# Influenza A virus during pregnancy disrupts maternal intestinal immunity and fetal cortical development in a dose- and time-dependent manner

**DOI:** 10.1038/s41380-024-02648-9

**Published:** 2024-07-03

**Authors:** Ashley M. Otero, Meghan G. Connolly, Rafael J. Gonzalez-Ricon, Selena S. Wang, Jacob M. Allen, Adrienne M. Antonson

**Affiliations:** 1 Neuroscience Program, University of Illinois Urbana-Champaign, Urbana, IL USA; 2 Department of Animal Sciences, University of Illinois Urbana-Champaign, Urbana, IL USA; 3 Stark Neurosciences Research Institute, Indiana University School of Medicine, Indianapolis, IN USA; 4 Department of Kinesiology and Community Health, University of Illinois Urbana-Champaign, Urbana, IL USA

**Keywords:** Neuroscience, Molecular biology, Cell biology

## Abstract

Epidemiological studies link exposure to viral infection during pregnancy, including influenza A virus (IAV) infection, with increased incidence of neurodevelopmental disorders (NDDs) in offspring. Models of maternal immune activation (MIA) using viral mimetics demonstrate that activation of maternal intestinal T helper 17 (T_H_17) cells, which produce effector cytokine interleukin (IL)-17, leads to aberrant fetal brain development, such as neocortical malformations. Fetal microglia and border-associated macrophages (BAMs) also serve as potential cellular mediators of MIA-induced cortical abnormalities. However, neither the inflammation-induced T_H_17 cell pathway nor fetal brain-resident macrophages have been thoroughly examined in models of live viral infection during pregnancy. Here, we inoculated pregnant mice with two infectious doses of IAV and evaluated peak innate and adaptive immune responses in the dam and fetus. While respiratory IAV infection led to dose-dependent maternal colonic shortening and microbial dysregulation, there was no elevation in intestinal T_H_17 cells nor IL-17. Systemically, IAV resulted in consistent dose- and time-dependent increases in IL-6 and IFN-γ. Fetal cortical abnormalities and global changes in fetal brain transcripts were observable in the high-but not the moderate-dose IAV group. Profiling of fetal microglia and BAMs revealed dose- and time-dependent differences in the numbers of meningeal but not choroid plexus BAMs, while microglial numbers and proliferative capacity of Iba1^+^ cells remained constant. Fetal brain-resident macrophages increased phagocytic CD68 expression, also in a dose- and time-dependent fashion. Taken together, our findings indicate that certain features of MIA are conserved between mimetic and live virus models, while others are not. Overall, we provide consistent evidence of an infection severity threshold for downstream maternal inflammation and fetal cortical abnormalities, which recapitulates a key feature of the epidemiological data and further underscores the importance of using live pathogens in NDD modeling to better evaluate the complete immune response and to improve translation to the clinic.

## Introduction

Influenza A virus (IAV) is a highly contagious respiratory pathogen that annually infects 5–10% of the global population [1]. Most individuals have mild symptoms; however, IAV infection during pregnancy poses a substantially increased risk of morbidity and mortality in both mother and infant [2–4] due to pregnancy-mediated changes to the maternal immune landscape [5]. Gestational IAV infection also has the potential to cause long-lasting negative health outcomes in the developing offspring. Epidemiological studies demonstrate that IAV infection during pregnancy increases the prevalence of offspring neurodevelopmental disorders (NDDs) like schizophrenia [6–8], bipolar disorder [9], and autism spectrum disorder (ASD) [10] (as reviewed in [11]).

Early rodent models of prenatal exposure to IAV found that offspring developed neuropathology similar to that seen in ASD and schizophrenia [12–14]. Notably, the cause of these brain abnormalities was determined to be from the maternal anti-viral inflammatory response rather than vertical transmission of the virus from mother to fetus [15]. Pathogen mimetics, such as bacterial endotoxin lipopolysaccharide (LPS) or synthetic dsRNA polyinosinic-polycytidylic acid (poly I:C), are currently popular choices for maternal immune activation (MIA) modeling due to the controlled and predictable innate immune response elicited, which enables researchers to target specific fetal developmental periods. While these mimetic models recapitulate NDD-like behavioral and neuropathological offspring phenotypes [16, 17], they fail to recreate the full spectrum of pathogen-induced pathological conditions. Unlike poly I:C, live viruses like IAV actively replicate within infected tissue and elicit a complex cascade of innate *and* adaptive immune responses [18–20]. Thus, certain characteristics of important downstream immune signaling cascades differ between poly I:C and live IAV. However, some canonical inflammatory signaling cascades may be conserved between the two models. Poly I:C-initiated MIA models first implicated interleukin (IL)-6, a pleiotropic inflammatory cytokine, in offspring NDDs [21–23]. Similarly, our group and others have also observed IAV-induced increases in IL-6 [24–26], yet the various mechanisms by which maternally derived IL-6 may directly impact offspring neurodevelopmental processes are still being revealed [23, 27]. IL-6 can also act upstream of effector cytokine IL-17, which has been more recently implicated as the major driver of fetal brain abnormalities. In poly I:C-induced MIA, increased production of IL-17 results from activation of pre-existing maternal intestinal T helper (T_H_)-17 cells [28–32]. While IL-17-producing intestinal T_H_17 cells have also been identified as potential drivers of IAV-mediated intestinal injury [33], this occurs on a very different time scale (6-8 days following IAV inoculation versus 24–48 h following poly I:C injection) and requires differentiation and propagation of naive CD4^+^ T cells into pathogenic T_H_17 cells [33, 34]. Therefore, we hypothesized that IAV infection during pregnancy would lead to activation of maternal intestinal T_H_17 cells and increased IL-17 production, albeit through unique innate and adaptive mechanisms.

Still, the mechanisms by which discrete maternal intestinal immune responses might perturb the fetal brain remain to be fully elucidated. Recent work from mimetic-induced MIA models indicates that elevated maternal IL-6 and IL-17 lead directly to fetal brain abnormalities by binding receptors on neurons, as indicated by increased transcription of neuronal IL-6 and IL-17 receptors [29]. While these changes appear to disrupt synaptogenesis and brain connectivity [23, 27], it is unclear how this proposed receptor-ligand binding sequence results in fetal cortical malformations. Furthermore, it is unclear whether a similar signaling cascade is activated during maternal IAV infection or if there are additional inflammatory signals at play.

Mounting evidence indicates that fetal microglia and border-associated macrophages (BAMs) respond to maternally derived inflammation [35, 36] and could be contributing to neocortical developmental abnormalities [37]. Microglia are present in the embryonic brain at the onset of neurogenesis [38], which places them at the center of early neuronal support. One of their many roles during development includes shaping the neocortex by stimulating neural precursor cell proliferation [39] and by subsequent phagocytosis of excess neural precursor cells [40]. At least one study directly implicates fetal microglia in mediating interneuron deficits during MIA [37]. Less is known about the role of BAMs during MIA, although recent evidence suggests they play a significant role in propagating MIA-induced inflammatory signaling at the embryonic choroid plexus [36]. Critically, fetal microglia and BAMs have never been examined during IAV-induced maternal inflammation. We hypothesized that maternal IAV infection would lead to notable shifts in fetal microglia and BAM phenotypes concomitant with disrupted neocortical development.

To test these hypotheses, we used an established rodent model of gestational viral infection with mouse-adapted IAV [24]. By comparing moderate and high IAV challenge doses in pregnant dams during peak innate and adaptive immunity (2- and 7-days post inoculation (dpi), respectively), we demonstrate that a maternal infection severity threshold exists for the onset of fetal brain abnormalities. Furthermore, while high-dose IAV induced fetal cortical abnormalities and increased the number of BAMs and brain-resident macrophage phagocytic capacity, IAV infection failed to increase circulating levels of maternal IL-17A and did not induce a pathogenic intestinal T_H_17 cell phenotype. These findings indicate that gestational IAV infection leads to aberrant fetal cortical development in the absence of upregulated levels of maternal IL-17.

## Materials and methods

### Animals

Singly housed male and pair-housed nulliparous female C57BL/6NTac mice obtained from Taconic Biosciences (Germantown, NY) at 9-to-10 weeks old were acclimated to the University of Illinois Urbana-Champaign animal facilities for a minimum of one week. Following acclimation, mice were trio-bred for 2-to-4 days. The presence of a vaginal plug was designated as gestational day (GD)0.5. Animals were maintained on a 12 h light–dark cycle, and body weights were recorded daily. A total of 60 pregnant dams across five biological replicates were used to examine tissues at 2 dpi (GD11.5). 29 pregnant dams across three biological replicates were used to examine tissues at 7 dpi (GD16.5). A separate cohort of 10 pregnant dams was used for bulk RNA-sequencing of the fetal brain at 7 dpi. All animal research was approved by and performed in accordance with The Institutional Animal Care and Use Committee (IACUC) at the University of Illinois Urbana-Champaign.

### Influenza A viral inoculation

Mouse-adapted IAV (subtype H3N2, strain X31) was provided by Dr. Jacob Yount at The Ohio State University. On GD9.5, pregnant dams were randomly assigned to infected or control groups. Murine infection at GD9.5 approximates the end of the first trimester in humans [41], a time when the risk of IAV infection leading to aberrant offspring neurodevelopmental outcomes is high [7]. They were then anesthetized by inhalation of isoflurane before intranasal inoculation with either a moderate dose of 10^3^ tissue culture infectious dose (TCID_50_) IAV (X31_mod_) or a high dose of 10^4^ TCID_50_ IAV (X31_hi_) in sterile saline. Control animals were inoculated with sterile saline (Con). Across three biological replicates at 2 dpi, 13 pregnant dams served as controls, 14 were inoculated with X31_mod_, and 12 were inoculated with X31_hi_. An additional two biological replicates at 2 dpi were used for colonic flow cytometry analysis, and 7 pregnant dams served as controls, 7 were inoculated with X31_mod_, and 7 were inoculated with X31_hi_. Across three identical replicates at 7 dpi, 10 pregnant dams served as controls, 9 were inoculated with X31_mod_, and 10 were inoculated with X31_hi_.

### Tissue collection

Tissues were collected and examined at either 2 or 7 dpi. Pregnant dams were euthanized by CO_2_ inhalation, and tissues were excised under sterile conditions. Maternal whole blood was obtained through blind cardiac puncture and subsequently centrifuged at 2000 × *g* at 4 °C for 10 min. Maternal serum was aliquoted and stored at −80 °C until analysis. Dams whose tissues were used for immunohistochemistry at 2 dpi were transcardially perfused with 25 mL sterile saline following blood collection. The uterus was removed from the dam and placed in ice cold phosphate-buffered saline (pH = 7.4; PBS), then placentae and fetuses were separated and cleaned. Fetuses were immersion fixed in 10% neutral buffered formalin (NBF) and stored at 4 °C. The ileum and right lung were collected from each pregnant dam and snap frozen on dry ice before storing at −80˚C until further processing. The left lung was immersion fixed in 10% NBF and stored at 4 °C until histopathology processing. Maternal colon length was measured before collection. The colon was then snap frozen and stored at −80 °C or placed in ice cold media for further processing (see section “Flow cytometry”).

### Lung histology

Dam lung tissue was processed at the University of Illinois Urbana-Champaign Veterinary Diagnostic Laboratory. Tissues were paraffin-embedded and stained with hematoxylin and eosin (H&E). Slides were evaluated by a board-certified veterinary comparative pathologist, Dr. Shih-Hsuan Hsiao, who was blinded to treatment groups. Semi-quantitative scoring of lung histopathology was performed using a rubric from previously published scoring methods [42]. The following lesions were independently scored for each sample: bronchitis, interstitial inflammation, edema, endothelialitis, pleuritis, and thrombus formation. Each lesion type was graded on a scale of 0 to 4 (0: absent, 1: mild, 2: moderate, 3: severe, 4: very severe). The total histopathological score is expressed as the sum of scores for all parameters with a maximum score of 24.

### Quantitative real-time PCR (qPCR)

RNA was isolated using TRIzol Reagent per the manufacturer’s protocol (Invitrogen, Carlsbad, CA, Catalog no. 15-596-026). RNA integrity and concentration were determined using the Nanodrop Nd-8000 Spectrophotometer (Thermo Fisher Scientific). Four micrograms of cDNA per sample was synthesized using the High-Capacity cDNA Reverse Transcription Kit (Applied Biosystems, Foster City, CA, Catalog no. 43-688-13) and Mastercycler Pro Thermal Cycler (Eppendorf) and was subsequently diluted 1:5 in DEPC water. qPCR was performed on a QuantStudio 5 Real-Time PCR System (Thermo Fisher Scientific) using PowerTrack SYBR Green Master Mix (Applied Biosystems, Catalog no. A46111). Data were analyzed using the 2^−ΔΔCt^ method against housekeeping genes (for mouse cDNA) or total Eubacteria (for microbial DNA) and presented as relative expression compared to control. *Rplp0* and *Hprt1* were used as housekeeping genes for mouse ileal and lung cDNA, respectively, and did not differ between treatment groups (*p*-value = 0.94, *p*-value = 0.28, respectively). Custom primer information is listed in Supplementary Table S1 (Integrated DNA Technologies, Coralville, IA).

### Protein immunoassays

Protein was isolated using Tissue Protein Extraction Reagent (T-PER) per the manufacturer’s protocol (Thermo Fisher Scientific, Waltham, MA, Catalog no. 78510). Protein concentration was measured using Pierce BCA Protein Assay (Thermo Fisher Scientific, Catalog no. PI23225) and read on BioTek ELx800 Microplate Reader (Agilent). Serum samples were analyzed using LEGENDplex MU Inflammatory Panel (13-plex) per the manufacturer’s protocol (BioLegend, San Diego, CA, Catalog no. 740446). All samples were run on the Attune NxT Flow Cytometer (Thermo Fisher Scientific) and analyzed on Qognit software (San Carlos, CA).

### Immunohistochemistry

Upon tissue collection, embryonic day (E)11.5 fetuses and E16.5 fetal heads were immersion-fixed in 10% NBF for 24 h at 4 °C. They were then decanted and washed twice in PBS followed by immersion in 30% sucrose solution with sodium azide for at least 48 h at 4 °C. E16.5 brains were dissected from heads after fixation and cryoprotection; E11.5 fetuses were kept whole. Cryoprotected tissue was embedded in OCT tissue-tek (Thermo Fisher Scientific, Catalog no. NC9159334) and stored long-term at −80 °C. 60 µm sagittal sections of E11.5 fetuses and 25 µm coronal sections of E16.5 fetal brains were cryosectioned. Free-floating batch staining was performed for each staining configuration. Sections were washed three times in PBS with 0.05% Tween-20 (Thermo Fisher Scientific, Catalog no. PRH5152; PBST) for 5 min each time and subsequently incubated with blocking buffer (5% goat serum [R&D Systems, Minneapolis, MN, Catalog no. S13110], 1% bovine serum albumin [Thermo Fisher Scientific, Catalog no. 126609100GM], 0.3% Triton-X 100 [Thermo Fisher Scientific, Catalog no. ICN19485450] in PBST) for 1 h at room temperature. For stains with a mouse host, sections were blocked with Mouse-on-Mouse IgG Blocking Solution for 1 h at room temperature (1:30; Thermo Fisher Scientific, Catalog no. R37621). Sections were incubated overnight at 4 °C with primary antibodies—rabbit anti-Iba1 (1:1000; Wako Chemicals U.S.A, Richmond, VA, Catalog no. 019-19741), rat anti-CD206 (1:1000; Biorad, Hercules, CA, Catalog no. MCA2235GA), rat anti-Ki67 (1:500; Invitrogen, Catalog no. 14-5698-82), rat anti-CD68 (1:1000; Biorad MCA1957GA), rabbit anti-TBR1 (1:1000; Abcam, Cambridge, UK, Catalog no. ab183032), or mouse anti-SATB2 (1:300; Abcam, Catalog no. ab51502). Sections were washed three times in PBST and incubated with secondary antibodies—Alexa Fluor 594 goat anti-rabbit IgG H&L (1:250; Jackson ImmunoResearch, West Grove, PA, Catalog no. 111-585-003), Alexa Fluor 488 goat anti-rat IgG H&L (1:250; Jackson ImmunoResearch, Catalog no. 112-545-003), or Alexa Fluor 488 goat anti-mouse IgG H&L (1:400; Thermo Fisher Scientific, Catalog no. A-11001)—for 2 h followed by staining in DAPI (Thermo Fisher Scientific, Catalog no. EN62248) for 1 min. Sections were mounted with Fluoromount-G Mounting Medium (Thermo Fisher Scientific, Catalog no. 5018788) and stored long-term at 4 °C.

### Imaging and image analysis

All images were acquired using a ZEISS AxioScan.Z1 slide scanner with system configurations as follows: Colibri 7 LED light source: 385 nm, 430 nm, 511 nm, 555 nm, 590 nm, 630 nm; Objectives: 5×/0.25 10×/0.45, 20×/0.8, 40×/0.5 Pol and 50×/0.8 Pol; Filter: GFP, DsRed, Cy5, DAPI/GFP/CY3/Cy5, and CFP/FP/mCherry; Camera: Hamamatsu Orca Flash, AxioCam IC (CCD AxioCam IC Color camera) and Hitachi HV-F202SCL. Images were subjected to the same conditions for each staining configuration. Image files were blinded and analyzed using ZEISS ZEN 3.0 Blue software (Oberkochen, DE). Cell counts were performed manually and normalized by whole brain area (mm^2^) for Iba1^+^CD206^−^ (microglia), Iba1^+^CD206^+^ (BAMs), Iba1^+^Ki67^+^ (proliferating Iba1^+^ cells), and Iba1^+^CD68^+^ (phagocytic Iba1^+^ cells). Mean fluorescence intensity (MFI) and cell counts were performed using ImageJ on a 300 × 300 µm^2^ region of interest (ROI) for SATB2 (upper excitatory neurons) and TBR1 (deep excitatory neurons) in E16.5 fetal brains. The ROI was further divided into 10 equal laminar bins, and the signal intensity of each bin was normalized relative to the total signal intensity of the ROI. The location of the primary somatosensory cortex region was determined based on the distance from the retrosplenial cortex relative to the length of the dorsal midline. E11.5 brain regions were identified using Chen et al. (2017) [43] and Kaufman’s Atlas of Mouse Development [44]. E16.5 brain regions were identified using Uta Schambra’s Prenatal Mouse Brain Atlas where coronal 15/16 was used for caudal regions and coronal 10/11 was used for rostral regions [45].

### Flow cytometry

Flow cytometry was performed on 2 and 7 dpi colonic lamina propria lymphocytes (LPLs) based on previously published methods [46]. The colon was flushed with FACS buffer (2% FBS [Thermo Fisher Scientific, Catalog no. MT35010CV], 2 mM EDTA [Thermo Fisher Scientific, Catalog no. 15-575-020] in 1 × HBSS [Thermo Fisher Scientific, Catalog no. 14175145]). Mesenteric fat was removed, and the colon was longitudinally bisected. Colons were incubated in EDTA-DTT buffer (2% FBS, 1 mM EDTA, 1 mM DTT [Sigma Aldrich, St. Louis, MO, Catalog no. 10708984001] in 1× HBSS) at 37 °C, 250 rpm for 20 min to remove epithelial cells and intraepithelial lymphocytes. Colons were subsequently incubated in digestion buffer (2% FBS, 50 µg/mL DNase I [Sigma Aldrich, Catalog no. 10104159001], and 62.5 µg/mL Liberase [Sigma Aldrich, Catalog no. 5401127011] in RPMI-1640 [Corning, Corning, NY, Catalog no. 15-040-CV]) at 37 °C, 250 rpm for 45 min. The resulting cells were isolated using a 100 µm cell strainer followed by a 40/80 Percoll (Thermo Fisher Scientific, Catalog no. 45001753) gradient. Cells were resuspended in T cell culture media (10% FBS, 50 µg/mL gentamicin sulfate [Corning, Catalog no. 30-005-CR], 2× GlutaMAX [Thermo Fisher Scientific, Catalog no. 35050061], 1× Penicillin/Streptomycin [Corning, Catalog no. 30002Cl], and 55 µM 2-Beta-Mercaptoethanol [Thermo Fisher Scientific Catalog no. 21-985-023] in RPMI-1640) and counted using Invitrogen Countess Automated Cell Counter and adjusted so there were 1 × 10^6^-2 × 10^6^ cells/100 µl. Cells were then cultured for 3 h in T cell stimulation media (1000 ng/mL PMA [Sigma Aldrich, Catalog no. P1585-1MG], 2 µM Ionomycin [Sigma Aldrich, Catalog no. I0634-1MG], and 2 µg/mL GolgiPlug [BD Biosciences, Franklin Lakes, NJ, Catalog no. 555029] in T cell culture media) at 37 °C, 5% CO_2_. Cells were stained with LIVE/DEAD Fixable Aqua (Thermo Fisher Scientific, Catalog no. L34966), Rat anti-mouse CD16/32 Fc Block (BD Biosciences, Catalog no. 553142), and surface markers CD4 anti-mouse BB700 (BD Biosciences, Catalog no. 566407) and CD45 anti-mouse APC-Cy7 (BD Biosciences, Catalog no. 557659). Cells were then fixed and permeabilized using FOXP3 Transcription Factor Staining Buffer Set (Thermo Fisher Scientific, Catalog no. 00-5523-00) and stained for intracellular markers RORγt anti-mouse PE-CF594 (BD Biosciences, Catalog no. 562684), Tbet anti-mouse APC (Thermo Fisher Scientific, Catalog no. 17-5825-82), IL-17A anti-mouse Alexa Fluor 488 (BioLegend, Catalog no. 506910), IL-17F anti-mouse PE (BD Biosciences, Catalog no. 561627), and IFN-γ anti-mouse Brilliant Violet 421 (BioLegend, Catalog no. 505830). Cells were run on the Attune NxT Flow Cytometer. Fluorescence minus ones (FMOs) were used daily for gating. Compensation was done using UltraComp compensation beads (Thermo Fisher Scientific, Catalog no. 501129040). Flow analysis was done using FlowJo (Ashland, OR). Absolute cell counts were calculated as the percent of the gated subset multiplied by the total number of lymphocytes per 100 µl sample.

### 16S sequencing

DNA extraction was performed using a QIAamp Fast DNA Stool Mini Kit (Qiagen, Valencia, CA, Catalog no. 51604) following the manufacturer’s instructions, with slight modifications as previously described [47]. Briefly, 20-40 mg of stool was incubated for 45 min at 37 °C in lysozyme buffer (22 mg/ml lysozyme, 20 mM Tris-HCl, 2 mM EDTA, 1.2% Triton-x, pH 8.0), then bead-beat for 150 s with 0.1 mm zirconia beads. Samples were incubated at 95°C for 5 min with InhibitEX Buffer, then incubated at 70 °C for 10 min with Proteinase K and Buffer AL. Following this step, the QIAamp Fast DNA Stool Mini Kit isolation protocol was followed, beginning with the ethanol step. DNA was quantified with the Qubit 2.0 Fluorometer (Life Technologies) using the dsDNA Broad Range Assay Kit.

After extraction and DNA quality assurance through gel electrophoresis, library construction was completed using a Fluidigm Access Array system in the Functional Genomics Unit of the Roy J. Carver Biotechnology Center at the University of Illinois Urbana-Champaign. After library construction, 250 bp of the V4 region of the 16SrRNA gene were amplified and sequenced at the WM Keck Center for Biotechnology at the University of Illinois Urbana-Champaign using an Illumina MiSeq2000. The V4 region of the 16S rRNA gene was amplified using primers 515F (5′-GTGYCAGCMGCCGCGGTAA-3′) and 806R (5′-GGACTACNVGGGTWTCTAAT-3′). PCR reactions were conducted in triplicate and resulting amplicons were pooled.

Illumina libraries were generated from the pooled amplicons and paired-end (2 × 250 nt) sequencing was performed on an Illumina MiSeq. After sequencing and barcode trimming, raw sequence data (FASTQs) underwent quality control using DADA2, trimming low-quality bases with a cutoff Phred score >30. DADA2 was used for denoising, merging paired-end reads, and inferring amplicon sequence variants (ASVs). ASVs were mapped to SILVA rRNA database version 138.1 with QIIME2. Alpha diversity metrics (Shannon’s Index) were calculated to assess within-sample diversity. Beta diversity metrics (Unweighted Unifrac) were calculated to assess between-sample diversity. Downstream statistical analysis and data visualization were completed with MicrobiomeAnalyst. Taxa abundance data were filtered as follows: minimum count = 4, prevalence in samples = 20%, and percentage to remove = 10%. Data were then transformed using the centered log-ratio (CLR) method to mitigate compositional biases and improve interpretability. Negative binomial regression models were employed to explore relationships between dam influenza status and the gut microbiome.

### Bulk RNA sequencing

RNA from pooled E16.5 fetal whole brains was extracted using the RNeasy Mini Kit (Qiagen, Catalog no. 74004). RNA quality and integrity were then determined by 28S/18S rRNA analysis with the Agilent 2100 Bioanalyzer. All samples scored an RNA Quality Number over 7 indicating little to no signs of degradation (Fig. S1A). RNA sequencing was performed at the Roy J. Carver Biotechnology Center. RNA-Seq libraries were prepared using the KAPA Stranded RNA-Seq Library Preparation Kit with an average fragment length of 100 bp. Libraries were pooled and quantified using qPCR and were then sequenced on one S Prime (SP) lane for 101 cycles from one end of the fragments on a NovaSeq 6000 (Illumina). FASTQ files were generated from the raw sequencing runs and demultiplexed with the bcl2fastq v2.20 Conversion Software (Illumina). Adapters were trimmed from the 3′-end of the reads, and FASTQC v0.11.8 indicated no adapter sequence contamination and average per-base quality scores over 30 in all samples (Fig. S1B).

All reference files were downloaded from the National Center for Biotechnology Information (NCBI) ftp site. The *Mus musculus* H3N2 transcriptome file “GCF_000001635.27_GRCm39_rna.fna.gz; influenza_A_X-31_H3N2_rna.fa” from NCBI Annotation 109 was used for quasi-mapping and count generation. This transcriptome is derived from genome GRCm39 (Mouse); A/X-31(H3N2). Since the quasi-mapping step only uses transcript sequences, the gene model file “GCF_000001635.27_GRCm39_genomic.gff.gz; influenza_A_X-31_H3N2.gff3” was used to generate transcript-gene mapping table (“trx_Egids_GRCm39_annot109.txt”) for gene-level counts. Our raw sequencing data can be found at GEO (accession no. GSE262291).

Salmon version 1.4.0 was used to quasi-map reads to the transcriptome and quantify the abundance of each transcript. The transcriptome was first indexed using the decoy-aware method in Salmon with the entire genome file “GCF_000001635.27_GRCm39_genomic.fna.gz; influenza_A_X-31_H3N2_genes.fa” as the decoy sequence. Then quasi-mapping was performed to map reads to the transcriptome with additional arguments –seqBias and –gcBias to correct sequence-specific and GC content biases, –numBootstraps=30 to compute bootstrap transcript abundance estimates, and –validateMappings to help improve the accuracy of mappings. Gene-level counts were then estimated based on transcript-level counts using the bias-corrected counts without an offset method from the ‘tximport’ package in R. The percentage of reads mapped to the transcriptome for all samples was between 70-80% (Fig. S2A). Normalization of samples was between 0.97-1.03 using the trimmed mean of M values (TMM) method (Fig. S2B). Remove Unwanted Variation (RUV) analysis was performed on the data. RUV is a method to estimate factors that can be added to the statistical model (co-variates) assuming that these factors are spurious technical variations in the samples and not biologically related. Additional normalization using RUV is often necessary for RNA-Seq experiments and helps to improve biological insights [48]. Clustering after RUV removal was done using the limma package in R (Fig. S2C). The limma-trend method was used to find differentially expressed genes using a model of ~ treatment + 4 RUV factors [49]. *P*-values of differentially expressed genes were adjusted using Benjamini-Hochberg’s correction for false discovery (*p* < 0.1). The Database for Annotation, Visualization, and Integrated Discovery (DAVID) was used to find the top ten significantly enriched up and downregulated Gene Ontology (GO) pathways. Independent qPCR of six biologically relevant genes was used to validate RNA-seq findings (Fig. S2E, F).

### Statistics

Dam was treated as the experimental unit for all outcomes, and one randomly selected fetus per litter was used for each experimental outcome. An a priori power analysis was conducted using G*Power v3.1.9.6 for sample size estimation based on data from IL-6 serum with an effect size F = 0.86. With a significance criterion of α = 0.05 and power = 0.80, the minimum sample size needed with this effect size is N = 18, and our minimum obtained sample size was N = 21. All data were analyzed using GraphPad Prism 9 Software (San Diego, CA) with significance set at alpha = 0.05 unless otherwise specified. Dam body weights across time were analyzed using repeated measures two-way ANOVA with Geisser-Greenhouse correction for sphericity and Tukey correction for multiple comparisons. Con, X31_mod_, and X31_hi_ groups were compared using one-way ANOVA assuming Gaussian distribution and homogeneity of variance of residuals, and Tukey correction for multiple comparisons was used. For data with unequal variance, Brown-Forsythe ANOVA was performed with Dunnet T3 correction for multiple comparisons. For non-parametric data, Kruskal-Wallis ANOVA was performed with Dunn’s correction for multiple comparisons. Kruskal-Wallis was used in the case that residuals did not meet normality or homogeneity of variance. Outliers were identified and removed using the ROUT method with Q = 1%.

## Results

### Respiratory IAV infection leads to maternal inflammation at the site of infection and systemically in a dose- and time-dependent manner

**Fig. 1:**
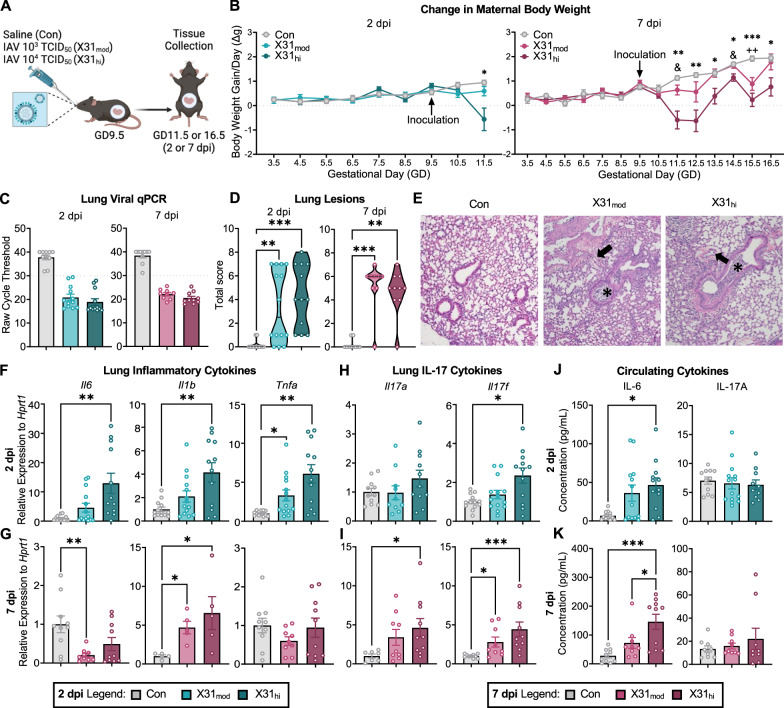
Respiratory IAV infection alters maternal lung inflammation and circulating cytokines in a dose- and time-dependent manner. **A** Experimental schematic. **B** IAV inoculation at GD9.5 suppressed body weight gain per day from 2-to-7 dpi in X31_hi_ dams only (repeated measures 2-way ANOVA, the main effect of time = p < 0.001; * = Con vs X31_hi_, & = X31_mod_ vs X31_hi_, + = Con vs X31_mod_). **C** The presence of IAV-X31 in lungs at 2 and 7 dpi was evaluated using qPCR with a cycle threshold of ≤ 30 cycles as confirmed infection (dotted line). **D** Quantification of H&E pathological scoring showed elevated lung lesion scores in infected dams. The scoring criteria are listed in the methods with additional scoring values in Supplementary Table S3. **E** Representative photomicrographs of H&E-stained lung sections. Asterisks (*) indicate bronchi filled with clusters of neutrophils with cellular debris, and arrows (→) indicate arterial and venous endothelia with rolling neutrophils. Genes encoding for classic pro-inflammatory cytokines IL-6, IL-1β, and TNF-α in maternal lungs were **F** upregulated in a dose-dependent manner at 2 dpi whereas **G** only *Il1b* was upregulated at 7 dpi. IL-17 genes were upregulated in the maternal lung at **H** 2 and **I** 7 dpi in a dose-dependent manner. Maternal cytokines in circulation at **J** 2 and **K** 7 dpi. Pro-inflammatory cytokine IL-6 was upregulated in moderate- and high-dose dams proportional to dosage at both time points. IL-17A was not upregulated in circulation at either endpoint. IAV = influenza A virus, GD = gestational day, *dpi* = days post-inoculation, Con = saline control, X31_mod_ = IAV-X31 10^3^ TCID_50_, X31_hi_ = IAV-X31 10^4^ TCID_50_. Groups were compared using one-way ANOVA with Tukey post hoc for multiple comparisons. For data containing residuals with unequal variance, Brown-Forsythe and Welch’s ANOVA with Dunnett T3 post hoc multiple comparisons was used. For non-parametric data, Kruskal–Wallis ANOVA with Dunn’s correction for multiple comparisons was used. Data are means ± SEM; one symbol = p < 0.05, two symbols = p < 0.01, three symbols = *p* < 0.001; dots represent individual dams; n = 9–14 per treatment group. See Supplementary Tables S3–5 for complete statistical analysis of all data collected for this figure (individual mean ± SEM per group, p-values, hypothesis test used, and test statistic).

To capture the complete immune response, we evaluated pregnant dams and fetuses at 2 and 7 dpi to approximate the peak of both innate and adaptive anti-viral responses, respectively [50, 51]. We previously reported that fetal brain neuroinflammatory transcripts are unaltered by inoculation with a moderate infectious titer of IAV (X31_mod_) [24]; thus, we included an additional high infectious titer (X31_hi_) in the current study (Fig. 1A). To determine whether the infectious dose of IAV impacts maternal symptomology and pathology, dam body weights were monitored daily (Fig. S3), and anti-viral immune response was examined in the lungs and systemically. Dam body weight gain per day was significantly decreased from 2-to-7 dpi in X31_hi_ dams whereas X31_mod_ dams exhibited a decrease in body weight gain per day only at 6 dpi (Fig. 1B). Both moderate- and high-dose dams demonstrated a spike in body weight gain per day at 5 dpi followed by a subsequent dip at 6 dpi, which could indicate a potential shift from innate to adaptive immunity [51]. IAV infection did not affect litter size or number of fetal resorptions at either time point, which is consistent with our previous findings [24] (Supplementary Table S2). The presence of viral RNA was confirmed in infected maternal lungs, although there was no difference in gene expression between high and moderate doses (Fig. 1C). The lack of change in viral RNA between infected groups was expected as the initial viral dose is not indicative of viral load or symptomology [52–54]. Histopathological scoring of H&E-stained maternal lungs showed increased total lung lesion scores regardless of viral dose (Fig. 1D-E). Specifically, scores for bronchitis, interstitial inflammation, and endothelialitis were upregulated throughout the infection (Supplementary Table S3). Genes encoding for classic pro-inflammatory cytokines IL-6, IL-1β, and tumor necrosis factor-alpha (TNF-α) were upregulated in the lungs in a dose-dependent manner at 2 dpi (Fig. 1F). Only *Il1b* remained elevated at 7 dpi (Fig. 1G, Supplementary Table S4). Type II antiviral interferon-gamma (IFN-γ) was upregulated in X31_hi_ dams only during acute infection (Fig. S4A). Type I and type II interferons were downregulated in IAV-infected lungs at 7 dpi, which is consistent with prior kinetic studies of IAV-X31 [55] (Fig. S4B). We then evaluated IL-17 expression in the lungs. Elevated IL-17 signaling during IAV infection is shown to cause acute lung injury while also playing a protective role against secondary bacterial infections [56, 57]. In line with these findings, we observed an increase in *Il17f* at 2 dpi (Fig. 1H) and an increase in *Il17a* and *Il17f* at 7 dpi in a dose-dependent manner (Fig. 1I).

To evaluate the systemic impacts of IAV across viral replication, we measured maternal serum cytokine concentrations (Supplementary Table S5). IL-6 was upregulated in a dose-dependent manner at 2 and 7 dpi (Fig. 1J, K). Anti-inflammatory cytokine IL-10 differed across groups at 2 dpi (Fig. S4C, D). IFN-γ and IL-23–a cytokine required for the commitment and propagation of T_H_17 cells [58]–were also upregulated in a dose-dependent manner at 7 dpi (Fig. S4D). Notably, IL-17A was not upregulated at either time point (Fig. 1J, K), which differs from poly I:C-induced MIA studies showing an elevation of IL-17A in maternal circulation [29, 30]. However, this is consistent with a lack of elevated IL-17A in the serum of IAV-infected male mice [33].

### Respiratory IAV infection disrupts maternal intestinal immunity in a dose- and time-dependent manner

**Fig. 2:**
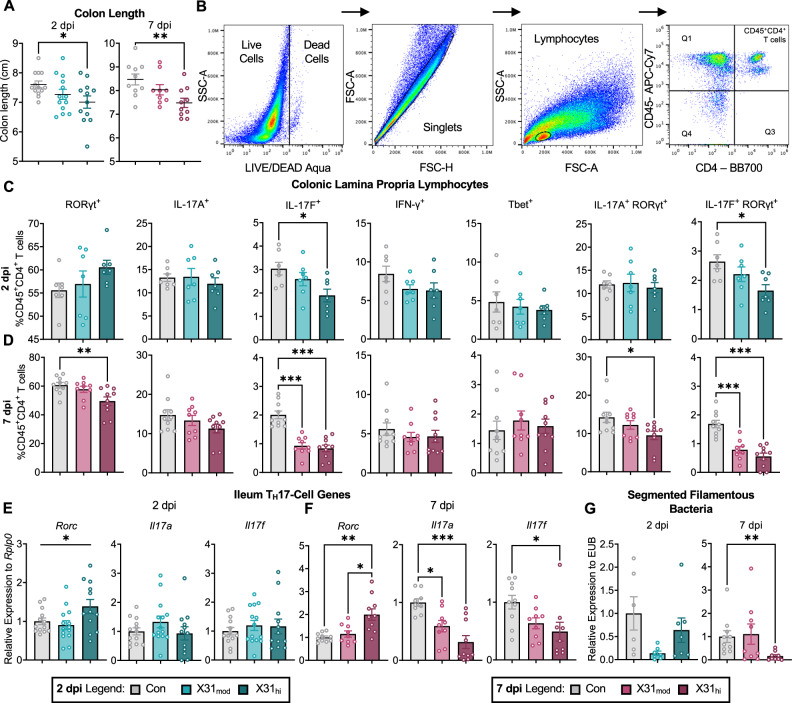
Respiratory IAV infection dysregulates maternal intestinal immunity in a dose- and time-dependent manner. **A** IAV reduced colon length in high-dose dams as early as 2 dpi, and this persisted at 7 dpi. **B** Example flow cytometry gating for colonic LPLs. Gating on CD45^+^CD4^+^ T cells in the colon revealed **C** downregulation of IL-17F^+^ and IL-17F^+^RORγt^+^ T cells at 2 dpi which persisted into **D** 7 dpi in addition to downregulation of RORγt^+^ and IL-17A^+^RORγt^+^ T cells. qPCR of the ileum, the terminal end of the small intestine, confirms findings in the colon where **E** little changes were observed at 2 dpi and **F** downregulation in *Il17a* and *Il17f* was seen at 7 dpi. Notably, *Rorc* transcription did not coincide with decreased RORγt protein expression. **G** Relative gene expression via qPCR showed a decrease in SFB, a bacterial regulator of T_H_17 cells, in the colon contents of high-dose dams at 7 dpi. IAV = influenza A virus, dpi = days post-inoculation, LPL = lamina propria lymphocytes, Con = saline control, X31_mod_ = IAV-X31 10^3^ TCID_50_, X31_hi_ = IAV-X31 10^4^ TCID_50,_ SSC-A = side scatter-area, FSC-A = forward scatter-area. Groups were compared with one-way ANOVA with Tukey post hoc for multiple comparisons. For data containing residuals with unequal variance, Brown-Forsythe and Welch’s ANOVA with Dunnett T3 post hoc multiple comparisons was used. For non-parametric data, Kruskal-Wallis ANOVA with Dunn’s correction for multiple comparisons was used. Data are means ± SEM; *p < 0.05, **p < 0.01, ***p < 0.001; dots represent individual dams; n = 7–14 per treatment group. See Supplementary Tables S6–7 for complete statistical analysis of all data collected for this figure (individual mean ± SEM per group, p-values, hypothesis test used, and test statistic).

We evaluated the maternal intestines to determine if alterations in T_H_17 cells are present during gestational IAV, similar to poly I:C-induced MIA [29, 30], and if these T_H_17 cells transition to a pathogenic phenotype, similar to IAV-infected male mice [33]. At 2 dpi, high-dose dams exhibit colonic shortening, which persists out to 7 dpi, a finding that has been found in previous mouse models of IAV [24, 33] (Fig. 2A). Colonic shortening is a hallmark of intestinal inflammation and colitis [59]. Furthermore, while fewer T_H_17 cells reside in the colon compared to the small intestine, colonic T_H_17 cells are more susceptible to developing pathogenic phenotypes [34, 60]. Therefore, we decided to phenotype colonic lamina propria T_H_17 cells in IAV-infected dams (Fig. 2B, Supplementary Table S6). CD45^+^CD4^+^ lymphocytes were gated on markers for homeostatic T_H_17 (RORγt, IL-17A, IL-17F), T_H_1 (Tbet, IFN-γ), and pathogenic T_H_17 (RORγt, Tbet, IFN-γ,IL-17A) cells [34, 61]. We observed a downregulation in the percentage of IL-17F^+^ and IL-17F^+^RORγt^+^ colonic T cells from X31_hi_ dams at 2 dpi (Fig. 2C). This finding persisted into 7 dpi with an additional decrease in RORγt^+^ and IL-17A^+^RORγt^+^ T cells (Figs. 2D, S5A). When quantifying absolute cell count, we observed no differences at 2 dpi and downregulation in RORγt, IL-17A, IL-17F, IFN-γ and double positive RORγt cells at 7 dpi (Fig. S4E, F, Supplementary Table S6). Therefore, not only are the relative percentages of T_H_17 cell populations downregulated, but so are the absolute cell counts with respect to the total lymphocyte population. Further gating on CD45^+^CD4^+^RORγt^+^ and CD45^+^CD4^+^Tbet^+^ T cells revealed downregulation in classic T_H_17 (IL-17F^+^) cells at 2 and 7 dpi and no changes in classic T_H_1 cells, respectively (Fig. S5B–E). Additionally, there was no evidence to indicate an increase in pathogenic colonic T_H_17 cells when gating for RORγt^+^Tbet^+^ and IL-17A^+^IFN-γ^+^ double-positive cells (Fig. S5F, G).

To determine if the downregulation of IL-17 was specific to the colon, we looked at gene expression in the ileum of the small intestine, which is where the majority of homeostatic T_H_17 cells reside [62, 63] and the typical intestinal region of interest in MIA studies [28, 29]. qPCR of the ileum revealed a similar pattern to colonic flow cytometry findings: no transcripts differed at 2 dpi whereas both *Il17a* and *Il17f* decreased in the IAV groups at 7 dpi (Fig. 2E, F). There was also a decrease in ileal *Il22*, another cytokine produced by T_H_17 cells (Supplementary Table S7). Contrastingly, transcription of *Rorc*, the gene encoding for T_H_17 cell transcription factor RORγt, was upregulated at both time points in high-dose dams only (Fig. 2E, F). We previously observed this same upregulation in *Rorc* in both the colon and ileum of moderate-dose dams at 7 dpi [24]. Thus, increased transcription of *Rorc* does not coincide with an increased number of RORγt^+^ cells in this model. We then looked upstream at T_H_17-cell priming genes and found upregulation in ileal *Il6*, downregulation in *Tgfb1* and *Il1b*, and no changes in *Il23a* at 7 dpi (Supplementary Table S7). Altogether, these data demonstrate that gestational IAV alters intestinal immunity throughout infection despite a lack of viral replication within the intestines (Supplementary Table S7). Notably, it appears that maternal colonic shortening cannot be explained by IL-17-producing T_H_17 cells in our model of IAV infection.

### Respiratory IAV infection alters the maternal colonic microbiome

Intestinal T_H_17 cells rely on segmented filamentous bacteria (SFB), which regulate the development of homeostatic T_H_17 cell populations in the small intestine [63]. Some have found that the presence of SFB in pregnant mice is necessary to induce activation of T_H_17 cells and is also required for neuropathological and behavioral abnormalities in offspring from poly-I:C-induced MIA [28, 30]. Others have recently demonstrated offspring behavioral alterations in the absence of maternal SFB [64]. We observed a decrease in SFB gene expression in X31_hi_ dams at 7 dpi (Fig. 2G), which corroborates what was previously found in the intestinal contents of IAV-infected male mice [33].

Intestinal microbial dysbiosis as a characteristic of respiratory IAV infection has been found in both human and animal models [33, 65–67]. To evaluate potential microbial disruption, we performed 16S rRNA sequencing on the colon contents of control and IAV-infected mice at 2 and 7 dpi. There was no difference between X31_mod_ and X31_hi_ treatment groups when evaluating within-sample diversity (alpha diversity) or between-sample diversity (beta diversity) at 2 dpi (p-and q-value = 0.75; p- and q-value = 0.25, respectively) or 7 dpi (p- and q-value = 0.93; p- and q-value = 0.74, respectively); therefore, X31 treatment groups were collapsed. Alpha diversity was only different at 2 dpi (Fig. S6A) whereas beta-diversity was different at both endpoints (Fig. S6B, C). Taxonomic analysis revealed no differentially expressed bacteria at the genus level when adjusting for false discovery rate (Fig. S6D). However, heat tree analysis verified the upregulation in *Bacteroides* along with the downregulation of several genus-level microbes associated with the Firmicutes phylum (Fig. S6E, F). A decrease in Firmicutes and upregulation in Bacteroidetes (the phyla *Bacteroides* falls under) was previously reported in mice with respiratory syncytial virus (RSV) infection [66] and in humans with IAV infection [68]. Altogether, these data verify colonic microbial alterations in gestating dams upon exposure to IAV.

### Gestational IAV infection leads to cortical abnormalities in the fetal brain

**Fig. 3:**
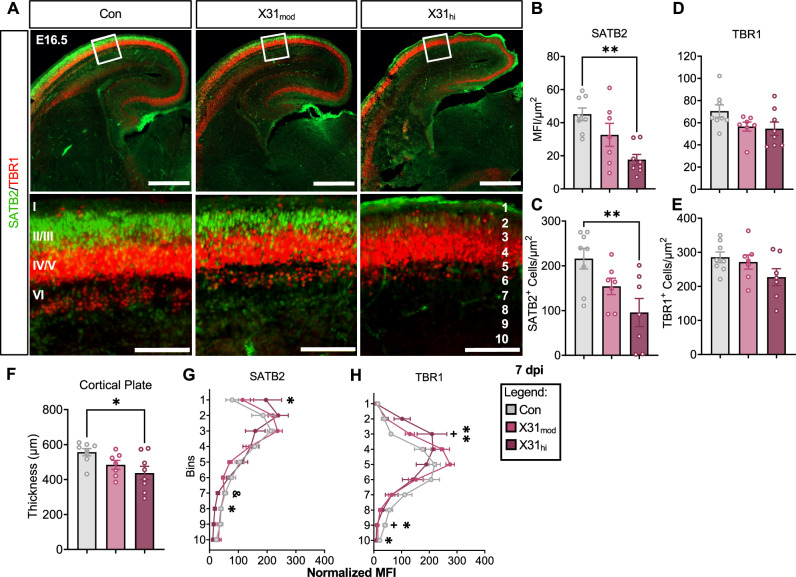
Respiratory IAV infection during pregnancy impacts cortical development in fetal brains from high- but not moderate-dose dams at E16.5, 7 dpi. **A** Representative images of E16.5, 7 dpi fetal brains stained for SATB2 (green) and TBR1 (red). Close-up images were taken in the right-hemisphere somatosensory cortex in a 300 × 300 µm^2^ ROI. Top scale bars = 500 µm; bottom scale bars = 100 µm. I–VI in the bottom left image represents cortical layers 1–6, and 1–10 in the bottom right image represents the bins. **B**, **C** MFI and cell count per µm^2^ of SATB2, an upper excitatory neuronal marker, is decreased in the fetal brains of high-dose mothers. **D**, **E** MFI and cell counts of TBR1, a deep excitatory neuronal marker, were not statistically different between groups (p-value = 0.10). **F** Prenatal exposure to IAV-X31 reduced cortical thickness in fetal brains from X31_hi_ dams. Dividing the ROI into 10 equal cortical laminar bins showed altered cortical lamination in **G** SATB2 bins 1, 7, and 8 and **H** TBR1 bins 3, 9, and 10 (* = Con vs X31_hi_, & = X31_mod_ vs X31_hi_,+= Con vs X31_mod_). IAV = influenza A virus, dpi = days post-inoculation, E = embryonic day, ROI = region of interest, MFI = mean fluorescence intensity, Con = saline control, X31_mod_ = IAV-X31 10^3^ TCID_50_, X31_hi_ = IAV-X31 10^4^ TCID_50_. Groups were compared with one-way ANOVA with Tukey post hoc for multiple comparisons. For data containing residuals with unequal variance, Brown-Forsythe and Welch’s ANOVA with Dunnett T3 post hoc multiple comparisons was used. For non-parametric data, Kruskal–Wallis ANOVA with Dunn’s correction for multiple comparisons was used. Data are means ± SEM; one symbol = p < 0.05, two symbols = p < 0.01; dots represent one representative fetus per litter; n = 9–10 per treatment group. See Supplementary Table S8 for complete statistical analysis of all data collected for this figure (individual mean ± SEM per group, p-values, hypothesis test used, and test statistic).

We next wanted to see if maternal IAV infection led to cortical abnormalities as previously described in mimetic-induced MIA models [28, 29, 36, 69–72]. We performed immunohistochemistry on fetal brains at E16.5, as cortical layers are not distinguishable until E13.5 [43] (Supplementary Table S8). We examined the primary somatosensory cortex (Fig. 3A) based on prior MIA studies [71]. We observed a decrease in SATB2^+^ cells and SATB2 mean fluorescence intensity (MFI) in fetal brains from high-dose infected dams (Fig. 3B, C), and no changes in TBR1 cell count or fluorescence (Fig. 3D, E), which is consistent with prior studies [29]. Notably, we proceeded with MFI analysis for subsequent measurements since cell counts and MFI are highly correlated (Fig. S7A). SATB2 and TBR1 group differences were apparent in both the right and left hemispheres (Fig. S7B), did not differ between hemispheres (Fig. S7D), and displayed a similar pattern in a more rostral region along the anterior-posterior axis (Fig. S7C). We also saw a dose-dependent decrease in cortical plate thickness (Fig. 3F), a pathological indication of NDDs in humans [73–75]. We then divided this somatosensory cortical region of interest (ROI) into 10 equal laminar bins to determine the presence or absence of cortical malformations as previously described [29, 70–72]. Significant differences were observed in bins 1, 7, and 8 for SATB2 MFI (Fig. 3G) and bins 3, 9, and 10 for TBR1 MFI in a dose-dependent manner (Fig. 3H). Overall, these data indicate that prenatal respiratory IAV infection leads to perturbations in fetal cortical development in a dose-dependent manner, providing further support for the existence of an infection severity threshold.

### Bulk RNA sequencing of the fetal brain reveals IAV-dependent transcriptional changes related to synaptic signaling and neuronal development

**Fig. 4:**
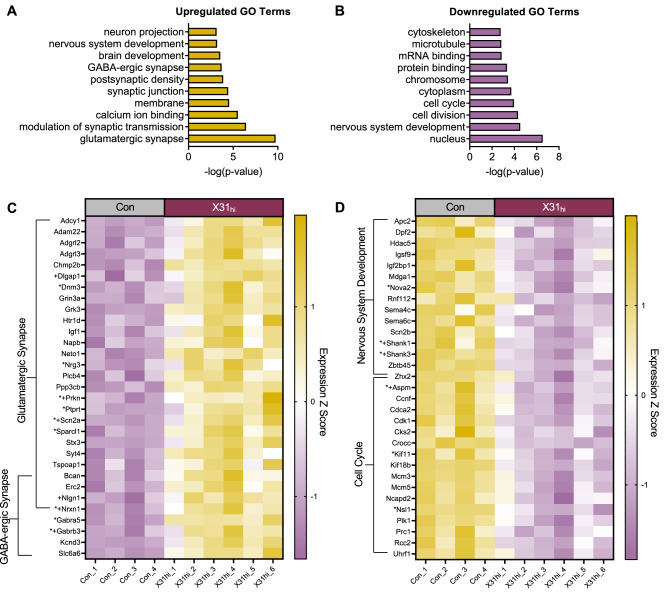
Bulk RNA-seq reveals genes enriched in neuronal development and synaptic signaling in fetal brains exposed to a high dose of prenatal IAV infection at E16.5, 7 dpi. Top ten significantly enriched **A** upregulated and **B** downregulated GO pathways in fetal brains prenatally exposed to a high dose of IAV, quantified by –log (p-value). Pathways were generated from the Database for Annotation, Visualization, and Integrated Discovery terms (DAVID). **C** Heatmap of genes differentially upregulated in the glutamatergic and GABA-ergic synapse pathways. **D** Heatmap of genes differentially downregulated in the nervous system development and cell cycle pathways. Genes with * represent candidate genes for neurodevelopmental disorders based on the Developmental Brain Disorder Gene Database (DBD). Genes with + represent candidate genes for Autism Spectrum Disorder based on the Simons Foundation Autism Research Initiative (SFARI) genes database. IAV = influenza A virus, dpi = days post-inoculation, Con = saline control, X31_hi_ = IAV-X31 10^4^ TCID_50,_ GO = gene ontology. Benjamini–Hochberg’s correction for false discovery (p < 0.1) was used to identify differentially expressed genes. Data are standardized logCPM values (Z score); dots represent one representative fetus per litter; n = 4–6 per treatment group. See Supplementary Tables S9–10 for additional statistical analysis of data collected for this figure.

Since a high dose of IAV was required to produce cortical abnormalities, and since we did not observe any changes in the 591 neuroinflammatory genes evaluated in X31_mod_ fetal brains from our previous study [24], we performed bulk RNA-sequencing on control and X31_hi_ fetal brains at E16.5, 7 dpi. Differential gene expression analysis revealed 211 differentially upregulated and 173 differentially downregulated genes (Fig. S2D). We then used DAVID to determine the top ten significantly enriched up- and down-regulated GO pathways. Upregulated GO pathways largely corresponded to synaptic signaling (Fig. 4A), and downregulated GO pathways largely corresponded to neuronal and cellular development (Fig. 4B; Supplementary Table S9). These top GO pathways are in alignment with GO pathways enriched in human NDDs [76, 77]. Furthermore, a myriad of differentially expressed genes in the selected pathways are candidate genes for NDDs based on the Developmental Brain Disorder Gene Database (DBD) and the Simons Foundation Autism Research Initiative (SFARI) database (Fig. 4C, D, as indicated by * and + symbols, respectively). In contrast to findings from poly I:C MIA models [29], transcription of *Il17ra* and *Il17rc* (encoding the receptor complex for IL-17A and F) was unchanged in X31_hi_ fetal brains (adj. p = 0.61 and 0.83, respectively). Furthermore, no transcriptional differences were observed for *Il6ra* (adj. p = 0.84) or interferon-related genes (Supplementary Table S10).

The glutamatergic synapse pathway (GO:0098978) was the most significantly enriched upregulated GO term (Fig. 4A). Excitatory synaptic cell-adhesion molecules, *Nrxn1* and *Nlgn1*, were upregulated. Interestingly, the gene encoding for excitatory postsynaptic scaffolding protein SHANK3 (*Shank3*; adj. p = 0.09) was downregulated. This may indicate an imbalance in the development of pre- and post-synaptic terminals during gestational IAV infection. The GABA-ergic synapse pathway (GO:0098982) was also upregulated, as evidenced by upregulation in GABA receptor genes *Gabrb3 and Gabra5* (Fig. 4C, Fig. S2E).

Nervous system development (GO:0007399) and cell cycle (GO:0007049) were among the most significantly enriched downregulated GO pathways (Fig. 4B). This was evident in the downregulation of genes encoding for proteins related to cortical lamination, like *Apc2* and *Mdga1* (Figs. 4D, S2F). *Apc2* is involved in neuronal migration and axonal projection [78], and *Mdga1* plays a role in upper excitatory neuronal migration [79]. Genes responsible for proper embryonic neuronal migration and development including *Aspm* [80], *Cdk1* [81], and *Cks2* [82]—each of which are also involved in cell cycling—were also downregulated (Fig. 4D). Notably, expression of *Satb2* and *Tbr1* was unchanged (adj. p = 0.61 and 0.49, respectively); however, transcription of nuclear genes does not always equate to translation. Overall, these data indicate that gene pathways related to synaptic signaling and neuronal development are among the most dysregulated in the developing brain following prenatal exposure to a high dose of IAV.

### Gestational IAV infection alters embryonic brain-resident macrophages in a dose- and time-dependent manner

**Fig. 5:**
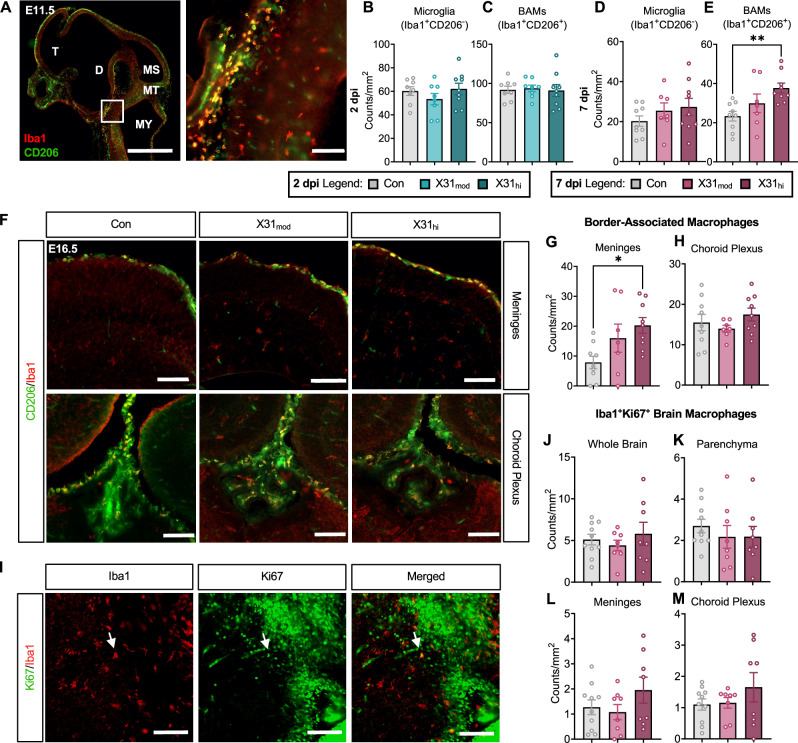
Border-associated macrophages but not microglia are upregulated in fetal brains from high-dose IAV dams. **A** Representative sagittal sections of the E11.5 fetal brain stained with Iba1 (red) and CD206 (green). Microglia are Iba1^+^CD206^-^ and BAMs are Iba1^+^CD206^+^. Left scale bar = 1000 µm; right scale bar = 100 µm. There were no changes in **B** microglia or **C** BAM count per mm^2^ at E11.5, 2 dpi. While there were no changes in **D** microglia count, **E** BAM count was upregulated in E16.5, 7 dpi fetal brains exposed to a high dose of IAV. **F** Representative coronal sections of the meninges and choroid plexus from each treatment group of Iba1 and CD206 co-stained E16.5 fetal brains. Scale bars = 100 µm. **G**, **H** Separation of choroid plexus and meningeal BAMs showed that only **G** meningeal BAMs were increased at 7 dpi. **I** Evaluation of E16.5 brain macrophage proliferation via Ki67 and Iba1 co-staining revealed no changes in double-positive cells in the **J** whole brain, **K** parenchyma, **L** meninges, or **M** choroid plexus. Arrows = representative co-staining; scale bars = 100 µm. IAV = influenza A virus, dpi = days post-inoculation, E = embryonic day, MFI = mean fluorescence intensity, T = telencephalon, D = diencephalon, MS = mesencephalon, MT = metencephalon MY = myencephalon, Con = saline control, X31_mod_ = IAV-X31 10^3^ TCID_50_, X31_hi_ = IAV-X31 10^4^ TCID_50_. Groups were compared using one-way ANOVA with Tukey post hoc for multiple comparisons. For data containing residuals with unequal variance, Brown-Forsythe and Welch’s ANOVA with Dunnett T3 post hoc multiple comparisons was used. Data are means ± SEM; *p < 0.05, **p < 0.01; dots represent one representative fetus per litter; n = 9–14 per treatment group. See Supplementary Table S11 for complete statistical analysis of all data collected for this figure (individual mean ± SEM per group, p-values, hypothesis test used, and test statistic).

While some genetic mutations may underly cortical malformations [83, 84], exactly what leads to perturbations in neocortical development during prenatal inflammation remains undetermined. Several studies implicate fetal microglia in MIA-related pathology [35, 40, 85–88]. Here, we hypothesized that an IAV immune insult would redirect microglia from their normal neurotrophic support functions, leading to neocortical abnormalities. To differentiate between microglia and BAMs, we co-stained fetal brain sections with CD206 and Iba1 at E11.5 and 16.5 (Fig. 5A, F, Supplementary Table S11). We observed no differences in number of microglia (CD206^-^Iba1^+^) across the whole brain at 2 (Fig. 5B) or 7 dpi (Fig. 5D). When assessed within and across specific fore-, mid-, and hind-brain regions (E11.5; Fig. S7E) or between the left and right hemispheres (E16.5; Fig. S7F), microglia density remained unchanged.

Little is known about BAMs under homeostatic conditions, and even less is known about their function during embryonic development [89]. However, these transcriptionally distinct macrophages migrate from the yolk sac around the same time as microglia and take up residence at the brain’s borders [90, 91]. Therefore, it is possible they also play a role in neuronal development. In line with this hypothesis, we observed an increase in BAMs (CD206^+^Iba1^+^) in fetal brains from X31_hi_ dams (Fig. 5E), which manifested at 7 dpi but was not apparent at 2 dpi (Fig. 5C, S7G). This observation was not restricted to a specific hemisphere (Fig. S7H). Previous studies have found increased trafficking of BAMs into the embryonic choroid plexus (ChP) during MIA [36]. While we did not observe a difference in the number of ChP BAMs (Fig. 5H), numbers of meningeal BAMs were significantly increased in fetal brains from high-dose IAV dams (Fig. 5G). Interestingly, *Mrc1*, the gene encoding for CD206, was not altered in our RNA sequencing dataset (adj. p = 0.88).

We then assessed whether or not prenatal IAV infection altered the proliferative and/or phagocytic capacity of brain-resident macrophages (Iba1^+^ cells), as seen in other MIA models [35, 36, 88, 92]. Cell proliferation, as measured by co-expression with nuclear Ki67 (Fig. 5I), was not altered in the whole brain at E16.5, 7 dpi (Fig. 5J). No changes were found when the brain was further partitioned into parenchymal, meningeal, and choroid plexus Iba1^+^Ki67^+^ brain macrophages, which serve as an approximation of microglia, meningeal BAMs, and ChP BAMs, respectively (Fig. 5K–M) [36]. Furthermore, the fluorescence intensity of Ki67-positive staining did not differ, indicating that overall cell proliferation was unchanged by prenatal IAV infection (Supplementary Table S11).

**Fig. 6:**
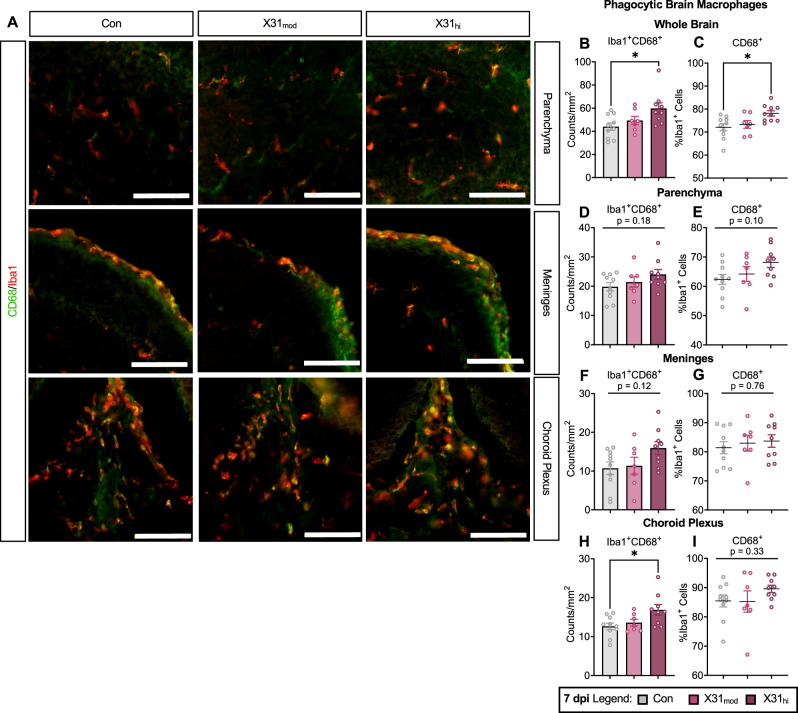
Brain-resident macrophages exhibit elevated levels of phagocytic markers in fetal brains from high-dose IAV dams. **A** Representative coronal sections of E16.5 fetal brains stained with Iba1 (red) and CD68 (green). Scale bars = 100 µm. Brain-resident macrophages (Iba1^+^ cells) showed increased phagocytic capacity in X31_hi_ fetal brains when evaluating **B** total counts of Iba1^+^CD68^+^ cells per mm^2^ and **C** percentage of Iba1^+^ cells that were also CD68^+^. **D**–**G** Parenchymal Iba1^+^ cells, which approximate microglia, and meningeal Iba1^+^ cells (meningeal BAMs) did not demonstrate a dose-dependent increase in phagocytic capacity. **H** Elevated counts of Iba1^+^CD68^+^ choroid plexus BAMs did not persist when evaluated as a **I** percentage of Iba1^+^ cells that were also CD68^+^. IAV = influenza A virus, dpi = days post-inoculation, E = embryonic day, BAM = border-associated macrophage, Con = saline control, X31_mod_ = IAV-X31 10^3^ TCID_50_, X31_hi_ = IAV-X31 10^4^ TCID_50_, Groups were compared using one-way ANOVA with Tukey post hoc for multiple comparisons. Data are means ± SEM; * = p < 0.05; dots represent one representative fetus per litter; n = 9–10 per treatment group. See Supplementary Table S11 for complete statistical analysis of all data collected for this figure (individual mean ± SEM per group, p-values, hypothesis test used, and test statistic).

In contrast, phagocytic capacity, as measured by Iba1^+^ co-expression with lysosomal CD68 (Fig. 6A), was increased in X31_hi_ fetal brains at E16.5 (Fig. 6B, C**;** represented by total double-positive cell counts per area and as a percentage of single-positive Iba1 cells). Analysis of Iba1^+^CD68^+^ cell numbers in the parenchyma and meninges revealed no differences across the three treatment groups (Fig. 6D, F); however, independent t-tests comparing Con and X31_hi_ indicate high-dose IAV-induced increases in CD68 co-expression only in the meninges (Fig. S7I, J). Interestingly, overall counts of Iba1^+^CD68^+^ BAMs increased in the choroid plexus (Fig. 6H) despite no difference in the abundance of Iba1^+^CD206^+^ BAMs (Fig. 5H). To determine whether these observations were due to increased overall cell number versus phagocytic CD68 expression, we evaluated double-positive cells as a percentage of single-positive Iba1 cells. Comparison of parenchymal Iba1^+^ cells across three treatment groups did not reach significance (Fig. 6E), while independent t-tests comparing Con and X31_hi_ groups indicate high-dose increases in the percent of Iba1 cells positive for CD68 (Fig. S7I). Conversely, meningeal Iba1^+^ cells did not differ in CD68 expression (Figs. 6G, S7J), indicating the Iba1^+^CD68^+^ increase is likely due to elevation in overall numbers of meningeal BAMs as seen in Fig. 5G. Lastly, the increase in Iba1^+^CD68^+^ choroid plexus BAMs does not coincide with elevated CD68 expression as a percentage of Iba1^+^ cells (Figs. 6I, S7K). Further investigation is needed to determine whether this discrepancy reflects a biologically relevant feature of Iba1^+^ cell distribution in the embryonic ChP following IAV infection. Overall, these data demonstrate consistent changes in BAM numbers and potential phagocytic capacity in the developing brain during late-stage IAV infection, with few changes in resident parenchymal Iba1^+^ microglia.

## Discussion

The United States has seen a significant increase in the incidence of NDDs over the past two decades [93]. As genetic risk factors comprise a small percentage of NDD etiologies, it is important to evaluate additional risk factors. In the 1990s, S.H. Fatemi and colleagues developed a mouse model of prenatal exposure to a neurotropic strain of IAV to evaluate the epidemiological link between IAV and offspring NDDs [12–14]. Since then, the majority of animal studies have used immunostimulants to identify potential mechanisms of gestational maternal inflammation. In this study, we use a non-neurotropic strain of IAV that recapitulates seasonal influenza infections in humans [94]. Critically, our work reveals that an infection severity threshold exists in tissues downstream of the site of infection, which is a concept that is also evident in poly I:C MIA models [95, 96] and reflects differences in disease severity seen in seasonal influenza outbreaks. While IAV dose did not impact maternal lung lesions or viral transcripts, it played a significant role in fetal brain abnormalities, which corroborates our previous findings indicating protection of the fetal brain during moderately pathogenic IAV infection [24]. IAV infection severity also dictated downstream maternal intestinal immune dysfunction, which coincided with mild but significant colonic T_H_17 cell suppression.

IAV infection can trigger adaptive immune responses that arise from a disruption in endogenous microbes, which appear to be at the crux of IAV-induced intestinal inflammation (as reviewed in [97]). Gastroenteritis-like symptoms, such as colonic shortening and shifts in the intestinal microbiome, are often evident during respiratory IAV infection even though the virus does not infect intestinal tissue [65]. Importantly, microbe depletion prior to IAV inoculation has been shown to diminish IAV-induced IL-17A production and intestinal injury [33]. Overall, the evidence suggests that microbial disruption precedes intestinal inflammation during respiratory IAV infection, leading to the differentiation of naïve CD4^+^ cells into pathogenic T_H_17 cells [60]. More importantly, infection-induced dysbiosis is a clinically relevant phenotype that is not fully recapitulated in poly I:C MIA models, wherein activation of pre-existing homeostatic T_H_17 cells, following poly I:C recognition by dendritic cells, precedes poly I:C-induced gut dysbiosis [20, 28, 98]. While our data indicate persistent colonic shortening throughout IAV replication and changes in microbial composition, we observe reduced production of IL-17 and lower numbers of colonic T_H_17 cells post-IAV infection. Although this suggests a deviation from previously published observations with highly pathogenic IAV strain PR8 [33], it is possible that IL-17 production could be elevated at some point between 2 and 7 dpi in our X31 model, or that the more moderately pathogenic strain X31 does not induce severe intestinal immune shifts comparable to PR8. It is also possible that pregnancy-specific immune responses to respiratory IAV infection (as reviewed in [99]) also lead to a unique intestinal immune phenotype following respiratory IAV infection, although this remains to be tested.

In poly I:C-induced MIA models, pharmacological inhibition and genetic knockdowns of maternal IL-17A are sufficient to halt the development of offspring cortical patches and behavioral deficits [29]. These studies found that offspring abnormalities are also absent in poly I:C-challenged animals that do not harbor SFB, and thus do not have sufficient populations of T_H_17 cells to mount an IL-17 response [28, 30]. A recent study contradicted these findings and determined that SFB is not necessary to induce behavioral abnormalities in offspring [64]. Similarly, we observe cortical abnormalities in our model of IAV-induced MIA despite persistent downregulation of intestinal IL-17 responses and SFB transcripts. Notably, while anti-viral IL-17 responses in maternal lungs were within normal ranges for IAV infection, circulating IL-17 levels remained low and did not differ between groups, which is consistent with our previous findings [24] and similar to other IAV studies [33]. This suggests that the alterations in neocortical development observed in our model are inducible via alternative, and potentially IL-17-independent, mechanisms. While further testing is required to parse this out, it is important to note the consistent elevation in IL-6 across maternal tissue types (lung, serum, intestine) in our model. Indeed, the idea that maternal IL-6 mediates offspring neurodevelopmental abnormalities predates the more recent interest in IL-17A. Early poly I:C-induced MIA studies revealed that pharmacological inhibition and genetic depletion of IL-6 prevented certain aspects of behavioral deficits in offspring [21]. Genetic ablation of the IL-6 receptor on placental trophoblasts in poly I:C-challenged dams prevented offspring behavioral abnormalities and neuropathologies [23]. Recent studies further bolster the idea that IL-6 is capable of directly mediating brain development via prenatal programming of synaptogenesis (as seen in mice) and via dysregulation of radial glia (as seen in human brain organoids) [27, 100]. There is also strong evidence documenting the negative effects of anti-viral interferon signaling on placental and fetal development during maternal infection [101]. The type I and II interferon signaling patterns observed in our model may be acting via similar mechanisms. Therefore, it is possible that maternal IL-6 and/or IFN signaling—rather than IL-17—could be driving downstream intestinal inflammation and disrupted fetal neocortical phenotypes in our model.

Alterations in neocortical development can have life-long negative consequences for behavior and neural processing, and disordered cortical lamination is common in NDDs [102]. Here, we observed a reduction in superficial SATB2^+^ neurons in the neocortex of E16.5 fetuses exposed to high but not moderate dose maternal IAV infection. Several mimetic-induced MIA models report the same reduction in fetal SATB2^+^ neurons and improper cortical layering of SATB2 and TBR1 before the onset of behavioral deficits [29, 70, 72, 103], suggesting that this phenomenon is conserved across disparate models of MIA. The importance of SATB2 can be appreciated when observing patients with genetic mutations and deletions of *Satb2*. This SATB2-associated syndrome causes a myriad of complications, including autistic-like behavior and speech impairments [104]. Interestingly, influenza vaccination in early pregnancy has been shown to prevent a reduction in embryonic SATB2 in a rodent model of LPS-induced MIA [72]. While it stands to reason that maternal vaccination against IAV before infection would similarly protect against SATB2 abnormalities in our model, this still needs to be tested. Although others have proposed that upregulation of IL-17 receptors on embryonic neurons precedes SATB2 abnormalities [71], we observed no differences in IL-17 receptor transcripts in the embryonic brain. Overall, SATB2^+^ neurons appear to be conserved cellular targets of maternal inflammation and are likely disrupted through an IL-17-independent mechanism in our model.

Our RNA-seq data from X31_hi_ fetal brains bolsters the evidence for altered cortical lamination. For instance, we see a reduction in genes *Apc2, Mdga1*, and *Usp11*. Genetic knockout or loss of function studies performed on each of these genes reveal their importance in regulating early neuronal layering in the murine cortex. Knockout of *Apc2* leads to improper cortical lamination of TBR1^+^ neurons at E16.5 [78]. Knockout of *Usp11*, which encodes a protein responsible for cortical neurogenesis and migration, leads to reduced migration of neuronal progenitors into superficial layers (e.g., reduction in SATB2 in layers II-IV) [105]. Loss of function of *Mdga1* indicated that this gene is critical for radial migration of upper layer neurons [79]. Overall, the downregulation of these genes, among others, supports the idea that severe maternal IAV infection disrupts processes related to neocortical formation and neuron migration.

As cortical neurogenesis precedes synaptogenesis [38], alterations in cortical lamination are often accompanied by dysregulated synaptic signaling [106, 107]. The observed upregulation in glutamatergic synapse-related genes in our model corroborates evidence from a recent study demonstrating that direct intracerebroventricular injection of IL-6 into the embryonic brain elevates genetic programs of synaptogenesis in cortical glutamatergic neurons [27]. Poly I:C-induced MIA studies also describe upregulated glutamatergic synapse density in cortical upper layer [108] and deep layer [109] neurons. Critically, our data are also in alignment with clinical studies that demonstrate dysregulation in both cortical lamination and synapse development and function (e.g., excitatory/inhibitory imbalance) in patients with NDDs [102, 110]. Future studies are needed to determine whether IAV-induced changes in fetal brain organization and developmental processing persist postnatally and whether they correspond with NDD-related behaviors.

We hypothesized that microglia and BAMs would be prime cellular candidates for orchestrating cortical pathologies. At the time of our maternal IAV challenge, yolk-sac-derived microglia are migrating to the embryonic brain where they aid in critical neurotrophic support functions including cortical development [39, 40] and cortical synaptic formation [111]. The role microglia play in NDDs is debated; however, numerous studies report that microglia are impacted by MIA [37, 87, 112]. While our histological analysis of embryonic microglia indicates that their migration patterns and proliferation are not altered by maternal IAV infection, the percent of CD68^+^ parenchymal Iba1 cells is increased in the high-dose IAV group compared to controls. Overall counts of CD68^+^ Iba1 cells were also increased across the whole fetal brain, indicating a pro-phagocytic shift in the brain-resident macrophage population. Moving forward, functional assays are needed to confirm whether microglial/macrophage activities are shifted in the immediate days following infection. Indeed, microglial density and proliferative capacity may not directly correlate with phagocytic function nor with the production of neurotrophic or inflammatory mediators, each of which has been shown to modulate critical neurodevelopmental processes (as reviewed in [20]). Furthermore, a limitation of our study is the use of one randomly selected fetus per litter, which does not represent potential within-litter variability or sex differences [113]. While MIA-induced sex differences are most apparent in offspring behavioral outcomes, several studies have also shown sex-specific differences in placental transcripts [114] and in offspring microglial outcomes [115, 116]. Therefore, parsing out sex differences in our model will be an important next step.

Interestingly, the dose-dependent increased density of BAMs indicates a macrophage-specific response to IAV-induced maternal inflammation. While BAMs do not infiltrate the brain parenchyma to interact with early neuronal cells like microglia do, they are in direct contact with the periphery and thus play a critical role in immune defense [90, 117]. At least one study demonstrates that poly I:C-induced MIA directly impacts the homeostatic function of embryonic brain macrophages, leading to increases in ChP macrophage populations [36]. These authors propose that an accumulation of macrophages at the ChP and ventricular zone indirectly contributes to the cortical malformations observed in their model, whereby increased recruitment of phagocytes into the brain parenchyma disrupts neural progenitor proliferation at the cortex [36]. Direct examination of this proposed mechanism, however, is still needed. Notably, we observed an increased abundance of CD206^+^ macrophages in the meninges, concomitant with increased expression of Iba1^+^CD68^+^ cells in X31_hi_ fetuses compared to control. Interestingly, despite indications that CD68 expression may also increase in ChP BAMs in high-dose IAV fetuses, overall numbers of CD206^+^ macrophages did not differ in this region, which contrasts with recent poly I:C findings [36]. To our knowledge, no study has specifically evaluated embryonic meningeal BAMs following maternal inflammation, nor has any study compared BAM subsets in this context. Further work is needed to elucidate the role meningeal and ChP BAMs might play in regulating prenatal neuronal patterning following maternal IAV infection and the mechanisms by which they might fulfill these roles.

Taken together, our data suggest that IAV-induced cortical malformations and altered macrophage populations in the embryonic brain arise independently of maternal IL-17 signaling at 2 and 7 dpi. Importantly, we demonstrate that a higher infectious dose of IAV is necessary to induce downstream changes in the maternal intestine and the fetal brain, further bolstering our previous findings [24]. Overall, the discrepancies and similarities we observed between our model of live IAV-induced MIA and models of mimetic-induced MIA highlight the importance of using live pathogens to evaluate the complete immune response and to improve translation to the clinic. Understanding these model differences is critical for effectively delineating translationally relevant NDD etiologies and determining susceptibility and resiliency of offspring to developing NDDs following MIA [118].

## Supplementary information

Supplemental Figures

Supplemental Table S1

Supplemental Table S2

Supplemental Table S3

Supplemental Table S4

Supplemental Table S5

Supplemental Table S6

Supplemental Table S7

Supplemental Table S8

Supplemental Table S9

Supplemental Table S10

Supplemental Table S11

MIA Reporting Guidelines

## Data Availability

Bulk RNA-Sequencing data were deposited into the Gene Expression Omnibus (GEO) database under the accession number GSE262291 and are available at the following URL: https://www.ncbi.nlm.nih.gov/geo/query/acc.cgi?acc=GSE262291.
