# Supplementary material for: Influenza A virus during pregnancy disrupts maternal intestinal immunity and fetal cortical development in a dose- and time-dependent manner
Source: Mol Psychiatry. 2024 Jul 3;30(1):13–28. doi: 10.1038/s41380-024-02648-9 (PMC11649561; doi:10.1038/s41380-024-02648-9)
Supplement: Supplementary file 1 — Supplemental Figures [file 41380_2024_2648_MOESM1_ESM.pdf]

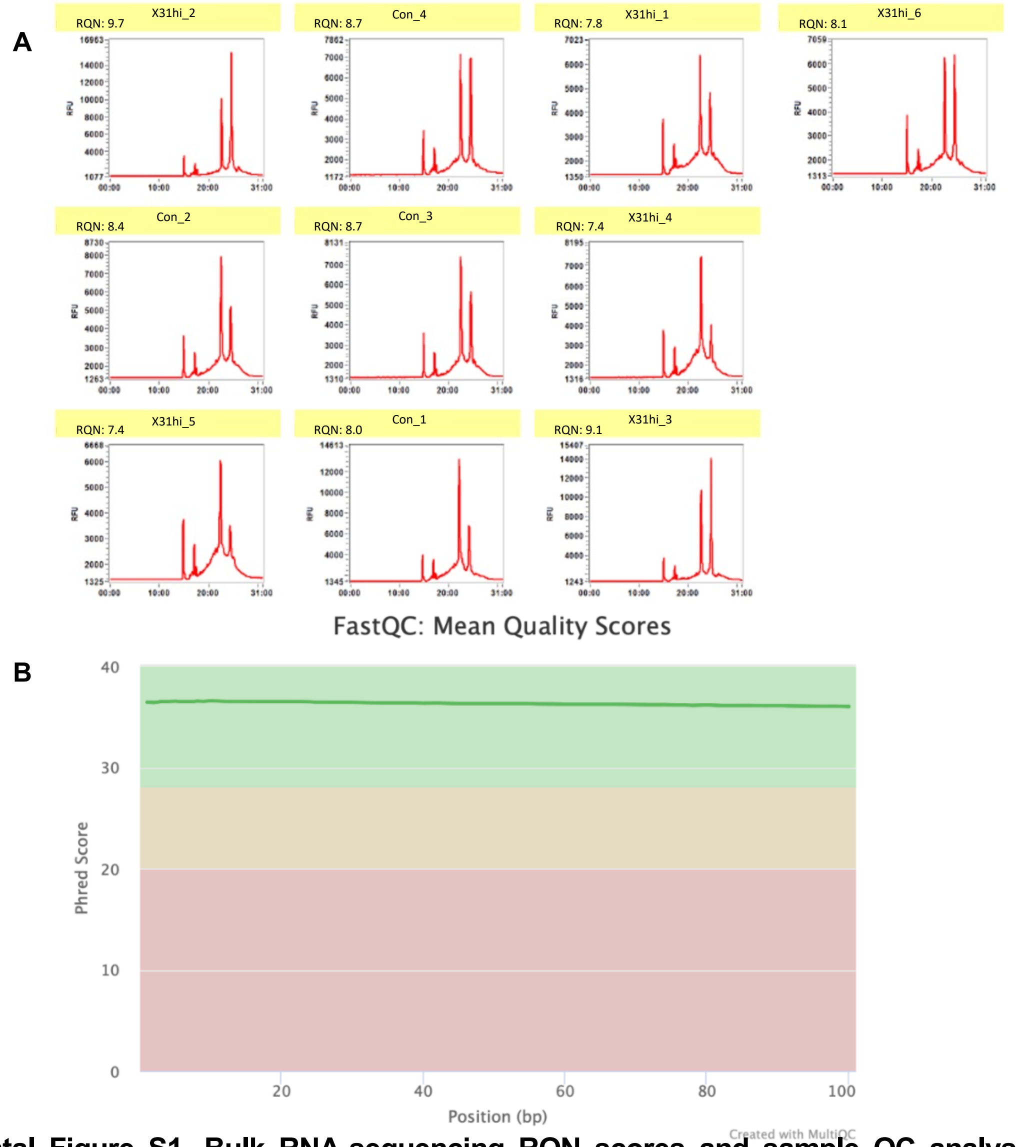

Supplemental Figure S1. Bulk RNA-sequencing RQN scores and sample QC analysis. (A) RNA quality scores (RQN) for each cDNA sample prior to sequencing. **(B)** FASTQC on individual samples summarized by MultiQC v 1.9 showing average per-base read quality scores over 30 in all samples. *Con* = saline control, $X31_{hi}$ = IAV-X31 10^4 $TCID_{50}$; n = 4-6 per treatment group.

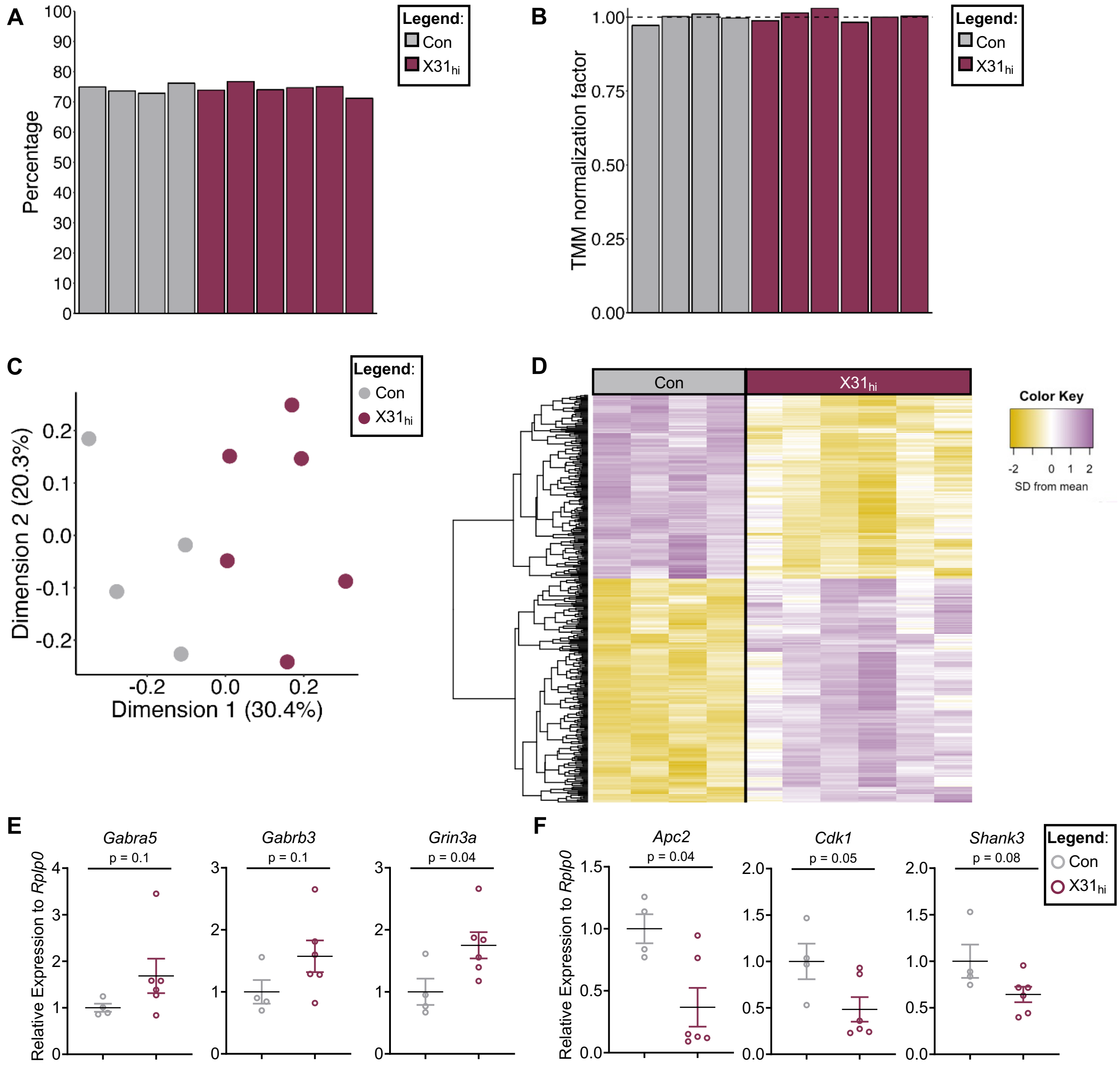

A
Percentage
Legend:
Con
X31hi
B
TMM normalization factor
Legend:
Con
X31hi
C
Dimension 2 (20.3%)
Dimension 1 (30.4%)
Legend:
Con
X31hi
D
Con
X31hi
Color Key
SD from mean
E
Relative Expression to Rplp0
Gabra5
p = 0.1
Gabrb3
p = 0.1
Grin3a
p = 0.04
F
Relative Expression to Rplp0
Apc2
p = 0.04
Cdk1
p = 0.05
Shank3
p = 0.08
Legend:
Con
X31hi

Supplemental Figure S2. Bulk RNA-sequencing alignment and differential gene expression in E16.5 fetal brains. (A) The percentage of reads mapped to the transcriptome. **(B)** Normalization of samples using the trimmed mean of M values (TMM) method. **(C)** Multidimensional clustering of the top 5,000 most variable genes after removing the effects of 4 RUV factors. Dimension 1 explains 30.4% of the variation. **(D)** Visualization of differential gene expression showing 211 upregulated genes and 173 downregulated genes when comparing fetal brains from Con and $X31_{hi}$ dams (Benjamini-Hochberg FDR correction, $p < 0.1$). **(E-F)** qPCR confirmation of **(E)** upregulated synaptic signaling genes and **(F)** downregulated neuronal development genes. *IAV* = influenza A virus, *E* = embryonic day, *RUV* = remove unwanted variance, *FDR* = false discovery rate, *Con* = saline control, $X31_{hi}$ = IAV-X31 10^4 $TCID_{50}$; n = 4-6 per treatment group.

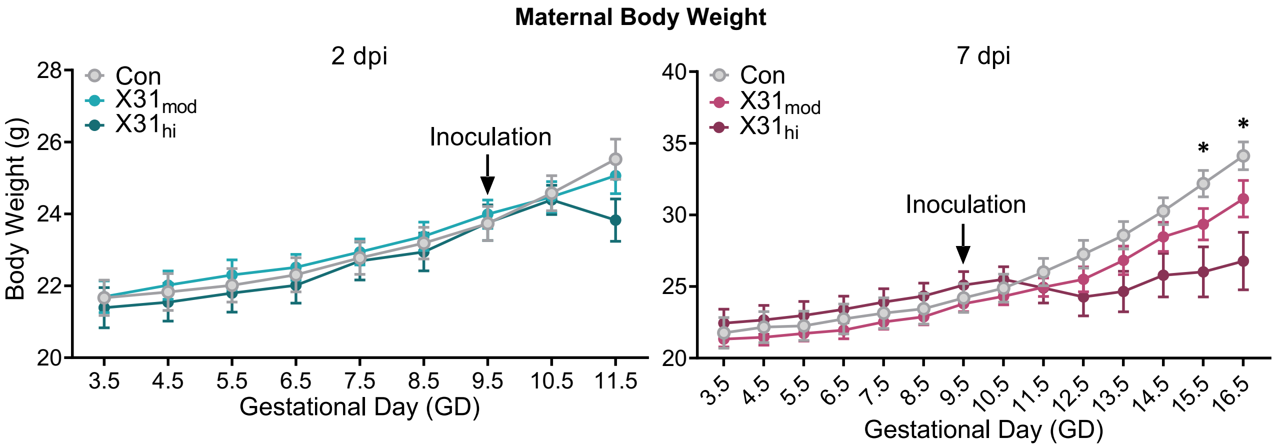

Supplemental Figure S3. Maternal body weight at 2 and 7 dpi. Maternal body weight was significantly different between control and IAV-X31$_{hi}$ dams starting at GD15.5, 6 dpi (repeated measures two-way ANOVA, the main effect of time = $p < 0.001$; * = Con vs X31$_{hi}$). *IAV* = influenza A virus, *dpi* = days post-inoculation, *Con* = saline control, *X31$_{mod}$* = IAV-X31 10^3 TCID$_{50}$, *X31$_{hi}$* = IAV-X31 10^4 TCID$_{50}$; n = 9-14 per treatment group.

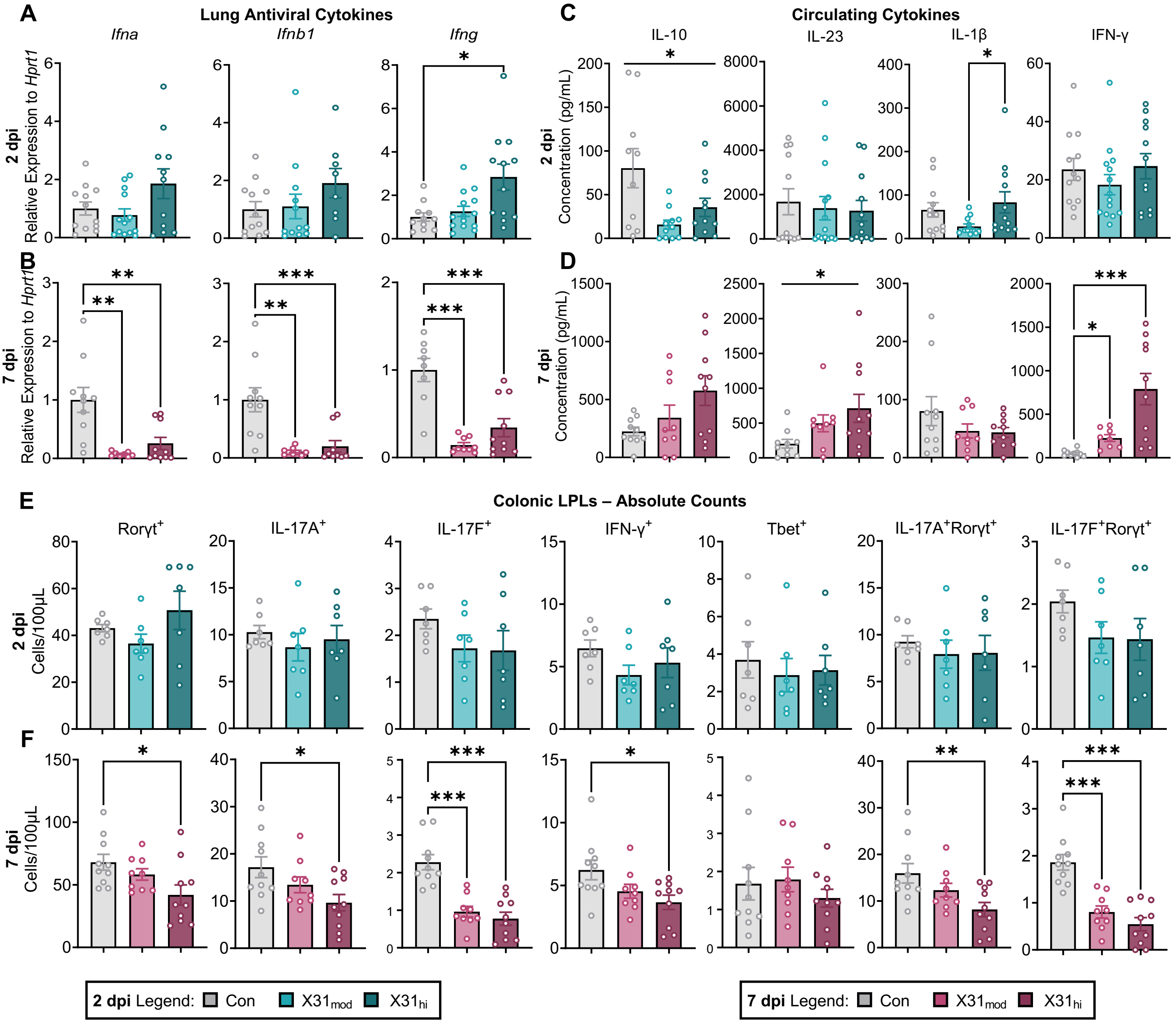

A
Lung Antiviral Cytokines
Ifna
Ifnb1
Ifng
2 dpi
Relative Expression to Hprt1
B
7 dpi
Relative Expression to Hprt1
C
Circulating Cytokines
IL-10
IL-23
IL-1β
IFN-γ
2 dpi
Concentration (pg/mL)
D
7 dpi
Concentration (pg/mL)
E
Colonic LPLs – Absolute Counts
Rorγt+
IL-17A+
IL-17F+
IFN-γ+
Tbet+
IL-17A+Rorγt+
IL-17F+Rorγt+
2 dpi
Cells/100μL
F
7 dpi
Cells/100μL
2 dpi Legend: Con X31mod X31hi
7 dpi Legend: Con X31mod X31hi

Supplemental Figure S4. Maternal lung interferons, circulating cytokines, and absolute counts of colonic T cells at 2 and 7 dpi. (A-B) The antiviral gene encoding for IFN-γ was upregulated in the maternal lung at **(A)** 2 dpi in $X31_{hi}$ dams only. **(B)** At 7 dpi, there was a significant decrease in type I and type II interferons in the IAV-infected maternal lung. **(C-D)** A 13-plex mouse inflammation panel (LEGENDplex) was performed on maternal serum at **(C)** 2 dpi and **(D)** 7 dpi. Type II antiviral interferon, IFN-γ, was upregulated in a dose-dependent manner at 7 dpi only. IL-23, an inflammatory cytokine and a molecule required for the commitment of pathogenic T_H17 cells, was upregulated at 7 dpi. Anti-inflammatory cytokine IL-10 was significantly different amongst treatment groups at 2 dpi. **(E-F)** Absolute cell counts via flow cytometry of colonic LPLs showed no changes at **(E)** 2 dpi and downregulation in everything except Tbet at **(F)** 7 dpi. *IAV* = influenza A virus, *dpi* = days post-inoculation, *LPL* = lamina propria lymphocytes, *Con* = saline control, $X31_{mod}$ = IAV-X31 10^3 $TCID_{50}$, $X31_{hi}$ = IAV-X31 10^4 $TCID_{50.}$ Groups were compared with one-way ANOVA with Tukey post hoc for multiple comparisons. For data containing residuals with unequal variance, Brown-Forsythe and Welch's ANOVA with Dunnett T3 post hoc multiple comparisons was used. For non-parametric data, Kruskal-Wallis ANOVA with Dunn's correction for multiple comparisons was used. Data are means ± SEM; * = $p < 0.05$, ** = $p < 0.01$, *** = $p < 0.001$; dots represent individual dams; n = 9-14 per treatment group. See Supp. Table S4-6 for complete statistical analysis of all data collected for this figure (individual mean ± SEM per group, p-values, hypothesis test used, and test statistic).

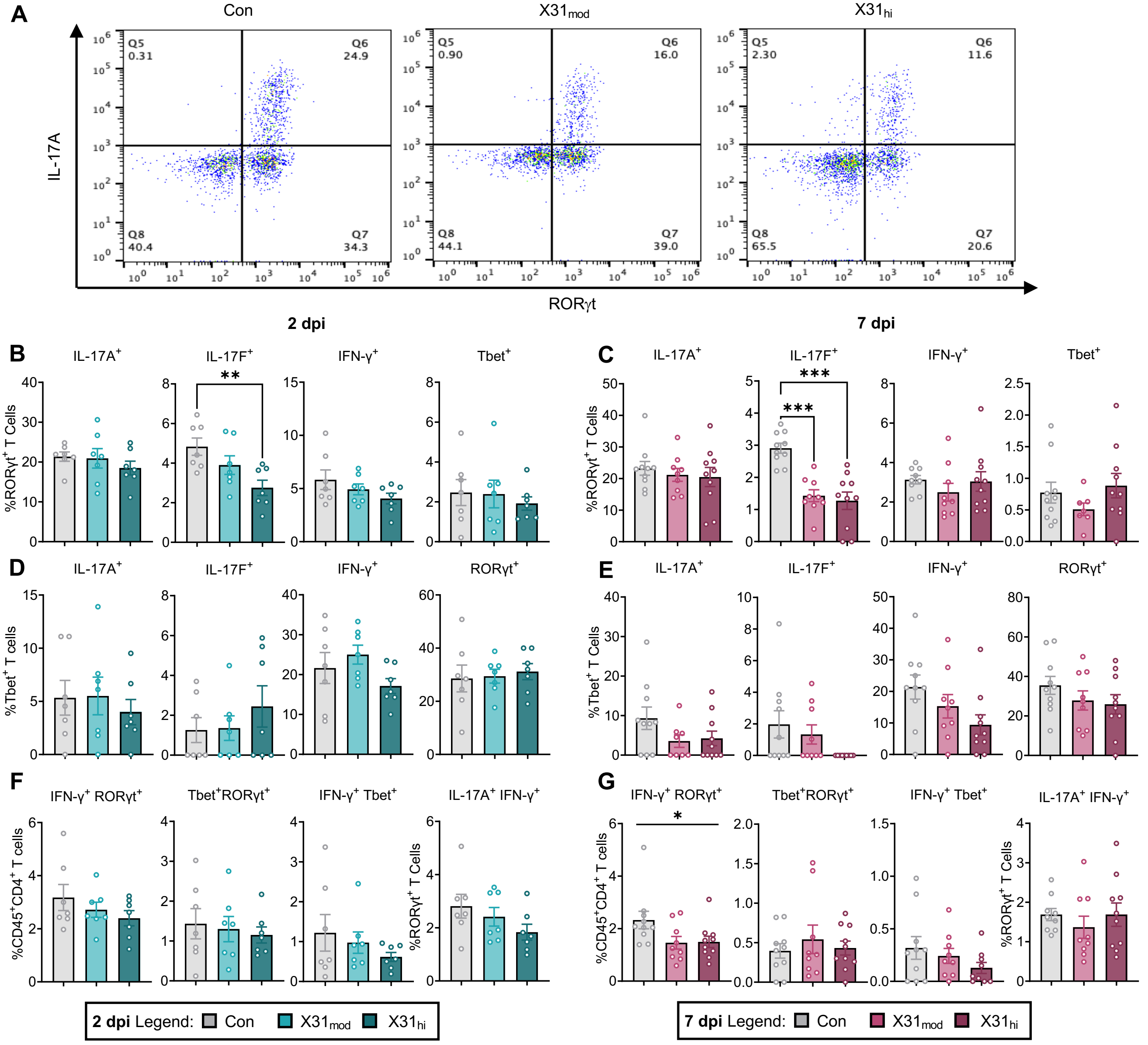
A
Con
X31mod
X31hi
Q5 0.31
Q6 24.9
Q8 40.4
Q7 34.3
Q5 0.90
Q6 16.0
Q8 44.1
Q7 39.0
Q5 2.30
Q6 11.6
Q8 65.5
Q7 20.6
IL-17A
RORγt
2 dpi
7 dpi
B
C
D
E
F
G
IL-17A+
IL-17F+
IFN-γ+
Tbet+
RORγt+
%RORγt+ T Cells
%Tbet+ T cells
%CD45+CD4+ T cells
%RORγt+ T Cells
IFN-γ+ RORγt+
Tbet+RORγt+
IFN-γ+ Tbet+
IL-17A+ IFN-γ+
**
***
*
2 dpi Legend: Con X31mod X31hi
7 dpi Legend: Con X31mod X31hi

Supplemental Figure S5. Maternal colonic T_H17 and T_H1 cells at 2 and 7 dpi. (A) Representative flow cytometry dot plots at 7 dpi of $CD45^+CD4^+$ colonic LPLs quadrant gated on IL-17A and RORγt expression. Q6 represents double positive cells. **(B-C)** $CD45^+CD4^+ROR\gamma t^+$ (T_H17 cells) at **(B)** 2 and **(C)** 7 dpi gated on IL-17A, IL-17F, IFN-γ, and Tbet. There is a decrease in IL-17F at both time points. **(D-E)** $CD45^+CD4^+Tbet^+$ (T_H1 cells) at **(D)** 2 and **(E)** 7 dpi gated on IL-17A, IL-17F, IFN-γ, and Rorγt. **(F-G)** Additional quadrant gating on $CD45^+CD4^+$ and $CD45^+CD4^+ROR\gamma t^+$ T cells at **(F)** 2 and **(G)** 7 dpi. *IAV* = influenza A virus, *dpi* = days post-inoculation, , *LPL* = lamina propria lymphocytes, *Con* = saline control, $X31_{mod}$ = IAV-X31 10^3 $TCID_{50}$, $X31_{hi}$ = IAV-X31 10^4 $TCID_{50}$. Groups were compared with one-way ANOVA with Tukey post hoc for multiple comparisons. For data containing residuals with unequal variance, Brown-Forsythe and Welch's ANOVA with Dunnett T3 post hoc multiple comparisons was used. For non-parametric data, Kruskal-Wallis ANOVA with Dunn's correction for multiple comparisons was used. Data are means ± SEM; * = $p < 0.05$, ** = $p < 0.01$, *** = $p < 0.001$; dots represent individual dams; n = 7-10 per treatment group. See Supp. Table S6 for complete statistical analysis of all data collected for this figure (individual mean ± SEM per group, p-values, hypothesis test used, and test statistic).

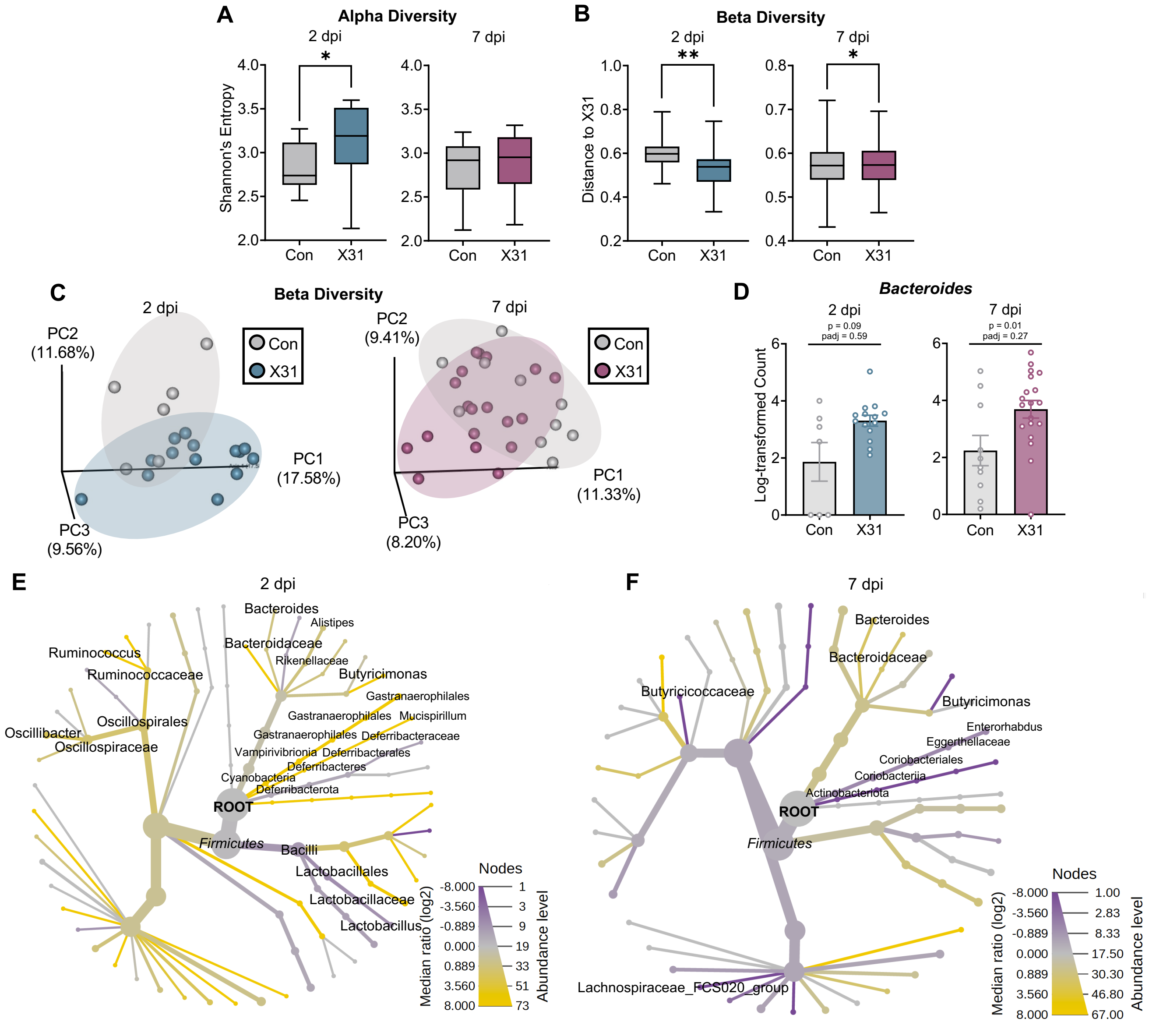
A
Alpha Diversity
2 dpi
7 dpi
Shannon's Entropy
Con
X31
B
Beta Diversity
Distance to X31
C
PC1 (17.58%)
PC2 (11.68%)
PC3 (9.56%)
PC1 (11.33%)
PC2 (9.41%)
PC3 (8.20%)
D
Bacteroides
p = 0.09
padj = 0.59
p = 0.01
padj = 0.27
Log-transformed Count
E
F
ROOT
Firmicutes
Bacteroides
Alistipes
Bacteroidaceae
Rikenellaceae
Butyricimonas
Ruminococcus
Ruminococcaceae
Oscillospirales
Oscillibacter
Oscillospiraceae
Gastranaerophilales
Mucispirillum
Deferribacteraceae
Vampirivibrionia
Deferribacterales
Deferribacteres
Cyanobacteria
Deferribacterota
Bacilli
Lactobacillales
Lactobacillaceae
Lactobacillus
Butyricicoccaceae
Enterorhabdus
Eggerthellaceae
Coriobacteriales
Coriobacteriia
Actinobacteriota
Lachnospiraceae_FCS020_group
Nodes
Median ratio (log2)
Abundance level

Supplemental Figure S6. Respiratory IAV infection alters the maternal colonic microbiome. (A) Alpha diversity (within-sample diversity) was different between Con and X31 dams at 2 dpi only. **(B)** Beta diversity (between-sample diversity) was different between Con and X31 dams at 2 and 7 dpi. **(C)** PCA based on unweighted UniFrac distance metrics. The ovals in the figures do not represent statistical significance but serve as a visual guide to group differences. **(D)** While centered-log ratio analysis did not reveal a statistical increase in *Bacteroides* in IAV-infected dams at either time-point (DESeq2, padj threshold <0.05), **(E-F)** heat-tree analysis at **(E)** 2 and **(F)** 7 dpi revealed taxonomic differences between microbial communities, including *Bacteroides* (Wilcoxon Rank Sum test; italicized = not significant). *PCA* = principal component analysis, *IAV* = influenza A virus, *dpi* = days post inoculation, *Con* = saline control, *X31* = IAV-X31 10^3 $TCID_{50}$ and IAV-X31 10^4 $TCID_{50}$. Shannon's index was used to calculate alpha diversity, and unweighted UniFrac was used to calculate beta diversity. Data are means ± SEM; * = $p < 0.05$, ** $p < 0.01$; dots represent individual dams; n = 7-10 per treatment group.

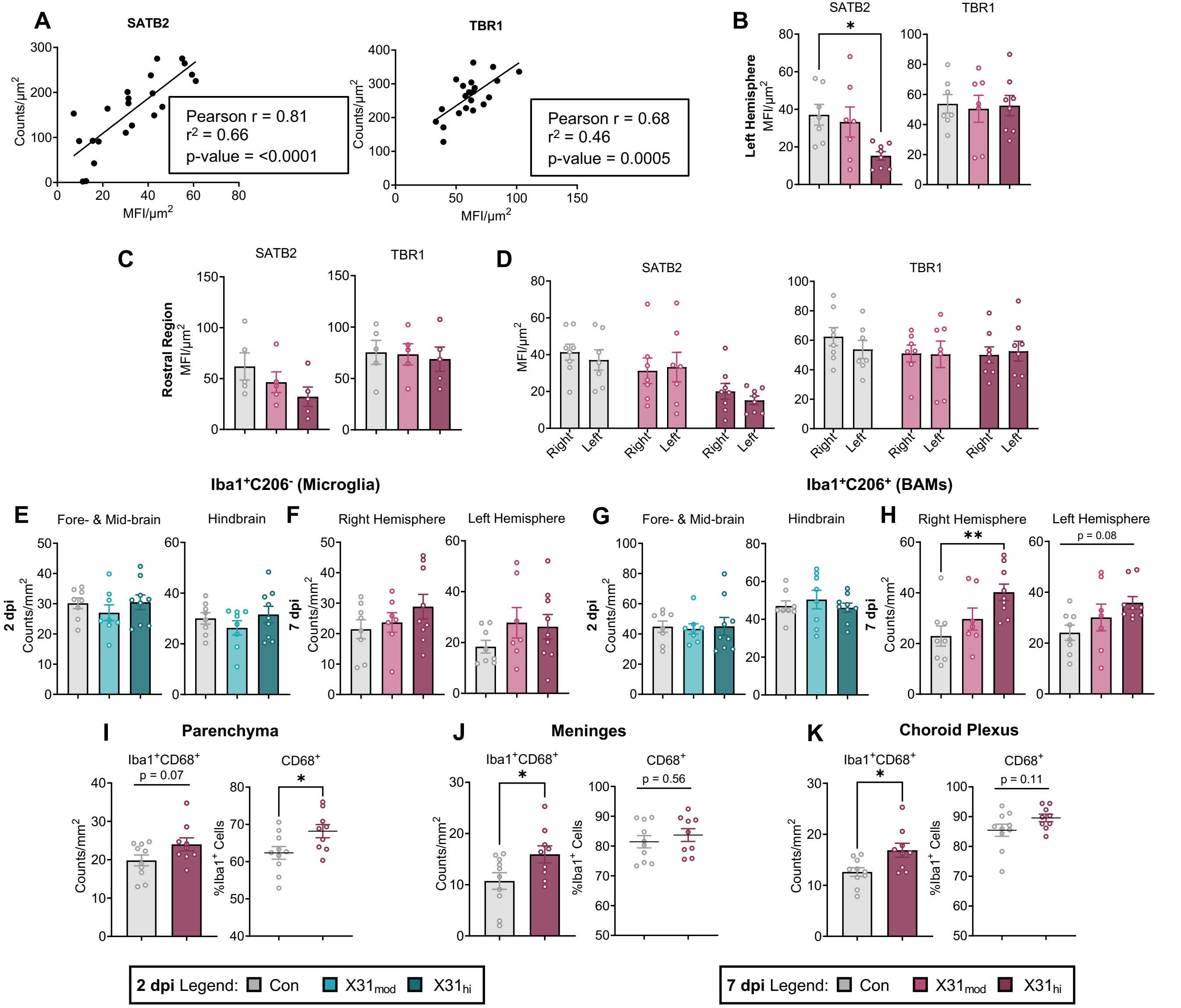

A
SATB2
Pearson r = 0.81
r2 = 0.66
p-value = <0.0001
Counts/µm2
MFI/µm2
TBR1
Pearson r = 0.68
r2 = 0.46
p-value = 0.0005
B
Left Hemisphere
C
Rostral Region
D
Right
Left
Iba1+C206- (Microglia)
Iba1+C206+ (BAMs)
E
Fore- & Mid-brain
Hindbrain
2 dpi
F
Right Hemisphere
Left Hemisphere
7 dpi
G
H
p = 0.08
I
Parenchyma
Iba1+CD68+
p = 0.07
CD68+
%Iba1+ Cells
J
Meninges
p = 0.56
K
Choroid Plexus
p = 0.11
2 dpi Legend: Con X31mod X31hi
7 dpi Legend: Con X31mod X31hi

Supplemental Figure S7. Regional analysis of E11.5 and 16.5 fetal brains. (A) Correlation between SATB2 and TBR1 MFI and cell counts. **(B)** MFI of SATB2 and TBR1 in the left hemispheres at E16.5, 7 dpi reveal a downregulation in $SATB2^+$ neurons in fetal brains from $X31_{hi}$ dams (see Fig. 3 for right hemisphere data). **(C)** Analysis of SATB2 and TBR1 expression in a rostral brain region. **(D)** No hemispheric differences within treatment groups were observed for SATB2 MFI (multiple unpaired t-test; p-value = 0.25, 0.96, and 0.54 for con, $X31_{mod}$, and $X31_{hi}$ comparisons, respectively) or TBR1 MFI (multiple unpaired T-tests; p=value = 0.07, 0.55, and 0.83 for con, $X31_{mod}$, and $X31_{hi}$ comparisons, respectively). **(E)** $Iba1^+CD206^-$ (microglia) staining of the E11.5 (2 dpi) fetal brain split into fore- and mid-brain (telencephalon, diencephalon, and mesencephalon) and hindbrain (metencephalon and myencephalon) revealed no differences. **(F)** Additionally, there were no differences in microglia count at E16.5, 7 dpi when dividing by hemisphere. **(G)** $Iba1^+CD206^+$ (BAM) staining of E11.5 fetal brains revealed no differences between brain regions. **(H)** The right hemisphere showed a slightly higher upregulation of BAMs in $X31_{hi}$ vs Con fetal brains at E16.5. **(I-K)** Unpaired t-tests between Con and $X31_{hi}$ fetal brains. **(I)** High-dose parenchymal macrophages have more $Iba1^+$ cells that co-express CD68. **(J)** Elevation in $Iba1^+CD68^+$ cells in the meninges is due to an overall increase in meningeal BAM numbers. **(K)** Elevation in $Iba1^+CD68^+$ cells in the choroid plexus (ChP) appears to be due to an increase in ChP BAMs; however, there is a trending increase in $Iba1^+$ ChP cells that co-express CD68. *IAV* = influenza A virus, *dpi* = days post-inoculation, *E* = embryonic day, *MFI* = mean fluorescence intensity, BAM = border-associated macrophages, *Con* = saline control, $X31_{mod}$ = IAV-X31 10^3 $TCID_{50}$, $X31_{hi}$ = IAV-X31 10^4 $TCID_{50}$. Groups were compared with one-way ANOVA with Tukey post hoc for multiple comparisons unless otherwise specified. For data containing residuals with unequal variance, Brown-Forsythe and Welch's ANOVA with Dunnett T3 post hoc multiple comparisons was used. Data are means ± SEM; * = $p < 0.05$, ** = $p < 0.01$; dots represent one representative fetus per litter; n = 9-14 per treatment group. See Supp. Table S8 and S11 for complete statistical analysis of all data collected for this figure (individual mean ± SEM per group, p-values, hypothesis test used, and test statistic).
