# Supplementary material for: Influenza A virus during pregnancy disrupts maternal intestinal immunity and fetal cortical development in a dose- and time-dependent manner
Source: Mol Psychiatry. 2024 Jul 3;30(1):13–28. doi: 10.1038/s41380-024-02648-9 (PMC11649561; doi:10.1038/s41380-024-02648-9)
Supplement: Supplementary file 2 — Supplemental Table S1 [file 41380_2024_2648_MOESM2_ESM.pdf]

Supplemental Table S1. Gene Primers.

Gene	Forward Sequence	Reverse Sequence
Il6	GACTGATGCTGGTGACAAC	ATCCTCTGTGAAGTCTCCTC
Tgfb1	AGCGGACTACTATGCTAAAGAGGTC	TCTCATAGATGGCGTTGTTGC
Il1b	GCACTACAGGCTCCGAGATGAAC	TTGTCGTTGCTTGGTTCTCCTTGT
Il23a	CAAGGACAACAGCCAGTTCTGCTT	AGGCTCCCCTTTGAAGATGTCAGA
Il17a	ACTACCTCAACCGTTCCA	GAGGGATATCTATCAGGGTC
Il17f	ACCAAAACCAGGGCATTTCT	CGAGTGATGTTGTAATCCCATG
Il22	CCTGACCAAACTCAGCAATC	GCCTTCTGACATTCTTCTGG
Il17ra	CTGTATGACCTGGAGGCTTTCTG	CGAGTAGACGATCCAGACCTTC
Rorc	CCTTGCAAGATCTGTGGGGAC	TGCAGGAGTAGGCCACATTACA
Tnf	GTGCCTATGTCTCAGCCTCTT	GCCATAGAACTGATGAGAGGGAG
Il15	GTAGGTCTCCCTAAAACAGAGGC	TCCAGGAGAAAGCAGTTCATTGC
Il10	GCTGAAGACCCTCAGGATGCG	CCTGCTCCACTGCCTTGCTCT
Ifna	GCAATGACCTCCATCAGCAG	GTGGAAGTATGTCCTCACAGCC
Ifnb1	GCCTTTGCCATCCAAGAGATGC	ACACTGTCTGCTGGTGGAGTTC
Ifng	GGTCAACAACCCACAGGTCC	ACTCCTTTTCCGCTTCCTGAG
Reg3b	GTTTCAGATACCACAGACCTGG	TTGAGCACAGATACGAGGTGT
Reg3g	AAAGCAGTGGTAACAGTGG	CACCTCTGTTGGGTTCATAG
Duox2	ACCCTGGACCTCTATTCAG	ACAGCCCATTCCTAGTGT
Cldn1	GCCATCTACGAGGGACTGTG	ACTAATGTCGCCAGACCTGAAA
Cldn2	TCTCTGTGGTGGGCATGAGA	CCAGCGGCGAGTAGAAGTC
Cldn5	CAGTTAAGGCACGGGTAGCA	GCCCAGCTCGTACTTCTGTG
Ocln	GAGTGAAGAGTACATGGCTGCT	TTCTCCCGCAACTGGCATC
X31 NP	AGGCACCAAACGGTCTTACG	TTCCGACGGATGCTCTGATT
*Rplp0**	GGACCCGAGAAGACCTCCTT	GCACATCACTCAGAATTTCAATGG
*Hprt1**	CAAACTTTGCTTTCCCTGGT	TCTGGCCTGTATCCAACACTTC
Eubacteria	ACTCCTACGGGAGGCAGCAGT	ATTACCGCGGCTGCTGCG
SFB	GACGCTGAGGCATGAGAGCAT	GACGGCACGGATTGTTATTCA

Forward and reverse sequences of user-designed gene primers. * = housekeeping genes.
