# Supplementary material for: Influenza A virus during pregnancy disrupts maternal intestinal immunity and fetal cortical development in a dose- and time-dependent manner
Source: Mol Psychiatry. 2024 Jul 3;30(1):13–28. doi: 10.1038/s41380-024-02648-9 (PMC11649561; doi:10.1038/s41380-024-02648-9)
Supplement: Supplementary file 3 — Supplemental Table S2 [file 41380_2024_2648_MOESM3_ESM.pdf]

**Supplemental Table S2**. Litter Characteristics at 2 and 7 dpi.

| Timepoint | Characteristic | Control | $X31_{mod}$ | $X31_{hi}$ | p-value | Test | Statistic |
|---|---|---|---|---|---|---|---|
| 2 dpi | Litter size | 7.85 ± 0.83 | 8.64 ± 0.46 | 8.83 ± 0.39 | 0.82 | K-W | H(2) = 0.40 |
| | Viable Pups | 7.31 ± 0.75 | 7.93 ± 0.49 | 7.58 ± 0.45 | 0.42 | K-W | H(2) = 1.72 |
| | Resorptions | 0.54 ± 0.18 | 0.71 ± 0.19 | 1.25 ± 0.33 | 0.18 | K-W | H(2) = 3.46 |
| 7 dpi | Litter size | 8.4 ± 0.50 | 8.56 ± 0.47 | 9.00 ± 0.37 | 0.68 | K-W | H(2) = 0.77 |
| | Viable Pups | 7.80 ± 0.49 | 8.11 ± 0.46 | 7.70 ± 0.54 | 0.69 | K-W | H(2) = 0.73 |
| | Resorptions | 0.60 ± 0.27 | 0.44 ± 0.18 | 1.30 ± 0.37 | 0.17 | K-W | H(2) = 3.51 |

No changes in total litter size, pup viability, or fetal resorptions at 2 or 7 dpi. Viable pups are resorptions subtracted from litter size. *Dpi* = days-post-inoculation, $X31_{mod}$ = IAV-X31 $10^3$ $TCID_{50}$, $X31_{hi}$ = IAV-X31 $10^4$ $TCID_{50}$. Kruskal-Wallis (K-W) ANOVA with Dunn’s correction for multiple comparisons was used. Data are means ± SEM; 2 dpi n = 12-14 and 7 dpi n = 9-10 dams per treatment group.
