# Supplementary material for: Influenza A virus during pregnancy disrupts maternal intestinal immunity and fetal cortical development in a dose- and time-dependent manner
Source: Mol Psychiatry. 2024 Jul 3;30(1):13–28. doi: 10.1038/s41380-024-02648-9 (PMC11649561; doi:10.1038/s41380-024-02648-9)
Supplement: Supplementary file 4 — Supplemental Table S3 [file 41380_2024_2648_MOESM4_ESM.pdf]

Supplemental Table S3. Lung Lesion Scores at 2 and 7 dpi.

Timepoint	Characteristic	Control	$X31_{mod}$	$X31_{hi}$	p-value	Test	Statistic
2 dpi	Bronchitis	0.08 ± 0.08	1.64 ± 0.32	2.00 ± 0.28	**0.0001**	K-W	H(2) = 20.85
	Interstitial Inflammation	0.08 ± 0.08	0.93 ± 0.27	1.25 ± 0.30	**0.006**	K-W	H(2) = 10.11
	Edema	0.00 ± 0.00	0.00 ± 0.00	0.00 ± 0.00	-	-	-
	Endothelialitis	0.00 ± 0.00	0.86 ± 0.25	1.08 ± 0.23	**0.001**	K-W	H(2) = 13.16
	Pleuritis	0.00 ± 0.00	0.00 ± 0.00	0.00 ± 0.00	-	-	-
	Thrombus Formation	0.00 ± 0.00	0.00 ± 0.00	0.00 ± 0.00	-	-	-
	Total score	0.15 ± 0.10	3.43 ± 0.82	4.33 ± 0.76	**0.0001**	K-W	H(2) = 19.60
7 dpi	Bronchitis	0.00 ± 0.00	0.89 ± 0.11	0.90 ± 0.10	**0.0001**	K-W	H(2) = 20.88
	Interstitial Inflammation	0.00 ± 0.00	2.33 ± 0.37	2.50 ± 0.31	**0.0001**	K-W	H(2) = 18.16
	Edema	0.20 ± 0.13	0.44 ± 0.18	0.20 ± 0.13	0.41	K-W	H(2) = 1.79
	Endothelialitis	0.00 ± 0.00	1.00 ± 0.17	0.90 ± 0.10	**0.0001**	K-W	H(2) = 20.01
	Pleuritis	0.00 ± 0.00	0.56 ± 0.18	0.20 ± 0.13	**0.02**	K-W	H(2) = 7.85
	Thrombus Formation	0.00 ± 0.00	0.00 ± 0.00	0.00 ± 0.00	-	-	-
	Total score	0.20 ± 0.13	5.22 ± 0.68	4.70 ± 0.60	**0.0003**	K-W	H(2) = 16.16

Lung lesion score quantification showed bronchitis, interstitial inflammation, and endothelialitis in IAV-infected dams at 2 dpi. No changes were noted in 11 of 13 control dams. Mild bronchitis, characterized by an occasional influx of neutrophils in bronchi, was noted in 1 of 13 control dams. Mild interstitial pneumonia was noted in 1 of 13 control dams. Mild bronchitis was noted in 4 of 14 $X31_{mod}$ dams. Moderate (2 of 14) to marked (5 of 14) suppurative bronchopneumonia in $X31_{mod}$ dams is characterized by sections of bronchi filled with clusters of degenerative neutrophils with cellular debris and arterial and venous endothelial attached with rolls of neutrophils. Mild bronchitis was noted in 4 of 12 $X31_{hi}$ dams. Mild (1 of 12), moderate (2 of 12), to marked (5 of 12) suppurative bronchopneumonia was noted in $X31_{hi}$ dams. Lung lesion score quantification showed bronchitis, interstitial inflammation, edema, endothelialitis, and pleuritis in IAV-infected dams at 7 dpi. No changes were noted in 8 of 10 control dams. Mild periarteriolitis, as characterized by infiltration of macrophages and lymphocytes in the periarteriolar interstitium, was found in 2 control dams. Marked subacute bronchointerstitial pneumonia, as characterized by clusters of degenerative neutrophils and foamy macrophages in alveoli and bronchi; infiltration of lymphocytes, neutrophils, and macrophages in periarteriolar and alveolar interstitium; attachment of rolling neutrophils to arterial and venous endothelia, was found in 8 of 9 $X31_{mod}$ dams. One $X31_{mod}$ dam did not have pathology. Mild (2 of 10) to marked (7 of 10) subacute bronchointerstitial pneumonia was noted in $X31_{hi}$ dams. One $X31_{hi}$ dam did not have pathology.

IAV = influenza A virus, dpi = days post-inoculation, $X31_{mod}$ = IAV-X31 10^3 $TCID_{50}$, $X31_{hi}$ = IAV-X31 10^4 $TCID_{50}$. Kruskal-Wallis (K-W) ANOVA with Dunn's correction for multiple comparisons was used. Data are means ± SEM; bold font = $p < 0.05$; 2 dpi n = 12-14, 7 dpi n = 9-10 dams per treatment group.
