# Supplementary material for: Influenza A virus during pregnancy disrupts maternal intestinal immunity and fetal cortical development in a dose- and time-dependent manner
Source: Mol Psychiatry. 2024 Jul 3;30(1):13–28. doi: 10.1038/s41380-024-02648-9 (PMC11649561; doi:10.1038/s41380-024-02648-9)
Supplement: Supplementary file 5 — Supplemental Table S4 [file 41380_2024_2648_MOESM5_ESM.pdf]

Supplemental Table S4. Lung qPCR at 2 and 7 dpi.

Timepoint	Gene	Control	$X31_{mod}$	$X31_{hi}$	p-value	Test	Statistic
2 dpi	*Il6*	1.00 ± 0.24	4.59 ± 1.49	12.93 ± 3.45	**0.002**	K-W	H(2) = 12.07
	Tnf	1.00 ± 0.09	3.31 ± 0.71	6.08 ± 1.18	**0.001**	B-F + W	F*(2, 18.94) = 10.02
	Il1b	1.00 ± 0.19	2.09 ± 0.48	4.15 ± 0.83	**0.003**	B-F + W	F*(2, 19.91) = 7.84
	Ifna	1.00 ± 0.22	0.77 ± 0.22	1.85 ± 0.51	0.09	B-F + W	F*(2, 18.19) = 2.69
	Ifnb1	1.00 ± 0.26	1.10 ± 0.42	1.90 ± 0.51	0.28	K-W	H(2) = 2.51
	Ifng	1.00 ± 0.19	1.26 ± 0.23	2.84 ± 0.60	**0.020**	K-W	H(2) = 7.76
	Il17a	1.00 ± 0.13	0.97 ± 0.24	1.47 ± 0.29	0.24	One-way	F(2, 29) = 1.50
	Il17f	1.00 ± 0.11	1.37 ± 0.22	2.35 ± 0.39	**0.006**	B-F + W	F*(2, 20.13) = 6.80
7 dpi	*Il6*	1.00 ± 0.21	0.20 ± 0.05	0.49 ± 0.17	**0.008**	One-way	F(2, 26) = 5.89
	Tnf	1.00 ± 0.19	0.60 ± 0.11	0.94 ± 0.25	0.31	B-F+W	F*(2, 20.26) = 1.24
	Il1b	1.00 ± 0.17	4.69 ± 0.80	6.55 ± 2.13	**0.002**	K-W	H(2) = 9.50
	Ifna	1.00 ± 0.21	0.07 ± 0.01	0.26 ± 0.10	**0.001**	K-W	H(2) = 13.68
	Ifnb1	1.00 ± 0.21	0.11 ± 0.02	0.20 ± 0.10	**0.0005**	K-W	H(2) = 15.08
	Ifng	1.00 ± 0.13	0.14 ± 0.03	0.34 ± 0.10	**0.0001**	One-way	F(2, 24) = 20.18
	Il17a	1.00 ± 0.25	3.39 ± 1.02	4.61 ± 1.20	**0.04**	K-W	H(2) = 6.54
	Il17f	1.00 ± 0.07	2.80 ± 0.64	4.44 ± 0.93	**0.0005**	K-W	H(2) = 15.14

Relative inflammatory cytokine gene expression in lung tissue at 2 and 7 dpi. *IAV* = influenza A virus, *dpi* = days post-inoculation, $X31_{mod}$ = IAV-X31 10^3 $TCID_{50}$, $X31_{hi}$ = IAV-X31 10^4 $TCID_{50}$, housekeeping gene = *Hprt1* . One-way ANOVA is the default statistical test unless residuals fail to meet normality (use K-W = Kruskal-Wallis) or homogeneity of variance (use B-F + W = Brown-Forsythe + Welch). Data are means ± SEM; bold font = $p < 0.05$, 2 dpi n = 12-14, 7 dpi n = 9-10 per treatment group.
