# Supplementary material for: Influenza A virus during pregnancy disrupts maternal intestinal immunity and fetal cortical development in a dose- and time-dependent manner
Source: Mol Psychiatry. 2024 Jul 3;30(1):13–28. doi: 10.1038/s41380-024-02648-9 (PMC11649561; doi:10.1038/s41380-024-02648-9)
Supplement: Supplementary file 6 — Supplemental Table S5 [file 41380_2024_2648_MOESM6_ESM.pdf]

Supplemental Table S5.Serum Protein Concentrations at 2 and 7 dpi.

**Timepoint**	**Protein**	**Control**	**$X31_{mod}$**	**$X31_{hi}$**	**p-value**	**Test**	**Statistic**
2 dpi	IL-1α	14.6 ± 1.97	9.36 ± 1.19	10.6 ± 2.11	0.11	K-W	H(2) = 4.30
	IL-1β	66.0 ± 16.5	27.9 ± 5.64	83.1 ± 24.4	**0.03**	K-W	H(2) = 6.76
	IL-6	6.81 ± 1.57	36.1 ± 10.5	46.5 ± 9.37	**0.013**	K-W	H(2) = 8.69
	IL-10	80.3 ± 22.3	15.8 ± 5.19	35.5 ± 10.4	**0.03**	B-F + W	F*(2, 14.16) = 5.16
	IL-12p70	3.75 ± 0.88	2.11 ± 0.53	3.93 ± 1.03	0.26	K-W	H(2) = 2.68
	IL-17A	7.04 ± 0.62	6.57 ± 0.96	6.31 ± 0.86	0.52	K-W	H(2) = 1.31
	IL-23	1679 ± 587	1385 ± 529	1273 ± 467	0.95	K-W	H(2) = 0.10
	IL-27	351 ± 82.5	247 ± 50.5	290 ± 58.4	0.66	K-W	H(2) = 0.85
	IFN-β	55.5 ± 7.38	62.9 ± 5.21	75.3 ± 8.07	0.10	K-W	H(2) = 4.68
	IFN-γ	23.6 ± 3.76	18.3 ± 3.48	24.6 ± 4.34	0.41	K-W	H(2) = 1.77
	TNF-α	56.3 ± 10.4	66.7 ± 13.3	86.4 ± 20.8	0.67	K-W	H(2) = 0.81
	CCL2	22.7 ± 2.81	22.1 ± 1.85	21.8 ± 2.29	0.91	K-W	H(2) = 0.19
	GM-CSF	4.46 ± 0.86	4.02 ± 0.58	5.68 ± 0.88	0.36	K-W	H(2) = 2.04
7 dpi	IL-1α	6.71 ± 1.88	10.4 ± 3.83	5.20 ± 1.10	0.89	K-W	H(2) = 0.23
	IL-1β	80.1 ± 24.9	46.0 ± 12.1	43.5 ± 8.41	0.72	K-W	H(2) = 0.67
	IL-6	27.8 ± 7.31	72.3 ± 18.8	146 ± 26.3	**0.0006**	One-way	F(2, 26) = 9.96
	IL-10	225 ± 37.5	342 ± 109	577 ± 128	0.06	B-F+W	F*(2, 18.39) = 3.39
	IL-12p70	4.33 ± 1.91	25.2 ± 8.43	13.0 ± 4.45	0.11	K-W	H(2) = 4.32
	IL-17A	13.3 ± 2.66	15.9 ± 2.50	22.1 ± 9.21	0.72	K-W	H(2) = 0.68
	IL-23	203 ± 62.1	496 ± 122	711 ± 201	**0.04**	K-W	H(2) = 6.31
	IL-27	2323 ± 460	2203 ± 420	1384 ± 330	0.13	K-W	H(2) = 4.07
	IFN-β	240 ± 71.4	256 ± 86.2	356 ± 99.0	0.82	K-W	H(2) = 0.39
	IFN-γ	51.1 ± 12.1	226 ± 39.2	788 ± 180	**0.0002**	K-W	H(2) = 17.60
	TNF-α	102 ± 13.5	136 ± 21.5	140 ± 14.5	0.23	One-way	F(2, 25) = 1.57
	CCL2	35.8 ± 8.25	32.4 ± 8.37	43.9 ± 6.43	0.52	K-W	H(2) = 1.31
	GM-CSF	29.2 ± 8.28	29.6 ± 6.37	31.7 ± 8.38	0.87	K-W	H(2) = 0.28

Inflammatory protein concentrations (pg/mL) in dam serum at 2 and 7 dpi. *IAV* = influenza A virus, *dpi* = days post-inoculation, $X31_{mod}$ = IAV-X31 10^3 $TCID_{50}$, $X31_{hi}$ = IAV-X31 10^4 $TCID_{50}$. One-way ANOVA is the default statistical test unless residuals fail to meet normality (use K-W = Kruskal-Wallis) or homogeneity of variance (use B-F + W = Brown-Forsythe + Welch). Data are means ± SEM; bold font = p < 0.05, 2 dpi n = 12-14, 7 dpi n = 9-10 per treatment group.
