# Supplementary material for: Influenza A virus during pregnancy disrupts maternal intestinal immunity and fetal cortical development in a dose- and time-dependent manner
Source: Mol Psychiatry. 2024 Jul 3;30(1):13–28. doi: 10.1038/s41380-024-02648-9 (PMC11649561; doi:10.1038/s41380-024-02648-9)
Supplement: Supplementary file 7 — Supplemental Table S6 [file 41380_2024_2648_MOESM7_ESM.pdf]

Supplemental Table S6. Colon LPL Flow Cytometry at 2 and 7 dpi.

Timepoint	Cell type	Gated on	Control	$X31_{mod}$	$X31_{hi}$	p-value	Test	Statistic
2 dpi (% subset)	CD45+CD4+	RORγt+	55.6 ± 1.57	56.9 ± 2.82	60.6 ± 1.50	0.24	One-way	F(2, 18) = 1.56
		IL-17A+	13.3 ± 0.82	13.5 ± 1.80	11.9 ± 1.31	0.70	One-way	F(2, 18) = 0.36
		IL-17F+	3.04 ± 0.27	2.59 ± 0.28	1.89 ± 0.27	**0.03**	One-way	F(2, 18) = 4.39
		IFN-γ+	8.45 ± 0.99	6.49 ± 0.54	6.28 ± 0.99	0.18	One-way	F(2, 18) = 1.89
		Tbet+	4.83 ± 1.31	4.22 ± 0.95	3.79 ± 0.55	0.76	One-way	F(2, 18) = 0.28
		RORγt+IL-17A+	11.9 ± 0.76	12.3 ± 1.89	11.2 ± 1.10	0.86	One-way	F(2, 18) = 0.15
		RORγt+IL-17F+	2.64 ± 0.24	2.21 ± 0.25	1.65 ± 0.20	**0.02**	One-way	F(2, 18) = 4.65
		RORgt+IFN-γ+	3.18 ± 0.49	2.71 ± 0.29	2.40 ± 0.29	0.34	One-way	F(2, 18) = 1.14
		Tbet+RORγt+	1.43 ± 0.38	1.30 ± 0.32	1.15 ± 0.20	0.81	One-way	F(2, 18) = 0.21
		Tbet+IFN-γ+	1.22 ± 0.46	0.97 ± 0.27	0.62 ± 0.11	0.66	K-W	H(2) = 0.89
	CD45+CD4+RORγt+	Tbet+	2.47 ± 0.65	2.39 ± 0.69	1.92 ± 0.33	0.77	One-way	F(2, 18) = 0.26
		IL-17A+	21.4 ± 1.16	20.9 ± 2.45	18.5 ± 1.73	0.51	One-way	F(2, 18) = 0.69
		IL-17F+	4.83 ± 0.44	3.90 ± 0.47	2.75 ± 0.39	**0.01**	One-way	F(2, 18) = 5.81
		IFN-γ+	5.82 ± 0.92	4.92 ± 0.51	4.06 ± 0.50	0.21	One-way	F(2, 18) = 1.73
		IL-17A+ IFN-γ+	2.81 ± 0.45	2.41 ± 0.35	1.83 ± 0.30	0.20	One-way	F(2, 18) = 1.76
	CD45+CD4+Tbet+	RORγt+	28.6 ± 5.05	29.4 ± 2.61	31.1 ± 3.00	0.88	One-way	F(2, 18) = 0.13
		IL-17A+	5.34 ± 1.63	5.51 ± 1.77	3.99 ± 1.19	0.75	One-way	F(2, 18) = 0.29
		IL-17F+	1.26 ± 0.63	1.36 ± 0.62	2.44 ± 1.04	0.71	K-W	H(2) = 0.75
		IFN-γ+	21.6 ± 3.88	25.0 ± 2.37	17.1 ± 1.87	0.17	One-way	F(2, 18) = 1.94
7 dpi (% subset)	CD45+CD4+	RORγt+	60.7 ± 1.89	57.8 ± 2.15	49.5 ± 2.99	**0.007**	One-way	F(2, 26) = 5.99
		IL-17A+	14.7 ± 1.37	13.3 ± 1.28	11.3 ± 1.10	0.16	One-way	F(2, 26) = 1.99
		IL-17F+	2.01 ± 0.14	0.93 ± 0.11	0.84 ± 0.11	**0.001**	One-way	F(2, 26) = 29.5
		IFN-γ+	5.60 ± 0.78	4.59 ± 0.58	4.66 ± 0.77	0.48	K-W	H(2) = 1.48
		Tbet+	1.45 ± 0.31	1.78 ± 0.33	1.59 ± 0.24	0.73	One-way	F(2, 26) = 0.32
		RORγt+IL-17A+	14.2 ± 1.35	12.2 ± 1.11	9.50 ± 1.09	**0.03**	One-way	F(2, 26) = 4.02
		RORγt+IL-17F+	1.68 ± 0.13	0.78 ± 0.11	0.55 ± 0.12	**0.001**	One-way	F(2, 26) = 24.99
		RORgt+IFN-γ+	2.32 ± 0.33	1.46 ± 0.24	1.50 ± 0.22	**0.03**	K-W	H(2) = 6.73
		Tbet+RORγt+	0.40 ± 0.09	0.54 ± 0.18	0.43 ± 0.09	0.96	K-W	H(2) = 0.09
		Tbet+IFN-γ+	0.32 ± 0.11	0.24 ± 0.07	0.13 ± 0.05	0.33	K-W	H(2) = 2.22
		Tbet+	0.77 ± 0.16	0.51 ± 0.10	0.88 ± 0.19	0.30	K-W	H(2) = 2.42
		IL-17A+	23.2 ± 2.17	21.2 ± 2.04	20.5 ± 3.03	0.71	One-way	F(2, 26) = 0.34

	CD45+CD4+RORγt+	IL-17F+	2.91 ± 0.16	1.43 ± 0.19	1.27 ± 0.27	**0.001**	One-way	F(2, 26) = 18.55
		IFN-γ+	3.13 ± 0.21	2.50 ± 0.45	3.04 ± 0.50	0.21	K-W	H(2) = 3.09
		IL-17A+ IFN-γ+	1.69 ± 0.15	1.37 ± 0.28	1.69 ± 0.29	0.60	One-way	F(2, 25) = 0.52
	CD45+CD4+Tbet+	RORγt+	35.5 ± 4.55	27.8 ± 4.84	25.9 ± 4.88	0.33	One-way	F(2, 26) = 1.17
		IL-17A+	9.33 ± 2.84	3.55 ± 1.59	4.22 ± 1.85	0.22	K-W	H(2) = 2.30
		IL-17F+	1.97 ± 0.86	1.33 ± 0.61	0.00 ± 0.00	0.05	K-W	H(2) = 5.89
		IFN-γ+	21.4 ± 3.82	15.3 ± 3.73	9.39 ± 3.18	0.07	One-way	F(2, 26) = 2.90
2 dpi (absolute counts)	CD45+CD4+	RORγt+	43.1 ± 1.55	36.5 ± 4.03	50.7 ± 8.17	0.22	B-F + W	F*(2, 9.25) = 1.77
		IL-17A+	10.3 ± 0.71	8.66 ± 1.46	9.52 ± 1.47	0.67	One-way	F(2, 18) = 0.40
		IL-17F+	2.35 ± 0.21	1.72 ± 0.28	1.68 ± 0.42	0.27	One-way	F(2, 18) = 1.40
		IFN-γ+	6.46 ± 0.67	4.33 ± 0.78	5.31 ± 1.19	0.28	One-way	F(2, 18) = 1.38
		Tbet+	3.69 ± 0.97	2.87 ± 0.89	3.14 ± 0.79	0.79	K-W	H(2) = 0.50
		RORγt+IL-17A+	9.25 ± 0.64	7.92 ± 1.50	8.07 ± 1.86	0.77	One-way	F(2, 18) = 0.26
		RORγt+IL-17F+	2.04 ± 0.18	1.46 ± 0.25	1.43 ± 0.33	0.21	One-way	F(2, 18) = 1.69
		RORgt+IFN-γ+	2.43 ± 0.34	1.76 ± 0.28	2.10 ± 0.47	0.46	One-way	F(2, 18) = 0.81
		Tbet+RORγt+	1.10 ± 0.29	0.89 ± 0.29	1.02 ± 0.29	0.88	One-way	F(2, 18) = 0.13
		Tbet+IFN-γ+	0.93 ± 0.34	0.43 ± 0.08	0.49 ± 0.13	0.74	K-W	H(2) = 0.63
7 dpi (absolute counts)	CD45+CD4+	RORγt+	68.0 ± 6.32	58.3 ± 4.55	41.9 ± 7.99	**0.04**	K-W	H(2) = 6.39
		IL-17A+	17.2 ± 2.20	13.4 ± 1.67	9.64 ± 1.74	**0.03**	One-way	F(2, 26) = 4.03
		IL-17F+	2.28 ± 0.64	0.96 ± 0.14	0.77 ± 0.17	**0.0001**	One-way	F(2, 26) = 22.1
		IFN-γ+	6.23 ± 0.79	4.53 ± 0.55	3.66 ± 0.57	**0.03**	One-way	F(2, 26) = 4.17
		Tbet+	1.68 ± 0.43	1.79 ± 0.32	1.30 ± 0.23	0.66	K-W	H(2) = 0.84
		RORγt+IL-17A+	16.0 ± 2.08	12.3 ± 1.47	8.16 ± 1.53	**0.01**	One-way	F(2, 26) = 5.23
		RORγt+IL-17F+	1.86 ± 0.17	0.80 ± 0.13	0.55 ± 0.14	**0.0001**	One-way	F(2, 26) = 23.0
		RORgt+IFN-γ+	2.27 ± 0.26	1.40 ± 0.19	1.19 ± 0.22	**0.006**	One-way	F(2, 25) = 6.37
		Tbet+RORγt+	0.48 ± 0.12	0.54 ± 0.17	0.35 ± 0.07	0.85	K-W	H(2) = 0.33
		Tbet+IFN-γ+	0.34 ± 0.11	0.24 ± 0.07	0.14 ± 0.05	0.22	One-way	F(2, 26) = 1.58

Flow cytometric analysis of colonic lamina propria lymphocytes (LPLs) at 2 and 7 dpi. *IAV* = influenza A virus, *dpi* = days post-inoculation, $X31_{mod}$ = IAV-X31 10^3 $TCID_{50}$, $X31_{hi}$ = IAV-X31 10^4 $TCID_{50.}$ One-way ANOVA is the default statistical test unless residuals fail to meet normality (use K-W = Kruskal-Wallis) or homogeneity of variance (use B-F + W = Brown-Forsythe + Welch). Data are means ± SEM; bold font = p < 0.05, 2 dpi n = 7, 7 dpi n = 9-10 per treatment group.
