# Supplementary material for: Influenza A virus during pregnancy disrupts maternal intestinal immunity and fetal cortical development in a dose- and time-dependent manner
Source: Mol Psychiatry. 2024 Jul 3;30(1):13–28. doi: 10.1038/s41380-024-02648-9 (PMC11649561; doi:10.1038/s41380-024-02648-9)
Supplement: Supplementary file 8 — Supplemental Table S7 [file 41380_2024_2648_MOESM8_ESM.pdf]

Supplemental Table S7. Ileum qPCR at 2 and 7 dpi.

Timepoint	Gene	Control	$X31_{mod}$	$X31_{hi}$	p-value	Test	Statistic
2 dpi	*Il6*	1.00 ± 0.11	1.52 ± 0.34	2.52 ± 0.67	0.08	K-W	H(2) = 5.05
	Tgfb1	1.00 ± 0.05	1.00 ± 0.08	1.04 ± 0.08	0.93	One-way	F(2, 36) = 0.07
	Il1b	1.00 ± 0.11	0.86 ± 0.12	0.61 ± 0.10	0.06	K-W	H(2) = 5.58
	Il23a	1.00 ± 0.05	0.93 ± 0.10	1.10 ± 0.12	0.36	K-W	H(2) = 2.05
	Il17a	1.00 ± 0.12	1.33 ± 0.20	0.93 ± 0.23	0.28	K-W	H(2) = 2.54
	Il17f	1.00 ± 0.13	1.20 ± 0.17	1.16 ± 0.25	0.74	K-W	H(2) = 0.60
	Il22	1.00 ± 0.16	0.71 ± 0.16	0.26 ± 0.09	**0.007**	One-way	F(2, 34) = 5.71
	Il17ra	1.00 ± 0.05	0.83 ± 0.04	1.02 ± 0.12	0.15	B-F + W	F*(2, 15.52) = 2.14
	Rorc	1.00 ± 0.09	0.90 ± 0.11	1.38 ± 0.18	**0.03**	One-way	F(2, 36) = 3.73
	Tnf	1.00 ± 0.07	1.13 ± 0.10	1.23 ± 0.15	0.36	One-way	F(2, 35) = 1.06
	Il15	1.00 ± 0.09	0.92 ± 0.14	1.18 ± 0.25	0.57	B-F + W	F*(2, 20.72) = 0.58
	Il10	1.00 ± 0.11	1.05 ± 0.14	1.27 ± 0.19	0.42	One-way	F(2, 33) = 0.90
	Ifna	1.00 ± 0.14	1.24 ± 0.31	1.80 ± 0.38	0.17	K-W	H(2) = 3.50
	Ifnb1	1.00 ± 0.15	1.01 ± 0.21	1.58 ± 0.24	0.09	One-way	F(2, 33) = 2.54
	Ifng	1.00 ± 0.09	1.09 ± 0.11	1.17 ± 0.10	0.49	K-W	H(2) = 1.45
	Reg3b	1.00 ± 0.11	1.10 ± 0.18	1.13 ± 0.39	0.53	K-W	H(2) =1.28
	Reg3g	1.00 ± 0.13	1.01 ± 0.17	0.27 ± 0.11	**0.003**	One-way	F(2, 33) = 6.96
	Duox2	1.00 ± 0.10	0.74 ± 0.10	0.62 ± 0.20	0.07	K-W	H(2) = 5.22
	Cldn1	1.00 ± 0.08	1.16 ± 0.19	1.56 ± 0.46	0.99	K-W	H(2) = 0.01
	Cldn2	1.00 ± 0.08	1.08 ± 0.16	0.72 ± 0.09	0.10	B-F + W	F*(2, 26.40) = 0.10
	Cldn5	1.00 ± 0.07	1.24 ± 0.18	1.00 ± 0.12	0.88	K-W	H(2) = 0.25
	Ocln	1.00 ± 0.08	0.75 ± 0.09	0.86 ± 0.10	0.15	One-way	F(2, 36) = 2.02
	X31 NP	N.D	N.D	N.D	-	-	-
	Il6	1.00 ± 0.13	0.94 ± 0.14	2.68 ± 0.33	**0.0001**	B-F + W	F*(2, 14.63) = 19.88
	Tgfb1	1.00 ± 0.08	0.75 ± 0.04	0.78 ± 0.10	**0.03**	K-W	H(2) = 6.91
	Il1b	1.00 ± 0.13	0.63 ± 0.12	0.57 ± 0.10	**0.03**	One-way	F(2, 25) = 4.06
	Il23a	1.00 ± 0.06	0.77 ± 0.10	0.87 ± 0.11	0.24	One-way	F(2, 25) = 1.51
	Il17a	1.00 ± 0.06	0.59 ± 0.10	0.32 ± 0.11	**0.0001**	One-way	F(2, 25) = 13.13
	Il17f	1.00 ± 0.12	0.63 ± 0.09	0.49 ± 0.16	**0.02**	One-way	F(2, 25) = 4.38
	Il22	1.00 ± 0.15	0.52 ± 0.16	0.37 ± 0.14	**0.01**	K-W	H(2) = 8.57

7 dpi	*Il17ra*	1.00 ± 0.14	0.73 ± 0.07	1.12 ± 0.07	**0.01**	K-W	H(2) = 9.29
	Rorc	1.00 ± 0.06	1.15 ± 0.14	1.99 ± 0.25	**0.002**	B-F + W	F*(2, 14.88) = 10.45
	Tnf	1.00 ± 0.07	0.92 ± 0.16	0.85 ± 0.14	0.29	K-W	H(2) = 2.51
	Il15	1.00 ± 0.09	1.98 ± 0.48	6.41 ± 1.35	**0.0001**	K-W	H(2) = 17.94
	Il10	1.00 ± 0.10	0.78 ± 0.10	0.54 ± 0.11	**0.02**	One-way	F(2, 25) = 4.85
	Ifna	1.00 ± 0.16	0.69 ± 0.15	1.72 ± 0.23	**0.003**	One-way	F(2, 24) = 7.71
	Ifnb1	1.00 ± 0.17	0.60 ± 0.09	1.87 ± 0.36	**0.006**	K-W	H(2) = 10.40
	Ifng	1.00 ± 0.11	1.23 ± 0.21	2.12 ± 0.39	**0.02**	B-F + W	F*(2, 13.54) = 5.19
	Reg3b	1.00 ± 0.23	0.53 ± 0.09	0.09 ± 0.04	**0.0004**	K-W	H(2) = 10.06
	Reg3g	1.00 ± 0.14	0.85 ± 0.21	0.05 ± 0.02	**0.0003**	K-W	H(2) = 16.48
	Duox2	1.00 ± 0.09	0.84 ± 0.15	0.32 ± 0.12	**0.001**	One-way	F(2, 26) = 9.11
	Cldn1	1.00 ± 0.13	1.42 ± 0.22	1.70 ± 0.34	0.15	One-way	F(2, 26) = 2.06
	Cldn2	1.00 ± 0.10	1.00 ± 0.18	0.60 ± 0.16	**0.04**	K-W	H(2) = 6.23
	Cldn5	1.00 ± 0.20	0.37 ± 0.08	0.35 ± 0.04	**0.005**	B-F + W	F*(2, 12.13) = 8.33
	Ocln	1.00 ± 0.13	1.51 ± 0.26	2.64 ± 0.66	**0.03**	K-W	H(2) = 6.76
	X31 NP	N.D	N.D	N.D	-	-	-

Relative immune, antimicrobial, and barrier integrity gene expression in ileum tissue at 2 and 7 dpi. *IAV* = influenza A virus, *dpi* = days post-inoculation, $X31_{mod}$ = IAV-X31 10^3 $TCID_{50}$, $X31_{hi}$ = IAV-X31 10^4 $TCID_{50}$, housekeeping gene = *Rplp0*. One-way ANOVA is the default statistical test unless residuals fail to meet normality (use K-W = Kruskal-Wallis) or homogeneity of variance (use B-F + W = Brown-Forsythe + Welch). Data are means ± SEM; bold font = p < 0.05, 2 dpi n = 12-14, 7 dpi n = 9-10 per treatment group.
