# Supplementary material for: Influenza A virus during pregnancy disrupts maternal intestinal immunity and fetal cortical development in a dose- and time-dependent manner
Source: Mol Psychiatry. 2024 Jul 3;30(1):13–28. doi: 10.1038/s41380-024-02648-9 (PMC11649561; doi:10.1038/s41380-024-02648-9)
Supplement: Supplementary file 9 — Supplemental Table S8 [file 41380_2024_2648_MOESM9_ESM.pdf]

Supplemental Table S8. Quantification of fetal brain cortical layers at E16.5, 7 dpi.

Brain Region	Analysis	Cell Markers	Control	$X31_{mod}$	$X31_{hi}$	p-value	Test	Statistic
Right hemisphere	MFI	SATB2	45.1 ± 3.83	32.7 ± 6.94	17.7 ± 3.16	**0.002**	One-way	F(2, 20) = 8.86
		TBR1	70.4 ± 5.62	56.5 ± 4.08	54.5 ± 6.03	0.10	One-way	F(2, 20) = 2.63
	Counts	SATB2	216 ± 23.1	154 ± 18.3	95.5 ± 31.3	**0.009**	One-way	F(2, 19) = 6.07
		TBR1	285 ± 15.6	271 ± 20.8	227 ± 24.9	0.14	One-way	F(2, 19) = 2.23
Left Hemisphere	MFI	SATB2	37.1 ± 5.52	33.2 ± 8.00	15.2 ± 2.28	**0.02**	One-way	F(2, 19) = 4.65
		TBR1	53.7 ± 6.16	50.5 ± 8.99	52.6 ± 6.75	0.95	One-way	F(2, 19) = 0.05
Rostral Region	MFI	SATB2	61.9 ± 13.4	46.4 ± 10.2	32.2 ± 9.48	0.16	K-W	H(3) = 3.66
		TBR1	75.4 ± 11.7	73.4 ± 10.4	68.7 ± 11.8	0.91	One-way	F(2, 12) = 0.09
Bin 1	MFI	SATB2	78.7 ± 21.9	115 ± 38.5	196 ± 54.2	0.05	K-W	H(2) = 6.01
Bin 2			186 ± 39.9	219 ± 14.3	239 ± 35.0	0.55	K-W	H(2) = 1.20
Bin 3			214 ± 13.0	237 ± 16.3	159 ± 34.3	0.09	B-F + W	F*(2, 11.94) = 2.92
Bin 4			157 ± 16.9	141 ± 13.6	143 ± 27.2	0.83	One-way	F(2, 20) = 0.18
Bin 5			105 ± 16.3	71.3 ± 7.58	114 ± 19.1	0.17	One-way	F(2, 20) = 1.96
Bin 6			77.3 ± 13.3	46.8 ± 4.63	67.0 ± 8.63	0.12	One-way	F(2, 20) = 2.38
Bin 7			54.2 ± 8.83	51.6 ± 3.61	29.0 ± 6.05	**0.03**	B-F + W	F(2, 14.81) = 4.59
Bin 8			39.7 ± 7.26	39.3 ± 6.51	18.5 ± 5.57	**0.05**	One-way	F(2, 20) = 3.56
Bin 9			37.7 ± 8.77	34.2 ± 8.49	14.2 ± 5.60	0.09	One-way	F(2, 20) = 2.79
Bin 10			23.7 ± 3.58	31.3 ± 9.46	11.6 ± 4.40	0.1	One-way	F(2, 19) = 2.63
Bin 1	MFI	TBR1	14.4 ± 6.17	9.21 ± 2.60	8.11 ± 1.41	0.98	K-W	H(2) = 0.05
Bin 2			37.4 ± 10.3	46.0 ± 6.55	102 ± 29.2	0.13	K-W	H(2) = 4.03
Bin 3			61.9 ± 3.73	130 ± 16.8	210 ± 53.4	**0.0009**	K-W	H(2) = 11.32
Bin 4			176 ± 29.8	247 ± 25.7	214 ± 27.3	0.20	K-W	H(2) = 3.23
Bin 5			219 ± 20.4	275 ± 15.4	189 ± 36.4	0.10	One-way	F(2, 20) = 2.61
Bin 6			207 ± 30.0	152 ± 27.3	140 ± 36.7	0.30	One-way	F(2, 20) = 1.26
Bin 7			111 ± 26.3	64.1 ± 11.0	64.6 ± 23.1	0.23	One-way	F(2, 20) = 1.57
Bin 8			55.9 ± 12.1	22.8 ± 5.48	32.3 ± 11.6	0.10	K-W	H(2) = 4.61
Bin 9			40.3 ± 7.52	14.0 ± 2.27	10.8 ± 3.89	**0.003**	B-F + W	F*(2, 11.86) = 10.33
Bin 10			21.3 ± 3.35	15.1 ± 3.44	8.35 ± 2.32	**0.03**	K-W	H(2) = 7.25

Quantification of SATB2 and TBR1 in E16.5, 7 dpi fetal brains. *MFI* = mean fluorescence intensity, *IAV* = influenza A virus, *dpi* = days post-inoculation, $X31_{mod}$ = IAV-X31 10^3 $TCID_{50}$, $X31_{hi}$ = IAV-X31 10^4 $TCID_{50}$. One-way ANOVA is the default statistical test unless residuals fail to meet normality (use K-W = Kruskal-Wallis) or homogeneity of variance (use B-F + W = Brown-Forsythe + Welch). Data are means ± SEM; bold font = $p < 0.05$, 7 dpi n = 9-10 per treatment group.
