# Supplementary material for: Influenza A virus during pregnancy disrupts maternal intestinal immunity and fetal cortical development in a dose- and time-dependent manner
Source: Mol Psychiatry. 2024 Jul 3;30(1):13–28. doi: 10.1038/s41380-024-02648-9 (PMC11649561; doi:10.1038/s41380-024-02648-9)
Supplement: Supplementary file 10 — Supplemental Table S9 [file 41380_2024_2648_MOESM10_ESM.pdf]

Supplemental Table S9. Fetal brain transcript GO Terms at E16.5, 7 dpi.

	Go term (direct)	GO	Category	Count	%	P-value	- log (P-value)
Upregulated	glutamatergic synapse	GO:0098978	CC	27	12.8	1.80E-10	9.745
	modulation of synaptic transmission	GO:0050804	BP	11	5.2	3.70E-07	6.432
	calcium ion binding	GO:0005509	MF	22	10.4	3.00E-06	5.523
	membrane	GO:0016020	CC	98	46.4	2.90E-05	4.538
	synapse	GO:0045202	CC	24	11.4	3.60E-05	4.444
	postsynaptic density	GO:0014069	CC	13	6.2	1.30E-04	3.886
	GABA-ergic synapse	GO:0098982	CC	8	3.8	2.00E-04	3.699
	brain development	GO:0007420	BP	11	5.2	2.80E-04	3.553
	nervous system development	GO:0007399	BP	13	6.2	6.30E-04	3.201
	neuron projection	GO:0043005	CC	15	7.1	7.20E-04	3.143
Downregulated	nucleus	GO:0005634	CC	79	45.7	2.90E-07	6.538
	nervous system development	GO:0007399	BP	14	8.1	3.00E-05	4.523
	cell division	GO:0051301	BP	13	7.5	4.70E-05	4.328
	cell cycle	GO:0007049	BP	16	9.2	1.10E-04	3.959
	cytoplasm	GO:0005737	CC	78	45.1	1.90E-04	3.721
	chromosome	GO:0005694	CC	14	8.1	3.80E-04	3.420
	protein binding	GO:0005515	MF	61	35.3	4.70E-04	3.328
	mRNA binding	GO:0003729	MF	9	5.2	1.40E-03	2.854
	microtubule	GO:0005874	CC	10	12.7	1.50E-03	2.824
	cytoskeleton	GO:0005856	CC	22	9.8	1.70E-03	2.770

Top 10 significantly enriched upregulated and downregulated GO terms from DAVID. *Dpi* = days post inoculation, *GO* = gene ontology, *DAVID* = Database for Annotation, Visualization, and Integrated Discovery terms, *CC* = cellular component, *BP* = biological process, *MF* = molecular function.
