# Supplementary material for: Influenza A virus during pregnancy disrupts maternal intestinal immunity and fetal cortical development in a dose- and time-dependent manner
Source: Mol Psychiatry. 2024 Jul 3;30(1):13–28. doi: 10.1038/s41380-024-02648-9 (PMC11649561; doi:10.1038/s41380-024-02648-9)
Supplement: Supplementary file 11 — Supplemental Table S10 [file 41380_2024_2648_MOESM11_ESM.pdf]

Supplemental Table S10. RNA-sequencing analysis of fetal brain transcripts at E16.5, 7 dpi.

ENTREZID	Symbol	Product	TopToBottom	logFC	AveExpr	regFC	P.Value	adj.P.Val
100039060	Gm2026	predicted gene 2026, transcript variant X4	227	1.70432	3.293212	3.258753	1.277E-06	0.016258
12696	Cirbp	cold inducible RNA binding protein, transcript variant	312	0.410084	7.506424	1.328764	1.9983E-06	0.016258
213084	Cdkl3	cyclin-dependent kinase-like 3, transcript variant X33	206	0.487585	4.811875	1.402096	7.2551E-06	0.031297
665828	Gm10654	predicted gene 10654	313	0.796852	2.753356	1.737306	9.6371E-06	0.031297
19652	Rbm3	RNA binding motif (RNP1, RRM) protein 3, transcript	342	0.524075	8.244099	1.438011	1.2974E-05	0.031297
12406	Serpinh1	serine (or cysteine) peptidase inhibitor, clade H, mem	70	-0.36077	7.355881	-1.28411	1.3821E-05	0.031297
115490184	Gm42427	predicted gene 42427	193	0.985795	3.495802	1.980405	1.7292E-05	0.031297
232345	A2m	alpha-2-macroglobulin	208	0.513801	4.063634	1.427807	0.00001761	0.031297
71951	Gpc2	glypican 2 (cerebroglycan)	103	-0.36468	8.301291	-1.28759	1.9761E-05	0.031297
11464	Actc1	actin, alpha, cardiac muscle 1	51	-1.54499	2.995887	-2.91801	2.0794E-05	0.031297
19395	Rasgrp2	RAS, guanyl releasing protein 2, transcript variant 1	371	0.436296	5.154734	1.353126	2.2517E-05	0.031297
232223	Txnrd3	thioredoxin reductase 3, transcript variant 1	209	0.503318	4.112832	1.41747	2.3363E-05	0.031297
234967	Slc36a4	solute carrier family 36 (proton/amino acid symporter)	314	0.320272	6.176468	1.248566	2.5004E-05	0.031297
22042	Tfrc	transferrin receptor, transcript variant 1	249	0.61498	6.28116	1.531537	2.8511E-05	0.033138
54006	Deaf1	DEAF1, transcription factor, transcript variant 1	318	0.29603	7.117908	1.227761	3.4195E-05	0.037095
24059	Slco2a1	solute carrier organic anion transporter family, memb	309	0.641047	2.973252	1.55946	5.0473E-05	0.051331
233887	Zfp553	zinc finger protein 553, transcript variant 1	104	-0.34874	5.837976	-1.27344	5.7587E-05	0.054657
105245249	Gm40728	predicted gene, 40728, transcript variant X2	30	-2.31126	-0.65052	-4.96318	6.597E-05	0.054657
118568783	LOC118568783	igE-binding protein-like, transcript variant X1	68	-0.28498	5.928674	-1.21839	6.9042E-05	0.054657
140486	Igf2bp1	insulin-like growth factor 2 mRNA binding protein 1	154	-0.42656	5.186122	-1.34403	7.4324E-05	0.054657
22371	Vwf	Von Willebrand factor, transcript variant X1	279	0.669605	4.428503	1.590637	7.8072E-05	0.054657
17022	Lum	lumican	166	-0.57508	4.392067	-1.48976	8.0236E-05	0.054657
57265	Fzd2	frizzled class receptor 2	152	-0.36964	5.186931	-1.29203	8.3108E-05	0.054657
13602	Sparcl1	SPARC-like 1, transcript variant 3	183	0.324014	8.016795	1.251809	8.4677E-05	0.054657
68942	Chmp2b	charged multivesicular body protein 2B	306	0.278708	6.242796	1.213108	8.6416E-05	0.054657
16973	Lrp5	low density lipoprotein receptor-related protein 5	19	-0.41327	4.655054	-1.3317	8.8321E-05	0.054657
56533	Rgs17	regulator of G-protein signaling 17, transcript variant	270	0.379313	5.677592	1.300722	9.3004E-05	0.054657
18846	Plxna3	plexin A3, transcript variant X5	67	-0.23538	7.306416	-1.17722	9.6955E-05	0.054657
20394	Scg5	secretogranin V	302	0.265886	6.53796	1.202374	9.7409E-05	0.054657
320129	Grk3	G protein-coupled receptor kinase 3, transcript varian	317	0.298037	5.811972	1.229471	0.00010572	0.057344
102632200	Gm30340	predicted gene, 30340, transcript variant X5	202	0.622927	3.26819	1.539996	0.00010982	0.057645
140904	Caln1	calneuron 1, transcript variant X1	378	0.327604	5.645391	1.254928	0.00012241	0.059191
239706	Mettl22	methyltransferase like 22	271	0.514152	3.953725	1.428155	0.00012597	0.059191
230316	Megf9	multiple EGF-like-domains 9	292	0.658529	3.880917	1.578472	0.00014481	0.059191
16987	Lss	lanosterol synthase, transcript variant X1	344	0.291244	6.190246	1.223695	0.00014483	0.059191
330908	Opcml	opioid binding protein/cell adhesion molecule-like	319	0.274538	6.662474	1.209607	0.00014735	0.059191
11434	Acr	acrosin prepropeptide, transcript variant X1	239	0.592568	3.47303	1.507929	0.00014817	0.059191
15168	Hcn3	hyperpolarization-activated, cyclic nucleotide-gated K	65	-0.28097	5.816308	-1.21501	0.00014886	0.059191
628147	Gm2004	predicted gene 2004, transcript variant X1	341	0.722771	2.511725	1.650349	0.00014963	0.059191
209378	Itih5	inter-alpha (globulin) inhibitor H5	315	0.404185	4.497328	1.323341	0.00015425	0.059191
233871	Atxn2l	ataxin 2-like, transcript variant X9	18	-0.25515	8.119656	-1.19346	0.00015617	0.059191
110886	Gabra5	gamma-aminobutyric acid (GABA) A receptor, subuni	207	0.305089	5.427103	1.235495	0.0001564	0.059191
260297	Prrt1	proline-rich transmembrane protein 1, transcript varia	165	-0.35603	5.514578	-1.2799	0.00015804	0.059191
52323	Klhl7	kelch-like 7, transcript variant 1	323	0.252266	7.898614	1.191076	0.00016005	0.059191
108169008	Gm46891	predicted gene, 46891	210	1.066324	1.041009	2.094091	0.00016858	0.059766
227541	Camk1d	calcium/calmodulin-dependent protein kinase ID, tran	370	0.314807	5.142789	1.243845	0.00016896	0.059766
545667	Shisal2a	shisa like 2A	298	1.366686	0.343062	2.578775	0.00017391	0.06021
15505	Hsph1	heat shock 105kDa/110kDa protein 1, transcript varia	6	-0.31984	7.130273	-1.24819	0.00018293	0.060294
224897	Dpp9	dipeptidylpeptidase 9, transcript variant 2	21	-0.28786	5.878323	-1.22083	0.00018356	0.060294
109929	Zbtb25	zinc finger and BTB domain containing 25, transcript	308	0.309702	5.099783	1.239451	0.00018556	0.060294
74762	Mdga1	MAM domain containing glycosylphosphatidylinositol	10	-0.29676	7.019575	-1.22838	0.00018898	0.060294
110196	Fdps	farnesyl diphosphate synthetase, transcript variant 2	269	0.253626	7.336104	1.1922	0.00019386	0.060664
547150	6820431F20Rik	RIKEN cDNA 6820431F20 gene	259	0.234692	8.021544	1.176655	0.00019891	0.06107
224661	Slc26a8	solute carrier family 26, member 8, transcript variant	242	0.946715	2.040021	1.927478	0.00021228	0.062799
54139	Irf6	interferon regulatory factor 6, transcript variant X2	131	-1.22755	0.6545	-2.34169	0.00021532	0.062799
432530	Adcy1	adenylate cyclase 1	382	0.330646	7.27407	1.257576	0.00021704	0.062799
69150	Snx4	sorting nexin 4	311	0.264615	6.273138	1.201315	0.00021998	0.062799
17957	Napb	N-ethylmaleimide sensitive fusion protein attachment	182	0.371959	5.967581	1.294109	0.0002383	0.065044
21366	Slc6a6	solute carrier family 6 (neurotransmitter transporter, t	377	0.243572	7.397297	1.18392	0.00023849	0.065044
407788	BC051142	cDNA sequence BC051142, transcript variant X37	372	0.608749	2.726846	1.524936	0.00024677	0.065044
215615	Rnpep	arginyl aminopeptidase (aminopeptidase B), transcrip	105	-0.37349	5.804133	-1.29548	0.0002519	0.065044
53761	Prrc2a	proline-rich coiled-coil 2A, transcript variant 1	129	-0.30343	9.006175	-1.23407	0.00025272	0.065044
16549	Khsrp	KH-type splicing regulatory protein	50	-0.26413	7.608606	-1.20091	0.00025937	0.065044
102640338	Gm26782	predicted gene, 26782, transcript variant X6	31	-0.24215	6.509117	-1.18275	0.00026091	0.065044
231807	Map11	microtubule associated protein 11	72	-0.25883	6.087059	-1.19651	0.00026134	0.065044
93724	Pcdhga12	protocadherin gamma subfamily A, 12	52	-0.35457	4.600533	-1.2786	0.00029226	0.065044

18140	Uhrf1	ubiquitin-like, containing PHD and RING finger domai	81	-0.41549	5.528416	-1.33375	0.00029456	0.065044
246317	Neto1	neuropilin (NRP) and tolloid (TLL)-like 1, transcript va	234	0.282853	5.543342	1.216598	0.00029812	0.065044
110876	Scn2a	sodium channel, voltage-gated, type II, alpha, transcr	380	0.318368	6.622992	1.246919	0.00030353	0.065044
13447	Doc2b	double C2, beta	1	-0.33108	5.428248	-1.25795	0.0003044	0.065044
68394	Ccdc163	coiled-coil domain containing 163, transcript variant X	79	-0.65138	2.971608	-1.57067	0.00030664	0.065044
360216	Zranb1	zinc finger, RAN-binding domain containing 1, transcı	284	0.261365	7.042788	1.198612	0.00031157	0.065044
263764	Creg2	cellular repressor of E1A-stimulated genes 2	339	0.426679	4.220391	1.344136	0.00031507	0.065044
108911	Rcc2	regulator of chromosome condensation 2	90	-0.24706	7.583771	-1.18678	0.0003162	0.065044
50790	Acsl4	acyl-CoA synthetase long-chain family member 4, tra	336	0.330222	5.228661	1.257207	0.00032385	0.065044
211739	Vstm2a	V-set and transmembrane domain containing 2A, trar	343	0.26495	5.973284	1.201594	0.00032539	0.065044
21951	Tnks	tankyrase, TRF1-interacting ankyrin-related ADP-ribo	280	0.840132	5.10094	1.790214	0.00032755	0.065044
102635290	Ttc39aos1	Ttc39a opposite strand RNA 1	324	0.830686	1.862818	1.778531	0.00033065	0.065044
59090	Midn	midnolin, transcript variant 4	57	-0.23644	8.414532	-1.17808	0.00033112	0.065044
18767	Pkia	protein kinase inhibitor, alpha	248	0.310932	8.915235	1.240509	0.00033155	0.065044
104831	Ptpn23	protein tyrosine phosphatase, non-receptor type 23	2	-0.26541	6.679378	-1.20198	0.00033471	0.065044
233863	Gtf3c1	general transcription factor III C 1	127	-0.28668	6.859868	-1.21983	0.00033781	0.065044
56275	Rbm14	RNA binding motif protein 14	37	-0.30862	5.715383	-1.23852	0.00034217	0.065044
100039123	Gm14295	predicted gene 14295, transcript variant X2	275	0.435978	4.673815	1.352827	0.00034229	0.065044
53321	Cntnap1	contactin associated protein-like 1, transcript variant)	32	-0.41064	3.919788	-1.32927	0.00034282	0.065044
68026	Pclaf	PCNA clamp associated factor	84	-0.38125	5.436685	-1.30247	0.00034377	0.065044
14580	Gfap	glial fibrillary acidic protein, transcript variant 2	303	0.905465	1.209445	1.873149	0.00036133	0.067333
67789	Dalrd3	DALR anticodon binding domain containing 3, transcı	137	-0.29778	5.449551	-1.22925	0.00036414	0.067333
234076	Tmco3	transmembrane and coiled-coil domains 3, transcript	163	-0.31359	4.913732	-1.2428	0.00037208	0.068028
66368	Rtca	RNA 3'-terminal phosphate cyclase	334	0.327758	5.836165	1.255061	0.00039433	0.070227
67285	Cwc27	CWC27 spliceosome-associated protein, transcript va	304	0.360238	4.667225	1.283638	0.00039593	0.070227
329502	Pla2g4e	phospholipase A2, group IVE, transcript variant X1	198	0.478092	3.392307	1.3929	0.00040199	0.070227
233210	Prr12	proline rich 12	23	-0.22656	7.18907	-1.17004	0.00040451	0.070227
110308	Krt5	keratin 5	171	-2.762	1.901765	-6.78338	0.00040774	0.070227
14680	Gnal	guanine nucleotide binding protein, alpha stimulating,	340	0.227604	7.126975	1.170889	0.00041553	0.070227
71472	Usp19	ubiquitin specific peptidase 19, transcript variant 2	75	-0.2236	6.943599	-1.16765	0.00041674	0.070227
230872	Crocc	ciliary rootlet coiled-coil, rootletin, transcript variant X:	16	-0.31321	4.794833	-1.24247	0.00042367	0.070227
230796	Wdtc1	WD and tetratricopeptide repeats 1, transcript variant	17	-0.25471	6.189781	-1.1931	0.00042698	0.070227
232334	Vgll4	vestigial like family member 4, transcript variant X4	94	-0.31144	5.658407	-1.24095	0.00044221	0.070227
26908	Eif2s3y	eukaryotic translation initiation factor 2, subunit 3, stru	194	0.47746	5.51029	1.39229	0.00044427	0.070227
93842	Igsf9	immunoglobulin superfamily, member 9, transcript va	128	-0.33778	6.205562	-1.26381	0.00044487	0.070227
67117	Dynlt3	dynein light chain Tctex-type 3	184	0.310712	5.077541	1.240319	0.00044498	0.070227
102791	Tcta	T cell leukemia translocation altered gene, transcript	358	0.413269	4.194767	1.331699	0.00044539	0.070227
380728	Kcnh4	potassium voltage-gated channel, subfamily H (eag-r	144	-0.34018	4.745116	-1.26591	0.00045088	0.070227
277154	Nynrin	NYN domain and retroviral integrase containing	118	-0.28134	6.287395	-1.21533	0.00045591	0.070227
17218	Mcm5	minichromosome maintenance complex component 5	86	-0.39486	5.332059	-1.31481	0.00045748	0.070227
73061	Cldn34c1	claudin 34C1, transcript variant 3	301	0.334513	4.683478	1.260952	0.00046651	0.070944
12305	Ddr1	discoidin domain receptor family, member 1, transcrip	76	-0.23059	7.212547	-1.17331	0.00048757	0.072815
14254	Flt1	FMS-like tyrosine kinase 1, transcript variant 2	196	0.431309	4.993163	1.348457	0.00048776	0.072815
56874	Rnf32	ring finger protein 32, transcript variant 1	20	-0.47328	3.234295	-1.38826	0.00049622	0.073404
50997	Mpp2	membrane protein, palmitoylated 2 (MAGUK p55 sub	33	-0.25424	5.915687	-1.19271	0.00051504	0.073949
70439	Taf15	TATA-box binding protein associated factor 15	160	-0.24641	7.062353	-1.18625	0.00052	0.073949
14701	Gng12	guanine nucleotide binding protein (G protein), gamm	316	0.277949	5.368742	1.21247	0.00052085	0.073949
69719	Cad	carbamoyl-phosphate synthetase 2, aspartate transca	55	-0.33115	5.446516	-1.25801	0.00052898	0.073949
69352	Necab1	N-terminal EF-hand calcium binding protein 1, transcı	374	0.346965	4.949537	1.271882	0.00053234	0.073949
241520	Fam171b	family with sequence similarity 171, member B	326	0.260147	7.300038	1.1976	0.0005342	0.073949
269713	Clip2	CAP-GLY domain containing linker protein 2, transcri	8	-0.22844	7.720788	-1.17157	0.00053992	0.073949
233271	Luzp2	leucine zipper protein 2	251	0.36871	6.162092	1.291197	0.0005439	0.073949
17306	Sypl2	synaptophysin-like 2	64	-0.80798	1.479566	-1.75076	0.00055034	0.073949
11781	Ap4m1	adaptor-related protein complex AP-4, mu 1, transcrip	3	-0.26536	5.729177	-1.20194	0.00055218	0.073949
52857	Gramd1a	GRAM domain containing 1A, transcript variant X6	150	-0.26528	7.974268	-1.20187	0.00056197	0.073949
68272	Rbm28	RNA binding motif protein 28	161	-0.33531	5.885569	-1.26165	0.00056549	0.073949
18971	Pold1	polymerase (DNA directed), delta 1, catalytic subunit	60	-0.36745	4.450057	-1.29007	0.00056777	0.073949
319162	H2aw	H2A.W histone	169	-0.42879	4.193614	-1.3461	0.00057099	0.073949
26360	Angptl2	angiopoietin-like 2	146	-0.51748	3.949103	-1.43145	0.00057205	0.073949
70375	Ica1l	islet cell autoantigen 1-like, transcript variant 2	179	0.352053	5.533913	1.276376	0.00057262	0.073949
15184	Hdac5	histone deacetylase 5, transcript variant 2	22	-0.21243	6.653714	-1.15864	0.00057759	0.074004
20360	Sema6c	sema domain, transmembrane domain (TM), and cyto	153	-0.22136	6.678514	-1.16583	0.0005854	0.074221
216850	Kdm6b	KDM1 lysine (K)-specific demethylase 6B	96	-0.22946	7.36073	-1.17239	0.00059246	0.074221
68488	1110002J07Rik	RIKEN cDNA 1110002J07 gene, transcript variant 1	14	-0.76405	1.866829	-1.69825	0.00059297	0.074221
67493	Mettl16	methyltransferase like 16	134	-0.3736	5.426187	-1.29558	0.00060315	0.074487
66190	Acer3	alkaline ceramidase 3, transcript variant 1	258	0.483347	3.372178	1.397983	0.00060862	0.074487
20908	Stx3	syntaxin 3, transcript variant X6	381	0.330842	5.127383	1.257747	0.00060882	0.074487
50754	Fbxw7	F-box and WD-40 domain protein 7, transcript variant	322	0.257164	6.084425	1.195127	0.00061919	0.074653

70591	5730455P16Rik	RIKEN cDNA 5730455P16 gene	214	0.310486	5.569633	1.240126	0.00062468	0.074653
210027	Slc35f3	solute carrier family 35, member F3, transcript variant	187	0.452206	3.439851	1.368131	0.00062496	0.074653
14114	Fbln1	fibulin 1, transcript variant 2	59	-0.31467	5.344209	-1.24373	0.00062853	0.074653
70887	Dmrtc1a	DMRT-like family C1a, transcript variant 2	236	1.486823	-0.35186	2.802711	0.00066175	0.076898
75769	Plppr5	phospholipid phosphatase related 5, transcript varian	346	0.319963	4.923981	1.248299	0.00066185	0.076898
12659	Ovgp1	oviductal glycoprotein 1	212	0.427657	3.598309	1.345047	0.0006656	0.076898
244810	AW551984	expressed sequence AW551984, transcript variant X	181	0.252291	7.531011	1.191097	0.00066634	0.076898
233406	Prc1	protein regulator of cytokinesis 1, transcript variant 1(	135	-0.34913	5.501988	-1.27379	0.0006818	0.077797
11861	Arl4a	ADP-ribosylation factor-like 4A, transcript variant 1	290	0.314884	6.330848	1.243912	0.00068592	0.077797
18817	Plk1	polo like kinase 1	85	-0.42448	4.44708	-1.34209	0.00068847	0.077797
22359	Vldlr	very low density lipoprotein receptor, transcript variar	296	0.283891	6.149934	1.217474	0.0006995	0.077834
12449	Ccnf	cyclin F	151	-0.47827	4.232361	-1.39307	0.00070381	0.077834
109593	Lmo3	LIM domain only 3, transcript variant 3	288	0.24066	7.368205	1.181533	0.00070787	0.077834
11496	Adam22	a disintegrin and metallopeptidase domain 22, transc	278	0.250048	6.535816	1.189246	0.00072644	0.077834
80288	Bcl9l	B cell CLL/lymphoma 9-like, transcript variant X1	9	-0.26344	7.029696	-1.20034	0.00072658	0.077834
16370	Irs4	insulin receptor substrate 4	287	0.466653	3.512902	1.3819	0.0007273	0.077834
67952	Tomm20	translocase of outer mitochondrial membrane 20	365	0.221565	7.824944	1.165997	0.00072749	0.077834
52552	Parp8	poly (ADP-ribose) polymerase family, member 8	338	0.223736	6.159153	1.167753	0.00073455	0.077834
67723	Cep83os	centrosomal protein 83, opposite strand	274	0.57381	3.008834	1.488449	0.00073553	0.077834
228869	Ncoa5	nuclear receptor coactivator 5	114	-0.2459	7.142694	-1.18583	0.00073663	0.077834
102634933	Gm26871	predicted gene, 26871, transcript variant X13	364	0.297779	6.094768	1.229251	0.00074905	0.078635
78751	Zc3h6	zinc finger CCCH type containing 6	294	0.454065	4.122904	1.369895	0.00076545	0.078921
14402	Gabrb3	gamma-aminobutyric acid (GABA) A receptor, subuni	250	0.248257	7.527027	1.187771	0.0007681	0.078921
12534	Cdk1	cyclin-dependent kinase 1	89	-0.28875	5.126417	-1.22159	0.00076902	0.078921
22099	Tsn	translin, transcript variant 2	189	0.209848	7.70231	1.156566	0.00077141	0.078921
238988	Erc2	ELKS/RAB6-interacting/CAST family member 2, trans	180	0.232847	6.844664	1.175151	0.00077601	0.078921
235542	Ppp2r3a	protein phosphatase 2, regulatory subunit B'', alpha, t	204	0.213406	6.926889	1.159422	0.0007841	0.079247
23934	Ly6h	lymphocyte antigen 6 complex, locus H, transcript var	285	0.229957	6.927353	1.1728	0.00080437	0.080794
210583	Gm4767	predicted gene 4767	190	1.872668	1.949641	3.662091	0.00082193	0.080832
56543	Kcnd3	potassium voltage-gated channel, Shal-related family	277	0.243923	6.272343	1.184208	0.00082252	0.080832
108899	2700081O15Rik	RIKEN cDNA 2700081O15 gene, transcript variant X	56	-0.23793	8.173019	-1.1793	0.0008261	0.080832
12316	Aspm	abnormal spindle microtubule assembly, transcript va	155	-0.36405	4.302307	-1.28703	0.00082702	0.080832
71101	Uvssa	UV stimulated scaffold protein A, transcript variant 4	276	0.356655	4.506047	1.280454	0.00083682	0.080832
66270	Retreg1	reticulophagy regulator 1, transcript variant 5	211	0.339163	4.601864	1.265023	0.00083933	0.080832
118567624	LOC118567624	uncharacterized LOC118567624	235	0.93139	1.039332	1.907113	0.00083951	0.080832
58807	Slco1c1	solute carrier organic anion transporter family, memb	201	0.300848	4.792866	1.231868	0.00086133	0.082438
66878	Riok3	RIO kinase 3	177	0.220449	6.862067	1.165096	0.00087053	0.082438
268890	Lsamp	limbic system-associated membrane protein, transcrip	252	0.291388	7.245954	1.223817	0.00087321	0.082438
17076	Ly75	lymphocyte antigen 75	331	0.808598	2.021612	1.751509	0.0008895	0.082438
102640519	Gm36560	predicted gene, 36560, transcript variant X14	143	-1.35455	0.011627	-2.55717	0.00088997	0.082438
235610	Atrip	ATR interacting protein	240	0.779539	2.522666	1.716582	0.00089405	0.082438
545817	Cyp2w1	cytochrome P450, family 2, subfamily w, polypeptide	168	-2.06122	-0.74347	-4.17338	0.00090554	0.082438
109620	Dsp	desmoplakin	173	-1.20245	2.585701	-2.3013	0.00090633	0.082438
319387	Adgrl3	adhesion G protein-coupled receptor L3, transcript va	267	0.250798	7.181729	1.189865	0.00091698	0.082438
80334	Kcnip4	Kv channel interacting protein 4, transcript variant X5	353	0.255866	5.553606	1.194052	0.00092114	0.082438
26436	Psg16	pregnancy specific glycoprotein 16, transcript variant	376	0.671159	2.243503	1.592352	0.00092986	0.082438
330267	Thsd7a	thrombospondin, type I, domain containing 7A	273	0.269191	5.790703	1.205132	0.00093055	0.082438
73094	Sgip1	SH3-domain GRB2-like (endophilin) interacting protei	268	0.209077	6.769138	1.155948	0.0009329	0.082438
15552	Htr1d	5-hydroxytryptamine (serotonin) receptor 1D, transcri	225	1.208398	1.439089	2.310809	0.00093549	0.082438
668303	Kif26a	kinesin family member 26A	107	-0.28611	6.548604	-1.21935	0.00093672	0.082438
12177	Bnip3l	BCL2/adenovirus E1B interacting protein 3-like, trans	300	0.189959	7.42741	1.140731	0.00095791	0.082438
330941	AI593442	expressed sequence AI593442, transcript variant 1	247	0.26682	5.807755	1.203153	0.00096127	0.082438
66864	Clec14a	C-type lectin domain family 14, member a	359	0.385041	3.591177	1.305897	0.00096824	0.082438
97212	Hadha	hydroxyacyl-CoA dehydrogenase trifunctional multier	73	-0.21854	6.389632	-1.16355	0.00097884	0.082438
228807	Zfp341	zinc finger protein 341, transcript variant X1	74	-0.32489	4.27428	-1.25257	0.00098144	0.082438
56316	Ggcx	gamma-glutamyl carboxylase	15	-0.34458	4.156656	-1.26978	0.00098157	0.082438
18798	Plcb4	phospholipase C, beta 4, transcript variant X21	354	0.26503	5.596218	1.201661	0.00098254	0.082438
16665	Krt15	keratin 15	172	-1.67226	2.102152	-3.18714	0.00098438	0.082438
72821	Scn2b	sodium channel, voltage-gated, type II, beta	11	-0.38381	4.298604	-1.30478	0.00099336	0.082438
15381	Hnrnpc	heterogeneous nuclear ribonucleoprotein C, transcrip	147	-0.23897	8.308822	-1.18015	0.00100146	0.082438
320700	A930033H14Rik	RIKEN cDNA A930033H14 gene	237	0.745714	1.651971	1.676804	0.00100304	0.082438
494468	Armcx5	armadillo repeat containing, X-linked 5	355	0.408977	3.630579	1.327744	0.00100459	0.082438
384569	Nova2	NOVA alternative splicing regulator 2	93	-0.22245	8.409884	-1.16671	0.00100692	0.082438
54392	Ncapg	non-SMC condensin I complex, subunit G	45	-0.3582	3.916784	-1.28183	0.00100828	0.082438
14218	Sh3pxd2a	SH3 and PX domains 2A, transcript variant X3	44	-0.25849	5.867834	-1.19623	0.00100993	0.082438
97827	Exd2	exonuclease 3'-5' domain containing 2	159	-0.26304	5.425058	-1.2	0.00101326	0.082438
214742	Rcor3	REST corepressor 3, transcript variant X8	348	0.311922	5.013113	1.24136	0.00102237	0.082766
12032	Bcan	brevican, transcript variant X9	264	0.30433	5.682887	1.234845	0.00104223	0.083956

108058	Camk2d	calcium/calmodulin-dependent protein kinase II, delta	244	0.327077	6.340562	1.25447	0.00104822	0.084023
11877	Arvcf	armadillo repeat gene deleted in velocardiofacial sync	13	-0.2173	6.664529	-1.16256	0.00105551	0.084193
240057	Syngap1	synaptic Ras GTPase activating protein 1 homolog (r	46	-0.21359	7.774221	-1.15957	0.00107386	0.084937
70382	Kctd2	potassium channel tetramerisation domain containing	174	0.264194	5.503774	1.200965	0.00107528	0.084937
17395	Mmp9	matrix metallopeptidase 9	4	-0.34428	4.400445	-1.26952	0.0010819	0.085047
14470	Rabac1	Rab acceptor 1 (prenylated)	293	0.284288	5.300785	1.217809	0.00109491	0.085422
20591	Kdm5c	lysine (K)-specific demethylase 5C	99	-0.22788	7.051451	-1.17111	0.00109717	0.085422
387609	Zhx2	zinc fingers and homeoboxes 2, transcript variant X2	108	-0.35035	5.151717	-1.27487	0.00111683	0.085696
268755	Mir124a-1hg	Mir124-1 host gene (non-protein coding)	113	-0.25405	8.163143	-1.19255	0.00111723	0.085696
229543	Ints3	integrator complex subunit 3, transcript variant 1	122	-0.23808	6.429402	-1.17942	0.00111982	0.085696
20927	Abcc8	ATP-binding cassette, sub-family C (CFTR/MRP), me	91	-0.30583	4.368158	-1.23613	0.00112473	0.085696
19708	Dpf2	D4, zinc and double PHD fingers family 2, transcript v	156	-0.25606	6.447621	-1.19421	0.0011298	0.085696
192236	Hps1	HPS1, biogenesis of lysosomal organelles complex 3	117	-0.36794	4.388055	-1.29051	0.00113462	0.085696
107770	Tm6sf2	transmembrane 6 superfamily member 2, transcript v	77	-1.18388	0.482768	-2.27187	0.00113756	0.085696
21973	Top2a	topoisomerase (DNA) II alpha	87	-0.27829	6.687988	-1.21276	0.00114894	0.086155
110695	Aldh7a1	aldehyde dehydrogenase family 7, member A1, trans	7	-0.27534	5.61565	-1.21028	0.001182	0.087003
230085	Phf24	PHD finger protein 24, transcript variant X9	379	0.218346	6.838123	1.163399	0.00118299	0.087003
16998	Ltbp3	latent transforming growth factor beta binding protein	97	-0.26496	5.294315	-1.2016	0.00118446	0.087003
78808	Stxbp5	syntaxin binding protein 5 (tomosyn)	368	0.326919	5.689095	1.254332	0.00119101	0.087003
23805	Apc2	APC regulator of WNT signaling pathway 2, transcrip	28	-0.19362	8.930716	-1.14363	0.00119172	0.087003
269209	Stk36	serine/threonine kinase 36, transcript variant X1	126	-0.32835	4.604609	-1.25557	0.00119767	0.087003
21752	Tert	telomerase reverse transcriptase, transcript variant 1	63	-0.45709	2.944703	-1.37277	0.00119914	0.087003
50873	Prkn	parkin RBR E3 ubiquitin protein ligase, transcript vari	384	0.549329	3.389878	1.463405	0.00120303	0.087003
50758	Fbxl17	F-box and leucine-rich repeat protein 17	321	0.215839	6.973757	1.161379	0.00121026	0.087119
414126	G630018N14Rik	RIKEN cDNA G630018N14 gene, transcript variant X	286	0.773494	1.604518	1.709404	0.00122407	0.087119
231070	Insig1	insulin induced gene 1	369	0.327983	6.487739	1.255257	0.00122956	0.087119
72668	Skida1	SKI/DACH domain containing 1	66	-0.25491	4.905865	-1.19326	0.00123294	0.087119
320865	Cdh18	cadherin 18, transcript variant X3	218	0.598971	3.563419	1.514636	0.00123583	0.087119
83671	Sytl2	synaptotagmin-like 2, transcript variant X10	241	0.518607	2.936439	1.432572	0.00123675	0.087119
241514	Zfp804a	zinc finger protein 804A	328	0.36719	4.472024	1.289839	0.00125022	0.087458
20872	Stk16	serine/threonine kinase 16, transcript variant X2	47	-0.2477	5.735122	-1.18731	0.00125611	0.087458
11669	Aldh2	aldehyde dehydrogenase 2, mitochondrial, transcript	42	-0.25859	6.196027	-1.19631	0.00125769	0.087458
16551	Kif11	kinesin family member 11	80	-0.30306	5.395479	-1.23375	0.00126893	0.087864
330149	Hfm1	HFM1, ATP-dependent DNA helicase homolog, trans	226	0.417603	3.520033	1.335706	0.00127808	0.088123
71263	Mro	maestro, transcript variant 2	310	0.468461	2.896031	1.383632	0.0012967	0.089029
105445	Dock9	dedicator of cytokinesis 9, transcript variant 3	327	0.237594	6.153242	1.179025	0.00131163	0.089579
74038	Brip1os	BRCA1 interacting protein C-terminal helicase 1, opp	109	-0.31367	5.260844	-1.24286	0.00131572	0.089579
108687	Edem2	ER degradation enhancer, mannosidase alpha-like 2,	54	-0.25486	5.105033	-1.19322	0.00132489	0.089663
12176	Bnip3	BCL2/adenovirus E1B interacting protein 3	195	0.382548	5.534327	1.303642	0.00133013	0.089663
14955	H19	H19, imprinted maternally expressed transcript, trans	148	-0.19931	8.960943	-1.14815	0.00133348	0.089663
668253	Dleu2	deleted in lymphocytic leukemia, 2	133	-0.9768	0.955533	-1.9681	0.00134532	0.089798
19281	Ptprt	protein tyrosine phosphatase, receptor type, T, transc	383	0.277949	6.784959	1.21247	0.00134653	0.089798
19042	Ppm1a	protein phosphatase 1A, magnesium dependent, alph	345	0.198985	7.505461	1.147891	0.00135408	0.089814
20719	Serpinb6a	serine (or cysteine) peptidase inhibitor, clade B, mem	192	0.315541	4.615645	1.244478	0.00136822	0.089814
224705	Vps52	VPS52 GARP complex subunit, transcript variant X2	5	-0.24111	6.192149	-1.1819	0.0013721	0.089814
58234	Shank3	SH3 and multiple ankyrin repeat domains 3	25	-0.22181	6.046687	-1.16619	0.00137617	0.089814
20983	Syt4	synaptotagmin IV	291	0.189159	8.139977	1.140099	0.00138609	0.089814
108912	Cdca2	cell division cycle associated 2, transcript variant 2	145	-0.36807	4.053216	-1.29062	0.00139252	0.089814
76580	Mib2	mindbomb E3 ubiquitin protein ligase 2, transcript var	35	-0.22292	5.801488	-1.16709	0.00140296	0.089814
17215	Mcm3	minichromosome maintenance complex component 3	115	-0.28426	5.110776	-1.21779	0.0014044	0.089814
103967	Dnm3	dynamin 3, transcript variant 1	257	0.255803	6.858474	1.194	0.0014162	0.089814
432450	Nkain2	Na+/K+ transporting ATPase interacting 2, transcript	289	0.278405	5.352843	1.212853	0.0014185	0.089814
11829	Aqp4	aquaporin 4, transcript variant 3	333	0.535089	3.226141	1.449031	0.0014291	0.089814
16682	Krt4	keratin 4	142	-1.28237	0.108473	-2.43239	0.00144043	0.089814
224997	Dlgap1	DLG associated protein 1, transcript variant 3	375	0.229229	6.30102	1.172208	0.00144319	0.089814
20467	Sin3b	transcriptional regulator, SIN3B (yeast), transcript var	261	0.216977	6.712826	1.162295	0.00144618	0.089814
29869	Ulk2	unc-51 like kinase 2	361	0.237433	7.488501	1.178893	0.00144714	0.089814
67417	Ears2	glutamyl-tRNA synthetase 2, mitochondrial	61	-0.42508	3.818262	-1.34265	0.00145365	0.089814
99011	Pomt1	protein-O-mannosyltransferase 1	157	-0.27597	4.913006	-1.21081	0.00145528	0.089814
545253	Gm5820	predicted gene 5820, transcript variant X5	199	1.26439	1.117578	2.402256	0.0014583	0.089814
224008	Spidr	scaffolding protein involved in DNA repair, transcript v	43	-0.46351	3.148394	-1.37889	0.00146271	0.089814
18749	Prkacb	protein kinase, cAMP dependent, catalytic, beta, tran	266	0.183499	8.228891	1.135635	0.0014643	0.089814
101214	Tra2a	transformer 2 alpha, transcript variant 1	136	-0.25124	7.53865	-1.19023	0.00147658	0.089814
71766	Raver1	ribonucleoprotein, PTB-binding 1	27	-0.20794	6.349166	-1.15504	0.00148566	0.089814
78752	Csgalnact2	chondroitin sulfate N-acetylgalactosaminyltransferase	238	0.36056	3.994304	1.283924	0.0014867	0.089814
27261	Dok3	docking protein 3, transcript variant X1	38	-0.56207	2.966142	-1.47639	0.00148691	0.089814
18183	Nrg3	neuregulin 3, transcript variant X9	362	0.327075	5.527428	1.254467	0.00149023	0.089814
243961	Shank1	SH3 and multiple ankyrin repeat domains 1, transcrip	29	-0.191	8.277758	-1.14156	0.00149028	0.089814

54357	Epb41l4b	erythrocyte membrane protein band 4.1 like 4b, trans	175	0.380269	4.348053	1.301584	0.00150456	0.08992
17831	Muc2	mucin 2	216	1.06554	0.63853	2.092953	0.00150935	0.08992
230793	Ahdc1	AT hook, DNA binding motif, containing 1, transcript v	24	-0.20448	6.530236	-1.15227	0.00151331	0.08992
64144	Mllt1	myeloid/lymphoid or mixed-lineage leukemia; transloc	123	-0.21307	7.198238	-1.15915	0.00151491	0.08992
101055907	Gm15246	predicted gene 15246, transcript variant X2	217	0.349594	4.035827	1.274202	0.00152174	0.08992
68298	Ncapd2	non-SMC condensin I complex, subunit D2	112	-0.31183	6.928306	-1.24128	0.0015252	0.08992
19056	Ppp3cb	protein phosphatase 3, catalytic subunit, beta isoform	299	0.168764	8.191091	1.124095	0.00153633	0.090249
236733	Usp11	ubiquitin specific peptidase 11, transcript variant 1	124	-0.21512	7.048469	-1.1608	0.00154805	0.090611
109154	Mlec	malectin	120	-0.24293	7.008286	-1.18339	0.00155458	0.090667
54131	Irf3	interferon regulatory factor 3, transcript variant X2	141	-0.33292	4.562635	-1.25956	0.00158082	0.090953
338365	Slc41a2	solute carrier family 41, member 2, transcript variant 2	351	0.366491	4.168388	1.289213	0.00158693	0.090953
15490	Hsd17b7	hydroxysteroid (17-beta) dehydrogenase 7	347	0.310299	4.535012	1.239965	0.00159081	0.090953
58178	Sorcs1	sortilin-related VPS10 domain containing receptor 1,	260	0.289414	4.945792	1.222143	0.00159622	0.090953
74165	Fbxl22	F-box and leucine-rich repeat protein 22, transcript va	158	-1.00744	0.536266	-2.01034	0.00160156	0.090953
70218	Kif18b	kinesin family member 18B	69	-0.35181	3.698652	-1.27616	0.00160287	0.090953
59025	Usp14	ubiquitin specific peptidase 14, transcript variant 2	337	0.178556	6.865461	1.13175	0.00160314	0.090953
27416	Abcc5	ATP-binding cassette, sub-family C (CFTR/MRP), me	162	-0.19353	7.194242	-1.14356	0.0016042	0.090953
434128	Pnmal2	PNMA-like 2	12	-0.17522	8.298356	-1.12914	0.00161312	0.091141
18952	Septin4	septin 4, transcript variant X12	232	0.377175	3.746203	1.298796	0.00163849	0.091926
20353	Sema4c	sema domain, immunoglobulin domain (Ig), transmem	49	-0.23316	6.557419	-1.17541	0.00164478	0.091926
66724	Tab3	TGF-beta activated kinase 1/MAP3K7 binding protein	330	0.270163	5.452512	1.205944	0.00164646	0.091926
217517	Stxbp6	syntaxin binding protein 6 (amisyn), transcript variant	185	0.265407	4.987735	1.201975	0.00165041	0.091926
12325	Camk2g	calcium/calmodulin-dependent protein kinase II gamm	262	0.220933	6.983805	1.165487	0.00165805	0.091926
16000	Igf1	insulin-like growth factor 1, transcript variant 4	256	0.341553	4.76302	1.26712	0.00166733	0.091926
116621581	Derpc	DERPC proline and glycine rich nuclear protein, trans	78	-0.22745	6.88449	-1.17076	0.00167177	0.091926
56372	1110004F10Rik	RIKEN cDNA 1110004F10 gene	272	0.193686	7.309629	1.143682	0.00167241	0.091926
213945	Col28a1	collagen, type XXVIII, alpha 1, transcript variant X3	357	0.737067	1.449335	1.666784	0.00169966	0.091926
329584	Slc2a4rg-ps	Slc2a4 regulator, pseudogene	116	-0.46891	3.843405	-1.38406	0.00170669	0.091926
26570	Slc7a11	solute carrier family 7 (cationic amino acid transporte	295	0.431691	4.097422	1.348813	0.00170971	0.091926
110956	D17H6S56E-5	DNA segment, Chr 17, human D6S56E 5	102	-0.32019	4.87905	-1.24849	0.00171361	0.091926
66830	Nacc1	nucleus accumbens associated 1, BEN and BTB (PO	121	-0.19597	6.895561	-1.14549	0.00171446	0.091926
20887	Sult1a1	sulfotransferase family 1A, phenol-preferring, membe	373	0.513046	2.74307	1.42706	0.00172035	0.091926
110265	Msra	methionine sulfoxide reductase A, transcript variant 6	230	0.243191	5.092993	1.183608	0.00172518	0.091926
79043	Spsb3	splA/ryanodine receptor domain and SOCS box conta	231	0.259989	5.243995	1.19747	0.00173561	0.091926
74464	Zswim5	zinc finger SWIM-type containing 5	92	-0.28109	5.753709	-1.21511	0.00174375	0.091926
104871	Spata7	spermatogenesis associated 7, transcript variant 1	186	0.313732	4.378098	1.242919	0.00174807	0.091926
68926	Ubap2	ubiquitin-associated protein 2	98	-0.20219	6.96566	-1.15045	0.00175444	0.091926
52589	Ncald	neurocalcin delta, transcript variant X37	200	0.208872	7.242331	1.155784	0.00175674	0.091926
68024	H2bc4	H2B clustered histone 4, transcript variant 1	41	-0.54666	2.823356	-1.4607	0.00175683	0.091926
208643	Eif4g1	eukaryotic translation initiation factor 4, gamma 1, tra	100	-0.21248	8.551627	-1.15868	0.00175905	0.091926
268859	Rbfox1	RNA binding protein, fox-1 homolog (C. elegans) 1, tr	265	0.184911	7.729539	1.136747	0.00176231	0.091926
67088	Cand2	cullin-associated and neddylation-dissociated 2 (puta	149	-0.26202	5.086737	-1.19916	0.00176259	0.091926
21849	Trim28	tripartite motif-containing 28	53	-0.16535	8.48213	-1.12143	0.00177207	0.092061
70564	Prxl2a	peroxiredoxin like 2A, transcript variant 10	191	0.287208	5.810549	1.220277	0.00177649	0.092061
231659	Gcn1	GCN1 activator of EIF2AK4	26	-0.19178	6.770491	-1.14217	0.00180919	0.092786
81905	Cacng8	calcium channel, voltage-dependent, gamma subunit	39	-0.29331	4.650862	-1.22545	0.00180967	0.092786
381126	Garem1	GRB2 associated regulator of MAPK1 subtype 1	215	0.511347	3.237115	1.42538	0.00181102	0.092786
381318	Nsl1	NSL1, MIS12 kinetochore complex component	58	-0.47784	2.852278	-1.39266	0.0018133	0.092786
18189	Nrxn1	neurexin I, transcript variant 9	246	0.180382	8.187997	1.133184	0.00182125	0.092901
239857	Cadm2	cell adhesion molecule 2, transcript variant X5	366	0.263265	6.873272	1.200192	0.0018329	0.093203
319518	Pdpr	pyruvate dehydrogenase phosphatase regulatory sub	349	0.278663	5.222275	1.21307	0.0018443	0.09349
21858	Timp2	tissue inhibitor of metalloproteinase 2	356	0.177135	7.502604	1.130637	0.00187149	0.094035
16332	Inppl1	inositol polyphosphate phosphatase-like 1, transcript	83	-0.33665	5.086628	-1.26282	0.00187258	0.094035
230959	Ajap1	adherens junction associated protein 1, transcript var	219	0.299223	4.731792	1.230481	0.00187667	0.094035
236732	Rbm10	RNA binding motif protein 10, transcript variant X1	110	-0.2229	6.338388	-1.16708	0.0018788	0.094035
20720	Serpine2	serine (or cysteine) peptidase inhibitor, clade E, mem	283	0.267071	6.739207	1.203362	0.00189519	0.094035
106042	Prickle1	prickle planar cell polarity protein 1, transcript variant	233	0.261365	4.992373	1.198612	0.00189523	0.094035
12282	Hyou1	hypoxia up-regulated 1	119	-0.19127	6.754173	-1.14177	0.00190441	0.094035
52609	Cbx7	chromobox 7	197	0.445728	3.154134	1.362001	0.00190478	0.094035
241489	Pde11a	phosphodiesterase 11A	360	0.625356	1.685811	1.542592	0.00190706	0.094035
18595	Pdgfra	platelet derived growth factor receptor, alpha polypep	253	0.265114	5.613339	1.201731	0.00192459	0.094613
102640344	A730060N03Rik	RIKEN cDNA A730060N03 gene, transcript variant X	350	0.731897	1.613725	1.660822	0.00195623	0.095774
67163	Ccdc47	coiled-coil domain containing 47	223	0.207006	6.66976	1.15429	0.00195999	0.095774
170823	Glmn	glomulin, FKBP associated protein, transcript variant	178	0.333013	4.503603	1.259642	0.00198545	0.096607
214084	Slc18a2	solute carrier family 18 (vesicular monoamine), meml	36	-0.29084	4.770827	-1.22335	0.0019889	0.096607
52468	Ctdsp2	CTD (carboxy-terminal domain, RNA polymerase II, p	138	-0.19895	6.988838	-1.14786	0.00201408	0.097539
235493	Fam214a	family with sequence similarity 214, member A, transc	307	0.211361	5.600378	1.15778	0.00202693	0.097747
67092	Gatm	glycine amidinotransferase (L-arginine:glycine amidin	213	0.267293	4.970733	1.203547	0.0020304	0.097747

26446	Psmb3	proteasome (prosome, macropain) subunit, beta type	203	0.20683	6.247426	1.15415	0.0020507	0.098002
382014	Ano8	anoctamin 8	48	-0.21318	6.491199	-1.15924	0.00205803	0.098002
228545	Vps18	VPS18 CORVET/HOPS core subunit, transcript varia	132	-0.24645	5.043899	-1.18629	0.00205955	0.098002
99633	Adgrl2	adhesion G protein-coupled receptor L2, transcript va	325	0.18377	7.994713	1.135848	0.00205978	0.098002
52666	Arhgef25	Rho guanine nucleotide exchange factor (GEF) 25, tr	101	-0.23079	6.121348	-1.17348	0.00206966	0.098185
22785	Slc30a4	solute carrier family 30 (zinc transporter), member 4,	332	0.24825	5.399264	1.187766	0.00207794	0.098291
232879	Zbtb45	zinc finger and BTB domain containing 45, transcript	71	-0.24082	4.926795	-1.18166	0.00210483	0.099275
12295	Cacnb1	calcium channel, voltage-dependent, beta 1 subunit,	95	-0.19517	6.692213	-1.14486	0.0021199	0.099696
71803	Slc25a18	solute carrier family 25 (mitochondrial carrier), memb	224	0.530393	2.919229	1.444322	0.002132	0.099733
72902	Spock3	sparc/osteonectin, cwcv and kazal-like domains prote	363	0.272899	5.445845	1.208233	0.00213294	0.099733
100043597	Srcap	Snf2-related CREBBP activator protein	130	-0.24	7.661022	-1.18099	0.00214333	0.099795
20203	S100b	S100 protein, beta polypeptide, neural	352	0.94773	0.988711	1.928835	0.00215067	0.099795
80748	BC004004	cDNA sequence BC004004, transcript variant 1	222	0.24213	5.68028	1.182737	0.00215265	0.099795
115489454	Gm52512	predicted gene, 52512	221	0.875986	2.759563	1.835262	0.00217184	0.099869
192167	Nlgn1	neuroligin 1, transcript variant 4	335	0.211854	6.186789	1.158175	0.0021836	0.099869
100041581	Zkscan16	zinc finger with KRAB and SCAN domains 16, transc	297	0.360066	3.949124	1.283485	0.00218466	0.099869
22671	Rnf112	ring finger protein 112, transcript variant 3	140	-0.27218	5.96481	-1.20763	0.00218742	0.099869
14814	Grin2d	glutamate receptor, ionotropic, NMDA2D (epsilon 4),	34	-0.2416	5.659924	-1.1823	0.00219226	0.099869
68188	Sympk	symplekin, transcript variant 1	82	-0.22605	6.751527	-1.16963	0.00220086	0.099869
14661	Glud1	glutamate dehydrogenase 1	263	0.196611	7.620222	1.146003	0.00220458	0.099869
58869	Pex5l	peroxisomal biogenesis factor 5-like, transcript varian	282	0.514727	3.028614	1.428724	0.00221335	0.099869
16664	Krt14	keratin 14, transcript variant 1	170	-1.86582	1.160387	-3.64474	0.00221403	0.099869
170757	Adgrl4	adhesion G protein-coupled receptor L4	176	0.413924	3.904244	1.332305	0.00222202	0.099869
69548	2310015A10Rik	RIKEN cDNA 2310015A10 gene	255	0.484601	3.287974	1.399199	0.00224466	0.099869
83924	Gpr137b	G protein-coupled receptor 137B, transcript variant X	320	0.263933	5.062413	1.200747	0.00224492	0.099869
67039	Rbm25	RNA binding motif protein 25, transcript variant 3	305	0.170928	7.941068	1.125783	0.00225358	0.099869
71781	Slc16a14	solute carrier family 16 (monocarboxylic acid transpo	281	0.330557	4.417989	1.257499	0.00226282	0.099869
269637	Cnpy1	canopy FGF signaling regulator 1, transcript variant 2	243	0.328078	4.813244	1.25534	0.00227353	0.099869
67538	Zswim3	zinc finger SWIM-type containing 3	139	-0.39204	3.456901	-1.31225	0.0022755	0.099869
75686	Nudt16	nudix (nucleoside diphosphate linked moiety X)-type	245	0.387315	3.485345	1.307957	0.00228183	0.099869
66197	Cks2	CDC28 protein kinase regulatory subunit 2	167	-0.41917	3.891361	-1.33716	0.00228467	0.099869
246710	Rhobtb2	Rho-related BTB domain containing 2	40	-0.19689	6.268107	-1.14623	0.00229358	0.099869
207777	Tspoap1	TSPO associated protein 1	220	0.218455	7.237267	1.163487	0.00229456	0.099869
12227	Btg2	BTG anti-proliferation factor 2	106	-0.27487	5.112127	-1.20988	0.00229789	0.099869
100043189	Gm4285	predicted gene 4285	228	0.631411	1.61341	1.549079	0.0023194	0.099869
11545	Parp1	poly (ADP-ribose) polymerase family, member 1	111	-0.25689	7.124681	-1.1949	0.00232409	0.099869
242443	Grin3a	glutamate receptor ionotropic, NMDA3A, transcript va	254	0.26211	6.189845	1.199231	0.00232557	0.099869
223732	Rtl6	retrotransposon Gag like 6	125	-0.19529	6.793325	-1.14495	0.00233213	0.099869
244867	Arhgap20	Rho GTPase activating protein 20, transcript variant	329	0.276147	5.486265	1.210956	0.0023373	0.099869
115487808	LOC115487808	uncharacterized LOC115487808, transcript variant X	88	-0.67026	2.192417	-1.59136	0.00233825	0.099869
102633156	Gm4631	predicted gene 4631, transcript variant X7	367	0.45883	3.449499	1.374427	0.00233984	0.099869
68625	Cfap57	cilia and flagella associated protein 57	164	-0.78296	1.371618	-1.72065	0.00234299	0.099869
11864	Arnt2	aryl hydrocarbon receptor nuclear translocator 2, tran	205	0.170654	8.419239	1.125569	0.00235026	0.099869
192187	Stab1	stabilin 1	62	-0.33729	4.748995	-1.26338	0.00235215	0.099869
71207	Nudt4	nudix (nucleoside diphosphate linked moiety X)-type	229	0.175065	7.358583	1.129015	0.00235566	0.099869
26950	Vsnl1	visinin-like 1, transcript variant 1	188	0.207406	6.375079	1.15461	0.0023568	0.099869
13688	Eif4ebp2	eukaryotic translation initiation factor 4E binding prote	NA	-0.40396	5.316931	-1.32313	0.00237214	0.100225
68925	Rpap1	RNA polymerase II associated protein 1, transcript va	NA	-0.29898	4.587721	-1.23028	0.00237751	0.100225
94229	Slc4a10	solute carrier family 4, sodium bicarbonate cotranspo	NA	0.319984	5.292257	1.248316	0.00239271	0.100605
20320	Nptn	neuroplastin, transcript variant 1	NA	0.16641	7.345063	1.122262	0.00240625	0.100914
14479	Usp15	ubiquitin specific peptidase 15, transcript variant 2	NA	0.209706	6.571566	1.156453	0.00241394	0.100976
22691	Zscan2	zinc finger and SCAN domain containing 2, transcript	NA	-0.25861	5.031316	-1.19633	0.00242095	0.101009
19266	Ptprd	protein tyrosine phosphatase, receptor type, D, trans	NA	0.189614	7.990064	1.140458	0.00244083	0.101335
102141	Snx25	sorting nexin 25, transcript variant 3	NA	0.272144	4.929802	1.207602	0.00244653	0.101335
13400	Dmpk	dystrophia myotonica-protein kinase, transcript variar	NA	-0.31402	4.341895	-1.24317	0.0024587	0.101335
18536	Pcm1	pericentriolar material 1	NA	0.20475	7.217028	1.152486	0.0024629	0.101335
53322	Nucb2	nucleobindin 2, transcript variant X1	NA	0.302696	4.379056	1.233448	0.00246676	0.101335
18573	Pde1a	phosphodiesterase 1A, calmodulin-dependent, transc	NA	0.380689	3.955739	1.301963	0.00246788	0.101335
14415	Gad1	glutamate decarboxylase 1, transcript variant 1	NA	0.196611	7.302348	1.146003	0.00248134	0.101335
244418	Prag1	PEAK1 related kinase activating pseudokinase 1	NA	-0.23062	5.496155	-1.17334	0.00248313	0.101335
269109	Dpp10	dipeptidylpeptidase 10	NA	0.311676	5.83963	1.241149	0.00248479	0.101335
12831	Col5a1	collagen, type V, alpha 1	NA	-0.23538	5.851132	-1.17722	0.00250049	0.101532
213262	Fstl5	follistatin-like 5, transcript variant 2	NA	0.274271	5.898212	1.209383	0.0025093	0.101532
11816	Apoe	apolipoprotein E, transcript variant 1	NA	0.323525	7.081454	1.251384	0.00251354	0.101532
208846	Daam1	dishevelled associated activator of morphogenesis 1,	NA	0.172355	7.257946	1.126897	0.00251458	0.101532
13731	Emp2	epithelial membrane protein 2	NA	0.293452	4.137788	1.225569	0.00252097	0.101538
381148	Prob1	proline rich basic protein 1	NA	-0.45698	2.634985	-1.37266	0.00254537	0.102049
73804	Kif2c	kinesin family member 2C, transcript variant 1	NA	-0.33967	4.116162	-1.26547	0.00255095	0.102049

380669	Lin28b	lin-28 homolog B (C. elegans), transcript variant X2	NA	0.358516	4.528832	1.282106	0.00255247	0.102049
207952	Klhl25	kelch-like 25, transcript variant 2	NA	-0.24096	5.06235	-1.18178	0.00256421	0.102086
52906	Ahi1	Abelson helper integration site 1, transcript variant 1	NA	0.193843	7.84938	1.143806	0.00256596	0.102086
192192	Shkbp1	Sh3kbp1 binding protein 1	NA	-0.23552	5.296553	-1.17733	0.00258144	0.102288
101490	Inpp5f	inositol polyphosphate-5-phosphatase F, transcript va	NA	0.161403	8.761482	1.118374	0.00258785	0.102288
630579	Zfp808	zinc finger protein 808	NA	-0.72435	1.719542	-1.65215	0.00258989	0.102288
227292	Ctdsp1	CTD (carboxy-terminal domain, RNA polymerase II, p	NA	-0.23823	5.580983	-1.17955	0.00259782	0.102309
74777	Selenon	selenoprotein N	NA	-0.18108	6.187978	-1.13373	0.00260374	0.102309
230098	Arhgef39	Rho guanine nucleotide exchange factor (GEF) 39	NA	-0.52248	2.160248	-1.43642	0.00262067	0.102309
12416	Cbx2	chromobox 2	NA	-0.27233	5.338814	-1.20776	0.00262127	0.102309
228357	Lrp4	low density lipoprotein receptor-related protein 4, tran	NA	-0.23199	4.876126	-1.17445	0.00262227	0.102309
16508	Kcnd2	potassium voltage-gated channel, Shal-related family	NA	0.293883	6.413504	1.225935	0.00263525	0.102309
104069	Sncb	synuclein, beta, transcript variant 1	NA	0.221197	6.330648	1.1657	0.00264373	0.102309
219228	Pcdh17	protocadherin 17, transcript variant X1	NA	0.236449	6.867843	1.178089	0.00264456	0.102309
108143	Taf9	TATA-box binding protein associated factor 9, transcr	NA	0.181515	6.868687	1.134074	0.00265145	0.102309
109272	Mybpc1	myosin binding protein C, slow-type, transcript varian	NA	-0.47188	2.938227	-1.38691	0.00265599	0.102309
394435	Ugt1a6b	UDP glucuronosyltransferase 1 family, polypeptide A	NA	1.389574	1.403672	2.620012	0.00266222	0.102309
19366	Rad54l	RAD54 like (S. cerevisiae), transcript variant X1	NA	-0.42938	3.187747	-1.34666	0.00266588	0.102309
330260	Pon2	paraoxonase 2	NA	0.272222	4.471489	1.207667	0.0027104	0.103229
378702	Serf2	small EDRK-rich factor 2, transcript variant 2	NA	-0.17794	6.850039	-1.13127	0.00271629	0.103229
30938	Fgd3	FYVE, RhoGEF and PH domain containing 3, transcr	NA	-0.30552	4.402192	-1.23587	0.00271922	0.103229
72981	Thap12	THAP domain containing 12, transcript variant 1	NA	0.212644	6.648869	1.15881	0.00272065	0.103229
66977	Nuf2	NUF2, NDC80 kinetochore complex component, tran	NA	-0.31877	4.371993	-1.24726	0.00272593	0.103229
21928	Tnfaip2	tumor necrosis factor, alpha-induced protein 2	NA	-0.90818	0.403329	-1.87667	0.00273267	0.103229
243621	Iqsec3	IQ motif and Sec7 domain 3, transcript variant 2	NA	0.22925	6.006821	1.172225	0.00273607	0.103229
105244828	Gm40364	predicted gene, 40364	NA	-2.91741	-1.22675	-7.55491	0.00274223	0.103229
231861	Tnrc18	trinucleotide repeat containing 18, transcript variant X	NA	-0.18388	8.229408	-1.13594	0.00275681	0.103229
319984	Jph4	junctophilin 4	NA	-0.1744	8.124071	-1.12849	0.00275831	0.103229
14766	Adgrg1	adhesion G protein-coupled receptor G1, transcript va	NA	-0.16838	7.591903	-1.1238	0.00275964	0.103229
50876	Tmod2	tropomodulin 2, transcript variant X1	NA	0.183191	8.364946	1.135392	0.00279224	0.104013
100042519	Gm3883	predicted gene 3883	NA	2.452992	-0.88607	5.475506	0.00279405	0.104013
56380	Arid3b	AT rich interactive domain 3B (BRIGHT-like), transcri	NA	-0.3737	3.324555	-1.29567	0.00279976	0.104013
209011	Sirt7	sirtuin 7, transcript variant 1	NA	-0.26129	4.662188	-1.19855	0.00280935	0.104132
234353	Psd3	pleckstrin and Sec7 domain containing 3, transcript v	NA	0.20725	6.033372	1.154486	0.00282037	0.104302
66949	Trim59	tripartite motif-containing 59	NA	-0.20653	5.684518	-1.15391	0.00286133	0.105555
16475	Ajuba	ajuba LIM protein	NA	-0.29848	3.907802	-1.22985	0.00287102	0.105555
20662	Sos1	SOS Ras/Rac guanine nucleotide exchange factor 1,	NA	0.189165	6.48051	1.140104	0.00287606	0.105555
72750	Fam117b	family with sequence similarity 117, member B	NA	0.164253	7.588543	1.120586	0.00288019	0.105555
21750	Terf2	telomeric repeat binding factor 2, transcript variant X	NA	-0.1843	6.370249	-1.13627	0.00289291	0.105783
68203	Diras2	DIRAS family, GTP-binding RAS-like 2, transcript var	NA	0.199916	5.542538	1.148631	0.00290189	0.105874
100042178	Prdm16os	Prdm16 opposite strand transcript	NA	-0.64815	2.620602	-1.56716	0.00291455	0.105989
13549	Dyrk1b	dual-specificity tyrosine-(Y)-phosphorylation regulate	NA	-0.20662	6.760256	-1.15398	0.00292644	0.105989
78784	Celf3	CUGBP, Elav-like family member 3, transcript variant	NA	-0.19564	7.870134	-1.14523	0.0029302	0.105989
20818	Srprb	signal recognition particle receptor, B subunit	NA	-0.22148	5.60556	-1.16593	0.00293111	0.105989
239017	Ogdhl	oxoglutarate dehydrogenase-like	NA	-0.23306	5.123392	-1.17533	0.00294379	0.106145
240334	Pcyox1l	prenylcysteine oxidase 1 like	NA	-0.27581	4.991888	-1.21067	0.00294846	0.106145
74111	Rbm19	RNA binding motif protein 19	NA	-0.29869	4.370556	-1.23003	0.00295617	0.106187
23948	Mmp17	matrix metallopeptidase 17	NA	-0.23818	5.366222	-1.1795	0.00298332	0.106926
320795	Pkn1	protein kinase N1, transcript variant X1	NA	-0.25494	7.016628	-1.19329	0.00300314	0.107219
14407	Gabrg3	gamma-aminobutyric acid (GABA) A receptor, subuni	NA	0.440747	3.528579	1.357307	0.00300466	0.107219
434232	Iqck	IQ motif containing K, transcript variant 2	NA	0.927785	0.426867	1.902353	0.00301355	0.107246
73181	Nfatc4	nuclear factor of activated T cells, cytoplasmic, calcin	NA	-0.31623	4.270471	-1.24507	0.00301861	0.107246
228071	Sestd1	SEC14 and spectrin domains 1	NA	0.172946	7.633795	1.127359	0.00303638	0.107642
11931	Atp1b1	ATPase, Na+/K+ transporting, beta 1 polypeptide	NA	0.229676	7.881675	1.172571	0.00307331	0.108485
13614	Edn1	endothelin 1	NA	0.839971	0.866426	1.790014	0.00307348	0.108485
224824	Pex6	peroxisomal biogenesis factor 6, transcript variant 1	NA	-0.25072	5.228233	-1.1898	0.00308842	0.108776
18549	Pcsk2	proprotein convertase subtilisin/kexin type 2	NA	0.250787	5.769656	1.189856	0.00314872	0.110187
14560	Gdf10	growth differentiation factor 10	NA	0.301147	3.897899	1.232124	0.00315128	0.110187
20475	Six5	sine oculis-related homeobox 5	NA	-0.4382	3.263039	-1.35492	0.00315444	0.110187
20878	Aurka	aurora kinase A, transcript variant 2	NA	-0.33297	3.949727	-1.2596	0.00315555	0.110187
229007	Zgpat	zinc finger, CCCH-type with G patch domain, transcri	NA	-0.29689	5.261863	-1.22849	0.00316858	0.110405
433926	Lrrc8b	leucine rich repeat containing 8 family, member B, tra	NA	0.244992	6.277047	1.185086	0.00320912	0.110513
66646	Rpe	ribulose-5-phosphate-3-epimerase, transcript variant	NA	-0.28586	4.689931	-1.21914	0.00321377	0.110513
20512	Slc1a3	solute carrier family 1 (glial high affinity glutamate tra	NA	0.174407	7.565031	1.1285	0.00321526	0.110513
93699	Pcdhgb1	protocadherin gamma subfamily B, 1	NA	-0.33803	3.751967	-1.26403	0.00322654	0.110513
67333	Stk35	serine/threonine kinase 35, transcript variant 1	NA	-0.2641	4.969624	-1.20089	0.00322976	0.110513
68635	1110025M09Rik	RIKEN cDNA 1110025M09 gene	NA	0.690409	1.514316	1.613741	0.00323423	0.110513
208198	Btbd2	BTB (POZ) domain containing 2, transcript variant 2	NA	-0.20774	6.202571	-1.15487	0.00323432	0.110513

545474	Scrt2	scratch family zinc finger 2	NA	-0.28348	6.558819	-1.21712	0.00324667	0.110513
378430	Nanos2	nanos C2HC-type zinc finger 2	NA	0.622169	1.75662	1.539187	0.00325538	0.110513
28185	Tomm70a	translocase of outer mitochondrial membrane 70A	NA	0.210573	7.500266	1.157148	0.00325973	0.110513
319996	Golm2	golgi membrane protein 2, transcript variant 5	NA	0.190988	6.980462	1.141545	0.00326094	0.110513
18008	Nes	nestin	NA	-0.35663	7.341552	-1.28043	0.0032618	0.110513
118452	Baalc	brain and acute leukemia, cytoplasmic, transcript vari	NA	0.248215	4.770105	1.187737	0.00327002	0.110513
380959	Alg10b	asparagine-linked glycosylation 10B (alpha-1,2-gluco	NA	0.688926	4.46059	1.612083	0.00327123	0.110513
14936	Gys1	glycogen synthase 1, muscle	NA	-0.25437	4.7947	-1.19282	0.00327656	0.110513
13211	Dhx9	DEAH (Asp-Glu-Ala-His) box polypeptide 9, transcrip	NA	-0.18682	8.197836	-1.13825	0.00328034	0.110513
12235	Bub1	BUB1, mitotic checkpoint serine/threonine kinase, tra	NA	-0.33247	4.247642	-1.25917	0.00329823	0.110886
23908	Hs2st1	heparan sulfate 2-O-sulfotransferase 1, transcript var	NA	0.166777	6.798348	1.122548	0.00330864	0.111006
22004	Tpm2	tropomyosin 2, beta, transcript variant X1	NA	-0.42701	3.962468	-1.34445	0.00332858	0.111446
14248	Flii	flightless I actin binding protein, transcript variant 1	NA	-0.19348	6.298025	-1.14352	0.00333999	0.111498
52202	Rbm34	RNA binding motif protein 34, transcript variant 2	NA	0.246599	4.822299	1.186407	0.00334935	0.111498
242748	Disp3	dispatched RND transporter family member 3, transcı	NA	-0.26287	5.090914	-1.19986	0.00336214	0.111498
241688	Dzank1	double zinc ribbon and ankyrin repeat domains 1, traı	NA	0.375389	5.342211	1.297189	0.00336379	0.111498
70296	Tbc1d13	TBC1 domain family, member 13	NA	-0.20468	6.355476	-1.15243	0.00336441	0.111498
320816	Ankrd16	ankyrin repeat domain 16, transcript variant X12	NA	0.294768	5.612668	1.226688	0.00338216	0.111636
238037	Wdcp	WD repeat and coiled coil containing, transcript variaı	NA	-0.3531	3.591988	-1.2773	0.00338577	0.111636
16907	Lmnb2	lamin B2, transcript variant 1	NA	-0.1986	5.818037	-1.14759	0.00340123	0.111636
17968	Ncam2	neural cell adhesion molecule 2, transcript variant X1	NA	0.261096	5.666702	1.198389	0.00340264	0.111636
193043	Zfp3	zinc finger protein 3	NA	0.343526	3.43853	1.268854	0.00340287	0.111636
17918	Myo5a	myosin VA, transcript variant X8	NA	0.18889	7.733147	1.139887	0.0034189	0.111936
271305	Phf21b	PHD finger protein 21B, transcript variant 3	NA	-0.23641	6.364049	-1.17806	0.00342973	0.111948
26900	Ddx3y	DEAD box helicase 3, Y-linked	NA	0.541671	4.544534	1.455657	0.00343302	0.111948
15507	Hspb1	heat shock protein 1	NA	-0.61753	2.567432	-1.53425	0.00344514	0.112008
229791	Plppr4	phospholipid phosphatase related 4	NA	0.244327	5.629421	1.18454	0.00346062	0.112008
20254	Scg2	secretogranin II, transcript variant 1	NA	0.225219	6.832834	1.168955	0.00346252	0.112008
234366	Gatad2a	GATA zinc finger domain containing 2A, transcript va	NA	-0.23592	6.130928	-1.17766	0.00347627	0.112008
19346	Rab6a	RAB6A, member RAS oncogene family, transcript va	NA	0.15685	8.233152	1.11485	0.00348078	0.112008
76787	Ppfia3	protein tyrosine phosphatase, receptor type, f polype	NA	-0.1676	6.44754	-1.12318	0.00348406	0.112008
69137	Vstm5	V-set and transmembrane domain containing 5	NA	0.368244	3.882636	1.290781	0.00348501	0.112008
242584	Wdr78	WD repeat domain 78	NA	-0.40066	3.312457	-1.32011	0.00348993	0.112008
23807	Arih2	ariadne RBR E3 ubiquitin protein ligase 2, transcript v	NA	0.207278	5.627496	1.154508	0.0035184	0.112618
19139	Prps1	phosphoribosyl pyrophosphate synthetase 1	NA	0.192955	5.836823	1.143103	0.00352278	0.112618
14027	Evpl	envoplakin	NA	-0.77505	1.686532	-1.71125	0.00354202	0.112843
53886	Cdkl2	cyclin-dependent kinase-like 2 (CDC2-related kinase)	NA	0.321134	4.249337	1.249312	0.00355582	0.112843
57247	Zfp276	zinc finger protein (C2H2 type) 276	NA	-0.24321	4.632328	-1.18362	0.00355678	0.112843
243300	Nyap1	neuronal tyrosine-phosphorylated phosphoinositide 3	NA	-0.16751	7.866391	-1.12312	0.00356788	0.112843
244144	Usp35	ubiquitin specific peptidase 35, transcript variant X4	NA	-0.3174	4.229736	-1.24609	0.00357142	0.112843
16440	Itpr3	inositol 1,4,5-triphosphate receptor 3, transcript variaı	NA	0.471352	2.769287	1.386408	0.00357143	0.112843
22129	Ttc3	tetratricopeptide repeat domain 3, transcript variant X	NA	0.186125	9.839259	1.137704	0.00358728	0.112927
26422	Nbea	neurobeachin, transcript variant X22	NA	0.155491	8.299399	1.113801	0.00358993	0.112927
68918	1190005I06Rik	RIKEN cDNA 1190005I06 gene	NA	0.816526	1.495245	1.76116	0.0036059	0.112927
63985	Gmfb	glia maturation factor, beta	NA	0.200435	7.417042	1.149045	0.00360966	0.112927
14804	Grid2	glutamate receptor, ionotropic, delta 2, transcript vari	NA	0.363856	4.275918	1.286861	0.00362539	0.112927
21828	Thbs4	thrombospondin 4	NA	-0.54326	2.048541	-1.45727	0.00363403	0.112927
17283	Men1	multiple endocrine neoplasia 1, transcript variant 3	NA	-0.17277	6.234738	-1.12722	0.00363501	0.112927
228960	Stx16	syntaxin 16, transcript variant 8	NA	-0.22461	6.558644	-1.16846	0.00363902	0.112927
15357	Hmgcr	3-hydroxy-3-methylglutaryl-Coenzyme A reductase, tı	NA	0.162842	8.037876	1.119491	0.00364687	0.112927
14394	Gabra1	gamma-aminobutyric acid (GABA) A receptor, subuni	NA	0.268955	4.620556	1.204935	0.00365481	0.112927
24064	Spry2	sprouty RTK signaling antagonist 2	NA	0.256124	4.893202	1.194266	0.00365639	0.112927
11815	Apod	apolipoprotein D, transcript variant 1	NA	0.204935	5.450792	1.152635	0.00365735	0.112927
104401	Pcnx3	pecanex homolog 3, transcript variant X7	NA	-0.16991	6.285802	-1.12499	0.00366906	0.113
234396	Ankle1	ankyrin repeat and LEM domain containing 1, transcr	NA	-0.68418	2.860268	-1.60679	0.00368324	0.113
77976	Nuak1	NUAK family, SNF1-like kinase, 1, transcript variant X	NA	-0.24451	6.89712	-1.18469	0.00368551	0.113
74103	Nebl	nebulette, transcript variant 5	NA	0.438343	4.176441	1.355047	0.00368751	0.113
171212	Galnt10	polypeptide N-acetylgalactosaminyltransferase 10	NA	-0.3584	4.742918	-1.282	0.00369974	0.113162
16007	Ccn1	cellular communication network factor 1	NA	-0.35919	3.349514	-1.2827	0.00371445	0.11334
494448	Cbx6	chromobox 6	NA	-0.15205	7.444062	-1.11115	0.00371968	0.11334
70005	Znf41-ps	ZNF41, pseudogene	NA	0.628959	3.058813	1.546448	0.00372646	0.11334
72927	Hepacam	hepatocyte cell adhesion molecule	NA	0.34212	4.384378	1.267618	0.00374684	0.113426
110595	Timp4	tissue inhibitor of metalloproteinase 4, transcript varia	NA	0.524666	2.373857	1.4386	0.00375011	0.113426
224938	Pja2	praja ring finger ubiquitin ligase 2, transcript variant 2	NA	0.189673	8.03171	1.140505	0.00376663	0.113426
246104	Rhbdl3	rhomboid like 3	NA	-0.38184	4.524918	-1.303	0.00376993	0.113426
19186	Psme1	proteasome (prosome, macropain) activator subunit 1	NA	0.188439	6.27465	1.13953	0.00377977	0.113426
56430	Clip1	CAP-GLY domain containing linker protein 1, transcri	NA	0.158401	7.040272	1.116049	0.00379913	0.113426
103012	Firre	functional intergenic repeating RNA element	NA	0.237871	5.629936	1.179251	0.00380108	0.113426

100503002	Gm38424	predicted gene, 38424, transcript variant X3	NA	-0.5985	1.829755	-1.51414	0.00380822	0.113426
12842	Col1a1	collagen, type I, alpha 1	NA	-0.41908	8.039648	-1.33708	0.0038107	0.113426
20603	Sms	spermine synthase, transcript variant 2	NA	0.16218	7.665563	1.118977	0.00381716	0.113426
68180	Hyi	hydroxypyruvate isomerase (putative), transcript varia	NA	0.525013	2.744973	1.438946	0.00381826	0.113426
16542	Kdr	kinase insert domain protein receptor, transcript varia	NA	0.192671	5.860691	1.142878	0.00382306	0.113426
66158	Rtl8a	retrotransposon Gag like 8A	NA	0.17165	6.822568	1.126346	0.00383931	0.113426
68655	Fndc1	fibronectin type III domain containing 1, transcript var	NA	0.397689	3.399213	1.317396	0.00384213	0.113426
12405	Cbln2	cerebellin 2 precursor protein, transcript variant 5	NA	0.220007	5.950964	1.16474	0.00386518	0.113426
14886	Gtf2i	general transcription factor II I, transcript variant 11	NA	-0.18388	8.300371	-1.13594	0.00386521	0.113426
73852	D3Ertd751e	DNA segment, Chr 3, ERATO Doi 751, expressed, tra	NA	-0.42824	2.737253	-1.34559	0.00387355	0.113426
106763	Ttbk1	tau tubulin kinase 1, transcript variant X2	NA	-0.18799	6.962743	-1.13918	0.00387369	0.113426
76187	Adhfe1	alcohol dehydrogenase, iron containing, 1, transcript	NA	0.518822	2.487303	1.432785	0.00387566	0.113426
329739	Fam102b	family with sequence similarity 102, member B	NA	0.242844	6.525105	1.183323	0.0038778	0.113426
52250	Reep1	receptor accessory protein 1	NA	0.169119	8.442818	1.124372	0.00388347	0.113426
22377	Wbp1	WW domain binding protein 1, transcript variant 1	NA	-0.2095	5.935471	-1.15629	0.00390554	0.113426
268697	Ccnb1	cyclin B1	NA	-0.28657	5.284671	-1.21974	0.00390571	0.113426
15499	Hsf1	heat shock factor 1, transcript variant 3	NA	-0.26288	5.134538	-1.19987	0.00391373	0.113426
207227	Stxbp5l	syntaxin binding protein 5-like, transcript variant X14	NA	0.304851	4.341754	1.235291	0.00391848	0.113426
170728	Rtn4ip1	reticulon 4 interacting protein 1, transcript variant 1	NA	-0.33887	3.725938	-1.26476	0.00393574	0.113426
319752	B230209E15Rik	RIKEN cDNA B230209E15 gene	NA	0.419472	3.028015	1.337438	0.00393873	0.113426
72699	Lime1	Lck interacting transmembrane adaptor 1	NA	-0.24876	5.673302	-1.18818	0.00394603	0.113426
11924	Neurog2	neurogenin 2, transcript variant X1	NA	-0.36204	5.54688	-1.28524	0.00395493	0.113426
17847	Usp34	ubiquitin specific peptidase 34, transcript variant X7	NA	0.233303	7.408147	1.175523	0.00395704	0.113426
74106	Dcaf6	DDB1 and CUL4 associated factor 6	NA	0.206722	5.531912	1.154063	0.003959	0.113426
114615	Elac1	elaC ribonuclease Z 1	NA	0.259239	4.353015	1.196848	0.0039667	0.113426
71838	Phf7	PHD finger protein 7, transcript variant 1	NA	0.380149	3.076727	1.301476	0.00396807	0.113426
208908	Ccdc62	coiled-coil domain containing 62, transcript variant X1	NA	0.470393	2.501489	1.385487	0.00397033	0.113426
432442	Akap7	A kinase (PRKA) anchor protein 7, transcript variant 2	NA	0.225026	5.459936	1.168798	0.00398374	0.113426
67307	Pbld2	phenazine biosynthesis-like protein domain containin	NA	0.630908	1.423754	1.548539	0.00398406	0.113426
18772	Pkp1	plakophilin 1, transcript variant 1	NA	-1.52375	0.792612	-2.87538	0.00399084	0.113426
320563	Islr2	immunoglobulin superfamily containing leucine-rich re	NA	-0.20024	9.431674	-1.14889	0.00399803	0.113426
259300	Ehd2	EH-domain containing 2, transcript variant X1	NA	-0.40599	4.165929	-1.325	0.00400114	0.113426
27206	Nrk	Nik related kinase, transcript variant X1	NA	-0.4322	3.033139	-1.34929	0.00401572	0.113603
216049	Zfp365	zinc finger protein 365	NA	0.379287	3.560358	1.300699	0.00402903	0.113603
13510	Dsg1a	desmoglein 1 alpha, transcript variant X1	NA	-2.04797	-0.6089	-4.13522	0.00403544	0.113603
26420	Mapk9	mitogen-activated protein kinase 9, transcript variant	NA	0.21096	6.992343	1.157458	0.00405952	0.113603
116837	Rims1	regulating synaptic membrane exocytosis 1, transcrip	NA	0.220222	5.924702	1.164912	0.00406061	0.113603
68117	Apool	apolipoprotein O-like, transcript variant 1	NA	-0.40877	3.371309	-1.32755	0.00406082	0.113603
13641	Efnb1	ephrin B1	NA	-0.26969	6.463556	-1.20555	0.00406138	0.113603
227699	Nup188	nucleoporin 188, transcript variant 1	NA	-0.22296	5.660314	-1.16712	0.00406325	0.113603
56421	Pfkp	phosphofructokinase, platelet, transcript variant X7	NA	0.204374	8.085123	1.152186	0.0040742	0.113714
20586	Smarca4	SWI/SNF related, matrix associated, actin dependent	NA	-0.1473	8.796952	-1.10749	0.00409668	0.113822
214575	Tdrd5	tudor domain containing 5, transcript variant 2	NA	0.812781	0.643302	1.756594	0.00409687	0.113822
227399	Ppip5k2	diphosphoinositol pentakisphosphate kinase 2, transc	NA	0.19745	6.625038	1.146669	0.00411919	0.113822
116847	Prelp	proline arginine-rich end leucine-rich repeat	NA	-0.34482	3.609263	-1.26999	0.00411973	0.113822
118568556	LOC118568556	uncharacterized LOC118568556	NA	0.927148	0.353273	1.901513	0.00413672	0.113822
105247050	Gm42226	predicted gene, 42226	NA	-0.28153	4.25091	-1.21548	0.00413858	0.113822
67579	Cpeb4	cytoplasmic polyadenylation element binding protein	NA	0.234493	6.855779	1.176494	0.0041496	0.113822
224727	Bag6	BCL2-associated athanogene 6, transcript variant X1	NA	-0.16091	8.372545	-1.11799	0.00415055	0.113822
217705	Fam161b	family with sequence similarity 161, member B, transc	NA	-0.29131	4.166645	-1.22375	0.00415416	0.113822
16801	Arhgef1	Rho guanine nucleotide exchange factor (GEF) 1, tra	NA	-0.21364	6.377387	-1.15961	0.00415511	0.113822
98170	Tmem132a	transmembrane protein 132A, transcript variant 1	NA	-0.16631	7.946846	-1.12218	0.00416066	0.113822
77097	Tanc2	tetratricopeptide repeat, ankyrin repeat and coiled-co	NA	0.182651	7.295953	1.134968	0.00417275	0.113822
319263	Pcmtd1	protein-L-isoaspartate (D-aspartate) O-methyltransfer	NA	0.194427	6.263215	1.14427	0.00417285	0.113822
20541	Slc8a1	solute carrier family 8 (sodium/calcium exchanger), m	NA	0.176332	7.345178	1.130007	0.00417599	0.113822
216613	Ccdc85a	coiled-coil domain containing 85A, transcript variant X	NA	0.212985	5.657123	1.159084	0.00420063	0.113949
331532	Tceal5	transcription elongation factor A (SII)-like 5, transcript	NA	0.257199	4.594176	1.195156	0.00420648	0.113949
16009	Igfbp3	insulin-like growth factor binding protein 3	NA	0.289403	5.604045	1.222134	0.00420709	0.113949
268354	Tafa2	TAFA chemokine like family member 2, transcript var	NA	0.211755	6.874583	1.158096	0.00420866	0.113949
19736	Rgs4	regulator of G-protein signaling 4	NA	0.20096	5.949837	1.149463	0.00422357	0.114163
27999	Fam3c	family with sequence similarity 3, member C	NA	0.215932	6.315013	1.161454	0.00425622	0.114773
215654	Cdh12	cadherin 12, transcript variant X8	NA	0.311869	4.109739	1.241314	0.00426027	0.114773
18612	Etv4	ets variant 4, transcript variant X7	NA	0.346695	3.586904	1.271644	0.00427538	0.11499
321008	Zswim9	zinc finger SWIM-type containing 9	NA	-0.36487	4.06343	-1.28777	0.00428964	0.115183
226169	Pprc1	peroxisome proliferative activated receptor, gamma, c	NA	-0.23313	5.34954	-1.17538	0.00430578	0.115236
434234	Rexo5	RNA exonuclease 5, transcript variant X7	NA	-0.43457	2.975362	-1.35151	0.00431122	0.115236
208715	Hmgcs1	3-hydroxy-3-methylglutaryl-Coenzyme A synthase 1,	NA	0.155672	9.441268	1.11394	0.00431285	0.115236
20249	Scd1	stearoyl-Coenzyme A desaturase 1	NA	0.295427	5.137535	1.227248	0.00432816	0.115383

20104	Rps6	ribosomal protein S6	NA	-0.25253	4.938274	-1.1913	0.00433255	0.115383
19714	Rev3l	REV3 like, DNA directed polymerase zeta catalytic su	NA	0.218561	7.378612	1.163573	0.00433975	0.115386
17289	Mertk	MER proto-oncogene tyrosine kinase, transcript varia	NA	0.438103	2.617186	1.354821	0.00435174	0.115487
104383	Rcor2	REST corepressor 2, transcript variant 2	NA	-0.20676	6.69724	-1.15409	0.004365	0.115487
67358	1700093K21Rik	RIKEN cDNA 1700093K21 gene, transcript variant 1	NA	1.116434	-0.09202	2.168104	0.00436696	0.115487
80294	Pofut2	protein O-fucosyltransferase 2	NA	-0.20516	5.239314	-1.15281	0.00438084	0.115487
14376	Ganab	alpha glucosidase 2 alpha neutral subunit, transcript	NA	-0.16742	7.463504	-1.12305	0.00438177	0.115487
100042149	Gm3696	predicted gene 3696, transcript variant 3	NA	0.413686	3.664427	1.332085	0.00439263	0.115487
21367	Cntn2	contactin 2, transcript variant X1	NA	-0.20448	8.424246	-1.15227	0.00439322	0.115487
227800	Rabgap1	RAB GTPase activating protein 1, transcript variant 5	NA	0.170344	7.046158	1.125327	0.00442335	0.115908
18854	Pml	promyelocytic leukemia, transcript variant 1	NA	-0.35633	3.687283	-1.28017	0.00442348	0.115908
18829	Ccl21a	chemokine (C-C motif) ligand 21A (serine)	NA	-1.73072	-0.96499	-3.31894	0.00443256	0.115959
68612	Ube2c	ubiquitin-conjugating enzyme E2C	NA	-0.28927	4.883658	-1.22202	0.00446481	0.116462
20729	Spin1	spindlin 1, transcript variant 1	NA	0.14003	8.465962	1.101928	0.00446611	0.116462
75404	Arhgap36	Rho GTPase activating protein 36, transcript variant X	NA	0.382311	3.024385	1.303428	0.00451418	0.117528
238328	Vash1	vasohibin 1	NA	-0.22038	8.155464	-1.16504	0.00453262	0.117639
11501	Adam8	a disintegrin and metallopeptidase domain 8, transcri	NA	-0.47409	2.713355	-1.38904	0.00453538	0.117639
14235	Foxm1	forkhead box M1	NA	-0.35058	4.970353	-1.27508	0.00454014	0.117639
64291	Osbpl1a	oxysterol binding protein-like 1A, transcript variant 7	NA	0.205711	5.472782	1.153254	0.00455155	0.117681
64009	Syne1	spectrin repeat containing, nuclear envelope 1, transc	NA	0.193848	5.805309	1.14381	0.00456784	0.117681
211548	Nomo1	nodal modulator 1	NA	-0.17141	6.499101	-1.12616	0.00456929	0.117681
74182	Gpcpd1	glycerophosphocholine phosphodiesterase 1, transcr	NA	0.22869	6.24515	1.17177	0.00457071	0.117681
50932	Mink1	misshapen-like kinase 1 (zebrafish), transcript varian	NA	-0.17881	6.377474	-1.13195	0.00458679	0.11788
77018	Col25a1	collagen, type XXV, alpha 1, transcript variant 3	NA	0.249268	4.663096	1.188604	0.0045929	0.11788
14167	Fgf12	fibroblast growth factor 12, transcript variant 2	NA	0.197602	5.479596	1.146791	0.00461341	0.118066
83965	Enpp5	ectonucleotide pyrophosphatase/phosphodiesterase	NA	0.159704	6.974415	1.117058	0.00461467	0.118066
72333	Palld	palladin, cytoskeletal associated protein, transcript va	NA	-0.2129	5.09931	-1.15901	0.00464103	0.118554
19365	Rad52	RAD52 homolog, DNA repair protein, transcript varia	NA	-0.29455	3.962588	-1.2265	0.00466095	0.118834
378431	Txlnb	taxilin beta	NA	-0.73797	1.08273	-1.66783	0.00466659	0.118834
223601	Cyrib	CYFIP related Rac1 interactor B, transcript variant 5	NA	0.171283	7.436209	1.12606	0.00467895	0.118962
102632340	Gm30439	predicted gene, 30439, transcript variant X4	NA	0.458215	2.553211	1.373841	0.00469086	0.119031
52563	Cdc23	CDC23 cell division cycle 23	NA	-0.15705	6.850544	-1.11501	0.00470686	0.119031
402767	A830052D11Rik	RIKEN cDNA A830052D11 gene	NA	0.975503	-0.0127	1.966327	0.00471305	0.119031
68552	Smim14	small integral membrane protein 14, transcript varian	NA	0.163001	6.635838	1.119614	0.00471597	0.119031
67549	Gpr89	G protein-coupled receptor 89, transcript variant X2	NA	-0.33571	4.375639	-1.262	0.00472091	0.119031
225020	Fez2	fasciculation and elongation protein zeta 2 (zygin II),	NA	0.214187	5.03053	1.16005	0.00472555	0.119031
12833	Col6a1	collagen, type VI, alpha 1	NA	-0.20259	5.842001	-1.15076	0.00473407	0.119061
268903	Nrip1	nuclear receptor interacting protein 1, transcript varia	NA	0.223009	5.471333	1.167166	0.00478753	0.119867
232286	Tmf1	TATA element modulatory factor 1	NA	0.217833	5.248431	1.162985	0.00479328	0.119867
208084	Pif1	PIF1 5'-to-3' DNA helicase, transcript variant 4	NA	-0.38708	3.153685	-1.30774	0.00479505	0.119867
67150	Rnf141	ring finger protein 141, transcript variant X2	NA	0.244492	5.721511	1.184676	0.00480726	0.119867
54403	Slc4a4	solute carrier family 4 (anion exchanger), member 4,	NA	0.309002	5.114371	1.238851	0.0048073	0.119867
52377	Rcn3	reticulocalbin 3, EF-hand calcium binding domain, tra	NA	-0.23756	4.666032	-1.179	0.00481214	0.119867
76952	Nt5c2	5'-nucleotidase, cytosolic II, transcript variant 4	NA	0.201967	6.322832	1.150266	0.00481767	0.119867
17172	Ascl1	achaete-scute family bHLH transcription factor 1	NA	-0.2849	5.228302	-1.21833	0.00483151	0.120028
16667	Krt17	keratin 17	NA	-1.11872	0.09132	-2.17154	0.00484076	0.120074
22750	Zfp9	zinc finger protein 9	NA	0.22899	5.247373	1.172014	0.00485796	0.120318
55948	Sfn	stratifin	NA	-0.75739	2.174722	-1.69044	0.00488683	0.120849
71206	Katnal2	katanin p60 subunit A-like 2, transcript variant 1	NA	-0.53601	2.60905	-1.44996	0.0049202	0.121205
381405	Zfp663	zinc finger protein 663, transcript variant 3	NA	-0.35621	3.622303	-1.28006	0.00492308	0.121205
73072	Prr36	proline rich 36, transcript variant X8	NA	-0.15809	7.396875	-1.11581	0.00492559	0.121205
74178	Stk40	serine/threonine kinase 40, transcript variant 2	NA	-0.23802	5.551023	-1.17937	0.00493102	0.121205
16438	Itpr1	inositol 1,4,5-trisphosphate receptor 1, transcript varia	NA	0.202759	5.123781	1.150898	0.00496255	0.121796
225642	Grp	gastrin releasing peptide	NA	0.648527	1.473381	1.567567	0.00498316	0.122047
12801	Cnr1	cannabinoid receptor 1 (brain), transcript variant X8	NA	0.175529	7.582851	1.129379	0.00499267	0.122047
78506	Micu3	mitochondrial calcium uptake family, member 3	NA	0.211018	5.425988	1.157504	0.00499527	0.122047
13605	Ect2	ect2 oncogene, transcript variant 2	NA	-0.26622	4.411575	-1.20265	0.00501385	0.122317
77605	H2az2	H2A.Z histone variant 2, transcript variant 1	NA	-0.23066	6.04253	-1.17337	0.0050328	0.122359
271278	BC024139	cDNA sequence BC024139	NA	-0.31653	3.805259	-1.24533	0.00504239	0.122359
109263	Rlf	rearranged L-myc fusion sequence, transcript variant	NA	0.221621	6.07616	1.166043	0.00504808	0.122359
71667	Tmem248	transmembrane protein 248, transcript variant X1	NA	0.177939	5.589886	1.131267	0.00505669	0.122359
233103	Garre1	granule associated Rac and RHOG effector 1, transc	NA	0.17736	5.77655	1.130813	0.00505828	0.122359
72852	Mblac2	metallo-beta-lactamase domain containing 2	NA	0.207835	5.331739	1.154954	0.00507223	0.122359
74105	Gga2	golgi associated, gamma adaptin ear containing, ARF	NA	-0.19321	6.220731	-1.1433	0.00507343	0.122359
66972	Slc25a23	solute carrier family 25 (mitochondrial carrier; phosph	NA	-0.1654	7.722171	-1.12148	0.00507573	0.122359
20269	Scn3a	sodium channel, voltage-gated, type III, alpha, transc	NA	0.235776	7.133571	1.17754	0.00508465	0.122393
22138	Ttn	titin, transcript variant X11	NA	-0.69089	2.937046	-1.61428	0.00509969	0.122526
12527	Cd9	CD9 antigen	NA	0.198621	5.239889	1.147601	0.0051054	0.122526

546143	Ccpg1os	cell cycle progression 1, opposite strand	NA	-0.48225	2.591324	-1.39692	0.00512196	0.122526
110789	Adgrv1	adhesion G protein-coupled receptor V1, transcript va	NA	-0.26725	4.305761	-1.20351	0.00512316	0.122526
223527	Eny2	ENY2 transcription and export complex 2 subunit, tra	NA	0.17004	6.73444	1.12509	0.00512786	0.122526
12614	Celsr1	cadherin, EGF LAG seven-pass G-type receptor 1, tr	NA	-0.25583	5.718221	-1.19402	0.00513912	0.122616
12915	Atf6b	activating transcription factor 6 beta	NA	-0.19092	6.241684	-1.1415	0.00515209	0.122734
58243	Nap1l5	nucleosome assembly protein 1-like 5	NA	0.260465	7.104615	1.197864	0.00517043	0.122734
17216	Mcm2	minichromosome maintenance complex component 2	NA	-0.24113	5.346449	-1.18192	0.00517202	0.122734
68943	Pink1	PTEN induced putative kinase 1	NA	0.179574	6.733314	1.132549	0.00517427	0.122734
26413	Mapk1	mitogen-activated protein kinase 1, transcript variant	NA	0.147879	8.035812	1.10794	0.00518384	0.122782
18795	Plcb1	phospholipase C, beta 1, transcript variant 1	NA	0.226677	6.056133	1.170136	0.0052186	0.123219
14632	Gli1	GLI-Kruppel family member GLI1	NA	-0.35422	3.232725	-1.27829	0.00521937	0.123219
68567	Cgref1	cell growth regulator with EF hand domain 1, transcri	NA	0.287389	4.45449	1.220429	0.00522674	0.123219
67771	Arpc5	actin related protein 2/3 complex, subunit 5	NA	0.159578	7.699932	1.11696	0.00523455	0.123219
13527	Dtna	dystrobrevin alpha, transcript variant X36	NA	0.220861	5.69236	1.165429	0.00524015	0.123219
56753	Tacstd2	tumor-associated calcium signal transducer 2	NA	-1.03761	0.595522	-2.05282	0.00526518	0.123557
20440	St6gal1	beta galactoside alpha 2,6 sialyltransferase 1, transc	NA	-0.27051	5.999503	-1.20623	0.0052797	0.123557
102637366	Gm10037	predicted gene 10037, transcript variant X1	NA	-0.6236	1.816461	-1.54072	0.00528899	0.123557
68977	Haghl	hydroxyacylglutathione hydrolase-like, transcript varia	NA	0.195317	5.845277	1.144976	0.00529783	0.123557
140721	Caskin2	CASK-interacting protein 2, transcript variant X1	NA	-0.25441	4.899441	-1.19285	0.00531117	0.123557
65973	Asph	aspartate-beta-hydroxylase, transcript variant 10	NA	0.240184	5.759188	1.181144	0.00532654	0.123557
140570	Plxnb2	plexin B2, transcript variant X7	NA	-0.15628	7.696905	-1.11441	0.00533307	0.123557
382867	Zfp488	zinc finger protein 488	NA	0.765317	0.807758	1.699743	0.00533796	0.123557
20399	Sh2b1	SH2B adaptor protein 1, transcript variant 5	NA	-0.16637	6.583475	-1.12223	0.00534197	0.123557
100503393	Gm14827	predicted gene 14827	NA	-0.39927	3.428121	-1.31884	0.00534216	0.123557
230249	Ecpas	Ecm29 proteasome adaptor and scaffold, transcript v	NA	0.197091	6.771423	1.146384	0.00534409	0.123557
15931	Ids	iduronate 2-sulfatase, transcript variant X6	NA	0.200818	8.122769	1.14935	0.00534564	0.123557
23879	Fxr2	fragile X mental retardation, autosomal homolog 2	NA	-0.16303	7.017118	-1.11964	0.00536046	0.1236
240119	St6gal2	beta galactoside alpha 2,6 sialyltransferase 2	NA	0.250661	6.375238	1.189752	0.00536267	0.1236
21453	Tcof1	treacle ribosome biogenesis factor 1, transcript variar	NA	-0.2066	5.634185	-1.15397	0.00537096	0.123616
17425	Foxk1	forkhead box K1, transcript variant X1	NA	-0.28353	6.241134	-1.21717	0.00541063	0.124353
229512	Smg5	Smg-5 homolog, nonsense mediated mRNA decay fa	NA	-0.19061	6.380055	-1.14124	0.00542608	0.124458
435366	Platr25	pluripotency associated transcript 25, transcript varia	NA	-0.22669	4.721209	-1.17015	0.00543051	0.124458
100503041	Pdzd7	PDZ domain containing 7, transcript variant X2	NA	-0.42328	2.940904	-1.34097	0.00545736	0.124898
77938	Fam53b	family with sequence similarity 53, member B, transc	NA	-0.28005	4.605556	-1.21424	0.00546958	0.125002
13386	Dlk1	delta like non-canonical Notch ligand 1, transcript var	NA	-0.19467	6.34288	-1.14446	0.00548939	0.125084
229709	Ahcyl1	S-adenosylhomocysteine hydrolase-like 1, transcript	NA	0.184857	7.71611	1.136704	0.00549288	0.125084
195727	Nhs	NHS actin remodeling regulator, transcript variant X1	NA	0.270067	4.503674	1.205864	0.0055168	0.125084
22668	Sf1	splicing factor 1, transcript variant X11	NA	-0.15087	7.978612	-1.11024	0.00551683	0.125084
74764	Klc4	kinesin light chain 4, transcript variant 2	NA	-0.21704	4.958879	-1.16235	0.00551817	0.125084
213550	Dis3l	DIS3 like exosome 3'-5' exoribonuclease, transcript v	NA	-0.21366	5.106587	-1.15963	0.0055193	0.125084
19348	Kif20a	kinesin family member 20A, transcript variant 3	NA	-0.31214	4.59443	-1.24155	0.00553482	0.125255
76884	Cyfip2	cytoplasmic FMR1 interacting protein 2, transcript var	NA	0.149366	7.993731	1.109082	0.00554765	0.125255
75273	Pelp1	proline, glutamic acid and leucine rich protein 1	NA	-0.18356	5.699281	-1.13568	0.00554994	0.125255
14682	Gnaq	guanine nucleotide binding protein, alpha q polypepti	NA	0.159863	8.659457	1.117181	0.00558371	0.125759
18675	Phex	phosphate regulating endopeptidase homolog, X-link	NA	0.873165	0.786968	1.831676	0.00558775	0.125759
71059	Hexim2	hexamethylene bis-acetamide inducible 2, transcript	NA	0.301293	3.963557	1.232249	0.00559998	0.12586
26554	Cul3	cullin 3, transcript variant X2	NA	0.186015	7.598217	1.137617	0.00561143	0.125927
17534	Mrc2	mannose receptor, C type 2	NA	-0.26009	4.58725	-1.19755	0.00561841	0.125927
16897	Llgl1	LLGL1 scribble cell polarity complex component, tran	NA	-0.16745	6.492451	-1.12307	0.00564011	0.126103
279653	Pcdh19	protocadherin 19, transcript variant X3	NA	0.218964	6.826368	1.163897	0.00564177	0.126103
20538	Slc6a2	solute carrier family 6 (neurotransmitter transporter, r	NA	0.520514	2.287757	1.434466	0.00566629	0.126131
225164	Mib1	mindbomb E3 ubiquitin protein ligase 1, transcript var	NA	0.233301	6.870978	1.175521	0.00566895	0.126131
212168	Zswim4	zinc finger SWIM-type containing 4	NA	-0.18222	6.031186	-1.13463	0.00567552	0.126131
105244946	Gm40468	predicted gene, 40468	NA	-0.52916	2.01937	-1.44309	0.00568667	0.126131
245555	Nexmif	neurite extension and migration factor, transcript vari	NA	0.199887	6.434448	1.148608	0.00568899	0.126131
231803	Mepce	methylphosphate capping enzyme	NA	-0.18265	5.65021	-1.13496	0.0056963	0.126131
26934	Racgap1	Rac GTPase-activating protein 1, transcript variant 2	NA	-0.20762	6.21779	-1.15478	0.00569729	0.126131
240641	Kif20b	kinesin family member 20B, transcript variant X4	NA	-0.40376	3.336636	-1.32295	0.00570864	0.12619
68842	Tulp4	tubby like protein 4, transcript variant X15	NA	0.140973	8.316226	1.102649	0.00571544	0.12619
11883	Arsa	arylsulfatase A	NA	-0.25513	4.710045	-1.19344	0.00572557	0.126242
52187	Rragd	Ras-related GTP binding D, transcript variant 1	NA	0.195162	6.063385	1.144853	0.00575507	0.126477
319405	D430036J16Rik	RIKEN cDNA D430036J16 gene, transcript variant 3	NA	0.714266	1.519121	1.640648	0.00575909	0.126477
18786	Plaa	phospholipase A2, activating protein	NA	0.217626	6.548531	1.162818	0.00575953	0.126477
75734	Mff	mitochondrial fission factor, transcript variant 8	NA	0.159974	7.432474	1.117267	0.00576835	0.126499
216011	Lrrc20	leucine rich repeat containing 20, transcript variant X	NA	-0.23974	5.171343	-1.18078	0.00580721	0.12718
74868	Tmem65	transmembrane protein 65, transcript variant X2	NA	0.161164	6.889762	1.118189	0.00583892	0.127448
14677	Gnai1	guanine nucleotide binding protein (G protein), alpha	NA	0.171319	6.678199	1.126087	0.005841	0.127448
20867	Stip1	stress-induced phosphoprotein 1	NA	-0.18042	7.50869	-1.13321	0.00584294	0.127448

17436	Me1	malic enzyme 1, NADP(+)-dependent, cytosolic, trans	NA	0.229352	5.820219	1.172308	0.00586698	0.127801
18191	Nrxn3	neurexin III, transcript variant 3	NA	0.167166	7.145169	1.12285	0.00589033	0.128138
225280	Ino80c	INO80 complex subunit C	NA	0.24156	4.869806	1.18227	0.00591606	0.128145
24100	Tpra1	transmembrane protein, adipocyte asscociated 1	NA	-0.25033	4.284147	-1.18948	0.00591951	0.128145
245595	Zfp711	zinc finger protein 711, transcript variant X4	NA	0.187893	5.658328	1.139099	0.00592	0.128145
170942	Erdr1	erythroid differentiation regulator 1, transcript variant	NA	-3.03976	1.169675	-8.22356	0.00592213	0.128145
108071	Grm5	glutamate receptor, metabotropic 5, transcript variant	NA	0.21387	6.366276	1.159795	0.00594306	0.128361
320845	A230056P14Rik	RIKEN cDNA A230056P14 gene	NA	-0.58124	1.876039	-1.49614	0.00595471	0.128361
19418	Rasgrf2	RAS protein-specific guanine nucleotide-releasing fac	NA	0.281836	4.377236	1.215741	0.0059558	0.128361
93840	Vangl2	VANGL planar cell polarity 2, transcript variant X6	NA	-0.14843	7.218659	-1.10837	0.00596501	0.12839
71722	Cic	capicua transcriptional repressor, transcript variant X	NA	-0.16192	7.868678	-1.11877	0.00599207	0.128802
16319	Incenp	inner centromere protein, transcript variant 2	NA	-0.23948	5.356937	-1.18057	0.00601737	0.12913
67849	Cdca5	cell division cycle associated 5	NA	-0.42368	2.881722	-1.34134	0.00602897	0.12913
67109	Zfp787	zinc finger protein 787, transcript variant 2	NA	0.297914	4.099157	1.229366	0.00603128	0.12913
627626	Ptchd4	patched domain containing 4, transcript variant X6	NA	0.282541	3.866475	1.216335	0.00603967	0.12913
232947	Ppp1r37	protein phosphatase 1, regulatory subunit 37	NA	-0.17109	6.643224	-1.12591	0.00605575	0.12913
209737	Kif15	kinesin family member 15	NA	-0.28495	3.987687	-1.21837	0.00605799	0.12913
70426	Tekt5	tektin 5	NA	0.463931	2.21758	1.379295	0.00606882	0.12913
71927	Itfg1	integrin alpha FG-GAP repeat containing 1	NA	0.155623	7.624889	1.113902	0.00607083	0.12913
226830	Smyd2	SET and MYND domain containing 2	NA	0.178549	5.432077	1.131745	0.00609297	0.129161
50793	Orc3	origin recognition complex, subunit 3, transcript variai	NA	0.197185	5.832529	1.146459	0.00609938	0.129161
238205	Lrfn5	leucine rich repeat and fibronectin type III domain cor	NA	0.180853	6.220258	1.133554	0.00610868	0.129161
66141	Ifitm3	interferon induced transmembrane protein 3	NA	0.421921	2.606095	1.33971	0.00611692	0.129161
19302	Pex2	peroxisomal biogenesis factor 2, transcript variant 9	NA	0.20005	5.211641	1.148738	0.00611695	0.129161
320007	Sidt1	SID1 transmembrane family, member 1, transcript va	NA	0.299196	3.771797	1.230458	0.00613013	0.129161
12564	Cdh8	cadherin 8, transcript variant 2	NA	0.184947	6.467297	1.136775	0.00613281	0.129161
78586	Srbd1	S1 RNA binding domain 1	NA	-0.2379	4.932901	-1.17928	0.00615057	0.129161
26754	Cops5	COP9 signalosome subunit 5, transcript variant 1	NA	0.168124	6.68246	1.123597	0.00616142	0.129161
231279	Guf1	GUF1 homolog, GTPase, transcript variant X1	NA	0.20222	5.921513	1.150467	0.00616867	0.129161
252875	Mios	meiosis regulator for oocyte development	NA	0.274995	5.206383	1.20999	0.00617331	0.129161
235461	Mindy2	MINDY lysine 48 deubiquitinase 2, transcript variant '	NA	0.213228	6.004861	1.159279	0.00617458	0.129161
18640	Pfkfb2	6-phosphofructo-2-kinase/fructose-2,6-biphosphatase	NA	0.245929	5.627227	1.185856	0.00619239	0.129161
54673	Sh3glb1	SH3-domain GRB2-like B1 (endophilin), transcript va	NA	0.180275	8.026804	1.1331	0.00619322	0.129161
16906	Lmnb1	lamin B1	NA	-0.18398	7.899634	-1.13601	0.00620322	0.129161
414093	A830082N09Rik	RIKEN cDNA A830082N09 gene	NA	0.2226	5.048821	1.166835	0.00620598	0.129161
108000	Cenpf	centromere protein F	NA	-0.2754	5.114331	-1.21033	0.00620721	0.129161
54561	Nap1l3	nucleosome assembly protein 1-like 3	NA	0.183488	5.563252	1.135626	0.0062362	0.129598
93700	Pcdhgb2	protocadherin gamma subfamily B, 2	NA	-0.33802	4.053752	-1.26402	0.00625633	0.129731
19263	Ptprb	protein tyrosine phosphatase, receptor type, B	NA	0.308543	4.091617	1.238456	0.00626726	0.129731
11517	Adcyap1r1	adenylate cyclase activating polypeptide 1 receptor 1	NA	0.169581	7.154495	1.124732	0.00626793	0.129731
666060	Frmpd1	FERM and PDZ domain containing 1, transcript varia	NA	0.528085	2.072418	1.442014	0.00627447	0.129731
15467	Eif2ak1	eukaryotic translation initiation factor 2 alpha kinase '	NA	-0.17553	5.999553	-1.12938	0.00628881	0.129862
66044	Dtd1	D-tyrosyl-tRNA deacylase 1	NA	0.209576	5.056016	1.156348	0.00632508	0.130244
70584	Pak4	p21 (RAC1) activated kinase 4	NA	-0.21953	4.597009	-1.16435	0.00633249	0.130244
14168	Fgf13	fibroblast growth factor 13, transcript variant 3	NA	0.172689	7.049965	1.127157	0.00633665	0.130244
103098	Slc6a15	solute carrier family 6 (neurotransmitter transporter),	NA	0.159186	6.523814	1.116657	0.00634081	0.130244
58799	Crbn	cereblon, transcript variant 2	NA	0.182473	5.811672	1.134827	0.00634731	0.130244
231003	Klhl17	kelch-like 17	NA	-0.21612	5.207062	-1.16161	0.00637668	0.130682
20408	Sh3gl3	SH3-domain GRB2-like 3, transcript variant X1	NA	0.194571	5.354721	1.144384	0.00638646	0.130718
14806	Grik2	glutamate receptor, ionotropic, kainate 2 (beta 2), trai	NA	0.186681	6.715782	1.138143	0.00641843	0.131054
224647	Ilrun	inflammation and lipid regulator with UBA-like and NE	NA	-0.19981	6.504677	-1.14855	0.0064234	0.131054
52837	Tmx4	thioredoxin-related transmembrane protein 4	NA	0.174272	7.627402	1.128395	0.00642705	0.131054
110611	Hdlbp	high density lipoprotein (HDL) binding protein, transcı	NA	-0.18074	7.509445	-1.13347	0.00643872	0.131128
74741	C2cd5	C2 calcium-dependent domain containing 5, transcrip	NA	-0.17314	6.495415	-1.12751	0.00646747	0.131446
108075	Ltbp4	latent transforming growth factor beta binding protein	NA	-0.22208	5.285354	-1.16642	0.00647053	0.131446
76371	2810408B13Rik	RIKEN cDNA 2810408B13 gene, transcript variant X'	NA	-0.40597	2.503191	-1.32498	0.00649405	0.13176
320873	Cdh10	cadherin 10, transcript variant 2	NA	0.202962	4.994392	1.151059	0.00653984	0.13241
100310872	Dynlt1a	dynein light chain Tctex-type 1A	NA	-0.21235	5.646253	-1.15858	0.00655887	0.13241
102632087	Gm13373	predicted gene 13373, transcript variant X7	NA	-1.2135	-0.63805	-2.31899	0.00655992	0.13241
106840	Unc119b	unc-119 lipid binding chaperone B	NA	-0.19025	5.540039	-1.14096	0.00657101	0.13241
319582	Trmt9b	tRNA methyltransferase 9B, transcript variant X1	NA	0.374194	2.920486	1.296115	0.0065713	0.13241
69657	2310047D07Rik	RIKEN cDNA 2310047D07 gene, transcript variant X:	NA	-1.03395	0.057798	-2.04762	0.00657983	0.13241
208898	Unc13c	unc-13 homolog C, transcript variant X3	NA	0.378643	3.701633	1.300118	0.00658306	0.13241
12606	Cebpa	CCAAT/enhancer binding protein (C/EBP), alpha, trai	NA	-0.65759	1.999014	-1.57744	0.0065996	0.132532
12877	Cpeb1	cytoplasmic polyadenylation element binding protein	NA	0.484617	2.42739	1.399215	0.00661227	0.132532
19143	St14	suppression of tumorigenicity 14 (colon carcinoma), t	NA	-0.74022	0.746669	-1.67043	0.00661384	0.132532
67956	Kmt5a	lysine methyltransferase 5A, transcript variant X3	NA	-0.24444	6.113303	-1.18463	0.00662371	0.132532
108067	Eif2b3	eukaryotic translation initiation factor 2B, subunit 3, tr	NA	0.27868	4.450131	1.213084	0.00662986	0.132532

22698	Zfp39	zinc finger protein 39	NA	0.239934	4.17056	1.180939	0.0066609	0.132865
210801	Unc5d	unc-5 netrin receptor D, transcript variant 4	NA	0.224263	5.986513	1.16818	0.00666284	0.132865
219135	Mtmr6	myotubularin related protein 6	NA	0.179815	6.367074	1.132738	0.00668386	0.132881
51944	Knstrn	kinetochore-localized astrin/SPAG5 binding	NA	-0.29248	4.599765	-1.22474	0.00670257	0.132881
54711	Plagl2	pleiomorphic adenoma gene-like 2	NA	-0.21631	4.86251	-1.16176	0.00670333	0.132881
72397	Rbm12b1	RNA binding motif protein 12 B1, transcript variant X5	NA	-0.27691	4.408172	-1.2116	0.0067096	0.132881
23849	Klf6	Kruppel-like factor 6	NA	0.174578	6.511433	1.128634	0.00671398	0.132881
24132	Zfp53	zinc finger protein 53	NA	0.307411	3.673862	1.237485	0.00671573	0.132881
24083	Natd1	N-acetyltransferase domain containing 1	NA	-0.2163	5.880565	-1.16175	0.00672247	0.132881
54644	Otud5	OTU domain containing 5, transcript variant 3	NA	-0.18009	6.832556	-1.13296	0.00673367	0.132881
56429	Dpt	dermatopontin	NA	-1.36156	0.381078	-2.56963	0.00673868	0.132881
13629	Eef2	eukaryotic translation elongation factor 2	NA	-0.16713	11.07737	-1.12282	0.00675898	0.132881
68087	Dcakd	dephospho-CoA kinase domain containing	NA	-0.1647	7.039641	-1.12094	0.00676242	0.132881
20284	Scrg1	scrapie responsive gene 1	NA	0.574227	1.69512	1.48888	0.00676358	0.132881
52679	E2f7	E2F transcription factor 7, transcript variant 1	NA	-0.35868	3.743288	-1.28225	0.00677354	0.132881
68770	Phtf2	putative homeodomain transcription factor 2, transcri	NA	0.209608	5.249554	1.156374	0.006783	0.132881
76373	Zfp773	zinc finger protein 773	NA	-0.28712	3.82266	-1.2202	0.00678612	0.132881
217463	Snx13	sorting nexin 13	NA	0.239191	5.34298	1.18033	0.0068022	0.133035
17883	Myh3	myosin, heavy polypeptide 3, skeletal muscle, embry	NA	-0.92476	3.39718	-1.89837	0.00684527	0.133233
107569	Nt5c3	5'-nucleotidase, cytosolic III, transcript variant 2	NA	0.165155	6.188605	1.121287	0.00685009	0.133233
73711	Mvb12a	multivesicular body subunit 12A	NA	0.291144	3.881444	1.22361	0.0068647	0.133233
241794	Kcng1	potassium voltage-gated channel, subfamily G, meml	NA	-0.28177	3.745176	-1.21569	0.006871	0.133233
360213	Trim46	tripartite motif-containing 46, transcript variant X1	NA	-0.14802	6.695589	-1.10805	0.00687728	0.133233
269878	Megf8	multiple EGF-like-domains 8	NA	-0.15054	7.001839	-1.10999	0.00688048	0.133233
12805	Cntn1	contactin 1, transcript variant 3	NA	0.234561	6.66868	1.176549	0.00688073	0.133233
102636905	Gm26908	predicted gene, 26908, transcript variant X9	NA	1.090804	-0.38534	2.129927	0.00688188	0.133233
268510	Mgat5b	mannoside acetylglucosaminyltransferase 5, isoenzy	NA	-0.1528	7.089017	-1.11172	0.00688602	0.133233
78339	Ttyh3	tweety family member 3, transcript variant 2	NA	-0.15747	8.985434	-1.11533	0.00690264	0.133252
50492	Thop1	thimet oligopeptidase 1	NA	-0.25083	5.21905	-1.1899	0.00691058	0.133252
18590	Pdgfa	platelet derived growth factor, alpha, transcript varian	NA	0.249395	4.68796	1.188709	0.00691154	0.133252
71678	Brox	BRO1 domain and CAAX motif containing, transcript	NA	0.141462	7.115782	1.103022	0.00695748	0.133765
57913	Pidd1	p53 induced death domain protein 1, transcript variar	NA	-0.3428	3.203153	-1.26822	0.00696034	0.133765
74025	Nphp3	nephronophthisis 3 (adolescent), transcript variant 1	NA	0.293974	3.588348	1.226013	0.00696284	0.133765
105980075	Gm20716	predicted gene 20716	NA	0.199268	7.282227	1.148116	0.00698537	0.133887
11966	Atp6v1b2	ATPase, H+ transporting, lysosomal V1 subunit B2	NA	0.145293	7.864492	1.105955	0.00699672	0.133887
497097	Xkr4	X-linked Kx blood group related 4, transcript variant X	NA	0.227761	6.356934	1.171016	0.00700084	0.133887
17428	Mnt	max binding protein	NA	-0.15838	6.225333	-1.11603	0.00700927	0.133887
68675	Fam172a	family with sequence similarity 172, member A, trans	NA	0.172069	5.931495	1.126673	0.00701031	0.133887
102182	Prmt9	protein arginine methyltransferase 9, transcript variar	NA	0.219134	4.672911	1.164035	0.00703998	0.134189
14793	Cdca3	cell division cycle associated 3	NA	-0.27649	4.722489	-1.21125	0.00704263	0.134189
16476	Jun	jun proto-oncogene	NA	-0.19665	5.690016	-1.14603	0.00706852	0.134457
11652	Akt2	thymoma viral proto-oncogene 2, transcript variant X5	NA	-0.2268	6.516317	-1.17024	0.00707322	0.134457
239410	A930017M01Rik	RIKEN cDNA A930017M01 gene	NA	0.421415	2.832411	1.33924	0.00712469	0.135278
71302	Arhgap26	Rho GTPase activating protein 26, transcript variant X	NA	0.217719	4.754507	1.162894	0.00715124	0.135623
12331	Cap1	CAP, adenylate cyclase-associated protein 1 (yeast),	NA	-0.15725	7.513644	-1.11516	0.00716918	0.135806
70747	Tspan2	tetraspanin 2, transcript variant 2	NA	0.33047	4.240553	1.257423	0.00718568	0.135909
12236	Bub1b	BUB1B, mitotic checkpoint serine/threonine kinase	NA	-0.33476	4.864711	-1.26117	0.00719801	0.135909
67298	Gprasp1	G protein-coupled receptor associated sorting protein	NA	0.1556	8.95677	1.113885	0.00719969	0.135909
18755	Prkch	protein kinase C, eta, transcript variant 1	NA	0.263216	3.915124	1.200151	0.00722195	0.135954
23789	Coro1b	coronin, actin binding protein 1B, transcript variant X	NA	-0.1695	6.143782	-1.12467	0.00722327	0.135954
105239	Rnf44	ring finger protein 44, transcript variant X19	NA	-0.16303	7.333869	-1.11963	0.00722714	0.135954
66084	Rmnd1	required for meiotic nuclear division 1 homolog, trans	NA	-0.26052	4.185764	-1.19791	0.00724797	0.136061
101358	Fbxl14	F-box and leucine-rich repeat protein 14	NA	-0.21318	5.640676	-1.15924	0.00725042	0.136061
21871	Atp6v0a2	ATPase, H+ transporting, lysosomal V0 subunit A2	NA	-0.17968	5.657355	-1.13263	0.0072579	0.136061
56386	B4galt6	UDP-Gal:betaGlcNAc beta 1,4-galactosyltransferase,	NA	0.194606	6.410419	1.144412	0.00727908	0.136074
70024	Mcm10	minichromosome maintenance 10 replication initiatior	NA	-0.43075	2.952644	-1.34794	0.00728288	0.136074
223723	Ttll12	tubulin tyrosine ligase-like family, member 12	NA	-0.1974	5.863057	-1.14663	0.00728966	0.136074
105245357	Gm40827	predicted gene, 40827	NA	-0.59102	1.446968	-1.50631	0.00729205	0.136074
18491	Pappa	pregnancy-associated plasma protein A	NA	0.492491	1.880102	1.406872	0.00731314	0.136273
11549	Adra1a	adrenergic receptor, alpha 1a, transcript variant X11	NA	0.383264	3.590841	1.304289	0.0073303	0.136273
11766	Ap1g2	adaptor protein complex AP-1, gamma 2 subunit, trar	NA	0.316622	3.370564	1.245411	0.00734185	0.136273
67398	Srpr	signal recognition particle receptor ('docking protein')	NA	-0.16664	5.771055	-1.12244	0.00735626	0.136273
11964	Atp6v1a	ATPase, H+ transporting, lysosomal V1 subunit A, tra	NA	0.165026	7.451366	1.121186	0.00735734	0.136273
69113	Alkbh3	alkB homolog 3, alpha-ketoglutarate-dependent dioxy	NA	0.329114	3.994385	1.256242	0.00735862	0.136273
56381	Spen	spen family transcription repressor, transcript variant	NA	-0.18734	6.91063	-1.13866	0.00736138	0.136273
93723	Pcdhga11	protocadherin gamma subfamily A, 11	NA	-0.29594	5.36392	-1.22768	0.00738986	0.136645
329152	Hecw2	HECT, C2 and WW domain containing E3 ubiquitin p	NA	0.261467	4.503443	1.198697	0.00740607	0.13679
22360	Nrsn1	neurensin 1	NA	0.198062	5.445463	1.147156	0.00741843	0.136835

80876	Ifitm2	interferon induced transmembrane protein 2	NA	0.24284	4.849384	1.18332	0.00743139	0.136835
66248	Alg5	asparagine-linked glycosylation 5 (dolichyl-phosphate	NA	0.251995	4.556327	1.190853	0.00743375	0.136835
233071	Arhgap33	Rho GTPase activating protein 33, transcript variant 3	NA	-0.1401	8.311835	-1.10198	0.00745073	0.136896
432486	Gnptab	N-acetylglucosamine-1-phosphate transferase, alpha	NA	0.244542	6.236028	1.184717	0.00746086	0.136896
17992	Ndufa4	Ndufa4, mitochondrial complex associated	NA	0.156898	6.91087	1.114887	0.00746233	0.136896
70750	Kdsr	3-ketodihydrosphingosine reductase, transcript variar	NA	0.240873	5.06439	1.181707	0.0074922	0.137157
233902	Fbxl19	F-box and leucine-rich repeat protein 19, transcript va	NA	-0.13881	7.72874	-1.10099	0.00749685	0.137157
64297	Gprc5b	G protein-coupled receptor, family C, group 5, memb	NA	-0.27042	5.008558	-1.20616	0.00750181	0.137157
20527	Slc2a3	solute carrier family 2 (facilitated glucose transporter)	NA	0.210557	5.891264	1.157135	0.00753049	0.137527
12444	Ccnd2	cyclin D2, transcript variant X5	NA	-0.24678	8.583349	-1.18656	0.00754685	0.137671
103836	Zfp692	zinc finger protein 692, transcript variant 2	NA	-0.23118	4.634836	-1.17379	0.00757462	0.137754
70233	Cd2bp2	CD2 cytoplasmic tail binding protein 2, transcript vari	NA	-0.17407	6.87018	-1.12824	0.00757638	0.137754
100126243	A030001D20Rik	RIKEN cDNA A030001D20 gene	NA	-0.55385	1.534964	-1.468	0.00757679	0.137754
231855	Ap5z1	adaptor-related protein complex 5, zeta 1 subunit, tra	NA	-0.32352	4.018033	-1.25138	0.0075983	0.137991
245578	Pcdh11x	protocadherin 11 X-linked, transcript variant 1	NA	0.251263	5.23732	1.190249	0.00761443	0.138005
72549	Reep4	receptor accessory protein 4, transcript variant X1	NA	-0.36041	2.958526	-1.28379	0.00761604	0.138005
20979	Syt1	synaptotagmin I, transcript variant X5	NA	0.146354	7.813622	1.106769	0.00762591	0.13803
18511	Pax9	paired box 9	NA	-1.35299	-0.92989	-2.55441	0.00764307	0.138088
27493	A230006K03Rik	RIKEN cDNA A230006K03 gene	NA	0.232044	4.928851	1.174498	0.00765168	0.138088
76282	Gpt	glutamic pyruvic transaminase, soluble	NA	-0.34791	3.007802	-1.27272	0.00766269	0.138088
230779	Serinc2	serine incorporator 2, transcript variant 1	NA	-0.22013	4.934328	-1.16484	0.00766306	0.138088
14395	Gabra2	gamma-aminobutyric acid (GABA) A receptor, subuni	NA	0.154018	6.57924	1.112664	0.00768137	0.138117
29819	Stau2	staufen double-stranded RNA binding protein 2, trans	NA	0.148825	7.15021	1.108666	0.00768605	0.138117
27078	B9d1	B9 protein domain 1, transcript variant 1	NA	-0.33192	3.252957	-1.25869	0.00769013	0.138117
58810	Akr1a1	aldo-keto reductase family 1, member A1 (aldehyde r	NA	0.136451	8.408817	1.099198	0.00774933	0.139027
211712	Pcdh9	protocadherin 9, transcript variant X2	NA	0.225728	7.361131	1.169368	0.00776711	0.139052
19387	Rangap1	RAN GTPase activating protein 1, transcript variant 2	NA	-0.17714	7.405119	-1.13064	0.00777531	0.139052
319991	Kif6	kinesin family member 6, transcript variant X1	NA	-0.61158	1.232852	-1.52794	0.00777981	0.139052
234736	Rfwd3	ring finger and WD repeat domain 3	NA	-0.20558	5.50454	-1.15315	0.00779078	0.139052
65113	Ndfip1	Nedd4 family interacting protein 1, transcript variant 2	NA	0.162367	8.535291	1.119122	0.00779347	0.139052
20520	Slc22a5	solute carrier family 22 (organic cation transporter), m	NA	0.249636	4.102563	1.188907	0.00782686	0.139419
654472	Gm12070	predicted gene 12070	NA	0.725166	0.881771	1.653091	0.00783887	0.139419
353187	Nr1d2	nuclear receptor subfamily 1, group D, member 2	NA	0.344568	4.072731	1.269771	0.00784772	0.139419
100039684	5031434O11Rik	RIKEN cDNA 5031434O11 gene, transcript variant 2	NA	0.600394	1.874422	1.51613	0.00785493	0.139419
72169	Trim29	tripartite motif-containing 29	NA	-1.55133	0.126058	-2.93087	0.00785691	0.139419
93960	Nkd1	naked cuticle 1, transcript variant 1	NA	-0.18214	5.491174	-1.13456	0.00787301	0.13951
68092	Ncbp2	nuclear cap binding protein subunit 2	NA	-0.17934	6.487179	-1.13236	0.00788316	0.13951
22658	Pcgf2	polycomb group ring finger 2, transcript variant 3	NA	0.145757	7.240258	1.106311	0.00788773	0.13951
23801	Aloxe3	arachidonate lipoxygenase 3	NA	0.531298	2.012716	1.445229	0.00790463	0.139517
270906	Prr11	proline rich 11	NA	-0.37129	4.121598	-1.29351	0.00790526	0.139517
72972	Ccser2	coiled-coil serine rich 2, transcript variant 2	NA	0.164966	6.725809	1.12114	0.00792481	0.13971
13206	Ddx4	DEAD box helicase 4, transcript variant 2	NA	-0.65212	0.915986	-1.57147	0.00793986	0.139824
105689	Mycbp2	MYC binding protein 2, E3 ubiquitin protein ligase, tra	NA	0.174203	8.122146	1.128341	0.00796252	0.140071
56194	Prpf40a	pre-mRNA processing factor 40A, transcript variant 5	NA	0.154165	6.828258	1.112778	0.00798033	0.140233
66272	Cox16	cytochrome c oxidase assembly protein 16, transcript	NA	-0.3092	4.086316	-1.23902	0.0080133	0.140661
210673	Prrt3	proline-rich transmembrane protein 3, transcript varia	NA	-0.43402	2.904771	-1.35099	0.00802323	0.140683
14228	Fkbp4	FK506 binding protein 4	NA	-0.1518	7.836813	-1.11096	0.00808059	0.141449
15893	Ica1	islet cell autoantigen 1, transcript variant X1	NA	0.191556	5.405473	1.141995	0.0080843	0.141449
227210	Ccnyl1	cyclin Y-like 1	NA	0.19321	5.278643	1.143305	0.00810991	0.141669
107934	Celsr3	cadherin, EGF LAG seven-pass G-type receptor 3, tr	NA	-0.14201	7.474914	-1.10344	0.0081143	0.141669
269629	Lhfpl3	lipoma HMGIC fusion partner-like 3, transcript variant	NA	0.283726	4.640922	1.217334	0.00813506	0.14188
70533	Btf3l4	basic transcription factor 3-like 4, transcript variant X	NA	0.159213	7.263552	1.116678	0.00816032	0.142168
233651	Dchs1	dachsous cadherin related 1	NA	-0.14701	7.463763	-1.10727	0.00818329	0.142276
269582	Clspn	claspin, transcript variant X6	NA	-0.26553	3.786683	-1.20208	0.008184	0.142276
57908	Zfp318	zinc finger protein 318, transcript variant X1	NA	0.221491	6.492578	1.165938	0.00821229	0.142615
329165	Abi2	abl-interactor 2, transcript variant 1	NA	0.171528	7.732323	1.126251	0.00822205	0.142632
102632094	Gm30259	predicted gene, 30259	NA	0.828819	1.305609	1.776231	0.00824586	0.142893
22067	Trpc5	transient receptor potential cation channel, subfamily	NA	0.302554	3.871387	1.233326	0.00829835	0.143232
18071	Nhlh1	nescient helix loop helix 1	NA	-0.38791	3.478466	-1.3085	0.00830027	0.143232
19205	Ptbp1	polypyrimidine tract binding protein 1, transcript varia	NA	-0.15614	7.009881	-1.1143	0.00830793	0.143232
74229	Paqr8	progestin and adipoQ receptor family member VIII, tra	NA	0.179443	5.894529	1.132446	0.00830848	0.143232
241112	Catip	ciliogenesis associated TTC17 interacting protein, tra	NA	0.764974	0.683779	1.69934	0.00831374	0.143232
56357	Ivd	isovaleryl coenzyme A dehydrogenase	NA	-0.18716	5.750505	-1.13852	0.00831824	0.143232
21770	Ppp2r5d	protein phosphatase 2, regulatory subunit B', delta, tr	NA	-0.14749	6.828411	-1.10764	0.0083682	0.14394
52696	Zwint	ZW10 interactor, transcript variant 2	NA	0.1285	8.61011	1.093156	0.00839632	0.144271
14020	Evi5	ecotropic viral integration site 5, transcript variant 1	NA	0.178611	5.953387	1.131794	0.00841319	0.144409
67216	Mboat2	membrane bound O-acyltransferase domain containi	NA	0.192556	6.270689	1.142786	0.00844138	0.144625
72108	Ddhd2	DDHD domain containing 2, transcript variant X12	NA	0.238674	5.533316	1.179907	0.0084436	0.144625

19272	Ptprk	protein tyrosine phosphatase, receptor type, K, transc	NA	0.182521	5.995815	1.134865	0.00846323	0.144675
56455	Dynll1	dynein light chain LC8-type 1	NA	-0.15557	7.722906	-1.11386	0.00847707	0.144675
76206	Gpr165	G protein-coupled receptor 165	NA	0.483543	1.936443	1.398173	0.00848004	0.144675
100316903	Gm20605	predicted gene 20605	NA	-0.29935	4.999908	-1.23059	0.00848205	0.144675
217944	Rapgef5	Rap guanine nucleotide exchange factor (GEF) 5, tra	NA	0.191237	6.386445	1.141743	0.00851877	0.144806
270066	Slc35e1	solute carrier family 35, member E1	NA	-0.20369	5.827159	-1.15164	0.00852388	0.144806
21420	Tfap2c	transcription factor AP-2, gamma, transcript variant 1	NA	-0.5068	3.67555	-1.4209	0.00853915	0.144806
14405	Gabrg1	gamma-aminobutyric acid (GABA) A receptor, subuni	NA	0.287501	4.304743	1.220524	0.00854525	0.144806
94214	Spock2	sparc/osteonectin, cwcv and kazal-like domains prote	NA	-0.18557	8.839478	-1.13727	0.00854941	0.144806
20667	Sox12	SRY (sex determining region Y)-box 12	NA	-0.15473	7.977869	-1.11321	0.00855702	0.144806
72795	Ttc19	tetratricopeptide repeat domain 19, transcript variant	NA	0.212769	5.143843	1.158911	0.00856125	0.144806
252972	Tpcn1	two pore channel 1, transcript variant X3	NA	-0.21591	5.567427	-1.16144	0.00856921	0.144806
171543	Bmf	BCL2 modifying factor, transcript variant 4	NA	-0.2731	4.030498	-1.2084	0.00857877	0.144806
67680	Sdhb	succinate dehydrogenase complex, subunit B, iron su	NA	0.160838	6.677783	1.117936	0.00858596	0.144806
228839	Tgif2	TGFB-induced factor homeobox 2, transcript variant)	NA	-0.22409	5.009947	-1.16804	0.0085932	0.144806
243385	Gprin3	GPRIN family member 3, transcript variant X1	NA	0.259199	4.565789	1.196814	0.00860859	0.144806
56332	Amotl2	angiomotin-like 2	NA	-0.15681	6.544769	-1.11482	0.00860974	0.144806
53883	Celsr2	cadherin, EGF LAG seven-pass G-type receptor 2, tra	NA	-0.13991	7.346149	-1.10184	0.00861457	0.144806
110959	Nudt19	nudix (nucleoside diphosphate linked moiety X)-type	NA	0.190318	5.287047	1.141015	0.00862933	0.144806
380921	Dgkh	diacylglycerol kinase, eta, transcript variant X10	NA	0.259595	5.126735	1.197142	0.00863214	0.144806
382018	Unc13a	unc-13 homolog A	NA	-0.16852	7.062156	-1.12391	0.00866205	0.14512
320772	Mdga2	MAM domain containing glycosylphosphatidylinositol	NA	0.239268	5.771029	1.180394	0.00867717	0.14512
268515	Bahcc1	BAH domain and coiled-coil containing 1	NA	-0.14892	6.605283	-1.10874	0.00867871	0.14512
433809	Rnf207	ring finger protein 207, transcript variant X6	NA	0.690559	1.11713	1.613909	0.00870354	0.14512
227632	Kcnt1	potassium channel, subfamily T, member 1, transcrip	NA	0.218155	5.288361	1.163245	0.00870914	0.14512
105244034	Gm33887	predicted gene, 33887	NA	0.246489	6.18561	1.186317	0.00871286	0.14512
12428	Ccna2	cyclin A2	NA	-0.24109	5.932218	-1.18189	0.00871324	0.14512
381157	Greb1l	growth regulation by estrogen in breast cancer-like	NA	0.357269	3.373883	1.280999	0.00872918	0.145236
75799	4930444P10Rik	RIKEN cDNA 4930444P10 gene, transcript variant X4	NA	-0.67065	0.858406	-1.59179	0.00873925	0.145255
271457	Rab5a	RAB5A, member RAS oncogene family	NA	0.168345	7.033961	1.123769	0.00877622	0.145721
66171	Pgls	6-phosphogluconolactonase, transcript variant 1	NA	0.211993	4.940847	1.158288	0.00880144	0.145899
75718	Vwa5b1	von Willebrand factor A domain containing 5B1, trans	NA	0.355737	3.142614	1.279639	0.00880649	0.145899
12308	Calb2	calbindin 2, transcript variant 1	NA	0.199779	5.723307	1.148522	0.00882017	0.145899
22141	Tub	tubby bipartite transcription factor	NA	0.139515	7.825794	1.101535	0.0088228	0.145899
17993	Ndufs4	NADH:ubiquinone oxidoreductase core subunit S4	NA	0.15436	6.096679	1.112928	0.0088349	0.145951
19704	Upf1	UPF1 regulator of nonsense transcripts homolog (yea	NA	-0.17549	6.461621	-1.12935	0.00887192	0.146275
11807	Apoa2	apolipoprotein A-II, transcript variant 4	NA	1.05141	-0.39659	2.072555	0.0088804	0.146275
99887	Tlcd4	TLC domain containing 4, transcript variant X6	NA	0.381006	3.373465	1.30225	0.00888533	0.146275
229445	Ctso	cathepsin O	NA	0.363115	3.019834	1.2862	0.00889051	0.146275
102637720	Gm16244	predicted gene 16244	NA	0.792427	0.676882	1.731985	0.00890991	0.146303
76843	Dtl	denticleless E3 ubiquitin protein ligase, transcript vari	NA	-0.26304	3.990272	-1.20001	0.00891281	0.146303
76936	Hnrnpm	heterogeneous nuclear ribonucleoprotein M, transcrip	NA	-0.14311	7.842298	-1.10428	0.00892567	0.146303
235106	Ntm	neurotrimin, transcript variant 7	NA	0.177464	7.52638	1.130894	0.00892818	0.146303
12821	Col17a1	collagen, type XVII, alpha 1, transcript variant 1	NA	-0.98589	0.744339	-1.98054	0.00893989	0.146348
12215	Bsg	basigin, transcript variant 2	NA	0.156075	8.33713	1.114251	0.00896141	0.146383
327766	Tmem26	transmembrane protein 26	NA	0.476657	2.7699	1.391516	0.00896803	0.146383
14811	Grin2a	glutamate receptor, ionotropic, NMDA2A (epsilon 1)	NA	0.421275	3.167299	1.33911	0.00896903	0.146383
20442	St3gal1	ST3 beta-galactoside alpha-2,3-sialyltransferase 1	NA	-0.23604	5.811702	-1.17775	0.00899032	0.146526
13178	Dck	deoxycytidine kinase	NA	-0.17068	5.987321	-1.12559	0.00899666	0.146526
16425	Itih2	inter-alpha trypsin inhibitor, heavy chain 2	NA	0.453884	2.473364	1.369723	0.00900478	0.146526
105785	Kdelr3	KDEL (Lys-Asp-Glu-Leu) endoplasmic reticulum prote	NA	0.458375	2.259005	1.373994	0.00903666	0.146898
319262	Fchsd1	FCH and double SH3 domains 1, transcript variant X1	NA	-0.21579	4.712963	-1.16134	0.00905086	0.146982
270096	Mon1b	MON1 homolog B, secretory traffciking associated, tr	NA	-0.20796	5.440691	-1.15505	0.00916478	0.148683
226977	Actr1b	ARP1 actin-related protein 1B, centractin beta, transc	NA	-0.14659	6.909346	-1.10695	0.00919025	0.148948
56878	Rbms1	RNA binding motif, single stranded interacting protein	NA	0.227798	5.969248	1.171047	0.00921651	0.14914
12317	Calr	calreticulin	NA	-0.14876	9.018568	-1.10862	0.00922707	0.14914
17907	Mylpf	myosin light chain, phosphorylatable, fast skeletal mu	NA	-1.01717	2.808293	-2.02395	0.00923819	0.14914
232811	Kmt5c	lysine methyltransferase 5C, transcript variant 3	NA	-0.23359	5.658866	-1.17575	0.00923877	0.14914
23797	Akt3	thymoma viral proto-oncogene 3	NA	0.124418	8.17152	1.090068	0.00929864	0.149815
118567509	LOC118567509	uncharacterized LOC118567509	NA	-0.81227	1.121419	-1.75598	0.00930181	0.149815
231863	Fbxl18	F-box and leucine-rich repeat protein 18, transcript va	NA	-0.21031	6.000327	-1.15694	0.0093261	0.149815
236794	Slc9a6	solute carrier family 9 (sodium/hydrogen exchanger),	NA	0.214729	6.997899	1.160486	0.0093313	0.149815
17105	Lyz2	lysozyme 2	NA	-0.50264	2.155746	-1.4168	0.00935367	0.149815
72713	Angptl1	angiopoietin-like 1	NA	-0.76132	0.444242	-1.69504	0.00935856	0.149815
58212	Srrm3	serine/arginine repetitive matrix 3, transcript variant X	NA	-0.16873	6.219999	-1.12407	0.00935984	0.149815
225872	Npas4	neuronal PAS domain protein 4, transcript variant X1	NA	-0.27796	4.027452	-1.21248	0.00935997	0.149815
15950	Ifi203	interferon activated gene 203, transcript variant 3	NA	0.533924	1.759617	1.447862	0.00936343	0.149815
64209	Herpud1	homocysteine-inducible, endoplasmic reticulum stres	NA	0.198013	4.967732	1.147117	0.00938278	0.149881

102638631	Gm35150	predicted gene, 35150, transcript variant X1	NA	0.386915	2.853447	1.307594	0.00938598	0.149881
14387	Gaa	glucosidase, alpha, acid, transcript variant 1	NA	-0.14798	6.451302	-1.10802	0.00941933	0.150233
56407	Trpc4ap	transient receptor potential cation channel, subfamily	NA	-0.13512	7.345813	-1.09819	0.0094265	0.150233
74343	Crtc2	CREB regulated transcription coactivator 2, transcript	NA	-0.19128	5.138181	-1.14178	0.00945217	0.150304
105943584	Gm45927	predicted gene, 45927, transcript variant 1	NA	-0.40056	3.634568	-1.32002	0.00945264	0.150304
236193	Zfp709	zinc finger protein 709	NA	0.308094	3.383008	1.238071	0.00945864	0.150304
73689	Bloc1s2	biogenesis of lysosomal organelles complex-1, subur	NA	0.197487	5.564681	1.146699	0.00947807	0.150466
20604	Sst	somatostatin	NA	0.202732	5.684906	1.150876	0.00950004	0.150562
20135	Rrm2	ribonucleotide reductase M2	NA	-0.21392	6.029001	-1.15983	0.00950266	0.150562
338351	Akap17b	A kinase (PRKA) anchor protein 17B, transcript varia	NA	0.26037	4.620887	1.197786	0.00955884	0.151305
235527	Plscr4	phospholipid scramblase 4	NA	0.608251	1.452675	1.52441	0.00956952	0.151327
208171	Tmprss7	transmembrane serine protease 7, transcript variant)	NA	-0.65536	0.995503	-1.57501	0.00957991	0.151344
78514	Arhgap10	Rho GTPase activating protein 10, transcript variant	NA	-0.40954	2.824922	-1.32827	0.00960402	0.151578
64652	Nisch	nischarin, transcript variant 1	NA	-0.12452	8.946203	-1.09015	0.00962441	0.151752
71962	Castor1	cytosolic arginine sensor for mTORC1 subunit 1	NA	0.666123	0.843889	1.586803	0.00965485	0.152085
380855	Rsl1	regulator of sex limited protein 1	NA	-0.49115	2.471539	-1.40556	0.00966744	0.152136
100226	Stx12	syntaxin 12	NA	0.136179	7.626518	1.098991	0.00974291	0.153031
17855	Mvk	mevalonate kinase, transcript variant X2	NA	0.205125	5.139803	1.152786	0.00974315	0.153031
74081	Cep350	centrosomal protein 350, transcript variant X9	NA	0.188069	5.780742	1.139238	0.00980349	0.153723
84035	Kremen1	kringle containing transmembrane protein 1	NA	-0.28769	4.118648	-1.22068	0.00980791	0.153723
228033	Atp5g3	ATP synthase, H+ transporting, mitochondrial F0 con	NA	0.139184	7.509053	1.101282	0.00981731	0.153723
57782	Rbak	RB-associated KRAB zinc finger, transcript variant 2	NA	0.233116	4.699483	1.175371	0.00982496	0.153723
59045	Stard3	START domain containing 3, transcript variant X3	NA	-0.21356	4.458147	-1.15955	0.00984196	0.153841
14712	Gnpat	glyceronephosphate O-acyltransferase	NA	-0.15809	6.24371	-1.11581	0.00985935	0.153892
100129	Gpr153	G protein-coupled receptor 153, transcript variant 1	NA	-0.17336	5.449804	-1.12768	0.00986965	0.153892
320472	Ppm1e	protein phosphatase 1E (PP2C domain containing), t	NA	0.16374	7.169656	1.120187	0.00987359	0.153892
100503583	Fsbp	fibrinogen silencer binding protein	NA	0.520898	2.600642	1.434848	0.00992494	0.15453
242687	Wasf2	WASP family, member 2, transcript variant X1	NA	-0.21736	5.316522	-1.16261	0.00994064	0.15453
404710	Iqgap3	IQ motif containing GTPase activating protein 3	NA	-0.36724	3.546954	-1.28988	0.00995998	0.15453
106877	Afap1l1	actin filament associated protein 1-like 1	NA	0.33373	4.18968	1.260267	0.00996178	0.15453
68235	Mturn	maturin, neural progenitor differentiation regulator ho	NA	0.129291	8.12129	1.093756	0.00996202	0.15453
20775	Sqle	squalene epoxidase	NA	0.130339	7.529035	1.094551	0.00998486	0.154737
66011	Ranbp17	RAN binding protein 17, transcript variant 1	NA	0.277334	3.702221	1.211954	0.01006541	0.155729
76132	Faxc	failed axon connections homolog, transcript variant X	NA	0.145964	7.665324	1.10647	0.01007656	0.155729
624866	Lekr1	leucine, glutamate and lysine rich 1, transcript varian	NA	-0.36107	2.703031	-1.28438	0.01007761	0.155729
319642	Rab9b	RAB9B, member RAS oncogene family	NA	0.19355	5.163936	1.143574	0.0100992	0.15581
65970	Lima1	LIM domain and actin binding 1, transcript variant X1	NA	-0.24512	5.54697	-1.18519	0.01010342	0.15581
13175	Dclk1	doublecortin-like kinase 1, transcript variant X3	NA	0.152169	9.151601	1.111239	0.01011158	0.15581
629595	Gm6988	predicted gene 6988	NA	0.179912	5.604744	1.132815	0.01014149	0.155973
19342	Rab4b	RAB4B, member RAS oncogene family	NA	0.208696	4.88242	1.155643	0.0101607	0.155973
78303	H2bu2	H2B.U histone 2	NA	-0.22942	5.045754	-1.17236	0.01016115	0.155973
66713	Actr2	ARP2 actin-related protein 2, transcript variant 2	NA	0.188421	8.11596	1.139516	0.01016371	0.155973
21677	Tead2	TEA domain family member 2, transcript variant 5	NA	-0.19968	6.002176	-1.14845	0.01017005	0.155973
11789	Apc	APC, WNT signaling pathway regulator, transcript va	NA	0.151632	8.63546	1.110825	0.01019449	0.156105
76866	Morn1	MORN repeat containing 1, transcript variant X39	NA	0.321378	3.451223	1.249523	0.01020269	0.156105
55979	Agpat1	1-acylglycerol-3-phosphate O-acyltransferase 1 (lyso	NA	-0.14031	6.988188	-1.10214	0.01020746	0.156105
654432	Gm7334	predicted gene 7334	NA	-1.91835	0.08346	-3.77992	0.01026068	0.156772
242425	Gabbr2	gamma-aminobutyric acid (GABA) B receptor, 2	NA	0.161716	6.286533	1.118617	0.0102853	0.15688
30941	Usp21	ubiquitin specific peptidase 21	NA	-0.20965	5.913606	-1.15641	0.01028708	0.15688
102636049	Gm33228	predicted gene, 33228, transcript variant X3	NA	0.961776	-0.13019	1.947706	0.01033085	0.157256
107971	Frs3	fibroblast growth factor receptor substrate 3	NA	0.234269	4.610974	1.17631	0.01033102	0.157256
12822	Col18a1	collagen, type XVIII, alpha 1, transcript variant 2	NA	-0.17367	5.761309	-1.12792	0.01036795	0.157474
68192	Leprotl1	leptin receptor overlapping transcript-like 1	NA	-0.14714	6.119974	-1.10737	0.01036963	0.157474
17933	Myt1l	myelin transcription factor 1-like, transcript variant 7	NA	0.126181	8.011842	1.091401	0.01037442	0.157474
114142	Foxp2	forkhead box P2, transcript variant X1	NA	0.163987	6.871015	1.120379	0.0103976	0.157679
56077	Dgke	diacylglycerol kinase, epsilon, transcript variant X1	NA	0.182541	5.781483	1.134881	0.01041494	0.157795
20620	Plk2	polo like kinase 2	NA	0.14285	7.205675	1.104084	0.01044599	0.158118
16834	Cog1	component of oligomeric golgi complex 1	NA	-0.18418	5.487633	-1.13617	0.01048259	0.158513
20562	Slit1	slit guidance ligand 1, transcript variant X4	NA	-0.14598	6.385988	-1.10648	0.0105146	0.158513
70300	Fuz	fuzzy planar cell polarity protein	NA	-0.34935	3.057989	-1.27398	0.01051597	0.158513
78246	Phf23	PHD finger protein 23, transcript variant 4	NA	-0.16792	6.04653	-1.12343	0.01052029	0.158513
26918	Ern2	endoplasmic reticulum (ER) to nucleus signalling 2, tr	NA	0.929366	-0.03099	1.904439	0.01052893	0.158513
74528	Mgme1	mitochondrial genome maintenance exonuclease 1, t	NA	-0.34463	3.126902	-1.26983	0.01054828	0.158513
19242	Ptn	pleiotrophin	NA	0.152745	9.138649	1.111683	0.01055173	0.158513
109113	Uhrf2	ubiquitin-like, containing PHD and RING finger domai	NA	-0.15933	6.21396	-1.11677	0.0105702	0.158513
26875	Pclo	piccolo (presynaptic cytomatrix protein), transcript va	NA	0.23384	6.929818	1.175961	0.01057615	0.158513
12611	Cebpg	CCAAT/enhancer binding protein (C/EBP), gamma	NA	-0.18974	5.888332	-1.14056	0.01058943	0.158513
18636	Cfp	complement factor properdin	NA	-0.35928	3.66144	-1.28279	0.01059934	0.158513

23989	Med24	mediator complex subunit 24, transcript variant 6	NA	-0.18047	6.470696	-1.13325	0.01060236	0.158513
319530	Zfp750	zinc finger protein 750	NA	-1.19963	-0.35343	-2.2968	0.01061099	0.158513
20472	Six2	sine oculis-related homeobox 2	NA	-0.58799	1.336892	-1.50315	0.01061657	0.158513
108083	Pip4k2b	phosphatidylinositol-5-phosphate 4-kinase, type II, be	NA	-0.15935	8.24002	-1.11679	0.0106255	0.158513
209225	Zfp710	zinc finger protein 710, transcript variant 1	NA	-0.21464	5.166833	-1.16041	0.01062791	0.158513
237362	Npffr1	neuropeptide FF receptor 1	NA	0.649892	1.213779	1.569051	0.01064384	0.158605
216848	Chd3	chromodomain helicase DNA binding protein 3	NA	-0.19125	9.617876	-1.14175	0.01065477	0.158623
235050	Zfp810	zinc finger protein 810	NA	0.223929	5.185486	1.16791	0.01067874	0.158727
22276	Uros	uroporphyrinogen III synthase, transcript variant 4	NA	-0.23237	4.680044	-1.17476	0.0106813	0.158727
225655	Prelid3a	PRELI domain containing 3A	NA	0.209915	5.015446	1.15662	0.01079682	0.160181
20807	Srf	serum response factor	NA	-0.20605	5.542579	-1.15352	0.0107988	0.160181
72003	Synpr	synaptoporin, transcript variant 2	NA	0.229052	4.804351	1.172064	0.01083301	0.16049
68126	Fahd2a	fumarylacetoacetate hydrolase domain containing 2A	NA	0.330419	3.32874	1.257378	0.01083939	0.16049
104111	Adcy3	adenylate cyclase 3, transcript variant 1	NA	-0.19635	4.742819	-1.1458	0.01085552	0.160496
72171	Shq1	SHQ1 homolog (S. cerevisiae), transcript variant X1	NA	-0.38468	2.495206	-1.30557	0.01086588	0.160496
77629	Sphkap	SPHK1 interactor, AKAP domain containing, transcrip	NA	0.16656	5.434685	1.122379	0.01087984	0.160496
68349	Ndufs3	NADH:ubiquinone oxidoreductase core subunit S3	NA	0.157882	6.060871	1.115648	0.01088337	0.160496
241694	Ralgapa2	Ral GTPase activating protein, alpha subunit 2 (catal	NA	0.171696	5.546833	1.126382	0.01088912	0.160496
12876	Cpe	carboxypeptidase E	NA	0.188668	9.195845	1.139711	0.0109154	0.160512
77733	Rnf170	ring finger protein 170, transcript variant 1	NA	0.229236	4.679135	1.172214	0.01092096	0.160512
54195	Gucy1b1	guanylate cyclase 1, soluble, beta 1, transcript varian	NA	0.155336	5.971989	1.113681	0.01093169	0.160512
22601	Yap1	yes-associated protein 1, transcript variant 2	NA	-0.16853	5.397723	-1.12391	0.01093474	0.160512
102638296	Gm17102	predicted gene 17102	NA	0.673549	0.592129	1.594992	0.01093954	0.160512
104940697	Dalir	DNMT1 associated long intergenic non-coding RNA	NA	0.784183	0.26982	1.722116	0.01097572	0.160775
13612	Edil3	EGF-like repeats and discoidin I-like domains 3, trans	NA	0.20412	6.217247	1.151983	0.0109772	0.160775
353310	Zfp703	zinc finger protein 703, transcript variant 1	NA	-0.1778	5.648045	-1.13116	0.0110266	0.161353
216835	Usp43	ubiquitin specific peptidase 43, transcript variant X2	NA	0.401428	2.807944	1.320814	0.01105553	0.161631
56795	Arl10	ADP-ribosylation factor-like 10, transcript variant 1	NA	-0.13966	6.933915	-1.10165	0.01106893	0.161682
21859	Timp3	tissue inhibitor of metalloproteinase 3	NA	0.219446	5.892785	1.164286	0.01108258	0.161736
105247002	Gm42187	predicted gene, 42187	NA	0.444968	2.407756	1.361284	0.01110924	0.161874
18670	Abcb4	ATP-binding cassette, sub-family B (MDR/TAP), men	NA	1.026616	-0.00585	2.03724	0.01111836	0.161874
11775	Ap3b2	adaptor-related protein complex 3, beta 2 subunit	NA	-0.14392	7.029339	-1.1049	0.01114341	0.161874
16579	Kifap3	kinesin-associated protein 3, transcript variant X1	NA	0.126891	8.129897	1.091938	0.0111462	0.161874
71648	Optn	optineurin, transcript variant X1	NA	0.278847	4.453912	1.213225	0.01117341	0.161874
338352	Nell1	NEL-like 1	NA	0.226224	4.538125	1.169769	0.01118503	0.161874
234542	Rtbdn	retbindin, transcript variant X1	NA	0.349835	3.092468	1.274415	0.01118988	0.161874
21894	Tln1	talin 1, transcript variant X5	NA	-0.19149	5.616936	-1.14195	0.0112205	0.161874
319415	Hs3st5	heparan sulfate (glucosamine) 3-O-sulfotransferase 5	NA	0.247295	3.950193	1.18698	0.01122557	0.161874
52838	Dnlz	DNL-type zinc finger, transcript variant 1	NA	-0.21654	4.922557	-1.16194	0.0112304	0.161874
60613	Kcnq4	potassium voltage-gated channel, subfamily Q, meml	NA	-0.2605	3.922156	-1.1979	0.01124046	0.161874
211986	Tmem18	transmembrane protein 18	NA	0.219748	4.949219	1.16453	0.01124694	0.161874
208777	Sned1	sushi, nidogen and EGF-like domains 1, transcript va	NA	0.184103	4.902836	1.13611	0.01126439	0.161874
16800	Arhgef2	rho/rac guanine nucleotide exchange factor (GEF) 2,	NA	-0.17955	7.288341	-1.13253	0.0112652	0.161874
69131	Cdk12	cyclin-dependent kinase 12, transcript variant 2	NA	-0.15768	5.985168	-1.11549	0.01129084	0.161874
380718	Mks1	MKS transition zone complex subunit 1	NA	-0.32516	3.455646	-1.25281	0.01129315	0.161874
52432	Ppp2r2d	protein phosphatase 2, regulatory subunit B, delta, tra	NA	0.153909	6.691433	1.11258	0.01129548	0.161874
111828493	Atrip-trex1	Atrip-Trex1 readthrough	NA	-0.264	4.421448	-1.2008	0.01131797	0.161874
68490	Zfp579	zinc finger protein 579, transcript variant X1	NA	-0.16376	6.057163	-1.1202	0.01132274	0.161874
260315	Nav3	neuron navigator 3, transcript variant X9	NA	0.14534	6.773356	1.105991	0.01132588	0.161874
208968	Zfp280c	zinc finger protein 280C, transcript variant 3	NA	0.20314	5.224815	1.151201	0.01133329	0.161874
14673	Gna12	guanine nucleotide binding protein, alpha 12	NA	-0.22664	5.50773	-1.17011	0.01133766	0.161874
116904	Alpk3	alpha-kinase 3, transcript variant X2	NA	-0.92679	0.143642	-1.90105	0.01134317	0.161874
13385	Dlg4	discs large MAGUK scaffold protein 4, transcript varia	NA	-0.13523	8.541668	-1.09827	0.01138738	0.161874
94217	Lrp1b	low density lipoprotein-related protein 1B, transcript v	NA	0.318231	3.581417	1.246801	0.01139259	0.161874
102800312	Lncppara	long noncoding RNA near Ppara	NA	0.601353	1.159696	1.517139	0.01139364	0.161874
67017	Fam210b	family with sequence similarity 210, member B	NA	0.203819	6.08291	1.151743	0.01139883	0.161874
224742	Abcf1	ATP-binding cassette, sub-family F (GCN20), membe	NA	-0.14821	6.827795	-1.10819	0.01140669	0.161874
100043040	1110002L01Rik	RIKEN cDNA 1110002L01 gene	NA	-0.27269	4.40964	-1.20806	0.01141408	0.161874
100044236	Copg2os2	coatomer protein complex, subunit gamma 2, opposit	NA	0.320906	5.544476	1.249114	0.01141526	0.161874
19141	Lgmn	legumain, transcript variant 1	NA	0.152406	5.988993	1.111421	0.01141799	0.161874
29870	Gtse1	G two S phase expressed protein 1, transcript variant	NA	-0.33023	3.28609	-1.25721	0.01141923	0.161874
18578	Pde4b	phosphodiesterase 4B, cAMP specific, transcript vari	NA	0.159533	5.863948	1.116926	0.01142032	0.161874
553095	Gm17750	predicted gene, 17750	NA	0.250282	4.404451	1.189439	0.01143359	0.161921
19879	Slc22a8	solute carrier family 22 (organic anion transporter), m	NA	0.276005	3.889304	1.210837	0.01146677	0.162171
56508	Rapgef4	Rap guanine nucleotide exchange factor (GEF) 4, tra	NA	0.240274	5.583299	1.181217	0.01147119	0.162171
14489	Mtpn	myotrophin	NA	0.12267	8.299814	1.088748	0.0115518	0.163078
105853	Mal2	mal, T cell differentiation protein 2	NA	0.243459	4.518416	1.183827	0.01155557	0.163078
29809	Rabgap1l	RAB GTPase activating protein 1-like, transcript varia	NA	0.137041	7.134222	1.099648	0.0115733	0.163078

76686	Clip3	CAP-GLY domain containing linker protein 3, transcri	NA	-0.13143	8.774805	-1.09538	0.01158324	0.163078
207921	Fam228b	family with sequence similarity 228, member B, transc	NA	0.393269	2.571856	1.313366	0.01158998	0.163078
22385	Baz1b	bromodomain adjacent to zinc finger domain, 1B	NA	-0.16994	7.194539	-1.12501	0.01159587	0.163078
223332	Ranbp3l	RAN binding protein 3-like	NA	0.387267	2.970528	1.307913	0.01160545	0.163078
232333	Slc6a1	solute carrier family 6 (neurotransmitter transporter, (	NA	0.151953	7.462309	1.111072	0.01164112	0.163438
27388	Ptdss2	phosphatidylserine synthase 2, transcript variant X4	NA	-0.20891	5.075235	-1.15581	0.01166115	0.163578
102638248	Gm34857	predicted gene, 34857, transcript variant X2	NA	0.983744	-0.24517	1.977591	0.01168527	0.16373
72026	Trmu	tRNA 5-methylaminomethyl-2-thiouridylate methyltrar	NA	0.216574	4.224006	1.161971	0.0116942	0.16373
68795	Ubr3	ubiquitin protein ligase E3 component n-recognin 3, t	NA	0.200434	6.878807	1.149044	0.01170222	0.16373
98732	Rab3gap2	RAB3 GTPase activating protein subunit 2	NA	0.164375	5.771354	1.120681	0.01171425	0.163758
234664	Nae1	NEDD8 activating enzyme E1 subunit 1	NA	0.158265	5.93277	1.115945	0.01177074	0.164223
102639918	Gm36117	predicted gene, 36117, transcript variant 2	NA	0.321894	4.117436	1.24997	0.01178758	0.164223
59021	Rab2a	RAB2A, member RAS oncogene family	NA	0.13529	8.465005	1.098313	0.01179574	0.164223
11350	Abl1	c-abl oncogene 1, non-receptor tyrosine kinase, trans	NA	-0.15223	6.611448	-1.11129	0.01179721	0.164223
53330	Vamp4	vesicle-associated membrane protein 4, transcript va	NA	0.183048	5.804114	1.13528	0.01179796	0.164223
26931	Ppp2r5c	protein phosphatase 2, regulatory subunit B', gamma	NA	0.1465	7.290998	1.106881	0.01189562	0.16527
72946	Lrrc47	leucine rich repeat containing 47	NA	-0.15816	6.171922	-1.11586	0.01190095	0.16527
99326	Garnl3	GTPase activating RANGAP domain-like 3, transcript	NA	0.157096	5.899736	1.115041	0.01190608	0.16527
18231	Nxph1	neurexophilin 1	NA	0.220103	5.670451	1.164817	0.01192296	0.16527
109979	Art3	ADP-ribosyltransferase 3, transcript variant 2	NA	0.631928	1.105691	1.549635	0.01192813	0.16527
140919	Slc17a6	solute carrier family 17 (sodium-dependent inorganic	NA	0.191013	6.892055	1.141565	0.01193413	0.16527
30957	Mapk8ip3	mitogen-activated protein kinase 8 interacting protein	NA	-0.1381	7.59702	-1.10046	0.01197873	0.165466
14109	Fau	Finkel-Biskis-Reilly murine sarcoma virus (FBR-MuSV	NA	0.141682	7.346641	1.10319	0.01198835	0.165466
59035	Carm1	coactivator-associated arginine methyltransferase 1,	NA	-0.149	7.011017	-1.1088	0.01199052	0.165466
75196	Ankrd7	ankyrin repeat domain 7, transcript variant X1	NA	-0.74445	0.956294	-1.67533	0.01199123	0.165466
14633	Gli2	GLI-Kruppel family member GLI2	NA	-0.27371	4.089965	-1.20891	0.01200168	0.165466
347740	Norad	non-coding RNA activated by DNA damage	NA	0.161909	9.316195	1.118766	0.01200928	0.165466
72745	Tmem161b	transmembrane protein 161B, transcript variant 3	NA	0.225642	4.909959	1.169298	0.01202224	0.16548
56632	Sphk2	sphingosine kinase 2, transcript variant X2	NA	-0.20725	4.73218	-1.15449	0.01203068	0.16548
246257	Ovca2	candidate tumor suppressor in ovarian cancer 2	NA	-0.2319	4.37884	-1.17438	0.01206162	0.16567
16348	Invs	inversin, transcript variant X9	NA	-0.24941	3.765275	-1.18872	0.01207513	0.16567
104015	Synj1	synaptojanin 1, transcript variant X47	NA	0.173792	6.826856	1.12802	0.01207582	0.16567
13002	Dnajc5	DnaJ heat shock protein family (Hsp40) member C5,	NA	0.136958	8.155348	1.099584	0.01208522	0.16567
270685	Mthfd1l	methylenetetrahydrofolate dehydrogenase (NADP+ d	NA	0.35943	3.422323	1.282919	0.01213182	0.166113
208884	Zdhhc9	zinc finger, DHHC domain containing 9	NA	-0.20854	6.479681	-1.15552	0.01213796	0.166113
20265	Scn1a	sodium channel, voltage-gated, type I, alpha, transcri	NA	0.283357	4.859729	1.217023	0.01216149	0.166296
66673	Sorcs3	sortilin-related VPS10 domain containing receptor 3	NA	0.246859	4.777891	1.186621	0.01217635	0.166359
237615	Ankrd52	ankyrin repeat domain 52	NA	-0.24159	6.384375	-1.1823	0.01221453	0.166694
192212	Prom2	prominin 2, transcript variant 1	NA	-1.14432	-0.68305	-2.21042	0.01222134	0.166694
327942	Pigl	phosphatidylinositol glycan anchor biosynthesis, clas	NA	0.265301	4.204628	1.201887	0.01223191	0.166698
381310	Stum	mechanosensory transduction mediator	NA	0.223667	5.449963	1.167698	0.0122603	0.166945
66645	Pspc1	paraspeckle protein 1, transcript variant 1	NA	0.139187	6.545641	1.101285	0.01228245	0.167107
12843	Col1a2	collagen, type I, alpha 2	NA	-0.23227	8.013065	-1.17468	0.01232094	0.167491
24045	Scamp3	secretory carrier membrane protein 3, transcript varia	NA	0.193416	5.174695	1.143468	0.01236714	0.167884
381598	2610005L07Rik	RIKEN cDNA 2610005L07 gene	NA	0.216928	5.299637	1.162256	0.01238159	0.167884
108797	Mex3b	mex3 RNA binding family member B	NA	-0.17232	7.067821	-1.12687	0.01239494	0.167884
103284	Zc3h10	zinc finger CCCH type containing 10	NA	-0.23638	4.314728	-1.17803	0.01239793	0.167884
66218	Ndufb9	NADH:ubiquinone oxidoreductase subunit B9, transc	NA	0.158551	6.120365	1.116166	0.01240146	0.167884
64011	Nrgn	neurogranin	NA	0.26273	3.888438	1.199747	0.01241273	0.167897
108829	Jmjd1c	jumonji domain containing 1C, transcript variant X16	NA	0.1694	6.539752	1.124591	0.01245238	0.168213
52028	Bbs1	Bardet-Biedl syndrome 1 (human)	NA	-0.19379	5.147691	-1.14377	0.01245675	0.168213
19058	Ppp3r1	protein phosphatase 3, regulatory subunit B, alpha is	NA	0.127926	7.936416	1.092722	0.01250028	0.168402
100504412	Kcnmb4os2	potassium large conductance calcium-activated chan	NA	0.623987	1.41471	1.541128	0.01250041	0.168402
22201	Uba1	ubiquitin-like modifier activating enzyme 1, transcript	NA	-0.14132	9.486141	-1.10291	0.01250179	0.168402
434280	Sox1ot	Sox1 overlapping transcript	NA	-0.19294	5.416695	-1.14309	0.01252807	0.168547
19739	Rgs9	regulator of G-protein signaling 9, transcript variant 2	NA	0.195114	5.451449	1.144815	0.01253329	0.168547
636808	Cntnap5a	contactin associated protein-like 5A, transcript varian	NA	0.220058	4.86507	1.16478	0.01255455	0.168693
102640268	Gm2956	predicted gene 2956	NA	1.74865	-0.83413	3.360439	0.01258492	0.168962
241568	Lrrc4c	leucine rich repeat containing 4C, transcript variant 3	NA	0.160944	6.094388	1.118019	0.01262214	0.169322
69981	Tmem30a	transmembrane protein 30A	NA	0.154049	7.232848	1.112688	0.01265959	0.169425
73826	Poldip3	polymerase (DNA-directed), delta interacting protein	NA	-0.12872	6.945475	-1.09332	0.01268514	0.169425
225288	Fhod3	formin homology 2 domain containing 3, transcript va	NA	0.183981	5.558472	1.136014	0.01269219	0.169425
93961	B3galt5	UDP-Gal:betaGlcNAc beta 1,3-galactosyltransferase,	NA	0.393044	2.904211	1.313161	0.01271224	0.169425
277396	Klhl23	kelch-like 23, transcript variant 3	NA	0.139162	7.204997	1.101265	0.01271705	0.169425
20469	Sipa1	signal-induced proliferation associated gene 1, transc	NA	-0.22093	4.422637	-1.16549	0.01271847	0.169425
15211	Hexa	hexosaminidase A	NA	-0.18852	5.125151	-1.1396	0.01272922	0.169425
21423	Tcf3	transcription factor 3, transcript variant 8	NA	-0.14465	6.695047	-1.10546	0.01273082	0.169425
12226	Btg1	BTG anti-proliferation factor 1	NA	0.135653	7.245468	1.09859	0.01273092	0.169425

239796	Mb21d2	Mab-21 domain containing 2	NA	0.152076	5.939366	1.111168	0.01273392	0.169425
16687	Krt6a	keratin 6A	NA	-1.17571	0.869543	-2.25904	0.0127659	0.169456
233208	Scaf1	SR-related CTD-associated factor 1, transcript varian	NA	-0.12807	7.651521	-1.09283	0.01278786	0.169456
14807	Grik3	glutamate receptor, ionotropic, kainate 3	NA	-0.21882	7.065619	-1.16378	0.01280289	0.169456
12653	Chgb	chromogranin B	NA	0.208981	6.657987	1.155871	0.01281465	0.169456
77116	Mtmr2	myotubularin related protein 2, transcript variant X10	NA	0.169169	6.061629	1.12441	0.01282696	0.169456
102632538	C230034O21Rik	RIKEN cDNA C230034O21 gene	NA	0.301234	3.475642	1.232198	0.01283674	0.169456
21343	Taf6	TATA-box binding protein associated factor 6, transc	NA	-0.18847	5.40864	-1.13955	0.01283889	0.169456
26404	Map3k12	mitogen-activated protein kinase kinase kinase 12, tr	NA	-0.17035	6.803453	-1.12533	0.01284516	0.169456
211651	Fancd2	Fanconi anemia, complementation group D2, transcri	NA	-0.35176	2.701269	-1.27612	0.01284701	0.169456
218977	Dlgap5	DLG associated protein 5	NA	-0.40736	3.416247	-1.32625	0.01284711	0.169456
27360	Add3	adducin 3 (gamma), transcript variant 2	NA	0.182338	5.64861	1.134722	0.0128877	0.169456
19055	Ppp3ca	protein phosphatase 3, catalytic subunit, alpha isofor	NA	0.138988	8.061898	1.101132	0.01290168	0.169456
60425	Doc2g	double C2, gamma	NA	0.300216	3.476114	1.231328	0.01290972	0.169456
12304	Pdia4	protein disulfide isomerase associated 4, transcript va	NA	-0.15901	6.776064	-1.11652	0.01291854	0.169456
67023	Use1	unconventional SNARE in the ER 1 homolog (S. cere	NA	0.185963	5.31543	1.137576	0.01293142	0.169456
22634	Plagl1	pleiomorphic adenoma gene-like 1, transcript variant	NA	-0.13325	7.127015	-1.09676	0.01293149	0.169456
14401	Gabrb2	gamma-aminobutyric acid (GABA) A receptor, subuni	NA	0.196583	6.594514	1.145981	0.01293573	0.169456
321000	Lrif1	ligand dependent nuclear receptor interacting factor 1	NA	0.213268	4.690681	1.159311	0.01295149	0.169456
22445	Xlr3a	X-linked lymphocyte-regulated 3A, transcript variant X	NA	-0.68185	0.93667	-1.6042	0.01295785	0.169456
77578	Bcl9	B cell CLL/lymphoma 9, transcript variant X2	NA	-0.16574	7.490795	-1.12174	0.01296056	0.169456
16490	Kcna2	potassium voltage-gated channel, shaker-related sub	NA	0.259027	4.676492	1.196671	0.01296354	0.169456
101476	Plekha1	pleckstrin homology domain containing, family A (pho	NA	0.131781	7.036088	1.095645	0.01296583	0.169456
233870	Tufm	Tu translation elongation factor, mitochondrial, transc	NA	-0.15858	6.238125	-1.11619	0.01297578	0.169456
14732	Gpam	glycerol-3-phosphate acyltransferase, mitochondrial,	NA	0.162341	5.811329	1.119102	0.01304759	0.169898
68144	5031426D15Rik	RIKEN cDNA 5031426D15 gene	NA	0.309374	3.511699	1.23917	0.0130495	0.169898
20503	Slc16a7	solute carrier family 16 (monocarboxylic acid transpo	NA	-0.26258	4.42763	-1.19963	0.01305214	0.169898
22779	Ikzf2	IKAROS family zinc finger 2	NA	-0.30719	3.22327	-1.2373	0.01306796	0.169898
110033	Kif22	kinesin family member 22	NA	-0.23489	4.536343	-1.17682	0.01309806	0.169898
68184	Denr	density-regulated protein, transcript variant X1	NA	0.161638	6.12515	1.118556	0.01309889	0.169898
15183	Hdac3	histone deacetylase 3	NA	0.136792	7.003853	1.099457	0.01310753	0.169898
23912	Rhof	ras homolog family member F (in filopodia), transcript	NA	-0.12443	7.351804	-1.09007	0.01310878	0.169898
22185	U2af2	U2 small nuclear ribonucleoprotein auxiliary factor (U	NA	-0.13789	8.190182	-1.1003	0.01311092	0.169898
23897	Hax1	HCLS1 associated X-1, transcript variant 1	NA	-0.19866	5.271256	-1.14763	0.01312858	0.169898
232878	Zscan22	zinc finger and SCAN domain containing 22, transcrip	NA	-0.30113	3.790412	-1.23211	0.01313113	0.169898
328424	Kcnrg	potassium channel regulator, transcript variant 1	NA	0.647795	0.909433	1.566771	0.01313602	0.169898
12834	Col6a2	collagen, type VI, alpha 2, transcript variant 1	NA	-0.19194	5.668517	-1.1423	0.01315544	0.169898
77862	Thyn1	thymocyte nuclear protein 1, transcript variant X1	NA	0.187224	5.37772	1.138571	0.01315579	0.169898
74004	Jakmip3	janus kinase and microtubule interacting protein 3	NA	0.301153	3.909465	1.232129	0.01317993	0.169996
108167638	Gm46102	predicted gene, 46102	NA	0.933438	-0.29885	1.909821	0.01318433	0.169996
68043	Eef1akmt1	EEF1A alpha lysine methyltransferase 1, transcript va	NA	-0.27173	3.874244	-1.20725	0.01320941	0.170185
434156	Eid2b	EP300 interacting inhibitor of differentiation 2B	NA	0.19528	5.254424	1.144946	0.01325154	0.170449
74200	Khdc4	KH domain containing 4, pre-mRNA splicing factor, tr	NA	0.140529	6.980739	1.102309	0.01325543	0.170449
20499	Slc12a7	solute carrier family 12, member 7, transcript variant	NA	-0.20752	4.546919	-1.1547	0.01326134	0.170449
228536	Bahd1	bromo adjacent homology domain containing 1, trans	NA	-0.16098	5.442472	-1.11805	0.01328328	0.170596
81896	Ift122	intraflagellar transport 122, transcript variant 2	NA	-0.20719	4.910509	-1.15444	0.0133044	0.170698
12293	Cacna2d1	calcium channel, voltage-dependent, alpha2/delta sul	NA	0.136729	7.21861	1.09941	0.0133122	0.170698
77569	Limch1	LIM and calponin homology domains 1, transcript var	NA	0.138499	6.601526	1.100759	0.01333016	0.170722
66695	Aspn	asporin, transcript variant 1	NA	-0.69825	2.194799	-1.62254	0.01334151	0.170722
64293	Stk32b	serine/threonine kinase 32B	NA	-0.18498	6.557696	-1.1368	0.01336464	0.170722
72656	Ints8	integrator complex subunit 8, transcript variant X2	NA	-0.16967	5.64452	-1.1248	0.01337514	0.170722
626596	Rgs22	regulator of G-protein signalling 22, transcript variant	NA	0.814423	0.344668	1.758594	0.0133857	0.170722
76917	Flywch2	FLYWCH family member 2, transcript variant 2	NA	0.374793	3.095624	1.296654	0.01338605	0.170722
14585	Gfra1	glial cell line derived neurotrophic factor family recept	NA	0.210453	5.506999	1.157051	0.01339428	0.170722
100503859	1110015O18Rik	RIKEN cDNA 1110015O18 gene	NA	0.34446	2.871025	1.269675	0.01340444	0.170722
23963	Tenm1	teneurin transmembrane protein 1, transcript variant X	NA	0.228721	5.642274	1.171795	0.0134142	0.170722
100233208	Gm10778	predicted gene 10778	NA	-0.57193	2.908763	-1.48651	0.01341895	0.170722
102640970	Gm36907	predicted gene, 36907, transcript variant X4	NA	0.349078	2.78253	1.273747	0.01343743	0.170823
20377	Sfrp1	secreted frizzled-related protein 1	NA	-0.15791	6.52817	-1.11567	0.01348878	0.171297
212483	Fam193b	family with sequence similarity 193, member B, trans	NA	-0.14463	6.689585	-1.10545	0.01350562	0.171297
399566	Btbd6	BTB (POZ) domain containing 6, transcript variant 2	NA	0.183813	5.041234	1.135882	0.01350625	0.171297
218333	Ice1	interactor of little elongation complex ELL subunit 1	NA	0.145821	7.129252	1.10636	0.01353951	0.171585
98660	Atp1a2	ATPase, Na+/K+ transporting, alpha 2 polypeptide	NA	0.137167	7.588703	1.099743	0.01355718	0.171675
105245369	Gm3625	predicted gene 3625	NA	0.859262	0.267359	1.81411	0.01358844	0.171817
14000	Drosha	drosha, ribonuclease type III, transcript variant 2	NA	-0.15902	7.454946	-1.11653	0.01359875	0.171817
111970	Dlx1as	distal-less homeobox 1, antisense	NA	-0.34671	5.874565	-1.27166	0.0136025	0.171817
114570	Crip3	cysteine-rich protein 3, transcript variant X10	NA	-0.45343	2.549168	-1.36929	0.01361066	0.171817
67125	Tspan31	tetraspanin 31	NA	-0.15089	6.033079	-1.11026	0.01362804	0.171845

210573	Tmem151b	transmembrane protein 151B	NA	-0.13339	6.806105	-1.09687	0.01364825	0.171845
80904	Dtx3	deltex 3, E3 ubiquitin ligase, transcript variant X13	NA	-0.14664	7.816821	-1.10699	0.01364975	0.171845
11717	Ampd3	adenosine monophosphate deaminase 3, transcript v	NA	0.24802	3.685685	1.187576	0.01365505	0.171845
12443	Ccnd1	cyclin D1, transcript variant 2	NA	-0.17783	5.968676	-1.13118	0.01368989	0.17215
66266	Eapp	E2F-associated phosphoprotein, transcript variant 1	NA	0.165281	5.840221	1.121384	0.01374513	0.172661
100303732	Zfp967	zinc finger protein 967, transcript variant 2	NA	1.987475	0.113894	3.965424	0.01376109	0.172661
13872	Ercc3	excision repair cross-complementing rodent repair de	NA	0.168558	5.08887	1.123934	0.01376239	0.172661
18472	Pafah1b1	platelet-activating factor acetylhydrolase, isoform 1b,	NA	0.153952	8.711575	1.112613	0.01378093	0.172761
67161	Sclt1	sodium channel and clathrin linker 1, transcript variar	NA	0.237538	3.978023	1.178979	0.01379739	0.172834
22260	Nr1h2	nuclear receptor subfamily 1, group H, member 2, tra	NA	-0.20816	5.03425	-1.15522	0.01383759	0.173111
75599	Pcdh1	protocadherin 1, transcript variant X3	NA	-0.15481	6.419735	-1.11328	0.01384528	0.173111
22321	Vars	valyl-tRNA synthetase, transcript variant X1	NA	-0.16046	6.447627	-1.11765	0.01386155	0.173111
94352	Loxl2	lysyl oxidase-like 2	NA	-0.24198	3.775541	-1.18261	0.01386832	0.173111
18676	Phf2	PHD finger protein 2	NA	-0.15143	6.952617	-1.11067	0.01389531	0.173111
100504191	Gm16576	predicted gene 16576	NA	-0.66129	1.109795	-1.58149	0.01389593	0.173111
76709	Arpc2	actin related protein 2/3 complex, subunit 2, transcrip	NA	0.1311	7.655339	1.095128	0.0139088	0.173111
18519	Kat2b	K(lysine) acetyltransferase 2B, transcript variant 2	NA	0.193139	4.785967	1.143248	0.01392196	0.173111
100678	Psph	phosphoserine phosphatase	NA	0.253451	3.775335	1.192055	0.01392934	0.173111
72514	Fgfbp3	fibroblast growth factor binding protein 3	NA	0.230614	5.573202	1.173334	0.01393026	0.173111
75965	Zdhhc20	zinc finger, DHHC domain containing 20, transcript va	NA	0.162758	6.052461	1.119425	0.0139485	0.173111
23964	Tenm2	teneurin transmembrane protein 2, transcript variant	NA	0.142706	7.670424	1.103974	0.01395979	0.173111
76872	Ccdc116	coiled-coil domain containing 116, transcript variant X	NA	0.362127	2.94394	1.28532	0.01396322	0.173111
71919	Rpap3	RNA polymerase II associated protein 3	NA	0.171441	5.263188	1.126183	0.01396855	0.173111
13829	Dmtn	dematin actin binding protein, transcript variant X14	NA	-0.15707	6.325415	-1.11502	0.01397909	0.173111
72925	Marchf1	membrane associated ring-CH-type finger 1, transcri	NA	0.231309	5.834713	1.1739	0.01399998	0.173177
102638715	Gm35209	predicted gene, 35209, transcript variant X1	NA	-1.36562	-0.56136	-2.57688	0.01400568	0.173177
100038514	Gm11837	predicted gene 11837, transcript variant 2	NA	0.384238	2.268602	1.30517	0.01405729	0.173527
16880	Lifr	LIF receptor alpha, transcript variant 1	NA	0.229566	5.02133	1.172482	0.01406066	0.173527
320256	Dlec1	deleted in lung and esophageal cancer 1, transcript v	NA	-0.55933	1.048265	-1.47358	0.01406602	0.173527
74513	Neto2	neuropilin (NRP) and tolloid (TLL)-like 2, transcript va	NA	0.166334	7.366757	1.122203	0.01408522	0.173629
11606	Agt	angiotensinogen (serpin peptidase inhibitor, clade A,	NA	0.82445	0.025861	1.770859	0.01409563	0.173629
12454	Ccnk	cyclin K	NA	-0.17019	6.109393	-1.12521	0.01415025	0.17417
110891	Slc8a2	solute carrier family 8 (sodium/calcium exchanger), m	NA	-0.16275	5.809056	-1.11942	0.01416334	0.174199
320500	Tmem215	transmembrane protein 215, transcript variant 2	NA	0.860362	-0.08987	1.815494	0.01418987	0.174313
30785	Cttnbp2	cortactin binding protein 2, transcript variant 2	NA	0.142865	6.536859	1.104096	0.01419403	0.174313
69906	Slc25a32	solute carrier family 25, member 32, transcript varian	NA	0.232054	4.223649	1.174506	0.01420658	0.174336
242915	Garem2	GRB2 associated regulator of MAPK1 subtype 2	NA	-0.21954	4.622706	-1.16437	0.01424111	0.174628
192119	Dicer1	dicer 1, ribonuclease type III	NA	0.140442	6.677055	1.102243	0.01430075	0.175227
72345	Amer1	APC membrane recruitment 1	NA	0.200712	5.538606	1.149266	0.01431186	0.175231
226421	Rab7b	RAB7B, member RAS oncogene family, transcript va	NA	-0.91928	0.101888	-1.89117	0.01432369	0.175244
21912	Tspan7	tetraspanin 7	NA	0.139661	7.854186	1.101646	0.01436773	0.17552
74340	Ahcyl2	S-adenosylhomocysteine hydrolase-like 2, transcript	NA	0.153316	5.528469	1.112123	0.01436776	0.17552
216825	Usp22	ubiquitin specific peptidase 22	NA	0.120266	9.202598	1.086935	0.01439519	0.175671
101540	Prkd2	protein kinase D2, transcript variant 1	NA	-0.28669	3.502247	-1.21983	0.01440171	0.175671
18751	Prkcb	protein kinase C, beta, transcript variant 1	NA	0.169151	7.281781	1.124397	0.01448022	0.176333
170767	Rfxap	regulatory factor X-associated protein	NA	0.185166	5.194018	1.136948	0.01448899	0.176333
319832	Tmem229a	transmembrane protein 229A	NA	0.300312	3.582834	1.231411	0.01449384	0.176333
665858	Gm7827	predicted gene 7827	NA	0.619568	1.487419	1.536415	0.01450615	0.176333
53357	Pla2g6	phospholipase A2, group VI, transcript variant X22	NA	-0.2682	4.682469	-1.2043	0.01451023	0.176333
12337	Capn5	calpain 5, transcript variant X6	NA	-0.20027	5.541888	-1.14891	0.01452774	0.176392
319586	Celf5	CUGBP, Elav-like family member 5, transcript variant	NA	-0.13218	8.514633	-1.09595	0.01454058	0.176392
15270	H2ax	H2A.X variant histone	NA	-0.21083	5.970333	-1.15736	0.01455678	0.176392
268996	Ss18	SS18, nBAF chromatin remodeling complex subunit,	NA	-0.19717	5.677037	-1.14645	0.01455842	0.176392
210933	Adgrb3	adhesion G protein-coupled receptor B3, transcript va	NA	0.147607	6.532071	1.107731	0.01460164	0.176784
22428	Dctn6	dynactin 6, transcript variant X3	NA	0.174405	6.092858	1.128499	0.01464051	0.17699
223267	Ggact	gamma-glutamylamine cyclotransferase, transcript va	NA	0.41944	1.961891	1.337409	0.01465919	0.17699
60534	Fancg	Fanconi anemia, complementation group G, transcrip	NA	-0.22392	4.207184	-1.16791	0.01465943	0.17699
13388	Dll1	delta like canonical Notch ligand 1, transcript variant	NA	-0.20494	4.973785	-1.15264	0.01466211	0.17699
234023	Arglu1	arginine and glutamate rich 1	NA	0.176636	6.948194	1.130246	0.01468591	0.177011
107371	Exoc6	exocyst complex component 6, transcript variant X1	NA	0.16622	5.137863	1.122115	0.01469049	0.177011
246228	Vwa1	von Willebrand factor A domain containing 1, transcri	NA	-0.34229	4.881836	-1.26777	0.01469969	0.177011
232232	Hdac11	histone deacetylase 11	NA	-0.14893	6.098947	-1.10874	0.01471219	0.177011
223649	Nrbp2	nuclear receptor binding protein 2, transcript variant 1	NA	0.153151	7.191549	1.111996	0.01471828	0.177011
243616	Slc6a11	solute carrier family 6 (neurotransmitter transporter, (	NA	0.173979	6.559597	1.128166	0.01475703	0.177346
68728	Trp53inp2	transformation related protein 53 inducible nuclear pr	NA	-0.13817	7.454835	-1.10051	0.014768	0.177347
75744	Svip	small VCP/p97-interacting protein, transcript variant 2	NA	0.198292	4.701556	1.147339	0.01479619	0.177554
15525	Hspa4	heat shock protein 4	NA	-0.14234	7.960809	-1.1037	0.01481461	0.177626
332110	Mapk15	mitogen-activated protein kinase 15, transcript varian	NA	0.375601	2.668778	1.29738	0.01482398	0.177626

12626	Cetn3	centrin 3	NA	0.163123	6.817379	1.119709	0.01484174	0.177708
16795	Large1	LARGE xylosyl- and glucuronyltransferase 1, transcri	NA	0.15414	6.330391	1.112758	0.0148762	0.177989
192232	Hps4	HPS4, biogenesis of lysosomal organelles complex 3	NA	-0.23869	4.519198	-1.17992	0.01488895	0.178011
100038725	Cep85l	centrosomal protein 85-like	NA	0.217359	4.68361	1.162603	0.01494381	0.17838
102636309	Gssos2	glutathione synthase, opposite strand 2, transcript va	NA	0.317094	3.137624	1.245818	0.01495589	0.17838
74359	4931414P19Rik	RIKEN cDNA 4931414P19 gene, transcript variant X	NA	-0.39636	3.381354	-1.31618	0.01497228	0.17838
100952	Emilin1	elastin microfibril interfacer 1	NA	-0.24606	4.078076	-1.18596	0.01498246	0.17838
66863	Lztr1	leucine-zipper-like transcriptional regulator, 1, transcr	NA	-0.14224	6.748887	-1.10362	0.01498638	0.17838
14809	Grik5	glutamate receptor, ionotropic, kainate 5 (gamma 2),	NA	-0.12207	7.634028	-1.0883	0.01499959	0.17838
14170	Fgf15	fibroblast growth factor 15	NA	-0.31822	3.559746	-1.24679	0.01500136	0.17838
209478	Tbc1d12	TBC1D12: TBC1 domain family, member 12, transcri	NA	0.23869	4.974525	1.179921	0.01500752	0.17838
230721	Pabpc4	poly(A) binding protein, cytoplasmic 4, transcript varia	NA	-0.13712	6.62223	-1.0997	0.01502614	0.178471
209334	Gen1	GEN1, Holliday junction 5' flap endonuclease	NA	-0.29515	2.968044	-1.22701	0.01504899	0.178612
320106	Slc38a11	solute carrier family 38, member 11, transcript variant	NA	0.508036	1.348114	1.422112	0.01506145	0.17863
57321	Terf2ip	telomeric repeat binding factor 2, interacting protein	NA	0.138351	6.225497	1.100647	0.01509469	0.178893
102636923	Gm33862	predicted gene, 33862	NA	-0.34144	3.031016	-1.26702	0.0151076	0.178908
269702	Mphosph9	M-phase phosphoprotein 9, transcript variant X12	NA	0.179017	5.102336	1.132112	0.01512257	0.178908
12725	Clcn3	chloride channel, voltage-sensitive 3, transcript variar	NA	0.179172	6.904751	1.132234	0.01512889	0.178908
16391	Irf9	interferon regulatory factor 9, transcript variant 2	NA	-0.25255	4.09798	-1.19131	0.0151479	0.178988
209131	Snx30	sorting nexin family member 30, transcript variant X2	NA	0.193414	5.543405	1.143467	0.01515769	0.178988
403187	Opa3	optic atrophy 3	NA	-0.25889	4.469432	-1.19656	0.01518557	0.179188
12544	Cdc45	cell division cycle 45, transcript variant 1	NA	-0.39654	3.251073	-1.31635	0.01522976	0.179579
403201	5330416C01Rik	RIKEN cDNA 5330416C01 gene, transcript variant 1	NA	-0.9598	-0.4927	-1.94504	0.01528152	0.180032
66464	Taf12	TATA-box binding protein associated factor 12	NA	-0.23474	4.338334	-1.17669	0.01530048	0.180032
16210	Impact	impact, RWD domain protein, transcript variant 2	NA	0.222395	6.023017	1.166668	0.01530873	0.180032
67976	Trabd	TraB domain containing, transcript variant 1	NA	-0.16944	4.998338	-1.12462	0.01532426	0.180032
74185	Gbe1	glucan (1,4-alpha-), branching enzyme 1	NA	0.23617	3.711048	1.177861	0.01532533	0.180032
99526	Usp53	ubiquitin specific peptidase 53, transcript variant X9	NA	0.315733	3.499973	1.244644	0.01533459	0.180032
63828	Fn3k	fructosamine 3 kinase, transcript variant 2	NA	-0.51288	1.748681	-1.42689	0.01534753	0.180054
115486509	Gm12764	predicted gene 12764	NA	-1.16066	-0.59025	-2.2356	0.01536389	0.180116
320038	Dlx6os1	distal-less homeobox 6, opposite strand 1, transcript	NA	-0.30797	5.644696	-1.23796	0.01539467	0.180255
66522	Pgpep1	pyroglutamyl-peptidase I, transcript variant X4	NA	-0.17472	5.439554	-1.12875	0.01539789	0.180255
338349	Cntln	centlein, centrosomal protein, transcript variant X8	NA	0.23027	4.409642	1.173054	0.01542909	0.180368
353237	Pcdhac2	protocadherin alpha subfamily C, 2	NA	0.376146	5.311545	1.29787	0.01542971	0.180368
113868	Acaa1a	acetyl-Coenzyme A acyltransferase 1A, transcript var	NA	-0.15875	5.418015	-1.11632	0.0154514	0.180492
13871	Ercc2	excision repair cross-complementing rodent repair de	NA	-0.17089	5.601949	-1.12575	0.01549262	0.180843
102633283	Gm31145	predicted gene, 31145, transcript variant X9	NA	-0.44473	1.902431	-1.36106	0.01552627	0.181106
110784	Nr3c2	nuclear receptor subfamily 3, group C, member 2	NA	0.402423	2.678586	1.321726	0.01554143	0.181153
242747	Zfp933	zinc finger protein 933, transcript variant X1	NA	-0.22297	4.779425	-1.16714	0.01556868	0.181341
17122	Mxd4	Max dimerization protein 4	NA	-0.22822	4.239561	-1.17139	0.01558938	0.18136
20621	Snn	stannin	NA	0.115114	8.146444	1.083061	0.01560378	0.18136
17084	Ly86	lymphocyte antigen 86	NA	0.505802	1.436698	1.419912	0.01561488	0.18136
26897	Acot1	acyl-CoA thioesterase 1	NA	0.203601	5.263853	1.151569	0.0156242	0.18136
110751	Adam33	a disintegrin and metallopeptidase domain 33, transc	NA	-0.81822	0.83317	-1.76323	0.01562599	0.18136
11419	Asic1	acid-sensing (proton-gated) ion channel 1, transcript	NA	-0.15625	7.025967	-1.11439	0.01568985	0.181938
20602	Ncor2	nuclear receptor co-repressor 2, transcript variant 3	NA	-0.1244	7.282813	-1.09005	0.01571121	0.181938
109676	Ank2	ankyrin 2, brain, transcript variant 3	NA	0.14471	8.458739	1.105508	0.01572802	0.181938
115487949	LOC115487949	uncharacterized LOC115487949, transcript variant X	NA	0.355396	2.915474	1.279337	0.01572855	0.181938
15452	Hprt	hypoxanthine guanine phosphoribosyl transferase	NA	0.193312	5.535553	1.143386	0.01573173	0.181938
26433	Plod3	procollagen-lysine, 2-oxoglutarate 5-dioxygenase 3	NA	-0.15583	5.77606	-1.11406	0.01578779	0.182035
170812	Ahsp	alpha hemoglobin stabilizing protein	NA	-0.58642	2.207002	-1.50152	0.01580133	0.182035
20742	Sptbn1	spectrin beta, non-erythrocytic 1, transcript variant 1	NA	0.142365	8.921158	1.103713	0.01580208	0.182035
26757	Dpysl4	dihydropyrimidinase-like 4	NA	0.145797	7.138065	1.106342	0.01580955	0.182035
68275	Rpa1	replication protein A1, transcript variant 2	NA	-0.16263	5.992465	-1.11932	0.01581269	0.182035
70997	Spef1	sperm flagellar 1	NA	-0.17328	5.394248	-1.12762	0.01581362	0.182035
104570	Ppp4r3b	protein phosphatase 4 regulatory subunit 3B, transcri	NA	0.152918	6.149648	1.111816	0.01581842	0.182035
102634887	Gm13112	predicted gene 13112	NA	0.603481	1.011394	1.519379	0.0158308	0.182049
14245	Lpin1	lipin 1, transcript variant X17	NA	0.169835	5.204168	1.124929	0.01586918	0.182361
73610	Zfp433	zinc finger protein 433, transcript variant X5	NA	-0.95614	3.306774	-1.94011	0.01592065	0.182823
234686	Fhod1	formin homology 2 domain containing 1	NA	-0.32507	2.961549	-1.25272	0.01604598	0.184075
53619	Blcap	bladder cancer associated protein, transcript variant	NA	0.132917	7.414642	1.096508	0.01606011	0.184075
21887	Tle3	transducin-like enhancer of split 3, transcript variant	NA	-0.12749	7.034999	-1.0924	0.01606355	0.184075
18933	Prrx1	paired related homeobox 1, transcript variant 1	NA	-0.24599	4.206473	-1.18591	0.01612166	0.184366
11752	Anxa8	annexin A8, transcript variant 1	NA	-1.28148	0.041569	-2.43089	0.01612588	0.184366
66875	Swt1	SWT1 RNA endoribonuclease homolog (S. cerevisiae	NA	-0.2311	3.858705	-1.17373	0.01615343	0.184366
231452	Sdad1	SDA1 domain containing 1	NA	0.164689	5.275001	1.120925	0.01615812	0.184366
232313	Gxylt2	glucoside xylosyltransferase 2, transcript variant X1	NA	-0.33463	2.834854	-1.26105	0.01616925	0.184366
20384	Srsf5	serine and arginine-rich splicing factor 5, transcript va	NA	0.136918	8.143441	1.099554	0.01618217	0.184366

108903	Tbcd	tubulin-specific chaperone d	NA	0.135074	6.194774	1.098149	0.01618434	0.184366
230075	Ndufb6	NADH:ubiquinone oxidoreductase subunit B6	NA	0.229894	5.215386	1.172749	0.01618815	0.184366
68255	Tmem86b	transmembrane protein 86B, transcript variant X1	NA	-0.36132	3.229526	-1.2846	0.01619096	0.184366
56541	Habp4	hyaluronic acid binding protein 4	NA	0.185838	5.310245	1.137477	0.0162174	0.184413
76670	Cfap70	cilia and flagella associated protein 70, transcript vari	NA	0.695433	0.658697	1.61937	0.01622443	0.184413
216238	Eea1	early endosome antigen 1	NA	0.206563	5.325643	1.153936	0.01622907	0.184413
17220	Mcm7	minichromosome maintenance complex component 7	NA	-0.22426	6.579809	-1.16818	0.01625438	0.18442
58193	Extl2	exostosin-like glycosyltransferase 2, transcript varian	NA	0.177033	4.915513	1.130557	0.01626329	0.18442
76257	Slc38a3	solute carrier family 38, member 3, transcript variant :	NA	0.160666	5.303523	1.117803	0.01627491	0.18442
241589	D430041D05Rik	RIKEN cDNA D430041D05 gene, transcript variant 2	NA	0.135008	6.748132	1.098099	0.01627501	0.18442
17219	Mcm6	minichromosome maintenance complex component 6	NA	-0.14495	6.073963	-1.1057	0.01628874	0.184439
109077	Ints5	integrator complex subunit 5	NA	-0.19568	4.935812	-1.14526	0.01629934	0.184439
216846	Cntrob	centrobin, centrosomal BRCA2 interacting protein	NA	-0.19638	5.314226	-1.14582	0.01633075	0.184544
14313	Fst	follistatin, transcript variant 1	NA	0.372312	2.856855	1.294426	0.01633136	0.184544
74192	Arpc5l	actin related protein 2/3 complex, subunit 5-like	NA	0.181153	5.761387	1.13379	0.01634362	0.184555
214424	Parp16	poly (ADP-ribose) polymerase family, member 16	NA	0.326692	3.024033	1.254134	0.01639164	0.184969
20441	St3gal3	ST3 beta-galactoside alpha-2,3-sialyltransferase 3, tr	NA	-0.16665	5.20669	-1.12245	0.01642871	0.185258
23859	Dlg2	discs large MAGUK scaffold protein 2, transcript varia	NA	0.147101	7.109032	1.107342	0.01644361	0.185298
13618	Ednrb	endothelin receptor type B, transcript variant 1	NA	0.203572	6.672052	1.151546	0.01651527	0.185755
75050	Kif27	kinesin family member 27, transcript variant 2	NA	0.3567	2.847577	1.280494	0.01651971	0.185755
270156	Nkapd1	NKAP domain containing 1, transcript variant 2	NA	0.179856	5.083106	1.132771	0.01652852	0.185755
269642	Nat8l	N-acetyltransferase 8-like	NA	-0.16501	5.767131	-1.12117	0.01656134	0.185755
17158	Man2a1	mannosidase 2, alpha 1	NA	0.242919	5.060667	1.183384	0.01656163	0.185755
212127	Proser1	proline and serine rich 1	NA	-0.15583	5.637405	-1.11406	0.01656865	0.185755
246198	Mllt6	myeloid/lymphoid or mixed-lineage leukemia; transloc	NA	-0.16904	7.08025	-1.12431	0.01657277	0.185755
12891	Cpne6	copine VI, transcript variant 2	NA	0.350871	2.920548	1.27533	0.01658894	0.185755
18639	Pfkfb1	6-phosphofructo-2-kinase/fructose-2,6-biphosphatase	NA	0.457414	1.607093	1.373078	0.01660734	0.185755
73828	Dcaf4	DDB1 and CUL4 associated factor 4, transcript variar	NA	-0.1716	5.32712	-1.12631	0.01661459	0.185755
14570	Arhgdig	Rho GDP dissociation inhibitor (GDI) gamma	NA	0.252797	4.382996	1.191515	0.01662858	0.185755
353156	Egfl7	EGF-like domain 7, transcript variant b	NA	0.236318	3.959255	1.177983	0.01663276	0.185755
28019	Ing4	inhibitor of growth family, member 4, transcript varian	NA	-0.14664	6.475746	-1.10698	0.01663859	0.185755
69207	Srsf11	serine and arginine-rich splicing factor 11, transcript v	NA	0.138045	7.774083	1.100413	0.01664396	0.185755
109168	Atl3	atlastin GTPase 3, transcript variant 2	NA	0.203186	4.91294	1.151238	0.01667107	0.18593
76809	Bri3bp	Bri3 binding protein, transcript variant 1	NA	0.143277	6.613621	1.104411	0.01668574	0.185966
230257	Ptbp3	polypyrimidine tract binding protein 3, transcript varia	NA	0.174888	5.477613	1.128877	0.01672231	0.186151
216856	Nlgn2	neuroligin 2, transcript variant 2	NA	-0.12527	8.429814	-1.09071	0.01672591	0.186151
263803	Pkn3	protein kinase N3, transcript variant X7	NA	-0.50815	1.546835	-1.42223	0.01673669	0.186151
71452	Ankrd40	ankyrin repeat domain 40, transcript variant 1	NA	-0.15146	5.766391	-1.11069	0.01675285	0.186204
117146	Ube3b	ubiquitin protein ligase E3B, transcript variant 1	NA	-0.14723	6.442332	-1.10744	0.01678126	0.186392
73024	Emc7	ER membrane protein complex subunit 7	NA	0.17353	5.40778	1.127815	0.01682431	0.186705
105352	Dusp22	dual specificity phosphatase 22, transcript variant 1	NA	0.24822	4.396333	1.187741	0.01683234	0.186705
22763	Zfr	zinc finger RNA binding protein, transcript variant 2	NA	0.121531	7.576001	1.087888	0.01685338	0.186811
76251	Ercc6l2	excision repair cross-complementing rodent repair de	NA	0.215557	4.572588	1.161152	0.01689161	0.187107
22375	Wars	tryptophanyl-tRNA synthetase, transcript variant 2	NA	-0.14246	6.490768	-1.10378	0.01690416	0.187119
54366	Ctnnal1	catenin (cadherin associated protein), alpha-like 1	NA	0.23674	4.416677	1.178327	0.01693978	0.187386
68187	Fam135a	family with sequence similarity 135, member A, trans	NA	0.190535	5.845335	1.141187	0.01699798	0.187841
23834	Cdc6	cell division cycle 6, transcript variant 2	NA	-0.36239	2.862843	-1.28555	0.01700408	0.187841
73822	Mfsd12	major facilitator superfamily domain containing 12	NA	-0.23691	4.21154	-1.17847	0.01706825	0.18838
69692	Hddc2	HD domain containing 2, transcript variant X1	NA	0.205925	4.30432	1.153426	0.01707598	0.18838
106039	Gga1	golgi associated, gamma adaptin ear containing, ARF	NA	-0.16687	5.985589	-1.12262	0.01709708	0.188485
330450	Far2	fatty acyl CoA reductase 2, transcript variant 1	NA	0.204737	4.55043	1.152477	0.01712026	0.188588
20665	Sox10	SRY (sex determining region Y)-box 10	NA	0.282258	3.189631	1.216096	0.01713605	0.188588
12561	Cdh4	cadherin 4, transcript variant 1	NA	0.133951	7.130519	1.097294	0.01714123	0.188588
329015	Atg2a	autophagy related 2A	NA	-0.15489	5.356378	-1.11334	0.01717211	0.1888
20358	Sema6a	sema domain, transmembrane domain (TM), and cyto	NA	0.134488	7.217309	1.097703	0.01721244	0.188907
224344	Rbm11	RNA binding motif protein 11	NA	0.483086	2.099078	1.39773	0.01722164	0.188907
241638	Lzts3	leucine zipper, putative tumor suppressor family mem	NA	-0.18934	5.490182	-1.14024	0.0172281	0.188907
232813	Shisa7	shisa family member 7, transcript variant X2	NA	-0.1435	7.2762	-1.10458	0.01722823	0.188907
56335	Mettl3	methyltransferase like 3	NA	-0.25973	4.714748	-1.19726	0.01724128	0.188923
103768	Tubg2	tubulin, gamma 2	NA	0.176739	4.905221	1.130326	0.01726056	0.189007
98685	Trmt1l	tRNA methyltransferase 1 like, transcript variant X2	NA	-0.20123	5.249783	-1.14968	0.01727356	0.189022
654467	Gm10052	predicted pseudogene 10052	NA	1.080319	1.492199	2.114503	0.01731951	0.189397
15417	Hoxb9	homeobox B9	NA	1.729873	-0.70011	3.316986	0.01736553	0.189563
16782	Lamc2	laminin, gamma 2, transcript variant X1	NA	0.380207	2.45706	1.301528	0.01736878	0.189563
67533	Ppfibp1	PTPRF interacting protein, binding protein 1 (liprin be	NA	0.22358	4.833067	1.167628	0.01738525	0.189563
14862	Gstm1	glutathione S-transferase, mu 1, transcript variant 2	NA	0.240722	5.196497	1.181584	0.01740176	0.189563
19268	Ptprf	protein tyrosine phosphatase, receptor type, F, transc	NA	-0.11763	7.406567	-1.08495	0.01741238	0.189563
232035	Ccser1	coiled-coil serine rich 1, transcript variant 2	NA	0.170375	5.101261	1.125351	0.01741962	0.189563

241556	Tspan18	tetraspanin 18, transcript variant X1	NA	-0.16236	5.780954	-1.11912	0.01742538	0.189563
76551	Ccdc6	coiled-coil domain containing 6, transcript variant X2	NA	0.147021	6.559325	1.107281	0.01742791	0.189563
72735	Eldr	Egfr long non-coding downstream RNA, transcript va	NA	-0.68524	1.070873	-1.60797	0.01745122	0.18966
101694	Mir9-3hg	Mir9-3 host gene, transcript variant 4	NA	0.170378	5.784332	1.125353	0.01746014	0.18966
269037	Ctif	CBP80/20-dependent translation initiation factor, tran	NA	-0.14897	6.846952	-1.10877	0.01747201	0.189663
107587	Osr2	odd-skipped related 2, transcript variant 2	NA	-1.2631	-0.72849	-2.40012	0.017497	0.189807
108168382	Gm46626	predicted gene, 46626	NA	-0.58203	1.066315	-1.49695	0.01753951	0.19008
269704	Zfp664	zinc finger protein 664	NA	-0.12663	7.237138	-1.09174	0.01755106	0.19008
29856	Smtn	smoothelin, transcript variant 2	NA	-0.26479	4.31208	-1.20146	0.01755713	0.19008
74123	Foxp4	forkhead box P4, transcript variant 3	NA	-0.12931	6.940589	-1.09377	0.0176416	0.190747
71949	Cers5	ceramide synthase 5	NA	0.171733	5.975612	1.12641	0.01764219	0.190747
217265	Abca5	ATP-binding cassette, sub-family A (ABC1), member	NA	0.206228	4.767118	1.153668	0.01766936	0.190914
209540	Rtl9	retrotransposon Gag like 9, transcript variant X2	NA	0.44684	1.895194	1.363051	0.01769891	0.191076
12380	Cast	calpastatin, transcript variant X4	NA	0.289187	3.579098	1.221951	0.0177079	0.191076
56199	Abcb10	ATP-binding cassette, sub-family B (MDR/TAP), men	NA	0.164336	5.34645	1.12065	0.01776616	0.191457
219181	Akap11	A kinase (PRKA) anchor protein 11, transcript variant	NA	0.14651	7.16495	1.106889	0.0177667	0.191457
12123	Hrk	harakiri, BCL2 interacting protein (contains only BH3	NA	0.20567	4.811807	1.153222	0.01778756	0.191502
12671	Chrm3	cholinergic receptor, muscarinic 3, cardiac, transcript	NA	0.234369	3.814318	1.176392	0.01779637	0.191502
50918	Myadm	myeloid-associated differentiation marker, transcript v	NA	-0.16519	5.635416	-1.12131	0.01780618	0.191502
20913	Stxbp4	syntaxin binding protein 4	NA	-0.19425	4.89775	-1.14413	0.01782436	0.191571
14917	Gucy2c	guanylate cyclase 2c, transcript variant 2	NA	0.886983	-0.45036	1.849305	0.0178616	0.191844
102635781	Idi1-ps1	isopentenyl-diphosphate delta isomerase, pseudoger	NA	0.157645	6.021806	1.115465	0.01788636	0.191983
333564	Fndc3c1	fibronectin type III domain containing 3C1, transcript	NA	-0.84242	0.4961	-1.79305	0.01792392	0.192196
269023	Zfp608	zinc finger protein 608, transcript variant X4	NA	0.129622	6.320172	1.094007	0.01792981	0.192196
106200	Txndc11	thioredoxin domain containing 11, transcript variant 1	NA	0.227841	4.655918	1.171081	0.01795149	0.192291
319655	Podxl2	podocalyxin-like 2, transcript variant 2	NA	0.115743	8.430623	1.083533	0.01796232	0.192291
20924	Supt5	suppressor of Ty 5, DSIF elongation factor subunit	NA	-0.14893	7.393219	-1.10875	0.01800842	0.192658
16323	Inhba	inhibin beta-A	NA	0.307221	3.324953	1.237322	0.01802544	0.192713
66447	Mgst3	microsomal glutathione S-transferase 3	NA	0.177277	5.150125	1.130748	0.01808636	0.193111
74387	4932438H23Rik	RIKEN cDNA 4932438H23 gene, transcript variant X	NA	1.391186	0.418073	2.622942	0.01808855	0.193111
53600	Timm23	translocase of inner mitochondrial membrane 23	NA	0.134835	6.20752	1.097967	0.0180982	0.193111
320376	Bcorl1	BCL6 co-repressor-like 1, transcript variant X6	NA	-0.22375	5.667356	-1.16777	0.01816629	0.193517
76137	Mcur1	mitochondrial calcium uniporter regulator 1	NA	0.274902	3.605521	1.209912	0.0181758	0.193517
11459	Acta1	actin, alpha 1, skeletal muscle, transcript variant 2	NA	-0.65055	4.040564	-1.56976	0.01818325	0.193517
381634	Gm1043	predicted gene 1043, transcript variant X2	NA	-0.24709	4.534379	-1.18681	0.01818508	0.193517
218629	Dhx29	DEAH (Asp-Glu-Ala-His) box polypeptide 29	NA	-0.1598	5.522048	-1.11713	0.01823296	0.193517
12850	Coq7	demethyl-Q 7, transcript variant 1	NA	0.233349	4.131174	1.17556	0.01823331	0.193517
71710	Lrrcc1	leucine rich repeat and coiled-coil domain containing	NA	0.243063	5.094134	1.183503	0.01824916	0.193517
211329	Ncoa7	nuclear receptor coactivator 7, transcript variant 2	NA	0.182829	5.185968	1.135108	0.01826373	0.193517
668525	Gm9222	predicted gene 9222, transcript variant 13	NA	0.226289	5.450932	1.169822	0.01826706	0.193517
109161	Ube2q2	ubiquitin-conjugating enzyme E2Q family member 2,	NA	0.153771	5.870835	1.112474	0.01826743	0.193517
17155	Man1a	mannosidase 1, alpha, transcript variant X1	NA	0.260213	4.196695	1.197656	0.01829039	0.193517
66704	Rbm4b	RNA binding motif protein 4B	NA	-0.25222	6.493475	-1.19104	0.01829582	0.193517
16763	Lad1	ladinin	NA	-1.01736	0.465296	-2.02421	0.01829635	0.193517
108062	Cstf2	cleavage stimulation factor, 3' pre-RNA subunit 2, tra	NA	0.141345	6.696701	1.102933	0.01830279	0.193517
320557	Fam169a	family with sequence similarity 169, member A, trans	NA	0.209601	5.760918	1.156368	0.01832959	0.193675
383348	Kctd16	potassium channel tetramerisation domain containing	NA	0.257624	4.088783	1.195508	0.01835323	0.193799
71275	Noxred1	NADP+ dependent oxidoreductase domain containing	NA	-0.70786	0.469145	-1.63338	0.01837595	0.193913
67978	Tctn2	tectonic family member 2	NA	-0.23053	4.126213	-1.17327	0.01839548	0.193948
71330	Rcbtb1	regulator of chromosome condensation (RCC1) and I	NA	0.137898	6.539911	1.1003	0.0184031	0.193948
74126	Syvn1	synovial apoptosis inhibitor 1, synoviolin, transcript va	NA	-0.14748	5.645175	-1.10763	0.01842592	0.194063
216965	Taok1	TAO kinase 1, transcript variant X6	NA	0.15529	7.242167	1.113646	0.01844821	0.194172
171171	Ntng2	netrin G2, transcript variant a	NA	-0.16659	4.954231	-1.1224	0.01846438	0.194216
102635867	Gm33097	predicted gene, 33097	NA	0.625137	1.012099	1.542357	0.01849432	0.194405
107895	Mgat5	mannoside acetylglucosaminyltransferase 5, transcrip	NA	0.190513	5.392037	1.14117	0.01851848	0.194507
232237	Fgd5	FYVE, RhoGEF and PH domain containing 5	NA	0.208396	4.762368	1.155403	0.01855257	0.194507
110380	Shroom2	shroom family member 2, transcript variant X3	NA	0.176088	6.852302	1.129817	0.01855424	0.194507
74008	Arsg	arylsulfatase G, transcript variant 3	NA	0.379771	2.755299	1.301136	0.01856678	0.194507
115487215	Gm7628	predicted gene 7628	NA	0.498652	1.413	1.412893	0.01856791	0.194507
102278	Cpne7	copine VII, transcript variant X2	NA	0.324464	2.673996	1.252199	0.01858618	0.194507
269643	Ppp2r2c	protein phosphatase 2, regulatory subunit B, gamma,	NA	0.122608	7.313395	1.088701	0.01858766	0.194507
26949	Vat1	vesicle amine transport 1	NA	-0.11545	7.703692	-1.08332	0.01860833	0.194532
69554	Klhdc2	kelch domain containing 2	NA	0.119644	7.525345	1.086467	0.01861397	0.194532
104082	Wdr7	WD repeat domain 7, transcript variant 2	NA	0.153261	6.576013	1.11208	0.01863903	0.194624
23917	Impdh1	inosine monophosphate dehydrogenase 1, transcript	NA	-0.16736	5.691568	-1.12301	0.01865074	0.194624
101706	Numa1	nuclear mitotic apparatus protein 1	NA	-0.20823	7.127986	-1.15527	0.01865863	0.194624
214812	Zfp609	zinc finger protein 609, transcript variant X2	NA	-0.17127	7.025996	-1.12605	0.01868575	0.194782
242384	Lingo2	leucine rich repeat and Ig domain containing 2, trans	NA	0.176475	4.935681	1.130119	0.01871199	0.194931

12859	Cox5b	cytochrome c oxidase subunit 5B	NA	0.147756	6.466096	1.107845	0.01876695	0.19497
59029	Psmd14	proteasome (prosome, macropain) 26S subunit, non-	NA	0.136557	6.95052	1.099278	0.01879528	0.19497
544922	Zkscan4	zinc finger with KRAB and SCAN domains 4	NA	0.288467	3.265806	1.221342	0.01881874	0.19497
56367	Scoc	short coiled-coil protein, transcript variant 1	NA	0.118442	6.9724	1.085562	0.01883034	0.19497
14816	Grm1	glutamate receptor, metabotropic 1, transcript variant	NA	0.18835	4.56956	1.13946	0.01883772	0.19497
11990	Atrn	attractin	NA	0.165634	6.688801	1.121659	0.01886466	0.19497
17879	Myh1	myosin, heavy polypeptide 1, skeletal muscle, adult	NA	0.947651	-0.56773	1.928729	0.01886505	0.19497
102632845	Gm30810	predicted gene, 30810, transcript variant X5	NA	0.751232	0.202107	1.68323	0.0188757	0.19497
22027	Hsp90b1	heat shock protein 90, beta (Grp94), member 1	NA	-0.13128	8.476226	-1.09527	0.01888379	0.19497
11567	Avil	advillin	NA	0.585619	1.757176	1.500682	0.01888798	0.19497
217682	Plekhd1	pleckstrin homology domain containing, family D (with	NA	0.401734	1.979972	1.321095	0.01888851	0.19497
241919	Slc7a14	solute carrier family 7 (cationic amino acid transporte	NA	0.251471	4.834049	1.19042	0.01890533	0.19497
230674	Kdm4a	lysine (K)-specific demethylase 4A, transcript variant	NA	-0.15945	7.39345	-1.11686	0.0189054	0.19497
50766	Crim1	cysteine rich transmembrane BMP regulator 1 (chord	NA	0.2148	5.698951	1.160543	0.0189142	0.19497
94230	Cpsf1	cleavage and polyadenylation specific factor 1, transc	NA	-0.15507	6.399452	-1.11347	0.01891453	0.19497
66179	Ogfod3	2-oxoglutarate and iron-dependent oxygenase domai	NA	0.300903	3.027697	1.231915	0.01892547	0.19497
12971	Crym	crystallin, mu	NA	0.219872	4.130132	1.16463	0.01894435	0.19497
104798	Sfta3-ps	surfactant associated 3, pseudogene	NA	-0.48312	2.271505	-1.39776	0.01895819	0.19497
106766	Stap2	signal transducing adaptor family member 2, transcrip	NA	0.409802	1.889326	1.328503	0.01897164	0.19497
115488611	Gm52229	predicted gene, 52229	NA	0.173383	5.473783	1.1277	0.01899303	0.19497
30953	Schip1	schwannomin interacting protein 1, transcript variant	NA	0.167816	6.537574	1.123356	0.01899326	0.19497
224902	Safb2	scaffold attachment factor B2, transcript variant X12	NA	-0.14679	6.428491	-1.1071	0.01902212	0.19497
15528	Hspe1	heat shock protein 1 (chaperonin 10)	NA	-0.13706	6.208614	-1.09966	0.01903208	0.19497
19043	Ppm1b	protein phosphatase 1B, magnesium dependent, beta	NA	0.126996	6.739149	1.092018	0.01904008	0.19497
11841	Arf2	ADP-ribosylation factor 2, transcript variant 2	NA	0.132403	7.246671	1.096118	0.01904042	0.19497
19159	Cyth3	cytohesin 3, transcript variant X3	NA	0.148894	5.835874	1.108719	0.0190476	0.19497
67042	Ift27	intraflagellar transport 27	NA	0.208101	4.758431	1.155167	0.01904834	0.19497
76220	6530402F18Rik	RIKEN cDNA 6530402F18 gene	NA	-0.26713	4.406549	-1.20342	0.01905125	0.19497
214895	Lman2l	lectin, mannose-binding 2-like, transcript variant X4	NA	-0.23502	6.025646	-1.17693	0.01910524	0.195399
54338	Slc23a2	solute carrier family 23 (nucleobase transporters), me	NA	0.140446	5.978922	1.102246	0.019188	0.196123
16569	Kif3b	kinesin family member 3B	NA	0.152729	6.491126	1.111671	0.01923679	0.196468
101943	Sf3b3	splicing factor 3b, subunit 3	NA	-0.13107	7.287033	-1.0951	0.01927222	0.196468
99870	AW047730	expressed sequence AW047730	NA	0.303892	3.213811	1.23447	0.01927515	0.196468
53893	Nudt5	nudix (nucleoside diphosphate linked moiety X)-type	NA	-0.21454	4.440945	-1.16034	0.0192767	0.196468
14266	Aff2	AF4/FMR2 family, member 2, transcript variant X5	NA	0.140902	5.945086	1.102595	0.01928219	0.196468
76014	Zc3h18	zinc finger CCCH-type containing 18, transcript variai	NA	-0.14336	6.213966	-1.10447	0.01935099	0.196956
59095	Fxyd6	FXYD domain-containing ion transport regulator 6	NA	0.12975	8.251507	1.094104	0.01935432	0.196956
21812	Tgfbr1	transforming growth factor, beta receptor I, transcript	NA	0.128631	6.536175	1.093256	0.01938246	0.197004
15081	H3f3b	H3.3 histone B	NA	0.129619	10.14876	1.094005	0.01938316	0.197004
17748	Mt1	metallothionein 1	NA	0.358903	2.824	1.28245	0.01939574	0.197008
102632914	Gm30866	predicted gene, 30866	NA	-0.73782	0.48407	-1.66766	0.0194226	0.197104
71690	Esm1	endothelial cell-specific molecule 1	NA	0.599455	0.798938	1.515144	0.01944191	0.197104
118568062	LOC118568062	uncharacterized LOC118568062	NA	0.43751	1.952786	1.354264	0.0194605	0.197104
105948585	Gm45928	predicted gene, 45928, transcript variant 2	NA	-1.58364	-1.1241	-2.99724	0.01946335	0.197104
100382	AW011738	expressed sequence AW011738	NA	0.478557	1.54904	1.393349	0.0194657	0.197104
100169868	Gm3173	predicted gene 3173, transcript variant 5	NA	0.435291	3.144963	1.352184	0.01948648	0.197192
97487	Cmtm4	CKLF-like MARVEL transmembrane domain containi	NA	0.182324	6.125031	1.13471	0.01951509	0.197358
118568180	LOC118568180	uncharacterized LOC118568180, transcript variant X	NA	1.25872	-0.58818	2.392834	0.01953299	0.197415
68067	Mrnip	MRN complex interacting protein	NA	0.234992	3.776582	1.1769	0.01954493	0.197415
12649	Chek1	checkpoint kinase 1	NA	-0.30295	3.326778	-1.23366	0.01958194	0.19764
67014	Riox2	ribosomal oxygenase 2	NA	-0.34216	3.358386	-1.26765	0.01959949	0.19764
74094	Tjap1	tight junction associated protein 1, transcript variant X	NA	-0.2346	4.61672	-1.17658	0.01960366	0.19764
626415	4930467E23Rik	RIKEN cDNA 4930467E23 gene, transcript variant X	NA	-0.4269	2.593903	-1.34434	0.0196184	0.197666
320209	Ddx11	DEAD/H box helicase 11, transcript variant 2	NA	-0.39441	3.36637	-1.31441	0.01965339	0.197896
71946	Endod1	endonuclease domain containing 1, transcript variant	NA	0.207271	4.94529	1.154502	0.01966861	0.197927
77045	Bcl7a	B cell CLL/lymphoma 7A	NA	-0.14796	7.465094	-1.108	0.01969476	0.198067
105244006	Gm39701	predicted gene, 39701	NA	-0.3903	2.433721	-1.31067	0.0197096	0.198094
108653	Rimklb	ribosomal modification protein rimK-like family memb	NA	0.108706	8.009284	1.078261	0.01972351	0.198112
240322	Adamts19	a disintegrin-like and metallopeptidase (reprolysin typ	NA	0.47445	2.065166	1.389388	0.01974718	0.198145
56320	Dbn1	drebrin 1, transcript variant 3	NA	-0.12816	9.314346	-1.0929	0.01975113	0.198145
56554	Raet1d	retinoic acid early transcript delta	NA	-0.79321	0.221881	-1.73292	0.01982385	0.198659
73229	Zfp983	zinc finger protein 983, transcript variant X1	NA	-0.18135	4.784959	-1.13395	0.01982688	0.198659
13599	Ecel1	endothelin converting enzyme-like 1, transcript variar	NA	-0.22408	4.499818	-1.16803	0.01988265	0.198903
240505	Cdc42bpg	CDC42 binding protein kinase gamma (DMPK-like)	NA	-0.29241	3.771546	-1.22468	0.01988352	0.198903
13433	Dnmt1	DNA methyltransferase (cytosine-5) 1, transcript varia	NA	-0.12735	7.265505	-1.09229	0.01988788	0.198903
101471	Phrf1	PHD and ring finger domains 1	NA	-0.17054	6.54074	-1.12548	0.01994607	0.199076
19062	Inpp5k	inositol polyphosphate 5-phosphatase K	NA	0.163874	5.268597	1.120292	0.0199506	0.199076
319970	B230323A14Rik	RIKEN cDNA B230323A14 gene	NA	0.604719	0.591128	1.520683	0.0199524	0.199076

67101	2310039H08Rik	RIKEN cDNA 2310039H08 gene	NA	0.337417	2.719352	1.263493	0.0199541	0.199076
21419	Tfap2b	transcription factor AP-2 beta, transcript variant 1	NA	0.19499	7.275586	1.144716	0.01998091	0.199221
108169011	Gm46892	predicted gene, 46892	NA	-0.30672	3.883281	-1.23689	0.02002271	0.199516
54401	Ywhab	tyrosine 3-monooxygenase/tryptophan 5-monooxyge	NA	0.122192	9.010729	1.088387	0.02005541	0.199653
330474	Zc3h4	zinc finger CCCH-type containing 4	NA	-0.14786	6.940082	-1.10792	0.02006097	0.199653
24012	Rgs7	regulator of G protein signaling 7, transcript variant X	NA	0.188982	5.176456	1.139959	0.02009765	0.199895
110279	Bcr	BCR activator of RhoGEF and GTPase	NA	-0.1844	7.206938	-1.13634	0.02011281	0.199924
218442	Serinc5	serine incorporator 5	NA	0.145724	5.779769	1.106285	0.02014059	0.200078
94232	Ubqln4	ubiquilin 4	NA	-0.12788	6.982391	-1.09269	0.02021494	0.200654
78283	Map7d2	MAP7 domain containing 2, transcript variant 11	NA	0.157817	5.520009	1.115598	0.02024311	0.200654
100505034	Gm29695	predicted gene, 29695, transcript variant X2	NA	-0.22012	4.789855	-1.16483	0.02027242	0.200654
68152	Fam133b	family with sequence similarity 133, member B	NA	0.176657	5.236266	1.130262	0.02028418	0.200654
69202	Ptms	parathymosin	NA	0.11454	9.404825	1.08263	0.02028888	0.200654
18632	Pex11b	peroxisomal biogenesis factor 11 beta, transcript vari	NA	0.229183	4.905697	1.172171	0.0202896	0.200654
12797	Cnn1	calponin 1, transcript variant X1	NA	1.070571	-0.9251	2.100264	0.02030347	0.200654
13660	Ehd1	EH-domain containing 1	NA	-0.14011	6.857994	-1.10199	0.02030637	0.200654
245877	Map7d1	MAP7 domain containing 1, transcript variant 3	NA	-0.12191	7.880098	-1.08817	0.02032705	0.200654
67430	4921536K21Rik	RIKEN cDNA 4921536K21 gene	NA	-0.60981	0.842657	-1.52606	0.02032731	0.200654
67177	Cdt1	chromatin licensing and DNA replication factor 1	NA	-0.24698	3.523417	-1.18672	0.02033417	0.200654
20218	Khdrbs1	KH domain containing, RNA binding, signal transduct	NA	-0.11652	8.553231	-1.08412	0.02037484	0.200933
380785	Begain	brain-enriched guanylate kinase-associated, transcrip	NA	0.161368	5.906286	1.118347	0.02039359	0.200996
17136	Mag	myelin-associated glycoprotein, transcript variant 7	NA	0.549562	1.008495	1.463641	0.02044181	0.201254
243842	Bicra	BRD4 interacting chromatin remodeling complex ass	NA	-0.18695	5.914731	-1.13835	0.02044453	0.201254
83397	Akap12	A kinase (PRKA) anchor protein (gravin) 12	NA	0.185055	6.786798	1.13686	0.02046505	0.201335
75782	Lca5	Leber congenital amaurosis 5 (human), transcript var	NA	0.193914	4.85027	1.143863	0.02048764	0.201435
105244666	Gm40232	predicted gene, 40232	NA	-0.7368	0.111197	-1.66648	0.0205299	0.201564
69890	Zfp219	zinc finger protein 219, transcript variant X1	NA	-0.13824	6.501765	-1.10056	0.02053282	0.201564
13543	Dvl2	dishevelled segment polarity protein 2	NA	-0.18987	5.035095	-1.14066	0.02053793	0.201564
67204	Eif2s2	eukaryotic translation initiation factor 2, subunit 2 (be	NA	0.120898	7.390951	1.087411	0.02057119	0.201769
12294	Cacna2d3	calcium channel, voltage-dependent, alpha2/delta su	NA	0.202843	5.430935	1.150965	0.0205916	0.201847
66249	Pno1	partner of NOB1 homolog	NA	0.196157	4.699269	1.145642	0.0206057	0.201864
66131	Tipin	timeless interacting protein, transcript variant X4	NA	0.182091	4.935247	1.134527	0.02063225	0.202002
56626	Poll	polymerase (DNA directed), lambda, transcript varian	NA	-0.28013	3.367403	-1.21431	0.02065124	0.202033
66816	Thap2	THAP domain containing, apoptosis associated prote	NA	-0.21256	5.040176	-1.15874	0.02067903	0.202033
17391	Mmp24	matrix metallopeptidase 24	NA	-0.14157	7.047833	-1.1031	0.02069064	0.202033
104776	Aldh6a1	aldehyde dehydrogenase family 6, subfamily A1, tran	NA	0.193014	5.05166	1.14315	0.0206918	0.202033
214951	Rhbdl1	rhomboid like 1	NA	0.246816	3.959466	1.186586	0.02069743	0.202033
54723	Tfip11	tuftelin interacting protein 11	NA	-0.16185	5.595903	-1.11872	0.02073929	0.202279
97165	Hmgb2	high mobility group box 2, transcript variant 2	NA	-0.18199	7.135775	-1.13445	0.02074748	0.202279
93898	Cers1	ceramide synthase 1	NA	-0.19363	5.027689	-1.14364	0.02078146	0.202387
353211	Prune2	prune homolog 2	NA	0.283809	4.008137	1.217405	0.02078343	0.202387
50817	Capn15	calpain 15, transcript variant X5	NA	-0.17735	5.541771	-1.13081	0.02081122	0.202536
20361	Sema7a	sema domain, immunoglobulin domain (Ig), and GPI	NA	-0.2163	5.887203	-1.16175	0.02087026	0.202989
236727	Slc9a7	solute carrier family 9 (sodium/hydrogen exchanger),	NA	0.23924	5.081999	1.180371	0.02088717	0.203032
22589	Atrx	ATRX, chromatin remodeler	NA	0.130169	8.178527	1.094422	0.02091466	0.203178
67412	Soga3	SOGA family member 3	NA	-0.15457	6.324914	-1.11309	0.02095014	0.203401
22151	Tubb2a	tubulin, beta 2A class IIA	NA	0.148028	10.05794	1.108054	0.0209719	0.203491
319371	D030028A08Rik	RIKEN cDNA D030028A08 gene	NA	-0.26201	3.614103	-1.19915	0.02103506	0.203982
252868	Odf4	outer dense fiber of sperm tails 4	NA	0.729866	0.449117	1.658485	0.02107295	0.204228
433406	Gm13363	predicted gene 13363	NA	-1.59759	-0.6413	-3.02637	0.02110659	0.204295
223642	Zc3h3	zinc finger CCCH type containing 3	NA	-0.22164	5.024638	-1.16606	0.02112213	0.204295
118568526	LOC118568526	uncharacterized LOC118568526	NA	0.405378	2.024401	1.324436	0.02113794	0.204295
230936	Phf13	PHD finger protein 13	NA	-0.19292	5.198676	-1.14308	0.02114728	0.204295
70719	Arhgap45	Rho GTPase activating protein 45, transcript variant 2	NA	-0.64705	1.459631	-1.56596	0.02114858	0.204295
69739	2410004I01Rik	RIKEN cDNA 2410004I01 gene	NA	-0.81641	-0.2196	-1.76102	0.02115515	0.204295
101869	Unc45a	unc-45 myosin chaperone A	NA	-0.14525	5.662255	-1.10592	0.02121425	0.204658
268977	Ltbp1	latent transforming growth factor beta binding protein	NA	-0.23185	3.794791	-1.17434	0.02126071	0.204658
70357	Kcnip1	Kv channel-interacting protein 1, transcript variant X1	NA	0.173873	5.763771	1.128083	0.02126219	0.204658
67866	Wfdc1	WAP four-disulfide core domain 1	NA	0.610653	1.051677	1.52695	0.02126433	0.204658
22770	Zhx1	zinc fingers and homeoboxes 1, transcript variant 1	NA	0.14055	6.093809	1.102325	0.02126898	0.204658
67171	Dram2	DNA-damage regulated autophagy modulator 2, tran	NA	0.194688	4.948779	1.144477	0.02127008	0.204658
115487361	Gm51748	predicted gene, 51748	NA	-0.33136	2.613449	-1.2582	0.02128084	0.204658
17295	Met	met proto-oncogene, transcript variant X2	NA	0.29698	3.179155	1.22857	0.02129524	0.204676
109658	Txlna	taxilin alpha, transcript variant 2	NA	-0.17796	6.458984	-1.13128	0.02132464	0.204796
55936	Ctps2	cytidine 5'-triphosphate synthase 2, transcript variant	NA	0.14615	5.931915	1.106613	0.02133928	0.204796
13592	Ebf2	early B cell factor 2, transcript variant 3	NA	0.244944	4.730808	1.185047	0.02134549	0.204796
102635707	2600014E21Rik	RIKEN cDNA 2600014E21 gene, transcript variant X	NA	-0.28585	3.320247	-1.21913	0.02140597	0.205108
100039495	Gm15706	predicted gene 15706	NA	0.290917	3.067593	1.223418	0.02140651	0.205108

319555	Nwd1	NACHT and WD repeat domain containing 1, transcri	NA	0.215284	4.260855	1.160933	0.02141584	0.205108
17690	Msi1	musashi RNA-binding protein 1, transcript variant 1	NA	-0.11264	7.78979	-1.0812	0.02143684	0.205188
55960	Ebag9	estrogen receptor-binding fragment-associated gene	NA	0.216143	4.41833	1.161624	0.0214587	0.205277
102339	Cog4	component of oligomeric golgi complex 4, transcript v	NA	-0.17079	5.611486	-1.12567	0.02149775	0.20553
67886	Camsap2	calmodulin regulated spectrin-associated protein fam	NA	0.131761	7.557467	1.09563	0.02151902	0.205612
242785	Klhl21	kelch-like 21	NA	-0.37874	2.926149	-1.3002	0.02153612	0.205655
72119	Tpx2	TPX2, microtubule-associated, transcript variant X1	NA	-0.24935	5.604272	-1.18867	0.02155008	0.205667
14619	Gjb2	gap junction protein, beta 2	NA	-0.32103	3.61256	-1.24923	0.02158079	0.205805
22290	Uty	ubiquitously transcribed tetratricopeptide repeat conta	NA	0.459254	3.608935	1.37483	0.02160257	0.205805
102633149	9430053O09Rik	RIKEN cDNA 9430053O09 gene, transcript variant X	NA	0.198867	4.682091	1.147797	0.02161163	0.205805
233744	Spon1	spondin 1, (f-spondin) extracellular matrix protein	NA	0.203617	6.100372	1.151582	0.02162446	0.205805
74016	Phf19	PHD finger protein 19, transcript variant X5	NA	-0.41817	2.404474	-1.33623	0.02162771	0.205805
68603	Pmvk	phosphomevalonate kinase, transcript variant 1	NA	0.167171	5.613551	1.122855	0.02164428	0.205842
230088	Fam214b	family with sequence similarity 214, member B, transc	NA	-0.15257	5.970564	-1.11155	0.02167224	0.205873
80750	N4bp1	NEDD4 binding protein 1	NA	-0.15893	5.972691	-1.11646	0.02168005	0.205873
66821	Bcs1l	BCS1-like (yeast), transcript variant 2	NA	-0.25939	4.065298	-1.19697	0.02168548	0.205873
240880	Scyl3	SCY1-like 3 (S. cerevisiae), transcript variant 2	NA	0.233932	4.888249	1.176036	0.02170005	0.205891
75951	4930578M01Rik	RIKEN cDNA 4930578M01 gene	NA	-0.90734	-0.05291	-1.87559	0.02171595	0.205922
241175	Cntnap5b	contactin associated protein-like 5B, transcript varian	NA	0.193709	4.584206	1.1437	0.02173697	0.205929
26443	Psma6	proteasome subunit alpha 6	NA	0.136175	7.022611	1.098987	0.02174203	0.205929
68259	Ift80	intraflagellar transport 80	NA	-0.20784	4.377127	-1.15496	0.02176685	0.20593
23928	Lamc3	laminin gamma 3	NA	-0.21069	3.932692	-1.15724	0.02176743	0.20593
12238	Commd3	COMM domain containing 3	NA	0.140982	6.020362	1.102656	0.02180542	0.20617
20266	Scn1b	sodium channel, voltage-gated, type I, beta	NA	0.346818	3.033337	1.271753	0.02182932	0.206276
214106	4933430I17Rik	RIKEN cDNA 4933430I17 gene, transcript variant X2	NA	0.58253	1.471951	1.497473	0.0218687	0.206528
16341	Eif3e	eukaryotic translation initiation factor 3, subunit E	NA	0.122872	7.580196	1.088901	0.02188623	0.206565
333193	Proser3	proline and serine rich 3, transcript variant 3	NA	-0.2473	3.858567	-1.18698	0.02189804	0.206565
11512	Adcy6	adenylate cyclase 6, transcript variant 2	NA	-0.13842	6.214533	-1.1007	0.02191285	0.206585
252838	Tox	thymocyte selection-associated high mobility group b	NA	0.1674	5.838769	1.123032	0.02193091	0.206636
380969	Nckap5l	NCK-associated protein 5-like	NA	-0.15518	5.85479	-1.11356	0.02196738	0.206691
217473	Ankmy2	ankyrin repeat and MYND domain containing 2	NA	0.142104	6.023921	1.103514	0.02196951	0.206691
57745	Zfp112	zinc finger protein 112, transcript variant X5	NA	0.397381	2.435667	1.317114	0.0219884	0.206691
381067	Zfp229	zinc finger protein 229, transcript variant X5	NA	0.257251	4.315516	1.195199	0.021993	0.206691
277360	Prex1	phosphatidylinositol-3,4,5-trisphosphate-dependent F	NA	-0.138	6.309368	-1.10038	0.02200384	0.206691
16803	Lbp	lipopolysaccharide binding protein	NA	0.441965	1.784265	1.358453	0.02201296	0.206691
108811	Ccdc122	coiled-coil domain containing 122, transcript variant 1	NA	0.860465	-0.33167	1.815624	0.02207467	0.206777
235036	Ppan	peter pan homolog	NA	-0.19633	4.654512	-1.14578	0.02208688	0.206777
74770	Hhatl	hedgehog acyltransferase-like, transcript variant X6	NA	0.764403	0.290483	1.698667	0.0221143	0.206777
74360	Cep57	centrosomal protein 57, transcript variant 1	NA	0.163072	5.209396	1.119669	0.02212546	0.206777
15135	Hbb-y	hemoglobin Y, beta-like embryonic chain	NA	-0.56002	2.90101	-1.47429	0.02213766	0.206777
433759	Hdac1	histone deacetylase 1	NA	-0.17108	6.089931	-1.1259	0.02214389	0.206777
108946	Zzz3	zinc finger, ZZ domain containing 3, transcript variant	NA	0.170644	6.195812	1.125561	0.02214415	0.206777
226251	Ablim1	actin-binding LIM protein 1, transcript variant X32	NA	0.121994	7.061972	1.088238	0.02215031	0.206777
98366	Smap1	small ArfGAP 1, transcript variant 2	NA	0.119093	7.101428	1.086052	0.02215132	0.206777
432842	LOC432842	uncharacterized LOC432842	NA	0.389748	2.09893	1.310165	0.02216685	0.206777
56089	Ramp3	receptor (calcitonin) activity modifying protein 3	NA	0.518358	2.137679	1.432325	0.0221715	0.206777
330361	Gcfc2	GC-rich sequence DNA binding factor 2, transcript va	NA	0.256701	3.958456	1.194743	0.02217466	0.206777
70099	Smc4	structural maintenance of chromosomes 4, transcript	NA	-0.17939	6.363655	-1.1324	0.02222199	0.20702
666892	Gm8350	predicted gene 8350	NA	0.495516	1.568702	1.409825	0.02224483	0.20702
26431	Git2	GIT ArfGAP 2, transcript variant 2	NA	-0.14715	5.742374	-1.10738	0.02225191	0.20702
16531	Kcnma1	potassium large conductance calcium-activated chan	NA	0.169202	6.315764	1.124436	0.02226728	0.20702
108934	Smim13	small integral membrane protein 13	NA	0.155246	6.177384	1.113611	0.02227395	0.20702
118567384	LOC118567384	UDP-glucuronic acid decarboxylase 1-like	NA	0.588745	1.68688	1.503938	0.022277	0.20702
19041	Ppl	periplakin	NA	-1.37066	0.665789	-2.58589	0.02231032	0.207211
12567	Cdk4	cyclin-dependent kinase 4, transcript variant 1	NA	-0.12188	8.073319	-1.08815	0.0223329	0.207302
67418	Ppil4	peptidylprolyl isomerase (cyclophilin)-like 4	NA	0.152397	5.945097	1.111415	0.02235926	0.207429
99738	Kcnc4	potassium voltage gated channel, Shaw-related subfa	NA	0.239662	4.540479	1.180716	0.02246982	0.208232
74136	Sec14l1	SEC14-like lipid binding 1, transcript variant 2	NA	-0.17869	6.975536	-1.13186	0.02248526	0.208232
64176	Sv2b	synaptic vesicle glycoprotein 2 b, transcript variant 7	NA	0.197021	4.615268	1.146329	0.02249792	0.208232
118567333	LOC118567333	uncharacterized LOC118567333	NA	0.821797	-0.33376	1.767606	0.02250823	0.208232
80733	Car15	carbonic anhydrase 15	NA	-0.51254	1.358782	-1.42656	0.02250983	0.208232
14704	Gng3	guanine nucleotide binding protein (G protein), gamm	NA	0.138703	7.991013	1.100915	0.02253057	0.208305
353169	Slc2a12	solute carrier family 2 (facilitated glucose transporter)	NA	0.356405	3.018064	1.280231	0.02255036	0.20837
115487446	Gm51797	predicted gene, 51797	NA	-0.64102	0.602015	-1.55943	0.02259088	0.208626
66790	Grtp1	GH regulated TBC protein 1, transcript variant 1	NA	0.266077	3.174113	1.202533	0.02261127	0.208696
26913	Gprin1	G protein-regulated inducer of neurite outgrowth 1, tra	NA	-0.11403	7.878875	-1.08225	0.0226465	0.208876
102442	Dennd4a	DENN/MADD domain containing 4A	NA	0.178729	5.010201	1.131887	0.02265646	0.208876
77521	Mtus2	microtubule associated tumor suppressor candidate 2	NA	0.161216	5.429583	1.118229	0.02267366	0.208914

14077	Fabp3	fatty acid binding protein 3, muscle and heart	NA	0.168346	5.337267	1.123769	0.02270374	0.208914
103677	Smg6	Smg-6 homolog, nonsense mediated mRNA decay fa	NA	-0.14076	5.83766	-1.10249	0.02272127	0.208914
102636387	Gm33467	predicted gene, 33467, transcript variant X1	NA	-0.63689	1.36892	-1.55497	0.02272472	0.208914
66871	Cpne8	copine VIII, transcript variant 1	NA	0.262336	4.040639	1.199419	0.02272482	0.208914
57875	Angptl4	angiopoietin-like 4	NA	-0.3621	2.906928	-1.2853	0.02274764	0.209006
330173	2610524H06Rik	RIKEN cDNA 2610524H06 gene	NA	0.202016	4.47003	1.150305	0.02279068	0.209283
20901	Strap	serine/threonine kinase receptor associated protein	NA	0.15741	7.473377	1.115283	0.02281391	0.209378
100038560	Gm10687	predicted gene 10687	NA	0.666498	0.242962	1.587215	0.02286164	0.209611
76857	Spopl	speckle-type BTB/POZ protein-like, transcript variant	NA	0.260622	4.837544	1.197995	0.02286503	0.209611
104479	Ccdc117	coiled-coil domain containing 117	NA	-0.2232	4.618616	-1.16732	0.02290032	0.209816
78771	Mctp1	multiple C2 domains, transmembrane 1, transcript va	NA	0.324396	3.140135	1.25214	0.02301448	0.210562
27967	Cherp	calcium homeostasis endoplasmic reticulum protein,	NA	-0.13922	6.54756	-1.10131	0.02302523	0.210562
654824	Ankrd37	ankyrin repeat domain 37	NA	0.332939	2.853683	1.259576	0.02302932	0.210562
66775	Hacd4	3-hydroxyacyl-CoA dehydratase 4, transcript variant	NA	0.389258	2.167423	1.309719	0.02303347	0.210562
102639700	Gm21297	predicted gene, 21297	NA	0.289636	3.100923	1.222332	0.02313409	0.210969
213582	Map9	microtubule-associated protein 9, transcript variant 1	NA	0.139574	7.424683	1.10158	0.02314293	0.210969
193736	Zbtb12	zinc finger and BTB domain containing 12, transcript	NA	-0.14945	6.62949	-1.10915	0.02314507	0.210969
19653	Rbm4	RNA binding motif protein 4, transcript variant 5	NA	-0.20299	5.11137	-1.15108	0.02315514	0.210969
100042277	Gm3764	predicted gene 3764, transcript variant 3	NA	0.173233	6.927098	1.127582	0.02316865	0.210969
381353	Ajm1	apical junction component 1	NA	-0.19845	4.908008	-1.14747	0.02318025	0.210969
235606	Apeh	acylpeptide hydrolase	NA	-0.19021	4.878174	-1.14093	0.0231812	0.210969
24070	Mpdu1	mannose-P-dolichol utilization defect 1, transcript var	NA	0.165419	5.28625	1.121492	0.02318172	0.210969
75564	Rsph9	radial spoke head 9 homolog (Chlamydomonas)	NA	0.285889	3.611803	1.219162	0.02322894	0.211281
12297	Cacnb3	calcium channel, voltage-dependent, beta 3 subunit,	NA	-0.10722	7.779724	-1.07715	0.02324327	0.211288
115487962	Gm51968	predicted gene, 51968	NA	0.182907	4.613011	1.135169	0.02326053	0.211288
373852	4833422C13Rik	RIKEN cDNA 4833422C13 gene, transcript variant 3	NA	0.513415	1.119328	1.427425	0.02327728	0.211288
233789	Smg1	SMG1 homolog, phosphatidylinositol 3-kinase-relatec	NA	0.147205	7.012998	1.107422	0.02328172	0.211288
227835	Gtdc1	glycosyltransferase-like domain containing 1, transcri	NA	0.152105	5.692219	1.111189	0.02331488	0.211471
97961	Nol12	nucleolar protein 12, transcript variant X3	NA	0.173956	4.721065	1.128148	0.02333328	0.21152
72655	Snhg5	small nucleolar RNA host gene 5	NA	-0.17499	5.055795	-1.12895	0.02336141	0.211572
73047	Camk2n2	calcium/calmodulin-dependent protein kinase II inhibi	NA	0.164988	6.222955	1.121157	0.02338567	0.211572
192786	Rapgef6	Rap guanine nucleotide exchange factor (GEF) 6, tra	NA	0.176501	5.567253	1.130139	0.02338665	0.211572
21937	Tnfrsf1a	tumor necrosis factor receptor superfamily, member	NA	-0.24673	3.916854	-1.18651	0.0233918	0.211572
12283	Cab39	calcium binding protein 39, transcript variant X3	NA	0.125306	7.642988	1.090739	0.02341565	0.211572
20257	Stmn2	stathmin-like 2	NA	0.115843	10.04294	1.083608	0.02341994	0.211572
75687	Ripor1	RHO family interacting cell polarization regulator 1, tr	NA	-0.13263	6.421596	-1.09629	0.02342997	0.211572
240185	Jcad	junctional cadherin 5 associated	NA	0.168794	5.13695	1.124119	0.02346163	0.21174
83767	Wasf1	WASP family, member 1, transcript variant 1	NA	0.113488	7.225132	1.081841	0.02350442	0.212009
13347	Dffa	DNA fragmentation factor, alpha subunit, transcript va	NA	-0.2181	4.26304	-1.1632	0.02352019	0.212034
14545	Gdap1	ganglioside-induced differentiation-associated-proteir	NA	0.12445	6.920405	1.090092	0.02354063	0.2121
546100	Gm5914	predicted gene 5914, transcript variant 2	NA	0.627792	1.184163	1.545198	0.02358751	0.212222
230582	Cyb5rl	cytochrome b5 reductase-like, transcript variant X17	NA	0.412481	2.040214	1.330973	0.02360977	0.212222
14567	Gdi1	guanosine diphosphate (GDP) dissociation inhibitor 1	NA	-0.10838	9.427173	-1.07801	0.02363357	0.212222
319508	Syt15	synaptotagmin XV, transcript variant a	NA	0.60134	0.7079	1.517125	0.0236352	0.212222
72825	Mon1a	MON1 homolog A, secretory traffciking associated	NA	-0.23643	3.794251	-1.17808	0.02363811	0.212222
19274	Ptprm	protein tyrosine phosphatase, receptor type, M, trans	NA	0.175016	4.998504	1.128977	0.0236407	0.212222
320064	D130017N08Rik	RIKEN cDNA D130017N08 gene	NA	-0.36896	2.937682	-1.29142	0.02364547	0.212222
320685	Dctd	dCMP deaminase, transcript variant 3	NA	-0.24753	4.128677	-1.18718	0.02369005	0.212488
15312	Hmgn1	high mobility group nucleosomal binding domain 1	NA	-0.12069	8.292152	-1.08726	0.02370119	0.212488
13860	Eps8	epidermal growth factor receptor pathway substrate 8	NA	0.194902	4.291663	1.144647	0.02372089	0.212547
277939	C2cd3	C2 calcium-dependent domain containing 3, transcrip	NA	-0.2187	5.498793	-1.16369	0.02387129	0.213777
17885	Myh8	myosin, heavy polypeptide 8, skeletal muscle, perinat	NA	-0.66438	3.411339	-1.58489	0.02389033	0.21383
20610	Sumo3	small ubiquitin-like modifier 3, transcript variant 1	NA	0.12248	7.346181	1.088605	0.02391385	0.213923
224079	Atp13a4	ATPase type 13A4, transcript variant X16	NA	0.630031	0.982436	1.547598	0.02392992	0.213949
110962	Mbd6	methyl-CpG binding domain protein 6, transcript varia	NA	-0.12835	6.624549	-1.09304	0.02396873	0.214179
54725	Cadm1	cell adhesion molecule 1, transcript variant 2	NA	0.117039	8.224524	1.084507	0.02400144	0.214308
54216	Pcdh7	protocadherin 7, transcript variant 1	NA	0.199349	6.334444	1.14818	0.02400955	0.214308
110446	Acat1	acetyl-Coenzyme A acetyltransferase 1	NA	0.114504	7.530218	1.082603	0.02406035	0.214644
107272	Psat1	phosphoserine aminotransferase 1, transcript variant	NA	-0.14755	6.759185	-1.10769	0.02409024	0.214793
223970	Rmi2	RecQ mediated genome instability 2	NA	-0.5048	1.521916	-1.41893	0.02410812	0.214834
20020	Polr2a	polymerase (RNA) II (DNA directed) polypeptide A	NA	-0.12203	7.378129	-1.08827	0.02413896	0.214933
74114	Crot	carnitine O-octanoyltransferase	NA	0.200887	4.267392	1.149405	0.02414954	0.214933
11828	Aqp3	aquaporin 3	NA	-0.78151	0.207186	-1.71892	0.02415881	0.214933
12398	Cbfa2t3	CBFA2/RUNX1 translocation partner 3, transcript var	NA	-0.1505	6.053185	-1.10995	0.02418012	0.215005
16975	Lrp8	low density lipoprotein receptor-related protein 8, apc	NA	0.124712	8.126597	1.09029	0.02421479	0.215196
105782	Scrib	scribbled planar cell polarity, transcript variant X11	NA	-0.12605	6.6036	-1.0913	0.02426959	0.215565
12835	Col6a3	collagen, type VI, alpha 3, transcript variant X4	NA	-0.16492	5.546968	-1.1211	0.02430002	0.215614
13845	Ephb3	Eph receptor B3	NA	-0.21539	4.56399	-1.16102	0.02430167	0.215614

27407	Abcf2	ATP-binding cassette, sub-family F (GCN20), membe	NA	-0.14104	6.258172	-1.1027	0.02433507	0.215793
14758	Gpm6b	glycoprotein m6b, transcript variant 5	NA	0.112576	8.625501	1.081157	0.02438705	0.215924
320840	Negr1	neuronal growth regulator 1, transcript variant 1	NA	0.205934	7.467477	1.153433	0.02439767	0.215924
320405	Cadps2	Ca2+-dependent activator protein for secretion 2, trar	NA	0.237882	4.038502	1.179261	0.02439875	0.215924
14628	Ostm1	osteopetrosis associated transmembrane protein 1	NA	0.181042	5.125316	1.133702	0.02440296	0.215924
20336	Exoc4	exocyst complex component 4, transcript variant 1	NA	-0.13451	6.513359	-1.09772	0.0244167	0.215929
16599	Klf3	Kruppel-like factor 3 (basic), transcript variant 1	NA	0.161679	5.181549	1.118588	0.02444353	0.21594
71609	Tradd	TNFRSF1A-associated via death domain	NA	-0.43925	1.446606	-1.3559	0.02445483	0.21594
14133	Fcna	ficolin A, transcript variant X1	NA	0.823221	0.528184	1.769352	0.02446417	0.21594
102639169	Gm20387	predicted gene 20387, transcript variant X2	NA	0.528444	1.629055	1.442373	0.02447113	0.21594
77411	Esrp2	epithelial splicing regulatory protein 2, transcript varia	NA	-1.12925	-0.13358	-2.18745	0.02450747	0.216006
72357	2210016L21Rik	RIKEN cDNA 2210016L21 gene	NA	0.164531	5.74533	1.120801	0.02451097	0.216006
20969	Sdc1	syndecan 1	NA	-0.22354	4.591144	-1.1676	0.02453033	0.216006
71711	Mus81	MUS81 structure-specific endonuclease subunit, tran	NA	-0.20536	4.50911	-1.15297	0.02453166	0.216006
109880	Braf	Braf transforming gene	NA	0.134734	6.390096	1.097891	0.02456731	0.21602
14827	Pdia3	protein disulfide isomerase associated 3	NA	-0.10834	8.258872	-1.07799	0.02457775	0.21602
70433	Draxin	dorsal inhibitory axon guidance protein	NA	-0.20407	7.305392	-1.15194	0.02458625	0.21602
329559	Zfp335	zinc finger protein 335, transcript variant X2	NA	-0.16893	5.496005	-1.12423	0.02458636	0.21602
11421	Ace	angiotensin I converting enzyme (peptidyl-dipeptidas	NA	0.183013	4.393446	1.135252	0.02461495	0.216021
66124	Josd2	Josephin domain containing 2, transcript variant 7	NA	-0.33194	3.028685	-1.25871	0.02464107	0.216021
228829	Phf20	PHD finger protein 20	NA	0.133726	6.665521	1.097124	0.02464935	0.216021
214922	Slc39a2	solute carrier family 39 (zinc transporter), member 2,	NA	-0.23389	3.764123	-1.176	0.02465284	0.216021
30056	Timm9	translocase of inner mitochondrial membrane 9, trans	NA	0.167135	4.700465	1.122826	0.0246578	0.216021
16594	Klc2	kinesin light chain 2, transcript variant 3	NA	-0.13678	6.640086	-1.09945	0.02467589	0.216021
242509	Bnc2	basonuclin 2, transcript variant 1	NA	0.244121	4.477527	1.184371	0.02467943	0.216021
21976	Top3b	topoisomerase (DNA) III beta, transcript variant X6	NA	-0.16594	5.415193	-1.1219	0.02469815	0.216041
235380	Dmxl2	Dmx-like 2, transcript variant X2	NA	0.134754	6.86966	1.097906	0.02470822	0.216041
269774	Aak1	AP2 associated kinase 1, transcript variant X29	NA	0.131639	7.011521	1.095538	0.02473066	0.216121
16012	Igfbp6	insulin-like growth factor binding protein 6	NA	0.753991	0.037087	1.686452	0.02480129	0.216622
53626	Insm1	insulinoma-associated 1	NA	-0.21768	6.482241	-1.16286	0.02487035	0.217109
12385	Ctnna1	catenin (cadherin associated protein), alpha 1	NA	-0.12555	6.455163	-1.09093	0.02491698	0.217156
381822	Lockd	lncRNA downstream of Cdkn1b, transcript variant 2	NA	-0.45305	1.917196	-1.36893	0.02492346	0.217156
67122	Nrarp	Notch-regulated ankyrin repeat protein	NA	-0.17397	5.518394	-1.12816	0.02493749	0.217156
76217	Jakmip2	janus kinase and microtubule interacting protein 2	NA	0.140411	7.030689	1.102219	0.02493919	0.217156
74006	Dnm1l	dynamin 1-like, transcript variant X16	NA	0.120792	7.636474	1.087332	0.0249425	0.217156
319616	Fzd10os	frizzled class receptor 10, opposite strand	NA	-0.56563	0.874961	-1.48004	0.02498299	0.21719
212999	Tnpo2	transportin 2 (importin 3, karyopherin beta 2b), transc	NA	-0.13744	7.505652	-1.09995	0.02498531	0.21719
68916	Cdkal1	CDK5 regulatory subunit associated protein 1-like 1, t	NA	-0.2196	4.001643	-1.16441	0.02498644	0.21719
74498	Gorasp1	golgi reassembly stacking protein 1	NA	-0.1862	4.452965	-1.13776	0.02511302	0.218147
381314	Iars2	isoleucine-tRNA synthetase 2, mitochondrial	NA	0.150439	5.354266	1.109907	0.02513298	0.218147
11695	Alx4	aristaless-like homeobox 4	NA	-0.36837	2.895831	-1.2909	0.02514443	0.218147
93734	Mpv17l	Mpv17 transgene, kidney disease mutant-like, transcı	NA	0.247896	4.645128	1.187474	0.02515908	0.218147
26409	Map3k7	mitogen-activated protein kinase kinase kinase 7, trai	NA	0.131591	6.793724	1.095501	0.02516362	0.218147
56228	Ube2j1	ubiquitin-conjugating enzyme E2J 1, transcript varian	NA	0.128759	6.565228	1.093353	0.02517731	0.21815
77794	Adamtsl2	ADAMTS-like 2	NA	-0.3386	2.709798	-1.26453	0.02521752	0.218382
71141	4933407L21Rik	RIKEN cDNA 4933407L21 gene	NA	0.457668	1.491638	1.37332	0.02526264	0.218407
104806	Fancm	Fanconi anemia, complementation group M, transcrip	NA	0.237572	4.100456	1.179007	0.02526432	0.218407
623279	Dok6	docking protein 6	NA	0.173096	5.824403	1.127476	0.02527609	0.218407
67010	Rbm7	RNA binding motif protein 7, transcript variant 1	NA	0.159206	5.439626	1.116672	0.02528209	0.218407
67181	Ctdnep1	CTD nuclear envelope phosphatase 1	NA	-0.12386	6.220478	-1.08964	0.02529497	0.218407
71448	Tmem80	transmembrane protein 80, transcript variant X2	NA	-0.22318	4.210735	-1.16731	0.02530099	0.218407
70312	Cactin	cactin, spliceosome C complex subunit	NA	-0.2094	5.033799	-1.15621	0.02532109	0.218465
50765	Tfr2	transferrin receptor 2, transcript variant 1	NA	0.584138	1.894953	1.499143	0.02536258	0.218602
73845	Ankrd42	ankyrin repeat domain 42	NA	-0.24453	3.83748	-1.18471	0.02538243	0.218602
69698	Slc52a3	solute carrier protein family 52, member 3, transcript	NA	0.65256	0.572011	1.571955	0.02541923	0.218602
17135	Mafk	v-maf musculoaponeurotic fibrosarcoma oncogene fa	NA	-0.22834	4.159654	-1.17149	0.02543325	0.218602
23955	Nek4	NIMA (never in mitosis gene a)-related expressed kin	NA	-0.17927	4.546157	-1.13231	0.02544289	0.218602
57741	Noc2l	NOC2 like nucleolar associated transcriptional repres	NA	-0.11606	6.527026	-1.08377	0.02544595	0.218602
110796	Tshz1	teashirt zinc finger family member 1, transcript varian	NA	0.161751	6.124582	1.118644	0.0254482	0.218602
54418	Fmn2	formin 2	NA	0.15318	5.541613	1.112018	0.02547585	0.218602
20617	Snca	synuclein, alpha, transcript variant 2	NA	0.149755	6.452635	1.109381	0.02548258	0.218602
76089	Rapgef2	Rap guanine nucleotide exchange factor (GEF) 2, tra	NA	0.140419	6.203041	1.102225	0.02549183	0.218602
110460	Acat2	acetyl-Coenzyme A acetyltransferase 2	NA	0.106529	7.669099	1.076635	0.02549541	0.218602
269615	Plch2	phospholipase C, eta 2, transcript variant X11	NA	-0.16844	5.244662	-1.12385	0.02549822	0.218602
69908	Rab3b	RAB3B, member RAS oncogene family	NA	0.152169	5.402937	1.111239	0.0255603	0.218998
665001	Gm14391	predicted gene 14391, transcript variant X1	NA	0.235762	3.578147	1.177529	0.02557128	0.218998
118567396	LOC118567396	uncharacterized LOC118567396	NA	-0.95027	-0.0838	-1.93224	0.02559149	0.219056
209707	Lcorl	ligand dependent nuclear receptor corepressor-like, t	NA	0.171514	6.092242	1.126239	0.0256453	0.219401

613258	A730017L22Rik	RIKEN cDNA A730017L22 gene	NA	-0.3136	3.010377	-1.2428	0.02569548	0.219603
327762	Dna2	DNA replication helicase/nuclease 2, transcript variar	NA	-0.32312	3.372495	-1.25103	0.02569593	0.219603
12140	Fabp7	fatty acid binding protein 7, brain	NA	0.122275	9.220276	1.08845	0.02577803	0.219986
20191	Ryr2	ryanodine receptor 2, cardiac, transcript variant X20	NA	0.21538	4.903297	1.16101	0.025785	0.219986
229877	Rap1gds1	RAP1, GTP-GDP dissociation stimulator 1, transcript	NA	0.123499	6.838515	1.089373	0.02581701	0.219986
72039	Mccc1	methylcrotonoyl-Coenzyme A carboxylase 1 (alpha),	NA	-0.17096	4.705968	-1.12581	0.02582199	0.219986
14081	Acsl1	acyl-CoA synthetase long-chain family member 1, tra	NA	0.160774	4.808422	1.117887	0.02582849	0.219986
76795	Tbc1d9b	TBC1 domain family, member 9B, transcript variant X	NA	-0.13251	6.250574	-1.0962	0.02582855	0.219986
72961	Slc17a7	solute carrier family 17 (sodium-dependent inorganic	NA	-0.24788	4.805421	-1.18746	0.02583532	0.219986
74100	Arpp21	cyclic AMP-regulated phosphoprotein, 21, transcript v	NA	0.168769	5.996111	1.124099	0.02586227	0.220084
102633763	Gm31513	predicted gene, 31513, transcript variant X1	NA	0.430568	1.767929	1.347764	0.0258739	0.220084
18295	Ogn	osteoglycin	NA	0.20191	4.53417	1.15022	0.02597943	0.22079
17454	Mov10	Mov10 RISC complex RNA helicase, transcript variar	NA	-0.30796	3.60746	-1.23796	0.02598405	0.22079
67516	Kctd4	potassium channel tetramerisation domain containing	NA	0.246346	3.63942	1.186199	0.02600135	0.220821
19734	Rgs16	regulator of G-protein signaling 16	NA	-0.18054	4.521084	-1.13331	0.02604775	0.2211
57438	Marchf7	membrane associated ring-CH-type finger 7, transcrip	NA	0.133208	6.560871	1.09673	0.02610726	0.22149
57267	Apba3	amyloid beta (A4) precursor protein-binding, family A	NA	-0.22379	3.714442	-1.1678	0.02612917	0.22156
72023	Cyb561d1	cytochrome b-561 domain containing 1, transcript var	NA	0.213792	3.856222	1.159733	0.0261556	0.221666
74374	Clec16a	C-type lectin domain family 16, member A, transcript	NA	-0.12019	6.565712	-1.08688	0.02617834	0.221666
66098	Chchd6	coiled-coil-helix-coiled-coil-helix domain containing 6,	NA	0.154439	5.132111	1.112989	0.02619007	0.221666
59022	Edf1	endothelial differentiation-related factor 1	NA	-0.14985	5.896037	-1.10945	0.02621517	0.221666
234915	Cep126	centrosomal protein 126, transcript variant X5	NA	0.249001	4.03195	1.188384	0.02622104	0.221666
20817	Srpk2	serine/arginine-rich protein specific kinase 2, transcri	NA	0.111712	7.548515	1.080509	0.0262236	0.221666
93761	Smarca1	SWI/SNF related, matrix associated, actin dependent	NA	0.179456	5.854739	1.132457	0.02623706	0.221666
19944	Rpl29	ribosomal protein L29, transcript variant 1	NA	0.108361	8.209386	1.078003	0.02629123	0.222009
11303	Abca1	ATP-binding cassette, sub-family A (ABC1), member	NA	0.144914	6.035436	1.105665	0.02632276	0.222086
244579	Tox3	TOX high mobility group box family member 3, transc	NA	0.124786	6.304737	1.090346	0.02634742	0.222086
93696	Chrac1	chromatin accessibility complex 1	NA	0.241658	3.568939	1.182351	0.02635031	0.222086
14030	Ewsr1	Ewing sarcoma breakpoint region 1, transcript varian	NA	-0.11679	8.63633	-1.08432	0.02635494	0.222086
64658	Mrps25	mitochondrial ribosomal protein S25	NA	-0.23089	4.523451	-1.17356	0.02638464	0.222221
66953	Cdca7	cell division cycle associated 7	NA	-0.25128	5.472485	-1.19026	0.02640259	0.222257
20614	Snap25	synaptosomal-associated protein 25, transcript variar	NA	0.109814	7.728229	1.079089	0.02646941	0.222704
67465	Sf3a1	splicing factor 3a, subunit 1	NA	-0.15087	7.097331	-1.11024	0.02650166	0.222817
104112	Acly	ATP citrate lyase, transcript variant 2	NA	-0.10408	8.282319	-1.07481	0.02651023	0.222817
21422	Tfcp2	transcription factor CP2, transcript variant X5	NA	-0.17216	5.254729	-1.12675	0.02659096	0.22307
22341	Vegfc	vascular endothelial growth factor C	NA	0.373144	2.456404	1.295172	0.0265969	0.22307
73940	Hapln2	hyaluronan and proteoglycan link protein 2, transcript	NA	0.808236	-0.29235	1.751069	0.02659724	0.22307
26383	Fto	fat mass and obesity associated	NA	-0.12988	7.75152	-1.09421	0.02661922	0.22307
11847	Arg2	arginase type II	NA	0.244419	3.381848	1.184615	0.02662347	0.22307
68082	Dusp19	dual specificity phosphatase 19	NA	-0.31171	2.857622	-1.24118	0.02664565	0.22307
23984	Pde10a	phosphodiesterase 10A, transcript variant 3	NA	0.155579	6.191023	1.113869	0.02667366	0.22307
28006	Washc2	WASH complex subunit 2`	NA	0.133652	6.308677	1.097067	0.02668666	0.22307
433323	Sgpp2	sphingosine-1-phosphate phosphatase 2	NA	0.554752	0.932236	1.468916	0.02669985	0.22307
72065	Rap2c	RAP2C, member of RAS oncogene family	NA	0.138933	6.1393	1.101091	0.02671066	0.22307
102644	Oaf	out at first homolog	NA	-0.3868	2.462246	-1.30749	0.02671327	0.22307
117981786	Gm9884	predicted gene 9884	NA	0.731136	-0.00609	1.659946	0.02671717	0.22307
69745	Pold4	polymerase (DNA-directed), delta 4, transcript varian	NA	0.550578	2.262837	1.464673	0.02671855	0.22307
67569	Mgat4c	MGAT4 family, member C, transcript variant 1	NA	0.296995	3.86024	1.228583	0.02680365	0.223666
19291	Purb	purine rich element binding protein B	NA	0.15674	6.617663	1.114766	0.02681967	0.223685
102635315	Gm32687	predicted gene, 32687, transcript variant 1	NA	0.337343	3.38769	1.263428	0.02690452	0.224278
242109	Zfp697	zinc finger protein 697	NA	0.133182	5.874445	1.09671	0.02694194	0.224452
57376	Smarce1	SWI/SNF related, matrix associated, actin dependent	NA	-0.10726	8.200073	-1.07718	0.02695299	0.224452
21877	Tk1	thymidine kinase 1, transcript variant 1	NA	-0.25661	3.665337	-1.19467	0.0269718	0.224494
433408	Gm13375	predicted gene 13375	NA	0.274901	3.637547	1.209911	0.0269874	0.224509
66902	Mtap	methylthioadenosine phosphorylase	NA	-0.17914	4.619932	-1.13221	0.02702548	0.224566
381549	Zfp69	zinc finger protein 69, transcript variant 1	NA	0.372184	2.161011	1.29431	0.02703836	0.224566
243548	Prickle2	prickle planar cell polarity protein 2, transcript variant	NA	0.152093	5.596868	1.111181	0.02704633	0.224566
17686	Msh3	mutS homolog 3, transcript variant X2	NA	0.196477	4.945109	1.145897	0.02704953	0.224566
232089	Elmod3	ELMO/CED-12 domain containing 3, transcript varian	NA	-0.20975	3.921528	-1.15649	0.02707853	0.224692
73738	Haus7	HAUS augmin-like complex, subunit 7, transcript vari	NA	0.182324	4.635072	1.13471	0.02715003	0.225067
26429	Orc5	origin recognition complex, subunit 5	NA	0.190256	4.743391	1.140966	0.02715129	0.225067
21687	Tek	TEK receptor tyrosine kinase, transcript variant 3	NA	0.198651	4.134021	1.147625	0.02723689	0.225661
20334	Sec23a	SEC23 homolog A, COPII coat complex component,	NA	0.137703	7.199747	1.100152	0.02726338	0.225766
22317	Vamp1	vesicle-associated membrane protein 1, transcript va	NA	0.219283	4.300288	1.164155	0.02729569	0.22588
208628	Kntc1	kinetochore associated 1, transcript variant X6	NA	-0.30619	3.374207	-1.23644	0.02730493	0.22588
67432	Hoga1	4-hydroxy-2-oxoglutarate aldolase 1	NA	0.830525	0.600255	1.778333	0.02738512	0.226331
71233	Enkur	enkurin, TRPC channel interacting protein	NA	0.365818	2.199172	1.288612	0.02738727	0.226331
276952	Rasl10b	RAS-like, family 10, member B, transcript variant 4	NA	-0.14093	6.609438	-1.10262	0.02741008	0.226405

72736	Tmx1	thioredoxin-related transmembrane protein 1	NA	0.115208	6.614014	1.083131	0.02745926	0.226696
210808	Lacc1	laccase domain containing 1, transcript variant X3	NA	0.454975	1.716481	1.370759	0.02748987	0.226713
12825	Col3a1	collagen, type III, alpha 1	NA	-0.19552	8.19365	-1.14513	0.02754581	0.226713
100763	Ube3c	ubiquitin protein ligase E3C	NA	0.127965	6.506968	1.092751	0.02754829	0.226713
236285	Lancl3	LanC lantibiotic synthetase component C-like 3 (bact	NA	0.464655	2.110807	1.379987	0.02755775	0.226713
231225	Tapt1	transmembrane anterior posterior transformation 1	NA	0.173246	5.614963	1.127593	0.02756202	0.226713
226791	Lyplal1	lysophospholipase-like 1	NA	-0.2977	2.883555	-1.22918	0.02757871	0.226713
80286	Tusc3	tumor suppressor candidate 3, transcript variant X8	NA	0.146515	7.50763	1.106892	0.02758814	0.226713
22594	Xrcc1	X-ray repair complementing defective repair in Chine	NA	-0.17574	5.3764	-1.12955	0.02758836	0.226713
236790	Ints6l	integrator complex subunit 6 like, transcript variant X	NA	0.17222	5.604301	1.126791	0.02760008	0.226713
26390	Mapkbp1	mitogen-activated protein kinase binding protein 1, tr	NA	0.123234	6.218752	1.089174	0.02760064	0.226713
102635429	Gm20655	predicted gene 20655, transcript variant X3	NA	0.72359	0.012436	1.651286	0.02761974	0.226755
18641	Pfkl	phosphofructokinase, liver, B-type, transcript variant	NA	-0.13397	6.762342	-1.09731	0.02764249	0.226827
30052	Pcsk1n	proprotein convertase subtilisin/kexin type 1 inhibitor	NA	0.197612	5.939038	1.146798	0.02766858	0.226927
16774	Lama3	laminin, alpha 3, transcript variant X6	NA	-0.36098	2.077679	-1.2843	0.02768671	0.226958
23965	Tenm3	teneurin transmembrane protein 3, transcript variant	NA	0.159354	7.447562	1.116787	0.02770029	0.226958
235281	Scn3b	sodium channel, voltage-gated, type III, beta, transcri	NA	0.121322	7.820358	1.087731	0.02775318	0.227252
74143	Opa1	OPA1, mitochondrial dynamin like GTPase, transcript	NA	0.144614	6.948332	1.105435	0.02776403	0.227252
71684	Rbm43	RNA binding motif protein 43, transcript variant 6	NA	0.314992	2.669421	1.244005	0.02780201	0.227448
16647	Kpna2	karyopherin (importin) alpha 2	NA	-0.16058	6.848109	-1.11774	0.0278382	0.22763
12566	Cdk2	cyclin-dependent kinase 2, transcript variant 2	NA	-0.18482	4.41053	-1.13667	0.02787388	0.227708
17260	Mef2c	myocyte enhancer factor 2C, transcript variant 10	NA	0.156524	7.406073	1.114598	0.02787574	0.227708
12369	Casp7	caspase 7	NA	0.233078	4.149088	1.175339	0.02796458	0.227928
14555	Gpd1	glycerol-3-phosphate dehydrogenase 1 (soluble)	NA	-0.44853	2.995762	-1.36465	0.02797047	0.227928
242819	Rundc3b	RUN domain containing 3B, transcript variant 2	NA	0.152797	6.129822	1.111722	0.02797927	0.227928
12661	Chl1	cell adhesion molecule L1-like, transcript variant X14	NA	0.127563	8.756878	1.092446	0.02800838	0.227928
100042235	Gm3739	predicted gene 3739	NA	0.279297	3.573485	1.213603	0.02800846	0.227928
102638362	Gm34945	predicted gene, 34945	NA	-0.93451	0.241338	-1.91125	0.02800871	0.227928
77634	Snapc3	small nuclear RNA activating complex, polypeptide 3,	NA	-0.14956	6.624156	-1.10923	0.02802506	0.227928
14594	Ggta1	glycoprotein galactosyltransferase alpha 1, 3, transcr	NA	-0.22271	3.667707	-1.16692	0.02804666	0.227928
67623	Tm7sf3	transmembrane 7 superfamily member 3, transcript v	NA	0.18853	5.088091	1.139602	0.02804757	0.227928
381629	Atraid	all-trans retinoic acid induced differentiation factor, tr	NA	0.189587	5.259439	1.140437	0.02806122	0.227928
207920	Esrp1	epithelial splicing regulatory protein 1, transcript varia	NA	-0.60672	0.653948	-1.52279	0.02806444	0.227928
211389	Suox	sulfite oxidase	NA	-0.22059	3.628924	-1.16521	0.02808338	0.227928
67983	Pdzd9	PDZ domain containing 9, transcript variant X3	NA	0.301453	2.896623	1.232385	0.02811047	0.227928
72287	Plekhf1	pleckstrin homology domain containing, family F (with	NA	1.047803	-0.50247	2.067379	0.0281185	0.227928
70152	Mettl7a1	methyltransferase like 7A1	NA	-0.26704	3.198594	-1.20334	0.02811948	0.227928
20779	Src	Rous sarcoma oncogene, transcript variant X10	NA	-0.11985	7.152356	-1.08662	0.02814322	0.227928
624086	A230045G11Rik	RIKEN cDNA A230045G11 gene	NA	-0.27586	2.961617	-1.21072	0.02815244	0.227928
319801	Tigar	Trp53 induced glycolysis regulatory phosphatase	NA	-0.19416	4.874182	-1.14406	0.0281548	0.227928
228026	Pdk1	pyruvate dehydrogenase kinase, isoenzyme 1, transc	NA	0.242237	5.614769	1.182825	0.02819306	0.228124
27103	Eif2ak4	eukaryotic translation initiation factor 2 alpha kinase	NA	0.177044	4.61317	1.130565	0.02822939	0.228242
73724	Mcee	methylmalonyl CoA epimerase, transcript variant 1	NA	-0.2725	3.097981	-1.2079	0.02823564	0.228242
67893	Tmem86a	transmembrane protein 86A	NA	0.246161	3.499853	1.186047	0.02825419	0.228278
71675	0610010F05Rik	RIKEN cDNA 0610010F05 gene, transcript variant X	NA	0.153453	6.993834	1.112229	0.02828267	0.228395
71599	Senp8	SUMO/sentrin specific peptidase 8, transcript variant	NA	-0.26534	3.335372	-1.20192	0.02831406	0.228535
114713	Rasa2	RAS p21 protein activator 2	NA	0.169177	5.07473	1.124417	0.0283701	0.228773
229841	Cenpe	centromere protein E	NA	-0.20783	4.563984	-1.15495	0.02837162	0.228773
79221	Hdac9	histone deacetylase 9, transcript variant X35	NA	0.210236	4.431815	1.156878	0.02840149	0.228813
18708	Pik3r1	phosphoinositide-3-kinase regulatory subunit 1, trans	NA	0.135703	7.240572	1.098628	0.02842049	0.228813
66615	Atg4b	autophagy related 4B, cysteine peptidase, transcript	NA	-0.13564	6.131893	-1.09858	0.02842063	0.228813
24055	Sh3bp2	SH3-domain binding protein 2, transcript variant X1	NA	-0.14414	5.651488	-1.10507	0.02843289	0.228813
73040	2900052N01Rik	RIKEN cDNA 2900052N01 gene	NA	0.651366	0.154941	1.570655	0.02845399	0.22887
11737	Anp32a	acidic (leucine-rich) nuclear phosphoprotein 32 family	NA	-0.15353	7.988538	-1.11229	0.02852597	0.229225
67967	Pold3	polymerase (DNA-directed), delta 3, accessory subur	NA	-0.14103	5.784699	-1.10269	0.02852856	0.229225
67043	Syap1	synapse associated protein 1	NA	0.151234	5.50597	1.110519	0.02854037	0.229225
21956	Tnnt2	troponin T2, cardiac, transcript variant 9	NA	-0.7077	0.895653	-1.63319	0.02856087	0.229226
237611	Stac3	SH3 and cysteine rich domain 3, transcript variant 2	NA	-0.38997	2.096019	-1.31037	0.02859315	0.229226
231123	Haus3	HAUS augmin-like complex, subunit 3	NA	-0.24198	3.595407	-1.18261	0.02859358	0.229226
56273	Pex14	peroxisomal biogenesis factor 14	NA	0.175716	4.954222	1.129525	0.02859687	0.229226
76547	Tmem101	transmembrane protein 101	NA	-0.17353	4.543704	-1.12781	0.02864356	0.229352
71897	Lypd6b	LY6/PLAUR domain containing 6B, transcript variant	NA	0.237202	3.788959	1.178705	0.02864634	0.229352
50795	Sh3bgr	SH3-binding domain glutamic acid-rich protein	NA	0.652303	0.935092	1.571675	0.02865927	0.229352
14319	Fth1	ferritin heavy polypeptide 1, transcript variant 1	NA	0.11736	8.651266	1.084748	0.02867523	0.229352
66086	Cep20	centrosomal protein 20	NA	0.148506	5.823888	1.108421	0.02868618	0.229352
55947	Dclre1a	DNA cross-link repair 1A, transcript variant 4	NA	0.227084	3.492706	1.170467	0.02869932	0.229352
99031	Osbpl6	oxysterol binding protein-like 6, transcript variant X14	NA	0.160829	5.553117	1.11793	0.02872605	0.229352
319915	A830049F12Rik	RIKEN cDNA A830049F12 gene	NA	0.790962	-0.17519	1.730228	0.02874788	0.229352

102632229	Gm30364	predicted gene, 30364, transcript variant X4	NA	0.753128	0.001335	1.685443	0.02875185	0.229352
279561	Wnk3	WNK lysine deficient protein kinase 3, transcript varia	NA	0.153161	5.737061	1.112004	0.0287574	0.229352
16589	Uhmk1	U2AF homology motif (UHM) kinase 1, transcript vari	NA	0.14415	6.117067	1.10508	0.02876763	0.229352
211429	Pla2g4b	phospholipase A2, group IVB (cytosolic)	NA	-0.42911	4.079794	-1.34641	0.02879616	0.229467
70354	Secisbp2l	SECIS binding protein 2-like	NA	0.139392	6.558159	1.101441	0.02883484	0.229605
213436	Rtl3	retrotransposon Gag like 3	NA	-0.44006	1.71007	-1.35666	0.02885228	0.229605
100042679	Gm16386	predicted gene 16386	NA	0.455843	2.948579	1.371584	0.02886068	0.229605
71704	Arhgef3	Rho guanine nucleotide exchange factor (GEF) 3, tra	NA	0.239096	3.44971	1.180253	0.02888299	0.229605
22776	Zim1	zinc finger, imprinted 1, transcript variant X3	NA	0.367155	3.310352	1.289807	0.02888404	0.229605
12326	Camk4	calcium/calmodulin-dependent protein kinase IV, tran	NA	0.16098	5.947777	1.118046	0.02891527	0.229741
17184	Matr3	matrin 3	NA	0.102465	9.390539	1.073607	0.02894286	0.229772
225289	AW554918	expressed sequence AW554918	NA	0.185573	4.684253	1.137269	0.02894738	0.229772
108147	Atic	5-aminoimidazole-4-carboxamide ribonucleotide form	NA	-0.13749	5.851262	-1.09999	0.02896653	0.229812
237213	Glra2	glycine receptor, alpha 2 subunit, transcript variant 1	NA	0.121913	6.406473	1.088176	0.02902218	0.230141
12373	Casq2	calsequestrin 2, transcript variant 2	NA	-0.73972	1.382932	-1.66985	0.02907992	0.230486
17698	Msn	moesin	NA	-0.12347	5.998662	-1.08935	0.02910721	0.230486
18844	Plxna1	plexin A1	NA	-0.10989	7.98167	-1.07915	0.02914948	0.230486
54383	Phc2	polyhomeotic 2, transcript variant X8	NA	-0.12082	7.240415	-1.08735	0.02915132	0.230486
102633301	Gm31160	predicted gene, 31160, transcript variant X1	NA	0.725874	0.163299	1.653902	0.02916862	0.230486
210925	Ints9	integrator complex subunit 9, transcript variant 1	NA	-0.17138	4.753693	-1.12613	0.02918339	0.230486
22422	Wnt7b	wingless-type MMTV integration site family, member	NA	-0.19589	6.49703	-1.14543	0.02920908	0.230486
67731	Fbxo32	F-box protein 32	NA	0.23791	3.724381	1.179283	0.02921263	0.230486
59044	Rnf130	ring finger protein 130, transcript variant 3	NA	0.144488	7.926097	1.105338	0.02921695	0.230486
15926	Idh1	isocitrate dehydrogenase 1 (NADP+), soluble, transc	NA	0.099149	8.640199	1.071141	0.02922422	0.230486
278304	Zfp385c	zinc finger protein 385C, transcript variant 5	NA	0.736824	-0.00467	1.666503	0.02923142	0.230486
11984	Atp6v0c	ATPase, H+ transporting, lysosomal V0 subunit C, tra	NA	0.122904	8.485402	1.088925	0.02923571	0.230486
170822	Usp33	ubiquitin specific peptidase 33, transcript variant X1	NA	0.112375	6.977082	1.081006	0.02925652	0.230539
78785	Clip4	CAP-GLY domain containing linker protein family, me	NA	0.178172	4.63596	1.13145	0.02930745	0.230699
329003	Zfp516	zinc finger protein 516, transcript variant X11	NA	-0.1793	4.866041	-1.13233	0.02931479	0.230699
21925	Tnnc2	troponin C2, fast	NA	-0.87358	1.0727	-1.8322	0.02932637	0.230699
67304	3110070M22Rik	RIKEN cDNA 3110070M22 gene	NA	0.81539	-0.14753	1.759773	0.02933361	0.230699
328035	Fads6	fatty acid desaturase domain family, member 6	NA	-0.31505	2.734466	-1.24406	0.02938602	0.230974
213011	Zfp583	zinc finger protein 583	NA	-0.24849	3.910209	-1.18796	0.02939692	0.230974
53413	Exoc7	exocyst complex component 7, transcript variant 1	NA	-0.12082	6.346329	-1.08735	0.02941425	0.230998
271786	Galnt13	polypeptide N-acetylgalactosaminyltransferase 13, tra	NA	0.254777	3.616993	1.193151	0.02942916	0.231004
15587	Hyal2	hyaluronoglucosaminidase 2	NA	-0.15604	4.991664	-1.11423	0.0294636	0.231065
414872	Zyg11b	zyg-ll family member B, cell cycle regulator	NA	0.109709	7.817416	1.079011	0.02946529	0.231065
19942	Rpl27	ribosomal protein L27	NA	0.114658	7.840664	1.082718	0.02948926	0.231109
17257	Mecp2	methyl CpG binding protein 2, transcript variant 1	NA	0.123513	7.427601	1.089384	0.02949936	0.231109
66880	Rsrc1	arginine/serine-rich coiled-coil 1, transcript variant 2	NA	0.133722	5.662046	1.09712	0.02951624	0.23113
76897	Ralyl	RALY RNA binding protein-like, transcript variant X13	NA	0.12708	5.968004	1.092081	0.02954749	0.231263
100102	Pcsk9	proprotein convertase subtilisin/kexin type 9	NA	0.37998	1.786009	1.301324	0.0296255	0.231744
12550	Cdh1	cadherin 1	NA	-0.3625	3.221742	-1.28566	0.02964541	0.231744
18212	Ntrk2	neurotrophic tyrosine kinase, receptor, type 2, transc	NA	0.118017	7.635206	1.085242	0.0296516	0.231744
66674	Spryd7	SPRY domain containing 7, transcript variant 1	NA	0.144274	5.315594	1.105174	0.02967669	0.231761
13858	Eps15	epidermal growth factor receptor pathway substrate 1	NA	0.142646	6.837864	1.103928	0.02970009	0.231761
238130	Dock4	dedicator of cytokinesis 4, transcript variant X7	NA	0.15718	5.613618	1.115105	0.02970927	0.231761
21853	Timeless	timeless circadian clock 1, transcript variant 3	NA	-0.19679	4.504133	-1.14614	0.02971435	0.231761
11658	Alcam	activated leukocyte cell adhesion molecule, transcript	NA	0.191405	6.760512	1.141875	0.02972503	0.231761
23997	Psmd13	proteasome (prosome, macropain) 26S subunit, non-	NA	0.115137	6.751971	1.083078	0.0297434	0.231793
545391	Catspere2	cation channel sperm associated auxiliary subunit ep	NA	0.285153	3.523713	1.21854	0.02978513	0.231984
11516	Adcyap1	adenylate cyclase activating polypeptide 1, transcript	NA	0.248023	4.027037	1.187578	0.02980769	0.231984
104444	Rexo2	RNA exonuclease 2, transcript variant 1	NA	-0.19807	4.376352	-1.14716	0.02981739	0.231984
66236	1500011B03Rik	RIKEN cDNA 1500011B03 gene, transcript variant 1	NA	0.154826	5.907665	1.113288	0.02982819	0.231984
56215	Acin1	apoptotic chromatin condensation inducer 1, transcrip	NA	-0.1245	8.204108	-1.09013	0.02983917	0.231984
64136	Sdf2l1	stromal cell-derived factor 2-like 1	NA	-0.3084	3.013733	-1.23833	0.02989247	0.232288
58909	Fam13a	family with sequence similarity 13, member A	NA	0.226195	3.806373	1.169746	0.02991378	0.232342
12662	Chm	choroidermia (RAB escort protein 1), transcript variar	NA	0.18435	4.945789	1.136305	0.02997559	0.232711
12927	Bcar1	breast cancer anti-estrogen resistance 1, transcript va	NA	-0.14473	6.184829	-1.10553	0.03007468	0.233262
20843	Stag2	stromal antigen 2, transcript variant X10	NA	0.147438	6.498873	1.107601	0.03012375	0.233262
20174	Ruvbl2	RuvB-like protein 2	NA	-0.12221	6.144945	-1.0884	0.03012861	0.233262
224171	Cip2a	cell proliferation regulating inhibitor of protein phosph	NA	-0.26131	4.364974	-1.19856	0.03013179	0.233262
110532	Adarb1	adenosine deaminase, RNA-specific, B1, transcript va	NA	0.137387	5.334487	1.099911	0.03015471	0.233262
380660	Acss3	acyl-CoA synthetase short-chain family member 3, tra	NA	0.437675	1.381587	1.35442	0.03016523	0.233262
13730	Emp1	epithelial membrane protein 1, transcript variant 1	NA	-0.2148	5.054291	-1.16054	0.03016621	0.233262
70348	Ube2cbp	ubiquitin-conjugating enzyme E2C binding protein	NA	0.345172	2.116658	1.270302	0.03016797	0.233262
22253	Unc5c	unc-5 netrin receptor C, transcript variant 2	NA	0.214046	5.288007	1.159937	0.03017729	0.233262
268445	Ankrd13b	ankyrin repeat domain 13b, transcript variant X15	NA	-0.10366	7.68326	-1.0745	0.03021395	0.233262

20928	Abcc9	ATP-binding cassette, sub-family C (CFTR/MRP), me	NA	0.233536	4.720524	1.175713	0.03022141	0.233262
15562	Htr4	5 hydroxytryptamine (serotonin) receptor 4, transcript	NA	0.399223	2.310419	1.318797	0.03023011	0.233262
14137	Fdft1	farnesyl diphosphate farnesyl transferase 1, transcrip	NA	0.123942	7.787882	1.089708	0.03023283	0.233262
100978	Nfxl1	nuclear transcription factor, X-box binding-like 1, tran	NA	0.167093	4.926577	1.122794	0.03026465	0.233386
26430	Parg	poly (ADP-ribose) glycohydrolase, transcript variant X	NA	0.132742	5.694253	1.096375	0.03027762	0.233386
21961	Tns1	tensin 1, transcript variant X42	NA	-0.16008	5.309566	-1.11735	0.03032877	0.233639
100502846	Gm19410	predicted gene, 19410	NA	0.695417	0.063731	1.619353	0.0303508	0.233639
19377	Rai1	retinoic acid induced 1, transcript variant X2	NA	-0.10555	7.278432	-1.0759	0.03036332	0.233639
219134	Shisa2	shisa family member 2	NA	-0.1978	4.408439	-1.14695	0.03036788	0.233639
245297	Gm4983	predicted gene 4983, transcript variant X1	NA	0.390485	2.100422	1.310834	0.03040618	0.233823
65962	Slc9a3r2	solute carrier family 9 (sodium/hydrogen exchanger),	NA	0.254238	4.710692	1.192706	0.03042327	0.233832
234094	Arhgef10	Rho guanine nucleotide exchange factor (GEF) 10, tr	NA	-0.21006	4.003584	-1.15674	0.03043974	0.233832
27428	Shroom3	shroom family member 3, transcript variant X2	NA	-0.24478	3.949672	-1.18491	0.03045242	0.233832
54384	Mtmr7	myotubularin related protein 7	NA	0.132854	5.706801	1.096461	0.03048075	0.233832
78906	Misp	mitotic spindle positioning, transcript variant X4	NA	-0.61851	0.399055	-1.53529	0.03048474	0.233832
74302	Mtmr3	myotubularin related protein 3, transcript variant X5	NA	0.125215	6.753002	1.09067	0.03051527	0.233832
71709	Syde1	synapse defective 1, Rho GTPase, homolog 1 (C. ele	NA	-0.2257	3.60249	-1.16935	0.03051906	0.233832
16828	Ldha	lactate dehydrogenase A, transcript variant 1	NA	0.182906	8.030402	1.135168	0.03052229	0.233832
51960	Kctd18	potassium channel tetramerisation domain containing	NA	0.197238	4.662411	1.146502	0.03056038	0.233861
213121	Ankrd35	ankyrin repeat domain 35, transcript variant X1	NA	-0.33653	2.402154	-1.26271	0.03056245	0.233861
237868	Sarm1	sterile alpha and HEAT/Armadillo motif containing 1,	NA	-0.21267	6.436835	-1.15883	0.0305968	0.233861
207592	Tbc1d16	TBC1 domain family, member 16, transcript variant 2	NA	-0.14428	8.353349	-1.10518	0.03060349	0.233861
108167626	Gm46093	predicted gene, 46093	NA	0.678912	0.689448	1.600932	0.03063265	0.233861
70729	Nos1ap	nitric oxide synthase 1 (neuronal) adaptor protein, tra	NA	-0.16947	4.825229	-1.12465	0.03065059	0.233861
231986	Jazf1	JAZF zinc finger 1, transcript variant X2	NA	0.145229	5.768614	1.105906	0.03065728	0.233861
63993	Slc5a7	solute carrier family 5 (choline transporter), member 7	NA	0.33578	2.482894	1.262059	0.03066131	0.233861
72157	Pgm1	phosphoglucomutase 1	NA	0.138647	5.664682	1.100872	0.03067152	0.233861
70155	Ogfrl1	opioid growth factor receptor-like 1, transcript variant	NA	0.141116	5.792488	1.102758	0.0306811	0.233861
67469	Abhd5	abhydrolase domain containing 5, transcript variant 1	NA	0.166694	4.528374	1.122483	0.03069565	0.233861
108079	Prkaa2	protein kinase, AMP-activated, alpha 2 catalytic subu	NA	0.166634	5.500583	1.122437	0.03069851	0.233861
67399	Pdlim7	PDZ and LIM domain 7, transcript variant d	NA	-0.15309	5.276422	-1.11195	0.03073141	0.2339
107449	Unc5b	unc-5 netrin receptor B, transcript variant 2	NA	-0.18238	4.443365	-1.13475	0.03073244	0.2339
26926	Aifm1	apoptosis-inducing factor, mitochondrion-associated	NA	-0.15601	4.938206	-1.1142	0.03078252	0.234109
382620	Tmed8	transmembrane p24 trafficking protein 8	NA	0.194206	5.192682	1.144094	0.03079757	0.234109
21969	Top1	topoisomerase (DNA) I	NA	-0.1258	7.848435	-1.09111	0.030803	0.234109
211612	Ptchd1	patched domain containing 1, transcript variant X5	NA	0.263946	4.402636	1.200758	0.03085427	0.234351
118567516	LOC118567516	uncharacterized LOC118567516, transcript variant X	NA	-0.38288	2.027102	-1.30395	0.03088875	0.234351
72621	Pdzd11	PDZ domain containing 11, transcript variant 1	NA	-0.1562	5.121139	-1.11435	0.03089031	0.234351
102632	Acad11	acyl-Coenzyme A dehydrogenase family, member 11	NA	-0.28753	3.109193	-1.22055	0.03089257	0.234351
329416	Nostrin	nitric oxide synthase trafficker	NA	0.586385	1.191309	1.501479	0.03094149	0.234613
54153	Rasa4	RAS p21 protein activator 4, transcript variant 1	NA	0.263233	3.571614	1.200166	0.03095604	0.234614
30947	Adat1	adenosine deaminase, tRNA-specific 1, transcript var	NA	-0.22371	3.794805	-1.16773	0.03103309	0.234992
66235	Eif1ax	eukaryotic translation initiation factor 1A, X-linked	NA	0.129044	7.400826	1.093569	0.03104535	0.234992
16563	Kif2a	kinesin family member 2A, transcript variant 2	NA	0.111457	7.447629	1.080319	0.03107324	0.234992
230848	Zbtb40	zinc finger and BTB domain containing 40, transcript	NA	-0.2849	3.846999	-1.21832	0.03109379	0.234992
109241	Mbd5	methyl-CpG binding domain protein 5, transcript varia	NA	0.184408	5.096658	1.136351	0.03110843	0.234992
13649	Egfr	epidermal growth factor receptor, transcript variant X	NA	0.295487	4.387523	1.227299	0.03111132	0.234992
12388	Ctnnd1	catenin (cadherin associated protein), delta 1, transcr	NA	-0.12923	7.65118	-1.09371	0.03111162	0.234992
71988	Esco2	establishment of sister chromatid cohesion N-acetyltr	NA	-0.3135	3.235819	-1.24272	0.03113342	0.234992
52123	Agpat5	1-acylglycerol-3-phosphate O-acyltransferase 5 (lyso	NA	-0.12832	5.973409	-1.09302	0.03113587	0.234992
16601	Klf9	Kruppel-like factor 9	NA	0.251794	3.123431	1.190687	0.03116705	0.235004
621976	Tmem170b	transmembrane protein 170B, transcript variant 2	NA	0.153883	6.657833	1.11256	0.0311831	0.235004
72190	2510009E07Rik	RIKEN cDNA 2510009E07 gene	NA	0.122274	6.602589	1.088449	0.03119332	0.235004
74600	Mrpl47	mitochondrial ribosomal protein L47	NA	0.227339	3.800884	1.170674	0.0312079	0.235004
17472	Gbp4	guanylate binding protein 4, transcript variant 5	NA	-0.56067	0.77834	-1.47495	0.03120968	0.235004
217695	Zfyve1	zinc finger, FYVE domain containing 1, transcript vari	NA	-0.14453	5.638713	-1.10537	0.03123993	0.235046
224109	Nrros	negative regulator of reactive oxygen species, transcr	NA	-0.29794	2.999591	-1.22939	0.03125452	0.235046
105246591	Gm41861	predicted gene, 41861	NA	-0.47174	1.314295	-1.38678	0.03125857	0.235046
239652	Zfp641	zinc finger protein 641, transcript variant X7	NA	0.179994	4.724619	1.132879	0.03129519	0.235213
320940	Atp11c	ATPase, class VI, type 11C, transcript variant X2	NA	0.16896	5.855155	1.124248	0.03132685	0.235342
208144	Dhx37	DEAH (Asp-Glu-Ala-His) box polypeptide 37	NA	-0.19101	4.782215	-1.14156	0.03134494	0.235365
14183	Fgfr2	fibroblast growth factor receptor 2, transcript variant 3	NA	-0.14159	5.567455	-1.10312	0.03135889	0.235365
215474	Sec22c	SEC22 homolog C, vesicle trafficking protein, transcr	NA	-0.1404	5.739551	-1.10221	0.03146743	0.236071
12540	Cdc42	cell division cycle 42, transcript variant 1	NA	0.097561	9.090645	1.069963	0.03148503	0.236094
66840	Wdr45b	WD repeat domain 45B	NA	0.14214	5.965859	1.10354	0.03155292	0.236494
192652	Wdr81	WD repeat domain 81	NA	-0.18166	4.647153	-1.13419	0.03158411	0.236583
70472	Atad2	ATPase family, AAA domain containing 2	NA	-0.21327	4.34861	-1.15931	0.03160825	0.236583
56726	Sh3bgrl	SH3-binding domain glutamic acid-rich protein like	NA	0.097944	8.139386	1.070247	0.03160843	0.236583

107995	Cdc20	cell division cycle 20	NA	-0.21274	4.844129	-1.15889	0.0316366	0.236685
69046	Isca1	iron-sulfur cluster assembly 1	NA	0.123029	6.62006	1.089019	0.03170519	0.237
52398	Septin11	septin 11, transcript variant X22	NA	-0.11011	7.885433	-1.07931	0.0317175	0.237
68404	Nrn1	neuritin 1, transcript variant 1	NA	-0.13537	6.216325	-1.09838	0.03172233	0.237
75617	Rps25	ribosomal protein S25	NA	0.118737	7.982693	1.085784	0.03178125	0.237289
12333	Capn1	calpain 1, transcript variant 1	NA	-0.25812	3.231463	-1.19592	0.03179211	0.237289
13000	Csnk2a2	casein kinase 2, alpha prime polypeptide, transcript v	NA	0.136703	6.22528	1.09939	0.03180479	0.237289
214290	Tut7	terminal uridylyl transferase 7, transcript variant X9	NA	0.137822	6.105874	1.100243	0.03182209	0.237309
51795	Srpx	sushi-repeat-containing protein, transcript variant 1	NA	-0.44151	1.692897	-1.35803	0.03185589	0.23735
12778	Ackr3	atypical chemokine receptor 3, transcript variant 2	NA	-0.18208	4.429339	-1.13452	0.03187112	0.23735
69537	Dnase1l1	deoxyribonuclease 1-like 1, transcript variant 2	NA	0.639887	0.883768	1.558207	0.03187131	0.23735
26914	Macroh2a1	macroH2A.1 histone, transcript variant 2	NA	-0.10698	7.736396	-1.07697	0.0318891	0.237374
228662	Btbd3	BTB (POZ) domain containing 3, transcript variant 2	NA	0.138391	6.630207	1.100677	0.03192246	0.237468
76130	Las1l	LAS1-like (S. cerevisiae), transcript variant 2	NA	0.121886	5.990193	1.088157	0.03193682	0.237468
63856	Taf8	TATA-box binding protein associated factor 8, transcı	NA	-0.16268	4.76801	-1.11937	0.03194558	0.237468
27418	Mkln1	muskelin 1, intracellular mediator containing kelch mc	NA	0.140157	6.595773	1.102025	0.03201526	0.237878
12785	Cnbp	cellular nucleic acid binding protein, transcript variant	NA	0.101322	8.044942	1.072756	0.03206792	0.23816
100038570	Prcd	photoreceptor disc component, transcript variant X6	NA	0.726435	-0.18432	1.654545	0.03208582	0.238185
12632	Cfl2	cofilin 2, muscle	NA	0.099992	7.778718	1.071768	0.03215383	0.238549
65114	Vps35	VPS35 retromer complex component	NA	0.104841	7.386992	1.075376	0.03216428	0.238549
320265	Tafa1	TAFA chemokine like family member 1	NA	0.198842	4.104638	1.147777	0.03219438	0.238582
70036	Dancr	differentiation antagonizing non-protein coding RNA,	NA	0.26724	3.225194	1.203503	0.03219804	0.238582
106947	Slc39a3	solute carrier family 39 (zinc transporter), member 3,	NA	-0.16225	5.924395	-1.11903	0.03225147	0.238865
210992	Lpcat1	lysophosphatidylcholine acyltransferase 1, transcript	NA	0.130184	6.760682	1.094433	0.03226559	0.238865
14747	Cmklr1	chemokine-like receptor 1, transcript variant X1	NA	-0.45672	1.404045	-1.37242	0.03230318	0.238873
280411	Lix1l	Lix1-like	NA	-0.15365	6.433162	-1.11238	0.03230721	0.238873
268721	Zswim8	zinc finger SWIM-type containing 8, transcript variant	NA	-0.10499	7.405038	-1.07549	0.03231069	0.238873
229759	Olfm3	olfactomedin 3, transcript variant X1	NA	0.19234	4.292045	1.142615	0.03232766	0.23889
56631	Trim17	tripartite motif-containing 17, transcript variant X2	NA	-0.27331	3.949721	-1.20858	0.03239518	0.239146
239546	Zfp647	zinc finger protein 647, transcript variant 2	NA	0.221277	4.523137	1.165765	0.03240126	0.239146
56873	Lmbr1	limb region 1	NA	0.139907	5.421122	1.101834	0.03241898	0.239146
11565	Adssl1	adenylosuccinate synthetase like 1	NA	0.371015	1.85999	1.293263	0.03242109	0.239146
12359	Cat	catalase	NA	-0.12394	6.521914	-1.0897	0.03245078	0.239256
234847	Spg7	SPG7, paraplegin matrix AAA peptidase subunit, tran	NA	0.142359	5.423098	1.103708	0.03246845	0.239278
214048	Larp1b	La ribonucleoprotein domain family, member 1B, tran	NA	0.266379	3.809406	1.202785	0.03255309	0.23972
21767	Tex264	testis expressed gene 264, transcript variant 2	NA	0.167734	4.58109	1.123293	0.03257307	0.23972
269003	Sap130	Sin3A associated protein, transcript variant 1	NA	-0.12794	6.224988	-1.09274	0.03261481	0.23972
80981	Arl4d	ADP-ribosylation factor-like 4D	NA	-0.22467	4.181191	-1.16851	0.03262108	0.23972
70984	4931406C07Rik	RIKEN cDNA 4931406C07 gene, transcript variant 3	NA	0.152861	5.218699	1.111772	0.03263308	0.23972
30805	Slc22a4	solute carrier family 22 (organic cation transporter), m	NA	0.38946	2.03202	1.309903	0.03263748	0.23972
19283	Ptprz1	protein tyrosine phosphatase, receptor type Z, polype	NA	0.133361	8.38946	1.096846	0.03264567	0.23972
69408	Dnajc17	DnaJ heat shock protein family (Hsp40) member C17	NA	0.274822	3.80374	1.209844	0.0326462	0.23972
268373	Ppia	peptidylprolyl isomerase A	NA	0.110797	10.00948	1.079824	0.03269014	0.239893
94090	Trim9	tripartite motif-containing 9, transcript variant X16	NA	0.149731	6.84372	1.109363	0.03270104	0.239893
320595	Phf8	PHD finger protein 8, transcript variant X1	NA	-0.1374	5.500506	-1.09992	0.032714	0.239893
70427	Mier2	MIER family member 2, transcript variant 1	NA	-0.1452	5.18899	-1.10588	0.03273239	0.23992
21815	Tgif1	TGFB-induced factor homeobox 1, transcript variant 2	NA	-0.25804	3.007961	-1.19585	0.03278111	0.24007
56438	Rbx1	ring-box 1	NA	0.11134	7.220666	1.080231	0.03278239	0.24007
13593	Ebf3	early B cell factor 3, transcript variant X27	NA	0.194481	7.418452	1.144312	0.03281312	0.240187
19799	Rn4.5s	4.5S RNA	NA	-0.9452	1.1291	-1.92545	0.03284515	0.240313
18671	Abcb1a	ATP-binding cassette, sub-family B (MDR/TAP), men	NA	0.328703	2.897716	1.255884	0.03289944	0.240602
16981	Lrrn3	leucine rich repeat protein 3, neuronal, transcript vari	NA	0.12556	6.897343	1.090931	0.03294162	0.240802
66202	1110059G10Rik	RIKEN cDNA 1110059G10 gene	NA	0.189448	4.148838	1.140328	0.03297764	0.240957
319675	Cep295	centrosomal protein 295, transcript variant X20	NA	0.147145	5.386469	1.107376	0.03301723	0.241058
64008	Aqp9	aquaporin 9, transcript variant 1	NA	0.767936	-0.29169	1.702832	0.03303472	0.241058
66520	2610001J05Rik	RIKEN cDNA 2610001J05 gene	NA	0.188767	4.906949	1.139789	0.03303587	0.241058
108705	Pttg1ip	pituitary tumor-transforming 1 interacting protein	NA	0.133325	5.635466	1.096818	0.03306471	0.241131
13426	Dync1i1	dynein cytoplasmic 1 intermediate chain 1, transcript	NA	0.176075	5.902892	1.129806	0.03307554	0.241131
13806	Eno1	enolase 1, alpha non-neuron, transcript variant 3	NA	0.143713	8.901653	1.104745	0.03318567	0.241702
72290	Lsm11	U7 snRNP-specific Sm-like protein LSM11	NA	0.120967	6.297603	1.087463	0.03318837	0.241702
16568	Kif3a	kinesin family member 3A, transcript variant 1	NA	0.106182	8.140897	1.076376	0.03320026	0.241702
26381	Esrrg	estrogen-related receptor gamma, transcript variant 4	NA	0.143883	5.595997	1.104875	0.03321319	0.241702
21402	Skp1	S-phase kinase-associated protein 1	NA	0.099145	8.671521	1.071138	0.03326329	0.241796
68166	Spire1	spire type actin nucleation factor 1, transcript variant	NA	0.127621	6.91627	1.092491	0.03327069	0.241796
242126	Slc22a15	solute carrier family 22 (organic anion/cation transpor	NA	0.2215	3.910845	1.165945	0.03327364	0.241796
80911	Acox3	acyl-Coenzyme A oxidase 3, pristanoyl, transcript var	NA	0.158442	4.950435	1.116081	0.03328553	0.241796
12615	Cenpa	centromere protein A, transcript variant 2	NA	-0.19474	4.349085	-1.14452	0.03335996	0.242228
14432	Gap43	growth associated protein 43	NA	0.151983	9.382939	1.111095	0.03338779	0.242288

16592	Fabp5	fatty acid binding protein 5, epidermal, transcript varia	NA	0.106076	8.135233	1.076297	0.03340313	0.242288
74134	Cyp2s1	cytochrome P450, family 2, subfamily s, polypeptide	NA	0.504004	1.127257	1.418144	0.03343167	0.242288
230753	Thrap3	thyroid hormone receptor associated protein 3, transc	NA	-0.11299	7.850509	-1.08146	0.03343957	0.242288
12404	Cbln1	cerebellin 1 precursor protein	NA	0.142759	6.153379	1.104014	0.03344872	0.242288
22330	Vcl	vinculin	NA	0.14046	5.336999	1.102257	0.03346107	0.242288
16593	Klc1	kinesin light chain 1, transcript variant b	NA	0.099672	8.875856	1.07153	0.0334725	0.242288
22193	Ube2e3	ubiquitin-conjugating enzyme E2E 3, transcript variar	NA	0.114928	7.924818	1.082921	0.03352519	0.242562
80515	Rnf227	ring finger protein 227	NA	0.115915	6.90498	1.083662	0.03362812	0.243199
108052	Slc14a1	solute carrier family 14 (urea transporter), member 1,	NA	0.437553	1.449853	1.354305	0.03368015	0.243215
100039795	Ildr2	immunoglobulin-like domain containing receptor 2, tra	NA	0.157901	5.175045	1.115663	0.03368311	0.243215
108167848	Gm12258	predicted gene 12258, transcript variant 1	NA	0.156028	5.041153	1.114215	0.03368439	0.243215
66882	Bzw1	basic leucine zipper and W2 domains 1, transcript va	NA	0.098657	8.74476	1.070776	0.03371038	0.243215
320587	Tmem88b	transmembrane protein 88B	NA	0.539237	0.840851	1.453204	0.03371813	0.243215
102637582	Gm34353	predicted gene, 34353, transcript variant X2	NA	0.4428	1.356645	1.35924	0.0337475	0.243215
243937	Zfp536	zinc finger protein 536, transcript variant X7	NA	-0.15052	5.044952	-1.10997	0.03375202	0.243215
233552	Gdpd5	glycerophosphodiester phosphodiesterase domain cc	NA	-0.15048	5.715236	-1.10994	0.03375673	0.243215
18618	Pemt	phosphatidylethanolamine N-methyltransferase, trans	NA	0.571629	0.863873	1.486201	0.03379605	0.243215
234595	Slc38a7	solute carrier family 38, member 7, transcript variant	NA	-0.17602	5.07053	-1.12976	0.03380507	0.243215
57028	Pdxp	pyridoxal (pyridoxine, vitamin B6) phosphatase	NA	0.12938	6.516459	1.093824	0.03380571	0.243215
67581	Tbc1d23	TBC1 domain family, member 23, transcript variant X	NA	0.134527	6.503359	1.097733	0.03382683	0.243215
15902	Id2	inhibitor of DNA binding 2	NA	0.152341	7.245583	1.111372	0.03383398	0.243215
66839	Dele1	DAP3 binding cell death enhancer 1	NA	-0.15756	5.36585	-1.1154	0.03386912	0.243215
17356	Afdn	afadin, adherens junction formation factor, transcript	NA	0.11368	7.775191	1.081984	0.03389483	0.243215
102639013	Gm14246	predicted gene 14246, transcript variant X3	NA	0.644669	0.382229	1.56338	0.03389985	0.243215
237178	Ppef1	protein phosphatase with EF hand calcium-binding dc	NA	0.819185	-0.05395	1.764409	0.03390829	0.243215
73144	Mir100hg	Mir100 Mirlet7a-2 Mir125b-1 cluster host gene, transc	NA	0.109215	8.505122	1.078641	0.03391222	0.243215
72569	Bbs5	Bardet-Biedl syndrome 5 (human), transcript variant	NA	0.228409	3.607384	1.171542	0.0339402	0.243215
18141	Nup50	nucleoporin 50	NA	-0.13282	5.9454	-1.09644	0.03395566	0.243215
100504734	Gm16794	predicted gene, 16794, transcript variant X4	NA	1.035822	-0.07896	2.050281	0.03395978	0.243215
19280	Ptprs	protein tyrosine phosphatase, receptor type, S, transc	NA	-0.10127	9.555208	-1.07272	0.0339725	0.243215
73288	Vps50	VPS50 EARP/GARPII complex subunit, transcript var	NA	0.130512	5.860898	1.094682	0.03397411	0.243215
493800	9330162012Rik	cDNA RIKEN 9330162012 gene	NA	0.660904	0.212157	1.581073	0.03400085	0.243299
74127	Krt80	keratin 80	NA	-0.95016	-0.31577	-1.93209	0.03409352	0.243805
66209	Inip	INTS3 and NABP interacting protein	NA	-0.14209	5.198728	-1.1035	0.03412999	0.243805
19089	Prkcsh	protein kinase C substrate 80K-H, transcript variant 1	NA	-0.13563	6.463611	-1.09857	0.03413585	0.243805
115486899	Gm51551	predicted gene, 51551, transcript variant X1	NA	-0.64344	0.762848	-1.56205	0.03414579	0.243805
320662	Casc1	cancer susceptibility candidate 1, transcript variant X	NA	-0.60122	0.612622	-1.517	0.03414654	0.243805
67710	Polr2g	polymerase (RNA) II (DNA directed) polypeptide G	NA	0.13769	5.262614	1.100142	0.03418377	0.243964
56742	Psrc1	proline/serine-rich coiled-coil 1, transcript variant 2	NA	-0.21621	3.757226	-1.16167	0.03421328	0.244068
319554	Idi1	isopentenyl-diphosphate delta isomerase	NA	0.171325	5.883064	1.126093	0.0342858	0.244448
20592	Kdm5d	lysine (K)-specific demethylase 5D, transcript variant	NA	0.384021	4.087576	1.304974	0.03431178	0.244448
414085	9330151L19Rik	RIKEN cDNA 9330151L19 gene	NA	0.204946	4.031658	1.152643	0.03434546	0.244448
319513	Pced1a	PC-esterase domain containing 1A, transcript variant	NA	-0.22344	4.231996	-1.16752	0.03435051	0.244448
56469	Pias1	protein inhibitor of activated STAT 1, transcript varian	NA	0.147834	5.959138	1.107904	0.03438017	0.244448
52014	Nus1	NUS1 dehydrodolichyl diphosphate synthase subunit	NA	0.114739	6.559128	1.082779	0.03438022	0.244448
71878	Fam83d	family with sequence similarity 83, member D	NA	-0.25209	3.015222	-1.19094	0.03438189	0.244448
241764	L3mbtl1	L3MBTL1 histone methyl-lysine binding protein, trans	NA	0.218806	4.268834	1.16377	0.03439507	0.244448
56460	Pkp3	plakophilin 3, transcript variant X1	NA	-1.0653	-0.24444	-2.0926	0.03441838	0.244448
381112	Arhgef33	Rho guanine nucleotide exchange factor (GEF) 33	NA	0.579458	1.329052	1.494288	0.03443627	0.244448
118568796	LOC118568796	uncharacterized LOC118568796	NA	0.441144	2.062925	1.35768	0.03443734	0.244448
14115	Fbln2	fibulin 2, transcript variant 2	NA	0.184934	4.66507	1.136765	0.03444687	0.244448
229227	4932438A13Rik	RIKEN cDNA 4932438A13 gene	NA	0.159428	6.422241	1.116844	0.03448614	0.24462
56209	Gde1	glycerophosphodiester phosphodiesterase 1	NA	0.130877	6.370661	1.094959	0.03451512	0.244637
67057	Yaf2	YY1 associated factor 2, transcript variant X1	NA	0.136134	5.758321	1.098957	0.03452857	0.244637
19076	Prim2	DNA primase, p58 subunit	NA	-0.16951	4.683723	-1.12468	0.0345517	0.244637
71523	8430429K09Rik	RIKEN cDNA 8430429K09 gene	NA	0.300683	3.116503	1.231727	0.03455487	0.244637
269180	Inpp4a	inositol polyphosphate-4-phosphatase, type I, transcr	NA	0.127986	6.459889	1.092767	0.03456375	0.244637
66599	Rdm1	RAD52 motif 1	NA	-0.36026	2.499176	-1.28366	0.03462664	0.244763
14734	Gpc3	glypican 3	NA	0.127947	5.875599	1.092737	0.03464635	0.244763
320469	9930014A18Rik	RIKEN cDNA 9930014A18 gene	NA	0.376096	2.499764	1.297825	0.03465612	0.244763
18128	Notch1	notch 1	NA	-0.11792	6.385838	-1.08517	0.03466215	0.244763
59042	Cope	coatomer protein complex, subunit epsilon	NA	0.150576	5.832228	1.110013	0.03466275	0.244763
216190	Appl2	adaptor protein, phosphotyrosine interaction, PH dom	NA	0.170943	5.363311	1.125794	0.03467173	0.244763
231672	Fbxw8	F-box and WD-40 domain protein 8	NA	-0.12524	6.3621	-1.09069	0.03475686	0.245257
27364	Srr	serine racemase, transcript variant X3	NA	-0.12071	6.217344	-1.08727	0.03478991	0.245384
105245412	Gm40872	predicted gene, 40872	NA	-0.65342	0.27257	-1.57289	0.03481736	0.245408
26556	Homer1	homer scaffolding protein 1, transcript variant d	NA	0.126332	6.227372	1.091515	0.03482339	0.245408
100041283	Gm3252	predicted gene 3252, transcript variant X8	NA	0.996025	0.096931	1.994498	0.03497889	0.246251

21952	Tnni1	troponin I, skeletal, slow 1, transcript variant 1	NA	-0.6614	0.769764	-1.58162	0.03498693	0.246251
231912	Katnal1	katanin p60 subunit A-like 1	NA	-0.14355	5.726986	-1.10462	0.03498848	0.246251
214189	Scgn	secretagogin, EF-hand calcium binding protein	NA	-0.66411	0.178255	-1.58459	0.03502	0.246366
52469	Coa3	cytochrome C oxidase assembly factor 3	NA	-0.13789	5.238387	-1.10029	0.03506063	0.246459
74237	Tubgcp2	tubulin, gamma complex associated protein 2, transcr	NA	0.145379	5.982858	1.106021	0.03506343	0.246459
100038746	Gm1976	predicted gene 1976, transcript variant 2	NA	0.278463	3.451316	1.212902	0.03509746	0.246591
23939	Mapk7	mitogen-activated protein kinase 7, transcript variant	NA	-0.15243	6.266646	-1.11144	0.03512136	0.246612
21400	Tcea2	transcription elongation factor A (SII), 2, transcript va	NA	0.222939	4.10442	1.167109	0.03513559	0.246612
20698	Sphk1	sphingosine kinase 1, transcript variant 5	NA	0.47143	1.371653	1.386483	0.03514583	0.246612
13875	Erf	Ets2 repressor factor	NA	-0.18371	4.798831	-1.1358	0.03523078	0.247101
56812	Dnajb2	DnaJ heat shock protein family (Hsp40) member B2,	NA	-0.13424	5.396306	-1.09751	0.03524619	0.247103
83814	Nedd4l	neural precursor cell expressed, developmentally dov	NA	-0.11613	7.755275	-1.08382	0.03526965	0.247129
272009	Srsf12	serine and arginine-rich splicing factor 12, transcript v	NA	0.175954	5.43921	1.129711	0.03528024	0.247129
14675	Gna14	guanine nucleotide binding protein, alpha 14	NA	-0.89612	-0.1855	-1.86105	0.03533887	0.24716
246707	Emilin2	elastin microfibril interfacer 2, transcript variant 2	NA	-0.38195	1.750363	-1.3031	0.03534948	0.24716
72106	Jmjd8	jumonji domain containing 8	NA	-0.13224	5.636708	-1.096	0.03536767	0.24716
71085	Arhgap19	Rho GTPase activating protein 19, transcript variant	NA	-0.21803	3.966135	-1.16314	0.03539131	0.24716
223697	Sun2	Sad1 and UNC84 domain containing 2, transcript var	NA	-0.18822	6.071259	-1.13936	0.03540773	0.24716
66343	Tmem177	transmembrane protein 177	NA	-0.24191	3.742758	-1.18255	0.03540965	0.24716
66194	Pycrl	pyrroline-5-carboxylate reductase-like	NA	-0.18759	4.702035	-1.13886	0.03541953	0.24716
328309	Gm9776	predicted gene 9776	NA	0.382993	1.980612	1.304044	0.03542129	0.24716
319317	Snhg11	small nucleolar RNA host gene 11	NA	0.166767	9.961871	1.12254	0.03542147	0.24716
73124	Golim4	golgi integral membrane protein 4, transcript variant 2	NA	0.137447	5.201431	1.099957	0.03545631	0.247275
16480	Jup	junction plakoglobin, transcript variant X1	NA	-0.11489	6.211946	-1.0829	0.03546829	0.247275
231207	Cpeb2	cytoplasmic polyadenylation element binding protein	NA	0.207807	6.140748	1.154932	0.03550106	0.247398
66170	Chchd5	coiled-coil-helix-coiled-coil-helix domain containing 5	NA	-0.3093	2.942494	-1.2391	0.0355567	0.247578
54486	Hpgds	hematopoietic prostaglandin D synthase	NA	0.336255	2.02019	1.262475	0.03555738	0.247578
16728	L1cam	L1 cell adhesion molecule, transcript variant 1	NA	-0.11487	8.370559	-1.08288	0.03559363	0.247724
227580	C1ql3	C1q-like 3	NA	0.250791	3.859299	1.18986	0.0356421	0.247956
105246807	Gm42031	predicted gene, 42031	NA	-0.37275	2.360577	-1.29482	0.03567127	0.248053
432467	Hnrnph3	heterogeneous nuclear ribonucleoprotein H3, transcri	NA	-0.13051	6.975569	-1.09468	0.0357087	0.248197
15510	Hspd1	heat shock protein 1 (chaperonin), transcript variant 1	NA	-0.11916	7.989184	-1.0861	0.0357226	0.248197
229504	Isg20l2	interferon stimulated exonuclease gene 20-like 2	NA	-0.15148	5.532421	-1.11071	0.03574446	0.248243
622404	Ccdc107	coiled-coil domain containing 107	NA	0.234883	3.772545	1.176811	0.03580918	0.248587
68229	Spindoc	spindlin interactor and repressor of chromatin binding	NA	-0.12553	6.195138	-1.09091	0.03585266	0.248625
14226	Fkbp1b	FK506 binding protein 1b, transcript variant 1	NA	0.272286	3.687268	1.20772	0.03586067	0.248625
217201	Rundc1	RUN domain containing 1, transcript variant X1	NA	-0.15618	5.008234	-1.11433	0.035866	0.248625
58249	Fibp	fibroblast growth factor (acidic) intracellular binding p	NA	0.160062	5.4788	1.117336	0.03587577	0.248625
69727	Usp46	ubiquitin specific peptidase 46, transcript variant X2	NA	0.130225	6.138534	1.094464	0.03589466	0.248643
11518	Add1	adducin 1 (alpha), transcript variant 2	NA	-0.11167	8.313487	-1.08048	0.03591312	0.248643
381813	Prmt8	protein arginine N-methyltransferase 8, transcript vari	NA	0.150806	5.374576	1.11019	0.03592425	0.248643
80985	Trim44	tripartite motif-containing 44	NA	0.11392	8.236584	1.082164	0.03595484	0.248749
100043257	Rbm3-ps	RNA binding motif (RNP1, RRM) protein 3	NA	0.828341	1.206496	1.775643	0.03598149	0.2488
105940408	Gm20498	predicted gene 20498, transcript variant 2	NA	0.361115	3.403908	1.284418	0.03599281	0.2488
11426	Macf1	microtubule-actin crosslinking factor 1, transcript vari	NA	0.137926	8.597371	1.100322	0.03604259	0.248953
224813	Lrrc73	leucine rich repeat containing 73	NA	0.235828	3.818939	1.177582	0.03606032	0.248953
100504361	Gm14230	predicted gene 14230, transcript variant X1	NA	-0.22382	3.73694	-1.16782	0.03606079	0.248953
107746	Rapgef1	Rap guanine nucleotide exchange factor (GEF) 1, tra	NA	0.099306	7.418125	1.071258	0.03608124	0.248988
12299	Cacng1	calcium channel, voltage-dependent, gamma subunit	NA	0.70339	0.617566	1.628326	0.03614173	0.2493
52120	Hgsnat	heparan-alpha-glucosaminide N-acetyltransferase	NA	0.188834	4.629952	1.139842	0.03619798	0.249582
72148	Tdrp	testis development related protein, transcript variant 4	NA	0.273197	3.042803	1.208483	0.03623033	0.249629
71728	Stk11ip	serine/threonine kinase 11 interacting protein, transcr	NA	-0.1707	4.922713	-1.1256	0.0362379	0.249629
16467	Atcay	ataxia, cerebellar, Cayman type	NA	-0.14586	7.979504	-1.10639	0.03625588	0.249629
19244	Ptp4a2	protein tyrosine phosphatase 4a2, transcript variant 1	NA	0.09633	8.018115	1.06905	0.03629508	0.249629
22423	Wnt8b	wingless-type MMTV integration site family, member	NA	-0.69703	1.069365	-1.62117	0.03630539	0.249629
100040671	Gm2897	predicted gene 2897	NA	0.269783	4.734609	1.205626	0.03631223	0.249629
227737	Niban2	niban apoptosis regulator 2	NA	-0.13472	6.051103	-1.09788	0.03632677	0.249629
239408	Tmem74	transmembrane protein 74, transcript variant X1	NA	0.159697	4.877175	1.117052	0.03633135	0.249629
11994	Pcdh15	protocadherin 15, transcript variant L	NA	0.212146	4.454027	1.15841	0.03634287	0.249629
50911	Exosc9	exosome component 9	NA	0.128169	5.592512	1.092906	0.03636292	0.249661
18541	Pcnt	pericentrin (kendrin), transcript variant X19	NA	-0.15013	4.867793	-1.10967	0.03643021	0.249991
26909	Exo1	exonuclease 1	NA	-0.32201	2.704839	-1.25007	0.0364566	0.249991
74244	Atg7	autophagy related 7, transcript variant 3	NA	-0.21229	4.156081	-1.15853	0.03645978	0.249991
66242	Mrps16	mitochondrial ribosomal protein S16, transcript variar	NA	0.202297	3.830309	1.150529	0.03647261	0.249991
212153	Ccdc191	coiled-coil domain containing 191, transcript variant X	NA	-0.34855	2.661103	-1.27328	0.03649183	0.249991
56772	Mllt11	myeloid/lymphoid or mixed-lineage leukemia; translo	NA	0.100744	9.277687	1.072326	0.03650315	0.249991
231093	Agbl5	ATP/GTP binding protein-like 5, transcript variant 9	NA	-0.1231	5.785278	-1.08907	0.03655918	0.25027
80909	Castor2	cytosolic arginine sensor for mTORC1 subunit 2	NA	-0.14492	5.876535	-1.10567	0.03661672	0.250558

241732	Tspyl3	TSPY-like 3	NA	0.144149	5.334364	1.105078	0.03671245	0.250983
20671	Sox17	SRY (sex determining region Y)-box 17, transcript va	NA	0.289842	2.764603	1.222506	0.0367157	0.250983
230917	Tmem201	transmembrane protein 201, transcript variant 2	NA	-0.1176	6.139911	-1.08492	0.03672502	0.250983
70573	Tbccd1	TBCC domain containing 1, transcript variant X7	NA	0.208659	3.901287	1.155614	0.03674665	0.251025
22439	Xk	X-linked Kx blood group	NA	0.208599	3.726883	1.155566	0.03678702	0.251195
27045	Nit1	nitrilase 1, transcript variant X1	NA	-0.17228	5.221445	-1.12684	0.03682882	0.251375
29875	Iqgap1	IQ motif containing GTPase activating protein 1	NA	0.124808	5.626729	1.090363	0.03688775	0.251672
224814	Abcc10	ATP-binding cassette, sub-family C (CFTR/MRP), me	NA	-0.20721	3.838501	-1.15445	0.03692045	0.251789
15366	Hmmr	hyaluronan mediated motility receptor (RHAMM), trar	NA	-0.25172	3.684985	-1.19063	0.03694844	0.251875
16553	Kif13a	kinesin family member 13A	NA	0.134442	5.433936	1.097668	0.03700926	0.25211
329384	Ptrh1	peptidyl-tRNA hydrolase 1 homolog, transcript varian	NA	-0.62914	0.651583	-1.54665	0.03701399	0.25211
13844	Ephb2	Eph receptor B2, transcript variant 2	NA	-0.10575	7.613661	-1.07605	0.03704153	0.252133
18704	Pik3c2a	phosphatidylinositol-4-phosphate 3-kinase catalytic s	NA	0.190525	4.895183	1.141179	0.03704834	0.252133
268934	Grm4	glutamate receptor, metabotropic 4, transcript variant	NA	-0.18346	4.528588	-1.13561	0.03708495	0.252277
212514	Spice1	spindle and centriole associated protein 1, transcript	NA	-0.21885	4.052653	-1.1638	0.03715051	0.252527
14601	Ghrh	growth hormone releasing hormone, transcript varian	NA	0.84043	-0.02293	1.790584	0.03717544	0.252527
20823	Ssb	Sjogren syndrome antigen B, transcript variant 2	NA	0.099058	7.602525	1.071074	0.03718676	0.252527
74108	Parn	poly(A)-specific ribonuclease (deadenylation nucleas	NA	0.141978	5.155072	1.103417	0.0371961	0.252527
399568	Cdin1	CDAN1 interacting nuclease 1, transcript variant 2	NA	-0.21037	4.523179	-1.15699	0.03720397	0.252527
233045	Gm26604	predicted gene, 26604	NA	0.397407	1.693021	1.317139	0.03722623	0.252527
68510	Ints1	integrator complex subunit 1, transcript variant X3	NA	-0.12702	6.15934	-1.09204	0.03723032	0.252527
84095	Pi4k2a	phosphatidylinositol 4-kinase type 2 alpha	NA	-0.15302	5.616745	-1.11189	0.03725057	0.252559
100504589	Gm11454	predicted gene 11454	NA	-0.4895	1.431332	-1.40396	0.0373531	0.253138
243819	Ppp6r1	protein phosphatase 6, regulatory subunit 1	NA	-0.12987	6.631876	-1.0942	0.03736717	0.253138
102637602	Gm34369	predicted gene, 34369, transcript variant X1	NA	0.685765	-0.17319	1.608555	0.03742115	0.253399
102632821	Gm13778	predicted gene 13778, transcript variant X3	NA	-0.4787	1.627536	-1.39348	0.037455	0.253451
13682	Eif4a2	eukaryotic translation initiation factor 4A2, transcript	NA	-0.11104	9.106007	-1.08	0.03746	0.253451
223666	Arhgap39	Rho GTPase activating protein 39, transcript variant	NA	-0.13215	6.170409	-1.09592	0.03752552	0.253686
76485	Glt8d1	glycosyltransferase 8 domain containing 1, transcript	NA	-0.16348	5.768532	-1.11999	0.037526	0.253686
109349	Fam163b	family with sequence similarity 163, member B	NA	0.228592	3.649001	1.171691	0.03759432	0.25385
74383	Ubap2l	ubiquitin-associated protein 2-like, transcript variant	NA	-0.12692	7.657011	-1.09196	0.03760455	0.25385
68550	Tefm	transcription elongation factor, mitochondrial	NA	0.288799	2.933451	1.221623	0.03761156	0.25385
93705	Pcdhgb8	protocadherin gamma subfamily B, 8, transcript varia	NA	-0.36071	2.454231	-1.28405	0.03761266	0.25385
28000	Prpf19	pre-mRNA processing factor 19, transcript variant 2	NA	-0.10894	7.704244	-1.07843	0.03765162	0.254008
244431	Sgcz	sarcoglycan zeta, transcript variant X3	NA	0.602535	1.662294	1.518382	0.03767572	0.254065
67860	S100a16	S100 calcium binding protein A16, transcript variant	NA	0.23844	3.661929	1.179717	0.03774499	0.254427
68626	Elac2	elaC ribonuclease Z 2, transcript variant 3	NA	-0.14247	5.31408	-1.1038	0.0378052	0.254727
101113	Snx21	sorting nexin family member 21, transcript variant X5	NA	0.232247	4.153086	1.174663	0.03785824	0.25483
228012	Tlk1	tousled-like kinase 1, transcript variant X6	NA	0.142179	6.466499	1.103571	0.0378653	0.25483
331524	Xkrx	X-linked Kx blood group related, X-linked, transcript v	NA	0.738485	0.464514	1.668423	0.0378806	0.25483
67160	Eef1g	eukaryotic translation elongation factor 1 gamma	NA	-0.10622	9.085289	-1.07641	0.03790466	0.25483
73750	Whrn	whirlin, transcript variant 1	NA	-0.25111	4.644288	-1.19013	0.03791675	0.25483
58235	Nectin1	nectin cell adhesion molecule 1, transcript variant 1	NA	-0.12298	6.651441	-1.08898	0.03794993	0.25483
269378	Ahcy	S-adenosylhomocysteine hydrolase	NA	-0.14092	6.02231	-1.10261	0.03795054	0.25483
11944	Atp4a	ATPase, H+/K+ exchanging, gastric, alpha polypeptic	NA	0.645466	0.252346	1.564244	0.03795804	0.25483
116733	Vps4a	vacuolar protein sorting 4A	NA	0.107991	6.909651	1.077726	0.03797559	0.25483
19737	Rgs5	regulator of G-protein signaling 5, transcript variant 1	NA	0.118485	6.752056	1.085594	0.03798133	0.25483
81535	Sgpp1	sphingosine-1-phosphate phosphatase 1	NA	0.200495	4.759513	1.149092	0.03799273	0.25483
64340	Dhx38	DEAH (Asp-Glu-Ala-His) box polypeptide 38, transcri	NA	-0.16072	5.508757	-1.11785	0.03804938	0.254903
67547	Slc39a8	solute carrier family 39 (metal ion transporter), memb	NA	0.194388	4.245178	1.144238	0.03805341	0.254903
232854	Zfp418	zinc finger protein 418	NA	-0.33927	2.118412	-1.26512	0.03806482	0.254903
67266	Dipk1a	divergent protein kinase domain 1A	NA	0.167779	5.224167	1.123328	0.03806626	0.254903
54616	Extl3	exostosin-like glycosyltransferase 3, transcript varian	NA	-0.11702	6.865784	-1.08449	0.0381451	0.255203
76832	Hyls1	HYLS1, centriolar and ciliogenesis associated	NA	-0.18725	4.165176	-1.13859	0.03819116	0.255203
18707	Pik3cd	phosphatidylinositol-4,5-bisphosphate 3-kinase catal	NA	-0.14588	5.379612	-1.1064	0.03819291	0.255203
269695	Rnft2	ring finger protein, transmembrane 2, transcript varia	NA	-0.13285	6.564775	-1.09646	0.03819311	0.255203
225027	Srsf7	serine and arginine-rich splicing factor 7, transcript v	NA	-0.11268	7.892969	-1.08123	0.03820262	0.255203
208869	Dock3	dedicator of cyto-kinesis 3, transcript variant X10	NA	0.122589	6.667509	1.088687	0.03821884	0.255203
237411	Zfp938	zinc finger protein 938, transcript variant 2	NA	0.197053	4.30594	1.146354	0.03822623	0.255203
115487829	Gm17586	predicted gene, 17586	NA	0.406971	1.747233	1.325899	0.03823651	0.255203
103743	Tmem98	transmembrane protein 98	NA	-0.2051	3.808583	-1.15276	0.03828749	0.255438
320534	Tmem104	transmembrane protein 104	NA	-0.22886	3.674342	-1.17191	0.0383197	0.25546
67203	Nde1	nudE neurodevelopment protein 1, transcript variant	NA	-0.22869	4.918483	-1.17177	0.03833428	0.25546
269784	Cntn4	contactin 4, transcript variant 1	NA	0.234593	4.353626	1.176575	0.03833783	0.25546
66302	Rmdn1	regulator of microtubule dynamics 1, transcript varian	NA	0.251404	3.108424	1.190365	0.03837378	0.25547
226778	Mark1	MAP/microtubule affinity regulating kinase 1, transcri	NA	0.10575	7.153848	1.076054	0.03839046	0.25547
53422	Ybx2	Y box protein 2, transcript variant X5	NA	-0.24681	3.978421	-1.18658	0.03841565	0.25547
16432	Itm2b	integral membrane protein 2B	NA	0.129713	8.029544	1.094076	0.0384199	0.25547

70604	Dnajb14	DnaJ heat shock protein family (Hsp40) member B14	NA		0.199084	4.040784	1.147969	0.03842697	0.25547
235293	Sc5d	sterol-C5-desaturase, transcript variant X1	NA		0.167147	6.377048	1.122836	0.03843349	0.25547
20842	Stag1	stromal antigen 1, transcript variant 1	NA		0.134112	6.488505	1.097417	0.03846679	0.255495
330657	Prss53	protease, serine 53, transcript variant X1	NA		-0.7666	0.455222	-1.70125	0.03848593	0.255495
16969	Zbtb7a	zinc finger and BTB domain containing 7a, transcript	NA		-0.18313	5.148652	-1.13535	0.03852546	0.255495
26398	Map2k4	mitogen-activated protein kinase kinase 4, transcript	NA		0.11478	6.797327	1.08281	0.03853555	0.255495
118567335	LOC118567335	uncharacterized LOC118567335, transcript variant X	NA		-0.16562	4.595195	-1.12165	0.03854413	0.255495
210982	Bicral	BRD4 interacting chromatin remodeling complex ass	NA		-0.11718	6.570032	-1.08461	0.03854583	0.255495
11793	Atg5	autophagy related 5, transcript variant 1	NA		0.167435	4.824626	1.12306	0.03854715	0.255495
224088	Atp13a3	ATPase type 13A3, transcript variant X1	NA		0.17105	6.172303	1.125877	0.03858386	0.255588
72605	Car10	carbonic anhydrase 10, transcript variant 3	NA		0.138813	5.780985	1.100999	0.03859262	0.255588
26877	B3galt1	UDP-Gal:betaGlcNAc beta 1,3-galactosyltransferase,	NA		0.17217	5.74561	1.126752	0.03862999	0.255731
21769	Zfand3	zinc finger, AN1-type domain 3, transcript variant X1	NA		-0.12189	6.783946	-1.08816	0.03866944	0.255742
668917	Zfp133-ps	zinc finger protein 133, pseudogene	NA		-0.40507	2.060315	-1.32415	0.03868033	0.255742
71701	Pnpt1	polyribonucleotide nucleotidyltransferase 1, transcript	NA		0.181165	4.83249	1.133799	0.03869884	0.255742
26557	Homer2	homer scaffolding protein 2, transcript variant 2	NA		0.149879	5.37915	1.109477	0.03870681	0.255742
235312	C1qtnf5	C1q and tumor necrosis factor related protein 5, trans	NA		0.307378	2.474261	1.237457	0.0387102	0.255742
67414	Mfn1	mitofusin 1	NA		0.123521	6.2641	1.08939	0.03875193	0.255755
16451	Jak1	Janus kinase 1, transcript variant X1	NA		0.150081	6.634436	1.109632	0.03876544	0.255755
381511	Pdp1	pyruvate dehyrogenase phosphatase catalytic subuni	NA		0.125819	6.219641	1.091127	0.03879346	0.255755
328092	Dtd2	D-tyrosyl-tRNA deacylase 2, transcript variant 2	NA		0.260795	3.154172	1.198139	0.03879628	0.255755
59289	Ackr2	atypical chemokine receptor 2, transcript variant 1	NA		0.507344	1.623905	1.421431	0.03882474	0.255755
69440	Dennd6b	DENN/MADD domain containing 6B	NA		0.124271	6.516676	1.089957	0.03884748	0.255755
16779	Lamb2	laminin, beta 2	NA		-0.1666	5.164975	-1.12241	0.03885506	0.255755
56454	Aldh18a1	aldehyde dehydrogenase 18 family, member A1, tran	NA		-0.17343	6.27052	-1.12774	0.03886521	0.255755
15165	Hcn1	hyperpolarization activated cyclic nucleotide gated pc	NA		0.33035	2.964746	1.257318	0.03886857	0.255755
76967	2700049A03Rik	RIKEN cDNA 2700049A03 gene, transcript variant X	NA		-0.16997	4.351733	-1.12503	0.03888373	0.255755
76306	Slc18b1	solute carrier family 18, subfamily B, member 1, trans	NA		-0.19757	4.475714	-1.14677	0.03890491	0.255755
56433	Vps29	VPS29 retromer complex component, transcript varia	NA		0.112676	6.459677	1.081232	0.03891444	0.255755
217653	Mis18bp1	MIS18 binding protein 1	NA		-0.21378	3.809649	-1.15972	0.0389165	0.255755
66448	Mrpl20	mitochondrial ribosomal protein L20, transcript varian	NA		0.197316	5.328022	1.146563	0.03895974	0.255833
223918	Spryd3	SPRY domain containing 3	NA		0.145741	5.954169	1.106299	0.03895983	0.255833
18408	Slc25a15	solute carrier family 25 (mitochondrial carrier ornithin	NA		-0.21974	3.43896	-1.16453	0.03900245	0.25601
71835	Lancl2	LanC (bacterial lantibiotic synthetase component C)-l	NA		0.134163	6.771763	1.097456	0.0390229	0.256041
214764	Edrf1	erythroid differentiation regulatory factor 1, transcript	NA		0.15176	4.929742	1.110924	0.03904986	0.256114
12509	Cd59a	CD59a antigen, transcript variant 2	NA		0.262245	3.258375	1.199343	0.03907292	0.256162
13637	Efna2	ephrin A2	NA		-0.13786	5.410304	-1.10027	0.03911478	0.256333
17221	Cd46	CD46 antigen, complement regulatory protein, transc	NA		0.296766	2.456458	1.228388	0.03918624	0.256698
74637	Shpk	sedoheptulokinase	NA		0.432326	1.305751	1.349408	0.03927791	0.257195
242960	Fbxl5	F-box and leucine-rich repeat protein 5, transcript var	NA		0.110594	6.556711	1.079673	0.03932177	0.257379
381677	Vgf	VGF nerve growth factor inducible, transcript variant	NA		0.275274	4.53628	1.210224	0.03945694	0.258134
382207	Jade3	jade family PHD finger 3, transcript variant 1	NA		0.165006	4.856695	1.121171	0.03946894	0.258134
209743	Minar1	membrane integral NOTCH2 associated receptor 1, t	NA		0.250022	3.539203	1.189225	0.03949453	0.258198
11431	Acp1	acid phosphatase 1, soluble, transcript variant X1	NA		0.128765	5.863864	1.093357	0.03953824	0.25826
105246117	Gm41458	predicted gene, 41458, transcript variant X1	NA		0.786864	-0.40112	1.72532	0.03954816	0.25826
67778	Zfp639	zinc finger protein 639, transcript variant X3	NA		0.145121	5.571695	1.105823	0.03957595	0.25826
107065	Lrrtm2	leucine rich repeat transmembrane neuronal 2	NA		0.138748	5.075234	1.100949	0.03958432	0.25826
216395	Rxylt1	ribitol xylosyltransferase 1, transcript variant X3	NA		0.166681	4.451888	1.122473	0.0395919	0.25826
14897	Trip12	thyroid hormone receptor interactor 12, transcript vari	NA		0.113291	7.801276	1.081693	0.03959922	0.25826
105522	Ankrd28	ankyrin repeat domain 28, transcript variant X9	NA		0.16419	5.999567	1.120537	0.03963045	0.258352
56698	Phax	phosphorylated adaptor for RNA export, transcript va	NA		0.120988	6.084582	1.087479	0.0396686	0.258352
16646	Kpna1	karyopherin (importin) alpha 1, transcript variant X1	NA		0.130447	6.875288	1.094633	0.03966862	0.258352
67974	Ccny	cyclin Y, transcript variant X1	NA		0.111385	6.74963	1.080265	0.03967959	0.258352
12537	Cdk11b	cyclin-dependent kinase 11B, transcript variant X13	NA		0.129336	6.643542	1.09379	0.03970599	0.258352
103534	Mgat4b	mannoside acetylglucosaminyltransferase 4, isoenzy	NA		0.116877	6.752597	1.084385	0.03970856	0.258352
54189	Rabep1	rabaptin, RAB GTPase binding effector protein 1, trar	NA		0.106414	7.166617	1.076549	0.03975428	0.258546
226896	Tfap2d	transcription factor AP-2, delta, transcript variant 1	NA		0.146926	5.141264	1.107208	0.03979236	0.25869
171469	Gpr37l1	G protein-coupled receptor 37-like 1	NA		0.712835	-0.19777	1.639021	0.0398467	0.258815
242608	Podn	podocan, transcript variant 3	NA		0.417249	1.795331	1.335379	0.03985596	0.258815
70974	Pgm2l1	phosphoglucomutase 2-like 1, transcript variant X1	NA		0.158615	7.740904	1.116215	0.03985926	0.258815
224170	Dzip3	DAZ interacting protein 3, zinc finger, transcript variai	NA		-0.13055	6.753969	-1.09471	0.0399361	0.25921
18181	Nrf1	nuclear respiratory factor 1, transcript variant 1	NA		-0.12405	5.591021	-1.08979	0.03998239	0.259398
22288	Utrn	utrophin, transcript variant X14	NA		0.140654	5.152609	1.102404	0.03999686	0.259398
66848	Fuca2	fucosidase, alpha-L- 2, plasma, transcript variant 2	NA		0.18471	4.656766	1.136588	0.04007788	0.25982
22158	Tulp3	tubby-like protein 3	NA		-0.15499	5.072856	-1.11342	0.04009996	0.259859
60525	Acss2	acyl-CoA synthetase short-chain family member 2	NA		-0.18089	4.296712	-1.13358	0.04015768	0.260051
235339	Dlat	dihydrolipoamide S-acetyltransferase (E2 component	NA		0.120526	5.997768	1.087131	0.04017297	0.260051
72535	Aldh1b1	aldehyde dehydrogenase 1 family, member B1	NA		-0.20066	4.12313	-1.14922	0.04017756	0.260051

74149	Zfp946	zinc finger protein 946, transcript variant 2	NA	-0.13522	5.657347	-1.09826	0.04022232	0.260184
237898	Usp32	ubiquitin specific peptidase 32, transcript variant X3	NA	0.153918	6.565691	1.112587	0.0402333	0.260184
237360	Adamts14	a disintegrin-like and metallopeptidase (reprolysin typ	NA	-0.52728	1.041552	-1.44121	0.04028802	0.260184
102636048	Gm38485	predicted gene, 38485, transcript variant X1	NA	0.812284	0.593772	1.75599	0.04029158	0.260184
269788	Lhfpl4	lipoma HMGIC fusion partner-like protein 4	NA	-0.11759	7.555727	-1.08492	0.04030126	0.260184
277463	Gpr107	G protein-coupled receptor 107	NA	-0.17026	5.666652	-1.12526	0.04030412	0.260184
18575	Pde1c	phosphodiesterase 1C, transcript variant 5	NA	0.16656	4.598162	1.122379	0.04032151	0.260184
264895	Acsf2	acyl-CoA synthetase family member 2	NA	-0.19333	3.815597	-1.1434	0.04034948	0.260184
13026	Pcyt1a	phosphate cytidylyltransferase 1, choline, alpha isofo	NA	0.12602	5.986115	1.091279	0.04035247	0.260184
102637243	Gm34110	predicted gene, 34110, transcript variant X1	NA	0.538039	1.141732	1.451998	0.04035792	0.260184
216443	Mars1	methionine-tRNA synthetase 1, transcript variant 2	NA	-0.11795	6.085541	-1.08519	0.04038037	0.260226
14702	Gng2	guanine nucleotide binding protein (G protein), gamm	NA	0.099379	9.816393	1.071312	0.04041543	0.260348
243897	Ggn	gametogenetin, transcript variant 1	NA	-0.60006	0.169255	-1.51578	0.0404601	0.26041
26968	Islr	immunoglobulin superfamily containing leucine-rich re	NA	-0.18436	4.626834	-1.13631	0.0404863	0.26041
69456	Commd10	COMM domain containing 10	NA	0.165364	4.810062	1.121449	0.04050606	0.26041
14118	Fbn1	fibrillin 1	NA	0.176413	5.122985	1.13007	0.0405234	0.26041
24044	Scamp2	secretory carrier membrane protein 2, transcript varia	NA	-0.1569	4.963959	-1.11489	0.04053016	0.26041
13548	Dyrk1a	dual-specificity tyrosine-(Y)-phosphorylation regulate	NA	0.103494	6.952372	1.074372	0.04060835	0.26041
449521	Zfp213	zinc finger protein 213	NA	-0.21268	3.674579	-1.15884	0.0406089	0.26041
68018	Cert1	ceramide transporter 1, transcript variant 2	NA	0.1621	5.804281	1.118915	0.0406133	0.26041
19328	Rab12	RAB12, member RAS oncogene family, transcript var	NA	0.113471	6.961644	1.081828	0.04061958	0.26041
108927	Lhfp	lipoma HMGIC fusion partner	NA	0.149681	5.11769	1.109324	0.04062112	0.26041
13690	Eif4g2	eukaryotic translation initiation factor 4, gamma 2, tra	NA	0.111281	10.77381	1.080187	0.0406528	0.26041
19094	Mapk11	mitogen-activated protein kinase 11	NA	-0.15134	5.378733	-1.1106	0.04066591	0.26041
67998	Retreg3	reticulophagy regulator family member 3, transcript va	NA	-0.13647	6.385885	-1.09921	0.04069377	0.26041
16173	Il18	interleukin 18, transcript variant 2	NA	0.290327	3.025387	1.222917	0.04070002	0.26041
13392	Dlx2	distal-less homeobox 2	NA	-0.18203	5.57874	-1.13448	0.04070359	0.26041
69270	Gins1	GINS complex subunit 1 (Psf1 homolog), transcript va	NA	-0.26721	3.294681	-1.20348	0.04071751	0.26041
75146	Mfsd13a	major facilitator superfamily domain containing 13a	NA	-0.23691	3.101116	-1.17847	0.04072897	0.26041
53610	Nono	non-POU-domain-containing, octamer binding protein	NA	-0.12039	8.906372	-1.08703	0.04074185	0.26041
59050	Nsa2	NSA2 ribosome biogenesis homolog	NA	0.104309	6.993386	1.074979	0.04074336	0.26041
17151	Ccndbp1	cyclin D-type binding-protein 1	NA	0.134138	5.540568	1.097437	0.04074505	0.26041
27368	Tbl2	transducin (beta)-like 2, transcript variant 1	NA	-0.15098	4.73715	-1.11032	0.04084619	0.260954
380684	Nefh	neurofilament, heavy polypeptide	NA	0.245571	3.511484	1.185562	0.04087667	0.260987
66087	Emc3	ER membrane protein complex subunit 3	NA	0.119847	6.008802	1.08662	0.04088345	0.260987
20273	Scn8a	sodium channel, voltage-gated, type VIII, alpha, trans	NA	0.116094	6.485447	1.083796	0.04091654	0.261096
235459	Gtf2a2	general transcription factor II A, 2, transcript variant 2	NA	0.14218	5.623656	1.103572	0.04099903	0.261407
93706	Pcdhgc3	protocadherin gamma subfamily C, 3	NA	-0.13006	7.772994	-1.09434	0.04101068	0.261407
668553	Gm9237	predicted pseudogene 9237, transcript variant X1	NA	0.641393	0.667401	1.559834	0.04102235	0.261407
244813	Bsx	brain specific homeobox, transcript variant X2	NA	-0.46557	1.1232	-1.38086	0.04102955	0.261407
67707	Mrpl24	mitochondrial ribosomal protein L24	NA	-0.17876	5.323471	-1.13191	0.04105875	0.26149
630836	2010315B03Rik	RIKEN cDNA 2010315B03 gene, transcript variant 3	NA	0.248983	3.669377	1.188369	0.04114243	0.261836
76539	Fam204a	family with sequence similarity 204, member A, transc	NA	0.162919	4.810239	1.11955	0.04114517	0.261836
280635	Emilin3	elastin microfibril interfacer 3, transcript variant 2	NA	-0.44464	1.251187	-1.36097	0.0412536	0.262423
69538	Antxr1	anthrax toxin receptor 1	NA	0.172071	4.866849	1.126675	0.04131435	0.262668
83768	Dpp7	dipeptidylpeptidase 7, transcript variant X1	NA	-0.25828	3.013346	-1.19605	0.04132442	0.262668
75472	Cfap126	cilia and flagella associated protein 126, transcript va	NA	0.348426	2.30868	1.273171	0.04136684	0.262819
21679	Tead4	TEA domain family member 4, transcript variant 2	NA	-0.63661	0.068708	-1.55467	0.04138049	0.262819
20975	Synj2	synaptojanin 2, transcript variant 7	NA	0.344988	2.042693	1.270141	0.04140904	0.262898
217378	Dnajc27	DnaJ heat shock protein family (Hsp40) member C27	NA	0.137573	5.189007	1.100053	0.04146836	0.263172
277414	Trp53i11	transformation related protein 53 inducible protein 11	NA	-0.11035	7.497584	-1.07949	0.04151939	0.263393
56744	Pf4	platelet factor 4	NA	0.4525	2.182805	1.368409	0.04157794	0.263539
19697	Rela	v-rel reticuloendotheliosis viral oncogene homolog A	NA	-0.14032	5.0455	-1.10215	0.04158966	0.263539
113002583	Shld3	shieldin complex subunit 3	NA	0.309018	2.323708	1.238864	0.04161136	0.263539
56191	Tro	trophinin, transcript variant 4	NA	0.115158	7.338323	1.083094	0.04162651	0.263539
279028	Adamts13	a disintegrin-like and metallopeptidase (reprolysin typ	NA	-0.25858	3.244416	-1.1963	0.04163376	0.263539
68897	Disp1	dispatched RND transporter family member 1, transc	NA	-0.24249	3.780254	-1.18303	0.04163957	0.263539
192663	Abcg4	ATP binding cassette subfamily G member 4	NA	-0.15442	5.414872	-1.11297	0.04175542	0.264078
245537	Nlgn3	neuroligin 3	NA	-0.1415	5.851321	-1.10305	0.04175714	0.264078
69749	Epb41l4aos	erythrocyte membrane protein band 4.1 like 4a, oppo	NA	0.270544	2.614367	1.206262	0.04179644	0.264177
278240	Spin2c	spindlin family, member 2C	NA	0.227023	3.247758	1.170417	0.04180529	0.264177
13205	Ddx3x	DEAD box helicase 3, X-linked	NA	0.135123	8.581519	1.098187	0.04182502	0.264199
102637674	Gm34425	predicted gene, 34425, transcript variant X2	NA	-0.56596	0.745188	-1.48037	0.04185741	0.26425
11603	Agrn	agrin, transcript variant X14	NA	-0.09119	8.764779	-1.06525	0.04186555	0.26425
52477	Angel2	angel homolog 2, transcript variant 2	NA	0.11408	6.152753	1.082285	0.04195855	0.264415
105242884	Gm38970	predicted gene, 38970	NA	-0.55336	1.321176	-1.4675	0.04196455	0.264415
64406	Sp5	trans-acting transcription factor 5	NA	0.285439	2.898862	1.218781	0.04197125	0.264415
109754	Cyb5r3	cytochrome b5 reductase 3, transcript variant X1	NA	0.132735	6.53799	1.096371	0.04197518	0.264415

53601	Pcdh12	protocadherin 12	NA	-0.27549	3.033066	-1.21041	0.04197997	0.264415
67911	Zfp169	zinc finger protein 169, transcript variant X4	NA	-0.1851	4.852631	-1.13689	0.04198919	0.264415
140723	Cacng5	calcium channel, voltage-dependent, gamma subunit	NA	0.241603	3.581036	1.182305	0.0420143	0.264471
545554	Ankrd34a	ankyrin repeat domain 34A	NA	-0.17734	4.151148	-1.13079	0.04204037	0.26452
67628	Anp32b	acidic (leucine-rich) nuclear phosphoprotein 32 family	NA	-0.13335	6.932622	-1.09684	0.04206175	0.26452
14412	Slc6a13	solute carrier family 6 (neurotransmitter transporter, (	NA	0.237295	4.549689	1.17878	0.04207674	0.26452
70853	Vwa3b	von Willebrand factor A domain containing 3B, transc	NA	-0.78621	-0.03503	-1.72453	0.04210239	0.26452
269132	Colgalt2	collagen beta(1-O)galactosyltransferase 2	NA	0.271384	2.891584	1.206965	0.04210335	0.26452
170799	Rtkn2	rhotekin 2, transcript variant 1	NA	-0.25013	3.14996	-1.18931	0.04213065	0.264589
212285	Arap2	ArfGAP with RhoGAP domain, ankyrin repeat and PH	NA	0.247774	3.395842	1.187374	0.04217679	0.264633
69305	Dcps	decapping enzyme, scavenger	NA	-0.15162	4.851377	-1.11082	0.04217962	0.264633
75317	Parpbp	PARP1 binding protein	NA	-0.32922	2.406095	-1.25633	0.04218655	0.264633
118568705	LOC118568705	MLV-related proviral Env polyprotein-like, transcript v	NA	-0.16538	4.698501	-1.12146	0.04220278	0.264633
654804	4732471J01Rik	RIKEN cDNA 4732471J01 gene, transcript variant 1	NA	0.530365	0.470669	1.444295	0.04222376	0.264663
68379	Ciz1	CDKN1A interacting zinc finger protein 1, transcript v	NA	-0.1437	5.990948	-1.10473	0.04227369	0.264682
237400	Mex3d	mex3 RNA binding family member D	NA	-0.11562	6.628084	-1.08344	0.04227391	0.264682
100039210	Gm2102	predicted gene 2102	NA	0.927304	-0.48926	1.901719	0.04228031	0.264682
74511	Lrrc17	leucine rich repeat containing 17	NA	-0.28113	2.909909	-1.21514	0.04229183	0.264682
69754	Fbxo7	F-box protein 7, transcript variant 1	NA	0.24123	3.724322	1.182	0.04233884	0.264749
69387	Dnajb13	DnaJ heat shock protein family (Hsp40) member B13	NA	-0.5533	0.776563	-1.46744	0.04234804	0.264749
70797	Ankib1	ankyrin repeat and IBR domain containing 1, transcrip	NA	0.145269	6.072653	1.105937	0.04235279	0.264749
228769	Psmf1	proteasome (prosome, macropain) inhibitor subunit 1	NA	0.124782	5.555143	1.090343	0.04236763	0.264749
108168244	Gm46519	predicted gene, 46519	NA	-0.63675	0.534944	-1.55483	0.04254245	0.265654
59079	Erbin	Erbb2 interacting protein, transcript variant 3	NA	0.158511	6.407481	1.116135	0.04254711	0.265654
100040048	Ccl27b	chemokine (C-C motif) ligand 27b, transcript variant X	NA	0.659311	0.42459	1.579328	0.04256141	0.265654
12006	Axin2	axin 2	NA	-0.13724	5.662047	-1.0998	0.04258677	0.26571
72404	Wdr44	WD repeat domain 44, transcript variant 2	NA	0.18005	4.165512	1.132923	0.04266694	0.266108
211586	Tfdp2	transcription factor Dp 2, transcript variant 5	NA	0.153639	5.287436	1.112372	0.04279611	0.266812
237940	Aoc2	amine oxidase, copper containing 2 (retina-specific),	NA	-0.26814	3.26694	-1.20426	0.04290043	0.26736
13010	Cst3	cystatin C	NA	0.146227	6.588281	1.106671	0.04292064	0.267383
105245389	Gm40853	predicted gene, 40853, transcript variant X7	NA	0.707346	-0.16627	1.632798	0.04294781	0.26745
107476	Acaca	acetyl-Coenzyme A carboxylase alpha	NA	0.119891	7.269061	1.086653	0.04309918	0.26829
107684	Coro2a	coronin, actin binding protein 2A, transcript variant 2	NA	0.171265	4.493995	1.126045	0.04312868	0.268371
13190	Dct	dopachrome tautomerase	NA	-0.70938	1.358723	-1.6351	0.04318289	0.268504
76608	Hectd3	HECT domain E3 ubiquitin protein ligase 3	NA	-0.14136	5.940394	-1.10295	0.04318304	0.268504
83762	Otof	otoferlin, transcript variant X6	NA	-0.19347	3.681871	-1.14351	0.04324302	0.268774
52132	Ccdc97	coiled-coil domain containing 97, transcript variant 1	NA	-0.16433	5.796436	-1.12065	0.04331691	0.269131
67561	Wdr48	WD repeat domain 48	NA	-0.10905	6.443746	-1.07852	0.04336473	0.269325
109108	Slc30a9	solute carrier family 30 (zinc transporter), member 9,	NA	0.119797	6.821899	1.086582	0.04340152	0.269408
635702	Naaladl2	N-acetylated alpha-linked acidic dipeptidase-like 2	NA	0.595429	0.397391	1.510922	0.0434195	0.269408
333639	Mamld1	mastermind-like domain containing 1, transcript varia	NA	0.168974	4.462694	1.124259	0.0434521	0.269408
319184	H2bc12	H2B clustered histone 12	NA	-1.04373	-0.62461	-2.06155	0.04347048	0.269408
77128	Crebrf	CREB3 regulatory factor, transcript variant X7	NA	0.170781	4.705826	1.125668	0.04347072	0.269408
22433	Xbp1	X-box binding protein 1, transcript variant 1	NA	-0.11477	6.19384	-1.0828	0.04347746	0.269408
380702	Shisa6	shisa family member 6	NA	0.151946	4.640681	1.111067	0.04351771	0.269555
320492	A830018L16Rik	RIKEN cDNA A830018L16 gene, transcript variant 4	NA	0.267627	3.270709	1.203826	0.04358733	0.269883
65105	Arl6ip4	ADP-ribosylation factor-like 6 interacting protein 4	NA	0.173289	4.890042	1.127626	0.04364245	0.269942
14724	Gp1bb	glycoprotein Ib, beta polypeptide, transcript variant 1	NA	0.259659	4.135621	1.197196	0.04366492	0.269942
228482	Arhgap11a	Rho GTPase activating protein 11A	NA	-0.26642	5.424078	-1.20282	0.04367031	0.269942
68525	Evc2	EvC ciliary complex subunit 2	NA	-0.22618	3.215075	-1.16974	0.04369083	0.269942
227624	Rabl6	RAB, member RAS oncogene family-like 6	NA	-0.13289	6.136165	-1.09649	0.04369756	0.269942
665180	Clec2l	C-type lectin domain family 2, member L	NA	0.185959	4.613901	1.137573	0.0437011	0.269942
240888	Gpr161	G protein-coupled receptor 161, transcript variant X4	NA	0.111564	6.83917	1.080399	0.04371299	0.269942
68066	Slc25a39	solute carrier family 25, member 39, transcript varian	NA	-0.11954	5.776367	-1.08639	0.04374896	0.270062
70767	Prpf3	pre-mRNA processing factor 3, transcript variant X1	NA	-0.14582	5.194479	-1.10636	0.04379583	0.270147
18150	Npm3	nucleoplasmin 3	NA	-0.17701	4.311851	-1.13054	0.04379592	0.270147
210710	Gab3	growth factor receptor bound protein 2-associated pro	NA	0.643404	0.01732	1.56201	0.0438823	0.270577
20688	Sp4	trans-acting transcription factor 4, transcript variant 1	NA	0.124501	6.182723	1.090131	0.04392188	0.27068
67299	Dock7	dedicator of cytokinesis 7, transcript variant X10	NA	0.101071	7.420849	1.072569	0.04395907	0.27068
216560	Wdpcp	WD repeat containing planar cell polarity effector, tra	NA	-0.25329	3.275443	-1.19192	0.04396259	0.27068
213027	Evi5l	ecotropic viral integration site 5 like, transcript variant	NA	-0.10627	7.157369	-1.07644	0.04397664	0.27068
20661	Sort1	sortilin 1, transcript variant X3	NA	-0.12824	7.195736	-1.09296	0.0439821	0.27068
22402	Ccn4	cellular communication network factor 4, transcript va	NA	-0.36324	2.386982	-1.28631	0.04403159	0.270801
66166	S100a14	S100 calcium binding protein A14, transcript variant 1	NA	-0.89143	-0.2848	-1.85501	0.0440351	0.270801
664883	Nova1	NOVA alternative splicing regulator 1, transcript varia	NA	0.142488	7.79558	1.103807	0.04409648	0.271044
66849	Ppp1r2	protein phosphatase 1, regulatory inhibitor subunit 2	NA	0.123773	6.787806	1.08958	0.04410788	0.271044
208117	Aph1b	aph1 homolog B, gamma secretase subunit	NA	0.167014	5.495363	1.122733	0.04413118	0.271084
18082	Nipsnap1	nipsnap homolog 1	NA	0.104722	6.728087	1.075288	0.0441801	0.271283

50524	Sall2	spalt like transcription factor 2, transcript variant 2	NA	-0.10746	6.88272	-1.07733	0.04423068	0.271437
223646	Naprt	nicotinate phosphoribosyltransferase, transcript varia	NA	-0.53543	0.57707	-1.44938	0.04424014	0.271437
59046	Arpp19	cAMP-regulated phosphoprotein 19, transcript varian	NA	0.148792	7.170177	1.108641	0.04427033	0.271437
105005	Lratd1	LRAT domain containing 1	NA	-0.12045	6.579329	-1.08707	0.04427368	0.271437
22690	Zfp28	zinc finger protein 28	NA	0.162181	4.583841	1.118977	0.04428873	0.271437
30946	Abt1	activator of basal transcription 1	NA	-0.1851	3.955255	-1.1369	0.04434744	0.271662
81898	Sf3b1	splicing factor 3b, subunit 1	NA	0.105003	8.507234	1.075496	0.04435877	0.271662
268465	Eme1	essential meiotic structure-specific endonuclease 1, t	NA	-0.34771	2.232774	-1.27254	0.04438552	0.271724
72807	Zfp429	zinc finger protein 429	NA	-0.32791	2.12426	-1.25519	0.04446745	0.272105
66684	Tceal8	transcription elongation factor A (SII)-like 8, transcript	NA	0.116801	6.166799	1.084328	0.04448129	0.272105
71096	Sntg1	syntrophin, gamma 1, transcript variant 4	NA	0.335339	3.046239	1.261674	0.04449883	0.27211
16539	Kcns2	K+ voltage-gated channel, subfamily S, 2, transcript v	NA	0.31432	3.050652	1.243425	0.0445374	0.272244
216831	Arhgap44	Rho GTPase activating protein 44, transcript variant 2	NA	0.166298	4.849043	1.122175	0.04456646	0.272304
67264	Ndufb8	NADH:ubiquinone oxidoreductase subunit B8, transcr	NA	0.130696	6.372767	1.094822	0.04458079	0.272304
226610	Fam78b	family with sequence similarity 78, member B, transcr	NA	-0.12749	6.06479	-1.09239	0.04462998	0.272502
594844	Tceal3	transcription elongation factor A (SII)-like 3, transcript	NA	0.156158	5.359538	1.114315	0.04468553	0.272739
20272	Scn7a	sodium channel, voltage-gated, type VII, alpha	NA	0.592335	0.5518	1.507685	0.04471905	0.272758
74334	Ranbp10	RAN binding protein 10, transcript variant X6	NA	-0.13522	5.810378	-1.09826	0.04472846	0.272758
70892	Ttll7	tubulin tyrosine ligase-like family, member 7, transcrip	NA	0.187048	4.873396	1.138432	0.04473888	0.272758
56523	Pmfbp1	polyamine modulated factor 1 binding protein 1, trans	NA	0.350254	2.478822	1.274785	0.0447652	0.272803
69161	Manbal	mannosidase, beta A, lysosomal-like	NA	0.161878	4.993276	1.118743	0.04478617	0.272803
74410	Ttll11	tubulin tyrosine ligase-like family, member 11, transcr	NA	0.268563	2.800516	1.204608	0.0447965	0.272803
18607	Pdpk1	3-phosphoinositide dependent protein kinase 1, trans	NA	0.127144	6.490743	1.092129	0.04492695	0.273464
72469	Plcd3	phospholipase C, delta 3, transcript variant 1	NA	0.371868	1.948186	1.294027	0.04493874	0.273464
56371	Fzr1	fizzy and cell division cycle 20 related 1	NA	-0.11525	6.445211	-1.08316	0.04499091	0.273594
20773	Sptlc2	serine palmitoyltransferase, long chain base subunit 2	NA	0.137478	6.057598	1.099981	0.04501025	0.273594
20370	Sez6	seizure related gene 6, transcript variant X6	NA	-0.10694	8.208619	-1.07694	0.04501049	0.273594
17299	Mettl1	methyltransferase like 1, transcript variant X1	NA	-0.30952	3.314138	-1.23929	0.04503899	0.273665
69737	Ttl	tubulin tyrosine ligase	NA	0.117375	6.327602	1.084759	0.04506886	0.273744
71564	Izumo4	IZUMO family member 4, transcript variant X3	NA	0.194456	3.954942	1.144293	0.04508816	0.273759
211378	6720489N17Rik	RIKEN cDNA 6720489N17 gene, transcript variant 1	NA	-0.26156	3.297565	-1.19877	0.04510877	0.273782
380686	Cnrip1	cannabinoid receptor interacting protein 1	NA	0.114037	6.264663	1.082253	0.04515248	0.273821
244059	Chd2	chromodomain helicase DNA binding protein 2	NA	0.154163	6.439589	1.112776	0.04516138	0.273821
14086	Fscn1	fascin actin-bundling protein 1	NA	-0.09991	8.971873	-1.0717	0.04518739	0.273821
81703	Jdp2	Jun dimerization protein 2, transcript variant 3	NA	0.245792	3.2271	1.185743	0.0451992	0.273821
13853	Epm2a	epilepsy, progressive myoclonic epilepsy, type 2 gen	NA	0.221183	4.126466	1.165689	0.04519937	0.273821
19054	Ppp2r3d	protein phosphatase 2 (formerly 2A), regulatory subu	NA	-0.12252	6.022045	-1.08863	0.04523499	0.273935
12055	Bcl7c	B cell CLL/lymphoma 7C, transcript variant 1	NA	0.248253	6.910007	1.187768	0.04525898	0.273967
216742	Fnip1	folliculin interacting protein 1, transcript variant X2	NA	0.163854	5.015234	1.120276	0.04528643	0.273967
19165	Psen2	presenilin 2, transcript variant 1	NA	0.237628	3.340904	1.179052	0.04529371	0.273967
66753	Erlec1	endoplasmic reticulum lectin 1, transcript variant X5	NA	0.14078	5.73478	1.102501	0.04532256	0.273967
13007	Csrp1	cysteine and glycine-rich protein 1, transcript variant 1	NA	0.171032	4.865679	1.125863	0.04532439	0.273967
69726	Smyd3	SET and MYND domain containing 3	NA	0.163897	4.713251	1.120309	0.04540226	0.274141
66865	Pmpca	peptidase (mitochondrial processing) alpha	NA	-0.11336	6.880449	-1.08174	0.0454103	0.274141
66515	Cul7	cullin 7	NA	-0.11707	6.600123	-1.08453	0.0454283	0.274141
223693	Tmem184b	transmembrane protein 184b, transcript variant 3	NA	-0.12422	5.528455	-1.08992	0.04543711	0.274141
217980	Larp4b	La ribonucleoprotein domain family, member 4B, tran	NA	0.127218	6.798516	1.092186	0.04543871	0.274141
74352	Zfp84	zinc finger protein 84	NA	-0.14608	5.364159	-1.10656	0.0454543	0.274141
19719	Rfng	RFNG O-fucosylpeptide 3-beta-N-acetylglucosaminyl	NA	-0.11527	6.431559	-1.08318	0.04549007	0.274255
21968	Tom1	target of myb1 trafficking protein, transcript variant 1	NA	0.1829	4.26248	1.135164	0.0455299	0.274318
81907	Tmem108	transmembrane protein 108, transcript variant 1	NA	-0.16648	5.110897	-1.12232	0.04553428	0.274318
116891	Derl2	Der1-like domain family, member 2, transcript variant	NA	-0.18808	5.387956	-1.13925	0.04557913	0.274351
66616	Snx9	sorting nexin 9	NA	0.197959	3.99286	1.147074	0.04558016	0.274351
17318	Mid1	midline 1, transcript variant 6	NA	-0.54137	5.094718	-1.45535	0.04559333	0.274351
319757	Smo	smoothened, frizzled class receptor, transcript varian	NA	-0.12747	5.62237	-1.09238	0.04560723	0.274351
76365	Tbx18	T-box18	NA	0.223304	3.563534	1.167404	0.04565756	0.274553
213491	Szrd1	SUZ RNA binding domain containing 1, transcript var	NA	-0.13407	6.415632	-1.09739	0.04568203	0.274598
384763	Zfp667	zinc finger protein 667, transcript variant X4	NA	0.181934	4.067982	1.134404	0.0457073	0.274601
117148	Necab2	N-terminal EF-hand calcium binding protein 2, transcr	NA	0.159441	5.253021	1.116855	0.04571772	0.274601
102639192	Gm35558	predicted gene, 35558, transcript variant X2	NA	0.290664	2.429394	1.223203	0.04573303	0.274601
100041194	Ahnak2	AHNAK nucleoprotein 2, transcript variant 2	NA	0.325938	2.350928	1.25348	0.04580583	0.274843
327951	Cyb5d1	cytochrome b5 domain containing 1	NA	-0.12792	5.438021	-1.09272	0.04580725	0.274843
14679	Gnai3	guanine nucleotide binding protein (G protein), alpha	NA	0.111148	7.290585	1.080087	0.04587163	0.275128
58208	Bcl11b	B cell leukemia/lymphoma 11B, transcript variant 3	NA	-0.13608	8.030367	-1.09892	0.04590765	0.275243
333605	Frmpd4	FERM and PDZ domain containing 4, transcript varia	NA	0.333566	2.707619	1.260124	0.0460079	0.275552
208092	Chmp6	charged multivesicular body protein 6	NA	0.143365	5.014292	1.104478	0.04601137	0.275552
18477	Prdx1	peroxiredoxin 1	NA	0.100959	7.096293	1.072486	0.04601621	0.275552
320460	Vwc2l	von Willebrand factor C domain-containing protein 2-l	NA	0.226192	3.454718	1.169743	0.04602696	0.275552

18023	Nfe2l1	nuclear factor, erythroid derived 2,-like 1, transcript va	NA	-0.1266	7.25114	-1.09172	0.04605197	0.275552
13193	Dcx	doublecortin, transcript variant 4	NA	0.115875	10.06145	1.083632	0.04606077	0.275552
100125931	Phtf1os	putative homeodomain transcription factor 1, opposite	NA	0.389968	2.114356	1.310364	0.04611357	0.275675
240283	Dmxl1	Dmx-like 1	NA	0.210599	5.499592	1.157168	0.04611522	0.275675
319650	A430108G06Rik	RIKEN cDNA A430108G06 gene	NA	0.413313	1.516064	1.33174	0.04618498	0.275989
20519	Slc22a3	solute carrier family 22 (organic cation transporter), m	NA	0.465553	1.157007	1.380846	0.04621513	0.275989
11479	Acvr1b	activin A receptor, type 1B	NA	-0.12195	7.025247	-1.0882	0.04627378	0.275989
69219	Ddah1	dimethylarginine dimethylaminohydrolase 1, transcrip	NA	0.10327	7.427329	1.074205	0.04627668	0.275989
27041	G3bp1	GTPase activating protein (SH3 domain) binding prot	NA	-0.13884	7.374499	-1.10102	0.04627978	0.275989
71819	Kif23	kinesin family member 23	NA	-0.18666	4.186928	-1.13813	0.04628728	0.275989
229906	Gtf2b	general transcription factor IIB	NA	0.13695	5.257225	1.099578	0.04629316	0.275989
57785	Rangrf	RAN guanine nucleotide release factor, transcript var	NA	-0.28129	2.93574	-1.21528	0.04630344	0.275989
67349	1700086P04Rik	RIKEN cDNA 1700086P04 gene, transcript variant X	NA	0.779249	0.685256	1.716237	0.04632539	0.276019
102638638	Gm14114	predicted gene 14114, transcript variant X4	NA	0.407512	1.691648	1.326396	0.04642233	0.276434
66053	Ppil2	peptidylprolyl isomerase (cyclophilin)-like 2, transcript	NA	-0.12379	6.25128	-1.08959	0.04642907	0.276434
23877	Fiz1	Flt3 interacting zinc finger protein 1, transcript variant	NA	-0.15214	5.52002	-1.11122	0.04645734	0.276501
319469	A230056J06Rik	RIKEN cDNA A230056J06 gene	NA	-0.56111	0.601215	-1.4754	0.04652822	0.276822
320011	Uggt1	UDP-glucose glycoprotein glucosyltransferase 1	NA	-0.1097	6.322212	-1.079	0.04657037	0.276971
13169	Dbnl	drebrin-like, transcript variant 3	NA	-0.11319	6.579556	-1.08162	0.04661765	0.277151
94109	Csmd1	CUB and Sushi multiple domains 1, transcript variant	NA	0.217397	4.479559	1.162634	0.04666542	0.277234
231413	Grsf1	G-rich RNA sequence binding factor 1, transcript vari	NA	0.13213	6.694458	1.09591	0.04667088	0.277234
170716	Cyp4f13	cytochrome P450, family 4, subfamily f, polypeptide 1	NA	0.418205	1.768746	1.336264	0.0466951	0.277234
73100	2900092D14Rik	RIKEN cDNA 2900092D14 gene	NA	0.130636	5.846081	1.094776	0.04671473	0.277234
14964	H2-D1	histocompatibility 2, D region locus 1	NA	0.197899	4.71395	1.147027	0.04671679	0.277234
22171	Tyms	thymidylate synthase, transcript variant 1	NA	-0.17154	4.394825	-1.12626	0.04673887	0.277259
102636641	Gm26618	predicted gene, 26618, transcript variant X2	NA	0.427205	1.745389	1.344626	0.04675946	0.277259
70405	Calml3	calmodulin-like 3	NA	-1.30212	-1.17448	-2.46591	0.04677217	0.277259
67199	Pfdn1	prefoldin 1	NA	0.142793	5.33265	1.104041	0.04680368	0.277272
75221	Dpp3	dipeptidylpeptidase 3, transcript variant 1	NA	-0.11395	6.122073	-1.08219	0.04682484	0.277272
72685	Dnajc6	DnaJ heat shock protein family (Hsp40) member C6,	NA	0.149678	6.866381	1.109322	0.04682545	0.277272
56229	Thsd1	thrombospondin, type I, domain 1, transcript variant 3	NA	0.199842	3.860618	1.148572	0.04687573	0.277458
330627	Trim66	tripartite motif-containing 66, transcript variant X4	NA	0.358023	2.6825	1.281668	0.04689095	0.277458
13644	Efs	embryonal Fyn-associated substrate	NA	-0.16894	4.991632	-1.12423	0.04693918	0.277642
320333	D830030K20Rik	RIKEN cDNA D830030K20 gene, transcript variant 1	NA	0.265727	3.928936	1.202242	0.04696169	0.277656
16324	Inhbb	inhibin beta-B	NA	-0.18924	4.19802	-1.14016	0.04697564	0.277656
14073	Faah	fatty acid amide hydrolase, transcript variant 1	NA	-0.17298	4.252783	-1.12739	0.04702816	0.277808
56175	Bace2	beta-site APP-cleaving enzyme 2, transcript variant X	NA	0.467183	1.06703	1.382408	0.04703542	0.277808
107568	Wwp1	WW domain containing E3 ubiquitin protein ligase 1,	NA	0.240188	5.124352	1.181146	0.04705318	0.277812
18457	Bloc1s6	biogenesis of lysosomal organelles complex-1, subur	NA	0.129731	5.749179	1.09409	0.04711479	0.278071
432516	Myo1a	myosin IA	NA	-0.40585	1.212709	-1.32487	0.04713123	0.278071
77744	Bora	bora, aurora kinase A activator, transcript variant 1	NA	-0.25017	3.760984	-1.18935	0.04717647	0.278173
93702	Pcdhgb5	protocadherin gamma subfamily B, 5	NA	-0.21259	3.908769	-1.15877	0.04718277	0.278173
15944	Irgm1	immunity-related GTPase family M member 1, transc	NA	0.455495	1.23479	1.371254	0.04728936	0.278576
27221	Chaf1a	chromatin assembly factor 1, subunit A (p150)	NA	-0.25601	4.332397	-1.19417	0.04730225	0.278576
71750	R3hdm2	R3H domain containing 2, transcript variant X23	NA	-0.10032	7.424567	-1.07201	0.04732787	0.278576
239217	Kctd12	potassium channel tetramerisation domain containing	NA	0.154719	7.357568	1.113204	0.04733049	0.278576
17258	Mef2a	myocyte enhancer factor 2A, transcript variant X8	NA	0.126446	6.672088	1.091602	0.04733674	0.278576
18213	Ntrk3	neurotrophic tyrosine kinase, receptor, type 3, transc	NA	0.107012	7.585817	1.076996	0.04736733	0.278656
12035	Bcat1	branched chain aminotransferase 1, cytosolic, transc	NA	-0.11191	6.618101	-1.08066	0.04743922	0.278677
108664	Atp6v1h	ATPase, H+ transporting, lysosomal V1 subunit H, tra	NA	0.096481	7.083151	1.069163	0.04744295	0.278677
118568306	LOC118568306	igE-binding protein-like	NA	-0.52226	1.317904	-1.4362	0.04745296	0.278677
27966	Rrp9	ribosomal RNA processing 9, U3 small nucleolar RN/	NA	-0.17938	4.428042	-1.13239	0.04746245	0.278677
108167931	Gm11650	predicted gene 11650	NA	0.450748	1.429266	1.366749	0.04748404	0.278677
66538	Rps19bp1	ribosomal protein S19 binding protein 1	NA	0.172328	4.077767	1.126875	0.04748933	0.278677
55988	Snx12	sorting nexin 12, transcript variant 1	NA	-0.14603	6.229143	-1.10652	0.04749759	0.278677
15980	Ifngr2	interferon gamma receptor 2	NA	0.116804	6.448371	1.08433	0.04750806	0.278677
68731	Rbfa	ribosome binding factor A	NA	0.175399	4.403709	1.129277	0.04754244	0.278779
13194	Ddb1	damage specific DNA binding protein 1	NA	-0.09491	8.539048	-1.068	0.04758832	0.278947
98488	Gtf3c3	general transcription factor IIIC, polypeptide 3	NA	0.132136	5.194948	1.095915	0.04768825	0.279405
56724	Cript	cysteine-rich PDZ-binding protein	NA	0.120452	5.887193	1.087075	0.04770618	0.279405
19679	Pitpnm2	phosphatidylinositol transfer protein, membrane-asso	NA	-0.11812	6.456203	-1.08532	0.04774068	0.279405
56420	Ppp4c	protein phosphatase 4, catalytic subunit, transcript va	NA	-0.10946	6.073661	-1.07883	0.04774528	0.279405
244585	Rpgrip1l	Rpgrip1-like, transcript variant X8	NA	-0.17781	4.356226	-1.13117	0.04775227	0.279405
76108	Rap2a	RAS related protein 2a	NA	0.11601	6.366194	1.083733	0.04781442	0.279668
18166	Npy1r	neuropeptide Y receptor Y1, transcript variant 2	NA	0.219825	3.360575	1.164592	0.04784408	0.279741
68948	Fam216a	family with sequence similarity 216, member A	NA	0.118908	5.810196	1.085913	0.04786758	0.279741
74370	Rptor	regulatory associated protein of MTOR, complex 1, tr	NA	-0.11451	5.6768	-1.08261	0.0478784	0.279741
52662	Ldlrad4	low density lipoprotein receptor class A domain conta	NA	0.16546	4.762214	1.121524	0.04789693	0.279748

18752	Prkcg	protein kinase C, gamma, transcript variant X2	NA		0.244049	3.799222	1.184312	0.04793765	0.279886
14348	Fut9	fucosyltransferase 9	NA		0.151393	6.251019	1.110642	0.04799994	0.280047
15117	Has2	hyaluronan synthase 2	NA		0.297298	2.409902	1.228841	0.04800423	0.280047
102641609	Gm38565	predicted gene, 38565, transcript variant X1	NA		-0.56142	0.199234	-1.47572	0.04803001	0.280047
73824	Snhg6	small nucleolar RNA host gene 6	NA		0.203099	3.806496	1.151169	0.04803416	0.280047
101883	Igflr1	IGF-like family receptor 1	NA		-0.29855	2.775732	-1.22991	0.04807616	0.280192
20585	Hltf	helicase-like transcription factor, transcript variant 1	NA		-0.13493	4.893353	-1.09804	0.04812506	0.280376
329217	Panct2	pluripotency-associated noncoding transcript 2	NA		-0.41298	1.313017	-1.33144	0.04817873	0.280589
11520	Plin2	perilipin 2, transcript variant X3	NA		-0.17308	4.615016	-1.12746	0.04822159	0.280738
69215	Sat2	spermidine/spermine N1-acetyl transferase 2, transcr	NA		0.240671	3.266345	1.181542	0.04827669	0.280821
115490116	Gm52771	predicted gene, 52771	NA		-0.76017	0.024194	-1.69369	0.04827789	0.280821
15983	Ifrd2	interferon-related developmental regulator 2	NA		-0.24216	3.113369	-1.18276	0.04828773	0.280821
12367	Casp3	caspase 3, transcript variant X1	NA		0.098095	8.167806	1.070359	0.04830964	0.280848
23831	Car14	carbonic anhydrase 14, transcript variant 1	NA		-0.35133	2.65323	-1.27573	0.04834064	0.280928
18570	Pdcd6	programmed cell death 6, transcript variant 2	NA		0.141937	5.479635	1.103386	0.04836598	0.280975
22230	Ufd1	ubiquitin recognition factor in ER-associated degrada	NA		0.105555	6.181664	1.075908	0.04845483	0.281391
12492	Scarb2	scavenger receptor class B, member 2	NA		0.129078	7.601029	1.093594	0.04854876	0.28174
231380	Uba6	ubiquitin-like modifier activating enzyme 6	NA		-0.16016	4.650387	-1.11741	0.04854952	0.28174
109135	Plekha5	pleckstrin homology domain containing, family A men	NA		0.113617	6.241736	1.081937	0.04858568	0.281775
545428	Ccdc141	coiled-coil domain containing 141, transcript variant X	NA		0.249055	3.264905	1.188428	0.04859026	0.281775
67011	Mettl6	methyltransferase like 6, transcript variant 1	NA		0.169323	4.801841	1.12453	0.04864959	0.282019
77531	Anks1b	ankyrin repeat and sterile alpha motif domain contain	NA		0.129674	6.33181	1.094046	0.04871469	0.282206
15925	Ide	insulin degrading enzyme, transcript variant X2	NA		0.119135	5.610886	1.086083	0.04873269	0.282206
53885	Nphp1	nephronophthisis 1 (juvenile) homolog (human), trans	NA		0.206287	4.201946	1.153715	0.048734	0.282206
70430	Tbce	tubulin-specific chaperone E, transcript variant X11	NA		0.116867	5.95312	1.084377	0.04875847	0.282248
20474	Six4	sine oculis-related homeobox 4, transcript variant 1	NA		-0.53909	1.128974	-1.45306	0.04879868	0.282357
21908	Tlx1	T cell leukemia, homeobox 1	NA		-0.86263	-0.773	-1.81835	0.04881215	0.282357
71361	Aifm2	apoptosis-inducing factor, mitochondrion-associated :	NA		0.268518	2.969249	1.20457	0.04889634	0.282708
69136	Tusc1	tumor suppressor candidate 1	NA		0.319923	3.328609	1.248264	0.04890749	0.282708
381694	B3glct	beta-3-glucosyltransferase	NA		0.135439	5.4884	1.098427	0.04902782	0.283206
212390	Klhl32	kelch-like 32, transcript variant 1	NA		0.205464	4.433644	1.153057	0.04903177	0.283206
213783	Plekhg1	pleckstrin homology domain containing, family G (witl	NA		0.16514	4.397006	1.121275	0.04908222	0.283206
100504323	Gm20172	predicted gene, 20172	NA		-0.76746	-0.27815	-1.70227	0.04909591	0.283206
213742	Xist	inactive X specific transcripts, transcript variant 1	NA		-0.44527	8.451342	-1.36157	0.04911033	0.283206
268878	Atp13a5	ATPase type 13A5, transcript variant 1	NA		0.439657	1.856709	1.356282	0.04911516	0.283206
118568718	LOC118568718	NADH-ubiquinone oxidoreductase chain 5-like	NA		0.504152	0.925912	1.41829	0.04911955	0.283206
70441	Mir124-2hg	Mir124-2 host gene (non-protein coding)	NA		0.190431	3.95514	1.141104	0.04914923	0.283206
399101	Snhg3	small nucleolar RNA host gene 3	NA		0.256617	3.241391	1.194674	0.04915029	0.283206
68936	Smim11	small integral membrane protein 11, transcript variant	NA		-0.21938	3.309273	-1.16423	0.04920292	0.283315
13842	Epha8	Eph receptor A8	NA		-0.1424	4.918795	-1.10374	0.04920397	0.283315
11937	Atp2a1	ATPase, Ca++ transporting, cardiac muscle, fast twitc	NA		-0.5837	1.413964	-1.49869	0.0492399	0.283326
244721	Zfp846	zinc finger protein 846, transcript variant X2	NA		0.124504	5.99116	1.090133	0.04924082	0.283326
17750	Mt2	metallothionein 2	NA		0.373373	2.76823	1.295378	0.04929157	0.283496
59043	Wsb2	WD repeat and SOCS box-containing 2	NA		0.106149	7.724093	1.076351	0.04933272	0.283496
226075	Glis3	GLIS family zinc finger 3, transcript variant X2	NA		0.291388	2.81047	1.223817	0.0493513	0.283496
108013	Celf4	CUGBP, Elav-like family member 4, transcript variant	NA		0.100938	8.759123	1.072471	0.0493562	0.283496
14453	Gas2	growth arrest specific 2, transcript variant 2	NA		0.254466	3.773601	1.192894	0.04937844	0.283496
212943	Tent5a	terminal nucleotidyltransferase 5A, transcript variant :	NA		0.255107	3.605687	1.193424	0.04939126	0.283496
74522	Morc2a	microrchidia 2A, transcript variant 2	NA		0.103113	6.750607	1.074088	0.04940963	0.283496
67045	Riok2	RIO kinase 2	NA		0.139085	5.085889	1.101206	0.04940965	0.283496
235587	Parp3	poly (ADP-ribose) polymerase family, member 3, tran	NA		0.687288	0.166976	1.610254	0.04946387	0.283707
77087	Ankrd11	ankyrin repeat domain 11, transcript variant X1	NA		0.1149	7.531705	1.0829	0.04952085	0.283754
79456	Recql4	RecQ protein-like 4	NA		-0.29367	2.491411	-1.22575	0.04953587	0.283754
53890	Sart3	squamous cell carcinoma antigen recognized by T ce	NA		-0.11366	6.18858	-1.08197	0.04954029	0.283754
16952	Anxa1	annexin A1	NA		-0.45142	3.125115	-1.36739	0.04954482	0.283754
78938	Fbxo34	F-box protein 34, transcript variant X3	NA		0.149977	4.68004	1.109552	0.04955925	0.283754
21376	Tbrg1	transforming growth factor beta regulated gene 1	NA		0.132647	5.791988	1.096303	0.04959633	0.283795
78816	Gmip	Gem-interacting protein	NA		-0.14002	5.957733	-1.10192	0.04961197	0.283795
232889	Pla2g4c	phospholipase A2, group IVC (cytosolic, calcium-inde	NA		-0.53536	1.37005	-1.4493	0.04962983	0.283795
22026	Nr2c2	nuclear receptor subfamily 2, group C, member 2, tra	NA		0.11915	7.211005	1.086094	0.04963627	0.283795
102642321	Gm38604	predicted gene, 38604	NA		-0.30286	2.435216	-1.23358	0.04972133	0.284182
105504	Exoc5	exocyst complex component 5, transcript variant X1	NA		0.13589	6.797719	1.098771	0.0497888	0.284375
66155	Ufc1	ubiquitin-fold modifier conjugating enzyme 1, transcri	NA		0.152256	5.728621	1.111306	0.04979004	0.284375
21917	Tmpo	thymopoietin, transcript variant 7	NA		-0.10933	7.801409	-1.07873	0.04988348	0.284734
22275	Urod	uroporphyrinogen decarboxylase	NA		-0.17314	4.537424	-1.12751	0.0498879	0.284734
26371	Ciao1	cytosolic iron-sulfur protein assembly 1	NA		-0.15804	5.185719	-1.11577	0.04997623	0.285138
77583	Notum	notum palmitoleoyl-protein carboxylesterase, transcri	NA		0.434446	1.146937	1.351391	0.0500063	0.285209
66602	Oip5os1	Opa interacting protein 5, opposite strand 1, transcrip	NA		0.263829	3.889192	1.200661	0.05002839	0.285235

106393	Srl	sarcalumenin, transcript variant X2	NA	-0.24833	5.651973	-1.18783	0.05013456	0.285741
12552	Cdh11	cadherin 11	NA	0.122855	6.589985	1.088887	0.05019471	0.285929
69329	Cfap206	cilia and flagella associated protein 206, transcript va	NA	0.507871	1.687926	1.42195	0.05020273	0.285929
208440	Dip2c	disco interacting protein 2 homolog C	NA	0.119135	6.44313	1.086083	0.05025347	0.286006
100504663	Atg14	autophagy related 14	NA	-0.14795	4.491538	-1.10799	0.05025367	0.286006
67291	Ccdc137	coiled-coil domain containing 137	NA	-0.1669	4.436823	-1.12265	0.05029111	0.286006
67727	Stx17	syntaxin 17, transcript variant X2	NA	0.165961	5.081687	1.121913	0.05029164	0.286006
380977	A330009N23Rik	RIKEN cDNA A330009N23 gene, transcript variant 2	NA	-0.62484	0.114176	-1.54204	0.0503042	0.286006
50907	Preb	prolactin regulatory element binding, transcript varian	NA	-0.1538	6.184784	-1.11249	0.05037684	0.286319
57743	Sec61a2	Sec61, alpha subunit 2 (S. cerevisiae), transcript vari	NA	0.103682	6.379593	1.074513	0.0504068	0.28636
72542	Pgam5	phosphoglycerate mutase family member 5, transcrip	NA	0.120173	6.11415	1.086865	0.05041923	0.28636
16188	Il3ra	interleukin 3 receptor, alpha chain, transcript variant)	NA	0.462756	1.922688	1.378172	0.05049055	0.286569
19744	Rheb	Ras homolog enriched in brain	NA	0.110321	6.618484	1.079469	0.05049128	0.286569
74205	Acsl3	acyl-CoA synthetase long-chain family member 3, tra	NA	0.144763	6.548113	1.105549	0.05053493	0.286717
382253	Cdkl5	cyclin-dependent kinase-like 5	NA	0.501327	1.549836	1.415515	0.05076158	0.287903
19651	Rbl2	RB transcriptional corepressor like 2, transcript variar	NA	0.137289	5.294214	1.099836	0.05080688	0.288059
219149	Xkr6	X-linked Kx blood group related 6, transcript variant X	NA	-0.23435	5.396206	-1.17638	0.05082852	0.288081
20220	Sap18	Sin3-associated polypeptide 18	NA	-0.12918	5.116531	-1.09367	0.05089104	0.288335
102631735	A630039O03Rik	RIKEN cDNA A630039O03 gene, transcript variant X	NA	0.526611	0.87257	1.440541	0.05096376	0.288585
84505	Setdb1	SET domain, bifurcated 1, transcript variant 2	NA	-0.10637	6.664518	-1.07652	0.0509705	0.288585
26419	Mapk8	mitogen-activated protein kinase 8, transcript variant	NA	0.111211	7.425656	1.080134	0.05102357	0.288715
106707	Rpusd1	RNA pseudouridylate synthase domain containing 1,	NA	0.203934	3.750489	1.151835	0.0510343	0.288715
71313	Fsip1	fibrous sheath-interacting protein 1, transcript variant	NA	-0.43543	1.523767	-1.35232	0.05105812	0.288715
208768	Sde2	SDE2 telomere maintenance homolog (S. pombe)	NA	-0.13219	5.195772	-1.09595	0.05106446	0.288715
22224	Usp10	ubiquitin specific peptidase 10, transcript variant 1	NA	-0.11734	6.726301	-1.08473	0.05119613	0.289245
100273	Osbpl9	oxysterol binding protein-like 9, transcript variant X12	NA	0.101132	6.398194	1.072615	0.05122445	0.289245
53618	Fut8	fucosyltransferase 8, transcript variant 1	NA	0.148454	6.155575	1.108381	0.0512318	0.289245
75553	Zc3h14	zinc finger CCCH type containing 14, transcript variar	NA	0.09807	6.931273	1.070341	0.05123289	0.289245
103172	Chchd10	coiled-coil-helix-coiled-coil-helix domain containing 1(	NA	0.248189	3.406669	1.187716	0.05124721	0.289245
76688	Arfrp1	ADP-ribosylation factor related protein 1, transcript va	NA	-0.1426	5.516569	-1.10389	0.05128881	0.289296
66184	Rps4l	ribosomal protein S4-like	NA	0.16575	5.389237	1.121749	0.05129168	0.289296
170740	Zfp287	zinc finger protein 287, transcript variant X15	NA	-0.14398	5.010001	-1.10495	0.05139066	0.289754
17995	Ndufv1	NADH:ubiquinone oxidoreductase core subunit V1	NA	0.109733	6.690267	1.079028	0.05146816	0.29009
67739	Slc48a1	solute carrier family 48 (heme transporter), member 1	NA	0.165686	5.747016	1.121699	0.05151691	0.290264
12982	Csf2ra	colony stimulating factor 2 receptor, alpha, low-affinity	NA	-0.14163	5.710651	-1.10315	0.05157811	0.290472
13830	Stom	stomatin	NA	0.200303	3.998328	1.14894	0.05159607	0.290472
68839	Ankrd46	ankyrin repeat domain 46, transcript variant 3	NA	0.115068	6.987057	1.083026	0.05160726	0.290472
21960	Tnr	tenascin R, transcript variant X10	NA	0.164475	5.654648	1.120758	0.05164862	0.290585
12069	Bex2	brain expressed X-linked 2	NA	0.097221	7.452338	1.069711	0.05166623	0.290585
211652	Wwc1	WW, C2 and coiled-coil domain containing 1, transcri	NA	-0.11818	6.295785	-1.08536	0.05169614	0.290585
16911	Lmo4	LIM domain only 4, transcript variant 3	NA	0.155265	6.950897	1.113626	0.05170721	0.290585
14800	Gria2	glutamate receptor, ionotropic, AMPA2 (alpha 2), trar	NA	0.127833	8.95902	1.092651	0.05171675	0.290585
319748	Zfp865	zinc finger protein 865, transcript variant X3	NA	-0.13193	5.221831	-1.09576	0.05174574	0.290648
14651	Hagh	hydroxyacyl glutathione hydrolase, transcript variant '	NA	0.159556	4.16664	1.116944	0.05180441	0.290866
12904	Crabp2	cellular retinoic acid binding protein II	NA	-0.21484	3.29874	-1.16058	0.05182366	0.290866
118568509	LOC118568509	uncharacterized LOC118568509	NA	0.731126	-0.34579	1.659935	0.05183828	0.290866
67035	Dnajb4	DnaJ heat shock protein family (Hsp40) member B4,	NA	0.185671	5.310514	1.137346	0.0519366	0.291318
243725	Ppp1r9a	protein phosphatase 1, regulatory subunit 9A, transcr	NA	0.115035	6.984633	1.083001	0.05198636	0.291496
67194	2700038G22Rik	RIKEN cDNA 2700038G22 gene, transcript variant 1	NA	-0.3224	2.272218	-1.25041	0.05203172	0.291591
12488	Cd2ap	CD2-associated protein	NA	0.143343	5.384003	1.104461	0.05204201	0.291591
71885	Faap100	Fanconi anemia core complex associated protein 10(	NA	-0.14524	5.744108	-1.10592	0.05208592	0.291591
214230	Pak6	p21 (RAC1) activated kinase 6, transcript variant 1	NA	-0.10129	6.460153	-1.07274	0.05209106	0.291591
51886	Fubp1	far upstream element (FUSE) binding protein 1, trans	NA	-0.12317	8.163767	-1.08913	0.05209322	0.291591
102640125	Gm36267	predicted gene, 36267, transcript variant X3	NA	0.731068	-0.57465	1.659867	0.05213657	0.291591
20964	Syn1	synapsin I, transcript variant b	NA	-0.09326	7.329229	-1.06678	0.05214071	0.291591
78933	Agbl4	ATP/GTP binding protein-like 4, transcript variant 3	NA	0.356062	1.638195	1.279927	0.05214661	0.291591
381022	Kmt2d	lysine (K)-specific methyltransferase 2D, transcript va	NA	-0.1088	7.471366	-1.07833	0.05222815	0.291864
104248	Cabin1	calcineurin binding protein 1, transcript variant X7	NA	-0.12768	6.42735	-1.09254	0.05223137	0.291864
225339	Ammecr1l	AMME chromosomal region gene 1-like, transcript va	NA	-0.11156	6.317114	-1.0804	0.05234267	0.292383
14860	Gsta4	glutathione S-transferase, alpha 4	NA	0.222186	3.090903	1.1665	0.05236189	0.292383
20679	Sox6	SRY (sex determining region Y)-box 6, transcript vari	NA	-0.14091	5.592677	-1.1026	0.05237962	0.292383
234413	Zfp961	zinc finger protein 961	NA	-0.21029	4.331978	-1.15692	0.05239604	0.292383
66105	Ube2d3	ubiquitin-conjugating enzyme E2D 3, transcript variar	NA	0.098961	8.381642	1.071002	0.05244798	0.2925
18027	Nfia	nuclear factor I/A, transcript variant 1	NA	-0.09989	7.694154	-1.07169	0.05245294	0.2925
117197	Bloc1s4	biogenesis of lysosomal organelles complex-1, subur	NA	0.222564	3.180962	1.166805	0.05248146	0.292558
329178	Unc80	unc-80, NALCN activator, transcript variant X23	NA	0.18752	4.586617	1.138804	0.05251579	0.292593
230393	Focad	focadhesin, transcript variant X5	NA	0.138553	4.756073	1.100801	0.05252911	0.292593
71706	Slc46a3	solute carrier family 46, member 3, transcript variant :	NA	0.348616	1.604475	1.273339	0.05255956	0.292593

18005	Nek2	NIMA (never in mitosis gene a)-related expressed kin	NA	-0.22451	3.568149	-1.16838	0.05258146	0.292593
56637	Gsk3b	glycogen synthase kinase 3 beta, transcript variant X	NA	0.094779	8.268446	1.067902	0.05259244	0.292593
83603	Elovl4	elongation of very long chain fatty acids (FEN1/Elo2,	NA	0.117938	6.361762	1.085183	0.0525955	0.292593
67252	Cap2	CAP, adenylate cyclase-associated protein, 2 (yeast)	NA	-0.20393	4.478063	-1.15183	0.0526348	0.292711
223739	5031439G07Rik	RIKEN cDNA 5031439G07 gene, transcript variant X	NA	-0.11224	7.723457	-1.08091	0.05265506	0.292724
14571	Gpd2	glycerol phosphate dehydrogenase 2, mitochondrial,	NA	0.144805	5.373398	1.105581	0.05269852	0.292765
319278	Shfl	shiftless antiviral inhibitor of ribosomal frameshifting,	NA	0.290911	2.684084	1.223412	0.05271583	0.292765
66056	Zfp524	zinc finger protein 524, transcript variant X1	NA	-0.34633	2.055053	-1.27132	0.05271646	0.292765
107515	Lgr4	leucine-rich repeat-containing G protein-coupled rece	NA	0.239414	4.874697	1.180513	0.05276162	0.292916
110109	Nop2	NOP2 nucleolar protein	NA	-0.13294	5.417887	-1.09652	0.05278315	0.292936
115488374	Gm52150	predicted gene, 52150	NA	-0.3489	1.667121	-1.27359	0.05282859	0.293088
93887	Pcdhb16	protocadherin beta 16	NA	0.345844	2.016242	1.270895	0.05291171	0.293137
18563	Pcx	pyruvate carboxylase, transcript variant 1	NA	-0.14289	5.177372	-1.10411	0.05292395	0.293137
225876	Kdm2a	lysine (K)-specific demethylase 2A, transcript variant	NA	-0.13057	6.81144	-1.09473	0.05292635	0.293137
11674	Aldoa	aldolase A, fructose-bisphosphate, transcript variant 2	NA	0.117478	8.230193	1.084837	0.0529311	0.293137
19663	Rbpms	RNA binding protein gene with multiple splicing, trans	NA	0.259525	2.866896	1.197084	0.05293297	0.293137
78593	Nrip3	nuclear receptor interacting protein 3	NA	0.197181	3.652713	1.146456	0.05295205	0.293137
115488373	LOC115488373	uncharacterized LOC115488373	NA	0.36429	2.125571	1.287248	0.05296359	0.293137
268933	Wdr24	WD repeat domain 24	NA	-0.16983	4.573578	-1.12493	0.05305559	0.293547
53602	Hpcal1	hippocalcin-like 1, transcript variant 1	NA	0.13585	5.522321	1.09874	0.05308977	0.293636
56348	Hsd17b12	hydroxysteroid (17-beta) dehydrogenase 12	NA	0.098162	6.569791	1.070409	0.05311109	0.293654
74239	Iqce	IQ motif containing E, transcript variant 1	NA	-0.13917	5.262919	-1.10127	0.05313938	0.293685
118567859	LOC118567859	uncharacterized LOC118567859	NA	0.572078	0.214241	1.486664	0.05315562	0.293685
20465	Sim2	single-minded family bHLH transcription factor 2	NA	-0.61577	-0.12279	-1.53238	0.05317077	0.293685
98956	Nat10	N-acetyltransferase 10	NA	-0.14795	5.184434	-1.10799	0.05320996	0.293801
67972	Atp2b1	ATPase, Ca++ transporting, plasma membrane 1, tra	NA	0.12987	7.58681	1.094195	0.05323831	0.293858
69904	2610027F03Rik	RIKEN cDNA 2610027F03 gene, transcript variant X	NA	-0.66719	0.618951	-1.58797	0.05325767	0.293865
58202	Nelfb	negative elongation factor complex member B, transc	NA	-0.12531	5.916967	-1.09074	0.053328	0.293983
209645	Bend7	BEN domain containing 7, transcript variant X3	NA	0.287371	2.674841	1.220414	0.05335088	0.293983
353170	Txlng	taxilin gamma, transcript variant X1	NA	0.189942	4.47663	1.140718	0.05335257	0.293983
320237	Smim10l2a	small integral membrane protein 10 like 2A	NA	0.164262	4.895143	1.120593	0.05335933	0.293983
14735	Gpc4	glypican 4, transcript variant X1	NA	-0.13579	5.079458	-1.09869	0.05336937	0.293983
20181	Rxra	retinoid X receptor alpha, transcript variant X1	NA	-0.14398	4.802602	-1.10495	0.05349734	0.29454
66083	Setd6	SET domain containing 6, transcript variant 2	NA	0.208256	3.976527	1.155291	0.05350657	0.29454
16485	Kcna1	potassium voltage-gated channel, shaker-related sub	NA	0.232933	3.313868	1.175222	0.05354403	0.29455
225467	Pggt1b	protein geranylgeranyltransferase type I, beta subuni	NA	0.12718	5.488843	1.092157	0.05354819	0.29455
72575	C430049B03Rik	RIKEN cDNA C430049B03 gene, transcript variant 1	NA	-0.34683	1.968222	-1.27177	0.05356275	0.29455
56736	Rnf14	ring finger protein 14, transcript variant 2	NA	0.106525	7.787542	1.076632	0.0536025	0.294572
73699	Ppp2r1b	protein phosphatase 2, regulatory subunit A, beta, tra	NA	-0.13622	5.800579	-1.09902	0.05360875	0.294572
29876	Clic4	chloride intracellular channel 4 (mitochondrial)	NA	-0.13109	6.923987	-1.09512	0.05364235	0.294572
100503000	Zfp993	zinc finger protein 993	NA	0.939888	-0.13396	1.918379	0.05367045	0.294572
15284	Hlx	H2.0-like homeobox	NA	-0.58844	0.663531	-1.50362	0.05367515	0.294572
67607	Zfp788	zinc finger protein 788, transcript variant 6	NA	0.118859	5.590388	1.085876	0.05367537	0.294572
21788	Tfpi	tissue factor pathway inhibitor, transcript variant 4	NA	0.180906	3.981662	1.133596	0.05372697	0.294756
12447	Ccne1	cyclin E1	NA	-0.29233	3.194771	-1.22462	0.05376429	0.294766
102639645	Gm534	predicted gene 534, transcript variant X5	NA	0.63369	0.405403	1.551529	0.05376505	0.294766
269604	Gpr157	G protein-coupled receptor 157, transcript variant X1	NA	0.380715	1.665688	1.301987	0.05380001	0.29478
72693	Zcchc12	zinc finger, CCHC domain containing 12, transcript va	NA	0.131365	7.913915	1.095329	0.05384465	0.29478
210162	Zkscan2	zinc finger with KRAB and SCAN domains 2, transcri	NA	0.154011	6.189616	1.112659	0.05384481	0.29478
171508	Creld1	cysteine-rich with EGF-like domains 1, transcript vari	NA	-0.15618	5.259368	-1.11433	0.0538481	0.29478
97130	C77080	expressed sequence C77080, transcript variant 4	NA	-0.24646	4.571045	-1.18629	0.05385825	0.29478
67050	Nkap	NFKB activating protein	NA	0.133313	5.574526	1.096809	0.05387992	0.294787
216151	Polrmt	polymerase (RNA) mitochondrial (DNA directed)	NA	-0.14113	4.721958	-1.10277	0.05392383	0.294787
93882	Pcdhb11	protocadherin beta 11	NA	0.33104	1.876691	1.25792	0.05393114	0.294787
69871	Ppp1r35	protein phosphatase 1, regulatory subunit 35, transcr	NA	-0.19445	3.612835	-1.14429	0.05393204	0.294787
53421	Sec61a1	Sec61 alpha 1 subunit (S. cerevisiae)	NA	-0.10959	7.161461	-1.07892	0.05396235	0.294792
641376	Tomm40l	translocase of outer mitochondrial membrane 40-like	NA	-0.14302	5.152313	-1.10421	0.05396914	0.294792
210172	Zfp526	zinc finger protein 526, transcript variant X3	NA	-0.25102	3.912745	-1.19005	0.05400474	0.294888
70239	Gtf3c5	general transcription factor IIIC, polypeptide 5, transc	NA	-0.15342	5.371034	-1.1122	0.0540381	0.294971
100503043	Armcx4	armadillo repeat containing, X-linked 4, transcript vari	NA	0.123648	7.325523	1.089486	0.05407132	0.295053
74325	Cltb	clathrin, light polypeptide (Lcb), transcript variant 1	NA	0.105067	6.486742	1.075545	0.0541063	0.295145
109264	Me3	malic enzyme 3, NADP(+)-dependent, mitochondrial,	NA	0.235909	3.345863	1.177649	0.05420306	0.295574
13019	Ctf1	cardiotrophin 1, transcript variant 2	NA	-0.4098	1.177073	-1.3285	0.05423978	0.295675
66734	Map1lc3a	microtubule-associated protein 1 light chain 3 alpha,	NA	0.131419	6.537138	1.095371	0.05435131	0.296089
19355	Rad1	RAD1 checkpoint DNA exonuclease, transcript variar	NA	-0.18068	3.927127	-1.13342	0.05436672	0.296089
20733	Spint2	serine protease inhibitor, Kunitz type 2, transcript vari	NA	0.199545	4.797641	1.148336	0.05437032	0.296089
102636055	Gm38486	predicted gene, 38486, transcript variant X4	NA	0.526439	0.818881	1.440369	0.05439985	0.296151
56703	Pigo	phosphatidylinositol glycan anchor biosynthesis, clas	NA	-0.15974	4.724948	-1.11709	0.05442219	0.296173

68735	Mrps18c	mitochondrial ribosomal protein S18C	NA		0.169423	4.368099	1.124608	0.05444949	0.296223
11722	Amy1	amylase 1, salivary, transcript variant 1	NA		0.237835	4.470443	1.179222	0.05447632	0.29627
68794	Flnc	filamin C, gamma, transcript variant 2	NA		-0.18202	4.829252	-1.13447	0.05451915	0.296403
52276	Cdca8	cell division cycle associated 8	NA		-0.16282	4.51507	-1.11947	0.05457342	0.296592
75552	Paqr9	progestin and adipoQ receptor family member IX	NA		0.164925	4.89683	1.121108	0.0545902	0.296592
23792	Adam23	a disintegrin and metallopeptidase domain 23, transc	NA		0.165158	5.821491	1.121288	0.05465612	0.296601
72193	Scaf11	SR-related CTD-associated factor 11, transcript varia	NA		0.127702	6.236315	1.092552	0.05465653	0.296601
67425	Eps8l1	EPS8-like 1, transcript variant X8	NA		-0.33866	2.22395	-1.26458	0.05467318	0.296601
75565	Sgf29	SAGA complex associated factor 29	NA		0.184994	4.032755	1.136812	0.05469575	0.296601
18754	Prkce	protein kinase C, epsilon	NA		0.094305	7.567459	1.067551	0.05471891	0.296601
66917	Chordc1	cysteine and histidine-rich domain (CHORD)-containi	NA		-0.1189	5.767757	-1.08591	0.05472382	0.296601
27801	Zdhhc8	zinc finger, DHHC domain containing 8, transcript var	NA		-0.13679	5.834576	-1.09946	0.05472517	0.296601
21425	Tfeb	transcription factor EB, transcript variant X3	NA		-0.41415	1.36616	-1.33251	0.05475096	0.296601
13837	Epha3	Eph receptor A3, transcript variant 1	NA		-0.16936	6.042207	-1.12456	0.05475594	0.296601
58200	Ppp1r1a	protein phosphatase 1, regulatory inhibitor subunit 1A	NA		0.1265	5.583248	1.091642	0.05481645	0.29683
192185	Nadk	NAD kinase, transcript variant 2	NA		-0.12359	6.303467	-1.08945	0.05489809	0.297173
20513	Slc1a6	solute carrier family 1 (high affinity aspartate/glutama	NA		0.211367	3.746034	1.157785	0.05494372	0.297321
216860	Neurl4	neuralized E3 ubiquitin protein ligase 4, transcript var	NA		-0.11069	7.274976	-1.07975	0.05498507	0.297446
229363	Gmps	guanine monophosphate synthetase, transcript variar	NA		0.128262	6.738258	1.092976	0.05500621	0.297461
29871	Scmh1	sex comb on midleg homolog 1, transcript variant 3	NA		-0.13614	6.234686	-1.09896	0.05511065	0.297623
16400	Itga3	integrin alpha 3, transcript variant 2	NA		-0.15268	4.963516	-1.11163	0.05511976	0.297623
216964	Trp53i13	transformation related protein 53 inducible protein 13	NA		-0.29565	2.300219	-1.22744	0.0551316	0.297623
327814	Ppfia2	protein tyrosine phosphatase, receptor type, f polype	NA		0.110744	6.798652	1.079785	0.05514593	0.297623
94184	Pdxdc1	pyridoxal-dependent decarboxylase domain containir	NA		0.102775	6.420814	1.073837	0.05515447	0.297623
320191	Hook3	hook microtubule tethering protein 3	NA		0.116557	6.549955	1.084144	0.05515857	0.297623
20682	Sox9	SRY (sex determining region Y)-box 9	NA		-0.11692	5.940246	-1.08442	0.05516422	0.297623
20652	Soat1	sterol O-acyltransferase 1	NA		-0.21428	3.524292	-1.16013	0.05521092	0.297777
213753	Zfp598	zinc finger protein 598, transcript variant X1	NA		-0.11825	5.851848	-1.08541	0.05526956	0.297907
19376	Rab34	RAB34, member RAS oncogene family, transcript var	NA		0.145348	5.064896	1.105997	0.05528828	0.297907
102371	Myzap	myocardial zonula adherens protein	NA		-0.39833	1.217945	-1.31799	0.05528998	0.297907
69568	Vkorc1l1	vitamin K epoxide reductase complex, subunit 1-like	NA		0.099572	6.669329	1.071455	0.05536407	0.298207
93871	Brwd1	bromodomain and WD repeat domain containing 1, tr	NA		-0.09517	7.592372	-1.06819	0.05540699	0.298293
100502861	Ccdc13	coiled-coil domain containing 13, transcript variant X2	NA		-0.40701	1.727318	-1.32593	0.05541665	0.298293
69693	Pof1b	premature ovarian failure 1B	NA		-1.1011	-1.12521	-2.14518	0.05548966	0.298587
12740	Cldn4	claudin 4	NA		-0.9299	-0.59477	-1.90514	0.05556236	0.298804
71853	Pdia6	protein disulfide isomerase associated 6	NA		-0.10387	7.542683	-1.07466	0.05559907	0.298804
232791	Cnot3	CCR4-NOT transcription complex, subunit 3	NA		-0.10373	6.474388	-1.07455	0.05560462	0.298804
11983	Atpif1	ATPase inhibitory factor 1, transcript variant X2	NA		0.099505	7.114164	1.071406	0.05561118	0.298804
208820	Triqk	triple QxxK/R motif containing, transcript variant 2	NA		0.219208	3.34353	1.164094	0.05562184	0.298804
105844	Card10	caspase recruitment domain family, member 10	NA		-0.29945	2.724782	-1.23067	0.05567271	0.298979
110172	Slc35b1	solute carrier family 35, member B1, transcript varian	NA		0.12094	5.69769	1.087443	0.0557476	0.299112
78912	Sp2	Sp2 transcription factor, transcript variant 2	NA		-0.16552	4.509116	-1.12157	0.05576461	0.299112
207615	Wdr37	WD repeat domain 37, transcript variant X3	NA		0.146971	6.323036	1.107242	0.05577062	0.299112
380654	Cfap54	cilia and flagella associated protein 54, transcript vari	NA		0.314348	2.232223	1.24345	0.05579698	0.299112
14526	Gcg	glucagon	NA		-0.62595	0.027629	-1.54323	0.05579792	0.299112
26938	St6galnac5	ST6 (alpha-N-acetyl-neuraminyl-2,3-beta-galactosyl-1	NA		0.200763	4.338271	1.149306	0.05580785	0.299112
211623	Plac9a	placenta specific 9a	NA		0.367921	1.71085	1.290492	0.05584364	0.299206
214855	Arid5a	AT rich interactive domain 5A (MRF1-like), transcript	NA		-0.40891	1.559978	-1.32768	0.05594739	0.299628
74277	Chic2	cysteine-rich hydrophobic domain 2	NA		0.145085	4.737103	1.105796	0.05600895	0.299628
665578	Gm7697	predicted gene 7697, transcript variant X3	NA		-0.82508	-0.2568	-1.77163	0.05603871	0.299628
102631665	Gm29948	predicted gene, 29948	NA		0.362897	1.463668	1.286006	0.05607032	0.299628
74186	Ccdc3	coiled-coil domain containing 3	NA		0.197217	3.507186	1.146485	0.05607361	0.299628
70227	Zfp619	zinc finger protein 619	NA		-0.23811	2.863073	-1.17944	0.05608021	0.299628
71373	Prr16	proline rich 16	NA		0.371774	1.849468	1.293943	0.05609115	0.299628
338467	Morc3	microrchidia 3	NA		0.139722	5.343397	1.101692	0.05609734	0.299628
50908	C1s1	complement component 1, s subcomponent 1, transc	NA		0.492738	0.52133	1.407113	0.05609864	0.299628
18777	Lypla1	lysophospholipase 1, transcript variant 1	NA		-0.15765	5.060342	-1.11547	0.05610833	0.299628
353236	Pcdhac1	protocadherin alpha subfamily C, 1	NA		0.55847	2.764735	1.472706	0.05614111	0.299628
12332	Capg	capping protein (actin filament), gelsolin-like, transcri	NA		0.436481	1.585517	1.3533	0.05614917	0.299628
69585	Hjv	hemojuvelin BMP co-receptor	NA		-0.52052	0.91614	-1.43448	0.0561619	0.299628
57912	Cdc42se1	CDC42 small effector 1, transcript variant 2	NA		-0.1206	6.11005	-1.08719	0.05621473	0.299717
76498	Paqr4	progestin and adipoQ receptor family member IV	NA		-0.16642	5.009251	-1.12227	0.05622585	0.299717
109006	Ciapin1	cytokine induced apoptosis inhibitor 1, transcript varia	NA		-0.12787	5.902473	-1.09268	0.05627651	0.299717
16971	Lrp1	low density lipoprotein receptor-related protein 1	NA		-0.0927	8.415179	-1.06637	0.05628938	0.299717
66611	Ribc1	RIB43A domain with coiled-coils 1	NA		-0.557	0.114338	-1.47121	0.05631067	0.299717
59004	Pias4	protein inhibitor of activated STAT 4	NA		-0.15481	4.604035	-1.11328	0.05634653	0.299717
74157	Cmtr1	cap methyltransferase 1	NA		-0.10953	6.327688	-1.07888	0.0563548	0.299717
76260	Ttc8	tetratricopeptide repeat domain 8, transcript variant 2	NA		-0.15669	4.380948	-1.11473	0.05637019	0.299717

68094	Smarcc2	SWI/SNF related, matrix associated, actin dependent	NA		-0.09673	8.984902	-1.06935	0.05637145	0.299717
78887	Sfi1	Sfi1 homolog, spindle assembly associated (yeast), t	NA		-0.18949	4.364602	-1.14036	0.05638408	0.299717
233073	U2af1l4	U2 small nuclear RNA auxiliary factor 1-like 4	NA		-0.1831	4.26418	-1.13532	0.05639595	0.299717
108655	Foxp1	forkhead box P1, transcript variant 3	NA		0.097406	7.069292	1.069848	0.05639964	0.299717
66383	Iscu	iron-sulfur cluster assembly enzyme, transcript variar	NA		0.158488	4.411047	1.116117	0.05648468	0.300039
18164	Nptx1	neuronal pentraxin 1	NA		-0.15109	4.826809	-1.11041	0.05649696	0.300039
14396	Gabra3	gamma-aminobutyric acid (GABA) A receptor, subuni	NA		0.114345	6.294573	1.082484	0.05652094	0.300068
64095	Gpr35	G protein-coupled receptor 35, transcript variant X2	NA		-0.3044	2.249833	-1.23491	0.05654499	0.300098
22241	Ulk1	unc-51 like kinase 1, transcript variant 2	NA		-0.09273	7.170381	-1.06639	0.05662314	0.300415
226154	Lzts2	leucine zipper, putative tumor suppressor 2, transcrip	NA		-0.13623	5.065787	-1.09903	0.05667625	0.300598
66314	Tpd52l2	tumor protein D52-like 2, transcript variant X1	NA		0.112087	5.831166	1.080791	0.05671788	0.300651
70153	2210016F16Rik	RIKEN cDNA 2210016F16 gene	NA		-0.19447	3.814876	-1.14431	0.05672329	0.300651
16538	Kcns1	K+ voltage-gated channel, subfamily S, 1	NA		0.422932	1.322109	1.340649	0.05674167	0.300651
20480	Clpb	ClpB caseinolytic peptidase B, transcript variant 2	NA		-0.12929	5.288807	-1.09375	0.05687034	0.301178
54170	Rragc	Ras-related GTP binding C	NA		0.11705	5.95457	1.084515	0.05687814	0.301178
108707	Fam207a	family with sequence similarity 207, member A	NA		-0.13414	5.176492	-1.09744	0.05693812	0.301398
66406	Sac3d1	SAC3 domain containing 1	NA		0.192825	3.725726	1.143	0.05701859	0.301726
171207	Arhgap4	Rho GTPase activating protein 4, transcript variant 3	NA		-0.39044	1.675009	-1.31079	0.0571234	0.302114
20255	Scg3	secretogranin III, transcript variant 2	NA		0.092133	7.4992	1.065945	0.05712911	0.302114
223254	Farp1	FERM, RhoGEF (Arhgef) and pleckstrin domain prote	NA		0.095198	7.214655	1.068212	0.05721861	0.302292
68558	Ankra2	ankyrin repeat, family A (RFXANK-like), 2, transcript	NA		0.139226	6.17803	1.101314	0.05722037	0.302292
74356	4931428F04Rik	RIKEN cDNA 4931428F04 gene, transcript variant 2	NA		-0.08785	7.858772	-1.06279	0.05723883	0.302292
55981	Pigb	phosphatidylinositol glycan anchor biosynthesis, clas	NA		0.185672	3.826251	1.137347	0.05725289	0.302292
320213	Senp5	SUMO/sentrin specific peptidase 5, transcript variant	NA		0.119207	6.159754	1.086137	0.05725569	0.302292
108832	Tmem74b	transmembrane protein 74B, transcript variant X2	NA		0.149286	5.804942	1.109021	0.05728877	0.302369
75751	Ipo4	importin 4, transcript variant 1	NA		-0.10315	6.37103	-1.07411	0.05732558	0.302385
118567533	LOC118567533	uncharacterized LOC118567533	NA		-0.45271	2.258258	-1.36861	0.05732894	0.302385
18044	Nfya	nuclear transcription factor-Y alpha, transcript variant	NA		-0.18656	6.239517	-1.13805	0.05740256	0.302675
238024	Fn3krp	fructosamine 3 kinase related protein, transcript varia	NA		-0.16895	4.473247	-1.12424	0.05746308	0.302834
14783	Grb10	growth factor receptor bound protein 10, transcript va	NA		-0.09459	7.587624	-1.06776	0.05747059	0.302834
231712	Trafd1	TRAF type zinc finger domain containing 1, transcript	NA		-0.11694	6.510552	-1.08443	0.05750597	0.302834
20335	Sec61g	SEC61, gamma subunit, transcript variant 1	NA		0.140769	5.989356	1.102493	0.05752805	0.302834
72388	Ripk4	receptor-interacting serine-threonine kinase 4	NA		-0.55235	0.564378	-1.46648	0.05752967	0.302834
239719	Mrtfb	myocardin related transcription factor B, transcript va	NA		0.136699	5.376529	1.099386	0.05754432	0.302834
14544	Gda	guanine deaminase, transcript variant X2	NA		0.23686	3.505192	1.178425	0.05756922	0.302867
235682	Zfp445	zinc finger protein 445, transcript variant 1	NA		0.110911	7.718964	1.07991	0.05760678	0.302966
75580	Zbtb4	zinc finger and BTB domain containing 4, transcript v	NA		0.146988	5.205367	1.107255	0.05767691	0.303174
208994	Fam83b	family with sequence similarity 83, member B	NA		-0.89741	-0.87721	-1.86271	0.05769593	0.303174
320642	A630066F11Rik	RIKEN cDNA A630066F11 gene	NA		0.604049	0.598536	1.519977	0.05773019	0.303174
53332	Mtmr1	myotubularin related protein 1, transcript variant X7	NA		0.140606	5.422701	1.102368	0.0577514	0.303174
15204	Herc2	HECT and RLD domain containing E3 ubiquitin prote	NA		0.129903	7.462436	1.09422	0.05781417	0.303174
228140	Tnks1bp1	tankyrase 1 binding protein 1	NA		-0.11897	6.150327	-1.08596	0.05782757	0.303174
269585	Zscan20	zinc finger and SCAN domains 20, transcript variant)	NA		-0.23098	3.707744	-1.17363	0.05782917	0.303174
118567337	LOC118567337	uncharacterized LOC118567337, transcript variant X	NA		0.712976	-0.41029	1.639181	0.05784753	0.303174
433466	Jmjd7	jumonji domain containing 7	NA		0.325077	2.720237	1.252731	0.05784805	0.303174
664862	Gpr137b-ps	G protein-coupled receptor 137B, pseudogene	NA		0.15833	4.362616	1.115995	0.05786276	0.303174
22352	Vim	vimentin	NA		-0.09206	8.721589	-1.06589	0.05787663	0.303174
68917	Hint2	histidine triad nucleotide binding protein 2	NA		0.27448	2.614898	1.209558	0.05787924	0.303174
223455	Marchf6	membrane associated ring-CH-type finger 6	NA		0.115133	8.308973	1.083075	0.05788856	0.303174
224674	Slc37a1	solute carrier family 37 (glycerol-3-phosphate transpo	NA		0.218126	3.532686	1.163221	0.0579082	0.30318
18073	Nid1	nidogen 1	NA		0.09893	6.738928	1.070979	0.05798513	0.303485
227157	Mpp4	membrane protein, palmitoylated 4 (MAGUK p55 sub	NA		-0.56661	0.683441	-1.48104	0.05800939	0.303514
217869	Eif5	eukaryotic translation initiation factor 5, transcript vari	NA		0.111345	8.285273	1.080234	0.0581076	0.303803
12386	Ctnna2	catenin (cadherin associated protein), alpha 2, transc	NA		0.088552	7.977512	1.063303	0.05811213	0.303803
330096	Shisa3	shisa family member 3	NA		-0.24494	3.530854	-1.18504	0.05812889	0.303803
101055840	Gm29729	predicted gene, 29729	NA		0.279639	2.406509	1.213891	0.05813935	0.303803
72155	Cenpn	centromere protein N	NA		-0.24819	3.046788	-1.18772	0.05823965	0.304129
246103	Atxn7	ataxin 7, transcript variant X6	NA		0.197975	4.708846	1.147087	0.05825061	0.304129
240427	Setbp1	SET binding protein 1	NA		0.103657	7.185818	1.074494	0.0583288	0.304129
11440	Chrna6	cholinergic receptor, nicotinic, alpha polypeptide 6	NA		0.448415	0.84127	1.36454	0.05833957	0.304129
268936	Brpf3	bromodomain and PHD finger containing, 3, transcrip	NA		-0.1441	5.346039	-1.10504	0.05835238	0.304129
73368	Col20a1	collagen, type XX, alpha 1, transcript variant X1	NA		-0.27548	2.392547	-1.2104	0.05835326	0.304129
277854	Depdc5	DEP domain containing 5, transcript variant 3	NA		-0.13981	5.408434	-1.10176	0.05835386	0.304129
319504	Nrcam	neuronal cell adhesion molecule, transcript variant X4	NA		0.104836	7.901226	1.075372	0.05835766	0.304129
224792	Adgrf5	adhesion G protein-coupled receptor F5, transcript va	NA		0.144536	4.597646	1.105375	0.05838792	0.304129
243911	Kirrel2	kirre like nephrin family adhesion molecule 2, transcri	NA		-0.51105	0.731408	-1.42508	0.0583886	0.304129
231151	Tada2b	transcriptional adaptor 2B, transcript variant X2	NA		0.140139	5.250913	1.102011	0.05843696	0.304284
11513	Adcy7	adenylate cyclase 7, transcript variant 4	NA		0.31598	2.891525	1.244857	0.05854344	0.304741

319909	Ism1	isthmin 1, angiogenesis inhibitor	NA	-0.41124	1.05148	-1.32983	0.05857009	0.304782
80718	Rab27b	RAB27B, member RAS oncogene family, transcript v	NA	0.245908	3.285035	1.185839	0.05859967	0.304838
68957	Paqr6	progestin and adipoQ receptor family member VI, tra	NA	0.722736	-0.4323	1.650308	0.05865288	0.304968
17217	Mcm4	minichromosome maintenance complex component 4	NA	-0.17256	5.415218	-1.12705	0.05866387	0.304968
78920	Dlst	dihydrolipoamide S-succinyltransferase (E2 compone	NA	-0.1036	7.203013	-1.07445	0.05868088	0.304968
26965	Cul1	cullin 1, transcript variant X2	NA	0.090742	7.310607	1.064918	0.05870045	0.304972
240514	Ccdc85b	coiled-coil domain containing 85B, transcript variant 1	NA	0.161159	4.694081	1.118185	0.05886651	0.305738
67897	Rnmt	RNA (guanine-7-) methyltransferase, transcript varia	NA	0.111405	6.417638	1.08028	0.05891129	0.305873
20932	Surf4	surfeit gene 4	NA	-0.1008	6.767924	-1.07237	0.05894809	0.305905
100715	Tent2	terminal nucleotidyltransferase 2, transcript variant 1	NA	0.123653	5.618554	1.08949	0.05900323	0.305905
18515	Pbx2	pre B cell leukemia homeobox 2	NA	-0.10744	6.417837	-1.07732	0.05901057	0.305905
269261	Rpl12	ribosomal protein L12	NA	0.096765	8.321388	1.069373	0.05901239	0.305905
70363	Fam135b	family with sequence similarity 135, member B, trans	NA	0.181596	3.916687	1.134138	0.05902405	0.305905
67111	Naaa	N-acylethanolamine acid amidase, transcript variant	NA	0.200452	3.623945	1.149058	0.05903037	0.305905
106585	Ankrd12	ankyrin repeat domain 12, transcript variant X12	NA	0.103705	7.530503	1.07453	0.05913187	0.306297
672553	Gm9567	predicted gene 9567, transcript variant X3	NA	-0.36418	2.670195	-1.28715	0.05914365	0.306297
71389	Chd6	chromodomain helicase DNA binding protein 6, trans	NA	0.107556	6.855883	1.077401	0.05918793	0.306355
115487955	Gm51961	predicted gene, 51961	NA	0.389229	1.424095	1.309693	0.05919257	0.306355
102632137	Gm30290	predicted gene, 30290, transcript variant X2	NA	-0.74046	0.172372	-1.67071	0.05921638	0.306381
12445	Ccnd3	cyclin D3, transcript variant 1	NA	-0.12067	5.463142	-1.08724	0.05923824	0.306397
216152	Plppr3	phospholipid phosphatase related 3, transcript varian	NA	-0.0865	8.517389	-1.06179	0.05930757	0.306633
19025	Ctsa	cathepsin A, transcript variant X1	NA	-0.10949	5.96553	-1.07884	0.05933177	0.306633
56431	Dstn	destrin	NA	0.101326	8.394841	1.072759	0.05934051	0.306633
20024	Sub1	SUB1 homolog, transcriptional regulator, transcript va	NA	0.094626	7.752197	1.067788	0.05936277	0.306651
329160	9130024F11Rik	RIKEN cDNA 9130024F11 gene, transcript variant 2	NA	0.21179	5.128008	1.158124	0.05938359	0.306661
15547	Trmt2a	TRM2 tRNA methyltransferase 2A, transcript variant	NA	-0.13084	5.589517	-1.09493	0.0595031	0.307089
71732	Vps11	VPS11, CORVET/HOPS core subunit, transcript varia	NA	0.124206	5.613811	1.089908	0.05951604	0.307089
241308	Ralgps1	Ral GEF with PH domain and SH3 binding motif 1, tra	NA	0.102848	7.119044	1.073891	0.05952309	0.307089
219158	Ccar2	cell cycle activator and apoptosis regulator 2	NA	-0.13203	7.289858	-1.09583	0.05959019	0.307154
140559	Igsf8	immunoglobulin superfamily, member 8	NA	-0.12437	6.064612	-1.09003	0.05960352	0.307154
73162	Otud3	OTU domain containing 3, transcript variant 2	NA	-0.14633	4.50696	-1.10675	0.05960755	0.307154
100041678	Gm3500	predicted gene 3500	NA	0.327359	2.614573	1.254714	0.05961105	0.307154
66589	Ube2v1	ubiquitin-conjugating enzyme E2 variant 1, transcript	NA	0.086476	7.648908	1.061773	0.05963925	0.307197
18032	Nfix	nuclear factor I/X, transcript variant 12	NA	-0.1347	9.208435	-1.09786	0.05965718	0.307197
105841	Dennd3	DENN/MADD domain containing 3, transcript variant	NA	0.335895	2.51715	1.26216	0.05970796	0.307361
13405	Dmd	dystrophin, muscular dystrophy, transcript variant 4	NA	0.134857	5.051426	1.097984	0.0597407	0.307366
102638882	Gm35339	predicted gene, 35339	NA	-0.29411	2.643706	-1.22613	0.05974667	0.307366
115486930	Gm51571	predicted gene, 51571	NA	0.871881	-0.01412	1.830048	0.05981506	0.307506
108902	B4gat1	beta-1,4-glucuronyltransferase 1	NA	0.143381	5.517012	1.104491	0.05982805	0.307506
94220	Cnnm4	cyclin M4	NA	-0.18763	3.633173	-1.13889	0.05983064	0.307506
67467	Gpalpp1	GPALPP motifs containing 1	NA	0.118081	5.502998	1.08529	0.0598693	0.307608
77288	9430021M05Rik	RIKEN cDNA 9430021M05 gene	NA	0.299003	2.669933	1.230294	0.0599008	0.307672
50878	Stag3	stromal antigen 3, transcript variant X5	NA	0.287156	3.307684	1.220232	0.05993779	0.307678
14397	Gabra4	gamma-aminobutyric acid (GABA) A receptor, subuni	NA	0.183573	4.01924	1.135693	0.05993963	0.307678
12870	Cp	ceruloplasmin, transcript variant 4	NA	0.201879	3.791767	1.150196	0.05998573	0.307817
20202	S100a9	S100 calcium binding protein A9 (calgranulin B), tran	NA	0.606521	0.077385	1.522583	0.06003471	0.307971
108097	Prkab2	protein kinase, AMP-activated, beta 2 non-catalytic su	NA	0.132597	5.122042	1.096265	0.06010749	0.308208
66300	Inafm1	InaF motif containing 1	NA	0.23753	4.184354	1.178973	0.06012393	0.308208
15976	Ifnar2	interferon (alpha and beta) receptor 2, transcript varia	NA	0.17336	4.478804	1.127681	0.06013772	0.308208
94213	Ddx50	DExD box helicase 50, transcript variant 3	NA	0.133494	6.358344	1.096947	0.06022309	0.308388
105245255	Gm40733	predicted gene, 40733, transcript variant 1	NA	0.406814	1.480752	1.325754	0.06022557	0.308388
11821	Aprt	adenine phosphoribosyl transferase	NA	0.157154	4.806029	1.115085	0.0602677	0.308388
56440	Snx1	sorting nexin 1	NA	-0.15527	5.972044	-1.11363	0.06028541	0.308388
104174	Gldc	glycine decarboxylase	NA	-0.15146	4.329654	-1.1107	0.06028567	0.308388
26400	Map2k7	mitogen-activated protein kinase kinase 7, transcript	NA	-0.10431	6.207517	-1.07498	0.06030253	0.308388
12354	Car7	carbonic anhydrase 7, transcript variant 2	NA	0.629981	0.015123	1.547545	0.06032112	0.308388
245596	Hdx	highly divergent homeobox, transcript variant 2	NA	0.521672	0.729761	1.435618	0.06033603	0.308388
14433	Gapdh	glyceraldehyde-3-phosphate dehydrogenase, transcr	NA	0.098823	10.4397	1.070899	0.06035796	0.308388
432582	Ccdc92b	coiled-coil domain containing 92B	NA	-0.19388	4.250096	-1.14384	0.06037927	0.308388
66836	Tmem223	transmembrane protein 223	NA	0.150541	4.981461	1.109985	0.06038123	0.308388
118567948	LOC118567948	uncharacterized LOC118567948	NA	0.596777	-0.14071	1.512334	0.06040533	0.308414
13367	Diaph1	diaphanous related formin 1, transcript variant 1	NA	0.103154	5.973446	1.074119	0.06046083	0.308601
100502766	Kifc1	kinesin family member C1	NA	-0.19803	3.977024	-1.14713	0.06055307	0.30889
12313	Calm1	calmodulin 1, transcript variant 2	NA	0.091407	9.9431	1.065408	0.06056356	0.30889
320158	Zmat4	zinc finger, matrin type 4, transcript variant X8	NA	0.186441	4.152164	1.137953	0.06057453	0.30889
16201	Ilf3	interleukin enhancer binding factor 3, transcript varia	NA	-0.09836	7.98679	-1.07056	0.06062292	0.308925
103080	Septin10	septin 10, transcript variant 2	NA	0.164725	4.212536	1.120952	0.06063025	0.308925
21406	Tcf12	transcription factor 12, transcript variant 4	NA	0.103143	7.058269	1.074111	0.0606564	0.308925

320184	Lrrc58	leucine rich repeat containing 58	NA	0.113247	7.031312	1.08166	0.06065722	0.308925
17687	Msh5	mutS homolog 5, transcript variant X20	NA	-0.5908	0.836128	-1.50609	0.06068045	0.308946
435965	Lrp3	low density lipoprotein receptor-related protein 3, trar	NA	-0.09368	6.777525	-1.06709	0.06087814	0.309856
14756	Gpld1	glycosylphosphatidylinositol specific phospholipase D	NA	0.269017	2.744203	1.204986	0.06092494	0.309946
17702	Msx2	msh homeobox 2	NA	0.552344	0.131036	1.466466	0.06095052	0.309946
72723	Zfp74	zinc finger protein 74	NA	0.142234	4.792131	1.103613	0.06095302	0.309946
102124	Enkd1	enkurin domain containing 1	NA	-0.222	3.196764	-1.16635	0.06099194	0.310009
210766	Brcc3	BRCA1/BRCA2-containing complex, subunit 3, transc	NA	-0.17864	4.920978	-1.13182	0.06102154	0.310009
320404	Itpkb	inositol 1,4,5-trisphosphate 3-kinase B, transcript vari	NA	-0.17357	4.590702	-1.12785	0.06102254	0.310009
234814	Mthfsd	methenyltetrahydrofolate synthetase domain containi	NA	-0.13121	5.001747	-1.09521	0.06104301	0.310016
223669	Zfp7	zinc finger protein 7, transcript variant 2	NA	-0.19034	3.913382	-1.14103	0.06108821	0.310149
74717	Spata17	spermatogenesis associated 17, transcript variant X2	NA	0.636254	0.592099	1.554288	0.06114409	0.31033
70511	Eef2kmt	eukaryotic elongation factor 2 lysine methyltransferas	NA	-0.36292	1.901418	-1.28602	0.06116204	0.31033
217371	Rab40b	Rab40B, member RAS oncogene family	NA	0.22141	2.99567	1.165873	0.06119472	0.310399
545182	Gm9861	predicted gene 9861, transcript variant X1	NA	-0.3344	1.970711	-1.26085	0.06123851	0.310493
280645	B3gat2	beta-1,3-glucuronyltransferase 2 (glucuronosyltransfe	NA	0.15313	4.676425	1.111979	0.0612514	0.310493
16351	Ipp	IAP promoted placental gene, transcript variant X1	NA	0.220683	3.5197	1.165285	0.06128771	0.310505
56531	Ylpm1	YLP motif containing 1, transcript variant 1	NA	0.106745	7.427601	1.076796	0.06129191	0.310505
574404	Gm14685	predicted gene 14685	NA	1.094359	-0.46073	2.135182	0.06133642	0.310634
76559	Atg2b	autophagy related 2B	NA	0.106045	5.80969	1.076274	0.06138201	0.310666
105245570	Gm26803	predicted gene, 26803, transcript variant X6	NA	0.550642	1.524789	1.464737	0.06139162	0.310666
20655	Sod1	superoxide dismutase 1, soluble	NA	0.139909	7.085553	1.101836	0.06140014	0.310666
66793	Efcab1	EF-hand calcium binding domain 1	NA	0.256675	2.633506	1.194722	0.06164703	0.311671
216795	Wnt9a	wingless-type MMTV integration site family, member	NA	-0.30904	2.002386	-1.23888	0.0616719	0.311671
207495	Baiap2l2	BAI1-associated protein 2-like 2, transcript variant X5	NA	0.595932	0.953389	1.511448	0.06169177	0.311671
66396	Ccdc82	coiled-coil domain containing 82	NA	0.16644	5.127648	1.122286	0.06169788	0.311671
21340	Taf1b	TATA-box binding protein associated factor, RNA pol	NA	0.126142	4.892022	1.091371	0.06173394	0.311671
72662	Dis3	DIS3 homolog, exosome endoribonuclease and 3'-5'	NA	0.165648	4.963279	1.12167	0.0617398	0.311671
18034	Nfkb2	nuclear factor of kappa light polypeptide gene enhanc	NA	-0.38534	1.736481	-1.30617	0.06175193	0.311671
21816	Tgm1	transglutaminase 1, K polypeptide, transcript variant)	NA	-0.77028	-0.32815	-1.70561	0.06177096	0.311671
108167321	Gm10479	predicted gene 10479, transcript variant X4	NA	0.687317	0.564033	1.610286	0.06177105	0.311671
102633433	Gm29478	predicted gene 29478, transcript variant X10	NA	-0.34602	2.309132	-1.27105	0.06179782	0.311709
16469	Jrk	jerky	NA	-0.21954	3.611041	-1.16437	0.06187999	0.311821
108671	Dnajc9	DnaJ heat shock protein family (Hsp40) member C9	NA	-0.11751	6.009982	-1.08486	0.06189842	0.311821
72121	Dennd2d	DENN/MADD domain containing 2D, transcript variar	NA	-0.79152	-0.25119	-1.7309	0.06191011	0.311821
100434	Slc44a1	solute carrier family 44, member 1, transcript variant)	NA	0.182194	5.079638	1.134608	0.0619343	0.311821
19246	Ptpn1	protein tyrosine phosphatase, non-receptor type 1	NA	-0.11637	6.211838	-1.084	0.06193789	0.311821
102639576	Gm35856	predicted gene, 35856	NA	0.463882	0.843703	1.379248	0.06194895	0.311821
241525	Ypel4	yippee like 4, transcript variant X2	NA	-0.16733	4.63015	-1.12298	0.06199603	0.311821
107747	Aldh1l1	aldehyde dehydrogenase 1 family, member L1, transc	NA	0.144696	5.685938	1.105497	0.06200272	0.311821
635253	Usp51	ubiquitin specific protease 51, transcript variant X2	NA	0.303059	2.517028	1.233758	0.06202016	0.311821
606735	A330069E16Rik	RIKEN cDNA A330069E16 gene	NA	0.590391	0.106899	1.505655	0.06202407	0.311821
243764	Chrm2	cholinergic receptor, muscarinic 2, cardiac, transcript	NA	0.219239	3.268791	1.164119	0.06203081	0.311821
67899	Cmc1	COX assembly mitochondrial protein 1	NA	0.2078	3.611503	1.154926	0.06205885	0.311824
170729	Scrt1	scratch family zinc finger 1	NA	-0.12575	6.872911	-1.09108	0.06206968	0.311824
229644	Trim45	tripartite motif-containing 45, transcript variant 2	NA	-0.16696	4.065443	-1.12269	0.0621054	0.311832
75607	Wnk2	WNK lysine deficient protein kinase 2, transcript varia	NA	-0.09648	7.444443	-1.06916	0.06212662	0.311832
105245924	Gm16136	predicted gene 16136, transcript variant 2	NA	-1.04156	-1.00889	-2.05845	0.06212874	0.311832
20402	Zfp106	zinc finger protein 106, transcript variant X6	NA	0.093829	7.109345	1.067199	0.06218505	0.311986
59013	Hnrnph1	heterogeneous nuclear ribonucleoprotein H1, transcri	NA	-0.10484	9.443095	-1.07538	0.06221388	0.311986
74137	Nuak2	NUAK family, SNF1-like kinase, 2, transcript variant)	NA	-0.20433	3.983825	-1.15215	0.06222616	0.311986
19017	Ppargc1a	peroxisome proliferative activated receptor, gamma, (	NA	0.171296	4.665667	1.126069	0.06223615	0.311986
105246164	Gm41498	predicted gene, 41498	NA	-0.76622	0.03873	-1.70081	0.06226666	0.312043
244238	Mrgpre	MAS-related GPR, member E	NA	-0.24055	3.90058	-1.18144	0.06230069	0.312117
100504221	Efcab8	EF-hand calcium binding domain 8, transcript variant	NA	0.428775	1.530049	1.34609	0.06234683	0.312228
54137	Acrbp	proacrosin binding protein, transcript variant 2	NA	-0.24108	3.793583	-1.18188	0.06239416	0.312228
110862	Kcnq3	potassium voltage-gated channel, subfamily Q, meml	NA	0.124716	6.263268	1.090293	0.06239725	0.312228
218975	Mapk1ip1l	mitogen-activated protein kinase 1 interacting protein	NA	-0.10553	7.081476	-1.07589	0.06242584	0.312228
20637	Snrnp70	small nuclear ribonucleoprotein 70 (U1), transcript va	NA	-0.09427	8.743385	-1.06752	0.06243048	0.312228
21987	Tpd52l1	tumor protein D52-like 1, transcript variant 1	NA	0.223135	3.415283	1.167267	0.06243784	0.312228
74563	Rasgef1c	RasGEF domain family, member 1C, transcript variar	NA	0.174012	3.987818	1.128191	0.06248237	0.312307
545539	Gm15417	predicted gene 15417, transcript variant 1	NA	0.605105	-0.03828	1.52109	0.0624973	0.312307
24052	Sgcd	sarcoglycan, delta (dystrophin-associated glycoprotei	NA	0.267392	3.600791	1.20363	0.0625133	0.312307
107373	Fam111a	family with sequence similarity 111, member A, transc	NA	-0.18623	4.002101	-1.13779	0.06253982	0.312307
93889	Pcdhb18	protocadherin beta 18	NA	0.246922	3.019743	1.186673	0.06254975	0.312307
102637845	Gm34552	predicted gene, 34552, transcript variant X2	NA	0.518826	1.088064	1.432789	0.06261	0.312443
83703	Dbr1	debranching RNA lariats 1, transcript variant 1	NA	0.160273	4.243111	1.117498	0.06261535	0.312443
71947	Tmem94	transmembrane protein 94, transcript variant X7	NA	-0.12782	6.085967	-1.09264	0.06271219	0.312819

17975	Ncl	nucleolin, transcript variant X1	NA	0.086005	8.928324	1.061427	0.06274201	0.312819
78388	Mvp	major vault protein	NA	0.311122	1.844079	1.240672	0.06274832	0.312819
70420	Arpin	actin-related protein 2/3 complex inhibitor	NA	-0.23892	3.61244	-1.18011	0.06279089	0.312869
260299	Cadm4	cell adhesion molecule 4	NA	0.117893	8.048723	1.085149	0.06279691	0.312869
110304	Glra3	glycine receptor, alpha 3 subunit, transcript variant 2	NA	0.316572	1.819783	1.245368	0.06282789	0.312903
216233	Socs2	suppressor of cytokine signaling 2, transcript variant 4	NA	0.097874	6.264812	1.070195	0.0628427	0.312903
320365	Fry	FRY microtubule binding protein, transcript variant X1	NA	0.155734	6.785557	1.113988	0.06287976	0.312903
218341	Rfesd	Rieske (Fe-S) domain containing, transcript variant 1	NA	0.193799	3.400004	1.143771	0.06288067	0.312903
71673	Rnf215	ring finger protein 215, transcript variant 1	NA	0.136406	5.124322	1.099164	0.06293562	0.313081
100040938	Gm3052	predicted gene 3052	NA	-0.21968	3.929082	-1.16448	0.06298273	0.313112
102631576	Gm29879	predicted gene, 29879, transcript variant X7	NA	0.759527	0.78448	1.692936	0.06300949	0.313112
73516	1700086O06Rik	RIKEN cDNA 1700086O06 gene, transcript variant 3	NA	0.380877	1.595975	1.302133	0.06301289	0.313112
74342	Lrrtm1	leucine rich repeat transmembrane neuronal 1, transc	NA	-0.14506	5.175926	-1.10578	0.06301887	0.313112
53412	Ppp1r3c	protein phosphatase 1, regulatory subunit 3C	NA	0.191882	3.946056	1.142253	0.06316517	0.313457
212531	Sh3bgrl2	SH3 domain binding glutamic acid-rich protein like 2	NA	-0.20751	3.587744	-1.15469	0.06317385	0.313457
233781	Xylt1	xylosyltransferase 1	NA	0.384254	1.873635	1.305184	0.0631837	0.313457
320360	Ric3	RIC3 acetylcholine receptor chaperone, transcript var	NA	0.117589	5.406766	1.08492	0.06319953	0.313457
102633984	Gm31677	predicted gene, 31677, transcript variant X1	NA	0.606259	-0.07176	1.522306	0.06320197	0.313457
83962	Btbd1	BTB (POZ) domain containing 1	NA	0.099974	7.350223	1.071755	0.06321849	0.313457
84036	Kcnn1	potassium intermediate/small conductance calcium-a	NA	-0.18084	4.110411	-1.13354	0.06322302	0.313457
20262	Stmn3	stathmin-like 3	NA	0.089116	8.263552	1.063718	0.06332533	0.313868
64706	Scube1	signal peptide, CUB domain, EGF-like 1, transcript va	NA	-0.13501	5.78791	-1.0981	0.06334611	0.313876
71978	Ppp2r2a	protein phosphatase 2, regulatory subunit B, alpha, tr	NA	0.097036	6.957104	1.069574	0.06346847	0.314184
320078	Olfml2b	olfactomedin-like 2B	NA	-0.20828	3.421838	-1.15531	0.06347667	0.314184
20509	Slc19a1	solute carrier family 19 (folate transporter), member 1	NA	0.164589	4.202716	1.120847	0.06350195	0.314184
66521	Rwdd1	RWD domain containing 1	NA	0.12454	5.677745	1.09016	0.06350591	0.314184
76478	Haus8	4HAUS augmin-like complex, subunit 8, transcript var	NA	-0.17671	4.398424	-1.1303	0.06351254	0.314184
67118	Bfar	bifunctional apoptosis regulator, transcript variant 1	NA	-0.11297	5.623372	-1.08145	0.06355535	0.314184
67371	Gtf3c6	general transcription factor IIIC, polypeptide 6, alpha,	NA	0.125269	5.657404	1.090711	0.06356138	0.314184
16004	Igf2r	insulin-like growth factor 2 receptor	NA	-0.0998	6.130051	-1.07163	0.06357896	0.314184
54611	Pde3a	phosphodiesterase 3A, cGMP inhibited, transcript var	NA	0.210321	3.829633	1.156945	0.06358203	0.314184
329154	Ankrd44	ankyrin repeat domain 44	NA	0.137979	5.411269	1.100362	0.06364434	0.314385
244694	Kdm4d	lysine (K)-specific demethylase 4D	NA	0.868377	-0.76281	1.825608	0.0636614	0.314385
230991	Fndc10	fibronectin type III domain containing 10	NA	0.215532	3.801537	1.161132	0.06369926	0.314476
74766	Yipf2	Yip1 domain family, member 2, transcript variant X1	NA	-0.20521	3.393637	-1.15285	0.06378361	0.314797
118567621	LOC118567621	MLV-related proviral Env polyprotein-like, transcript v	NA	0.237133	3.066464	1.178648	0.0638479	0.315019
20196	S100a13	S100 calcium binding protein A13	NA	0.492815	1.024459	1.407188	0.06388856	0.315124
217125	Samd14	sterile alpha motif domain containing 14, transcript va	NA	-0.0842	8.178044	-1.0601	0.06397609	0.31546
211660	Cspp1	centrosome and spindle pole associated protein 1, tra	NA	0.107632	5.763507	1.077458	0.0640008	0.315487
277468	Slc39a12	solute carrier family 39 (zinc transporter), member 12	NA	0.321213	1.951023	1.24938	0.06409612	0.315651
56277	Tmem45a	transmembrane protein 45a, transcript variant X6	NA	-0.49385	1.782545	-1.4082	0.06410678	0.315651
19044	Ppox	protoporphyrinogen oxidase	NA	-0.14041	4.609409	-1.10222	0.06410748	0.315651
235431	Coro2b	coronin, actin binding protein, 2B, transcript variant X	NA	-0.10135	6.828826	-1.07277	0.06412566	0.315651
225929	Patl1	protein associated with topoisomerase II homolog 1 (	NA	-0.10789	5.606137	-1.07765	0.0641332	0.315651
20981	Syt3	synaptotagmin III, transcript variant 1	NA	-0.12807	5.058223	-1.09283	0.06415065	0.315651
30877	Gnl3	guanine nucleotide binding protein-like 3 (nucleolar),	NA	0.123074	5.090001	1.089053	0.06419763	0.315786
93881	Pcdhb10	protocadherin beta 10	NA	0.363216	1.401181	1.28629	0.06423378	0.315786
105246763	Gm42005	predicted gene, 42005	NA	-0.54905	0.455934	-1.46312	0.06423626	0.315786
50884	Nckap1	NCK-associated protein 1, transcript variant 2	NA	0.136939	8.007356	1.09957	0.06435015	0.316187
12189	Brca1	breast cancer 1, early onset, transcript variant X5	NA	-0.24063	3.494056	-1.18151	0.06437263	0.316187
232339	Ankrd26	ankyrin repeat domain 26, transcript variant X8	NA	0.146594	4.622081	1.106953	0.06437609	0.316187
434175	Ccnb1-ps	cyclin B1, pseudogene	NA	-1.14409	0.528906	-2.21007	0.06445158	0.316462
545030	Wdfy4	WD repeat and FYVE domain containing 4	NA	-0.71874	-0.13853	-1.64574	0.0644987	0.316503
14802	Gria4	glutamate receptor, ionotropic, AMPA4 (alpha 4), trar	NA	0.1163	6.467249	1.083952	0.0644988	0.316503
19732	Rgl2	ral guanine nucleotide dissociation stimulator-like 2	NA	-0.10858	5.951686	-1.07817	0.06453065	0.316564
118568318	LOC118568318	uncharacterized LOC118568318	NA	-0.5367	0.364606	-1.45065	0.0645597	0.316611
66397	Sar1b	secretion associated Ras related GTPase 1B	NA	0.102088	5.906511	1.073325	0.06460798	0.31664
93709	Pcdhga1	protocadherin gamma subfamily A, 1	NA	0.260047	3.873624	1.197518	0.06461227	0.31664
75099	Lysmd4	LysM, putative peptidoglycan-binding, domain contair	NA	-0.17455	5.347544	-1.12861	0.06462959	0.31664
19777	Uri1	URI1, prefoldin-like chaperone, transcript variant X2	NA	0.126231	5.685004	1.091438	0.06464339	0.31664
27403	Abca7	ATP-binding cassette, sub-family A (ABC1), member	NA	-0.15855	4.117275	-1.11616	0.06467331	0.316691
67241	Smc6	structural maintenance of chromosomes 6, transcript	NA	0.10377	6.286875	1.074578	0.06472173	0.316817
66965	Ctu2	cytosolic thiouridylase subunit 2, transcript variant X3	NA	0.153784	4.279955	1.112483	0.06473806	0.316817
100302648	Gm11944	predicted gene 11944	NA	-0.63556	-0.09705	-1.55354	0.06476944	0.316869
107939	Pom121	nuclear pore membrane protein 121, transcript varian	NA	-0.10812	6.606953	-1.07782	0.06480291	0.316869
19215	Ptgds	prostaglandin D2 synthase (brain), transcript variant X	NA	0.279046	6.113106	1.213393	0.06484181	0.316869
66420	Polr2e	polymerase (RNA) II (DNA directed) polypeptide E	NA	0.114214	5.339972	1.082385	0.0648593	0.316869
207607	Ccdc40	coiled-coil domain containing 40, transcript variant X4	NA	-0.17925	4.051971	-1.13229	0.06486305	0.316869

100034363	Tmsb15b2	thymosin beta 15b2	NA	0.472764	1.775422	1.387765	0.06486553	0.316869
110351	Rap1gap	Rap1 GTPase-activating protein, transcript variant 3	NA	0.119676	6.130257	1.086491	0.06496001	0.317189
101095	Zfp282	zinc finger protein 282	NA	-0.10537	5.994621	-1.07577	0.06496992	0.317189
108169145	Gm44026	predicted gene, 44026	NA	0.531624	0.985964	1.445555	0.06499181	0.317201
243043	Kctd8	potassium channel tetramerisation domain containing	NA	0.196549	3.500068	1.145954	0.06504178	0.317349
240087	Mdc1	mediator of DNA damage checkpoint 1, transcript var	NA	-0.14713	5.812226	-1.10736	0.06514953	0.31778
100201	Tmem64	transmembrane protein 64	NA	0.182735	4.639291	1.135034	0.06522152	0.318036
75570	Nhej1	non-homologous end joining factor 1	NA	-0.3578	1.560164	-1.28147	0.06525624	0.31811
68273	Pomgnt1	protein O-linked mannose beta 1,2-N-acetylglucosam	NA	-0.1065	6.141577	-1.07661	0.06532962	0.318297
212163	8030462N17Rik	RIKEN cDNA 8030462N17 gene, transcript variant 2	NA	0.154575	4.746766	1.113094	0.06536738	0.318297
100737	Dcun1d4	DCN1, defective in cullin neddylation 1, domain conta	NA	0.104771	6.21111	1.075324	0.06536919	0.318297
433956	Dnaaf5	dynein, axonemal assembly factor 5, transcript variar	NA	-0.1915	3.660909	-1.14195	0.06537304	0.318297
19659	Rbp1	retinol binding protein 1, cellular	NA	-0.13788	5.073764	-1.10028	0.06544956	0.318575
102638461	Gm26793	predicted gene, 26793	NA	-0.37989	1.632171	-1.30125	0.06550965	0.318772
108168813	Gm46775	predicted gene, 46775	NA	-0.47794	0.638148	-1.39276	0.06553115	0.318781
57230	Sap30bp	SAP30 binding protein	NA	-0.12479	5.579731	-1.09035	0.06561092	0.318972
67027	Mkrn2	makorin, ring finger protein, 2	NA	0.137223	5.517101	1.099786	0.06562006	0.318972
225055	Fbxo11	F-box protein 11, transcript variant X1	NA	0.097121	7.595596	1.069637	0.06565259	0.318972
52588	Tspan14	tetraspanin 14, transcript variant 2	NA	-0.1051	6.12101	-1.07557	0.06565889	0.318972
227058	Dnah7b	dynein, axonemal, heavy chain 7B, transcript variant	NA	0.2498	3.237945	1.189043	0.06570841	0.318972
67870	Enoph1	enolase-phosphatase 1, transcript variant X1	NA	0.137637	5.150992	1.100102	0.06572667	0.318972
625249	Gpx4	glutathione peroxidase 4, transcript variant 1	NA	0.143715	6.76793	1.104746	0.06573444	0.318972
319880	Tmcc3	transmembrane and coiled coil domains 3, transcript	NA	0.174436	4.16113	1.128523	0.06574656	0.318972
19337	Rab33a	RAB33A, member RAS oncogene family	NA	0.203582	4.979677	1.151554	0.06574673	0.318972
230895	Vps13d	vacuolar protein sorting 13D, transcript variant 1	NA	0.124334	5.848219	1.090005	0.06580167	0.318979
74718	Snx16	sorting nexin 16, transcript variant X1	NA	-0.1178	5.654663	-1.08508	0.06581325	0.318979
668272	Gm9079	predicted gene 9079	NA	0.368764	1.430962	1.291246	0.06585376	0.318979
11475	Acta2	actin, alpha 2, smooth muscle, aorta	NA	0.205149	3.797262	1.152806	0.06585634	0.318979
54630	Prickle3	prickle planar cell polarity protein 3	NA	-0.397	1.161587	-1.31677	0.06588079	0.318979
81630	Zbtb22	zinc finger and BTB domain containing 22	NA	0.158849	4.999294	1.116396	0.06588115	0.318979
68453	Gpihbp1	GPI-anchored HDL-binding protein 1, transcript varia	NA	0.96594	-0.57902	1.953336	0.06589633	0.318979
11732	Ank	progressive ankylosis	NA	0.110823	5.577659	1.079844	0.06590518	0.318979
74112	Usp16	ubiquitin specific peptidase 16, transcript variant X5	NA	0.135669	5.345795	1.098602	0.06600808	0.319383
241846	Lsm14b	LSM family member 14B, transcript variant 1	NA	-0.08597	7.951256	-1.0614	0.06604664	0.319444
102636088	Gm33257	predicted gene, 33257, transcript variant X3	NA	0.225697	3.483312	1.169342	0.06606004	0.319444
11787	Apbb2	amyloid beta (A4) precursor protein-binding, family B	NA	0.09818	6.420145	1.070422	0.06610737	0.319578
102632019	Gm26777	predicted gene, 26777, transcript variant X2	NA	0.257428	2.724853	1.195346	0.06617721	0.319589
56463	Snd1	staphylococcal nuclease and tudor domain containing	NA	-0.10192	6.738397	-1.0732	0.06617757	0.319589
230861	Eif4g3	eukaryotic translation initiation factor 4 gamma, 3, tra	NA	0.090845	8.063252	1.064994	0.06619597	0.319589
234358	Zfp930	zinc finger protein 930	NA	0.175192	3.96548	1.129115	0.06620135	0.319589
18747	Prkaca	protein kinase, cAMP dependent, catalytic, alpha, tra	NA	-0.09227	7.636363	-1.06604	0.06620796	0.319589
80907	Lactb	lactamase, beta	NA	0.188215	3.978972	1.139353	0.06626407	0.319765
18440	P2rx6	purinergic receptor P2X, ligand-gated ion channel, 6,	NA	-0.48517	0.637688	-1.39975	0.06634148	0.319807
93691	Klf7	Kruppel-like factor 7 (ubiquitous), transcript variant X	NA	0.089367	7.831377	1.063903	0.06634351	0.319807
83796	Smarcd2	SWI/SNF related, matrix associated, actin dependent	NA	-0.17434	4.330202	-1.12845	0.06637015	0.319807
17758	Map4	microtubule-associated protein 4, transcript variant 1	NA	0.091746	8.590022	1.065659	0.06638873	0.319807
20403	Itsn2	intersectin 2, transcript variant X7	NA	0.126317	5.070732	1.091504	0.06639556	0.319807
381305	Rc3h1	RING CCCH (C3H) domains 1	NA	0.120322	6.008995	1.086977	0.06642262	0.319807
230101	Gba2	glucosidase beta 2	NA	-0.09866	6.343968	-1.07078	0.06643338	0.319807
74559	Elovl7	ELOVL family member 7, elongation of long chain fat	NA	0.354286	1.959823	1.278353	0.06644218	0.319807
232664	Ccdc136	coiled-coil domain containing 136, transcript variant X	NA	0.129077	6.107758	1.093594	0.06644967	0.319807
76156	Fam131b	family with sequence similarity 131, member B, trans	NA	-0.10388	6.225456	-1.07466	0.06657424	0.319883
14184	Fgfr3	fibroblast growth factor receptor 3, transcript variant 3	NA	-0.13097	5.494689	-1.09503	0.06659511	0.319883
100503802	Gm10419	predicted gene 10419	NA	0.347297	1.568898	1.272175	0.06664723	0.319883
72828	Ubash3b	ubiquitin associated and SH3 domain containing, B, t	NA	0.131838	5.452218	1.095688	0.06666101	0.319883
244853	Nxpe4	neurexophilin and PC-esterase domain family, memb	NA	0.224388	3.719	1.168282	0.06666512	0.319883
12442	Ccnb2	cyclin B2	NA	-0.18415	4.205484	-1.13615	0.06667272	0.319883
14370	Fzd8	frizzled class receptor 8	NA	-0.1967	3.85222	-1.14608	0.06667407	0.319883
72677	2810049E08Rik	RIKEN cDNA 2810049E08 gene	NA	0.37255	1.752084	1.294639	0.06667614	0.319883
17954	Nap1l2	nucleosome assembly protein 1-like 2	NA	0.127966	5.83327	1.092752	0.06668245	0.319883
270151	Nlrx1	NLR family member X1, transcript variant 3	NA	-0.2846	2.363127	-1.21807	0.06671008	0.319883
338337	Cog3	component of oligomeric golgi complex 3	NA	0.114907	5.348474	1.082905	0.06671023	0.319883
619309	Muc3a	mucin 3A, cell surface associated, transcript variant X	NA	0.434991	1.672971	1.351902	0.06671681	0.319883
109019	Nabp1	nucleic acid binding protein 1, transcript variant 2	NA	-0.29742	2.989685	-1.22894	0.0667302	0.319883
74198	Dtx2	deltex 2, E3 ubiquitin ligase, transcript variant 4	NA	-0.16385	4.086869	-1.12027	0.06674064	0.319883
217335	Fbf1	Fas (TNFRSF6) binding factor 1, transcript variant X4	NA	-0.14861	6.799089	-1.1085	0.06682871	0.320143
22234	Ugcg	UDP-glucose ceramide glucosyltransferase	NA	0.118943	6.745069	1.085939	0.06684667	0.320143
57752	Tacc2	transforming, acidic coiled-coil containing protein 2, tr	NA	0.109665	6.97653	1.078977	0.06685386	0.320143

69539	Trnp1	TMF1-regulated nuclear protein 1	NA	0.219164	3.748295	1.164059	0.06689093	0.320226
94246	Arid4b	AT rich interactive domain 4B (RBP1-like), transcript	NA	0.107589	6.448305	1.077426	0.06695475	0.320438
29867	Cabp1	calcium binding protein 1, transcript variant 3	NA	0.179066	4.351042	1.13215	0.06699497	0.32053
213819	Casd1	CAS1 domain containing 1, transcript variant 4	NA	0.124919	6.30521	1.090446	0.06701344	0.32053
56384	Letm1	leucine zipper-EF-hand containing transmembrane pr	NA	0.099821	6.34132	1.07164	0.06706579	0.320643
56198	Heyl	hairy/enhancer-of-split related with YRPW motif-like	NA	-0.26436	3.735831	-1.20111	0.0670765	0.320643
83766	Actl6b	actin-like 6B, transcript variant X8	NA	-0.10442	6.821032	-1.07506	0.06713511	0.320829
118568416	LOC118568416	uncharacterized LOC118568416	NA	0.286313	2.601562	1.219519	0.06716291	0.320868
74116	Pi16	peptidase inhibitor 16	NA	-0.37128	1.6624	-1.2935	0.06720329	0.320917
99138	Stard7	START domain containing 7, transcript variant 1	NA	0.102779	7.175964	1.07384	0.06721269	0.320917
107351	Kank1	KN motif and ankyrin repeat domains 1, transcript var	NA	0.227867	3.446205	1.171103	0.06723558	0.320918
81489	Dnajb1	DnaJ heat shock protein family (Hsp40) member B1,	NA	0.105842	5.757918	1.076123	0.06725241	0.320918
269589	Sytl1	synaptotagmin-like 1	NA	-0.80944	-0.23369	-1.75253	0.06730925	0.321012
26611	Rcn2	reticulocalbin 2, transcript variant 1	NA	0.103184	7.048615	1.074142	0.06731159	0.321012
74478	Snx29	sorting nexin 29, transcript variant X12	NA	-0.11968	5.478897	-1.0865	0.06734004	0.321054
13996	Etohd2	ethanol decreased 2	NA	-0.55157	0.235122	-1.46568	0.06740533	0.32117
210148	Slc30a6	solute carrier family 30 (zinc transporter), member 6,	NA	-0.2051	3.883283	-1.15277	0.06743338	0.32117
18488	Cntn3	contactin 3	NA	0.218449	3.8101	1.163482	0.06744054	0.32117
72515	Wdr43	WD repeat domain 43	NA	0.113452	5.726292	1.081814	0.06744846	0.32117
17901	Myl1	myosin, light polypeptide 1, transcript variant 1f	NA	-0.62384	2.534898	-1.54097	0.06749247	0.32117
56464	Ctsf	cathepsin F	NA	0.156394	4.854895	1.114498	0.06749277	0.32117
102640822	Nr6a1os	nuclear receptor subfamily 6, group A, member 1, op	NA	-0.2517	2.583401	-1.19061	0.06750258	0.32117
50787	Hs6st3	heparan sulfate 6-O-sulfotransferase 3	NA	0.626318	1.159728	1.54362	0.06755212	0.321306
102639568	A930005G22Rik	RIKEN cDNA A930005G22 gene, transcript variant 2	NA	0.518423	0.267679	1.432389	0.06757071	0.321306
108961	E2f8	E2F transcription factor 8, transcript variant X1	NA	-0.29899	3.426875	-1.23028	0.06768378	0.32175
78733	Troap	trophinin associated protein, transcript variant 1	NA	-0.27581	2.868172	-1.21067	0.06773341	0.321892
16923	Sh2b3	SH2B adaptor protein 3, transcript variant X5	NA	0.152563	4.109505	1.111542	0.06781121	0.322168
71720	Osbpl3	oxysterol binding protein-like 3, transcript variant X2	NA	0.198665	3.500149	1.147636	0.06787099	0.322224
76238	Grhpr	glyoxylate reductase/hydroxypyruvate reductase, tran	NA	0.270788	3.031687	1.206467	0.06790572	0.322224
11856	Arhgap6	Rho GTPase activating protein 6, transcript variant 2	NA	0.350112	1.622667	1.274659	0.06791099	0.322224
66970	Ssbp2	single-stranded DNA binding protein 2, transcript vari	NA	0.123214	7.556076	1.089159	0.06791796	0.322224
11744	Anxa11	annexin A11	NA	-0.26739	2.60125	-1.20363	0.06792935	0.322224
68732	Carmil1	capping protein regulator and myosin 1 linker 1, trans	NA	0.116015	5.491546	1.083738	0.06794186	0.322224
105243412	Gm39342	predicted gene, 39342	NA	0.308733	2.173565	1.238619	0.06798919	0.322317
18679	Phka1	phosphorylase kinase alpha 1, transcript variant 2	NA	0.242971	3.95307	1.183427	0.06800103	0.322317
218734	3830406C13Rik	RIKEN cDNA 3830406C13 gene, transcript variant 5	NA	0.149712	4.592793	1.109348	0.06815849	0.32285
15259	Hipk3	homeodomain interacting protein kinase 3, transcript	NA	0.157431	5.651712	1.1153	0.06820333	0.32285
17977	Ncoa1	nuclear receptor coactivator 1, transcript variant X12	NA	0.120352	6.530425	1.087	0.06821946	0.32285
18968	Pola1	polymerase (DNA directed), alpha 1, transcript varian	NA	-0.22931	3.817071	-1.17227	0.06821988	0.32285
14281	Fos	FBJ osteosarcoma oncogene	NA	0.508761	0.739931	1.422827	0.06822091	0.32285
72587	Pan3	PAN3 poly(A) specific ribonuclease subunit, transcrip	NA	0.106638	5.944928	1.076717	0.0682326	0.32285
12751	Tpp1	tripeptidyl peptidase I	NA	-0.17478	5.022503	-1.12879	0.06825426	0.322859
23936	Lynx1	Ly6/neurotoxin 1	NA	-0.20486	3.630859	-1.15257	0.06837761	0.323146
54194	Akap8l	A kinase (PRKA) anchor protein 8-like, transcript vari	NA	-0.11198	6.53342	-1.08071	0.06837965	0.323146
54122	Uevld	UEV and lactate/malate dehyrogenase domains	NA	0.170474	4.462099	1.125428	0.06839376	0.323146
68312	Gstm7	glutathione S-transferase, mu 7, transcript variant 1	NA	-0.17501	4.729593	-1.12897	0.06840747	0.323146
628308	Zfp970	zinc finger protein 970	NA	0.209883	3.638671	1.156594	0.06841432	0.323146
18389	Oprl1	opioid receptor-like 1, transcript variant 8	NA	0.111346	6.339048	1.080236	0.06843927	0.32317
81600	Chia1	chitinase, acidic 1	NA	0.533738	0.523851	1.447675	0.0684754	0.323192
66661	Srp72	signal recognition particle 72	NA	0.087309	7.048729	1.062387	0.06853069	0.323192
108123	Napg	N-ethylmaleimide sensitive fusion protein attachment	NA	0.102536	6.123694	1.073659	0.06854714	0.323192
21787	Tfg	Trk-fused gene, transcript variant 1	NA	0.117877	6.553583	1.085137	0.06855118	0.323192
14870	Gstp1	glutathione S-transferase, pi 1	NA	0.103423	6.347137	1.074319	0.06857218	0.323192
231946	Fam221a	family with sequence similarity 221, member A, trans	NA	0.459701	1.408329	1.375257	0.06857263	0.323192
69527	Mrps9	mitochondrial ribosomal protein S9	NA	0.139673	4.923253	1.101655	0.06858306	0.323192
99152	Anapc2	anaphase promoting complex subunit 2	NA	-0.10078	6.899569	-1.07235	0.06863617	0.323349
57316	C1d	C1D nuclear receptor co-repressor, transcript variant	NA	0.099087	6.438087	1.071095	0.06870201	0.323501
224481	Tfb1m	transcription factor B1, mitochondrial	NA	-0.28368	2.828693	-1.21729	0.06870821	0.323501
19012	Plpp1	phospholipid phosphatase 1, transcript variant 1	NA	0.138877	4.663016	1.101048	0.06881234	0.323882
118568390	LOC118568390	uncharacterized LOC118568390	NA	0.309193	1.819894	1.239015	0.06882888	0.323882
242259	Slc44a5	solute carrier family 44, member 5, transcript variant	NA	0.197947	5.078782	1.147065	0.06885049	0.32389
50769	Atp8a2	ATPase, aminophospholipid transporter-like, class I,	NA	0.222078	3.565792	1.166412	0.06898107	0.324341
16581	Kifc2	kinesin family member C2, transcript variant X4	NA	0.143344	6.250099	1.104462	0.06899281	0.324341
18791	Plat	plasminogen activator, tissue	NA	0.107671	5.721287	1.077487	0.06900617	0.324341
276846	Pigs	phosphatidylinositol glycan anchor biosynthesis, clas	NA	-0.10804	5.740985	-1.07776	0.06922661	0.325283
12418	Cbx4	chromobox 4	NA	-0.13178	5.621622	-1.09565	0.06925659	0.325283
21355	Tap2	transporter 2, ATP-binding cassette, sub-family B (MI	NA	0.547441	0.52282	1.461491	0.0692666	0.325283
56085	Ubqln1	ubiquilin 1, transcript variant 2	NA	-0.10277	7.559309	-1.07384	0.06933702	0.32552

109331	Rnf20	ring finger protein 20, transcript variant 3	NA	0.092548	6.580603	1.066252	0.06941324	0.325783
68549	Sgo2a	shugoshin 2A, transcript variant 2	NA	-0.1939	3.634641	-1.14385	0.06944495	0.325783
104001	Rtn1	reticulon 1, transcript variant 2	NA	0.101536	10.08273	1.072915	0.06949123	0.325783
20874	Slk	STE20-like kinase, transcript variant 2	NA	0.130458	5.742518	1.094641	0.06949271	0.325783
22762	Zfpm2	zinc finger protein, multitype 2, transcript variant 2	NA	0.141815	5.109003	1.103292	0.06949314	0.325783
668661	2410002F23Rik	RIKEN cDNA 2410002F23 gene	NA	0.10591	7.201797	1.076173	0.0695743	0.325863
665033	Col6a5	collagen, type VI, alpha 5	NA	0.398119	1.067859	1.317789	0.06959109	0.325863
77371	Sec24a	Sec24 related gene family, member A (S. cerevisiae)	NA	0.160167	5.017887	1.117417	0.06959323	0.325863
69582	Plekhm2	pleckstrin homology domain containing, family M (witl	NA	-0.1271	5.476714	-1.09209	0.06960002	0.325863
226180	Ina	internexin neuronal intermediate filament protein, alpl	NA	-0.09274	9.354636	-1.06639	0.06962409	0.325863
109246	Tspan9	tetraspanin 9, transcript variant 2	NA	-0.13281	5.741339	-1.09643	0.0696385	0.325863
23994	Dazap2	DAZ associated protein 2	NA	0.098196	6.802318	1.070434	0.06965044	0.325863
56327	Arl2	ADP-ribosylation factor-like 2	NA	0.137144	4.50705	1.099726	0.06973576	0.326161
56278	Gkap1	G kinase anchoring protein 1, transcript variant X4	NA	0.159461	5.098097	1.11687	0.06975415	0.326161
330671	B4galnt4	beta-1,4-N-acetyl-galactosaminyl transferase 4	NA	-0.09556	6.843841	-1.06848	0.06982984	0.326421
105651	Ppp1r3e	protein phosphatase 1, regulatory subunit 3E	NA	-0.22207	3.278472	-1.16641	0.0698523	0.326432
80913	Pum2	pumilio RNA-binding family member 2, transcript vari:	NA	0.091814	8.082813	1.065709	0.06987299	0.326435
432763	Prr7	proline rich 7 (synaptic)	NA	0.158494	4.19859	1.116122	0.06989808	0.326459
17250	Abcc1	ATP-binding cassette, sub-family C (CFTR/MRP), me	NA	0.145755	4.580093	1.10631	0.06992631	0.326497
21808	Tgfb2	transforming growth factor, beta 2, transcript variant ´	NA	0.134603	5.018974	1.097791	0.06995703	0.326546
75137	Rprd2	regulation of nuclear pre-mRNA domain containing 2,	NA	0.093886	6.32688	1.067241	0.07003893	0.326835
71740	Nectin4	nectin cell adhesion molecule 4, transcript variant 1	NA	-0.3105	1.761111	-1.24013	0.07007076	0.32689
16855	Lgals4	lectin, galactose binding, soluble 4	NA	0.366183	1.680148	1.288938	0.07010991	0.326937
319613	Sybu	syntabulin (syntaxin-interacting), transcript variant e	NA	0.111229	6.617576	1.080148	0.07012222	0.326937
75452	Ascc2	activating signal cointegrator 1 complex subunit 2, tra	NA	-0.13189	5.66452	-1.09573	0.07014121	0.326937
100502698	Rubcn	RUN domain and cysteine-rich domain containing, Be	NA	-0.12155	6.166162	-1.0879	0.0702219	0.32722
140810	Ttbk2	tau tubulin kinase 2, transcript variant 1	NA	0.115114	6.722187	1.083061	0.07024293	0.327224
13039	Ctsl	cathepsin L, transcript variant X1	NA	0.123785	6.404535	1.08959	0.07035474	0.327651
170930	Sumo2	small ubiquitin-like modifier 2	NA	0.085986	8.83556	1.061413	0.07042301	0.327875
12169	Bmx	BMX non-receptor tyrosine kinase	NA	0.703227	-0.12929	1.628143	0.07045406	0.327926
319764	A730046J19Rik	RIKEN cDNA A730046J19 gene	NA	0.58449	0.370886	1.499508	0.0705122	0.328018
19192	Psme3	proteaseome (prosome, macropain) activator subunit	NA	-0.12396	7.061964	-1.08972	0.07051415	0.328018
52357	Wwc2	WW, C2 and coiled-coil domain containing 2, transcri	NA	0.13303	4.822443	1.096595	0.07054492	0.328067
71529	Kazn	kazrin, periplakin interacting protein, transcript varian	NA	-0.11463	6.410248	-1.0827	0.07060536	0.328254
60595	Actn4	actinin alpha 4, transcript variant X1	NA	-0.10021	6.82795	-1.07193	0.07066224	0.328309
245403	Dcaf12l2	DDB1 and CUL4 associated factor 12-like 2	NA	0.417544	0.921434	1.335652	0.07066865	0.328309
74347	Meak7	MTOR associated protein, eak-7 homolog, transcript	NA	-0.20102	3.780958	-1.14951	0.07067766	0.328309
240613	9930021J03Rik	RIKEN cDNA 9930021J03 gene, transcript variant X9	NA	0.126098	6.3514	1.091338	0.07074307	0.328392
69617	Pitrm1	pitrilysin metallepetidase 1, transcript variant 2	NA	-0.11358	5.748528	-1.08191	0.07077359	0.328392
72726	Tbcc	tubulin-specific chaperone C	NA	0.18592	3.782496	1.137542	0.07078627	0.328392
80884	Maged2	MAGE family member D2, transcript variant 1	NA	0.08622	7.697989	1.061585	0.07080117	0.328392
233905	Zfp646	zinc finger protein 646, transcript variant X4	NA	-0.1494	5.50086	-1.10911	0.07080834	0.328392
100042480	Nhsl2	NHS-like 2, transcript variant X16	NA	0.179359	4.408676	1.132381	0.07081655	0.328392
71339	5430400D12Rik	RIKEN cDNA 5430400D12 gene	NA	0.420184	1.089897	1.338098	0.07084442	0.328427
14733	Gpc1	glypican 1	NA	-0.09325	7.692356	-1.06677	0.070894	0.328564
76273	Ndfip2	Nedd4 family interacting protein 2, transcript variant 1	NA	0.121604	6.129241	1.087944	0.07103107	0.329105
207304	Hectd1	HECT domain E3 ubiquitin protein ligase 1	NA	0.101492	7.189399	1.072882	0.07114514	0.329512
668382	Pabpc1l2b	poly(A) binding protein, cytoplasmic 1-like 2B	NA	-0.12595	4.841894	-1.09122	0.0711713	0.329512
74626	Tmem81	transmembrane protein 81, transcript variant X1	NA	1.003921	-0.50405	2.005443	0.07118742	0.329512
118568782	LOC118568782	ribosome biogenesis regulatory protein homolog	NA	0.485138	1.16361	1.39972	0.07119993	0.329512
214133	Tet2	tet methylcytosine dioxygenase 2, transcript variant X	NA	0.13173	5.586715	1.095607	0.07125192	0.329578
58194	Sh3kbp1	SH3-domain kinase binding protein 1, transcript varia	NA	0.096928	6.193828	1.069494	0.07125467	0.329578
17345	Mki67	antigen identified by monoclonal antibody Ki 67, trans	NA	-0.17557	6.739915	-1.12941	0.07130527	0.329668
75957	Mir17hg	Mir17 host gene (non-protein coding)	NA	-0.1867	3.955797	-1.13816	0.07132714	0.329668
57869	Adck2	aarF domain containing kinase 2	NA	-0.15553	4.626697	-1.11383	0.07133492	0.329668
118568432	LOC118568432	uncharacterized LOC118568432	NA	-1.03988	-0.1811	-2.05606	0.07138476	0.329731
20405	Sh3gl1	SH3-domain GRB2-like 1, transcript variant 1	NA	-0.1127	5.703704	-1.08125	0.07138894	0.329731
14751	Gpi1	glucose-6-phosphate isomerase 1	NA	0.127847	7.919302	1.092662	0.07143763	0.329829
319807	Nwd2	NACHT and WD repeat domain containing 2	NA	0.188072	4.725465	1.13924	0.07145087	0.329829
77980	Sbf1	SET binding factor 1, transcript variant 2	NA	-0.0942	6.973487	-1.06748	0.07150135	0.329922
69638	Enho	energy homeostasis associated	NA	0.177498	5.15968	1.13092	0.07152451	0.329922
71445	5530601H04Rik	RIKEN cDNA 5530601H04 gene	NA	-0.20164	3.183896	-1.15001	0.07153795	0.329922
12455	Ccnt1	cyclin T1, transcript variant 1	NA	0.119288	6.300109	1.086199	0.07157023	0.329922
76100	5830454E08Rik	RIKEN cDNA 5830454E08 gene	NA	0.597229	-0.17575	1.512808	0.0715724	0.329922
229905	Kyat3	kynurenine aminotransferase 3, transcript variant X1	NA	-0.34449	1.80177	-1.2697	0.07162013	0.330049
252870	Usp7	ubiquitin specific peptidase 7, transcript variant X2	NA	0.08511	7.52553	1.060769	0.0716774	0.330193
23938	Map2k5	mitogen-activated protein kinase kinase 5, transcript '	NA	0.122116	5.188393	1.08833	0.07169192	0.330193
13813	Eomes	eomesodermin, transcript variant 1	NA	-0.19139	6.209521	-1.14186	0.07177489	0.330481

66624	Spcs2	signal peptidase complex subunit 2 homolog (S. cere	NA	-0.09394	6.666588	-1.06728	0.0718468	0.330691
207393	Elfn2	leucine rich repeat and fibronectin type III, extracellul	NA	-0.11033	6.209138	-1.07947	0.07186107	0.330691
270163	Myo9a	myosin IXa, transcript variant X12	NA	0.101441	6.787149	1.072845	0.07199573	0.331097
103554	Psme4	proteasome (prosome, macropain) activator subunit 4	NA	0.104151	6.782063	1.074862	0.0720165	0.331097
69709	Ptrhd1	peptidyl-tRNA hydrolase domain containing 1	NA	0.233481	2.918764	1.175668	0.07201991	0.331097
235184	Msantd2	Myb/SANT-like DNA-binding domain containing 2	NA	-0.09842	6.336806	-1.0706	0.07203118	0.331097
319448	Fndc3a	fibronectin type III domain containing 3A, transcript va	NA	0.12678	6.628865	1.091854	0.07206537	0.331097
381379	Med19	mediator complex subunit 19	NA	0.117375	5.804106	1.08476	0.07207139	0.331097
11770	Fabp4	fatty acid binding protein 4, adipocyte	NA	-0.58088	0.398573	-1.49576	0.07210288	0.331148
414118	Zmiz1os1	Zmiz1 opposite strand 1, transcript variant X11	NA	-0.24288	3.315162	-1.18335	0.07214528	0.331235
17035	Lxn	latexin	NA	0.134931	5.164752	1.09804	0.07223033	0.331235
109294	Prex2	phosphatidylinositol-3,4,5-trisphosphate-dependent F	NA	0.223395	3.117019	1.167478	0.07223059	0.331235
52521	Zfp622	zinc finger protein 622	NA	0.131082	5.180226	1.095115	0.07225653	0.331235
382252	Bclaf3	Bclaf1 and Thrap3 family member 3, transcript varian	NA	0.136649	4.88726	1.099349	0.07226748	0.331235
230661	Tesk2	testis-specific kinase 2	NA	-0.29318	1.992851	-1.22534	0.07227001	0.331235
22718	Zfp60	zinc finger protein 60, transcript variant 2	NA	-0.1034	6.397741	-1.0743	0.07227867	0.331235
66117	Fmc1	formation of mitochondrial complex V assembly facto	NA	0.251878	3.451083	1.190756	0.07228456	0.331235
216156	Wdr18	WD repeat domain 18	NA	-0.10914	5.517983	-1.07859	0.07240225	0.331552
21817	Tgm2	transglutaminase 2, C polypeptide	NA	-0.31056	3.642228	-1.24019	0.07240708	0.331552
207278	Fchsd2	FCH and double SH3 domains 2, transcript variant X	NA	0.094404	6.890037	1.067624	0.07241488	0.331552
26445	Psmb2	proteasome (prosome, macropain) subunit, beta type	NA	0.114099	6.39609	1.082299	0.07244232	0.331584
70911	Phyhipl	phytanoyl-CoA hydroxylase interacting protein-like, tr	NA	0.114204	6.934043	1.082378	0.07262897	0.332345
140546	Eri3	exoribonuclease 3, transcript variant 3	NA	0.090357	7.004268	1.064634	0.07267169	0.332409
69104	Marchf5	membrane associated ring-CH-type finger 5, transcri	NA	0.099288	7.126831	1.071245	0.07269884	0.332409
320495	Ipcef1	interaction protein for cytohesin exchange factors 1, t	NA	0.238682	2.652091	1.179914	0.07271042	0.332409
16419	Itgb5	integrin beta 5, transcript variant 1	NA	-0.1448	4.972957	-1.10558	0.07272478	0.332409
77505	Dnhd1	dynein heavy chain domain 1	NA	-0.24487	2.61415	-1.18498	0.07279623	0.332494
118567607	LOC118567607	uncharacterized LOC118567607, transcript variant X	NA	0.378711	1.32609	1.30018	0.07280707	0.332494
66282	Tma16	translation machinery associated 16, transcript variar	NA	0.204879	3.317318	1.15259	0.07281513	0.332494
17896	Myl4	myosin, light polypeptide 4, transcript variant 2	NA	-0.39725	2.012143	-1.317	0.07282502	0.332494
320825	Samd5	sterile alpha motif domain containing 5	NA	0.483466	1.408355	1.398098	0.07285854	0.332554
13869	Erbb4	erb-b2 receptor tyrosine kinase 4	NA	0.138703	5.806629	1.100915	0.07289032	0.332606
24069	Sufu	SUFU negative regulator of hedgehog signaling, tran	NA	-0.14012	5.431572	-1.10199	0.07301055	0.333061
100503292	Gm12279	predicted gene 12279, transcript variant X1	NA	0.237129	2.704466	1.178645	0.07304105	0.333107
74711	Ttll9	tubulin tyrosine ligase-like family, member 9, transcri	NA	0.291599	2.03169	1.223996	0.07307719	0.333158
71900	Tmem106b	transmembrane protein 106B	NA	0.155593	6.243505	1.11388	0.07310267	0.333158
18432	Mybbp1a	MYB binding protein (P160) 1a	NA	-0.11824	6.641426	-1.08541	0.07312841	0.333158
15260	Hira	histone cell cycle regulator, transcript variant X1	NA	-0.1066	6.667125	-1.07669	0.07313421	0.333158
211006	Sepsecs	Sep (O-phosphoserine) tRNA:Sec (selenocysteine) tF	NA	0.17221	4.25268	1.126783	0.07317914	0.333269
57776	Ttyh1	tweety family member 1, transcript variant X3	NA	0.112357	7.440306	1.080993	0.07324826	0.333438
330914	Arhgap32	Rho GTPase activating protein 32, transcript variant 2	NA	0.103615	6.756517	1.074462	0.07325844	0.333438
68380	0610042G04Rik	RIKEN cDNA 0610042G04 gene	NA	0.467104	1.037816	1.382332	0.07327776	0.333438
403395	Clec3a	C-type lectin domain family 3, member a	NA	0.734974	-0.7272	1.664368	0.0733331	0.333597
22223	Uchl1	ubiquitin carboxy-terminal hydrolase L1	NA	0.085041	9.430908	1.060718	0.0733808	0.333721
105245375	Gm40841	predicted gene, 40841, transcript variant X1	NA	0.169597	5.426763	1.124744	0.07341524	0.333784
56055	Gtpbp2	GTP binding protein 2, transcript variant 1	NA	-0.10899	6.242672	-1.07847	0.07344222	0.33381
106014	Tafa5	TAFA chemokine like family member 5, transcript var	NA	0.106114	6.345526	1.076325	0.07346204	0.33381
20877	Aurkb	aurora kinase B	NA	-0.1778	4.270387	-1.13116	0.07355949	0.334001
269514	Fbxl4	F-box and leucine-rich repeat protein 4, transcript var	NA	0.230782	3.946851	1.173471	0.07357405	0.334001
12366	Casp2	caspase 2	NA	-0.11626	5.684618	-1.08392	0.07357956	0.334001
214240	Disp2	dispatched RND tramsporter family member 2, transc	NA	-0.10244	7.32572	-1.07359	0.0735861	0.334001
50917	Galns	galactosamine (N-acetyl)-6-sulfate sulfatase, transcri	NA	0.221356	3.06869	1.165829	0.0736782	0.334246
80517	Herpud2	HERPUD family member 2	NA	-0.11885	5.31795	-1.08587	0.07368113	0.334246
106583	Scaf8	SR-related CTD-associated factor 8	NA	0.099373	6.271347	1.071308	0.07380724	0.334724
20192	Ryr3	ryanodine receptor 3	NA	0.132016	4.973984	1.095824	0.07384899	0.334773
382090	Cep162	centrosomal protein 162, transcript variant X2	NA	-0.18729	4.427929	-1.13863	0.07386107	0.334773
19951	Rpl32	ribosomal protein L32	NA	0.09432	8.363586	1.067562	0.07387965	0.334773
66844	Ormdl2	ORM1-like 2 (S. cerevisiae), transcript variant 3	NA	-0.27646	2.662235	-1.21122	0.07393588	0.334904
52822	Rufy3	RUN and FYVE domain containing 3, transcript varia	NA	0.080163	8.77035	1.057137	0.07396475	0.334904
72440	Rhno1	RAD9-HUS1-RAD1 interacting nuclear orphan 1, trar	NA	-0.11768	5.563747	-1.08499	0.07397032	0.334904
20021	Polr2c	polymerase (RNA) II (DNA directed) polypeptide C	NA	-0.12539	5.514655	-1.0908	0.07404479	0.335148
100689703	9330133O14Rik	RIKEN cDNA 9330133O14 gene	NA	0.154122	4.142412	1.112744	0.07412272	0.335382
239420	Csmd3	CUB and Sushi multiple domains 3	NA	0.199571	4.415101	1.148356	0.07415915	0.335382
102631674	Gm28913	predicted gene 28913, transcript variant X1	NA	0.634971	0.430014	1.552907	0.07415949	0.335382
19051	Ppp1r17	protein phosphatase 1, regulatory subunit 17	NA	0.40505	1.27191	1.324134	0.07417889	0.335382
105245342	Gm40814	predicted gene, 40814	NA	-0.41666	2.465879	-1.33484	0.07423606	0.335489
11637	Ak2	adenylate kinase 2, transcript variant 2	NA	-0.13592	5.660431	-1.09879	0.07424384	0.335489
67067	Romo1	reactive oxygen species modulator 1, transcript varia	NA	0.139689	4.722123	1.101668	0.07426924	0.335511

67453	Slc25a46	solute carrier family 25, member 46, transcript variant	NA	0.098793	6.417947	1.070877	0.07430579	0.335525
13929	Amz2	archaelysin family metallopeptidase 2, transcript varia	NA	-0.09845	7.586802	-1.07062	0.07431374	0.335525
18130	Ints6	integrator complex subunit 6, transcript variant X7	NA	-0.13623	4.919143	-1.09903	0.07434277	0.335563
64580	Ndst4	N-deacetylase/N-sulfotransferase (heparin glucosami	NA	0.190381	3.662151	1.141065	0.07442404	0.335823
72567	Bclaf1	BCL2-associated transcription factor 1, transcript vari	NA	0.096535	7.645549	1.069202	0.07444258	0.335823
56193	Plek	pleckstrin	NA	0.226628	3.159115	1.170097	0.07446225	0.335823
278279	Tmtc2	transmembrane and tetratricopeptide repeat containir	NA	0.126341	4.812068	1.091522	0.07448465	0.335831
53868	Rab25	RAB25, member RAS oncogene family	NA	-0.99591	-0.75182	-1.99434	0.07454646	0.33585
71508	Zfp935	zinc finger protein 935, transcript variant 1	NA	-0.17567	3.886611	-1.12949	0.07455647	0.33585
20286	Zc3h7b	zinc finger CCCH type containing 7B	NA	-0.11654	8.446115	-1.08413	0.074579	0.33585
380928	Lmo7	LIM domain only 7, transcript variant X46	NA	0.161123	4.578849	1.118157	0.07458074	0.33585
17388	Mmp15	matrix metallopeptidase 15	NA	-0.1214	5.781389	-1.08779	0.07459198	0.33585
100503003	Gm17484	predicted gene, 17484	NA	-0.31956	2.390413	-1.24795	0.0746517	0.335931
12211	Birc6	baculoviral IAP repeat-containing 6	NA	0.116601	6.872902	1.084178	0.07467046	0.335931
72584	Cul4b	cullin 4B, transcript variant 1	NA	0.11117	6.132827	1.080104	0.07471188	0.335931
108168941	Gm46853	predicted gene, 46853	NA	0.630501	-0.10385	1.548103	0.07473056	0.335931
105244498	Gm40095	predicted gene, 40095	NA	-0.52739	0.304167	-1.44132	0.07473916	0.335931
22121	Rpl13a	ribosomal protein L13A	NA	0.09309	9.089111	1.066652	0.07476423	0.335931
12181	Bop1	block of proliferation 1	NA	-0.12157	5.586381	-1.08792	0.07477502	0.335931
19716	Bex1	brain expressed X-linked 1	NA	0.097237	6.212649	1.069723	0.07477531	0.335931
12530	Cdc25a	cell division cycle 25A	NA	-0.16522	4.93385	-1.12134	0.07487679	0.336238
118568482	LOC118568482	uncharacterized LOC118568482	NA	0.184915	3.690716	1.13675	0.07488484	0.336238
633640	Tmem267	transmembrane protein 267, transcript variant 1	NA	-0.20835	3.826483	-1.15536	0.0749446	0.336413
67869	Paip2	polyadenylate-binding protein-interacting protein 2, tr	NA	-0.08705	7.410974	-1.0622	0.07497472	0.336456
109232	Sccpdh	saccharopine dehydrogenase (putative)	NA	0.107411	6.243556	1.077293	0.07502887	0.336606
269529	Fbxo10	F-box protein 10, transcript variant 2	NA	-0.12169	5.671707	-1.08801	0.07510215	0.336842
29864	Rnf11	ring finger protein 11	NA	0.096381	6.833921	1.069088	0.07519018	0.337101
240752	Pik3c2b	phosphatidylinositol-4-phosphate 3-kinase catalytic su	NA	-0.10261	6.052305	-1.07371	0.0752013	0.337101
243867	Fbxo46	F-box protein 46	NA	-0.17762	4.360879	-1.13102	0.07525094	0.33723
232441	Rerg	RAS-like, estrogen-regulated, growth-inhibitor, transc	NA	0.309562	2.643492	1.239332	0.07531235	0.337413
330143	LOC330143	uncharacterized LOC330143	NA	0.554754	0.163818	1.468918	0.07533512	0.337414
381544	Armh1	armadillo-like helical domain containing 1	NA	0.604858	-0.0629	1.520829	0.07536247	0.337414
66089	Rmnd5b	required for meiotic nuclear division 5 homolog B	NA	0.124983	5.065224	1.090495	0.07538972	0.337414
56516	Rbms2	RNA binding motif, single stranded interacting proteir	NA	-0.1382	4.92202	-1.10053	0.0754011	0.337414
100504049	Gm15559	predicted gene 15559	NA	-0.34611	1.76168	-1.27113	0.07543727	0.337414
69195	Tmem121	transmembrane protein 121, transcript variant X1	NA	0.211305	3.82712	1.157735	0.07545377	0.337414
118568737	LOC118568737	uncharacterized LOC118568737	NA	0.476796	0.489096	1.39165	0.07547365	0.337414
216131	Trappc10	trafficking protein particle complex 10	NA	0.116657	6.347698	1.08422	0.07547854	0.337414
56219	Extl1	exostosin-like glycosyltransferase 1, transcript variant	NA	0.539845	0.31969	1.453816	0.07550169	0.337425
70599	Itprid2	ITPR interacting domain containing 2, transcript varia	NA	0.150667	4.386321	1.110082	0.07559149	0.337733
68477	Rmnd5a	required for meiotic nuclear division 5 homolog A, trai	NA	0.121866	6.200397	1.088142	0.07565077	0.337905
67894	Dennd10	DENN domain containing 10, transcript variant 5	NA	0.142543	4.856898	1.103849	0.07570489	0.338054
67119	Ccdc159	coiled-coil domain containing 159, transcript variant 2	NA	-0.46201	1.614979	-1.37746	0.07576927	0.338249
13166	Dbh	dopamine beta hydroxylase	NA	0.395621	1.11501	1.315509	0.07580423	0.338255
70495	Atp6ap2	ATPase, H+ transporting, lysosomal accessory protei	NA	0.097877	6.750539	1.070197	0.07582377	0.338255
76229	Vmn2r29	vomeronasal 2, receptor 29, transcript variant 2	NA	-0.30543	1.811104	-1.23579	0.07583299	0.338255
24075	Taf10	TATA-box binding protein associated factor 10	NA	-0.17262	4.436983	-1.1271	0.07589806	0.338365
74440	Cmip	c-Maf inducing protein, transcript variant 1	NA	-0.08358	8.678789	-1.05964	0.07591144	0.338365
59033	Slc4a8	solute carrier family 4 (anion exchanger), member 8,	NA	0.105323	6.023685	1.075735	0.07591991	0.338365
14057	Sfxn1	sideroflexin 1, transcript variant X1	NA	0.122245	6.581445	1.088427	0.07597567	0.338457
93701	Pcdhgb4	protocadherin gamma subfamily B, 4	NA	-0.21885	4.05453	-1.16381	0.0759823	0.338457
107975	Pacs1	phosphofurin acidic cluster sorting protein 1, transcrip	NA	-0.09126	6.72654	-1.0653	0.07600831	0.33848
319266	A130010J15Rik	RIKEN cDNA A130010J15 gene, transcript variant 3	NA	0.238467	3.365929	1.179738	0.07604345	0.338531
17294	Mest	mesoderm specific transcript, transcript variant X2	NA	-0.12906	11.42413	-1.09358	0.07606138	0.338531
30046	Zfp292	zinc finger protein 292, transcript variant X3	NA	0.097902	6.737879	1.070216	0.07613391	0.338624
238384	Slc24a4	solute carrier family 24 (sodium/potassium/calcium ex	NA	0.551762	0.394389	1.465875	0.07616407	0.338624
17986	Ndp	Norrie disease (pseudoglioma) (human)	NA	-0.24964	2.727199	-1.18891	0.07617262	0.338624
665845	Gm7819	predicted gene 7819	NA	1.194427	-1.34545	2.28854	0.07617614	0.338624
20510	Slc1a1	solute carrier family 1 (neuronal/epithelial high affinity	NA	0.10416	5.66529	1.074868	0.07619295	0.338624
320456	B330016D10Rik	RIKEN cDNA B330016D10 gene	NA	0.250812	2.689157	1.189877	0.07620759	0.338624
53625	B3gnt2	UDP-GlcNAc:betaGal beta-1,3-N-acetylglucosaminylt	NA	0.273253	2.896831	1.20853	0.07623627	0.338624
68524	Wipf2	WAS/WASL interacting protein family, member 2	NA	-0.10761	5.629171	-1.07745	0.07626729	0.338624
77652	Zfp955a	zinc finger protein 955A, transcript variant X1	NA	0.182344	4.936563	1.134726	0.07626957	0.338624
57816	Tesc	tescalcin, transcript variant 1	NA	-0.40728	1.257231	-1.32618	0.07634097	0.338849
80889	Tlnrd1	talin rod domain containing 1	NA	-0.12716	5.209156	-1.09214	0.07638632	0.338934
76179	Usp31	ubiquitin specific peptidase 31	NA	0.109639	5.476382	1.078958	0.07640186	0.338934
108652	Slc35b3	solute carrier family 35, member B3, transcript varian	NA	0.179099	4.445181	1.132176	0.07642678	0.338952
14672	Gna11	guanine nucleotide binding protein, alpha 11	NA	0.10176	6.954402	1.073082	0.07647677	0.339061

12497	Entpd6	ectonucleoside triphosphate diphosphohydrolase 6, t	NA	0.106936	5.557051	1.076939	0.07649864	0.339061
210544	Tbc1d31	TBC1 domain family, member 31, transcript variant X	NA	-0.18571	3.72957	-1.13737	0.07651386	0.339061
101118	Tmem168	transmembrane protein 168, transcript variant X2	NA	0.140296	4.737273	1.102131	0.07657624	0.339245
237459	Cdk17	cyclin-dependent kinase 17, transcript variant X1	NA	0.089785	6.776163	1.064212	0.07664163	0.339443
240261	Ccdc112	coiled-coil domain containing 112	NA	0.153912	5.130041	1.112582	0.07670965	0.339616
210417	Thsd7b	thrombospondin, type I, domain containing 7B	NA	0.174696	3.869875	1.128726	0.07672484	0.339616
12955	Cryab	crystallin, alpha B, transcript variant 2	NA	-0.26347	2.55494	-1.20037	0.07674339	0.339616
66240	Kcne1l	potassium voltage-gated channel, Isk-related family,	NA	0.191306	3.494759	1.141797	0.07679542	0.339754
22390	Wee1	WEE 1 homolog 1 (S. pombe), transcript variant 1	NA	-0.16905	4.109951	-1.12432	0.07688552	0.340027
83398	Ndst3	N-deacetylase/N-sulfotransferase (heparan glucosam	NA	0.151496	4.960793	1.11072	0.07689882	0.340027
70891	Spdya	speedy/RINGO cell cycle regulator family, member A	NA	0.457875	0.738898	1.373518	0.07695265	0.340172
52163	Camk1	calcium/calmodulin-dependent protein kinase I	NA	0.130136	5.267013	1.094397	0.07703145	0.340321
27054	Sec23b	SEC23 homolog B, COPII coat complex component,	NA	-0.1149	5.526791	-1.0829	0.07704672	0.340321
208518	Cep78	centrosomal protein 78	NA	0.124608	4.761646	1.090211	0.07706984	0.340321
317757	Gimap5	GTPase, IMAP family member 5	NA	0.70942	-0.16753	1.635146	0.07706991	0.340321
18693	Pick1	protein interacting with C kinase 1, transcript variant 2	NA	-0.1171	5.595344	-1.08455	0.07711545	0.340429
320020	6330415G19Rik	RIKEN cDNA 6330415G19 gene, transcript variant 1	NA	0.551188	-0.0561	1.465292	0.07714691	0.340476
27784	Commd8	COMM domain containing 8, transcript variant 1	NA	0.105876	5.729624	1.076147	0.07718388	0.340547
22169	Cmpk2	cytidine monophosphate (UMP-CMP) kinase 2, mitoc	NA	0.166575	4.027922	1.12239	0.07720606	0.340552
214459	Fnbp1l	formin binding protein 1-like, transcript variant 2	NA	0.095644	9.3745	1.068542	0.07725309	0.340593
18802	Plcd4	phospholipase C, delta 4, transcript variant 1	NA	-0.3037	2.226504	-1.2343	0.07726731	0.340593
219105	Zmym5	zinc finger, MYM-type 5, transcript variant X7	NA	0.098259	6.050514	1.070481	0.07727804	0.340593
74150	Slc35f5	solute carrier family 35, member F5, transcript varian	NA	0.138608	4.755527	1.100842	0.07737469	0.340872
208111	Zfp976	zinc finger protein 976	NA	-0.26924	2.466645	-1.20518	0.07739835	0.340872
243931	Tshz3	teashirt zinc finger family member 3, transcript varian	NA	0.113409	6.03372	1.081781	0.07740431	0.340872
70846	Ttc6	tetratricopeptide repeat domain 6, transcript variant X	NA	0.499732	0.709429	1.41395	0.07750178	0.341209
18526	Pcdh10	protocadherin 10, transcript variant 3	NA	0.129846	7.154925	1.094177	0.07755761	0.341318
232491	Pyroxd1	pyridine nucleotide-disulphide oxidoreductase domai	NA	0.14586	4.676241	1.10639	0.07756855	0.341318
57896	Krcc1	lysine-rich coiled-coil 1, transcript variant 2	NA	0.158056	4.042634	1.115782	0.077627	0.341464
53897	Gal3st1	galactose-3-O-sulfotransferase 1, transcript variant 1	NA	0.466581	1.437297	1.38183	0.07764364	0.341464
102635108	Gm32528	predicted gene, 32528	NA	0.605941	0.396386	1.521971	0.07771833	0.3417
381921	Taok2	TAO kinase 2, transcript variant X1	NA	-0.10272	7.504416	-1.0738	0.07777505	0.341857
53860	Septin9	septin 9, transcript variant 4	NA	-0.09237	7.254829	-1.06612	0.07787822	0.342185
17161	Maoa	monoamine oxidase A	NA	0.108379	5.592347	1.078017	0.07789157	0.342185
68128	Fam120aos	family with sequence similarity 120A, opposite strand	NA	0.168193	4.399084	1.12365	0.07796461	0.342413
70772	Ggnbp1	gametogenetin binding protein 1, transcript variant 1	NA	0.390284	1.669686	1.310651	0.07807063	0.342755
68327	Tsr3	TSR3 20S rRNA accumulation, transcript variant 1	NA	0.161368	4.524328	1.118347	0.07808452	0.342755
70832	4921504A21Rik	RIKEN cDNA 4921504A21 gene, transcript variant 2	NA	0.771517	-0.51061	1.707064	0.0781471	0.342937
19249	Ptpn13	protein tyrosine phosphatase, non-receptor type 13	NA	-0.13613	4.538138	-1.09895	0.07823041	0.34321
319934	Sbf2	SET binding factor 2	NA	0.111158	7.115184	1.080095	0.07835174	0.343649
234463	Tmem184c	transmembrane protein 184C, transcript variant 2	NA	-0.09965	5.913839	-1.07151	0.0784095	0.34381
207704	Gtpbp10	GTP-binding protein 10 (putative), transcript variant 1	NA	0.152696	4.686374	1.111645	0.07846336	0.343954
56349	Net1	neuroepithelial cell transforming gene 1, transcript va	NA	-0.14992	4.553933	-1.10951	0.0785027	0.343957
75276	Ppp1r1c	protein phosphatase 1, regulatory inhibitor subunit 1C	NA	0.415796	1.501023	1.334035	0.07850633	0.343957
320982	Arl4c	ADP-ribosylation factor-like 4C	NA	-0.08977	7.324417	-1.0642	0.07853098	0.343972
544717	1190007I07Rik	RIKEN cDNA 1190007I07 gene, transcript variant 1	NA	-0.35637	2.157865	-1.2802	0.07860395	0.344199
11820	App	amyloid beta (A4) precursor protein, transcript varian	NA	0.091284	9.285107	1.065318	0.07865213	0.344317
13052	Cxadr	coxsackie virus and adenovirus receptor, transcript va	NA	0.111502	8.62626	1.080352	0.07867572	0.344328
227327	B3gnt7	UDP-GlcNAc:betaGal beta-1,3-N-acetylglucosaminyl	NA	-0.26443	2.607208	-1.20117	0.0787723	0.344591
170707	Usp48	ubiquitin specific peptidase 48, transcript variant X22	NA	0.084499	6.899471	1.060319	0.07877829	0.344591
18822	Plod1	procollagen-lysine, 2-oxoglutarate 5-dioxygenase 1	NA	-0.13174	4.6049	-1.09562	0.07882873	0.344596
414801	Itprip	inositol 1,4,5-triphosphate receptor interacting protei	NA	-0.2509	3.056047	-1.18995	0.07883838	0.344596
224794	Enpp4	ectonucleotide pyrophosphatase/phosphodiesterase	NA	0.23087	3.028981	1.173543	0.07884281	0.344596
52440	Tax1bp1	Tax1 (human T cell leukemia virus type I) binding pro	NA	0.082165	7.089359	1.058605	0.07889521	0.344622
115488283	LOC115488283	uncharacterized LOC115488283, transcript variant X	NA	0.788844	0.293877	1.72769	0.07891408	0.344622
56758	Mbnl1	muscleblind like splicing factor 1, transcript variant X	NA	0.142568	5.257065	1.103868	0.07893685	0.344622
11881	Arsb	arylsulfatase B	NA	0.154603	4.948586	1.113115	0.07893743	0.344622
108168785	G630016G05Rik	RIKEN cDNA G630016G05 gene	NA	0.619666	0.176057	1.536519	0.07897386	0.344622
74125	Armc8	armadillo repeat containing 8, transcript variant 2	NA	0.113334	6.474073	1.081726	0.07897586	0.344622
13824	Epb41l4a	erythrocyte membrane protein band 4.1 like 4a, trans	NA	-0.1081	5.406843	-1.07781	0.07902493	0.344744
69823	Fyttd1	forty-two-three domain containing 1, transcript varian	NA	0.1209	6.870048	1.087413	0.0790619	0.344781
52683	Ncaph2	non-SMC condensin II complex, subunit H2, transcrip	NA	-0.10372	6.684027	-1.07454	0.07910643	0.344781
54601	Foxo4	forkhead box O4	NA	-0.15154	4.815163	-1.11075	0.07911866	0.344781
70808	4632415L05Rik	RIKEN cDNA 4632415L05 gene	NA	0.165947	4.18081	1.121902	0.07913205	0.344781
320939	5930403L14Rik	RIKEN cDNA 5930403L14 gene	NA	-0.19637	4.494097	-1.14581	0.0791811	0.344781
219024	Pip4p1	phosphatidylinositol-4,5-bisphosphate 4-phosphatase	NA	0.128823	5.368585	1.093401	0.07918167	0.344781
27276	Plekhb1	pleckstrin homology domain containing, family B (eve	NA	0.218431	3.084811	1.163468	0.07920007	0.344781
100503185	Btbd8	BTB (POZ) domain containing 8, transcript variant 3	NA	0.112179	5.286019	1.080859	0.07920308	0.344781

68744	Zfp740	zinc finger protein 740, transcript variant 1	NA	-0.09629	7.09448	-1.06902	0.07922458	0.344783
110816	Pwp2	PWP2 periodic tryptophan protein homolog (yeast)	NA	-0.12653	4.944722	-1.09167	0.07924624	0.344785
228778	6820408C15Rik	RIKEN cDNA 6820408C15 gene, transcript variant 6	NA	-0.41424	0.85093	-1.33259	0.07930568	0.344785
73447	Wdr13	WD repeat domain 13, transcript variant X1	NA	0.093911	7.590203	1.06726	0.07931562	0.344785
14042	Ext1	exostosin glycosyltransferase 1	NA	0.135538	5.263111	1.098502	0.07931628	0.344785
103199	Fig4	FIG4 phosphoinositide 5-phosphatase	NA	0.113343	5.218012	1.081732	0.0793615	0.344785
74270	Usp20	ubiquitin specific peptidase 20, transcript variant X1	NA	-0.11717	5.488281	-1.08461	0.07939382	0.344785
21991	Tpi1	triosephosphate isomerase 1	NA	0.119115	7.853413	1.086068	0.07941432	0.344785
107477	Guca1b	guanylate cyclase activator 1B, transcript variant X1	NA	-0.66673	-0.38472	-1.58747	0.07945615	0.344785
620016	Gm12669	predicted gene 12669	NA	0.234882	4.411773	1.17681	0.07946623	0.344785
14158	Fer	fer (fms/fps related) protein kinase, transcript variant	NA	-0.13499	5.694661	-1.09809	0.07948622	0.344785
227197	Ndufs1	NADH:ubiquinone oxidoreductase core subunit S1, tr	NA	0.091973	6.631156	1.065827	0.07949865	0.344785
70620	Ube2v2	ubiquitin-conjugating enzyme E2 variant 2, transcript	NA	0.089329	7.034616	1.063875	0.07950064	0.344785
108837	Ibtk	inhibitor of Bruton agammaglobulinemia tyrosine kina	NA	0.104394	5.8685	1.075043	0.07955157	0.344785
13998	Fgd6	FYVE, RhoGEF and PH domain containing 6	NA	0.175398	3.753401	1.129276	0.07955272	0.344785
11668	Aldh1a1	aldehyde dehydrogenase family 1, subfamily A1, tran	NA	0.232078	3.140577	1.174526	0.07955598	0.344785
68153	Gtf2e2	general transcription factor II E, polypeptide 2 (beta s	NA	-0.11923	5.274394	-1.08615	0.07956403	0.344785
115489917	Gm52688	predicted gene, 52688	NA	0.728502	-0.57623	1.656918	0.07969164	0.345177
106821	Oard1	O-acyl-ADP-ribose deacylase 1, transcript variant 5	NA	0.158189	4.474148	1.115885	0.07969714	0.345177
170936	Zfp369	zinc finger protein 369, transcript variant X3	NA	-0.11533	5.801175	-1.08322	0.0798147	0.345595
100043381	Gm14308	predicted gene 14308, transcript variant X4	NA	-1.83782	1.689989	-3.57468	0.07985016	0.345631
15460	Hr	lysine demethylase and nuclear receptor corepressor	NA	-0.27862	3.119524	-1.21304	0.07987006	0.345631
67063	Pgap4	post-GPI attachment to proteins GalNAc transferase	NA	0.111727	5.837343	1.080521	0.07988677	0.345631
24051	Sgcb	sarcoglycan, beta (dystrophin-associated glycoprotei	NA	0.137727	5.298213	1.10017	0.07993399	0.345677
23857	Dmtf1	cyclin D binding myb-like transcription factor 1, transc	NA	-0.09672	6.357012	-1.06934	0.07993992	0.345677
11836	Araf	Araf proto-oncogene, serine/threonine kinase, transc	NA	0.091405	7.317238	1.065407	0.08001919	0.345754
20970	Sdc3	syndecan 3, transcript variant X4	NA	-0.09544	9.23844	-1.06839	0.08003759	0.345754
20663	Sos2	SOS Ras/Rho guanine nucleotide exchange factor 2,	NA	0.113646	5.458388	1.081959	0.08004159	0.345754
226999	Slc9a2	solute carrier family 9 (sodium/hydrogen exchanger),	NA	0.609592	-0.11883	1.525828	0.08004279	0.345754
433485	Syndig1	synapse differentiation inducing 1, transcript variant 4	NA	0.2216	3.618304	1.166026	0.08018157	0.345983
70088	Meaf6	MYST/Esa1-associated factor 6, transcript variant 4	NA	-0.09818	7.223602	-1.07042	0.08018615	0.345983
26414	Mapk10	mitogen-activated protein kinase 10, transcript varian	NA	0.083865	7.233906	1.059853	0.08021231	0.345983
233328	Lrrk1	leucine-rich repeat kinase 1, transcript variant X1	NA	-0.2619	2.674198	-1.19905	0.08022004	0.345983
71963	Cdca4	cell division cycle associated 4, transcript variant 2	NA	-0.12351	4.943527	-1.08938	0.08025614	0.345983
100134990	Selenok-ps1	selenoprotein K, pseudogene 1	NA	0.560866	0.060138	1.475155	0.08029667	0.345983
15586	Hyal1	hyaluronoglucosaminidase 1, transcript variant 2	NA	-0.23879	2.74147	-1.18001	0.08031698	0.345983
22755	Zfp93	zinc finger protein 93, transcript variant 2	NA	-0.17967	3.62812	-1.13263	0.08035665	0.345983
74256	Cyld	CYLD lysine 63 deubiquitinase, transcript variant X23	NA	0.14334	5.228293	1.104459	0.08036842	0.345983
15574	Hus1	HUS1 checkpoint clamp component, transcript varian	NA	0.156629	3.900083	1.114679	0.08036888	0.345983
72124	Seh1l	SEH1-like (S. cerevisiae, transcript variant 1	NA	0.126179	5.94756	1.0914	0.08040877	0.345983
76781	Mettl4	methyltransferase like 4, transcript variant 3	NA	-0.1276	5.102096	-1.09247	0.08042593	0.345983
13728	Mark2	MAP/microtubule affinity regulating kinase 2, transcri	NA	-0.09012	6.501999	-1.06446	0.08043112	0.345983
77644	C330007P06Rik	RIKEN cDNA C330007P06 gene	NA	-0.118	4.946737	-1.08523	0.08043738	0.345983
319545	D430020J02Rik	RIKEN cDNA D430020J02 gene	NA	0.310767	1.883911	1.240367	0.08046258	0.345983
107392	Brms1	breast cancer metastasis-suppressor 1, transcript var	NA	-0.1427	4.1751	-1.10397	0.08047727	0.345983
433367	Gm5532	predicted gene 5532	NA	-0.53148	-0.02076	-1.44541	0.08048065	0.345983
66309	Tmem128	transmembrane protein 128, transcript variant 1	NA	0.141999	5.145927	1.103433	0.08048451	0.345983
17347	Mknk2	MAP kinase-interacting serine/threonine kinase 2	NA	-0.12754	6.244729	-1.09243	0.08049983	0.345983
73102	Slc22a23	solute carrier family 22, member 23, transcript varian	NA	0.087173	6.695236	1.062287	0.08052485	0.346
70097	Sash1	SAM and SH3 domain containing 1, transcript variant	NA	0.163841	5.406751	1.120266	0.08054758	0.346006
18211	Ntrk1	neurotrophic tyrosine kinase, receptor, type 1	NA	0.640256	0.915086	1.558606	0.08064084	0.34623
380629	Heca	hdc homolog, cell cycle regulator, transcript variant X	NA	0.128731	5.80484	1.093332	0.08064234	0.34623
53902	Rcan3	regulator of calcineurin 3	NA	0.097801	6.304046	1.070141	0.0806687	0.346252
242406	Rgp1	RAB6A GEF compex partner 1	NA	-0.15284	5.888371	-1.11176	0.08078783	0.346672
14919	Gucy2e	guanylate cyclase 2e, transcript variant X2	NA	-0.5322	0.005982	-1.44613	0.080919	0.347143
16918	Mycl	v-myc avian myelocytomatosis viral oncogene lung c	NA	0.092998	6.840935	1.066584	0.08095627	0.347211
30951	Cbx8	chromobox 8	NA	-0.20156	3.518345	-1.14994	0.08098213	0.347231
320709	Tmem117	transmembrane protein 117	NA	0.250397	3.136741	1.189535	0.08103487	0.347291
434179	Zfp975	zinc finger protein 975	NA	-0.2321	3.02492	-1.17454	0.08103885	0.347291
231130	Tnip2	TNFAIP3 interacting protein 2	NA	0.265255	2.207453	1.201849	0.08111177	0.347392
22343	Lin7c	lin-7 homolog C (C. elegans)	NA	0.117644	8.175509	1.084962	0.0811402	0.347392
237930	Ttll6	tubulin tyrosine ligase-like family, member 6	NA	-0.4682	0.45792	-1.38338	0.08115478	0.347392
58240	Hs1bp3	HCLS1 binding protein 3	NA	-0.20516	3.645602	-1.15281	0.08115512	0.347392
102637100	Gm26911	predicted gene, 26911, transcript variant X3	NA	0.393237	1.231899	1.313337	0.08118095	0.347392
226178	Wbp1l	WW domain binding protein 1 like, transcript variant 2	NA	-0.1037	6.220667	-1.07452	0.08119409	0.347392
52064	Coq5	coenzyme Q5 methyltransferase	NA	-0.13437	4.898518	-1.09761	0.08121181	0.347392
74549	Mau2	MAU2 sister chromatid cohesion factor, transcript var	NA	0.086498	7.28811	1.06179	0.08134977	0.347891
15185	Hdac6	histone deacetylase 6, transcript variant X3	NA	-0.10447	6.517515	-1.0751	0.0813858	0.347953

12857	Cox4i1	cytochrome c oxidase subunit 4I1, transcript variant 1	NA	0.097926	7.855736	1.070234	0.08144436	0.348044
67657	Rabl3	RAB, member RAS oncogene family-like 3	NA	0.185848	3.535738	1.137485	0.08144979	0.348044
16648	Kpna3	karyopherin (importin) alpha 3	NA	0.113074	6.929596	1.08153	0.08155444	0.3484
102639066	Gm35469	predicted gene, 35469, transcript variant X1	NA	-0.44896	0.627965	-1.36505	0.08160758	0.348535
73122	Tgfbrap1	transforming growth factor, beta receptor associated	NA	0.106585	5.981103	1.076676	0.08163788	0.348566
73075	Ppil6	peptidylprolyl isomerase (cyclophilin)-like 6	NA	0.453341	0.503797	1.369207	0.0817071	0.348566
56280	Mrpl37	mitochondrial ribosomal protein L37	NA	-0.11535	5.355042	-1.08324	0.08170853	0.348566
98910	Usp6nl	USP6 N-terminal like, transcript variant X5	NA	0.120185	5.56679	1.086874	0.08171455	0.348566
57815	Spata5	spermatogenesis associated 5, transcript variant X3	NA	-0.15454	4.341515	-1.11306	0.08172352	0.348566
192285	Phf21a	PHD finger protein 21A, transcript variant 1	NA	-0.11724	6.696056	-1.08466	0.0817876	0.348566
328967	Arhgef37	Rho guanine nucleotide exchange factor (GEF) 37, tr	NA	0.275603	2.445179	1.2105	0.08180801	0.348566
114679	Selenom	selenoprotein M	NA	0.22485	4.0754	1.168656	0.08184188	0.348566
108115	Slco4a1	solute carrier organic anion transporter family, memb	NA	0.422848	0.866677	1.340571	0.08185817	0.348566
232821	Ccdc106	coiled-coil domain containing 106, transcript variant 1	NA	0.129437	4.641091	1.093867	0.08185913	0.348566
227867	Epc2	enhancer of polycomb homolog 2, transcript variant X	NA	0.124247	6.41979	1.089939	0.081862	0.348566
72881	Zdhhc4	zinc finger, DHHC domain containing 4, transcript var	NA	0.208193	4.469248	1.155241	0.08187196	0.348566
240776	Kcnt2	potassium channel, subfamily T, member 2, transcrip	NA	0.226762	2.952224	1.170206	0.08189748	0.348584
67702	Rnf149	ring finger protein 149, transcript variant X2	NA	0.151049	4.686353	1.110377	0.08207829	0.349218
69192	Dhx16	DEAH (Asp-Glu-Ala-His) box polypeptide 16	NA	-0.10988	5.390915	-1.07914	0.082102	0.349218
94186	Strn3	striatin, calmodulin binding protein 3, transcript variar	NA	0.105087	6.744856	1.075559	0.08213549	0.349218
76820	Cyria	CYFIP related Rac1 interactor A, transcript variant X2	NA	0.095807	7.682605	1.068663	0.08214343	0.349218
75292	Prkd3	protein kinase D3, transcript variant 3	NA	0.11204	5.666886	1.080755	0.08217498	0.349218
195522	Zfp691	zinc finger protein 691, transcript variant 2	NA	-0.22549	2.66808	-1.16918	0.08217534	0.349218
68738	Acss1	acyl-CoA synthetase short-chain family member 1	NA	-0.13329	4.797983	-1.09679	0.08220326	0.349226
223752	Gramd4	GRAM domain containing 4, transcript variant 1	NA	-0.10199	6.688173	-1.07326	0.08222017	0.349226
170829	Tram2	translocating chain-associating membrane protein 2	NA	-0.56562	0.017831	-1.48002	0.08231609	0.349543
216618	Cfap36	cilia and flagella associated protein 36	NA	0.096397	6.027651	1.0691	0.08236527	0.349632
22143	Tuba1b	tubulin, alpha 1B	NA	-0.082	9.632849	-1.05849	0.08238006	0.349632
217127	Kat7	K(lysine) acetyltransferase 7, transcript variant X2	NA	0.098684	6.374498	1.070796	0.08243783	0.349786
76740	Efr3a	EFR3 homolog A, transcript variant X1	NA	0.127388	5.35066	1.092314	0.08250148	0.349965
30928	Zbtb18	zinc finger and BTB domain containing 18, transcript	NA	0.094048	8.71298	1.067361	0.08256775	0.350024
17116	Mab21l1	mab-21-like 1	NA	0.132242	6.047333	1.095996	0.08257641	0.350024
228787	Xkr7	X-linked Kx blood group related 7, transcript variant X	NA	-0.13135	6.307107	-1.09532	0.0825867	0.350024
13138	Dag1	dystroglycan 1, transcript variant 5	NA	0.087295	7.193492	1.062376	0.08262369	0.350024
266781	Snx17	sorting nexin 17	NA	-0.0958	6.211621	-1.06866	0.0826362	0.350024
241324	Crb2	crumbs family member 2	NA	-0.18894	4.072691	-1.13993	0.08264463	0.350024
68554	Cebpzos	CCAAT/enhancer binding protein (C/EBP), zeta, oppo	NA	0.182996	3.90465	1.135239	0.08281704	0.350663
68098	Rchy1	ring finger and CHY zinc finger domain containing 1,	NA	0.105443	5.584339	1.075825	0.08289309	0.350747
12960	Crybb1	crystallin, beta B1, transcript variant X1	NA	0.501453	0.38924	1.415639	0.08289791	0.350747
19341	Rab4a	RAB4A, member RAS oncogene family	NA	0.132582	5.148908	1.096254	0.08290144	0.350747
101056047	Gm28455	predicted gene 28455, transcript variant 2	NA	0.208912	3.078002	1.155816	0.08293991	0.350818
268902	Robo2	roundabout guidance receptor 2, transcript variant X6	NA	0.089502	7.791475	1.064003	0.08309068	0.351296
12905	Cradd	CASP2 and RIPK1 domain containing adaptor with de	NA	-0.21707	2.952118	-1.16237	0.0830959	0.351296
381104	Prickle4	prickle planar cell polarity protein 4	NA	0.277897	2.334277	1.212426	0.08313847	0.351305
667742	Piezo2	piezo-type mechanosensitive ion channel component	NA	0.24867	3.0374	1.188111	0.08314122	0.351305
56535	Pex3	peroxisomal biogenesis factor 3, transcript variant 1	NA	0.126114	4.821341	1.09135	0.08325945	0.351713
214952	Rhot2	ras homolog family member T2, transcript variant 1	NA	-0.10128	5.628087	-1.07273	0.08328683	0.351737
72555	Shisa9	shisa family member 9, transcript variant 2	NA	0.119079	5.335293	1.086041	0.08339816	0.35195
98221	Eif3m	eukaryotic translation initiation factor 3, subunit M	NA	0.10104	6.690927	1.072546	0.08340028	0.35195
101985	Usb1	U6 snRNA biogenesis 1	NA	-0.13265	4.595317	-1.0963	0.083402	0.35195
12319	Car8	carbonic anhydrase 8	NA	0.229897	3.995079	1.172752	0.08349537	0.352252
22393	Wfs1	wolframin ER transmembrane glycoprotein	NA	-0.17728	3.49727	-1.13075	0.08352694	0.352294
74183	Perm1	PPARGC1 and ESRR induced regulator, muscle 1, tr	NA	-0.38515	1.204839	-1.306	0.08364259	0.35269
14199	Fhl1	four and a half LIM domains 1, transcript variant X1	NA	0.085577	7.254827	1.061112	0.0837065	0.352868
70012	Cep85	centrosomal protein 85	NA	-0.14555	4.075262	-1.10615	0.08377338	0.352981
15331	Hmgn2	high mobility group nucleosomal binding domain 2	NA	-0.08479	9.086136	-1.06053	0.08377654	0.352981
76376	Slc24a2	solute carrier family 24 (sodium/potassium/calcium ex	NA	0.217737	3.238519	1.162908	0.08381378	0.353046
21351	Taldo1	transaldolase 1	NA	-0.12619	6.859323	-1.09141	0.08388296	0.353159
102635939	Gm33148	predicted gene, 33148, transcript variant X3	NA	0.587628	1.502036	1.502774	0.08390944	0.353159
224432	Scaf4	SR-related CTD-associated factor 4, transcript varian	NA	-0.1167	6.096289	-1.08426	0.08392285	0.353159
68512	Tomm5	translocase of outer mitochondrial membrane 5, trans	NA	0.136591	4.447739	1.099304	0.0839463	0.353159
21886	Tle2	transducin-like enhancer of split 2, transcript variant X	NA	-0.13732	4.85947	-1.09986	0.08396444	0.353159
14017	Evi2a	ecotropic viral integration site 2a, transcript variant 2	NA	0.461297	0.53149	1.376779	0.084022	0.353159
68617	Mtcl1	microtubule crosslinking factor 1, transcript variant 1	NA	-0.10097	5.651269	-1.0725	0.08407809	0.353159
211896	Depdc7	DEP domain containing 7	NA	-0.49948	0.380441	-1.41371	0.08408181	0.353159
14082	Fadd	Fas (TNFRSF6)-associated via death domain	NA	-0.20127	3.187831	-1.14971	0.0840914	0.353159
58246	Slc35b4	solute carrier family 35, member B4	NA	-0.12147	5.434	-1.08785	0.08409461	0.353159
225207	Zfp521	zinc finger protein 521, transcript variant 1	NA	0.096658	6.258417	1.069294	0.08413505	0.353159

240168	Rasgrp3	RAS, guanyl releasing protein 3, transcript variant 3	NA		0.200041	3.267167	1.148731	0.08417873	0.353159
102639048	Gm35456	predicted gene, 35456, transcript variant X4	NA		-0.50473	0.400596	-1.41885	0.08426044	0.353159
107829	Thoc5	THO complex 5, transcript variant X1	NA		-0.103	5.973211	-1.07401	0.08427308	0.353159
67898	Pef1	penta-EF hand domain containing 1	NA		0.1271	4.939139	1.092097	0.08427421	0.353159
98363	Efhd1	EF hand domain containing 1, transcript variant 1	NA		0.525085	1.062475	1.439018	0.08427908	0.353159
13121	Cyp51	cytochrome P450, family 51	NA		0.094137	7.581545	1.067427	0.08428524	0.353159
225912	Cyb561a3	cytochrome b561 family, member A3, transcript varia	NA		-0.19009	3.917053	-1.14083	0.08430231	0.353159
20462	Tra2b	transformer 2 beta, transcript variant X1	NA		0.082964	7.993202	1.059192	0.08430813	0.353159
28250	Slco1a4	solute carrier organic anion transporter family, memb	NA		-0.3188	1.972991	-1.24729	0.08431647	0.353159
72650	2810006K23Rik	RIKEN cDNA 2810006K23 gene, transcript variant 1	NA		-0.19358	3.573696	-1.1436	0.08431988	0.353159
101772	Ano1	anoctamin 1, calcium activated chloride channel, tran	NA		-0.2264	3.485447	-1.16991	0.08433464	0.353159
76614	Immt	inner membrane protein, mitochondrial, transcript var	NA		0.095976	7.031211	1.068788	0.08435261	0.353159
232976	Zfp574	zinc finger protein 574, transcript variant X1	NA		-0.1357	4.951288	-1.09862	0.08437463	0.353159
66841	Etfdh	electron transferring flavoprotein, dehydrogenase	NA		0.115596	5.085754	1.083423	0.08438902	0.353159
74396	4933407K13Rik	RIKEN cDNA 4933407K13 gene	NA		-0.25351	2.697122	-1.1921	0.08440493	0.353159
71745	Cul2	cullin 2, transcript variant X2	NA		0.106679	6.233394	1.076747	0.08443366	0.353189
331461	Il1rapl1	interleukin 1 receptor accessory protein-like 1	NA		0.277102	2.745371	1.211759	0.08447616	0.353247
69077	Psmd11	proteasome (prosome, macropain) 26S subunit, non-	NA		-0.1018	6.939021	-1.07311	0.08449108	0.353247
70325	Pigw	phosphatidylinositol glycan anchor biosynthesis, clas	NA		-0.22831	2.893393	-1.17146	0.08454123	0.353366
57810	Cdon	cell adhesion molecule-related/down-regulated by on	NA		-0.12397	6.327058	-1.08973	0.08460727	0.353475
19891	Rpa2	replication protein A2	NA		-0.13216	4.571478	-1.09594	0.08461058	0.353475
56397	Morf4l2	mortality factor 4 like 2, transcript variant 6	NA		0.084542	8.665574	1.060351	0.0846474	0.353538
101544	Zfp575	zinc finger protein 575, transcript variant X1	NA		0.192054	3.772261	1.142389	0.08469649	0.353652
13498	Atn1	atrophin 1	NA		-0.08858	8.007996	-1.06333	0.08471931	0.353656
78830	Slc25a12	solute carrier family 25 (mitochondrial carrier, Aralar),	NA		0.095845	6.412078	1.068691	0.08475212	0.353703
27205	Podxl	podocalyxin-like	NA		-0.10771	5.819801	-1.07752	0.0848202	0.353896
100040852	Gm3002	predicted gene 3002	NA		0.21148	5.44381	1.157876	0.08490587	0.35415
15205	Hes1	hes family bHLH transcription factor 1	NA		-0.19951	3.691336	-1.14831	0.08493828	0.35415
241624	Exd1	exonuclease 3'-5' domain containing 1, transcript vari	NA		0.425068	0.615352	1.342636	0.08494635	0.35415
12745	Clgn	calmegin, transcript variant 2	NA		0.154248	4.05993	1.112841	0.08501258	0.354335
66427	Cyb5b	cytochrome b5 type B	NA		0.081143	7.473372	1.057856	0.08516914	0.354897
241134	Nyap2	neuronal tyrosine-phophorylated phosphoinositide 3-I	NA		0.122345	5.180774	1.088503	0.08521108	0.354922
68563	Dpm3	dolichyl-phosphate mannosyltransferase polypeptide	NA		0.246436	2.335797	1.186273	0.08522488	0.354922
100041151	Gm3636	predicted gene 3636	NA		0.124599	5.034559	1.090205	0.08524054	0.354922
72749	Tonsl	tonsoku-like, DNA repair protein	NA		-0.18012	3.609994	-1.13298	0.08530198	0.355001
12005	Axin1	axin 1, transcript variant 1	NA		-0.10437	5.76224	-1.07503	0.08530333	0.355001
170731	Mfn2	mitofusin 2, transcript variant 2	NA		-0.10136	6.995496	-1.07278	0.08534806	0.355021
237730	Fbll1	fibrillarin-like 1	NA		0.171661	4.548657	1.126354	0.08536819	0.355021
18626	Per1	period circadian clock 1, transcript variant 2	NA		-0.1112	5.247646	-1.08012	0.08537415	0.355021
102637563	Gm34340	predicted gene, 34340, transcript variant X2	NA		0.69079	-0.32936	1.614167	0.08540988	0.355021
52502	Carhsp1	calcium regulated heat stable protein 1, transcript var	NA		-0.08334	7.29897	-1.05947	0.08546084	0.355021
50874	Tmod4	tropomodulin 4	NA		-0.685	0.410168	-1.60771	0.08547048	0.355021
77963	Hook1	hook microtubule tethering protein 1, transcript variar	NA		0.140617	5.991528	1.102377	0.08552478	0.355021
217370	Cybc1	cytochrome b 245 chaperone 1, transcript variant 5	NA		-0.11666	5.134117	-1.08422	0.08553472	0.355021
208228	Mob3a	MOB kinase activator 3A	NA		-0.12663	5.626166	-1.09174	0.08555188	0.355021
21804	Tgfb1i1	transforming growth factor beta 1 induced transcript 1	NA		-0.14927	3.922079	-1.10901	0.0855593	0.355021
230815	Man1c1	mannosidase, alpha, class 1C, member 1, transcript	NA		-0.16071	4.419889	-1.11784	0.08557298	0.355021
56291	Styx	serine/threonine/tyrosine interaction protein	NA		0.138796	5.645327	1.100986	0.08558374	0.355021
20401	Sh3bp1	SH3-domain binding protein 1, transcript variant 1	NA		-0.2551	2.385104	-1.19342	0.08559172	0.355021
20624	Eftud2	elongation factor Tu GTP binding domain containing :	NA		-0.08719	6.945196	-1.0623	0.0856358	0.355114
74453	Cfap53	cilia and flagella associated protein 53, transcript vari	NA		0.444756	0.516186	1.361084	0.0857419	0.355391
109115	Supt3	SPT3, SAGA and STAGA complex component, trans	NA		0.181314	3.86798	1.133916	0.08574643	0.355391
27386	Npas3	neuronal PAS domain protein 3, transcript variant 2	NA		0.123681	6.238508	1.089511	0.08578162	0.355447
93722	Pcdhga10	protocadherin gamma subfamily A, 10	NA		-0.15109	4.86912	-1.11041	0.085983	0.356153
209268	Igsf1	immunoglobulin superfamily, member 1, transcript va	NA		0.229504	3.438984	1.172432	0.08599584	0.356153
211482	Efhb	EF hand domain family, member B, transcript variant	NA		0.33368	1.347587	1.260224	0.08603655	0.356199
18145	Npc1	NPC intracellular cholesterol transporter 1	NA		0.105886	5.613888	1.076155	0.08605983	0.356199
230737	Gnl2	guanine nucleotide binding protein-like 2 (nucleolar)	NA		0.121531	6.100215	1.087889	0.08607408	0.356199
66246	Osgep	O-sialoglycoprotein endopeptidase	NA		-0.11611	5.044351	-1.0838	0.08611559	0.356199
67979	Atad1	ATPase family, AAA domain containing 1, transcript \	NA		0.097662	6.991095	1.070038	0.08614934	0.356199
67200	Ccdc77	coiled-coil domain containing 77, transcript variant X8	NA		-0.21836	3.314877	-1.16341	0.08617445	0.356199
20678	Sox5	SRY (sex determining region Y)-box 5, transcript vari	NA		-0.12527	6.815604	-1.09071	0.08618876	0.356199
244668	Sipa1l2	signal-induced proliferation-associated 1 like 2, trans	NA		-0.10849	6.424569	-1.0781	0.0861975	0.356199
69126	Rbis	ribosomal biogenesis factor, transcript variant 2	NA		0.123982	5.639141	1.089739	0.08620413	0.356199
231532	Arhgap24	Rho GTPase activating protein 24, transcript variant :	NA		0.224701	3.402097	1.168535	0.08629995	0.356505
22256	Ung	uracil DNA glycosylase, transcript variant 2	NA		-0.19831	3.136828	-1.14735	0.0863907	0.356698
54380	Smarcal1	SWI/SNF related matrix associated, actin dependent	NA		-0.11091	5.92528	-1.07991	0.08640358	0.356698
227231	Cps1	carbamoyl-phosphate synthetase 1, transcript variant	NA		0.573823	-0.22198	1.488463	0.08642647	0.356698

50781	Dkk3	dickkopf WNT signaling pathway inhibitor 3, transcrip	NA	0.152875	4.952785	1.111783	0.08643427	0.356698
21385	Tbx2	T-box 2	NA	-0.22493	3.129901	-1.16872	0.08651592	0.356882
12565	Cdh9	cadherin 9	NA	0.222325	3.424688	1.166612	0.08652285	0.356882
229714	Gpr61	G protein-coupled receptor 61	NA	-0.26493	2.331099	-1.20158	0.08667447	0.357417
102631889	Gm16638	predicted gene, 16638, transcript variant 1	NA	-0.26199	2.613009	-1.19913	0.08674844	0.357494
11370	Acadvl	acyl-Coenzyme A dehydrogenase, very long chain	NA	-0.11811	5.055216	-1.08531	0.08676651	0.357494
68295	Aar2	AAR2 splicing factor homolog, transcript variant X1	NA	0.121103	5.274147	1.087566	0.086771	0.357494
22670	Trim26	tripartite motif-containing 26, transcript variant 4	NA	-0.13005	5.295004	-1.09433	0.08678117	0.357494
105988	Espl1	extra spindle pole bodies 1, separase, transcript varia	NA	-0.18534	3.675343	-1.13709	0.08682224	0.35751
17263	Meg3	maternally expressed 3, transcript variant 1	NA	0.103611	11.65869	1.07446	0.08682879	0.35751
11957	Atp5j	ATP synthase, H+ transporting, mitochondrial F0 com	NA	0.092446	6.532202	1.066176	0.08686986	0.35758
73419	Armt1	acidic residue methyltransferase 1	NA	-0.17827	3.617067	-1.13153	0.08688974	0.35758
14933	Gk	glycerol kinase, transcript variant X1	NA	0.164509	3.91438	1.120784	0.08692196	0.357622
102640806	Gm36787	predicted gene, 36787, transcript variant X1	NA	0.528855	-0.11298	1.442784	0.08704308	0.35803
74451	Pgs1	phosphatidylglycerophosphate synthase 1	NA	-0.12478	5.232165	-1.09034	0.08707831	0.358084
353208	Zfp931	zinc finger protein 931, transcript variant X2	NA	-0.2306	3.046825	-1.17332	0.08720723	0.358501
239606	Slc2a13	solute carrier family 2 (facilitated glucose transporter)	NA	0.163683	4.330528	1.120143	0.08722386	0.358501
68631	Cryl1	crystallin, lambda 1	NA	0.276066	2.524608	1.210889	0.08726364	0.358574
17691	Sik1	salt inducible kinase 1	NA	-0.10112	5.905163	-1.07261	0.08730432	0.358651
21463	Tcp11	t-complex protein 11, transcript variant 1	NA	0.447217	0.656803	1.363407	0.08743561	0.3591
19876	Robo1	roundabout guidance receptor 1	NA	0.102825	7.680811	1.073874	0.0874927	0.359156
101861	Ints4	integrator complex subunit 4, transcript variant 1	NA	-0.118	5.499761	-1.08523	0.08749351	0.359156
56716	Mlst8	MTOR associated protein, LST8 homolog (S. cerevis	NA	-0.13281	5.083449	-1.09643	0.08752719	0.359204
69641	Wdr20	WD repeat domain 20, transcript variant 1	NA	0.140474	4.817433	1.102267	0.08763311	0.359548
104692	Gm12108	predicted gene 12108	NA	0.466534	0.958861	1.381785	0.08767456	0.359627
16558	Kif16b	kinesin family member 16B, transcript variant X6	NA	0.173584	3.430336	1.127857	0.08773601	0.359788
20416	Shc1	src homology 2 domain-containing transforming prote	NA	-0.16158	4.683293	-1.11851	0.08776099	0.3598
14257	Flt4	FMS-like tyrosine kinase 4	NA	-0.22856	2.858532	-1.17167	0.08780653	0.359896
100302730	5830417I10Rik	RIKEN cDNA 5830417I10 gene	NA	-0.17527	5.033407	-1.12918	0.08783742	0.359925
12064	Bdnf	brain derived neurotrophic factor, transcript variant 3	NA	0.255387	2.625523	1.193656	0.0878579	0.359925
68421	Lmbrd1	LMBR1 domain containing 1, transcript variant 2	NA	0.114742	5.732883	1.082781	0.08796072	0.360256
414101	E130317F20Rik	RIKEN cDNA E130317F20 gene	NA	0.223831	2.827018	1.16783	0.08799574	0.360309
212307	Mapre2	microtubule-associated protein, RP/EB family, memb	NA	0.076843	8.488243	1.054707	0.0880324	0.360368
207818	Smagp	small cell adhesion glycoprotein, transcript variant 2	NA	-0.46897	0.87287	-1.38412	0.08807665	0.360449
215378	Brinp3	bone morphogenetic protein/retinoic acid inducible ne	NA	0.159104	4.573812	1.116594	0.08809654	0.360449
331424	Cdr1os	cerebellar degeneration related antigen 1, opposite st	NA	0.325897	1.346993	1.253443	0.08815032	0.360547
72543	Mvb12b	multivesicular body subunit 12B, transcript variant X1	NA	-0.10244	8.100132	-1.07359	0.08816482	0.360547
229599	Ciart	circadian associated repressor of transcription	NA	0.394493	1.486822	1.314481	0.08820418	0.360552
83945	Dnaja3	DnaJ heat shock protein family (Hsp40) member A3,	NA	-0.11538	5.51446	-1.08326	0.08821036	0.360552
21648	Dynlt1b	dynein light chain Tctex-type 1B	NA	0.122315	7.563854	1.08848	0.08836077	0.361048
12531	Cdc25b	cell division cycle 25B, transcript variant 2	NA	-0.16317	5.036969	-1.11974	0.08837599	0.361048
224405	Cyyr1	cysteine and tyrosine-rich protein 1, transcript variant	NA	0.209213	3.043957	1.156058	0.08843381	0.361126
18377	Omg	oligodendrocyte myelin glycoprotein	NA	0.248641	2.478456	1.188087	0.08843958	0.361126
102075	Plekhg4	pleckstrin homology domain containing, family G (witl	NA	-0.18188	3.27329	-1.13436	0.08855032	0.361488
73422	Prox2	prospero homeobox 2, transcript variant X1	NA	0.511012	0.080188	1.42505	0.08859618	0.361584
105246404	Gm41696	predicted gene, 41696, transcript variant X3	NA	0.661429	-0.35923	1.581648	0.08862233	0.3616
18293	Ogdh	oxoglutarate (alpha-ketoglutarate) dehydrogenase (lip	NA	-0.10471	7.23968	-1.07527	0.0887099	0.361867
102323	Dcun1d2	DCN1, defective in cullin neddylation 1, domain conta	NA	-0.11693	5.221901	-1.08443	0.08875111	0.361913
234593	Ndrg4	N-myc downstream regulated gene 4, transcript varia	NA	0.090199	8.115601	1.064517	0.08878082	0.361913
53978	Lpar2	lysophosphatidic acid receptor 2	NA	-0.14438	4.290308	-1.10526	0.08879616	0.361913
226043	Cbwd1	COBW domain containing 1, transcript variant 1	NA	-0.16755	3.729805	-1.12315	0.08881022	0.361913
104349	Zfp119a	zinc finger protein 119a	NA	-0.24893	2.767003	-1.18833	0.08888229	0.362101
103806	Maml1	mastermind like transcriptional coactivator 1	NA	-0.11741	4.920517	-1.08479	0.08890067	0.362101
22036	Traip	TRAF-interacting protein	NA	0.160995	4.004039	1.118058	0.08894597	0.362194
15463	Agfg1	ArfGAP with FG repeats 1, transcript variant 4	NA	0.099328	6.883526	1.071274	0.08900875	0.362325
67821	Atp1b4	ATPase, (Na+)/K+ transporting, beta 4 polypeptide, tı	NA	-0.75151	-0.16279	-1.68355	0.08902247	0.362325
240892	Dusp27	dual specificity phosphatase 27 (putative), transcript \	NA	-0.32491	1.512329	-1.25259	0.08921073	0.36296
102774	Bbs4	Bardet-Biedl syndrome 4 (human), transcript variant ⁻	NA	0.132349	5.208114	1.096077	0.08924103	0.36296
353047	Plekhm1	pleckstrin homology domain containing, family M (witl	NA	-0.16257	4.930223	-1.11928	0.08924548	0.36296
77595	Nup210l	nucleoporin 210-like	NA	-0.42519	0.758361	-1.34275	0.0892807	0.363012
102638029	Gm34696	predicted gene, 34696, transcript variant X2	NA	-0.47683	0.660188	-1.39168	0.08931999	0.363081
57294	Rps27	ribosomal protein S27, transcript variant 2	NA	0.098581	8.024088	1.07072	0.08938144	0.36324
56839	Lgi1	leucine-rich repeat LGI family, member 1, transcript v	NA	0.114221	5.056679	1.08239	0.08942679	0.363334
628779	Hs3st4	heparan sulfate (glucosamine) 3-O-sulfotransferase 4	NA	0.139546	5.975739	1.101558	0.08946404	0.363395
105246496	4930578L24Rik	RIKEN cDNA 4930578L24 gene	NA	0.535684	-0.04143	1.449629	0.08950006	0.363418
50527	Ero1a	endoplasmic reticulum oxidoreductase 1 alpha	NA	0.115078	5.48301	1.083034	0.08951441	0.363418
105246126	Gm41466	predicted gene, 41466	NA	-0.61594	0.167721	-1.53256	0.08962996	0.363796
212974	Pgghg	protein glucosylgalactosylhydroxylysine glucosidase,	NA	-0.23649	3.37587	-1.17812	0.08966769	0.363859

11489	Adam12	a disintegrin and metallopeptidase domain 12 (meltrir	NA	0.156101	4.063829	1.114272	0.08981189	0.364353
78908	Igsf3	immunoglobulin superfamily, member 3, transcript va	NA	0.082793	7.350227	1.059067	0.08984376	0.364391
58521	Eid1	EP300 interacting inhibitor of differentiation 1	NA	0.07605	9.17988	1.054128	0.08998106	0.364857
18481	Pak3	p21 (RAC1) activated kinase 3, transcript variant 4	NA	0.092644	7.992757	1.066322	0.09000932	0.364881
50935	St6galnac6	ST6 (alpha-N-acetyl-neuraminyl-2,3-beta-galactosyl-1	NA	-0.13304	4.739065	-1.0966	0.09010107	0.365159
245622	Fam199x	family with sequence similarity 199, X-linked	NA	0.192577	4.488394	1.142803	0.09012966	0.365159
231868	E130309D02Rik	RIKEN cDNA E130309D02 gene	NA	-0.14918	4.792244	-1.10894	0.09015463	0.365159
75296	Cep43	centrosomal protein 43, transcript variant 2	NA	0.146524	4.726411	1.1069	0.09016774	0.365159
673094	Cd99	CD99 antigen	NA	0.155369	5.021	1.113706	0.09020842	0.365233
215708	Miga1	mitoguardin 1, transcript variant X4	NA	0.110989	5.469799	1.079968	0.09030011	0.365513
74998	Rab11fip2	RAB11 family interacting protein 2 (class I), transcript	NA	0.109666	5.990594	1.078978	0.09039664	0.365813
20525	Slc2a1	solute carrier family 2 (facilitated glucose transporter)	NA	0.125479	6.391856	1.09087	0.09082889	0.367227
13819	Epas1	endothelial PAS domain protein 1	NA	0.118166	5.619186	1.085354	0.09083706	0.367227
66588	Cmpk1	cytidine monophosphate (UMP-CMP) kinase 1	NA	0.099577	7.40343	1.071459	0.09085082	0.367227
17196	Mbp	myelin basic protein, transcript variant 8	NA	0.169629	5.121122	1.124769	0.09085225	0.367227
83669	Wdr6	WD repeat domain 6	NA	-0.09326	8.690311	-1.06678	0.09085893	0.367227
68597	Ccdc167	coiled-coil domain containing 167, transcript variant 1	NA	-0.21501	3.832257	-1.16071	0.09089352	0.367276
14681	Gnao1	guanine nucleotide binding protein, alpha O, transcrip	NA	-0.0777	9.187537	-1.05533	0.09094786	0.367404
68514	Micu2	mitochondrial calcium uptake 2	NA	0.095798	6.004548	1.068656	0.09100316	0.367536
67168	Lpar6	lysophosphatidic acid receptor 6	NA	-0.22189	3.041261	-1.16626	0.09104511	0.367602
76117	Arhgap15	Rho GTPase activating protein 15, transcript variant X	NA	0.381463	0.839847	1.302663	0.09106454	0.367602
56013	Srcin1	SRC kinase signaling inhibitor 1, transcript variant X4	NA	-0.0817	7.706779	-1.05826	0.09113857	0.367727
140795	P2ry14	purinergic receptor P2Y, G-protein coupled, 14, trans	NA	0.389281	0.755958	1.309741	0.09114076	0.367727
69186	Tmem256	transmembrane protein 256	NA	-0.1805	4.126639	-1.13328	0.09119699	0.367863
54196	Pabpn1	poly(A) binding protein, nuclear 1	NA	0.139223	8.161271	1.101311	0.09123612	0.367887
207181	Rbms3	RNA binding motif, single stranded interacting protein	NA	0.108483	6.325536	1.078094	0.09131311	0.367887
118567618	LOC118567618	uncharacterized LOC118567618	NA	-0.26395	2.424192	-1.20076	0.09133439	0.367887
27096	Trappc3	trafficking protein particle complex 3	NA	0.095007	6.298675	1.068071	0.09133843	0.367887
19353	Rac1	Rac family small GTPase 1, transcript variant 2	NA	0.087683	8.540261	1.062662	0.09134837	0.367887
118567527	LOC118567527	uncharacterized LOC118567527, transcript variant X	NA	-0.48979	0.285981	-1.40424	0.09135743	0.367887
78832	Cacul1	CDK2 associated, cullin domain 1, transcript variant 1	NA	0.097566	6.221919	1.069967	0.09136134	0.367887
18613	Pecam1	platelet/endothelial cell adhesion molecule 1, transcri	NA	0.114082	5.104483	1.082286	0.0914337	0.367902
381062	Ermard	ER membrane associated RNA degradation, transcrip	NA	0.123297	4.795047	1.089221	0.091444	0.367902
224836	Usp49	ubiquitin specific peptidase 49	NA	-0.11442	6.367694	-1.08254	0.09144552	0.367902
243659	Styk1	serine/threonine/tyrosine kinase 1, transcript variant X	NA	0.326329	2.119257	1.253819	0.09145829	0.367902
381110	Rmdn2	regulator of microtubule dynamics 2, transcript varian	NA	0.283972	2.530017	1.217543	0.09148409	0.367902
103425	Ncln	nicalin	NA	-0.11163	5.755767	-1.08045	0.09151646	0.367902
28042	Selenoi	selenoprotein I, transcript variant 1	NA	0.099325	6.378052	1.071272	0.09153186	0.367902
14360	Fyn	Fyn proto-oncogene, transcript variant 1	NA	0.089279	7.975822	1.063839	0.09154596	0.367902
18552	Pcsk5	proprotein convertase subtilisin/kexin type 5, transcrip	NA	0.184328	3.958981	1.136288	0.09165152	0.368199
66373	Lsm5	LSM5 homolog, U6 small nuclear RNA and mRNA de	NA	0.142816	4.362888	1.104058	0.09166511	0.368199
216739	Acsl6	acyl-CoA synthetase long-chain family member 6, tra	NA	0.093773	6.373219	1.067158	0.09170415	0.368265
20229	Sat1	spermidine/spermine N1-acetyl transferase 1, transcr	NA	0.181293	4.410199	1.1339	0.09182573	0.368662
101497	Plekhg2	pleckstrin homology domain containing, family G (with	NA	-0.15846	5.94187	-1.11609	0.09187535	0.368728
20844	Stam	signal transducing adaptor molecule (SH3 domain an	NA	0.100011	6.370384	1.071781	0.09190073	0.368728
77781	Epm2aip1	EPM2A (laforin) interacting protein 1	NA	0.118615	7.503402	1.085692	0.09195849	0.368728
234373	Sugp2	SURP and G patch domain containing 2, transcript va	NA	-0.10795	6.833301	-1.0777	0.09199693	0.368728
18021	Nfatc3	nuclear factor of activated T cells, cytoplasmic, calcin	NA	0.124675	4.71372	1.090262	0.09201102	0.368728
11307	Abcg1	ATP binding cassette subfamily G member 1	NA	-0.13012	4.592838	-1.09439	0.09202644	0.368728
107771	Bmyc	brain expressed myelocytomatosis oncogene	NA	0.144123	4.590345	1.105059	0.09205565	0.368728
209601	Erich3	glutamate rich 3	NA	0.37789	1.163064	1.29944	0.0920726	0.368728
239102	Zfhx2	zinc finger homeobox 2, transcript variant X3	NA	-0.11482	6.644864	-1.08284	0.09207628	0.368728
212517	Cfap44	cilia and flagella associated protein 44, transcript vari	NA	0.307961	2.554651	1.237957	0.0920896	0.368728
17228	Cma1	chymase 1, mast cell	NA	-1.0442	-0.05656	-2.06223	0.09209941	0.368728
18484	Pam	peptidylglycine alpha-amidating monooxygenase, tra	NA	0.09256	6.609928	1.066261	0.09211399	0.368728
14760	Gpr19	G protein-coupled receptor 19, transcript variant 3	NA	-0.17687	4.233355	-1.13043	0.09222467	0.369021
22142	Tuba1a	tubulin, alpha 1A	NA	0.098948	13.19093	1.070993	0.09223257	0.369021
71712	Dram1	DNA-damage regulated autophagy modulator 1, trans	NA	0.493426	0.474563	1.407784	0.09228933	0.369077
102641704	Gm11690	predicted gene 11690	NA	-0.4384	0.543591	-1.3551	0.09229202	0.369077
75425	Tti1	TELO2 interacting protein 1	NA	-0.20313	4.632659	-1.15119	0.09233961	0.369177
50724	Sap30l	SAP30-like	NA	0.262125	3.988414	1.199244	0.0923878	0.36918
665119	Sec14l5	SEC14-like lipid binding 5, transcript variant X3	NA	-0.39789	0.826069	-1.31758	0.09239605	0.36918
245857	Ssh3	slingshot protein phosphatase 3, transcript variant 2	NA	0.17296	4.590772	1.12737	0.09240848	0.36918
68194	Ndufb4	NADH:ubiquinone oxidoreductase subunit B4	NA	0.16937	3.805549	1.124567	0.09246576	0.369318
215445	Rab11fip3	RAB11 family interacting protein 3 (class II), transcrip	NA	-0.12133	6.714028	-1.08774	0.09252239	0.369454
12864	Cox6c	cytochrome c oxidase subunit 6C	NA	0.085778	6.756553	1.06126	0.09268574	0.369889
67824	Nmral1	NmrA-like family domain containing 1, transcript varia	NA	-0.12474	4.863358	-1.09031	0.09271394	0.369889
19261	Sirpa	signal-regulatory protein alpha, transcript variant 9	NA	0.093813	6.277863	1.067187	0.09271917	0.369889

20338	Sel1l	sel-1 suppressor of lin-12-like (C. elegans), transcript	NA	0.09445	6.900391	1.067658	0.09274198	0.369889
59126	Nek6	NIMA (never in mitosis gene a)-related expressed kin	NA	0.112224	5.301968	1.080894	0.0927659	0.369889
104027	Synpo	synaptopodin, transcript variant X11	NA	-0.17931	3.396263	-1.13234	0.09276788	0.369889
170768	Pfkfb3	6-phosphofructo-2-kinase/fructose-2,6-biphosphatase	NA	0.112639	6.236704	1.081204	0.09286233	0.370175
66826	Taz	tafazzin, transcript variant 2	NA	0.12039	5.093341	1.087029	0.09298049	0.370556
110834	Chrna3	cholinergic receptor, nicotinic, alpha polypeptide 3	NA	-0.14895	3.944038	-1.10876	0.09313898	0.371091
66293	Snhg16	small nucleolar RNA host gene 16, transcript variant :	NA	0.283416	3.324117	1.217073	0.09317812	0.371091
56697	Akap10	A kinase (PRKA) anchor protein 10, transcript variant	NA	-0.12083	5.41566	-1.08736	0.09320379	0.371091
229780	Trmt13	tRNA methyltransferase 13, transcript variant X3	NA	0.144018	4.244913	1.104978	0.09322842	0.371091
14667	Gm2a	GM2 ganglioside activator protein	NA	0.127075	5.231447	1.092077	0.09327265	0.371091
213019	Pdlim2	PDZ and LIM domain 2, transcript variant X4	NA	0.362766	1.648134	1.285889	0.09328553	0.371091
11803	Aplp1	amyloid beta (A4) precursor-like protein 1	NA	0.088037	9.111785	1.062923	0.09328904	0.371091
11845	Arf6	ADP-ribosylation factor 6	NA	-0.12332	6.546247	-1.08924	0.09329735	0.371091
228836	Dlgap4	DLG associated protein 4, transcript variant X23	NA	-0.08011	8.402669	-1.0571	0.09334857	0.371204
11566	Adss	adenylosuccinate synthetase, non muscle	NA	0.085824	6.781263	1.061294	0.09339599	0.371236
100502630	Gm15545	predicted gene 15545	NA	-0.53219	0.307948	-1.44612	0.09340753	0.371236
103266	Tmem263	transmembrane protein 263	NA	0.119163	5.72118	1.086105	0.09346212	0.371236
11810	Apobec1	apolipoprotein B mRNA editing enzyme, catalytic poly	NA	0.360945	1.36351	1.284267	0.09348016	0.371236
69549	2310009B15Rik	RIKEN cDNA 2310009B15 gene	NA	0.324289	2.233349	1.252047	0.09348931	0.371236
12986	Csf3r	colony stimulating factor 3 receptor (granulocyte), tra	NA	-0.44942	0.409611	-1.36549	0.0934935	0.371236
17448	Mdh2	malate dehydrogenase 2, NAD (mitochondrial)	NA	0.080585	7.775565	1.057447	0.09368655	0.371866
218811	Sec24c	Sec24 related gene family, member C (S. cerevisiae)	NA	-0.09143	7.080406	-1.06542	0.09371373	0.371866
100043072	A230070E04Rik	RIKEN cDNA A230070E04 gene	NA	-0.25424	2.610317	-1.19271	0.09372071	0.371866
28081	Fam104a	family with sequence similarity 104, member A, trans	NA	0.116018	5.246485	1.08374	0.09379565	0.372073
20044	Rps14	ribosomal protein S14	NA	0.127906	8.50246	1.092706	0.09382099	0.372083
233724	Tmem41b	transmembrane protein 41B	NA	0.098248	5.671906	1.070472	0.09389415	0.372254
11352	Abl2	v-abl Abelson murine leukemia viral oncogene 2 (arg	NA	0.120035	6.038532	1.086761	0.09391	0.372254
320634	Ocrl	OCRL, inositol polyphosphate-5-phosphatase	NA	0.11694	6.155021	1.084432	0.09399518	0.372501
12453	Ccni	cyclin I	NA	-0.09225	8.740603	-1.06603	0.09402549	0.372531
235567	Dnajc13	DnaJ heat shock protein family (Hsp40) member C13	NA	0.118202	5.939639	1.085381	0.094061	0.37258
26377	Dapp1	dual adaptor for phosphotyrosine and 3-phosphoinos	NA	0.251218	2.152178	1.190211	0.09409385	0.372591
214663	Slc25a29	solute carrier family 25 (mitochondrial carrier, palmito	NA	-0.17653	3.290982	-1.13016	0.09412402	0.372591
100041574	9030025P20Rik	RIKEN cDNA 9030025P20 gene	NA	0.29865	2.920687	1.229993	0.09413825	0.372591
100072	Camta1	calmodulin binding transcription activator 1, transcript	NA	0.098339	8.155329	1.07054	0.09415529	0.372591
269152	Kif26b	kinesin family member 26B, transcript variant X1	NA	0.127315	6.592829	1.092259	0.09425513	0.372622
238725	Gpr150	G protein-coupled receptor 150	NA	-0.54021	-0.11691	-1.45418	0.09426496	0.372622
68870	Ak8	adenylate kinase 8	NA	0.449356	0.582214	1.36543	0.09426515	0.372622
14823	Grm8	glutamate receptor, metabotropic 8, transcript variant	NA	0.181744	3.321004	1.134254	0.09426551	0.372622
14420	Galc	galactosylceramidase	NA	0.131527	4.857482	1.095453	0.09427751	0.372622
75135	4930526I15Rik	RIKEN cDNA 4930526I15 gene, transcript variant 3	NA	0.406276	1.741497	1.325261	0.09431739	0.372643
57296	Psmd8	proteasome (prosome, macropain) 26S subunit, non-	NA	0.097234	7.054289	1.06972	0.0943673	0.372643
16574	Kif5c	kinesin family member 5C	NA	0.081117	9.396916	1.057837	0.09438094	0.372643
94190	Ophn1	oligophrenin 1, transcript variant 2	NA	0.105037	6.081606	1.075522	0.09439364	0.372643
12021	Bard1	BRCA1 associated RING domain 1	NA	-0.19937	3.12822	-1.1482	0.09439736	0.372643
30949	Lcmt1	leucine carboxyl methyltransferase 1, transcript varia	NA	0.100928	5.659966	1.072463	0.09447446	0.372768
69519	Rwdd2a	RWD domain containing 2A, transcript variant 1	NA	0.257507	2.684857	1.195411	0.09447491	0.372768
19727	Rfxank	regulatory factor X-associated ankyrin-containing pro	NA	-0.15659	4.243994	-1.11465	0.09450598	0.372773
382111	Susd5	sushi domain containing 5	NA	0.553149	0.424711	1.467285	0.09452184	0.372773
93681	Zkscan8	zinc finger with KRAB and SCAN domains 8, transcri	NA	0.143004	4.72376	1.104202	0.09456389	0.372848
22339	Vegfa	vascular endothelial growth factor A, transcript varian	NA	0.149867	6.696706	1.109467	0.09461273	0.372901
11677	Akr1b3	aldo-keto reductase family 1, member B3 (aldose red	NA	-0.11718	5.47572	-1.08461	0.09462328	0.372901
228025	Gm17250	predicted gene, 17250, transcript variant X4	NA	-0.45213	0.50281	-1.36806	0.09467271	0.373002
106338	Nsun3	NOL1/NOP2/Sun domain family member 3, transcript	NA	0.193652	3.69422	1.143655	0.09469452	0.373002
22404	Wiz	widely-interspaced zinc finger motifs, transcript variar	NA	-0.07946	7.307246	-1.05662	0.09481219	0.373375
67267	Uqcc2	ubiquinol-cytochrome c reductase complex assembly	NA	0.129647	5.064508	1.094026	0.09487459	0.373465
70223	Nars	asparaginyl-tRNA synthetase, transcript variant 1	NA	0.080138	7.152186	1.057119	0.09488105	0.373465
71340	Riok1	RIO kinase 1, transcript variant X3	NA	0.135965	4.931064	1.098828	0.09491948	0.373526
22751	Zfp90	zinc finger protein 90, transcript variant X1	NA	0.103205	6.013443	1.074157	0.09502392	0.373847
27643	Ubl4a	ubiquitin-like 4A, transcript variant 1	NA	0.109481	5.543531	1.07884	0.09508397	0.373992
234515	Inpp4b	inositol polyphosphate-4-phosphatase, type II, transc	NA	0.19569	3.194804	1.145272	0.09511871	0.374039
18554	Pcsk7	proprotein convertase subtilisin/kexin type 7, transcri	NA	0.134887	5.032969	1.098007	0.09516623	0.374135
216157	Tmem259	transmembrane protein 259, transcript variant 2	NA	0.11935	6.414333	1.086245	0.09523479	0.374314
333433	Gpd1l	glycerol-3-phosphate dehydrogenase 1-like	NA	0.096299	6.208682	1.069028	0.09528105	0.374385
68815	Btbd10	BTB (POZ) domain containing 10, transcript variant X	NA	0.098341	6.064589	1.070542	0.09530086	0.374385
105387	Akr1c14	aldo-keto reductase family 1, member C14	NA	-0.5721	0.178831	-1.48669	0.09532177	0.374385
94089	Trim7	tripartite motif-containing 7, transcript variant X6	NA	0.363381	1.516726	1.286438	0.09538468	0.374541
327744	E130307A14Rik	RIKEN cDNA E130307A14 gene	NA	0.12055	5.107292	1.087149	0.09541735	0.374579
319190	H2bc21	H2B clustered histone 21	NA	-0.15725	4.141977	-1.11516	0.09545543	0.374638

14339	Aktip	thymoma viral proto-oncogene 1 interacting protein, t	NA	0.097791	6.478355	1.070133	0.09549755	0.374667
18131	Notch3	notch 3	NA	-0.11327	5.448181	-1.08168	0.09550881	0.374667
68910	Zfp467	zinc finger protein 467, transcript variant X6	NA	-0.14018	5.00977	-1.10204	0.09558134	0.374861
18739	Pitpnm1	phosphatidylinositol transfer protein, membrane-asso	NA	0.107752	6.360113	1.077548	0.09562797	0.374954
73242	Atat1	alpha tubulin acetyltransferase 1, transcript variant 3	NA	-0.09379	8.610292	-1.06717	0.09569361	0.375017
381306	BC055324	cDNA sequence BC055324, transcript variant 1	NA	-0.18875	3.151919	-1.13978	0.09570184	0.375017
102639318	Gm35658	predicted gene, 35658, transcript variant X1	NA	0.569437	0.027703	1.483944	0.09575349	0.375017
107751	Prrxl1	paired related homeobox protein-like 1, transcript var	NA	0.49042	2.272636	1.404854	0.09579914	0.375017
12180	Smyd1	SET and MYND domain containing 1, transcript varia	NA	-0.35448	1.715081	-1.27852	0.09580034	0.375017
170719	Oxr1	oxidation resistance 1, transcript variant 3	NA	0.090366	6.147037	1.06464	0.0958075	0.375017
78781	Zc3hav1	zinc finger CCCH type, antiviral 1, transcript variant 3	NA	0.34323	1.443136	1.268593	0.09580915	0.375017
193838	Eme2	essential meiotic structure-specific endonuclease sub	NA	-0.32299	2.221651	-1.25092	0.09585237	0.375017
68556	Uckl1	uridine-cytidine kinase 1-like 1, transcript variant 2	NA	-0.16794	4.281688	-1.12345	0.09588453	0.375017
13435	Dnmt3a	DNA methyltransferase 3A, transcript variant 2	NA	-0.0857	7.593263	-1.0612	0.09589252	0.375017
330177	Taok3	TAO kinase 3, transcript variant X3	NA	-0.10253	6.055796	-1.07366	0.09591034	0.375017
75040	Efcab10	EF-hand calcium binding domain 10	NA	0.591938	-0.3002	1.50727	0.09593146	0.375017
213556	Plekhh2	pleckstrin homology domain containing, family H (with	NA	0.178585	3.315331	1.131773	0.09594357	0.375017
102632465	Gm13391	predicted gene 13391, transcript variant X2	NA	-1.00961	-0.75139	-2.01336	0.09607362	0.375435
71069	Stox2	storkhead box 2, transcript variant X7	NA	0.095423	7.979125	1.068379	0.09610531	0.375436
434198	B130024G19Rik	RIKEN cDNA B130024G19 gene	NA	0.318448	1.445126	1.246989	0.09612015	0.375436
433771	Micos10	mitochondrial contact site and cristae organizing syst	NA	0.096021	6.050587	1.068822	0.09617367	0.375555
14612	Gja4	gap junction protein, alpha 4	NA	0.195366	3.244249	1.145015	0.09624866	0.375747
72180	Zfp661	zinc finger protein 661, transcript variant X7	NA	0.204674	3.72961	1.152426	0.0962968	0.375747
224090	Tmem44	transmembrane protein 44	NA	0.099243	7.148643	1.071212	0.09630668	0.375747
212647	Aldh4a1	aldehyde dehydrogenase 4 family, member A1	NA	-0.11833	5.04129	-1.08548	0.09634871	0.375747
64058	Perp	PERP, TP53 apoptosis effector	NA	-0.24386	3.563822	-1.18415	0.09636099	0.375747
237886	Slfn9	schlafen 9, transcript variant X1	NA	-0.20755	3.418941	-1.15473	0.09636129	0.375747
20452	St8sia4	ST8 alpha-N-acetyl-neuraminide alpha-2,8-sialyltrans	NA	0.104164	6.203698	1.074872	0.09640341	0.375782
51897	Atg13	autophagy related 13, transcript variant 4	NA	-0.10518	5.778133	-1.07563	0.09641662	0.375782
108069	Grm3	glutamate receptor, metabotropic 3	NA	0.14916	4.189635	1.108923	0.09652293	0.376107
67890	Ufm1	ubiquitin-fold modifier 1	NA	0.090315	6.355685	1.064603	0.09661245	0.376355
14687	Gnaz	guanine nucleotide binding protein, alpha z subunit, t	NA	0.103271	6.220302	1.074206	0.09663282	0.376355
545276	Gal3st3	galactose-3-O-sulfotransferase 3	NA	-0.1263	5.072932	-1.09149	0.09669831	0.376519
100042498	Mir22hg	Mir22 host gene (non-protein coding)	NA	0.266086	2.526189	1.202541	0.09674118	0.376596
233895	Prr14	proline rich 14, transcript variant X5	NA	-0.09448	6.421785	-1.06768	0.09678606	0.376681
239364	Tspyl5	testis-specific protein, Y-encoded-like 5	NA	0.116071	5.130131	1.083779	0.09682414	0.376739
50849	Rnf10	ring finger protein 10, transcript variant 2	NA	-0.0908	8.410151	-1.06496	0.09689137	0.37691
67769	Gpatch2	G patch domain containing 2, transcript variant X1	NA	-0.13643	4.810755	-1.09918	0.09701715	0.377183
11899	Astn1	astrotactin 1, transcript variant 2	NA	0.078513	7.825865	1.055929	0.09702249	0.377183
29817	Igfbp7	insulin-like growth factor binding protein 7, transcript	NA	0.156581	4.442135	1.114642	0.09703093	0.377183
20656	Sod2	superoxide dismutase 2, mitochondrial	NA	-0.09158	6.396776	-1.06553	0.09706499	0.377219
327900	Ubtd2	ubiquitin domain containing 2, transcript variant 1	NA	0.099934	6.665089	1.071724	0.09708664	0.377219
98496	Pid1	phosphotyrosine interaction domain containing 1, tran	NA	0.108647	5.758749	1.078217	0.09711916	0.377255
240084	Cchcr1	coiled-coil alpha-helical rod protein 1, transcript varia	NA	-0.21799	3.332094	-1.16311	0.09714638	0.377271
114604	Prdm15	PR domain containing 15, transcript variant X17	NA	0.129398	4.680945	1.093837	0.09720698	0.377346
12952	Cry1	cryptochrome 1 (photolyase-like)	NA	0.113212	5.073083	1.081634	0.09721209	0.377346
381038	Parl	presenilin associated, rhomboid-like	NA	0.144517	4.906897	1.10536	0.09727477	0.377463
67015	Ccdc91	coiled-coil domain containing 91, transcript variant 1	NA	-0.14896	5.048059	-1.10877	0.09728867	0.377463
69875	Ndufa11	NADH:ubiquinone oxidoreductase subunit A11	NA	0.109562	5.20594	1.0789	0.09732655	0.37752
233876	Hirip3	HIRA interacting protein 3	NA	-0.16469	5.30495	-1.12093	0.09736885	0.377588
66106	Smpx	small muscle protein, X-linked, transcript variant 1	NA	-0.62047	-0.35052	-1.53737	0.09740533	0.377588
242736	Pramel12	PRAME like 12, transcript variant 2	NA	-0.12197	4.664912	-1.08822	0.09741352	0.377588
73658	Spns1	spinster homolog 1, transcript variant 1	NA	-0.11127	5.584615	-1.08018	0.09758494	0.378162
12539	Cdc37	cell division cycle 37, transcript variant 2	NA	-0.09572	6.913765	-1.0686	0.09761308	0.378181
67622	Mxra7	matrix-remodelling associated 7, transcript variant 1	NA	0.122652	4.87754	1.088734	0.09767419	0.378328
20085	Rps19	ribosomal protein S19, transcript variant 1	NA	0.094131	7.796414	1.067422	0.09770706	0.378365
102680	Slc6a20a	solute carrier family 6 (neurotransmitter transporter),	NA	0.283328	2.108569	1.216999	0.0977341	0.37838
100040846	5930403N24Rik	RIKEN cDNA 5930403N24 gene, transcript variant 2	NA	-0.39058	0.979901	-1.31092	0.09779821	0.378538
80749	Lrfn1	leucine rich repeat and fibronectin type III domain cor	NA	-0.10382	6.115099	-1.07461	0.09798962	0.379188
78365	Lhx1os	LIM homeobox 1, opposite strand	NA	0.31328	2.924682	1.242529	0.09802806	0.379247
67938	Myl12b	myosin, light chain 12B, regulatory	NA	0.078993	7.478088	1.05628	0.09813762	0.379576
68468	Ly6g6c	lymphocyte antigen 6 complex, locus G6C	NA	-1.1193	-0.96942	-2.17241	0.09815979	0.379576
234374	Ddx49	DEAD box helicase 49, transcript variant 1	NA	0.110864	5.101665	1.079875	0.09820763	0.37959
14467	Nipsnap2	nipsnap homolog 2	NA	0.097662	6.133615	1.070038	0.09821363	0.37959
80892	Zfhx4	zinc finger homeodomain 4, transcript variant X2	NA	0.153344	6.418136	1.112144	0.09823342	0.37959
14367	Fzd5	frizzled class receptor 5, transcript variant 2	NA	-0.25117	2.440334	-1.19018	0.09827797	0.379646
11308	Abi1	abl-interactor 1, transcript variant 6	NA	0.08313	6.632471	1.059314	0.09829463	0.379646
227656	Rexo4	REX4, 3'-5' exonuclease, transcript variant 1	NA	-0.14497	5.078815	-1.10571	0.09833666	0.379719

11409	Acads	acyl-Coenzyme A dehydrogenase, short chain	NA	-0.17934	3.476187	-1.13237	0.09862715	0.380595
13371	Dio2	deiodinase, iodothyronine, type II	NA	0.284624	1.971749	1.218093	0.09863424	0.380595
75770	Brsk2	BR serine/threonine kinase 2, transcript variant 3	NA	-0.07844	7.323264	-1.05588	0.09864227	0.380595
20646	Snrpn	small nuclear ribonucleoprotein N, transcript variant 1	NA	0.078094	8.808246	1.055623	0.09865711	0.380595
224523	4732491K20Rik	RIKEN cDNA 4732491K20 gene	NA	0.291995	1.676651	1.224332	0.09869627	0.380656
114714	Rad51c	RAD51 paralog C, transcript variant X1	NA	-0.20485	2.949145	-1.15256	0.0987626	0.380758
71602	Myo1e	myosin IE, transcript variant X1	NA	-0.17752	3.623098	-1.13094	0.09876962	0.380758
234214	Sorbs2	sorbin and SH3 domain containing 2, transcript variar	NA	-0.07942	7.072494	-1.05659	0.09893708	0.381235
244654	Mtss2	MTSS I-BAR domain containing 2, transcript variant ´	NA	-0.09014	8.244083	-1.06447	0.09894025	0.381235
66226	Trappc2	trafficking protein particle complex 2, transcript variar	NA	0.138563	4.947338	1.100808	0.09896787	0.381251
22063	Trpc1	transient receptor potential cation channel, subfamily	NA	0.158713	4.066701	1.116291	0.09899434	0.381263
70315	Hdac8	histone deacetylase 8, transcript variant 1	NA	-0.18715	4.045033	-1.13851	0.09903469	0.381311
210104	Zfp658	zinc finger protein 658	NA	-0.13552	4.36431	-1.09849	0.09909132	0.381311
78004	Prr15	proline rich 15, transcript variant X2	NA	0.328861	1.495814	1.256022	0.09909182	0.381311
664805	Skor2	SKI family transcriptional corepressor 2, transcript va	NA	0.235238	2.539999	1.177101	0.09910055	0.381311
11735	Ank3	ankyrin 3, epithelial, transcript variant 9	NA	0.077459	9.058665	1.055158	0.09915076	0.381414
74243	Slx4ip	SLX4 interacting protein, transcript variant 2	NA	-0.11999	4.741171	-1.08673	0.09924078	0.38167
56811	Dkk2	dickkopf WNT signaling pathway inhibitor 2	NA	-0.3266	1.815929	-1.25405	0.09930562	0.381805
68911	Pygo2	pygopus 2, transcript variant 3	NA	-0.10077	6.173594	-1.07234	0.09933577	0.381805
20918	Eif1	eukaryotic translation initiation factor 1	NA	0.073413	8.505218	1.052203	0.09942798	0.381805
26878	B3galt2	UDP-Gal:betaGlcNAc beta 1,3-galactosyltransferase,	NA	0.116623	4.621853	1.084194	0.09942884	0.381805
71323	Rassf8	Ras association (RalGDS/AF-6) domain family (N-ter	NA	0.188964	4.088198	1.139944	0.09944154	0.381805
94192	C1galt1	core 1 synthase, glycoprotein-N-acetylgalactosamine	NA	0.35576	1.551145	1.279659	0.0994522	0.381805
11863	Arnt	aryl hydrocarbon receptor nuclear translocator, transc	NA	-0.12039	5.616563	-1.08703	0.09947508	0.381805
66412	Arrdc4	arrestin domain containing 4, transcript variant 1	NA	0.160582	5.278928	1.117738	0.09948092	0.381805
66717	Ccdc96	coiled-coil domain containing 96	NA	-0.24742	2.22359	-1.18708	0.09949011	0.381805
381085	Tbc1d22b	TBC1 domain family, member 22B, transcript variant	NA	-0.0968	6.109838	-1.0694	0.09951057	0.381805
192662	Arhgdia	Rho GDP dissociation inhibitor (GDI) alpha, transcrip	NA	-0.08174	8.302535	-1.05829	0.09959807	0.382051
224585	Zfp160	zinc finger protein 160	NA	0.117285	5.286351	1.084692	0.09969367	0.382094
118568691	LOC118568691	uncharacterized LOC118568691	NA	0.606996	-0.17585	1.523085	0.09970228	0.382094
12335	Capn3	calpain 3, transcript variant a	NA	-0.56444	-0.01524	-1.47882	0.0997025	0.382094
21813	Tgfbr2	transforming growth factor, beta receptor II, transcript	NA	0.131377	4.574698	1.095338	0.09977533	0.382094
118446	Gjc3	gap junction protein, gamma 3, transcript variant 1	NA	0.515395	0.696871	1.429385	0.09979174	0.382094
18020	Nfatc2ip	nuclear factor of activated T cells, cytoplasmic, calcin	NA	-0.1597	3.800347	-1.11705	0.09979178	0.382094
57295	Icmt	isoprenylcysteine carboxyl methyltransferase, transcr	NA	-0.11578	5.399887	-1.08356	0.09979244	0.382094
75422	Mettl5	methyltransferase like 5, transcript variant 1	NA	-0.2319	2.896211	-1.17438	0.09979711	0.382094
12307	Calb1	calbindin 1	NA	0.118518	4.780447	1.085619	0.09982559	0.382113
19367	Rad9a	RAD9 checkpoint clamp component A	NA	-0.14879	3.938356	-1.10864	0.09987734	0.382186
102632484	Gm26691	predicted gene, 26691, transcript variant X2	NA	0.536932	0.006724	1.450884	0.09989176	0.382186
20230	Satb1	special AT-rich sequence binding protein 1, transcript	NA	0.082701	6.460146	1.058999	0.09995452	0.382337
109815	Selenos	selenoprotein S, transcript variant 1	NA	0.123986	4.680997	1.089741	0.10005712	0.382532
353172	Gars	glycyl-tRNA synthetase	NA	0.079648	7.536031	1.05676	0.10007642	0.382532
241263	Gpr158	G protein-coupled receptor 158	NA	0.191024	3.442101	1.141574	0.10008916	0.382532
66730	4921531C22Rik	RIKEN cDNA 4921531C22 gene	NA	-0.31691	1.619835	-1.24566	0.10011763	0.382532
70458	2610318N02Rik	RIKEN cDNA 2610318N02 gene, transcript variant 2	NA	-0.25803	2.449873	-1.19584	0.10012307	0.382532
14561	Gdf11	growth differentiation factor 11	NA	-0.10313	5.343304	-1.0741	0.10043165	0.383621
233064	Wdr62	WD repeat domain 62, transcript variant 2	NA	-0.20669	3.119759	-1.15404	0.10047937	0.383671
338363	Tmem241	transmembrane protein 241, transcript variant 1	NA	-0.19907	3.162634	-1.14796	0.10052841	0.383671
73545	1700094D03Rik	RIKEN cDNA 1700094D03 gene	NA	-0.16595	3.814395	-1.12191	0.10053073	0.383671
22325	Vav2	vav 2 oncogene, transcript variant X3	NA	-0.09118	6.147287	-1.06524	0.10053924	0.383671
320311	Rnf152	ring finger protein 152, transcript variant X1	NA	0.112294	6.751351	1.080946	0.10057989	0.383736
15118	Has3	hyaluronan synthase 3, transcript variant 2	NA	-0.14711	4.207835	-1.10735	0.10065285	0.383925
105847	Lmf2	lipase maturation factor 2, transcript variant X1	NA	-0.14757	4.994887	-1.10771	0.10081872	0.384369
77889	Lbh	limb-bud and heart	NA	-0.09379	7.241677	-1.06717	0.10082797	0.384369
20740	Sptan1	spectrin alpha, non-erythrocytic 1, transcript variant X	NA	0.084934	9.526593	1.060639	0.1008403	0.384369
18117	Emc8	ER membrane protein complex subunit 8	NA	0.102588	5.835353	1.073698	0.10087346	0.384406
71780	Isyna1	myo-inositol 1-phosphate synthase A1	NA	-0.12552	5.819085	-1.0909	0.10093081	0.384534
19099	Mapk8ip1	mitogen-activated protein kinase 8 interacting protein	NA	-0.08072	7.934346	-1.05755	0.10100264	0.384718
227446	Relch	RAB11 binding and LisH domain, coiled-coil and HEA	NA	0.119128	6.036515	1.086078	0.10105518	0.384808
242557	Atg4c	autophagy related 4C, cysteine peptidase, transcript '	NA	0.13141	4.484633	1.095364	0.10109995	0.384808
22791	Dnajc2	DnaJ heat shock protein family (Hsp40) member C2	NA	0.111172	5.895342	1.080105	0.10113672	0.384808
70351	Ppp4r1	protein phosphatase 4, regulatory subunit 1, transcrip	NA	0.099041	6.405327	1.071061	0.10115755	0.384808
23918	Impdh2	inosine monophosphate dehydrogenase 2, transcript	NA	-0.09377	6.729632	-1.06715	0.10115798	0.384808
329274	Fam163a	family with sequence similarity 163, member A	NA	-0.21315	2.673899	-1.15922	0.10116823	0.384808
16835	Ldlr	low density lipoprotein receptor, transcript variant 1	NA	0.124728	5.057767	1.090302	0.10122564	0.384937
71703	Armcx3	armadillo repeat containing, X-linked 3, transcript vari	NA	0.082582	6.644565	1.058912	0.10128695	0.38508
100317	AU040320	expressed sequence AU040320, transcript variant 3	NA	-0.10697	5.820664	-1.07697	0.10137227	0.385149
106504	Stk38	serine/threonine kinase 38, transcript variant 1	NA	0.08181	6.578955	1.058345	0.10139117	0.385149

67381	Med4	mediator complex subunit 4	NA	0.127471	5.415401	1.092377	0.1014019	0.385149
328329	Mast4	microtubule associated serine/threonine kinase famil	NA	0.116069	6.286246	1.083778	0.1014081	0.385149
72306	Zfp777	zinc finger protein 777, transcript variant X1	NA	0.121347	4.977912	1.08775	0.10142353	0.385149
99151	Cercam	cerebral endothelial cell adhesion molecule	NA	0.167194	4.061372	1.122873	0.1014485	0.385154
98402	Sh3bp4	SH3-domain binding protein 4	NA	-0.13897	5.123103	-1.10112	0.10150644	0.385192
12412	Cbx1	chromobox 1, transcript variant 2	NA	-0.10129	7.218496	-1.07273	0.10150797	0.385192
319197	Gpr4	G protein-coupled receptor 4	NA	0.297262	2.0497	1.228811	0.10152963	0.385192
100036521	Umad1	UMAP1-MVP12 associated (UMA) domain containing	NA	0.115366	4.832376	1.08325	0.10157738	0.385284
18514	Pbx1	pre B cell leukemia homeobox 1, transcript variant a	NA	0.074102	8.793308	1.052706	0.101775	0.385943
14805	Grik1	glutamate receptor, ionotropic, kainate 1, transcript v	NA	0.120545	5.189877	1.087146	0.10181562	0.386007
67698	Fam174a	family with sequence similarity 174, member A	NA	0.176908	3.566708	1.130459	0.10184465	0.386028
246691	Prok1	prokineticin 1, transcript variant 1	NA	0.627124	-0.02577	1.544483	0.10201836	0.386582
78672	9530057J20Rik	RIKEN cDNA 9530057J20 gene	NA	-0.49252	0.834019	-1.4069	0.10203848	0.386582
66978	Luc7l	Luc7-like, transcript variant 2	NA	0.084498	7.461269	1.060319	0.10208905	0.386653
320606	3632454L22Rik	RIKEN cDNA 3632454L22 gene, transcript variant 1	NA	0.499233	0.573065	1.413462	0.10210477	0.386653
56739	Rec8	REC8 meiotic recombination protein, transcript variar	NA	-0.23879	3.084664	-1.18	0.10218162	0.386854
12848	Cops2	COP9 signalosome subunit 2, transcript variant 2	NA	0.082322	6.883609	1.05872	0.10222544	0.38693
68592	Syf2	SYF2 homolog, RNA splicing factor (S. cerevisiae)	NA	0.115188	5.150382	1.083116	0.10226213	0.386964
546611	Klhl33	kelch-like 33, transcript variant 2	NA	0.311207	1.668777	1.240745	0.10238552	0.386964
67468	Mmd	monocyte to macrophage differentiation-associated	NA	0.08213	7.549973	1.05858	0.10239601	0.386964
218194	Phactr1	phosphatase and actin regulator 1, transcript variant :	NA	0.088116	7.381693	1.062981	0.10241364	0.386964
330355	Dnah6	dynein, axonemal, heavy chain 6	NA	-0.28735	1.853041	-1.2204	0.10242359	0.386964
102639549	Gm38521	predicted gene, 38521	NA	-0.51465	0.35092	-1.42865	0.10244358	0.386964
170459	Stard4	StAR-related lipid transfer (START) domain containin	NA	0.14263	4.987588	1.103915	0.1024623	0.386964
18813	Pa2g4	proliferation-associated 2G4	NA	-0.10064	7.530889	-1.07225	0.1024652	0.386964
17126	Smad2	SMAD family member 2, transcript variant X1	NA	0.090064	6.720543	1.064417	0.10249581	0.386964
67248	Rpl39	ribosomal protein L39	NA	0.087189	7.293591	1.062298	0.10252719	0.386964
76025	Cant1	calcium activated nucleotidase 1, transcript variant 4	NA	-0.09592	5.77609	-1.06874	0.10252842	0.386964
15975	Ifnar1	interferon (alpha and beta) receptor 1	NA	0.082927	6.683659	1.059165	0.10253064	0.386964
23802	Amfr	autocrine motility factor receptor, transcript variant X1	NA	0.086678	7.247337	1.061922	0.10254366	0.386964
12424	Cck	cholecystokinin, transcript variant 1	NA	0.265937	2.814867	1.202416	0.10258676	0.386989
18230	Nxn	nucleoredoxin	NA	-0.1037	5.250849	-1.07452	0.10259786	0.386989
108167513	Gm46027	predicted gene, 46027	NA	-0.27977	2.675762	-1.214	0.10264197	0.387066
12715	Ckm	creatine kinase, muscle	NA	-0.78297	1.317516	-1.72067	0.10266956	0.38708
213993	Ccdc186	coiled-coil domain containing 186, transcript variant X	NA	-0.10657	5.628867	-1.07667	0.10271485	0.387157
100504399	C130021I20Rik	Riken cDNA C130021I20 gene	NA	-0.20074	3.789264	-1.14929	0.10273746	0.387157
19153	Prx	periaxin, transcript variant 1	NA	-0.246	2.447012	-1.18591	0.10280031	0.387304
226841	Vash2	vasohibin 2, transcript variant 2	NA	0.076309	7.087689	1.054317	0.1028331	0.387338
67054	Paics	phosphoribosylaminoimidazole carboxylase, phosphc	NA	-0.08551	7.251353	-1.06106	0.10293455	0.38763
68002	Sdhaf4	succinate dehydrogenase complex assembly factor 4	NA	0.173195	3.920194	1.127553	0.10301035	0.387801
54367	Zfp326	zinc finger protein 326, transcript variant 1	NA	0.090698	5.912865	1.064885	0.10302763	0.387801
14156	Fen1	flap structure specific endonuclease 1, transcript vari	NA	-0.13252	4.450242	-1.09621	0.10306322	0.387828
14130	Fcgr2b	Fc receptor, IgG, low affinity IIb, transcript variant 2	NA	0.487294	0.125366	1.401813	0.10308223	0.387828
12070	Bex3	brain expressed X-linked 3, transcript variant 1	NA	0.081653	7.533987	1.05823	0.10320482	0.388189
384059	Tlr12	toll-like receptor 12	NA	-0.37943	1.133666	-1.30083	0.1032487	0.388189
231915	Uspl1	ubiquitin specific peptidase like 1, transcript variant 3	NA	0.115489	5.755921	1.083342	0.10324986	0.388189
100383	Bsdc1	BSD domain containing 1	NA	-0.11207	5.827153	-1.08078	0.10333209	0.388408
69773	Timm29	translocase of inner mitochondrial membrane 29	NA	0.088108	6.757183	1.062975	0.10341742	0.388639
14599	Gh	growth hormone, transcript variant X1	NA	0.999824	-0.92441	1.999756	0.10348365	0.388798
67247	Mtarc2	mitochondrial amidoxime reducing component 2, tran	NA	0.145644	4.971294	1.106224	0.10354634	0.388876
76900	Ssbp4	single stranded DNA binding protein 4, transcript vari	NA	0.100429	6.134176	1.072092	0.10356973	0.388876
67791	6530411M01Rik	RIKEN cDNA 6530411M01 gene	NA	0.614977	-0.40352	1.531534	0.10357593	0.388876
118568531	LOC118568531	uncharacterized LOC118568531	NA	0.249259	2.754963	1.188597	0.10363038	0.388932
13800	Enah	ENAH actin regulator, transcript variant X21	NA	-0.07751	7.9868	-1.05519	0.10363884	0.388932
14894	Cfap20	cilia and flagella associated protein 20	NA	0.127442	5.278394	1.092355	0.10379341	0.389348
66168	Grina	glutamate receptor, ionotropic, N-methyl D-aspartate-	NA	0.098604	7.386576	1.070737	0.10380817	0.389348
102639908	Gm36109	predicted gene, 36109	NA	0.295466	2.058748	1.227281	0.1038224	0.389348
100041290	D4Ertd617e	DNA segment, Chr 4, ERATO Doi 617, expressed	NA	0.77208	-0.1474	1.70773	0.10386257	0.389348
244650	Phlpp2	PH domain and leucine rich repeat protein phosphata	NA	0.108446	5.237868	1.078066	0.1038691	0.389348
108153	Adamts7	a disintegrin-like and metallopeptidase (reprolysin typ	NA	-0.15007	4.741311	-1.10962	0.1039719	0.389643
50880	Scly	selenocysteine lyase, transcript variant X7	NA	-0.21724	3.152915	-1.16251	0.10411436	0.389958
140703	Emid1	EMI domain containing 1, transcript variant 1	NA	0.191681	3.080133	1.142094	0.10412969	0.389958
56417	Adar	adenosine deaminase, RNA-specific, transcript variar	NA	-0.08086	6.891226	-1.05765	0.10417164	0.389958
115488029	LOC115488029	uncharacterized LOC115488029	NA	0.352322	1.292719	1.276614	0.10417592	0.389958
68953	Chmp2a	charged multivesicular body protein 2A, transcript var	NA	0.097567	6.096007	1.069968	0.10419863	0.389958
241296	Lrrc8a	leucine rich repeat containing 8A VRAC subunit A	NA	-0.09066	5.951666	-1.06486	0.10424508	0.389958
30934	Tor1b	torsin family 1, member B	NA	-0.13037	4.913824	-1.09457	0.10426801	0.389958
233315	Mtmr10	myotubularin related protein 10	NA	0.146088	4.081493	1.106565	0.10427606	0.389958

18412	Sqstm1	sequestosome 1, transcript variant 1	NA	0.082854	7.711119	1.059111	0.10428478	0.389958
224143	Poglut1	protein O-glucosyltransferase 1, transcript variant 1	NA	-0.11028	5.529918	-1.07944	0.10429549	0.389958
22083	Ctr9	CTR9 homolog, Paf1/RNA polymerase II complex coı	NA	-0.08198	6.615927	-1.05847	0.10433971	0.389962
13395	Dlx5	distal-less homeobox 5, transcript variant 1	NA	-0.12588	4.806686	-1.09117	0.10434454	0.389962
68708	Rabl2	RAB, member RAS oncogene family-like 2	NA	-0.11589	5.218771	-1.08365	0.1044911	0.39039
67936	Wdr55	WD repeat domain 55, transcript variant 1	NA	-0.16278	4.227811	-1.11944	0.10450709	0.39039
233987	Zfp958	zinc finger protein 958, transcript variant 2	NA	0.134	4.352213	1.097332	0.10455207	0.390469
16337	Insr	insulin receptor, transcript variant 1	NA	0.090338	6.582857	1.06462	0.10459523	0.390497
235533	Gk5	glycerol kinase 5 (putative), transcript variant X3	NA	0.238354	2.370682	1.179646	0.10462842	0.390497
432720	Akr1c19	aldo-keto reductase family 1, member C19	NA	0.454875	0.594313	1.370664	0.10463175	0.390497
56207	Uchl5	ubiquitin carboxyl-terminal esterase L5, transcript var	NA	0.150372	4.842263	1.109856	0.10468243	0.390539
239096	Cdh24	cadherin-like 24, transcript variant X1	NA	-0.11759	5.732841	-1.08492	0.10471864	0.390539
118567910	LOC118567910	uncharacterized LOC118567910	NA	0.59577	-0.08486	1.511279	0.10472682	0.390539
75424	Zfp820	zinc finger protein 820	NA	0.272266	1.830474	1.207703	0.10473882	0.390539
20355	Sema4f	sema domain, immunoglobulin domain (Ig), TM doma	NA	-0.11779	5.088326	-1.08507	0.1047813	0.390607
52856	Mtg2	mitochondrial ribosome associated GTPase 2, transc	NA	-0.14401	4.14405	-1.10497	0.10495306	0.391158
666907	Ms4a4a	membrane-spanning 4-domains, subfamily A, membe	NA	-0.45873	0.631154	-1.37433	0.10498002	0.391169
69790	Med30	mediator complex subunit 30	NA	0.173638	4.652308	1.127899	0.10505566	0.391352
118567463	LOC118567463	uncharacterized LOC118567463	NA	0.439595	0.77619	1.356224	0.10507736	0.391352
22688	Zfp26	zinc finger protein 26, transcript variant X6	NA	0.120014	5.801935	1.086746	0.1052982	0.392085
51875	Tmem141	transmembrane protein 141, transcript variant 2	NA	0.227308	2.938008	1.170649	0.10533894	0.392112
245269	Nim1k	NIM1 serine/threonine protein kinase	NA	0.211281	3.473358	1.157716	0.10535366	0.392112
209630	Frmd4a	FERM domain containing 4A, transcript variant 3	NA	0.072484	7.918566	1.051526	0.10545885	0.392414
107328	Trpt1	tRNA phosphotransferase 1, transcript variant 2	NA	-0.22356	2.979988	-1.16761	0.10552815	0.392434
16524	Kcnj9	potassium inwardly-rectifying channel, subfamily J, m	NA	-0.19141	3.05966	-1.14188	0.1055342	0.392434
104662	Tsr1	TSR1 20S rRNA accumulation	NA	0.100627	5.376358	1.07224	0.10555487	0.392434
236848	Tmem185a	transmembrane protein 185A, transcript variant 1	NA	-0.11189	5.064218	-1.08064	0.10559687	0.392434
57266	Cxcl14	chemokine (C-X-C motif) ligand 14, transcript variant	NA	-0.25925	3.632804	-1.19685	0.10561341	0.392434
16560	Kif1a	kinesin family member 1A, transcript variant 4	NA	0.093493	9.368609	1.06695	0.10562482	0.392434
76295	Atp11b	ATPase, class VI, type 11B, transcript variant 2	NA	0.12243	6.011836	1.088567	0.10564657	0.392434
216028	Lrrtm3	leucine rich repeat transmembrane neuronal 3	NA	0.126828	4.504998	1.09189	0.10567654	0.392434
665113	Tnik	TRAF2 and NCK interacting kinase, transcript variant	NA	0.086476	7.364241	1.061773	0.1056812	0.392434
118567381	LOC118567381	uncharacterized LOC118567381	NA	0.58179	0.214408	1.496705	0.10570657	0.392438
102633986	Gm31679	predicted gene, 31679	NA	0.448411	0.324617	1.364536	0.10578483	0.392639
21685	Tef	thyrotroph embryonic factor, transcript variant X5	NA	0.119846	5.692574	1.086619	0.10588476	0.392921
64294	Itm2c	integral membrane protein 2C	NA	0.089283	6.812574	1.063841	0.10599538	0.393176
68975	Med27	mediator complex subunit 27, transcript variant 1	NA	-0.14062	4.514608	-1.10238	0.10600195	0.393176
244879	Npat	nuclear protein in the AT region, transcript variant X2	NA	0.131719	4.78508	1.095598	0.10614527	0.393618
320024	Nceh1	neutral cholesterol ester hydrolase 1, transcript variar	NA	0.173569	3.473993	1.127845	0.10623187	0.393849
243312	Elfn1	leucine rich repeat and fibronectin type III, extracellul	NA	-0.1631	4.158561	-1.11969	0.10626004	0.393864
319455	Pld5	phospholipase D family, member 5, transcript variant	NA	0.23711	2.452039	1.178629	0.10636007	0.394145
217558	G2e3	G2/M-phase specific E3 ubiquitin ligase, transcript va	NA	-0.11373	5.729594	-1.08202	0.1065397	0.394721
72898	Asphd2	aspartate beta-hydroxylase domain containing 2, tran	NA	0.127891	5.971594	1.092695	0.10684746	0.395771
12912	Creb1	cAMP responsive element binding protein 1, transcrip	NA	-0.08818	6.751638	-1.06303	0.1068953	0.395804
78658	Ncapd3	non-SMC condensin II complex, subunit D3, transcrip	NA	-0.11921	4.948612	-1.08614	0.10690512	0.395804
381903	Alg8	asparagine-linked glycosylation 8 (alpha-1,3-glucosyl	NA	-0.19131	3.511532	-1.1418	0.10704255	0.396223
19895	Rpia	ribose 5-phosphate isomerase A	NA	-0.13815	4.527869	-1.10049	0.10710869	0.396378
170760	Acbd3	acyl-Coenzyme A binding domain containing 3	NA	0.121682	5.676641	1.088002	0.10713472	0.396384
108168251	Gm46526	predicted gene, 46526	NA	0.518608	-0.2139	1.432572	0.10719562	0.396519
245386	Tmem255a	transmembrane protein 255A, transcript variant 3	NA	0.178515	4.240166	1.131718	0.10723257	0.396566
104103	Airn	antisense Igf2r RNA, transcript variant 2	NA	0.557925	0.303429	1.472151	0.10727596	0.396636
215015	Fam20b	family with sequence similarity 20, member B	NA	0.089855	6.691084	1.064263	0.10733675	0.39673
332397	Nanos1	nanos C2HC-type zinc finger 1	NA	0.169432	5.472379	1.124615	0.10736145	0.39673
260302	Gga3	golgi associated, gamma adaptin ear containing, ARI	NA	-0.11995	6.072598	-1.0867	0.10737453	0.39673
68743	Anln	anillin, actin binding protein, transcript variant X3	NA	-0.14859	3.895559	-1.10849	0.10740504	0.396752
66442	Spc25	SPC25, NDC80 kinetochore complex component, hoı	NA	-0.1743	4.293159	-1.12841	0.1075357	0.397085
76867	Rhbdd1	rhomboid domain containing 1, transcript variant 1	NA	-0.21986	2.527585	-1.16462	0.10757291	0.397085
102636765	Gm33746	predicted gene, 33746, transcript variant X1	NA	-0.41227	0.467041	-1.33078	0.10758348	0.397085
233335	Synm	synemin, intermediate filament protein, transcript vari	NA	0.163519	3.890291	1.120015	0.10760132	0.397085
22160	Twist1	twist basic helix-loop-helix transcription factor 1	NA	-0.34027	1.935712	-1.26599	0.10762355	0.397085
18208	Ntn1	netrin 1	NA	-0.11139	6.371927	-1.08027	0.10764141	0.397085
233204	Tbc1d17	TBC1 domain family, member 17, transcript variant X	NA	-0.11025	5.397814	-1.07941	0.1076872	0.397164
64661	Krtdap	keratinocyte differentiation associated protein, transc	NA	-1.28146	0.030686	-2.43085	0.10771573	0.397179
13835	Epha1	Eph receptor A1	NA	-0.56884	0.050504	-1.48333	0.10786136	0.397577
66092	Ghitm	growth hormone inducible transmembrane protein, tr	NA	0.084203	6.868805	1.060102	0.10787255	0.397577
19228	Pth1r	parathyroid hormone 1 receptor, transcript variant X1	NA	-0.23057	3.496302	-1.1733	0.10798621	0.397906
233912	Armc5	armadillo repeat containing 5	NA	-0.16659	3.611277	-1.1224	0.10810085	0.398238
277743	Fam131c	family with sequence similarity 131, member C	NA	0.156465	4.279428	1.114553	0.10813195	0.398262

72043	Sulf2	sulfatase 2, transcript variant X2	NA	0.095582	7.329988	1.068497	0.10819178	0.398369
73192	Xpot	exportin, tRNA (nuclear export receptor for tRNAs), tr	NA	0.093445	6.336895	1.066915	0.10820996	0.398369
13383	Dlg1	discs large MAGUK scaffold protein 1, transcript varia	NA	0.096532	5.744734	1.0692	0.10823909	0.398386
68140	Tigd2	tigger transposable element derived 2	NA	0.173689	3.515582	1.127939	0.10827921	0.398444
13992	Khdrbs3	KH domain containing, RNA binding, signal transduct	NA	-0.08704	7.216013	-1.06219	0.10837371	0.398674
17865	Mybl2	myeloblastosis oncogene-like 2	NA	-0.19846	3.483101	-1.14747	0.10839085	0.398674
66492	Zmat2	zinc finger, matrin type 2	NA	-0.09359	6.663104	-1.06702	0.10842603	0.398711
269181	Mgat4a	mannoside acetylglucosaminyltransferase 4, isoenzy	NA	0.124793	4.852404	1.090351	0.10848474	0.398711
50783	Lsm4	LSM4 homolog, U6 small nuclear RNA and mRNA de	NA	0.147828	4.930031	1.107901	0.10849879	0.398711
93692	Glrx	glutaredoxin, transcript variant 1	NA	0.143507	4.511546	1.104587	0.10850653	0.398711
118568632	LOC118568632	igE-binding protein-like	NA	0.92875	-0.79126	1.903626	0.10852326	0.398711
217218	Atxn7l3	ataxin 7-like 3, transcript variant 2	NA	-0.0842	7.844434	-1.0601	0.1086231	0.398988
18016	Nf2	neurofibromin 2, transcript variant 3	NA	-0.11449	7.642214	-1.08259	0.10865702	0.398995
71721	Fam13c	family with sequence similarity 13, member C, transc	NA	0.118203	5.328763	1.085382	0.10874223	0.398995
68566	Caly	calcyon neuron-specific vesicular protein, transcript v	NA	0.116301	5.541325	1.083952	0.10875094	0.398995
620760	2900079G21Rik	RIKEN cDNA 2900079G21 gene, transcript variant 1	NA	0.17963	3.320564	1.132593	0.10875521	0.398995
78267	Klhdc8b	kelch domain containing 8B, transcript variant 2	NA	-0.13516	5.259209	-1.09822	0.10875917	0.398995
12575	Cdkn1a	cyclin-dependent kinase inhibitor 1A (P21), transcript	NA	0.144097	5.283624	1.105039	0.10878777	0.398995
118567551	LOC118567551	uncharacterized LOC118567551	NA	0.443735	0.619628	1.360121	0.10879662	0.398995
68523	Ciao2b	cytosolic iron-sulfur assembly component 2B	NA	-0.14608	4.417646	-1.10656	0.10883939	0.399047
71435	Arhgap21	Rho GTPase activating protein 21, transcript variant 2	NA	0.095482	7.471646	1.068422	0.10886017	0.399047
110639	Prps2	phosphoribosyl pyrophosphate synthetase 2, transcri	NA	0.150946	4.355369	1.110297	0.10888464	0.399047
72739	Zkscan3	zinc finger with KRAB and SCAN domains 3, transcri	NA	-0.09937	5.933155	-1.0713	0.10891981	0.399086
215690	Nav1	neuron navigator 1	NA	0.091801	8.677996	1.065699	0.1089547	0.399124
20598	Smpd2	sphingomyelin phosphodiesterase 2, neutral	NA	0.152167	4.054203	1.111237	0.10901734	0.399125
231238	Sel1l3	sel-1 suppressor of lin-12-like 3 (C. elegans), transcri	NA	-0.10165	5.25328	-1.073	0.10902377	0.399125
15277	Hk2	hexokinase 2	NA	-0.19005	3.35346	-1.14081	0.10902854	0.399125
14088	Fancc	Fanconi anemia, complementation group C, transcrip	NA	-0.17037	3.333748	-1.12535	0.10909262	0.399184
115489117	Gm52428	predicted gene, 52428	NA	-0.56693	-0.28	-1.48136	0.10909566	0.399184
66291	Smim8	small integral membrane protein 8, transcript variant 4	NA	0.185155	3.445884	1.136939	0.10911827	0.399184
64138	Ctsz	cathepsin Z	NA	-0.13077	4.910776	-1.09488	0.10919939	0.399391
101502	Hsd3b7	hydroxy-delta-5-steroid dehydrogenase, 3 beta- and s	NA	-0.22307	2.806277	-1.16722	0.10922978	0.399413
100504467	Gm16596	predicted gene, 16596, transcript variant 2	NA	-0.39479	0.803645	-1.31475	0.10934821	0.399692
15550	Htr1a	5-hydroxytryptamine (serotonin) receptor 1A	NA	0.22769	2.623856	1.170958	0.10936938	0.399692
100034748	A930017K11Rik	RIKEN cDNA A930017K11 gene, transcript variant X	NA	0.426788	0.837042	1.344237	0.1093986	0.399692
231801	Agfg2	ArfGAP with FG repeats 2, transcript variant 3	NA	-0.13121	4.188147	-1.09521	0.10940427	0.399692
330953	Hcn4	hyperpolarization-activated, cyclic nucleotide-gated K	NA	-0.09201	6.021194	-1.06586	0.10960885	0.400349
634731	Susd1	sushi domain containing 1, transcript variant X4	NA	0.263108	1.922065	1.200061	0.10966562	0.400422
213402	Armc2	armadillo repeat containing 2	NA	-0.19974	3.000805	-1.14849	0.10967795	0.400422
233877	Kctd13	potassium channel tetramerisation domain containing	NA	0.136247	6.255325	1.099042	0.10978958	0.40058
277432	Vstm2l	V-set and transmembrane domain containing 2-like	NA	0.147437	5.929197	1.1076	0.10979101	0.40058
73274	Gpbp1	GC-rich promoter binding protein 1, transcript variant	NA	0.07514	7.340067	1.053463	0.10979528	0.40058
243864	Mill2	MHC I like leukocyte 2, transcript variant X5	NA	0.396018	1.470213	1.315871	0.10984357	0.400652
114896	Afg3l1	AFG3-like AAA ATPase 1, transcript variant 1	NA	-0.11885	5.854019	-1.08587	0.10986413	0.400652
218311	Zfp455	zinc finger protein 455	NA	-0.30036	1.722501	-1.23145	0.1099349	0.40082
73016	Kremen2	kringle containing transmembrane protein 2, transcrip	NA	-0.61289	0.11215	-1.52932	0.11000632	0.400991
116701	Fgfrl1	fibroblast growth factor receptor-like 1, transcript varia	NA	-0.16048	3.665113	-1.11766	0.11013173	0.401358
118567340	LOC118567340	uncharacterized LOC118567340	NA	0.113254	6.605788	1.081665	0.11020906	0.401415
67236	Cinp	cyclin-dependent kinase 2 interacting protein, transcr	NA	-0.1207	4.811643	-1.08726	0.11025937	0.401415
244608	Ccdc113	coiled-coil domain containing 113	NA	0.374148	0.918384	1.296074	0.11026521	0.401415
12832	Col5a2	collagen, type V, alpha 2	NA	-0.11918	5.97469	-1.08612	0.11028175	0.401415
268780	Egflam	EGF-like, fibronectin type III and laminin G domains,	NA	0.169095	3.813349	1.124353	0.11028581	0.401415
223593	Washc5	WASH complex subunit 5	NA	0.09018	5.899539	1.064503	0.11032721	0.401415
66313	Smurf2	SMAD specific E3 ubiquitin protein ligase 2, transcrip	NA	0.091186	6.214692	1.065246	0.11034103	0.401415
68671	Pcyt2	phosphate cytidylyltransferase 2, ethanolamine, trans	NA	-0.08611	5.987309	-1.06151	0.11036848	0.401415
219103	Cenpj	centromere protein J	NA	-0.17803	4.104397	-1.13134	0.11036945	0.401415
108907	Nusap1	nucleolar and spindle associated protein 1, transcript	NA	-0.16461	4.854677	-1.12086	0.11052349	0.401828
66454	Nmnat1	nicotinamide nucleotide adenylyltransferase 1, transc	NA	-0.22207	2.658183	-1.1664	0.11055909	0.401828
72415	Sgo1	shugoshin 1	NA	-0.20375	3.487238	-1.15169	0.11056886	0.401828
110058	Syt17	synaptotagmin XVII, transcript variant X6	NA	0.139958	3.906757	1.101873	0.11058176	0.401828
67384	Bag4	BCL2-associated athanogene 4	NA	0.110334	5.467607	1.079478	0.11063189	0.40184
446101	Xrra1	X-ray radiation resistance associated 1	NA	-0.54201	-0.07202	-1.456	0.11063437	0.40184
50877	Neu3	neuraminidase 3	NA	-0.32487	1.491984	-1.25255	0.11068881	0.401948
16772	Lama1	laminin, alpha 1, transcript variant X3	NA	0.145128	4.516427	1.105828	0.11073114	0.402012
11758	Prdx6	peroxiredoxin 6, transcript variant X1	NA	-0.09589	6.058754	-1.06873	0.11081159	0.402131
217835	Rin3	Ras and Rab interactor 3, transcript variant 2	NA	-0.19904	2.93501	-1.14793	0.11081335	0.402131
58182	Prokr1	prokineticin receptor 1, transcript variant X1	NA	-0.33978	1.572087	-1.26557	0.1108818	0.40229
21802	Tgfa	transforming growth factor alpha	NA	0.11075	5.138999	1.07979	0.11113578	0.403121

223604	Kcnk9	potassium channel, subfamily K, member 9	NA	0.150951	5.287028	1.110301	0.11122547	0.403134
217258	Abca8a	ATP-binding cassette, sub-family A (ABC1), member	NA	0.248992	2.234156	1.188376	0.11122726	0.403134
68142	Ino80	INO80 complex subunit	NA	0.102886	5.202073	1.07392	0.11127491	0.403134
103850	Nt5m	5',3'-nucleotidase, mitochondrial	NA	0.144326	4.35463	1.105214	0.11127725	0.403134
671535	Parp10	poly (ADP-ribose) polymerase family, member 10, tra	NA	-0.36153	1.428965	-1.28479	0.1112802	0.403134
11784	Apba2	amyloid beta (A4) precursor protein-binding, family A	NA	-0.1026	7.250522	-1.07371	0.11128802	0.403134
11449	Chrng	cholinergic receptor, nicotinic, gamma polypeptide, tr	NA	-0.71323	-0.96111	-1.63947	0.11138732	0.403404
67074	Mon2	MON2 homolog, regulator of endosome to Golgi traffi	NA	0.090755	6.271361	1.064927	0.11143255	0.403478
17828	Bloc1s5	biogenesis of lysosomal organelles complex-1, subur	NA	0.141727	4.332843	1.103225	0.11147411	0.403489
21953	Tnni2	troponin I, skeletal, fast 2	NA	-0.69358	0.89457	-1.61729	0.11148513	0.403489
11502	Adam9	a disintegrin and metallopeptidase domain 9 (meltrin	NA	0.110187	5.228369	1.079368	0.11152	0.403525
226744	Cnst	consortin, connexin sorting protein	NA	-0.09786	5.705852	-1.07019	0.11156472	0.403593
230603	Ttc39a	tetratricopeptide repeat domain 39A, transcript varian	NA	-0.40004	0.869758	-1.31954	0.11158843	0.403593
216881	Wscd1	WSC domain containing 1, transcript variant 3	NA	0.101408	5.829084	1.07282	0.11161638	0.403605
105242721	Gm38863	predicted gene, 38863	NA	0.630334	-0.13675	1.547923	0.11164666	0.403625
195208	Dcdc2a	doublecortin domain containing 2a, transcript variant	NA	0.262047	2.610238	1.199179	0.11173595	0.403858
239435	Aard	alanine and arginine rich domain containing protein	NA	0.328066	1.539299	1.255329	0.11182744	0.403942
67729	Mansc1	MANSC domain containing 1	NA	0.400645	0.984192	1.320098	0.11183177	0.403942
26399	Map2k6	mitogen-activated protein kinase kinase 6, transcript	NA	-0.13133	6.207346	-1.0953	0.11186547	0.403942
12314	Calm2	calmodulin 2, transcript variant 1	NA	0.088093	9.695876	1.062965	0.11188046	0.403942
278672	Duxbl1	double homeobox B-like 1, transcript variant X15	NA	0.190903	4.040048	1.141478	0.11190379	0.403942
328365	Zmiz1	zinc finger, MIZ-type containing 1, transcript variant X	NA	0.07742	8.615196	1.05513	0.11190819	0.403942
50773	Nt5c	5',3'-nucleotidase, cytosolic	NA	0.15909	4.287968	1.116583	0.1119925	0.403997
504193	Npcd	neuronal pentraxin chromo domain, transcript variant	NA	0.435067	6.914818	1.351974	0.11199459	0.403997
23829	C1ql1	complement component 1, q subcomponent-like 1	NA	0.30301	2.639794	1.233715	0.11204346	0.403997
227723	Prrc2b	proline-rich coiled-coil 2B, transcript variant 2	NA	0.074349	9.311528	1.052886	0.1120438	0.403997
56298	Atl2	atlastin GTPase 2, transcript variant X1	NA	0.096668	5.755816	1.069301	0.11207086	0.403997
56363	Tmeff2	transmembrane protein with EGF-like and two follista	NA	0.108221	5.412451	1.077898	0.11207236	0.403997
18087	Nktr	natural killer tumor recognition sequence, transcript v	NA	0.093366	7.035926	1.066856	0.11214564	0.40416
67120	Ttc14	tetratricopeptide repeat domain 14, transcript variant	NA	0.095271	6.330299	1.068266	0.11216719	0.40416
245000	Atr	ataxia telangiectasia and Rad3 related, transcript vari	NA	0.134535	4.765286	1.097739	0.11219335	0.404164
16008	Igfbp2	insulin-like growth factor binding protein 2, transcript	NA	0.087454	7.078632	1.062493	0.11240504	0.404837
15388	Hnrnpl	heterogeneous nuclear ribonucleoprotein L	NA	-0.08932	8.593947	-1.06387	0.11258874	0.405409
105245382	Gm9241	predicted gene 9241	NA	-0.59826	-0.29736	-1.51389	0.11277776	0.405989
18606	Enpp2	ectonucleotide pyrophosphatase/phosphodiesterase	NA	0.100414	5.642378	1.072081	0.11279962	0.405989
14788	Gpr162	G protein-coupled receptor 162, transcript variant 2	NA	-0.08365	6.670119	-1.0597	0.11286731	0.406098
243755	Slc13a4	solute carrier family 13 (sodium/sulfate symporters), r	NA	0.243461	2.885248	1.183829	0.11287982	0.406098
74252	Armc1	armadillo repeat containing 1, transcript variant 1	NA	0.098144	6.113169	1.070395	0.11299124	0.406379
102443350	Xndc1	Xrcc1 N-terminal domain containing 1, transcript varia	NA	-0.13609	4.820796	-1.09892	0.11301891	0.406379
17248	Mdm4	transformed mouse 3T3 cell double minute 4, transcr	NA	0.090809	7.027846	1.064967	0.11303298	0.406379
75571	Spata9	spermatogenesis associated 9, transcript variant X5	NA	0.656647	-0.27116	1.576415	0.1130771	0.406448
104601	Mycbpap	MYCBP associated protein	NA	-0.30635	1.454894	-1.23657	0.11313822	0.40645
17920	Myo6	myosin VI	NA	0.118708	5.441247	1.085762	0.11316615	0.40645
81904	Cacng7	calcium channel, voltage-dependent, gamma subunit	NA	-0.10042	7.261366	-1.07208	0.11320751	0.40645
118567439	LOC118567439	protein enabled homolog	NA	1.973182	0.759205	3.92633	0.11328095	0.40645
100126229	Gm12758	predicted gene 12758	NA	0.409367	1.051682	1.328103	0.11328154	0.40645
102635133	Gm10777	predicted gene 10777, transcript variant X4	NA	0.22879	2.882697	1.171852	0.11329796	0.40645
108169069	Gm16001	predicted gene 16001	NA	0.559325	-0.303	1.47358	0.11334385	0.40645
53331	Stx7	syntaxin 7, transcript variant 2	NA	0.070709	7.728408	1.050233	0.11341242	0.40645
12228	Btg3	BTG anti-proliferation factor 3, transcript variant 2	NA	0.147533	3.81052	1.107674	0.11341278	0.40645
28075	Desi1	desumoylating isopeptidase 1, transcript variant 1	NA	-0.09759	6.132941	-1.06999	0.11342211	0.40645
14357	Dtx1	deltex 1, E3 ubiquitin ligase	NA	-0.07571	6.977431	-1.05388	0.11342375	0.40645
217648	Gm527	predicted gene 527	NA	0.255471	2.120561	1.193725	0.11342525	0.40645
102632812	Gm30789	predicted gene, 30789	NA	0.301606	1.854673	1.232515	0.11343425	0.40645
14457	Gas7	growth arrest specific 7, transcript variant X5	NA	0.081481	7.107573	1.058104	0.11343811	0.40645
239570	Ttc38	tetratricopeptide repeat domain 38, transcript variant	NA	-0.211	2.524456	-1.15749	0.11345226	0.40645
20658	Son	Son DNA binding protein, transcript variant 1	NA	0.07583	8.734784	1.053967	0.11348433	0.406475
69710	Arap1	ArfGAP with RhoGAP domain, ankyrin repeat and PH	NA	-0.16164	3.815431	-1.11856	0.11357908	0.406628
74158	Josd1	Josephin domain containing 1	NA	-0.09205	5.818238	-1.06588	0.11361522	0.406628
67391	Fundc2	FUN14 domain containing 2	NA	0.105118	5.549091	1.075583	0.11363113	0.406628
224020	Pi4ka	phosphatidylinositol 4-kinase alpha, transcript variant	NA	-0.08126	6.948507	-1.05794	0.11363593	0.406628
72354	Ttc4	tetratricopeptide repeat domain 4, transcript variant 1	NA	-0.09342	6.070579	-1.06689	0.11365183	0.406628
72759	Tmem135	transmembrane protein 135, transcript variant X2	NA	0.120443	5.106954	1.087069	0.11368004	0.406639
26561	Mmp23	matrix metallopeptidase 23, transcript variant X1	NA	-0.31647	1.497674	-1.24528	0.11373361	0.406653
73754	Thap1	THAP domain containing, apoptosis associated prote	NA	-0.15075	3.880897	-1.11014	0.1137338	0.406653
53620	Vamp5	vesicle-associated membrane protein 5, transcript va	NA	0.34165	1.489082	1.267205	0.11380911	0.406782
67495	Tmem167b	transmembrane protein 167B	NA	-0.09538	6.117276	-1.06835	0.11384234	0.406782
66925	Sdhd	succinate dehydrogenase complex, subunit D, integra	NA	0.085586	6.144679	1.061119	0.11386752	0.406782

237222	Ofd1	OFD1, centriole and centriolar satellite protein	NA	0.139066	4.542468	1.101192	0.11388075	0.406782
70044	Tut1	terminal uridylyl transferase 1, U6 snRNA-specific	NA	-0.12518	4.763719	-1.09065	0.11389506	0.406782
69774	Ms4a6b	membrane-spanning 4-domains, subfamily A, membe	NA	0.424978	0.646873	1.342552	0.11402238	0.407048
16597	Klf12	Kruppel-like factor 12, transcript variant X5	NA	0.085549	6.640606	1.061092	0.11403092	0.407048
15251	Hif1a	hypoxia inducible factor 1, alpha subunit, transcript va	NA	0.106955	7.02959	1.076953	0.11404438	0.407048
14389	Gab2	growth factor receptor bound protein 2-associated pro	NA	0.121812	6.193105	1.088101	0.11421246	0.407558
66350	Pla2g12a	phospholipase A2, group XIIA, transcript variant 1	NA	0.178974	3.913614	1.132078	0.11430121	0.407743
72899	Macrod2	mono-ADP ribosylhydrolase 2, transcript variant 2	NA	0.096233	5.820375	1.068979	0.11431429	0.407743
66254	Dimt1	DIM1 dimethyladenosine transferase 1-like (S. cerevi	NA	-0.15312	4.508664	-1.11197	0.11440206	0.407881
170833	Hook2	hook microtubule tethering protein 2, transcript variar	NA	0.122864	4.971894	1.088895	0.11440311	0.407881
100040322	3830408C21Rik	RIKEN cDNA 3830408C21 gene	NA	0.30727	1.696779	1.237364	0.11452667	0.408232
319192	H2ac19	H2A clustered histone 19	NA	-0.9511	0.775348	-1.93335	0.11457205	0.408261
71837	1700003E16Rik	RIKEN cDNA 1700003E16 gene	NA	-0.27242	2.139589	-1.20783	0.11459988	0.408261
20623	Snrk	SNF related kinase, transcript variant 5	NA	0.084105	6.577225	1.06003	0.11465577	0.408261
320661	D5Ertd579e	DNA segment, Chr 5, ERATO Doi 579, expressed	NA	0.088986	6.725065	1.063622	0.11465811	0.408261
67037	Pmf1	polyamine-modulated factor 1, transcript variant 1	NA	-0.1791	3.622242	-1.13218	0.11466025	0.408261
21955	Tnnt1	troponin T1, skeletal, slow, transcript variant 1	NA	-0.34521	1.745484	-1.27034	0.11470887	0.408344
106565	Dlk2	delta like non-canonical Notch ligand 2, transcript var	NA	-0.13992	4.426024	-1.10184	0.11475791	0.408353
12508	Cd53	CD53 antigen	NA	0.343913	1.389924	1.269195	0.11477142	0.408353
27425	Atp5l	ATP synthase, H+ transporting, mitochondrial F0 com	NA	0.106335	6.896547	1.07649	0.11480173	0.408353
13078	Cyp1b1	cytochrome P450, family 1, subfamily b, polypeptide	NA	0.157207	3.752058	1.115126	0.11485167	0.408353
13390	Dlx1	distal-less homeobox 1	NA	-0.12013	6.991795	-1.08683	0.11485252	0.408353
59006	Myoz2	myozenin 2, transcript variant 1	NA	-0.70052	-0.16944	-1.6251	0.1148618	0.408353
665433	H2ac23	H2A clustered histone 23	NA	-0.15882	4.992045	-1.11637	0.11496274	0.408622
19325	Rab10	RAB10, member RAS oncogene family	NA	0.071404	8.185806	1.050739	0.11500758	0.408693
117150	Pip4k2c	phosphatidylinositol-5-phosphate 4-kinase, type II, ga	NA	-0.1231	4.783532	-1.08907	0.11506132	0.408794
68952	Tlcd3b	TLC domain containing 3B, transcript variant 1	NA	-0.10991	7.568381	-1.07916	0.11514977	0.40893
208647	Creb3l2	cAMP responsive element binding protein 3-like 2	NA	-0.26288	2.188809	-1.19987	0.1151498	0.40893
67164	Lipt2	lipoyl(octanoyl) transferase 2 (putative)	NA	0.233476	2.518576	1.175664	0.11518071	0.408951
105377	Slf1	SMC5-SMC6 complex localization factor 1, transcript	NA	0.109093	5.384257	1.07855	0.11525952	0.409141
56323	Dnajb5	DnaJ heat shock protein family (Hsp40) member B5,	NA	-0.08652	6.676506	-1.0618	0.11530193	0.409166
271849	Shc4	SHC (Src homology 2 domain containing) family, mer	NA	0.262533	2.682127	1.199583	0.11532341	0.409166
102640722	Gm36722	predicted gene, 36722, transcript variant X1	NA	0.429529	0.827627	1.346794	0.11534191	0.409166
100043424	Morrbid	myeloid RNA regulator of BCL2L11 induced cell deat	NA	0.580771	-0.20956	1.495649	0.11540449	0.409281
22421	Wnt7a	wingless-type MMTV integration site family, member	NA	-0.13303	4.742013	-1.09659	0.11542482	0.409281
240614	Ranbp6	RAN binding protein 6	NA	0.104086	5.835508	1.074813	0.11548351	0.4094
22183	Zrsr1	zinc finger (CCCH type), RNA binding motif and serin	NA	0.11445	4.968354	1.082562	0.1155561	0.409476
140493	Kcnn3	potassium intermediate/small conductance calcium-a	NA	0.238521	3.112439	1.179783	0.11560279	0.409476
56784	Ralgapa1	Ral GTPase activating protein, alpha subunit 1, trans	NA	0.093745	6.54426	1.067137	0.11560406	0.409476
320590	Svopl	SV2 related protein homolog (rat)-like	NA	0.543927	-0.24347	1.457936	0.11560537	0.409476
69480	Ttc9	tetratricopeptide repeat domain 9	NA	0.088978	6.081951	1.063616	0.11567181	0.4096
631624	Gm7072	predicted gene 7072, transcript variant 2	NA	0.120352	4.687293	1.087	0.11569073	0.4096
66975	Trappc13	trafficking protein particle complex 13, transcript varia	NA	-0.10537	5.619801	-1.07577	0.11577939	0.409628
100416706	Zfp729b	zinc finger protein 729b	NA	0.155876	4.024769	1.114098	0.11578667	0.409628
102633579	Gm31370	predicted gene, 31370	NA	-0.52102	0.249122	-1.43497	0.11579271	0.409628
13507	Dsc3	desmocollin 3, transcript variant X2	NA	-0.41993	0.617901	-1.33786	0.11579933	0.409628
60411	Cenpk	centromere protein K, transcript variant 5	NA	-0.24327	2.410889	-1.18367	0.11584689	0.409707
53972	Ngef	neuronal guanine nucleotide exchange factor, transcr	NA	0.136113	4.529949	1.09894	0.11590171	0.409812
27412	Peg12	paternally expressed 12	NA	-0.24021	2.935046	-1.18116	0.11599469	0.40985
19821	Rnf2	ring finger protein 2, transcript variant 4	NA	0.080302	6.920361	1.057239	0.11602027	0.40985
12936	Pcdha4	protocadherin alpha 4	NA	0.158463	3.936486	1.116097	0.11602305	0.40985
224860	Plcl2	phospholipase C-like 2	NA	0.089127	6.030854	1.063726	0.11604361	0.40985
69487	Ndufaf5	NADH:ubiquinone oxidoreductase complex assembly	NA	0.140552	4.604504	1.102327	0.11610724	0.40985
64654	Fgf23	fibroblast growth factor 23	NA	0.467289	0.044035	1.382509	0.11612339	0.40985
100041735	Gm3488	predicted gene, 3488, transcript variant 2	NA	1.073668	3.272444	2.104778	0.1161247	0.40985
16512	Kcnh3	potassium voltage-gated channel, subfamily H (eag-r	NA	-0.25006	2.591789	-1.18926	0.11613802	0.40985
74051	Steap2	six transmembrane epithelial antigen of prostate 2, tra	NA	0.200025	3.071262	1.148718	0.11613941	0.40985
74043	Pex26	peroxisomal biogenesis factor 26, transcript variant X	NA	-0.13161	4.854958	-1.09551	0.11618162	0.409911
666244	Tmsb15b1	thymosin beta 15b1	NA	0.303201	1.88258	1.233879	0.11627373	0.410147
56737	Alg2	asparagine-linked glycosylation 2 (alpha-1,3-mannos	NA	0.073969	8.104132	1.052609	0.11635104	0.410176
72093	2010320M18Rik	RIKEN cDNA 2010320M18 gene	NA	0.221766	3.097489	1.16616	0.11637022	0.410176
26457	Slc27a1	solute carrier family 27 (fatty acid transporter), memb	NA	-0.08917	5.974456	-1.06376	0.11638991	0.410176
14084	Faf1	Fas-associated factor 1	NA	0.086534	6.285132	1.061816	0.11640142	0.410176
21869	Nkx2-1	NK2 homeobox 1	NA	-0.14782	4.697963	-1.10789	0.11640807	0.410176
12479	Cd1d1	CD1d1 antigen, transcript variant 4	NA	-0.14874	4.344778	-1.1086	0.11651365	0.410434
218215	Rnf144b	ring finger protein 144B, transcript variant 2	NA	0.351863	2.240137	1.276208	0.11653191	0.410434
66997	Psmd12	proteasome (prosome, macropain) 26S subunit, non-	NA	0.092912	6.468022	1.066521	0.11671611	0.410994
74287	Kcmf1	potassium channel modulatory factor 1, transcript var	NA	0.09778	6.154819	1.070125	0.11682716	0.411173

80743	Vps16	VSP16 CORVET/HOPS core subunit, transcript varia	NA	0.087093	5.909411	1.062228	0.11685813	0.411173
115488490	LOC115488490	galectin-related protein-like	NA	0.363788	1.514887	1.2868	0.11688269	0.411173
100039596	Tcf24	transcription factor 24	NA	0.438296	0.513905	1.355003	0.11688664	0.411173
16401	Itga4	integrin alpha 4	NA	0.138933	3.875969	1.10109	0.11689321	0.411173
13382	Dld	dihydrolipoamide dehydrogenase	NA	0.080458	6.787149	1.057353	0.1169503	0.411285
268448	Phf12	PHD finger protein 12, transcript variant X3	NA	-0.07834	6.694991	-1.0558	0.11697701	0.411288
18222	Numb	NUMB endocytic adaptor protein, transcript variant X4	NA	-0.13711	4.661183	-1.0997	0.11700167	0.411288
668662	Gm9292	predicted gene 9292, transcript variant X16	NA	0.232312	3.113086	1.174716	0.11712385	0.411628
68572	Mrpl58	mitochondrial ribosomal protein L58, transcript varian	NA	0.112888	5.113302	1.081391	0.11715167	0.411637
102642612	Gm38618	predicted gene, 38618	NA	-0.17868	3.664605	-1.13185	0.11720556	0.411738
212516	Efcab12	EF-hand calcium binding domain 12	NA	-0.33765	1.77655	-1.26369	0.1172491	0.411758
230577	Pars2	prolyl-tRNA synthetase (mitochondrial)(putative), tran	NA	0.259298	3.081687	1.196896	0.11726194	0.411758
17385	Mmp11	matrix metallopeptidase 11, transcript variant 1	NA	0.185914	4.01982	1.137538	0.11736289	0.412009
108168338	Gm46594	predicted gene, 46594, transcript variant X1	NA	-0.51847	0.107429	-1.43244	0.11740932	0.412009
224826	Ubr2	ubiquitin protein ligase E3 component n-recognin 2, tı	NA	0.088162	6.153849	1.063015	0.11740941	0.412009
93711	Pcdhga3	protocadherin gamma subfamily A, 3	NA	0.112829	4.974889	1.081346	0.11753171	0.412292
67672	0610040B10Rik	RIKEN cDNA 0610040B10 gene	NA	0.445606	0.441385	1.361886	0.11754073	0.412292
72472	Slc16a10	solute carrier family 16 (monocarboxylic acid transpoı	NA	-0.21376	2.781005	-1.1597	0.11761375	0.412403
279706	Nup62cl	nucleoporin 62 C-terminal like, transcript variant X4	NA	0.375726	0.793452	1.297493	0.11762292	0.412403
14417	Gad2	glutamic acid decarboxylase 2	NA	0.096403	6.758061	1.069105	0.11771892	0.41265
72349	Dusp3	dual specificity phosphatase 3 (vaccinia virus phosph	NA	0.105611	5.072945	1.07595	0.11791378	0.413085
74230	1700016K19Rik	RIKEN cDNA 1700016K19 gene	NA	-0.45252	0.634658	-1.36842	0.11791446	0.413085
19299	Abcd3	ATP-binding cassette, sub-family D (ALD), member 3	NA	0.10712	6.503291	1.077076	0.11799603	0.413085
208164	Fam180a	family with sequence similarity 180, member A	NA	0.613332	-0.48996	1.529789	0.11799648	0.413085
75740	Egfem1	EGF-like and EMI domain containing 1, transcript var	NA	0.330319	2.023609	1.257292	0.11802778	0.413085
66136	Znrd1	zinc ribbon domain containing 1, transcript variant 2	NA	-0.17665	3.910602	-1.13026	0.11806798	0.413085
277333	Gm5069	predicted pseudogene 5069	NA	0.409585	0.831549	1.328304	0.11808915	0.413085
102634450	Gm32031	predicted gene, 32031	NA	0.455257	0.646311	1.371027	0.11809977	0.413085
218763	Lrrc3b	leucine rich repeat containing 3B	NA	0.152995	3.545742	1.111875	0.11810851	0.413085
100503799	Gm7931	predicted pseudogene 7931	NA	-0.77347	-0.28412	-1.70937	0.11811061	0.413085
268512	Slc26a11	solute carrier family 26, member 11, transcript variant	NA	-0.2097	2.598804	-1.15645	0.11812208	0.413085
68032	Emc4	ER membrane protein complex subunit 4	NA	0.112412	5.38669	1.081034	0.11816047	0.41313
11529	Adh7	alcohol dehydrogenase 7 (class IV), mu or sigma poly	NA	-0.62877	-0.36215	-1.54625	0.11838481	0.413825
19982	Rpl36a	ribosomal protein L36A	NA	0.081554	7.342211	1.058157	0.11843368	0.413907
217379	Ubxn2a	UBX domain protein 2A	NA	0.094025	5.679626	1.067344	0.11852099	0.41408
71436	Flrt3	fibronectin leucine rich transmembrane protein 3, trar	NA	0.10272	5.644321	1.073796	0.11853398	0.41408
72658	2700097O09Rik	RIKEN cDNA 2700097O09 gene, transcript variant X	NA	-0.15786	4.382201	-1.11563	0.11865022	0.414245
234138	Tti2	TELO2 interacting protein 2, transcript variant 1	NA	-0.14577	4.56185	-1.10632	0.11865142	0.414245
11609	Agtr2	angiotensin II receptor, type 2	NA	-0.31335	2.90914	-1.24259	0.11865765	0.414245
72326	2500004C02Rik	RIKEN cDNA 2500004C02 gene	NA	-0.20385	3.087931	-1.15177	0.11873885	0.41444
21338	Tacr3	tachykinin receptor 3	NA	0.277035	2.336246	1.211702	0.11879832	0.414462
102635428	Gm26614	predicted gene, 26614, transcript variant X3	NA	0.405393	0.613893	1.32445	0.11880137	0.414462
67026	Thap4	THAP domain containing 4, transcript variant 1	NA	0.11358	4.833556	1.08191	0.11882157	0.414462
442803	A830005F24Rik	RIKEN cDNA A830005F24 gene	NA	0.602184	-0.30471	1.518012	0.11886841	0.414536
19361	Rad51	RAD51 recombinase, transcript variant 1	NA	-0.17481	3.505199	-1.12881	0.11889694	0.414547
71481	Alpk1	alpha-kinase 1, transcript variant X9	NA	-0.28663	2.137861	-1.21979	0.11899338	0.414794
78465	Ccdc190	coiled-coil domain containing 190, transcript variant 1	NA	0.217235	3.220429	1.162503	0.11902717	0.414823
230784	Sesn2	sestrin 2	NA	-0.22723	2.634092	-1.17058	0.11906744	0.414871
16518	Kcnj2	potassium inwardly-rectifying channel, subfamily J, m	NA	0.170019	3.197134	1.125073	0.11911676	0.414871
74521	Ppp4r4	protein phosphatase 4, regulatory subunit 4, transcrip	NA	0.201792	3.202329	1.150126	0.1191298	0.414871
72154	Zfp157	zinc finger protein 157	NA	0.080419	6.478319	1.057325	0.11914292	0.414871
72129	Pex13	peroxisomal biogenesis factor 13, transcript variant 1	NA	-0.10099	5.296426	-1.07251	0.11917262	0.414886
74330	Dnajc14	DnaJ heat shock protein family (Hsp40) member C14	NA	-0.0832	6.244499	-1.05936	0.11923972	0.41496
69640	Fam83g	family with sequence similarity 83, member G	NA	-0.61759	-0.65603	-1.5343	0.11924492	0.41496
14674	Gna13	guanine nucleotide binding protein, alpha 13, transcri	NA	0.109608	6.010137	1.078935	0.11928926	0.415026
20425	Shmt1	serine hydroxymethyltransferase 1 (soluble), transcri	NA	-0.22313	3.134785	-1.16727	0.11936333	0.415175
19091	Prkg1	protein kinase, cGMP-dependent, type I, transcript va	NA	0.178687	3.356598	1.131853	0.11938314	0.415175
13848	Ephb6	Eph receptor B6, transcript variant 1	NA	-0.10539	5.611826	-1.07578	0.11941726	0.415205
21826	Thbs2	thrombospondin 2	NA	0.19687	3.407472	1.146209	0.11948708	0.415258
19691	Recql	RecQ protein-like, transcript variant 4	NA	0.113193	4.676271	1.081619	0.11948772	0.415258
72461	Prcp	prolylcarboxypeptidase (angiotensinase C)	NA	-0.14062	4.24122	-1.10238	0.11950905	0.415258
19088	Prkar2b	protein kinase, cAMP dependent regulatory, type II b	NA	0.07489	7.889242	1.053281	0.1195444	0.415292
22350	Ezr	ezrin	NA	-0.09965	6.606205	-1.07151	0.11961326	0.415303
12038	Bche	butyrylcholinesterase	NA	0.336395	2.140879	1.262598	0.11963246	0.415303
20185	Ncor1	nuclear receptor co-repressor 1, transcript variant 1	NA	0.073951	7.72369	1.052596	0.11967253	0.415303
231464	Cnot6l	CCR4-NOT transcription complex, subunit 6-like, trar	NA	0.124308	5.966739	1.089985	0.11968691	0.415303
115489486	Gm52533	predicted gene, 52533	NA	0.44108	0.555483	1.35762	0.11969843	0.415303
100504421	2900076A07Rik	RIKEN cDNA 2900076A07 gene, transcript variant 2	NA	0.215667	2.587845	1.161241	0.1197008	0.415303

73338	Itpripl1	inositol 1,4,5-triphosphate receptor interacting proteir	NA	-0.22845	3.27395	-1.17158	0.11981973	0.415444
107767	Scamp1	secretory carrier membrane protein 1, transcript varia	NA	0.097949	7.493489	1.070251	0.11982232	0.415444
56843	Trpm5	transient receptor potential cation channel, subfamily	NA	-0.39105	1.362549	-1.31135	0.11982722	0.415444
18719	Pip5k1b	phosphatidylinositol-4-phosphate 5-kinase, type 1 bet	NA	0.263615	1.972032	1.200483	0.11984365	0.415444
20724	Serpinb5	serine (or cysteine) peptidase inhibitor, clade B, mem	NA	-0.82177	-0.49995	-1.76757	0.11999289	0.415873
115486979	Gm51604	predicted gene, 51604	NA	-0.32924	1.248463	-1.25635	0.12005644	0.416005
101867	Rrp8	ribosomal RNA processing 8, transcript variant X1	NA	-0.14832	4.497029	-1.10828	0.12008792	0.416025
319480	Itga11	integrin alpha 11	NA	-0.23092	2.327255	-1.17358	0.12014092	0.416041
231997	Fkbp14	FK506 binding protein 14, transcript variant 1	NA	-0.15417	3.498903	-1.11278	0.12016182	0.416041
14923	Guk1	guanylate kinase 1, transcript variant X1	NA	0.135655	5.164336	1.098592	0.12017289	0.416041
244216	Zfp771	zinc finger protein 771, transcript variant 1	NA	-0.20571	3.805667	-1.15325	0.12019478	0.416041
15166	Hcn2	hyperpolarization-activated, cyclic nucleotide-gated K	NA	0.143992	4.359175	1.104959	0.1202817	0.416253
66962	Swsap1	SWIM type zinc finger 7 associated protein 1	NA	-0.14657	3.747619	-1.10694	0.12039702	0.416543
102639683	LOC102639683	uncharacterized LOC102639683	NA	-0.30955	1.566693	-1.23932	0.12041662	0.416543
17156	Man1a2	mannosidase, alpha, class 1A, member 2	NA	0.116494	6.226654	1.084097	0.12048432	0.416651
67246	Resf1	retroelement silencing factor 1, transcript variant X6	NA	-0.10769	4.98733	-1.0775	0.12049902	0.416651
52575	Trmt10c	tRNA methyltransferase 10C, mitochondrial RNase P	NA	0.122218	4.410977	1.088407	0.12056601	0.416794
15415	Hoxb7	homeobox B7, transcript variant X1	NA	0.891555	0.12989	1.855175	0.12063227	0.416935
70790	Ubr5	ubiquitin protein ligase E3 component n-recognin 5, t	NA	0.087319	7.689369	1.062394	0.12069272	0.417046
66860	Tanc1	tetratricopeptide repeat, ankyrin repeat and coiled-co	NA	-0.14316	4.031098	-1.10432	0.12071579	0.417046
11548	Adra1b	adrenergic receptor, alpha 1b, transcript variant 1	NA	0.20769	3.035811	1.154838	0.12076406	0.417124
329908	Usp24	ubiquitin specific peptidase 24, transcript variant X4	NA	0.112607	6.340848	1.08118	0.12081507	0.417212
14815	Nr3c1	nuclear receptor subfamily 3, group C, member 1, tra	NA	0.139698	4.196001	1.101675	0.12091935	0.417483
100929	Tyw1	tRNA-yW synthesizing protein 1 homolog (S. cerevisi	NA	-0.13741	4.525053	-1.09993	0.12095297	0.417511
213773	Tbl3	transducin (beta)-like 3	NA	-0.10642	5.16001	-1.07655	0.12109848	0.417925
228421	Kif18a	kinesin family member 18A	NA	-0.20492	3.158839	-1.15262	0.12115803	0.418008
216505	Pik3ip1	phosphoinositide-3-kinase interacting protein 1	NA	0.136008	4.380985	1.098861	0.12117416	0.418008
18125	Nos1	nitric oxide synthase 1, neuronal	NA	-0.14806	5.280706	-1.10808	0.12121221	0.418051
66205	Cd302	CD302 antigen, transcript variant 1	NA	0.251792	2.168638	1.190685	0.12132284	0.418308
72068	Cnot2	CCR4-NOT transcription complex, subunit 2, transcri	NA	-0.07964	6.723226	-1.05676	0.12133817	0.418308
214791	Sertad4	SERTA domain containing 4, transcript variant 2	NA	0.092189	6.042365	1.065987	0.12140563	0.418452
109346	Ankrd39	ankyrin repeat domain 39, transcript variant 3	NA	0.215845	2.620859	1.161384	0.12147447	0.418601
225583	Minar2	membrane integral NOTCH2 associated receptor 2, t	NA	0.089914	6.568533	1.064307	0.12154075	0.418654
72167	Thumpd2	THUMP domain containing 2	NA	0.175006	3.116915	1.128969	0.12157555	0.418654
81003	Trim23	tripartite motif-containing 23, transcript variant 1	NA	0.126609	5.919773	1.091725	0.12157669	0.418654
72194	Fbxl20	F-box and leucine-rich repeat protein 20	NA	0.08394	6.187455	1.059909	0.12159288	0.418654
74039	Nfam1	Nfat activating molecule with ITAM motif 1, transcript	NA	-0.31141	1.431099	-1.24092	0.12164711	0.418752
12633	Cflar	CASP8 and FADD-like apoptosis regulator, transcript	NA	-0.13826	4.254796	-1.10058	0.12172862	0.418944
71268	Lrrfip2	leucine rich repeat (in FLII) interacting protein 2, trans	NA	0.104974	5.584546	1.075475	0.12177393	0.419012
93714	Pcdhga6	protocadherin gamma subfamily A, 6	NA	0.127069	5.161675	1.092073	0.12184107	0.419092
75415	Arhgap12	Rho GTPase activating protein 12, transcript variant)	NA	0.109446	6.115341	1.078814	0.1218488	0.419092
236930	Ercc6l	excision repair cross-complementing rodent repair de	NA	-0.22649	2.384972	-1.16999	0.12192143	0.419103
93747	Echs1	enoyl Coenzyme A hydratase, short chain, 1, mitocho	NA	0.091826	5.847287	1.065718	0.1219366	0.419103
13361	Dhfr	dihydrofolate reductase	NA	-0.14823	4.092549	-1.10821	0.12195136	0.419103
17187	Max	Max protein, transcript variant 2	NA	-0.07986	6.351475	-1.05691	0.12202113	0.419103
73834	Atp6v1d	ATPase, H+ transporting, lysosomal V1 subunit D	NA	0.092284	6.613576	1.066056	0.12202695	0.419103
72199	Mms19	MMS19 cytosolic iron-sulfur assembly component, tra	NA	0.104404	5.445014	1.07505	0.12202854	0.419103
22709	Zfp51	zinc finger protein 51, transcript variant 1	NA	-0.17999	3.362081	-1.13288	0.12209112	0.419103
14799	Gria1	glutamate receptor, ionotropic, AMPA1 (alpha 1), trar	NA	0.083069	7.32503	1.059269	0.1221001	0.419103
29877	Hdgfl3	HDGF like 3	NA	0.073322	8.287958	1.052137	0.12210911	0.419103
21331	T2	brachyury 2, transcript variant X23	NA	0.353884	1.251316	1.277997	0.12210958	0.419103
140498	Rxfp2	relaxin/insulin-like family peptide receptor 2, transcrip	NA	0.527829	-0.30049	1.441758	0.12215568	0.419173
66526	Tceanc2	transcription elongation factor A (SII) N-terminal and	NA	-0.10956	4.983292	-1.0789	0.12224294	0.419308
77832	Tchp	trichoplein, keratin filament binding	NA	-0.1405	4.40683	-1.10229	0.12225262	0.419308
242474	Tmem245	transmembrane protein 245, transcript variant X2	NA	0.141541	5.730501	1.103083	0.12227254	0.419308
100042150	Nrg2	neuregulin 2, transcript variant 1	NA	0.157823	3.597808	1.115602	0.12234816	0.419479
23850	Pappa2	pappalysin 2	NA	-0.18594	3.276151	-1.13756	0.12237897	0.419497
11491	Adam17	a disintegrin and metallopeptidase domain 17, transc	NA	-0.11804	4.951029	-1.08526	0.12241984	0.419548
70432	Rufy2	RUN and FYVE domain-containing 2, transcript varia	NA	0.096716	6.010738	1.069337	0.12250126	0.419604
20227	Sart1	squamous cell carcinoma antigen recognized by T ce	NA	-0.091	6.513664	-1.06511	0.12250447	0.419604
68941	1110018N20Rik	RIKEN cDNA 1110018N20 gene, transcript variant X	NA	0.261529	2.08119	1.198748	0.12251352	0.419604
435145	Shisa8	shisa family member 8	NA	0.458206	0.520847	1.373833	0.1226205	0.419882
68628	Fbxw9	F-box and WD-40 domain protein 9	NA	0.119658	5.262845	1.086477	0.12272393	0.420148
27401	Skp2	S-phase kinase-associated protein 2, transcript variar	NA	-0.13813	4.819116	-1.10048	0.12282407	0.420402
78777	Tepsin	TEPSIN, adaptor related protein complex 4 accessor	NA	-0.12705	4.239482	-1.09206	0.12289872	0.420569
67845	Rnf115	ring finger protein 115	NA	0.09011	5.826703	1.064451	0.12297883	0.420755
14009	Etv1	ets variant 1, transcript variant X10	NA	0.099955	5.465895	1.07174	0.12301247	0.420782
224650	Anks1	ankyrin repeat and SAM domain containing 1, transcr	NA	-0.08961	6.404192	-1.06408	0.12304796	0.420815

245638	Tbc1d8b	TBC1 domain family, member 8B	NA	-0.30037	2.341815	-1.23146	0.12308648	0.420858
56044	Rala	v-ral simian leukemia viral oncogene A (ras related)	NA	0.073116	7.696351	1.051987	0.12337957	0.421649
74107	Cep55	centrosomal protein 55, transcript variant 3	NA	-0.20361	3.425544	-1.15158	0.12339305	0.421649
110094	Phka2	phosphorylase kinase alpha 2, transcript variant X6	NA	0.125127	4.695994	1.090604	0.12339718	0.421649
14884	Gtf2h1	general transcription factor II H, polypeptide 1, transc	NA	0.110742	5.506076	1.079784	0.12342138	0.421649
233033	Samd4b	sterile alpha motif domain containing 4B	NA	-0.08598	6.286683	-1.06141	0.12349378	0.421807
68196	Hsbp1	heat shock factor binding protein 1	NA	0.069886	7.944569	1.049634	0.12355921	0.421868
224598	Zfp758	zinc finger protein 758, transcript variant X5	NA	-0.13453	4.07992	-1.09774	0.12356344	0.421868
66648	Tpgs2	tubulin polyglutamylase complex subunit 2, transcript	NA	0.079438	6.499868	1.056606	0.12361177	0.421945
94227	Pi15	peptidase inhibitor 15	NA	0.451334	0.524951	1.367304	0.12369198	0.42213
18619	Penk	preproenkephalin, transcript variant 2	NA	0.152474	4.514533	1.111474	0.12377563	0.422327
19053	Ppp2cb	protein phosphatase 2 (formerly 2A), catalytic subuni	NA	0.094631	7.237882	1.067792	0.12387333	0.422572
70369	Bag5	BCL2-associated athanogene 5, transcript variant 1	NA	0.091906	5.492664	1.065777	0.12391182	0.422607
16519	Kcnj3	potassium inwardly-rectifying channel, subfamily J, m	NA	0.113535	4.548819	1.081876	0.12393564	0.422607
66085	Eif3f	eukaryotic translation initiation factor 3, subunit F	NA	0.091688	7.920436	1.065616	0.12399702	0.422728
227682	Trub2	TruB pseudouridine (psi) synthase family member 2,	NA	0.138291	4.738717	1.1006	0.12408468	0.422938
66665	Msantd3	Myb/SANT-like DNA-binding domain containing 3, tra	NA	0.100582	6.251193	1.072206	0.12415536	0.42309
15466	Hrh2	histamine receptor H2, transcript variant X3	NA	0.395981	1.081766	1.315837	0.12421657	0.423125
244672	Cwf19l2	CWF19-like 2, cell cycle control (S. pombe)	NA	0.139438	4.365728	1.101476	0.12425459	0.423125
217935	Wdr60	WD repeat domain 60	NA	-0.1305	4.237206	-1.09467	0.12426648	0.423125
106952	Arap3	ArfGAP with RhoGAP domain, ankyrin repeat and PH	NA	-0.18796	4.094028	-1.13915	0.12426945	0.423125
11782	Ap4s1	adaptor-related protein complex AP-4, sigma 1, trans	NA	0.130081	4.619906	1.094355	0.12429638	0.423125
677884	Pakap	paralemmin A kinase anchor protein, transcript variar	NA	0.084031	6.75202	1.059975	0.12432172	0.423125
320806	Gfm2	G elongation factor, mitochondrial 2, transcript varian	NA	-0.10902	5.149999	-1.0785	0.12438512	0.423253
17756	Map2	microtubule-associated protein 2, transcript variant X	NA	0.071015	10.13821	1.050456	0.12443663	0.423264
216154	Med16	mediator complex subunit 16, transcript variant 1	NA	-0.11876	5.499308	-1.0858	0.12444042	0.423264
15500	Hsf2	heat shock factor 2	NA	0.089836	6.221958	1.064249	0.12454607	0.423391
13353	Dgcr6	DiGeorge syndrome critical region gene 6, transcript	NA	0.107058	5.237883	1.077029	0.12456296	0.423391
192193	Edem1	ER degradation enhancer, mannosidase alpha-like 1,	NA	0.102523	4.98369	1.07365	0.1245682	0.423391
118449	Synpo2	synaptopodin 2	NA	-0.30446	2.120829	-1.23495	0.12458183	0.423391
223722	Mcat	malonyl CoA:ACP acyltransferase (mitochondrial)	NA	0.133277	4.203849	1.096782	0.12462122	0.423436
22222	Ubr1	ubiquitin protein ligase E3 component n-recognin 1, t	NA	0.09536	5.859044	1.068332	0.12481589	0.423647
78248	Armcx1	armadillo repeat containing, X-linked 1, transcript vari	NA	0.09762	6.332322	1.070007	0.12481678	0.423647
74551	Pck2	phosphoenolpyruvate carboxykinase 2 (mitochondria	NA	-0.13935	4.162829	-1.10141	0.1248181	0.423647
237010	Klhl4	kelch-like 4, transcript variant 2	NA	0.233648	3.237444	1.175804	0.12482407	0.423647
20444	St3gal2	ST3 beta-galactoside alpha-2,3-sialyltransferase 2, tr	NA	-0.08129	6.229054	-1.05796	0.12484881	0.423647
24074	Taf7	TATA-box binding protein associated factor 7	NA	-0.12215	4.584423	-1.08836	0.12488859	0.423647
110197	Dgkg	diacylglycerol kinase, gamma, transcript variant X4	NA	0.222343	2.842411	1.166627	0.12495927	0.423647
56436	Adrm1	adhesion regulating molecule 1	NA	0.087389	6.149397	1.062446	0.12499762	0.423647
18108	Nmt2	N-myristoyltransferase 2, transcript variant 4	NA	0.077861	6.875065	1.055452	0.12500033	0.423647
108168413	Gm46652	predicted gene, 46652, transcript variant X2	NA	0.615536	-0.38007	1.532127	0.12501205	0.423647
12789	Cnga2	cyclic nucleotide gated channel alpha 2, transcript va	NA	0.654472	-0.68048	1.57404	0.12501458	0.423647
71955	Ist1	increased sodium tolerance 1 homolog (yeast), trans	NA	0.070809	7.294099	1.050306	0.12501624	0.423647
19185	Psmd4	proteasome (prosome, macropain) 26S subunit, non-	NA	0.078488	6.667461	1.055911	0.12502176	0.423647
13684	Eif4e	eukaryotic translation initiation factor 4E, transcript va	NA	0.08023	7.405876	1.057187	0.12510757	0.42385
77827	Krba1	KRAB-A domain containing 1, transcript variant X5	NA	-0.09512	5.746159	-1.06815	0.12516783	0.423966
22059	Trp53	transformation related protein 53, transcript variant 1	NA	-0.09998	6.088076	-1.07176	0.12525264	0.424104
20482	Skil	SKI-like, transcript variant 3	NA	0.104665	5.976533	1.075245	0.12526069	0.424104
56306	Sinhcaf	SIN3-HDAC complex associated factor, transcript var	NA	-0.11852	6.003064	-1.08562	0.12532261	0.424225
68857	Dtwd2	DTW domain containing 2, transcript variant 1	NA	0.233314	2.278612	1.175532	0.12542336	0.424477
235300	Tlcd5	TLC domain containing 5, transcript variant 1	NA	-0.11525	4.85089	-1.08316	0.12544915	0.424477
67610	Rspry1	ring finger and SPRY domain containing 1, transcript	NA	0.118908	5.75599	1.085913	0.12549353	0.424539
15199	Hebp1	heme binding protein 1	NA	0.24428	2.074981	1.184501	0.12555969	0.424674
72284	LTO1	ABCE maturation factor, transcript variant 2	NA	-0.15063	4.607358	-1.11006	0.12561852	0.424785
67065	Polr3d	polymerase (RNA) III (DNA directed) polypeptide D, t	NA	-0.11948	5.050253	-1.08634	0.12566096	0.424812
243780	Dennd11	DENN domain containing 11	NA	-0.09964	6.022658	-1.07151	0.12572875	0.424812
217684	Susd6	sushi domain containing 6, transcript variant X1	NA	-0.13274	5.076194	-1.09638	0.12572943	0.424812
11605	Gla	galactosidase, alpha	NA	0.20741	3.108518	1.154614	0.12573109	0.424812
100048534	Cfap43	cilia and flagella associated protein 43, transcript vari	NA	0.278071	2.054842	1.212573	0.1257682	0.42485
20745	Spock1	sparc/osteonectin, cwcv and kazal-like domains prote	NA	0.08773	7.200415	1.062697	0.12581188	0.424909
67260	Cers4	ceramide synthase 4, transcript variant X3	NA	0.095873	6.154976	1.068712	0.12600456	0.425395
381801	Tatdn2	TatD DNase domain containing 2, transcript variant 1	NA	0.098547	5.479548	1.070694	0.12600825	0.425395
14400	Gabrb1	gamma-aminobutyric acid (GABA) A receptor, subuni	NA	0.110071	5.283148	1.079281	0.12606519	0.425491
270162	Elmod1	ELMO/CED-12 domain containing 1	NA	0.07556	6.83826	1.05377	0.12608886	0.425491
12343	Capza2	capping protein (actin filament) muscle Z-line, alpha 2	NA	0.07279	7.460825	1.051749	0.12613723	0.425566
68497	Arel1	apoptosis resistant E3 ubiquitin protein ligase 1, trans	NA	0.074539	6.688284	1.053025	0.12619856	0.425685
68027	Tmem178	transmembrane protein 178	NA	0.122729	4.690705	1.088792	0.12625416	0.425784
52715	Ccdc43	coiled-coil domain containing 43	NA	0.114764	5.02235	1.082798	0.1262906	0.425819

66596	Gtf3a	general transcription factor III A	NA	0.099347	5.18647	1.071288	0.12646047	0.426238
11666	Abcd1	ATP-binding cassette, sub-family D (ALD), member 1	NA	-0.14351	4.227687	-1.10459	0.12648728	0.426238
109910	Zfp91	zinc finger protein 91	NA	0.078841	7.216032	1.056169	0.12651117	0.426238
320207	Pik3r5	phosphoinositide-3-kinase regulatory subunit 5, trans	NA	0.540509	-0.31433	1.454486	0.12651966	0.426238
109637	Upk1a	uroplakin 1A	NA	0.502561	0.157158	1.416726	0.12656444	0.42627
11514	Adcy8	adenylate cyclase 8, transcript variant 1	NA	0.174628	3.943309	1.128674	0.12658174	0.42627
66292	Mrps21	mitochondrial ribosomal protein S21, transcript variar	NA	-0.11419	4.931041	-1.08237	0.12664493	0.426395
15511	Hspa1b	heat shock protein 1B	NA	-0.31772	2.321279	-1.24636	0.12669957	0.426486
21780	Tfam	transcription factor A, mitochondrial	NA	0.087951	5.680445	1.06286	0.12672455	0.426486
67604	Get4	golgi to ER traffic protein 4, transcript variant 1	NA	0.100079	5.582959	1.071832	0.12682585	0.426739
269254	Setx	senataxin, transcript variant X2	NA	0.086062	6.61035	1.061469	0.12687402	0.426813
66743	Rnf220	ring finger protein 220, transcript variant X11	NA	0.073091	7.151266	1.051968	0.12700297	0.427045
50706	Postn	periostin, osteoblast specific factor, transcript variant	NA	-0.08913	5.61734	-1.06373	0.12706442	0.427045
102635071	Gm26944	predicted gene, 26944, transcript variant X4	NA	-0.17881	3.189797	-1.13195	0.12706966	0.427045
67900	Mtfp1	mitochondrial fission process 1	NA	-0.2406	1.990475	-1.18148	0.12707523	0.427045
68802	Mypn	myopalladin	NA	-0.44939	0.467166	-1.36546	0.12707756	0.427045
66208	Nenf	neuron derived neurotrophic factor	NA	0.140233	4.577473	1.102083	0.1271004	0.427045
19654	Rbm6	RNA binding motif protein 6, transcript variant X7	NA	-0.11146	6.921676	-1.08032	0.12724985	0.427459
18196	Nsg1	neuron specific gene family member 1, transcript vari	NA	0.070974	9.492551	1.050426	0.12742815	0.427969
74351	Ddx23	DEAD box helicase 23, transcript variant X1	NA	-0.08999	6.427741	-1.06437	0.12750527	0.428071
319670	Eml5	echinoderm microtubule associated protein like 5	NA	0.10018	6.357088	1.071907	0.12752999	0.428071
94249	Slc24a3	solute carrier family 24 (sodium/potassium/calcium e:	NA	0.099778	5.906669	1.071609	0.12753747	0.428071
668880	Stard9	START domain containing 9, transcript variant X7	NA	0.169835	4.221394	1.12493	0.12758265	0.428135
72425	Katnbl1	katanin p80 subunit B like 1	NA	0.132611	5.063975	1.096276	0.12762141	0.428176
239691	AU021092	expressed sequence AU021092	NA	0.193606	2.760365	1.143618	0.12765634	0.428205
67437	Ssr3	signal sequence receptor, gamma	NA	0.070961	7.961741	1.050416	0.12777111	0.428378
192174	Rwdd4a	RWD domain containing 4A	NA	-0.10677	5.260717	-1.07681	0.12777399	0.428378
56354	Dnajc7	DnaJ heat shock protein family (Hsp40) member C7,	NA	0.075373	7.799729	1.053633	0.12778676	0.428378
63986	Gmfg	glia maturation factor, gamma, transcript variant 2	NA	0.478228	0.270756	1.393032	0.12785121	0.428476
20788	Srebf2	sterol regulatory element binding factor 2, transcript v	NA	-0.07335	7.746007	-1.05216	0.12789554	0.428476
434130	Ccdc8	coiled-coil domain containing 8	NA	-0.16172	4.219221	-1.11862	0.12790662	0.428476
16443	Itsn1	intersectin 1 (SH3 domain protein 1A), transcript varia	NA	-0.08857	7.928642	-1.06332	0.12793118	0.428476
21391	Tbxas1	thromboxane A synthase 1, platelet, transcript varian	NA	-0.42999	0.374627	-1.34722	0.12796879	0.428476
330319	Wipf3	WAS/WASL interacting protein family, member 3, trai	NA	-0.11106	4.745815	-1.08002	0.12797412	0.428476
72361	Ces2g	carboxylesterase 2G, transcript variant X1	NA	-0.95745	-1.02632	-1.94188	0.12803138	0.42858
115487346	Gm51737	predicted gene, 51737	NA	0.589427	-0.07254	1.504649	0.12814003	0.428771
77264	Zfp142	zinc finger protein 142, transcript variant X7	NA	-0.14414	4.865817	-1.10507	0.12814126	0.428771
70676	Gulp1	GULP, engulfment adaptor PTB domain containing 1	NA	-0.16745	3.248829	-1.12307	0.12825043	0.428961
271127	Adamts16	a disintegrin-like and metallopeptidase (reprolysin typ	NA	0.20475	2.580502	1.152487	0.12825057	0.428961
192950	Nacad	NAC alpha domain containing	NA	-0.08049	6.863246	-1.05738	0.12828446	0.428986
72293	Nkd2	naked cuticle 2, transcript variant 1	NA	0.170309	4.383368	1.125299	0.12843561	0.429403
20897	Stra6	stimulated by retinoic acid gene 6, transcript variant X	NA	-0.16466	3.476669	-1.1209	0.12848617	0.429484
19241	Tmsb4x	thymosin, beta 4, X chromosome	NA	0.073261	10.17007	1.052092	0.12854971	0.429608
269116	Nfasc	neurofascin, transcript variant X35	NA	-0.09862	7.789097	-1.07075	0.12862899	0.429785
68014	Zwilch	zwilch kinetochore protein	NA	-0.2067	3.471317	-1.15404	0.12874622	0.430075
77011	Ticrr	TOPBP1-interacting checkpoint and replication regula	NA	-0.19502	3.303777	-1.14474	0.12876866	0.430075
101122	Rpusd3	RNA pseudouridylate synthase domain containing 3,	NA	0.224518	2.153968	1.168387	0.12883163	0.430196
235028	Zfp426	zinc finger protein 426, transcript variant 2	NA	0.090956	5.598325	1.065076	0.12885796	0.430196
56314	Zfp113	zinc finger protein 113	NA	-0.13821	4.999624	-1.10054	0.12903551	0.43065
102641516	Gm38561	predicted gene, 38561	NA	-0.14912	3.920391	-1.10889	0.1290468	0.43065
11774	Ap3b1	adaptor-related protein complex 3, beta 1 subunit	NA	-0.09894	5.516784	-1.07099	0.12917247	0.430793
24088	Tlr2	toll-like receptor 2	NA	0.405152	0.407532	1.324229	0.12917251	0.430793
76246	Rtf1	RTF1, Paf1/RNA polymerase II complex component	NA	0.073623	7.238166	1.052356	0.12919821	0.430793
70350	Basp1	brain abundant, membrane attached signal protein 1	NA	0.082718	10.03906	1.059011	0.12924368	0.430793
217366	Lrrc45	leucine rich repeat containing 45	NA	-0.1257	5.635011	-1.09104	0.12925641	0.430793
80986	Ckap2	cytoskeleton associated protein 2	NA	-0.15385	4.458267	-1.11254	0.1293025	0.430793
11757	Prdx3	peroxiredoxin 3	NA	-0.09518	5.420281	-1.0682	0.12930635	0.430793
53604	Zpbp	zona pellucida binding protein, transcript variant X4	NA	0.396726	0.567055	1.316517	0.12931554	0.430793
215387	Ncaph	non-SMC condensin I complex, subunit H	NA	-0.18489	4.423678	-1.13673	0.12932907	0.430793
22061	Trp63	transformation related protein 63, transcript variant 5	NA	-0.64854	0.175477	-1.56759	0.12935441	0.430793
105835	Sgsm3	small G protein signaling modulator 3, transcript varia	NA	-0.11029	5.207392	-1.07945	0.12939077	0.430826
18975	Polg	polymerase (DNA directed), gamma, transcript variar	NA	-0.10905	5.540427	-1.07851	0.12948538	0.431053
72401	Slc43a1	solute carrier family 43, member 1, transcript variant	NA	-0.29879	1.279706	-1.23011	0.12953249	0.431072
94191	Adarb2	adenosine deaminase, RNA-specific, B2, transcript v	NA	0.106366	4.802453	1.076513	0.12958108	0.431072
73991	Atl1	atlastin GTPase 1	NA	0.088491	6.342403	1.063258	0.12958994	0.431072
13163	Daxx	Fas death domain-associated protein, transcript varia	NA	-0.11354	5.501046	-1.08188	0.12959701	0.431072
66108	Ndufa9	NADH:ubiquinone oxidoreductase subunit A9	NA	0.079932	6.235266	1.056969	0.12968902	0.43116
320896	C330020E22Rik	RIKEN cDNA C330020E22 gene	NA	0.376433	0.561558	1.298128	0.12970101	0.43116

78428	Pym1	PYM homolog 1, exon junction complex associated fa	NA	0.104327	5.041819	1.074993	0.12970306	0.43116
72434	Lypd3	Ly6/Plaur domain containing 3	NA	-0.64282	-0.39642	-1.56138	0.12988745	0.431636
56176	Pigp	phosphatidylinositol glycan anchor biosynthesis, clas:	NA	0.12033	5.11119	1.086984	0.12990894	0.431636
16988	Lst1	leukocyte specific transcript 1	NA	0.5311	-0.24699	1.44503	0.12992595	0.431636
19015	Ppard	peroxisome proliferator activator receptor delta, trans	NA	-0.14288	4.307646	-1.10411	0.13000633	0.431815
268980	Strn	striatin, calmodulin binding protein, transcript variant :	NA	0.116287	5.528685	1.083942	0.13003727	0.43183
394432	Ugt1a7c	UDP glucuronosyltransferase 1 family, polypeptide A'	NA	0.514216	0.02727	1.428218	0.13028304	0.432558
17308	Mgat1	mannoside acetylglucosaminyltransferase 1, transcrip	NA	-0.09132	5.530303	-1.06535	0.13040002	0.432858
399609	C130046K22Rik	RIKEN cDNA C130046K22 gene, transcript variant 2	NA	-0.18629	3.173645	-1.13784	0.13061095	0.433422
74754	Dhcr24	24-dehydrocholesterol reductase	NA	0.072037	7.020834	1.0512	0.13062339	0.433422
66193	Pithd1	PITH (C-terminal proteasome-interacting domain of th	NA	0.124117	4.922152	1.08984	0.13074845	0.433553
233826	Palb2	partner and localizer of BRCA2, transcript variant X2	NA	-0.26089	2.114046	-1.19822	0.13076575	0.433553
11657	Alb	albumin	NA	-0.83006	-1.41378	-1.77776	0.1307786	0.433553
319478	Cxxc4	CXXC finger 4	NA	0.109838	6.949643	1.079107	0.13078345	0.433553
102635775	Gm33027	predicted gene, 33027, transcript variant 2	NA	0.609048	-0.41334	1.525252	0.1307959	0.433553
11799	Birc5	baculoviral IAP repeat-containing 5, transcript variant	NA	-0.18387	4.261137	-1.13592	0.13085054	0.433644
16977	Lrrc23	leucine rich repeat containing 23, transcript variant X:	NA	-0.2514	2.122188	-1.19036	0.13087746	0.433644
320879	B230217O12Rik	RIKEN cDNA B230217O12 gene	NA	0.322337	1.38791	1.250355	0.13090335	0.433644
545490	Zfp973	zinc finger protein 973	NA	0.700112	0.100859	1.624631	0.13097544	0.433764
52700	Txndc17	thioredoxin domain containing 17	NA	-0.09559	5.270017	-1.0685	0.13099302	0.433764
94221	Gopc	golgi associated PDZ and coiled-coil motif containing	NA	0.119944	5.560474	1.086692	0.13103932	0.433829
73571	1700096K18Rik	RIKEN cDNA 1700096K18 gene	NA	0.236469	2.306491	1.178106	0.13108533	0.433894
237928	Phospho1	phosphatase, orphan 1	NA	-0.34125	1.957751	-1.26686	0.1311628	0.433989
328801	Zfp414	zinc finger protein 414, transcript variant 1	NA	0.105272	5.061139	1.075697	0.13117769	0.433989
442804	Kirrel3os	kirre like nephrin family adhesion molecule 3, opposit	NA	-0.44279	0.188376	-1.35923	0.13120657	0.433989
94093	Trim33	tripartite motif-containing 33, transcript variant X1	NA	0.09583	6.800727	1.06868	0.13122811	0.433989
68107	Cntd1	cyclin N-terminal domain containing 1	NA	-0.44821	-0.01732	-1.36435	0.13127649	0.433989
69004	6330418K02Rik	RIKEN cDNA 6330418K02 gene	NA	-0.34489	1.206123	-1.27006	0.13128189	0.433989
22335	Vdac3	voltage-dependent anion channel 3, transcript variant	NA	0.07118	7.756815	1.050575	0.13131971	0.433989
15200	Hbegf	heparin-binding EGF-like growth factor	NA	0.175593	3.938281	1.129429	0.13132741	0.433989
20404	Sh3gl2	SH3-domain GRB2-like 2	NA	0.082859	6.493709	1.059115	0.13138198	0.434042
60599	Trp53inp1	transformation related protein 53 inducible nuclear pr	NA	-0.08971	5.856063	-1.06416	0.1314849	0.434042
240025	Dact2	dishevelled-binding antagonist of beta-catenin 2, tran	NA	0.228289	2.140537	1.171445	0.13148947	0.434042
78255	Ralgps2	Ral GEF with PH domain and SH3 binding motif 2, tra	NA	0.084394	7.007219	1.060242	0.13149061	0.434042
230734	Yrdc	yrdC domain containing (E.coli)	NA	0.125592	4.629669	1.090955	0.13151776	0.434042
14635	Galk1	galactokinase 1	NA	0.152525	4.193322	1.111513	0.1315273	0.434042
224671	Btbd9	BTB (POZ) domain containing 9, transcript variant 1	NA	0.077383	6.645402	1.055102	0.13155815	0.434042
76983	Scfd1	Sec1 family domain containing 1, transcript variant 1	NA	0.108473	5.4318	1.078086	0.1315623	0.434042
105180375	Tmem265	transmembrane protein 265	NA	0.158342	3.684249	1.116004	0.13158356	0.434042
71091	Cdkl1	cyclin-dependent kinase-like 1 (CDC2-related kinase)	NA	0.164713	3.185243	1.120943	0.13163725	0.434131
54126	Arhgef7	Rho guanine nucleotide exchange factor (GEF7), tran	NA	0.068863	7.744936	1.04889	0.13172998	0.434349
13244	Degs1	delta(4)-desaturase, sphingolipid 1, transcript variant	NA	0.115774	5.449791	1.083556	0.13193171	0.434922
83453	Chrdl1	chordin-like 1, transcript variant 2	NA	0.271461	2.310852	1.207029	0.13195748	0.434922
72147	Zbtb46	zinc finger and BTB domain containing 46, transcript	NA	0.132763	4.752774	1.096392	0.13200924	0.435005
216134	Pdxk	pyridoxal (pyridoxine, vitamin B6) kinase	NA	-0.10087	5.874176	-1.07242	0.13225763	0.435735
216805	Flcn	folliculin, transcript variant X2	NA	-0.10668	5.357776	-1.07675	0.13228712	0.435744
269033	4930503L19Rik	RIKEN cDNA 4930503L19 gene, transcript variant 6	NA	-0.12147	4.403801	-1.08784	0.13232614	0.435784
56699	Cdc42ep4	CDC42 effector protein (Rho GTPase binding) 4, tran	NA	-0.07435	6.461901	-1.05289	0.13240368	0.435952
218138	Gmds	GDP-mannose 4, 6-dehydratase	NA	0.130615	4.032115	1.09476	0.13244965	0.436015
17319	Mif	macrophage migration inhibitory factor (glycosylation-	NA	0.14105	6.743394	1.102708	0.13258109	0.436359
108156	Mthfd1	methylenetetrahydrofolate dehydrogenase (NADP+ d	NA	-0.11782	5.225306	-1.08509	0.13262534	0.436416
107993	Bfsp2	beaded filament structural protein 2, phakinin, transcr	NA	0.536296	0.052404	1.450245	0.13269459	0.436481
16865	Eif2d	eukaryotic translation initiation factor 2D, transcript va	NA	0.105628	5.458174	1.075963	0.13269873	0.436481
75089	Uhrf1bp1l	UHRF1 (ICBP90) binding protein 1-like	NA	0.110874	5.924172	1.079882	0.13278218	0.436668
71665	Fuca1	fucosidase, alpha-L- 1, tissue	NA	0.118593	4.900765	1.085675	0.13286517	0.436852
545260	Arsi	arylsulfatase i	NA	-0.27911	1.629058	-1.21345	0.13294518	0.437027
70381	Tecpr1	tectonin beta-propeller repeat containing 1, transcript	NA	-0.09398	6.219998	-1.06731	0.13304944	0.437232
93736	Aff4	AF4/FMR2 family, member 4, transcript variant X1	NA	0.081857	7.757494	1.058379	0.13306388	0.437232
100710	Pds5b	PDS5 cohesin associated factor B, transcript variant	NA	0.084897	6.794424	1.060612	0.13308828	0.437232
53623	Gria3	glutamate receptor, ionotropic, AMPA3 (alpha 3), tran	NA	0.094913	5.537331	1.068001	0.13325296	0.437685
66482	Exoc2	exocyst complex component 2, transcript variant 1	NA	0.084788	6.06223	1.060532	0.133331	0.437851
77106	Tmem181a	transmembrane protein 181A, transcript variant 1	NA	0.085746	6.7514	1.061237	0.13335714	0.437851
117149	Tirap	toll-interleukin 1 receptor (TIR) domain-containing ad:	NA	0.255617	2.409673	1.193846	0.13339913	0.437862
22628	Ywhag	tyrosine 3-monooxygenase/tryptophan 5-monooxygei	NA	-0.07618	10.00753	-1.05422	0.1334145	0.437862
234594	Cnot1	CCR4-NOT transcription complex, subunit 1, transcri	NA	0.084722	6.987017	1.060483	0.13350026	0.438055
72103	Aplf	aprataxin and PNKP like factor, transcript variant 3	NA	0.185146	3.568681	1.136932	0.13370726	0.438646
22134	Tgoln1	trans-golgi network protein	NA	0.0969	5.687958	1.069473	0.13374655	0.438687
114230	Aipl1	aryl hydrocarbon receptor-interacting protein-like 1	NA	0.814034	-0.76321	1.75812	0.13387835	0.43903

56427	Tubd1	tubulin, delta 1, transcript variant 2	NA	-0.22238	2.673446	-1.16666	0.13405168	0.43951
15223	Foxj1	forkhead box J1	NA	-0.1412	4.426254	-1.10282	0.13408817	0.439541
208158	Map6d1	MAP6 domain containing 1	NA	-0.2072	2.375127	-1.15444	0.1342001	0.439761
207474	Kctd12b	potassium channel tetramerisation domain containing	NA	0.17636	3.221305	1.130029	0.13421693	0.439761
19250	Ptpn14	protein tyrosine phosphatase, non-receptor type 14	NA	-0.20367	3.404664	-1.15162	0.1342363	0.439761
66686	Dcbld1	discoidin, CUB and LCCL domain containing 1, trans	NA	0.114884	4.467017	1.082888	0.13432925	0.439977
270118	Maml2	mastermind like transcriptional coactivator 2, transcri	NA	0.108358	5.155585	1.078001	0.13437912	0.439992
105243585	Gm39469	predicted gene, 39469	NA	0.195322	2.792507	1.144979	0.13441283	0.439992
18423	Otx1	orthodenticle homeobox 1	NA	-0.12046	4.297637	-1.08708	0.13442997	0.439992
21778	Tex9	testis expressed gene 9, transcript variant X15	NA	0.133092	4.712942	1.096641	0.13444197	0.439992
77771	Csrnp3	cysteine-serine-rich nuclear protein 3, transcript varia	NA	0.098866	7.298537	1.070931	0.13450208	0.4401
225028	Map4k3	mitogen-activated protein kinase kinase kinase kinas	NA	0.081163	6.485481	1.057871	0.13453308	0.440113
22221	Ubp1	upstream binding protein 1, transcript variant X17	NA	0.069134	7.262071	1.049086	0.13459576	0.440165
20544	Slc9a1	solute carrier family 9 (sodium/hydrogen exchanger),	NA	-0.09221	5.321454	-1.066	0.13460311	0.440165
116939	Pnpla3	patatin-like phospholipase domain containing 3, trans	NA	0.184589	3.190059	1.136493	0.13468712	0.440351
71520	Grap	GRB2-related adaptor protein	NA	0.249591	2.225765	1.18887	0.13471935	0.440368
382571	Kcnf1	potassium voltage-gated channel, subfamily F, memb	NA	0.163871	3.79262	1.120289	0.13484108	0.440678
214425	Cilp	cartilage intermediate layer protein, nucleotide pyrop	NA	-0.52732	0.16338	-1.44125	0.1349048	0.440769
100503890	Pet100	PET100 homolog	NA	-0.16046	3.200935	-1.11765	0.13492323	0.440769
72128	2610008E11Rik	RIKEN cDNA 2610008E11 gene, transcript variant X	NA	0.085637	6.449	1.061156	0.13504436	0.44096
76222	Magef1	MAGE family member F1	NA	0.137015	4.196156	1.099627	0.13509059	0.44096
67803	Limd2	LIM domain containing 2, transcript variant 2	NA	-0.09391	5.94197	-1.06726	0.13509572	0.44096
214580	Pstk	phosphoseryl-tRNA kinase, transcript variant 1	NA	0.195815	3.206552	1.145371	0.13511779	0.44096
209239	Gan	giant axonal neuropathy	NA	0.273587	1.792649	1.20881	0.13515111	0.44096
226527	Cryzl2	crystallin zeta like 2, transcript variant 1	NA	-0.2278	2.184466	-1.17105	0.13516098	0.44096
14773	Grk5	G protein-coupled receptor kinase 5, transcript varian	NA	0.177654	4.496243	1.131043	0.13517141	0.44096
116940	Tgs1	trimethylguanosine synthase 1	NA	0.095236	5.937827	1.06824	0.13525226	0.441086
67738	Ppid	peptidylprolyl isomerase D (cyclophilin D), transcript	NA	-0.08591	6.600506	-1.06136	0.13526406	0.441086
77857	9430065F17Rik	RIKEN cDNA 9430065F17 gene, transcript variant 1	NA	0.864993	-0.22085	1.821331	0.13531329	0.441158
102866	Pls3	plastin 3 (T-isoform), transcript variant 2	NA	0.093488	5.490984	1.066947	0.13546875	0.441517
19072	Prep	prolyl endopeptidase, transcript variant X1	NA	-0.07552	6.450163	-1.05374	0.13549456	0.441517
117934532	Gm48552	predicted gene, 48552	NA	1.544671	-0.07935	2.917374	0.13552473	0.441517
11958	Atp5k	ATP synthase, H+ transporting, mitochondrial F1F0 c	NA	0.147871	5.66985	1.107933	0.13553199	0.441517
319944	Taf2	TATA-box binding protein associated factor 2	NA	0.10223	5.94727	1.073431	0.13563002	0.441748
22746	Zfp85	zinc finger protein 85	NA	0.150239	3.451682	1.109753	0.13565981	0.441756
67427	Rps20	ribosomal protein S20	NA	0.066494	7.890628	1.047169	0.13582985	0.442085
228876	Zfp334	zinc finger protein 334	NA	0.113089	5.879674	1.081541	0.13585778	0.442085
666173	Vps13b	vacuolar protein sorting 13B	NA	0.113355	4.92403	1.081741	0.1358584	0.442085
67629	Spc24	SPC24, NDC80 kinetochore complex component, ho	NA	-0.24042	3.074991	-1.18133	0.13586935	0.442085
112403	Dxo	decapping exoribonuclease, transcript variant 2	NA	-0.12435	4.568965	-1.09002	0.13608023	0.44261
100504166	4933421O10Rik	RIKEN cDNA 4933421O10 gene	NA	0.190862	2.897299	1.141445	0.13608515	0.44261
433940	Fam222a	family with sequence similarity 222, member A	NA	-0.14433	4.002023	-1.10522	0.13615126	0.442718
210376	Mtmr9	myotubularin related protein 9, transcript variant X1	NA	0.08268	7.137337	1.058983	0.13617285	0.442718
118567918	LOC118567918	MLV-related proviral Env polyprotein-like, transcript v	NA	0.183181	3.530497	1.135384	0.13623913	0.442845
68073	Atpsckmt	ATP synthase C subunit lysine N-methyltransferase,	NA	-0.19227	4.239261	-1.14256	0.1362837	0.442902
16206	Lrig1	leucine-rich repeats and immunoglobulin-like domain	NA	0.121213	5.343953	1.087649	0.13633773	0.442957
18167	Npy2r	neuropeptide Y receptor Y2, transcript variant 1	NA	-0.3298	1.407224	-1.25684	0.13635534	0.442957
22724	Zbtb7b	zinc finger and BTB domain containing 7B, transcript	NA	-0.2944	2.00538	-1.22638	0.13641605	0.443066
54713	Fezf2	Fez family zinc finger 2	NA	-0.15166	5.944446	-1.11085	0.13663348	0.443524
118568335	LOC118568335	uncharacterized LOC118568335, transcript variant X	NA	0.541411	-0.4387	1.455396	0.13663749	0.443524
66069	Snupn	snurportin 1	NA	-0.13897	4.15135	-1.10111	0.13663876	0.443524
57258	Xpo4	exportin 4, transcript variant X1	NA	0.122594	4.864893	1.088691	0.13675895	0.443756
102635461	Gm32793	predicted gene, 32793, transcript variant X1	NA	0.60093	-0.4295	1.516694	0.1367647	0.443756
229474	Fhdc1	FH2 domain containing 1, transcript variant 2	NA	-0.17239	3.049138	-1.12693	0.13686865	0.443925
26951	Zw10	zw10 kinetochore protein	NA	-0.11867	4.826234	-1.08573	0.13695916	0.443925
100503572	Bbip1	BBSome interacting protein 1, transcript variant 2	NA	0.093503	5.835557	1.066957	0.13698245	0.443925
76561	Snx7	sorting nexin 7, transcript variant 1	NA	0.199836	3.277848	1.148568	0.13699746	0.443925
15558	Htr2a	5-hydroxytryptamine (serotonin) receptor 2A	NA	0.656505	-0.00832	1.576259	0.13701013	0.443925
100503823	Gm16973	predicted gene, 16973, transcript variant 1	NA	0.130671	4.013967	1.094803	0.13712857	0.443925
75007	Mindy1	MINDY lysine 48 deubiquitinase 1, transcript variant	NA	-0.10711	5.204005	-1.07707	0.13715776	0.443925
70178	Abhd17c	abhydrolase domain containing 17C	NA	0.089126	6.599554	1.063725	0.13717202	0.443925
57432	Zc3h8	zinc finger CCCH type containing 8, transcript variant	NA	0.165646	3.442298	1.121669	0.13720236	0.443925
17927	Myod1	myogenic differentiation 1	NA	-0.58254	-0.71682	-1.49748	0.1372124	0.443925
15932	Idua	iduronidase, alpha-L, transcript variant 1	NA	-0.16492	3.416813	-1.12111	0.1372281	0.443925
231086	Hadhb	hydroxyacyl-CoA dehydrogenase trifunctional multier	NA	-0.09946	5.381101	-1.07137	0.1372532	0.443925
68047	Mpnd	MPN domain containing	NA	-0.07623	6.396389	-1.05426	0.13726312	0.443925
18007	Neo1	neogenin, transcript variant 1	NA	0.070236	7.880676	1.049888	0.13728106	0.443925
69882	Ints14	integrator complex subunit 14, transcript variant 2	NA	-0.11842	5.310459	-1.08555	0.13729832	0.443925

56442	Serinc1	serine incorporator 1	NA	0.075944	8.878387	1.05405	0.13735362	0.443925
66058	Tmem176a	transmembrane protein 176A, transcript variant 1	NA	0.209114	2.467434	1.155978	0.13736589	0.443925
69534	Avpi1	arginine vasopressin-induced 1	NA	0.313877	1.158656	1.243043	0.13737543	0.443925
214899	Kdm5a	lysine (K)-specific demethylase 5A	NA	0.087938	6.141328	1.06285	0.1373789	0.443925
67105	Timm21	translocase of inner mitochondrial membrane 21, trar	NA	-0.11333	4.539299	-1.08172	0.13738462	0.443925
27215	Azi2	5-azacytidine induced gene 2, transcript variant X9	NA	0.086153	6.164619	1.061536	0.13738969	0.443925
71746	Rgl3	ral guanine nucleotide dissociation stimulator-like 3	NA	-0.40004	0.656219	-1.31954	0.13741767	0.443927
84652	Fam126a	family with sequence similarity 126, member A, trans	NA	0.100473	6.445874	1.072125	0.13753483	0.444196
11993	Aup1	ancient ubiquitous protein 1, transcript variant 1	NA	-0.11586	5.376814	-1.08362	0.13755544	0.444196
75454	Phpt1	phosphohistidine phosphatase 1, transcript variant X	NA	0.150039	5.273068	1.1096	0.13759967	0.44425
26456	Sema4g	sema domain, immunoglobulin domain (Ig), transmen	NA	0.073935	7.478128	1.052584	0.13765714	0.444348
103963	Rpn1	ribophorin I	NA	-0.06924	7.318593	-1.04917	0.13774906	0.444514
67443	Map1lc3b	microtubule-associated protein 1 light chain 3 beta, tr	NA	0.066546	7.718374	1.047207	0.1378343	0.444514
233056	Zfp790	zinc finger protein 790, transcript variant 1	NA	-0.12237	5.060651	-1.08852	0.13784851	0.444514
19219	Ptger4	prostaglandin E receptor 4 (subtype EP4), transcript	NA	0.472914	0.385198	1.38791	0.13787665	0.444514
94064	Mrpl27	mitochondrial ribosomal protein L27	NA	0.115727	4.547266	1.083521	0.13790227	0.444514
20166	Rtkn	rhotekin, transcript variant 1	NA	-0.19253	3.408045	-1.14276	0.1379043	0.444514
194655	Klf11	Kruppel-like factor 11	NA	0.132777	4.880018	1.096402	0.13791059	0.444514
216725	Adamts2	a disintegrin-like and metallopeptidase (reprolysin typ	NA	0.19977	3.16216	1.148515	0.13792718	0.444514
77891	Ube2s	ubiquitin-conjugating enzyme E2S	NA	-0.12624	7.21589	-1.09144	0.1379646	0.444547
170442	Bbox1	butyrobetaine (gamma), 2-oxoglutarate dioxygenase	NA	0.816395	-0.68215	1.761	0.13799263	0.444549
225266	Klhl14	kelch-like 14, transcript variant X1	NA	0.209944	2.461997	1.156644	0.13802004	0.444549
381045	Ccdc58	coiled-coil domain containing 58, transcript variant 1	NA	0.165381	3.595862	1.121462	0.13810443	0.444727
16950	Loxl3	lysyl oxidase-like 3	NA	-0.24408	2.95569	-1.18434	0.13813005	0.444727
68970	Dcaf12	DDB1 and CUL4 associated factor 12, transcript varia	NA	-0.07265	6.638188	-1.05164	0.13817315	0.444778
115487775	Gm51898	predicted gene, 51898, transcript variant X2	NA	-0.44604	0.770796	-1.3623	0.13820938	0.444807
56488	Nxt1	NTF2-related export protein 1, transcript variant 2	NA	-0.18437	3.664025	-1.13632	0.13828962	0.444902
68865	Arv1	ARV1 homolog, fatty acid homeostasis modulator, tra	NA	-0.09667	5.01703	-1.0693	0.13829377	0.444902
57370	B4galt3	UDP-Gal:betaGlcNAc beta 1,4-galactosyltransferase,	NA	-0.09059	5.880815	-1.0648	0.13852146	0.445489
69309	Slc16a13	solute carrier family 16 (monocarboxylic acid transpo	NA	-0.14417	3.627164	-1.1051	0.13854025	0.445489
81018	Rnf114	ring finger protein 114, transcript variant 3	NA	-0.08552	6.276856	-1.06107	0.13858211	0.445489
53870	Cntn6	contactin 6	NA	0.13646	4.322926	1.099205	0.13859108	0.445489
118568634	LOC118568634	igE-binding protein-like	NA	-0.36771	2.285668	-1.2903	0.1386492	0.445489
140500	Acap3	ArfGAP with coiled-coil, ankyrin repeat and PH doma	NA	-0.08748	6.645921	-1.06252	0.13866554	0.445489
20193	S100a1	S100 calcium binding protein A1	NA	0.326045	1.674979	1.253572	0.13866768	0.445489
12585	Cdr2	cerebellar degeneration-related 2, transcript variant 1	NA	-0.12117	5.116205	-1.08762	0.13877297	0.445639
27411	Slc14a2	solute carrier family 14 (urea transporter), member 2,	NA	0.171228	3.103933	1.126016	0.13878843	0.445639
408064	BC064078	cDNA sequence BC064078	NA	0.481288	0.518894	1.395989	0.13883325	0.445639
21762	Psmd2	proteasome (prosome, macropain) 26S subunit, non-	NA	-0.06977	8.055817	-1.04955	0.13884497	0.445639
22130	Ttf1	transcription termination factor, RNA polymerase I	NA	-0.14979	4.033205	-1.10941	0.13887534	0.445639
68127	B230217C12Rik	RIKEN cDNA B230217C12 gene, transcript variant 7	NA	-0.16522	3.591519	-1.12134	0.13890132	0.445639
20916	Sucla2	succinate-Coenzyme A ligase, ADP-forming, beta sul	NA	0.082193	6.34928	1.058626	0.13890608	0.445639
384261	Gm5296	predicted gene 5296	NA	0.189549	2.824232	1.140407	0.1389681	0.44575
19820	Rlim	ring finger protein, LIM domain interacting, transcript	NA	0.077536	6.52439	1.055214	0.1390263	0.445827
56299	Fkbpl	FK506 binding protein-like	NA	0.224052	2.647135	1.168009	0.139047	0.445827
330502	Zfp82	zinc finger protein 82, transcript variant 1	NA	-0.15702	3.877161	-1.11498	0.13913222	0.44595
12563	Cdh6	cadherin 6	NA	0.135244	5.674084	1.098278	0.13913999	0.44595
19011	Endou	endonuclease, polyU-specific, transcript variant X3	NA	-0.71847	-0.3741	-1.64544	0.13920322	0.445978
66230	Mrps28	mitochondrial ribosomal protein S28	NA	0.211955	2.658296	1.158257	0.13922098	0.445978
233865	Katnip	katanin interacting protein, transcript variant 2	NA	-0.15309	4.45377	-1.11194	0.1392587	0.445978
230673	Ipo13	importin 13	NA	-0.08955	5.975518	-1.06404	0.13927086	0.445978
67630	Samd8	sterile alpha motif domain containing 8, transcript var	NA	0.092757	6.352157	1.066406	0.13933897	0.445978
118567441	LOC118567441	uncharacterized LOC118567441	NA	-0.33183	0.853015	-1.25861	0.13933907	0.445978
234549	Heatr3	HEAT repeat containing 3	NA	0.09505	5.373763	1.068102	0.13934085	0.445978
100043600	Gm4544	predicted gene 4544	NA	-0.53338	-0.09407	-1.44731	0.1394289	0.446146
252973	Grhl2	grainyhead like transcription factor 2, transcript variar	NA	-0.54912	-0.5976	-1.46319	0.13944819	0.446146
20650	Sntb2	syntrophin, basic 2, transcript variant 2	NA	0.29177	1.813921	1.224141	0.13948767	0.446175
57259	Tob2	transducer of ERBB2, 2	NA	-0.13456	4.780269	-1.09776	0.13951189	0.446175
230837	Asap3	ArfGAP with SH3 domain, ankyrin repeat and PH dor	NA	-0.1722	3.363277	-1.12678	0.13974231	0.446759
75745	Rian	RNA imprinted and accumulated in nucleus	NA	0.077523	9.794399	1.055204	0.13975514	0.446759
13619	Phc1	polyhomeotic 1, transcript variant 2	NA	0.071999	7.175479	1.051172	0.13980027	0.446759
66255	Hsbp1l1	heat shock factor binding protein 1-like 1	NA	0.517091	-0.29194	1.431066	0.1398812	0.446759
216197	Ckap4	cytoskeleton-associated protein 4	NA	-0.07202	6.68238	-1.05119	0.13988558	0.446759
94332	Cadm3	cell adhesion molecule 3	NA	-0.07514	8.518307	-1.05346	0.13988778	0.446759
214579	Aldh5a1	aldhehyde dehydrogenase family 5, subfamily A1	NA	0.10464	5.816557	1.075226	0.13988799	0.446759
69190	Dym	dymeclin	NA	0.109335	5.318188	1.078731	0.13991419	0.446759
19122	Prnp	prion protein, transcript variant 1	NA	0.083952	7.413212	1.059918	0.14000779	0.44697
53376	Usp2	ubiquitin specific peptidase 2, transcript variant 2	NA	0.183108	3.014466	1.135327	0.14004328	0.446996

245684	Cnksr2	connector enhancer of kinase suppressor of Ras 2, tr	NA		0.118157	4.539908	1.085347	0.14019216	0.447383
109674	Ampd2	adenosine monophosphate deaminase 2, transcript v	NA		-0.08631	5.852651	-1.06165	0.14028691	0.447598
22782	Slc30a1	solute carrier family 30 (zinc transporter), member 1	NA		0.151907	4.862595	1.111037	0.14036774	0.447742
69895	Snhg8	small nucleolar RNA host gene 8	NA		0.165247	3.165306	1.121358	0.14038712	0.447742
67557	Larp6	La ribonucleoprotein domain family, member 6	NA		0.13164	4.189848	1.095539	0.14051725	0.447985
17475	Mpdz	multiple PDZ domain crumbs cell polarity complex co	NA		-0.09029	5.499535	-1.06459	0.14051849	0.447985
232337	Zfp637	zinc finger protein 637, transcript variant 4	NA		0.124001	5.446605	1.089753	0.14058094	0.448004
66912	Bzw2	basic leucine zipper and W2 domains 2	NA		0.078801	8.117327	1.05614	0.14060776	0.448004
217864	Rcor1	REST corepressor 1	NA		-0.11701	5.392693	-1.08448	0.14065911	0.448004
74152	Stra6l	STRA6-like, transcript variant 1	NA		0.304925	1.465347	1.235355	0.14073689	0.448004
16876	Lhx9	LIM homeobox protein 9, transcript variant 1	NA		0.117622	6.734952	1.084945	0.14076174	0.448004
382793	Mtx3	metaxin 3, transcript variant X9	NA		0.111901	5.521031	1.080651	0.14077116	0.448004
328977	Zfp532	zinc finger protein 532, transcript variant 2	NA		-0.06792	7.044711	-1.0482	0.14079683	0.448004
12909	Crcp	calcitonin gene-related peptide-receptor component p	NA		0.093656	5.277632	1.067071	0.14082281	0.448004
236573	Gbp9	guanylate-binding protein 9, transcript variant 1	NA		0.262102	1.836263	1.199224	0.14084289	0.448004
18518	Igbp1	immunoglobulin (CD79A) binding protein 1	NA		0.107924	5.366888	1.077676	0.14086743	0.448004
68544	Trir	telomerase RNA component interacting RNase, trans	NA		0.095464	6.544351	1.068409	0.14088871	0.448004
72654	Ccdc12	coiled-coil domain containing 12	NA		-0.10575	4.754272	-1.07605	0.14089778	0.448004
12261	C1qbp	complement component 1, q subcomponent binding p	NA		0.086744	6.317911	1.06197	0.14093817	0.448004
53858	Rwdd2b	RWD domain containing 2B	NA		0.152507	3.379244	1.111499	0.14096583	0.448004
234730	Fcsk	fucose kinase, transcript variant 1	NA		0.164059	3.300663	1.120435	0.14100653	0.448004
383766	Tldc2	TBC/LysM associated domain containing 2, transcript	NA		0.454474	0.563745	1.370283	0.14100948	0.448004
215789	Phactr2	phosphatase and actin regulator 2, transcript variant 1	NA		0.105216	4.674981	1.075656	0.14101496	0.448004
105247240	Gm42372	predicted gene, 42372	NA		-0.09777	5.815961	-1.07012	0.14102009	0.448004
15108	Hsd17b10	hydroxysteroid (17-beta) dehydrogenase 10	NA		-0.09997	5.261484	-1.07175	0.14111409	0.448216
208836	Fanci	Fanconi anemia, complementation group I, transcript	NA		-0.20471	2.554697	-1.15246	0.14120885	0.44835
272636	Esyt3	extended synaptotagmin-like protein 3, transcript vari	NA		0.369114	0.548957	1.291559	0.14126827	0.44835
229663	Csde1	cold shock domain containing E1, RNA binding, trans	NA		0.066425	8.837212	1.047119	0.14127132	0.44835
268747	Carmil3	capping protein regulator and myosin 1 linker 3, trans	NA		-0.08668	7.468387	-1.06192	0.14129054	0.44835
209773	Dennd2a	DENN/MADD domain containing 2A, transcript variar	NA		-0.09591	5.687657	-1.06874	0.14132704	0.44835
57916	Tnfrsf13b	tumor necrosis factor receptor superfamily, member 1	NA		0.573774	-0.41111	1.488412	0.1413438	0.44835
217588	Mbip	MAP3K12 binding inhibitory protein 1	NA		0.127692	4.468313	1.092544	0.14134921	0.44835
67166	Arl8b	ADP-ribosylation factor-like 8B	NA		0.079737	6.940258	1.056826	0.14145144	0.448471
72599	Pdia5	protein disulfide isomerase associated 5	NA		0.174303	3.148765	1.128419	0.14145993	0.448471
66377	Ndufc1	NADH:ubiquinone oxidoreductase subunit C1	NA		0.118403	4.53782	1.085533	0.14155418	0.448471
210622	Pamr1	peptidase domain containing associated with muscle	NA		0.206638	2.323204	1.153995	0.14156353	0.448471
15569	Elavl2	ELAV like RNA binding protein 1, transcript variant 4	NA		0.08706	7.782624	1.062204	0.1415788	0.448471
15160	Serpind1	serine (or cysteine) peptidase inhibitor, clade D, mem	NA		0.373468	1.088103	1.295463	0.14160016	0.448471
12153	Bmp1	bone morphogenetic protein 1, transcript variant 3	NA		0.078243	6.213138	1.055732	0.14160117	0.448471
17970	Ncf2	neutrophil cytosolic factor 2, transcript variant X1	NA		-0.2982	1.344418	-1.22961	0.14162986	0.448471
194590	Reps2	RALBP1 associated Eps domain containing protein 2	NA		0.110156	4.976243	1.079345	0.14163535	0.448471
20832	Ssr4	signal sequence receptor, delta, transcript variant 1	NA		0.108449	4.864765	1.078069	0.1417246	0.448522
22192	Ube2m	ubiquitin-conjugating enzyme E2M, transcript variant	NA		0.079613	7.074989	1.056735	0.14173215	0.448522
232187	Smyd5	SET and MYND domain containing 5	NA		-0.10484	5.574991	-1.07537	0.14173433	0.448522
225743	Rnf165	ring finger protein 165	NA		-0.09421	7.192816	-1.06748	0.14182182	0.448703
102636180	Gm33320	predicted gene, 33320, transcript variant X1	NA		-0.55674	0.209664	-1.47094	0.14185872	0.448703
272027	Tstd2	thiosulfate sulfurtransferase (rhodanese)-like domain	NA		-0.11389	5.118397	-1.08214	0.1419114	0.448703
237823	Pfas	phosphoribosylformylglycinamidine synthase (FGAR	NA		-0.09344	5.438396	-1.06691	0.14193303	0.448703
436062	Cibar2	CBY1 interacting BAR domain containing 2	NA		-0.46247	-0.09745	-1.3779	0.14195087	0.448703
68737	Angel1	angel homolog 1	NA		-0.15059	3.735546	-1.11002	0.14195688	0.448703
78558	Htra3	HtrA serine peptidase 3, transcript variant X4	NA		0.160814	3.659021	1.117917	0.14198771	0.448713
50776	Polg2	polymerase (DNA directed), gamma 2, accessory sub	NA		-0.20809	2.532427	-1.15516	0.14202196	0.448734
319195	Rpl17	ribosomal protein L17, transcript variant X4	NA		0.07213	9.038828	1.051267	0.14221886	0.449269
67452	Pnpla8	patatin-like phospholipase domain containing 8	NA		0.087034	6.834416	1.062184	0.14231522	0.44937
321022	Cdv3	carnitine deficiency-associated gene expressed in ve	NA		0.082133	7.48003	1.058582	0.14231988	0.44937
240058	Cpne5	copine V, transcript variant X1	NA		0.110073	5.129918	1.079283	0.14236656	0.44937
20821	Trim21	tripartite motif-containing 21, transcript variant 2	NA		0.366572	0.640645	1.289286	0.14237493	0.44937
75805	Nln	neurolysin (metallopeptidase M3 family), transcript va	NA		0.083061	6.129822	1.059263	0.14238883	0.44937
214779	Zfp879	zinc finger protein 879, transcript variant X1	NA		-0.24974	2.353657	-1.18899	0.14246827	0.449533
331474	Rtl5	retrotransposon Gag like 5	NA		-0.09439	5.804278	-1.06762	0.14253555	0.449659
110355	Grk2	G protein-coupled receptor kinase 2, transcript varian	NA		-0.08539	7.08156	-1.06097	0.14265806	0.449958
78232	Trappc6b	trafficking protein particle complex 6B, transcript varia	NA		0.101753	5.952808	1.073077	0.14278513	0.45018
319924	Apba1	amyloid beta (A4) precursor protein binding, family A,	NA		-0.0934	7.459564	-1.06688	0.14284914	0.45018
68479	Phf5a	PHD finger protein 5A	NA		-0.10959	5.464363	-1.07892	0.14286979	0.45018
242506	Frmd3	FERM domain containing 3, transcript variant 1	NA		0.12336	4.653313	1.089269	0.14287959	0.45018
75801	4930447C04Rik	RIKEN cDNA 4930447C04 gene, transcript variant X	NA		0.29785	1.93488	1.229311	0.14288031	0.45018
72012	1600020E01Rik	RIKEN cDNA 1600020E01 gene, transcript variant 1	NA		0.198724	3.609813	1.147682	0.14291792	0.45018
70829	Ccdc93	coiled-coil domain containing 93, transcript variant 1	NA		0.104333	5.200681	1.074997	0.14292209	0.45018

76959	Chmp5	charged multivesicular body protein 5	NA	-0.07426	6.512953	-1.05282	0.14297364	0.450255
74763	Naa60	N(alpha)-acetyltransferase 60, NatF catalytic subunit,	NA	-0.08127	6.376068	-1.05795	0.14312253	0.450564
407786	Taf9b	TATA-box binding protein associated factor 9B, trans	NA	0.130891	3.911071	1.09497	0.14312725	0.450564
65100	Zic5	zinc finger protein of the cerebellum 5	NA	0.236388	2.299938	1.178039	0.14317395	0.450624
114873	Dscaml1	DS cell adhesion molecule like 1	NA	-0.11822	5.791711	-1.0854	0.14322725	0.450638
19179	Psmc1	protease (prosome, macropain) 26S subunit, ATPase	NA	0.069395	7.267082	1.049276	0.14326189	0.450638
12496	Entpd2	ectonucleoside triphosphate diphosphohydrolase 2	NA	0.277759	1.703799	1.21231	0.14326788	0.450638
16353	Ipw	imprinted gene in the Prader-Willi syndrome region	NA	-0.28662	3.089858	-1.21978	0.1432892	0.450638
66213	Med7	mediator complex subunit 7, transcript variant X4	NA	0.112746	4.762199	1.081284	0.1433454	0.450709
13482	Dpp4	dipeptidylpeptidase 4, transcript variant 1	NA	-0.26734	1.863645	-1.20359	0.1433671	0.450709
15374	Jpt1	Jupiter microtubule associated homolog 1	NA	0.073705	8.954358	1.052416	0.14341541	0.450774
101351	Eogt	EGF domain-specific O-linked N-acetylglucosamine (	NA	0.111632	4.83841	1.08045	0.143554	0.451021
211914	Asap2	ArfGAP with SH3 domain, ankyrin repeat and PH dor	NA	0.078564	6.015606	1.055966	0.14357119	0.451021
433791	Zfp992	zinc finger protein 992	NA	0.3747	1.865966	1.29657	0.1435771	0.451021
224022	Slc7a4	solute carrier family 7 (cationic amino acid transporte	NA	-0.13489	4.059842	-1.09801	0.14368553	0.451274
668158	Ccdc85c	coiled-coil domain containing 85C	NA	-0.08004	6.414495	-1.05705	0.14382424	0.451623
24057	Sh3yl1	Sh3 domain YSC-like 1, transcript variant 1	NA	0.127475	5.024068	1.09238	0.14387189	0.451685
13723	Emb	embigin	NA	0.167871	3.486718	1.123399	0.14395468	0.451857
217946	Cdca7l	cell division cycle associated 7 like	NA	-0.1981	2.756733	-1.14719	0.14398209	0.451857
54132	Pdlim1	PDZ and LIM domain 1 (elfin)	NA	-0.24209	2.03157	-1.1827	0.14408623	0.452069
17826	Fam89b	family with sequence similarity 89, member B, transcı	NA	0.101657	5.919125	1.073005	0.14415756	0.452069
68095	Ociad1	OCIA domain containing 1, transcript variant X9	NA	0.073635	7.581841	1.052365	0.14416006	0.452069
21954	Tnni3	troponin I, cardiac 3, transcript variant X1	NA	0.412538	1.185458	1.331025	0.14417422	0.452069
16874	Lhx6	LIM homeobox protein 6, transcript variant 4	NA	-0.11215	5.84504	-1.08084	0.14420685	0.452069
53325	Banp	BTG3 associated nuclear protein, transcript variant 2	NA	-0.16221	5.063285	-1.119	0.14425378	0.452069
109136	Mmaa	methylmalonic aciduria (cobalamin deficiency) type A	NA	-0.12061	4.83367	-1.0872	0.14426846	0.452069
383787	Ankrd63	ankyrin repeat domain 63	NA	0.434878	0.430649	1.351796	0.14427749	0.452069
66357	Ostc	oligosaccharyltransferase complex subunit (non-catal	NA	-0.08323	6.023822	-1.05939	0.14431725	0.452069
53379	Hnrnpa2b1	heterogeneous nuclear ribonucleoprotein A2/B1, tran	NA	-0.08445	10.46471	-1.06028	0.1443446	0.452069
56389	Stx5a	syntaxin 5A, transcript variant 2	NA	-0.08492	6.037597	-1.06063	0.14435528	0.452069
191578	Helq	helicase, POLQ-like, transcript variant X12	NA	0.131921	3.932598	1.095751	0.14438613	0.452078
13368	Dffb	DNA fragmentation factor, beta subunit	NA	0.161422	3.457399	1.118389	0.14444388	0.452129
22640	Zfp1	zinc finger protein 1, transcript variant 1	NA	0.110755	4.872054	1.079794	0.14447809	0.452129
105246894	Gm11753	predicted gene 11753	NA	0.469496	1.099344	1.384626	0.1445009	0.452129
78655	Eif3j1	eukaryotic translation initiation factor 3, subunit J1	NA	0.088353	6.152235	1.063156	0.14452713	0.452129
270049	Galntl6	UDP-N-acetyl-alpha-D-galactosamine:polypeptide N-	NA	0.140988	3.951663	1.10266	0.14454125	0.452129
218756	Slc4a7	solute carrier family 4, sodium bicarbonate cotranspo	NA	0.119469	6.029961	1.086335	0.14458856	0.452136
68955	Srrm4	serine/arginine repetitive matrix 4	NA	-0.07939	7.15909	-1.05657	0.14459889	0.452136
217837	Itpk1	inositol 1,3,4-triphosphate 5/6 kinase	NA	-0.10045	5.793405	-1.07211	0.14462845	0.452141
18799	Plcd1	phospholipase C, delta 1, transcript variant 2	NA	-0.16098	3.787489	-1.11805	0.14480753	0.452603
225160	Thoc1	THO complex 1, transcript variant X1	NA	0.090408	6.024125	1.064671	0.14487166	0.452603
12286	Cacna1a	calcium channel, voltage-dependent, P/Q type, alpha	NA	0.090305	5.872223	1.064596	0.14487501	0.452603
237175	Adgrg2	adhesion G protein-coupled receptor G2, transcript va	NA	0.240408	2.406236	1.181327	0.14491322	0.452603
75209	Sv2c	synaptic vesicle glycoprotein 2c, transcript variant X2	NA	0.178236	5.194109	1.131499	0.14491538	0.452603
13191	Dctn1	dynactin 1, transcript variant X6	NA	-0.07203	7.897398	-1.05119	0.14504614	0.45277
94219	Cnnm2	cyclin M2, transcript variant 1	NA	-0.17192	3.409117	-1.12656	0.1450572	0.45277
28028	Mrpl50	mitochondrial ribosomal protein L50	NA	0.081924	5.95518	1.058429	0.14507306	0.45277
13434	Trdmt1	tRNA aspartic acid methyltransferase 1, transcript vaı	NA	0.27796	3.629838	1.212479	0.14508	0.45277
11842	Arf3	ADP-ribosylation factor 3, transcript variant 1	NA	-0.07846	7.99594	-1.05589	0.14512772	0.452832
110809	Srsf1	serine and arginine-rich splicing factor 1, transcript va	NA	-0.08343	8.959894	-1.05954	0.14518233	0.452915
21357	Tarbp2	TARBP2, RISC loading complex RNA binding subuni	NA	0.121679	5.186558	1.088	0.14521889	0.452943
105787	Prkaa1	protein kinase, AMP-activated, alpha 1 catalytic subu	NA	0.110895	5.176256	1.079898	0.1452704	0.452984
16450	Jag2	jagged 2, transcript variant X1	NA	-0.08272	6.075892	-1.05901	0.1452912	0.452984
72661	Serp2	stress-associated endoplasmic reticulum protein fami	NA	0.107935	5.091368	1.077685	0.14531577	0.452984
271564	Vps13a	vacuolar protein sorting 13A	NA	-0.12681	4.340215	-1.09188	0.14546682	0.453363
224111	Ubxn7	UBX domain protein 7	NA	0.083156	7.065549	1.059333	0.14549296	0.453363
242662	Rims3	regulating synaptic membrane exocytosis 3, transcrip	NA	0.101805	6.91209	1.073115	0.14557007	0.453515
54375	Azin1	antizyme inhibitor 1, transcript variant 2	NA	0.087022	7.981852	1.062175	0.14559756	0.453515
195018	Zzef1	zinc finger, ZZ-type with EF hand domain 1, transcrip	NA	0.095728	5.820046	1.068605	0.14567169	0.453598
13179	Dcn	decorin, transcript variant 1	NA	-0.12938	5.603406	-1.09382	0.14567987	0.453598
235574	Atp2c1	ATPase, Ca++-sequestering, transcript variant X2	NA	0.094774	6.958245	1.067898	0.1457926	0.453722
16842	Lef1	lymphoid enhancer binding factor 1, transcript variant	NA	0.109855	5.130635	1.07912	0.14579649	0.453722
22044	Trh	thyrotropin releasing hormone	NA	0.341721	1.312259	1.267267	0.14582088	0.453722
18035	Nfkbia	nuclear factor of kappa light polypeptide gene enhanc	NA	0.121408	4.279092	1.087796	0.14585417	0.453722
52830	Pnrc2	proline-rich nuclear receptor coactivator 2	NA	-0.07792	6.431332	-1.0555	0.14585928	0.453722
117599	Helb	helicase (DNA) B	NA	0.220198	2.636208	1.164894	0.14590464	0.453777
71310	Tbc1d9	TBC1 domain family, member 9, transcript variant X1	NA	0.071857	7.1682	1.051069	0.1460132	0.454016
212377	Mms22l	MMS22-like, DNA repair protein, transcript variant 1	NA	-0.19015	3.435092	-1.14089	0.14609027	0.454016

105245359	4930470G03Rik	RIKEN cDNA 4930470G03 gene	NA	0.462934	0.173338	1.378342	0.14609157	0.454016
19047	Ppp1cc	protein phosphatase 1 catalytic subunit gamma, trans	NA	0.074752	8.307075	1.05318	0.14609321	0.454016
11982	Atp10a	ATPase, class V, type 10A, transcript variant X4	NA	-0.21799	2.374289	-1.16311	0.14617021	0.454094
68077	Nop53	NOP53 ribosome biogenesis factor	NA	0.078895	6.505025	1.056209	0.14618463	0.454094
16600	Klf4	Kruppel-like factor 4 (gut)	NA	-0.20283	3.334909	-1.15096	0.14622634	0.454094
22351	Vill	villin-like, transcript variant X4	NA	-0.3054	1.195161	-1.23576	0.14622979	0.454094
14312	Brd2	bromodomain containing 2, transcript variant X3	NA	-0.07752	8.260777	-1.0552	0.14627791	0.454148
70454	Cenpl	centromere protein L, transcript variant 1	NA	-0.18161	2.952162	-1.13415	0.14630307	0.454148
67179	Ccdc25	coiled-coil domain containing 25	NA	0.095194	5.258182	1.068209	0.14642913	0.454453
212123	Dcaf15	DDB1 and CUL4 associated factor 15, transcript varia	NA	0.088767	5.979849	1.063461	0.14649385	0.454567
56525	Zfp235	zinc finger protein 235, transcript variant X1	NA	0.122923	4.251993	1.088939	0.1466	0.454809
14181	Fgfbp1	fibroblast growth factor binding protein 1, transcript va	NA	-0.27131	1.806798	-1.20691	0.14666695	0.45493
26405	Map3k2	mitogen-activated protein kinase kinase kinase 2, tran	NA	0.123786	5.496143	1.08959	0.14671014	0.45495
76457	Ccdc134	coiled-coil domain containing 134, transcript variant 3	NA	-0.18641	3.663844	-1.13793	0.14672919	0.45495
14977	Slc39a7	solute carrier family 39 (zinc transporter), member 7,	NA	-0.08057	6.77633	-1.05744	0.14680503	0.455058
73827	Tmem198b	transmembrane protein 198b, transcript variant 1	NA	-0.1258	4.211344	-1.09111	0.14681985	0.455058
110749	Chaf1b	chromatin assembly factor 1, subunit B (p60), transcr	NA	0.14463	4.160939	1.105447	0.14688194	0.455163
218639	Arl15	ADP-ribosylation factor-like 15	NA	0.080391	6.051014	1.057304	0.1469367	0.455246
666257	Zfp660	zinc finger protein 660, transcript variant 1	NA	-0.1669	3.859553	-1.12265	0.14702256	0.455426
213208	Il20rb	interleukin 20 receptor beta	NA	0.42852	0.902273	1.345852	0.14714044	0.455648
13134	Dach1	dachshund family transcription factor 1, transcript var	NA	0.118389	5.424219	1.085522	0.14715038	0.455648
13846	Ephb4	Eph receptor B4, transcript variant 2	NA	-0.10628	4.778905	-1.07645	0.14719623	0.455651
26403	Map3k11	mitogen-activated protein kinase kinase kinase 11, tra	NA	-0.11228	4.487334	-1.08093	0.14722287	0.455651
118567688	LOC118567688	uncharacterized LOC118567688, transcript variant X1	NA	-0.53502	-0.14508	-1.44896	0.14723527	0.455651
216119	Ybey	ybeY metallopeptidase	NA	-0.19123	2.877704	-1.14173	0.14734427	0.455901
17133	Maff	v-maf musculoaponeurotic fibrosarcoma oncogene fa	NA	0.223441	2.316812	1.167515	0.14745041	0.456143
99167	Ssx2ip	synovial sarcoma, X 2 interacting protein, transcript v	NA	0.096475	5.141443	1.069158	0.14751526	0.456242
69358	Lrrc51	leucine rich repeat containing 51, transcript variant 3	NA	-0.30041	1.357399	-1.23149	0.14754675	0.456242
18616	Peg3	paternally expressed 3, transcript variant X9	NA	0.102472	8.076017	1.073612	0.14756649	0.456242
22294	Uxt	ubiquitously expressed prefoldin like chaperone	NA	-0.26721	2.306094	-1.20348	0.14770377	0.45658
75202	Spaca6	sperm acrosome associated 6, transcript variant X21	NA	0.106591	5.971442	1.076681	0.14791267	0.457139
75767	Rab11fip1	RAB11 family interacting protein 1 (class I), transcript	NA	-0.28603	1.509865	-1.21928	0.1481147	0.457645
23942	Mta2	metastasis-associated gene family, member 2	NA	-0.08252	7.294088	-1.05886	0.1481559	0.457645
244631	Pskh1	protein serine kinase H1	NA	-0.13471	4.208542	-1.09787	0.14816093	0.457645
72313	Fryl	FRY like transcription coactivator, transcript variant X	NA	0.086462	6.357566	1.061763	0.14832391	0.457865
57813	Tk2	thymidine kinase 2, mitochondrial, transcript variant 1	NA	0.111029	5.32997	1.079998	0.14833452	0.457865
268958	Capn11	calpain 11, transcript variant X4	NA	-0.57807	1.521678	-1.49285	0.14834839	0.457865
78455	Helz	helicase with zinc finger domain, transcript variant 1	NA	-0.08735	6.132512	-1.06242	0.14836324	0.457865
66966	Trit1	tRNA isopentenyltransferase 1, transcript variant X3	NA	0.119016	4.687173	1.085994	0.1483856	0.457865
233011	Itpkc	inositol 1,4,5-trisphosphate 3-kinase C	NA	-0.1516	4.035795	-1.1108	0.14840096	0.457865
115488510	Gm52202	predicted gene, 52202	NA	-0.31102	1.729186	-1.24058	0.14865238	0.458554
433868	3110082J24Rik	RIKEN cDNA 3110082J24 gene	NA	-0.41222	0.3645	-1.33073	0.14870165	0.458619
66929	Asf1b	anti-silencing function 1B histone chaperone	NA	-0.22673	2.812957	-1.17018	0.14878036	0.458775
56370	Tagln3	transgelin 3	NA	-0.06998	7.260262	-1.0497	0.14882038	0.458811
71787	Trnau1ap	tRNA selenocysteine 1 associated protein 1	NA	-0.11444	4.785858	-1.08255	0.14888698	0.45893
21938	Tnfrsf1b	tumor necrosis factor receptor superfamily, member 1	NA	0.276923	2.218599	1.211608	0.14893124	0.458956
76192	Abhd12	abhydrolase domain containing 12, transcript variant	NA	0.092987	6.170327	1.066576	0.14896805	0.458956
78309	Cul9	cullin 9	NA	-0.10187	5.452365	-1.07316	0.14901347	0.458956
16548	Khk	ketohexokinase, transcript variant 4	NA	0.2933	1.407997	1.22544	0.14904364	0.458956
56459	Sae1	SUMO1 activating enzyme subunit 1, transcript varia	NA	-0.08358	7.140428	-1.05964	0.14905839	0.458956
109900	Asl	argininosuccinate lyase, transcript variant X1	NA	0.169446	3.959009	1.124626	0.14908594	0.458956
93712	Pcdhga4	protocadherin gamma subfamily A, 4	NA	0.110309	4.634956	1.079459	0.14909314	0.458956
75786	Ckap5	cytoskeleton associated protein 5, transcript variant 1	NA	0.068891	7.536604	1.04891	0.14913442	0.458997
11671	Aldh3a2	aldehyde dehydrogenase family 3, subfamily A2, tran	NA	0.094523	4.973913	1.067713	0.1492137	0.459124
15560	Htr2c	5-hydroxytryptamine (serotonin) receptor 2C, transcri	NA	0.187022	3.863213	1.138411	0.14923237	0.459124
108073	Grm7	glutamate receptor, metabotropic 7, transcript variant	NA	0.12275	4.735467	1.088809	0.14934045	0.459355
20869	Stk11	serine/threonine kinase 11, transcript variant 1	NA	-0.0816	6.510387	-1.05819	0.14936385	0.459355
81799	C1qtnf3	C1q and tumor necrosis factor related protein 3, trans	NA	-0.38943	1.080516	-1.30987	0.14951043	0.459588
108167562	Gm46060	predicted gene, 46060	NA	-0.30831	1.653211	-1.23826	0.14952874	0.459588
114249	Npnt	nephronectin, transcript variant 5	NA	0.1019	5.226947	1.073186	0.14954389	0.459588
106628	Trip10	thyroid hormone receptor interactor 10, transcript vari	NA	-0.22238	3.174747	-1.16665	0.14957898	0.459588
230972	Arhgef16	Rho guanine nucleotide exchange factor (GEF) 16	NA	0.447537	0.348686	1.36371	0.14958926	0.459588
11306	Abcb7	ATP-binding cassette, sub-family B (MDR/TAP), mem	NA	-0.09808	5.08176	-1.07035	0.14960911	0.459588
11886	Asah1	N-acylsphingosine amidohydrolase 1	NA	0.084537	5.768248	1.060347	0.14966457	0.459672
77552	Shisa4	shisa family member 4	NA	-0.11226	4.63772	-1.08092	0.14981803	0.460032
67774	Borcs5	BLOC-1 related complex subunit 5, transcript variant	NA	0.136948	4.126279	1.099577	0.14985562	0.460032
29863	Pde7b	phosphodiesterase 7B, transcript variant 1	NA	-0.13848	3.614949	-1.10075	0.14990863	0.460032
72826	Fam76b	family with sequence similarity 76, member B, transcr	NA	0.12656	5.9332	1.091688	0.14990903	0.460032

22088	Tsg101	tumor susceptibility gene 101, transcript variant 1	NA	0.069794	7.461971	1.049567	0.14992306	0.460032
107242	AI837181	expressed sequence AI837181, transcript variant 1	NA	0.09741	5.018716	1.069851	0.15003138	0.46023
211673	Arfgef1	ADP-ribosylation factor guanine nucleotide-exchange	NA	0.083427	6.392288	1.059532	0.15004427	0.46023
66307	Isoc1	isochorismatase domain containing 1	NA	0.126196	5.76152	1.091412	0.15010776	0.460338
232875	Zscan18	zinc finger and SCAN domain containing 18, transcrip	NA	0.105775	5.193895	1.076072	0.15025146	0.460632
16329	Inpp1	inositol polyphosphate-1-phosphatase, transcript vari	NA	0.125739	4.839843	1.091066	0.15028335	0.460632
69702	Ndufaf1	NADH:ubiquinone oxidoreductase complex assembly	NA	0.128198	4.0417	1.092928	0.15029772	0.460632
72930	Ppp2r2b	protein phosphatase 2, regulatory subunit B, beta, tra	NA	0.071105	7.735561	1.050521	0.15031676	0.460632
106672	AI413582	expressed sequence AI413582, transcript variant X3	NA	0.223399	2.870337	1.167481	0.15035721	0.460652
57342	Parva	parvin, alpha, transcript variant X1	NA	-0.08432	5.783226	-1.06019	0.15038013	0.460652
108160	Fam50a	family with sequence similarity 50, member A	NA	0.099684	5.176071	1.071539	0.15046755	0.460759
18983	Cnot7	CCR4-NOT transcription complex, subunit 7, transcri	NA	-0.07646	7.428459	-1.05443	0.15047271	0.460759
99480	Dnttip2	deoxynucleotidyltransferase, terminal, interacting pro	NA	0.101604	5.337303	1.072966	0.1505753	0.460759
14219	Ccn2	cellular communication network factor 2	NA	0.159367	3.144353	1.116797	0.15062202	0.460759
73945	Otud4	OTU domain containing 4, transcript variant X1	NA	0.103529	6.207169	1.074399	0.15062775	0.460759
67300	Cltc	clathrin, heavy polypeptide (Hc), transcript variant 2	NA	0.07808	8.635216	1.055612	0.15063311	0.460759
75172	Ccdc146	coiled-coil domain containing 146	NA	-0.41214	0.234878	-1.33066	0.15063946	0.460759
93790	Nipa2	non imprinted in Prader-Willi/Angelman syndrome 2 h	NA	0.110256	5.279577	1.07942	0.15064147	0.460759
13639	Efna4	ephrin A4	NA	0.160746	3.212836	1.117865	0.15067288	0.460768
67512	Agpat2	1-acylglycerol-3-phosphate O-acyltransferase 2 (lyso	NA	0.283298	1.20919	1.216974	0.15081464	0.461115
320933	D230017M19Rik	RIKEN cDNA D230017M19 gene	NA	-0.40612	0.379314	-1.32512	0.15087456	0.461212
230233	Elp1	elongator complex protein 1	NA	0.079642	6.789586	1.056756	0.15093115	0.461233
67151	Psmd9	proteasome (prosome, macropain) 26S subunit, non-	NA	0.109774	4.996662	1.079059	0.15093826	0.461233
54721	Tyk2	tyrosine kinase 2, transcript variant X6	NA	-0.13339	4.263124	-1.09687	0.1509879	0.461298
17313	Mgp	matrix Gla protein	NA	0.261956	1.651516	1.199103	0.15105789	0.461424
68724	Arl8a	ADP-ribosylation factor-like 8A	NA	0.071494	8.439521	1.050804	0.15108587	0.461424
233107	Kctd15	potassium channel tetramerisation domain containing	NA	-0.07696	6.396734	-1.05479	0.15112936	0.461464
22025	Nr2c1	nuclear receptor subfamily 2, group C, member 1	NA	-0.11835	4.201667	-1.0855	0.15118448	0.461464
217692	Sipa1l1	signal-induced proliferation-associated 1 like 1, trans	NA	-0.08367	5.979493	-1.05971	0.15121196	0.461464
13024	Ctla2a	cytotoxic T lymphocyte-associated protein 2 alpha, tr	NA	0.243959	2.281709	1.184238	0.15121233	0.461464
66366	Ergic3	ERGIC and golgi 3, transcript variant 2	NA	0.074759	6.848862	1.053185	0.15137176	0.461864
51885	Tubgcp4	tubulin, gamma complex associated protein 4, transc	NA	-0.12532	5.154006	-1.09075	0.15144299	0.461995
213948	Atg9b	autophagy related 9B, transcript variant X1	NA	0.402295	1.355406	1.321609	0.15149891	0.462079
76898	B3gat1	beta-1,3-glucuronyltransferase 1 (glucuronosyltransfe	NA	-0.07451	8.4564	-1.053	0.15165036	0.462454
56786	Tmem9b	TMEM9 domain family, member B	NA	0.084938	5.610628	1.060642	0.15169147	0.462493
231798	Lrch4	leucine-rich repeats and calponin homology (CH) dor	NA	-1.06222	2.385607	-2.08814	0.1517797	0.4626
12667	Chrd	chordin, transcript variant X14	NA	0.14542	3.919942	1.106053	0.15178982	0.4626
66855	Tcf25	transcription factor 25 (basic helix-loop-helix), transcr	NA	0.06692	8.532185	1.047478	0.15181184	0.4626
53320	Folh1	folate hydrolase 1, transcript variant X4	NA	0.518365	-0.07031	1.432331	0.15195349	0.462945
115488170	Rpl27a-ps4	ribosomal protein L27A, pseudogene 4	NA	0.081505	5.816247	1.058121	0.152005	0.462994
20666	Sox11	SRY (sex determining region Y)-box 11	NA	-0.08826	10.67816	-1.06309	0.15205088	0.462994
99237	Tm9sf4	transmembrane 9 superfamily protein member 4	NA	-0.08191	6.368982	-1.05842	0.15205498	0.462994
380916	Lrch1	leucine-rich repeats and calponin homology (CH) dor	NA	0.117645	4.721819	1.084963	0.15208798	0.463008
106025	Sharpin	SHANK-associated RH domain interacting protein	NA	0.109304	4.865771	1.078708	0.15226001	0.463445
13867	Erbb3	erb-b2 receptor tyrosine kinase 3	NA	-0.27756	2.100491	-1.21215	0.15235898	0.463659
241989	Pabpc4l	poly(A) binding protein, cytoplasmic 4-like, transcript	NA	-0.26149	1.451333	-1.19872	0.15239988	0.463697
71929	Tmem123	transmembrane protein 123, transcript variant X1	NA	-0.12046	4.958764	-1.08708	0.15248814	0.463879
19763	Ring1	ring finger protein 1	NA	-0.10602	4.506613	-1.07625	0.15263217	0.46423
448850	Znhit3	zinc finger, HIT type 3, transcript variant 1	NA	0.14172	3.654589	1.10322	0.15269676	0.464333
115486940	Gm51579	predicted gene, 51579	NA	0.57989	-0.044	1.494736	0.15272907	0.464333
54381	Cpq	carboxypeptidase Q, transcript variant 2	NA	0.170807	3.054509	1.125688	0.15281811	0.464333
66513	Tab1	TGF-beta activated kinase 1/MAP3K7 binding protein	NA	-0.09501	5.040327	-1.06807	0.15285149	0.464333
238331	Zdhhc22	zinc finger, DHHC-type containing 22, transcript varia	NA	0.173867	3.731258	1.128078	0.15285653	0.464333
107435	Hat1	histone aminotransferase 1	NA	-0.11868	5.000241	-1.08574	0.15291843	0.464333
236904	Klhl15	kelch-like 15, transcript variant X6	NA	0.144529	3.855728	1.10537	0.15294451	0.464333
11998	Avp	arginine vasopressin	NA	0.432896	0.435893	1.349941	0.15295044	0.464333
665931	Rplp2-ps1	ribosomal protein, large P2, pseudogene 1	NA	0.150659	3.789894	1.110076	0.15295291	0.464333
14087	Fanca	Fanconi anemia, complementation group A, transcrip	NA	-0.20483	2.846064	-1.15255	0.15296771	0.464333
76574	Mfsd2a	major facilitator superfamily domain containing 2A	NA	0.105178	4.782558	1.075627	0.15300214	0.464333
435684	Shf	Src homology 2 domain containing F, transcript varia	NA	0.101896	5.319371	1.073183	0.15300858	0.464333
12395	Runx1t1	RUNX1 translocation partner 1, transcript variant X9	NA	0.064242	7.890088	1.045536	0.15311714	0.464489
50883	Chek2	checkpoint kinase 2, transcript variant 1	NA	-0.19286	2.690041	-1.14303	0.15313022	0.464489
19417	Rasgrf1	RAS protein-specific guanine nucleotide-releasing fa	NA	0.173906	3.676526	1.128109	0.15314558	0.464489
69269	Scnm1	sodium channel modifier 1, transcript variant 1	NA	-0.13295	4.440885	-1.09653	0.15324652	0.464708
108168942	Gm46854	predicted gene, 46854	NA	-0.38044	0.880166	-1.30174	0.15327485	0.464708
56448	Cyp2d22	cytochrome P450, family 2, subfamily d, polypeptide	NA	0.318223	1.460745	1.246794	0.15339578	0.464988
99712	Cept1	choline/ethanolaminephosphotransferase 1, transcrip	NA	-0.09548	5.077822	-1.06842	0.15343254	0.465013
102635783	Gm11266	predicted gene 11266	NA	-0.1883	4.017523	-1.13942	0.15348793	0.465094

22717	Zfp59	zinc finger protein 59	NA	0.138983	4.109067	1.101128	0.15358309	0.465296
102633432	Gm31262	predicted gene, 31262	NA	-0.31668	2.166497	-1.24546	0.15379652	0.465856
66425	Pcp4l1	Purkinje cell protein 4-like 1	NA	0.170745	3.524948	1.12564	0.15386059	0.46591
17930	Myom2	myomesin 2	NA	-0.59829	0.34791	-1.51392	0.1538717	0.46591
27058	Srp9	signal recognition particle 9	NA	0.106209	5.856137	1.076396	0.15392442	0.46595
170758	Rac3	Rac family small GTPase 3	NA	0.086709	6.713933	1.061945	0.15394207	0.46595
28084	Vps25	vacuolar protein sorting 25, transcript variant 2	NA	0.092649	5.581555	1.066326	0.1539948	0.465962
241201	Cdh7	cadherin 7, type 2, transcript variant 3	NA	0.146287	4.030485	1.106717	0.15400354	0.465962
74322	Cxxc1	CXXC finger 1 (PHD domain), transcript variant 1	NA	-0.08521	5.985301	-1.06084	0.15407645	0.466096
23881	G3bp2	GTPase activating protein (SH3 domain) binding prot	NA	0.062704	8.493032	1.044421	0.15415754	0.466144
66691	Gapvd1	GTPase activating protein and VPS9 domains 1, tran	NA	0.071041	6.981114	1.050474	0.15418001	0.466144
107817	Jmjd6	jumonji domain containing 6, transcript variant 2	NA	-0.08827	5.492466	-1.06309	0.15419238	0.466144
71685	Galnt14	polypeptide N-acetylgalactosaminyltransferase 14	NA	0.123162	3.918615	1.08912	0.15421136	0.466144
252903	Ap1s3	adaptor-related protein complex AP-1, sigma 3	NA	0.264825	1.403951	1.20149	0.15423551	0.466144
67287	Parp6	poly (ADP-ribose) polymerase family, member 6, tran	NA	-0.06565	7.639636	-1.04655	0.15430305	0.466262
75778	Them4	thioesterase superfamily member 4	NA	0.10353	5.006633	1.074399	0.15439561	0.466369
234825	Klhdc4	kelch domain containing 4	NA	-0.09215	5.229848	-1.06595	0.15439593	0.466369
14583	Gfpt1	glutamine fructose-6-phosphate transaminase 1	NA	0.076211	7.059344	1.054245	0.15449949	0.466595
102633424	Gm31255	predicted gene, 31255	NA	0.152014	3.532416	1.111119	0.1546018	0.466768
69188	Kmt2e	lysine (K)-specific methyltransferase 2E	NA	0.084656	8.080773	1.060435	0.15461408	0.466768
56048	Lgals8	lectin, galactose binding, soluble 8, transcript variant	NA	0.125384	4.505499	1.090798	0.15473301	0.467041
16576	Kif7	kinesin family member 7, transcript variant X6	NA	-0.14801	3.292456	-1.10804	0.15483581	0.467264
233726	Ipo7	importin 7, transcript variant 1	NA	-0.08731	7.417019	-1.06239	0.15490142	0.467375
668218	Bin2	bridging integrator 2, transcript variant 1	NA	-0.47691	0.203742	-1.39176	0.15493087	0.467378
14270	Srgap2	SLIT-ROBO Rho GTPase activating protein 2	NA	0.076251	7.412379	1.054275	0.15502904	0.467567
77782	Polq	polymerase (DNA directed), theta, transcript variant 2	NA	-0.17993	3.171785	-1.13283	0.15506676	0.467567
66570	Cenpm	centromere protein M, transcript variant X3	NA	-0.25653	2.52832	-1.1946	0.15507979	0.467567
217700	Acot6	acyl-CoA thioesterase 6, transcript variant X1	NA	0.20434	2.378119	1.152159	0.15513004	0.46762
13449	Dok2	docking protein 2, transcript variant X1	NA	-0.29542	1.359194	-1.22724	0.15515659	0.46762
18201	Nsmaf	neutral sphingomyelinase (N-SMase) activation asso	NA	0.082544	5.47939	1.058884	0.15518356	0.46762
81500	Sil1	endoplasmic reticulum chaperone SIL1 homolog (S. c	NA	0.145002	3.625317	1.105732	0.15525947	0.467723
72022	Slc35f2	solute carrier family 35, member F2, transcript varian	NA	0.147826	3.745963	1.107899	0.1552752	0.467723
66556	Drap1	Dr1 associated protein 1 (negative cofactor 2 alpha),	NA	0.142859	6.550777	1.104091	0.15541218	0.468049
243771	Parp12	poly (ADP-ribose) polymerase family, member 12	NA	0.28864	2.111501	1.221488	0.15551663	0.468277
71960	Myh14	myosin, heavy polypeptide 14, transcript variant 3	NA	-0.23463	2.148641	-1.1766	0.15558127	0.468382
70373	Gpatch2l	G patch domain containing 2 like, transcript variant 2	NA	0.077386	5.874235	1.055105	0.15560931	0.468382
78798	Eml4	echinoderm microtubule associated protein like 4, tra	NA	0.068851	6.643158	1.048881	0.15569276	0.468442
67228	Dph7	diphthamine biosynethesis 7, transcript variant 1	NA	0.103571	4.55155	1.074429	0.15569402	0.468442
102631989	Gm30177	predicted gene, 30177	NA	-0.28181	1.757387	-1.21572	0.1557441	0.468442
11435	Chrna1	cholinergic receptor, nicotinic, alpha polypeptide 1 (m	NA	-0.62673	-0.45537	-1.54406	0.15577247	0.468442
12257	Tspo	translocator protein	NA	-0.40249	0.619967	-1.32179	0.15577305	0.468442
68553	Col6a4	collagen, type VI, alpha 4, transcript variant X1	NA	0.32269	1.242862	1.25066	0.1558711	0.468577
70753	6330415B21Rik	RIKEN cDNA 6330415B21 gene	NA	-0.17573	3.154723	-1.12954	0.15587543	0.468577
17068	Ly6d	lymphocyte antigen 6 complex, locus D	NA	-1.11417	-0.89441	-2.1647	0.15598507	0.46882
77428	9430083A17Rik	RIKEN cDNA 9430083A17 gene	NA	0.240352	1.85117	1.181281	0.15604788	0.468908
226419	Dyrk3	dual-specificity tyrosine-(Y)-phosphorylation regulate	NA	-0.12447	4.231059	-1.09011	0.15607224	0.468908
83701	Srrt	serrate RNA effector molecule homolog (Arabidopsis	NA	-0.08347	7.430244	-1.05956	0.15618967	0.469107
72135	Pygo1	pygopus 1	NA	0.078254	6.768996	1.05574	0.15619606	0.469107
13380	Dkk1	dickkopf WNT signaling pathway inhibitor 1	NA	-0.48838	-0.38611	-1.40287	0.15628669	0.469224
56710	Brinp1	bone morphogenic protein/retinoic acid inducible neu	NA	0.081161	6.021347	1.057869	0.15629269	0.469224
100042875	Gm12059	predicted gene 12059, transcript variant X1	NA	0.333359	0.82003	1.259944	0.15632942	0.469248
14976	Pfdn6	prefoldin subunit 6, transcript variant 1	NA	0.091729	5.111195	1.065647	0.15638947	0.469264
66869	Zfp869	zinc finger protein 869, transcript variant 2	NA	0.093672	5.185184	1.067083	0.1563926	0.469264
330301	Zfp786	zinc finger protein 786	NA	-0.16873	4.27954	-1.12407	0.15642209	0.469266
11429	Aco2	aconitase 2, mitochondrial	NA	-0.07444	8.121232	-1.05295	0.1564652	0.469309
74388	Dpp8	dipeptidylpeptidase 8, transcript variant X3	NA	0.075794	7.272807	1.053941	0.15658284	0.469573
17380	Mme	membrane metallo endopeptidase, transcript variant	NA	0.205535	3.096979	1.153114	0.15663792	0.469573
29811	Ndrg2	N-myc downstream regulated gene 2, transcript varia	NA	0.078514	7.039523	1.05593	0.15665011	0.469573
52615	Suz12	SUZ12 polycomb repressive complex 2 subunit, trans	NA	0.079009	6.319053	1.056292	0.15670313	0.469573
15563	Htr5a	5-hydroxytryptamine (serotonin) receptor 5A	NA	0.190925	2.894603	1.141496	0.1567048	0.469573
118568532	LOC118568532	uncharacterized LOC118568532	NA	0.320601	1.192071	1.248851	0.15674312	0.469573
18430	Oxtr	oxytocin receptor	NA	0.284276	1.646494	1.217799	0.15675507	0.469573
93685	Entpd7	ectonucleoside triphosphate diphosphohydrolase 7	NA	0.139406	3.790144	1.101451	0.15679001	0.469591
66928	Dmac1	distal membrane arm assembly complex 1	NA	-0.11891	4.557295	-1.08591	0.15686321	0.469613
67490	Ufl1	UFM1 specific ligase 1, transcript variant 1	NA	0.105425	5.05321	1.075811	0.15687829	0.469613
17537	Meis3	Meis homeobox 3, transcript variant X25	NA	0.086361	6.246073	1.061689	0.15688391	0.469613
27053	Asns	asparagine synthetase	NA	0.108628	5.659702	1.078203	0.15700897	0.469752
381853	Gipr	gastric inhibitory polypeptide receptor, transcript varia	NA	-0.13716	4.548076	-1.09974	0.15703518	0.469752

15416	Hoxb8	homeobox B8, transcript variant X3	NA	1.052217	2.388583	2.073714	0.1570454	0.469752
217039	Ggnbp2	gametogenetin binding protein 2, transcript variant X1	NA	0.076134	6.648275	1.054189	0.1570569	0.469752
21371	Tbca	tubulin cofactor A	NA	0.079488	6.142242	1.056643	0.15707463	0.469752
17913	Myo1c	myosin IC, transcript variant 3	NA	-0.10581	4.733521	-1.07609	0.15729715	0.470229
12727	Clcn4	chloride channel, voltage-sensitive 4, transcript variar	NA	0.07121	7.856389	1.050597	0.15731826	0.470229
12808	Cobl	cordon-bleu WH2 repeat, transcript variant 3	NA	0.092202	5.386375	1.065996	0.15734378	0.470229
654818	Smco3	single-pass membrane protein with coiled-coil domair	NA	0.39589	0.331799	1.315755	0.15739844	0.470229
67888	Tmem100	transmembrane protein 100, transcript variant X2	NA	0.193243	2.765674	1.143331	0.15741502	0.470229
210297	Lrch2	leucine-rich repeats and calponin homology (CH) dor	NA	0.094091	6.482338	1.067393	0.15742872	0.470229
11364	Acadm	acyl-Coenzyme A dehydrogenase, medium chain	NA	0.099271	5.200501	1.071232	0.15747641	0.470229
64657	Mrps10	mitochondrial ribosomal protein S10, transcript variar	NA	0.107901	4.665815	1.077659	0.15750128	0.470229
326618	Tpm4	tropomyosin 4	NA	-0.06808	7.35228	-1.04832	0.15755225	0.470229
14171	Fgf17	fibroblast growth factor 17, transcript variant X4	NA	-0.35333	0.878191	-1.27751	0.1575534	0.470229
118568343	LOC118568343	igE-binding protein-like	NA	-0.52686	0.743754	-1.44079	0.15756178	0.470229
99377	Sall4	spalt like transcription factor 4, transcript variant a	NA	0.399419	0.234188	1.318977	0.15758098	0.470229
99470	Magi3	membrane associated guanylate kinase, WW and PD	NA	0.094098	5.376009	1.067398	0.15766884	0.470371
12741	Cldn5	claudin 5	NA	0.117003	5.696848	1.08448	0.15770637	0.470371
17766	Nudt1	nudix (nucleoside diphosphate linked moiety X)-type	NA	-0.22089	2.732854	-1.16545	0.15775068	0.470371
70646	Naa30	N(alpha)-acetyltransferase 30, NatC catalytic subunit	NA	0.099322	6.107768	1.07127	0.15776848	0.470371
215693	Zmat1	zinc finger, matrin type 1	NA	-0.13079	3.882256	-1.09489	0.15778267	0.470371
14176	Fgf5	fibroblast growth factor 5, transcript variant 2	NA	0.465883	-0.32023	1.381162	0.15781638	0.470371
20708	Serpinb6b	serine (or cysteine) peptidase inhibitor, clade B, mem	NA	0.380034	1.04716	1.301372	0.15783722	0.470371
103978	Gpc5	glypican 5	NA	0.23295	2.440846	1.175235	0.15788831	0.470371
234695	Carmil2	capping protein regulator and myosin 1 linker 2	NA	0.12549	4.972678	1.090878	0.1578889	0.470371
67288	Srek1ip1	splicing regulatory glutamine/lysine-rich protein 1inter	NA	0.119896	4.147027	1.086657	0.15794973	0.470466
66296	Haus2	HAUS augmin-like complex, subunit 2, transcript varia	NA	0.119325	4.928042	1.086226	0.15805469	0.470693
58188	Vstm2b	V-set and transmembrane domain containing 2B	NA	0.098706	4.739128	1.070813	0.15808663	0.470702
67095	Trak1	trafficking protein, kinesin binding 1, transcript variant	NA	0.066848	7.316402	1.047426	0.15818063	0.470896
217031	Tada2a	transcriptional adaptor 2A	NA	0.102956	5.139808	1.073972	0.1584735	0.471408
75355	4930553P18Rik	RIKEN cDNA 4930553P18 gene	NA	-0.36906	0.526173	-1.29151	0.1584983	0.471408
67472	Mtfr1	mitochondrial fission regulator 1, transcript variant 2	NA	-0.10072	4.794814	-1.07231	0.15850804	0.471408
18642	Pfkm	phosphofructokinase, muscle, transcript variant 1	NA	-0.0852	7.130212	-1.06083	0.15852212	0.471408
18569	Pdcd4	programmed cell death 4, transcript variant 1	NA	-0.07281	6.366649	-1.05176	0.15852789	0.471408
109652	Acy1	aminoacylase 1, transcript variant 1	NA	-0.1732	3.452638	-1.12756	0.15852893	0.471408
20725	Serpinb8	serine (or cysteine) peptidase inhibitor, clade B, mem	NA	-0.53744	-0.34645	-1.45139	0.15855989	0.471408
70593	Evx1os	even skipped homeotic gene 1, opposite strand	NA	-0.38708	0.462489	-1.30775	0.15858458	0.471408
105246933	Gm42127	predicted gene, 42127	NA	-0.23135	2.123581	-1.17393	0.15868431	0.471618
252966	Cables2	CDK5 and Abl enzyme substrate 2, transcript variant	NA	0.079439	5.936553	1.056607	0.15880014	0.471805
76792	Spring1	SREBF pathway regulator in golgi 1	NA	-0.1178	4.509767	-1.08508	0.15881648	0.471805
27883	Tango2	transport and golgi organization 2, transcript variant X	NA	-0.10232	4.705563	-1.0735	0.15883402	0.471805
320466	A230103J11Rik	RIKEN cDNA A230103J11 gene, transcript variant 2	NA	0.349674	0.536795	1.274273	0.15891579	0.471962
67062	Slc25a53	solute carrier family 25, member 53, transcript variant	NA	0.15712	3.286991	1.115059	0.15894759	0.47197
22642	Zbtb17	zinc finger and BTB domain containing 17, transcript	NA	-0.09663	5.435864	-1.06927	0.15940004	0.472973
226499	Odr4	odr4 GPCR localization factor homolog, transcript var	NA	0.087942	5.211325	1.062853	0.15946271	0.472973
72393	Faim2	Fas apoptotic inhibitory molecule 2, transcript variant	NA	-0.12801	5.039994	-1.09278	0.15948003	0.472973
26407	Map3k4	mitogen-activated protein kinase kinase kinase 4, trai	NA	-0.08373	5.543458	-1.05975	0.15951383	0.472973
68070	Pdzd2	PDZ domain containing 2, transcript variant X17	NA	0.12889	4.714616	1.093452	0.15953112	0.472973
382019	Zfp882	zinc finger protein 882	NA	-0.13056	4.548811	-1.09472	0.15956743	0.472973
71242	Spata24	spermatogenesis associated 24, transcript variant 2	NA	0.259205	1.588628	1.196819	0.1595818	0.472973
217951	Tmem196	transmembrane protein 196, transcript variant 1	NA	0.179388	3.310351	1.132404	0.15959326	0.472973
20419	Shcbp1	Shc SH2-domain binding protein 1	NA	-0.20534	3.459302	-1.15296	0.15960058	0.472973
106489	Sft2d1	SFT2 domain containing 1, transcript variant 2	NA	0.123659	4.602605	1.089495	0.15962316	0.472973
223696	Tomm22	translocase of outer mitochondrial membrane 22	NA	0.07123	6.263777	1.050612	0.15962498	0.472973
67492	Zfand4	zinc finger, AN1-type domain 4, transcript variant 2	NA	0.197405	3.370803	1.146634	0.15963413	0.472973
68614	Letmd1	LETM1 domain containing 1, transcript variant 4	NA	-0.08299	5.869892	-1.05921	0.15974822	0.47317
106618	Wdr90	WD repeat domain 90	NA	-0.09976	4.799577	-1.07159	0.15977798	0.47317
19882	Mst1r	macrophage stimulating 1 receptor (c-met-related tyro	NA	-0.33442	1.535851	-1.26087	0.15979945	0.47317
71227	Daw1	dynein assembly factor with WDR repeat domains 1,	NA	-0.46091	0.590109	-1.37641	0.15981692	0.47317
243382	Ppm1k	protein phosphatase 1K (PP2C domain containing), tı	NA	-0.12631	4.226025	-1.0915	0.15984692	0.473172
76464	Knl1	kinetochore scaffold 1, transcript variant X2	NA	-0.1486	4.044178	-1.10849	0.15988716	0.473194
13849	Ephx1	epoxide hydrolase 1, microsomal, transcript variant 1	NA	0.196319	2.776416	1.145771	0.15991846	0.473194
18689	Phxr4	per-hexamer repeat gene 4	NA	-0.48907	-0.30628	-1.40354	0.15994151	0.473194
53599	Cd164	CD164 antigen	NA	-0.08087	6.398057	-1.05766	0.15999877	0.473278
243963	Zfp473	zinc finger protein 473, transcript variant 5	NA	-0.34216	0.924679	-1.26765	0.16009948	0.473489
16905	Lmna	lamin A, transcript variant 1	NA	0.147412	4.777867	1.107581	0.16033667	0.474105
224630	Bnip1	BCL2/adenovirus E1B interacting protein 1, transcript	NA	-0.11619	4.608667	-1.08387	0.16039024	0.474136
67225	Rnpc3	RNA-binding region (RNP1, RRM) containing 3, trans	NA	0.121808	5.32719	1.088098	0.16040561	0.474136
93687	Csnk1a1	casein kinase 1, alpha 1, transcript variant 4	NA	0.077721	8.643057	1.055349	0.1604927	0.474308

209032	Zc3hav1l	zinc finger CCCH-type, antiviral 1-like	NA	-0.08762	5.502956	-1.06262	0.16067115	0.474646
16497	Kcnab1	potassium voltage-gated channel, shaker-related sub	NA	0.151881	3.110601	1.111017	0.16068289	0.474646
12160	Bmp5	bone morphogenetic protein 5	NA	0.191257	2.654512	1.141758	0.16069483	0.474646
238333	Samd15	sterile alpha motif domain containing 15	NA	0.510655	0.039924	1.424697	0.16074	0.474694
12824	Col2a1	collagen, type II, alpha 1, transcript variant 2	NA	-0.11919	6.532142	-1.08612	0.16082976	0.474872
193280	C030037D09Rik	RIKEN cDNA C030037D09 gene	NA	-0.30217	1.474072	-1.233	0.16091519	0.475038
70546	Zdhhc2	zinc finger, DHHC domain containing 2, transcript var	NA	0.10062	5.912687	1.072234	0.16096239	0.475092
211770	Trib1	tribbles pseudokinase 1	NA	-0.11599	4.42284	-1.08372	0.16101757	0.475113
103733	Tubg1	tubulin, gamma 1	NA	-0.08699	5.624232	-1.06215	0.16104771	0.475113
242377	Pm20d2	peptidase M20 domain containing 2	NA	0.156396	3.380857	1.1145	0.16105731	0.475113
114642	Brdt	bromodomain, testis-specific, transcript variant X5	NA	0.177858	3.093793	1.131203	0.16112105	0.475215
19279	Ptprr	protein tyrosine phosphatase, receptor type, R, transc	NA	0.134659	4.060043	1.097834	0.16119805	0.475356
69227	Selenot	selenoprotein T	NA	0.072263	7.320554	1.051364	0.16124905	0.475401
21915	Dtymk	deoxythymidylate kinase, transcript variant X1	NA	0.159463	5.780505	1.116871	0.16127177	0.475401
53310	Dlg3	discs large MAGUK scaffold protein 3, transcript varia	NA	-0.07444	6.616133	-1.05295	0.16144673	0.475704
67963	Npc2	NPC intracellular cholesterol transporter 2	NA	-0.08114	6.516335	-1.05786	0.16150446	0.475704
234740	Tmem231	transmembrane protein 231, transcript variant 1	NA	-0.12646	4.286797	-1.09161	0.16152914	0.475704
24117	Wif1	Wnt inhibitory factor 1	NA	-0.22118	2.589143	-1.16569	0.1615524	0.475704
118568701	LOC118568701	uncharacterized LOC118568701	NA	-0.37472	0.903317	-1.29658	0.16155894	0.475704
68050	Akirin1	akirin 1	NA	0.081107	6.219227	1.057829	0.1615734	0.475704
260305	Nphp4	nephronophthisis 4 (juvenile) homolog (human), trans	NA	-0.14823	3.51203	-1.10821	0.16160704	0.475704
210998	Fam91a1	family with sequence similarity 91, member A1, transc	NA	0.115853	6.283642	1.083615	0.16160842	0.475704
101056029	Gm7206	predicted pseudogene 7206	NA	-0.39016	0.474948	-1.31054	0.16164514	0.475726
239134	Gucy1b2	guanylate cyclase 1, soluble, beta 2, transcript varian	NA	-0.30849	1.139911	-1.23841	0.16172007	0.475816
100526796	Cahm	colon adenocarcinoma hypermethylated RNA	NA	-0.31206	1.088557	-1.24148	0.16173406	0.475816
234135	Nsd3	nuclear receptor binding SET domain protein 3, trans	NA	0.074135	6.76274	1.05273	0.16191249	0.476255
18600	Padi2	peptidyl arginine deiminase, type II	NA	0.341321	1.377794	1.266916	0.16196967	0.476337
231642	Alkbh2	alkB homolog 2, alpha-ketoglutarate-dependent dioxy	NA	-0.29248	1.82842	-1.22474	0.16203891	0.476411
12977	Csf1	colony stimulating factor 1 (macrophage), transcript v	NA	-0.19284	3.823141	-1.14301	0.16205355	0.476411
76223	Agbl3	ATP/GTP binding protein-like 3, transcript variant 4	NA	0.21765	3.365433	1.162838	0.16209535	0.476448
118568621	LOC118568621	uncharacterized LOC118568621	NA	0.341109	0.591021	1.26673	0.16218539	0.476578
20190	Ryr1	ryanodine receptor 1, skeletal muscle, transcript varia	NA	-0.12876	4.469234	-1.09336	0.16219806	0.476578
67671	Rpl38	ribosomal protein L38, transcript variant X1	NA	0.134028	7.210101	1.097353	0.16229741	0.476735
665562	Rpl31-ps12	ribosomal protein L31, pseudogene 1 2	NA	0.18484	6.157	1.136691	0.16235482	0.476735
338366	Mia3	melanoma inhibitory activity 3, transcript variant X7	NA	0.071541	6.70304	1.050839	0.16236531	0.476735
15239	Hgs	HGF-regulated tyrosine kinase substrate, transcript v	NA	-0.07584	6.611328	-1.05398	0.16237181	0.476735
14391	Gabpb1	GA repeat binding protein, beta 1, transcript variant X	NA	-0.09454	5.651719	-1.06772	0.1623982	0.476735
66626	Cdip1	cell death inducing Trp53 target 1, transcript variant X	NA	0.074936	6.632008	1.053314	0.16256406	0.477095
30960	Vapa	vesicle-associated membrane protein, associated pro	NA	0.078536	7.345618	1.055946	0.16259732	0.477095
67052	Ndc80	NDC80 kinetochore complex component	NA	-0.16177	3.465293	-1.11866	0.1626259	0.477095
108086	Rnf216	ring finger protein 216, transcript variant X4	NA	-0.09877	5.52494	-1.07086	0.16263811	0.477095
102634078	LOC102634078	uncharacterized LOC102634078	NA	0.235471	3.033295	1.177291	0.16269944	0.477189
66148	Dnajc15	DnaJ heat shock protein family (Hsp40) member C15	NA	0.140671	3.677045	1.102418	0.16278405	0.477351
11444	Chrnb2	cholinergic receptor, nicotinic, beta polypeptide 2 (ne	NA	-0.07568	6.478432	-1.05386	0.16286132	0.477452
238252	Gpr135	G protein-coupled receptor 135	NA	0.167355	3.406247	1.122997	0.16287726	0.477452
170711	Otud7a	OTU domain containing 7A, transcript variant X2	NA	-0.12633	4.969893	-1.09152	0.16292001	0.477492
72475	Ssbp3	single-stranded DNA binding protein 3, transcript vari	NA	-0.07423	8.362596	-1.0528	0.16300515	0.477655
140709	Col26a1	collagen, type XXVI, alpha 1, transcript variant 2	NA	0.098554	5.344082	1.0707	0.16304159	0.477676
75124	Nxnl2	nucleoredoxin-like 2, transcript variant X1	NA	0.406954	0.207867	1.325883	0.16312014	0.47782
217216	Hrob	homologous recombination factor with OB-fold	NA	-0.26118	1.819705	-1.19846	0.16315531	0.477836
68074	A930013F10Rik	RIKEN cDNA A930013F10 gene	NA	0.119703	4.60107	1.086511	0.16318435	0.477836
100043232	3110099E03Rik	RIKEN cDNA 3110099E03 gene	NA	0.482223	-0.0461	1.396894	0.16324734	0.47792
19290	Pura	purine rich element binding protein A	NA	0.127385	5.628248	1.092312	0.16327165	0.47792
83964	Jam3	junction adhesion molecule 3	NA	-0.0987	5.041503	-1.0708	0.1633275	0.477993
11688	Alox8	arachidonate 8-lipoxygenase	NA	0.529447	-0.48373	1.443376	0.16335528	0.477993
19330	Rab18	RAB18, member RAS oncogene family, transcript var	NA	0.066464	7.349056	1.047147	0.16340245	0.478045
80707	Wwox	WW domain-containing oxidoreductase	NA	0.195333	3.484791	1.144988	0.16355861	0.478392
228765	Sdcbp2	syndecan binding protein (syntenin) 2, transcript varia	NA	0.491198	-0.11955	1.405611	0.16357991	0.478392
68278	Ddx39a	DEAD box helicase 39a, transcript variant 2	NA	-0.10845	5.367599	-1.07807	0.16361224	0.4784
56741	Igdcc4	immunoglobulin superfamily, DCC subclass, member	NA	0.084137	6.04852	1.060053	0.1637169	0.478573
56279	Dipk1b	divergent protein kinase domain 1B	NA	0.116365	5.36536	1.084	0.16373004	0.478573
72462	Rrp1b	ribosomal RNA processing 1B, transcript variant 2	NA	-0.09955	4.901152	-1.07144	0.16385099	0.47884
100037283	Rnaset2a	ribonuclease T2A, transcript variant 1	NA	-0.56186	3.182263	-1.47617	0.1638908	0.478871
74393	Map10	microtubule-associated protein 10	NA	0.16974	3.282455	1.124856	0.16396489	0.479001
17929	Myom1	myomesin 1, transcript variant X1	NA	-0.2069	2.120588	-1.15421	0.16401974	0.479075
268860	Abat	4-aminobutyrate aminotransferase, transcript variant	NA	0.084479	6.698718	1.060305	0.16408036	0.47911
12499	Entpd5	ectonucleoside triphosphate diphosphohydrolase 5, tr	NA	0.115916	4.393096	1.083663	0.16409052	0.47911
234267	Gpm6a	glycoprotein m6a, transcript variant 2	NA	0.066918	9.666638	1.047477	0.16414021	0.479169

16439	Itpr2	inositol 1,4,5-triphosphate receptor 2, transcript varia	NA	0.146918	3.417596	1.107201	0.16417608	0.479188
24086	Tlk2	tousled-like kinase 2 (Arabidopsis), transcript variant	NA	0.081256	6.321955	1.057939	0.16433809	0.4795
71994	Cnn3	calponin 3, acidic	NA	-0.06938	8.244636	-1.04927	0.16434203	0.4795
215113	Slc43a2	solute carrier family 43, member 2, transcript variant	NA	-0.12087	5.446188	-1.08739	0.16439878	0.47958
14688	Gnb1	guanine nucleotide binding protein (G protein), beta 1	NA	0.063102	10.01587	1.04471	0.16443544	0.479601
99512	Wdr47	WD repeat domain 47, transcript variant X7	NA	0.067488	7.259564	1.047891	0.16450278	0.479711
69162	Sec31a	Sec31 homolog A (S. cerevisiae), transcript variant 7	NA	-0.07091	6.944441	-1.05038	0.16462932	0.479776
68149	Otub2	OTU domain, ubiquitin aldehyde binding 2, transcript	NA	-0.11961	4.366291	-1.08644	0.16463319	0.479776
98733	Obsl1	obscurin-like 1, transcript variant X5	NA	-0.12804	5.108048	-1.09281	0.16466584	0.479776
73914	Irak3	interleukin-1 receptor-associated kinase 3, transcript	NA	0.522772	-0.48682	1.436713	0.16467206	0.479776
20833	Ssrp1	structure specific recognition protein 1, transcript vari	NA	-0.06945	7.512035	-1.04932	0.16467233	0.479776
118568050	LOC118568050	protein C1orf43 homolog	NA	0.600428	-0.333	1.516166	0.16481066	0.479994
17714	Grpel2	GrpE-like 2, mitochondrial	NA	0.080603	5.698804	1.05746	0.1648419	0.479994
215751	Ginm1	glycoprotein integral membrane 1, transcript variant 2	NA	0.106675	4.344986	1.076744	0.1648467	0.479994
18996	Pou4f1	POU domain, class 4, transcription factor 1	NA	0.125703	4.836625	1.091039	0.16486513	0.479994
66154	Tmem14c	transmembrane protein 14C, transcript variant 1	NA	-0.12429	4.925104	-1.08997	0.1649711	0.480216
18029	Nfic	nuclear factor I/C, transcript variant 1	NA	-0.09054	5.773011	-1.06477	0.16502722	0.480232
101809	Spred3	sprouty-related EVH1 domain containing 3	NA	-0.11181	5.630526	-1.08058	0.1650355	0.480232
229473	Tmem131l	transmembrane 131 like, transcript variant X4	NA	-0.09371	6.998284	-1.06711	0.16508259	0.480283
100504231	Gm15708	predicted gene 15708	NA	0.30819	1.115691	1.238153	0.16517034	0.480383
215707	Ccdc92	coiled-coil domain containing 92	NA	0.107149	5.016949	1.077097	0.16522713	0.480383
72162	Dhx36	DEAH (Asp-Glu-Ala-His) box polypeptide 36, transcri	NA	0.0848	7.17772	1.060541	0.16523275	0.480383
20840	Stac	src homology three (SH3) and cysteine rich domain, t	NA	0.221408	2.961543	1.165871	0.16523506	0.480383
69870	Polr3gl	polymerase (RNA) III (DNA directed) polypeptide G li	NA	0.092378	5.209101	1.066126	0.16531969	0.480488
229699	Slc16a4	solute carrier family 16 (monocarboxylic acid transpo	NA	-0.44824	0.399734	-1.36438	0.16533031	0.480488
20274	Scn9a	sodium channel, voltage-gated, type IX, alpha, transc	NA	0.13971	4.688893	1.101684	0.16541701	0.480602
54710	Hs3st3b1	heparan sulfate (glucosamine) 3-O-sulfotransferase 3	NA	-0.13165	3.781137	-1.09554	0.16542837	0.480602
12361	Cask	calcium/calmodulin-dependent serine protein kinase (	NA	0.065218	7.421722	1.046243	0.16545989	0.480608
18555	Cdk16	cyclin-dependent kinase 16, transcript variant 1	NA	-0.07247	7.679895	-1.05151	0.16554533	0.48077
14533	Bloc1s1	biogenesis of lysosomal organelles complex-1, subur	NA	0.096057	4.874161	1.068848	0.16558451	0.480774
115488789	LOC115488789	keratin-associated protein 9-1-like	NA	0.202866	2.393348	1.150983	0.16560902	0.480774
270672	Map3k15	mitogen-activated protein kinase kinase kinase 15	NA	0.338968	0.970551	1.264852	0.16563555	0.480774
67784	Plxnd1	plexin D1	NA	-0.08304	6.322539	-1.05925	0.16579766	0.481159
80898	Erap1	endoplasmic reticulum aminopeptidase 1	NA	0.225003	2.456756	1.16878	0.1658517	0.48123
27369	Dguok	deoxyguanosine kinase, transcript variant 1	NA	-0.1381	3.867108	-1.10045	0.16597945	0.481451
243846	Ccdc9	coiled-coil domain containing 9, transcript variant 2	NA	-0.107	5.425355	-1.07698	0.16604596	0.481451
26378	Decr2	2-4-dienoyl-Coenzyme A reductase 2, peroxisomal	NA	-0.1231	4.59324	-1.08908	0.16605265	0.481451
246738	Dnajc28	DnaJ heat shock protein family (Hsp40) member C28	NA	-0.17398	2.872624	-1.12817	0.16606297	0.481451
18738	Pitpna	phosphatidylinositol transfer protein, alpha	NA	0.064856	7.706732	1.045981	0.1660758	0.481451
97775	D930048N14Rik	RIKEN cDNA D930048N14 gene	NA	0.30786	1.352342	1.23787	0.1661055	0.481451
102216272	Ak6	adenylate kinase 6	NA	-0.1208	4.261122	-1.08734	0.16620885	0.481601
114715	Spred1	sprouty protein with EVH-1 domain 1, related sequen	NA	0.088382	6.631057	1.063177	0.16621625	0.481601
77113	Klhl2	kelch-like 2, Mayven, transcript variant X4	NA	0.072542	6.02636	1.051568	0.1663593	0.48193
223499	Dcaf13	DDB1 and CUL4 associated factor 13	NA	0.081117	5.495933	1.057837	0.16639604	0.481931
544696	Tbc1d32	TBC1 domain family, member 32	NA	0.140335	3.416498	1.102161	0.16643312	0.481931
54519	Apbb1ip	amyloid beta (A4) precursor protein-binding, family B	NA	0.278602	1.430316	1.213019	0.16644857	0.481931
319463	C230057M02Rik	RIKEN cDNA C230057M02 gene, transcript variant X	NA	-0.13653	3.757001	-1.09925	0.16651962	0.482051
99889	Arfip1	ADP-ribosylation factor interacting protein 1, transcrip	NA	0.094523	5.024902	1.067712	0.16662094	0.482258
28113	Tinf2	Terf1 (TRF1)-interacting nuclear factor 2	NA	0.124621	4.124697	1.090221	0.16674671	0.482536
77300	Raph1	Ras association (RalGDS/AF-6) and pleckstrin homol	NA	0.074	6.886964	1.052631	0.16688863	0.482789
320210	A230077H06Rik	RIKEN cDNA A230077H06 gene	NA	0.151784	3.398999	1.110942	0.1668932	0.482789
65020	Zfp110	zinc finger protein 110, transcript variant X2	NA	-0.11778	4.878191	-1.08507	0.16701695	0.483005
71306	Mfap3l	microfibrillar-associated protein 3-like, transcript varia	NA	0.174053	3.526716	1.128223	0.16709472	0.483005
15227	Foxf1	forkhead box F1	NA	0.36311	0.911379	1.286196	0.16709971	0.483005
68193	Rpl24	ribosomal protein L24	NA	0.098836	8.262127	1.070909	0.16712168	0.483005
68427	Slc39a13	solute carrier family 39 (metal ion transporter), memb	NA	-0.15999	4.304766	-1.11728	0.16718956	0.483005
76784	Mtif2	mitochondrial translational initiation factor 2, transcrip	NA	-0.0967	5.255035	-1.06933	0.16721928	0.483005
75646	Rai14	retinoic acid induced 14, transcript variant X1	NA	-0.09952	5.444594	-1.07142	0.16724579	0.483005
212547	Nepro	nucleolus and neural progenitor protein, transcript va	NA	0.135132	3.680054	1.098194	0.16727278	0.483005
21771	Utp4	UTP4 small subunit processome component, transcri	NA	-0.09342	5.043531	-1.0669	0.16730337	0.483005
73093	3110006O06Rik	RIKEN cDNA 3110006O06 gene	NA	-0.44503	0.184319	-1.36134	0.16731938	0.483005
102635047	Gm32483	predicted gene, 32483, transcript variant X2	NA	0.159509	3.468225	1.116907	0.1673595	0.483005
230757	5730409E04Rik	RIKEN cDNA 5730409E04Rik gene, transcript varian	NA	0.112802	5.268368	1.081326	0.16737388	0.483005
105243290	Gm39252	predicted gene, 39252, transcript variant X2	NA	-0.79207	-0.44387	-1.73156	0.16738006	0.483005
93683	Glce	glucuronyl C5-epimerase, transcript variant 2	NA	0.109619	4.98666	1.078944	0.16738343	0.483005
56700	Glmp	glycosylated lysosomal membrane protein	NA	-0.11357	4.497472	-1.0819	0.1674331	0.483062
17344	Pias2	protein inhibitor of activated STAT 2, transcript varian	NA	0.073519	6.743502	1.05228	0.16750831	0.483194
14456	Gas6	growth arrest specific 6	NA	-0.09709	5.380284	-1.06962	0.1675819	0.483224

11682	Alk	anaplastic lymphoma kinase, transcript variant X3	NA	0.207547	3.805964	1.154723	0.16760866	0.483224
320271	Scai	suppressor of cancer cell invasion	NA	0.122371	6.130412	1.088523	0.16762811	0.483224
76252	Atp6v0e2	ATPase, H+ transporting, lysosomal V0 subunit E2, tı	NA	0.071272	7.530424	1.050642	0.16763761	0.483224
20848	Stat3	signal transducer and activator of transcription 3, tran	NA	0.090954	5.4012	1.065074	0.16769028	0.48329
102633666	AV099323	expressed sequence AV099323, transcript variant X2	NA	-0.32194	1.721271	-1.25001	0.16781333	0.483559
213484	Nudt18	nudix (nucleoside diphosphate linked moiety X)-type	NA	0.148309	3.536306	1.108269	0.16786573	0.483624
71772	Plbd2	phospholipase B domain containing 2	NA	-0.09818	4.956267	-1.07042	0.16840158	0.484946
115488204	Gm45623	predicted gene 45623	NA	0.18873	2.470847	1.13976	0.16842872	0.484946
70683	Utp20	UTP20 small subunit processome component	NA	-0.11527	4.449759	-1.08318	0.16843812	0.484946
52480	Snhg14	small nucleolar RNA host gene 14	NA	0.20926	6.610905	1.156095	0.16852052	0.484946
269683	E130006D01Rik	RIKEN cDNA E130006D01 gene, transcript variant 1	NA	0.23245	2.029652	1.174829	0.16852548	0.484946
13006	Smc3	structural maintenance of chromosomes 3	NA	0.065386	7.076444	1.046365	0.16856552	0.484946
353342	Peg13	paternally expressed 13	NA	-0.06384	8.315366	-1.04524	0.1685818	0.484946
66140	Ska2	spindle and kinetochore associated complex subunit	NA	-0.10461	4.966042	-1.0752	0.16859263	0.484946
67621	Bend5	BEN domain containing 5, transcript variant 1	NA	0.094538	5.307771	1.067723	0.1685928	0.484946
76890	Memo1	mediator of cell motility 1	NA	0.077945	6.241867	1.055513	0.1686264	0.484957
595136	Ndufs5	NADH:ubiquinone oxidoreductase core subunit S5	NA	0.087251	5.609524	1.062344	0.16867647	0.485016
60532	Wtap	Wilms tumour 1-associating protein, transcript variant	NA	0.068715	7.128202	1.048782	0.16882152	0.485347
170762	Nup155	nucleoporin 155	NA	0.086918	5.681318	1.062099	0.16889809	0.485481
233813	Vwa3a	von Willebrand factor A domain containing 3A, transc	NA	0.2075	2.247002	1.154685	0.16895189	0.48555
54208	Arl6ip1	ADP-ribosylation factor-like 6 interacting protein 1	NA	-0.06225	7.980325	-1.04409	0.16901941	0.4856
14325	Ftl1	ferritin light polypeptide 1	NA	-0.07434	10.02016	-1.05288	0.16902884	0.4856
102639899	Gm36101	predicted gene, 36101, transcript variant X2	NA	-0.42491	0.385944	-1.34249	0.16907377	0.485643
115486536	Gm51491	predicted gene, 51491	NA	-0.31867	1.466285	-1.24718	0.16914024	0.48573
192986	Cyb5d2	cytochrome b5 domain containing 2	NA	0.197638	2.484454	1.14682	0.16916355	0.48573
329828	Myorg	myogenesis regulating glycosidase (putative)	NA	0.157806	3.862857	1.115589	0.16919953	0.485747
100534287	Dchs2	dachsous cadherin related 2, transcript variant X1	NA	0.154118	3.50179	1.112741	0.16937961	0.486178
72114	Zbed3	zinc finger, BED type containing 3, transcript variant 2	NA	-0.1028	5.426083	-1.07385	0.16957205	0.486428
791411	Gm13199	predicted gene 13199	NA	-0.4235	0.487061	-1.34118	0.1695965	0.486428
623474	Rad54b	RAD54 homolog B (S. cerevisiae), transcript variant ´	NA	-0.19747	2.564744	-1.14668	0.16960983	0.486428
21353	Tank	TRAF family member-associated Nf-kappa B activato	NA	0.117548	4.480385	1.084889	0.16961785	0.486428
70466	Ckap2l	cytoskeleton associated protein 2-like	NA	-0.13942	4.585939	-1.10146	0.16963482	0.486428
654470	Tctn1	tectonic family member 1, transcript variant 1	NA	-0.09738	4.867509	-1.06983	0.16964586	0.486428
71279	Slc29a3	solute carrier family 29 (nucleoside transporters), mei	NA	-0.11265	4.394477	-1.08121	0.16968995	0.48645
17175	Masp2	mannan-binding lectin serine peptidase 2, transcript \	NA	-0.61733	-0.25676	-1.53404	0.16971706	0.48645
319594	Hif1an	hypoxia-inducible factor 1, alpha subunit inhibitor	NA	-0.09245	5.730406	-1.06618	0.1697432	0.48645
666427	Gm8098	predicted gene 8098, transcript variant 3	NA	0.403117	0.700956	1.322362	0.16978043	0.486471
102633593	AA387200	expressed sequence AA387200, transcript variant X1	NA	-0.39256	1.346515	-1.31272	0.16993349	0.486824
170749	Mtmr4	myotubularin related protein 4, transcript variant X7	NA	0.080362	6.946094	1.057283	0.16997905	0.486868
70827	Trak2	trafficking protein, kinesin binding 2, transcript variant	NA	0.072342	6.203523	1.051422	0.17001946	0.486898
14696	Gnb4	guanine nucleotide binding protein (G protein), beta 4	NA	0.080834	6.12908	1.057629	0.17012589	0.487117
118568308	LOC118568308	igE-binding protein-like	NA	0.252584	2.165976	1.191339	0.17017583	0.487175
12217	Bsn	bassoon	NA	0.0754	7.089399	1.053653	0.17029636	0.487434
12728	Clcn5	chloride channel, voltage-sensitive 5, transcript variar	NA	0.107676	5.071077	1.077491	0.17043689	0.487686
18706	Pik3ca	phosphatidylinositol-4,5-bisphosphate 3-kinase cataly	NA	0.081053	6.769323	1.057789	0.1704445	0.487686
52004	Cdk2ap2	CDK2-associated protein 2	NA	-0.14944	3.833725	-1.10914	0.1705061	0.487716
20937	Suv39h1	suppressor of variegation 3-9 1, transcript variant 1	NA	-0.08312	5.638528	-1.0593	0.1705149	0.487716
328949	Mcc	mutated in colorectal cancers, transcript variant 2	NA	0.149267	3.657823	1.109006	0.17056783	0.487782
239170	Fam160b2	family with sequence similarity 160, member B2	NA	-0.10658	4.84657	-1.07667	0.17066334	0.487903
57814	Kcne4	potassium voltage-gated channel, Isk-related subfam	NA	-0.55369	0.420437	-1.46784	0.17067017	0.487903
207375	Fam120c	family with sequence similarity 120, member C	NA	0.090325	5.365245	1.06461	0.17077673	0.488122
72301	Shisal1	shisa like 1, transcript variant 1	NA	0.084207	5.930619	1.060105	0.17085408	0.488217
18796	Plcb2	phospholipase C, beta 2, transcript variant X1	NA	-0.4085	0.386168	-1.32731	0.17087532	0.488217
237625	Pla2g3	phospholipase A2, group III, transcript variant X7	NA	0.31227	1.544659	1.24166	0.17090117	0.488217
12576	Cdkn1b	cyclin-dependent kinase inhibitor 1B	NA	-0.10491	7.00807	-1.07543	0.17096961	0.488217
320277	Spef2	sperm flagellar 2, transcript variant 1	NA	0.276509	1.510674	1.21126	0.17101226	0.488217
76915	Mnd1	meiotic nuclear divisions 1, transcript variant X1	NA	-0.46246	0.319361	-1.37789	0.17103813	0.488217
75475	Oplah	5-oxoprolinase (ATP-hydrolysing), transcript variant X	NA	-0.15851	3.333771	-1.11613	0.17106246	0.488217
53608	Map3k6	mitogen-activated protein kinase kinase kinase 6	NA	-0.35458	0.957406	-1.27861	0.17111221	0.488217
60533	Cd274	CD274 antigen	NA	-0.35218	0.727631	-1.27649	0.17111781	0.488217
76927	Tsacc	TSSK6 activating co-chaperone, transcript variant 2	NA	0.386872	0.326679	1.307556	0.17113843	0.488217
320473	Heatr5b	HEAT repeat containing 5B	NA	-0.10997	5.292297	-1.07921	0.17115216	0.488217
11921	Atoh1	atonal bHLH transcription factor 1	NA	0.429739	0.718794	1.34699	0.17117881	0.488217
319207	Pgbd1	piggyBac transposable element derived 1, transcript \	NA	-0.19884	2.426615	-1.14778	0.17123034	0.488217
11973	Atp6v1e1	ATPase, H+ transporting, lysosomal V1 subunit E1	NA	0.067641	6.973877	1.048001	0.17125521	0.488217
66795	Atg10	autophagy related 10	NA	-0.18932	2.616071	-1.14023	0.17127649	0.488217
270120	Fat3	FAT atypical cadherin 3, transcript variant X6	NA	0.078164	6.531065	1.055674	0.17129017	0.488217
78560	Adgra2	adhesion G protein-coupled receptor A2	NA	-0.11741	4.719481	-1.08479	0.1713657	0.48822

100113398	Adat3	adenosine deaminase, tRNA-specific 3	NA	-0.29059	1.179882	-1.22314	0.17136714	0.48822
386655	Eid2	EP300 interacting inhibitor of differentiation 2	NA	0.094769	5.529142	1.067895	0.17138111	0.48822
67141	Fbxo5	F-box protein 5	NA	-0.16863	3.779427	-1.12399	0.1716695	0.488956
15482	Hspa1l	heat shock protein 1-like	NA	-0.55648	-0.33998	-1.47068	0.17170829	0.488981
70160	Vps36	vacuolar protein sorting 36, transcript variant 1	NA	0.072842	5.923017	1.051787	0.17180047	0.489158
13609	S1pr1	sphingosine-1-phosphate receptor 1	NA	0.090142	5.038859	1.064475	0.17184047	0.489166
101513	Mob2	MOB kinase activator 2, transcript variant 1	NA	0.114095	4.186953	1.082296	0.171945	0.489166
68332	Sdhaf1	succinate dehydrogenase complex assembly factor 1	NA	0.174689	3.942695	1.128721	0.17197738	0.489166
23794	Adamts5	a disintegrin-like and metallopeptidase (reprolysin typ	NA	0.152816	3.491026	1.111737	0.17198564	0.489166
102607	Snx19	sorting nexin 19	NA	-0.09212	5.613711	-1.06593	0.17201651	0.489166
329735	4933431E20Rik	RIKEN cDNA 4933431E20 gene	NA	0.080929	5.507092	1.057699	0.17205015	0.489166
11927	Atox1	antioxidant 1 copper chaperone	NA	0.124519	4.204826	1.090144	0.17205746	0.489166
13518	Dst	dystonin, transcript variant X29	NA	0.089049	7.523131	1.063669	0.17212777	0.489166
18014	Neurog1	neurogenin 1	NA	-0.30068	1.071746	-1.23173	0.17215353	0.489166
93671	Cd163	CD163 antigen, transcript variant 2	NA	0.484441	0.669151	1.399044	0.17215457	0.489166
263876	Spata2	spermatogenesis associated 2, transcript variant X5	NA	-0.08214	5.635177	-1.05858	0.17215797	0.489166
67046	Tbc1d7	TBC1 domain family, member 7, transcript variant 1	NA	0.103876	4.775177	1.074657	0.17217527	0.489166
66381	Rnf113a2	ring finger protein 113A2	NA	0.15399	3.527153	1.112643	0.17219412	0.489166
20324	Cavin2	caveolae associated 2	NA	0.181241	3.325307	1.133859	0.1723763	0.489468
50794	Klf13	Kruppel-like factor 13	NA	-0.07838	6.090302	-1.05583	0.17240085	0.489468
27965	Spg21	SPG21, maspardin, transcript variant 1	NA	0.075927	6.067412	1.054038	0.17240636	0.489468
76982	Vxn	vexin	NA	0.097045	5.611876	1.06958	0.17242064	0.489468
21375	Tbr1	T-box brain transcription factor 1	NA	-0.1272	7.233265	-1.09217	0.17248324	0.48956
107733	Mrpl41	mitochondrial ribosomal protein L41	NA	0.10872	4.754276	1.078271	0.17259542	0.489748
69398	Cdhr4	cadherin-related family member 4	NA	-0.4351	-0.03059	-1.35201	0.1726273	0.489748
22084	Tsc2	TSC complex subunit 2, transcript variant X21	NA	-0.06731	6.949423	-1.04776	0.17267278	0.489748
544678	Cfap74	cilia and flagella associated protein 74, transcript vari	NA	0.207119	2.605456	1.154381	0.17267834	0.489748
20479	Vps4b	vacuolar protein sorting 4B	NA	0.086554	5.560934	1.061831	0.1727106	0.489748
18039	Nefl	neurofilament, light polypeptide	NA	0.1028	7.884977	1.073856	0.17272995	0.489748
217737	Ahsa1	AHA1, activator of heat shock protein ATPase 1	NA	-0.07494	6.786209	-1.05332	0.17288221	0.489946
66609	Cryzl1	crystallin, zeta (quinone reductase)-like 1, transcript v	NA	0.082087	5.442415	1.058548	0.17289164	0.489946
282663	Serpinb1b	serine (or cysteine) peptidase inhibitor, clade B, mem	NA	0.25242	1.768152	1.191203	0.17291949	0.489946
85030	Tnfrsf25	tumor necrosis factor receptor superfamily, member 2	NA	-0.3562	0.737743	-1.28005	0.17296024	0.489946
208618	Etl4	enhancer trap locus 4, transcript variant X26	NA	-0.13481	4.128649	-1.09795	0.17297479	0.489946
18242	Oat	ornithine aminotransferase	NA	-0.08469	6.252355	-1.06046	0.17298042	0.489946
13601	Ecm1	extracellular matrix protein 1, transcript variant 1	NA	-0.16251	2.887352	-1.11923	0.17302973	0.489958
72999	Insig2	insulin induced gene 2, transcript variant 2	NA	0.107992	5.056082	1.077727	0.17304506	0.489958
52639	Wipi1	WD repeat domain, phosphoinositide interacting 1	NA	0.18726	3.178259	1.138599	0.1730772	0.489964
70575	Gfod2	glucose-fructose oxidoreductase domain containing 2	NA	-0.07686	6.070082	-1.05472	0.17314173	0.490061
76375	Det1	de-etiolated homolog 1 (Arabidopsis)	NA	-0.14632	3.51014	-1.10674	0.17324053	0.490166
227801	Dennd1a	DENN/MADD domain containing 1A, transcript variar	NA	-0.08368	5.978901	-1.05971	0.17332213	0.490166
320054	9230116N13Rik	RIKEN cDNA 9230116N13 gene	NA	0.450443	-0.15755	1.36646	0.17334551	0.490166
70834	Spag9	sperm associated antigen 9, transcript variant 4	NA	0.075615	8.287625	1.05381	0.17335312	0.490166
66176	Nat9	N-acetyltransferase 9 (GCN5-related, putative), trans	NA	0.247987	2.938231	1.187549	0.1733546	0.490166
74978	Lrriq1	leucine-rich repeats and IQ motif containing 1, transc	NA	0.25182	1.906667	1.190708	0.17337728	0.490166
118567402	LOC118567402	uncharacterized LOC118567402	NA	0.435536	0.088056	1.352413	0.17338954	0.490166
228942	Cbln4	cerebellin 4 precursor protein	NA	0.103418	4.621686	1.074315	0.17344667	0.490242
73218	Sppl2b	signal peptide peptidase like 2B, transcript variant 1	NA	-0.11468	5.38107	-1.08273	0.17351652	0.490354
107029	Me2	malic enzyme 2, NAD(+)-dependent, mitochondrial, tr	NA	-0.08649	5.463138	-1.06178	0.17357799	0.490426
353235	Pcdha8	protocadherin alpha 8	NA	0.64747	0.610357	1.566419	0.17360203	0.490426
108167650	Gm46110	predicted gene, 46110, transcript variant X4	NA	-0.52452	-0.37806	-1.43845	0.17370483	0.490572
67839	Gpsm1	G-protein signalling modulator 1 (AGS3-like, C. elega	NA	0.075971	7.413667	1.054071	0.173714	0.490572
213760	Prepl	prolyl endopeptidase-like, transcript variant X1	NA	0.066987	7.689332	1.047527	0.17378305	0.490617
72843	Prdm4	PR domain containing 4, transcript variant X6	NA	0.077619	5.986762	1.055275	0.17382901	0.490617
102637020	Gm33933	predicted gene, 33933	NA	-0.24719	1.798078	-1.1869	0.17383284	0.490617
20589	Ighmbp2	immunoglobulin mu binding protein 2, transcript varia	NA	0.135984	4.044581	1.098842	0.17385078	0.490617
16498	Kcnab2	potassium voltage-gated channel, shaker-related sub	NA	-0.11521	4.823203	-1.08313	0.17406541	0.490948
109181	Trip11	thyroid hormone receptor interactor 11, transcript vari	NA	0.082703	5.22814	1.059	0.17407302	0.490948
115488623	6720473M11Rik	RIKEN cDNA 6720473M11 gene	NA	-0.11366	4.964134	-1.08197	0.17409181	0.490948
54120	Gipc2	GIPC PDZ domain containing family, member 2, trans	NA	0.330418	0.929448	1.257378	0.17411303	0.490948
214253	Etnk2	ethanolamine kinase 2	NA	0.194659	3.098841	1.144453	0.17411885	0.490948
68915	Vars2	valyl-tRNA synthetase 2, mitochondrial	NA	-0.096	4.818273	-1.06881	0.17420256	0.491031
96979	Ptges2	prostaglandin E synthase 2	NA	0.118724	4.569908	1.085774	0.17420863	0.491031
627280	Vmn1r90	vomeronasal 1 receptor 90	NA	1.000279	-0.39249	2.000386	0.174262	0.491097
16852	Lgals1	lectin, galactose binding, soluble 1	NA	-0.13736	4.975031	-1.09989	0.17440156	0.491381
69893	Coa7	cytochrome c oxidase assembly factor 7	NA	-0.12996	4.134585	-1.09427	0.17442336	0.491381
75458	Cklf	chemokine-like factor, transcript variant X2	NA	-0.14142	3.61874	-1.10299	0.17446399	0.49141
115490496	LOC115490496	small nucleolar RNA U89	NA	0.268531	1.815006	1.204581	0.17452726	0.491439

330830	Drc7	dynein regulatory complex subunit 7, transcript variar	NA	-0.25399	1.39262	-1.1925	0.1745347	0.491439
22019	Tpp2	tripeptidyl peptidase II, transcript variant 2	NA	0.08276	6.225209	1.059042	0.174576	0.491471
13616	Edn3	endothelin 3	NA	-0.16621	2.706961	-1.12211	0.17466836	0.491646
18025	Nfe2l3	nuclear factor, erythroid derived 2, like 3	NA	0.14483	3.974586	1.105601	0.17474105	0.491765
50498	Ebi3	Epstein-Barr virus induced gene 3, transcript variant :	NA	0.335437	0.705238	1.26176	0.17491035	0.492127
382406	Poc1b	POC1 centriolar protein B, transcript variant 1	NA	-0.10127	4.787769	-1.07271	0.17497532	0.492127
14194	Fh1	fumarate hydratase 1	NA	0.099358	5.937388	1.071297	0.17500077	0.492127
66274	Lyrm9	LYR motif containing 9	NA	0.096008	4.72682	1.068812	0.17501361	0.492127
241322	Zbtb6	zinc finger and BTB domain containing 6	NA	0.084959	5.660047	1.060658	0.17502075	0.492127
19286	Pts	6-pyruvoyl-tetrahydropterin synthase, transcript variar	NA	0.11026	4.792832	1.079423	0.17505253	0.492131
118568130	LOC118568130	uncharacterized LOC118568130	NA	-0.27729	1.508617	-1.21191	0.17519427	0.492444
93684	Selenof	selenoprotein F	NA	0.081428	7.428529	1.058065	0.17524578	0.492464
76142	Ppp1r14c	protein phosphatase 1, regulatory inhibitor subunit 14	NA	0.079748	5.882591	1.056833	0.17529089	0.492464
105245076	Gm40578	predicted gene, 40578, transcript variant X1	NA	0.236926	1.718107	1.178479	0.17529196	0.492464
210135	Zfp180	zinc finger protein 180, transcript variant 1	NA	-0.08698	5.511661	-1.06214	0.17536297	0.492545
14998	H2-DMa	histocompatibility 2, class II, locus DMa, transcript va	NA	0.183947	2.987231	1.135987	0.17539209	0.492545
115486887	Gm51541	predicted gene, 51541	NA	0.327574	1.413871	1.254901	0.17541147	0.492545
66679	Rae1	ribonucleic acid export 1, transcript variant X2	NA	0.077522	5.952949	1.055204	0.1754744	0.492616
12287	Cacna1b	calcium channel, voltage-dependent, N type, alpha 1I	NA	0.085305	6.875018	1.060912	0.17549749	0.492616
215705	Arrdc1	arrestin domain containing 1, transcript variant X8	NA	0.125533	4.110034	1.09091	0.17558114	0.492711
170625	Snx18	sorting nexin 18	NA	0.094927	5.03937	1.068011	0.17561574	0.492711
100568459	Bc1	brain cytoplasmic RNA 1	NA	0.101457	4.45078	1.072856	0.17562205	0.492711
66290	Atp6v1g1	ATPase, H+ transporting, lysosomal V1 subunit G1	NA	0.07116	7.299569	1.050561	0.17573156	0.492921
72322	Xpo5	exportin 5	NA	-0.07102	6.388187	-1.05046	0.175771	0.492921
320267	Fubp3	far upstream element (FUSE) binding protein 3, trans	NA	0.075883	5.798235	1.054006	0.17579121	0.492921
97031	Tprn	taperin	NA	0.127613	3.70401	1.092485	0.17581817	0.492921
66403	Asf1a	anti-silencing function 1A histone chaperone	NA	0.095436	4.938674	1.068388	0.17587247	0.492988
28105	Trim36	tripartite motif-containing 36, transcript variant 1	NA	-0.09762	5.075591	-1.07001	0.17600656	0.493279
74206	Sipa1l3	signal-induced proliferation-associated 1 like 3	NA	-0.10469	5.651777	-1.07526	0.17606655	0.493362
116873	Stim2	stromal interaction molecule 2, transcript variant 2	NA	0.081635	5.663789	1.058217	0.17624045	0.493765
56788	Scube2	signal peptide, CUB domain, EGF-like 2	NA	0.334835	1.153068	1.261233	0.17642322	0.494159
18383	Tnfrsf11b	tumor necrosis factor receptor superfamily, member '	NA	0.311498	0.972421	1.240996	0.1764432	0.494159
666899	Gm12191	predicted gene 12191	NA	-0.3184	2.811894	-1.24695	0.17648325	0.494159
109205	Sobp	sine oculis binding protein	NA	-0.06934	7.236226	-1.04923	0.17654317	0.494159
108168283	Gm46548	predicted gene, 46548	NA	0.24857	3.507136	1.188029	0.17654614	0.494159
19116	Prlr	prolactin receptor, transcript variant X4	NA	0.260972	1.743101	1.198285	0.17657323	0.494159
72948	Tppp	tubulin polymerization promoting protein	NA	-0.1299	4.536017	-1.09422	0.17662061	0.494159
14885	Gtf2h4	general transcription factor II H, polypeptide 4	NA	-0.14189	3.714445	-1.10335	0.17662417	0.494159
17886	Myh9	myosin, heavy polypeptide 9, non-muscle	NA	-0.08641	6.453218	-1.06173	0.17676286	0.494315
103573	Xpo1	exportin 1, transcript variant 2	NA	-0.07417	7.813547	-1.05275	0.17677036	0.494315
67048	Vma21	VMA21 vacuolar H+-ATPase homolog (S. cerevisiae)	NA	0.088552	6.165127	1.063303	0.17677096	0.494315
16520	Kcnj4	potassium inwardly-rectifying channel, subfamily J, m	NA	0.3756	0.515365	1.297379	0.17687706	0.494526
118568563	LOC118568563	uncharacterized LOC118568563, transcript variant X	NA	-0.09867	4.726653	-1.07079	0.17690965	0.494533
73204	3110056K07Rik	RIKEN cDNA 3110056K07 gene, transcript variant 2	NA	-0.20352	2.881428	-1.15151	0.17698322	0.494563
67665	Dctn4	dynactin 4, transcript variant 2	NA	0.065391	6.905986	1.046368	0.17700373	0.494563
232236	Ccdc174	coiled-coil domain containing 174	NA	0.132121	4.747553	1.095904	0.17701178	0.494563
240255	Ythdc2	YTH domain containing 2, transcript variant X3	NA	0.114947	4.732267	1.082936	0.17709478	0.494662
22295	Cdh23	cadherin 23 (otocadherin), transcript variant X5	NA	-0.17901	2.687276	-1.1321	0.17710785	0.494662
67188	2700046G09Rik	RIKEN cDNA 2700046G09 gene	NA	0.284593	1.135665	1.218066	0.17715253	0.494692
64113	Moap1	modulator of apoptosis 1, transcript variant 1	NA	0.337194	4.389648	1.263297	0.17717949	0.494692
69612	Kansl2	KAT8 regulatory NSL complex subunit 2, transcript va	NA	0.065866	6.987183	1.046713	0.17723644	0.494766
100169	Phactr4	phosphatase and actin regulator 4, transcript variant :	NA	-0.10504	5.215688	-1.07552	0.1772849	0.494816
66121	Chchd1	coiled-coil-helix-coiled-coil-helix domain containing 1	NA	-0.12098	5.105892	-1.08747	0.17741421	0.495092
13036	Ctsh	cathepsin H, transcript variant 1	NA	0.152778	3.215996	1.111708	0.17745993	0.495135
237553	Trhde	TRH-degrading enzyme	NA	0.159024	3.509875	1.116532	0.17757555	0.495373
58911	Sumf1	sulfatase modifying factor 1	NA	0.148924	3.833603	1.108743	0.17760863	0.49538
214063	Dnajc16	DnaJ heat shock protein family (Hsp40) member C16	NA	-0.08674	5.217719	-1.06197	0.1777911	0.495804
102640021	Gm36195	predicted gene, 36195	NA	-0.49588	-0.18027	-1.41018	0.17784328	0.495836
56249	Actr8	ARP8 actin-related protein 8	NA	-0.09093	5.524795	-1.06506	0.1778635	0.495836
66845	Mrpl33	mitochondrial ribosomal protein L33	NA	0.112345	4.325965	1.080984	0.17790541	0.495868
399603	Lratd2	LRAT domain containing 1	NA	-0.14165	4.948188	-1.10316	0.17797554	0.495978
104910	Slc25a47	solute carrier family 25, member 47, transcript variant	NA	0.261283	1.926908	1.198544	0.17807479	0.49617
18740	Pitx1	paired-like homeodomain transcription factor 1	NA	-0.4162	0.435785	-1.3344	0.17811194	0.496189
497652	Acd	adrenocortical dysplasia, transcript variant 1	NA	0.090426	5.383421	1.064684	0.17831579	0.496516
63958	Ube4b	ubiquitination factor E4B	NA	-0.0681	7.479653	-1.04834	0.17831629	0.496516
654805	F930015N05Rik	RIKEN cDNA F930015N05 gene	NA	0.403499	0.071923	1.322712	0.17835945	0.496516
378466	Gm10033	predicted gene 10033, transcript variant 4	NA	-0.18085	2.884344	-1.13355	0.17838919	0.496516
98710	Rabif	RAB interacting factor	NA	0.083556	5.47925	1.059626	0.17841158	0.496516

194126	Mtmr11	myotubularin related protein 11	NA	0.19603	3.008664	1.145541	0.17841239	0.496516
74580	Pyroxd2	pyridine nucleotide-disulphide oxidoreductase domain	NA	-0.24371	1.921996	-1.18403	0.17876008	0.497238
70423	Tspan15	tetraspanin 15	NA	0.166114	3.564333	1.122032	0.17876523	0.497238
102640310	Gm36409	predicted gene, 36409, transcript variant X1	NA	0.371027	0.40092	1.293273	0.17879537	0.497238
18115	Nnt	nicotinamide nucleotide transhydrogenase, transcript	NA	-0.11258	5.45014	-1.08116	0.17880619	0.497238
15982	Ifrd1	interferon-related developmental regulator 1	NA	0.080362	5.636656	1.057283	0.17884057	0.497238
329324	Syt14	synaptotagmin XIV, transcript variant 2	NA	0.091036	5.308197	1.065135	0.17885519	0.497238
672511	Rnf213	ring finger protein 213	NA	0.230809	2.304795	1.173493	0.17890558	0.497293
105278	Cdk20	cyclin-dependent kinase 20	NA	-0.13484	3.735413	-1.09797	0.17895117	0.497334
219170	Fam216b	family with sequence similarity 216, member B	NA	0.355477	0.400517	1.279408	0.17906999	0.497493
100502849	Gm19412	predicted gene, 19412	NA	-0.3669	0.406173	-1.28958	0.17909024	0.497493
16561	Kif1b	kinesin family member 1B, transcript variant X11	NA	0.066871	9.063065	1.047442	0.17917812	0.497493
381760	Ssbp1	single-stranded DNA binding protein 1, transcript vari	NA	-0.08674	5.330489	-1.06197	0.17920661	0.497493
14366	Fzd4	frizzled class receptor 4, transcript variant X1	NA	0.142657	3.397295	1.103937	0.17922237	0.497493
13196	Asap1	ArfGAP with SH3 domain, ankyrin repeat and PH dor	NA	0.071408	6.084748	1.050741	0.17923135	0.497493
115490412	Gm52904	predicted gene, 52904	NA	0.575916	-0.46525	1.490624	0.17924595	0.497493
70592	5730480H06Rik	RIKEN cDNA 5730480H06 gene	NA	0.253016	1.910893	1.191696	0.17927508	0.497493
71151	Eri2	exoribonuclease 2, transcript variant X3	NA	-0.11843	4.500632	-1.08555	0.17928346	0.497493
98828	Cdc123	cell division cycle 123, transcript variant 1	NA	-0.0793	6.386359	-1.0565	0.1793695	0.497647
20849	Stat4	signal transducer and activator of transcription 4, tran	NA	0.630698	-0.52403	1.548314	0.17956988	0.498062
219189	Vwa8	von Willebrand factor A domain containing 8, transcri	NA	0.114902	4.300186	1.082902	0.17959026	0.498062
268470	Ube2z	ubiquitin-conjugating enzyme E2Z	NA	-0.07573	6.867559	-1.05389	0.17961089	0.498062
69019	Spcs1	signal peptidase complex subunit 1 homolog (S. cere	NA	0.093928	5.953989	1.067272	0.17964682	0.498077
12050	Bcl2l2	BCL2-like 2	NA	-0.38813	6.398747	-1.30869	0.17979371	0.498399
68644	Abhd14a	abhydrolase domain containing 14A, transcript varian	NA	-0.12309	4.541714	-1.08907	0.17983189	0.498408
268319	BC025920	cDNA sequence BC025920	NA	-0.19841	2.560755	-1.14743	0.17989408	0.498408
107448	Unc5a	unc-5 netrin receptor A, transcript variant 2	NA	-0.08329	5.351198	-1.05943	0.17990543	0.498408
16873	Lhx5	LIM homeobox protein 5	NA	-0.10511	4.668082	-1.07558	0.17992796	0.498408
223658	Mroh1	maestro heat-like repeat family member 1, transcript	NA	-0.11162	5.147762	-1.08044	0.17994992	0.498408
67703	Kirrel3	kirre like nephrin family adhesion molecule 3, transcri	NA	-0.08319	6.769302	-1.05936	0.18006552	0.498643
13669	Eif3a	eukaryotic translation initiation factor 3, subunit A	NA	0.06426	8.079812	1.045548	0.1801364	0.498736
14050	Eya3	EYA transcriptional coactivator and phosphatase 3, tr	NA	-0.08837	5.428216	-1.06317	0.18016042	0.498736
217232	Cdc27	cell division cycle 27, transcript variant X5	NA	0.077556	6.417158	1.055229	0.18019693	0.498752
228998	Arfgap1	ADP-ribosylation factor GTPase activating protein 1,	NA	-0.07202	6.096638	-1.05119	0.18028709	0.498904
117589	Asb7	ankyrin repeat and SOCS box-containing 7, transcrip	NA	0.089628	4.799902	1.064096	0.18031298	0.498904
68606	Ppm1f	protein phosphatase 1F (PP2C domain containing), tı	NA	-0.09429	5.475792	-1.06754	0.18036465	0.498962
20846	Stat1	signal transducer and activator of transcription 1, tran	NA	-0.14801	3.531328	-1.10804	0.18042988	0.499057
241944	Zfp267	zinc finger protein 267	NA	0.099276	5.261052	1.071236	0.18047232	0.49909
21429	Ubtf	upstream binding transcription factor, RNA polymeras	NA	-0.07312	7.403334	-1.05199	0.18055086	0.499222
226856	Lpgat1	lysophosphatidylglycerol acyltransferase 1, transcript	NA	0.087073	6.895824	1.062213	0.18059164	0.49925
226409	Zranb3	zinc finger, RAN-binding domain containing 3, transcı	NA	-0.1388	3.376897	-1.10099	0.18063121	0.499275
231002	Plekhn1	pleckstrin homology domain containing, family N mer	NA	-0.18948	3.118291	-1.14035	0.18080622	0.499674
633395	Gm10548	predicted gene 10548	NA	-0.31293	0.983687	-1.24223	0.1808513	0.499713
93717	Pcdhga9	protocadherin gamma subfamily A, 9	NA	-0.15658	4.168745	-1.11464	0.18093664	0.499864
268291	Rnf217	ring finger protein 217	NA	0.134309	4.002583	1.097567	0.18097454	0.499884
21407	Tcf15	transcription factor 15	NA	0.480002	-0.22829	1.394746	0.18103817	0.499893
27981	Rsrp1	arginine/serine rich protein 1	NA	-0.11134	7.527556	-1.08023	0.18105194	0.499893
110593	Prdm2	PR domain containing 2, with ZNF domain, transcript	NA	-0.08905	6.884718	-1.06367	0.18106986	0.499893
80291	Rilpl2	Rab interacting lysosomal protein-like 2	NA	0.136797	3.477286	1.099461	0.1811119	0.499924
54610	Tbc1d8	TBC1 domain family, member 8, transcript variant 1	NA	0.108127	4.407345	1.077828	0.18115725	0.499965
66960	Mindy3	MINDY lysine 48 deubiquitinase 3, transcript variant)	NA	0.084946	5.288153	1.060648	0.18123517	0.500095
18559	Pctp	phosphatidylcholine transfer protein, transcript varian	NA	-0.3198	0.812078	-1.24816	0.18126851	0.500102
100503652	Gm16845	predicted gene, 16845, transcript variant 2	NA	0.438742	0.014284	1.355422	0.18135276	0.50025
20847	Stat2	signal transducer and activator of transcription 2	NA	-0.09062	4.987928	-1.06483	0.18157004	0.500682
56016	Hebp2	heme binding protein 2	NA	-0.21621	1.990645	-1.16168	0.1815711	0.500682
242894	Actr3b	ARP3 actin-related protein 3B, transcript variant X2	NA	0.091696	4.815994	1.065622	0.18162271	0.50074
18205	Ntf3	neurotrophin 3, transcript variant 2	NA	0.27901	1.466695	1.213362	0.18172263	0.500751
245527	Eda2r	ectodysplasin A2 receptor, transcript variant 2	NA	-0.29838	1.024403	-1.22976	0.18175089	0.500751
76497	Ppp1r11	protein phosphatase 1, regulatory inhibitor subunit 11	NA	-0.09105	5.481068	-1.06515	0.18175998	0.500751
67003	Uqcrc2	ubiquinol cytochrome c reductase core protein 2	NA	0.076916	7.34986	1.054761	0.18176593	0.500751
20529	Slc31a1	solute carrier family 31, member 1	NA	0.0769	5.914202	1.054749	0.18180913	0.500751
78934	4930581F22Rik	RIKEN cDNA 4930581F22 gene	NA	-0.40669	0.267045	-1.32564	0.18181139	0.500751
18173	Slc11a1	solute carrier family 11 (proton-coupled divalent meta	NA	-0.43731	0.287453	-1.35408	0.18189689	0.500896
11622	Ahr	aryl-hydrocarbon receptor, transcript variant 2	NA	0.159615	2.972996	1.116989	0.18199755	0.500896
105242900	Gm38983	predicted gene, 38983, transcript variant X2	NA	-0.16107	3.484328	-1.11812	0.18203484	0.500896
102640779	2900052L18Rik	RIKEN cDNA 2900052L18 gene	NA	0.140016	3.735466	1.101918	0.18203534	0.500896
100041530	Gm10409	predicted gene 10409	NA	0.144804	4.632559	1.10558	0.18203852	0.500896
213391	Rassf4	Ras association (RalGDS/AF-6) domain family memb	NA	0.116876	4.151561	1.084384	0.1820489	0.500896

64451	Dip2a	disco interacting protein 2 homolog A, transcript varia	NA	0.08412	6.106225	1.060041	0.18213617	0.501052
60530	Fignl1	fidgetin-like 1, transcript variant X5	NA	-0.14532	3.763224	-1.10597	0.18216892	0.501057
12695	Patj	PATJ, crumbs cell polarity complex component, trans	NA	0.133891	3.774961	1.097249	0.18244256	0.501725
12041	Bckdk	branched chain ketoacid dehydrogenase kinase	NA	-0.08573	5.113289	-1.06122	0.18255111	0.501938
75894	Adal	adenosine deaminase-like, transcript variant 1	NA	0.101887	4.695685	1.073176	0.18262491	0.502011
118567987	LOC118567987	zinc finger protein 665-like	NA	-0.25791	1.594814	-1.19575	0.18263913	0.502011
77422	C330018D20Rik	RIKEN cDNA C330018D20 gene	NA	-0.14961	3.751218	-1.10927	0.18289529	0.50263
21974	Top2b	topoisomerase (DNA) II beta	NA	0.076396	8.658418	1.054381	0.18298626	0.502795
241311	Zbtb34	zinc finger and BTB domain containing 34, transcript	NA	0.090077	5.4839	1.064427	0.18302819	0.502825
18647	Cdk14	cyclin-dependent kinase 14, transcript variant X5	NA	0.079862	6.375298	1.056917	0.18315362	0.503085
72973	Fbxo47	F-box protein 47, transcript variant X6	NA	0.299726	0.840388	1.230911	0.18327057	0.503297
109202	A930024E05Rik	RIKEN cDNA A930024E05 gene	NA	-0.20852	2.75523	-1.1555	0.18330237	0.503297
228361	Ambra1	autophagy/beclin 1 regulator 1, transcript variant X2	NA	-0.07605	6.473344	-1.05413	0.1833534	0.503297
319742	Mpzl3	myelin protein zero-like 3	NA	-0.3617	0.976822	-1.28494	0.18335443	0.503297
17681	Msc	musculin, transcript variant 1	NA	0.363912	0.163917	1.286911	0.18343377	0.50343
66231	Thoc7	THO complex 7, transcript variant 5	NA	0.086107	5.945728	1.061502	0.18356833	0.503714
18508	Pax6	paired box 6, transcript variant 6	NA	-0.07779	5.760375	-1.0554	0.18361679	0.503762
20446	St6galnac2	ST6 (alpha-N-acetyl-neuraminyl-2,3-beta-galactosyl-'	NA	-0.35794	0.736927	-1.2816	0.18369677	0.50388
16870	Lhx2	LIM homeobox protein 2, transcript variant X5	NA	-0.07944	6.98022	-1.05661	0.18372187	0.50388
18186	Nrp1	neuropilin 1, transcript variant 1	NA	0.069855	7.155713	1.049611	0.18382009	0.503998
105670	Rcbtb2	regulator of chromosome condensation (RCC1) and I	NA	0.070967	6.142945	1.050421	0.18383002	0.503998
66407	Mrps15	mitochondrial ribosomal protein S15	NA	-0.11597	4.537045	-1.08371	0.18385758	0.503998
15488	Hsd17b4	hydroxysteroid (17-beta) dehydrogenase 4	NA	-0.07013	6.401883	-1.04981	0.1838906	0.504003
54004	Diaph2	diaphanous related formin 2	NA	0.14276	3.740184	1.104015	0.18399421	0.504202
17999	Nedd4	neural precursor cell expressed, developmentally dov	NA	-0.05931	9.963414	-1.04197	0.18417613	0.504548
319564	C230012O17Rik	RIKEN cDNA C230012O17 gene, transcript variant X	NA	0.431442	0.062057	1.34858	0.18426451	0.504548
72787	Ndc1	NDC1 transmembrane nucleoporin, transcript variant	NA	0.092966	5.324719	1.066561	0.18434099	0.504548
67884	Cfap410	cilia and flagella associated protein 410	NA	0.127566	4.321842	1.092449	0.1843751	0.504548
320091	Ano4	anoctamin 4, transcript variant 1	NA	0.152295	3.582034	1.111336	0.18437837	0.504548
12421	Rb1cc1	RB1-inducible coiled-coil 1, transcript variant X5	NA	0.086486	6.166906	1.061781	0.18438435	0.504548
269633	Wdr86	WD repeat domain 86, transcript variant X1	NA	-0.19679	2.481542	-1.14615	0.18439048	0.504548
107513	Ssr1	signal sequence receptor, alpha, transcript variant 2	NA	0.068618	7.384501	1.048711	0.18439149	0.504548
102641372	Gm38553	predicted gene, 38553	NA	0.279716	1.29122	1.213956	0.18440977	0.504548
72413	Kcnmb2	potassium large conductance calcium-activated chan	NA	0.164898	3.486897	1.121087	0.18455082	0.504548
78895	Pus7l	pseudouridylate synthase 7-like	NA	0.170381	2.619302	1.125356	0.18457209	0.504548
93694	Clec2d	C-type lectin domain family 2, member d	NA	-0.29172	1.600032	-1.2241	0.18461001	0.504548
18634	Pex7	peroxisomal biogenesis factor 7, transcript variant 1	NA	0.135245	3.775016	1.098279	0.1846543	0.504548
68364	0610030E20Rik	RIKEN cDNA 0610030E20 gene	NA	-0.10519	4.699865	-1.07563	0.18468535	0.504548
17939	Naga	N-acetyl galactosaminidase, alpha, transcript variant	NA	-0.12267	3.980202	-1.08875	0.18468642	0.504548
57754	Cend1	cell cycle exit and neuronal differentiation 1, transcrip	NA	0.09554	6.099306	1.068465	0.18471577	0.504548
74293	1700095J03Rik	RIKEN cDNA 1700095J03 gene	NA	-0.32866	1.044755	-1.25585	0.18471877	0.504548
100041953	Sap18b	Sin3-associated polypeptide 18B	NA	0.074374	5.864445	1.052904	0.18473399	0.504548
67726	Fam114a2	family with sequence similarity 114, member A2, tran	NA	-0.07901	6.346515	-1.05629	0.1847564	0.504548
15182	Hdac2	histone deacetylase 2	NA	-0.06891	8.401936	-1.04893	0.18476444	0.504548
100503142	B230303A05Rik	RIKEN cDNA B230303A05 gene	NA	0.384402	0.36103	1.305318	0.18479448	0.504548
269437	Plch1	phospholipase C, eta 1, transcript variant 1	NA	0.119744	4.253993	1.086542	0.18480246	0.504548
115488807	Gm52310	predicted gene, 52310	NA	0.252108	1.602764	1.190946	0.1848578	0.504614
239789	Gmnc	geminin coiled-coil domain containing, transcript varia	NA	0.291848	1.309304	1.224207	0.18493318	0.504711
85308	Emc9	ER membrane protein complex subunit 9	NA	0.203003	2.357153	1.151092	0.18495531	0.504711
235469	Zfp280d	zinc finger protein 280D, transcript variant X1	NA	0.075171	6.012797	1.053486	0.18504362	0.504867
72690	Grrp1	glycine/arginine rich protein 1	NA	0.186456	2.57882	1.137965	0.18523658	0.505243
27140	Tlx3	T cell leukemia, homeobox 3	NA	0.338878	1.41662	1.264773	0.18527777	0.505243
66935	Cir1	corepressor interacting with RBPJ, 1	NA	0.089298	5.388169	1.063852	0.18529752	0.505243
381338	Lonrf2	LON peptidase N-terminal domain and ring finger 2	NA	0.069935	7.409034	1.04967	0.1853053	0.505243
328968	Carmn	cardiac mesoderm enhancer-associated non-coding I	NA	0.337446	0.494491	1.263518	0.18541854	0.505459
195733	Grhl1	grainyhead like transcription factor 1, transcript variar	NA	-0.32973	1.128414	-1.25677	0.18544696	0.505459
67226	Tmem19	transmembrane protein 19, transcript variant 1	NA	0.091591	5.331827	1.065545	0.18560908	0.505817
266690	Cyb5r4	cytochrome b5 reductase 4, transcript variant X1	NA	-0.07988	5.779918	-1.05693	0.18566858	0.505885
109700	Itga1	integrin alpha 1	NA	0.175499	2.909903	1.129355	0.18569639	0.505885
58801	Pmaip1	phorbol-12-myristate-13-acetate-induced protein 1	NA	0.22921	1.615406	1.172193	0.18583554	0.506174
574403	Insyn2b	inhibitory synaptic factor family member 2B	NA	0.147569	3.837957	1.107701	0.18586871	0.506174
16816	Lcat	lecithin cholesterol acyltransferase	NA	-0.353	0.714361	-1.27721	0.1859438	0.506174
327812	Gm15663	predicted gene 15663	NA	-0.14858	3.253982	-1.10848	0.18595795	0.506174
68895	Rasl11a	RAS-like, family 11, member A	NA	0.27917	1.621658	1.213497	0.18607113	0.506174
223701	Mrtfa	myocardin related transcription factor A, transcript va	NA	-0.09602	5.54088	-1.06882	0.18611355	0.506174
56615	Mgst1	microsomal glutathione S-transferase 1, transcript va	NA	0.134495	3.55324	1.097708	0.18611487	0.506174
22030	Traf2	TNF receptor-associated factor 2, transcript variant 2	NA	-0.12969	3.631005	-1.09406	0.18612505	0.506174
171286	Slc12a8	solute carrier family 12 (potassium/chloride transport	NA	0.442881	0.016276	1.359316	0.18614754	0.506174

233115	Dpy19l3	dpy-19-like 3 (C. elegans)	NA	-0.0918	5.782374	-1.0657	0.1861661	0.506174
76916	Timmdc1	translocase of inner mitochondrial membrane domain	NA	-0.11456	4.695919	-1.08265	0.18617146	0.506174
93703	Pcdhgb6	protocadherin gamma subfamily B, 6	NA	-0.10976	5.643175	-1.07905	0.18617554	0.506174
68337	Crip2	cysteine rich protein 2, transcript variant 2	NA	0.070278	6.968778	1.049919	0.18620944	0.506181
333654	Ppp1r13l	protein phosphatase 1, regulatory subunit 13 like, tra	NA	-0.24018	2.591681	-1.18114	0.18629666	0.50629
16478	Jund	jun D proto-oncogene, transcript variant 1	NA	0.087859	7.084968	1.062792	0.18631161	0.50629
319893	A230057D06Rik	RIKEN cDNA A230057D06 gene	NA	0.142947	3.672075	1.104158	0.18634554	0.506297
240756	Klhl12	kelch-like 12, transcript variant 1	NA	0.084932	5.383415	1.060638	0.18640145	0.506365
18516	Pbx3	pre B cell leukemia homeobox 3, transcript variant b	NA	0.067046	7.126536	1.047569	0.18644434	0.506397
56374	Tmem59	transmembrane protein 59	NA	0.074943	7.102489	1.05332	0.18650694	0.506482
66185	Virma	vir like m6A methyltransferase associated, transcript	NA	-0.07173	6.002732	-1.05097	0.18673142	0.507007
14013	Mecom	MDS1 and EVI1 complex locus, transcript variant X1	NA	0.163353	2.944851	1.119887	0.18678638	0.507012
12827	Col4a2	collagen, type IV, alpha 2	NA	-0.06732	7.761506	-1.04777	0.18681855	0.507012
105242928	Gm39000	predicted gene, 39000, transcript variant X2	NA	0.270613	1.50422	1.20632	0.1868425	0.507012
115489905	Gm52681	predicted gene, 52681, transcript variant X1	NA	0.289341	1.019474	1.222082	0.18687768	0.507012
12523	Cd84	CD84 antigen, transcript variant 1	NA	0.413484	0.594812	1.331898	0.18688885	0.507012
233065	Alkbh6	alkB homolog 6	NA	-0.11632	4.289665	-1.08397	0.18692209	0.507017
76073	Pcgf5	polycomb group ring finger 5, transcript variant 5	NA	0.150177	3.398427	1.109706	0.18699223	0.507123
118568126	LOC118568126	uncharacterized LOC118568126	NA	-0.34538	1.320772	-1.27048	0.18704498	0.507143
18109	Mycn	v-myc avian myelocytomatosis viral related oncogene	NA	-0.09302	6.320289	-1.0666	0.18709244	0.507143
77453	Dmrta2os	doublesex and mab-3 related transcription factor like	NA	-0.47953	-0.33486	-1.39429	0.18710056	0.507143
11630	Crybg1	crystallin beta-gamma domain containing 1, transcrip	NA	-0.48274	0.247712	-1.3974	0.18715285	0.507143
68721	1110032A03Rik	RIKEN cDNA 1110032A03 gene, transcript variant 1	NA	0.1436	4.38741	1.104658	0.1871553	0.507143
102640930	Gm36877	predicted gene, 36877	NA	0.459307	-0.27372	1.374881	0.18720721	0.50717
18591	Pdgfb	platelet derived growth factor, B polypeptide, transcri	NA	0.131475	3.972214	1.095413	0.18722912	0.50717
213054	Gabpb2	GA repeat binding protein, beta 2, transcript variant 2	NA	-0.09232	4.877207	-1.06608	0.18725909	0.50717
14229	Fkbp5	FK506 binding protein 5	NA	-0.07691	6.090464	-1.05476	0.18731209	0.50723
13643	Efnb3	ephrin B3	NA	-0.06318	7.865907	-1.04477	0.18734405	0.507232
13048	Cux2	cut-like homeobox 2, transcript variant X14	NA	-0.07162	6.712906	-1.0509	0.1876459	0.507875
12348	Car11	carbonic anhydrase 11, transcript variant 1	NA	-0.11317	5.465999	-1.0816	0.18764874	0.507875
319713	Ablim3	actin binding LIM protein family, member 3, transcript	NA	-0.0697	7.455662	-1.0495	0.18767546	0.507875
11911	Atf4	activating transcription factor 4, transcript variant 2	NA	0.061485	7.145428	1.04354	0.18778597	0.507965
103784	Wdr92	WD repeat domain 92	NA	-0.11323	3.982028	-1.08165	0.18782058	0.507965
216869	Arrb2	arrestin, beta 2, transcript variant 3	NA	-0.07107	6.032733	-1.0505	0.18782167	0.507965
98376	Gorab	golgin, RAB6-interacting, transcript variant 1	NA	0.11971	4.233199	1.086516	0.18783347	0.507965
115488130	LOC115488130	zinc finger protein 431-like	NA	0.106681	4.157175	1.076748	0.18792348	0.508049
74213	Rbm26	RNA binding motif protein 26, transcript variant 5	NA	0.068049	6.860991	1.048298	0.18793667	0.508049
11468	Actg2	actin, gamma 2, smooth muscle, enteric	NA	0.397138	0.103241	1.316893	0.18798813	0.508049
66915	Cops9	COP9 signalosome subunit 9, transcript variant 1	NA	0.084963	5.40075	1.060661	0.18798951	0.508049
67959	Puf60	poly-U binding splicing factor 60, transcript variant 2	NA	-0.07082	7.25172	-1.05031	0.18808066	0.508157
641340	Nrbf2	nuclear receptor binding factor 2	NA	0.104666	4.558822	1.075245	0.18809171	0.508157
21647	Tcte3	t-complex-associated testis expressed 3, transcript va	NA	0.206953	2.939963	1.154248	0.18821071	0.508163
19166	Psma2	proteasome subunit alpha 2	NA	0.074115	6.771036	1.052715	0.18822062	0.508163
54156	Egfl6	EGF-like-domain, multiple 6	NA	-0.28829	2.494024	-1.22119	0.18822488	0.508163
106582	Nrm	nurim (nuclear envelope membrane protein)	NA	-0.15026	3.388881	-1.10977	0.18824018	0.508163
53869	Rab11a	RAB11A, member RAS oncogene family	NA	0.081412	7.399222	1.058053	0.18825009	0.508163
15566	Htr7	5-hydroxytryptamine (serotonin) receptor 7, transcript	NA	0.150506	3.537476	1.109958	0.18850371	0.50871
105242667	Gm38820	predicted gene, 38820, transcript variant X1	NA	-0.2457	1.748724	-1.18567	0.18851533	0.50871
380912	Zfp395	zinc finger protein 395, transcript variant X2	NA	-0.14034	4.778261	-1.10216	0.18861164	0.508808
320027	Fstl4	follistatin-like 4, transcript variant X2	NA	0.133408	3.907544	1.096882	0.18861406	0.508808
235623	Scap	SREBF chaperone, transcript variant 2	NA	-0.07529	6.427427	-1.05357	0.18865559	0.508835
102639145	Gm17315	predicted gene, 17315	NA	0.477468	-0.27726	1.392298	0.18871947	0.50885
68046	2700062C07Rik	RIKEN cDNA 2700062C07 gene	NA	0.134592	3.84289	1.097782	0.18874336	0.50885
16881	Lig1	ligase I, DNA, ATP-dependent, transcript variant 2	NA	-0.10662	5.784116	-1.0767	0.18875466	0.50885
72836	Pot1b	protection of telomeres 1B, transcript variant X6	NA	-0.11108	4.542439	-1.08004	0.18881908	0.508861
16423	Cd47	CD47 antigen (Rh-related antigen, integrin-associate	NA	0.131347	5.482854	1.095316	0.1888216	0.508861
381510	Dpy19l4	dpy-19-like 4 (C. elegans)	NA	0.129102	5.025894	1.093612	0.18894766	0.509117
21982	Tmem165	transmembrane protein 165	NA	0.108875	5.756984	1.078387	0.18908503	0.509158
102640424	Gm36488	predicted gene, 36488, transcript variant X5	NA	-0.449	-0.28465	-1.36509	0.18912409	0.509158
56844	Tssc4	tumor-suppressing subchromosomal transferable frag	NA	0.086053	5.049758	1.061462	0.18912637	0.509158
54614	Prpf40b	pre-mRNA processing factor 40B, transcript variant 1	NA	-0.08737	6.662744	-1.06243	0.18913106	0.509158
211007	Trim41	tripartite motif-containing 41, transcript variant X2	NA	-0.08428	5.997817	-1.06016	0.189142	0.509158
433100	AA388235	expressed sequence AA388235	NA	-0.11533	4.586443	-1.08322	0.18916907	0.509158
15468	Prmt2	protein arginine N-methyltransferase 2, transcript vari	NA	-0.06486	7.264424	-1.04598	0.18919699	0.509158
71952	Riox1	ribosomal oxygenase 1	NA	-0.13744	3.828336	-1.09995	0.18922333	0.509158
227485	Cdh19	cadherin 19, type 2, transcript variant 1	NA	-0.43941	-0.2661	-1.35605	0.18928416	0.509158
118568152	LOC118568152	uncharacterized LOC118568152	NA	-0.33232	1.566087	-1.25903	0.18929429	0.509158
72507	Dzip1l	DAZ interacting protein 1-like, transcript variant X3	NA	-0.10544	4.275505	-1.07582	0.18939445	0.509158

268420	Alkbh5	alkB homolog 5, RNA demethylase	NA	-0.07688	6.027325	-1.05473	0.18941697	0.509158
73139	Cenpv	centromere protein V	NA	-0.12652	5.1973	-1.09166	0.18941789	0.509158
231668	Vsig10	V-set and immunoglobulin domain containing 10	NA	-0.2294	1.630404	-1.17235	0.18948012	0.509158
226089	Ric1	RAB6A GEF complex partner 1, transcript variant X2	NA	0.074126	5.684555	1.052723	0.18948227	0.509158
212706	N4bp3	NEDD4 binding protein 3, transcript variant X2	NA	0.15348	3.453513	1.112249	0.18953788	0.509158
225895	Taf6l	TATA-box binding protein associated factor 6 like, tra	NA	-0.11499	3.935861	-1.08297	0.18955604	0.509158
54377	Cacng4	calcium channel, voltage-dependent, gamma subunit	NA	-0.06162	8.30532	-1.04364	0.18957546	0.509158
102639375	Gm29514	predicted gene 29514, transcript variant 3	NA	-0.57025	-0.58911	-1.48478	0.18963304	0.509158
13733	Adgre1	adhesion G protein-coupled receptor E1, transcript va	NA	-0.20645	2.010407	-1.15384	0.18963878	0.509158
17761	Map7	microtubule-associated protein 7, transcript variant X	NA	0.130041	3.39658	1.094325	0.18964579	0.509158
54135	Lsr	lipolysis stimulated lipoprotein receptor, transcript var	NA	0.120757	3.760941	1.087305	0.1896674	0.509158
20514	Slc1a5	solute carrier family 1 (neutral amino acid transporter	NA	-0.15189	3.709927	-1.11102	0.18970979	0.509158
328099	Prps1l3	phosphoribosyl pyrophosphate synthetase 1-like 3	NA	-0.13221	4.876761	-1.09597	0.18974815	0.509158
67313	Inava	innate immunity activator, transcript variant X3	NA	-0.15574	2.892584	-1.11399	0.18979608	0.509158
629557	Gm6981	predicted pseudogene 6981	NA	0.387322	0.083879	1.307963	0.18983206	0.509158
12288	Cacna1c	calcium channel, voltage-dependent, L type, alpha 1(	NA	0.071988	6.342561	1.051164	0.18983759	0.509158
104271	Tex15	testis expressed gene 15	NA	0.17934	2.877775	1.132366	0.1898392	0.509158
19360	Rad50	RAD50 double strand break repair protein	NA	0.104175	4.15751	1.07488	0.18995597	0.509388
67389	C1qtnf12	C1q and tumor necrosis factor related 12	NA	-0.21296	1.908405	-1.15907	0.19001108	0.509451
68585	Rtn4	reticulon 4, transcript variant 5	NA	0.062663	9.092567	1.044392	0.19022464	0.509624
75990	5033421B08Rik	RIKEN cDNA 5033421B08 gene, transcript variant X	NA	0.139391	3.730539	1.10144	0.19024627	0.509624
116707477	Ipo11-lrrc70	Ipo11-lrrc70 readthrough	NA	0.155669	4.402433	1.113938	0.19025148	0.509624
50850	Spast	spastin, transcript variant 1	NA	0.0678	7.736444	1.048117	0.19029577	0.509624
74386	Rmi1	RecQ mediated genome instability 1, transcript variar	NA	-0.09014	4.869347	-1.06447	0.19032333	0.509624
385354	Frmd7	FERM domain containing 7	NA	0.466888	-0.12541	1.382125	0.19036729	0.509624
74052	Ttc21a	tetratricopeptide repeat domain 21A, transcript varian	NA	-0.23882	2.243455	-1.18003	0.19039918	0.509624
102637664	Gm34418	predicted gene, 34418, transcript variant X1	NA	-0.46262	0.159652	-1.37804	0.19041112	0.509624
66514	Asrgl1	asparaginase like 1	NA	0.091068	4.96514	1.065158	0.19043357	0.509624
30959	Ddx25	DEAD box helicase 25	NA	0.076692	5.67068	1.054597	0.19045416	0.509624
66361	Zfand1	zinc finger, AN1-type domain 1, transcript variant 1	NA	0.102965	4.461586	1.073979	0.19046327	0.509624
15360	Hmgcs2	3-hydroxy-3-methylglutaryl-Coenzyme A synthase 2	NA	0.124987	4.056543	1.090498	0.19047032	0.509624
66094	Lsm7	LSM7 homolog, U6 small nuclear RNA and mRNA de	NA	0.113647	4.519332	1.08196	0.19050634	0.509624
57837	Eral1	Era (G-protein)-like 1 (E. coli)	NA	-0.11449	4.411996	-1.08259	0.1905138	0.509624
14238	Foxf2	forkhead box F2	NA	-0.14467	3.448401	-1.10548	0.19056186	0.509668
20815	Srpk1	serine/arginine-rich protein specific kinase 1, transcri	NA	-0.07543	6.911484	-1.05368	0.19064092	0.509719
67857	Ppp6c	protein phosphatase 6, catalytic subunit, transcript va	NA	0.065952	6.370857	1.046775	0.1906436	0.509719
115488781	Gm52294	predicted gene, 52294	NA	-0.67724	0.202776	-1.59907	0.19073806	0.509888
232539	Klhl42	kelch-like 42	NA	0.114078	4.774041	1.082283	0.19077961	0.509916
100043143	Gm4258	predicted gene 4258, transcript variant X1	NA	-0.13734	5.315984	-1.09988	0.19081866	0.509936
21848	Trim24	tripartite motif-containing 24, transcript variant X1	NA	0.079913	6.5156	1.056954	0.19090212	0.509978
69066	1810010H24Rik	RIKEN cDNA 1810010H24 gene, transcript variant X(	NA	0.258482	2.466879	1.196219	0.19090327	0.509978
70247	Psmd1	proteasome (prosome, macropain) 26S subunit, non-	NA	0.062593	7.368286	1.044341	0.19092849	0.509978
107338	Gbf1	golgi-specific brefeldin A-resistance factor 1, transcri	NA	-0.08839	6.537747	-1.06319	0.19096726	0.509998
13796	Emx1	empty spiracles homeobox 1	NA	-0.13705	3.624186	-1.09965	0.19105316	0.510133
11878	Arx	aristaless related homeobox, transcript variant 1	NA	-0.09653	5.914407	-1.0692	0.19108045	0.510133
227731	Slc25a25	solute carrier family 25 (mitochondrial carrier, phosph	NA	-0.08523	5.728142	-1.06086	0.19111273	0.510136
19018	Scand1	SCAN domain-containing 1	NA	-0.1369	4.39297	-1.09954	0.19131007	0.510579
105245165	Gm40656	predicted gene, 40656	NA	-0.34961	0.862576	-1.27421	0.1914507	0.51087
12263	C2	complement component 2 (within H-2S)	NA	0.36851	0.233305	1.291019	0.19150856	0.510941
317717	Sec22a	SEC22 homolog A, vesicle trafficking protein, transcri	NA	0.12401	4.450289	1.08976	0.19155957	0.510993
667373	Ifit1bl1	interferon induced protein with tetratricpeptide repeat	NA	0.377844	0.160468	1.299398	0.1916697	0.511152
69159	Rhebl1	Ras homolog enriched in brain like 1, transcript varia	NA	0.128824	3.787779	1.093402	0.19168182	0.511152
71198	Otud1	OTU domain containing 1	NA	0.170891	2.99639	1.125754	0.19172693	0.511171
78523	Mrpl9	mitochondrial ribosomal protein L9	NA	-0.0969	5.527127	-1.06947	0.19175206	0.511171
26895	Cops7b	COP9 signalosome subunit 7B, transcript variant X1	NA	-0.08609	5.540824	-1.06149	0.19184113	0.511325
13488	Drd1	dopamine receptor D1, transcript variant 2	NA	-0.20463	2.797592	-1.15239	0.19188462	0.511357
381983	Lmtk3	lemur tyrosine kinase 3, transcript variant 1	NA	-0.0619	8.671352	-1.04384	0.19197654	0.511518
19724	Rfx1	regulatory factor X, 1 (influences HLA class II expres	NA	-0.0831	5.18504	-1.05929	0.19206075	0.511659
21384	Tbx15	T-box 15, transcript variant X1	NA	-0.25799	2.38493	-1.19581	0.1922136	0.511982
14085	Fah	fumarylacetoacetate hydrolase	NA	0.160738	3.424741	1.117859	0.19230228	0.512108
68713	Ifitm1	interferon induced transmembrane protein 1, transcri	NA	-0.27125	1.330616	-1.20685	0.19232357	0.512108
15361	Hmga1	high mobility group AT-hook 1, transcript variant 2	NA	0.074465	6.87579	1.052971	0.1924241	0.512209
109065	Dnaaf2	dynein, axonemal assembly factor 2	NA	-0.1221	3.756558	-1.08832	0.19244327	0.512209
56486	Gabarap	gamma-aminobutyric acid receptor associated protei	NA	-0.06803	7.777838	-1.04828	0.19245646	0.512209
12351	Car4	carbonic anhydrase 4	NA	0.289769	0.950248	1.222445	0.19248757	0.512209
71843	R3hcc1	R3H domain and coiled-coil containing 1, transcript v	NA	0.099626	5.093446	1.071496	0.1925764	0.51235
214685	Chadl	chondroadherin-like, transcript variant X3	NA	0.198729	3.327968	1.147687	0.19260368	0.51235
214642	Cped1	cadherin-like and PC-esterase domain containing 1, t	NA	0.137483	3.195443	1.099985	0.19263816	0.512358

233900	Rnf40	ring finger protein 40, transcript variant 1	NA	-0.06893	6.406715	-1.04894	0.19269901	0.512436
226594	Rcsd1	RCSD domain containing 1, transcript variant 2	NA	-0.17518	3.625039	-1.1291	0.1927974	0.512614
622434	Arhgef26	Rho guanine nucleotide exchange factor (GEF) 26	NA	0.098111	4.773983	1.070371	0.19290428	0.512815
12793	Cnih1	cornichon family AMPA receptor auxiliary protein 1, tr	NA	0.077462	6.429246	1.05516	0.19306591	0.51316
75568	Capsl	calcyphosine-like	NA	0.316522	0.762905	1.245325	0.19311157	0.513198
77974	Rdh12	retinol dehydrogenase 12, transcript variant 1	NA	-0.40901	0.104299	-1.32778	0.19329277	0.513596
67488	Calcoco1	calcium binding and coiled coil domain 1	NA	-0.09132	6.328755	-1.06534	0.19336378	0.5137
100503849	Sp3os	trans-acting transcription factor 3, opposite strand	NA	0.189499	2.251964	1.140368	0.19345242	0.513852
67843	Slc35a4	solute carrier family 35, member A4, transcript varian	NA	-0.08234	5.352277	-1.05874	0.19348961	0.513867
75619	Fastkd2	FAST kinase domains 2, transcript variant X1	NA	-0.13963	3.966065	-1.10162	0.1935416	0.513921
170753	Zfp704	zinc finger protein 704, transcript variant X1	NA	0.073483	7.370487	1.052254	0.19357356	0.513922
115488927	LOC115488927	uncharacterized LOC115488927	NA	0.337167	0.653967	1.263273	0.19369627	0.514092
56406	Ncoa6	nuclear receptor coactivator 6, transcript variant 1	NA	0.078412	6.891813	1.055855	0.1937009	0.514092
12262	C1qc	complement component 1, q subcomponent, C chain	NA	0.136543	3.381734	1.099268	0.19373802	0.514107
118451	Mrps2	mitochondrial ribosomal protein S2, transcript variant	NA	-0.09322	5.368579	-1.06675	0.19380763	0.514208
214901	Chtf18	CTF18, chromosome transmission fidelity factor 18	NA	-0.19897	2.884568	-1.14788	0.19398336	0.514576
26936	Mprip	myosin phosphatase Rho interacting protein, transcri	NA	-0.06079	8.476574	-1.04303	0.19404122	0.514576
77124	9130221H12Rik	RIKEN cDNA 9130221H12 gene	NA	-0.6917	1.934148	-1.61518	0.19406517	0.514576
72055	Slc38a10	solute carrier family 38, member 10, transcript varian	NA	-0.08895	6.830189	-1.06359	0.19409453	0.514576
226517	Smg7	Smg-7 homolog, nonsense mediated mRNA decay fa	NA	0.066754	6.759412	1.047357	0.19410426	0.514576
12826	Col4a1	collagen, type IV, alpha 1, transcript variant 1	NA	-0.0629	8.131426	-1.04456	0.1941971	0.514738
108168990	Gm46878	predicted gene, 46878, transcript variant X1	NA	-0.2356	2.061821	-1.17739	0.19423434	0.514753
68718	Rnf166	ring finger protein 166, transcript variant 2	NA	0.104578	4.541486	1.07518	0.19426596	0.514753
69786	Tprkb	Tp53rk binding protein, transcript variant 4	NA	0.083728	5.21367	1.059753	0.19432968	0.514838
13476	Reep5	receptor accessory protein 5	NA	0.058992	8.29837	1.041737	0.1945706	0.515308
102639358	Gm27197	predicted gene 27197, transcript variant X1	NA	-0.36256	0.16688	-1.2857	0.19458705	0.515308
22368	Trpv2	transient receptor potential cation channel, subfamily	NA	-0.13702	4.24826	-1.09963	0.19463937	0.515308
104184	Blmh	bleomycin hydrolase	NA	-0.06676	6.7741	-1.04736	0.19468345	0.515308
63959	Slc29a1	solute carrier family 29 (nucleoside transporters), me	NA	-0.10315	4.785004	-1.07412	0.19470581	0.515308
12168	Bmpr2	bone morphogenetic protein receptor, type II (serine/t	NA	0.080189	7.284939	1.057156	0.19470922	0.515308
66725	Lrrk2	leucine-rich repeat kinase 2	NA	0.208397	2.207639	1.155404	0.194748	0.515308
68949	Zfas1	zinc finger, NFX1-type containing 1, antisense RNA 1	NA	0.153447	4.513314	1.112224	0.19476059	0.515308
70408	Polr3f	polymerase (RNA) III (DNA directed) polypeptide F	NA	0.081178	5.093644	1.057882	0.19482469	0.515345
434147	D930028M14Rik	RIKEN cDNA D930028M14 gene, transcript variant 1	NA	-0.19563	2.393387	-1.14522	0.1948523	0.515345
17872	Ppp1r15a	protein phosphatase 1, regulatory subunit 15A	NA	0.139445	3.912838	1.101481	0.19486972	0.515345
622408	Mcidas	multiciliate differentiation and DNA synthesis associa	NA	-0.4119	0.246782	-1.33043	0.19501207	0.515579
74644	4930426D05Rik	RIKEN cDNA 4930426D05 gene	NA	-0.33089	0.612643	-1.25779	0.19504918	0.515579
14239	Foxs1	forkhead box S1	NA	0.357415	0.574633	1.281128	0.19505317	0.515579
54645	Gripap1	GRIP1 associated protein 1, transcript variant X27	NA	-0.08833	6.454602	-1.06314	0.19512699	0.515691
55935	Fnbp4	formin binding protein 4, transcript variant X3	NA	0.074544	6.96076	1.053028	0.19518427	0.515758
320751	D830014E11Rik	RIKEN cDNA D830014E11 gene, transcript variant X	NA	-0.49465	-0.14649	-1.40898	0.19534738	0.515852
207521	Dtx4	deltex 4, E3 ubiquitin ligase, transcript variant X1	NA	0.074631	7.107607	1.053092	0.19535468	0.515852
225432	Rbm27	RNA binding motif protein 27, transcript variant X4	NA	0.073457	5.83407	1.052235	0.1953927	0.515852
17274	Rab8a	RAB8A, member RAS oncogene family	NA	-0.07744	5.99336	-1.05515	0.19541291	0.515852
66714	4921524J17Rik	RIKEN cDNA 4921524J17 gene, transcript variant 1	NA	0.105815	4.769481	1.076102	0.1954448	0.515852
17751	Mt3	metallothionein 3	NA	0.152589	4.597742	1.111562	0.19545991	0.515852
666468	Atg4a	autophagy related 4A, cysteine peptidase, transcript	NA	0.15447	3.453807	1.113013	0.19547187	0.515852
104009	Qsox1	quiescin Q6 sulfhydryl oxidase 1, transcript variant 1	NA	-0.10181	4.987948	-1.07312	0.19550055	0.515852
14123	Fbrs	fibrosin	NA	-0.06774	6.437675	-1.04807	0.1955129	0.515852
13521	Slc26a2	solute carrier family 26 (sulfate transporter), member	NA	0.350029	0.711394	1.274586	0.19557009	0.515852
21335	Tacc3	transforming, acidic coiled-coil containing protein 3, tr	NA	-0.1007	5.037303	-1.0723	0.19561289	0.515852
241727	Snph	syntaphilin, transcript variant 3	NA	-0.08018	5.300882	-1.05715	0.19561967	0.515852
382034	Gse1	genetic suppressor element 1, coiled-coil protein, tra	NA	-0.08013	6.921043	-1.05711	0.19563184	0.515852
211556	Ap1ar	adaptor-related protein complex 1 associated regulat	NA	0.064212	6.543992	1.045513	0.19570543	0.515962
72578	2700054A10Rik	RIKEN cDNA 2700054A10 gene, transcript variant 1	NA	-0.12475	3.644581	-1.09032	0.19584316	0.516242
20280	Scp2	sterol carrier protein 2, liver	NA	0.064948	6.33804	1.046047	0.19589602	0.516297
75387	Sirt4	sirtuin 4, transcript variant X12	NA	-0.10567	4.322869	-1.07599	0.19608529	0.516713
664987	Gm14393	predicted gene 14393, transcript variant X2	NA	0.35462	1.733941	1.278649	0.19625583	0.517022
118568417	LOC118568417	uncharacterized LOC118568417	NA	-0.31366	1.136991	-1.24286	0.19631269	0.517022
68995	Mcts1	malignant T cell amplified sequence 1, transcript vari	NA	0.085625	5.703661	1.061147	0.19636791	0.517022
228911	Tshz2	teashirt zinc finger family member 2, transcript varian	NA	0.086973	6.51317	1.062139	0.19637193	0.517022
269470	Wdr3	WD repeat domain 3, transcript variant 1	NA	-0.08686	5.202429	-1.06205	0.19638312	0.517022
54524	Syt6	synaptotagmin VI, transcript variant X9	NA	0.087482	5.357886	1.062514	0.19645305	0.517022
319581	Xkr5	X-linked Kx blood group related 5, transcript variant X	NA	-0.33028	0.596122	-1.25726	0.1964545	0.517022
353234	Pcdha2	protocadherin alpha 2	NA	0.18193	4.189443	1.1344	0.19645687	0.517022
233549	Mogat2	monoacylglycerol O-acyltransferase 2	NA	-0.49731	-0.55453	-1.41157	0.19660502	0.517328
72518	2610307P16Rik	RIKEN cDNA 2610307P16 gene, transcript variant 1	NA	-0.22318	2.443547	-1.1673	0.19670138	0.517498
19183	Psmc3ip	proteasome (prosome, macropain) 26S subunit, ATP	NA	-0.13898	3.540617	-1.10113	0.19681377	0.517636

20743	Sptbn2	spectrin beta, non-erythrocytic 2	NA	-0.07256	8.08725	-1.05158	0.19683428	0.517636
231874	Ccz1	CCZ1 vacuolar protein trafficking and biogenesis ass	NA	0.075908	6.097747	1.054024	0.19684925	0.517636
237877	Atad5	ATPase family, AAA domain containing 5, transcript v	NA	0.109235	4.419003	1.078656	0.19694854	0.517734
68375	Ndufa8	NADH:ubiquinone oxidoreductase subunit A8	NA	0.075027	6.114296	1.053381	0.1969592	0.517734
13859	Eps15l1	epidermal growth factor receptor pathway substrate 1	NA	-0.06717	6.712903	-1.04766	0.19701529	0.517734
13489	Drd2	dopamine receptor D2	NA	-0.17353	3.198095	-1.12782	0.19704834	0.517734
56426	Pdcd10	programmed cell death 10	NA	0.086583	5.500165	1.061852	0.19707655	0.517734
69163	Mrpl44	mitochondrial ribosomal protein L44	NA	-0.11779	4.400381	-1.08507	0.19710596	0.517734
244421	Lonrf1	LON peptidase N-terminal domain and ring finger 1	NA	0.088514	5.313598	1.063275	0.19722165	0.517734
98878	Ehd4	EH-domain containing 4, transcript variant X1	NA	-0.13948	3.538866	-1.10151	0.19723045	0.517734
102635685	Gm32960	predicted gene, 32960, transcript variant 2	NA	0.388907	0.112785	1.309401	0.1972486	0.517734
242620	Dmrta2	doublesex and mab-3 related transcription factor like	NA	-0.1659	3.255181	-1.12187	0.19727204	0.517734
12122	Bid	BH3 interacting domain death agonist	NA	0.103169	4.387529	1.07413	0.19727763	0.517734
104175	Sbk1	SH3-binding kinase 1, transcript variant X1	NA	-0.06052	9.801721	-1.04284	0.19729124	0.517734
118567479	LOC118567479	uncharacterized LOC118567479	NA	0.336819	0.545091	1.262969	0.19730014	0.517734
68961	Phkg2	phosphorylase kinase, gamma 2 (testis), transcript va	NA	0.074346	5.469683	1.052883	0.19742333	0.517894
65255	Asb4	ankyrin repeat and SOCS box-containing 4, transcrip	NA	0.102111	4.364989	1.073343	0.19742492	0.517894
232933	Ccdc61	coiled-coil domain containing 61, transcript variant X1	NA	-0.13154	3.46685	-1.09546	0.19747159	0.517913
242691	Gpatch3	G patch domain containing 3	NA	-0.20383	2.297966	-1.15175	0.19752167	0.517913
66123	1110006O24Rik	RIKEN cDNA 1110006O24 gene	NA	0.439152	-0.0505	1.355807	0.19752761	0.517913
59052	Mettl9	methyltransferase like 9, transcript variant X2	NA	0.079181	7.29814	1.056418	0.19761804	0.518022
69528	1700030J22Rik	RIKEN cDNA 1700030J22 gene, transcript variant 1	NA	-0.17432	2.868193	-1.12843	0.19764021	0.518022
675812	Zfp605	zinc finger protein 605, transcript variant X2	NA	0.096859	5.032284	1.069442	0.19768981	0.518022
22612	Yes1	YES proto-oncogene 1, Src family tyrosine kinase, tra	NA	0.092018	4.930778	1.06586	0.19769643	0.518022
72775	Fance	Fanconi anemia, complementation group E, transcrip	NA	0.139329	3.72952	1.101393	0.19773525	0.518035
69632	Arhgef12	Rho guanine nucleotide exchange factor (GEF) 12, tr	NA	0.068439	7.820963	1.048581	0.19776519	0.518035
56079	Astn2	astrotactin 2, transcript variant 1	NA	0.098353	5.416423	1.07055	0.1978174	0.518041
17961	Nat2	N-acetyltransferase 2 (arylamine N-acetyltransferase	NA	0.330938	0.734468	1.257831	0.19786753	0.518041
270627	Taf1	TATA-box binding protein associated factor 1, transcr	NA	0.06792	6.168029	1.048204	0.19790832	0.518041
245038	Dclk3	doublecortin-like kinase 3	NA	0.158478	3.415849	1.116109	0.19791301	0.518041
232853	Zfp954	zinc finger protein 954	NA	-0.13546	4.090374	-1.09844	0.19794576	0.518041
54197	Rnf5	ring finger protein 5	NA	-0.07014	6.234417	-1.04982	0.1979646	0.518041
68763	1110038B12Rik	RIKEN cDNA 1110038B12 gene, transcript variant 1	NA	0.107497	4.816289	1.077358	0.19799035	0.518041
245607	Gprasp2	G protein-coupled receptor associated sorting protein	NA	0.072857	7.222536	1.051797	0.19819605	0.518496
329482	Dcdc5	doublecortin domain containing 5, transcript variant X	NA	-0.23457	1.468301	-1.17656	0.19842263	0.519005
67207	Lsm1	LSM1 homolog, mRNA degradation associated	NA	0.10066	4.926235	1.072264	0.1985225	0.519153
15414	Hoxb6	homeobox B6, transcript variant X1	NA	0.612437	1.122172	1.52884	0.19855372	0.519153
22379	Fmnl3	formin-like 3, transcript variant X2	NA	0.089767	4.855728	1.064199	0.1985746	0.519153
72938	Hspb11	heat shock protein family B (small), member 11, trans	NA	-0.19251	3.065147	-1.14275	0.1986892	0.519324
20516	Slc20a2	solute carrier family 20, member 2, transcript variant 1	NA	0.127974	4.668165	1.092758	0.1987039	0.519324
18969	Pola2	polymerase (DNA directed), alpha 2, transcript varian	NA	-0.12325	4.333555	-1.08918	0.19875261	0.519368
14979	H2-Ke6	H2-K region expressed gene 6	NA	-0.13198	4.316502	-1.09579	0.19886688	0.519583
67509	Saysd1	SAYSVFN motif domain containing 1	NA	-0.17051	2.730976	-1.12546	0.19891278	0.519616
72315	Ccdc74a	coiled-coil domain containing 74A, transcript variant X	NA	0.255354	1.332543	1.193628	0.19894326	0.519616
223770	Brd1	bromodomain containing 1	NA	-0.0641	6.73336	-1.04543	0.19904308	0.519793
118567768	LOC118567768	uncharacterized LOC118567768, transcript variant X	NA	0.291895	0.921649	1.224247	0.19909875	0.519855
56321	Aatf	apoptosis antagonizing transcription factor	NA	0.090509	5.448165	1.064746	0.19921475	0.520074
70884	Ccdc81	coiled-coil domain containing 81	NA	-0.36322	0.665147	-1.28629	0.19931494	0.520252
66667	Hspbap1	Hspb associated protein 1, transcript variant 1	NA	-0.1319	3.890428	-1.09573	0.19938772	0.520359
105244102	Gm13421	predicted gene 13421, transcript variant 2	NA	-0.18211	2.77895	-1.13454	0.19943688	0.520404
19358	Rad23a	RAD23 homolog A, nucleotide excision repair protein	NA	-0.09456	5.400602	-1.06774	0.19951641	0.520517
230648	Efcab14	EF-hand calcium binding domain 14, transcript variar	NA	-0.09427	4.734596	-1.06752	0.19954436	0.520517
227325	Dner	delta/notch-like EGF repeat containing	NA	0.070984	8.192878	1.050433	0.19967804	0.520775
24131	Ldb3	LIM domain binding 3, transcript variant X3	NA	-0.33808	2.316414	-1.26407	0.19970741	0.520775
13823	Epb41l3	erythrocyte membrane protein band 4.1 like 3, transc	NA	0.06865	6.728997	1.048735	0.19975749	0.520823
66687	Tbc1d15	TBC1 domain family, member 15, transcript variant X	NA	0.098162	5.015311	1.070409	0.19997087	0.521218
115490127	Gm10461	predicted gene 10461	NA	0.200735	2.4671	1.149283	0.20004467	0.521218
230676	Szt2	SZT2 subunit of KICSTOR complex	NA	-0.08547	5.650851	-1.06103	0.20005024	0.521218
27419	Naglu	alpha-N-acetylglucosaminidase (Sanfilippo disease II	NA	0.157278	3.167334	1.115181	0.20007175	0.521218
105245571	Gm41011	predicted gene, 41011	NA	-0.27933	1.067807	-1.21363	0.2000736	0.521218
68299	Vps53	VPS53 GARP complex subunit, transcript variant 1	NA	-0.06885	5.987586	-1.04888	0.2001014	0.521218
26382	Fgd2	FYVE, RhoGEF and PH domain containing 2, transcr	NA	0.254758	1.763099	1.193135	0.20013374	0.521219
11798	Xiap	X-linked inhibitor of apoptosis, transcript variant 4	NA	0.109499	6.524723	1.078854	0.20020397	0.521318
56401	P3h1	prolyl 3-hydroxylase 1, transcript variant 3	NA	-0.1195	4.169572	-1.08635	0.20026029	0.521382
214669	L3mbtl2	L3MBTL2 polycomb repressive complex 1 subunit, tra	NA	-0.09612	4.935902	-1.0689	0.20033622	0.521496
105244634	Gm12701	predicted gene 12701	NA	-0.33133	0.834558	-1.25817	0.20047022	0.521684
545758	Slc10a4-ps	solute carrier family 10 (sodium/bile acid cotransporte	NA	-0.33528	0.779556	-1.26163	0.20051353	0.521684
140740	Sec63	SEC63-like (S. cerevisiae), transcript variant 1	NA	0.070656	6.818675	1.050194	0.20053206	0.521684

16768	Lag3	lymphocyte-activation gene 3	NA	0.326547	1.833363	1.254009	0.20053669	0.521684
13110	Cyp2j6	cytochrome P450, family 2, subfamily j, polypeptide 6	NA	0.155776	3.289272	1.114021	0.20058855	0.521735
71913	Tmem79	transmembrane protein 79, transcript variant X2	NA	-0.61829	-0.38106	-1.53505	0.20066278	0.521845
381546	Ccdc24	coiled-coil domain containing 24, transcript variant X3	NA	-0.14714	3.826291	-1.10737	0.20069845	0.521854
100182	Akna	AT-hook transcription factor, transcript variant 2	NA	-0.16155	4.32489	-1.11849	0.20073441	0.521865
140577	Ankrd6	ankyrin repeat domain 6, transcript variant 3	NA	0.069879	5.896653	1.049629	0.20081121	0.521981
67295	Rab3c	RAB3C, member RAS oncogene family, transcript va	NA	0.068379	8.283633	1.048538	0.20092907	0.522204
20200	S100a6	S100 calcium binding protein A6 (calcyclin)	NA	0.219593	2.296538	1.164405	0.20112364	0.522603
108168876	Gm46809	predicted gene, 46809	NA	0.499266	-0.34601	1.413494	0.20115505	0.522603
278097	Armcx6	armadillo repeat containing, X-linked 6	NA	-0.08621	5.173585	-1.06158	0.20119702	0.522603
226976	Kansl3	KAT8 regulatory NSL complex subunit 3, transcript va	NA	-0.08378	6.335095	-1.05979	0.20121105	0.522603
68964	Ctc1	CTS telomere maintenance complex component 1, tr	NA	-0.076	6.431951	-1.05409	0.20124767	0.522614
20447	St6galnac3	ST6 (alpha-N-acetyl-neuraminyl-2,3-beta-galactosyl-1	NA	-0.10799	4.358435	-1.07773	0.20141775	0.522767
27528	Nrep	neuronal regeneration related protein, transcript varia	NA	0.064836	10.55292	1.045966	0.20141798	0.522767
76071	Jakmip1	janus kinase and microtubule interacting protein 1, tra	NA	0.081518	5.606222	1.058131	0.20148555	0.522767
328110	Prpf39	pre-mRNA processing factor 39, transcript variant 1	NA	0.07006	6.365479	1.04976	0.20148732	0.522767
234776	Atmin	ATM interactor, transcript variant X2	NA	0.085887	5.811981	1.06134	0.20150559	0.522767
74855	Fam228a	family with sequence similarity 228, member A, transc	NA	-0.33413	1.033658	-1.26062	0.20153446	0.522767
100113365	Nlgn4l	neuroligin 4-like, transcript variant X5	NA	-0.11524	5.611391	-1.08315	0.20159389	0.522767
18102	Nme1	NME/NM23 nucleoside diphosphate kinase 1, transcr	NA	0.059687	7.161748	1.04224	0.20163199	0.522767
12804	Cntfr	ciliary neurotrophic factor receptor, transcript variant 2	NA	-0.06586	6.140736	-1.04671	0.2016385	0.522767
22592	Ercc5	excision repair cross-complementing rodent repair de	NA	0.086677	5.589992	1.061921	0.20165404	0.522767
68304	Poglut3	protein O-glucosyltransferase 3	NA	-0.12629	3.493822	-1.09148	0.20165977	0.522767
74011	Slc25a27	solute carrier family 25, member 27, transcript variant	NA	0.071308	6.900894	1.050669	0.20179813	0.523001
115486436	LOC115486436	uncharacterized LOC115486436	NA	0.228702	1.610526	1.171781	0.20182585	0.523001
227102	Ormdl1	ORM1-like 1 (S. cerevisiae)	NA	-0.09233	4.838671	-1.06609	0.20190003	0.523001
115488787	Gm52298	predicted gene, 52298	NA	0.430887	1.162317	1.348062	0.20191827	0.523001
78914	Nadsyn1	NAD synthetase 1, transcript variant 3	NA	-0.18445	2.470127	-1.13638	0.2019498	0.523001
15441	Hp1bp3	heterochromatin protein 1, binding protein 3, transcrip	NA	0.06089	8.278677	1.043109	0.20198214	0.523001
327826	Frs2	fibroblast growth factor receptor substrate 2, transcrip	NA	0.089289	6.053143	1.063845	0.20199908	0.523001
319953	Ttll1	tubulin tyrosine ligase-like 1, transcript variant 1	NA	0.07213	6.253834	1.051268	0.2020237	0.523001
101197	Zfp956	zinc finger protein 956	NA	0.1133	4.688783	1.081699	0.20203946	0.523001
16832	Ldhb	lactate dehydrogenase B, transcript variant 1	NA	0.061816	7.31547	1.043779	0.20216271	0.523117
118567980	LOC118567980	uncharacterized LOC118567980	NA	-0.11312	3.975824	-1.08156	0.20217072	0.523117
320560	Dennd5b	DENN/MADD domain containing 5B, transcript variar	NA	0.084824	6.327597	1.060559	0.20218065	0.523117
105246931	Gm42125	predicted gene, 42125	NA	-0.45821	-0.27407	-1.37384	0.20226119	0.523124
18218	Dusp8	dual specificity phosphatase 8	NA	-0.05634	7.583431	-1.03982	0.20227592	0.523124
19731	Rgl1	ral guanine nucleotide dissociation stimulator,-like 1,	NA	-0.10388	4.89219	-1.07466	0.20227985	0.523124
66493	Mrpl51	mitochondrial ribosomal protein L51	NA	-0.0874	5.078095	-1.06246	0.20235706	0.523241
67306	Zc2hc1a	zinc finger, C2HC-type containing 1A	NA	0.065971	6.735913	1.046789	0.20248469	0.523454
20054	Rps15	ribosomal protein S15, transcript variant 2	NA	0.069655	8.426812	1.049465	0.20250404	0.523454
236900	Pdk3	pyruvate dehydrogenase kinase, isoenzyme 3	NA	-0.09903	4.690503	-1.07105	0.20261086	0.523647
107723	Slc12a6	solute carrier family 12, member 6, transcript variant	NA	0.068965	6.252466	1.048964	0.20270488	0.523807
17254	Slc3a2	solute carrier family 3 (activators of dibasic and neutr	NA	0.061404	6.773398	1.04348	0.20282943	0.524046
320713	Mysm1	myb-like, SWIRM and MPN domains 1, transcript var	NA	0.093074	5.011231	1.06664	0.20289281	0.524126
72236	Tsnaxip1	translin-associated factor X (Tsnax) interacting protei	NA	-0.51486	-0.16488	-1.42885	0.20297424	0.524253
353502	Hcfc1r1	host cell factor C1 regulator 1 (XPO1-dependent), tra	NA	0.109419	5.129942	1.078794	0.20307005	0.524381
338372	Map3k9	mitogen-activated protein kinase kinase kinase 9, tra	NA	0.086441	6.92618	1.061748	0.20308812	0.524381
69553	Ripor3	RIPOR family member 3, transcript variant 2	NA	-0.40035	0.150796	-1.31983	0.20323233	0.524634
382562	Pfn4	profilin family, member 4, transcript variant 1	NA	0.201536	2.955195	1.149922	0.20325037	0.524634
57278	Bcam	basal cell adhesion molecule	NA	-0.13064	3.843464	-1.09478	0.20331644	0.524682
13448	Dok1	docking protein 1, transcript variant 1	NA	-0.29478	0.961715	-1.2267	0.20336716	0.524682
21907	Nr2e1	nuclear receptor subfamily 2, group E, member 1	NA	-0.12494	4.679264	-1.09046	0.20337152	0.524682
78977	Popdc3	popeye domain containing 3, transcript variant X1	NA	0.440585	-0.15115	1.357154	0.20339803	0.524682
14450	Gart	phosphoribosylglycinamide formyltransferase, transcr	NA	-0.08245	5.878418	-1.05881	0.20354221	0.524971
214627	Tent4b	terminal nucleotidyltransferase 4B, transcript variant 4	NA	0.093724	6.040251	1.067121	0.20363195	0.525039
93837	Dach2	dachshund family transcription factor 2, transcript var	NA	0.153644	3.399812	1.112375	0.20364752	0.525039
105975	AU022754	expressed sequence AU022754	NA	0.360733	0.550155	1.284078	0.20369643	0.525039
11907	Ate1	arginyltransferase 1, transcript variant 1	NA	0.074858	6.176923	1.053258	0.20370896	0.525039
17119	Mxd1	MAX dimerization protein 1	NA	-0.07916	5.210025	-1.0564	0.20373027	0.525039
115487814	LOC115487814	uncharacterized LOC115487814	NA	0.194123	2.795833	1.144029	0.20379108	0.525055
272551	Gins2	GINS complex subunit 2 (Psf2 homolog)	NA	-0.17303	2.769485	-1.12743	0.20380094	0.525055
22156	Tuft1	tuftelin 1, transcript variant 2	NA	0.176938	2.492464	1.130482	0.20388611	0.525192
67685	Dnaaf4	dynein axonemal assembly factor 4, transcript variant	NA	-0.33764	1.559639	-1.26368	0.2039521	0.525224
59058	Bhlhe22	basic helix-loop-helix family, member e22	NA	-0.10621	6.722337	-1.0764	0.20399693	0.525224
233879	Asphd1	aspartate beta-hydroxylase domain containing 1, tran	NA	0.171526	3.08012	1.126249	0.20400738	0.525224
66591	Mad2l1bp	MAD2L1 binding protein	NA	0.170381	3.415365	1.125355	0.20404007	0.525224
12867	Cox7c	cytochrome c oxidase subunit 7C	NA	0.088392	5.651005	1.063184	0.20406107	0.525224

16985	Lsp1	lymphocyte specific 1, transcript variant X3	NA	0.20074	2.202482	1.149288	0.20409223	0.525224
100034675	Gm11335	predicted gene 11335, transcript variant 1	NA	-0.32563	0.51379	-1.25321	0.20413444	0.525249
19170	Psmb1	proteasome (prosome, macropain) subunit, beta type	NA	0.067464	7.028997	1.047873	0.20419339	0.525255
14871	Gstt1	glutathione S-transferase, theta 1, transcript variant 1	NA	0.232545	2.164785	1.174905	0.20420129	0.525255
27273	Pdk4	pyruvate dehydrogenase kinase, isoenzyme 4	NA	0.246077	1.459567	1.185978	0.20432513	0.525491
19378	Aldh1a2	aldehyde dehydrogenase family 1, subfamily A2	NA	0.132324	4.067591	1.096058	0.20436393	0.525507
67457	Frmd8	FERM domain containing 8, transcript variant X1	NA	-0.10983	4.112376	-1.0791	0.20451	0.5258
223754	Tbc1d22a	TBC1 domain family, member 22a, transcript variant	NA	-0.12157	4.100464	-1.08792	0.20459259	0.525898
56445	Dnaja2	DnaJ heat shock protein family (Hsp40) member A2	NA	0.059726	7.450676	1.042268	0.20463217	0.525898
56264	Cpxm1	carboxypeptidase X 1 (M14 family)	NA	0.110297	4.472318	1.079451	0.20467791	0.525898
22064	Trpc2	transient receptor potential cation channel, subfamily	NA	-0.1902	2.529992	-1.14092	0.20467974	0.525898
76022	Gon4l	gon-4-like (C.elegans), transcript variant X4	NA	-0.08261	6.630852	-1.05893	0.20478983	0.525898
19649	Robo3	roundabout guidance receptor 3	NA	-0.17668	3.240133	-1.13028	0.20480552	0.525898
70240	Ufsp1	UFM1-specific peptidase 1	NA	0.171901	2.701513	1.126542	0.20483353	0.525898
105243877	Gm39603	predicted gene, 39603, transcript variant X2	NA	-0.21254	2.037673	-1.15873	0.20485682	0.525898
74204	Xpo6	exportin 6, transcript variant X3	NA	0.065704	6.786246	1.046595	0.20486651	0.525898
26569	Slc27a4	solute carrier family 27 (fatty acid transporter), memb	NA	-0.07305	6.509446	-1.05194	0.20487128	0.525898
67393	Cxxc5	CXXC finger 5, transcript variant X2	NA	0.076867	6.138659	1.054725	0.20493372	0.525907
67331	Atp8b3	ATPase, class I, type 8B, member 3, transcript variar	NA	-0.3719	0.314793	-1.29406	0.20495591	0.525907
227195	Ino80d	INO80 complex subunit D, transcript variant 1	NA	0.078037	5.761303	1.055581	0.2049826	0.525907
17420	Mnat1	menage a trois 1, transcript variant X2	NA	-0.11556	4.015863	-1.0834	0.20500446	0.525907
13107	Cyp2f2	cytochrome P450, family 2, subfamily f, polypeptide 2	NA	-0.64177	-0.40451	-1.56024	0.20510214	0.525907
19664	Rbpj	recombination signal binding protein for immunoglobu	NA	-0.06831	6.761387	-1.04849	0.20528808	0.525907
15512	Hspa2	heat shock protein 2, transcript variant 1	NA	0.211262	2.305856	1.1577	0.20529119	0.525907
320150	Zdhhc17	zinc finger, DHHC domain containing 17, transcript va	NA	0.08028	6.464839	1.057223	0.20529731	0.525907
20183	Rxrg	retinoid X receptor gamma, transcript variant 1	NA	0.153475	4.022172	1.112245	0.20530004	0.525907
12873	Cpa3	carboxypeptidase A3, mast cell	NA	-0.78878	0.474627	-1.72761	0.20531997	0.525907
11302	Aatk	apoptosis-associated tyrosine kinase, transcript varia	NA	-0.06919	6.497186	-1.04913	0.2053318	0.525907
98758	Hnrnpf	heterogeneous nuclear ribonucleoprotein F, transcrip	NA	-0.06314	8.195392	-1.04474	0.20534021	0.525907
14660	Gls	glutaminase, transcript variant X5	NA	0.072382	7.23525	1.051451	0.20534749	0.525907
66859	Slc16a9	solute carrier family 16 (monocarboxylic acid transpo	NA	0.208	2.142799	1.155086	0.20538181	0.525907
67602	Necap1	NECAP endocytosis associated 1, transcript variant 1	NA	0.061906	6.620964	1.043844	0.20541507	0.525907
58198	Sall1	spalt like transcription factor 1, transcript variant X1	NA	-0.10632	5.452098	-1.07648	0.20542409	0.525907
74438	Clvs1	clavesin 1, transcript variant X3	NA	0.083586	6.040502	1.059648	0.20542443	0.525907
71966	Nkiras2	NFKB inhibitor interacting Ras-like protein 2	NA	-0.0738	5.854367	-1.05249	0.20554243	0.526127
102633836	Gm31566	predicted gene, 31566, transcript variant X3	NA	0.305028	0.85028	1.235443	0.20561485	0.526229
140481	Man2a2	mannosidase 2, alpha 2, transcript variant X2	NA	-0.06374	6.625509	-1.04517	0.20570413	0.526296
66508	Lamtor1	late endosomal/lysosomal adaptor, MAPK and MTOF	NA	0.077417	5.658556	1.055128	0.20571134	0.526296
332131	Krt78	keratin 78	NA	-0.83449	-1.60315	-1.78322	0.20573795	0.526296
72657	Selenoh	selenoprotein H, transcript variant 1	NA	-0.11383	5.359183	-1.08209	0.20580852	0.52636
68982	1500015A07Rik	RIKEN cDNA 1500015A07 gene	NA	0.188495	2.79462	1.139574	0.20582776	0.52636
72323	Asb6	ankyrin repeat and SOCS box-containing 6	NA	-0.11052	4.385725	-1.07962	0.20590728	0.526402
17936	Nab1	Ngfi-A binding protein 1, transcript variant X2	NA	0.085706	5.237657	1.061207	0.20590875	0.526402
102632048	Gm30223	predicted gene, 30223	NA	-0.25094	1.524041	-1.18998	0.20613979	0.526903
118567893	LOC118567893	MLV-related proviral Env polyprotein	NA	0.251664	2.916418	1.19058	0.20618689	0.526903
66628	Thg1l	tRNA-histidine guanylyltransferase 1-like (S. cerevisia	NA	-0.12417	3.730049	-1.08988	0.20622751	0.526903
100417675	Nlrp5-ps	NLR family, pyrin domain containing 5, pseudogene,	NA	-0.18911	2.478489	-1.14006	0.20625445	0.526903
240095	H2-M5	histocompatibility 2, M region locus 5, transcript varia	NA	0.110723	4.391814	1.079769	0.20628548	0.526903
56031	Ppie	peptidylprolyl isomerase E (cyclophilin E)	NA	-0.11442	4.328579	-1.08254	0.20629921	0.526903
67877	Naa20	N(alpha)-acetyltransferase 20, NatB catalytic subunit	NA	0.085984	5.321403	1.061411	0.20640167	0.527082
75659	Wdr54	WD repeat domain 54, transcript variant 1	NA	0.154998	2.818096	1.11342	0.2065602	0.527404
79201	Tnfrsf23	tumor necrosis factor receptor superfamily, member 2	NA	-0.26368	1.273149	-1.20054	0.20665055	0.527552
102423	Hinfp	histone H4 transcription factor, transcript variant X12	NA	-0.07399	5.520294	-1.05263	0.20670039	0.527597
72386	2610035D17Rik	RIKEN cDNA 2610035D17 gene	NA	-0.13169	3.945226	-1.09558	0.20676114	0.527669
217353	Tmc6	transmembrane channel-like gene family 6, transcript	NA	-0.29947	1.345399	-1.23069	0.20683504	0.527775
12111	Bgn	biglycan	NA	-0.10264	6.170645	-1.07374	0.20687337	0.52779
72350	Zc2hc1c	zinc finger, C2HC-type containing 1C	NA	0.329483	1.222698	1.256563	0.2069123	0.527806
118567871	LOC118567871	uncharacterized LOC118567871	NA	0.162301	3.122869	1.11907	0.20698268	0.52789
235323	Usp28	ubiquitin specific peptidase 28, transcript variant 1	NA	-0.08508	5.247356	-1.06074	0.2070099	0.52789
68705	Gtf2f2	general transcription factor IIF, polypeptide 2	NA	0.089565	4.851365	1.064049	0.20715725	0.528183
18845	Plxna2	plexin A2	NA	-0.08168	8.019276	-1.05825	0.20722129	0.528263
105247205	Gm42344	predicted gene, 42344	NA	-0.38621	0.687151	-1.30695	0.20734427	0.528494
105243235	Gm26721	predicted gene, 26721	NA	0.223503	1.586268	1.167565	0.20739887	0.52851
230379	Acer2	alkaline ceramidase 2, transcript variant X5	NA	0.125845	4.252499	1.091146	0.20741564	0.52851
66801	Prkrip1	Prkr interacting protein 1 (IL11 inducible)	NA	0.109245	4.227724	1.078663	0.20752359	0.528703
99349	Dnajc24	DnaJ heat shock protein family (Hsp40) member C24	NA	0.141418	3.023546	1.102989	0.20755798	0.528707
14461	Gata2	GATA binding protein 2, transcript variant 2	NA	-0.14416	3.445797	-1.10509	0.20761089	0.528759
16650	Kpna6	karyopherin (importin) alpha 6	NA	-0.07661	6.542724	-1.05454	0.20776212	0.529062

235442	Rab8b	RAB8B, member RAS oncogene family	NA	0.072718	6.069023	1.051696	0.20780216	0.529081
75578	Fggy	FGGY carbohydrate kinase domain containing, transc	NA	-0.20149	2.017144	-1.14988	0.20787013	0.529171
72823	Pard3b	par-3 family cell polarity regulator beta, transcript vari	NA	0.130763	3.565036	1.094873	0.20790945	0.529189
17997	Nedd1	neural precursor cell expressed, developmentally dov	NA	-0.13143	3.902912	-1.09538	0.20816484	0.529756
218454	Lhfpl2	lipoma HMGIC fusion partner-like 2, transcript variant	NA	0.088374	5.075619	1.063171	0.20824703	0.529758
53607	Snrpa	small nuclear ribonucleoprotein polypeptide A, transc	NA	-0.07098	6.654882	-1.05043	0.20825397	0.529758
15473	Rida	reactive intermediate imine deaminase A homolog	NA	-0.13581	3.193044	-1.09871	0.20826338	0.529758
102639713	Gm14421	predicted gene 14421	NA	0.362501	0.825822	1.285652	0.20837464	0.529958
234865	Nup133	nucleoporin 133	NA	0.093007	5.301791	1.066591	0.20841276	0.529972
75530	Lyrm7	LYR motif containing 7, transcript variant 1	NA	-0.18962	3.261152	-1.14047	0.20844909	0.529982
102634490	Gm32061	predicted gene, 32061	NA	-0.46116	-0.08319	-1.37664	0.20857126	0.53021
70560	Wars2	tryptophanyl tRNA synthetase 2 (mitochondrial)	NA	-0.13874	3.337696	-1.10094	0.20863261	0.530283
24066	Spry4	sprouty RTK signaling antagonist 4	NA	0.138424	3.596523	1.100702	0.2086657	0.530284
20356	Sema5a	sema domain, seven thrombospondin repeats (type 1	NA	-0.07314	5.824615	-1.052	0.20873504	0.530377
217303	Cd300a	CD300A molecule, transcript variant 1	NA	-0.35121	0.321703	-1.27563	0.208992	0.530947
100038847	Gm10406	predicted gene 10406	NA	0.136808	4.294532	1.09947	0.20910958	0.531131
100502982	BC037704	cDNA sequence BC037704	NA	0.467426	0.071848	1.38264	0.20916176	0.531131
57312	Mrps31	mitochondrial ribosomal protein S31	NA	0.119124	4.255807	1.086075	0.20917035	0.531131
100043766	Gm14057	predicted gene 14057	NA	0.160609	3.124451	1.117759	0.20920406	0.531131
194401	Mical3	microtubule associated monooxygenase, calponin an	NA	-0.09716	6.901746	-1.06966	0.20922758	0.531131
268932	Caskin1	CASK interacting protein 1, transcript variant X5	NA	-0.06478	7.113072	-1.04592	0.20930431	0.531139
56443	Arpc1a	actin related protein 2/3 complex, subunit 1A	NA	0.057641	7.339063	1.040762	0.20930982	0.531139
668894	Vinac1	vinculin/alpha-catenin family member 1, transcript vai	NA	0.340787	0.954549	1.266447	0.209343	0.531139
55984	Camkk1	calcium/calmodulin-dependent protein kinase kinase	NA	-0.103	4.872154	-1.07401	0.20938363	0.531139
11535	Adm	adrenomedullin	NA	0.449553	1.036284	1.365618	0.20942069	0.531139
212541	Rho	rhodopsin, transcript variant X1	NA	-0.49524	0.233053	-1.40955	0.20942646	0.531139
217262	Abca9	ATP-binding cassette, sub-family A (ABC1), member	NA	-0.11869	3.794055	-1.08574	0.20950582	0.53122
19277	Ptpro	protein tyrosine phosphatase, receptor type, O, trans	NA	0.06136	6.942822	1.043449	0.20955391	0.53122
107869	Cth	cystathionase (cystathionine gamma-lyase)	NA	-0.27736	0.883994	-1.21197	0.20955618	0.53122
51938	Ccdc39	coiled-coil domain containing 39, transcript variant X5	NA	0.128486	4.32883	1.093146	0.20962975	0.531323
68028	Rpl22l1	ribosomal protein L22 like 1, transcript variant 1	NA	-0.07319	5.647183	-1.05204	0.20973814	0.531471
72556	Zfp566	zinc finger protein 566	NA	0.137782	3.865605	1.100212	0.2097534	0.531471
14083	Ptk2	PTK2 protein tyrosine kinase 2, transcript variant X48	NA	0.071349	6.680092	1.050698	0.20983939	0.531606
18655	Pgk1	phosphoglycerate kinase 1	NA	0.082151	7.963345	1.058595	0.20990834	0.531698
64450	Gpr85	G protein-coupled receptor 85, transcript variant X7	NA	0.068626	6.906639	1.048717	0.20997015	0.531772
76863	Dcun1d5	DCN1, defective in cullin neddylation 1, domain conta	NA	0.083119	6.368343	1.059306	0.21012964	0.532082
118567336	LOC118567336	uncharacterized LOC118567336	NA	-0.27775	1.411468	-1.2123	0.21015814	0.532082
69327	1700007K13Rik	RIKEN cDNA 1700007K13 gene	NA	0.326587	0.568408	1.254043	0.21030156	0.532283
19134	Prpf4b	pre-mRNA processing factor 4B, transcript variant 1	NA	0.072809	7.222639	1.051763	0.21030265	0.532283
24060	Slc35a1	solute carrier family 35 (CMP-sialic acid transporter),	NA	0.083346	5.116888	1.059472	0.21038504	0.532336
16691	Krt8	keratin 8	NA	-0.28885	1.839696	-1.22166	0.21038906	0.532336
14265	Fmr1	FMRP translational regulator 1, transcript variant 3	NA	0.079576	6.485607	1.056707	0.21050442	0.532545
67229	Prpf18	pre-mRNA processing factor 18, transcript variant 2	NA	-0.0656	6.026542	-1.04652	0.21058704	0.532643
66932	Rexo1	REX1, RNA exonuclease 1	NA	-0.06764	6.389276	-1.048	0.21062588	0.532643
218461	Pde8b	phosphodiesterase 8B, transcript variant 6	NA	0.145344	3.201278	1.105994	0.21064162	0.532643
110829	Lims1	LIM and senescent cell antigen-like domains 1, trans	NA	0.07698	5.760962	1.054808	0.21070194	0.532713
52076	Tmem38b	transmembrane protein 38B	NA	0.146506	3.432712	1.106886	0.21074163	0.532731
12894	Cpt1a	carnitine palmitoyltransferase 1a, liver, transcript vari	NA	0.081491	4.933897	1.058111	0.2108565	0.532938
229011	Samd10	sterile alpha motif domain containing 10, transcript va	NA	-0.07139	6.418162	-1.05073	0.21093611	0.533057
226407	Rab3gap1	RAB3 GTPase activating protein subunit 1	NA	0.090888	5.171935	1.065026	0.21103743	0.53323
239839	Ccdc14	coiled-coil domain containing 14	NA	-0.16058	3.398709	-1.11773	0.21134254	0.533915
18247	Oaz2	ornithine decarboxylase antizyme 2, transcript varian	NA	0.055945	7.443256	1.03954	0.21139502	0.533915
22214	Ube2h	ubiquitin-conjugating enzyme E2H, transcript variant	NA	-0.07146	7.451197	-1.05078	0.21144595	0.533915
67917	Zcchc3	zinc finger, CCHC domain containing 3	NA	-0.09571	4.764679	-1.06859	0.21144683	0.533915
235130	Adamts15	a disintegrin-like and metallopeptidase (reprolysin typ	NA	0.123347	3.985682	1.089259	0.21147249	0.533915
16367	Irs1	insulin receptor substrate 1	NA	0.072163	6.300529	1.051291	0.21152483	0.533964
67503	1700001G17Rik	RIKEN cDNA 1700001G17 gene	NA	-0.39964	0.036581	-1.31918	0.21164239	0.534108
12631	Cfl1	cofilin 1, non-muscle	NA	0.057769	10.13286	1.040855	0.21164752	0.534108
26444	Psma7	proteasome subunit alpha 7, transcript variant 1	NA	0.087955	6.966044	1.062863	0.21176988	0.534269
54607	Socs6	suppressor of cytokine signaling 6	NA	-0.07989	5.049827	-1.05694	0.21177684	0.534269
15245	Hhip	Hedgehog-interacting protein	NA	0.207169	2.056188	1.154421	0.21186428	0.534338
54352	Irx5	Iroquois homeobox 5	NA	0.165093	3.582349	1.121238	0.21189001	0.534338
209416	Gpkow	G patch domain and KOW motifs	NA	0.070766	5.744966	1.050274	0.21193608	0.534338
107732	Mrpl10	mitochondrial ribosomal protein L10, transcript varian	NA	0.090326	5.076334	1.064611	0.21194087	0.534338
23971	Papss1	3'-phosphoadenosine 5'-phosphosulfate synthase 1,	NA	0.064673	6.845003	1.045848	0.21199025	0.534338
68366	Tmem129	transmembrane protein 129, transcript variant 1	NA	-0.10916	4.043825	-1.0786	0.2120464	0.534338
17279	Melk	maternal embryonic leucine zipper kinase, transcript	NA	-0.13772	3.858512	-1.10017	0.21206693	0.534338
218989	Tmem260	transmembrane protein 260, transcript variant 1	NA	0.094002	4.469356	1.067327	0.21211541	0.534338

232440	H2aj	H2J.A histone	NA	-0.09893	4.789862	-1.07098	0.21211581	0.534338
11596	Ager	advanced glycosylation end product-specific receptor	NA	0.246396	2.181299	1.18624	0.21213291	0.534338
319604	Fam168a	family with sequence similarity 168, member A, transc	NA	0.065399	8.022093	1.046374	0.21224291	0.534493
66496	Ppdpf	pancreatic progenitor cell differentiation and prolifera	NA	-0.0884	5.993076	-1.06319	0.21228262	0.534493
230157	Tmeff1	transmembrane protein with EGF-like and two follista	NA	0.072124	8.178938	1.051263	0.2123134	0.534493
74841	Usp38	ubiquitin specific peptidase 38, transcript variant X1	NA	0.086012	5.323672	1.061432	0.21232579	0.534493
102635561	Ggnbp2os	gametogenetin binding protein 2, opposite strand, tra	NA	0.219747	1.631512	1.16453	0.21244188	0.53462
14658	Glrb	glycine receptor, beta subunit, transcript variant 5	NA	0.09035	5.049578	1.064628	0.21245408	0.53462
11600	Angpt1	angiopoietin 1, transcript variant X1	NA	0.123063	3.684144	1.089044	0.21248486	0.53462
67695	Ost4	oligosaccharyltransferase complex subunit 4 (non-cat	NA	-0.1011	4.936392	-1.07259	0.21252053	0.53462
12018	Bak1	BCL2-antagonist/killer 1, transcript variant 1	NA	0.090461	5.252689	1.06471	0.21254047	0.53462
105859	Csdc2	cold shock domain containing C2, RNA binding	NA	0.084055	7.021968	1.059993	0.21290421	0.535394
23992	Prkra	protein kinase, interferon inducible double stranded R	NA	0.06794	6.08048	1.048219	0.21291392	0.535394
18072	Nhlh2	nescient helix loop helix 2, transcript variant X1	NA	-0.07502	5.394235	-1.05337	0.21295835	0.535423
67273	Ndufa10	NADH:ubiquinone oxidoreductase subunit A10	NA	0.071083	6.428264	1.050505	0.21301052	0.535472
268595	D430019H16Rik	RIKEN cDNA D430019H16 gene	NA	0.059167	8.318716	1.041864	0.21306506	0.535519
66660	Sltm	SAFB-like, transcription modulator, transcript variant	NA	0.069257	6.953705	1.049176	0.21309521	0.535519
19039	Lgals3bp	lectin, galactoside-binding, soluble, 3 binding protein	NA	0.235746	2.152873	1.177515	0.21319068	0.535652
237911	Brip1	BRCA1 interacting protein C-terminal helicase 1	NA	-0.20009	2.107664	-1.14877	0.21323051	0.535652
102635605	Gm32896	predicted gene, 32896	NA	0.465095	0.01132	1.380408	0.21332991	0.535652
64659	Mrps14	mitochondrial ribosomal protein S14, transcript variar	NA	-0.11268	4.302309	-1.08124	0.21334293	0.535652
14700	Gng10	guanine nucleotide binding protein (G protein), gamm	NA	0.076704	5.925948	1.054606	0.21334544	0.535652
19699	Reln	reelin, transcript variant 1	NA	0.06592	7.725338	1.046752	0.21334571	0.535652
105428	Fam149b	family with sequence similarity 149, member B, transc	NA	-0.07917	5.640976	-1.05641	0.21341449	0.535742
19762	Rit2	Ras-like without CAAX 2	NA	0.072841	5.365535	1.051786	0.21346238	0.535751
74653	Pomk	protein-O-mannose kinase	NA	-0.08449	4.944496	-1.06032	0.21348402	0.535751
17242	Mdk	midkine, transcript variant X1	NA	-0.08429	6.025581	-1.06017	0.21368855	0.536182
233147	Zfp939	zinc finger protein 939, transcript variant X15	NA	-0.10652	4.368737	-1.07663	0.21384571	0.536428
26922	Mecr	mitochondrial trans-2-enoyl-CoA reductase	NA	-0.14231	3.767102	-1.10367	0.2138525	0.536428
332937	Tfap2e	transcription factor AP-2, epsilon	NA	0.307388	1.044666	1.237465	0.21389629	0.536455
231717	Pheta1	PH domain containing endocytic trafficking adaptor 1	NA	-0.09822	4.996997	-1.07045	0.21400633	0.536606
328330	D130037M23Rik	RIKEN cDNA D130037M23 gene	NA	0.297311	1.027236	1.228852	0.21402253	0.536606
68667	Trpm4	transient receptor potential cation channel, subfamily	NA	0.13603	3.509229	1.098877	0.21406124	0.53662
102294	Cyp4v3	cytochrome P450, family 4, subfamily v, polypeptide 3	NA	0.258811	1.536513	1.196492	0.21409378	0.53662
100040591	Kcnj13	potassium inwardly-rectifying channel, subfamily J, m	NA	0.218433	1.987701	1.163469	0.21415673	0.536695
226040	Tmem252	transmembrane protein 252	NA	0.302172	1.484604	1.232999	0.21419345	0.536704
66690	Tmem186	transmembrane protein 186, transcript variant 1	NA	-0.09493	4.889507	-1.06801	0.21428829	0.536844
320997	Cyp4f39	cytochrome P450, family 4, subfamily f, polypeptide 3	NA	-0.50223	-0.31962	-1.4164	0.21431515	0.536844
26384	Gnpda1	glucosamine-6-phosphate deaminase 1	NA	-0.13471	4.208659	-1.09787	0.21436285	0.53688
68920	1110065P20Rik	RIKEN cDNA 1110065P20 gene, transcript variant 2	NA	0.133267	3.966178	1.096774	0.21460002	0.537392
215449	Rap1b	RAS related protein 1b	NA	0.06392	6.958852	1.045302	0.21467243	0.537475
108169181	LOC108169181	uncharacterized LOC108169181	NA	0.092819	7.011985	1.066452	0.21473915	0.537475
234069	Pcid2	PCI domain containing 2	NA	-0.07508	5.443998	-1.05342	0.21475407	0.537475
225870	Rin1	Ras and Rab interactor 1, transcript variant 3	NA	-0.23287	1.565139	-1.17517	0.21477838	0.537475
229503	Rrnad1	ribosomal RNA adenine dimethylase domain containi	NA	-0.0898	5.086603	-1.06422	0.21482789	0.537475
246229	Bivm	basic, immunoglobulin-like variable motif containing, t	NA	0.092804	5.411711	1.066441	0.21484453	0.537475
68385	Tlcd1	TLC domain containing 1, transcript variant 1	NA	0.200186	2.13618	1.148847	0.21487139	0.537475
259302	Srgap3	SLIT-ROBO Rho GTPase activating protein 3	NA	0.08076	7.675292	1.057575	0.21493826	0.537475
56350	Arl3	ADP-ribosylation factor-like 3, transcript variant 1	NA	-0.08034	6.241309	-1.05726	0.21497491	0.537475
58239	Dexi	dexamethasone-induced transcript	NA	0.140355	3.526151	1.102176	0.21499591	0.537475
69770	Fam174c	family with sequence similarity 174, member C	NA	-0.18852	2.024529	-1.1396	0.2149968	0.537475
268739	Arhgef40	Rho guanine nucleotide exchange factor (GEF) 40, tr	NA	-0.06829	5.867367	-1.04848	0.21515184	0.537575
75212	Rnf121	ring finger protein 121, transcript variant 1	NA	-0.08671	4.874191	-1.06195	0.21518344	0.537575
382427	Best3	bestrophin 3, transcript variant X3	NA	-0.43481	0.192749	-1.35173	0.21518881	0.537575
331392	Gm5124	predicted pseudogene 5124	NA	-0.09029	4.506299	-1.06458	0.21524137	0.537575
330064	Slc5a6	solute carrier family 5 (sodium-dependent vitamin tra	NA	0.0948	4.60638	1.067918	0.21524975	0.537575
102638940	Gm19605	predicted gene, 19605, transcript variant 1	NA	0.315611	0.916286	1.244538	0.21529065	0.537575
238799	Tnpo1	transportin 1, transcript variant X3	NA	0.067624	7.419572	1.047989	0.21531486	0.537575
217026	Heatr6	HEAT repeat containing 6	NA	-0.07283	5.608549	-1.05178	0.21531612	0.537575
21834	Thrb	thyroid hormone receptor beta, transcript variant X15	NA	0.226831	1.482496	1.170262	0.21536563	0.537575
170738	Kcnh7	potassium voltage-gated channel, subfamily H (eag-r	NA	0.103424	5.274636	1.07432	0.21536686	0.537575
58172	Sertad2	SERTA domain containing 2, transcript variant X1	NA	0.081314	5.177131	1.057982	0.21542399	0.537635
13852	Stx2	syntaxin 2, transcript variant 1	NA	-0.0864	4.736547	-1.06172	0.21552246	0.537798
68799	Rgmb	repulsive guidance molecule family member B, transc	NA	-0.05624	7.6018	-1.03975	0.21558028	0.53786
55951	Mpc1	mitochondrial pyruvate carrier 1, transcript variant 1	NA	0.100827	6.061925	1.072388	0.21579434	0.538233
319765	Igf2bp2	insulin-like growth factor 2 mRNA binding protein 2	NA	-0.09146	5.494002	-1.06545	0.2157979	0.538233
22590	Xpa	xeroderma pigmentosum, complementation group A	NA	0.164142	3.672928	1.120499	0.21582919	0.538233
621080	AI429214	expressed sequence AI429214	NA	0.200741	2.065984	1.149288	0.21590041	0.538328

74558	Gvin1	GTPase, very large interferon inducible 1, transcript v	NA	1.184427	-1.47012	2.272731	0.21595201	0.538375
108168741	Gm46732	predicted gene, 46732	NA	0.359352	0.42285	1.28285	0.21608302	0.538451
115489019	LOC115489019	uncharacterized LOC115489019	NA	0.540981	-0.57133	1.454961	0.21609247	0.538451
12861	Cox6a1	cytochrome c oxidase subunit 6A1	NA	0.090833	6.629915	1.064985	0.21609779	0.538451
50933	Uchl3	ubiquitin carboxyl-terminal esterase L3 (ubiquitin thiol	NA	0.076893	6.033548	1.054744	0.21612407	0.538451
72230	Zfp558	zinc finger protein 558, transcript variant 1	NA	0.06865	6.194129	1.048735	0.21618273	0.538451
105242554	Gm38733	predicted gene, 38733	NA	-0.2	1.79217	-1.1487	0.21625452	0.538451
17357	Marcksl1	MARCKS-like 1	NA	0.06098	10.32935	1.043174	0.21634425	0.538451
12988	Csk	c-src tyrosine kinase, transcript variant X3	NA	-0.0587	7.23823	-1.04152	0.21635865	0.538451
115486510	Gm45095	predicted gene 45095	NA	0.289433	1.307515	1.22216	0.21636721	0.538451
16528	Kcnk4	potassium channel, subfamily K, member 4, transcrip	NA	0.377603	0.019964	1.299182	0.21636869	0.538451
17932	Myt1	myelin transcription factor 1, transcript variant 3	NA	0.073414	6.139364	1.052203	0.21636872	0.538451
320910	Itgb8	integrin beta 8, transcript variant X1	NA	0.127764	5.068649	1.092599	0.21640502	0.538451
76947	Ndufaf6	NADH:ubiquinone oxidoreductase complex assembly	NA	0.138531	3.094107	1.100784	0.21654249	0.538451
269423	Abhd18	abhydrolase domain containing 18, transcript variant	NA	0.108002	4.194065	1.077734	0.21655672	0.538451
22627	Ywhae	tyrosine 3-monooxygenase/tryptophan 5-monooxyge	NA	-0.05897	10.53286	-1.04172	0.2165626	0.538451
629016	Zfp953	zinc finger protein 953	NA	0.281451	1.114677	1.215416	0.21659269	0.538451
69748	Aldh16a1	aldehyde dehydrogenase 16 family, member A1	NA	-0.12988	3.590924	-1.0942	0.21662615	0.538451
230163	Aldob	aldolase B, fructose-bisphosphate	NA	0.448751	-0.30246	1.364858	0.21663168	0.538451
319781	9530051G07Rik	RIKEN cDNA 9530051G07 gene	NA	-0.27033	1.125642	-1.20608	0.21664431	0.538451
14697	Gnb5	guanine nucleotide binding protein (G protein), beta 5	NA	0.081277	5.523767	1.057954	0.21664457	0.538451
102633560	Gm31356	predicted gene, 31356, transcript variant X3	NA	0.260463	1.444975	1.197863	0.21677263	0.538687
69499	Tsr2	TSR2 20S rRNA accumulation, transcript variant 2	NA	0.074779	5.358582	1.0532	0.21687658	0.538796
211134	Lzts1	leucine zipper, putative tumor suppressor 1, transcrip	NA	-0.11137	6.028336	-1.08025	0.21688247	0.538796
18190	Nrxn2	neurexin II, transcript variant 1	NA	-0.07379	8.475119	-1.05247	0.21701668	0.539047
18645	Pfn2	profilin 2	NA	0.061825	8.969792	1.043785	0.21710719	0.539136
239273	Abcc4	ATP-binding cassette, sub-family C (CFTR/MRP), me	NA	0.217432	2.537912	1.162662	0.21715773	0.539136
68316	Apoo	apolipoprotein O, transcript variant 1	NA	0.080553	4.991299	1.057423	0.21716389	0.539136
67286	Ift22	intraflagellar transport 22	NA	-0.10886	4.88365	-1.07838	0.21718501	0.539136
18126	Nos2	nitric oxide synthase 2, inducible, transcript variant 3	NA	-0.28402	0.893091	-1.21758	0.21740952	0.539611
66566	Ntpcr	nucleoside-triphosphatase, cancer-related, transcript	NA	0.133229	3.356957	1.096746	0.21753136	0.539752
16825	Ldb1	LIM domain binding 1, transcript variant 2	NA	-0.06205	8.038481	-1.04395	0.21753299	0.539752
102058	Exoc8	exocyst complex component 8	NA	-0.07525	5.314626	-1.05354	0.21761006	0.53976
100505386	Iqschfp	Iqcj and Schip1 fusion protein	NA	-0.24365	2.880504	-1.18399	0.21761988	0.53976
12571	Cdk6	cyclin-dependent kinase 6	NA	-0.12015	5.214526	-1.08685	0.21763556	0.53976
20448	St6galnac4	ST6 (alpha-N-acetyl-neuraminyl-2,3-beta-galactosyl-1	NA	-0.10266	4.301411	-1.07375	0.21768691	0.539805
270028	Fam155a	family with sequence similarity 155, member A, transc	NA	0.073385	6.606912	1.052183	0.21786894	0.540153
74159	Acbd5	acyl-Coenzyme A binding domain containing 5, transc	NA	0.079129	6.186884	1.05638	0.21789346	0.540153
74097	Pop7	processing of precursor 7, ribonuclease P family, (S.	NA	0.109497	3.8536	1.078852	0.21793053	0.540162
100039239	Gm2115	predicted gene 2115	NA	0.338226	1.039691	1.264201	0.21797051	0.540178
100042173	Rps15a-ps6	ribosomal protein S15A, pseudogene 6	NA	0.105576	4.283784	1.075924	0.21800335	0.540178
118568324	LOC118568324	uncharacterized LOC118568324	NA	-0.37859	0.448146	-1.30007	0.21805913	0.540234
380967	Tmem106c	transmembrane protein 106C, transcript variant 2	NA	0.086709	5.010553	1.061945	0.2181014	0.540257
14726	Pdpn	podoplanin, transcript variant 1	NA	-0.08758	5.321659	-1.06259	0.21815714	0.540307
22195	Ube2l3	ubiquitin-conjugating enzyme E2L 3	NA	-0.05734	7.551644	-1.04055	0.21818802	0.540307
68493	Ndufaf4	NADH:ubiquinone oxidoreductase complex assembly	NA	0.071507	5.370654	1.050814	0.2182285	0.540325
218973	Wdhd1	WD repeat and HMG-box DNA binding protein 1, tran	NA	0.114354	4.666197	1.08249	0.21827636	0.540361
67666	Hapln3	hyaluronan and proteoglycan link protein 3	NA	-0.34529	0.170109	-1.27041	0.21831079	0.540364
228913	Zfp217	zinc finger protein 217, transcript variant X5	NA	-0.12178	4.022001	-1.08808	0.21843553	0.540591
73379	Dcbld2	discoidin, CUB and LCCL domain containing 2, transc	NA	0.082134	6.01913	1.058583	0.21861874	0.540923
65246	Xpo7	exportin 7, transcript variant X3	NA	0.060589	6.634147	1.042892	0.21869693	0.540923
100126795	Gm10575	predicted gene 10575, transcript variant 1	NA	0.214734	1.686827	1.16049	0.21872275	0.540923
71799	Ptcd1	pentatricopeptide repeat domain 1	NA	-0.11533	4.615852	-1.08322	0.21873113	0.540923
67892	Coa6	cytochrome c oxidase assembly factor 6	NA	-0.16011	2.572316	-1.11738	0.21873622	0.540923
66400	Alkbh7	alkB homolog 7, transcript variant 1	NA	-0.14534	3.137566	-1.10599	0.21897626	0.541367
18174	Slc11a2	solute carrier family 11 (proton-coupled divalent meta	NA	0.084607	5.178943	1.060399	0.21900633	0.541367
105653	Phyhip	phytanoyl-CoA hydroxylase interacting protein	NA	0.161618	3.196911	1.118541	0.21903739	0.541367
118568565	LOC118568565	uncharacterized LOC118568565	NA	-0.21195	1.942592	-1.15825	0.21908092	0.541367
54003	Nell2	NEL-like 2, transcript variant 3	NA	-0.0621	7.966127	-1.04398	0.21912164	0.541367
68079	Pdcd2l	programmed cell death 2-like, transcript variant 1	NA	-0.13437	4.201852	-1.09761	0.21914715	0.541367
432508	Cpsf6	cleavage and polyadenylation specific factor 6, transc	NA	0.066015	8.06942	1.046821	0.21915735	0.541367
193452	Zfp184	zinc finger protein 184 (Kruppel-like), transcript variar	NA	0.113437	4.436491	1.081802	0.21918192	0.541367
11979	Atp7b	ATPase, Cu++ transporting, beta polypeptide, transcı	NA	0.181184	2.608281	1.133814	0.21922256	0.541386
22022	Tpst2	protein-tyrosine sulfotransferase 2, transcript variant :	NA	0.121528	3.82966	1.087886	0.21926384	0.541396
115487132	Gm51655	predicted gene, 51655	NA	0.372211	0.008971	1.294335	0.21931802	0.541396
69930	Zfp715	zinc finger protein 715, transcript variant X7	NA	0.092416	4.991797	1.066154	0.21932646	0.541396
108168747	Gm46735	predicted gene, 46735	NA	-0.27784	1.311465	-1.21238	0.21941934	0.541543
227671	Gbgt1	globoside alpha-1,3-N-acetylgalactosaminyltransfera	NA	-0.44365	0.322506	-1.36004	0.21953006	0.541734

60596	Gucy1a1	guanylate cyclase 1, soluble, alpha 1, transcript varia	NA		0.078946	6.39261	1.056246	0.21959771	0.541819
16403	Itga6	integrin alpha 6, transcript variant X1	NA		0.075897	5.666139	1.054016	0.21977416	0.542172
67971	Tppp3	tubulin polymerization-promoting protein family meml	NA		0.088574	4.99071	1.063319	0.21982953	0.542226
71238	Sdhaf3	succinate dehydrogenase complex assembly factor 3	NA		-0.17624	2.494698	-1.12994	0.21991927	0.542302
115489972	Gm52720	predicted gene, 52720	NA		0.392249	-0.16202	1.312437	0.21999138	0.542302
235661	Dync1li1	dynein cytoplasmic 1 light intermediate chain 1	NA		0.062065	6.549806	1.043959	0.21999268	0.542302
19718	Rfc2	replication factor C (activator 1) 2	NA		-0.06754	5.791094	-1.04793	0.22005037	0.542302
100125929	0610009E02Rik	RIKEN cDNA 0610009E02 gene, transcript variant 1	NA		-0.32458	0.709142	-1.2523	0.22009651	0.542302
24056	Sh3bp5	SH3-domain binding protein 5 (BTK-associated), tran	NA		0.062255	7.460342	1.044096	0.22010824	0.542302
17969	Ncf1	neutrophil cytosolic factor 1, transcript variant 2	NA		-0.23345	1.61516	-1.17564	0.22013265	0.542302
118567915	LOC118567915	uncharacterized LOC118567915, transcript variant X:	NA		0.386687	0.653761	1.307387	0.22015095	0.542302
58887	Repin1	replication initiator 1, transcript variant 5	NA		-0.08706	4.925371	-1.06221	0.22016014	0.542302
239157	Pnma2	paraneoplastic antigen MA2	NA		0.080939	5.322962	1.057706	0.22023054	0.542386
268391	A830031A19Rik	RIKEN cDNA A830031A19 gene	NA		-0.26967	1.091974	-1.20553	0.22026104	0.542386
381196	Gm960	predicted gene 960	NA		0.371204	0.184333	1.293431	0.22033345	0.542482
106633	Ift140	intraflagellar transport 140, transcript variant X2	NA		-0.0898	5.288935	-1.06422	0.22040005	0.542564
237711	Eml6	echinoderm microtubule associated protein like 6, tra	NA		0.103251	4.655838	1.074191	0.22045489	0.542617
217082	Hlf	hepatic leukemia factor, transcript variant X5	NA		0.106396	4.260548	1.076535	0.22053749	0.542685
102635086	Gm32510	predicted gene, 32510	NA		0.203858	2.472317	1.151774	0.22054912	0.542685
66432	Slc7a6os	solute carrier family 7, member 6 opposite strand, tra	NA		0.092934	5.19005	1.066537	0.22060336	0.542736
330133	Gm38413	predicted gene, 38413	NA		-0.46737	-0.58247	-1.38259	0.22065307	0.542743
12705	Cited1	Cbp/p300-interacting transactivator with Glu/Asp-rich	NA		0.225603	1.523752	1.169266	0.22082643	0.542743
102632060	Gm30233	predicted gene, 30233, transcript variant X1	NA		-0.29638	1.684745	-1.22806	0.22082756	0.542743
77254	Yif1b	Yip1 interacting factor homolog B (S. cerevisiae), trar	NA		0.107187	4.195035	1.077126	0.2208525	0.542743
18764	Pkd2	polycystin 2, transient receptor potential cation chann	NA		0.071343	5.665896	1.050695	0.22085634	0.542743
18709	Pik3r2	phosphoinositide-3-kinase regulatory subunit 2	NA		-0.07084	6.832841	-1.05033	0.22086108	0.542743
330963	Ankdd1a	ankyrin repeat and death domain containing 1A, trans	NA		0.233716	1.8965	1.17586	0.22087802	0.542743
16993	Lta4h	leukotriene A4 hydrolase, transcript variant 1	NA		0.073196	5.682613	1.052045	0.22090433	0.542743
76429	Lhpp	phospholysine phosphohistidine inorganic pyrophosp	NA		-0.10327	4.280126	-1.07421	0.22090635	0.542743
213332	Mfsd4b4	major facilitator superfamily domain containing 4B4, t	NA		0.117644	3.61717	1.084962	0.22097112	0.54282
68796	Tmem214	transmembrane protein 214	NA		-0.08725	5.554451	-1.06234	0.22100425	0.54282
78334	Cdk19	cyclin-dependent kinase 19, transcript variant X1	NA		0.082392	6.244098	1.058772	0.22113907	0.543069
226551	Suco	SUN domain containing ossification factor, transcript	NA		0.082411	5.877908	1.058786	0.22119207	0.543117
16412	Itgb1	integrin beta 1 (fibronectin receptor beta)	NA		0.055835	7.430477	1.039461	0.22125733	0.543132
14803	Grid1	glutamate receptor, ionotropic, delta 1, transcript vari	NA		0.088893	5.222488	1.063554	0.22126496	0.543132
170765	Ripply3	ripply transcriptional repressor 3	NA		0.438994	-0.00298	1.355659	0.22146012	0.543449
108682	Gpt2	glutamic pyruvate transaminase (alanine aminotransf	NA		-0.08084	5.513307	-1.05763	0.22146068	0.543449
18753	Prkcd	protein kinase C, delta, transcript variant 2	NA		-0.0926	4.584629	-1.06629	0.221501	0.543466
15442	Hpse	heparanase	NA		-0.44318	-0.25766	-1.35959	0.22158888	0.543582
17755	Map1b	microtubule-associated protein 1B	NA		0.076175	11.34135	1.05422	0.22163535	0.543582
272790	Magee2	MAGE family member E2	NA		0.097857	4.359125	1.070182	0.22164856	0.543582
327958	Pitpnm3	PITPNM family member 3, transcript variant X9	NA		0.117512	4.333036	1.084862	0.22173498	0.543712
233977	Ppfia1	protein tyrosine phosphatase, receptor type, f polype	NA		0.06988	5.906231	1.049629	0.22189281	0.544017
252864	Dusp15	dual specificity phosphatase-like 15, transcript varian	NA		0.153894	3.938748	1.112568	0.22199894	0.544125
19824	Trim10	tripartite motif-containing 10	NA		-0.39252	0.273584	-1.31268	0.22208547	0.544125
53895	Clpp	caseinolytic mitochondrial matrix peptidase proteolyti	NA		0.086053	5.018235	1.061462	0.22209722	0.544125
22354	Vipr1	vasoactive intestinal peptide receptor 1, transcript var	NA		0.347045	0.386377	1.271952	0.22211189	0.544125
414077	Wdr83os	WD repeat domain 83 opposite strand	NA		0.083063	5.002205	1.059265	0.22215485	0.544125
226139	Cox15	cytochrome c oxidase assembly protein 15	NA		-0.08907	5.19431	-1.06368	0.22215725	0.544125
109785	Pgm3	phosphoglucomutase 3, transcript variant X3	NA		-0.0976	4.574043	-1.06999	0.22217109	0.544125
68813	Dock5	dedicator of cytokinesis 5, transcript variant X1	NA		-0.22875	2.40135	-1.17182	0.22229122	0.544337
233833	Tnrc6a	trinucleotide repeat containing 6a	NA		0.061623	7.229486	1.043639	0.22237693	0.544465
240239	Gpr151	G protein-coupled receptor 151	NA		-0.38244	0.400725	-1.30355	0.22242534	0.544502
320712	Abi3bp	ABI gene family, member 3 (NESH) binding protein, t	NA		0.352057	0.723989	1.276379	0.22246907	0.544527
18203	Ntan1	N-terminal Asn amidase, transcript variant 4	NA		0.071012	5.817989	1.050453	0.22255119	0.544646
240263	Fem1c	fem 1 homolog c	NA		0.09559	4.449324	1.068502	0.22262339	0.544741
11474	Actn3	actinin alpha 3	NA		0.269485	1.457257	1.205377	0.22269634	0.54478
109212	Pimreg	PICALM interacting mitotic regulator	NA		-0.14014	4.476531	-1.10201	0.2227333	0.54478
19057	Ppp3cc	protein phosphatase 3, catalytic subunit, gamma isof	NA		0.105149	4.172361	1.075606	0.2227397	0.54478
140741	Gpr6	G protein-coupled receptor 6	NA		-0.36994	0.165219	-1.2923	0.22279571	0.544826
21349	Tal1	T cell acute lymphocytic leukemia 1, transcript varian	NA		0.111133	4.771253	1.080076	0.22282569	0.544826
213827	Arcn1	archain 1	NA		0.06313	7.391155	1.04473	0.22293706	0.544986
72935	Ddx41	DEAD box helicase 41	NA		-0.09105	5.365043	-1.06515	0.22297478	0.544986
170938	Zfp617	zinc finger protein 617	NA		0.071481	5.330722	1.050795	0.22299125	0.544986
102632444	Gm30515	predicted gene, 30515, transcript variant X13	NA		-0.36313	0.931452	-1.28621	0.22315976	0.545126
20170	Hps6	HPS6, biogenesis of lysosomal organelles complex 2	NA		0.278726	1.474829	1.213123	0.22316607	0.545126
26941	Slc9a3r1	solute carrier family 9 (sodium/hydrogen exchanger),	NA		0.097417	5.208741	1.069856	0.22319502	0.545126
72429	Dnajc25	DnaJ heat shock protein family (Hsp40) member C25	NA		0.129498	3.401495	1.093913	0.22320441	0.545126

67115	Rpl14	ribosomal protein L14	NA	0.059424	8.219967	1.042049	0.22321626	0.545126
64292	Ptges	prostaglandin E synthase	NA	0.178403	2.254272	1.13163	0.22333832	0.545302
102632990	A730098A19Rik	RIKEN cDNA A730098A19 gene	NA	0.669287	-0.71939	1.590286	0.22338981	0.545302
93686	Rbfox2	RNA binding protein, fox-1 homolog (C. elegans) 2, tr	NA	-0.05518	9.358675	-1.03899	0.22346666	0.545302
106564	Ppcs	phosphopantothenoylcysteine synthetase, transcript v	NA	0.156639	2.762827	1.114688	0.22350558	0.545302
71723	Dhx34	DEAH (Asp-Glu-Ala-His) box polypeptide 34, transcri	NA	-0.10817	4.048578	-1.07786	0.22350761	0.545302
636791	Gm9866	predicted gene 9866, transcript variant 3	NA	0.205921	1.825064	1.153423	0.22351636	0.545302
623078	Gm8108	predicted gene 8108, transcript variant X3	NA	0.420856	1.3865	1.338721	0.22352272	0.545302
54342	Gnpnat1	glucosamine-phosphate N-acetyltransferase 1, transc	NA	0.101229	4.323997	1.072687	0.22360105	0.545411
118567355	LOC118567355	zinc finger protein 844-like	NA	-0.29357	0.86876	-1.22567	0.2237275	0.545638
79264	Krit1	KRIT1, ankyrin repeat containing, transcript variant X	NA	-0.07914	5.643048	-1.05639	0.22411457	0.546434
16866	Lhb	luteinizing hormone beta, transcript variant X1	NA	-0.46485	0.808763	-1.38017	0.22412109	0.546434
27220	Cartpt	CART prepropeptide, transcript variant 2	NA	0.296688	0.938717	1.228322	0.22418099	0.546448
108167660	Gm25640	predicted gene, 25640	NA	-0.35698	0.403095	-1.28074	0.22419422	0.546448
50786	Hs6st2	heparan sulfate 6-O-sulfotransferase 2, transcript var	NA	0.08263	6.026613	1.058947	0.22432005	0.546514
320163	4930525G20Rik	RIKEN cDNA 4930525G20 gene	NA	-0.31945	0.455896	-1.24786	0.22432083	0.546514
60345	Nrip2	nuclear receptor interacting protein 2, transcript varia	NA	0.197552	2.317701	1.146751	0.22432965	0.546514
30050	Fbxw2	F-box and WD-40 domain protein 2, transcript variant	NA	0.066189	6.117393	1.046947	0.2243557	0.546514
56284	Mrpl19	mitochondrial ribosomal protein L19	NA	-0.08028	4.958922	-1.05722	0.22451404	0.54674
20905	Sts	steroid sulfatase	NA	-0.16684	2.9042	-1.1226	0.22451559	0.54674
240479	Dipk1c	divergent protein kinase domain 1C, transcript varian	NA	0.200298	2.825199	1.148936	0.22456776	0.546785
67005	Polr3k	polymerase (RNA) III (DNA directed) polypeptide K	NA	0.063219	6.430292	1.044794	0.22470144	0.54696
18633	Pex16	peroxisomal biogenesis factor 16, transcript variant 2	NA	0.117471	3.66972	1.084832	0.22472736	0.54696
117934533	Gm48551	predicted gene, 48551	NA	-0.49234	2.004147	-1.40672	0.22474041	0.54696
382030	Cnep1r1	CTD nuclear envelope phosphatase 1 regulatory sub	NA	0.08286	5.466871	1.059116	0.22483948	0.547119
110052	Dek	DEK proto-oncogene (DNA binding)	NA	0.061831	7.112762	1.043789	0.22494265	0.547289
115489729	Gm52629	predicted gene, 52629	NA	-0.34661	0.964967	-1.27157	0.22500111	0.547349
66931	1700010I14Rik	RIKEN cDNA 1700010I14 gene, transcript variant 1	NA	0.26236	1.112958	1.199439	0.22505457	0.547391
193796	Kdm4b	lysine (K)-specific demethylase 4B, transcript variant	NA	-0.06686	6.839238	-1.04743	0.22509235	0.547391
66147	Necap2	NECAP endocytosis associated 2, transcript variant 1	NA	0.086314	4.688625	1.061654	0.22511927	0.547391
56210	Rev1	REV1, DNA directed polymerase, transcript variant X	NA	0.079583	5.566498	1.056713	0.22520835	0.547526
29858	Pmm1	phosphomannomutase 1, transcript variant 1	NA	0.078811	5.170383	1.056147	0.22535593	0.547803
27267	Cars	cysteinyl-tRNA synthetase, transcript variant X5	NA	0.076452	5.79521	1.054422	0.22565628	0.548234
17309	Mgat3	mannoside acetylglucosaminyltransferase 3, transcrip	NA	-0.05726	7.037422	-1.04049	0.22566467	0.548234
11804	Aplp2	amyloid beta (A4) precursor-like protein 2, transcript v	NA	0.054733	8.058958	1.038667	0.22570794	0.548234
227707	BC005624	cDNA sequence BC005624	NA	-0.0816	5.521829	-1.05819	0.22572725	0.548234
66161	Pop4	processing of precursor 4, ribonuclease P/MRP famil	NA	0.088727	5.938743	1.063432	0.22573096	0.548234
18392	Orc1	origin recognition complex, subunit 1	NA	-0.29054	0.912884	-1.2231	0.22573557	0.548234
19035	Ppib	peptidylprolyl isomerase B	NA	0.062751	7.23136	1.044455	0.22579843	0.548243
21809	Tgfb3	transforming growth factor, beta 3	NA	-0.16599	3.634214	-1.12193	0.22586088	0.548243
192287	Slc25a36	solute carrier family 25, member 36, transcript varian	NA	0.081855	6.601197	1.058378	0.22586876	0.548243
100042143	Gm3693	predicted gene 3693	NA	0.289962	0.948182	1.222608	0.22587378	0.548243
19271	Ptprj	protein tyrosine phosphatase, receptor type, J, transc	NA	0.073593	5.534123	1.052335	0.22615487	0.548762
67804	Snx2	sorting nexin 2, transcript variant 1	NA	0.069397	6.165247	1.049278	0.22615536	0.548762
214058	Megf11	multiple EGF-like-domains 11, transcript variant X17	NA	0.106019	4.465885	1.076254	0.22619997	0.548789
71137	Rfx4	regulatory factor X, 4 (influences HLA class II expres	NA	0.105375	4.360839	1.075774	0.22631698	0.548991
71130	Sh2d6	SH2 domain containing 6, transcript variant X7	NA	0.41053	-0.30022	1.329174	0.2264274	0.549177
170759	Atp13a1	ATPase type 13A1	NA	-0.06906	6.040701	-1.04903	0.22662554	0.549575
216188	Aldh1l2	aldehyde dehydrogenase 1 family, member L2	NA	0.094835	5.623726	1.067944	0.2267846	0.549798
115487081	C130013N14Rik	RIKEN cDNA C130013N14 gene	NA	-0.35565	0.607301	-1.27956	0.22679071	0.549798
11819	Nr2f2	nuclear receptor subfamily 2, group F, member 2, trai	NA	0.054796	7.545534	1.038712	0.22681869	0.549798
67454	Ikbip	IKBKB interacting protein, transcript variant 1	NA	0.12221	3.709196	1.088401	0.22692244	0.549887
59026	Huwe1	HECT, UBA and WWE domain containing 1	NA	-0.07487	8.521757	-1.05327	0.22692284	0.549887
118567341	LOC118567341	uncharacterized LOC118567341	NA	0.10498	4.591886	1.07548	0.22703143	0.550068
67946	Spata6	spermatogenesis associated 6, transcript variant 2	NA	0.133646	3.397476	1.097063	0.2271129	0.550183
384061	Fndc5	fibronectin type III domain containing 5	NA	0.122014	3.736863	1.088253	0.22725861	0.550454
20437	Siah1a	siah E3 ubiquitin protein ligase 1A	NA	0.090157	6.10578	1.064486	0.22732445	0.550532
16494	Kcna6	potassium voltage-gated channel, shaker-related, sul	NA	0.073332	5.567759	1.052144	0.22741288	0.550588
17700	Mstn	myostatin	NA	0.340715	0.202819	1.266384	0.22743859	0.550588
13131	Dab1	disabled 1, transcript variant 2	NA	0.056923	7.257791	1.040245	0.22745022	0.550588
15370	Nr4a1	nuclear receptor subfamily 4, group A, member 1, tra	NA	0.142327	3.455353	1.103684	0.22748291	0.550588
239852	Zpld1	zona pellucida like domain containing 1, transcript va	NA	0.336102	0.495078	1.262342	0.22760668	0.550737
225049	Ttc7	tetratricopeptide repeat domain 7, transcript variant X	NA	-0.19041	2.57303	-1.14109	0.22761241	0.550737
19009	Pou6f1	POU domain, class 6, transcription factor 1, transcrip	NA	0.064091	7.194803	1.045426	0.22787129	0.551282
319211	Nol4	nucleolar protein 4, transcript variant 1	NA	0.077164	6.338727	1.054942	0.22792639	0.551333
270086	Ogfod1	2-oxoglutarate and iron-dependent oxygenase domai	NA	-0.10258	4.593632	-1.07369	0.22806112	0.551445
30935	Tor3a	torsin family 3, member A	NA	-0.22225	2.007705	-1.16655	0.22808421	0.551445
56456	Actl6a	actin-like 6A	NA	-0.08066	5.44562	-1.0575	0.2281298	0.551445

76411	Ift43	intraflagellar transport 43, transcript variant 1	NA	-0.13785	3.787669	-1.10027	0.22816436	0.551445
20845	Star	steroidogenic acute regulatory protein	NA	0.279443	0.900834	1.213727	0.22818379	0.551445
240067	Zfp952	zinc finger protein 952	NA	-0.08202	4.864222	-1.0585	0.22820817	0.551445
102448	Xylb	xylulokinase homolog (H. influenzae), transcript varia	NA	-0.16037	2.855122	-1.11757	0.22820973	0.551445
18744	Pja1	praja ring finger ubiquitin ligase 1, transcript variant 1	NA	0.056687	7.915458	1.040075	0.22825785	0.551449
225875	Lrfn4	leucine rich repeat and fibronectin type III domain cor	NA	-0.06502	6.148591	-1.0461	0.22827927	0.551449
226090	Ermp1	endoplasmic reticulum metallopeptidase 1, transcript	NA	0.093448	4.622951	1.066917	0.22832196	0.55147
192159	Prpf8	pre-mRNA processing factor 8	NA	-0.05322	8.117553	-1.03758	0.22844424	0.551642
17342	Mitf	melanogenesis associated transcription factor, transc	NA	0.187104	1.915241	1.138476	0.22849593	0.551642
100040563	Dynlt1c	dynein light chain Tctex-type 1C	NA	1.289498	3.36448	2.44443	0.22850678	0.551642
215418	Csrnp1	cysteine-serine-rich nuclear protein 1, transcript varia	NA	-0.25101	1.38253	-1.19004	0.22852865	0.551642
223838	Adamts20	a disintegrin-like and metallopeptidase (reprolysin typ	NA	0.157559	3.142228	1.115398	0.22863409	0.551727
399599	Ccdc87	coiled-coil domain containing 87	NA	-0.33877	0.255058	-1.26468	0.22863924	0.551727
14841	Haspin	histone H3 associated protein kinase	NA	-0.26413	1.831921	-1.20091	0.2286658	0.551727
67429	Nudcd1	NudC domain containing 1, transcript variant 2	NA	-0.09611	4.494133	-1.06888	0.22895816	0.55221
108168756	Gm46741	predicted gene, 46741	NA	-0.35982	0.325631	-1.28326	0.2289591	0.55221
13017	Ctbp2	C-terminal binding protein 2, transcript variant 3	NA	-0.06288	6.446399	-1.04455	0.22901115	0.55221
217430	Pqlc3	PQ loop repeat containing, transcript variant 1	NA	-0.36335	0.046405	-1.28641	0.22901619	0.55221
69372	Mocs3	molybdenum cofactor synthesis 3	NA	0.171921	2.671255	1.126558	0.22903564	0.55221
338371	Endov	endonuclease V, transcript variant 1	NA	-0.11883	5.472762	-1.08585	0.22919372	0.552439
69591	Mdrl	mitochondrial dynamic related lncRNA	NA	0.321284	0.561177	1.249442	0.2291982	0.552439
11898	Ass1	argininosuccinate synthetase 1	NA	0.180369	2.679462	1.133174	0.22923669	0.55245
66055	Sf3b6	splicing factor 3B, subunit 6	NA	0.068528	6.196347	1.048646	0.22932629	0.552462
16515	Kcnj12	potassium inwardly-rectifying channel, subfamily J, m	NA	-0.18261	2.870461	-1.13494	0.22934989	0.552462
70297	Gcc2	GRIP and coiled-coil domain containing 2	NA	0.084987	5.481703	1.060678	0.22935559	0.552462
407803	BC051226	cDNA sequence BC051226	NA	-0.17425	2.485133	-1.12838	0.22937779	0.552462
56216	Stx1b	syntaxin 1B, transcript variant X2	NA	-0.06766	7.774909	-1.04801	0.22958413	0.552694
216285	Alx1	ALX homeobox 1, transcript variant X2	NA	-0.51314	-0.38855	-1.42716	0.22959341	0.552694
18509	Pax7	paired box 7, transcript variant X3	NA	0.157577	3.964299	1.115412	0.22959747	0.552694
18717	Pip5k1c	phosphatidylinositol-4-phosphate 5-kinase, type 1 gai	NA	-0.06017	8.112973	-1.04259	0.22960983	0.552694
11933	Atp1b3	ATPase, Na+/K+ transporting, beta 3 polypeptide, tra	NA	0.05757	6.861544	1.040711	0.22978352	0.55303
19247	Ptpn11	protein tyrosine phosphatase, non-receptor type 11, t	NA	0.054983	7.169768	1.038847	0.2298446	0.553095
74376	Myo18b	myosin XVIIIb	NA	-0.27014	2.108025	-1.20592	0.22991156	0.553175
108958	Miga2	mitoguardin 2, transcript variant 2	NA	-0.09514	4.872104	-1.06817	0.2300833	0.553506
102634592	Gm32133	predicted gene, 32133, transcript variant X1	NA	0.359043	0.035078	1.282575	0.23023262	0.553783
71989	Rpusd4	RNA pseudouridylate synthase domain containing 4	NA	-0.11372	3.679692	-1.08201	0.23031643	0.553903
29806	Limd1	LIM domains containing 1	NA	-0.10083	4.635381	-1.07239	0.23041776	0.554018
12560	Cdh3	cadherin 3, transcript variant 1	NA	-0.18668	2.332744	-1.13814	0.23043239	0.554018
217310	Hid1	HID1 domain containing, transcript variant 2	NA	-0.06398	5.895523	-1.04534	0.2306076	0.554073
16848	Lfng	LFNG O-fucosylpeptide 3-beta-N-acetylglucosaminylt	NA	-0.09301	4.581992	-1.06659	0.23064977	0.554073
216724	Rufy1	RUN and FYVE domain containing 1	NA	0.086774	4.828423	1.061993	0.23067417	0.554073
223433	Otulinl	OTU deubiquitinase with linear linkage specificity like	NA	0.178228	2.963061	1.131493	0.23072583	0.554073
19172	Psmb4	proteasome (prosome, macropain) subunit, beta type	NA	0.085527	7.007225	1.061075	0.2307546	0.554073
83797	Smarcd1	SWI/SNF related, matrix associated, actin dependent	NA	-0.05827	7.634625	-1.04121	0.23075632	0.554073
215243	Traf3ip3	TRAF3 interacting protein 3	NA	-0.32438	0.230249	-1.25212	0.23075851	0.554073
67116	Cuedc2	CUE domain containing 2, transcript variant X7	NA	0.078884	6.623184	1.056201	0.23076242	0.554073
20321	Frrs1	ferric-chelate reductase 1, transcript variant X2	NA	0.151315	3.023186	1.110582	0.23080317	0.554073
56087	Dnah10	dynein, axonemal, heavy chain 10, transcript variant :	NA	0.296025	0.631292	1.227757	0.23080859	0.554073
270893	Tmem132e	transmembrane protein 132E	NA	0.109919	3.98396	1.079168	0.23087563	0.554073
80290	Gpr146	G protein-coupled receptor 146, transcript variant 1	NA	0.236088	1.766219	1.177794	0.23088096	0.554073
73683	Atg16l2	autophagy related 16-like 2 (S. cerevisiae), transcript	NA	-0.16394	3.49506	-1.12034	0.23089795	0.554073
56707	Zfp111	zinc finger protein 111, transcript variant 1	NA	-0.12479	3.975688	-1.09035	0.23096293	0.554148
98985	Clp1	CLP1, cleavage and polyadenylation factor I subunit,	NA	0.139586	3.778963	1.101589	0.23111495	0.554364
19087	Prkar2a	protein kinase, cAMP dependent regulatory, type II al	NA	0.069285	6.971636	1.049196	0.23112127	0.554364
23830	Capn10	calpain 10	NA	-0.0983	5.175118	-1.07051	0.2311943	0.554401
272322	Arntl2	aryl hydrocarbon receptor nuclear translocator-like 2,	NA	-0.25169	1.824698	-1.19061	0.23120474	0.554401
22272	Uqcrq	ubiquinol-cytochrome c reductase, complex III subuni	NA	0.079295	5.567822	1.056502	0.23129689	0.554467
66172	Med11	mediator complex subunit 11	NA	-0.10487	3.915829	-1.07539	0.23133273	0.554467
56353	Rybp	RING1 and YY1 binding protein	NA	0.069349	6.788798	1.049243	0.23137436	0.554467
70231	Gorasp2	golgi reassembly stacking protein 2, transcript variant	NA	0.065942	6.714009	1.046768	0.23141928	0.554467
235406	Snx33	sorting nexin 33	NA	-0.14167	2.78691	-1.10318	0.23143489	0.554467
74015	Fcho1	FCH domain only 1, transcript variant 2	NA	-0.06059	6.336777	-1.04289	0.23143905	0.554467
20911	Stxbp2	syntaxin binding protein 2, transcript variant X8	NA	0.080272	5.065377	1.057218	0.23147072	0.554467
207667	Skor1	SKI family transcriptional corepressor 1, transcript va	NA	0.2018	3.03691	1.150133	0.23151744	0.554497
93897	Fzd10	frizzled class receptor 10	NA	-0.12246	3.785789	-1.08859	0.23161192	0.554585
433424	Zeb2os	zinc finger E-box binding homeobox 2, opposite stran	NA	-0.19021	2.096012	-1.14093	0.23162341	0.554585
242860	Rsbn1l	round spermatid basic protein 1-like	NA	0.098158	5.060743	1.070406	0.23165649	0.554585
78943	Ern1	endoplasmic reticulum (ER) to nucleus signalling 1, tr	NA	0.119142	4.052156	1.086089	0.23173788	0.554629

14748	Gpr3	G-protein coupled receptor 3	NA	-0.36869	0.139654	-1.29118	0.23176161	0.554629
74577	Glb1l	galactosidase, beta 1-like, transcript variant X3	NA	0.109513	4.530329	1.078864	0.23179273	0.554629
112422	Zfp979	zinc finger protein 979	NA	0.21183	1.86092	1.158156	0.23182387	0.554629
17191	Mbd2	methyl-CpG binding domain protein 2, transcript varia	NA	-0.10093	5.245229	-1.07247	0.23188303	0.554629
238266	Syt16	synaptotagmin XVI, transcript variant 4	NA	0.069592	6.395396	1.04942	0.23190682	0.554629
109334	B230398E01Rik	RIKEN cDNA B230398E01 gene, transcript variant X	NA	0.605881	-0.50136	1.521908	0.2319133	0.554629
11624	Ahrr	aryl-hydrocarbon receptor repressor, transcript variar	NA	0.24813	1.480623	1.187667	0.23197087	0.554685
102632136	Gm20482	predicted gene 20482	NA	0.705975	-1.10044	1.631247	0.23201184	0.554701
16413	Itgb1bp1	integrin beta 1 binding protein 1, transcript variant X3	NA	0.10881	3.864558	1.078338	0.23208633	0.554798
102639384	Gm35711	predicted gene, 35711, transcript variant X3	NA	0.341488	0.638442	1.267063	0.23216679	0.554834
20980	Syt2	synaptotagmin II, transcript variant 2	NA	0.160837	3.448115	1.117936	0.23218082	0.554834
101489	Ric8a	RIC8 guanine nucleotide exchange factor A	NA	0.067521	5.991047	1.047915	0.23220379	0.554834
110596	Arhgef28	Rho guanine nucleotide exchange factor (GEF) 28, tr	NA	0.103678	4.414885	1.074509	0.23229843	0.554979
56195	Ptbp2	polypyrimidine tract binding protein 2, transcript varia	NA	0.068652	8.330749	1.048737	0.23234799	0.555016
230809	Pdik1l	PDLIM1 interacting kinase 1 like, transcript variant 2	NA	0.08701	5.477603	1.062167	0.23245353	0.555186
225608	Sh3tc2	SH3 domain and tetratricopeptide repeats 2	NA	0.24098	1.187794	1.181795	0.23250125	0.555219
55982	Paxip1	PAX interacting (with transcription-activation domain)	NA	-0.08078	5.814502	-1.05759	0.23260171	0.555351
19270	Ptprg	protein tyrosine phosphatase, receptor type, G, transc	NA	0.079649	6.652069	1.056761	0.23264993	0.555351
16847	Lepr	leptin receptor, transcript variant 3	NA	0.12694	3.231607	1.091975	0.23268627	0.555351
69156	Comtd1	catechol-O-methyltransferase domain containing 1	NA	0.156578	2.616609	1.114641	0.23269327	0.555351
67217	L3hypdh	L-3-hydroxyproline dehydratase (trans-)	NA	0.173771	2.297025	1.128003	0.23290443	0.555774
76612	Lrrc27	leucine rich repeat containing 27, transcript variant X	NA	-0.21945	1.955909	-1.16429	0.232956	0.555788
60527	Fads3	fatty acid desaturase 3	NA	-0.07611	5.120512	-1.05417	0.2329786	0.555788
263406	Plekhg3	pleckstrin homology domain containing, family G (with	NA	-0.18136	2.609825	-1.13396	0.23316952	0.556139
75690	Vsig10l	V-set and immunoglobulin domain containing 10 like,	NA	0.152177	3.177094	1.111245	0.23322231	0.556139
383295	Ypel5	yippee like 5, transcript variant 1	NA	0.070343	6.317438	1.049966	0.23323694	0.556139
66705	Dnase1l2	deoxyribonuclease 1-like 2, transcript variant X1	NA	-0.1451	3.123124	-1.10581	0.23327544	0.556139
107022	Gramd3	GRAM domain containing 3, transcript variant 2	NA	0.158502	3.202392	1.116128	0.2332969	0.556139
18301	Fxyd5	FXYD domain-containing ion transport regulator 5, tra	NA	0.146634	2.938108	1.106984	0.23337325	0.556166
20359	Sema6b	sema domain, transmembrane domain (TM), and cyto	NA	-0.07375	6.041544	-1.05245	0.23337664	0.556166
66910	Tmem107	transmembrane protein 107, transcript variant 1	NA	-0.17975	2.690744	-1.13269	0.23342216	0.556194
54152	Dnal4	dynein, axonemal, light chain 4	NA	-0.07637	4.98989	-1.05436	0.23354104	0.556395
76895	Bicd2	BICD cargo adaptor 2, transcript variant X1	NA	0.077654	6.287163	1.0553	0.23368308	0.556652
115490354	LOC115490354	uncharacterized LOC115490354	NA	-0.19126	2.171198	-1.14176	0.23382552	0.556904
665155	Srp54b	signal recognition particle 54B	NA	0.090022	6.918325	1.064386	0.23385706	0.556904
381352	Mamdc4	MAM domain containing 4, transcript variant X1	NA	-0.19482	2.174329	-1.14458	0.23399747	0.557091
14113	Fbl	fibrillarin	NA	-0.07679	6.273006	-1.05467	0.23400424	0.557091
102636000	Gm33195	predicted gene, 33195, transcript variant X19	NA	-0.34785	0.65611	-1.27266	0.23417857	0.557291
320226	Ccdc171	coiled-coil domain containing 171, transcript variant 2	NA	-0.17119	2.703522	-1.12599	0.23418517	0.557291
67268	Myl12a	myosin, light chain 12A, regulatory, non-sarcomeric, t	NA	-0.08024	5.602258	-1.05719	0.23419083	0.557291
15530	Hspg2	perlecan (heparan sulfate proteoglycan 2)	NA	-0.07072	5.865259	-1.05024	0.2342578	0.557305
269295	Rtn4rl2	reticulon 4 receptor-like 2, transcript variant X2	NA	0.125971	3.895592	1.091242	0.23426535	0.557305
237253	Lrp11	low density lipoprotein receptor-related protein 11, tra	NA	0.064718	6.06829	1.04588	0.23431212	0.55732
20910	Stxbp1	syntaxin binding protein 1, transcript variant 2	NA	0.058371	8.723629	1.041289	0.23434019	0.55732
56613	Rps6ka4	ribosomal protein S6 kinase, polypeptide 4	NA	-0.12345	3.505669	-1.08934	0.23438039	0.557334
67768	N6amt1	N-6 adenine-specific DNA methyltransferase 1 (putat	NA	0.111445	3.940269	1.08031	0.23453624	0.557623
26919	Zfp346	zinc finger protein 346, transcript variant X1	NA	0.090388	5.130688	1.064657	0.23459223	0.557641
73225	Fam118a	family with sequence similarity 118, member A, transc	NA	0.112494	4.639337	1.081095	0.23461238	0.557641
239393	Lrp12	low density lipoprotein-related protein 12, transcript v	NA	0.075811	6.274815	1.053953	0.23466441	0.557654
73067	Tmem192	transmembrane protein 192, transcript variant 1	NA	0.138801	3.056585	1.100989	0.23470118	0.557654
68044	Chac2	ChaC, cation transport regulator 2, transcript variant	NA	0.114192	3.476162	1.082369	0.23475416	0.557654
108167700	Gm46139	predicted gene, 46139	NA	0.445196	-0.18253	1.361499	0.23475473	0.557654
545725	Mterf1a	mitochondrial transcription termination factor 1a, tran	NA	-0.22204	2.231221	-1.16638	0.23486572	0.557792
66336	Cenpp	centromere protein P	NA	-0.23217	2.170543	-1.1746	0.23488128	0.557792
74190	Exoc3l4	exocyst complex component 3-like 4, transcript variar	NA	0.411175	-0.37491	1.329768	0.23493282	0.557833
57895	Ccdc126	coiled-coil domain containing 126, transcript variant 1	NA	0.157398	2.670481	1.115274	0.23503598	0.557948
320593	A230051N06Rik	RIKEN cDNA A230051N06 gene	NA	-0.4287	-0.29352	-1.34602	0.23505541	0.557948
16179	Irak1	interleukin-1 receptor-associated kinase 1, transcript	NA	0.069223	5.561631	1.049152	0.23508449	0.557948
72522	Atxn7l2	ataxin 7-like 2, transcript variant 1	NA	-0.08187	5.193599	-1.05839	0.23517738	0.558047
74775	Lmbr1l	limb region 1 like	NA	-0.08067	4.904993	-1.05751	0.23522256	0.558047
433294	Mettl21c	methyltransferase like 21C, transcript variant X2	NA	0.396773	0.040641	1.31656	0.23524393	0.558047
70370	Fbln7	fibulin 7	NA	0.27753	1.571015	1.212118	0.23526338	0.558047
19881	Rom1	rod outer segment membrane protein 1	NA	0.342462	0.468811	1.267919	0.23530848	0.558073
217038	Mrm1	mitochondrial rRNA methyltransferase 1	NA	-0.14728	3.191311	-1.10748	0.2354196	0.558202
117167	Steap4	STEAP family member 4	NA	0.570228	-0.96164	1.484758	0.23544036	0.558202
320343	Lypd6	LY6/PLAUR domain containing 6, transcript variant 1	NA	0.117322	4.11855	1.084719	0.23554365	0.558202
66540	Fam107b	family with sequence similarity 107, member B, transc	NA	0.085045	6.107575	1.060721	0.23558588	0.558202
76024	Gm11346	predicted gene 11346	NA	-0.33453	0.478628	-1.26097	0.23562451	0.558202

105243578	Gm39463	predicted gene, 39463	NA	0.21767	1.728095	1.162854	0.23562763	0.558202
67020	Tmem88	transmembrane protein 88	NA	0.228195	1.370233	1.171369	0.23565113	0.558202
67471	Gpatch1	G patch domain containing 1	NA	0.100113	4.657181	1.071857	0.23566625	0.558202
229715	Amigo1	adhesion molecule with Ig like domain 1, transcript va	NA	-0.08193	5.477732	-1.05843	0.23567153	0.558202
245007	Zbtb38	zinc finger and BTB domain containing 38, transcript	NA	0.072284	5.550131	1.05138	0.23582337	0.558474
100502829	Gm9899	predicted gene 9899	NA	0.171394	2.610836	1.126146	0.23585507	0.558474
66385	Ppp1r7	protein phosphatase 1, regulatory subunit 7	NA	0.068583	5.675124	1.048686	0.23592422	0.558502
108167918	Gm46290	predicted gene, 46290, transcript variant X3	NA	-0.08714	4.997405	-1.06226	0.23596069	0.558502
66181	Nop10	NOP10 ribonucleoprotein	NA	-0.11557	5.190875	-1.0834	0.23599208	0.558502
624549	Gm29683	predicted gene, 29683, transcript variant 1	NA	-0.29066	0.579349	-1.2232	0.23600424	0.558502
118567801	LOC118567801	uncharacterized LOC118567801	NA	-0.29756	1.409482	-1.22906	0.23606468	0.558564
112694759	Gm20517	predicted gene 20517, transcript variant 2	NA	-0.33075	1.460528	-1.25767	0.2361318	0.558573
22333	Vdac1	voltage-dependent anion channel 1, transcript variant	NA	0.059642	8.571603	1.042207	0.2361373	0.558573
16783	Lamp1	lysosomal-associated membrane protein 1, transcript	NA	0.051851	8.027863	1.036594	0.23624946	0.558734
94040	Clmn	calmin, transcript variant 2	NA	0.1285	3.680833	1.093157	0.23631984	0.558734
218793	Ube2e2	ubiquitin-conjugating enzyme E2E 2, transcript variar	NA	0.07021	5.755252	1.04987	0.23632969	0.558734
216516	Ccdc157	coiled-coil domain containing 157, transcript variant X	NA	-0.08727	4.650404	-1.06236	0.23637081	0.558734
66838	0610009L18Rik	RIKEN cDNA 0610009L18 gene	NA	-0.2972	0.58717	-1.22876	0.23643392	0.558734
13640	Efna5	ephrin A5, transcript variant 1	NA	0.084548	6.025889	1.060355	0.23643723	0.558734
12570	Cdk5r2	cyclin-dependent kinase 5, regulatory subunit 2 (p39)	NA	0.090412	6.64198	1.064674	0.23648143	0.558734
67071	Rps6ka6	ribosomal protein S6 kinase polypeptide 6, transcript	NA	0.072831	5.943481	1.051779	0.23650386	0.558734
69478	2300009A05Rik	RIKEN cDNA 2300009A05 gene	NA	0.207071	2.517108	1.154342	0.23651404	0.558734
246102	Rttn	rotatin, transcript variant X8	NA	-0.17997	3.130705	-1.13286	0.23656347	0.558769
72893	2900040C04Rik	RIKEN cDNA 2900040C04 gene	NA	0.325401	0.443754	1.253012	0.23661772	0.558816
102657	Cd276	CD276 antigen	NA	-0.06844	5.815347	-1.04858	0.23665922	0.558833
105246536	Gm41810	predicted gene, 41810	NA	0.310365	0.886777	1.240021	0.23673268	0.558925
240665	Ccnj	cyclin J, transcript variant X3	NA	-0.08342	5.051811	-1.05953	0.23679367	0.558988
66812	Ppcdc	phosphopantothenoylcysteine decarboxylase, transcr	NA	0.14459	3.749974	1.105416	0.23683211	0.558998
78038	Mccc2	methylcrotonoyl-Coenzyme A carboxylase 2 (beta)	NA	-0.09001	4.506776	-1.06438	0.23694261	0.559178
207839	Galnt6	polypeptide N-acetylgalactosaminyltransferase 6, trar	NA	-0.40992	-0.41183	-1.32861	0.23711455	0.559399
13838	Epha4	Eph receptor A4, transcript variant X2	NA	-0.07111	7.0551	-1.05053	0.23714971	0.559399
75406	Ndufs7	NADH:ubiquinone oxidoreductase core subunit S7, tr	NA	0.098044	4.775614	1.070321	0.23718366	0.559399
235559	Topbp1	topoisomerase (DNA) II binding protein 1	NA	0.070232	5.651574	1.049885	0.23723002	0.559399
56403	Syncrip	synaptotagmin binding, cytoplasmic RNA interacting		NA	0.061602	8.144898	1.043624	0.23723573	0.559399
15529	Sdc2	syndecan 2	NA	0.10066	4.858013	1.072264	0.23724287	0.559399
237988	Cdr2l	cerebellar degeneration-related protein 2-like	NA	-0.06374	6.004857	-1.04517	0.23731111	0.559479
100041273	Ndufb4c	NADH:ubiquinone oxidoreductase subunit B4C	NA	0.110168	4.840093	1.079354	0.23739478	0.559505
22127	Tsx	testis specific X-linked gene, transcript variant X1	NA	0.395394	-0.35093	1.315302	0.23744116	0.559505
15561	Htr3a	5-hydroxytryptamine (serotonin) receptor 3A, transcri	NA	0.167785	2.484957	1.123332	0.23745244	0.559505
56410	Cbln3	cerebellin 3 precursor protein	NA	0.468756	0.011757	1.383916	0.23748226	0.559505
13047	Cux1	cut-like homeobox 1, transcript variant 2	NA	0.061554	7.56998	1.04359	0.23749387	0.559505
192657	Ell2	elongation factor for RNA polymerase II 2	NA	-0.19011	1.913229	-1.14085	0.23754221	0.559538
66259	Camk2n1	calcium/calmodulin-dependent protein kinase II inhibi	NA	0.077255	7.113577	1.055009	0.23760363	0.559572
73431	1700052K11Rik	RIKEN cDNA 1700052K11 gene	NA	0.13245	3.080286	1.096153	0.23775585	0.559572
140742	Sesn1	sestrin 1, transcript variant 2	NA	0.066938	5.538079	1.047491	0.23776569	0.559572
19167	Psma3	proteasome subunit alpha 3, transcript variant X4	NA	0.054748	7.347706	1.038678	0.23777655	0.559572
242700	Ifnlr1	interferon lambda receptor 1	NA	-0.38524	0.071575	-1.30607	0.23779079	0.559572
319885	Zcchc7	zinc finger, CCHC domain containing 7, transcript var	NA	0.0874	5.164708	1.062454	0.23779444	0.559572
17181	Matn2	matrilin 2, transcript variant 1	NA	0.137304	3.341074	1.099848	0.23779739	0.559572
18080	Nin	ninein, transcript variant X10	NA	0.078939	5.927358	1.056241	0.23785921	0.559636
217830	Dglucy	D-glutamate cyclase, transcript variant X4	NA	-0.20765	1.767774	-1.15481	0.23791844	0.559669
21985	Tpd52	tumor protein D52, transcript variant X10	NA	0.086273	5.04901	1.061624	0.23794179	0.559669
72607	Usp13	ubiquitin specific peptidase 13 (isopeptidase T-3), tra	NA	0.110581	4.667958	1.079663	0.23808528	0.559885
66144	Atp6v1f	ATPase, H+ transporting, lysosomal V1 subunit F	NA	0.071908	5.537596	1.051106	0.23810232	0.559885
381823	Apold1	apolipoprotein L domain containing 1	NA	0.193903	2.919309	1.143854	0.23823054	0.559919
56295	Higd1a	HIG1 domain family, member 1A, transcript variant 5	NA	0.115179	5.334305	1.08311	0.23824888	0.559919
79362	Bhlhe41	basic helix-loop-helix family, member e41, transcript v	NA	0.189666	2.691408	1.140499	0.23826805	0.559919
68767	Washc1	WASH complex subunit 1, transcript variant 2	NA	-0.08874	5.49909	-1.06344	0.23828468	0.559919
23790	Coro1c	coronin, actin binding protein 1C	NA	-0.05943	7.953814	-1.04205	0.23828911	0.559919
105245381	Gm40847	predicted gene, 40847, transcript variant X1	NA	-0.40169	0.86338	-1.32105	0.23834262	0.559964
193003	Pirt	phosphoinositide-interacting regulator of transient rec	NA	0.382511	1.559647	1.303608	0.23843173	0.560089
76051	Ganc	glucosidase, alpha; neutral C	NA	0.190761	1.852455	1.141366	0.23846453	0.560089
74430	Cfap20dc	CFAP20 domain containing, transcript variant X4	NA	0.12684	3.273053	1.0919	0.23850947	0.560097
232599	Gm4876	predicted gene 4876	NA	0.322809	0.360991	1.250763	0.23853702	0.560097
16661	Krt10	keratin 10	NA	-0.76685	3.873552	-1.70155	0.23859163	0.56012
80859	Nfkbiz	nuclear factor of kappa light polypeptide gene enhanc	NA	0.203376	1.692271	1.151389	0.23863722	0.56012
77044	Arid2	AT rich interactive domain 2 (ARID, RFX-like)	NA	0.077315	7.011058	1.055052	0.23868269	0.56012
215951	Afg1l	AFG1 like ATPase, transcript variant X4	NA	-0.19215	2.599708	-1.14246	0.23868422	0.56012

67442	Retsat	retinol saturase (all trans retinol 13,14 reductase)	NA	0.194092	1.785025	1.144004	0.23876817	0.560236
330119	Adamts3	a disintegrin-like and metallopeptidase (reprolysin typ	NA	0.085324	5.191253	1.060926	0.23886195	0.560356
93884	Pcdhb13	protocadherin beta 13	NA	0.217223	1.43741	1.162493	0.23894247	0.560356
102632268	Gm26974	predicted gene, 26974, transcript variant X1	NA	0.334148	0.314438	1.260633	0.23900183	0.560356
80860	Ghdc	GH3 domain containing	NA	-0.22213	2.518313	-1.16645	0.23903015	0.560356
14200	Fhl2	four and a half LIM domains 2, transcript variant 1	NA	0.146786	3.376803	1.1071	0.23904347	0.560356
54422	Barhl1	BarH like homeobox 1, transcript variant X3	NA	0.090857	5.031825	1.065002	0.23905824	0.560356
70681	Abraxas1	BRCA1 A complex subunit, transcript variant X5	NA	0.165351	2.798526	1.121439	0.23906042	0.560356
19883	Rora	RAR-related orphan receptor alpha, transcript variant	NA	0.095836	5.445362	1.068685	0.23915596	0.560444
98845	Eps8l2	EPS8-like 2	NA	-0.31707	0.663353	-1.2458	0.23918318	0.560444
69956	Ptcd3	pentatricopeptide repeat domain 3, transcript variant	NA	0.067581	5.960471	1.047958	0.23920131	0.560444
207965	Vcpkmt	valosin containing protein lysine (K) methyltransferas	NA	-0.13121	4.423517	-1.09521	0.23925794	0.560456
71816	Rnf180	ring finger protein 180, transcript variant X9	NA	0.096472	5.237596	1.069156	0.23932674	0.560456
67797	Snrnp48	small nuclear ribonucleoprotein 48 (U11/U12), transc	NA	-0.12584	4.586216	-1.09114	0.239351	0.560456
22210	Ube2b	ubiquitin-conjugating enzyme E2B, transcript variant :	NA	-0.05891	6.891937	-1.04168	0.23937632	0.560456
67254	Bmerb1	bMERB domain containing 1	NA	0.055631	7.333473	1.039313	0.23937869	0.560456
106529	Tecr	trans-2,3-enoyl-CoA reductase, transcript variant 2	NA	0.055528	8.160467	1.03924	0.23944842	0.560459
20346	Sema3a	sema domain, immunoglobulin domain (Ig), short bas	NA	0.077472	5.371552	1.055167	0.23946309	0.560459
171486	Cd99l2	CD99 antigen-like 2, transcript variant 1	NA	-0.06239	6.643108	-1.04419	0.23948323	0.560459
108168925	Gm46841	predicted gene, 46841	NA	-0.20791	2.832319	-1.15501	0.23954232	0.560506
69940	Exoc1	exocyst complex component 1, transcript variant 1	NA	0.062163	6.228377	1.04403	0.23958593	0.560506
67157	2610301B20Rik	RIKEN cDNA 2610301B20 gene	NA	0.083947	5.071238	1.059914	0.23960657	0.560506
83409	Lamtor2	late endosomal/lysosomal adaptor, MAPK and MTOF	NA	0.11281	4.934495	1.081333	0.23995755	0.561109
72477	Tmem87b	transmembrane protein 87B, transcript variant 3	NA	0.080791	5.239216	1.057598	0.23997656	0.561109
108169076	Gm36266	predicted gene, 36266	NA	-0.31309	0.467997	-1.24237	0.2399901	0.561109
71805	Nup93	nucleoporin 93, transcript variant X1	NA	0.067847	6.101204	1.048151	0.24000221	0.561109
15903	Id3	inhibitor of DNA binding 3	NA	0.092452	4.816209	1.066181	0.24006649	0.561178
23887	Ggt5	gamma-glutamyltransferase 5, transcript variant X1	NA	0.207219	1.738842	1.154461	0.24018173	0.561317
102637776	Gm34502	predicted gene, 34502, transcript variant X4	NA	-0.33001	0.519663	-1.25702	0.24019472	0.561317
212276	Zfp748	zinc finger protein 748	NA	0.097792	4.527846	1.070134	0.24030629	0.561359
69957	Cdc16	CDC16 cell division cycle 16, transcript variant 1	NA	-0.06503	6.749092	-1.0461	0.24031457	0.561359
20595	Smn1	survival motor neuron 1, transcript variant 1	NA	0.089788	5.145148	1.064213	0.24033726	0.561359
20965	Syn2	synapsin II, transcript variant IIc	NA	0.071082	6.415664	1.050504	0.24035075	0.561359
75210	Prr3	proline-rich polypeptide 3, transcript variant 1	NA	-0.07079	6.032937	-1.05029	0.24045392	0.561485
19171	Psmb10	proteasome (prosome, macropain) subunit, beta type	NA	0.147144	3.035481	1.107375	0.24047358	0.561485
231162	Cytl1	cytokine-like 1	NA	-0.57304	-1.03764	-1.48765	0.24065866	0.561825
22045	Trhr	thyrotropin releasing hormone receptor	NA	0.231604	1.418412	1.17414	0.2407029	0.561825
67701	Wfdc2	WAP four-disulfide core domain 2, transcript variant 1	NA	-0.31599	0.400851	-1.24487	0.24072303	0.561825
104318	Csnk1d	casein kinase 1, delta, transcript variant 1	NA	-0.05375	7.842989	-1.03796	0.24078534	0.561852
11544	Adprh	ADP-ribosylarginine hydrolase	NA	-0.06004	6.494279	-1.0425	0.2408378	0.561852
386750	Slitrk3	SLIT and NTRK-like family, member 3, transcript vari	NA	0.076518	5.145273	1.05447	0.24086405	0.561852
666532	Zfp991	zinc finger protein 991	NA	0.177458	3.95429	1.13089	0.24087648	0.561852
232906	Arhgap35	Rho GTPase activating protein 35	NA	-0.06165	8.206488	-1.04366	0.2409073	0.561852
382221	Gm1141	predicted gene 1141	NA	-0.35633	0.279585	-1.28017	0.2409431	0.561855
319822	Smyd4	SET and MYND domain containing 4	NA	0.129119	3.187343	1.093626	0.24098245	0.561862
20418	Shc3	src homology 2 domain-containing transforming prote	NA	0.060906	6.658695	1.043121	0.24101525	0.561862
69082	Zc3h15	zinc finger CCCH-type containing 15	NA	-0.05319	7.061695	-1.03756	0.24109619	0.561896
68051	Nutf2	nuclear transport factor 2, transcript variant 1	NA	-0.06143	6.645758	-1.0435	0.24109888	0.561896
433004	B830017H08Rik	RIKEN cDNA B830017H08 gene	NA	0.275571	1.016057	1.210473	0.24124815	0.562164
57440	Ehd3	EH-domain containing 3	NA	0.077679	5.194653	1.055319	0.24128717	0.562174
74022	Glyr1	glyoxylate reductase 1 homolog (Arabidopsis), transc	NA	-0.06443	7.795526	-1.04567	0.24139908	0.562348
69668	Ccdc115	coiled-coil domain containing 115	NA	0.076066	5.05246	1.05414	0.24148178	0.562348
66469	Prxl2b	peroxiredoxin like 2B	NA	0.12554	3.410939	1.090916	0.24150856	0.562348
242362	Manea	mannosidase, endo-alpha	NA	0.086958	4.888619	1.062128	0.24151612	0.562348
66399	Tsfm	Ts translation elongation factor, mitochondrial	NA	-0.11338	3.839018	-1.08176	0.24155464	0.562348
67154	Mtdh	metadherin, transcript variant 3	NA	0.071137	6.943711	1.050544	0.24157736	0.562348
212980	Slc45a3	solute carrier family 45, member 3, transcript variant :	NA	-0.24616	1.331199	-1.18605	0.24160352	0.562348
73712	Dmkn	dermokine, transcript variant 5	NA	-0.52954	1.864783	-1.44347	0.24165092	0.562378
225994	Nmrk1	nicotinamide riboside kinase 1	NA	-0.19679	1.702094	-1.14615	0.24169796	0.562407
74427	Eaf1	ELL associated factor 1	NA	-0.09076	4.600776	-1.06493	0.24177561	0.56246
223989	Marf1	meiosis regulator and mRNA stability 1	NA	-0.05741	6.384922	-1.04059	0.24178991	0.56246
381280	Hjurp	Holliday junction recognition protein, transcript varian	NA	-0.07145	7.388069	-1.05077	0.24200018	0.562868
333670	Gm867	predicted gene 867, transcript variant X7	NA	-0.32442	0.243752	-1.25216	0.242153	0.563067
108168162	Gm43305	predicted gene 43305	NA	-0.16279	3.631015	-1.11945	0.24215493	0.563067
22759	Zfp97	zinc finger protein 97	NA	0.253297	1.79155	1.191928	0.24237809	0.563461
19645	Rb1	RB transcriptional corepressor 1	NA	-0.06814	6.235212	-1.04837	0.24239336	0.563461
18391	Sigmar1	sigma non-opioid intracellular receptor 1, transcript va	NA	-0.10565	4.819345	-1.07598	0.24257022	0.563791
269252	Gtf3c4	general transcription factor IIIC, polypeptide 4, transc	NA	0.057581	6.612519	1.04072	0.24269357	0.563846

58227	Fam184b	family with sequence similarity 184, member B	NA	0.13299	3.327234	1.096564	0.24273636	0.563846
56693	Crtap	cartilage associated protein	NA	-0.11829	4.03827	-1.08545	0.24274681	0.563846
666680	Gm8234	predicted gene 8234	NA	0.394717	0.165498	1.314685	0.24277233	0.563846
69922	Vrk2	vaccinia related kinase 2, transcript variant X1	NA	-0.20849	1.97893	-1.15548	0.24284636	0.563846
66606	Lrrc57	leucine rich repeat containing 57, transcript variant 3	NA	-0.0936	4.378303	-1.06703	0.2428516	0.563846
67303	3110045C21Rik	RIKEN cDNA 3110045C21 gene	NA	0.4	0.027669	1.319508	0.24285455	0.563846
381293	Kif14	kinesin family member 14	NA	-0.19053	2.873372	-1.14118	0.24287078	0.563846
17203	Mc5r	melanocortin 5 receptor	NA	0.218425	1.491543	1.163462	0.24291318	0.563864
16525	Kcnk1	potassium channel, subfamily K, member 1	NA	0.129039	3.959688	1.093565	0.24296589	0.563897
396184	Flrt1	fibronectin leucine rich transmembrane protein 1, trar	NA	-0.08673	5.47979	-1.06196	0.2430675	0.563897
243374	Gimap8	GTPase, IMAP family member 8, transcript variant 2	NA	0.281853	0.972585	1.215755	0.24311424	0.563897
319758	Rfx7	regulatory factor X, 7	NA	0.066492	6.70199	1.047168	0.24317727	0.563897
230514	Leprot	leptin receptor overlapping transcript, transcript variaı	NA	-0.06533	5.999892	-1.04632	0.24319435	0.563897
72722	Fam98a	family with sequence similarity 98, member A, transcı	NA	-0.06722	5.930867	-1.0477	0.2432137	0.563897
330938	Dixdc1	DIX domain containing 1, transcript variant X11	NA	0.074611	6.347872	1.053077	0.24323459	0.563897
52392	Macir	macrophage immunometabolism regulator, transcript	NA	0.070093	5.970808	1.049785	0.24329632	0.563897
93688	Klhl1	kelch-like 1	NA	0.111776	3.761933	1.080558	0.24330477	0.563897
224648	Uhrf1bp1	UHRF1 (ICBP90) binding protein 1	NA	0.096462	5.095237	1.069148	0.24333764	0.563897
14586	Gfra2	glial cell line derived neurotrophic factor family recept	NA	0.089044	4.975569	1.063665	0.24334014	0.563897
16798	Lats1	large tumor suppressor	NA	0.061756	6.446352	1.043735	0.24335657	0.563897
226432	Ipo9	importin 9, transcript variant 1	NA	0.055153	7.677892	1.038969	0.24343493	0.563897
666311	Zscan25	zinc finger and SCAN domain containing 25	NA	-0.09822	4.416872	-1.07045	0.24345129	0.563897
105246251	Gm41559	predicted gene, 41559, transcript variant X1	NA	-0.26949	1.317073	-1.20538	0.24347597	0.563897
73293	Ccdc103	coiled-coil domain containing 103, transcript variant 1	NA	0.302655	0.502535	1.233412	0.24348219	0.563897
328572	Ep300	E1A binding protein p300, transcript variant X4	NA	0.065656	6.489552	1.046561	0.24352084	0.563907
18823	Plp1	proteolipid protein (myelin) 1, transcript variant 2	NA	0.101943	4.134256	1.073218	0.24356624	0.563931
67808	Tprgl	transformation related protein 63 regulated like, trans	NA	0.060986	6.525804	1.043178	0.24365523	0.563948
56513	Pard6a	par-6 family cell polarity regulator alpha, transcript va	NA	0.118881	3.686554	1.085892	0.24366396	0.563948
233904	Setd1a	SET domain containing 1A	NA	-0.06306	6.206043	-1.04468	0.24367734	0.563948
102635994	Gm33190	predicted gene, 33190, transcript variant X1	NA	0.320183	0.549478	1.248489	0.24371903	0.563964
21416	Tcf7l2	transcription factor 7 like 2, T cell specific, HMG box,	NA	0.104546	8.020835	1.075156	0.24387719	0.56422
115490350	Gm52861	predicted gene, 52861, transcript variant X2	NA	-0.10524	4.456882	-1.07568	0.243899	0.56422
100048895	9330020H09Rik	RIKEN cDNA 9330020H09 gene	NA	0.232661	1.537573	1.175	0.24394053	0.564236
54397	Ppt2	palmitoyl-protein thioesterase 2, transcript variant 5	NA	-0.09384	4.375948	-1.0672	0.24400721	0.56431
67030	Fancl	Fanconi anemia, complementation group L, transcript	NA	0.118589	3.428285	1.085672	0.24408339	0.564405
380993	Zfat	zinc finger and AT hook domain containing, transcript	NA	-0.17458	3.057157	-1.12864	0.24411784	0.564405
70769	Nolc1	nucleolar and coiled-body phosphoprotein 1, transcrip	NA	-0.05901	6.553605	-1.04175	0.24421249	0.564494
17916	Myo1f	myosin IF	NA	-0.30673	0.64404	-1.2369	0.24425987	0.564494
268281	Shprh	SNF2 histone linker PHD RING helicase, transcript va	NA	0.092869	5.935876	1.066489	0.24426005	0.564494
12417	Cbx3	chromobox 3, transcript variant 1	NA	0.054427	8.59879	1.038447	0.24430031	0.564506
53871	Pkd2l2	polycystic kidney disease 2-like 2, transcript variant X	NA	0.428935	-0.1656	1.346239	0.24441803	0.564698
93762	Smarca5	SWI/SNF related, matrix associated, actin dependent	NA	-0.06339	6.821071	-1.04491	0.24458461	0.565003
434341	Nlrc5	NLR family, CARD domain containing 5, transcript va	NA	-0.55863	-0.63111	-1.47287	0.24466706	0.565113
19730	Ralgds	ral guanine nucleotide dissociation stimulator, transcr	NA	-0.06698	7.346507	-1.04752	0.24474752	0.565172
16571	Kif4	kinesin family member 4, transcript variant X3	NA	-0.1557	4.128903	-1.11396	0.24476211	0.565172
22591	Xpc	xeroderma pigmentosum, complementation group C	NA	0.096283	4.587004	1.069016	0.24487545	0.565227
232680	Cpa2	carboxypeptidase A2, pancreatic, transcript variant X	NA	-0.36244	0.545873	-1.2856	0.24488034	0.565227
18597	Pdha1	pyruvate dehydrogenase E1 alpha 1	NA	0.063339	7.240778	1.044881	0.24490131	0.565227
104394	E2f4	E2F transcription factor 4	NA	-0.07295	5.085248	-1.05186	0.24492471	0.565227
243813	Leng9	leukocyte receptor cluster (LRC) member 9	NA	-0.19893	1.866919	-1.14785	0.24509499	0.565488
14872	Gstt2	glutathione S-transferase, theta 2	NA	0.31015	0.625081	1.239836	0.24510721	0.565488
20583	Snai2	snail family zinc finger 2	NA	-0.2752	1.40366	-1.21016	0.24518255	0.565507
66905	Plin3	perilipin 3	NA	-0.12263	3.71527	-1.08872	0.24521597	0.565507
100732	Mapre3	microtubule-associated protein, RP/EB family, memb	NA	0.058655	6.713658	1.041494	0.24522005	0.565507
17202	Mc4r	melanocortin 4 receptor	NA	-0.17601	3.01352	-1.12975	0.24539222	0.565748
108657	Rnpepl1	arginyl aminopeptidase (aminopeptidase B)-like 1	NA	-0.0874	5.440491	-1.06245	0.24539398	0.565748
218490	Btf3	basic transcription factor 3, transcript variant 2	NA	0.052824	8.103179	1.037293	0.24560712	0.566101
14904	Gtpbp1	GTP binding protein 1	NA	-0.06425	6.455267	-1.04554	0.2456477	0.566101
102640858	Gm36826	predicted gene, 36826	NA	-0.37464	0.497891	-1.29652	0.24565136	0.566101
238690	Zfp458	zinc finger protein 458, transcript variant 2	NA	0.117107	3.497139	1.084558	0.24576203	0.566276
102636547	Gm33583	predicted gene, 33583, transcript variant X6	NA	-0.23217	2.008904	-1.1746	0.2458705	0.566324
74772	Atp13a2	ATPase type 13A2, transcript variant 2	NA	-0.06906	7.3357	-1.04903	0.24590139	0.566324
100042049	Gm15421	predicted gene 15421	NA	-0.13307	3.599179	-1.09662	0.24596953	0.566324
118568337	LOC118568337	igE-binding protein-like	NA	0.549891	-0.06379	1.463975	0.24597569	0.566324
74487	5430405H02Rik	RIKEN cDNA 5430405H02 gene, transcript variant 1	NA	-0.19345	2.16778	-1.1435	0.24603091	0.566324
232790	Oscar	osteoclast associated receptor, transcript variant 2	NA	0.30954	0.236218	1.239313	0.24604008	0.566324
226153	Twnk	twinkle mtDNA helicase, transcript variant 1	NA	-0.10868	4.715135	-1.07824	0.24604576	0.566324
101831	Faap24	Fanconi anemia core complex associated protein 24	NA	-0.1503	3.045223	-1.1098	0.24606156	0.566324

12013	Bach1	BTB and CNC homology 1, basic leucine zipper trans	NA	0.081118	6.176221	1.057837	0.24619536	0.566552
76074	Gbp8	guanylate-binding protein 8	NA	0.435997	-0.62698	1.352846	0.24632039	0.56676
170952	Prima1	proline rich membrane anchor 1, transcript variant X1	NA	-0.19473	2.009535	-1.14451	0.24637064	0.566795
56517	Slc22a21	solute carrier family 22 (organic cation transporter), m	NA	0.217767	2.015875	1.162932	0.24649866	0.566894
105246959	Gm42151	predicted gene, 42151, transcript variant 3	NA	-0.32987	0.533523	-1.2569	0.24650519	0.566894
66244	Nemf	nuclear export mediator factor	NA	0.080399	5.741149	1.05731	0.24651824	0.566894
115490131	Gm42517	predicted gene 42517	NA	0.137361	3.507024	1.099891	0.24658483	0.566967
70645	Oip5	Opa interacting protein 5	NA	0.193881	2.056165	1.143837	0.24663141	0.566994
54326	Elovl2	elongation of very long chain fatty acids (FEN1/Elo2,	NA	0.063978	6.085925	1.045344	0.24673303	0.567148
12291	Cacna1g	calcium channel, voltage-dependent, T type, alpha 1(	NA	-0.08091	6.619218	-1.05768	0.24683161	0.567294
73316	Calr3	calreticulin 3, transcript variant 1	NA	0.405583	-0.0664	1.324624	0.24687664	0.567318
76455	2310067E19Rik	RIKEN cDNA 2310067E19 gene	NA	-0.25525	2.856583	-1.19354	0.24698621	0.567447
328918	Zscan30	zinc finger and SCAN domain containing 30, transcrip	NA	0.173317	2.012191	1.127648	0.24701119	0.567447
16157	Il11ra1	interleukin 11 receptor, alpha chain 1, transcript varia	NA	-0.14115	3.854374	-1.10278	0.24705394	0.567447
102634512	Gm13791	predicted gene 13791, transcript variant X8	NA	0.371569	-0.25659	1.293759	0.24707432	0.567447
228846	D630003M21Rik	RIKEN cDNA D630003M21 gene, transcript variant 1	NA	0.165203	2.427213	1.121324	0.24714927	0.567447
69101	Ydjc	YdjC homolog (bacterial), transcript variant 1	NA	0.109702	3.708948	1.079005	0.24719643	0.567447
93888	Pcdhb17	protocadherin beta 17	NA	0.10297	4.010566	1.073982	0.24720821	0.567447
72137	Wdsub1	WD repeat, SAM and U-box domain containing 1, tra	NA	0.140633	3.314838	1.102389	0.24721199	0.567447
74349	Fam160a2	family with sequence similarity 160, member A2, tran	NA	-0.06795	5.661562	-1.04823	0.24727244	0.567506
67210	Gatad1	GATA zinc finger domain containing 1	NA	0.065482	6.019968	1.046435	0.24741794	0.567688
105246	Brd9	bromodomain containing 9, transcript variant 1	NA	-0.07208	6.610911	-1.05123	0.24742143	0.567688
67440	Mtpap	mitochondrial poly(A) polymerase, transcript variant 3	NA	0.112869	5.493393	1.081377	0.2474591	0.567694
75796	Cdyl2	chromodomain protein, Y chromosome-like 2	NA	0.145406	3.959848	1.106042	0.24751788	0.567735
21923	Tnc	tenascin C, transcript variant 5	NA	0.077323	7.772555	1.055059	0.24754688	0.567735
118567944	LOC118567944	uncharacterized LOC118567944	NA	-0.32814	0.818671	-1.2554	0.24766749	0.567932
110835	Chrna5	cholinergic receptor, nicotinic, alpha polypeptide 5, tr	NA	0.16808	2.328608	1.123562	0.24778982	0.568132
214254	Nudt15	nudix (nucleoside diphosphate linked moiety X)-type	NA	0.128193	3.104057	1.092924	0.2478652	0.568225
52463	Tet1	tet methylcytosine dioxygenase 1, transcript variant 1	NA	0.079899	5.321139	1.056944	0.24799221	0.56842
442834	D830031N03Rik	RIKEN cDNA D830031N03 gene	NA	0.299967	1.731921	1.231116	0.24801994	0.56842
52040	Ppp1r10	protein phosphatase 1, regulatory subunit 10, transcr	NA	-0.0829	6.289706	-1.05914	0.24805479	0.56842
100038489	Apela	apelin receptor early endogenous ligand	NA	-0.43277	-0.49358	-1.34982	0.24826542	0.568727
192166	Sardh	sarcosine dehydrogenase	NA	-0.13984	3.053404	-1.10178	0.24833803	0.568727
212281	Zfp729a	zinc finger protein 729a, transcript variant X5	NA	0.094464	4.331354	1.067669	0.24836608	0.568727
215085	Slc35f1	solute carrier family 35, member F1	NA	0.062953	6.908125	1.044602	0.24837696	0.568727
54128	Pmm2	phosphomannomutase 2, transcript variant 1	NA	0.112045	4.420982	1.080759	0.24838134	0.568727
320615	Dop1a	DOP1 leucine zipper like protein A, transcript variant	NA	0.065851	5.851902	1.046702	0.24843739	0.568727
15354	Hmgb3	high mobility group box 3, transcript variant 3	NA	0.052403	8.380611	1.036991	0.24845638	0.568727
16506	Kcnd1	potassium voltage-gated channel, Shal-related family	NA	0.131793	3.043715	1.095655	0.24846848	0.568727
16653	Kras	Kirsten rat sarcoma viral oncogene homolog	NA	0.066736	7.271308	1.047344	0.24861365	0.568971
211922	Dennd6a	DENN/MADD domain containing 6A, transcript variar	NA	-0.07603	5.156872	-1.05411	0.248659	0.568971
53605	Nap1l1	nucleosome assembly protein 1-like 1, transcript varia	NA	-0.04973	8.641719	-1.03507	0.24867989	0.568971
67272	Cmtm5	CKLF-like MARVEL transmembrane domain containi	NA	0.256703	0.960111	1.194745	0.24877655	0.569059
96935	Susd4	sushi domain containing 4, transcript variant 3	NA	0.076201	6.141638	1.054238	0.24878839	0.569059
102639598	Gm14296	predicted gene 14296, transcript variant 1	NA	-0.13855	4.962034	-1.1008	0.24894523	0.569308
66125	Sf3b5	splicing factor 3b, subunit 5	NA	-0.11539	4.26814	-1.08327	0.2489674	0.569308
98303	D630023F18Rik	RIKEN cDNA D630023F18 gene, transcript variant 2	NA	0.323421	0.766009	1.251295	0.24904312	0.569385
109079	Sephs1	selenophosphate synthetase 1, transcript variant 3	NA	0.065036	6.851726	1.046111	0.24910294	0.569385
80720	Pbx4	pre B cell leukemia homeobox 4, transcript variant 2	NA	0.123579	3.230591	1.089434	0.24910588	0.569385
108167377	LOC108167377	uncharacterized LOC108167377	NA	-0.27082	0.883559	-1.20649	0.24919778	0.569515
269336	Ccdc32	coiled-coil domain containing 32, transcript variant X1	NA	0.07748	4.793639	1.055173	0.24930733	0.569576
18162	Npr3	natriuretic peptide receptor 3, transcript variant X1	NA	0.132948	3.457645	1.096532	0.24932248	0.569576
68904	Abhd13	abhydrolase domain containing 13, transcript variant	NA	0.083715	5.191625	1.059743	0.24936145	0.569576
270201	Klhl18	kelch-like 18, transcript variant 1	NA	-0.07529	5.690514	-1.05357	0.24936701	0.569576
213211	Rnf26	ring finger protein 26	NA	-0.07767	5.037461	-1.05532	0.24939969	0.569576
100502841	Epg5	ectopic P-granules autophagy protein 5 homolog (C.	NA	0.100229	5.117727	1.071944	0.24954479	0.569828
21838	Thy1	thymus cell antigen 1, theta	NA	-0.1175	4.115538	-1.08485	0.24958552	0.569841
71790	Anxa9	annexin A9, transcript variant X3	NA	-0.24215	1.174591	-1.18275	0.2496412	0.569888
12677	Vsx2	visual system homeobox 2, transcript variant 2	NA	-0.28828	0.766793	-1.22118	0.24973516	0.570003
14165	Fgf10	fibroblast growth factor 10	NA	0.198439	2.09757	1.147456	0.24976143	0.570003
100504491	Dcdc2b	doublecortin domain containing 2b, transcript variant	NA	-0.21954	1.441784	-1.16437	0.24981609	0.570027
105246058	Gm41409	predicted gene, 41409	NA	0.670621	-0.75439	1.591759	0.2498423	0.570027
329575	Gm14325	predicted gene 14325	NA	0.11529	4.263465	1.083193	0.24998135	0.57018
17476	Mpeg1	macrophage expressed gene 1, transcript variant 2	NA	0.142224	3.007708	1.103605	0.25006301	0.57018
12823	Col19a1	collagen, type XIX, alpha 1	NA	0.166915	2.414896	1.122655	0.250068	0.57018
105663	Thtpa	thiamine triphosphatase	NA	0.067965	5.689215	1.048237	0.2500846	0.57018
102640081	Gm36236	predicted gene, 36236	NA	0.381642	0.115421	1.302824	0.25010586	0.57018
74187	Katnb1	katanin p80 (WD40-containing) subunit B 1, transcrip	NA	0.069094	5.454294	1.049058	0.25016638	0.57018

207781	C2cd2	C2 calcium-dependent domain containing 2	NA	-0.08457	4.463266	-1.06037	0.25017682	0.57018
239731	Rimbp3	RIMS binding protein 3	NA	0.223942	1.409393	1.167921	0.25026817	0.57018
14272	Fnta	farnesyltransferase, CAAX box, alpha	NA	0.063248	5.928793	1.044815	0.25033329	0.57018
241066	Carf	calcium response factor, transcript variant 4	NA	0.110685	4.182026	1.079741	0.25036107	0.57018
107831	Adgrb1	adhesion G protein-coupled receptor B1, transcript va	NA	-0.05565	7.378312	-1.03933	0.25037807	0.57018
71776	Tha1	threonine aldolase 1, transcript variant X1	NA	0.268964	1.131014	1.204943	0.25041687	0.57018
381107	Tmem232	transmembrane protein 232, transcript variant 4	NA	0.442201	-0.35514	1.358676	0.25045104	0.57018
72649	Tmem209	transmembrane protein 209, transcript variant 1	NA	-0.08728	5.245312	-1.06237	0.25047184	0.57018
51799	Rundc3a	RUN domain containing 3A, transcript variant 1	NA	-0.05312	7.507437	-1.03751	0.25049043	0.57018
12737	Cldn1	claudin 1	NA	0.130814	3.266242	1.094911	0.25049333	0.57018
319804	Glt1d1	glycosyltransferase 1 domain containing 1, transcript	NA	0.190057	3.777655	1.140808	0.25050508	0.57018
15193	Hdgfl2	HDGF like 2, transcript variant 2	NA	-0.06628	6.650651	-1.04701	0.25054062	0.570181
56470	Rgs19	regulator of G-protein signaling 19, transcript variant	NA	0.107634	4.379726	1.07746	0.2506499	0.57035
15551	Htr1b	5-hydroxytryptamine (serotonin) receptor 1B	NA	0.140823	3.424383	1.102534	0.25097276	0.570962
386612	Thoc6	THO complex 6, transcript variant 1	NA	-0.11043	4.024191	-1.07955	0.25107091	0.570962
214489	BC003965	cDNA sequence BC003965	NA	-0.07888	4.888598	-1.05619	0.25108964	0.570962
320965	4831440E17Rik	RIKEN cDNA 4831440E17 gene	NA	0.168911	2.304519	1.124209	0.25111726	0.570962
102632833	Gm30802	predicted gene, 30802	NA	-0.19724	1.769703	-1.1465	0.25112842	0.570962
67590	Tctn3	tectonic family member 3, transcript variant 1	NA	-0.11075	3.843138	-1.07979	0.25116345	0.570962
217069	Trim25	tripartite motif-containing 25	NA	-0.15933	2.951803	-1.11677	0.2512369	0.570962
12753	Clock	circadian locomotor output cycles kaput, transcript va	NA	0.094193	5.465324	1.067468	0.25126282	0.570962
227674	Ddx31	DEAD/H box helicase 31, transcript variant X2	NA	0.120082	3.77315	1.086797	0.25126606	0.570962
80891	Fcrls	Fc receptor-like S, scavenger receptor, transcript vari	NA	-0.13153	3.137522	-1.09546	0.25131658	0.570962
13840	Epha6	Eph receptor A6	NA	0.168632	2.601211	1.123992	0.25134193	0.570962
100504687	2810029C07Rik	RIKEN cDNA 2810029C07 gene	NA	0.130978	3.667897	1.095036	0.25138659	0.570962
15257	Hipk1	homeodomain interacting protein kinase 1, transcript	NA	0.057679	6.722293	1.04079	0.25139973	0.570962
109095	Rbm15b	RNA binding motif protein 15B	NA	-0.05386	7.327103	-1.03804	0.25145796	0.570962
98952	Fam102a	family with sequence similarity 102, member A	NA	-0.10199	4.668736	-1.07325	0.25149373	0.570962
53609	Clasrp	CLK4-associating serine/arginine rich protein, transcr	NA	-0.06362	6.112889	-1.04509	0.25150266	0.570962
214944	Mob3b	MOB kinase activator 3B, transcript variant X1	NA	-0.17941	2.758119	-1.13242	0.25157389	0.570962
20972	Syngr1	synaptogyrin 1, transcript variant 1a	NA	-0.06352	6.778624	-1.04501	0.2515808	0.570962
235439	Herc1	HECT and RLD domain containing E3 ubiquitin prote	NA	0.058736	7.467756	1.041553	0.25158523	0.570962
73728	Psd	pleckstrin and Sec7 domain containing, transcript var	NA	0.068569	7.756809	1.048676	0.25168298	0.571104
100504262	A730020E08Rik	RIKEN cDNA A730020E08 gene	NA	-0.1708	2.353224	-1.12568	0.25171917	0.571106
13645	Egf	epidermal growth factor, transcript variant 2	NA	0.252026	1.271671	1.190878	0.25178046	0.571166
233274	Siglech	sialic acid binding Ig-like lectin H, transcript variant X	NA	0.268651	0.738302	1.204681	0.25190566	0.571352
107566	Arl2bp	ADP-ribosylation factor-like 2 binding protein, transcri	NA	-0.05723	7.034518	-1.04047	0.25193289	0.571352
243538	Cfap100	cilia and flagella associated protein 100, transcript va	NA	-0.13437	2.961491	-1.09761	0.25223913	0.571937
81006	Gpr63	G protein-coupled receptor 63, transcript variant X1	NA	0.125591	3.431226	1.090954	0.2522902	0.571937
14314	Fstl1	follistatin-like 1	NA	0.058691	7.150184	1.04152	0.25229605	0.571937
13990	Smarcad1	SWI/SNF-related, matrix-associated actin-dependent	NA	0.077962	6.554625	1.055526	0.25240863	0.572112
210356	Nckap5	NCK-associated protein 5, transcript variant 2	NA	-0.10696	5.204845	-1.07696	0.25246983	0.572171
76719	Kansl1	KAT8 regulatory NSL complex subunit 1, transcript va	NA	0.053582	6.848214	1.037839	0.25260707	0.572402
73112	Abracl	ABRA C-terminal like, transcript variant 1	NA	-0.08158	5.580476	-1.05817	0.25275413	0.572517
268567	Tmem229b	transmembrane protein 229B, transcript variant X5	NA	-0.06424	7.206802	-1.04553	0.25276919	0.572517
225995	D030056L22Rik	RIKEN cDNA D030056L22 gene, transcript variant 1	NA	-0.0707	5.464619	-1.05023	0.25278344	0.572517
20955	Vamp7	vesicle-associated membrane protein 7, transcript va	NA	-0.08686	5.269383	-1.06206	0.25279849	0.572517
20540	Slc7a7	solute carrier family 7 (cationic amino acid transporte	NA	-0.25101	2.122667	-1.19004	0.25289262	0.572582
72201	Otud6b	OTU domain containing 6B	NA	0.067018	5.77544	1.047549	0.25289726	0.572582
234857	Spire2	spire type actin nucleation factor 2	NA	-0.08172	5.079681	-1.05828	0.252944	0.572608
22695	Zfp36	zinc finger protein 36	NA	-0.17103	2.630172	-1.12587	0.25308136	0.572805
101185	Pot1a	protection of telomeres 1A, transcript variant X2	NA	-0.09178	4.458337	-1.06568	0.25310172	0.572805
71984	Sars2	seryl-aminoacyl-tRNA synthetase 2	NA	-0.14914	3.468747	-1.10891	0.25314727	0.572829
267019	Rps15a	ribosomal protein S15A	NA	0.051014	8.248477	1.035993	0.25324545	0.572971
12721	Coro1a	coronin, actin binding protein 1A, transcript variant 2	NA	0.084855	5.404653	1.060581	0.25331798	0.573056
66419	Mrpl11	mitochondrial ribosomal protein L11	NA	0.065191	5.657098	1.046224	0.25338922	0.573137
319565	Syne2	spectrin repeat containing, nuclear envelope 2	NA	-0.09029	6.331377	-1.06459	0.25348433	0.573273
380683	Sec14l3	SEC14-like lipid binding 3	NA	0.232791	1.252486	1.175106	0.25353335	0.573304
100042237	Gm3740	predicted gene 3740, transcript variant X8	NA	-0.30593	1.022848	-1.23621	0.25364118	0.573468
230971	Megf6	multiple EGF-like-domains 6, transcript variant X3	NA	-0.09429	4.52037	-1.06754	0.25372328	0.573574
12858	Cox5a	cytochrome c oxidase subunit 5A	NA	0.104266	6.715184	1.074947	0.25384364	0.573766
19344	Rab5b	RAB5B, member RAS oncogene family	NA	-0.05342	7.408034	-1.03772	0.25394151	0.573862
74091	Npl	N-acetylneuraminate pyruvate lyase	NA	0.158323	2.781226	1.115989	0.25395633	0.573862
192160	Casc3	cancer susceptibility candidate 3	NA	-0.06204	7.03119	-1.04394	0.25420688	0.574348
329506	Ctdspl2	CTD (carboxy-terminal domain, RNA polymerase II, p	NA	0.109883	5.830989	1.079141	0.25424871	0.574363
108168877	Gm46810	predicted gene, 46810	NA	-0.19451	2.343729	-1.14434	0.25436132	0.574537
77798	A930009A15Rik	RIKEN cDNA A930009A15 gene, transcript variant X	NA	0.421719	-0.09872	1.339523	0.2544276	0.574607
73095	Slc25a42	solute carrier family 25, member 42	NA	-0.12863	3.712743	-1.09325	0.25448165	0.57465

235582	Glyctk	glycerate kinase, transcript variant X2	NA	-0.28827	0.853489	-1.22118	0.25452794	0.574661
226041	Pgm5	phosphoglucomutase 5	NA	0.125105	3.769286	1.090587	0.25465577	0.574661
71276	Ccdc57	coiled-coil domain containing 57, transcript variant X9	NA	0.113599	3.879308	1.081924	0.25468466	0.574661
20005	Rpl9	ribosomal protein L9	NA	0.061513	8.693745	1.04356	0.25472673	0.574661
71718	Telo2	telomere maintenance 2, transcript variant X3	NA	-0.08483	5.098976	-1.06056	0.25473658	0.574661
115488526	Gm52213	predicted gene, 52213	NA	-0.24572	1.182999	-1.18569	0.25477778	0.574661
100986	Akap9	A kinase (PRKA) anchor protein (yotiao) 9	NA	0.070651	8.045623	1.05019	0.25480232	0.574661
12048	Bcl2l1	BCL2-like 1, transcript variant 1	NA	-0.05353	6.853949	-1.0378	0.25480592	0.574661
110637	Grik4	glutamate receptor, ionotropic, kainate 4, transcript va	NA	0.127619	4.073728	1.092489	0.25480638	0.574661
217124	Ppp1r9b	protein phosphatase 1, regulatory subunit 9B	NA	-0.06214	8.332731	-1.04401	0.25483973	0.574661
67846	Tmem39a	transmembrane protein 39a, transcript variant X3	NA	-0.11055	4.048522	-1.07964	0.25496973	0.574863
26912	Gcat	glycine C-acetyltransferase (2-amino-3-ketobutyrate-c	NA	-0.15511	2.756492	-1.11351	0.25502867	0.574863
66625	Pnisr	PNN interacting serine/arginine-rich, transcript varian	NA	0.056241	7.789034	1.039753	0.2550353	0.574863
70047	Trnt1	tRNA nucleotidyl transferase, CCA-adding, 1, transcr	NA	0.072604	5.497543	1.051613	0.25515857	0.574965
24014	Rnasel	ribonuclease L (2', 5'-oligoisoadenylate synthetase-de	NA	0.0686	6.027834	1.048698	0.2551607	0.574965
28109	D10Wsu102e	DNA segment, Chr 10, Wayne State University 102, e	NA	0.07095	5.319302	1.050408	0.25518965	0.574965
13346	Des	desmin	NA	-0.31506	2.200955	-1.24406	0.25522188	0.574965
16885	Limk1	LIM-domain containing, protein kinase, transcript vari	NA	-0.06791	6.676833	-1.0482	0.25529937	0.575011
72997	Kantr	Kdm5c adjacent non-coding transcript, transcript varia	NA	-0.10133	4.910316	-1.07276	0.25531305	0.575011
13009	Csrp3	cysteine and glycine-rich protein 3, transcript variant	NA	-0.48792	-0.18668	-1.40242	0.25541033	0.575079
68971	Tamm41	TAM41 mitochondrial translocator assembly and main	NA	0.126699	3.893668	1.091793	0.25542036	0.575079
77407	Rab35	RAB35, member RAS oncogene family	NA	-0.07467	6.622149	-1.05312	0.25546599	0.575079
56075	Pdss1	prenyl (solanesyl) diphosphate synthase, subunit 1, tr	NA	0.100088	4.226081	1.071839	0.2554848	0.575079
11750	Anxa7	annexin A7, transcript variant 2	NA	-0.12462	4.370879	-1.09022	0.25553215	0.575106
432855	Zfhx2os	zinc finger homeobox 2, opposite strand	NA	-0.15822	3.657666	-1.11591	0.25560812	0.575127
66586	Crls1	cardiolipin synthase 1, transcript variant 2	NA	-0.07737	4.991599	-1.05509	0.2556221	0.575127
211323	Nrg1	neuregulin 1, transcript variant X22	NA	-0.10371	5.144451	-1.07453	0.25564733	0.575127
216971	Fam222b	family with sequence similarity 222, member B, transc	NA	-0.06949	5.639417	-1.04934	0.25573301	0.57524
76804	Kdm4c	lysine (K)-specific demethylase 4C, transcript variant	NA	0.075357	5.176793	1.053622	0.25581832	0.57528
67958	U2surp	U2 snRNP-associated SURP domain containing, tran	NA	0.058572	7.056081	1.041434	0.25582129	0.57528
225256	Dsg1b	desmoglein 1 beta, transcript variant X1	NA	-0.77433	-1.13912	-1.7104	0.25588787	0.57535
321003	Xpnpep3	X-prolyl aminopeptidase 3, mitochondrial, transcript v	NA	0.102416	4.166393	1.07357	0.25608351	0.57571
668225	Fignl2	fidgetin-like 2, transcript variant X2	NA	0.224692	1.656481	1.168528	0.25619477	0.575881
69349	1700008O03Rik	RIKEN cDNA 1700008O03 gene, transcript variant X	NA	0.514583	-0.15293	1.428581	0.25623121	0.575883
70292	Afap1	actin filament associated protein 1, transcript variant	NA	0.051527	7.105727	1.036361	0.2564136	0.576139
28080	Atp5o	ATP synthase, H+ transporting, mitochondrial F1 com	NA	0.070156	7.359374	1.049831	0.25641597	0.576139
16658	Mafb	v-maf musculoaponeurotic fibrosarcoma oncogene fa	NA	-0.08304	4.725646	-1.05925	0.25649383	0.576195
102635588	Gm32881	predicted gene, 32881, transcript variant X1	NA	0.4773	-0.47197	1.392136	0.25654698	0.576195
74019	Traf3ip1	TRAF3 interacting protein 1	NA	0.08156	4.706673	1.058161	0.2565471	0.576195
105246278	B230354K17Rik	RIKEN cDNA B230354K17 gene, transcript variant 2	NA	0.08809	4.702498	1.062962	0.25666305	0.576376
15425	Hoxc6	homeobox C6	NA	0.623518	-0.79294	1.540627	0.25675242	0.576421
72047	Ddx42	DEAD box helicase 42, transcript variant 1	NA	0.050767	7.158165	1.035815	0.25675371	0.576421
246081	Defb11	defensin beta 11	NA	-0.36587	-0.16929	-1.28866	0.25683084	0.576514
11797	Birc2	baculoviral IAP repeat-containing 2, transcript variant	NA	-0.07696	5.454882	-1.05479	0.25691141	0.576562
12322	Camk2a	calcium/calmodulin-dependent protein kinase II alpha	NA	-0.08831	5.960753	-1.06313	0.25692298	0.576562
102639765	Gm29260	predicted gene 29260, transcript variant X14	NA	-0.15139	3.957686	-1.11064	0.25698963	0.576583
73919	Lyrm1	LYR motif containing 1, transcript variant X8	NA	0.166054	2.748096	1.121985	0.25700314	0.576583
105246056	Gm9828	predicted gene 9828	NA	-0.29848	0.762773	-1.22985	0.2571022	0.576726
105246406	Gm41698	predicted gene, 41698, transcript variant X1	NA	0.353441	0.289394	1.277604	0.25714353	0.576739
72395	2610028E06Rik	RIKEN cDNA 2610028E06 gene	NA	0.329356	0.158413	1.256453	0.25720513	0.576797
20660	Sorl1	sortilin-related receptor, LDLR class A repeats-contai	NA	-0.06905	5.841377	-1.04903	0.25735026	0.576979
21824	Thbd	thrombomodulin	NA	-0.14292	3.645541	-1.10414	0.25735699	0.576979
212427	A730008H23Rik	RIKEN cDNA A730008H23 gene	NA	-0.18958	3.027982	-1.14043	0.25747059	0.577112
100125933	2310075K07Rik	RIKEN cDNA 2310075K07 gene	NA	0.37918	-0.14926	1.300602	0.25758687	0.577112
75725	Phf14	PHD finger protein 14, transcript variant X29	NA	0.053936	7.500129	1.038093	0.25760337	0.577112
54650	Sfmbt1	Scm-like with four mbt domains 1, transcript variant X	NA	0.090204	5.438444	1.06452	0.25765528	0.577112
65254	Dpysl5	dihydropyrimidinase-like 5, transcript variant 2	NA	-0.05426	9.676362	-1.03833	0.25766389	0.577112
71715	Dhx35	DEAH (Asp-Glu-Ala-His) box polypeptide 35, transcri	NA	-0.10305	4.419399	-1.07404	0.25771216	0.577112
67500	Ccar1	cell division cycle and apoptosis regulator 1, transcrip	NA	0.05394	6.973387	1.038096	0.2577419	0.577112
108168069	Gm46404	predicted gene, 46404	NA	-0.21832	1.407525	-1.16338	0.25776467	0.577112
74055	Plce1	phospholipase C, epsilon 1	NA	0.099131	4.383952	1.071128	0.25777557	0.577112
217664	Mgat2	mannoside acetylglucosaminyltransferase 2	NA	0.072222	5.351621	1.051335	0.25785504	0.577112
110253	Triobp	TRIO and F-actin binding protein, transcript variant 1	NA	-0.06652	6.377913	-1.04719	0.2578619	0.577112
12562	Cdh5	cadherin 5	NA	-0.06811	5.688513	-1.04834	0.25786866	0.577112
55963	Slc1a4	solute carrier family 1 (glutamate/neutral amino acid t	NA	0.052385	6.833549	1.036978	0.25787837	0.577112
22715	Zfp57	zinc finger protein 57, transcript variant 4	NA	0.052613	8.212108	1.037142	0.25791307	0.577112
100040298	Gm15501	predicted pseudogene 15501, transcript variant 1	NA	0.070514	6.285732	1.05009	0.2580409	0.577319
59091	Jph2	junctophilin 2, transcript variant 2	NA	-0.30147	1.466903	-1.2324	0.25808427	0.577337

68281	4930430F08Rik	RIKEN cDNA 4930430F08 gene	NA		0.127726	3.828925	1.09257	0.25818199	0.577476
69928	Cenps	centromere protein S	NA		0.1656	2.52035	1.121632	0.25821789	0.577477
67705	1810058I24Rik	RIKEN cDNA 1810058I24 gene, transcript variant 3	NA		0.092302	4.303502	1.06607	0.25846975	0.577853
70274	Ly6g6e	lymphocyte antigen 6 complex, locus G6E	NA		-0.30026	0.737903	-1.23137	0.2584892	0.577853
16840	Cnmd	chondromodulin, transcript variant 1	NA		-0.29665	1.594747	-1.22829	0.2584928	0.577853
270210	Zfp651	zinc finger protein 651, transcript variant X1	NA		-0.07373	5.79914	-1.05244	0.2585765	0.577961
74753	Trmo	tRNA methyltransferase O, transcript variant 2	NA		0.171881	2.502322	1.126527	0.2586682	0.578087
100040462	Mndal	myeloid nuclear differentiation antigen like, transcript	NA		0.201079	1.568443	1.149558	0.25883452	0.578354
106648	Cyp4f15	cytochrome P450, family 4, subfamily f, polypeptide 1	NA		0.27197	0.762596	1.207455	0.25885895	0.578354
66467	Gtf2h5	general transcription factor IIH, polypeptide 5, transcr	NA		0.061004	6.007254	1.043192	0.2591195	0.578857
77318	Ankrd55	ankyrin repeat domain 55, transcript variant 1	NA		0.197602	3.035014	1.146791	0.25931343	0.57921
115489723	LOC115489723	uncharacterized LOC115489723	NA		-0.45942	0.092782	-1.37499	0.25939129	0.579305
268709	Fam107a	family with sequence similarity 107, member A, transc	NA		0.237924	1.725404	1.179295	0.2595631	0.579564
229228	Nudt6	nudix (nucleoside diphosphate linked moiety X)-type	NA		0.289648	1.575106	1.222342	0.25963311	0.579564
99683	Sec24b	Sec24 related gene family, member B (S. cerevisiae)	NA		-0.05802	6.438324	-1.04104	0.25963462	0.579564
18142	Npas1	neuronal PAS domain protein 1	NA		0.197926	2.497825	1.147048	0.25964993	0.579564
330836	Slc7a6	solute carrier family 7 (cationic amino acid transporte	NA		0.070416	5.766323	1.050019	0.2597935	0.579779
76007	Zmym2	zinc finger, MYM-type 2, transcript variant 1	NA		0.065329	7.042672	1.046323	0.25983954	0.579779
223921	Aaas	achalasia, adrenocortical insufficiency, alacrimia	NA		-0.08438	4.579307	-1.06023	0.25986365	0.579779
15040	H2-T23	histocompatibility 2, T region locus 23	NA		0.187477	1.730203	1.138771	0.25988846	0.579779
382985	Rrm2b	ribonucleotide reductase M2 B (TP53 inducible), tran	NA		0.097845	4.210325	1.070173	0.26009999	0.580118
56043	Akr1e1	aldo-keto reductase family 1, member E1	NA		-0.09939	4.656485	-1.07132	0.26012514	0.580118
77040	Atg16l1	autophagy related 16-like 1 (S. cerevisiae), transcript	NA		0.064714	5.61017	1.045878	0.26014741	0.580118
102632262	Gm30388	predicted gene, 30388, transcript variant X2	NA		0.385947	0.02892	1.306718	0.26018984	0.580133
233489	Picalm	phosphatidylinositol binding clathrin assembly protein	NA		0.055088	7.915943	1.038922	0.26023652	0.580157
77739	Adamtsl1	ADAMTS-like 1, transcript variant X8	NA		0.156845	3.108042	1.114846	0.26028204	0.580179
53817	Ddx39b	DEAD box helicase 39b, transcript variant 1	NA		0.050203	8.484431	1.03541	0.26037572	0.580309
13025	Ctla2b	cytotoxic T lymphocyte-associated protein 2 beta, tra	NA		-0.33267	0.020979	-1.25934	0.26044253	0.580344
16532	Kcnu1	potassium channel, subfamily U, member 1, transcrip	NA		0.155681	2.46316	1.113947	0.26046311	0.580344
14230	Fkbp10	FK506 binding protein 10, transcript variant 1	NA		0.085195	4.629221	1.060831	0.26077828	0.580947
69217	Plekha4	pleckstrin homology domain containing, family A (pho	NA		0.236036	1.385406	1.177752	0.26080515	0.580947
73216	B9d1os	B9 protein domain 1, opposite strand	NA		0.658945	-0.54489	1.578928	0.26091351	0.58103
118568186	LOC118568186	uncharacterized LOC118568186	NA		0.276769	0.694322	1.211478	0.26091364	0.58103
69876	Thap3	THAP domain containing, apoptosis associated prote	NA		0.103695	3.667212	1.074522	0.26098512	0.581035
64704	Htra2	HtrA serine peptidase 2	NA		-0.08532	4.658798	-1.06093	0.2609954	0.581035
240753	Plekha6	pleckstrin homology domain containing, family A men	NA		-0.0678	6.510513	-1.04812	0.26102281	0.581035
20501	Slc16a1	solute carrier family 16 (monocarboxylic acid transpo	NA		0.094982	4.469108	1.068052	0.26110151	0.581041
102642625	Gm38620	predicted gene, 38620, transcript variant X2	NA		-0.40897	0.287815	-1.32774	0.26112087	0.581041
17120	Mad1l1	MAD1 mitotic arrest deficient 1-like 1, transcript varia	NA		-0.1327	4.560762	-1.09634	0.26113276	0.581041
12014	Bach2	BTB and CNC homology, basic leucine zipper transcr	NA		-0.06214	6.948802	-1.04401	0.26118944	0.581048
381644	Cep135	centrosomal protein 135, transcript variant X5	NA		-0.12914	3.428108	-1.09364	0.26120717	0.581048
66409	Rsl1d1	ribosomal L1 domain containing 1	NA		0.068716	6.708111	1.048783	0.26131371	0.581069
627049	Zfp800	zinc finger protein 800, transcript variant X8	NA		-0.09919	4.322107	-1.07117	0.26131751	0.581069
66938	Sh3d21	SH3 domain containing 21, transcript variant 2	NA		0.312871	0.738075	1.242178	0.26132539	0.581069
98238	Lrrc59	leucine rich repeat containing 59	NA		-0.06044	7.199327	-1.04278	0.2613598	0.581069
67732	Iah1	isoamyl acetate-hydrolyzing esterase 1 homolog	NA		-0.14006	3.419925	-1.10195	0.26141352	0.581109
227753	Gsn	gelsolin, transcript variant 2	NA		-0.08529	4.439149	-1.0609	0.26155993	0.581282
434784	Ldoc1	regulator of NFKB signaling	NA		0.243265	1.115148	1.183669	0.26156283	0.581282
14705	Bscl2	Berardinelli-Seip congenital lipodystrophy 2 (seipin), t	NA		-0.06902	5.665007	-1.049	0.26161499	0.581319
78412	Cyren	cell cycle regulator of NHEJ, transcript variant X1	NA		-0.13735	3.08157	-1.09988	0.26178898	0.581595
226016	Abhd17b	abhydrolase domain containing 17B	NA		0.088609	5.539802	1.063344	0.26182862	0.581595
78108	Particl	promoter of Mat2a antisense radiation induced circula	NA		0.11991	4.291325	1.086667	0.26185362	0.581595
213469	Lgi3	leucine-rich repeat LGI family, member 3	NA		0.205316	2.046663	1.152938	0.26188198	0.581595
208151	Tmem132b	transmembrane protein 132B	NA		0.058259	6.011136	1.041209	0.26201511	0.581749
18223	Numbl	numb-like, transcript variant X4	NA		-0.06289	6.363572	-1.04455	0.26204349	0.581749
118568386	LOC118568386	uncharacterized LOC118568386	NA		-0.5399	-0.1571	-1.45387	0.26205865	0.581749
56376	Pdlim5	PDZ and LIM domain 5, transcript variant 6	NA		0.093153	4.642722	1.066699	0.26221313	0.58194
13638	Efna3	ephrin A3, transcript variant 1	NA		0.075696	5.415609	1.05387	0.26221618	0.58194
20351	Sema4a	sema domain, immunoglobulin domain (Ig), transmen	NA		-0.08081	5.177111	-1.05761	0.26227625	0.581986
75029	Purg	purine-rich element binding protein G, transcript varia	NA		-0.0792	5.7825	-1.05643	0.26232504	0.581986
217827	Nrde2	nrde-2 necessary for RNA interference, domain conta	NA		-0.09687	4.304043	-1.06945	0.2623443	0.581986
74694	Tbc1d30	TBC1 domain family, member 30, transcript variant X	NA		0.063123	5.937881	1.044725	0.26244028	0.582119
68877	Maf1	MAF1 homolog, negative regulator of RNA polymeras	NA		0.061513	6.46281	1.04356	0.262495	0.582161
74610	Abcb8	ATP-binding cassette, sub-family B (MDR/TAP), men	NA		-0.09912	4.984709	-1.07112	0.26264543	0.582358
76131	Depdc1a	DEP domain containing 1a, transcript variant 3	NA		-0.19629	3.06287	-1.14575	0.26265527	0.582358
381284	Crocc2	ciliary rootlet coiled-coil, rootletin family member 2, tra	NA		0.295137	1.722763	1.227002	0.26283235	0.582671
52846	Cnot11	CCR4-NOT transcription complex, subunit 11	NA		-0.06998	5.310997	-1.0497	0.26291154	0.582768
216440	Os9	amplified in osteosarcoma, transcript variant 2	NA		-0.06234	7.08156	-1.04416	0.26297155	0.582811

227095	Hibch	3-hydroxyisobutyryl-Coenzyme A hydrolase	NA	0.091155	4.07919	1.065223	0.26300597	0.582811
381438	Gm5148	predicted gene 5148	NA	-0.13946	3.189928	-1.10149	0.2630757	0.582811
28040	D6Wsu163e	DNA segment, Chr 6, Wayne State University 163, e›	NA	-0.07808	4.838467	-1.05561	0.26312138	0.582811
69824	Glod5	glyoxalase domain containing 5, transcript variant X1	NA	0.460573	-0.7407	1.376089	0.26312677	0.582811
100039707	Mthfsl	5, 10-methenyltetrahydrofolate synthetase-like, transc	NA	0.164101	2.88003	1.120468	0.26314625	0.582811
83922	Cep41	centrosomal protein 41, transcript variant X8	NA	-0.11922	4.259731	-1.08615	0.26332702	0.583132
54632	Ftsj1	FtsJ RNA methyltransferase homolog 1 (E. coli), tran	NA	-0.08502	4.791879	-1.0607	0.26344752	0.583252
320678	Iffo1	intermediate filament family orphan 1, transcript varia	NA	-0.0798	4.681567	-1.05687	0.26345289	0.583252
245860	Atg9a	autophagy related 9A, transcript variant 2	NA	-0.0721	5.930371	-1.05125	0.26358342	0.583462
20362	Septin8	septin 8, transcript variant 1	NA	-0.06373	6.058892	-1.04516	0.2636484	0.583526
79555	BC005537	cDNA sequence BC005537	NA	0.074323	7.466168	1.052867	0.26375501	0.583616
101359	Prrt4	proline-rich transmembrane protein 4	NA	0.110408	3.980373	1.079533	0.26379548	0.583616
60597	Mapk8ip2	mitogen-activated protein kinase 8 interacting protein	NA	-0.06877	6.937225	-1.04882	0.26384949	0.583616
102634716	4930520E11Rik	RIKEN cDNA 4930520E11 gene	NA	-0.21492	1.72657	-1.16064	0.26392492	0.583616
102632031	Gm15728	predicted gene 15728	NA	0.212341	1.389298	1.158567	0.26398063	0.583616
100040870	Gm3005	predicted gene 3005, transcript variant 3	NA	0.154752	4.221278	1.11323	0.26398859	0.583616
100039332	Gm2164	predicted gene 2164	NA	0.122158	3.265722	1.088361	0.26401472	0.583616
268301	Sowahc	sosondowah ankyrin repeat domain family member C	NA	-0.09559	4.923306	-1.0685	0.26408377	0.583616
16068	Il18bp	interleukin 18 binding protein, transcript variant X2	NA	-0.10399	3.672549	-1.07474	0.26416185	0.583616
17777	Mttp	microsomal triglyceride transfer protein, transcript var	NA	-0.15731	2.958284	-1.1152	0.26416199	0.583616
15364	Hmga2	high mobility group AT-hook 2, transcript variant 1	NA	-0.14436	2.861917	-1.10524	0.26416268	0.583616
74309	Osbp2	oxysterol binding protein 2, transcript variant X29	NA	0.077268	5.085334	1.055018	0.2642148	0.583616
72508	Rps6kb1	ribosomal protein S6 kinase, polypeptide 1, transcript	NA	0.065236	6.264054	1.046256	0.26421647	0.583616
19130	Prox1	prospero homeobox 1, transcript variant X2	NA	-0.07153	5.560478	-1.05083	0.26429385	0.583616
118567401	LOC118567401	uncharacterized LOC118567401	NA	0.501447	-0.47544	1.415632	0.26429572	0.583616
108167831	Gm46241	predicted gene, 46241	NA	0.299457	0.868939	1.230681	0.26429659	0.583616
329977	Fhad1	forkhead-associated (FHA) phosphopeptide binding c	NA	0.23242	1.789448	1.174804	0.26429869	0.583616
234854	Cdk10	cyclin-dependent kinase 10, transcript variant 1	NA	-0.07923	5.222499	-1.05645	0.26452258	0.584031
107197	Uqcc3	ubiquinol-cytochrome c reductase complex assembly	NA	0.112737	3.517042	1.081278	0.26458979	0.584101
12817	Col13a1	collagen, type XIII, alpha 1, transcript variant X22	NA	-0.17256	2.341994	-1.12706	0.26468188	0.584133
102871	Radx	RPA1 related single stranded DNA binding protein, X	NA	0.293894	0.875098	1.225944	0.2647107	0.584133
13714	Elk4	ELK4, member of ETS oncogene family, transcript va	NA	0.201366	2.388549	1.149786	0.26471226	0.584133
107368	Pdzd8	PDZ domain containing 8	NA	0.087903	5.999887	1.062825	0.2648478	0.58433
238247	Arid4a	AT rich interactive domain 4A (RBP1-like), transcript	NA	0.065093	5.684341	1.046153	0.26494328	0.58433
24001	Tiam2	T cell lymphoma invasion and metastasis 2, transcrip	NA	-0.10971	6.668574	-1.07901	0.26494726	0.58433
107503	Atf5	activating transcription factor 5, transcript variant 2	NA	-0.11399	4.841765	-1.08222	0.26494894	0.58433
19207	Ptch2	patched 2, transcript variant 1	NA	-0.14412	3.15774	-1.10506	0.26498184	0.58433
12554	Cdh13	cadherin 13, transcript variant X1	NA	0.053652	6.977528	1.037889	0.2650459	0.58433
319783	A730056A06Rik	RIKEN cDNA A730056A06 gene, transcript variant 2	NA	0.142919	2.832644	1.104137	0.26506404	0.58433
102635441	Gm32778	predicted gene, 32778, transcript variant X3	NA	-0.52576	-0.46832	-1.43969	0.26508863	0.58433
66495	Ndufb3	NADH:ubiquinone oxidoreductase subunit B3	NA	-0.08268	5.325541	-1.05898	0.26516693	0.584423
57808	Rpl35a	ribosomal protein L35A, transcript variant 1	NA	0.048828	7.718523	1.034424	0.26521274	0.584445
66369	Dus2	dihydrouridine synthase 2, transcript variant 1	NA	-0.1214	3.75677	-1.08779	0.26531444	0.584551
68988	Prpf31	pre-mRNA processing factor 31, transcript variant 1	NA	-0.06547	5.730057	-1.04643	0.26533249	0.584551
233812	Mosmo	modulator of smoothened	NA	0.092182	5.467278	1.065981	0.26549905	0.584838
225887	Ndufs8	NADH:ubiquinone oxidoreductase core subunit S8, tr	NA	0.086183	5.294655	1.061558	0.26554637	0.584863
14828	Hspa5	heat shock protein 5, transcript variant 1	NA	-0.05609	8.333064	-1.03964	0.26573117	0.58519
13809	Enpep	glutamyl aminopeptidase	NA	0.174946	1.865163	1.128922	0.26576678	0.58519
18771	Pknox1	Pbx/knotted 1 homeobox, transcript variant 1	NA	0.078774	4.943382	1.05612	0.26588745	0.585377
378776	Pcsk2os1	proprotein convertase subtilisin/kexin type 2, opposite	NA	0.213265	1.255765	1.159309	0.26603111	0.585614
171210	Acot2	acyl-CoA thioesterase 2	NA	0.175052	2.594751	1.129005	0.266144	0.585654
56068	Ammecr1	Alport syndrome, mental retardation, midface hypopla	NA	0.113963	3.760449	1.082197	0.26614842	0.585654
67070	Lsm14a	LSM14A mRNA processing body assembly factor, tra	NA	-0.05587	6.687171	-1.03948	0.26615739	0.585654
20318	Sdf4	stromal cell derived factor 4, transcript variant 2	NA	0.066012	6.655361	1.046819	0.2663	0.585889
353499	Tmc4	transmembrane channel-like gene family 4	NA	-0.1746	2.543176	-1.12865	0.2665445	0.586312
103406	Zfr2	zinc finger RNA binding protein 2	NA	-0.08462	4.879112	-1.06041	0.26656445	0.586312
74023	Rd3	retinal degeneration 3, transcript variant 1	NA	-0.24435	1.106606	-1.18456	0.26665157	0.58638
194309	Vps37d	vacuolar protein sorting 37D, transcript variant 1	NA	0.095916	5.025547	1.068744	0.2666951	0.58638
18100	Mrpl40	mitochondrial ribosomal protein L40	NA	0.122788	4.402027	1.088837	0.26672065	0.58638
102638859	Gm35321	predicted gene, 35321, transcript variant X1	NA	0.245732	1.348175	1.185694	0.26673945	0.58638
72729	Cdc42se2	CDC42 small effector 2	NA	0.058789	7.008033	1.041591	0.2668144	0.586465
12818	Col14a1	collagen, type XIV, alpha 1, transcript variant 2	NA	-0.15239	2.941784	-1.11141	0.26685331	0.586467
234878	Map3k21	mitogen-activated protein kinase kinase kinase 21, tr	NA	0.225538	1.382326	1.169213	0.26691656	0.586467
329641	Sertm1	serine rich and transmembrane domain containing 1	NA	0.098191	4.44473	1.07043	0.26692307	0.586467
66643	Lix1	limb and CNS expressed 1	NA	-0.08168	5.341096	-1.05825	0.26708285	0.586739
11891	Rab27a	RAB27A, member RAS oncogene family, transcript v	NA	-0.16628	2.212015	-1.12216	0.26723608	0.586996
330164	C130026L21Rik	RIKEN cDNA C130026L21 gene	NA	0.289342	0.579056	1.222082	0.26755991	0.587628
17118	Marcks	myristoylated alanine rich protein kinase C substrate	NA	0.057937	10.29485	1.040976	0.26773709	0.587938

11607	Agtr1a	angiotensin II receptor, type 1a	NA	0.353746	0.4771	1.277875	0.26779758	0.587946
216565	Ehbp1	EH domain binding protein 1, transcript variant X29	NA	0.054869	6.696873	1.038765	0.26781328	0.587946
212539	Gm266	predicted gene 266	NA	0.305204	0.493148	1.235593	0.26796833	0.587958
69035	Zdhhc3	zinc finger, DHHC domain containing 3, transcript var	NA	-0.0582	6.579774	-1.04116	0.26797512	0.587958
17067	Ly6c1	lymphocyte antigen 6 complex, locus C1, transcript va	NA	0.267256	0.905263	1.203517	0.26799304	0.587958
212898	Dse	dermatan sulfate epimerase, transcript variant X5	NA	-0.11629	3.744972	-1.08395	0.26799452	0.587958
71844	Nupl1	nucleoporin like 1, transcript variant 1	NA	0.088472	5.684492	1.063243	0.26800533	0.587958
66593	Diablo	diablo, IAP-binding mitochondrial protein	NA	0.07317	5.565977	1.052025	0.2680354	0.587958
100134861	Gm44502	predicted readthrough transcript (NMD candidate), 44	NA	-0.99411	-1.26201	-1.99186	0.26812217	0.588069
319636	Fsd1l	fibronectin type III and SPRY domain containing 1-like	NA	0.066199	6.516407	1.046955	0.26820403	0.588169
22249	Unc13b	unc-13 homolog B, transcript variant X19	NA	0.068185	6.137756	1.048397	0.26831449	0.588332
116732	Tsga13	testis specific gene A13, transcript variant X1	NA	0.514844	-0.41384	1.42884	0.26838609	0.588353
100380944	Ino80dos	INO80 complex subunit D, opposite strand, transcript	NA	0.18413	1.808946	1.136132	0.26839631	0.588353
15476	Hs3st1	heparan sulfate (glucosamine) 3-O-sulfotransferase 1	NA	0.093845	4.373892	1.06721	0.26864264	0.588814
66237	Atp6v1g2	ATPase, H+ transporting, lysosomal V1 subunit G2, t	NA	0.064672	5.87124	1.045847	0.26870474	0.588828
213326	Scyl2	SCY1-like 2 (S. cerevisiae), transcript variant 3	NA	0.064225	6.039779	1.045523	0.26872148	0.588828
100040294	Gm2694	predicted gene 2694, transcript variant 2	NA	0.173619	2.155057	1.127884	0.26887768	0.589048
67673	Elob	elongin B	NA	0.066169	6.823884	1.046933	0.26889627	0.589048
110213	Tmbim6	transmembrane BAX inhibitor motif containing 6, tran	NA	-0.05433	7.747931	-1.03837	0.26893038	0.589048
225326	Pik3c3	phosphatidylinositol 3-kinase catalytic subunit type 3,	NA	0.069039	6.730006	1.049018	0.26901091	0.589073
55987	Cpxm2	carboxypeptidase X 2 (M14 family)	NA	-0.21036	1.404825	-1.15697	0.26901429	0.589073
73942	Fam151b	family with sequence similarity 151, member B	NA	0.186556	1.837384	1.138043	0.26909632	0.589099
98415	Nucks1	nuclear casein kinase and cyclin-dependent kinase su	NA	0.058947	7.958065	1.041705	0.26912376	0.589099
118568604	LOC118568604	uncharacterized LOC118568604, transcript variant X	NA	-0.24477	1.096826	-1.1849	0.26917846	0.589099
66054	Cndp2	CNDP dipeptidase 2 (metallopeptidase M20 family), t	NA	-0.06326	5.498919	-1.04482	0.26919192	0.589099
56318	Acpp	acid phosphatase, prostate, transcript variant 1	NA	0.37975	-0.1729	1.301116	0.26920723	0.589099
12042	Bcl10	B cell leukemia/lymphoma 10	NA	0.079863	4.728897	1.056918	0.2692809	0.589181
77994	Mir99ahg	Mir99a and Mirlet7c-1 host gene (non-protein coding)	NA	0.2316	1.874562	1.174136	0.26936323	0.589282
115488284	Gm3752	predicted gene 3752, transcript variant 2	NA	0.121319	5.268587	1.087729	0.26943757	0.589365
70419	2810408A11Rik	RIKEN cDNA 2810408A11 gene, transcript variant X1	NA	0.120483	3.865785	1.087099	0.26951247	0.58945
20450	St8sia2	ST8 alpha-N-acetyl-neuraminide alpha-2,8-sialyltrans	NA	0.050199	8.257525	1.035407	0.26956234	0.58948
629378	Dact3	dishevelled-binding antagonist of beta-catenin 3	NA	0.096304	5.521185	1.069032	0.26978771	0.589893
67283	Slc25a19	solute carrier family 25 (mitochondrial thiamine pyrop	NA	-0.07858	4.834932	-1.05598	0.26996171	0.590194
16514	Kcnj11	potassium inwardly rectifying channel, subfamily J, m	NA	-0.08857	4.38987	-1.06332	0.26999997	0.590199
74570	Zkscan1	zinc finger with KRAB and SCAN domains 1, transcrip	NA	0.058577	6.72662	1.041438	0.27012375	0.590345
170756	Slc8b1	solute carrier family 8 (sodium/lithium/calcium exchar	NA	-0.21309	1.416619	-1.15917	0.27013936	0.590345
57377	Mogs	mannosyl-oligosaccharide glucosidase	NA	-0.10127	4.917496	-1.07271	0.27026969	0.590362
381522	Ccdc180	coiled-coil domain containing 180, transcript variant X	NA	-0.38158	-0.30667	-1.30277	0.27030709	0.590362
116848	Baz2a	bromodomain adjacent to zinc finger domain, 2A	NA	0.06341	6.267393	1.044932	0.27033929	0.590362
76448	Ppp1r18	protein phosphatase 1, regulatory subunit 18, transcr	NA	-0.09397	5.952462	-1.0673	0.27038268	0.590362
19309	Pygm	muscle glycogen phosphorylase	NA	-0.19892	2.870587	-1.14784	0.27038492	0.590362
16564	Kif21a	kinesin family member 21A, transcript variant X28	NA	0.073807	7.416663	1.052491	0.27038497	0.590362
20014	Rpn2	ribophorin II, transcript variant X3	NA	-0.05839	7.193224	-1.0413	0.27040138	0.590362
66191	Ier3ip1	immediate early response 3 interacting protein 1	NA	0.079744	5.963633	1.05683	0.2704762	0.590447
74778	Rrp7a	ribosomal RNA processing 7 homolog A, transcript va	NA	-0.06431	6.27501	-1.04558	0.27062975	0.590703
68121	Cep70	centrosomal protein 70	NA	0.064304	5.475337	1.04558	0.27071465	0.590772
70804	Pgrmc2	progesterone receptor membrane component 2	NA	-0.07863	6.001417	-1.05601	0.27078975	0.590772
102857	Slc6a8	solute carrier family 6 (neurotransmitter transporter, c	NA	-0.06641	5.906387	-1.04711	0.27079043	0.590772
56434	Tspan3	tetraspanin 3	NA	0.091489	7.827533	1.065469	0.27081915	0.590772
102941	B630019K06Rik	RIKEN cDNA B630019K06 gene	NA	0.0832	5.916042	1.059365	0.2708942	0.590772
29805	Znhit2	zinc finger, HIT domain containing 2	NA	0.162252	3.200914	1.119033	0.27090944	0.590772
235134	Nfrkb	nuclear factor related to kappa B binding protein, tran	NA	0.065845	5.665265	1.046698	0.27091584	0.590772
67439	Xab2	XPA binding protein 2	NA	-0.06616	5.521687	-1.04692	0.27097332	0.590799
170770	Bbc3	BCL2 binding component 3, transcript variant 6	NA	0.108054	3.962267	1.077773	0.27100063	0.590799
102633888	Gm31606	predicted gene, 31606	NA	-0.07688	6.307952	-1.05474	0.2712608	0.591287
93838	Dqx1	DEAQ RNA-dependent ATPase	NA	-0.40418	-0.22178	-1.32333	0.27136055	0.591425
75812	Tasp1	taspase, threonine aspartase 1, transcript variant 2	NA	0.101915	3.744844	1.073197	0.27142952	0.591496
68709	Cilp2	cartilage intermediate layer protein 2	NA	0.16624	2.070234	1.12213	0.27150151	0.591574
80752	Fam20c	family with sequence similarity 20, member C, transcr	NA	-0.05532	6.393476	-1.03909	0.27190515	0.592302
56550	Ube2d2a	ubiquitin-conjugating enzyme E2D 2A	NA	0.055802	8.128263	1.039437	0.27193861	0.592302
59030	Mkks	McKusick-Kaufman syndrome, transcript variant 2	NA	-0.10009	4.288515	-1.07184	0.2719544	0.592302
74013	Rftn2	raftlin family member 2, transcript variant 1	NA	0.087186	4.312426	1.062296	0.2719812	0.592302
21672	Prdx2	peroxiredoxin 2, transcript variant 1	NA	-0.05055	8.860116	-1.03566	0.27205054	0.592373
65112	Pmepa1	prostate transmembrane protein, androgen induced 1	NA	-0.06994	5.449903	-1.04967	0.27219299	0.592574
19267	Ptpre	protein tyrosine phosphatase, receptor type, E, transc	NA	0.12576	3.549453	1.091082	0.27221543	0.592574
105638	Dph3	diphthamine biosynthesis 3, transcript variant 6	NA	0.070235	5.496164	1.049888	0.27237761	0.592664
20564	Slit3	slit guidance ligand 3	NA	-0.14831	2.676465	-1.10827	0.2724046	0.592664
105246303	Gm41607	predicted gene, 41607	NA	0.13873	3.912079	1.100935	0.27243057	0.592664

11946	Atp5a1	ATP synthase, H+ transporting, mitochondrial F1 com	NA	0.052853	9.42096	1.037314	0.27244359	0.592664
50755	Fbh1	F-box DNA helicase 1, transcript variant 1	NA	-0.05858	6.394343	-1.04144	0.27249471	0.592664
17387	Mmp14	matrix metallopeptidase 14 (membrane-inserted)	NA	-0.0618	6.693807	-1.04377	0.2724969	0.592664
100034361	Mfap1b	microfibrillar-associated protein 1B	NA	-0.09468	5.249927	-1.06783	0.27252503	0.592664
56452	Orc6	origin recognition complex, subunit 6, transcript variar	NA	0.069982	5.200434	1.049703	0.27254802	0.592664
115487182	Gm51684	predicted gene, 51684	NA	0.371656	0.196202	1.293837	0.27263239	0.592768
70661	Sik3	SIK family kinase 3, transcript variant X1	NA	0.062482	5.744341	1.044261	0.27276733	0.592778
76308	Rab1b	RAB1B, member RAS oncogene family	NA	0.058248	6.719317	1.0412	0.27279545	0.592778
109624	Cald1	caldesmon 1, transcript variant 2	NA	-0.05687	6.259899	-1.04021	0.27279789	0.592778
72813	2810454H06Rik	RIKEN cDNA 2810454H06 gene	NA	0.211267	1.676238	1.157705	0.2728039	0.592778
18160	Npr1	natriuretic peptide receptor 1	NA	-0.16042	2.381549	-1.11762	0.27282594	0.592778
12349	Car2	carbonic anhydrase 2, transcript variant 2	NA	0.119797	4.059051	1.086582	0.27285545	0.592778
69955	Fars2	phenylalanine-tRNA synthetase 2 (mitochondrial), tra	NA	0.099325	3.780355	1.071272	0.2731953	0.593344
26428	Orc4	origin recognition complex, subunit 4, transcript variar	NA	0.068966	5.783234	1.048965	0.27322276	0.593344
12847	Copa	coatomer protein complex subunit alpha, transcript va	NA	-0.04928	8.324185	-1.03474	0.2732278	0.593344
228852	Ppp1r16b	protein phosphatase 1, regulatory subunit 16B, transc	NA	0.076688	5.157918	1.054594	0.27330362	0.593344
80976	Syt13	synaptotagmin XIII	NA	-0.08192	6.736216	-1.05843	0.2733041	0.593344
117600	Srgap1	SLIT-ROBO Rho GTPase activating protein 1, transc	NA	0.07912	7.406469	1.056373	0.27333476	0.593344
67933	Hcfc2	host cell factor C2, transcript variant 2	NA	0.089828	5.141689	1.064243	0.27342106	0.593397
66890	Lman2	lectin, mannose-binding 2	NA	-0.06793	6.160288	-1.04821	0.27343221	0.593397
118567505	LOC118567505	ribosome-binding protein 1-like	NA	-0.27874	0.507745	-1.21313	0.2735158	0.593433
108167760	Gm50589	predicted gene, 50589, transcript variant X1	NA	-0.40258	0.249873	-1.32187	0.27353056	0.593433
67873	Mri1	methylthioribose-1-phosphate isomerase 1	NA	0.128913	3.316881	1.09347	0.2735581	0.593433
235504	Slc17a5	solute carrier family 17 (anion/sugar transporter), mer	NA	-0.11134	3.899056	-1.08023	0.27361205	0.59347
105246702	Gm41949	predicted gene, 41949	NA	0.185126	1.891886	1.136916	0.2736552	0.593485
18984	Por	P450 (cytochrome) oxidoreductase	NA	-0.05724	6.385434	-1.04047	0.27374867	0.59353
12460	Ccs	copper chaperone for superoxide dismutase	NA	0.098241	3.777682	1.070468	0.27374885	0.59353
76763	Mospd2	motile sperm domain containing 2, transcript variant ´	NA	0.077901	4.60861	1.055482	0.27385512	0.593681
68588	Cthrc1	collagen triple helix repeat containing 1, transcript var	NA	0.130128	3.624516	1.094391	0.27394633	0.5938
68134	Upf3b	UPF3 regulator of nonsense transcripts homolog B (y	NA	0.074136	6.219665	1.05273	0.27405732	0.593961
70730	6330409D20Rik	RIKEN cDNA 6330409D20 gene	NA	-0.25732	1.261763	-1.19526	0.27414217	0.594066
21350	Tal2	T cell acute lymphocytic leukemia 2	NA	-0.20003	2.314963	-1.14872	0.27422078	0.594119
100213	Rusc2	RUN and SH3 domain containing 2, transcript variant	NA	-0.05082	7.690296	-1.03586	0.27423981	0.594119
56294	Ptpn9	protein tyrosine phosphatase, non-receptor type 9	NA	0.056643	6.451646	1.040043	0.27433191	0.59424
67603	Dusp6	dual specificity phosphatase 6	NA	0.084005	4.943095	1.059956	0.27444494	0.594405
16775	Lama4	laminin, alpha 4	NA	0.075709	5.379297	1.053879	0.27452368	0.594497
212198	Wdr25	WD repeat domain 25, transcript variant X2	NA	-0.16579	2.49846	-1.12178	0.27457091	0.59452
69792	Med6	mediator complex subunit 6, transcript variant 3	NA	0.088586	4.200533	1.063327	0.27482407	0.594754
208366	Rpp40	ribonuclease P 40 subunit, transcript variant X1	NA	-0.18962	2.028464	-1.14047	0.27486854	0.594754
170571	Cntnap4	contactin associated protein-like 4	NA	0.086787	4.761236	1.062002	0.27487905	0.594754
237082	Nxt2	nuclear transport factor 2-like export factor 2, transcri	NA	0.09197	4.018295	1.065824	0.27488399	0.594754
108168907	Gm46829	predicted gene, 46829, transcript variant X2	NA	-0.43893	0.950702	-1.3556	0.27490847	0.594754
65945	Clstn1	calsyntenin 1, transcript variant X5	NA	0.05511	8.98312	1.038939	0.27493161	0.594754
171463	Il17rd	interleukin 17 receptor D, transcript variant X2	NA	0.107985	4.416533	1.077722	0.27497569	0.594754
70727	Rasgef1a	RasGEF domain family, member 1A, transcript variar	NA	0.118995	4.029295	1.085978	0.2749839	0.594754
74246	Gale	galactose-4-epimerase, UDP	NA	-0.10715	3.841009	-1.0771	0.27500807	0.594754
109889	Mzf1	myeloid zinc finger 1, transcript variant X4	NA	0.162658	2.682886	1.119347	0.27504852	0.594763
18002	Nedd8	neural precursor cell expressed, developmentally dov	NA	0.059303	6.041732	1.041963	0.27515972	0.594859
105244799	Gm40346	predicted gene, 40346	NA	-0.26017	0.836284	-1.19762	0.27519478	0.594859
19386	Ranbp2	RAN binding protein 2	NA	0.063357	6.243645	1.044894	0.27520278	0.594859
108043	Chrnb3	cholinergic receptor, nicotinic, beta polypeptide 3, trar	NA	0.336544	0.043266	1.262728	0.27534541	0.595034
118453	Mmp28	matrix metallopeptidase 28 (epilysin), transcript variar	NA	-0.15351	2.721229	-1.11227	0.27537735	0.595034
98870	AI182371	expressed sequence AI182371, transcript variant 3	NA	0.413098	-0.35051	1.331542	0.27539323	0.595034
118567328	LOC118567328	uncharacterized LOC118567328	NA	-0.39786	2.241342	-1.31756	0.27548039	0.595136
74554	9130002K18Rik	RIKEN cDNA 9130002K18 gene	NA	-0.15326	2.639824	-1.11208	0.27551372	0.595136
100503915	Smpd5	sphingomyelin phosphodiesterase 5	NA	-0.34438	0.006345	-1.2696	0.27556499	0.595168
211401	Mtss1	MTSS I-BAR domain containing 1, transcript variant X	NA	0.049551	7.785285	1.034942	0.2756191	0.595206
226359	C1ql2	complement component 1, q subcomponent-like 2	NA	-0.17747	2.327734	-1.1309	0.27570907	0.595321
232987	B9d2	B9 protein domain 2, transcript variant 1	NA	0.125226	3.219348	1.090679	0.27581245	0.595445
12928	Crk	v-crk avian sarcoma virus CT10 oncogene homolog, '	NA	0.056722	7.26838	1.0401	0.27583986	0.595445
244891	Scaper	S phase cyclin A-associated protein in the ER, transc	NA	0.086505	6.212842	1.061795	0.27595383	0.59556
100036569	Gm16525	predicted gene, 16525, transcript variant 3	NA	0.1228	3.714916	1.088846	0.27596618	0.59556
71707	Ubiad1	UbiA prenyltransferase domain containing 1	NA	-0.10413	3.871443	-1.07485	0.27606114	0.595686
55944	Eif3d	eukaryotic translation initiation factor 3, subunit D	NA	-0.05865	7.122698	-1.04149	0.27613941	0.595776
22704	Zfp46	zinc finger protein 46, transcript variant 2	NA	-0.06119	6.517429	-1.04333	0.27634757	0.596071
19142	Prss12	protease, serine 12 neurotrypsin (motopsin)	NA	0.125321	3.456301	1.090751	0.27634969	0.596071
102631930	A030010E16Rik	RIKEN cDNA A030010E16 gene, transcript variant X	NA	-0.28097	0.683838	-1.21501	0.27649397	0.596214
76788	Klhdc10	kelch domain containing 10, transcript variant 2	NA	0.05297	7.806455	1.037398	0.27653777	0.596214

67311	Nanp	N-acetylneuraminic acid phosphatase	NA	0.105471	3.947256	1.075846	0.27658767	0.596214
67337	Cstf1	cleavage stimulation factor, 3' pre-RNA, subunit 1, tra	NA	-0.06951	5.169054	-1.04936	0.2765877	0.596214
69962	Mettl18	methyltransferase like 18, transcript variant 2	NA	0.163636	2.183446	1.120107	0.27659893	0.596214
100043403	Gm14410	predicted gene 14410	NA	-0.10696	3.610919	-1.07696	0.27678091	0.596527
216001	Micu1	mitochondrial calcium uptake 1, transcript variant 3	NA	-0.09305	5.664774	-1.06662	0.27685179	0.59653
74168	Zdhhc16	zinc finger, DHHC domain containing 16, transcript va	NA	0.067693	5.352253	1.04804	0.27688837	0.59653
50799	Slc25a13	solute carrier family 25 (mitochondrial carrier, adenine	NA	-0.14013	2.791701	-1.102	0.27689207	0.59653
12075	Bfsp1	beaded filament structural protein 1, in lens-CP94, tra	NA	0.222401	1.240525	1.166674	0.27715346	0.597012
81840	Sorcs2	sortilin-related VPS10 domain containing receptor 2	NA	-0.07797	5.468125	-1.05553	0.2771894	0.597012
269019	Stk32a	serine/threonine kinase 32A	NA	0.194034	1.542678	1.143958	0.27735427	0.597237
99929	Tiparp	TCDD-inducible poly(ADP-ribose) polymerase	NA	0.077627	4.769064	1.055281	0.27736735	0.597237
72754	Arhgef10l	Rho guanine nucleotide exchange factor (GEF) 10-lik	NA	-0.07139	5.612503	-1.05073	0.27741733	0.597266
72900	Ndufv2	NADH:ubiquinone oxidoreductase core subunit V2, tr	NA	0.054987	6.717677	1.03885	0.27756342	0.597501
13496	Arid3a	AT rich interactive domain 3A (BRIGHT-like), transcri	NA	-0.13279	3.742636	-1.09641	0.27773629	0.597749
503610	Zdhhc18	zinc finger, DHHC domain containing 18	NA	0.068554	5.400581	1.048665	0.27778425	0.597749
12577	Cdkn1c	cyclin-dependent kinase inhibitor 1C (P57), transcript	NA	-0.06012	7.312226	-1.04255	0.27781928	0.597749
50721	Sirt6	sirtuin 6, transcript variant 1	NA	-0.08714	5.116711	-1.06226	0.27787797	0.597749
12759	Clu	clusterin	NA	0.069625	5.758896	1.049444	0.27791923	0.597749
73389	Hbp1	high mobility group box transcription factor 1, transcri	NA	-0.06181	5.64978	-1.04377	0.2779288	0.597749
102640300	Gm16083	predicted gene 16083, transcript variant X1	NA	0.298693	0.79658	1.230029	0.27799148	0.597749
71968	Wdr73	WD repeat domain 73, transcript variant 2	NA	0.071205	4.906578	1.050593	0.27803278	0.597749
320127	Dgki	diacylglycerol kinase, iota, transcript variant 2	NA	0.06799	5.752773	1.048255	0.27803529	0.597749
213236	Dnd1	DND microRNA-mediated repression inhibitor 1	NA	-0.29531	0.667701	-1.22714	0.27810677	0.597749
15958	Ifit2	interferon-induced protein with tetratricopeptide repea	NA	0.107166	3.605627	1.07711	0.27810751	0.597749
102638099	Gm34744	predicted gene, 34744, transcript variant X4	NA	-0.32628	0.083931	-1.25378	0.27812667	0.597749
260298	Fev	FEV transcription factor, ETS family member	NA	-0.4138	0.124446	-1.33219	0.27815594	0.597749
654359	Gm12338	predicted gene 12338	NA	-0.10073	5.729072	-1.07232	0.27822475	0.597818
70435	Inf2	inverted formin, FH2 and WH2 domain containing, tra	NA	-0.11776	4.151698	-1.08505	0.27830524	0.597845
100689	Spon2	spondin 2, extracellular matrix protein, transcript varia	NA	-0.25441	1.074727	-1.19285	0.27831082	0.597845
434204	Whamm	WAS protein homolog associated with actin, golgi me	NA	0.112149	3.949342	1.080837	0.27837394	0.597887
24061	Smc1a	structural maintenance of chromosomes 1A, transcrip	NA	-0.06469	7.069501	-1.04586	0.27843211	0.597887
210510	Tdrd6	tudor domain containing 6, transcript variant 3	NA	0.284774	0.501172	1.218219	0.27845952	0.597887
26572	Cops3	COP9 signalosome subunit 3	NA	0.052218	6.586405	1.036858	0.27847764	0.597887
14852	Gspt1	G1 to S phase transition 1, transcript variant 1	NA	0.05941	7.477601	1.042039	0.27853068	0.597922
70779	Prdm5	PR domain containing 5, transcript variant 1	NA	-0.16522	2.427904	-1.12134	0.27868394	0.598091
70144	Lrch3	leucine-rich repeats and calponin homology (CH) dor	NA	-0.05458	6.354363	-1.03856	0.27868902	0.598091
68797	Pdgfrl	platelet-derived growth factor receptor-like	NA	0.182212	1.768091	1.134622	0.27884431	0.598091
72931	Swi5	SWI5 recombination repair homolog (yeast), transcrip	NA	-0.05976	6.867986	-1.04229	0.27884543	0.598091
56743	Lat2	linker for activation of T cells family, member 2, trans	NA	0.280982	1.807436	1.215021	0.27884917	0.598091
19300	Abcd4	ATP-binding cassette, sub-family D (ALD), member 4	NA	-0.12121	3.518152	-1.08765	0.27885445	0.598091
230824	Grhl3	grainyhead like transcription factor 3	NA	0.322428	0.430379	1.250433	0.27886657	0.598091
19155	Npepps	aminopeptidase puromycin sensitive, transcript varian	NA	-0.05575	7.923652	-1.0394	0.27912489	0.598543
69847	Wnk4	WNK lysine deficient protein kinase 4, transcript varia	NA	-0.15007	2.631921	-1.10962	0.27925374	0.598543
74264	Rnf138rt1	ring finger protein 138, retrogene 1	NA	0.247972	0.77841	1.187537	0.27927076	0.598543
216766	Gemin5	gem nuclear organelle associated protein 5, transcrip	NA	0.078693	4.993108	1.056061	0.2793053	0.598543
12747	Clk1	CDC-like kinase 1, transcript variant 3	NA	0.070347	7.166414	1.049969	0.27933096	0.598543
228777	Nrsn2	neurensin 2, transcript variant X2	NA	0.096473	4.447822	1.069156	0.27933165	0.598543
229615	Pias3	protein inhibitor of activated STAT 3, transcript varian	NA	-0.07138	5.092218	-1.05072	0.27933466	0.598543
192198	Lrrc4	leucine rich repeat containing 4	NA	-0.07221	5.528223	-1.05133	0.27965516	0.598879
338370	Nalcn	sodium leak channel, non-selective	NA	0.070855	5.259494	1.050339	0.27966923	0.598879
21814	Tgfbr3	transforming growth factor, beta receptor III	NA	0.126919	3.461791	1.091959	0.2796794	0.598879
67078	Pgp	phosphoglycolate phosphatase	NA	0.088508	5.047815	1.06327	0.27969677	0.598879
104245	Slc6a5	solute carrier family 6 (neurotransmitter transporter, g	NA	0.21428	3.24622	1.160125	0.27971636	0.598879
14178	Fgf7	fibroblast growth factor 7	NA	-0.26883	0.785715	-1.20483	0.27973006	0.598879
69798	1810044D09Rik	RIKEN cDNA 1810044D09 gene	NA	0.488579	-0.29671	1.403062	0.27974934	0.598879
19383	Raly	hnRNP-associated with lethal yellow, transcript varian	NA	-0.05174	7.251564	-1.03652	0.27980907	0.598928
74140	Tm9sf1	transmembrane 9 superfamily member 1, transcript v	NA	-0.06765	5.226016	-1.048	0.27993418	0.599094
100503869	Mkln1os	muskelin 1, intracellular mediator containing kelch mo	NA	0.257208	0.870073	1.195163	0.27996911	0.599094
320951	Pisd	phosphatidylserine decarboxylase, transcript variant	NA	-0.06269	5.665054	-1.04441	0.28004534	0.599094
432999	A930007A09Rik	RIKEN cDNA A930007A09 gene	NA	0.156638	2.225187	1.114687	0.28006423	0.599094
18741	Pitx2	paired-like homeodomain transcription factor 2, trans	NA	-0.15561	3.445974	-1.1139	0.28007052	0.599094
380924	Olfm4	olfactomedin 4	NA	0.22331	1.494173	1.167409	0.28020042	0.599293
13642	Efnb2	ephrin B2, transcript variant 1	NA	-0.07788	7.416844	-1.05546	0.28026064	0.599343
110920	Hspa13	heat shock protein 70 family, member 13, transcript v	NA	0.073891	6.007486	1.052551	0.28031469	0.599352
72050	Poglut2	protein O-glucosyltransferase 2	NA	-0.0798	4.782129	-1.05687	0.2803835	0.599352
102640562	BB218582	expressed sequence BB218582	NA	0.276189	0.749684	1.210992	0.28042898	0.599352
67684	Luc7l3	LUC7-like 3 (S. cerevisiae), transcript variant 3	NA	0.052882	8.150006	1.037335	0.28046013	0.599352
74048	Vsir	V-set immunoregulatory receptor, transcript variant 1	NA	0.174978	2.464384	1.128947	0.2804776	0.599352

14792	Lpcat3	lysophosphatidylcholine acyltransferase 3, transcript	NA	0.091115	4.297234	1.065193	0.28048596	0.599352
226162	Dpcd	deleted in primary ciliary dyskinesia, transcript varian	NA	-0.09695	4.291434	-1.06951	0.28061718	0.599552
12943	Pcdha10	protocadherin alpha 10	NA	-0.14798	3.150254	-1.10802	0.28067005	0.599552
16656	Hivep3	human immunodeficiency virus type I enhancer bindi	NA	0.093635	4.92518	1.067055	0.28068984	0.599552
320782	Tmem154	transmembrane protein 154	NA	-0.33882	0.663079	-1.26472	0.28076178	0.599627
68219	Nudt21	nudix (nucleoside diphosphate linked moiety X)-type	NA	0.103898	5.850591	1.074673	0.2808355	0.599705
170472	Recql5	RecQ protein-like 5	NA	-0.09594	4.386828	-1.06876	0.28088868	0.59974
22217	Usp12	ubiquitin specific peptidase 12	NA	0.073786	6.089073	1.052475	0.28092998	0.59975
79566	Sh3bp5l	SH3 binding domain protein 5 like, transcript variant	NA	-0.0773	5.321702	-1.05504	0.28111121	0.599797
11690	Alox5ap	arachidonate 5-lipoxygenase activating protein, trans	NA	0.276363	0.95367	1.211138	0.28111216	0.599797
73341	Arhgef6	Rac/Cdc42 guanine nucleotide exchange factor (GEF	NA	0.125517	3.054889	1.090899	0.28113543	0.599797
19079	Prkab1	protein kinase, AMP-activated, beta 1 non-catalytic su	NA	0.073972	5.127445	1.052611	0.28114902	0.599797
54201	Zfp316	zinc finger protein 316	NA	-0.06445	5.924119	-1.04569	0.28116294	0.599797
67914	Coq9	coenzyme Q9	NA	0.08824	4.462383	1.063072	0.28117343	0.599797
56322	Timm22	translocase of inner mitochondrial membrane 22, trar	NA	-0.07443	4.824403	-1.05294	0.28125283	0.599888
330817	Dhps	deoxyhypusine synthase	NA	0.071292	4.818952	1.050657	0.28149419	0.600286
230904	Fbxo2	F-box protein 2	NA	0.196317	1.882586	1.145769	0.28151338	0.600286
70686	Dusp16	dual specificity phosphatase 16, transcript variant B1	NA	-0.0967	4.221406	-1.06932	0.28163427	0.600441
15368	Hmox1	heme oxygenase 1	NA	-0.14039	2.911942	-1.1022	0.28170459	0.600441
170750	Xpnpep1	X-prolyl aminopeptidase (aminopeptidase P) 1, solub	NA	0.0571	6.031529	1.040372	0.28173154	0.600441
56745	C1qtnf1	C1q and tumor necrosis factor related protein 1, trans	NA	-0.11503	3.57491	-1.083	0.28175857	0.600441
243369	Sspo	SCO-spondin, transcript variant X6	NA	0.403431	0.15423	1.32265	0.28177049	0.600441
16011	Igfbp5	insulin-like growth factor binding protein 5	NA	0.066537	8.140296	1.0472	0.28184625	0.60045
19352	Rabggtb	Rab geranylgeranyl transferase, b subunit, transcript	NA	-0.06618	5.457502	-1.04694	0.28184853	0.60045
75906	Fam184a	family with sequence similarity 184, member A, trans	NA	0.089414	4.331745	1.063938	0.28190621	0.60046
18120	Mrpl49	mitochondrial ribosomal protein L49	NA	-0.07373	5.227345	-1.05243	0.28192702	0.60046
17355	Aff1	AF4/FMR2 family, member 1, transcript variant X2	NA	-0.12234	3.361755	-1.0885	0.28201626	0.600572
328381	Sh2d4b	SH2 domain containing 4B, transcript variant X4	NA	-0.1341	3.474419	-1.09741	0.28213017	0.600686
207728	Pde2a	phosphodiesterase 2A, cGMP-stimulated, transcript v	NA	-0.06666	6.051515	-1.04729	0.2821638	0.600686
16205	Gimap1	GTPase, IMAP family member 1, transcript variant 1	NA	0.276888	0.467987	1.211578	0.28218072	0.600686
67980	Gnpda2	glucosamine-6-phosphate deaminase 2, transcript va	NA	0.09205	4.083435	1.065884	0.28226887	0.600795
666704	Samd1	sterile alpha motif domain containing 1	NA	-0.06683	5.9105	-1.04741	0.28245146	0.601027
66676	Tmed7	transmembrane p24 trafficking protein 7	NA	0.068601	6.898244	1.048699	0.28245178	0.601027
64213	St7	suppression of tumorigenicity 7, transcript variant 6	NA	-0.06821	5.395714	-1.04842	0.28256017	0.601105
71769	Bbs10	Bardet-Biedl syndrome 10 (human)	NA	-0.17542	2.440055	-1.12929	0.28256197	0.601105
78521	B230219D22Rik	RIKEN cDNA B230219D22 gene	NA	0.066428	7.35864	1.047121	0.28269236	0.601303
171429	Slc26a6	solute carrier family 26, member 6, transcript variant	NA	0.119396	3.245146	1.08628	0.28274428	0.601335
114606	Tle6	transducin-like enhancer of split 6, transcript variant	NA	-0.26896	0.769211	-1.20494	0.28278423	0.601341
16890	Lipe	lipase, hormone sensitive, transcript variant 1	NA	-0.09714	3.773465	-1.06965	0.28285777	0.601419
20605	Sstr1	somatostatin receptor 1	NA	0.104464	4.114994	1.075095	0.28298424	0.601597
118567450	LOC118567450	uncharacterized LOC118567450	NA	-0.39478	-0.15518	-1.31474	0.28301532	0.601597
23983	Pcbp1	poly(rC) binding protein 1	NA	-0.08561	8.116004	-1.06113	0.28316333	0.601833
76563	Qrsl1	glutaminyl-tRNA synthase (glutamine-hydrolyzing)-lik	NA	0.108801	3.61069	1.078331	0.28325748	0.601909
55989	Nop58	NOP58 ribonucleoprotein	NA	0.062596	6.00075	1.044343	0.28327303	0.601909
21803	Tgfb1	transforming growth factor, beta 1	NA	-0.13375	3.005662	-1.09714	0.28349288	0.602245
11418	Asic2	acid-sensing (proton-gated) ion channel 2, transcript	NA	0.082802	5.466315	1.059073	0.28354426	0.602245
72831	Dhx30	DEAH (Asp-Glu-Ala-His) box polypeptide 30, transcri	NA	-0.0509	7.04415	-1.03591	0.28357865	0.602245
18998	Pou4f3	POU domain, class 4, transcription factor 3	NA	-0.3082	-0.00027	-1.23816	0.28357903	0.602245
18159	Nppc	natriuretic peptide type C	NA	-0.29255	0.317503	-1.2248	0.28363706	0.602289
68114	Pwwp3a	PWWP domain containing 3A, DNA repair factor, trar	NA	-0.06363	5.594912	-1.04509	0.28381758	0.602504
214987	Chtf8	CTF8, chromosome transmission fidelity factor 8, trar	NA	0.721051	-0.275	1.648382	0.28382717	0.602504
110651	Rps6ka3	ribosomal protein S6 kinase polypeptide 3, transcript	NA	0.076138	6.057198	1.054192	0.28384906	0.602504
118568505	LOC118568505	uncharacterized LOC118568505	NA	-0.32004	0.001422	-1.24836	0.28403008	0.60278
69257	Elf2	E74-like factor 2, transcript variant X1	NA	-0.06608	6.387742	-1.04686	0.28405328	0.60278
14791	Emg1	EMG1 N1-specific pseudouridine methyltransferase	NA	-0.07574	5.510171	-1.0539	0.2843617	0.603317
19092	Prkg2	protein kinase, cGMP-dependent, type II	NA	0.189224	1.877102	1.140151	0.28442245	0.603317
269356	Slc4a11	solute carrier family 4, sodium bicarbonate transporte	NA	-0.32806	0.337786	-1.25533	0.28443054	0.603317
19294	Nectin2	nectin cell adhesion molecule 2, transcript variant 2	NA	0.08184	5.17206	1.058367	0.28445468	0.603317
228061	Agps	alkylglycerone phosphate synthase	NA	0.080881	6.005993	1.057664	0.2844939	0.603321
140499	Ube2j2	ubiquitin-conjugating enzyme E2J 2, transcript varian	NA	0.059193	6.06946	1.041883	0.28460879	0.60338
432939	Gm48957	predicted gene, 48957	NA	0.091215	4.332067	1.065267	0.28465045	0.60338
20973	Syngr2	synaptogyrin 2	NA	-0.16681	2.949791	-1.12257	0.28466341	0.60338
26886	Cenph	centromere protein H	NA	-0.13239	2.770424	-1.0961	0.28470852	0.60338
613262	BC029722	cDNA sequence BC029722	NA	0.097349	4.161667	1.069806	0.28473244	0.60338
16578	Kif9	kinesin family member 9, transcript variant X4	NA	-0.16928	2.325247	-1.1245	0.28474403	0.60338
245902	Ccdc15	coiled-coil domain containing 15	NA	0.091468	4.181661	1.065454	0.28493731	0.603621
22414	Wnt2b	wingless-type MMTV integration site family, member	NA	-0.23682	1.241466	-1.17839	0.28494198	0.603621
208104	Mlxip	MLX interacting protein, transcript variant 2	NA	-0.08389	6.052958	-1.05987	0.28496908	0.603621

53419	Corin	corin, serine peptidase, transcript variant X2	NA		0.320372	0.683946	1.248652	0.28516815	0.603964
100503842	2410022M11Rik	RIKEN cDNA 2410022M11 gene	NA		0.237868	0.962733	1.179249	0.28524068	0.604028
67136	Kbtbd4	kelch repeat and BTB (POZ) domain containing 4, tra	NA		0.062502	5.434242	1.044275	0.28527244	0.604028
19298	Pex19	peroxisomal biogenesis factor 19, transcript variant 1	NA		-0.05759	6.789465	-1.04072	0.28531006	0.604029
58223	Mmp19	matrix metallopeptidase 19, transcript variant 1	NA		0.345884	0.236109	1.27093	0.28555931	0.604407
171531	Mlph	melanophilin	NA		-0.3932	0.123108	-1.3133	0.28556755	0.604407
14630	Gclm	glutamate-cysteine ligase, modifier subunit	NA		0.114704	4.150733	1.082753	0.28560059	0.604407
20349	Sema3e	sema domain, immunoglobulin domain (Ig), short bas	NA		0.122069	3.058784	1.088294	0.28565514	0.604407
56282	Mrpl12	mitochondrial ribosomal protein L12	NA		0.077817	5.429989	1.05542	0.28569223	0.604407
67667	Alkbh8	alkB homolog 8, tRNA methyltransferase, transcript v	NA		0.076523	6.334531	1.054474	0.28571158	0.604407
71733	Susd2	sushi domain containing 2, transcript variant 2	NA		-0.1902	2.183389	-1.14092	0.28579382	0.604502
101187	Parp11	poly (ADP-ribose) polymerase family, member 11, tra	NA		-0.05283	6.322861	-1.0373	0.28590498	0.60462
232174	Cyp26b1	cytochrome P450, family 26, subfamily b, polypeptide	NA		-0.05712	5.907845	-1.04039	0.28593463	0.60462
66707	Nkapl	NFKB activating protein-like	NA		0.301359	0.413145	1.232305	0.28596074	0.60462
72113	Adck1	aarF domain containing kinase 1, transcript variant X	NA		0.074922	4.69565	1.053304	0.28622482	0.605056
102595	Plekho2	pleckstrin homology domain containing, family O mer	NA		-0.12946	3.333953	-1.09389	0.28624128	0.605056
57784	Bin3	bridging integrator 3, transcript variant 1	NA		0.115858	4.136284	1.08362	0.28637201	0.605253
105244212	Gm39860	predicted gene, 39860	NA		-0.52508	-0.2881	-1.43901	0.28644693	0.605265
20641	Snrpd1	small nuclear ribonucleoprotein D1	NA		0.066012	6.48302	1.046819	0.28653931	0.605265
11977	Atp7a	ATPase, Cu++ transporting, alpha polypeptide, trans	NA		-0.09572	4.858904	-1.0686	0.28654388	0.605265
19823	Rnf7	ring finger protein 7, transcript variant 1	NA		0.059359	6.25848	1.042003	0.28655259	0.605265
80886	Senp3	SUMO/sentrin specific peptidase 3, transcript variant	NA		-0.05448	6.589098	-1.03848	0.2865634	0.605265
66258	Mrps17	mitochondrial ribosomal protein S17, transcript variar	NA		0.068162	5.549619	1.04838	0.28660917	0.605283
50798	Gne	glucosamine (UDP-N-acetyl)-2-epimerase/N-acetylm	NA		-0.06691	5.116313	-1.04747	0.28668026	0.605336
68137	Kdelr1	KDEL (Lys-Asp-Glu-Leu) endoplasmic reticulum prot	NA		0.06628	6.55093	1.047013	0.28670873	0.605336
66401	Nudt2	nudix (nucleoside diphosphate linked moiety X)-type	NA		0.084282	4.334017	1.06016	0.28679755	0.605445
56095	Ftsj3	FtsJ RNA methyltransferase homolog 3 (E. coli)	NA		-0.0684	5.466333	-1.04855	0.286917	0.605619
106572	Rab31	RAB31, member RAS oncogene family, transcript var	NA		0.058835	6.374212	1.041624	0.28697768	0.605668
60441	Mrpl38	mitochondrial ribosomal protein L38	NA		0.081022	4.763349	1.057767	0.28725162	0.606168
270190	Ephb1	Eph receptor B1, transcript variant 1	NA		0.048702	7.064448	1.034334	0.28734962	0.606266
81909	Zfpl1	zinc finger like protein 1, transcript variant 2	NA		-0.09056	4.595856	-1.06479	0.28737274	0.606266
66234	Msmo1	methylsterol monoxygenase 1	NA		0.067878	6.719899	1.048174	0.28748486	0.606424
433938	Mn1	meningioma 1	NA		-0.06211	7.360395	-1.04399	0.28753235	0.606445
385674	Zfp174	zinc finger protein 174	NA		0.129293	3.195061	1.093758	0.28800329	0.60736
212073	Syne3	spectrin repeat containing, nuclear envelope family m	NA		0.382945	0.045597	1.304001	0.28817678	0.607635
69226	Snx24	sorting nexing 24, transcript variant 1	NA		0.116491	4.172631	1.084095	0.28820848	0.607635
226025	Trpm3	transient receptor potential cation channel, subfamily	NA		0.074601	6.226187	1.053069	0.28832005	0.607792
20481	Ski	ski sarcoma viral oncogene homolog (avian), transcri	NA		0.050343	7.187941	1.035511	0.28836647	0.607811
57738	Slc15a2	solute carrier family 15 (H+/peptide transporter), mem	NA		-0.12743	4.251477	-1.09235	0.28840984	0.607823
94253	Hecw1	HECT, C2 and WW domain containing E3 ubiquitin p	NA		0.060938	7.117971	1.043144	0.28850624	0.607858
71699	Slc41a3	solute carrier family 41, member 3, transcript variant	NA		0.17249	2.166161	1.127002	0.28851995	0.607858
68938	Aspscr1	alveolar soft part sarcoma chromosome region, candi	NA		0.071878	5.705712	1.051084	0.2885382	0.607858
71254	Naif1	nuclear apoptosis inducing factor 1	NA		-0.20658	1.381245	-1.15395	0.2887799	0.608288
100126034	Gm10644	predicted gene 10644	NA		-0.46195	-0.22864	-1.3774	0.2888242	0.608303
19826	Rnps1	RNA binding protein with serine rich domain 1, transc	NA		0.060824	7.953094	1.043061	0.2888865	0.608355
13143	Dapk2	death-associated protein kinase 2	NA		-0.3273	-0.05477	-1.25466	0.28899603	0.608384
282619	Sbsn	suprabasin, transcript variant X4	NA		0.241106	5.06976	1.181898	0.28900058	0.608384
76757	Trdn	triadin, transcript variant 1	NA		0.433384	-0.83122	1.350398	0.28901933	0.608384
20616	Snap91	synaptosomal-associated protein 91, transcript variar	NA		0.053173	7.727466	1.037544	0.28904985	0.608384
20947	Swap70	SWA-70 protein	NA		0.098975	3.797818	1.071012	0.28922675	0.608678
69917	Nabp2	nucleic acid binding protein 2, transcript variant 1	NA		0.057618	6.070492	1.040746	0.28927845	0.608692
18542	Pcolce	procollagen C-endopeptidase enhancer protein	NA		0.109992	3.454854	1.079222	0.28932728	0.608692
22232	Slc35a2	solute carrier family 35 (UDP-galactose transporter),	NA		-0.09645	4.473746	-1.06914	0.28934555	0.608692
192157	Socs7	suppressor of cytokine signaling 7	NA		-0.06291	6.917107	-1.04457	0.28965433	0.609143
381633	Gm1673	predicted gene 1673, transcript variant 2	NA		0.109197	4.074206	1.078628	0.28965563	0.609143
70839	P2ry12	purinergic receptor P2Y, G-protein coupled 12, trans	NA		0.164964	2.413413	1.121138	0.28967252	0.609143
118568198	LOC118568198	uncharacterized LOC118568198	NA		0.359346	-0.22301	1.282844	0.28979005	0.609312
109075	Exosc4	exosome component 4	NA		0.101409	4.254508	1.072821	0.28988564	0.609434
244713	Zfp317	zinc finger protein 317, transcript variant 1	NA		0.064545	5.723701	1.045755	0.29000768	0.609612
381979	Brsk1	BR serine/threonine kinase 1, transcript variant 1	NA		-0.04961	8.022371	-1.03498	0.29007309	0.609671
118568712	LOC118568712	uncharacterized LOC118568712, transcript variant X	NA		-0.44101	-0.50114	-1.35756	0.29013188	0.609673
328162	Trmt61a	tRNA methyltransferase 61A, transcript variant 1	NA		-0.15058	3.452829	-1.11001	0.29015945	0.609673
14256	Flt3l	FMS-like tyrosine kinase 3 ligand, transcript variant X	NA		-0.30284	0.671334	-1.23357	0.29022556	0.609673
16814	Lbx1	ladybird homeobox 1	NA		0.244414	1.964442	1.184612	0.29028172	0.609673
69248	2610035F20Rik	RIKEN cDNA 2610035F20 gene	NA		-0.33413	0.531363	-1.26061	0.29034466	0.609673
435376	Atp6ap1l	ATPase, H+ transporting, lysosomal accessory protei	NA		0.261274	0.650696	1.198537	0.29036707	0.609673
69885	Aunip	aurora kinase A and ninein interacting protein	NA		-0.2486	1.178782	-1.18805	0.2904317	0.609673
66411	Tbcb	tubulin folding cofactor B, transcript variant 2	NA		0.052389	6.520063	1.036981	0.29046019	0.609673

22278	Usf1	upstream transcription factor 1, transcript variant 2	NA	-0.05045	6.769281	-1.03558	0.29048442	0.609673
241915	Phc3	polyhomeotic 3, transcript variant 3	NA	0.068817	5.741611	1.048856	0.29048548	0.609673
107045	Lars	leucyl-tRNA synthetase	NA	0.060545	6.047833	1.04286	0.29048623	0.609673
18049	Ngf	nerve growth factor, transcript variant 2	NA	-0.35996	-0.10504	-1.28339	0.29056784	0.60973
66653	Brf2	BRF2, RNA polymerase III transcription initiation fact	NA	0.109162	3.902308	1.078601	0.29058842	0.60973
218820	Zfp503	zinc finger protein 503	NA	-0.0814	4.883509	-1.05804	0.29080398	0.609974
215890	Clvs2	clavesin 2, transcript variant 1	NA	0.118249	4.046664	1.085417	0.29080866	0.609974
52858	Cdipt	CDP-diacylglycerol--inositol 3-phosphatidyltransferas	NA	-0.05719	5.884245	-1.04044	0.29081713	0.609974
67902	Sumf2	sulfatase modifying factor 2, transcript variant X3	NA	-0.1093	3.963643	-1.0787	0.29097495	0.610193
67455	Klhl13	kelch-like 13, transcript variant 3	NA	0.049959	7.041049	1.035236	0.29099668	0.610193
67760	Slc38a2	solute carrier family 38, member 2, transcript variant	NA	0.07862	7.966879	1.056008	0.29109012	0.61031
228880	Zmynd8	zinc finger, MYND-type containing 8, transcript variar	NA	-0.0492	7.10905	-1.03469	0.29115605	0.610349
52686	Mettl2	methyltransferase like 2	NA	-0.08639	4.560242	-1.06171	0.29118337	0.610349
57915	Tbc1d1	TBC1 domain family, member 1, transcript variant X8	NA	-0.09771	4.871473	-1.07008	0.29123959	0.610388
231290	Slc10a4	solute carrier family 10 (sodium/bile acid cotransporte	NA	0.106536	3.784857	1.07664	0.29134173	0.61045
13803	Enc1	ectodermal-neural cortex 1, transcript variant X2	NA	-0.04803	9.595545	-1.03385	0.29139644	0.61045
18507	Pax5	paired box 5	NA	0.120229	3.941344	1.086908	0.29143054	0.61045
22334	Vdac2	voltage-dependent anion channel 2	NA	0.04917	7.233856	1.03467	0.29145344	0.61045
22031	Traf3	TNF receptor-associated factor 3, transcript variant X	NA	0.056723	6.114828	1.040101	0.2914571	0.61045
26568	Slc27a3	solute carrier family 27 (fatty acid transporter), memb	NA	-0.08605	4.755765	-1.06146	0.29150076	0.610463
21745	Tep1	telomerase associated protein 1	NA	-0.14301	2.401015	-1.1042	0.29171469	0.610833
67367	Paxbp1	PAX3 and PAX7 binding protein 1, transcript variant 2	NA	0.058077	6.818621	1.041077	0.29181	0.610954
620246	Gpr52	G protein-coupled receptor 52, transcript variant 1	NA	-0.19297	1.793261	-1.14311	0.29197955	0.611018
381678	Zcwpw1	zinc finger, CW type with PWWP domain 1, transcript	NA	0.135918	3.097136	1.098792	0.29202683	0.611018
63873	Trpv4	transient receptor potential cation channel, subfamily	NA	0.222989	1.068369	1.167149	0.29205459	0.611018
54141	Spag5	sperm associated antigen 5, transcript variant X6	NA	-0.11301	4.399536	-1.08148	0.29205824	0.611018
229320	Clrn1	clarin 1, transcript variant 2	NA	0.324496	0.055846	1.252227	0.29216651	0.611018
13070	Cyp11a1	cytochrome P450, family 11, subfamily a, polypeptide	NA	-0.25341	0.878635	-1.19202	0.29219032	0.611018
170789	Acot8	acyl-CoA thioesterase 8, transcript variant X5	NA	0.156461	2.562337	1.11455	0.29220174	0.611018
243510	Ccdc142	coiled-coil domain containing 142	NA	-0.12171	3.118791	-1.08802	0.29220308	0.611018
216363	Rab3ip	RAB3A interacting protein, transcript variant 1	NA	0.073619	5.248173	1.052353	0.29221457	0.611018
16909	Lmo2	LIM domain only 2, transcript variant 2	NA	0.075712	4.634607	1.053881	0.29221618	0.611018
20674	Sox2	SRY (sex determining region Y)-box 2	NA	-0.07174	7.00357	-1.05099	0.2922562	0.611023
16678	Krt1	keratin 1	NA	-0.60193	4.057318	-1.51774	0.29241882	0.611285
224092	Lsg1	large 60S subunit nuclear export GTPase 1	NA	0.078045	5.028773	1.055587	0.29259499	0.611499
224805	Aars2	alanyl-tRNA synthetase 2, mitochondrial, transcript va	NA	-0.09964	4.212242	-1.07151	0.29259792	0.611499
18019	Nfatc2	nuclear factor of activated T cells, cytoplasmic, calcin	NA	0.124055	3.056698	1.089794	0.29263432	0.611499
56297	Arl6	ADP-ribosylation factor-like 6, transcript variant 1	NA	0.080752	4.798411	1.057569	0.29270214	0.611547
18458	Pabpc1	poly(A) binding protein, cytoplasmic 1, transcript varia	NA	-0.05921	8.993831	-1.0419	0.29280064	0.611547
18538	Pcna	proliferating cell nuclear antigen	NA	-0.07087	6.807464	-1.05035	0.29280642	0.611547
231889	Bud31	BUD31 homolog, transcript variant 1	NA	0.06169	5.819025	1.043688	0.29280755	0.611547
76044	Ncapg2	non-SMC condensin II complex, subunit G2, transcrip	NA	-0.08349	4.362955	-1.05958	0.29288867	0.611563
218343	Ttc37	tetratricopeptide repeat domain 37, transcript variant	NA	0.083718	4.785762	1.059746	0.29292075	0.611563
74467	Pus10	pseudouridylate synthase 10, transcript variant X16	NA	0.089736	4.485753	1.064175	0.29303825	0.611563
11687	Alox15	arachidonate 15-lipoxygenase, transcript variant X1	NA	-0.10662	3.55059	-1.0767	0.29304802	0.611563
14049	Eya2	EYA transcriptional coactivator and phosphatase 2, tr	NA	-0.12608	3.02629	-1.09132	0.29310031	0.611563
70349	Copb1	coatomer protein complex, subunit beta 1	NA	0.059482	6.698542	1.042091	0.29312874	0.611563
210044	Adcy2	adenylate cyclase 2	NA	0.076667	4.746666	1.054579	0.29312894	0.611563
170771	Khdrbs2	KH domain containing, RNA binding, signal transduct	NA	0.059488	5.736553	1.042096	0.29313725	0.611563
226849	Ppp2r5a	protein phosphatase 2, regulatory subunit B', alpha	NA	0.080894	4.709421	1.057674	0.29315321	0.611563
70945	Mmrn1	multimerin 1, transcript variant 2	NA	-0.15657	2.183581	-1.11463	0.29321301	0.611609
70186	Fam162a	family with sequence similarity 162, member A	NA	-0.0866	4.564689	-1.06187	0.29328607	0.611669
50500	Ttpa	tocopherol (alpha) transfer protein, transcript variant	NA	0.225119	1.767727	1.168874	0.29331707	0.611669
212632	Iffo2	intermediate filament family orphan 2, transcript varia	NA	0.074239	4.897807	1.052806	0.29337715	0.611716
242705	E2f2	E2F transcription factor 2, transcript variant 2	NA	-0.11088	3.236484	-1.07989	0.29349103	0.611751
118568032	LOC118568032	uncharacterized LOC118568032, transcript variant X	NA	-0.11225	5.530915	-1.08091	0.29353253	0.611751
226418	Yod1	YOD1 deubiquitinase	NA	0.253293	2.027133	1.191925	0.2935422	0.611751
17703	Msx3	msh homeobox 3, transcript variant 1	NA	0.263801	0.527739	1.200638	0.29354412	0.611751
69291	1700001L05Rik	RIKEN cDNA 1700001L05 gene	NA	0.311296	0.800657	1.240822	0.29366237	0.611919
627352	Morf4l1b	mortality factor 4 like 1B	NA	-0.09691	5.766246	-1.06948	0.29387487	0.612283
212712	Satb2	special AT-rich sequence binding protein 2, transcript	NA	0.082165	6.609817	1.058606	0.29394109	0.612343
18783	Pla2g4a	phospholipase A2, group IVA (cytosolic, calcium-dep	NA	-0.24805	0.853174	-1.1876	0.29408512	0.612564
17765	Mtf2	metal response element binding transcription factor 2	NA	0.062786	6.628432	1.044481	0.29418481	0.612656
18198	Musk	muscle, skeletal, receptor tyrosine kinase, transcript v	NA	0.454856	-0.59886	1.370646	0.2942046	0.612656
93730	Lztfl1	leucine zipper transcription factor-like 1, transcript va	NA	-0.06268	5.498124	-1.04441	0.2943016	0.61278
56222	Cited4	Cbp/p300-interacting transactivator, with Glu/Asp-rich	NA	0.337011	-0.26845	1.263137	0.29440093	0.612782
18826	Lcp1	lymphocyte cytosolic protein 1, transcript variant X5	NA	0.082847	4.403166	1.059106	0.2944024	0.612782
665211	Gm14326	predicted gene 14326, transcript variant X1	NA	0.103358	5.13804	1.074271	0.29441531	0.612782

115489709	Gm43560	predicted gene 43560	NA	0.172671	2.536134	1.127143	0.29449272	0.61279
320749	D630041G03Rik	RIKEN cDNA D630041G03 gene	NA	-0.22986	1.260658	-1.17272	0.29449473	0.61279
56334	Tmed2	transmembrane p24 trafficking protein 2	NA	0.048854	7.566265	1.034443	0.29454326	0.612809
16172	Il17ra	interleukin 17 receptor A	NA	-0.13305	2.809093	-1.09661	0.29457921	0.612809
269994	Gsg1l	GSG1-like, transcript variant X2	NA	-0.08265	4.950722	-1.05896	0.2947624	0.613112
97484	Cog8	component of oligomeric golgi complex 8	NA	-0.09429	4.243338	-1.06754	0.29483992	0.613195
66276	1810009A15Rik	RIKEN cDNA 1810009A15 gene, transcript variant 1	NA	-0.10932	4.001281	-1.07872	0.29505965	0.613348
15372	Hmx2	H6 homeobox 2, transcript variant 2	NA	-0.25234	0.644714	-1.19114	0.29510573	0.613348
381232	Mirt1	myocardial infarction associated transcript 1	NA	0.298973	0.13505	1.230268	0.29516318	0.613348
633285	Rbm46	RNA binding motif protein 46, transcript variant X5	NA	0.248461	0.648246	1.18794	0.29517715	0.613348
70757	Hacd2	3-hydroxyacyl-CoA dehydratase 2	NA	0.101935	4.368971	1.073212	0.29518206	0.613348
208439	Klhl29	kelch-like 29	NA	-0.05264	6.223433	-1.03716	0.29518217	0.613348
238161	Akap6	A kinase (PRKA) anchor protein 6	NA	0.064911	6.913032	1.04602	0.29521555	0.613348
240816	Rgsl1	regulator of G-protein signaling like 1, transcript varia	NA	0.414558	-0.33589	1.33289	0.29524757	0.613348
113523645	Lrrc70	leucine rich repeat containing 70	NA	0.413866	-0.08893	1.332251	0.29526293	0.613348
19317	Qk	quaking, transcript variant 2	NA	0.068457	8.007357	1.048594	0.29535712	0.613348
68729	Trim37	tripartite motif-containing 37, transcript variant 2	NA	0.046566	7.507816	1.032804	0.29536349	0.613348
16402	Itga5	integrin alpha 5 (fibronectin receptor alpha), transcrip	NA	-0.09803	3.973106	-1.07031	0.29536572	0.613348
381582	Tmem240	transmembrane protein 240	NA	0.1337	4.129539	1.097104	0.29552964	0.613532
230777	Hcrtr1	hypocretin (orexin) receptor 1, transcript variant X1	NA	0.270516	0.980203	1.206239	0.29553016	0.613532
654424	Mccc1os	methylcrotonoyl-Coenzyme A carboxylase 1 (alpha),	NA	-0.31823	-0.11801	-1.2468	0.29562807	0.613657
233899	Ccdc189	coiled-coil domain containing 189, transcript variant 1	NA	0.176234	2.043987	1.129931	0.29602253	0.614361
16418	Eif6	eukaryotic translation initiation factor 6	NA	0.069005	5.41215	1.048993	0.2961318	0.614361
78937	Avl9	AVL9 cell migration associated	NA	0.068306	6.432278	1.048485	0.29623129	0.614361
11477	Acvr1	activin A receptor, type 1, transcript variant 3	NA	-0.06525	5.441751	-1.04627	0.29625677	0.614361
329782	4930570G19Rik	RIKEN cDNA 4930570G19 gene, transcript variant 1	NA	0.181309	2.575691	1.133912	0.29626443	0.614361
620592	Tmem28	transmembrane protein 28	NA	-0.08685	4.181109	-1.06205	0.2962649	0.614361
67871	Mrrf	mitochondrial ribosome recycling factor	NA	-0.11052	4.147509	-1.07962	0.29628236	0.614361
216198	Tcp11l2	t-complex 11 (mouse) like 2	NA	-0.08995	4.507119	-1.06433	0.29630397	0.614361
229593	Golph3l	golgi phosphoprotein 3-like, transcript variant 2	NA	0.070792	5.150644	1.050293	0.29630685	0.614361
19132	Prph	peripherin, transcript variant 3	NA	0.231249	3.850182	1.173851	0.29637286	0.61442
66410	Mterf3	mitochondrial transcription termination factor 3	NA	-0.07309	5.225857	-1.05197	0.29654997	0.614708
242800	Ttc34	tetratricopeptide repeat domain 34	NA	0.288143	0.380648	1.221067	0.29662968	0.614795
20350	Sema3f	sema domain, immunoglobulin domain (Ig), short bas	NA	-0.05873	5.970849	-1.04155	0.29667349	0.614808
27059	Sh3d19	SH3 domain protein D19, transcript variant X2	NA	-0.07705	5.479187	-1.05486	0.29683525	0.615065
69232	Qrich1	glutamine-rich 1, transcript variant 2	NA	0.05199	7.093053	1.036694	0.2968864	0.615092
71990	Ddx54	DEAD box helicase 54, transcript variant X1	NA	-0.07361	6.012487	-1.05235	0.29710315	0.615399
102633573	Gm31365	predicted gene, 31365, transcript variant X3	NA	0.299485	0.536514	1.230705	0.29717348	0.615399
243653	Clec1a	C-type lectin domain family 1, member a, transcript v	NA	0.29329	0.278527	1.225432	0.29718487	0.615399
121021	Cspg4	chondroitin sulfate proteoglycan 4	NA	-0.10477	4.128836	-1.07532	0.29718557	0.615399
629059	Fam124a	family with sequence similarity 124, member A	NA	0.062177	5.759754	1.04404	0.29730233	0.615562
69608	Sec24d	Sec24 related gene family, member D (S. cerevisiae)	NA	0.071115	4.714028	1.050528	0.29737434	0.615633
100009600	Zglp1	zinc finger, GATA-like protein 1	NA	-0.26867	0.507682	-1.2047	0.29754416	0.615878
70821	4921507P07Rik	RIKEN cDNA 4921507P07 gene, transcript variant X	NA	0.157026	2.423363	1.114987	0.2975683	0.615878
105242685	Gm38834	predicted gene, 38834	NA	-0.3814	-0.47938	-1.30261	0.29763253	0.615932
106795	Tcf19	transcription factor 19, transcript variant 3	NA	-0.12969	3.677704	-1.09406	0.29780028	0.616129
19201	Pstpip2	proline-serine-threonine phosphatase-interacting prot	NA	0.176153	1.792306	1.129867	0.29781488	0.616129
26396	Map2k2	mitogen-activated protein kinase kinase 2, transcript	NA	0.052426	6.503638	1.037007	0.29784097	0.616129
13636	Efna1	ephrin A1, transcript variant 1	NA	-0.17201	2.549826	-1.12663	0.29795925	0.616235
68581	Tmed10	transmembrane p24 trafficking protein 10	NA	-0.04762	7.448755	-1.03356	0.29805191	0.616235
105242444	Gm38709	predicted gene, 38709	NA	-0.36435	0.711744	-1.2873	0.29806358	0.616235
107823	Nsd2	nuclear receptor binding SET domain protein 2, trans	NA	0.049213	8.068124	1.0347	0.29807065	0.616235
118568202	LOC118568202	uncharacterized LOC118568202	NA	-0.34567	-0.17994	-1.27074	0.29809958	0.616235
12466	Cct6a	chaperonin containing Tcp1, subunit 6a (zeta)	NA	0.045831	8.595849	1.032278	0.29818161	0.616235
224440	Setd4	SET domain containing 4, transcript variant X8	NA	0.126964	3.279854	1.091993	0.29819598	0.616235
110557	H2-Q6	histocompatibility 2, Q region locus 6, transcript varia	NA	0.28096	0.334866	1.215003	0.29819962	0.616235
23888	Gpc6	glypican 6, transcript variant 2	NA	0.095135	5.02685	1.068165	0.29823301	0.616235
68151	Wls	wntless WNT ligand secretion mediator, transcript va	NA	0.089624	5.949301	1.064093	0.29848319	0.616549
547127	Tmem181b-ps	transmembrane protein 181B, pseudogene	NA	-0.05185	7.071545	-1.03659	0.29848474	0.616549
12121	Bicd1	BICD cargo adaptor 1, transcript variant 1	NA	0.079522	5.614372	1.056668	0.29849865	0.616549
216578	Papolg	poly(A) polymerase gamma	NA	0.080241	5.221071	1.057195	0.29858915	0.616656
74288	Spem1	sperm maturation 1	NA	-0.36589	0.204093	-1.28868	0.29862639	0.616656
66998	Psmd5	proteasome (prosome, macropain) 26S subunit, non-	NA	0.067903	5.791185	1.048192	0.29875483	0.616724
214601	Slc10a3	solute carrier family 10 (sodium/bile acid cotransporte	NA	-0.17223	1.778165	-1.1268	0.29875885	0.616724
207792	BC034090	cDNA sequence BC034090, transcript variant X13	NA	-0.13827	3.287716	-1.10059	0.29877308	0.616724
14790	Grcc10	gene rich cluster, C10 gene	NA	-0.06193	6.230094	-1.04386	0.29884022	0.616735
791294	Gm9958	predicted gene 9958	NA	0.21519	1.638513	1.160857	0.29887549	0.616735
225518	Prdm6	PR domain containing 6, transcript variant X2	NA	-0.18427	1.537921	-1.13624	0.29889189	0.616735

237339	L3mbtl3	L3MBTL3 histone methyl-lysine binding protein, trans	NA	0.05167	6.270982	1.036464	0.2989888	0.616827
50927	Nasp	nuclear autoantigenic sperm protein (histone-binding)	NA	-0.05687	7.527085	-1.0402	0.29901271	0.616827
107047	Psmg2	proteasome (prosome, macropain) assembly chaper(	NA	-0.09202	4.41176	-1.06586	0.2991658	0.617024
104080	Nxph4	neurexophilin 4	NA	0.088489	5.21359	1.063256	0.29921497	0.617024
98417	Cnih4	cornichon family AMPA receptor auxiliary protein 4	NA	-0.0841	5.002259	-1.06003	0.29922168	0.617024
26932	Ppp2r5e	protein phosphatase 2, regulatory subunit B', epsilon	NA	0.050556	7.014626	1.035664	0.29926653	0.617038
13831	Epc1	enhancer of polycomb homolog 1, transcript variant 1	NA	0.054813	6.720203	1.038724	0.2994042	0.617096
17000	Ltbr	lymphotoxin B receptor	NA	-0.10368	3.630981	-1.07451	0.29941369	0.617096
17984	Ndn	necdin, MAGE family member	NA	0.054432	9.223933	1.03845	0.29949623	0.617096
20855	Stc1	stanniocalcin 1	NA	0.124035	3.411684	1.089778	0.2994998	0.617096
102637698	Gm34441	predicted gene, 34441, transcript variant X1	NA	-0.30862	0.447593	-1.23852	0.29959353	0.617096
21681	Alyref	Aly/REF export factor	NA	-0.07878	6.009363	-1.05612	0.29959971	0.617096
94181	Nans	N-acetylneuraminic acid synthase (sialic acid synthas	NA	-0.06414	5.749731	-1.04546	0.29961504	0.617096
66629	Golph3	golgi phosphoprotein 3	NA	0.061152	6.411542	1.043298	0.29967109	0.617096
67763	Prpsap1	phosphoribosyl pyrophosphate synthetase-associate(	NA	-0.06002	6.141686	-1.04248	0.29969373	0.617096
76482	Rmc1	regulator of MON1-CCZ1	NA	-0.08647	4.370754	-1.06177	0.2996967	0.617096
16499	Kcnab3	potassium voltage-gated channel, shaker-related sub	NA	0.136466	2.897673	1.099209	0.29971179	0.617096
68810	Nexn	nexilin, transcript variant 2	NA	-0.15299	2.356569	-1.11187	0.29985049	0.617304
546840	Ldlrad1	low density lipoprotein receptor class A domain conta	NA	0.290034	1.074635	1.222669	0.29999207	0.617501
70461	Crtc3	CREB regulated transcription coactivator 3	NA	0.075104	4.768167	1.053437	0.3000225	0.617501
100042165	Thoc2l	THO complex subunit 2-like, transcript variant X6	NA	0.069037	5.790106	1.049016	0.30013345	0.617652
102632132	Gm30286	predicted gene, 30286	NA	0.384652	-0.12162	1.305545	0.30036342	0.618047
56468	Socs5	suppressor of cytokine signaling 5, transcript variant)	NA	0.069805	5.414393	1.049575	0.30046317	0.618097
240879	Mettl11b	methyltransferase like 11B, transcript variant X3	NA	-0.45196	-0.24134	-1.36789	0.30046397	0.618097
14166	Fgf11	fibroblast growth factor 11, transcript variant X3	NA	-0.08174	5.372081	-1.0583	0.30062306	0.618298
319965	Cc2d1b	coiled-coil and C2 domain containing 1B	NA	0.080856	5.097436	1.057645	0.30069414	0.618298
227933	Ccdc148	coiled-coil domain containing 148, transcript variant 3	NA	-0.12723	3.270965	-1.0922	0.30070169	0.618298
331026	Gmppb	GDP-mannose pyrophosphorylase B, transcript varia	NA	-0.11842	3.277646	-1.08554	0.30072037	0.618298
22326	Vax1	ventral anterior homeobox 1	NA	-0.10027	4.218982	-1.07198	0.30078726	0.618298
52653	Nudcd2	NudC domain containing 2, transcript variant 1	NA	0.085495	4.72982	1.061052	0.30078959	0.618298
236920	Stard8	START domain containing 8, transcript variant X10	NA	-0.13992	3.148297	-1.10185	0.30098692	0.618626
791275	Gm9885	predicted gene 9885	NA	0.181855	1.779657	1.134341	0.30113737	0.6188
11769	Ap1s1	adaptor protein complex AP-1, sigma 1	NA	0.059782	6.549864	1.042308	0.30114785	0.6188
112407	Egln3	egl-9 family hypoxia-inducible factor 3	NA	0.087577	4.141143	1.062584	0.30126411	0.618961
76560	Prss8	protease, serine 8 (prostasin), transcript variant X1	NA	-0.39793	-0.80749	-1.31761	0.30133935	0.618967
244667	Disc1	disrupted in schizophrenia 1, transcript variant X1	NA	-0.11898	3.304568	-1.08597	0.30135164	0.618967
20016	Polr1c	polymerase (RNA) I polypeptide C	NA	-0.07438	5.400482	-1.05291	0.30138111	0.618967
68052	Rps13	ribosomal protein S13	NA	0.086036	7.991463	1.061449	0.3015146	0.619163
270035	Letm2	leucine zipper-EF-hand containing transmembrane pı	NA	0.089296	3.837309	1.063851	0.3017677	0.619604
227619	Man1b1	mannosidase, alpha, class 1B, member 1	NA	-0.05598	6.477815	-1.03957	0.30199187	0.619979
22771	Zic1	zinc finger protein of the cerebellum 1, transcript varia	NA	0.061634	8.116052	1.043647	0.3020508	0.619979
72947	Phykpl	5-phosphohydroxy-L-lysine phospholyase, transcript	NA	-0.197	2.08378	-1.14631	0.30209618	0.619979
212032	Hk3	hexokinase 3, transcript variant 3	NA	-0.35427	0.004312	-1.27834	0.30210278	0.619979
102634309	Gm11419	predicted gene 11419, transcript variant X23	NA	0.132388	2.620732	1.096106	0.3021642	0.620027
12040	Bckdhb	branched chain ketoacid dehydrogenase E1, beta po	NA	-0.08657	4.251863	-1.06184	0.30224643	0.620087
231830	Micall2	MICAL-like 2	NA	-0.13253	4.085997	-1.09621	0.30226953	0.620087
27409	Abcg5	ATP binding cassette subfamily G member 5	NA	0.27298	0.501208	1.208301	0.3023223	0.620117
13999	Gm14288	predicted gene 14288	NA	1.018054	-0.91441	2.025185	0.30238061	0.620158
52504	Cenpo	centromere protein O, transcript variant X5	NA	0.128388	3.209147	1.093072	0.30243037	0.620182
68023	Pdf	peptide deformylase (mitochondrial)	NA	-0.09936	4.643601	-1.0713	0.30251367	0.620227
12162	Bmp7	bone morphogenetic protein 7	NA	-0.08136	4.564101	-1.05801	0.30252855	0.620227
20873	Plk4	polo like kinase 4, transcript variant 1	NA	-0.10012	4.107823	-1.07187	0.30264197	0.620382
67945	Rpl41	ribosomal protein L41	NA	0.046025	8.673478	1.032416	0.30272306	0.62047
100616095	Snhg18	small nucleolar RNA host gene 18	NA	-0.15236	2.722001	-1.11139	0.30276168	0.620471
114585	D17H6S53E	DNA segment, Chr 17, human D6S53E	NA	-0.12114	3.116852	-1.08759	0.30294667	0.620772
56501	Elf4	E74-like factor 4 (ets domain transcription factor), trai	NA	0.154805	2.236144	1.113271	0.30305205	0.620877
224904	Micos13	mitochondrial contact site and cristae organizing syst	NA	0.084734	4.788178	1.060492	0.30308506	0.620877
271508	Brd8dc	BRD8 domain containing, transcript variant 1	NA	0.15752	2.131513	1.115368	0.30311258	0.620877
232974	Erfl	ETS repressor factor like	NA	-0.14156	2.509054	-1.10309	0.3031921	0.620894
320632	Snrnp200	small nuclear ribonucleoprotein 200 (U5)	NA	0.047098	7.171982	1.033185	0.30320974	0.620894
242864	Napepld	N-acyl phosphatidylethanolamine phospholipase D, tı	NA	-0.11368	3.024359	-1.08198	0.30325141	0.620894
118567738	LOC118567738	uncharacterized LOC118567738	NA	-0.25684	1.164994	-1.19486	0.30327349	0.620894
330216	Mblac1	metallo-beta-lactamase domain containing 1	NA	-0.20213	1.982715	-1.15039	0.30344699	0.621171
68441	Rraga	Ras-related GTP binding A	NA	0.055753	6.702462	1.039401	0.30359033	0.621268
66523	2810004N23Rik	RIKEN cDNA 2810004N23 gene	NA	-0.15764	3.645536	-1.11546	0.30363251	0.621268
12156	Bmp2	bone morphogenetic protein 2	NA	0.186588	1.966249	1.138069	0.30364728	0.621268
74996	Usp47	ubiquitin specific peptidase 47, transcript variant 2	NA	0.04862	7.59751	1.034275	0.30370648	0.621268
20365	Serf1	small EDRK-rich factor 1	NA	-0.08169	5.836321	-1.05826	0.30372175	0.621268

20317	Serpinf1	serine (or cysteine) peptidase inhibitor, clade F, mem	NA	0.091195	3.829344	1.065252	0.30372318	0.621268
22145	Tuba4a	tubulin, alpha 4A, transcript variant 1	NA	0.108109	3.744999	1.077815	0.30376567	0.621276
192197	Bcas3	breast carcinoma amplified sequence 3, transcript va	NA	-0.07411	4.83565	-1.05271	0.30382511	0.62132
21345	Tagln	transgelin	NA	0.19654	2.118202	1.145946	0.30388559	0.621365
19212	Pter	phosphotriesterase related, transcript variant 1	NA	-0.18679	2.03766	-1.13823	0.30396286	0.621445
109284	R3hdm4	R3H domain containing 4, transcript variant 2	NA	0.052737	7.455763	1.037231	0.30406288	0.621497
57780	Fxyd7	FXYD domain-containing ion transport regulator 7	NA	0.110885	4.390325	1.07989	0.3040646	0.621497
74197	Gtf2e1	general transcription factor II E, polypeptide 1 (alpha	NA	-0.07723	5.211687	-1.05499	0.30451048	0.62222
70120	Yars2	tyrosyl-tRNA synthetase 2 (mitochondrial), transcript	NA	-0.1106	3.641443	-1.07968	0.30453616	0.62222
76779	Cluap1	clusterin associated protein 1	NA	0.070596	5.059055	1.05015	0.30455586	0.62222
70054	Ccdc89	coiled-coil domain containing 89	NA	0.332442	0.313478	1.259143	0.30458841	0.62222
50785	Hs6st1	heparan sulfate 6-O-sulfotransferase 1	NA	-0.04964	6.96461	-1.03501	0.30460953	0.62222
74413	Tc2n	tandem C2 domains, nuclear, transcript variant X7	NA	-0.37357	-0.11066	-1.29555	0.3047935	0.622518
210789	Tbc1d4	TBC1 domain family, member 4, transcript variant X1	NA	0.09102	4.188565	1.065123	0.30487372	0.622604
56717	Mtor	mechanistic target of rapamycin kinase, transcript var	NA	0.053504	6.422208	1.037782	0.30503374	0.622828
73174	Tbkbp1	TBK1 binding protein 1, transcript variant 6	NA	-0.07611	5.883853	-1.05417	0.30506038	0.622828
244757	Glb1l2	galactosidase, beta 1-like 2, transcript variant X4	NA	0.173444	1.640307	1.127747	0.3051367	0.622833
15254	Hint1	histidine triad nucleotide binding protein 1	NA	0.086293	7.254434	1.061639	0.3051394	0.622833
57444	Isg20	interferon-stimulated protein, transcript variant 1	NA	-0.35004	0.81161	-1.2746	0.30524398	0.622969
13478	Dpagt1	dolichyl-phosphate (UDP-N-acetylglucosamine) acety	NA	-0.0692	4.851288	-1.04914	0.3054662	0.623282
105246802	Gm42027	predicted gene, 42027, transcript variant X3	NA	-0.41643	0.182977	-1.33462	0.30551252	0.623282
239528	Ago2	argonaute RISC catalytic subunit 2	NA	0.159947	4.339147	1.117246	0.30561856	0.623282
76311	1110019D14Rik	RIKEN cDNA 1110019D14 gene	NA	-0.28944	0.400444	-1.22217	0.30562545	0.623282
320225	Catsperg1	cation channel sperm associated auxiliary subunit ga	NA	-0.16142	2.930407	-1.11838	0.30563897	0.623282
330602	Gm5115	predicted gene 5115	NA	0.276087	0.404556	1.210906	0.30566329	0.623282
319236	Trim12c	tripartite motif-containing 12C, transcript variant 2	NA	-0.15862	2.21237	-1.11622	0.3056654	0.623282
19362	Rad51ap1	RAD51 associated protein 1, transcript variant 1	NA	-0.13447	3.106284	-1.09769	0.30585773	0.623546
24068	Sra1	steroid receptor RNA activator 1, transcript variant 1	NA	0.07705	4.963657	1.054859	0.30587183	0.623546
118568233	LOC118568233	uncharacterized LOC118568233	NA	-0.35685	-0.31751	-1.28062	0.30594777	0.62355
381236	Lipo3	lipase, member O3, transcript variant X1	NA	0.096668	3.746853	1.069301	0.30595502	0.62355
59040	Rhot1	ras homolog family member T1, transcript variant 1	NA	0.058316	6.38139	1.04125	0.30600734	0.62355
18997	Pou4f2	POU domain, class 4, transcription factor 2	NA	0.095069	4.350522	1.068116	0.30602696	0.62355
68777	Tmem53	transmembrane protein 53, transcript variant 2	NA	0.29932	0.596595	1.230565	0.30607439	0.623569
115490415	Gm44240	predicted gene, 44240	NA	0.197961	1.531679	1.147076	0.30616414	0.623616
67733	Itgb3bp	integrin beta 3 binding protein (beta3-endonexin), tra	NA	-0.20151	2.27875	-1.1499	0.30618761	0.623616
66873	Tril	TLR4 interactor with leucine-rich repeats	NA	0.114072	4.625346	1.082279	0.30621282	0.623616
414095	B130034C11Rik	RIKEN cDNA B130034C11 gene	NA	-0.19668	1.340889	-1.14606	0.30630719	0.623692
67006	Cisd2	CDGSH iron sulfur domain 2	NA	0.086231	5.870331	1.061593	0.30632674	0.623692
75173	Tex38	testis expressed 38	NA	0.525705	-0.27266	1.439637	0.30641485	0.623794
212326	Fam149a	family with sequence similarity 149, member A	NA	0.097291	4.251644	1.069763	0.3065164	0.623922
246048	Chodl	chondrolectin, transcript variant 1	NA	0.105994	3.307667	1.076235	0.30669557	0.624209
243574	Kbtbd8	kelch repeat and BTB (POZ) domain containing 8, tra	NA	-0.16314	3.137679	-1.11972	0.3067512	0.624244
228432	Ano3	anoctamin 3, transcript variant 2	NA	0.201704	1.552705	1.150056	0.30680431	0.624274
17912	Myo1b	myosin IB, transcript variant 1	NA	0.049224	6.757705	1.034709	0.30689462	0.62438
243866	Meiosin	meiosis initiator, transcript variant 1	NA	-0.14028	2.934376	-1.10212	0.30694069	0.624395
52206	Anapc4	anaphase promoting complex subunit 4	NA	0.051681	6.539852	1.036472	0.30705241	0.624545
70456	Mpc2	mitochondrial pyruvate carrier 2	NA	0.068671	5.162293	1.04875	0.30710035	0.624564
67582	Slc25a26	solute carrier family 25 (mitochondrial carrier, phosph	NA	0.124889	2.714042	1.090424	0.30718971	0.624668
102635553	Gm15624	predicted gene 15624, transcript variant X4	NA	-0.23294	1.151966	-1.17523	0.30726412	0.624684
14388	Gab1	growth factor receptor bound protein 2-associated pro	NA	0.067715	5.531204	1.048056	0.30727437	0.624684
67991	Nacc2	nucleus accumbens associated 2, BEN and BTB (PO	NA	0.071343	4.763745	1.050694	0.30736607	0.624792
244373	Erlin2	ER lipid raft associated 2, transcript variant 7	NA	0.061532	6.702969	1.043574	0.30753597	0.625059
75547	Akap13	A kinase (PRKA) anchor protein 13, transcript variant	NA	-0.06852	4.989224	-1.04864	0.30758101	0.625073
64010	Sav1	salvador family WW domain containing 1	NA	-0.06399	5.627556	-1.04535	0.3076752	0.625186
100042945	Gm4120	predicted gene 4120	NA	0.365147	0.278114	1.288013	0.30778967	0.625341
71371	Arid5b	AT rich interactive domain 5B (MRF1-like), transcript	NA	0.078073	5.252512	1.055607	0.30785432	0.625394
432611	Dnaic2	dynein, axonemal, intermediate chain 2, transcript va	NA	0.185409	1.574055	1.137139	0.30794361	0.625467
207165	Bptf	bromodomain PHD finger transcription factor, transcr	NA	0.056513	7.396117	1.039949	0.30798801	0.625467
76854	Gper1	G protein-coupled estrogen receptor 1	NA	0.147341	2.326706	1.107526	0.30800567	0.625467
233020	Hipk4	homeodomain interacting protein kinase 4, transcript	NA	-0.34954	0.177027	-1.27416	0.30810123	0.625501
67420	Far1	fatty acyl CoA reductase 1, transcript variant X3	NA	0.071753	6.44871	1.050993	0.30810269	0.625501
70615	Ankrd24	ankyrin repeat domain 24, transcript variant X22	NA	-0.08314	4.877924	-1.05932	0.30820958	0.625501
230903	Fbxo44	F-box protein 44, transcript variant X4	NA	0.068845	5.266128	1.048876	0.30828115	0.625501
13508	Dscam	DS cell adhesion molecule, transcript variant X1	NA	0.053173	6.585731	1.037545	0.30834	0.625501
20492	Slbp	stem-loop binding protein, transcript variant 1	NA	-0.05527	5.805549	-1.03905	0.30834191	0.625501
100041012	Gm3095	predicted gene 3095, transcript variant X3	NA	0.283463	0.371427	1.217113	0.30845238	0.625501
11490	Adam15	a disintegrin and metallopeptidase domain 15 (metarg	NA	-0.06861	5.246432	-1.04871	0.30847539	0.625501
54393	Gabbr1	gamma-aminobutyric acid (GABA) B receptor, 1	NA	0.048055	8.638306	1.03387	0.30857161	0.625501

106326	Osbpl11	oxysterol binding protein-like 11	NA	0.070522	5.252053	1.050097	0.30857226	0.625501
232934	Mypop	Myb-related transcription factor, partner of profilin	NA	-0.09954	3.759942	-1.07143	0.30863408	0.625501
14390	Gabpa	GA repeat binding protein, alpha, transcript variant X	NA	-0.06555	5.56804	-1.04648	0.3086415	0.625501
212508	Mtg1	mitochondrial ribosome-associated GTPase 1	NA	0.109282	3.590389	1.078691	0.30867071	0.625501
14534	Kat2a	K(lysine) acetyltransferase 2A, transcript variant 2	NA	-0.0535	6.302256	-1.03778	0.30869826	0.625501
217109	Utp18	UTP18 small subunit processome component	NA	0.064974	5.326451	1.046066	0.30872957	0.625501
102639019	Gm35438	predicted gene, 35438, transcript variant X5	NA	-0.31654	0.298339	-1.24534	0.30873373	0.625501
232430	Crebl2	cAMP responsive element binding protein-like 2, tran	NA	0.117109	3.150614	1.08456	0.30876946	0.625501
19878	Rock2	Rho-associated coiled-coil containing protein kinase	NA	0.066565	6.175039	1.04722	0.30878633	0.625501
22381	Tceal9	transcription elongation factor A like 9	NA	0.068966	6.878632	1.048965	0.30880503	0.625501
21415	Tcf7l1	transcription factor 7 like 1 (T cell specific, HMG box)	NA	-0.10092	3.964937	-1.07246	0.30883259	0.625501
11747	Anxa5	annexin A5	NA	-0.07951	5.071823	-1.05666	0.30886009	0.625501
18037	Nfkbie	nuclear factor of kappa light polypeptide gene enhan	NA	-0.17397	1.862076	-1.12816	0.3088678	0.625501
229600	BC028528	cDNA sequence BC028528, transcript variant 2	NA	0.168425	2.017413	1.123831	0.30892209	0.625533
208188	Ghsr	growth hormone secretagogue receptor, transcript va	NA	0.350467	-0.18913	1.274973	0.30899485	0.625534
12568	Cdk5	cyclin-dependent kinase 5, transcript variant 1	NA	0.056431	6.265376	1.03989	0.30899964	0.625534
66359	Cox20	cytochrome c oxidase assembly protein 20, transcript	NA	-0.10157	4.329508	-1.07294	0.30904509	0.625548
235041	Kank2	KN motif and ankyrin repeat domains 2	NA	-0.10232	4.159039	-1.0735	0.30911371	0.625609
118567546	LOC118567546	uncharacterized LOC118567546	NA	-0.40835	-0.4212	-1.32716	0.3091821	0.625633
223650	Eppk1	epiplakin 1	NA	-0.18979	1.649744	-1.1406	0.30932113	0.625633
18604	Pdk2	pyruvate dehydrogenase kinase, isoenzyme 2, transc	NA	-0.09265	4.767258	-1.06632	0.30934214	0.625633
268481	Krt222	keratin 222, transcript variant 1	NA	-0.13989	2.710813	-1.10182	0.30935008	0.625633
18195	Nsf	N-ethylmaleimide sensitive fusion protein	NA	0.049042	7.198358	1.034577	0.30938164	0.625633
212880	Ddx46	DEAD box helicase 46	NA	0.052747	6.746478	1.037238	0.30942738	0.625633
54411	Atp6ap1	ATPase, H+ transporting, lysosomal accessory protei	NA	0.046572	7.593945	1.032808	0.30949106	0.625633
22697	Zscan21	zinc finger and SCAN domain containing 21, transcrip	NA	0.059844	6.383116	1.042353	0.30949294	0.625633
14007	Celf2	CUGBP, Elav-like family member 2, transcript variant	NA	0.055751	9.540509	1.0394	0.30951668	0.625633
20732	Spint1	serine protease inhibitor, Kunitz type 1	NA	-0.32355	-0.01924	-1.25141	0.30955437	0.625633
66664	Tmem41a	transmembrane protein 41a, transcript variant 2	NA	0.102169	3.719702	1.073386	0.30964887	0.625633
105246035	Gm41396	predicted gene, 41396, transcript variant X2	NA	0.201909	1.310451	1.150219	0.30966412	0.625633
67277	2900089D17Rik	RIKEN cDNA 2900089D17 gene, transcript variant X	NA	-0.35572	-0.15479	-1.27962	0.30968222	0.625633
56791	Ube2l6	ubiquitin-conjugating enzyme E2L 6	NA	-0.21795	3.881481	-1.16308	0.30971458	0.625633
217351	Tnrc6c	trinucleotide repeat containing 6C, transcript variant	NA	0.054041	7.442602	1.038169	0.30972778	0.625633
74201	Cep97	centrosomal protein 97, transcript variant 1	NA	-0.07679	5.276876	-1.05467	0.30978664	0.625633
232910	Ap2s1	adaptor-related protein complex 2, sigma 1 subunit	NA	0.056632	6.645746	1.040035	0.30986395	0.625633
66446	Exosc7	exosome component 7	NA	0.076179	4.512248	1.054222	0.30991982	0.625633
13542	Dvl1	dishevelled segment polarity protein 1, transcript vari	NA	-0.05615	6.464435	-1.03969	0.30998218	0.625633
269400	Rtel1	regulator of telomere elongation helicase 1, transcript	NA	0.080582	4.892922	1.057445	0.31000984	0.625633
216616	Efemp1	epidermal growth factor-containing fibulin-like extrace	NA	-0.15406	2.115284	-1.11269	0.31001354	0.625633
18669	Abcb1b	ATP-binding cassette, sub-family B (MDR/TAP), men	NA	-0.3279	-0.04596	-1.25519	0.31008386	0.625633
58233	Dnaja4	DnaJ heat shock protein family (Hsp40) member A4,	NA	0.101695	3.681303	1.073034	0.3100845	0.625633
20409	Ostf1	osteoclast stimulating factor 1	NA	0.110705	3.807426	1.079756	0.31009595	0.625633
626870	Gm11992	predicted gene 11992	NA	0.255867	0.569644	1.194053	0.3101039	0.625633
78925	Srd5a1	steroid 5 alpha-reductase 1	NA	0.087895	3.931195	1.062818	0.31012513	0.625633
114128	Laptm4b	lysosomal-associated protein transmembrane 4B	NA	0.076311	4.584316	1.054318	0.31020039	0.625658
67155	Smarca2	SWI/SNF related, matrix associated, actin dependent	NA	0.057298	7.717415	1.040515	0.31021431	0.625658
30933	Tor2a	torsin family 2, member A, transcript variant 2	NA	0.08011	4.622369	1.057099	0.31028003	0.625669
105247151	Gm42303	predicted gene, 42303	NA	-0.17913	1.697977	-1.1322	0.31030418	0.625669
53328	Pgrmc1	progesterone receptor membrane component 1	NA	0.045322	8.428697	1.031913	0.31033542	0.625669
545622	Ptpn3	protein tyrosine phosphatase, non-receptor type 3, tr	NA	0.122373	3.22751	1.088524	0.31039611	0.62568
230996	9430015G10Rik	RIKEN cDNA 9430015G10 gene, transcript variant 2	NA	-0.08309	5.12611	-1.05928	0.31041765	0.62568
170791	Rbm39	RNA binding motif protein 39, transcript variant 3	NA	0.045253	8.309392	1.031864	0.31051858	0.625806
11949	Atp5c1	ATP synthase, H+ transporting, mitochondrial F1 com	NA	0.055757	7.717393	1.039404	0.31061308	0.625919
102638038	Gm15631	predicted gene 15631	NA	-0.19017	1.711774	-1.1409	0.31073702	0.626091
66822	Fbxo25	F-box protein 25, transcript variant 3	NA	0.055393	5.959079	1.039142	0.31088228	0.626306
18041	Nfs1	nitrogen fixation gene 1 (S. cerevisiae)	NA	-0.0809	4.372563	-1.05768	0.31092796	0.626321
229782	Slc35a3	solute carrier family 35 (UDP-N-acetylglucosamine (U	NA	0.101864	4.41589	1.073159	0.31106554	0.62652
74044	Ttf2	transcription termination factor, RNA polymerase II	NA	-0.12638	3.25869	-1.09155	0.31116088	0.626577
70122	Mllt3	myeloid/lymphoid or mixed-lineage leukemia; translo	NA	-0.0502	6.932865	-1.03541	0.31117087	0.626577
27214	Dbf4	DBF4 zinc finger, transcript variant 1	NA	-0.08371	4.574651	-1.05974	0.31123081	0.626621
104923	Adi1	acireductone dioxygenase 1	NA	-0.08293	4.102177	-1.05917	0.31131576	0.626714
319277	Washc4	WASH complex subunit 4, transcript variant X7	NA	0.071722	5.243368	1.050971	0.31141995	0.626808
14299	Ncs1	neuronal calcium sensor 1	NA	-0.0629	7.81045	-1.04456	0.31146213	0.626808
52231	Ankzf1	ankyrin repeat and zinc finger domain containing 1, tr	NA	-0.07977	4.449721	-1.05685	0.31151123	0.626808
68507	Ppfia4	protein tyrosine phosphatase, receptor type, f polype	NA	0.063264	5.813643	1.044827	0.31156995	0.626808
69780	Smap2	small ArfGAP 2	NA	-0.04871	7.02086	-1.03434	0.31157126	0.626808
74405	Efhc2	EF-hand domain (C-terminal) containing 2, transcript	NA	0.216511	1.210227	1.16192	0.31160481	0.626808
320452	P4ha3	procollagen-proline, 2-oxoglutarate 4-dioxygenase (p	NA	-0.09474	3.636129	-1.06788	0.31163197	0.626808

245404	Dcaf12l1	DDB1 and CUL4 associated factor 12-like 1, transcrip	NA	0.109942	4.009115	1.079185	0.31173013	0.626928
66939	Aagab	alpha- and gamma-adaptin binding protein, transcript	NA	0.059928	5.90696	1.042414	0.3117782	0.626947
18386	Oprd1	opioid receptor, delta 1, transcript variant X1	NA	-0.29011	0.497168	-1.22273	0.31186347	0.627041
13555	E2f1	E2F transcription factor 1, transcript variant 1	NA	-0.09541	3.9612	-1.06837	0.31196144	0.62716
67369	Qpctl	glutaminyl-peptide cyclotransferase-like	NA	0.087288	4.226689	1.062371	0.31208063	0.627323
231014	Elapor2	endosome-lysosome associated apoptosis and autop	NA	0.110504	5.053893	1.079605	0.31218773	0.627403
118568167	LOC118568167	uncharacterized LOC118568167	NA	-0.31956	1.212208	-1.24795	0.31222503	0.627403
74617	Scpep1	serine carboxypeptidase 1	NA	-0.07006	4.878221	-1.04976	0.31223638	0.627403
17535	Mre11a	MRE11A homolog A, double strand break repair nucl	NA	0.088513	4.301987	1.063273	0.31230076	0.627455
56045	Samhd1	SAM domain and HD domain, 1, transcript variant 1	NA	-0.07633	4.761745	-1.05433	0.31247283	0.627484
76367	Trp53rkb	transformation related protein 53 regulating kinase B	NA	0.148415	2.742066	1.108351	0.3124832	0.627484
26905	Eif2s3x	eukaryotic translation initiation factor 2, subunit 3, stru	NA	-0.06161	8.17511	-1.04363	0.31252708	0.627484
102632066	Gm30238	predicted gene, 30238	NA	0.22499	1.323891	1.168769	0.31253751	0.627484
67448	Plxdc2	plexin domain containing 2, transcript variant X1	NA	0.086426	4.894895	1.061737	0.31254489	0.627484
381217	Fam189a2	family with sequence similarity 189, member A2	NA	0.119105	3.277962	1.086061	0.31254666	0.627484
69094	Tmem160	transmembrane protein 160	NA	0.150107	2.887704	1.109652	0.31263928	0.627593
66895	Pxdc1	PX domain containing 1, transcript variant 2	NA	0.123012	2.766659	1.089006	0.31296938	0.627982
235626	Setd2	SET domain containing 2	NA	0.06104	6.913856	1.043218	0.31299903	0.627982
75555	Nscme3l	NSE3 homolog, SMC5-SMC6 complex component lik	NA	-0.41429	-0.43415	-1.33264	0.31301801	0.627982
74053	Grip1	glutamate receptor interacting protein 1, transcript va	NA	0.062569	5.960967	1.044324	0.31302134	0.627982
270166	Clpx	caseinolytic mitochondrial matrix peptidase chaperon	NA	0.065175	5.298103	1.046212	0.3130262	0.627982
217203	Tmem106a	transmembrane protein 106A, transcript variant 4	NA	0.229238	0.899653	1.172215	0.31320958	0.628213
70333	Cd3eap	CD3E antigen, epsilon polypeptide associated protein	NA	0.080339	4.452732	1.057267	0.31325549	0.628213
13713	Elk3	ELK3, member of ETS oncogene family, transcript va	NA	0.077667	4.583537	1.05531	0.31325736	0.628213
100042074	Gm3650	predicted gene 3650	NA	0.122671	3.286671	1.088749	0.31330955	0.628228
320633	Zbtb26	zinc finger and BTB domain containing 26	NA	-0.10726	4.223942	-1.07718	0.31334186	0.628228
22065	Trpc3	transient receptor potential cation channel, subfamily	NA	0.106135	4.014746	1.076341	0.31342695	0.628321
11796	Birc3	baculoviral IAP repeat-containing 3, transcript variant	NA	0.202794	1.134833	1.150926	0.31367797	0.628659
12856	Cox17	cytochrome c oxidase assembly protein 17, copper cl	NA	-0.10103	3.497914	-1.07254	0.31369956	0.628659
16780	Lamb3	laminin, beta 3, transcript variant X1	NA	-0.23947	0.578235	-1.18056	0.31374001	0.628659
68226	Efcab2	EF-hand calcium binding domain 2	NA	0.097051	3.564901	1.069585	0.31378983	0.628659
110417	Pigh	phosphatidylinositol glycan anchor biosynthesis, clas	NA	-0.10231	3.47854	-1.07349	0.31381462	0.628659
102632344	Gm28876	predicted gene 28876, transcript variant X1	NA	-0.33284	1.181761	-1.25949	0.31382725	0.628659
21749	Terf1	telomeric repeat binding factor 1, transcript variant 1	NA	-0.09761	3.859279	-1.07	0.31387624	0.62868
227697	Dolk	dolichol kinase	NA	0.110106	3.511046	1.079308	0.31412861	0.629085
270624	Spin4	spindlin family, member 4	NA	0.262952	1.418487	1.199932	0.31415602	0.629085
214359	Tmem51	transmembrane protein 51	NA	-0.15944	2.022034	-1.11685	0.31427442	0.629242
68559	Pdrg1	p53 and DNA damage regulated 1	NA	0.054916	6.023245	1.038799	0.31431165	0.629242
69091	Vps26b	VPS26 retromer complex component B	NA	0.045334	7.402817	1.031922	0.31452222	0.629553
235044	Plppr2	phospholipid phosphatase related 2, transcript varian	NA	-0.05043	7.385696	-1.03558	0.31454428	0.629553
102637367	Gm34196	predicted gene, 34196, transcript variant X1	NA	-0.65402	-0.38379	-1.57355	0.31475694	0.629901
27392	Pign	phosphatidylinositol glycan anchor biosynthesis, clas	NA	0.072387	4.560135	1.051455	0.31483131	0.629971
272381	Lrrc4b	leucine rich repeat containing 4B	NA	-0.05787	7.921578	-1.04093	0.31491008	0.629971
241327	Olfml2a	olfactomedin-like 2A	NA	0.210834	1.255205	1.157357	0.3149148	0.629971
21927	Tnfaip1	tumor necrosis factor, alpha-induced protein 1 (endot	NA	-0.04929	6.562755	-1.03475	0.31494667	0.629971
380840	Lyrm4	LYR motif containing 4	NA	0.078771	4.260334	1.056118	0.31504691	0.630051
105244007	Gm20045	predicted gene, 20045	NA	-0.12509	3.30072	-1.09058	0.31506423	0.630051
76889	Coq8b	coenzyme Q8B, transcript variant X2	NA	-0.09687	4.54849	-1.06945	0.31524435	0.630274
268741	Tox4	TOX high mobility group box family member 4	NA	-0.05674	6.440335	-1.04011	0.31525753	0.630274
208922	Cpeb3	cytoplasmic polyadenylation element binding protein	NA	0.086708	5.207596	1.061944	0.31529209	0.630274
66632	Dph6	diphthamine biosynthesis 6, transcript variant 2	NA	0.096072	4.047456	1.068859	0.31555385	0.630554
22137	Ttk	Ttk protein kinase, transcript variant 1	NA	-0.12807	3.470887	-1.09283	0.31557407	0.630554
23986	Eci2	enoyl-Coenzyme A delta isomerase 2, transcript varia	NA	0.056741	5.810564	1.040114	0.31559414	0.630554
226970	Arhgef4	Rho guanine nucleotide exchange factor (GEF) 4, tra	NA	0.094265	5.10872	1.067521	0.31561567	0.630554
215210	Tmem120a	transmembrane protein 120A	NA	0.077629	4.690623	1.055282	0.31562572	0.630554
67755	Ddx47	DEAD box helicase 47	NA	-0.05569	5.618119	-1.03935	0.31572533	0.630635
211151	Churc1	churchill domain containing 1, transcript variant 1	NA	0.098439	4.13063	1.070614	0.31574387	0.630635
17772	Mtm1	X-linked myotubular myopathy gene 1, transcript vari	NA	0.145175	2.364135	1.105865	0.31585847	0.630787
77286	Nkrf	NF-kappaB repressing factor	NA	0.063681	5.069249	1.045129	0.3159453	0.63085
105722	Ano6	anoctamin 6, transcript variant X3	NA	0.062946	5.803319	1.044597	0.315968	0.63085
100037278	Niban3	niban apoptosis regulator 3, transcript variant 1	NA	-0.1384	2.696628	-1.10068	0.31617076	0.631178
106205	Zc3h7a	zinc finger CCCH type containing 7 A, transcript varia	NA	0.06215	5.822646	1.044021	0.31623825	0.631235
329679	Fnip2	folliculin interacting protein 2	NA	0.0684	5.768254	1.048553	0.31640534	0.631351
12638	Cftr	cystic fibrosis transmembrane conductance regulator	NA	0.234473	0.786056	1.176477	0.31641276	0.631351
11846	Arg1	arginase, liver	NA	0.377252	-0.25541	1.298866	0.31644561	0.631351
20347	Sema3b	sema domain, immunoglobulin domain (Ig), short bas	NA	-0.19292	2.049923	-1.14307	0.31647165	0.631351
20111	Rps6ka1	ribosomal protein S6 kinase polypeptide 1, transcript	NA	-0.07337	4.540501	-1.05217	0.31654434	0.631351
223881	Rnd1	Rho family GTPase 1	NA	0.094032	3.885201	1.067349	0.3165648	0.631351

12829	Col4a4	collagen, type IV, alpha 4, transcript variant X1	NA	0.20002	1.227201	1.148714	0.3166159	0.631351
108168820	Gm34294	predicted gene, 34294	NA	0.418475	-0.28705	1.336514	0.316637	0.631351
77015	Mpped2	metallophosphoesterase domain containing 2, transc	NA	-0.05378	6.830362	-1.03798	0.31664566	0.631351
319945	Flad1	flavin adenine dinucleotide synthetase 1, transcript va	NA	-0.10553	4.469667	-1.07589	0.31669627	0.631375
66537	Pomp	proteasome maturation protein	NA	0.048841	6.672777	1.034434	0.31678582	0.631438
22367	Vrk1	vaccinia related kinase 1, transcript variant 2	NA	0.073542	5.580418	1.052297	0.31683802	0.631438
98404	AI597479	expressed sequence AI597479	NA	0.060028	5.627198	1.042486	0.31684458	0.631438
15939	Ier5	immediate early response 5	NA	-0.06336	6.169334	-1.0449	0.31691679	0.631505
66647	Nsmce3	NSE3 homolog, SMC5-SMC6 complex component	NA	-0.09034	4.150808	-1.06462	0.31700756	0.631609
268564	Zbtb1	zinc finger and BTB domain containing 1, transcript v	NA	0.099149	4.458517	1.071142	0.31707088	0.631657
94245	Dtnbp1	dystrobrevin binding protein 1	NA	0.078614	5.360859	1.056003	0.31718508	0.631808
619665	Klf14	Kruppel-like factor 14	NA	0.38533	-0.38641	1.306159	0.31744463	0.632247
69354	Slc38a4	solute carrier family 38, member 4, transcript variant	NA	-0.11722	3.279723	-1.08464	0.31762945	0.632538
11989	Slc7a3	solute carrier family 7 (cationic amino acid transporte	NA	-0.17128	2.199084	-1.12606	0.31771002	0.632621
71461	Ptk7	PTK7 protein tyrosine kinase 7, transcript variant X3	NA	-0.06304	5.803179	-1.04466	0.317791	0.632662
237211	Fancb	Fanconi anemia, complementation group B, transcrip	NA	-0.14828	2.287227	-1.10825	0.3179343	0.632662
17064	Cd93	CD93 antigen	NA	0.068364	5.781901	1.048527	0.31799129	0.632662
108800	Ston2	stonin 2, transcript variant X11	NA	-0.09019	4.329235	-1.06451	0.31799367	0.632662
74316	Isca2	iron-sulfur cluster assembly 2	NA	0.088668	4.498851	1.063388	0.31800491	0.632662
67534	Ttll4	tubulin tyrosine ligase-like family, member 4, transcrip	NA	-0.05805	5.70868	-1.04106	0.31802439	0.632662
434350	9430091E24Rik	RIKEN cDNA 9430091E24 gene, transcript variant 2	NA	-0.19976	1.609183	-1.14851	0.31803902	0.632662
70425	Csnk1g3	casein kinase 1, gamma 3, transcript variant X6	NA	0.060348	6.445378	1.042717	0.31806477	0.632662
107686	Snrpd2	small nuclear ribonucleoprotein D2, transcript variant	NA	0.070114	5.847991	1.049799	0.31810851	0.632662
67872	Nsmce4a	NSE4 homolog A, SMC5-SMC6 complex component	NA	0.058652	5.582654	1.041492	0.3181196	0.632662
54402	Stk19	serine/threonine kinase 19	NA	0.120337	3.602107	1.086989	0.31818147	0.632708
20301	Ccl27a	chemokine (C-C motif) ligand 27A, transcript variant X	NA	-0.05398	6.682857	-1.03812	0.3182771	0.632821
12522	Cd83	CD83 antigen, transcript variant 1	NA	0.117742	3.407054	1.085036	0.31836364	0.632915
118567473	LOC118567473	uncharacterized LOC118567473	NA	0.279973	0.630132	1.214172	0.31847265	0.632988
115488487	Gm52186	predicted gene, 52186, transcript variant X1	NA	0.377532	-0.35629	1.299117	0.31847793	0.632988
13804	Endog	endonuclease G	NA	0.177052	2.046038	1.130571	0.31854687	0.633048
22177	Tyrobp	TYRO protein tyrosine kinase binding protein	NA	0.164641	1.892443	1.120887	0.31867084	0.633217
98432	Phlpp1	PH domain and leucine rich repeat protein phosphata	NA	0.063199	5.630714	1.04478	0.31872791	0.633253
12983	Csf2rb	colony stimulating factor 2 receptor, beta, low-affinity	NA	0.354235	0.277034	1.278307	0.31878497	0.633289
105242595	Gm34871	predicted gene, 34871, transcript variant X5	NA	-0.16625	2.136942	-1.12213	0.31914171	0.63392
67128	Ube2g1	ubiquitin-conjugating enzyme E2G 1	NA	0.052838	6.937723	1.037304	0.31930998	0.634177
64602	Ireb2	iron responsive element binding protein 2	NA	0.061513	7.323089	1.043559	0.31939441	0.634267
102632597	Gm28523	predicted gene 28523	NA	-0.19309	1.359409	-1.14321	0.31946848	0.634334
239554	Foxred2	FAD-dependent oxidoreductase domain containing 2	NA	-0.09473	5.013161	-1.06787	0.31950586	0.634334
67387	Unc50	unc-50 homolog, transcript variant 2	NA	0.07389	4.843835	1.052551	0.31966825	0.634579
236690	Nyx	nyctalopin, transcript variant X2	NA	0.30117	0.511082	1.232143	0.31986172	0.634885
15064	Mr1	major histocompatibility complex, class I-related, tran	NA	0.221861	1.267854	1.166237	0.31999215	0.635067
233405	Vps33b	vacuolar protein sorting 33B	NA	0.067237	4.967254	1.047708	0.32014401	0.635291
66364	Pigbos1	Pigb opposite strand 1	NA	-0.17548	2.457996	-1.12934	0.32032275	0.6354
56551	Txn2	thioredoxin 2	NA	-0.05444	6.010769	-1.03846	0.32032897	0.6354
67197	Zcrb1	zinc finger CCHC-type and RNA binding motif 1, trans	NA	0.057986	5.514449	1.041011	0.32034443	0.6354
14854	Gss	glutathione synthetase, transcript variant 1	NA	-0.11548	2.821674	-1.08334	0.32035512	0.6354
27374	Prmt5	protein arginine N-methyltransferase 5, transcript vari	NA	-0.05176	6.583135	-1.03653	0.32039778	0.635401
72978	Cnih3	cornichon family AMPA receptor auxiliary protein 3, tr	NA	-0.08229	4.490512	-1.0587	0.32045785	0.635401
17281	Fyco1	FYVE and coiled-coil domain containing 1, transcript	NA	-0.08881	4.495148	-1.0635	0.32047302	0.635401
434008	Tmem178b	transmembrane protein 178B, transcript variant 2	NA	0.056278	7.283198	1.03978	0.32067227	0.635719
18004	Nek1	NIMA (never in mitosis gene a)-related expressed kin	NA	-0.06558	5.473604	-1.0465	0.32079265	0.63588
75291	Zbtb3	zinc finger and BTB domain containing 3, transcript v	NA	0.148489	2.368803	1.108408	0.32097822	0.636118
270076	Gcdh	glutaryl-Coenzyme A dehydrogenase, transcript varia	NA	-0.08395	4.535277	-1.05992	0.32101522	0.636118
66892	Eif4e3	eukaryotic translation initiation factor 4E member 3	NA	-0.08235	4.295823	-1.05874	0.32104977	0.636118
67290	3110040N11Rik	RIKEN cDNA 3110040N11 gene, transcript variant X	NA	0.100936	3.369687	1.072469	0.32111492	0.636118
218699	Pxk	PX domain containing serine/threonine kinase, transc	NA	-0.05548	5.648825	-1.0392	0.32113947	0.636118
18104	Nqo1	NAD(P)H dehydrogenase, quinone 1	NA	0.154484	1.998882	1.113024	0.32114728	0.636118
27279	Tnfrsf12a	tumor necrosis factor receptor superfamily, member 1	NA	-0.30825	0.090679	-1.2382	0.32122059	0.636186
76901	Jade2	jade family PHD finger 2, transcript variant X5	NA	0.200327	1.887717	1.148959	0.32130351	0.6362
15526	Hspa9	heat shock protein 9	NA	0.045669	7.690306	1.032162	0.32130613	0.6362
56070	Tcerg1	transcription elongation regulator 1 (CA150), transcrip	NA	0.054945	7.071101	1.038819	0.32156813	0.636615
17967	Ncam1	neural cell adhesion molecule 1, transcript variant 1	NA	0.04631	9.83074	1.032621	0.32163251	0.636615
27226	Pla2g7	phospholipase A2, group VII (platelet-activating facto	NA	0.095971	4.622807	1.068784	0.32163312	0.636615
11632	Aip	aryl-hydrocarbon receptor-interacting protein, transcri	NA	-0.07288	5.073486	-1.05182	0.32188719	0.637041
269513	Nkain3	Na+/K+ transporting ATPase interacting 3, transcript	NA	0.083854	5.802186	1.059846	0.32221462	0.637611
58238	Fam181b	family with sequence similarity 181, member B	NA	0.089242	4.478403	1.063811	0.32235051	0.63774
105244703	Gm40262	predicted gene, 40262, transcript variant X1	NA	-0.16754	2.07748	-1.12314	0.32235806	0.63774
101488143	Hbb-bt	hemoglobin, beta adult t chain	NA	-0.22369	9.331908	-1.16772	0.32243331	0.637811

66353	Riiad1	regulatory subunit of type II PKA R-subunit (RIIa) dor	NA	0.188953	1.622868	1.139936	0.32266765	0.638164
78903	Wrnip1	Werner helicase interacting protein 1	NA	-0.05426	5.725196	-1.03833	0.32269016	0.638164
27384	Akr1c13	aldo-keto reductase family 1, member C13	NA	0.297553	0.062475	1.229058	0.32274904	0.638203
67332	Snrpd3	small nuclear ribonucleoprotein D3	NA	-0.05492	6.395013	-1.0388	0.32290604	0.638436
27402	Pdhx	pyruvate dehydrogenase complex, component X	NA	0.064867	5.500873	1.045989	0.32304068	0.638624
69535	Ten1	TEN1 telomerase capping complex subunit	NA	-0.1019	3.858045	-1.07319	0.32315486	0.638773
19073	Srgn	serglycin, transcript variant X2	NA	0.183482	2.176264	1.135621	0.32326622	0.638859
19214	Ptgdr	prostaglandin D receptor	NA	-0.32188	-0.20617	-1.24996	0.32333322	0.638859
19877	Rock1	Rho-associated coiled-coil containing protein kinase	NA	0.055534	6.190224	1.039244	0.32336665	0.638859
16709	Ktn1	kinectin 1, transcript variant 12	NA	0.054535	6.835559	1.038524	0.32340258	0.638859
223776	Selenoo	selenoprotein O	NA	0.079478	4.337613	1.056635	0.32341182	0.638859
14232	Fkbp8	FK506 binding protein 8, transcript variant 1	NA	-0.04923	7.430515	-1.03471	0.32344193	0.638859
338355	Fkbp15	FK506 binding protein 15, transcript variant X1	NA	-0.06333	5.063137	-1.04487	0.32347342	0.638859
108168870	Gm43549	predicted gene 43549	NA	-0.32673	0.3227	-1.25417	0.32351355	0.638861
71957	Ints11	integrator complex subunit 11	NA	-0.06476	5.418694	-1.04591	0.32356377	0.638878
30932	Zfp330	zinc finger protein 330, transcript variant 1	NA	0.05587	5.555447	1.039486	0.32362752	0.638878
19885	Rorc	RAR-related orphan receptor gamma, transcript varia	NA	-0.30107	0.184671	-1.23206	0.32363986	0.638878
100683	Trrap	transformation/transcription domain-associated protei	NA	-0.04494	7.21654	-1.03164	0.32372663	0.638971
102640747	Gm15423	predicted gene 15423, transcript variant X3	NA	-0.35872	0.414765	-1.28229	0.32379392	0.639017
192196	Luc7l2	LUC7-like 2 (S. cerevisiae), transcript variant 3	NA	0.047084	8.033265	1.033174	0.32382838	0.639017
78575	B430319G15Rik	RIKEN cDNA B430319G15 gene	NA	0.284317	0.918837	1.217833	0.32392427	0.639119
93742	Pard3	par-3 family cell polarity regulator, transcript variant 5	NA	-0.05595	5.563618	-1.03954	0.32395852	0.639119
76980	Ube2ql1	ubiquitin-conjugating enzyme E2Q family-like 1	NA	0.06197	7.523469	1.04389	0.32410123	0.639138
70791	Hars2	histidyl-tRNA synthetase 2, transcript variant X2	NA	0.06364	5.409099	1.045099	0.32410272	0.639138
11515	Adcy9	adenylate cyclase 9, transcript variant X2	NA	0.074452	4.432086	1.052961	0.32416596	0.639138
381199	Tmem151a	transmembrane protein 151A, transcript variant 1	NA	0.086391	4.302283	1.061711	0.32417499	0.639138
66959	Dusp26	dual specificity phosphatase 26 (putative), transcript	NA	0.064637	6.005819	1.045822	0.32419069	0.639138
234797	6430548M08Rik	RIKEN cDNA 6430548M08 gene, transcript variant X	NA	-0.05826	6.009096	-1.04121	0.32425305	0.639138
16889	Lipa	lysosomal acid lipase A, transcript variant 1	NA	-0.07437	4.654109	-1.0529	0.32426095	0.639138
210582	Coq10a	coenzyme Q10A, transcript variant 2	NA	0.08349	4.721095	1.059578	0.32432892	0.639138
21975	Top3a	topoisomerase (DNA) III alpha	NA	-0.09987	3.806716	-1.07168	0.32434293	0.639138
74249	Lrrc2	leucine rich repeat containing 2	NA	0.373724	-0.20287	1.295693	0.32436116	0.639138
20683	Sp1	trans-acting transcription factor 1, transcript variant X	NA	-0.05823	5.815768	-1.04119	0.3244415	0.639169
67263	Zswim6	zinc finger SWIM-type containing 6	NA	0.074399	6.156472	1.052922	0.3244848	0.639169
108168806	Gm45609	predicted gene 45609, transcript variant X2	NA	0.3562	0.231158	1.280049	0.32449449	0.639169
380768	Ccdc177	coiled-coil domain containing 177	NA	-0.0788	4.487513	-1.05614	0.32462367	0.639346
102634953	Gm32412	predicted gene, 32412, transcript variant X1	NA	-0.31244	0.070323	-1.24181	0.32486202	0.639738
19184	Psmc5	protease (prosome, macropain) 26S subunit, ATPase	NA	0.067417	6.827835	1.047839	0.32490583	0.639747
240690	St18	suppression of tumorigenicity 18, transcript variant X	NA	0.078746	4.11828	1.0561	0.32502255	0.639772
67526	Atg12	autophagy related 12	NA	0.059734	6.690581	1.042273	0.32503757	0.639772
279618	Gm715	predicted gene 715	NA	0.179087	1.673883	1.132167	0.3251054	0.639772
192654	Pla2g15	phospholipase A2, group XV, transcript variant 1	NA	-0.07717	4.469744	-1.05495	0.32513298	0.639772
110074	Dut	deoxyuridine triphosphatase, transcript variant 1	NA	-0.06778	5.481134	-1.04811	0.32516588	0.639772
67040	Ddx17	DEAD box helicase 17, transcript variant 3	NA	-0.04304	9.460724	-1.03028	0.32521644	0.639772
56398	Chp1	calcineurin-like EF hand protein 1	NA	-0.0616	6.40864	-1.04362	0.32522089	0.639772
94062	Mrpl3	mitochondrial ribosomal protein L3, transcript variant	NA	0.05936	5.884973	1.042003	0.32526611	0.639772
15464	Hrc	histidine rich calcium binding protein	NA	-0.24311	0.78234	-1.18354	0.32528647	0.639772
67889	Rbm18	RNA binding motif protein 18, transcript variant 1	NA	0.048284	6.531678	1.034034	0.32531192	0.639772
225898	Eml3	echinoderm microtubule associated protein like 3, tra	NA	0.100911	3.663823	1.07245	0.32545602	0.639978
57317	Srsf4	serine and arginine-rich splicing factor 4, transcript va	NA	-0.06142	6.945921	-1.04349	0.32551055	0.640008
78329	2310010J17Rik	RIKEN cDNA 2310010J17 gene, transcript variant 2	NA	0.283517	0.172935	1.217159	0.32559074	0.640089
64099	Parvg	parvin, gamma, transcript variant 2	NA	0.267923	0.460355	1.204073	0.32574664	0.640151
319901	Dsel	dermatan sulfate epimerase-like, transcript variant X2	NA	0.073887	4.467277	1.052549	0.3257674	0.640151
102640710	Gm36712	predicted gene, 36712	NA	-0.37183	-0.41083	-1.29399	0.32579072	0.640151
106014251	Gm28043	predicted gene, 28043	NA	0.210524	2.203083	1.157109	0.32579403	0.640151
27410	Abca3	ATP-binding cassette, sub-family A (ABC1), member	NA	-0.04875	6.502729	-1.03437	0.3259395	0.640151
102635552	Gm32856	predicted gene, 32856, transcript variant X3	NA	0.120826	3.13745	1.087357	0.32594953	0.640151
67345	Herc4	hect domain and RLD 4, transcript variant X10	NA	-0.0604	5.288318	-1.04276	0.32595277	0.640151
63913	Niban1	niban apoptosis regulator 1, transcript variant X1	NA	-0.23559	1.190985	-1.17738	0.32597618	0.640151
11601	Angpt2	angiopoietin 2	NA	0.151046	2.385731	1.110374	0.32597639	0.640151
105245904	Gm41284	predicted gene, 41284	NA	-0.06154	6.286857	-1.04358	0.32606884	0.640255
66204	Acyp1	acylphosphatase 1, erythrocyte (common) type, trans	NA	-0.11858	3.361983	-1.08567	0.32615152	0.640286
224912	Crb3	crumbs family member 3, transcript variant X1	NA	0.36138	-0.39145	1.284654	0.32616364	0.640286
270058	Map1s	microtubule-associated protein 1S	NA	-0.09403	5.141483	-1.06735	0.32620292	0.640286
225348	Wdr36	WD repeat domain 36, transcript variant 1	NA	0.073675	5.271462	1.052394	0.32630666	0.640404
56050	Cyp39a1	cytochrome P450, family 39, subfamily a, polypeptide	NA	-0.11043	3.383823	-1.07955	0.32634749	0.640404
66405	Mcts2	malignant T cell amplified sequence 2	NA	-0.11865	3.281386	-1.08572	0.32638074	0.640404
100861668	Gm21119	predicted gene, 21119, transcript variant X2	NA	-0.43057	-0.51621	-1.34776	0.32647003	0.640502

75339	Mphosph8	M-phase phosphoprotein 8, transcript variant 1	NA	-0.05547	5.978621	-1.0392	0.3265273	0.640537
78286	Nav2	neuron navigator 2, transcript variant X43	NA	0.049308	7.060867	1.034768	0.32657293	0.640549
216274	Cep290	centrosomal protein 290	NA	-0.10022	3.726024	-1.07194	0.32676879	0.640726
27008	Micall1	microtubule associated monooxygenase, calponin an	NA	0.058731	6.536441	1.041549	0.32680882	0.640726
71846	Syce2	synaptonemal complex central element protein 2, tra	NA	-0.14324	3.453979	-1.10438	0.32681473	0.640726
94043	Tm2d1	TM2 domain containing 1	NA	-0.1247	4.698803	-1.09028	0.32682053	0.640726
68271	Zfp85os	zinc finger protein 85, opposite strand	NA	-0.20262	1.315286	-1.15078	0.32688302	0.640771
66459	Pyurf	Pigy upstream reading frame	NA	0.08688	3.997246	1.062071	0.32701542	0.640815
68401	G6pc3	glucose 6 phosphatase, catalytic, 3	NA	-0.08177	4.510217	-1.05831	0.3270331	0.640815
56846	Necab3	N-terminal EF-hand calcium binding protein 3, transc	NA	-0.11667	3.118049	-1.08423	0.32705721	0.640815
56695	Pnkd	paroxysmal nonkinesiogenic dyskinesia, transcript va	NA	-0.05418	6.139288	-1.03827	0.32707518	0.640815
235330	Ttc12	tetratricopeptide repeat domain 12, transcript variant	NA	-0.17031	1.777629	-1.1253	0.3271021	0.640815
20822	Ro60	Ro60, Y RNA binding protein	NA	0.074195	6.860433	1.052773	0.32718002	0.640836
56213	Htra1	HtrA serine peptidase 1	NA	-0.11415	3.789221	-1.08234	0.327192	0.640836
213393	Depp1	DEPP1 autophagy regulator, transcript variant 1	NA	0.326438	1.697623	1.253913	0.32726339	0.640847
54125	Polm	polymerase (DNA directed), mu, transcript variant X2	NA	-0.10583	3.406794	-1.07611	0.32730618	0.640847
57750	Wdr12	WD repeat domain 12, transcript variant 2	NA	0.065749	5.187928	1.046628	0.32731578	0.640847
69740	Dph5	diphthamide biosynthesis 5, transcript variant X3	NA	0.127241	3.284739	1.092203	0.32749484	0.641121
26894	Cops7a	COP9 signalosome subunit 7A, transcript variant 4	NA	0.065654	5.289342	1.046559	0.32754925	0.64115
18011	Neurl1a	neuralized E3 ubiquitin protein ligase 1A, transcript v	NA	-0.07711	4.988787	-1.0549	0.32764123	0.641253
70799	Cep192	centrosomal protein 192, transcript variant X5	NA	-0.07815	4.940578	-1.05567	0.32773008	0.641287
229776	Cdc14a	CDC14 cell division cycle 14A, transcript variant 3	NA	-0.10501	3.161123	-1.0755	0.32773762	0.641287
11532	Adh5	alcohol dehydrogenase 5 (class III), chi polypeptide, t	NA	0.052694	7.034449	1.0372	0.3277869	0.641307
24128	Xrn2	5'-3' exoribonuclease 2, transcript variant 1	NA	0.047932	6.960496	1.033782	0.32785419	0.641361
102635868	Gm26728	predicted gene, 26728, transcript variant X1	NA	0.310917	0.025731	1.240496	0.32794047	0.64142
18424	Otx2	orthodenticle homeobox 2, transcript variant 3	NA	0.07666	5.429463	1.054573	0.32799859	0.64142
66169	Tomm7	translocase of outer mitochondrial membrane 7	NA	-0.06841	5.449601	-1.04856	0.32800264	0.64142
100705	Acacb	acetyl-Coenzyme A carboxylase beta, transcript varia	NA	0.179441	1.863625	1.132445	0.32809806	0.64153
102632116	Gm30273	predicted gene, 30273, transcript variant X3	NA	0.410524	-0.63984	1.329169	0.32815528	0.641565
74781	Wipi2	WD repeat domain, phosphoinositide interacting 2, tr	NA	-0.04812	6.331021	-1.03392	0.32823702	0.641647
68449	Tbc1d10b	TBC1 domain family, member 10b	NA	-0.05194	6.181418	-1.03666	0.3284038	0.641787
18643	Pfn1	profilin 1	NA	-0.07434	7.329257	-1.05288	0.32842299	0.641787
218630	Ccno	cyclin O	NA	-0.2972	0.072449	-1.22875	0.32842665	0.641787
69482	Nup35	nucleoporin 35, transcript variant 1	NA	-0.06854	5.019307	-1.04865	0.3284728	0.6418
75420	Secisbp2	SECIS binding protein 2, transcript variant 2	NA	-0.07678	4.505562	-1.05466	0.32854987	0.641873
320742	A230072C01Rik	RIKEN cDNA A230072C01 gene, transcript variant 2	NA	-0.121	3.276367	-1.08749	0.32860003	0.641894
66771	Gid4	GID complex subunit 4, VID24 homolog	NA	-0.06129	5.587288	-1.0434	0.32880292	0.642145
66196	Myo19	myosin XIX, transcript variant X4	NA	0.074396	4.47796	1.05292	0.32880723	0.642145
67615	Ube2r2	ubiquitin-conjugating enzyme E2R 2	NA	-0.05132	7.205414	-1.03621	0.32891564	0.642262
104859	Tecpr2	tectonin beta-propeller repeat containing 2, transcript	NA	0.061745	5.28171	1.043727	0.32898221	0.642262
51797	Ctps	cytidine 5'-triphosphate synthase, transcript variant 1	NA	-0.04834	6.350412	-1.03407	0.32901822	0.642262
57436	Gabarapl1	gamma-aminobutyric acid (GABA) A receptor-associa	NA	-0.04655	7.454396	-1.03279	0.32902533	0.642262
12144	Blm	Bloom syndrome, RecQ like helicase, transcript varia	NA	0.092693	3.918356	1.066359	0.32910533	0.642342
433022	Plcxd2	phosphatidylinositol-specific phospholipase C, X dom	NA	0.077923	5.265696	1.055497	0.32935406	0.64275
60322	Chst7	carbohydrate (N-acetylglucosamino) sulfotransferase	NA	-0.19287	1.846879	-1.14304	0.32951849	0.642994
118567769	LOC118567769	uncharacterized LOC118567769	NA	0.166689	2.095006	1.122479	0.32963546	0.643102
102632743	Gm30735	predicted gene, 30735, transcript variant X9	NA	-0.30074	0.109578	-1.23178	0.32971496	0.643102
215335	Slc36a1	solute carrier family 36 (proton/amino acid symporter	NA	-0.06977	5.210178	-1.04955	0.32971747	0.643102
68501	Nsmce2	NSE2/MMS21 homolog, SMC5-SMC6 complex SUM	NA	0.069634	5.000845	1.049451	0.32976091	0.643102
406218	Panx2	pannexin 2	NA	-0.07631	4.591281	-1.05432	0.32977157	0.643102
107376	E330013P04Rik	RIKEN cDNA E330013P04 gene	NA	-0.25212	0.76781	-1.19096	0.32981388	0.643107
68209	Rnaseh2c	ribonuclease H2, subunit C	NA	-0.13296	3.599218	-1.09654	0.32996213	0.643285
58520	Erg28	ergosterol biosynthesis 28, transcript variant 1	NA	0.066808	5.249273	1.047397	0.32998391	0.643285
20866	Stim1	stromal interaction molecule 1, transcript variant X2	NA	-0.07375	4.713872	-1.05245	0.3301218	0.643477
239659	C1ql4	complement component 1, q subcomponent-like 4, tr	NA	0.209496	2.703669	1.156284	0.33016259	0.643479
26372	Clcn6	chloride channel, voltage-sensitive 6	NA	-0.05214	6.622223	-1.0368	0.33041598	0.643752
19175	Psmb6	proteasome (prosome, macropain) subunit, beta type	NA	0.055398	6.880137	1.039146	0.33042883	0.643752
239337	Adamts12	a disintegrin-like and metallopeptidase (reprolysin typ	NA	0.117979	3.554503	1.085214	0.33043806	0.643752
208043	Setd1b	SET domain containing 1B	NA	-0.05587	6.558512	-1.03948	0.33046072	0.643752
20271	Scn5a	sodium channel, voltage-gated, type V, alpha, transc	NA	-0.17256	2.164022	-1.12706	0.3305152	0.643781
18101	Nmbr	neuromedin B receptor	NA	0.270537	0.354505	1.206257	0.33071161	0.644008
52443	Mrpl48	mitochondrial ribosomal protein L48, transcript varian	NA	0.06126	5.243107	1.043377	0.3307136	0.644008
56296	Dmrtb1	DMRT-like family B with proline-rich C-terminal, 1, tra	NA	-0.12785	3.744498	-1.09267	0.3307508	0.644008
100042332	2810410L24Rik	RIKEN cDNA 2810410L24 gene	NA	-0.12921	3.194429	-1.0937	0.33079355	0.644014
13116	Cyp46a1	cytochrome P450, family 46, subfamily a, polypeptide	NA	0.078504	4.029563	1.055922	0.33084678	0.644041
115488784	Gm5817	predicted gene 5817	NA	0.237785	0.62485	1.179181	0.330902	0.644071
77305	Wdr82	WD repeat domain containing 82, transcript variant X	NA	0.042203	7.976044	1.029685	0.33096215	0.644111
26939	Polr3e	polymerase (RNA) III (DNA directed) polypeptide E, t	NA	0.062131	5.298288	1.044006	0.33107081	0.644246

103711	Pnpo	pyridoxine 5'-phosphate oxidase	NA	-0.10221	4.248185	-1.07342	0.3311283	0.644281
226922	Kcnq5	potassium voltage-gated channel, subfamily Q, meml	NA	0.088617	3.769594	1.06335	0.33127362	0.644486
333050	Ksr2	kinase suppressor of ras 2	NA	0.180791	2.862038	1.133505	0.33132989	0.644519
23893	Grem2	gremlin 2, DAN family BMP antagonist	NA	0.101326	3.235069	1.072759	0.33141623	0.644559
19729	Slc50a1	solute carrier family 50 (sugar transporter), member 1	NA	0.096277	3.452047	1.069011	0.3314519	0.644559
22284	Usp9x	ubiquitin specific peptidase 9, X chromosome	NA	0.052192	8.168676	1.036839	0.33146956	0.644559
99730	Taf13	TATA-box binding protein associated factor 13	NA	0.08123	5.031574	1.05792	0.33161806	0.644771
20364	Selenow	selenoprotein W	NA	0.050009	8.223032	1.035271	0.33174796	0.644897
69524	Esam	endothelial cell-specific adhesion molecule, transcript	NA	0.076281	4.311891	1.054296	0.33176185	0.644897
72203	2610507I01Rik	RIKEN cDNA 2610507I01 gene	NA	-0.08777	4.369073	-1.06273	0.33187854	0.645046
67923	Eloc	elongin C, transcript variant X12	NA	0.050055	7.02933	1.035304	0.3319254	0.64506
12036	Bcat2	branched chain aminotransferase 2, mitochondrial, tr	NA	-0.08761	4.201042	-1.06261	0.33197128	0.645072
93715	Pcdhga7	protocadherin gamma subfamily A, 7	NA	0.090443	4.690095	1.064697	0.33217476	0.645391
68145	Etaa1	Ewing tumor-associated antigen 1, transcript variant	NA	0.08737	4.198435	1.062432	0.33225711	0.645474
107999	Gtpbp6	GTP binding protein 6 (putative), transcript variant X1	NA	-0.08396	4.51141	-1.05992	0.33236927	0.64553
17237	Mgrn1	mahogunin, ring finger 1, transcript variant 6	NA	0.048893	7.037881	1.034471	0.3324676	0.64553
236539	Phgdh	3-phosphoglycerate dehydrogenase	NA	0.051918	6.233166	1.036642	0.33246916	0.64553
102635458	Gm32790	predicted gene, 32790, transcript variant X8	NA	-0.21056	1.485341	-1.15714	0.3325475	0.64553
11859	Phox2a	paired-like homeobox 2a	NA	-0.42956	-0.14729	-1.34682	0.33254822	0.64553
231327	Ppat	phosphoribosyl pyrophosphate amidotransferase	NA	0.087929	4.329591	1.062843	0.33258102	0.64553
68034	Fam122a	family with sequence similarity 122, member A	NA	0.098875	4.207123	1.070938	0.33261003	0.64553
54351	Elp5	elongator acetyltransferase complex subunit 5, transc	NA	-0.05278	5.976529	-1.03726	0.33262172	0.64553
381406	Trp53rka	transformation related protein 53 regulating kinase A	NA	0.078866	4.793591	1.056188	0.33264311	0.64553
226470	Zbtb41	zinc finger and BTB domain containing 41	NA	0.070269	6.399078	1.049913	0.33274095	0.645581
328232	Gfod1	glucose-fructose oxidoreductase domain containing 1	NA	-0.0887	4.798934	-1.06341	0.33274873	0.645581
68458	Ppp1r14a	protein phosphatase 1, regulatory inhibitor subunit 14	NA	-0.29105	0.509749	-1.22353	0.33287202	0.645726
12836	Col7a1	collagen, type VII, alpha 1	NA	-0.15047	2.923989	-1.10993	0.33290283	0.645726
105245994	Gm41361	predicted gene, 41361, transcript variant X1	NA	-0.38439	0.841087	-1.3053	0.33298652	0.645766
69216	Svbp	small vasohibin binding protein, transcript variant 2	NA	0.078183	5.322063	1.055688	0.33300311	0.645766
239650	Ccdc184	coiled-coil domain containing 184	NA	-0.06224	6.065747	-1.04409	0.3332775	0.6462
83925	Trps1	transcriptional repressor GATA binding 1, transcript v	NA	-0.07182	4.729987	-1.05104	0.33330612	0.6462
58250	Chst11	carbohydrate sulfotransferase 11, transcript variant X	NA	-0.08675	5.295709	-1.06197	0.33341965	0.646242
217344	Rhbdf2	rhomboid 5 homolog 2, transcript variant 2	NA	0.245621	0.937838	1.185603	0.33342317	0.646242
332359	Tigd3	tigger transposable element derived 3, transcript varia	NA	-0.10059	3.846904	-1.07221	0.33344692	0.646242
238803	Zfp366	zinc finger protein 366	NA	0.333834	-0.01459	1.260359	0.3334905	0.64625
50916	Irx4	Iroquois homeobox 4, transcript variant X5	NA	0.224003	1.034381	1.16797	0.33362168	0.646418
13821	Epb41l1	erythrocyte membrane protein band 4.1 like 1, transc	NA	-0.0507	7.867866	-1.03577	0.33369217	0.646418
105244797	Gm40345	predicted gene, 40345	NA	0.304191	0.07588	1.234726	0.33369682	0.646418
56012	Pgam2	phosphoglycerate mutase 2	NA	-0.35746	0.173646	-1.28117	0.3339061	0.646747
232989	Hnrnpul1	heterogeneous nuclear ribonucleoprotein U-like 1, tra	NA	-0.04331	8.021014	-1.03048	0.33395022	0.646755
22720	Zfp62	zinc finger protein 62, transcript variant X4	NA	-0.05255	5.965672	-1.03709	0.3340328	0.646838
269113	Nup54	nucleoporin 54, transcript variant 2	NA	0.062593	5.534607	1.044341	0.33409984	0.646844
100702	Gbp6	guanylate binding protein 6	NA	-0.19555	1.796786	-1.14516	0.33411531	0.646844
246696	Slc25a28	solute carrier family 25, member 28, transcript varian	NA	0.082267	4.722808	1.05868	0.33418402	0.646854
80914	Uck2	uridine-cytidine kinase 2, transcript variant X3	NA	-0.04806	6.707545	-1.03388	0.3341997	0.646854
209212	Osgin2	oxidative stress induced growth inhibitor family memb	NA	-0.07771	4.196995	-1.05534	0.33425451	0.646883
20630	Snrpc	U1 small nuclear ribonucleoprotein C, transcript varia	NA	0.060889	5.671965	1.043109	0.33443127	0.646998
75665	Bicdl1	BICD family like cargo adaptor 1, transcript variant 2	NA	0.056218	5.568589	1.039736	0.33446395	0.646998
241627	Wdr76	WD repeat domain 76, transcript variant 2	NA	-0.09319	3.536171	-1.06673	0.3344754	0.646998
19713	Ret	ret proto-oncogene, transcript variant 4	NA	0.102433	4.369279	1.073582	0.33447827	0.646998
320869	Spata33	spermatogenesis associated 33, transcript variant X4	NA	0.158742	2.748929	1.116313	0.33452007	0.646998
192734	Lrrc75b	leucine rich repeat containing 75B, transcript variant	NA	-0.06777	6.442556	-1.0481	0.33455247	0.646998
229700	Rbm15	RNA binding motif protein 15, transcript variant X2	NA	-0.09962	5.114299	-1.07149	0.33462448	0.64706
12837	Col8a1	collagen, type VIII, alpha 1	NA	0.114349	3.275544	1.082487	0.33471711	0.647162
97064	Wwtr1	WW domain containing transcription regulator 1, tran	NA	0.067697	5.405329	1.048043	0.33479944	0.647228
237979	Sdk2	sidekick cell adhesion molecule 2, transcript variant X	NA	-0.06686	4.830882	-1.04743	0.33486765	0.647228
19275	Ptprn	protein tyrosine phosphatase, receptor type, N, transc	NA	0.06754	5.626964	1.047928	0.33487073	0.647228
16190	Il4ra	interleukin 4 receptor, alpha, transcript variant 1	NA	-0.13601	2.671458	-1.09886	0.33498143	0.647296
73062	Ppp1r16a	protein phosphatase 1, regulatory subunit 16A, transc	NA	-0.06087	5.42755	-1.04309	0.33502138	0.647296
24067	Srp54a	signal recognition particle 54A, transcript variant X1	NA	-0.12796	6.250928	-1.09275	0.33502493	0.647296
27055	Fkbp9	FK506 binding protein 9	NA	-0.05603	5.897493	-1.0396	0.33509767	0.64736
20449	St8sia1	ST8 alpha-N-acetyl-neuraminide alpha-2,8-sialyltrans	NA	0.053662	6.440525	1.037896	0.3352455	0.647556
329421	Myo3b	myosin IIIB	NA	-0.29224	0.35083	-1.22454	0.3353078	0.647556
574402	Gpr17	G protein-coupled receptor 17	NA	-0.10159	3.539711	-1.07296	0.33531873	0.647556
68927	Ptcd2	pentatricopeptide repeat domain 2	NA	-0.06259	4.88851	-1.04434	0.33537186	0.647582
208638	Slc25a38	solute carrier family 25, member 38	NA	-0.08107	4.441221	-1.0578	0.3354232	0.647604
108167721	LOC108167721	uncharacterized LOC108167721, transcript variant X	NA	-0.29665	0.343517	-1.22829	0.33561915	0.647773
212114	Nhlrc3	NHL repeat containing 3	NA	0.199441	1.665644	1.148253	0.33562165	0.647773

107734	Mrpl30	mitochondrial ribosomal protein L30, transcript varian	NA	0.073555	5.59236	1.052307	0.33566544	0.647773
18053	Ngfr	nerve growth factor receptor (TNFR superfamily, mer	NA	-0.08606	4.459301	-1.06146	0.33570163	0.647773
108888	Atad3a	ATPase family, AAA domain containing 3A	NA	-0.05486	5.545421	-1.03876	0.33570973	0.647773
237761	Sowaha	sosondowah ankyrin repeat domain family member A	NA	-0.16198	1.995721	-1.11882	0.33574936	0.647773
76824	Mtfr1l	mitochondrial fission regulator 1-like, transcript variar	NA	0.056478	6.447186	1.039924	0.33584008	0.647808
14958	H1f0	H1.0 linker histone	NA	-0.05772	8.090738	-1.04082	0.33584729	0.647808
27361	Msrb1	methionine sulfoxide reductase B1, transcript variant	NA	0.101411	3.800171	1.072822	0.33593089	0.647892
233893	Zfp764	zinc finger protein 764, transcript variant 1	NA	-0.11293	2.939877	-1.08142	0.33608927	0.648121
15891	Ibsp	integrin binding sialoprotein	NA	0.42586	1.87833	1.343373	0.33620334	0.648264
54151	Cyhr1	cysteine and histidine rich 1, transcript variant X5	NA	-0.04492	6.751636	-1.03163	0.33636525	0.648439
12266	C3	complement component 3, transcript variant X1	NA	-0.21666	1.30487	-1.16204	0.33638697	0.648439
100415901	Gm13648	predicted gene 13648	NA	-0.57003	-0.87106	-1.48455	0.33641747	0.648439
223773	Zbed4	zinc finger, BED type containing 4	NA	-0.05814	5.805673	-1.04112	0.33648718	0.648439
77031	Slc9a8	solute carrier family 9 (sodium/hydrogen exchanger),	NA	-0.09992	4.876592	-1.07172	0.33649339	0.648439
233168	AI987944	expressed sequence AI987944, transcript variant 2	NA	0.111721	2.883896	1.080516	0.33660352	0.648575
17168	Nprl3	nitrogen permease regulator-like 3, transcript variant	NA	0.07926	4.30968	1.056476	0.3367144	0.648706
106639	Vmac	vimentin-type intermediate filament associated coiled	NA	-0.09029	4.24412	-1.06458	0.33676753	0.648706
11941	Atp2b2	ATPase, Ca++ transporting, plasma membrane 2, tra	NA	0.048466	6.57931	1.034165	0.33681914	0.648706
12520	Cd81	CD81 antigen	NA	0.058815	7.154102	1.04161	0.33683124	0.648706
17179	Matk	megakaryocyte-associated tyrosine kinase, transcript	NA	0.089021	4.323537	1.063648	0.33691446	0.64879
97820	4833439L19Rik	RIKEN cDNA 4833439L19 gene, transcript variant 5	NA	-0.04359	7.398002	-1.03067	0.33703268	0.648851
73710	Tubb2b	tubulin, beta 2B class IIB	NA	0.049125	12.15483	1.034637	0.33708124	0.648851
17685	Msh2	mutS homolog 2	NA	-0.05718	5.984103	-1.04043	0.3370919	0.648851
100126824	Sco2	SCO2 cytochrome c oxidase assembly protein	NA	0.15286	3.00578	1.111771	0.3371056	0.648851
23950	Dnajb6	DnaJ heat shock protein family (Hsp40) member B6,	NA	0.043163	7.890151	1.03037	0.33725211	0.649056
78287	Rbsn	rabenosyn, RAB effector, transcript variant X2	NA	0.057772	5.833015	1.040857	0.33739225	0.649177
102632163	Gm30310	predicted gene, 30310, transcript variant X1	NA	-0.2799	0.971525	-1.21411	0.3374094	0.649177
14693	Gnb2	guanine nucleotide binding protein (G protein), beta 2	NA	-0.04191	8.664364	-1.02948	0.33743501	0.649177
228356	1110051M20Rik	RIKEN cDNA 1110051M20 gene, transcript variant X	NA	-0.05416	6.152318	-1.03826	0.33748471	0.649186
68969	Eif1b	eukaryotic translation initiation factor 1B	NA	0.072128	6.924856	1.051266	0.33751933	0.649186
17423	Ndst2	N-deacetylase/N-sulfotransferase (heparan glucosam	NA	0.069163	4.580132	1.049108	0.3376613	0.64923
231637	Ssh1	slingshot protein phosphatase 1, transcript variant 2	NA	0.056059	5.730863	1.039622	0.33769467	0.64923
78177	Ninl	ninein-like, transcript variant X10	NA	-0.09379	3.891332	-1.06717	0.33770784	0.64923
68001	Cfap298	cilia and flagella associate protien 298	NA	0.079013	4.548768	1.056295	0.33771558	0.64923
233913	Rusf1	RUS family member 1, transcript variant 2	NA	-0.09003	4.239951	-1.0644	0.33778024	0.64923
320165	Tacc1	transforming, acidic coiled-coil containing protein 1, tr	NA	0.067412	5.658928	1.047835	0.33778141	0.64923
13176	Dcc	deleted in colorectal carcinoma	NA	0.059106	7.065639	1.04182	0.33790896	0.649398
105244831	Gm40367	predicted gene, 40367	NA	-0.51174	1.555034	-1.42577	0.33796309	0.649426
15273	Hivep2	human immunodeficiency virus type I enhancer bindi	NA	0.06557	6.576342	1.046498	0.33808831	0.64954
229949	Ak5	adenylate kinase 5, transcript variant 1	NA	0.080499	4.093391	1.057384	0.33810246	0.64954
115488666	Gm52261	predicted gene, 52261	NA	-0.35667	-0.36733	-1.28047	0.33817958	0.649558
67187	Zmynd19	zinc finger, MYND domain containing 19, transcript va	NA	0.061027	5.876704	1.043208	0.33821937	0.649558
30944	Zfp354c	zinc finger protein 354C	NA	0.050847	6.8914	1.035873	0.33825067	0.649558
118568309	LOC118568309	uncharacterized LOC118568309, transcript variant X	NA	0.172525	1.511143	1.127029	0.33827135	0.649558
94066	Mrpl36	mitochondrial ribosomal protein L36	NA	-0.11599	3.890441	-1.08372	0.33849828	0.649871
103135	Pan2	PAN2 poly(A) specific ribonuclease subunit, transcrip	NA	0.061438	5.727823	1.043505	0.33851435	0.649871
75051	Ccdc173	coiled-coil domain containing 173, transcript variant X	NA	-0.16392	1.891949	-1.12033	0.33868264	0.650117
380713	Scarf1	scavenger receptor class F, member 1	NA	0.174278	2.177879	1.128399	0.33880061	0.650174
245944	Vps54	VPS54 GARP complex subunit, transcript variant X4	NA	0.05913	5.485062	1.041837	0.33882172	0.650174
16322	Inha	inhibin alpha, transcript variant X1	NA	0.072694	5.304262	1.051679	0.33888595	0.650174
71743	Coasy	Coenzyme A synthase, transcript variant 1	NA	0.069132	4.608519	1.049085	0.33890986	0.650174
18824	Plp2	proteolipid protein 2	NA	-0.08135	4.081427	-1.058	0.33891202	0.650174
225579	Slc27a6	solute carrier family 27 (fatty acid transporter), memb	NA	-0.24113	0.588939	-1.18192	0.3390437	0.65035
218203	Mylip	myosin regulatory light chain interacting protein	NA	0.104564	3.51072	1.075169	0.33914595	0.650469
56857	Slc37a2	solute carrier family 37 (glycerol-3-phosphate transpo	NA	0.195766	1.103136	1.145332	0.33926202	0.650595
67378	Bbs2	Bardet-Biedl syndrome 2 (human), transcript variant X	NA	0.070578	4.652553	1.050137	0.33929117	0.650595
242570	Raver2	ribonucleoprotein, PTB-binding 2	NA	0.099641	3.744415	1.071507	0.33933708	0.650606
28146	Serp1	stress-associated endoplasmic reticulum protein 1	NA	0.058196	6.481665	1.041163	0.33946403	0.650773
16523	Kcnj8	potassium inwardly-rectifying channel, subfamily J, m	NA	-0.11423	3.498286	-1.0824	0.33952281	0.650809
211961	Asxl3	additional sex combs like 3, transcriptional regulator,	NA	0.056657	6.136034	1.040053	0.33958141	0.650844
54204	Septin1	septin 1	NA	-0.17069	1.797726	-1.12559	0.33969766	0.650924
329877	Dennd4c	DENN/MADD domain containing 4C	NA	0.077571	4.442183	1.05524	0.33973601	0.650924
329872	Frem1	Fras1 related extracellular matrix protein 1, transcript	NA	0.097167	3.306674	1.069671	0.33974522	0.650924
242553	Kank4	KN motif and ankyrin repeat domains 4	NA	-0.13446	2.418533	-1.09768	0.33980789	0.650924
21846	Tie1	tyrosine kinase with immunoglobulin-like and EGF-lik	NA	-0.07128	4.456004	-1.05065	0.33992094	0.650924
74012	Rap2b	RAP2B, member of RAS oncogene family	NA	0.058831	5.794706	1.041622	0.33994067	0.650924
74041	Ddias	DNA damage-induced apoptosis suppressor, transcri	NA	0.112183	2.88658	1.080862	0.33996114	0.650924
245866	Ift52	intraflagellar transport 52, transcript variant 2	NA	-0.06598	4.918237	-1.04679	0.33996626	0.650924

17311	Kitl	kit ligand, transcript variant 1	NA	0.066518	6.903751	1.047186	0.34002041	0.650924
18479	Pak1	p21 (RAC1) activated kinase 1, transcript variant 1	NA	0.047028	7.73076	1.033135	0.34002309	0.650924
99696	Ankrd50	ankyrin repeat domain 50, transcript variant X1	NA	-0.053	6.395142	-1.03742	0.3400818	0.650943
224807	Tmem63b	transmembrane protein 63b, transcript variant X3	NA	0.04521	7.311201	1.031833	0.34011311	0.650943
69080	Gmppa	GDP-mannose pyrophosphorylase A, transcript varia	NA	-0.06774	4.901023	-1.04807	0.34024169	0.651113
71240	Osbpl7	oxysterol binding protein-like 7	NA	0.08171	4.07614	1.058272	0.34028533	0.65112
23923	Aadat	aminoadipate aminotransferase	NA	0.260965	0.321018	1.19828	0.34033166	0.651123
12290	Cacna1e	calcium channel, voltage-dependent, R type, alpha 1I	NA	0.052973	7.331517	1.0374	0.34039552	0.651123
13087	Cyp2a5	cytochrome P450, family 2, subfamily a, polypeptide !	NA	0.408059	0.42889	1.326899	0.340407	0.651123
228491	Zfp770	zinc finger protein 770	NA	0.076834	4.881708	1.054701	0.34047736	0.651181
115490366	Gm52873	predicted gene, 52873	NA	-0.33638	-0.38953	-1.26259	0.3406114	0.651315
17252	Rdh11	retinol dehydrogenase 11, transcript variant 1	NA	0.062004	5.568683	1.043915	0.34062763	0.651315
64242	Ngb	neuroglobin, transcript variant 2	NA	-0.23952	0.777158	-1.1806	0.34068714	0.65134
69008	Cab39l	calcium binding protein 39-like, transcript variant 2	NA	0.08031	4.598442	1.057245	0.34077172	0.65134
193813	Mcfd2	multiple coagulation factor deficiency 2, transcript var	NA	-0.06562	4.936118	-1.04654	0.34079943	0.65134
622320	Kctd21	potassium channel tetramerisation domain containing	NA	-0.14864	3.120215	-1.10853	0.34080084	0.65134
266632	Irak4	interleukin-1 receptor-associated kinase 4	NA	-0.302	0.654962	-1.23285	0.34122618	0.652077
26972	Spo11	SPO11 meiotic protein covalently bound to DSB, tran	NA	-0.30244	-0.01802	-1.23322	0.34145106	0.652404
27376	Slc25a10	solute carrier family 25 (mitochondrial carrier, dicarbo	NA	-0.07954	3.916822	-1.05668	0.34147769	0.652404
73680	Zbtb8a	zinc finger and BTB domain containing 8a	NA	0.084441	3.985676	1.060277	0.34162753	0.652569
12972	Cryz	crystallin, zeta, transcript variant 5	NA	0.114718	3.351734	1.082764	0.34164435	0.652569
102635209	Gm32604	predicted gene, 32604, transcript variant X2	NA	0.277712	0.179214	1.212271	0.3416879	0.652576
12338	Capn6	calpain 6	NA	-0.1256	3.960258	-1.09096	0.34177011	0.652596
18417	Cldn11	claudin 11	NA	-0.09186	4.753479	-1.06574	0.3417969	0.652596
232566	Amn1	antagonist of mitotic exit network 1, transcript variant	NA	0.080297	4.718757	1.057235	0.34182356	0.652596
66549	Aggf1	angiogenic factor with G patch and FHA domains 1	NA	-0.05672	5.867195	-1.0401	0.34187789	0.652596
12368	Casp6	caspase 6	NA	-0.09778	3.601879	-1.07013	0.34195469	0.652596
66994	Cep19	centrosomal protein 19	NA	0.07576	4.814379	1.053916	0.34199724	0.652596
115490436	Gm52920	predicted gene, 52920, transcript variant X2	NA	0.391728	-0.65118	1.311964	0.34200525	0.652596
67759	Plgrkt	plasminogen receptor, C-terminal lysine transmembra	NA	0.076231	4.896872	1.05426	0.34204842	0.652596
114143	Atp6v0b	ATPase, H+ transporting, lysosomal V0 subunit B	NA	-0.0648	6.696791	-1.04594	0.34210226	0.652596
22318	Vamp2	vesicle-associated membrane protein 2	NA	0.045034	8.623676	1.031707	0.34212303	0.652596
76789	Mzt1	mitotic spindle organizing protein 1	NA	0.053569	6.728137	1.037829	0.34214086	0.652596
170772	Glcci1	glucocorticoid induced transcript 1, transcript variant :	NA	-0.06174	5.87265	-1.04373	0.34217987	0.652596
18475	Pafah1b2	platelet-activating factor acetylhydrolase, isoform 1b,	NA	0.049387	8.368546	1.034825	0.34237669	0.652895
104303	Arl1	ADP-ribosylation factor-like 1, transcript variant X1	NA	0.047626	6.887956	1.033562	0.34243131	0.652923
268566	Gphn	gephyrin, transcript variant 1	NA	0.053995	6.108277	1.038135	0.34249526	0.652968
102635630	Gm32918	predicted gene, 32918, transcript variant X1	NA	-0.36427	-0.35987	-1.28723	0.34260342	0.653022
79464	Lias	lipoic acid synthetase, transcript variant 1	NA	0.063932	5.080654	1.045311	0.34260809	0.653022
76566	Rflnb	refilin B	NA	0.097765	4.216823	1.070114	0.34266865	0.653022
103466	Nt5dc3	5'-nucleotidase domain containing 3, transcript varian	NA	-0.05765	6.694679	-1.04077	0.34268419	0.653022
209837	Slc38a5	solute carrier family 38, member 5	NA	-0.1041	4.008685	-1.07482	0.34275057	0.653037
24115	Best1	bestrophin 1	NA	0.270984	0.528534	1.206631	0.34279544	0.653037
18551	Pcsk4	proprotein convertase subtilisin/kexin type 4, transcrip	NA	0.164631	1.744482	1.120879	0.34286391	0.653037
665775	Bod1l	biorientation of chromosomes in cell division 1-like, tr	NA	0.054777	6.579342	1.038699	0.34286481	0.653037
67445	C1qtnf4	C1q and tumor necrosis factor related protein 4	NA	0.115367	5.735249	1.08325	0.34289264	0.653037
15413	Hoxb5	homeobox B5	NA	0.339676	3.577875	1.265472	0.34304877	0.653258
26440	Psma1	proteasome subunit alpha 1	NA	0.046255	6.942671	1.032581	0.34325574	0.653576
74117	Actr3	ARP3 actin-related protein 3, transcript variant 3	NA	0.042139	8.2949	1.029639	0.34338221	0.653692
20250	Scd2	stearoyl-Coenzyme A desaturase 2	NA	0.047931	10.00495	1.033781	0.34341663	0.653692
554327	2610042L04Rik	RIKEN cDNA 2610042L04 gene	NA	0.110868	3.42644	1.079878	0.34343729	0.653692
67075	Magt1	magnesium transporter 1, transcript variant 2	NA	-0.06782	4.83681	-1.04813	0.34352686	0.653693
14548	Mrps33	mitochondrial ribosomal protein S33, transcript variar	NA	-0.06928	5.480222	-1.0492	0.34353294	0.653693
57275	Lenep	lens epithelial protein	NA	0.140086	2.038009	1.101971	0.34357445	0.653693
11416	Slc33a1	solute carrier family 33 (acetyl-CoA transporter), men	NA	-0.08053	4.57808	-1.05741	0.34359848	0.653693
102638289	Gm34889	predicted gene, 34889, transcript variant X1	NA	0.289193	-0.13855	1.221956	0.34368823	0.653718
69076	Triap1	TP53 regulated inhibitor of apoptosis 1	NA	-0.1121	4.106269	-1.0808	0.34372033	0.653718
74030	Rin2	Ras and Rab interactor 2, transcript variant X2	NA	0.075273	4.39666	1.05356	0.343758	0.653718
17153	Mal	myelin and lymphocyte protein, T cell differentiation p	NA	0.261147	2.011537	1.198431	0.3438863	0.653718
13132	Dab2	disabled 2, mitogen-responsive phosphoprotein, trans	NA	-0.07565	5.29136	-1.05383	0.34389495	0.653718
23969	Pacsin1	protein kinase C and casein kinase substrate in neuro	NA	-0.06455	5.52701	-1.04576	0.34390754	0.653718
78757	Rictor	RPTOR independent companion of MTOR, complex :	NA	0.065756	5.634191	1.046633	0.34392873	0.653718
54633	Pqbp1	polyglutamine binding protein 1, transcript variant 1	NA	0.048303	6.306112	1.034048	0.34393308	0.653718
245867	Pcmtd2	protein-L-isoaspartate (D-aspartate) O-methyltransfe	NA	-0.0508	6.219192	-1.03584	0.3440378	0.653841
108167440	AU020206	expressed sequence AU020206	NA	-0.12414	2.783617	-1.08986	0.34416316	0.653931
21341	Taf1c	TATA-box binding protein associated factor, RNA pol	NA	-0.07705	4.256399	-1.05486	0.3441689	0.653931
72614	Pih1d2	PIH1 domain containing 2	NA	0.251806	0.54149	1.190696	0.34420596	0.653931
212986	Scfd2	Sec1 family domain containing 2, transcript variant a	NA	-0.08586	3.944126	-1.06132	0.34427972	0.653995

237782	Smcr8	Smith-Magenis syndrome chromosome region, candi	NA	0.071787	5.051581	1.051017	0.34438602	0.654056
118567825	LOC118567825	uncharacterized LOC118567825	NA	-0.13091	2.690985	-1.09498	0.34439214	0.654056
78833	Gins3	GINS complex subunit 3 (Psf3 homolog)	NA	-0.14492	2.687954	-1.10567	0.34444221	0.654074
102639243	Gm35599	predicted gene, 35599, transcript variant X2	NA	-0.33058	-0.02873	-1.25752	0.34449113	0.654091
76293	Mfap4	microfibrillar-associated protein 4, transcript variant 3	NA	-0.07528	5.053997	-1.05356	0.34455923	0.654144
105243672	Gm39503	predicted gene, 39503	NA	0.290309	0.474736	1.222902	0.34471534	0.654351
73086	Rps6ka5	ribosomal protein S6 kinase, polypeptide 5, transcript	NA	0.068459	5.033692	1.048596	0.34474866	0.654351
50722	Dkkl1	dickkopf-like 1	NA	0.223341	1.062751	1.167434	0.34497151	0.65463
67511	Tmed9	transmembrane p24 trafficking protein 9	NA	0.055088	6.242456	1.038922	0.34497594	0.65463
67553	Gstcd	glutathione S-transferase, C-terminal domain contain	NA	0.077062	4.161468	1.054868	0.34512831	0.654782
100855	Tbc1d14	TBC1 domain family, member 14, transcript variant X	NA	0.049048	7.10751	1.034582	0.34513691	0.654782
76469	Cmya5	cardiomyopathy associated 5, transcript variant X1	NA	-0.22825	0.945975	-1.17141	0.34523551	0.654889
16565	Kif21b	kinesin family member 21B, transcript variant 1	NA	-0.05303	8.98719	-1.03744	0.34527348	0.654889
13864	Nr2f6	nuclear receptor subfamily 2, group F, member 6	NA	-0.098	4.405343	-1.07029	0.34557558	0.655279
20289	Scx	scleraxis	NA	0.282066	0.77113	1.215935	0.34564874	0.655279
18214	Ddr2	discoidin domain receptor family, member 2, transcrip	NA	0.068917	4.405183	1.048929	0.34566344	0.655279
64934	Pes1	pescadillo ribosomal biogenesis factor 1	NA	0.056847	6.049297	1.04019	0.34568419	0.655279
68695	Hddc3	HD domain containing 3, transcript variant X4	NA	-0.10909	4.082484	-1.07855	0.34569887	0.655279
269966	Nup98	nucleoporin 98, transcript variant X5	NA	-0.05382	5.740834	-1.03801	0.34573117	0.655279
242585	Slc35d1	solute carrier family 35 (UDP-glucuronic acid/UDP-N-	NA	0.093774	4.030152	1.067159	0.34577035	0.655279
229707	Strip1	striatin interacting protein 1	NA	-0.04832	7.422785	-1.03406	0.34580141	0.655279
73825	Ppp1r21	protein phosphatase 1, regulatory subunit 21	NA	0.056976	6.005657	1.040283	0.34609	0.655749
71148	Mier1	MEIR1 treanscription regulator, transcript variant 5	NA	0.052169	5.849446	1.036823	0.34614144	0.655771
67809	Rmdn3	regulator of microtubule dynamics 3	NA	-0.06627	5.045165	-1.047	0.34621524	0.655834
215008	Vezt	vezatin, adherens junctions transmembrane protein, t	NA	-0.05328	6.872972	-1.03762	0.34627043	0.655862
73032	Ttc9b	tetratricopeptide repeat domain 9B	NA	-0.064	5.088722	-1.04536	0.34631298	0.655866
118567348	LOC118567348	uncharacterized LOC118567348	NA	-0.17261	5.090685	-1.1271	0.34659274	0.656261
75426	Igfbpl1	insulin-like growth factor binding protein-like 1	NA	-0.05474	8.111645	-1.03867	0.34660209	0.656261
66618	Snrnp27	small nuclear ribonucleoprotein 27 (U4/U6.U5)	NA	0.064701	5.009614	1.045868	0.34683929	0.656634
14536	Nr6a1	nuclear receptor subfamily 6, group A, member 1, tra	NA	0.128529	2.898392	1.093178	0.34695923	0.656765
19211	Pten	phosphatase and tensin homolog	NA	0.05339	7.792697	1.037701	0.34698913	0.656765
70510	Rnf167	ring finger protein 167, transcript variant 1	NA	0.055557	5.849089	1.03926	0.34732718	0.657328
56190	Rbm38	RNA binding motif protein 38	NA	-0.10376	3.681597	-1.07457	0.34753612	0.657636
52174	Tmem222	transmembrane protein 222, transcript variant 1	NA	0.06163	5.452089	1.043644	0.34757087	0.657636
382056	Crtc1	CREB regulated transcription coactivator 1, transcript	NA	-0.04984	6.8955	-1.03515	0.34777079	0.657938
268469	Zfp652	zinc finger protein 652	NA	-0.12207	3.925573	-1.0883	0.347996	0.658058
104884	Tdp1	tyrosyl-DNA phosphodiesterase 1, transcript variant X	NA	-0.06424	5.263986	-1.04553	0.34806525	0.658058
17245	Mdm1	transformed mouse 3T3 cell double minute 1, transcr	NA	-0.06467	4.952462	-1.04585	0.34807308	0.658058
102633796	Gm31536	predicted gene, 31536, transcript variant X2	NA	-0.29983	0.042656	-1.231	0.34809222	0.658058
13349	Ackr1	atypical chemokine receptor 1 (Duffy blood group)	NA	0.158914	4.115424	1.116446	0.34811033	0.658058
100038709	Gm10789	predicted gene 10789	NA	-0.43166	-0.45082	-1.34879	0.34812544	0.658058
21885	Tle1	transducin-like enhancer of split 1, transcript variant X	NA	-0.05165	6.435416	-1.03645	0.34813158	0.658058
11992	Auh	AU RNA binding protein/enoyl-coenzyme A hydratase	NA	0.070505	4.703508	1.050084	0.34817934	0.658058
100042166	Gm3704	predicted gene 3704	NA	0.200563	0.994511	1.149147	0.3482019	0.658058
235497	Leo1	Leo1, Paf1/RNA polymerase II complex component	NA	0.055689	5.440231	1.039355	0.34824285	0.658058
192156	Mvd	mevalonate (diphospho) decarboxylase, transcript va	NA	0.091546	5.167227	1.065511	0.34830778	0.658058
19296	Pvt1	Pvt1 oncogene, transcript variant 2	NA	-0.15664	1.928837	-1.11468	0.34831965	0.658058
12234	Btrc	beta-transducin repeat containing protein, transcript v	NA	-0.04694	6.274641	-1.03307	0.34860557	0.658522
382083	Snx22	sorting nexin 22, transcript variant 2	NA	0.150345	2.830636	1.109835	0.34868279	0.658585
22122	Gfus	GDP-L-fucose synthase, transcript variant 2	NA	0.062886	4.923846	1.044553	0.34876031	0.658585
233410	Zfp592	zinc finger protein 592, transcript variant X1	NA	-0.07058	5.587502	-1.05014	0.34878926	0.658585
81845	Gpank1	G patch domain and ankyrin repeats 1, transcript vari	NA	-0.11262	3.640398	-1.08119	0.34880132	0.658585
231866	Zfp12	zinc finger protein 12, transcript variant X3	NA	-0.06175	5.445482	-1.04373	0.34884109	0.658585
110948	Hlcs	holocarboxylase synthetase (biotin- [propriony-Coenz	NA	0.100531	3.504099	1.072168	0.34888821	0.658597
80795	Selenok	selenoprotein K	NA	0.049021	6.530529	1.034562	0.34898463	0.658703
67139	Mis12	MIS12 kinetochore complex component, transcript va	NA	-0.06796	5.221042	-1.04823	0.34920062	0.659034
76000	5033430I15Rik	RIKEN cDNA 5033430I15 gene	NA	0.214663	1.241927	1.160432	0.34925372	0.659058
233571	P2ry6	pyrimidinergic receptor P2Y, G-protein coupled, 6	NA	0.300509	0.489877	1.231579	0.3493159	0.659099
118567748	LOC118567748	uncharacterized LOC118567748	NA	-0.18935	1.759633	-1.14025	0.34964399	0.65957
76582	Ipo11	importin 11, transcript variant 2	NA	0.088406	4.730934	1.063195	0.34967645	0.65957
20312	Cx3cl1	chemokine (C-X3-C motif) ligand 1	NA	0.058381	5.306186	1.041296	0.34968709	0.65957
235040	Atg4d	autophagy related 4D, cysteine peptidase, transcript v	NA	0.072981	4.355535	1.051888	0.34978052	0.65967
212996	Galnt17	polypeptide N-acetylgalactosaminyltransferase 17	NA	0.054545	5.631311	1.038532	0.34995361	0.659767
218441	Zfyve16	zinc finger, FYVE domain containing 16	NA	0.081764	4.576691	1.058311	0.34995849	0.659767
242669	Azin2	antizyme inhibitor 2, transcript variant X3	NA	0.14506	2.875552	1.105777	0.35004973	0.659767
246709	Rgs13	regulator of G-protein signaling 13	NA	0.320184	-0.00111	1.24849	0.35008136	0.659767
28200	Dhrs4	dehydrogenase/reductase (SDR family) member 4, tr	NA	-0.08402	3.771052	-1.05997	0.35011855	0.659767
320554	Tcp11l1	t-complex 11 like 1, transcript variant X1	NA	-0.05778	6.087619	-1.04086	0.35012554	0.659767

50868	Keap1	kelch-like ECH-associated protein 1, transcript varian	NA	-0.06639	5.796699	-1.04709	0.35014358	0.659767
67939	Prorsd1	prolyl-tRNA synthetase domain containing 1, transcrip	NA	-0.16766	1.644596	-1.12323	0.35015653	0.659767
238692	Zfp874a	zinc finger protein 874a	NA	-0.10341	3.194133	-1.07431	0.35028555	0.659934
115488649	Gm36513	predicted gene, 36513	NA	-0.29151	0.014195	-1.22392	0.35039827	0.659991
66511	Chtop	chromatin target of PRMT1, transcript variant 5	NA	-0.04193	7.917442	-1.02949	0.35040363	0.659991
76707	Clasp1	CLIP associating protein 1, transcript variant 5	NA	-0.05195	7.718207	-1.03667	0.35043781	0.659991
320714	Trappc11	trafficking protein particle complex 11	NA	-0.0501	5.88563	-1.03534	0.35053431	0.660022
19106	Eif2ak2	eukaryotic translation initiation factor 2-alpha kinase 2	NA	0.191131	2.725269	1.141658	0.35053514	0.660022
76880	6430411K18Rik	RIKEN cDNA 6430411K18 gen	NA	0.148449	2.82222	1.108377	0.3506188	0.660103
243274	Tmem132d	transmembrane protein 132D	NA	-0.13209	2.398295	-1.09588	0.35083046	0.660336
78774	Cfap61	cilia and flagella associated protein 61, transcript vari	NA	-0.2134	0.931775	-1.15942	0.35086178	0.660336
68760	Synpo2l	synaptopodin 2-like, transcript variant X2	NA	-0.3373	0.77596	-1.26339	0.35086428	0.660336
217337	Srp68	signal recognition particle 68	NA	-0.05579	6.167643	-1.03943	0.3510006	0.660516
216350	Tspan8	tetraspanin 8, transcript variant X1	NA	-0.35973	-0.61607	-1.28319	0.35104493	0.660523
77853	Msl2	MSL complex subunit 2, transcript variant 1	NA	0.05763	6.206093	1.040755	0.35119223	0.660724
66530	Ubxn6	UBX domain protein 6, transcript variant 3	NA	0.060342	5.954331	1.042713	0.35124075	0.660739
74597	4833418N02Rik	RIKEN cDNA 4833418N02 gene	NA	-0.25486	0.261464	-1.19322	0.35131783	0.660807
12023	Barx2	BarH-like homeobox 2	NA	-0.24476	0.62451	-1.1849	0.35137875	0.660845
278507	Wfikkn2	WAP, follistatin/kazal, immunoglobulin, kunitz and ne	NA	0.104106	3.727207	1.074828	0.35154936	0.660997
223828	Pphln1	periphilin 1, transcript variant X12	NA	0.055774	5.841519	1.039416	0.35156692	0.660997
381738	Drc1	dynein regulatory complex subunit 1	NA	-0.13261	2.776851	-1.09627	0.35158129	0.660997
22154	Tubb5	tubulin, beta 5 class I	NA	0.046117	11.64404	1.032482	0.35163062	0.661014
237859	Nsrp1	nuclear speckle regulatory protein 1	NA	0.071527	4.824176	1.050828	0.3517098	0.661086
232784	Zfp212	Zinc finger protein 212, transcript variant 1	NA	-0.07368	4.767816	-1.0524	0.35194651	0.661455
66881	Pcyox1	prenylcysteine oxidase 1, transcript variant 1	NA	0.049465	6.832906	1.034881	0.35207481	0.661601
102632124	Gm26901	predicted gene, 26901, transcript variant X4	NA	-0.33565	-0.03303	-1.26195	0.35210601	0.661601
73251	Setd7	SET domain containing (lysine methyltransferase) 7,	NA	0.053384	5.769887	1.037696	0.3521683	0.661635
105243688	Gm39518	predicted gene, 39518	NA	-0.22996	1.717574	-1.1728	0.35220506	0.661635
269275	Acvr1c	activin A receptor, type IC, transcript variant X1	NA	0.17791	1.38836	1.131244	0.35239512	0.661843
209815	Tbc1d25	TBC1 domain family, member 25, transcript variant 2	NA	-0.08392	4.233902	-1.05989	0.35239731	0.661843
105242738	Gm26799	predicted gene, 26799, transcript variant X1	NA	0.693443	-0.13998	1.617139	0.35247558	0.661847
75747	Sesn3	sestrin 3	NA	0.148267	3.277987	1.108237	0.35249838	0.661847
70603	Mutyh	mutY DNA glycosylase, transcript variant X7	NA	-0.1334	2.148022	-1.09688	0.35254439	0.661847
21847	Klf10	Kruppel-like factor 10, transcript variant 1	NA	0.081679	4.195625	1.058249	0.35256862	0.661847
70209	Tmem143	transmembrane protein 143	NA	-0.10032	3.33705	-1.07201	0.35260278	0.661847
108011	Ap4e1	adaptor-related protein complex AP-4, epsilon 1, tran	NA	0.061367	4.922542	1.043454	0.35268917	0.6619
100036768	Gm11696	predicted gene 11696, transcript variant 1	NA	0.177647	1.534724	1.131038	0.35271236	0.6619
67009	Ttc23	tetratricopeptide repeat domain 23, transcript variant	NA	-0.10548	3.166555	-1.07585	0.35285357	0.662089
229603	Otud7b	OTU domain containing 7B, transcript variant X5	NA	0.062292	4.958391	1.044124	0.35304048	0.662363
27028	Ermap	erythroblast membrane-associated protein, transcript	NA	-0.24302	0.31274	-1.18346	0.35323599	0.662552
118567442	LOC118567442	uncharacterized LOC118567442	NA	-0.22583	0.762061	-1.16945	0.35337611	0.662552
269614	Pank4	pantothenate kinase 4, transcript variant 3	NA	-0.07513	5.121112	-1.05345	0.35342719	0.662552
16891	Lipg	lipase, endothelial	NA	-0.10858	3.286216	-1.07816	0.35344339	0.662552
105242657	Hoxaas2	Hoxa cluster antisense RNA 2	NA	-0.35661	-0.7453	-1.28041	0.35345323	0.662552
26423	Nr5a1	nuclear receptor subfamily 5, group A, member 1, tra	NA	-0.27408	0.014062	-1.20922	0.3534566	0.662552
53424	Tsnax	translin-associated factor X	NA	0.043169	7.236058	1.030374	0.35346112	0.662552
215303	Camk1g	calcium/calmodulin-dependent protein kinase I gamm	NA	0.06693	5.177828	1.047485	0.35346709	0.662552
26425	Nubp1	nucleotide binding protein 1	NA	-0.0757	4.413229	-1.05387	0.35355048	0.662632
75763	Dcaf17	DDB1 and CUL4 associated factor 17, transcript varia	NA	-0.09291	4.80415	-1.06652	0.35367705	0.662793
243853	Fkrp	fukutin related protein, transcript variant 1	NA	-0.05903	5.120668	-1.04176	0.35373488	0.662825
110616	Atxn3	ataxin 3, transcript variant X4	NA	0.063082	4.825135	1.044695	0.35389984	0.662977
27993	Imp4	IMP4, U3 small nucleolar ribonucleoprotein	NA	-0.09082	4.312707	-1.06498	0.3539011	0.662977
15979	Ifngr1	interferon gamma receptor 1	NA	-0.08564	3.76298	-1.06116	0.35393815	0.662977
76954	Denn2b	DENN domain containing 2B, transcript variant 2	NA	-0.07232	4.458344	-1.05141	0.35402569	0.663053
77626	Smpd4	sphingomyelin phosphodiesterase 4, transcript variar	NA	-0.04997	5.862227	-1.03524	0.35405998	0.663053
69590	Gpx8	glutathione peroxidase 8 (putative)	NA	-0.08211	4.353487	-1.05857	0.35433486	0.66341
75871	Zfp821	zinc finger protein 821, transcript variant X3	NA	-0.04684	6.680342	-1.033	0.35445916	0.66341
209086	Samd9l	sterile alpha motif domain containing 9-like, transcript	NA	0.146252	1.959674	1.106691	0.35455922	0.66341
102638253	Gm34861	predicted gene, 34861, transcript variant X5	NA	0.207537	1.554664	1.154715	0.35457025	0.66341
20931	Surf2	surfeit gene 2	NA	0.071078	4.708634	1.050501	0.35459812	0.66341
67968	Ooep	oocyte expressed protein	NA	0.141649	2.111435	1.103165	0.35460294	0.66341
56325	Abcb9	ATP-binding cassette, sub-family B (MDR/TAP), men	NA	-0.08673	3.579587	-1.06196	0.35460675	0.66341
102637406	Gm34223	predicted gene, 34223, transcript variant X1	NA	-0.17862	1.552796	-1.1318	0.35461224	0.66341
54526	Syt10	synaptotagmin X, transcript variant X4	NA	0.256601	1.901588	1.194661	0.35462793	0.66341
93843	Pnck	pregnancy upregulated non-ubiquitously expressed C	NA	0.050845	6.01797	1.035871	0.35465878	0.66341
19218	Ptger3	prostaglandin E receptor 3 (subtype EP3), transcript v	NA	0.237582	0.579916	1.179015	0.35479008	0.663558
21930	Tnfaip6	tumor necrosis factor alpha induced protein 6	NA	-0.17329	1.59521	-1.12763	0.35481896	0.663558
75869	Arl5b	ADP-ribosylation factor-like 5B	NA	0.058552	6.005511	1.04142	0.35491229	0.663656

407785	Ndufs6	NADH:ubiquinone oxidoreductase core subunit S6, tr	NA	-0.09789	5.255983	-1.0702	0.35512827	0.663983
102637422	Gm34232	predicted gene, 34232	NA	-0.28654	2.540584	-1.21971	0.35526596	0.664153
56708	Clcf1	cardiotrophin-like cytokine factor 1, transcript variant	NA	0.264133	0.353635	1.200914	0.35530083	0.664153
22051	Trip6	thyroid hormone receptor interactor 6	NA	-0.08363	4.158569	-1.05968	0.3554217	0.664207
107372	C030016D13Rik	RIKEN cDNA C030016D13 gene	NA	0.347396	-0.39022	1.272262	0.35547259	0.664207
73635	Ptges3l	prostaglandin E synthase 3 like, transcript variant X2	NA	0.280362	0.556882	1.2145	0.3554949	0.664207
245670	Rragb	Ras-related GTP binding B	NA	0.05502	5.772672	1.038873	0.35549675	0.664207
70771	Gpr173	G-protein coupled receptor 173, transcript variant X1	NA	-0.07538	5.657645	-1.05364	0.35560919	0.664207
14369	Fzd7	frizzled class receptor 7	NA	-0.08109	4.450824	-1.05781	0.35561699	0.664207
18000	Septin2	septin 2, transcript variant X2	NA	0.045305	7.269222	1.031902	0.35562713	0.664207
71889	Epn3	epsin 3, transcript variant X1	NA	-0.24731	0.489942	-1.18699	0.35566968	0.664207
53418	B4galt2	UDP-Gal:betaGlcNAc beta 1,4- galactosyltransferase	NA	0.049547	6.782329	1.03494	0.35574823	0.664207
12311	Calcr	calcitonin receptor, transcript variant 1a	NA	0.138541	3.066226	1.100791	0.35577779	0.664207
67922	Fam32a	family with sequence similarity 32, member A	NA	0.046436	7.296606	1.03271	0.35577859	0.664207
20539	Slc7a5	solute carrier family 7 (cationic amino acid transporte	NA	0.048235	7.14246	1.033999	0.35585831	0.664251
68276	Toe1	target of EGR1, member 1 (nuclear)	NA	0.071745	4.384537	1.050987	0.35588353	0.664251
212167	Gsap	gamma-secretase activating protein, transcript varian	NA	-0.08714	3.684987	-1.06227	0.35593082	0.664263
100503099	Gm19554	predicted gene, 19554, transcript variant 2	NA	-0.13826	2.30076	-1.10058	0.35604577	0.664353
50723	Icosl	icos ligand	NA	-0.23659	0.504759	-1.1782	0.35608115	0.664353
67278	Pagr1a	PAXIP1 associated glutamate rich protein 1A	NA	0.063768	5.399346	1.045192	0.35610173	0.664353
75604	Tm4sf5	transmembrane 4 superfamily member 5	NA	-0.32854	-0.32103	-1.25575	0.356147	0.664361
231549	Lrrc8d	leucine rich repeat containing 8D, transcript variant X	NA	0.049731	6.102974	1.035072	0.35639844	0.664754
70551	Tmtc4	transmembrane and tetratricopeptide repeat containir	NA	0.067417	5.39641	1.047839	0.3565327	0.664814
231214	Cc2d2a	coiled-coil and C2 domain containing 2A, transcript va	NA	0.093284	4.054941	1.066796	0.35653821	0.664814
106794	Dhx57	DEAH (Asp-Glu-Ala-Asp/His) box polypeptide 57, trai	NA	-0.05104	6.899419	-1.03601	0.35655311	0.664814
69277	3300002I08Rik	RIKEN cDNA 3300002I08 gene, transcript variant X1	NA	-0.08262	5.181729	-1.05894	0.35682781	0.66525
76123	Gpsm2	G-protein signalling modulator 2 (AGS3-like, C. elega	NA	-0.0702	4.664252	-1.04987	0.35687033	0.665253
74585	Sppl3	signal peptide peptidase 3	NA	0.04464	7.022719	1.031425	0.35694422	0.665315
432769	Zfp708	zinc finger protein 708, transcript variant 2	NA	0.092841	3.650522	1.066468	0.35701005	0.665361
108167879	Gm46266	predicted gene, 46266	NA	0.26929	0.125622	1.205215	0.35713853	0.665521
194237	Rimkla	ribosomal modification protein rimK-like family memb	NA	0.054891	5.462509	1.038781	0.3572406	0.665521
20664	Sox1	SRY (sex determining region Y)-box 1	NA	-0.07093	6.303877	-1.05039	0.35726411	0.665521
76467	Msrb2	methionine sulfoxide reductase B2	NA	-0.17959	1.72971	-1.13256	0.35730655	0.665521
268390	Ahsa2	AHA1, activator of heat shock protein ATPase 2, tran	NA	-0.04563	6.995856	-1.03213	0.35732236	0.665521
14070	F8a	factor 8-associated gene A	NA	0.09206	3.518794	1.065891	0.35743808	0.665521
18139	Zfp638	zinc finger protein 638, transcript variant X27	NA	0.043597	7.085334	1.03068	0.35743809	0.665521
116870	Mta1	metastasis associated 1, transcript variant 5	NA	-0.04338	7.291094	-1.03053	0.35745357	0.665521
66175	Mustn1	musculoskeletal, embryonic nuclear protein 1	NA	-0.24291	0.385976	-1.18338	0.35746416	0.665521
100504178	Dhrs13os	dehydrogenase/reductase (SDR family) member 13,	NA	-0.21114	0.803106	-1.1576	0.357632	0.665758
18992	Pou3f2	POU domain, class 3, transcription factor 2	NA	0.047864	7.28469	1.033733	0.35781938	0.66603
115489417	LOC115489417	uncharacterized LOC115489417, transcript variant X	NA	0.123802	2.757528	1.089603	0.35791452	0.666127
70380	Mospd1	motile sperm domain containing 1, transcript variant 2	NA	0.06048	5.008956	1.042813	0.35795312	0.666127
71910	Plpp5	phospholipid phosphatase 5, transcript variant 3	NA	0.068716	4.53898	1.048783	0.35802349	0.66618
212919	Kctd7	potassium channel tetramerisation domain containing	NA	-0.07371	4.418073	-1.05242	0.35807056	0.66618
116838	Rims2	regulating synaptic membrane exocytosis 2, transcrip	NA	0.054414	6.040201	1.038438	0.35815908	0.66618
209195	Clic6	chloride intracellular channel 6	NA	-0.08578	4.45364	-1.06126	0.35821525	0.66618
101602	AI467606	expressed sequence AI467606	NA	0.279442	0.493791	1.213725	0.35824101	0.66618
21354	Tap1	transporter 1, ATP-binding cassette, sub-family B (MI	NA	0.188145	1.292335	1.139298	0.35824216	0.66618
69399	1700025G04Rik	RIKEN cDNA 1700025G04 gene	NA	0.067934	6.415983	1.048215	0.35828652	0.66618
232536	Mrps35	mitochondrial ribosomal protein S35	NA	0.073098	4.326681	1.051973	0.35832175	0.66618
276829	Smtnl2	smoothelin-like 2	NA	0.094017	3.295751	1.067338	0.35835031	0.66618
108077	Skiv2l	superkiller viralicidic activity 2-like (S. cerevisiae)	NA	-0.05043	5.985671	-1.03557	0.35841982	0.666233
107951	Cdk9	cyclin-dependent kinase 9 (CDC2-related kinase)	NA	0.04841	6.411688	1.034125	0.358487	0.666282
230376	Haus6	HAUS augmin-like complex, subunit 6	NA	0.070404	4.457273	1.050011	0.35874011	0.666676
66164	Nip7	NIP7, nucleolar pre-rRNA processing protein, transcr	NA	0.054736	5.360546	1.038669	0.35881612	0.666742
71833	Dcaf7	DDB1 and CUL4 associated factor 7	NA	0.040769	8.372015	1.028662	0.35895408	0.666922
102633898	Gm31613	predicted gene, 31613, transcript variant X2	NA	0.315458	0.001786	1.244407	0.35899614	0.666922
233490	Crebzf	CREB/ATF bZIP transcription factor, transcript varian	NA	-0.06619	6.476779	-1.04695	0.35903594	0.666922
67276	Eri1	exoribonuclease 1, transcript variant X1	NA	-0.05627	5.258283	-1.03978	0.35910623	0.666976
67683	Pbdc1	polysaccharide biosynthesis domain containing 1, tra	NA	-0.07641	4.473741	-1.05439	0.35919236	0.66706
246277	Csad	cysteine sulfinic acid decarboxylase, transcript varian	NA	-0.07268	4.241848	-1.05167	0.35924759	0.667086
72432	Spink5	serine peptidase inhibitor, Kazal type 5	NA	-0.76414	-1.41272	-1.69835	0.35937864	0.667196
52055	Rab11fip5	RAB11 family interacting protein 5 (class I), transcript	NA	-0.05492	5.80556	-1.0388	0.35938869	0.667196
245688	Rbbp7	retinoblastoma binding protein 7, chromatin remodeli	NA	0.042971	7.252096	1.030234	0.35953113	0.667384
76261	0610040J01Rik	RIKEN cDNA 0610040J01 gene	NA	0.201714	1.385507	1.150064	0.35963831	0.667507
20187	Ryk	receptor-like tyrosine kinase, transcript variant 1	NA	0.04868	6.200528	1.034318	0.35997745	0.66806
100206	Adprhl2	ADP-ribosylhydrolase like 2	NA	0.081933	4.46458	1.058436	0.36002884	0.66808
69556	Bod1	biorientation of chromosomes in cell division 1	NA	0.053143	6.431817	1.037523	0.3602221	0.668362

102638432	Gm35002	predicted gene, 35002	NA	0.096837	3.978646	1.069426	0.36039748	0.668611
19384	Ran	RAN, member RAS oncogene family	NA	-0.04251	8.331589	-1.0299	0.36060899	0.668927
320717	Pptc7	PTC7 protein phosphatase homolog	NA	0.085087	4.529844	1.060752	0.36073265	0.669046
16871	Lhx3	LIM homeobox protein 3, transcript variant X1	NA	-0.35894	-0.40204	-1.28248	0.36075933	0.669046
21934	Tnfrsf11a	tumor necrosis factor receptor superfamily, member '	NA	0.19538	1.562448	1.145026	0.36079639	0.669046
18163	Ctnnd2	catenin (cadherin associated protein), delta 2, transcr	NA	0.046099	7.774409	1.03247	0.3608563	0.669081
78651	Lsm6	LSM6 homolog, U6 small nuclear RNA and mRNA de	NA	-0.05524	5.873371	-1.03904	0.36097382	0.669223
219249	Tdrd3	tudor domain containing 3, transcript variant 1	NA	-0.05199	6.228973	-1.03669	0.36101576	0.669224
54638	Ccdc22	coiled-coil domain containing 22	NA	-0.05952	4.916022	-1.04212	0.36109522	0.669261
240396	Mex3c	mex3 RNA binding family member C	NA	-0.05447	6.509024	-1.03848	0.36111786	0.669261
100340	Smpdl3b	sphingomyelin phosphodiesterase, acid-like 3B	NA	0.336123	-0.30252	1.26236	0.36126301	0.66938
100041576	Gm3414	predicted gene 3414	NA	-0.43613	-1.11993	-1.35297	0.36127363	0.66938
67525	Trim62	tripartite motif-containing 62, transcript variant 3	NA	0.057118	5.548485	1.040385	0.36138707	0.66938
105244755	LOC105244755	uncharacterized LOC105244755, transcript variant X'	NA	-0.3114	-0.05873	-1.24091	0.36140443	0.66938
11800	Api5	apoptosis inhibitor 5, transcript variant 2	NA	0.048432	6.942943	1.03414	0.36148436	0.66938
244219	Zfp668	zinc finger protein 668, transcript variant X6	NA	0.053759	5.618951	1.037966	0.36154055	0.66938
109934	Abr	active BCR-related gene, transcript variant 4	NA	0.041247	8.248389	1.029003	0.36154354	0.66938
212670	Catsper2	cation channel, sperm associated 2, transcript varian	NA	0.134016	2.445008	1.097344	0.36158998	0.66938
11947	Atp5b	ATP synthase, H+ transporting mitochondrial F1 com	NA	-0.04164	9.79951	-1.02928	0.36169839	0.66938
15275	Hk1	hexokinase 1, transcript variant X5	NA	-0.04293	7.771437	-1.0302	0.36171673	0.66938
11761	Aox1	aldehyde oxidase 1	NA	0.295544	-0.15295	1.227348	0.36171906	0.66938
15516	Hsp90ab1	heat shock protein 90 alpha (cytosolic), class B meml	NA	-0.04348	10.86764	-1.03059	0.36173323	0.66938
224139	Golgb1	golgi autoantigen, golgin subfamily b, macrogolgin 1	NA	0.057239	6.262619	1.040473	0.36173913	0.66938
68778	Gucd1	guanylyl cyclase domain containing 1, transcript varia	NA	0.075998	4.699963	1.05409	0.36178997	0.66938
59003	Maea	macrophage erythroblast attacher	NA	0.051704	6.625674	1.036489	0.36179908	0.66938
225724	Mapk4	mitogen-activated protein kinase 4, transcript variant	NA	0.066306	4.881004	1.047032	0.36185497	0.669407
16886	Limk2	LIM motif-containing protein kinase 2, transcript varia	NA	-0.04475	7.289615	-1.03151	0.36191813	0.669448
13497	Drp2	dystrophin related protein 2, transcript variant X9	NA	-0.06203	6.380553	-1.04393	0.36196989	0.669467
57357	Srd5a3	steroid 5 alpha-reductase 3	NA	0.09706	3.156698	1.069591	0.36215245	0.669597
12840	Col9a2	collagen, type IX, alpha 2	NA	0.11232	5.364207	1.080965	0.36215295	0.669597
226519	Lamc1	laminin, gamma 1	NA	0.048239	6.368138	1.034002	0.36224476	0.669597
106052	Fbxo4	F-box protein 4	NA	0.135056	2.762684	1.098136	0.36225247	0.669597
213389	Prdm9	PR domain containing 9, transcript variant 1	NA	-0.14627	2.096137	-1.1067	0.36228476	0.669597
384309	Trim56	tripartite motif-containing 56	NA	0.225448	0.689713	1.16914	0.36228714	0.669597
232975	Atp1a3	ATPase, Na+/K+ transporting, alpha 3 polypeptide, tr	NA	-0.04518	9.417401	-1.03181	0.36242674	0.669779
229706	Slc6a17	solute carrier family 6 (neurotransmitter transporter),	NA	-0.05602	6.889085	-1.03959	0.36251419	0.669865
108068	Grm2	glutamate receptor, metabotropic 2	NA	-0.08051	3.956469	-1.05739	0.36267721	0.670038
21372	Tbl1x	transducin (beta)-like 1 X-linked, transcript variant X3	NA	0.047436	6.923341	1.033427	0.36278185	0.670038
68792	Srpx2	sushi-repeat-containing protein, X-linked 2, transcript	NA	-0.26734	0.625229	-1.20359	0.36283578	0.670038
55980	Impa1	inositol (myo)-1(or 4)-monophosphatase 1, transcript	NA	-0.05179	6.073046	-1.03655	0.3628733	0.670038
66573	Dzip1	DAZ interacting protein 1, transcript variant X11	NA	0.053716	7.478294	1.037935	0.36289727	0.670038
108167456	Gm45998	predicted gene, 45998	NA	0.258339	1.21707	1.196101	0.36290193	0.670038
105559	Mbnl2	muscleblind like splicing factor 2, transcript variant X:	NA	0.081725	5.0345	1.058283	0.36296066	0.670038
107526	Gimap4	GTPase, IMAP family member 4, transcript variant 1	NA	-0.23528	0.635065	-1.17713	0.36298841	0.670038
53311	Mybph	myosin binding protein H, transcript variant 1	NA	-0.34675	0.046755	-1.27169	0.36300047	0.670038
224250	Cldnd1	claudin domain containing 1, transcript variant 4	NA	-0.04918	6.353813	-1.03468	0.36301937	0.670038
54161	Copg1	coatomer protein complex, subunit gamma 1, transcri	NA	0.044913	7.447881	1.031621	0.36306565	0.670047
68636	Fahd1	fumarylacetoacetate hydrolase domain containing 1	NA	0.097517	3.307235	1.069931	0.36321034	0.670238
75234	Rnf19b	ring finger protein 19B, transcript variant 2	NA	0.055641	6.206817	1.039321	0.36335752	0.670275
76960	Bcas1	breast carcinoma amplified sequence 1, transcript va	NA	0.092548	3.345057	1.066251	0.36335779	0.670275
212439	AA986860	expressed sequence AA986860, transcript variant X1	NA	-0.26933	0.328894	-1.20525	0.36339293	0.670275
17829	Muc1	mucin 1, transmembrane	NA	-0.12203	2.407193	-1.08826	0.36339508	0.670275
17991	Ndufa2	NADH:ubiquinone oxidoreductase subunit A2	NA	0.078139	4.719847	1.055655	0.36362887	0.67063
12978	Csf1r	colony stimulating factor 1 receptor	NA	-0.08749	4.383373	-1.06252	0.36389778	0.670931
245450	Slitrk2	SLIT and NTRK-like family, member 2, transcript vari	NA	0.067517	4.754863	1.047911	0.36391103	0.670931
12394	Runx1	runt related transcription factor 1, transcript variant 2	NA	0.179173	1.314016	1.132235	0.36392249	0.670931
208583	Nek11	NIMA (never in mitosis gene a)-related expressed kin	NA	0.186777	1.233301	1.138218	0.36395725	0.670931
102634333	1110002E22Rik	RIKEN cDNA 1110002E22 gene, transcript variant X'	NA	-0.18054	1.288615	-1.13331	0.36426961	0.671431
67712	Slc25a37	solute carrier family 25, member 37	NA	-0.15947	4.630871	-1.11688	0.36451323	0.671804
71116	Stx18	syntaxin 18, transcript variant X2	NA	0.085481	4.319789	1.061042	0.36468328	0.672041
30791	Slc39a1	solute carrier family 39 (zinc transporter), member 1,	NA	-0.05461	5.62079	-1.03858	0.36474265	0.672075
244049	Mctp2	multiple C2 domains, transmembrane 2, transcript va	NA	0.297502	0.017422	1.229015	0.36481485	0.672132
108167693	Gm46137	predicted gene, 46137	NA	0.230102	0.763756	1.172918	0.36505788	0.672466
76522	Lsm8	LSM8 homolog, U6 small nuclear RNA associated	NA	-0.11596	5.067064	-1.0837	0.36510048	0.672466
320110	B230369F24Rik	RIKEN cDNA B230369F24 gene	NA	-0.18909	1.139677	-1.14005	0.36513655	0.672466
108105	B3gnt5	UDP-GlcNAc:betaGal beta-1,3-N-acetylglucosaminylt	NA	0.048981	5.864916	1.034534	0.36516166	0.672466
67498	Kcnv1	potassium channel, subfamily V, member 1	NA	0.109992	3.791406	1.079222	0.36561241	0.673193
78257	Lrrc9	leucine rich repeat containing 9, transcript variant X4	NA	0.168574	2.229952	1.123947	0.36563887	0.673193

226541	Klhl20	kelch-like 20	NA	-0.06149	5.18822	-1.04354	0.36577272	0.673363
100465	Mob3c	MOB kinase activator 3C	NA	-0.12543	2.584957	-1.09083	0.36599617	0.673698
70638	Fam189a1	family with sequence similarity 189, member A1, tran	NA	0.06789	4.958663	1.048183	0.36603736	0.673698
433586	Maml3	mastermind like transcriptional coactivator 3	NA	0.070997	4.552491	1.050442	0.36627295	0.673992
59049	Slc22a17	solute carrier family 22 (organic cation transporter), m	NA	0.041079	7.818406	1.028883	0.36631771	0.673992
56473	Fads2	fatty acid desaturase 2	NA	-0.04616	8.25433	-1.03251	0.36632182	0.673992
14062	F2r	coagulation factor II (thrombin) receptor	NA	-0.05319	6.166753	-1.03755	0.3664513	0.674154
27204	Syn3	synapsin III, transcript variant X3	NA	0.060852	6.965626	1.043081	0.36650082	0.674169
228714	Kat14	lysine acetyltransferase 14, transcript variant 1	NA	-0.06571	4.956649	-1.0466	0.36680875	0.674604
102093	Phkb	phosphorylase kinase beta, transcript variant 3	NA	0.063991	4.891536	1.045354	0.36685208	0.674604
69168	Bola1	bolA-like 1 (E. coli), transcript variant X1	NA	0.145785	2.82148	1.106332	0.36686748	0.674604
75723	Amotl1	angiomotin-like 1, transcript variant X4	NA	-0.06319	6.063155	-1.04477	0.36690299	0.674604
66689	Klhl28	kelch-like 28, transcript variant X1	NA	-0.06154	5.284769	-1.04358	0.36709613	0.674816
241075	Plekhm3	pleckstrin homology domain containing, family M, me	NA	-0.08867	4.085327	-1.06339	0.36710121	0.674816
69129	Pex11g	peroxisomal biogenesis factor 11 gamma, transcript v	NA	-0.29174	1.232046	-1.22412	0.36721338	0.674893
72020	Zfp654	zinc finger protein 654, transcript variant X1	NA	0.087474	4.693674	1.062508	0.36722634	0.674893
27398	Mrpl2	mitochondrial ribosomal protein L2, transcript variant	NA	0.094129	4.305313	1.067421	0.3673665	0.675001
75732	Iqcd	IQ motif containing D	NA	-0.2668	0.739882	-1.20314	0.36736799	0.675001
208292	Zfp871	zinc finger protein 871, transcript variant X1	NA	-0.05735	6.904003	-1.04055	0.36748712	0.675132
231724	Rad9b	RAD9 checkpoint clamp component B, transcript vari	NA	0.14057	3.512704	1.10234	0.36755303	0.675132
320609	Strip2	striatin interacting protein 2, transcript variant X1	NA	0.122103	3.062407	1.08832	0.3675924	0.675132
269120	Optc	opticin, transcript variant X4	NA	0.148865	1.972081	1.108697	0.36760515	0.675132
99650	4933434E20Rik	RIKEN cDNA 4933434E20 gene, transcript variant 1	NA	-0.05396	5.816498	-1.03811	0.36789205	0.675523
238271	Kcnh5	potassium voltage-gated channel, subfamily H (eag-r	NA	0.233025	0.589025	1.175297	0.36790099	0.675523
18746	Pkm	pyruvate kinase, muscle, transcript variant 1	NA	-0.04337	9.451519	-1.03052	0.36806519	0.675748
68342	Ndufb10	NADH:ubiquinone oxidoreductase subunit B10	NA	0.053309	5.889939	1.037642	0.36820565	0.675784
320844	Amigo3	adhesion molecule with Ig like domain 3	NA	-0.19052	1.069752	-1.14118	0.36820811	0.675784
68268	Zdhhc21	zinc finger, DHHC domain containing 21, transcript va	NA	0.076802	5.725107	1.054678	0.36820909	0.675784
83771	Tas1r3	taste receptor, type 1, member 3	NA	0.287809	-0.13287	1.220785	0.36831186	0.675894
12259	C1qa	complement component 1, q subcomponent, alpha po	NA	-0.11027	3.087327	-1.07943	0.3683521	0.675894
19946	Rpl30	ribosomal protein L30, transcript variant 1	NA	0.05365	7.845431	1.037887	0.36853794	0.6761
218613	Mier3	MIER family member 3	NA	0.077067	5.501553	1.054871	0.36854769	0.6761
14763	Gpr37	G protein-coupled receptor 37	NA	0.139574	2.871729	1.10158	0.36861421	0.676146
74776	Ppa2	pyrophosphatase (inorganic) 2, transcript variant 1	NA	0.060571	4.897818	1.042878	0.36878616	0.676385
57431	Dnajc4	DnaJ heat shock protein family (Hsp40) member C4,	NA	0.132974	2.841314	1.096552	0.36885895	0.67643
72168	Aifm3	apoptosis-inducing factor, mitochondrion-associated :	NA	-0.0964	3.794262	-1.0691	0.36897619	0.67643
12866	Cox7a2	cytochrome c oxidase subunit 7A2	NA	0.044012	6.652499	1.030977	0.36899052	0.67643
66854	Trim35	tripartite motif-containing 35	NA	0.041297	8.224559	1.029038	0.36899747	0.67643
234663	Dync1li2	dynein, cytoplasmic 1 light intermediate chain 2	NA	0.03827	7.923032	1.026882	0.36907085	0.67643
224829	Trerf1	transcriptional regulating factor 1, transcript variant 1	NA	-0.05566	5.359154	-1.03933	0.36909046	0.67643
227743	Mapkap1	mitogen-activated protein kinase associated protein 1	NA	-0.04824	6.075403	-1.034	0.36910173	0.67643
225913	Tkfc	triokinase, FMN cyclase, transcript variant X3	NA	0.068842	4.79675	1.048874	0.36925898	0.676642
70572	Ipo5	importin 5, transcript variant X2	NA	0.039316	7.6273	1.027626	0.36943968	0.676738
94047	Tmem121b	transmembrane protein 121B	NA	-0.06981	4.976691	-1.04958	0.36949383	0.676738
170644	Ubn1	ubinuclein 1, transcript variant 2	NA	-0.05527	6.133511	-1.03905	0.36950728	0.676738
27359	Sytl4	synaptotagmin-like 4, transcript variant 3	NA	0.22951	0.570161	1.172437	0.3695073	0.676738
13056	Cyb561	cytochrome b-561, transcript variant X2	NA	-0.06874	4.899153	-1.0488	0.36952673	0.676738
102632461	Gm30528	predicted gene, 30528, transcript variant X1	NA	-0.51016	0.28424	-1.42421	0.36956074	0.676738
213498	Arhgef11	Rho guanine nucleotide exchange factor (GEF) 11, tr	NA	0.049607	6.364974	1.034983	0.36978079	0.677036
67895	Ppa1	pyrophosphatase (inorganic) 1	NA	-0.04882	6.504896	-1.03442	0.36980669	0.677036
227638	Qsox2	quiescin Q6 sulfhydryl oxidase 2, transcript variant 1	NA	0.072003	4.529118	1.051175	0.36989779	0.677122
72098	Tmem68	transmembrane protein 68, transcript variant X1	NA	-0.05986	5.275765	-1.04236	0.36993677	0.677122
320202	Lefty2	left-right determination factor 2	NA	-0.19635	1.044064	-1.1458	0.37011462	0.677371
214854	Neurl3	neuralized E3 ubiquitin protein ligase 3	NA	0.242764	0.216906	1.183258	0.37017151	0.677384
22778	Ikzf1	IKAROS family zinc finger 1, transcript variant X19	NA	0.122434	3.126327	1.08857	0.37020499	0.677384
16164	Il13ra1	interleukin 13 receptor, alpha 1	NA	-0.10876	3.218586	-1.0783	0.3704463	0.677677
69747	Zswim7	zinc finger SWIM-type containing 7	NA	0.189396	1.413184	1.140286	0.37051321	0.677677
13924	Ptprv	protein tyrosine phosphatase, receptor type, V	NA	0.401734	-0.96084	1.321095	0.37052896	0.677677
100608	Noc4l	NOC4 like	NA	-0.0702	4.592688	-1.04986	0.37053162	0.677677
53414	Bysl	bystin-like	NA	-0.05674	5.458612	-1.04011	0.3706416	0.677788
23827	Bpnt1	3'(2'), 5'-bisphosphate nucleotidase 1, transcript varia	NA	-0.05468	5.280549	-1.03863	0.37067576	0.677788
243339	Tmem130	transmembrane protein 130	NA	-0.04802	8.283226	-1.03384	0.37083566	0.678004
74257	Tspan17	tetraspanin 17	NA	-0.10643	3.810293	-1.07656	0.37099769	0.678224
321007	Serac1	serine active site containing 1, transcript variant X10	NA	-0.07061	5.150586	-1.05016	0.37115657	0.678439
66435	Uggt2	UDP-glucose glycoprotein glucosyltransferase 2, tran	NA	0.073275	5.337823	1.052102	0.37127075	0.67851
18993	Pou3f3	POU domain, class 3, transcription factor 3	NA	-0.07485	7.784108	-1.05325	0.37127886	0.67851
108168771	Gm50595	predicted gene, 50595, transcript variant X11	NA	-0.25824	0.066611	-1.19602	0.37135007	0.678522
79196	Osbpl5	oxysterol binding protein-like 5, transcript variant 3	NA	-0.05827	5.599341	-1.04122	0.37136888	0.678522

72886	Yju2	YJU2 splicing factor	NA	-0.0983	3.242046	-1.07051	0.37145504	0.678603
19276	Ptprn2	protein tyrosine phosphatase, receptor type, N polype	NA	0.047712	6.726995	1.033624	0.37155461	0.67869
118568201	LOC118568201	uncharacterized LOC118568201	NA	-0.1916	1.403403	-1.14203	0.37158588	0.67869
21888	Tle4	transducin-like enhancer of split 4, transcript variant X	NA	0.047029	6.607522	1.033135	0.37169625	0.678792
60611	Foxj2	forkhead box J2	NA	-0.05636	5.462035	-1.03984	0.37172525	0.678792
209692	Dhtkd1	dehydrogenase E1 and transketolase domain contair	NA	0.082632	4.185236	1.058948	0.37188307	0.679004
54720	Rcan1	regulator of calcineurin 1, transcript variant 2	NA	-0.04604	6.268552	-1.03243	0.37194807	0.679046
76167	Snrnp35	small nuclear ribonucleoprotein 35 (U11/U12)	NA	-0.09473	4.090422	-1.06787	0.37202421	0.679109
70527	Stambp	STAM binding protein, transcript variant 2	NA	0.052103	5.630535	1.036775	0.3722876	0.679514
66983	Zfp830	zinc finger protein 830	NA	-0.07068	4.175123	-1.05021	0.37234204	0.679537
216558	Ugp2	UDP-glucose pyrophosphorylase 2, transcript variant	NA	0.050694	6.16985	1.035763	0.37270377	0.680121
105245391	Gm40855	predicted gene, 40855, transcript variant X1	NA	0.611212	-0.64525	1.527542	0.37279645	0.680189
233040	Fbxo27	F-box protein 27, transcript variant X2	NA	-0.14504	2.50443	-1.10576	0.37282461	0.680189
104110	Adcy4	adenylate cyclase 4, transcript variant X4	NA	-0.14027	2.421087	-1.10211	0.37287931	0.680205
66656	Eef1d	eukaryotic translation elongation factor 1 delta (guani	NA	-0.04557	6.563333	-1.03209	0.37291694	0.680205
66379	Cox14	cytochrome c oxidase assembly protein 14	NA	-0.08947	4.046868	-1.06398	0.37296645	0.68021
78603	B230216N24Rik	RIKEN cDNA B230216N24 gene	NA	-0.13438	2.276677	-1.09762	0.37303676	0.68021
72949	Ccnt2	cyclin T2	NA	0.06915	5.198199	1.049099	0.37304509	0.68021
242418	Dcaf10	DDB1 and CUL4 associated factor 10	NA	0.059133	5.103829	1.04184	0.37332284	0.680459
26987	Eif4e2	eukaryotic translation initiation factor 4E member 2, tı	NA	0.044851	6.461592	1.031576	0.37340159	0.680459
100504425	Tincr	TINCR ubiquitin domain containing	NA	0.209516	1.733534	1.1563	0.37340995	0.680459
72123	Ccdc71l	coiled-coil domain containing 71 like	NA	0.086762	5.069413	1.061984	0.37346931	0.680459
66567	2510022D24Rik	RIKEN cDNA 2510022D24 gene	NA	0.1231	3.323596	1.089073	0.3735064	0.680459
11778	Ap3s2	adaptor-related protein complex 3, sigma 2 subunit	NA	-0.04889	6.999832	-1.03447	0.37351079	0.680459
66934	Dsn1	DSN1 homolog, MIS12 kinetochore complex compon	NA	-0.10575	3.189932	-1.07606	0.37354084	0.680459
13665	Eif2s1	eukaryotic translation initiation factor 2, subunit 1 alpl	NA	-0.06007	6.233155	-1.04252	0.37355823	0.680459
17528	Mpz	myelin protein zero, transcript variant 2	NA	0.218564	0.994683	1.163575	0.37363163	0.680459
19894	Rph3a	rabphilin 3A, transcript variant 3	NA	-0.06146	6.362022	-1.04352	0.37365036	0.680459
207958	Alg11	asparagine-linked glycosylation 11 (alpha-1,2-manno	NA	0.054297	5.334382	1.038353	0.37366891	0.680459
105245595	Gm29543	predicted gene 29543	NA	-0.2379	0.501958	-1.17928	0.37368398	0.680459
13798	En1	engrailed 1	NA	0.090547	3.852951	1.064774	0.37392784	0.680827
72184	Klhl35	kelch-like 35	NA	0.177768	1.534542	1.131133	0.37405633	0.680985
70796	Zdhhc1	zinc finger, DHHC domain containing 1, transcript var	NA	-0.07379	4.262574	-1.05248	0.37415411	0.681087
327959	Xaf1	XIAP associated factor 1, transcript variant X1	NA	0.316109	-0.02941	1.244969	0.37427665	0.681234
433752	Frg2f1	FSHD region gene 2 family member 1	NA	0.137919	2.441476	1.100317	0.37436472	0.681318
74153	Uba7	ubiquitin-like modifier activating enzyme 7	NA	0.286462	0.016944	1.219646	0.37442697	0.681355
11928	Atp1a1	ATPase, Na+/K+ transporting, alpha 1 polypeptide	NA	-0.04046	7.455052	-1.02844	0.37464003	0.681666
16790	Anpep	alanyl (membrane) aminopeptidase	NA	0.101191	3.455442	1.072659	0.37471399	0.681725
68991	Ssu72	Ssu72 RNA polymerase II CTD phosphatase homolo	NA	0.064452	5.608657	1.045688	0.37490212	0.681991
72014	Btbd17	BTB (POZ) domain containing 17, transcript variant X	NA	0.12921	3.134611	1.093694	0.37501354	0.682102
12868	Cox8a	cytochrome c oxidase subunit 8A	NA	-0.08473	6.446468	-1.06049	0.37504712	0.682102
60321	Wbp11	WW domain binding protein 11	NA	-0.04627	6.678989	-1.03259	0.37534838	0.682299
50791	Magi2	membrane associated guanylate kinase, WW and PD	NA	0.053076	6.196825	1.037474	0.37542044	0.682299
545370	Hmcn1	hemicentin 1, transcript variant X3	NA	0.154027	2.632103	1.112671	0.37549505	0.682299
270084	Lpcat2	lysophosphatidylcholine acyltransferase 2, transcript	NA	0.110003	2.897952	1.07923	0.37549699	0.682299
408068	Zfp738	zinc finger protein 738	NA	0.068338	4.424722	1.048508	0.37551551	0.682299
235344	Sik2	salt inducible kinase 2, transcript variant X4	NA	0.052305	5.63171	1.03692	0.37552545	0.682299
118567938	LOC118567938	uncharacterized LOC118567938	NA	0.232272	0.403587	1.174683	0.37554304	0.682299
118568596	LOC118568596	uncharacterized LOC118568596	NA	-0.22306	2.57819	-1.1672	0.37556161	0.682299
381626	Rbm33	RNA binding motif protein 33	NA	0.053355	6.743293	1.037675	0.37559474	0.682299
75901	Dcp1a	decapping mRNA 1A	NA	0.063015	5.07078	1.044647	0.37560111	0.682299
72254	1700030K09Rik	RIKEN cDNA 1700030K09 gene	NA	-0.09391	4.047121	-1.06726	0.37561678	0.682299
19230	Twf1	twinfilin actin binding protein 1	NA	0.045097	6.341073	1.031752	0.37567534	0.682329
16716	Ky	kyphoscoliosis peptidase	NA	0.163613	1.901694	1.120089	0.37574454	0.682379
140630	Ube4a	ubiquitination factor E4A, transcript variant X6	NA	0.042691	6.61692	1.030033	0.37579507	0.682395
17131	Smad7	SMAD family member 7	NA	-0.11084	4.020937	-1.07985	0.3758866	0.682416
22793	Zyx	zyxin, transcript variant X4	NA	-0.05117	5.771906	-1.03611	0.37589079	0.682416
118567508	LOC118567508	uncharacterized LOC118567508	NA	0.21039	0.965786	1.157001	0.37607017	0.682642
67880	Dcxr	dicarbonyl L-xylulose reductase, transcript variant 1	NA	0.181003	1.236375	1.133672	0.37610847	0.682642
76508	Ube2d-ps	ubiquitin-conjugating enzyme E2D, pseudogene	NA	0.131079	2.650778	1.095113	0.3761411	0.682642
67949	Nifk	nucleolar protein interacting with the FHA domain of I	NA	0.055782	5.703979	1.039422	0.3761963	0.682666
102635629	Gm32917	predicted gene, 32917, transcript variant X2	NA	0.280009	0.084941	1.214203	0.37630577	0.682789
192169	Ufsp2	UFM1-specific peptidase 2	NA	0.060857	4.818804	1.043085	0.37660322	0.683203
272396	Tarsl2	threonyl-tRNA synthetase-like 2	NA	0.065697	4.885367	1.04659	0.37661834	0.683203
241656	Pak5	p21 (RAC1) activated kinase 5, transcript variant X6	NA	0.049985	6.026117	1.035254	0.37679163	0.68335
100043489	1300002E11Rik	RIKEN cDNA 1300002E11 gene, transcript variant 1	NA	0.098752	3.244299	1.070847	0.3768545	0.68335
100037258	Dnajc3	DnaJ heat shock protein family (Hsp40) member C3	NA	0.055265	5.383235	1.03905	0.37689959	0.68335
65115	Bean1	brain expressed, associated with Nedd4, 1, transcript	NA	0.086025	3.811918	1.061442	0.37690258	0.68335

109711	Actn1	actinin, alpha 1, transcript variant 1	NA	-0.0561	5.419603	-1.03965	0.37692837	0.68335
55934	Rp9	retinitis pigmentosa 9 (human)	NA	0.075283	4.776228	1.053567	0.37695128	0.68335
66437	Fis1	fission, mitochondrial 1, transcript variant 1	NA	0.046604	6.389466	1.032831	0.37705252	0.683458
102626	Mapkapk3	mitogen-activated protein kinase-activated protein kir	NA	0.202413	0.900128	1.150622	0.37722372	0.683581
56711	Plag1	pleiomorphic adenoma gene 1, transcript variant X8	NA	-0.11839	3.20928	-1.08552	0.37723653	0.683581
102636578	Gm33605	predicted gene, 33605, transcript variant X1	NA	-0.23501	1.220189	-1.17691	0.37724674	0.683581
54325	Elovl1	elongation of very long chain fatty acids (FEN1/Elo2,	NA	-0.08617	4.175094	-1.06155	0.37729572	0.683594
72993	Appl1	adaptor protein, phosphotyrosine interaction, PH dom	NA	0.047605	6.557773	1.033547	0.37736849	0.68365
71972	Dnmbp	dynamin binding protein, transcript variant 2	NA	-0.07478	4.195351	-1.0532	0.37748453	0.683784
67097	Rps10	ribosomal protein S10, transcript variant 1	NA	0.044369	7.869867	1.031232	0.37752713	0.683785
380698	Obscn	obscurin, cytoskeletal calmodulin and titin-interacting	NA	0.153829	2.149298	1.112518	0.37763169	0.683898
56496	Tspan6	tetraspanin 6	NA	0.044759	7.009413	1.031511	0.37787423	0.684182
18510	Pax8	paired box 8	NA	0.201295	1.820466	1.14973	0.37787622	0.684182
68303	Fam114a1	family with sequence similarity 114, member A1	NA	-0.11807	3.397088	-1.08528	0.37795622	0.684182
15208	Hes5	hes family bHLH transcription factor 5, transcript varia	NA	-0.0868	4.145728	-1.06201	0.37795672	0.684182
16430	Stt3a	STT3, subunit of the oligosaccharyltransferase compl	NA	-0.04825	6.153636	-1.03401	0.37802843	0.684236
574418	Serinc4	serine incorporator 4, transcript variant 1	NA	0.168213	1.41686	1.123666	0.37821236	0.684492
12532	Cdc25c	cell division cycle 25C	NA	-0.1075	3.115196	-1.07736	0.37836863	0.684594
23873	Faim	Fas apoptotic inhibitory molecule, transcript variant 1	NA	0.059806	4.944278	1.042326	0.37850618	0.684594
11593	Aga	aspartylglucosaminidase, transcript variant 1	NA	0.108424	2.772387	1.07805	0.37851372	0.684594
228355	Madd	MAP-kinase activating death domain, transcript varia	NA	-0.04549	6.902002	-1.03204	0.37851466	0.684594
69183	C1qtnf2	C1q and tumor necrosis factor related protein 2	NA	-0.13841	2.046994	-1.10069	0.37855906	0.684594
108989	Tpr	translocated promoter region, nuclear basket protein	NA	0.043866	7.164898	1.030873	0.37859992	0.684594
237073	Rbm41	RNA binding motif protein 41, transcript variant 2	NA	-0.07269	4.461998	-1.05168	0.37860044	0.684594
74167	Nudt9	nudix (nucleoside diphosphate linked moiety X)-type	NA	0.066333	5.138552	1.047052	0.3786053	0.684594
71883	Coq2	coenzyme Q2 4-hydroxybenzoate polyprenyltransfera	NA	0.065453	5.17302	1.046413	0.37880784	0.684713
66961	Neat1	nuclear paraspeckle assembly transcript 1 (non-prote	NA	0.083372	3.881532	1.059492	0.37885039	0.684713
75430	Anapc15	anaphase promoting complex C subunit 15, transcrip	NA	0.061026	4.976516	1.043207	0.37886353	0.684713
22441	Xlr	X-linked lymphocyte-regulated, transcript variant 2	NA	0.311531	-0.28478	1.241024	0.37888999	0.684713
107182	Btaf1	B-TFIID TATA-box binding protein associated factor	NA	-0.06514	5.490507	-1.04619	0.37903615	0.684713
212772	Arl14ep	ADP-ribosylation factor-like 14 effector protein, transc	NA	0.061044	5.062791	1.04322	0.3790606	0.684713
71827	Lrrc34	leucine rich repeat containing 34	NA	0.255977	0.545287	1.194144	0.3790986	0.684713
66071	Ethe1	ethylmalonic encephalopathy 1, transcript variant 2	NA	-0.11551	3.084767	-1.08336	0.37912898	0.684713
78115	4930445E18Rik	RIKEN cDNA 4930445E18 gene, transcript variant X	NA	0.219657	0.744467	1.164457	0.37918546	0.684713
69572	Mfsd3	major facilitator superfamily domain containing 3	NA	0.168108	1.880823	1.123584	0.37918602	0.684713
115488324	Gm52120	predicted gene, 52120	NA	0.2635	0.341525	1.200387	0.3792133	0.684713
100042450	Smim17	small integral membrane protein 17	NA	-0.11112	2.706643	-1.08006	0.37921833	0.684713
18099	Nlk	nemo like kinase	NA	0.049464	6.308064	1.03488	0.37922558	0.684713
269870	Zfp446	zinc finger protein 446, transcript variant 2	NA	-0.08446	4.055781	-1.06029	0.37926012	0.684713
13876	Erg	ETS transcription factor, transcript variant X7	NA	0.102155	3.080928	1.073376	0.37934851	0.684797
118568300	LOC118568300	igE-binding protein-like	NA	-0.35377	0.761782	-1.2779	0.37946969	0.684864
246694	Hps5	HPS5, biogenesis of lysosomal organelles complex 2	NA	-0.06663	4.764748	-1.04727	0.37946971	0.684864
230576	Ttc22	tetratricopeptide repeat domain 22, transcript variant	NA	0.234166	0.690413	1.176226	0.3798465	0.685468
12724	Clcn2	chloride channel, voltage-sensitive 2	NA	-0.05802	5.202905	-1.04104	0.37989871	0.685486
52009	Jpt2	Jupiter microtubule associated homolog 2	NA	-0.04911	6.214763	-1.03463	0.3800018	0.685547
24004	Rai2	retinoic acid induced 2, transcript variant 2	NA	-0.08089	4.303649	-1.05767	0.38001708	0.685547
67776	Vwa5a	von Willebrand factor A domain containing 5A, transc	NA	-0.09541	3.259818	-1.06837	0.3800839	0.685592
18187	Nrp2	neuropilin 2, transcript variant 2	NA	-0.04378	7.223385	-1.03081	0.38019608	0.685718
52690	Setd3	SET domain containing 3, transcript variant X1	NA	0.046221	6.945728	1.032557	0.38034556	0.685789
76425	Gid8	GID complex subunit 8, transcript variant 3	NA	0.054229	6.541266	1.038304	0.3803929	0.685789
52245	Commd2	COMM domain containing 2	NA	0.064075	4.540672	1.045415	0.38040129	0.685789
212728	Tarbp1	TAR RNA binding protein 1, transcript variant X1	NA	0.11088	3.291547	1.079887	0.38040629	0.685789
232748	Tcaf2	TRPM8 channel-associated factor 2	NA	-0.15028	1.941982	-1.10979	0.38047279	0.685789
68099	Cibar1	CBY1 interacting BAR domain containing 1, transcrip	NA	0.074136	5.742259	1.05273	0.38048807	0.685789
229658	Vangl1	VANGL planar cell polarity 1, transcript variant X1	NA	0.086458	3.69321	1.06176	0.38055508	0.685834
118567920	LOC118567920	uncharacterized LOC118567920	NA	-0.30322	-0.03026	-1.23389	0.380722	0.686026
338354	Zfp780b	zinc finger protein 780B, transcript variant X6	NA	0.059524	4.72499	1.042122	0.38076555	0.686026
18003	Nedd9	neural precursor cell expressed, developmentally dov	NA	0.067124	4.984709	1.047626	0.38082305	0.686026
68703	Rere	arginine glutamic acid dipeptide (RE) repeats, transcr	NA	0.040917	7.506289	1.028767	0.38083052	0.686026
230908	Tardbp	TAR DNA binding protein, transcript variant 1	NA	-0.04712	8.461909	-1.0332	0.3812781	0.686756
30945	Rnf19a	ring finger protein 19A	NA	0.049126	5.839157	1.034638	0.38140116	0.686902
104418	Dgkz	diacylglycerol kinase zeta, transcript variant 2	NA	-0.05001	6.664738	-1.03527	0.38174786	0.687403
100042335	Rps15a-ps5	ribosomal protein S15A, pseudogene 5	NA	-0.06158	5.052842	-1.04361	0.38185486	0.687403
102640250	Gm2885	predicted gene 2885	NA	-0.14723	1.62929	-1.10744	0.38190978	0.687403
242667	Dlgap3	DLG associated protein 3, transcript variant X3	NA	0.059165	6.475985	1.041863	0.38195682	0.687403
171567	Nme7	NME/NM23 family member 7, transcript variant 3	NA	-0.06924	4.342666	-1.04916	0.38198438	0.687403
105732	Fam83h	family with sequence similarity 83, member H, transc	NA	-0.11792	2.622475	-1.08517	0.38202412	0.687403
56399	Akap8	A kinase (PRKA) anchor protein 8, transcript variant	NA	0.043126	7.265874	1.030344	0.38208034	0.687403

20638	Snrpb	small nuclear ribonucleoprotein B	NA	0.057416	6.406381	1.0406	0.38215101	0.687403
71643	Zgrf1	zinc finger, GRF-type containing 1, transcript variant :	NA	0.113801	3.109069	1.082076	0.38216079	0.687403
66101	Ppih	peptidyl prolyl isomerase H, transcript variant 1	NA	-0.07343	4.534883	-1.05222	0.38216202	0.687403
74761	Mxra8	matrix-remodelling associated 8	NA	0.08776	3.803341	1.062719	0.38221049	0.687403
26558	Homer3	homer scaffolding protein 3, transcript variant 1	NA	0.063332	5.24677	1.044876	0.38224112	0.687403
26874	Abcd2	ATP-binding cassette, sub-family D (ALD), member 2	NA	-0.07563	4.287075	-1.05382	0.38226758	0.687403
240675	Vwa2	von Willebrand factor A domain containing 2	NA	0.275897	0.674725	1.210746	0.38227065	0.687403
93873	Pcdhb2	protocadherin beta 2	NA	0.308239	-0.169	1.238195	0.38234939	0.687468
80890	Trim2	tripartite motif-containing 2, transcript variant 1	NA	0.049803	8.217961	1.035124	0.38244378	0.687562
224619	Traf7	TNF receptor-associated factor 7, transcript variant 1	NA	-0.04273	6.776468	-1.03006	0.38260364	0.687774
20316	Sdf2	stromal cell derived factor 2, transcript variant 1	NA	0.066488	5.267568	1.047165	0.3828053	0.687915
332175	Zdhhc23	zinc finger, DHHC domain containing 23, transcript va	NA	0.087771	3.736683	1.062727	0.38293896	0.687915
22781	Ikzf4	IKAROS family zinc finger 4, transcript variant 1	NA	0.051983	5.716224	1.036689	0.38298619	0.687915
102640934	Gm36880	predicted gene, 36880, transcript variant X2	NA	-0.28185	-0.0677	-1.21575	0.38300727	0.687915
18089	Nkx2-3	NK2 homeobox 3	NA	-0.23895	0.420958	-1.18014	0.38301151	0.687915
320919	A230107N01Rik	RIKEN cDNA A230107N01 gene	NA	-0.17359	1.562267	-1.12786	0.38309616	0.687915
100039953	Gfy	golgi-associated olfactory signaling regulator	NA	-0.47246	-0.51375	-1.38748	0.38312462	0.687915
231470	Fras1	Fraser extracellular matrix complex subunit 1, transcr	NA	-0.0771	4.266849	-1.0549	0.38321694	0.687915
17149	Magoh	mago homolog, exon junction complex core compone	NA	-0.06197	5.487107	-1.04389	0.38321934	0.687915
110257	Hba-a2	hemoglobin alpha, adult chain 2	NA	-0.17823	9.260385	-1.13149	0.38325389	0.687915
12340	Capza1	capping protein (actin filament) muscle Z-line, alpha ´	NA	-0.03835	7.317085	-1.02694	0.383307	0.687915
277973	Slc9a5	solute carrier family 9 (sodium/hydrogen exchanger),	NA	-0.06062	5.503794	-1.04292	0.38331009	0.687915
23970	Pacsin2	protein kinase C and casein kinase substrate in neuro	NA	-0.0663	4.657748	-1.04703	0.38341402	0.687915
102637048	Gm38492	predicted gene, 38492, transcript variant X1	NA	0.276096	-0.11578	1.210914	0.38343535	0.687915
21645	Tcte1	t-complex-associated testis expressed 1	NA	0.228844	0.578232	1.171896	0.38350201	0.687915
234219	Helt	helt bHLH transcription factor	NA	0.233669	0.338224	1.175821	0.38352999	0.687915
102640879	Gm36839	predicted gene, 36839	NA	-0.1849	1.331825	-1.13674	0.38355164	0.687915
21827	Thbs3	thrombospondin 3, transcript variant X1	NA	-0.06279	5.507211	-1.04449	0.3836576	0.687915
230587	Glis1	GLIS family zinc finger 1, transcript variant X5	NA	-0.26107	0.453815	-1.19837	0.38368205	0.687915
11745	Anxa3	annexin A3	NA	0.110706	3.50141	1.079757	0.38372997	0.687915
74147	Ehhadh	enoyl-Coenzyme A, hydratase/3-hydroxyacyl Coenzy	NA	0.184692	1.24536	1.136575	0.38373062	0.687915
118568138	LOC118568138	uncharacterized LOC118568138	NA	-0.17834	1.684286	-1.13158	0.38373496	0.687915
387314	Tmtc1	transmembrane and tetratricopeptide repeat containir	NA	0.061698	4.756148	1.043694	0.38374689	0.687915
108089	Rnf144a	ring finger protein 144A, transcript variant X6	NA	0.046946	6.117635	1.033076	0.38378266	0.687915
93835	Amn	amnionless, transcript variant X1	NA	0.202792	0.850949	1.150924	0.38380909	0.687915
407790	Ndufa4l2	Ndufa4, mitochondrial complex associated like 2	NA	0.139098	2.467828	1.101216	0.38382066	0.687915
20224	Sar1a	secretion associated Ras related GTPase 1A, transcr	NA	0.049709	7.244881	1.035056	0.38384503	0.687915
328133	Slc39a9	solute carrier family 39 (zinc transporter), member 9,	NA	-0.04923	5.63531	-1.03471	0.38386597	0.687915
108148	Galnt2	polypeptide N-acetylgalactosaminyltransferase 2	NA	-0.04919	7.111552	-1.03468	0.38394838	0.687935
79565	Mettl27	methyltransferase like 27, transcript variant X7	NA	0.132935	2.36818	1.096522	0.38398183	0.687935
118568732	LOC118568732	uncharacterized LOC118568732	NA	0.232506	0.872868	1.174874	0.38400413	0.687935
268670	Zfp759	zinc finger protein 759, transcript variant X3	NA	-0.10561	3.447988	-1.07595	0.38409594	0.688024
14789	P3h3	prolyl 3-hydroxylase 3, transcript variant 2	NA	-0.05053	5.496786	-1.03564	0.38436787	0.688435
67096	Mmachc	methylmalonic aciduria cblC type, with homocystinuri	NA	-0.08121	4.387499	-1.0579	0.38462486	0.68882
54218	B3galt4	UDP-Gal:betaGlcNAc beta 1,3-galactosyltransferase,	NA	0.138239	2.455304	1.100561	0.38473451	0.68894
212391	Lcor	ligand dependent nuclear receptor corepressor, trans	NA	-0.06559	5.219697	-1.04652	0.38501013	0.689283
66861	Dnajc10	DnaJ heat shock protein family (Hsp40) member C10	NA	0.059686	6.376947	1.042239	0.38501047	0.689283
102635756	Gm15689	predicted gene 15689, transcript variant X2	NA	-0.4326	-0.79782	-1.34966	0.3851387	0.689436
72133	Trub1	TruB pseudouridine (psi) synthase family member 1,	NA	0.056414	5.033887	1.039878	0.38522512	0.689515
67515	Ttc33	tetratricopeptide repeat domain 33, transcript variant	NA	-0.07045	4.980857	-1.05004	0.38537198	0.689702
14972	H2-K1	histocompatibility 2, K1, K region, transcript variant 1	NA	0.13057	2.653381	1.094726	0.38560058	0.689952
50762	Fbxo6	F-box protein 6, transcript variant 5	NA	0.126234	3.073748	1.091441	0.3856225	0.689952
102060	Gadd45gip1	growth arrest and DNA-damage-inducible, gamma int	NA	-0.07869	4.277532	-1.05606	0.38563887	0.689952
54635	Pdgfc	platelet-derived growth factor, C polypeptide, transcri	NA	0.085924	3.622958	1.061367	0.38584182	0.690098
225362	Reep2	receptor accessory protein 2, transcript variant 1	NA	0.048028	6.438985	1.033851	0.38586537	0.690098
101206	Tada3	transcriptional adaptor 3, transcript variant X1	NA	0.05657	5.618467	1.03999	0.38593138	0.690098
18761	Prkcq	protein kinase C, theta	NA	0.093383	3.377706	1.066869	0.38594879	0.690098
72125	Amer2	APC membrane recruitment 2, transcript variant 2	NA	0.047929	7.139639	1.03378	0.38598757	0.690098
104721	Ddx1	DEAD box helicase 1	NA	0.042277	7.227492	1.029738	0.38599929	0.690098
215193	Utp25	UTP25 small subunit processome component, transc	NA	0.06338	5.107339	1.044911	0.3860172	0.690098
15013	H2-Q2	histocompatibility 2, Q region locus 2, transcript varia	NA	0.240631	0.608041	1.181509	0.38609347	0.690159
381359	Prdm12	PR domain containing 12	NA	0.112233	2.892521	1.0809	0.38637764	0.690589
26380	Esrrb	estrogen related receptor, beta, transcript variant X2	NA	-0.10142	3.26075	-1.07283	0.38641895	0.690589
218440	Ankrd34b	ankyrin repeat domain 34B	NA	0.137209	2.701065	1.099775	0.38650443	0.690666
78581	Utp23	UTP23 small subunit processome component	NA	-0.08334	3.929439	-1.05947	0.38663293	0.690778
211978	Zfyve26	zinc finger, FYVE domain containing 26, transcript va	NA	-0.07955	4.175986	-1.05669	0.38665216	0.690778
13685	Eif4ebp1	eukaryotic translation initiation factor 4E binding prote	NA	-0.17451	1.593938	-1.12858	0.38672203	0.690827
228859	Fitm2	fat storage-inducing transmembrane protein 2	NA	-0.11374	2.400722	-1.08203	0.38677223	0.690841

235604	Camkv	CaM kinase-like vesicle-associated	NA	-0.06456	6.357599	-1.04577	0.38694013	0.690941
19981	Rpl37a	ribosomal protein L37a	NA	0.051033	7.824144	1.036006	0.38697788	0.690941
60507	Qtrt1	queuine tRNA-ribosyltransferase catalytic subunit 1, t	NA	0.082741	4.084096	1.059028	0.38699149	0.690941
319158	H4c9	H4 clustered histone 9	NA	-0.20765	0.905473	-1.1548	0.38703547	0.690941
18146	Npdc1	neural proliferation, differentiation and control 1	NA	0.046563	7.755374	1.032802	0.38712412	0.690941
20345	Selplg	selectin, platelet (p-selectin) ligand, transcript variant	NA	-0.33495	-0.23746	-1.26133	0.38718205	0.690941
216136	Ilvbl	ilvB (bacterial acetolactate synthase)-like, transcript v	NA	0.07316	4.529331	1.052018	0.38728642	0.690941
213233	Tapbpl	TAP binding protein-like	NA	-0.15412	2.21807	-1.11275	0.38730057	0.690941
21401	Tcea3	transcription elongation factor A (SII), 3	NA	0.25025	0.357676	1.189413	0.38730839	0.690941
230594	Tut4	terminal uridylyl transferase 4, transcript variant X9	NA	0.044017	6.26728	1.030981	0.38737655	0.690941
234725	Zfp612	zinc finger protein 612, transcript variant X8	NA	-0.05226	5.190953	-1.03689	0.38739114	0.690941
216549	Aftph	aftiphilin, transcript variant 3	NA	0.051336	5.880648	1.036224	0.38741619	0.690941
320312	A430035B10Rik	RIKEN cDNA A430035B10 gene	NA	-0.1699	1.305687	-1.12498	0.38742653	0.690941
66443	Tnfaip8l1	tumor necrosis factor, alpha-induced protein 8-like 1	NA	-0.09917	3.445399	-1.07116	0.38743445	0.690941
100163	Pafah2	platelet-activating factor acetylhydrolase 2, transcript	NA	-0.09795	3.68958	-1.07025	0.38750868	0.690941
105245865	Gm41253	predicted gene, 41253, transcript variant X5	NA	-0.2477	0.388188	-1.18731	0.38757634	0.690941
75669	Pik3r4	phosphoinositide-3-kinase regulatory subunit 4	NA	-0.06009	4.927156	-1.04253	0.387581	0.690941
208982	Hmgcll1	3-hydroxymethyl-3-methylglutaryl-Coenzyme A lyase	NA	0.060918	4.952909	1.043129	0.38759249	0.690941
78935	Saal1	serum amyloid A-like 1	NA	-0.07784	4.450231	-1.05544	0.38773274	0.691035
67053	Rpp14	ribonuclease P 14 subunit	NA	0.057257	5.084217	1.040486	0.38774869	0.691035
18951	Septin5	septin 5	NA	-0.04119	8.62512	-1.02896	0.3878314	0.691035
27354	Nbn	nibrin	NA	0.06879	4.333257	1.048836	0.38786035	0.691035
56017	Slc2a8	solute carrier family 2, (facilitated glucose transporter	NA	0.128442	3.355135	1.093112	0.38790971	0.691035
66077	Aurkaip1	aurora kinase A interacting protein 1	NA	-0.06575	4.764867	-1.04663	0.38792267	0.691035
101488	Slco2b1	solute carrier organic anion transporter family, memb	NA	0.087194	3.722379	1.062302	0.38795876	0.691035
73373	Phospho2	phosphatase, orphan 2	NA	-0.06435	4.752622	-1.04561	0.38800432	0.691035
57080	Gtf2ird1	general transcription factor II I repeat domain-contain	NA	0.050636	6.392332	1.035721	0.38802783	0.691035
105244833	Gm40369	predicted gene, 40369, transcript variant X1	NA	1.176196	-0.82386	2.259802	0.38811265	0.691111
194908	Pld6	phospholipase D family, member 6, transcript variant	NA	-0.17871	1.44472	-1.13187	0.38816322	0.691125
20926	Supt6	SPT6, histone chaperone and transcription elongatio	NA	-0.04472	7.026009	-1.03148	0.38833663	0.691306
26401	Map3k1	mitogen-activated protein kinase kinase kinase 1, tra	NA	0.062609	4.893909	1.044353	0.38836517	0.691306
67148	Ramac	RNA guanine-7 methyltransferase activating subunit	NA	0.05271	6.157073	1.037211	0.3884425	0.691306
118568302	LOC118568302	uncharacterized LOC118568302	NA	-0.21475	0.606589	-1.1605	0.38849018	0.691306
67966	Zcchc10	zinc finger, CCHC domain containing 10	NA	0.112988	3.016366	1.081466	0.38859975	0.691306
229214	Qrfpr	pyroglutamylated RFamide peptide receptor	NA	0.257799	0.21463	1.195653	0.38860194	0.691306
67198	Spats2l	spermatogenesis associated, serine-rich 2-like, trans	NA	0.059226	5.410575	1.041907	0.38869629	0.691306
195434	Utp14b	UTP14B small subunit processome component, trans	NA	0.172231	2.072506	1.1268	0.38870576	0.691306
654440	A430046D13Rik	Riken cDNA A430046D13 gene, transcript variant 1	NA	-0.09701	3.539865	-1.06956	0.38875722	0.691306
75409	Slitrk5	SLIT and NTRK-like family, member 5, transcript vari	NA	0.057532	5.713913	1.040684	0.38881731	0.691306
70127	Dpf3	D4, zinc and double PHD fingers, family 3, transcript	NA	0.139839	2.767274	1.101782	0.38882016	0.691306
353242	Mrpl21	mitochondrial ribosomal protein L21, transcript varian	NA	0.071987	4.586814	1.051164	0.38882521	0.691306
65098	Zfand6	zinc finger, AN1-type domain 6, transcript variant X6	NA	-0.0486	6.168448	-1.03426	0.38884817	0.691306
100637	N4bp2l1	NEDD4 binding protein 2-like 1	NA	0.077865	3.837633	1.055455	0.38893929	0.691306
381113	Cdkl4	cyclin-dependent kinase-like 4, transcript variant 3	NA	-0.1328	2.356228	-1.09642	0.3889409	0.691306
54646	Ppp1r3f	protein phosphatase 1, regulatory subunit 3F, transcr	NA	0.086616	4.129965	1.061877	0.38900023	0.691306
14537	Gcnt1	glucosaminyl (N-acetyl) transferase 1, core 2, transcr	NA	-0.18673	1.048066	-1.13818	0.38901805	0.691306
102640451	Gm11767	predicted gene 11767, transcript variant X1	NA	0.129759	2.612578	1.094111	0.38902946	0.691306
53381	Prdx4	peroxiredoxin 4, transcript variant 2	NA	0.063134	4.928341	1.044733	0.3891022	0.69136
83602	Gtf2a1	general transcription factor II A, 1, transcript variant 1	NA	0.057851	6.655515	1.040914	0.38932175	0.691559
99375	Cul4a	cullin 4A, transcript variant 3	NA	0.047229	6.427115	1.033279	0.38933219	0.691559
246316	Lgi2	leucine-rich repeat LGI family, member 2, transcript v	NA	0.065245	5.166693	1.046263	0.3893417	0.691559
71870	Cfap45	cilia and flagella associated protein 45	NA	-0.19468	0.989921	-1.14447	0.38955094	0.691855
19345	Rab5c	RAB5C, member RAS oncogene family, transcript va	NA	0.047454	7.284483	1.033439	0.38964971	0.691899
78834	Zfp623	zinc finger protein 623	NA	-0.06406	4.628648	-1.04541	0.38966081	0.691899
12764	Cmas	cytidine monophospho-N-acetylneuraminic acid synth	NA	0.04335	6.93974	1.030504	0.38984694	0.692154
330503	Gm5113	predicted gene 5113	NA	0.067044	4.59798	1.047568	0.38996847	0.69226
14425	Galnt3	polypeptide N-acetylgalactosaminyltransferase 3, tra	NA	0.185835	1.021545	1.137475	0.38999189	0.69226
100384868	Gm37013	predicted gene, 37013	NA	-0.57011	-0.24111	-1.48464	0.39024003	0.692625
66868	Mfsd1	major facilitator superfamily domain containing 1	NA	0.059367	5.031116	1.042008	0.39032711	0.692638
72296	Rusc1	RUN and SH3 domain containing 1, transcript variant	NA	0.046332	6.956334	1.032636	0.39034158	0.692638
238673	Zfp367	zinc finger protein 367, transcript variant 2	NA	-0.08618	3.623497	-1.06156	0.39038564	0.692638
30051	Spdef	SAM pointed domain containing ets transcription fact	NA	0.275505	-0.05091	1.210418	0.3904177	0.692638
19216	Ptger1	prostaglandin E receptor 1 (subtype EP1), transcript	NA	-0.30209	0.326364	-1.23293	0.39053381	0.692769
71562	Afmid	arylformamidase, transcript variant 3	NA	0.118476	2.475371	1.085587	0.39065815	0.69287
320229	9530052C20Rik	RIKEN cDNA 9530052C20 gene	NA	0.297675	-0.29693	1.229162	0.39067591	0.69287
270091	Lrrc36	leucine rich repeat containing 36, transcript variant 3	NA	0.252932	-0.06382	1.191626	0.39081323	0.693038
15162	Hck	hemopoietic cell kinase, transcript variant 1	NA	0.236505	1.068504	1.178135	0.39097592	0.693188
58802	Kcnmb4	potassium large conductance calcium-activated chan	NA	0.072327	4.647514	1.051411	0.39098347	0.693188

432572	Specc1	sperm antigen with calponin homology and coiled-coi	NA	0.054232	5.322352	1.038307	0.39104119	0.693203
66552	Sppl2a	signal peptide peptidase like 2A, transcript variant X9	NA	0.060031	5.936493	1.042488	0.39107709	0.693203
108760	Galnt16	polypeptide N-acetylgalactosaminyltransferase 16, tra	NA	-0.05565	5.970407	-1.03932	0.39127335	0.693461
74142	Lonp1	lon peptidase 1, mitochondrial	NA	-0.04563	6.13021	-1.03214	0.39130779	0.693461
73173	Pcdh18	protocadherin 18	NA	0.05583	5.558103	1.039457	0.39155399	0.693641
83691	Crispld1	cysteine-rich secretory protein LCCL domain containi	NA	0.143411	2.843195	1.104513	0.39160898	0.693641
259277	Klk8	kallikrein related-peptidase 8, transcript variant X1	NA	0.158942	1.486387	1.116468	0.39161291	0.693641
74684	4930451G09Rik	RIKEN cDNA 4930451G09 gene, transcript variant 1	NA	-0.1294	2.101776	-1.09384	0.3916395	0.693641
19711	Resp18	regulated endocrine-specific protein 18, transcript var	NA	0.081673	4.636839	1.058244	0.39172714	0.693641
20868	Stk10	serine/threonine kinase 10, transcript variant 1	NA	-0.13295	2.588639	-1.09653	0.39173019	0.693641
70478	Mipep	mitochondrial intermediate peptidase, transcript varia	NA	0.061624	4.554608	1.04364	0.39175921	0.693641
17346	Mknk1	MAP kinase-interacting serine/threonine kinase 1, tra	NA	0.075499	4.039996	1.053725	0.39178329	0.693641
14205	Vegfd	vascular endothelial growth factor D, transcript varian	NA	-0.21671	0.592231	-1.16208	0.39179314	0.693641
73569	Vgll3	vestigial like family member 3, transcript variant 3	NA	-0.16872	2.284529	-1.12406	0.39196106	0.693863
117172	2310034C09Rik	RIKEN cDNA 2310034C09 gene	NA	-0.89232	-1.21424	-1.85616	0.39202791	0.693906
23876	Fbln5	fibulin 5, transcript variant X1	NA	0.087017	3.381991	1.062171	0.39207423	0.693913
246782	Atpaf2	ATP synthase mitochondrial F1 complex assembly fa	NA	-0.07125	4.613128	-1.05062	0.39217911	0.694023
71186	4933417D19Rik	RIKEN cDNA 4933417D19 gene	NA	0.247993	0.204925	1.187554	0.39222999	0.694037
231329	Polr2b	polymerase (RNA) II (DNA directed) polypeptide B	NA	0.041902	6.718745	1.02947	0.39229971	0.694085
30057	Timm8b	translocase of inner mitochondrial membrane 8B	NA	0.052403	5.624199	1.036991	0.39253266	0.694371
76499	Clasp2	CLIP associating protein 2, transcript variant 5	NA	0.045215	8.272189	1.031837	0.39254645	0.694371
433864	Nom1	nucleolar protein with MIF4G domain 1, transcript var	NA	0.058071	4.851881	1.041073	0.39278656	0.69472
12874	Cpd	carboxypeptidase D	NA	0.051307	5.907592	1.036203	0.39311445	0.695006
72611	Zfp655	zinc finger protein 655, transcript variant 1	NA	0.07839	5.075838	1.055839	0.39318408	0.695006
16331	Inpp5d	inositol polyphosphate-5-phosphatase D, transcript va	NA	-0.15067	2.318098	-1.11009	0.39322358	0.695006
11569	Aebp2	AE binding protein 2, transcript variant 2	NA	0.04763	5.888629	1.033566	0.39323169	0.695006
16818	Lck	lymphocyte protein tyrosine kinase, transcript variant	NA	-0.10784	2.789333	-1.07762	0.39323969	0.695006
66789	Alg14	asparagine-linked glycosylation 14, transcript variant	NA	-0.08298	3.945774	-1.05921	0.39325362	0.695006
50501	Prok2	prokineticin 2, transcript variant 2	NA	0.235129	0.227519	1.177012	0.39325957	0.695006
108167793	Gm46210	predicted gene, 46210	NA	0.277089	-0.06472	1.211748	0.39329173	0.695006
226823	Kctd3	potassium channel tetramerisation domain containing	NA	0.043729	6.519837	1.030775	0.39333272	0.695006
74071	Lmntd1	lamin tail domain containing 1, transcript variant X3	NA	0.298407	0.222211	1.229786	0.39349528	0.695201
59032	Ppp2r3c	protein phosphatase 2, regulatory subunit B'', gamma	NA	0.05575	5.557558	1.0394	0.39356937	0.695201
216792	Iba57	IBA57 homolog, iron-sulfur cluster assembly, transcri	NA	-0.09245	3.388265	-1.06618	0.39357142	0.695201
103583	Fbxw11	F-box and WD-40 domain protein 11, transcript variar	NA	0.037882	7.149737	1.026606	0.39364087	0.695229
73233	Zfp942	zinc finger protein 942	NA	0.063896	4.673166	1.045285	0.39367612	0.695229
56078	Car5b	carbonic anhydrase 5b, mitochondrial	NA	0.227955	0.743887	1.171173	0.39371503	0.695229
320247	C030023E24Rik	RIKEN cDNA C030023E24 gene	NA	0.101712	3.310965	1.073046	0.39394696	0.695235
18081	Ninj1	ninjurin 1	NA	0.140126	2.514825	1.102002	0.39397282	0.695235
14025	Bcl11a	B cell CLL/lymphoma 11A (zinc finger protein), transc	NA	-0.04876	8.13173	-1.03437	0.39398739	0.695235
269473	Lrig2	leucine-rich repeats and immunoglobulin-like domains	NA	0.054074	5.929624	1.038193	0.3939983	0.695235
75538	Fam71e1	family with sequence similarity 71, member E1, trans	NA	0.143101	3.732711	1.104276	0.39403034	0.695235
12505	Cd44	CD44 antigen, transcript variant 3	NA	-0.10653	2.98659	-1.07664	0.3941252	0.695235
103841	Cuedc1	CUE domain containing 1, transcript variant 1	NA	-0.045	6.503372	-1.03169	0.39412574	0.695235
71782	Ankle2	ankyrin repeat and LEM domain containing 2, transcr	NA	0.054039	5.47939	1.038167	0.39417443	0.695235
76438	Rftn1	raftlin lipid raft linker 1	NA	0.072484	4.448748	1.051526	0.39424885	0.695235
51800	Bok	BCL2-related ovarian killer	NA	0.081382	4.682224	1.058031	0.39425881	0.695235
63953	Dusp10	dual specificity phosphatase 10	NA	-0.10792	2.707637	-1.07768	0.39426534	0.695235
67169	Nradd	neurotrophin receptor associated death domain	NA	-0.11163	3.026561	-1.08045	0.39429199	0.695235
319653	Slc25a40	solute carrier family 25, member 40, transcript varian	NA	0.05866	5.381602	1.041498	0.39433572	0.695235
102639259	Gm35612	predicted gene, 35612	NA	0.339637	-0.35116	1.265438	0.39435903	0.695235
103712	6330403K07Rik	RIKEN cDNA 6330403K07 gene	NA	0.040391	8.139386	1.028393	0.3944813	0.695235
22410	Wnt10b	wingless-type MMTV integration site family, member	NA	0.337497	-0.6567	1.263562	0.39449322	0.695235
237412	Gm4924	predicted gene 4924, transcript variant X2	NA	-0.08303	3.703848	-1.05924	0.39459816	0.695235
66073	Txndc12	thioredoxin domain containing 12 (endoplasmic reticu	NA	-0.04883	5.500267	-1.03443	0.39460299	0.695235
58894	Zfp862-ps	zinc finger protein 862, pseudogene, transcript varian	NA	-0.09154	4.228076	-1.06551	0.39467809	0.695235
67711	Nsmce1	NSE1 homolog, SMC5-SMC6 complex component, tr	NA	0.082751	4.919168	1.059035	0.39473215	0.695235
235048	Zfp599	zinc finger protein 599	NA	-0.09977	3.491811	-1.0716	0.39474002	0.695235
70925	Cdkn2aip	CDKN2A interacting protein, transcript variant 1	NA	-0.06004	5.017901	-1.0425	0.39475624	0.695235
101592	Efl1	elongation factor like GTPase 1, transcript variant 1	NA	0.07281	4.341961	1.051763	0.39486277	0.695235
15234	Hgf	hepatocyte growth factor, transcript variant X1	NA	0.281469	0.526622	1.215432	0.3948867	0.695235
70999	Naa40	N(alpha)-acetyltransferase 40, NatD catalytic subunit	NA	-0.05022	5.899468	-1.03542	0.39498296	0.695235
75614	Rab26os	RAB26, member RAS oncogene family, opposite stra	NA	0.148905	1.950131	1.108728	0.39502076	0.695235
228607	Mavs	mitochondrial antiviral signaling protein, transcript var	NA	-0.11265	2.950016	-1.08121	0.39502134	0.695235
228598	Ebf4	early B cell factor 4	NA	-0.06154	4.490901	-1.04358	0.39502622	0.695235
408065	Zfp456	zinc finger protein 456	NA	0.217368	0.375207	1.16261	0.39503806	0.695235
76799	Tmem234	transmembrane protein 234, transcript variant 1	NA	0.048573	6.010832	1.034241	0.39507385	0.695235
12572	Cdk7	cyclin-dependent kinase 7	NA	0.054829	5.579933	1.038736	0.39507633	0.695235

54343	Atf7ip	activating transcription factor 7 interacting protein, tra	NA	-0.0402	7.326483	-1.02825	0.39508597	0.695235
27176	Rpl7a	ribosomal protein L7A	NA	0.040344	9.348411	1.028359	0.39514768	0.695269
17180	Matn1	matrilin 1, cartilage matrix protein	NA	-0.36751	-0.04994	-1.29013	0.39524762	0.695367
71742	Ulk3	unc-51-like kinase 3, transcript variant X8	NA	0.073939	4.589893	1.052587	0.39528911	0.695367
214239	Ccdc9b	coiled-coil domain containing 9B	NA	-0.11423	2.422038	-1.08239	0.39536232	0.695421
319710	Frmd6	FERM domain containing 6, transcript variant X3	NA	0.066562	4.469082	1.047218	0.39540602	0.695422
17540	Mrvi1	MRV integration site 1, transcript variant X6	NA	-0.17846	1.404795	-1.13167	0.39547628	0.695471
94045	P2rx5	purinergic receptor P2X, ligand-gated ion channel, 5,	NA	-0.25726	0.056448	-1.1952	0.39577162	0.69577
14160	Lgr5	leucine rich repeat containing G protein coupled rece	NA	0.214346	1.436568	1.160178	0.3957788	0.69577
20422	Sem1	SEM1, 26S proteasome complex subunit	NA	-0.04895	5.814027	-1.03451	0.39581661	0.69577
15258	Hipk2	homeodomain interacting protein kinase 2, transcript	NA	-0.05402	7.655163	-1.03815	0.39584045	0.69577
239985	Arid1b	AT rich interactive domain 1B (SWI-like), transcript va	NA	0.047794	6.816031	1.033683	0.39586057	0.69577
16151	Ikbkg	inhibitor of kappaB kinase gamma, transcript variant	NA	0.069555	4.920628	1.049393	0.39600037	0.695804
76829	Dok5	docking protein 5, transcript variant 1	NA	0.053957	5.290638	1.038109	0.39601363	0.695804
16997	Ltbp2	latent transforming growth factor beta binding protein	NA	-0.23526	0.367912	-1.17712	0.39602271	0.695804
319845	Bbs9	Bardet-Biedl syndrome 9 (human), transcript variant 4	NA	0.070254	4.398113	1.049901	0.39605046	0.695804
115488181	Gm52059	predicted gene, 52059	NA	0.272766	-0.06851	1.208122	0.39611316	0.695839
240120	Zfp119b	zinc finger protein 119b, transcript variant 1	NA	0.120729	2.576092	1.087284	0.39618732	0.695839
625121	Gm6556	predicted gene 6556	NA	0.162032	1.772644	1.118862	0.39619915	0.695839
226182	Taf5	TATA-box binding protein associated factor 5	NA	0.076877	4.222636	1.054733	0.39655751	0.696344
53416	Stk39	serine/threonine kinase 39, transcript variant X1	NA	-0.04902	5.851853	-1.03456	0.39661577	0.696344
105244557	Gm40150	predicted gene, 40150	NA	-0.21323	0.766381	-1.15928	0.39662787	0.696344
234371	Tmem161a	transmembrane protein 161A, transcript variant X1	NA	-0.06887	4.568604	-1.04889	0.39665763	0.696344
19415	Rasal1	RAS protein activator like 1 (GAP1 like), transcript va	NA	0.178431	1.709519	1.131652	0.39675671	0.696381
105245155	Gm40647	predicted gene, 40647	NA	0.065816	4.512767	1.046677	0.3967644	0.696381
26549	Itgb1bp2	integrin beta 1 binding protein 2	NA	-0.09328	3.200204	-1.0668	0.39690287	0.696549
13712	Elk1	ELK1, member of ETS oncogene family	NA	-0.06741	5.423698	-1.04784	0.39709716	0.696815
105372	Utp15	UTP15 small subunit processome component	NA	0.055518	5.306865	1.039232	0.39733575	0.697146
12651	Chkb	choline kinase beta, transcript variant 2	NA	0.072958	5.544278	1.051871	0.397394	0.697146
74411	Plpp6	phospholipid phosphatase 6	NA	0.071817	4.535737	1.051039	0.3974143	0.697146
241770	Rims4	regulating synaptic membrane exocytosis 4, transcrip	NA	-0.05154	5.960109	-1.03637	0.39757345	0.69735
19045	Ppp1ca	protein phosphatase 1 catalytic subunit alpha	NA	0.037049	7.85357	1.026013	0.39762478	0.697365
22273	Uqcrc1	ubiquinol-cytochrome c reductase core protein 1	NA	0.038413	7.016624	1.026984	0.39776719	0.697539
105243124	Gm15351	predicted gene 15351	NA	-0.19931	1.078139	-1.14815	0.39790586	0.697679
11975	Atp6v0a1	ATPase, H+ transporting, lysosomal V0 subunit A1, t	NA	-0.04009	7.824747	-1.02818	0.39794456	0.697679
102640043	Gm36210	predicted gene, 36210	NA	0.271693	-0.10073	1.207224	0.39797525	0.697679
210853	Zfp947	zinc finger protein 947	NA	0.20345	1.700929	1.151449	0.3980602	0.697752
20677	Sox4	SRY (sex determining region Y)-box 4	NA	0.041953	10.203	1.029507	0.3982622	0.698031
26362	Axl	AXL receptor tyrosine kinase, transcript variant X3	NA	-0.05935	4.858906	-1.042	0.39831943	0.69805
67865	Rgs10	regulator of G-protein signalling 10	NA	0.075165	3.873955	1.053481	0.39837151	0.69805
77877	6030458C11Rik	RIKEN cDNA 6030458C11 gene, transcript variant 3	NA	-0.05534	5.203438	-1.0391	0.39840138	0.69805
17965	Nbl1	NBL1, DAN family BMP antagonist, transcript variant	NA	0.083062	4.514776	1.059264	0.39854713	0.698222
210106	Tent4a	terminal nucleotidyltransferase 4A, transcript variant	NA	-0.04426	5.980486	-1.03116	0.39858573	0.698222
27393	Mrpl39	mitochondrial ribosomal protein L39, transcript varian	NA	0.071463	5.174888	1.050782	0.39885571	0.69861
110012	Tpgs1	tubulin polyglutamylase complex subunit 1	NA	0.108676	4.041055	1.078238	0.39900614	0.69861
56224	Tspan5	tetraspanin 5, transcript variant X3	NA	0.041095	7.020894	1.028895	0.3990228	0.69861
320701	Tafa4	TAFA chemokine like family member 4, transcript var	NA	0.180291	1.241136	1.133113	0.39903677	0.69861
102634055	Gm31734	predicted gene, 31734, transcript variant 3	NA	-0.27129	0.483908	-1.20689	0.39904442	0.69861
623273	Alms1-ps2	ALMS1, centrosome and basal body associated, pse	NA	0.194846	1.211385	1.144602	0.39921827	0.69861
14089	Fap	fibroblast activation protein	NA	-0.20707	1.257023	-1.15434	0.39925072	0.69861
108168987	Gm13205	predicted gene 13205	NA	0.195933	0.663902	1.145465	0.39926597	0.69861
75219	Dusp18	dual specificity phosphatase 18	NA	-0.05176	5.748761	-1.03653	0.39928462	0.69861
329628	Fat4	FAT atypical cadherin 4	NA	0.041641	6.949381	1.029284	0.39933343	0.69861
19223	Ptgis	prostaglandin I2 (prostacyclin) synthase	NA	-0.15353	1.736314	-1.11229	0.39933951	0.69861
56752	Aldh9a1	aldehyde dehydrogenase 9, subfamily A1	NA	0.057197	5.074779	1.040442	0.39935697	0.69861
70110	Ifi35	interferon-induced protein 35	NA	-0.23177	0.116663	-1.17427	0.39936542	0.69861
327987	Med13	mediator complex subunit 13	NA	0.047176	7.081419	1.03324	0.39945458	0.698691
12053	Bcl6	B cell leukemia/lymphoma 6, transcript variant X1	NA	-0.0873	3.374357	-1.06238	0.39957001	0.698731
100504641	1700101I11Rik	RIKEN cDNA 1700101I11 gene	NA	0.315374	-0.45183	1.244334	0.3995776	0.698731
226781	Slc30a10	solute carrier family 30, member 10	NA	0.059183	5.416153	1.041876	0.39960636	0.698731
54419	Cldn6	claudin 6	NA	-0.35511	-0.85772	-1.27908	0.39976387	0.698932
19252	Dusp1	dual specificity phosphatase 1	NA	-0.06995	4.843627	-1.04968	0.39985832	0.699022
15382	Hnrnpa1	heterogeneous nuclear ribonucleoprotein A1, transcri	NA	-0.04686	10.51026	-1.03302	0.39994308	0.699095
105242506	Gm38718	predicted gene, 38718	NA	0.296741	-0.49532	1.228366	0.40003572	0.69912
12916	Crem	cAMP responsive element modulator, transcript varia	NA	0.101295	3.342316	1.072736	0.40006263	0.69912
118567665	LOC118567665	uncharacterized LOC118567665	NA	-0.32841	-0.22446	-1.25563	0.40012265	0.69912
57373	Akip1	A kinase (PRKA) interacting protein 1	NA	0.137628	2.256213	1.100095	0.40017434	0.69912
17869	Myc	myelocytomatosis oncogene, transcript variant 1	NA	0.054621	5.097549	1.038587	0.40017942	0.69912

59002	Wrap73	WD repeat containing, antisense to Trp73	NA		-0.08089	4.46107	-1.05767	0.4002151	0.69912
102632164	Gm30311	predicted gene, 30311, transcript variant X2	NA		-0.28456	-0.12332	-1.21804	0.40027368	0.699122
208431	Shroom4	shroom family member 4, transcript variant 2	NA		0.10082	3.290998	1.072383	0.40030223	0.699122
231646	Myo1h	myosin 1H, transcript variant 2	NA		0.343347	-0.21176	1.268696	0.40039869	0.699146
16858	Lgals7	lectin, galactose binding, soluble 7	NA		-0.33879	1.809941	-1.26469	0.40040179	0.699146
21646	Tcte2	t-complex-associated testis expressed 2	NA		0.149428	1.518165	1.10913	0.4005018	0.699245
66612	Ormdl3	ORM1-like 3 (S. cerevisiae)	NA		-0.04477	6.290117	-1.03152	0.40055009	0.699254
56612	Pfdn5	prefoldin 5	NA		0.053703	6.747488	1.037926	0.4006942	0.699412
19889	Rp2	retinitis pigmentosa 2 homolog, transcript variant 1	NA		0.074052	4.356118	1.052669	0.40075772	0.699412
68755	Cgrrf1	cell growth regulator with ring finger domain 1, transc	NA		0.107206	2.568672	1.07714	0.40076951	0.699412
26395	Map2k1	mitogen-activated protein kinase kinase 1	NA		0.058058	5.352011	1.041063	0.40089439	0.699555
71375	Foxn3	forkhead box N3, transcript variant X13	NA		0.042423	6.646096	1.029842	0.4009425	0.699564
57340	Jph3	junctophilin 3	NA		-0.04576	6.402255	-1.03223	0.40107767	0.699725
20787	Srebf1	sterol regulatory element binding transcription factor '	NA		0.05812	5.222843	1.041108	0.40121816	0.699761
19264	Ptprc	protein tyrosine phosphatase, receptor type, C, transc	NA		-0.24893	0.817031	-1.18832	0.4012794	0.699761
11692	Gfer	growth factor, augmenter of liver regeneration, transc	NA		-0.06885	4.498047	-1.04888	0.40145687	0.699761
233733	Galnt18	polypeptide N-acetylgalactosaminyltransferase 18, tra	NA		-0.07977	3.601297	-1.05685	0.40147438	0.699761
13866	Erbb2	erb-b2 receptor tyrosine kinase 2	NA		-0.08219	3.739632	-1.05863	0.40148052	0.699761
66146	Maco1	macoilin 1, transcript variant 1	NA		0.037258	7.241973	1.026162	0.40149971	0.699761
76080	Ttpal	tocopherol (alpha) transfer protein-like, transcript vari	NA		0.050752	5.889613	1.035804	0.40150815	0.699761
105244124	Gm13404	predicted gene 13404, transcript variant X11	NA		0.200551	1.414864	1.149137	0.40158652	0.699761
22068	Trpc6	transient receptor potential cation channel, subfamily	NA		0.26319	0.071455	1.20013	0.40171081	0.699761
74365	Lonrf3	LON peptidase N-terminal domain and ring finger 3	NA		0.130014	2.754653	1.094304	0.40173064	0.699761
14284	Fosl2	fos-like antigen 2	NA		-0.07115	4.596841	-1.05056	0.40175657	0.699761
68565	Mrps18a	mitochondrial ribosomal protein S18A	NA		0.058047	4.741046	1.041056	0.40179042	0.699761
102639268	Gm13830	predicted gene 13830	NA		-0.21338	0.948452	-1.1594	0.40180017	0.699761
238871	Pde4d	phosphodiesterase 4D, cAMP specific, transcript vari	NA		0.044126	6.853941	1.031059	0.40180525	0.699761
544817	Arhgap27	Rho GTPase activating protein 27, transcript variant)	NA		0.108917	2.637517	1.078419	0.40185619	0.699761
68472	Tmem126b	transmembrane protein 126B	NA		-0.10443	3.15614	-1.07507	0.40186129	0.699761
115489886	LOC115489886	uncharacterized LOC115489886, transcript variant X	NA		0.155491	1.477378	1.113801	0.40186758	0.699761
209224	Enox2	ecto-NOX disulfide-thiol exchanger 2, transcript varia	NA		-0.09657	3.519829	-1.06923	0.40189564	0.699761
68021	Bphl	biphenyl hydrolase-like (serine hydrolase, breast epitl	NA		0.097222	3.697773	1.069712	0.40191545	0.699761
67885	Mtln	mitoregulin	NA		-0.12804	2.53772	-1.09281	0.40197214	0.699785
214345	Lrrc1	leucine rich repeat containing 1, transcript variant 2	NA		0.080173	3.974891	1.057145	0.40201806	0.69979
436100	Gm21814	predicted gene, 21814	NA		0.266168	-0.04084	1.202609	0.40209886	0.699856
68828	Sync	syncoilin, transcript variant X2	NA		-0.20445	1.03304	-1.15225	0.40227016	0.700025
381072	Abca17	ATP-binding cassette, sub-family A (ABC1), member	NA		-0.24794	0.067963	-1.18751	0.40228209	0.700025
66595	Aste1	asteroid homolog 1, transcript variant 4	NA		-0.10637	2.869423	-1.07652	0.40232865	0.700031
22756	Zfp94	zinc finger protein 94, transcript variant 1	NA		-0.08542	3.357863	-1.061	0.40252005	0.700289
22384	Eif4h	eukaryotic translation initiation factor 4H, transcript va	NA		-0.0382	8.137365	-1.02683	0.40260387	0.70036
14466	Gba	glucosidase, beta, acid, transcript variant 2	NA		-0.06101	4.650611	-1.0432	0.40267233	0.7004
117198	Ivns1abp	influenza virus NS1A binding protein, transcript variar	NA		0.038532	8.37525	1.027068	0.40271278	0.7004
57329	Otor	otoraplin	NA		-0.27306	0.085542	-1.20837	0.40286161	0.700513
67790	Rab39b	RAB39B, member RAS oncogene family	NA		0.055846	5.240926	1.039468	0.40286361	0.700513
57339	Jph1	junctophilin 1	NA		-0.11609	2.335812	-1.08379	0.40294638	0.70053
57390	Psors1c2	psoriasis susceptibility 1 candidate 2 (human), transc	NA		0.351422	0.214383	1.275817	0.40298491	0.70053
68922	Dnaic1	dynein, axonemal, intermediate chain 1, transcript va	NA		-0.20412	0.573723	-1.15198	0.40306597	0.70053
20852	Stat6	signal transducer and activator of transcription 6	NA		-0.10309	2.965978	-1.07407	0.40308101	0.70053
100039138	Gm2061	predicted gene 2061	NA		0.099376	2.869385	1.07131	0.40311758	0.70053
227615	Tmem203	transmembrane protein 203	NA		-0.14073	1.853802	-1.10246	0.40314671	0.70053
74455	Nsun6	NOL1/NOP2/Sun domain family member 6, transcript	NA		0.06922	4.279733	1.049149	0.40317504	0.70053
225363	Etf1	eukaryotic translation termination factor 1	NA		0.042797	6.973895	1.030109	0.40329193	0.700635
11481	Acvr2b	activin receptor IIB, transcript variant X6	NA		-0.0407	6.803656	-1.02861	0.40339041	0.700635
58244	Stx6	syntaxin 6	NA		0.045042	6.535318	1.031713	0.40341076	0.700635
78829	Tsc22d4	TSC22 domain family, member 4, transcript variant 2	NA		-0.05634	4.952359	-1.03983	0.40350347	0.700635
28071	Twistnb	twist basic helix-loop-helix transcription factor 1 neigh	NA		0.070391	4.681892	1.050001	0.4035184	0.700635
328287	Gm20554	predicted gene, 20554	NA		-0.1499	1.677171	-1.10949	0.40353401	0.700635
65102	Nif3l1	Ngg1 interacting factor 3-like 1 (S. pombe)	NA		0.07604	5.186328	1.054121	0.40353707	0.700635
66200	Commd6	COMM domain containing 6, transcript variant 1	NA		-0.08293	4.136996	-1.05917	0.40362205	0.700708
227394	Slco4c1	solute carrier organic anion transporter family, memb	NA		0.279579	-0.01218	1.213841	0.40369914	0.700767
66827	Ttc1	tetratricopeptide repeat domain 1	NA		0.065929	5.132149	1.046759	0.40380649	0.700879
68598	Dnajc8	DnaJ heat shock protein family (Hsp40) member C8	NA		0.054382	6.628867	1.038414	0.40392634	0.701012
18572	Pdcd11	programmed cell death 11	NA		-0.05955	5.318636	-1.04214	0.40420937	0.701428
170742	Sertad3	SERTA domain containing 3	NA		-0.13784	2.036889	-1.10026	0.40437001	0.701554
66306	Fam53c	family with sequence similarity 53, member C, transcr	NA		-0.04571	6.134417	-1.03219	0.40441121	0.701554
71514	Sfpq	splicing factor proline/glutamine rich (polypyrimidine t	NA		-0.04701	9.550361	-1.03312	0.40445961	0.701554
118567717	LOC118567717	uncharacterized LOC118567717	NA		-0.32417	1.2908	-1.25195	0.40451633	0.701554
70552	Lrrc56	leucine rich repeat containing 56, transcript variant 2	NA		-0.09259	3.474622	-1.06628	0.40458914	0.701554

110960	Tars	threonyl-tRNA synthetase	NA	0.04467	5.921565	1.031447	0.4046012	0.701554
69675	Pxdn	peroxidasin, transcript variant X3	NA	-0.04519	7.032262	-1.03182	0.40461336	0.701554
114863	Plpbp	pyridoxal phosphate binding protein, transcript varian	NA	-0.05749	5.20463	-1.04065	0.40462678	0.701554
217364	Engase	endo-beta-N-acetylglucosaminidase, transcript variar	NA	-0.09631	2.87499	-1.06903	0.40477825	0.701742
353190	Edc3	enhancer of mRNA decapping 3	NA	-0.0516	5.506961	-1.03642	0.40504331	0.701993
78703	Zfp972	zinc finger protein 972	NA	-0.30846	-0.11968	-1.23839	0.40505993	0.701993
14261	Fmo1	flavin containing monooxygenase 1, transcript variant	NA	0.069174	4.490923	1.049116	0.40507774	0.701993
17919	Myo5b	myosin VB, transcript variant X4	NA	0.098101	3.279572	1.070364	0.40509558	0.701993
105245071	Gm40573	predicted gene, 40573	NA	-0.16756	1.528774	-1.12316	0.40514257	0.702
66671	Ccnh	cyclin H, transcript variant 1	NA	-0.05511	5.251374	-1.03894	0.40526269	0.702133
18399	Slc22a6	solute carrier family 22 (organic anion transporter), m	NA	0.086051	3.935691	1.061461	0.40531263	0.702145
56738	Mocs1	molybdenum cofactor synthesis 1, transcript variant 2	NA	-0.09451	3.18335	-1.0677	0.40548867	0.702375
244682	Cntn5	contactin 5, transcript variant X26	NA	-0.04967	6.032701	-1.03503	0.40558322	0.702464
75731	Idnk	idnK gluconokinase homolog (E. coli), transcript varia	NA	0.069572	4.534179	1.049405	0.40568447	0.702537
74080	Nmnat3	nicotinamide nucleotide adenylyltransferase 3, transc	NA	0.264337	0.108036	1.201084	0.40575838	0.702537
66752	4933404O12Rik	RIKEN cDNA 4933404O12 gene	NA	-0.10315	3.508256	-1.07412	0.40576945	0.702537
100040769	Gm15506	predicted gene 15506	NA	0.131164	2.18079	1.095177	0.40581276	0.702537
102334	Ankrd10	ankyrin repeat domain 10, transcript variant 1	NA	0.0452	6.875035	1.031826	0.405841	0.702537
20963	Syk	spleen tyrosine kinase, transcript variant 1	NA	-0.11377	2.641487	-1.08205	0.40597623	0.702538
68215	Fam98b	family with sequence similarity 98, member B	NA	-0.04678	6.314788	-1.03295	0.40599641	0.702538
18088	Nkx2-2	NK2 homeobox 2, transcript variant 1	NA	-0.10792	3.331342	-1.07767	0.4060334	0.702538
67296	Socs4	suppressor of cytokine signaling 4	NA	-0.05495	5.241609	-1.03882	0.40604489	0.702538
104458	Rars	arginyl-tRNA synthetase	NA	-0.04974	5.982788	-1.03508	0.40605784	0.702538
100502732	Gm19343	predicted gene, 19343	NA	-0.16205	2.299032	-1.11887	0.40619865	0.702707
66942	Ddx18	DEAD box helicase 18	NA	-0.05037	5.36326	-1.03553	0.40654822	0.703237
19647	Rbbp6	retinoblastoma binding protein 6, ubiquitin ligase, trar	NA	0.052475	7.285019	1.037042	0.4067083	0.703401
21855	Timm17b	translocase of inner mitochondrial membrane 17b, tra	NA	-0.06114	4.915252	-1.04329	0.40677639	0.703401
232201	Arhgap25	Rho GTPase activating protein 25, transcript variant 2	NA	0.187577	1.25471	1.13885	0.40680965	0.703401
18792	Plau	plasminogen activator, urokinase	NA	-0.20396	1.07546	-1.15185	0.40686905	0.703401
68463	Mrpl14	mitochondrial ribosomal protein L14	NA	-0.09159	3.806995	-1.06554	0.40689367	0.703401
70451	Dhrs13	dehydrogenase/reductase (SDR family) member 13	NA	0.063037	4.665506	1.044663	0.40690205	0.703401
244653	Hydin	HYDIN, axonemal central pair apparatus protein, tran	NA	-0.18654	1.108052	-1.13803	0.40696152	0.703429
69256	Zfp397	zinc finger protein 397	NA	0.053108	6.087863	1.037497	0.40703145	0.703467
118567851	LOC118567851	uncharacterized LOC118567851	NA	0.290969	0.215575	1.223462	0.40707006	0.703467
69656	Pir	pirin, transcript variant 1	NA	0.203384	1.099972	1.151396	0.40716947	0.703552
317758	Gimap9	GTPase, IMAP family member 9, transcript variant X2	NA	0.234867	0.299289	1.176798	0.4072098	0.703552
13666	Eif2ak3	eukaryotic translation initiation factor 2 alpha kinase 3	NA	0.05446	5.03426	1.03847	0.40726324	0.703552
105244929	Gm40451	predicted gene, 40451, transcript variant X1	NA	0.238052	0.202394	1.179399	0.40734073	0.703552
210293	Dock10	dedicator of cytokinesis 10, transcript variant X26	NA	0.11641	3.086617	1.084034	0.40737028	0.703552
218581	Depdc1b	DEP domain containing 1B, transcript variant X1	NA	-0.1393	2.001444	-1.10137	0.40744437	0.703552
380714	Rph3al	rabphilin 3A-like (without C2 domains), transcript vari	NA	0.147881	1.582908	1.107941	0.40745992	0.703552
56404	Trip4	thyroid hormone receptor interactor 4, transcript varia	NA	-0.04844	5.384505	-1.03415	0.40746515	0.703552
30049	Scd3	stearoyl-coenzyme A desaturase 3	NA	-0.18755	1.5902	-1.13883	0.40757718	0.703613
103742	Mien1	migration and invasion enhancer 1	NA	-0.05086	5.262451	-1.03588	0.40762005	0.703613
224640	Lemd2	LEM domain containing 2	NA	-0.04887	5.575917	-1.03446	0.40768173	0.703613
13885	Esd	esterase D/formylglutathione hydrolase, transcript va	NA	0.050827	6.304073	1.035858	0.40768523	0.703613
66277	Klf15	Kruppel-like factor 15, transcript variant 1	NA	0.081526	3.608261	1.058137	0.40771664	0.703613
53382	Txnl1	thioredoxin-like 1	NA	0.041017	6.584181	1.028839	0.40777973	0.703647
66128	Mrps36	mitochondrial ribosomal protein S36, transcript variar	NA	0.071646	4.260606	1.050915	0.40801293	0.703901
320696	Ccdc158	coiled-coil domain containing 158, transcript variant 4	NA	-0.25287	0.248067	-1.19158	0.40807227	0.703901
245446	Slitrk4	SLIT and NTRK-like family, member 4, transcript vari	NA	0.088084	4.324094	1.062958	0.40808639	0.703901
74648	S100pbp	S100P binding protein, transcript variant 9	NA	0.047008	6.039471	1.03312	0.40809983	0.703901
76937	2810429I04Rik	RIKEN cDNA 2810429I04 gene, transcript variant 2	NA	0.221739	0.861032	1.166138	0.40829231	0.704064
171170	Mbnl3	muscleblind like splicing factor 3, transcript variant X8	NA	-0.13262	1.880922	-1.09628	0.40831395	0.704064
382543	Ankfn1	ankyrin-repeat and fibronectin type III domain contain	NA	0.260839	1.038667	1.198175	0.40832433	0.704064
18976	Pomc	pro-opiomelanocortin-alpha, transcript variant 4	NA	0.280313	1.224447	1.214458	0.40871706	0.704536
241950	Bbs12	Bardet-Biedl syndrome 12 (human), transcript variant	NA	0.091417	3.098753	1.065416	0.40871724	0.704536
102634756	Gm32262	predicted gene, 32262, transcript variant X2	NA	0.277992	-0.25741	1.212506	0.40872778	0.704536
93885	Pcdhb14	protocadherin beta 14	NA	-0.15842	1.553078	-1.11607	0.40891545	0.70478
71901	Fam219a	family with sequence similarity 219, member A, trans	NA	0.046977	7.091897	1.033098	0.40895613	0.70478
236576	Spry3	sprouty RTK signaling antagonist 3	NA	0.109366	3.046291	1.078754	0.4090434	0.704856
105244210	Gm39859	predicted gene, 39859, transcript variant 1	NA	0.244754	-0.0321	1.184891	0.40919718	0.705046
74319	Mettl23	methyltransferase like 23	NA	0.073655	4.326897	1.052379	0.40924903	0.705061
115490394	Gm52890	predicted gene, 52890	NA	-0.2668	0.80799	-1.20314	0.409334	0.705133
226026	Smc5	structural maintenance of chromosomes 5, transcript	NA	0.050595	5.787582	1.035692	0.40937733	0.705133
75516	Ttc32	tetratricopeptide repeat domain 32	NA	-0.18548	1.572428	-1.1372	0.40950213	0.705168
76166	Cplane2	ciliogenesis and planar polarity effector 2	NA	-0.36398	-0.4042	-1.28697	0.4095219	0.705168
378937	Lrrc24	leucine rich repeat containing 24	NA	-0.10717	2.840242	-1.07711	0.40952798	0.705168

68133	Gcsh	glycine cleavage system protein H (aminomethyl carr	NA		0.060276	5.171127	1.042665	0.40960121	0.70522
54217	Rpl36	ribosomal protein L36, transcript variant X1	NA		0.137033	7.07002	1.099641	0.40965934	0.705245
68285	C630043F03Rik	RIKEN cDNA C630043F03 gene	NA		0.082812	4.129096	1.05908	0.40988585	0.70556
68011	Snrpg	small nuclear ribonucleoprotein polypeptide G	NA		-0.04327	6.022441	-1.03045	0.40997561	0.70564
107239	Carns1	carnosine synthase 1, transcript variant X4	NA		-0.13558	1.966568	-1.09853	0.41014027	0.705775
68736	Tyw5	tRNA-yW synthesizing protein 5, transcript variant 3	NA		-0.09114	3.325343	-1.06521	0.41027969	0.705775
16002	Igf2	insulin-like growth factor 2, transcript variant 3	NA		0.042302	9.024419	1.029756	0.41035697	0.705775
14169	Fgf14	fibroblast growth factor 14, transcript variant X1	NA		0.068245	5.404689	1.048441	0.41035954	0.705775
64707	Suv39h2	suppressor of variegation 3-9 2, transcript variant X1	NA		-0.0868	3.837137	-1.06201	0.41043502	0.705775
68846	Rnf208	ring finger protein 208	NA		0.052583	5.616876	1.03712	0.4104618	0.705775
77057	Ston1	stonin 1, transcript variant X1	NA		-0.08265	3.995402	-1.05896	0.41048866	0.705775
231858	Radil	Ras association and DIL domains, transcript variant X	NA		-0.07415	3.969554	-1.05274	0.4105525	0.705775
235386	Hykk	hydroxylysine kinase 1	NA		-0.14973	1.920463	-1.10936	0.41055309	0.705775
105242443	Gm38708	predicted gene, 38708, transcript variant X4	NA		0.204046	0.812989	1.151924	0.41059124	0.705775
234479	Gm4890	predicted gene 4890, transcript variant 1	NA		0.251417	0.020843	1.190375	0.4105934	0.705775
56036	Ccnl2	cyclin L2, transcript variant X4	NA		-0.04474	7.29707	-1.0315	0.41063099	0.705775
381101	Dnph1	2'-deoxynucleoside 5'-phosphate N-hydrolase 1	NA		-0.13617	2.68643	-1.09899	0.41064275	0.705775
100604	Lrrc8c	leucine rich repeat containing 8 family, member C, tra	NA		-0.06009	4.977458	-1.04253	0.41071526	0.705775
67463	Poc5	POC5 centriolar protein, transcript variant X1	NA		-0.04971	5.43486	-1.03506	0.41074179	0.705775
14211	Smc2	structural maintenance of chromosomes 2, transcript	NA		-0.05466	5.641093	-1.03861	0.41076268	0.705775
18521	Pcbp2	poly(rC) binding protein 2, transcript variant X25	NA		-0.04213	8.80087	-1.02964	0.41079123	0.705775
436230	BC065397	cDNA sequence BC065397	NA		-0.13474	2.059648	-1.09789	0.41092501	0.70593
231600	Chfr	checkpoint with forkhead and ring finger domains, tra	NA		-0.04155	6.246066	-1.02922	0.41106539	0.706097
331188	Zfp781	zinc finger protein 781, transcript variant X1	NA		-0.11035	3.778867	-1.07949	0.41119971	0.706192
227746	Rabepk	Rab9 effector protein with kelch motifs, transcript vari	NA		0.088729	3.486277	1.063433	0.41123902	0.706192
20022	Polr2j	polymerase (RNA) II (DNA directed) polypeptide J	NA		0.068791	4.948749	1.048837	0.41125122	0.706192
58187	Cldn10	claudin 10, transcript variant b	NA		0.248634	0.258912	1.188082	0.41138024	0.706339
319727	A330035P11Rik	RIKEN cDNA A330035P11 gene	NA		0.253932	0.448452	1.192453	0.4115843	0.706615
73346	1700039I01Rik	RIKEN cDNA 1700039I01 gene, transcript variant X1	NA		-0.28068	-0.21967	-1.21476	0.41167823	0.706697
68310	Zmym1	zinc finger, MYM domain containing 1, transcript varia	NA		-0.0677	4.405815	-1.04804	0.41171887	0.706697
22342	Lin7b	lin-7 homolog B (C. elegans)	NA		-0.16626	1.311355	-1.12215	0.4118953	0.706925
75129	4930524J08Rik	RIKEN cDNA 4930524J08 gene	NA		-0.27416	-0.16269	-1.20929	0.41200476	0.706969
13401	Dmwd	dystrophia myotonica-containing WD repeat motif, tra	NA		-0.04818	6.847291	-1.03396	0.41201151	0.706969
67269	Agtpbp1	ATP/GTP binding protein 1, transcript variant 5	NA		0.050825	6.918268	1.035857	0.41205087	0.706969
76681	Trim12a	tripartite motif-containing 12A, transcript variant X7	NA		0.150482	1.532203	1.10994	0.41213839	0.707044
98711	Rdh10	retinol dehydrogenase 10 (all-trans)	NA		0.083754	4.01937	1.059772	0.41218244	0.707045
16669	Krt19	keratin 19, transcript variant 1	NA		-0.26233	1.552296	-1.19941	0.41228127	0.70714
53324	Nptx2	neuronal pentraxin 2	NA		0.094714	3.748116	1.067853	0.41243708	0.707232
272359	Irf2bp1	interferon regulatory factor 2 binding protein 1	NA		-0.04685	5.956739	-1.03301	0.41244588	0.707232
218544	Sgtb	small glutamine-rich tetratricopeptide repeat (TPR)-co	NA		0.06144	4.986437	1.043507	0.41246532	0.707232
68576	Lamtor5	late endosomal/lysosomal adaptor, MAPK and MTOF	NA		-0.05179	5.03813	-1.03655	0.41255816	0.707267
94091	Trim11	tripartite motif-containing 11, transcript variant X4	NA		-0.07778	4.418286	-1.05539	0.41257217	0.707267
99371	Arfgef2	ADP-ribosylation factor guanine nucleotide-exchange	NA		0.063923	4.849656	1.045304	0.41268932	0.707359
246133	Kcne2	potassium voltage-gated channel, Isk-related subfam	NA		-0.10772	3.889985	-1.07752	0.41287112	0.707359
69274	Ctdspl	CTD (carboxy-terminal domain, RNA polymerase II, p	NA		-0.06109	4.692759	-1.04325	0.41288266	0.707359
52013	R3hcc1l	R3H domain and coiled-coil containing 1 like	NA		-0.07445	4.100943	-1.05296	0.41288457	0.707359
19305	Pex5	peroxisomal biogenesis factor 5, transcript variant 6	NA		-0.04301	6.326884	-1.03026	0.41290574	0.707359
18784	Pla2g5	phospholipase A2, group V, transcript variant X2	NA		-0.28785	-0.35244	-1.22082	0.41291695	0.707359
78689	Naa35	N(alpha)-acetyltransferase 35, NatC auxiliary subunit	NA		-0.0409	6.496149	-1.02876	0.41293024	0.707359
230082	Nol6	nucleolar protein family 6 (RNA-associated)	NA		-0.04161	6.380422	-1.02926	0.41304622	0.707418
108995	Tbc1d10c	TBC1 domain family, member 10c, transcript variant)	NA		0.299055	-0.06291	1.230338	0.4130517	0.707418
217887	Clba1	clathrin binding box of aftiphilin containing 1	NA		0.103045	3.40554	1.074038	0.41319039	0.707529
108167790	Gm46207	predicted gene, 46207	NA		-0.28652	1.540355	-1.2197	0.41320706	0.707529
108686	Ccdc88a	coiled coil domain containing 88A, transcript variant 3	NA		0.041262	7.645551	1.029014	0.4132469	0.707529
18113	Nnmt	nicotinamide N-methyltransferase, transcript variant X	NA		-0.28206	-0.24469	-1.21593	0.41336583	0.707658
76737	Creld2	cysteine-rich with EGF-like domains 2	NA		-0.07365	4.520701	-1.05237	0.41352876	0.707862
230863	Sh2d5	SH2 domain containing 5	NA		-0.06331	4.21698	-1.04486	0.41370707	0.708024
20464	Sim1	single-minded family bHLH transcription factor 1, tran	NA		-0.15836	2.297164	-1.11602	0.41373111	0.708024
100038347	Fam174b	family with sequence similarity 174, member B	NA		-0.05631	4.7964	-1.03981	0.41379986	0.708024
100043213	5330434G04Rik	RIKEN cDNA 5330434G04 gene	NA		0.13647	2.440678	1.099212	0.41380004	0.708024
170743	Tlr7	toll-like receptor 7, transcript variant X1	NA		-0.22424	0.452072	-1.16816	0.41384078	0.708024
56405	Dusp14	dual specificity phosphatase 14	NA		-0.11914	2.868264	-1.08609	0.41392573	0.708071
66834	Acot13	acyl-CoA thioesterase 13	NA		0.091303	3.607594	1.065332	0.41396701	0.708071
75320	Etnk1	ethanolamine kinase 1	NA		0.065712	7.422431	1.046601	0.41405751	0.708071
73287	Cabcoco1	ciliary associated calcium binding coiled-coil 1	NA		0.246556	-0.0318	1.186371	0.41405819	0.708071
68212	Tmbim4	transmembrane BAX inhibitor motif containing 4	NA		0.072744	3.86752	1.051715	0.41409139	0.708071
102631739	Gm30003	predicted gene, 30003, transcript variant X5	NA		0.259486	0.078981	1.197052	0.41412948	0.708071
12490	Cd34	CD34 antigen, transcript variant 2	NA		-0.06845	5.104657	-1.04859	0.414396	0.708355

231670	Fbxo21	F-box protein 21, transcript variant 2	NA	-0.03581	7.739024	-1.02513	0.41444622	0.708355
320538	Ubn2	ubinuclein 2	NA	-0.05382	6.582062	-1.03801	0.41446693	0.708355
115486531	Gm35842	predicted gene, 35842	NA	-0.16884	1.090218	-1.12415	0.41456369	0.708355
231386	Ythdc1	YTH domain containing 1, transcript variant 1	NA	0.050104	6.856689	1.035339	0.41456429	0.708355
18973	Pole	polymerase (DNA directed), epsilon	NA	0.064638	4.327261	1.045823	0.4146162	0.708355
16826	Ldb2	LIM domain binding 2, transcript variant 1	NA	0.049872	6.037469	1.035173	0.41462263	0.708355
71567	Mcm9	minichromosome maintenance 9 homologous recomb	NA	-0.10318	3.145149	-1.07414	0.41464354	0.708355
13350	Dgat1	diacylglycerol O-acyltransferase 1, transcript variant X	NA	-0.07503	4.806979	-1.05338	0.41476596	0.708461
64379	Irx6	Iroquois homeobox 6, transcript variant 1	NA	0.143369	1.812986	1.104482	0.41479296	0.708461
76454	Fbxo31	F-box protein 31	NA	-0.04739	5.791403	-1.03339	0.41499595	0.708591
14187	Akr1b8	aldo-keto reductase family 1, member B8	NA	0.225344	1.14706	1.169056	0.41501727	0.708591
68190	Dubr	Dppa2 upstream binding RNA	NA	-0.1341	2.057952	-1.09741	0.41508035	0.708591
100415787	Tomm6os	translocase of outer mitochondrial membrane 6, oppo	NA	0.162484	1.100477	1.119213	0.41509176	0.708591
11840	Arf1	ADP-ribosylation factor 1, transcript variant 2	NA	0.034661	8.539177	1.024316	0.41515641	0.708591
20460	Stil	Scl/Tal1 interrupting locus, transcript variant 4	NA	0.080057	3.43183	1.05706	0.41517137	0.708591
66930	Fank1	fibronectin type 3 and ankyrin repeat domains 1, trans	NA	0.161325	1.355903	1.118314	0.41517365	0.708591
110173	Manba	mannosidase, beta A, lysosomal	NA	0.105139	2.963664	1.075598	0.41527068	0.708682
14634	Gli3	GLI-Kruppel family member GLI3, transcript variant X	NA	0.058408	4.951798	1.041316	0.41547654	0.708921
17978	Ncoa2	nuclear receptor coactivator 2, transcript variant a	NA	0.051673	6.276962	1.036466	0.41549748	0.708921
54124	Cks1b	CDC28 protein kinase 1b	NA	0.067984	4.722578	1.048251	0.41563593	0.709019
68365	Rab14	RAB14, member RAS oncogene family	NA	0.036905	7.981788	1.02591	0.41574987	0.709019
11546	Parp2	poly (ADP-ribose) polymerase family, member 2	NA	0.059176	5.08571	1.041871	0.41577493	0.709019
11305	Abca2	ATP-binding cassette, sub-family A (ABC1), member	NA	0.046942	6.457172	1.033073	0.41577732	0.709019
228983	Osbpl2	oxysterol binding protein-like 2, transcript variant X1	NA	0.044613	6.289835	1.031406	0.41578644	0.709019
14707	Gng5	guanine nucleotide binding protein (G protein), gamm	NA	-0.07319	5.11755	-1.05204	0.41581692	0.709019
102636700	Gm19531	predicted gene, 19531	NA	0.230135	0.261939	1.172945	0.41595587	0.709134
110521	Hivep1	human immunodeficiency virus type I enhancer bindi	NA	0.04918	6.318001	1.034676	0.41597143	0.709134
71764	C2cd2l	C2 calcium-dependent domain containing 2-like, trans	NA	-0.049	5.404048	-1.03455	0.41607173	0.709231
98193	Dcaf8	DDB1 and CUL4 associated factor 8, transcript varian	NA	-0.03763	6.76028	-1.02643	0.4161185	0.709236
242521	Klhl9	kelch-like 9	NA	-0.05242	7.400707	-1.037	0.41616754	0.709246
67619	Nob1	NIN1/RPN12 binding protein 1 homolog	NA	0.052411	5.092872	1.036996	0.41650056	0.709573
30853	Mlf2	myeloid leukemia factor 2, transcript variant 2	NA	-0.03951	8.141178	-1.02776	0.41650833	0.709573
12643	Chad	chondroadherin	NA	-0.37818	-1.22586	-1.2997	0.41657617	0.709573
237542	Osbpl8	oxysterol binding protein-like 8, transcript variant X1	NA	0.064697	6.440875	1.045865	0.4166184	0.709573
16398	Itga2	integrin alpha 2	NA	-0.12282	2.390344	-1.08886	0.41661853	0.709573
240121	Fsd1	fibronectin type 3 and SPRY domain-containing prote	NA	-0.04293	5.806092	-1.0302	0.41662125	0.709573
72465	Zfp131	zinc finger protein 131, transcript variant 5	NA	-0.04601	5.796407	-1.03241	0.41678651	0.70974
545902	Ptprh	protein tyrosine phosphatase, receptor type, H, transc	NA	0.165718	1.075216	1.121724	0.41680639	0.70974
102640166	Gm36299	predicted gene, 36299	NA	-0.23267	0.333595	-1.17501	0.41688825	0.709805
70882	Armc3	armadillo repeat containing 3, transcript variant 3	NA	-0.17771	0.990597	-1.13109	0.41704166	0.709946
230073	Ddx58	DEAD/H box helicase 58	NA	0.132356	2.081284	1.096082	0.41705852	0.709946
19060	Ppp5c	protein phosphatase 5, catalytic subunit	NA	0.045902	6.330661	1.032328	0.41714748	0.710023
102631905	Gm30122	predicted gene, 30122	NA	-0.26354	0.158477	-1.20042	0.41729593	0.71015
74477	4933427D14Rik	RIKEN cDNA 4933427D14 gene, transcript variant X:	NA	-0.04712	5.837188	-1.0332	0.41730935	0.71015
21922	Clec3b	C-type lectin domain family 3, member b	NA	0.189474	1.49421	1.140347	0.41745889	0.710331
15896	Icam2	intercellular adhesion molecule 2, transcript variant X	NA	0.120608	2.09279	1.087193	0.41756996	0.710396
56457	Clptm1	cleft lip and palate associated transmembrane protein	NA	-0.03953	6.914995	-1.02778	0.41758451	0.710396
18970	Polb	polymerase (DNA directed), beta, transcript variant X	NA	-0.04197	6.101375	-1.02952	0.41785078	0.710676
70397	Tmem70	transmembrane protein 70, transcript variant 1	NA	0.049776	5.156154	1.035104	0.4179032	0.710676
217194	Klhl11	kelch-like 11, transcript variant 2	NA	0.058998	5.678994	1.041742	0.41791664	0.710676
74769	Pik3cb	phosphatidylinositol-4,5-bisphosphate 3-kinase cataly	NA	0.06712	4.509733	1.047623	0.41803953	0.710676
22255	Uncx	UNC homeobox	NA	-0.08712	3.537422	-1.06225	0.41804103	0.710676
214137	Arhgap29	Rho GTPase activating protein 29, transcript variant X	NA	0.064401	5.377959	1.045651	0.41804229	0.710676
327655	Ppip5k1	diphosphoinositol pentakisphosphate kinase 1	NA	-0.07873	4.770408	-1.05609	0.41805478	0.710676
65256	Asb2	ankyrin repeat and SOCS box-containing 2, transcrip	NA	-0.32615	0.341394	-1.25367	0.41816601	0.71079
68035	Rbm42	RNA binding motif protein 42	NA	0.045086	5.939171	1.031744	0.41821962	0.710807
21766	Tex261	testis expressed gene 261, transcript variant X1	NA	-0.06681	5.47237	-1.0474	0.41831499	0.710873
72084	Pigx	phosphatidylinositol glycan anchor biosynthesis, clas	NA	0.0578	4.855746	1.040878	0.41834569	0.710873
70118	Srrd	SRR1 domain containing, transcript variant 4	NA	0.075728	3.806101	1.053893	0.41843014	0.710942
110078	Pygb	brain glycogen phosphorylase	NA	-0.03657	7.103711	-1.02567	0.41861746	0.711166
12310	Calca	calcitonin/calcitonin-related polypeptide, alpha, transc	NA	0.219642	0.628702	1.164445	0.41865915	0.711166
23947	Mid2	midline 2, transcript variant 3	NA	0.087623	4.545667	1.062618	0.41869311	0.711166
118568490	LOC118568490	uncharacterized LOC118568490	NA	0.846585	-0.14849	1.79824	0.41885082	0.711302
105014	Rdh14	retinol dehydrogenase 14 (all-trans and 9-cis)	NA	0.064295	4.221774	1.045574	0.41886059	0.711302
17925	Myo9b	myosin IXb, transcript variant X23	NA	-0.04623	6.362247	-1.03256	0.41895967	0.711396
72772	Rint1	RAD50 interactor 1, transcript variant X2	NA	-0.07294	4.831191	-1.05186	0.41900959	0.711407
69017	Prrt2	proline-rich transmembrane protein 2, transcript varia	NA	0.045887	6.264414	1.032318	0.41926806	0.711545
56527	Mast1	microtubule associated serine/threonine kinase 1, tra	NA	-0.03947	7.421129	-1.02773	0.41927654	0.711545

320692	9430037G07Rik	RIKEN cDNA 9430037G07 gene	NA	0.226076	0.311437	1.16965	0.41929239	0.711545
56347	Eif3c	eukaryotic translation initiation factor 3, subunit C	NA	0.039235	7.862593	1.027569	0.41930703	0.711545
223473	Nipal2	NIPA-like domain containing 2	NA	0.091706	3.157463	1.065629	0.41930936	0.711545
71934	Car13	carbonic anhydrase 13	NA	0.177403	0.948379	1.130846	0.41938791	0.711604
19204	Ptafr	platelet-activating factor receptor	NA	-0.28589	-0.17062	-1.21916	0.41943439	0.711608
64436	Inpp5e	inositol polyphosphate-5-phosphatase E, transcript va	NA	-0.05684	5.112522	-1.04018	0.41964714	0.711823
68879	Prpf6	pre-mRNA splicing factor 6	NA	-0.04611	6.354462	-1.03247	0.41964812	0.711823
16180	Il1rap	interleukin 1 receptor accessory protein, transcript va	NA	0.128405	3.472282	1.093085	0.41979781	0.711936
217893	Pacs2	phosphofurin acidic cluster sorting protein 2, transcrip	NA	0.038411	7.513678	1.026982	0.41980228	0.711936
68075	Lurap1	leucine rich adaptor protein 1	NA	0.244424	-0.08555	1.184619	0.41984834	0.71194
232969	Zfp428	zinc finger protein 428, transcript variant 3	NA	-0.04949	6.115164	-1.0349	0.41996029	0.712055
27277	Golga5	golgi autoantigen, golgin subfamily a, 5, transcript var	NA	0.057916	5.035108	1.040961	0.42013682	0.71228
93760	Arid1a	AT rich interactive domain 1A (SWI-like), transcript va	NA	-0.04028	8.167507	-1.02831	0.4202095	0.712329
338362	Ust	uronyl-2-sulfotransferase	NA	-0.07635	3.8819	-1.05435	0.42032692	0.712339
20713	Serpini1	serine (or cysteine) peptidase inhibitor, clade I, memb	NA	0.044224	7.154449	1.031129	0.42033132	0.712339
68039	Nmb	neuromedin B, transcript variant 2	NA	0.183413	1.203071	1.135567	0.42034678	0.712339
17222	Anapc1	anaphase promoting complex subunit 1, transcript va	NA	0.046061	7.009139	1.032442	0.42040232	0.712359
353282	Sfmbt2	Scm-like with four mbt domains 2, transcript variant 4	NA	0.124231	2.878611	1.089927	0.42054594	0.712446
231871	Daglb	diacylglycerol lipase, beta, transcript variant X2	NA	-0.06571	4.358748	-1.0466	0.42060306	0.712446
235402	Lingo1	leucine rich repeat and Ig domain containing 1, transc	NA	-0.04356	6.908869	-1.03066	0.42063649	0.712446
668173	Pex10	peroxisomal biogenesis factor 10	NA	-0.09917	2.902728	-1.07116	0.42065984	0.712446
70394	Kptn	kaptin	NA	-0.07316	3.980854	-1.05202	0.4207699	0.712446
69742	Tm2d2	TM2 domain containing 2	NA	0.055277	5.388812	1.039059	0.42077428	0.712446
68038	Chid1	chitinase domain containing 1, transcript variant 1	NA	-0.07357	4.985771	-1.05232	0.42079787	0.712446
240832	Tor1aip2	torsin A interacting protein 2, transcript variant 2	NA	-0.04189	6.365605	-1.02946	0.42080349	0.712446
16668	Krt18	keratin 18	NA	0.127968	2.240349	1.092754	0.42097267	0.712511
114565	Zbtb21	zinc finger and BTB domain containing 21, transcript	NA	-0.08109	4.39593	-1.05782	0.42101557	0.712511
30926	Glrx3	glutaredoxin 3, transcript variant 1	NA	-0.04073	6.932145	-1.02864	0.42114646	0.712511
68283	9530077C05Rik	RIKEN cDNA 9530077C05 gene, transcript variant 1	NA	-0.0854	3.4488	-1.06098	0.42115411	0.712511
105242394	Gm38678	predicted gene, 38678	NA	-0.28326	-0.03448	-1.21694	0.4211592	0.712511
212398	Frat2	frequently rearranged in advanced T cell lymphomas	NA	0.072767	4.134283	1.051732	0.42117498	0.712511
235283	Gramd1b	GRAM domain containing 1B, transcript variant X39	NA	0.050341	6.861655	1.035509	0.42118531	0.712511
229211	Acad9	acyl-Coenzyme A dehydrogenase family, member 9,	NA	-0.04579	5.516853	-1.03225	0.42119224	0.712511
14164	Fgf1	fibroblast growth factor 1, transcript variant X14	NA	0.1942	0.851473	1.144089	0.42173617	0.713357
21378	Tbrg3	transforming growth factor beta regulated gene 3	NA	-0.23259	0.914459	-1.17494	0.42195871	0.713659
668923	Zfp442	zinc finger protein 442, transcript variant X1	NA	-0.09838	3.228878	-1.07057	0.42213873	0.713888
78294	Rps27a	ribosomal protein S27A, transcript variant 1	NA	0.03989	8.7718	1.028035	0.42218206	0.713888
72102	Dusp11	dual specificity phosphatase 11 (RNA/RNP complex	NA	-0.03847	7.283848	-1.02703	0.42222893	0.713893
72007	Fndc3b	fibronectin type III domain containing 3B, transcript va	NA	0.065204	4.643252	1.046233	0.42229845	0.713919
100503311	Pifo	primary cilia formation, transcript variant 2	NA	0.167219	1.103599	1.122892	0.42233206	0.713919
73744	Man2c1	mannosidase, alpha, class 2C, member 1	NA	0.045259	5.644876	1.031868	0.42264395	0.714334
26943	Serinc3	serine incorporator 3, transcript variant X1	NA	0.038708	7.15332	1.027193	0.42266544	0.714334
71970	Zbed5	zinc finger, BED type containing 5, transcript variant	NA	-0.09069	4.152533	-1.06488	0.42283106	0.714487
13204	Dhx15	DEAH (Asp-Glu-Ala-His) box polypeptide 15, transcri	NA	0.037136	7.462653	1.026075	0.42284381	0.714487
226591	Tiprl	TIP41, TOR signalling pathway regulator-like (S. cere	NA	0.045125	6.186479	1.031772	0.42295716	0.714547
77920	A330102I10Rik	RIKEN cDNA A330102I10 gene, transcript variant 3	NA	-0.12677	2.313239	-1.09185	0.42296685	0.714547
102634170	Gm31819	predicted gene, 31819, transcript variant X4	NA	-0.19481	0.604568	-1.14457	0.42302165	0.714565
20473	Six3	sine oculis-related homeobox 3, transcript variant X1	NA	-0.06106	5.220651	-1.04324	0.42308945	0.714606
192970	Dhrs11	dehydrogenase/reductase (SDR family) member 11,	NA	0.148478	1.922375	1.108399	0.42315029	0.714634
15111	Hand2	heart and neural crest derivatives expressed 2	NA	-0.42548	-1.30842	-1.34302	0.42330757	0.714789
73103	3110009E18Rik	RIKEN cDNA 3110009E18 gene, transcript variant 1	NA	0.149569	1.359457	1.109238	0.42334363	0.714789
227522	Rpp38	ribonuclease P/MRP 38 subunit	NA	-0.10761	2.76757	-1.07744	0.42337391	0.714789
115485727	LOC115485727	uncharacterized LOC115485727, transcript variant X	NA	-0.19792	0.583473	-1.14704	0.42346001	0.714861
102638889	Gm35343	predicted gene, 35343, transcript variant X1	NA	0.193826	1.645704	1.143793	0.42351014	0.714871
210108	D130043K22Rik	RIKEN cDNA D130043K22 gene, transcript variant 1	NA	0.106335	2.721505	1.07649	0.42365922	0.715049
619441	Tnfsfm13	tumor necrosis factor (ligand) superfamily, membrane	NA	-0.61467	-1.02048	-1.53121	0.42372317	0.715082
71679	Atp5h	ATP synthase, H+ transporting, mitochondrial F0 com	NA	0.040157	6.981602	1.028226	0.42402097	0.715239
70103	Znhit1	zinc finger, HIT domain containing 1, transcript variar	NA	-0.05228	5.253953	-1.0369	0.42403487	0.715239
18226	Nup62	nucleoporin 62	NA	-0.04551	5.730488	-1.03205	0.42404899	0.715239
72033	Tsc22d2	TSC22 domain family, member 2, transcript variant 2	NA	0.050826	5.986431	1.035858	0.42416386	0.715239
408067	Zfp874b	zinc finger protein 874b	NA	-0.08587	3.533117	-1.06132	0.42416457	0.715239
98766	Ubac1	ubiquitin associated domain containing 1, transcript v	NA	0.043531	5.93091	1.030633	0.42417754	0.715239
118568781	LOC118568781	ribosome biogenesis regulatory protein homolog	NA	0.102058	3.199216	1.073303	0.42422725	0.715239
225115	Svil	supervillin, transcript variant 2	NA	-0.06117	4.70002	-1.04331	0.42426351	0.715239
19301	Pxmp2	peroxisomal membrane protein 2	NA	0.162496	1.321158	1.119222	0.42431204	0.715239
69259	Kctd5	potassium channel tetramerisation domain containing	NA	0.045108	6.152869	1.03176	0.42431657	0.715239
628850	6030426L16Rik	RIKEN cDNA 6030426L16 gene, transcript variant X	NA	0.185019	1.450313	1.136832	0.42438908	0.715239
75964	Trappc8	trafficking protein particle complex 8, transcript variar	NA	0.04202	5.87115	1.029554	0.42439691	0.715239

279572	Tlr13	toll-like receptor 13	NA	0.42676	-0.68793	1.344211	0.42440286	0.715239
226747	Ahctf1	AT hook containing transcription factor 1	NA	0.063088	5.895657	1.044699	0.4244679	0.715239
320204	Etfbkmt	electron transfer flavoprotein beta subunit lysine meth	NA	0.132816	1.952283	1.096432	0.42447528	0.715239
20128	Trim30a	tripartite motif-containing 30A, transcript variant X2	NA	0.215254	0.231588	1.160908	0.42471473	0.715562
20536	Slc4a3	solute carrier family 4 (anion exchanger), member 3,	NA	0.046331	6.395833	1.032635	0.42475518	0.715562
69106	Stoml1	stomatin-like 1, transcript variant 1	NA	0.052314	5.719517	1.036927	0.42481826	0.715594
223272	Itgbl1	integrin, beta-like 1	NA	0.135431	1.742777	1.098421	0.42486649	0.715602
11428	Aco1	aconitase 1, transcript variant X1	NA	-0.03963	6.160187	-1.02785	0.42497348	0.715708
12298	Cacnb4	calcium channel, voltage-dependent, beta 4 subunit,	NA	0.051404	5.299649	1.036273	0.42515021	0.715895
74020	Cpne4	copine IV, transcript variant X1	NA	0.0716	4.864205	1.050881	0.4252398	0.715895
102640192	Gm15510	predicted gene 15510	NA	0.224952	0.305893	1.168738	0.42524822	0.715895
100043580	Gm4532	predicted gene 4532	NA	-0.12895	2.228038	-1.0935	0.4252607	0.715895
544963	Iqgap2	IQ motif containing GTPase activating protein 2, trans	NA	-0.08709	4.048488	-1.06223	0.42533271	0.715942
11867	Arpc1b	actin related protein 2/3 complex, subunit 1B	NA	0.067753	3.939205	1.048083	0.42539978	0.715955
66431	Oxld1	oxidoreductase like domain containing 1, transcript va	NA	-0.22523	1.143781	-1.16897	0.42550778	0.715955
12237	Bub3	BUB3 mitotic checkpoint protein, transcript variant 1	NA	-0.04596	6.513549	-1.03237	0.42551509	0.715955
110198	Akr7a5	aldo-keto reductase family 7, member A5 (aflatoxin a	NA	0.068176	3.952034	1.04839	0.42552113	0.715955
17527	Mpv17	MpV17 mitochondrial inner membrane protein, transc	NA	-0.06756	4.50999	-1.04794	0.4256006	0.715955
64453	Zfp280b	zinc finger protein 280B	NA	-0.06679	4.304872	-1.04738	0.42564245	0.715955
56506	Cib2	calcium and integrin binding family member 2	NA	0.062307	4.742555	1.044134	0.42574062	0.715955
245469	Pdzd4	PDZ domain containing 4, transcript variant X2	NA	0.035925	7.984773	1.025214	0.42577054	0.715955
73723	Sh3bgrl3	SH3 domain binding glutamic acid-rich protein-like 3	NA	-0.05765	5.437793	-1.04077	0.42577269	0.715955
269682	Golga3	golgi autoantigen, golgin subfamily a, 3, transcript var	NA	0.043293	5.795	1.030463	0.42578011	0.715955
74255	Smu1	smu-1 suppressor of mec-8 and unc-52 homolog (C.	NA	0.041652	6.413319	1.029292	0.42596069	0.716184
70652	Tmem144	transmembrane protein 144, transcript variant 4	NA	-0.20827	0.822976	-1.1553	0.42607998	0.716251
102640374	Gm36447	predicted gene, 36447	NA	0.132969	1.717003	1.096548	0.42616239	0.716251
66268	Pigyl	phosphatidylinositol glycan anchor biosynthesis, clas	NA	0.087641	3.637626	1.062631	0.42628809	0.716251
192161	Pcdha9	protocadherin alpha 9	NA	-0.11007	4.233244	-1.07928	0.42633477	0.716251
105245750	Gm41149	predicted gene, 41149, transcript variant X1	NA	0.224154	0.434423	1.168092	0.42633513	0.716251
69181	Dyrk2	dual-specificity tyrosine-(Y)-phosphorylation regulate	NA	0.042246	6.654373	1.029716	0.42635109	0.716251
76416	Znrd1as	zinc ribbon domain containing 1, antisense, transcript	NA	0.166848	1.663875	1.122603	0.42635177	0.716251
108167437	Gm44812	predicted gene 44812, transcript variant X1	NA	-0.17072	1.428897	-1.12562	0.42635712	0.716251
214150	Ago3	argonaute RISC catalytic subunit 3, transcript variant	NA	-0.05237	5.083992	-1.03697	0.42639639	0.716251
78878	Ftx	Ftx transcript, Xist regulator (non-protein coding), tran	NA	0.068503	3.998824	1.048628	0.42652304	0.716321
227622	Paxx	non-homologous end joining factor	NA	0.073877	4.35848	1.052541	0.4266106	0.716321
105171	Arrdc3	arrestin domain containing 3, transcript variant 3	NA	0.0615	6.383414	1.04355	0.42667873	0.716321
224697	Adamts10	a disintegrin-like and metallopeptidase (reprolysin typ	NA	-0.04324	5.804948	-1.03043	0.42672732	0.716321
225358	Fam13b	family with sequence similarity 13, member B, transcr	NA	0.038968	6.717254	1.027378	0.42680488	0.716321
18787	Serpine1	serine (or cysteine) peptidase inhibitor, clade E, mem	NA	-0.16972	1.386638	-1.12484	0.42680582	0.716321
233890	Zfp768	zinc finger protein 768	NA	0.051218	5.018763	1.03614	0.42681736	0.716321
74616	Scrn3	secernin 3, transcript variant 2	NA	-0.09382	2.917207	-1.0672	0.42684175	0.716321
102636910	Gm33852	predicted gene, 33852, transcript variant X2	NA	0.162891	1.344933	1.119529	0.42684304	0.716321
140489	Bhlhe23	basic helix-loop-helix family, member e23	NA	-0.15424	1.586053	-1.11283	0.42687814	0.716321
102634642	Gm32178	predicted gene, 32178, transcript variant X3	NA	-0.20833	0.460833	-1.15535	0.42692547	0.716326
17152	Mak	male germ cell-associated kinase, transcript variant X	NA	0.156615	1.616806	1.114669	0.42713204	0.716572
231430	Cox18	cytochrome c oxidase assembly protein 18, transcript	NA	0.098022	3.060555	1.070305	0.42715998	0.716572
100041420	Gm3325	predicted gene 3325	NA	-0.07354	3.922344	-1.0523	0.42722149	0.716601
230451	Junos	jun proto-oncogene, opposite strand, transcript variar	NA	-0.14604	2.552418	-1.10653	0.42733975	0.716718
170761	Pdzd3	PDZ domain containing 3	NA	-0.32648	-0.33759	-1.25395	0.42737937	0.716718
70616	Sugp1	SURP and G patch domain containing 1, transcript va	NA	0.046828	5.826424	1.032991	0.42747652	0.716807
108168762	LOC108168762	uncharacterized LOC108168762	NA	-0.23292	0.808209	-1.17521	0.42766555	0.71705
218397	Rasa1	RAS p21 protein activator 1	NA	0.061349	6.655312	1.043441	0.42777759	0.717164
56212	Rhog	ras homolog family member G	NA	-0.09324	3.262443	-1.06677	0.42791818	0.717326
218865	Chdh	choline dehydrogenase, transcript variant 3	NA	-0.2133	0.447046	-1.15934	0.4280001	0.71739
118568340	LOC118568340	igE-binding protein-like	NA	-0.49793	-0.99116	-1.41219	0.42808336	0.717455
117160	Ttyh2	tweety family member 2	NA	0.067048	4.954314	1.047571	0.42829759	0.71774
69263	Rfc3	replication factor C (activator 1) 3	NA	0.053211	4.902439	1.037571	0.42850954	0.718022
18015	Nf1	neurofibromin 1	NA	0.054002	6.806723	1.03814	0.42865085	0.718156
68059	Tm9sf2	transmembrane 9 superfamily member 2	NA	0.040939	6.636231	1.028783	0.42867798	0.718156
50779	Rgs6	regulator of G-protein signaling 6, transcript variant 2	NA	0.055767	5.249088	1.039412	0.42903868	0.718621
69926	Dnah17	dynein, axonemal, heavy chain 17, transcript variant 1	NA	0.294748	-0.35952	1.22667	0.42904423	0.718621
17749	Polr2k	polymerase (RNA) II (DNA directed) polypeptide K, tr	NA	-0.06572	4.17009	-1.04661	0.42922494	0.718663
72042	Cotl1	coactosin-like 1 (Dictyostelium)	NA	-0.0692	7.241958	-1.04913	0.42925457	0.718663
66483	Rpl36al	ribosomal protein L36A-like	NA	-0.0593	6.154263	-1.04196	0.42928469	0.718663
17183	Matn4	matrilin 4, transcript variant 2	NA	0.194134	1.494295	1.144038	0.42935376	0.718663
226548	Aph1a	aph1 homolog A, gamma secretase subunit, transcrip	NA	0.044437	6.354396	1.031281	0.42936053	0.718663
102635142	Gm32555	predicted gene, 32555, transcript variant X2	NA	0.311711	-0.26841	1.241179	0.42936405	0.718663
115487094	Gm51627	predicted gene, 51627	NA	0.183861	1.294762	1.13592	0.42937835	0.718663

100042784	Prdm11	PR domain containing 11, transcript variant X5	NA	0.055365	4.948171	1.039122	0.42948684	0.718713
98386	Lbr	lamin B receptor, transcript variant X1	NA	0.05815	5.151889	1.04113	0.42949665	0.718713
319638	Nt5dc1	5'-nucleotidase domain containing 1, transcript variar	NA	0.170543	1.21453	1.125482	0.42959966	0.718812
218314	Zfp595	zinc finger protein 595, transcript variant 1	NA	-0.14184	1.71067	-1.10331	0.4297794	0.719039
225651	Mppe1	metallophosphoesterase 1, transcript variant X6	NA	0.143544	2.193901	1.104615	0.42997016	0.719066
229285	Spg20	spastic paraplegia 20, spartin (Troyer syndrome) hor	NA	-0.06177	4.856277	-1.04374	0.42998202	0.719066
60406	Sap30	sin3 associated polypeptide	NA	-0.07832	4.772195	-1.05579	0.43001368	0.719066
100040972	Tceal7	transcription elongation factor A (SII)-like 7	NA	0.146693	1.906738	1.107029	0.43003232	0.719066
69912	Nup43	nucleoporin 43	NA	-0.06906	3.897745	-1.04903	0.43008816	0.719066
12301	Cacybp	calcyclin binding protein	NA	0.036308	7.281655	1.025486	0.43010216	0.719066
226844	Flvcr1	feline leukemia virus subgroup C cellular receptor 1, t	NA	0.063201	4.722735	1.044781	0.4301335	0.719066
14787	Rhpn1	rhophilin, Rho GTPase binding protein 1, transcript va	NA	-0.10417	3.018907	-1.07487	0.43015706	0.719066
83435	Plekha3	pleckstrin homology domain-containing, family A (phc	NA	-0.06178	4.884289	-1.04375	0.4301933	0.719066
54160	Copg2	coatomer protein complex, subunit gamma 2	NA	-0.0426	6.09931	-1.02997	0.43025055	0.719088
12845	Comp	cartilage oligomeric matrix protein	NA	-0.35931	-0.8132	-1.28282	0.43032618	0.719131
53611	Vti1a	vesicle transport through interaction with t-SNAREs 1	NA	-0.05098	6.009059	-1.03597	0.43040244	0.719131
70605	Zdhhc24	zinc finger, DHHC domain containing 24, transcript va	NA	-0.07508	4.29205	-1.05342	0.43040938	0.719131
68355	2010204K13Rik	RIKEN cDNA 2010204K13 gene, transcript variant 2	NA	-0.15253	1.30074	-1.11152	0.43067589	0.719503
320253	Marchf3	membrane associated ring-CH-type finger 3, transcrip	NA	-0.11253	2.630879	-1.08112	0.43072068	0.719504
71474	Ppp6r2	protein phosphatase 6, regulatory subunit 2, transcrip	NA	0.042425	6.688769	1.029843	0.43083154	0.719586
51810	Hnrnpu	heterogeneous nuclear ribonucleoprotein U, transcrip	NA	-0.03713	9.67567	-1.02607	0.43085814	0.719586
11826	Aqp1	aquaporin 1	NA	0.064896	4.014325	1.046009	0.43096245	0.719645
118567497	LOC118567497	uncharacterized LOC118567497	NA	-0.1969	0.825661	-1.14624	0.43109159	0.719645
20133	Rrm1	ribonucleotide reductase M1	NA	-0.04969	6.425664	-1.03504	0.4311726	0.719645
230500	Efcab7	EF-hand calcium binding domain 7, transcript variant	NA	-0.11032	2.981107	-1.07947	0.43117524	0.719645
67531	5730408K05Rik	RIKEN cDNA 5730408K05 gene	NA	0.209213	0.457887	1.156057	0.43119071	0.719645
20533	Slc4a1	solute carrier family 4 (anion exchanger), member 1	NA	-0.21241	3.421429	-1.15862	0.43119854	0.719645
208146	Yeats2	YEATS domain containing 2, transcript variant X12	NA	-0.04015	6.051608	-1.02822	0.43120336	0.719645
102632331	Gm30431	predicted gene, 30431	NA	-0.24186	-0.05691	-1.18252	0.43159283	0.720221
12892	Cpox	coproporphyrinogen oxidase	NA	-0.06537	4.244708	-1.04635	0.43164281	0.720231
67887	Saraf	store-operated calcium entry-associated regulatory fa	NA	0.043072	6.944228	1.030305	0.43181608	0.720436
12628	Cfh	complement component factor h	NA	0.073338	4.59189	1.052148	0.43185452	0.720436
385668	Lca5l	Leber congenital amaurosis 5-like, transcript variant X	NA	0.155268	1.63247	1.113629	0.43203443	0.720562
67126	Atp5e	ATP synthase, H+ transporting, mitochondrial F1 com	NA	0.050127	6.128954	1.035356	0.43203587	0.720562
65257	Asb3	ankyrin repeat and SOCS box-containing 3, transcript	NA	0.069331	4.243669	1.04923	0.43210987	0.720562
15376	Foxa2	forkhead box A2, transcript variant 2	NA	0.202449	1.378153	1.15065	0.43212396	0.720562
118568312	LOC118568312	protein transport protein sec31-like	NA	0.272114	12.59644	1.207576	0.43215104	0.720562
115488138	Gm15912	predicted gene 15912	NA	0.278273	-0.30777	1.212743	0.43227081	0.72065
100043133	9130023H24Rik	RIKEN cDNA 9130023H24 gene	NA	-0.08589	3.218217	-1.06134	0.43231762	0.72065
67742	Samsn1	SAM domain, SH3 domain and nuclear localization si	NA	0.199213	0.572909	1.148072	0.43233699	0.72065
19401	Rara	retinoic acid receptor, alpha, transcript variant X4	NA	-0.06449	4.370581	-1.04571	0.43243963	0.720747
19363	Rad51b	RAD51 paralog B, transcript variant X18	NA	-0.1893	0.679714	-1.14021	0.43253137	0.720809
57434	Xrcc2	X-ray repair complementing defective repair in Chine	NA	-0.10342	3.082798	-1.07431	0.4325649	0.720809
71735	Lrwd1	leucine-rich repeats and WD repeat domain containin	NA	-0.06952	4.632351	-1.04936	0.43266717	0.720905
100504586	Gm20300	predicted gene, 20300	NA	0.066307	4.552716	1.047033	0.43276196	0.720989
66391	Zbtb11os1	zinc finger and BTB domain containing 11, opposite s	NA	0.191379	0.604361	1.141855	0.4328177	0.721008
66223	Mrpl35	mitochondrial ribosomal protein L35	NA	-0.06418	4.469265	-1.04549	0.43291786	0.721101
22033	Traf5	TNF receptor-associated factor 5	NA	-0.23854	0.543145	-1.1798	0.43311534	0.721305
73230	Bmper	BMP-binding endothelial regulator	NA	-0.06882	3.824481	-1.04886	0.43312886	0.721305
69700	Col22a1	collagen, type XXII, alpha 1	NA	-0.08339	4.225144	-1.0595	0.43329047	0.721358
76332	Cog2	component of oligomeric golgi complex 2	NA	0.053101	4.745121	1.037493	0.43329678	0.721358
53313	Atp2a3	ATPase, Ca++ transporting, ubiquitous, transcript var	NA	-0.21162	1.740822	-1.15799	0.43333611	0.721358
67282	Washc3	WASH complex subunit 3, transcript variant 1	NA	0.072885	3.94982	1.051818	0.43333776	0.721358
12167	Bmpr1b	bone morphogenetic protein receptor, type 1B, transc	NA	0.097091	3.350804	1.069615	0.43344063	0.721455
66079	Tmem42	transmembrane protein 42, transcript variant 1	NA	0.122939	2.707053	1.088951	0.43367342	0.721583
105244413	4930445K14Rik	RIKEN cDNA 4930445K14 gene	NA	-0.16187	1.481223	-1.11874	0.43374536	0.721583
71177	Ints13	integrator complex subunit 13, transcript variant 4	NA	-0.05591	5.3875	-1.03952	0.43389265	0.721583
67674	Trmt112	tRNA methyltransferase 11-2, transcript variant 2	NA	-0.05526	4.989148	-1.03905	0.43390002	0.721583
29816	Hip1r	huntingtin interacting protein 1 related	NA	-0.04471	5.922052	-1.03147	0.43395492	0.721583
102566	Ano10	anoctamin 10, transcript variant 1	NA	0.055329	4.964726	1.039096	0.4339852	0.721583
620499	Gm6158	predicted gene 6158	NA	-0.14016	4.355595	-1.10203	0.43399323	0.721583
233752	Insc	INSC spindle orientation adaptor protein, transcript va	NA	0.239231	0.321129	1.180363	0.43401006	0.721583
58805	Mlxipl	MLX interacting protein-like, transcript variant 2	NA	-0.20583	0.51156	-1.15335	0.43402299	0.721583
57276	Vsig2	V-set and immunoglobulin domain containing 2	NA	-0.22975	0.002715	-1.17263	0.43402742	0.721583
99296	Hrh3	histamine receptor H3, transcript variant X2	NA	-0.09924	2.610174	-1.07121	0.43405961	0.721583
232798	Leng8	leukocyte receptor cluster (LRC) member 8, transcrip	NA	-0.03601	8.312502	-1.02527	0.43407865	0.721583
320301	E530011L22Rik	RIKEN cDNA E530011L22 gene	NA	0.113177	2.4002	1.081608	0.43411763	0.721583
72667	Zfp444	zinc finger protein 444, transcript variant 1	NA	-0.053	5.48632	-1.03742	0.43418792	0.721583

16510	Kcnh1	potassium voltage-gated channel, subfamily H (eag-r	NA	-0.08405	3.35987	-1.05999	0.43422159	0.721583
104886	Rab15	RAB15, member RAS oncogene family, transcript vai	NA	-0.05561	6.002316	-1.0393	0.43427485	0.721583
66867	Hmg20a	high mobility group 20A	NA	-0.04498	6.896316	-1.03167	0.4342802	0.721583
219150	Hmbox1	homeobox containing 1, transcript variant X11	NA	0.038117	6.842357	1.026773	0.43431579	0.721583
100039691	Lncenc1	long non-coding RNA, embryonic stem cells express€	NA	0.15808	1.288386	1.115801	0.43454402	0.721822
53415	Htatip2	HIV-1 Tat interactive protein 2, transcript variant 5	NA	-0.21001	0.731972	-1.15669	0.43454797	0.721822
329790	A630034I12Rik	RIKEN cDNA A630034I12 gene, transcript variant X3	NA	-0.08079	3.437562	-1.0576	0.43466188	0.721847
11811	Apobec2	apolipoprotein B mRNA editing enzyme, catalytic poly	NA	-0.3386	-0.2193	-1.26453	0.43468487	0.721847
338368	Pheta2	PH domain containing endocytic trafficking adaptor 2	NA	-0.21127	0.522769	-1.1577	0.43469636	0.721847
622552	Gm20751	predicted gene, 20751	NA	0.203241	0.375096	1.151282	0.43475402	0.721869
22186	Uba52	ubiquitin A-52 residue ribosomal protein fusion produ	NA	0.045884	8.387457	1.032316	0.43485729	0.721967
231326	Aasdh	aminoadipate-semialdehyde dehydrogenase, transcri	NA	-0.07405	4.185682	-1.05267	0.43495611	0.722043
320506	Lmbrd2	LMBR1 domain containing 2, transcript variant X3	NA	0.061909	5.798843	1.043846	0.43499498	0.722043
320878	Mical2	microtubule associated monooxygenase, calponin an	NA	-0.09643	3.971351	-1.06912	0.43503606	0.722043
65960	Twsg1	twisted gastrulation BMP signaling modulator 1, trans	NA	-0.04294	6.22304	-1.03021	0.43522515	0.722238
271377	Zbtb11	zinc finger and BTB domain containing 11, transcript	NA	0.062275	4.840671	1.044111	0.43524257	0.722238
73247	Mrgbp	MRG/MORF4L binding protein, transcript variant 1	NA	0.052189	5.432233	1.036837	0.4353142	0.722283
11865	Arntl	aryl hydrocarbon receptor nuclear translocator-like, tr	NA	0.071215	4.38093	1.050601	0.43537436	0.722309
22694	Zfp35	zinc finger protein 35, transcript variant X1	NA	-0.05267	4.978437	-1.03718	0.43559482	0.722602
12389	Cav1	caveolin 1, caveolae protein, transcript variant X3	NA	0.061276	5.27412	1.043388	0.43575158	0.722756
19662	Rbp4	retinol binding protein 4, plasma, transcript variant 2	NA	0.225762	0.266062	1.169394	0.4358198	0.722756
236899	Pcyt1b	phosphate cytidylyltransferase 1, choline, beta isofor	NA	0.041045	6.299151	1.028858	0.43586245	0.722756
67881	Mdp1	magnesium-dependent phosphatase 1, transcript var	NA	0.049924	5.470195	1.03521	0.43594654	0.722756
23806	Arih1	ariadne RBR E3 ubiquitin protein ligase 1	NA	0.045262	7.300028	1.031871	0.43598476	0.722756
66885	Acadsb	acyl-Coenzyme A dehydrogenase, short/branched ch	NA	-0.04382	5.930865	-1.03084	0.43601932	0.722756
74318	Hopx	HOP homeobox, transcript variant 2	NA	0.085458	3.854244	1.061024	0.43603178	0.722756
100503323	A230087F16Rik	RIKEN cDNA A230087F16 gene, transcript variant X	NA	-0.22468	0.557575	-1.16852	0.43610221	0.722756
320924	Ccbe1	collagen and calcium binding EGF domains 1, transc	NA	0.082472	3.570532	1.058831	0.43610276	0.722756
102635484	Gm32810	predicted gene, 32810	NA	0.18383	0.893664	1.135896	0.43613237	0.722756
18628	Per3	period circadian clock 3, transcript variant X16	NA	-0.10324	2.873387	-1.07418	0.43640235	0.72313
83395	Sp6	trans-acting transcription factor 6, transcript variant 1	NA	-0.2453	0.401677	-1.18534	0.43662555	0.723421
69085	Zcchc9	zinc finger, CCHC domain containing 9	NA	0.07002	4.080502	1.049731	0.43672489	0.723421
68195	Rnaset2b	ribonuclease T2B	NA	0.112217	4.611008	1.080888	0.43680692	0.723421
12545	Cdc7	cell division cycle 7 (S. cerevisiae), transcript variant	NA	-0.04629	5.395218	-1.0326	0.43680979	0.723421
72017	Cyb5r1	cytochrome b5 reductase 1, transcript variant 1	NA	-0.0814	3.35464	-1.05805	0.43682873	0.723421
15384	Hnrnpab	heterogeneous nuclear ribonucleoprotein A/B, transc	NA	-0.04238	9.53073	-1.02981	0.43684486	0.723421
236082	Dhrsx	dehydrogenase/reductase (SDR family) X chromosor	NA	0.097886	3.425551	1.070204	0.43689573	0.723432
214968	Sema6d	sema domain, transmembrane domain (TM), and cyto	NA	0.046773	6.633209	1.032952	0.43716504	0.72378
231128	Fam193a	family with sequence homology 193, member A	NA	-0.03957	6.618568	-1.02781	0.4372661	0.72378
69802	Cox11	cytochrome c oxidase assembly protein 11, copper cl	NA	0.065326	4.353691	1.046321	0.43728279	0.72378
107581	Col16a1	collagen, type XVI, alpha 1, transcript variant X9	NA	-0.06443	4.66413	-1.04567	0.43728378	0.72378
213990	Agap3	ArfGAP with GTPase domain, ankyrin repeat and PH	NA	0.037209	7.410363	1.026127	0.43765627	0.724246
67390	Mrm3	mitochondrial rRNA methyltransferase 3	NA	-0.08426	3.105962	-1.06014	0.43774846	0.724246
118567972	LOC118567972	uncharacterized LOC118567972	NA	0.25253	-0.2584	1.191295	0.43776396	0.724246
242291	Bpnt2	3'(2'), 5'-bisphosphate nucleotidase 2	NA	0.045581	7.417347	1.032099	0.4377718	0.724246
387285	Hcrtr2	hypocretin (orexin) receptor 2, transcript variant 1	NA	0.122189	1.997344	1.088385	0.43778769	0.724246
329910	Acot11	acyl-CoA thioesterase 11, transcript variant X1	NA	-0.13236	2.308114	-1.09609	0.43786864	0.72427
98403	Zfp451	zinc finger protein 451, transcript variant X2	NA	0.060423	5.270187	1.042772	0.43789121	0.72427
216527	Ccm2	cerebral cavernous malformation 2, transcript variant	NA	-0.05804	5.263702	-1.04105	0.43800325	0.72438
67772	Chd8	chromodomain helicase DNA binding protein 8	NA	0.035001	7.233378	1.024557	0.43804694	0.72438
12339	Capn7	calpain 7, transcript variant 1	NA	-0.04247	6.616026	-1.02988	0.43821655	0.724521
68833	Pdcl3	phosducin-like 3	NA	0.048465	5.890794	1.034164	0.43822117	0.724521
76113	Lpo	lactoperoxidase, transcript variant X1	NA	-0.21668	0.391507	-1.16206	0.43829586	0.724539
12183	Bpgm	2,3-bisphosphoglycerate mutase, transcript variant 1	NA	0.048733	6.488694	1.034356	0.43832136	0.724539
66049	Rogdi	rogdi homolog	NA	-0.05153	4.945688	-1.03636	0.4384292	0.724606
14202	Fhl4	four and a half LIM domains 4	NA	0.178817	0.916912	1.131955	0.43845052	0.724606
13430	Dnm2	dynamin 2, transcript variant X15	NA	-0.04545	6.383467	-1.03201	0.43851659	0.724641
207683	Igsf11	immunoglobulin superfamily, member 11	NA	-0.06758	3.773966	-1.04796	0.43865365	0.724758
102633516	Peak1os	pseudopodium-enriched atypical kinase 1, opposite s	NA	-0.3275	-0.29025	-1.25484	0.43867637	0.724758
110599566	Eef1akmt4	EEF1A lysine methyltransferase 4	NA	-0.1932	1.337605	-1.1433	0.43873137	0.724775
118568495	LOC118568495	uncharacterized LOC118568495	NA	-0.16885	1.135206	-1.12416	0.43882046	0.724849
216344	Rab21	RAB21, member RAS oncogene family	NA	-0.07557	5.30958	-1.05378	0.43891454	0.724931
105855	Nckap1l	NCK associated protein 1 like	NA	0.12261	2.156304	1.088703	0.43896965	0.724948
232816	Zfp628	zinc finger protein 628, transcript variant X2	NA	0.071556	4.293074	1.050849	0.43923635	0.725259
192650	Cabp7	calcium binding protein 7	NA	-0.11428	2.426379	-1.08244	0.43924681	0.725259
11486	Ada	adenosine deaminase, transcript variant 2	NA	-0.23089	1.027663	-1.17356	0.43934992	0.725355
16792	Laptm5	lysosomal-associated protein transmembrane 5	NA	-0.0789	3.960964	-1.05621	0.43940156	0.725367
208076	Pknox2	Pbx/knotted 1 homeobox 2, transcript variant 1	NA	-0.06695	5.454816	-1.0475	0.43950167	0.725459

56790	Supt20	SPT20 SAGA complex component, transcript variant	NA	0.039399	6.227558	1.027686	0.43963694	0.725608
226098	Hectd2	HECT domain E3 ubiquitin protein ligase 2, transcript	NA	0.082406	4.932824	1.058783	0.43970208	0.725623
67864	Yipf4	Yip1 domain family, member 4	NA	0.059973	5.118071	1.042446	0.43975593	0.725623
12039	Bckdha	branched chain ketoacid dehydrogenase E1, alpha p	NA	0.053803	4.848533	1.037997	0.43979041	0.725623
76338	Rab2b	RAB2B, member RAS oncogene family	NA	0.045323	5.819968	1.031914	0.43982395	0.725623
76687	Spcs3	signal peptidase complex subunit 3 homolog (S. cere	NA	0.047764	5.818198	1.033662	0.43988545	0.72565
140477	Dmbx1	diencephalon/mesencephalon homeobox 1, transcrip	NA	0.086028	3.204178	1.061443	0.44000871	0.72578
208177	Phldb2	pleckstrin homology like domain, family B, member 2	NA	0.059036	4.525366	1.04177	0.44009316	0.725846
13681	Eif4a1	eukaryotic translation initiation factor 4A1, transcript v	NA	-0.04566	9.833052	-1.03216	0.44032913	0.726094
54598	Calcrl	calcitonin receptor-like, transcript variant X1	NA	0.118545	3.414181	1.08564	0.44038809	0.726094
74895	Ccdc181	coiled-coil domain containing 181	NA	-0.06643	4.499505	-1.04712	0.44039001	0.726094
68564	Nufip2	nuclear fragile X mental retardation protein interacting	NA	-0.04045	6.399784	-1.02843	0.44042194	0.726094
54130	Actr1a	ARP1 actin-related protein 1A, centractin alpha, trans	NA	0.034666	8.475428	1.02432	0.44064912	0.726395
100503632	Gm16892	predicted gene, 16892	NA	-0.25084	-0.20304	-1.1899	0.44076587	0.726514
68053	Ubxn2b	UBX domain protein 2B	NA	-0.05603	4.499394	-1.0396	0.44100517	0.726763
106931	Kctd1	potassium channel tetramerisation domain containing	NA	0.053087	5.015052	1.037483	0.44100907	0.726763
106264	0610012G03Rik	RIKEN cDNA 0610012G03 gene	NA	-0.05556	4.693334	-1.03927	0.44105126	0.726763
12400	Cbfb	core binding factor beta, transcript variant 4	NA	0.044684	6.087535	1.031458	0.4411273	0.726815
69994	Rsc1a1	regulatory solute carrier protein, family 1, member 1	NA	0.11798	3.143924	1.085214	0.44132929	0.727074
76366	Mtif3	mitochondrial translational initiation factor 3, transcrip	NA	-0.06664	4.028059	-1.04728	0.44147537	0.727241
66952	2310030G06Rik	RIKEN cDNA 2310030G06 gene	NA	0.253507	-0.00097	1.192101	0.441566	0.727317
56802	Gnasas1	GNAS antisense RNA 1	NA	-0.31359	-0.00886	-1.2428	0.44162254	0.727336
66663	Uba5	ubiquitin-like modifier activating enzyme 5	NA	0.040773	6.200557	1.028665	0.44170824	0.72737
105446	Gmpr2	guanosine monophosphate reductase 2	NA	-0.05482	4.551442	-1.03873	0.44173251	0.72737
104457	0610010K14Rik	RIKEN cDNA 0610010K14 gene, transcript variant 3	NA	-0.05499	5.11389	-1.03885	0.44184393	0.727454
56338	Txnip	thioredoxin interacting protein, transcript variant 1	NA	0.062823	6.36318	1.044508	0.44190133	0.727454
15401	Hoxa4	homeobox A4	NA	-0.25972	2.159813	-1.19725	0.4419268	0.727454
100088	Rcc1	regulator of chromosome condensation 1, transcript v	NA	-0.06321	4.96515	-1.04479	0.44196227	0.727454
75625	Mageh1	MAGE family member H1	NA	0.039467	5.947138	1.027734	0.44205692	0.727536
102634487	Gm32059	predicted gene, 32059	NA	-0.09985	2.476168	-1.07166	0.44216569	0.72761
100504350	Gm14169	predicted gene 14169	NA	0.204471	0.384295	1.152264	0.44219127	0.72761
67153	Rnaseh2b	ribonuclease H2, subunit B	NA	-0.06191	4.498169	-1.04385	0.44236084	0.727806
108167561	Gm46059	predicted gene, 46059, transcript variant X2	NA	0.353772	-0.2713	1.277898	0.44240231	0.727806
14595	B4galt1	UDP-Gal:betaGlcNAc beta 1,4- galactosyltransferase	NA	-0.06356	4.30037	-1.04504	0.44244458	0.727806
226255	Atrnl1	attractin like 1	NA	0.039216	6.422374	1.027555	0.44258133	0.727932
232533	Stk38l	serine/threonine kinase 38 like, transcript variant 1	NA	0.058128	4.316469	1.041114	0.44261085	0.727932
93890	Pcdhb19	protocadherin beta 19	NA	-0.1311	2.426972	-1.09513	0.44266979	0.727956
70314	Rabep2	rabaptin, RAB GTPase binding effector protein 2	NA	-0.07129	3.555589	-1.05065	0.44281953	0.728128
71041	Pcgf6	polycomb group ring finger 6, transcript variant X5	NA	-0.04765	5.660554	-1.03358	0.44301252	0.728215
73212	3110082I17Rik	RIKEN cDNA 3110082I17 gene	NA	-0.08897	2.981263	-1.06361	0.44301252	0.728215
78353	2500002B13Rik	RIKEN cDNA 2500002B13 gene, transcript variant 1	NA	-0.28245	0.46112	-1.21626	0.44301589	0.728215
78536	C030004G16Rik	RIKEN cDNA C030004G16 gene, transcript variant X	NA	0.215728	0.133691	1.16129	0.44305132	0.728215
68526	Gpr155	G protein-coupled receptor 155, transcript variant 1	NA	0.087942	4.261517	1.062853	0.44310679	0.728233
71834	Zbtb43	zinc finger and BTB domain containing 43, transcript	NA	0.057413	5.113117	1.040598	0.44323841	0.728274
245841	Polr2h	polymerase (RNA) II (DNA directed) polypeptide H, tr	NA	-0.0587	4.445782	-1.04153	0.44328313	0.728274
100041089	Gm3134	predicted gene 3134	NA	0.164477	1.046751	1.12076	0.44339415	0.728274
18712	Pim1	proviral integration site 1, transcript variant 1	NA	-0.10214	4.270596	-1.07337	0.44340713	0.728274
77683	Ehmt1	euchromatic histone methyltransferase 1, transcript v	NA	-0.04039	6.852743	-1.02839	0.44342642	0.728274
71538	Fbxo9	f-box protein 9, transcript variant 2	NA	0.044245	5.528752	1.031144	0.44345658	0.728274
94223	Dgcr8	DGCR8, microprocessor complex subunit	NA	0.042149	6.335162	1.029647	0.4434851	0.728274
67103	Ptgr1	prostaglandin reductase 1, transcript variant X1	NA	-0.12078	2.164976	-1.08732	0.44351554	0.728274
100043920	Fam205a4	family with sequence similarity 205, member A4, tran	NA	0.472444	0.342717	1.387458	0.44353484	0.728274
12306	Anxa2	annexin A2	NA	-0.04415	6.078462	-1.03108	0.44358627	0.728285
115490420	Gm52910	predicted gene, 52910	NA	-0.35351	-0.41421	-1.27767	0.44367961	0.728365
71368	5430431A17Rik	RIKEN cDNA 5430431A17 gene	NA	-0.27949	-0.35576	-1.21377	0.44375691	0.728384
494504	Apcdd1	adenomatosis polyposis coli down-regulated 1	NA	-0.03839	7.03962	-1.02697	0.44378046	0.728384
620376	Gm6145	predicted gene 6145, transcript variant X46	NA	0.133882	2.40191	1.097242	0.44396032	0.728564
109648	Npy	neuropeptide Y	NA	0.074998	4.125826	1.05336	0.44398018	0.728564
239318	Plcxd3	phosphatidylinositol-specific phospholipase C, X dom	NA	0.225437	0.900975	1.169131	0.44412659	0.728661
237387	Lrrc3	leucine rich repeat containing 3	NA	-0.06285	5.085958	-1.04452	0.44412838	0.728661
20524	Slc25a17	solute carrier family 25 (mitochondrial carrier, peroxis	NA	0.040748	6.267924	1.028647	0.44436104	0.728952
101700	Trim68	tripartite motif-containing 68, transcript variant 1	NA	0.108516	2.344432	1.078119	0.44440016	0.728952
102632635	Gm26843	predicted gene, 26843, transcript variant X2	NA	-0.20897	0.537192	-1.15586	0.44444024	0.728952
386649	Nsfl1c	NSFL1 (p97) cofactor (p47), transcript variant 1	NA	-0.04028	6.910485	-1.02831	0.44453604	0.729036
22282	Usf2	upstream transcription factor 2	NA	0.038323	6.709235	1.026919	0.4446464	0.729143
11702	Amd1	S-adenosylmethionine decarboxylase 1	NA	0.03723	6.584633	1.026142	0.44478614	0.729199
102635526	Gm32838	predicted gene, 32838, transcript variant X2	NA	0.185753	1.066813	1.13741	0.44478906	0.729199
102465959	Mir6236	microRNA 6236	NA	0.355655	2.109358	1.279566	0.44481508	0.729199

227720	Nup214	nucleoporin 214, transcript variant X3	NA	-0.04931	5.408314	-1.03477	0.4449687	0.729378
72511	2610316D01Rik	RIKEN cDNA 2610316D01 gene	NA	-0.11307	2.172811	-1.08153	0.44517045	0.729635
329659	E130311K13Rik	RIKEN cDNA E130311K13 gene, transcript variant 1	NA	0.110057	2.496306	1.079271	0.44524167	0.729678
100504285	Abhd12b	abhydrolase domain containing 12B, transcript varian	NA	0.186383	1.228683	1.137907	0.4453186	0.729721
118568293	LOC118568293	uncharacterized LOC118568293	NA	0.079066	3.847711	1.056334	0.44535732	0.729721
118568438	LOC118568438	uncharacterized LOC118568438, transcript variant X	NA	-0.1343	2.437494	-1.09756	0.44554468	0.729954
115489029	Gm52393	predicted gene, 52393	NA	-0.29889	-0.20935	-1.2302	0.4456193	0.730003
208659	Fam20a	family with sequence similarity 20, member A, transcı	NA	-0.13454	1.857506	-1.09774	0.44566955	0.730012
18950	Pnp	purine-nucleoside phosphorylase	NA	0.064208	3.982494	1.045511	0.44581202	0.730171
241528	Lrrc55	leucine rich repeat containing 55, transcript variant X	NA	-0.06075	5.069482	-1.04301	0.44599824	0.730403
381485	Trim55	tripartite motif-containing 55	NA	-0.35844	-0.97404	-1.28204	0.44612922	0.730475
54409	Ramp2	receptor (calcitonin) activity modifying protein 2	NA	0.068746	4.068223	1.048805	0.44617063	0.730475
15894	Icam1	intercellular adhesion molecule 1	NA	0.135514	1.744105	1.098484	0.44627278	0.730475
19899	Rpl18	ribosomal protein L18	NA	0.044501	7.810049	1.031326	0.44627674	0.730475
224656	Zfp523	zinc finger protein 523, transcript variant X3	NA	-0.03838	6.257467	-1.02696	0.44629843	0.730475
83815	Cenpq	centromere protein Q	NA	-0.06868	3.726275	-1.04876	0.44631683	0.730475
20363	Selenop	selenoprotein P, transcript variant 1	NA	0.035199	6.668604	1.024698	0.44637795	0.730475
17863	Myb	myeloblastosis oncogene, transcript variant 2	NA	-0.14026	2.443489	-1.1021	0.44643048	0.730475
232023	Vopp1	vesicular, overexpressed in cancer, prosurvival protei	NA	-0.03402	7.729518	-1.02386	0.44644604	0.730475
245545	Pabpc1l2a	poly(A) binding protein, cytoplasmic 1-like 2A	NA	-0.10014	3.847475	-1.07188	0.4465949	0.730645
12029	Bcl6b	B cell CLL/lymphoma 6, member B, transcript variant	NA	-0.09014	3.400685	-1.06447	0.44668966	0.730726
108689	Stn1	STN1, CST complex subunit	NA	-0.08254	4.030275	-1.05888	0.446738	0.730732
56876	Nsmf	NMDA receptor synaptonuclear signaling and neuron	NA	0.035492	6.966242	1.024906	0.44682761	0.730752
23856	Dido1	death inducer-obliterator 1, transcript variant 3	NA	0.043209	6.57339	1.030403	0.44684037	0.730752
330695	Ctxn1	cortexin 1	NA	0.047541	7.537597	1.033502	0.44694839	0.730856
102632948	Gm30893	predicted gene, 30893, transcript variant X2	NA	-0.22866	0.018684	-1.17174	0.44703727	0.730927
52915	Zmiz2	zinc finger, MIZ-type containing 2, transcript variant 2	NA	-0.03661	8.221139	-1.0257	0.44719493	0.731112
74392	Specc1l	sperm antigen with calponin homology and coiled-coi	NA	-0.03988	6.73774	-1.02803	0.4473433	0.731229
245424	Gpr101	G protein-coupled receptor 101	NA	0.206611	0.341325	1.153974	0.44740829	0.731229
20379	Sfrp4	secreted frizzled-related protein 4	NA	-0.18033	0.749202	-1.13314	0.44742591	0.731229
108167726	Gm37120	predicted gene, 37120	NA	-0.25973	0.31383	-1.19726	0.44748128	0.731229
13437	Dnpep	aspartyl aminopeptidase, transcript variant 2	NA	0.048097	5.691	1.0339	0.44749102	0.731229
22268	Upk1b	uroplakin 1B, transcript variant X1	NA	-0.42404	-0.08356	-1.34168	0.44772374	0.731522
52635	Esyt2	extended synaptotagmin-like protein 2	NA	0.045973	5.887001	1.032379	0.44784487	0.731522
12125	Bcl2l11	BCL2-like 11 (apoptosis facilitator), transcript variant	NA	0.070576	4.128911	1.050136	0.44786147	0.731522
216858	Kctd11	potassium channel tetramerisation domain containing	NA	-0.15126	1.53605	-1.11054	0.44788629	0.731522
24046	Scn11a	sodium channel, voltage-gated, type XI, alpha	NA	0.230096	0.0623	1.172913	0.4479179	0.731522
170460	Stard5	StAR-related lipid transfer (START) domain containin	NA	-0.10863	2.545832	-1.0782	0.44794048	0.731522
66139	Mymk	myomaker, myoblast fusion factor, transcript variant ´	NA	-0.36687	-0.86535	-1.28955	0.44806982	0.73166
11564	Adsl	adenylosuccinate lyase	NA	-0.05491	5.235711	-1.0388	0.44813226	0.731689
100040525	Tmem181c-ps	transmembrane protein 181C, pseudogene	NA	0.095802	3.420326	1.068659	0.44820496	0.731734
83997	Slmap	sarcolemma associated protein, transcript variant X8	NA	0.050398	5.674882	1.03555	0.4485601	0.73224
20333	Sec22b	SEC22 homolog B, vesicle trafficking protein	NA	0.042821	5.420634	1.030126	0.44882855	0.732477
70026	Tspo2	translocator protein 2	NA	-0.2431	0.018074	-1.18353	0.4488741	0.732477
71890	Mad2l2	MAD2 mitotic arrest deficient-like 2, transcript variant	NA	0.050416	5.190543	1.035564	0.44891751	0.732477
380836	Mrs2	MRS2 magnesium transporter, transcript variant X1	NA	0.045644	5.942879	1.032144	0.44902675	0.732477
625054	Gm6548	predicted gene 6548	NA	0.133308	2.48868	1.096806	0.44903583	0.732477
242083	Ppm1l	protein phosphatase 1 (formerly 2C)-like, transcript va	NA	0.040769	6.511481	1.028662	0.44909643	0.732477
59125	Nek7	NIMA (never in mitosis gene a)-related expressed kin	NA	0.056241	4.470135	1.039753	0.44915194	0.732477
56462	Mtch1	mitochondrial carrier 1, transcript variant 1	NA	0.033925	8.421867	1.023794	0.44915768	0.732477
76483	Lmf1	lipase maturation factor 1, transcript variant 1	NA	-0.08675	3.377548	-1.06198	0.44917404	0.732477
235441	Usp3	ubiquitin specific peptidase 3, transcript variant X11	NA	0.041053	6.415389	1.028864	0.44918084	0.732477
100379095	A830092H15Rik	RIKEN cDNA A830092H15 gene	NA	0.132414	1.863217	1.096126	0.44920037	0.732477
17846	Commd1	COMM domain containing 1, transcript variant 1	NA	-0.0509	4.959477	-1.03591	0.44933944	0.73262
140887	Lnx2	ligand of numb-protein X 2, transcript variant X2	NA	-0.08144	4.04539	-1.05808	0.44940449	0.73262
54613	St3gal6	ST3 beta-galactoside alpha-2,3-sialyltransferase 6, tr	NA	-0.09034	2.966247	-1.06462	0.44942332	0.73262
18175	Nrap	nebulin-related anchoring protein, transcript variant X	NA	-0.17684	1.12292	-1.1304	0.44958182	0.732805
100494	Zfand2a	zinc finger, AN1-type domain 2A, transcript variant 2	NA	0.04815	5.341291	1.033938	0.44968369	0.732871
216166	Plk5	polo like kinase 5, transcript variant X4	NA	0.137755	1.565118	1.100192	0.44975608	0.732871
13805	Eng	endoglin, transcript variant 1	NA	0.053818	5.085389	1.038009	0.44975736	0.732871
14569	Gdi2	guanosine diphosphate (GDP) dissociation inhibitor 2	NA	0.034693	8.594579	1.024339	0.44990241	0.732963
12374	Casr	calcium-sensing receptor, transcript variant X5	NA	0.246911	0.071856	1.186664	0.44990402	0.732963
240023	Pnldc1	poly(A)-specific ribonuclease (PARN)-like domain cor	NA	-0.18052	0.8675	-1.13329	0.45004635	0.733122
70626	5730522E02Rik	RIKEN cDNA 5730522E02 gene	NA	0.164829	0.933597	1.121034	0.45016074	0.733164
104263	Kdm3a	lysine (K)-specific demethylase 3A, transcript variant	NA	0.054415	6.273431	1.038438	0.45019042	0.733164
64378	Gpr88	G-protein coupled receptor 88	NA	0.060156	4.405206	1.042578	0.45020731	0.733164
13404	Dmc1	DNA meiotic recombinase 1, transcript variant 2	NA	-0.15061	1.363517	-1.11004	0.45041821	0.733377
70031	Cmtm8	CKLF-like MARVEL transmembrane domain containi	NA	0.167128	0.916533	1.122821	0.45042839	0.733377

53896	Slc7a10	solute carrier family 7 (cationic amino acid transporte	NA	-0.11271	2.66645	-1.08125	0.45060406	0.73351
118567764	LOC118567764	uncharacterized LOC118567764	NA	0.253144	-0.07498	1.191802	0.4506468	0.73351
320292	Rasgef1b	RasGEF domain family, member 1B, transcript variar	NA	-0.05491	6.056083	-1.0388	0.45065001	0.73351
50493	Txnrd1	thioredoxin reductase 1, transcript variant 2	NA	-0.03616	7.358754	-1.02538	0.45069022	0.73351
227696	Phyhd1	phytanoyl-CoA dioxygenase domain containing 1, tra	NA	-0.09422	2.927369	-1.06749	0.45077411	0.733573
101631	Pwwp2b	PWWP domain containing 2B, transcript variant 2	NA	-0.11166	2.065703	-1.08047	0.45125692	0.734149
140792	Colec12	collectin sub-family member 12	NA	0.053967	4.936258	1.038115	0.45127364	0.734149
217843	Unc79	unc-79 homolog, transcript variant X23	NA	-0.04383	6.042476	-1.03084	0.45129086	0.734149
20226	Sars	seryl-aminoacyl-tRNA synthetase, transcript variant 2	NA	-0.03586	7.45027	-1.02517	0.45133075	0.734149
20511	Slc1a2	solute carrier family 1 (glial high affinity glutamate trai	NA	-0.04045	6.967012	-1.02844	0.45135365	0.734149
330814	Adgrl1	adhesion G protein-coupled receptor L1, transcript va	NA	-0.03231	9.169391	-1.02265	0.45144229	0.73422
26365	Ceacam1	carcinoembryonic antigen-related cell adhesion mole	NA	0.263331	0.354483	1.200247	0.45160238	0.734407
242253	Wdr63	WD repeat domain 63	NA	-0.15337	1.076768	-1.11217	0.45169122	0.734478
72000	Lmntd2	lamin tail domain containing 2, transcript variant X13	NA	0.195742	0.464795	1.145313	0.45175818	0.734513
66510	Rnf181	ring finger protein 181, transcript variant 1	NA	0.042507	5.711958	1.029902	0.45190004	0.734552
19773	Rln1	relaxin 1	NA	0.245281	-0.01972	1.185323	0.45193527	0.734552
67867	Lrrc28	leucine rich repeat containing 28, transcript variant 3	NA	-0.05694	4.432719	-1.04026	0.45194045	0.734552
78891	Scyl1	SCY1-like 1 (S. cerevisiae), transcript variant 1	NA	-0.04994	6.032238	-1.03522	0.45196244	0.734552
100121	Tdrd7	tudor domain containing 7, transcript variant 1	NA	0.052071	4.844824	1.036752	0.45217566	0.734825
546752	Gm5977	predicted gene 5977	NA	0.08288	4.585176	1.05913	0.45225546	0.73485
71998	Slc25a35	solute carrier family 25, member 35, transcript varian	NA	-0.19672	1.273805	-1.14609	0.45229683	0.73485
76267	Fads1	fatty acid desaturase 1	NA	0.036568	7.744533	1.025671	0.45232658	0.73485
216859	Acap1	ArfGAP with coiled-coil, ankyrin repeat and PH doma	NA	0.305332	-0.3665	1.235703	0.45243067	0.734946
109169	Igip	IgA inducing protein	NA	0.085537	3.497054	1.061082	0.45248032	0.734953
114641	Rpl31	ribosomal protein L31, transcript variant 2	NA	-0.04078	8.35818	-1.02867	0.45253229	0.734964
71804	Mtfr2	mitochondrial fission regulator 2	NA	-0.15028	1.686734	-1.10979	0.45265918	0.735097
218877	Sema3g	sema domain, immunoglobulin domain (Ig), short bas	NA	-0.10136	2.605716	-1.07278	0.4528473	0.735329
26357	Abcg2	ATP binding cassette subfamily G member 2 (Junior	NA	0.055579	4.612687	1.039276	0.45296703	0.73545
170484	Nphs2	nephrosis 2, podocin, transcript variant X2	NA	0.338192	-0.63595	1.264171	0.45309677	0.735514
98314	D2hgdh	D-2-hydroxyglutarate dehydrogenase, transcript varia	NA	-0.074	4.291783	-1.05263	0.45309679	0.735514
386753	Dbpht2	DNA binding protein with his-thr domain	NA	-0.08853	2.987092	-1.06329	0.45320327	0.735518
27979	Eif3b	eukaryotic translation initiation factor 3, subunit B	NA	-0.0368	7.38697	-1.02584	0.45325578	0.735518
75764	Slx1b	SLX1 structure-specific endonuclease subunit homol	NA	0.052884	4.738164	1.037336	0.45329843	0.735518
16449	Jag1	jagged 1	NA	0.054024	4.649132	1.038156	0.45331778	0.735518
16392	Isl1	ISL1 transcription factor, LIM/homeodomain	NA	0.045712	5.108201	1.032192	0.45332562	0.735518
207212	Arhgef17	Rho guanine nucleotide exchange factor (GEF) 17	NA	-0.05005	5.921214	-1.0353	0.45337704	0.735529
67143	Ikzf5	IKAROS family zinc finger 5	NA	0.05855	4.935418	1.041419	0.45346326	0.735559
56382	Rab9	RAB9, member RAS oncogene family, transcript varia	NA	0.05629	5.166524	1.039788	0.45348644	0.735559
102631933	Gm30140	predicted gene, 30140, transcript variant X1	NA	-0.161	1.183266	-1.11806	0.45357089	0.735592
218518	Marveld2	MARVEL (membrane-associating) domain containing	NA	-0.13033	1.651602	-1.09455	0.45359708	0.735592
94180	Acsbg1	acyl-CoA synthetase bubblegum family member 1, tra	NA	-0.06122	4.194919	-1.04335	0.453674	0.735644
73692	Cplane1	ciliogenesis and planar polarity effector 1, transcript v	NA	0.054361	6.133366	1.038399	0.45375303	0.735698
66449	Pam16	presequence translocase-asssociated motor 16 hom	NA	-0.05879	4.113415	-1.04159	0.4538018	0.735704
60409	Trappc4	trafficking protein particle complex 4	NA	0.037933	6.261657	1.026642	0.45395623	0.735881
11980	Atp8a1	ATPase, aminophospholipid transporter (APLT), clas	NA	0.048109	6.69813	1.033909	0.45425458	0.736187
791292	Gm9936	predicted gene 9936	NA	0.127439	2.130504	1.092353	0.45425951	0.736187
22719	Zfp61	zinc finger protein 61	NA	-0.05076	4.80321	-1.03581	0.45434386	0.736187
217340	Rnf157	ring finger protein 157	NA	-0.03478	7.387674	-1.0244	0.45444062	0.736187
52829	Lurap1l	leucine rich adaptor protein 1-like	NA	0.105206	2.448392	1.075648	0.45445	0.736187
19206	Ptch1	patched 1, transcript variant 1	NA	0.05819	5.937228	1.041158	0.45447639	0.736187
105243582	Gm39467	predicted gene, 39467	NA	-0.27489	-0.31229	-1.2099	0.45449739	0.736187
12757	Clta	clathrin, light polypeptide (Lca), transcript variant 3	NA	-0.03563	7.496899	-1.025	0.45450703	0.736187
103537	Mbtd1	mbt domain containing 1, transcript variant X45	NA	0.043703	6.99543	1.030756	0.45459018	0.736249
15107	Hadh	hydroxyacyl-Coenzyme A dehydrogenase	NA	-0.05283	4.721385	-1.03729	0.45465341	0.736278
230767	Iqcc	IQ motif containing C	NA	-0.06645	3.939211	-1.04714	0.45471264	0.736301
100039786	Gm2423	predicted gene 2423	NA	0.100417	3.507116	1.072084	0.45476197	0.736307
64685	Nmi	N-myc (and STAT) interactor, transcript variant 3	NA	0.149134	1.291468	1.108904	0.45487246	0.736398
66995	Zcchc18	zinc finger, CCHC domain containing 18, transcript va	NA	0.042048	7.769792	1.029574	0.45493721	0.736398
66569	Gdpd1	glycerophosphodiester phosphodiesterase domain co	NA	0.040053	7.022853	1.028151	0.45495355	0.736398
68080	Gpn3	GPN-loop GTPase 3	NA	0.084466	3.914146	1.060295	0.45504486	0.736465
59288	Dctn5	dynactin 5	NA	0.037101	6.533649	1.02605	0.45512542	0.736465
170826	Ppargc1b	peroxisome proliferative activated receptor, gamma, (	NA	0.098362	3.058337	1.070558	0.45514491	0.736465
68653	Samm50	SAMM50 sorting and assembly machinery componer	NA	0.040992	6.259119	1.028821	0.4551762	0.736465
106068	Slc45a4	solute carrier family 45, member 4, transcript variant	NA	-0.05059	5.09127	-1.03569	0.45523908	0.736493
319322	Sf3b2	splicing factor 3b, subunit 2, transcript variant 1	NA	-0.03782	8.17715	-1.02656	0.45537218	0.736635
18010	Neu1	neuraminidase 1	NA	0.060659	4.606398	1.042942	0.45550276	0.736773
104099	Itga9	integrin alpha 9, transcript variant 2	NA	-0.08541	3.137988	-1.06099	0.45580673	0.737179
66278	Smim20	small integral membrane protein 20	NA	-0.08164	3.436264	-1.05822	0.4558442	0.737179

100169871	Gm10910	predicted gene 10910	NA	0.365793	-0.12713	1.28859	0.45593969	0.73726
72486	Obi1	ORC ubiquitin ligase 1	NA	-0.052	5.080477	-1.0367	0.45600106	0.737286
665563	Mthfd2l	methylenetetrahydrofolate dehydrogenase (NADP+ d	NA	-0.05044	5.170008	-1.03558	0.45606724	0.737294
115489130	LOC115489130	uncharacterized LOC115489130	NA	-0.24733	0.170167	-1.18701	0.45612213	0.737294
118567790	LOC118567790	uncharacterized LOC118567790	NA	-0.2379	-0.05894	-1.17928	0.45614199	0.737294
380664	Lemd3	LEM domain containing 3	NA	0.05319	5.262098	1.037557	0.45636985	0.737526
58523	Elp2	elongator acetyltransferase complex subunit 2, transc	NA	-0.03599	7.123876	-1.02526	0.45637598	0.737526
16504	Kcnc3	potassium voltage gated channel, Shaw-related subfa	NA	-0.0453	5.871977	-1.03189	0.4564978	0.73765
76294	Asb5	ankyrin repeat and SOCs box-containing 5, transcript	NA	0.330518	-0.09429	1.257465	0.45676787	0.738013
68251	Babam1	BRISC and BRCA1 A complex member 1, transcript v	NA	-0.04737	5.777637	-1.03338	0.4568404	0.738057
19082	Prkag1	protein kinase, AMP-activated, gamma 1 non-catalyti	NA	-0.04757	5.404162	-1.03352	0.45694455	0.738151
100037282	Rsph3b	radial spoke 3B homolog (Chlamydomonas)	NA	-0.0525	4.733753	-1.03706	0.45698971	0.738151
72805	Zfp839	zinc finger protein 839, transcript variant 2	NA	0.081102	3.308985	1.057826	0.45715277	0.738237
67862	2310033P09Rik	RIKEN cDNA 2310033P09 gene	NA	-0.08023	3.555297	-1.05719	0.45715787	0.738237
69149	Kbtbd3	kelch repeat and BTB (POZ) domain containing 3, tra	NA	0.113845	2.478249	1.082109	0.45717861	0.738237
67781	Ilf2	interleukin enhancer binding factor 2	NA	-0.04386	7.796497	-1.03087	0.45745927	0.738514
68147	Gar1	GAR1 ribonucleoprotein	NA	-0.07183	4.27279	-1.05105	0.45755176	0.738514
56218	Patz1	POZ (BTB) and AT hook containing zinc finger 1, tran	NA	-0.03935	6.351005	-1.02765	0.45764703	0.738514
54651	Usp27x	ubiquitin specific peptidase 27, X chromosome	NA	0.063567	4.418709	1.045046	0.45779298	0.738514
118567504	LOC118567504	uncharacterized LOC118567504, transcript variant X1	NA	-0.21964	-0.01109	-1.16444	0.45780278	0.738514
70650	Zcchc8	zinc finger, CCHC domain containing 8, transcript var	NA	-0.04763	5.975643	-1.03356	0.45787629	0.738514
211550	Tifa	TRAF-interacting protein with forkhead-associated do	NA	-0.13374	1.637143	-1.09714	0.45790111	0.738514
227715	Exosc2	exosome component 2	NA	-0.06121	4.803155	-1.04334	0.45792951	0.738514
102643128	Gm38660	predicted gene, 38660	NA	-0.13195	2.474166	-1.09578	0.45793756	0.738514
225896	Ubxn1	UBX domain protein 1, transcript variant 2	NA	0.041735	6.094522	1.029351	0.45797114	0.738514
97863	Fam8a1	family with sequence similarity 8, member A1, transcr	NA	0.048314	7.408826	1.034056	0.45800063	0.738514
77065	Ints7	integrator complex subunit 7, transcript variant X1	NA	-0.04439	5.943877	-1.03125	0.45802576	0.738514
12557	Cdh17	cadherin 17	NA	0.231652	-0.02661	1.174178	0.45804588	0.738514
170787	Hdac10	histone deacetylase 10, transcript variant 3	NA	-0.06825	4.309907	-1.04844	0.45805395	0.738514
94353	Hmgn3	high mobility group nucleosomal binding domain 3, tra	NA	0.040037	6.015344	1.02814	0.45805467	0.738514
213350	Gatd1	glutamine amidotransferase like class 1 domain conta	NA	-0.04651	4.99952	-1.03277	0.45807659	0.738514
66971	Cdk5rap1	CDK5 regulatory subunit associated protein 1	NA	-0.0982	3.291721	-1.07044	0.45813446	0.738534
67675	Cuta	cutA divalent cation tolerance homolog, transcript var	NA	0.061432	4.287303	1.043501	0.45834637	0.738656
20042	Rps12	ribosomal protein S12	NA	0.048267	8.231945	1.034022	0.45837421	0.738656
55991	Panx1	pannexin 1	NA	0.050057	6.032265	1.035306	0.45840552	0.738656
100302688	Gm17455	predicted gene, 17455, transcript variant X3	NA	-0.22012	0.109223	-1.16483	0.45847145	0.738656
106759	Ticam1	toll-like receptor adaptor molecule 1	NA	-0.13558	2.183853	-1.09853	0.45847218	0.738656
105245991	Gm41358	predicted gene, 41358	NA	0.128341	2.053487	1.093036	0.45852538	0.738656
75660	Lin37	lin-37 homolog (C. elegans), transcript variant 1	NA	0.06438	4.009076	1.045636	0.45852764	0.738656
18546	Pcp4	Purkinje cell protein 4	NA	0.050344	5.78538	1.035512	0.45861503	0.738678
19063	Ppt1	palmitoyl-protein thioesterase 1	NA	0.039898	5.818667	1.028041	0.45867683	0.738678
14004	Chchd2	coiled-coil-helix-coiled-coil-helix domain containing 2	NA	0.040453	6.961188	1.028437	0.4586777	0.738678
100073351	Yy2	Yy2 transcription factor	NA	0.120718	2.473533	1.087276	0.45886134	0.738715
328479	Gm5089	predicted gene 5089, transcript variant 2	NA	-0.17942	1.617641	-1.13243	0.45890196	0.738715
100502940	Colca2	COLCA2 homolog	NA	-0.26919	-0.44629	-1.20513	0.45898578	0.738715
69934	Trmt10b	tRNA methyltransferase 10B, transcript variant X5	NA	-0.06738	3.85424	-1.04781	0.45898621	0.738715
11858	Rnd2	Rho family GTPase 2	NA	0.04836	6.197095	1.034089	0.45904113	0.738715
22608	Ybx1	Y box protein 1	NA	0.037801	9.924625	1.026548	0.45904684	0.738715
11853	Rhoc	ras homolog family member C, transcript variant 1	NA	0.051259	4.65189	1.036169	0.45910907	0.738715
78376	Sapcd1	suppressor APC domain containing 1, transcript varia	NA	0.255135	0.092081	1.193447	0.45912392	0.738715
78304	Naa38	N(alpha)-acetyltransferase 38, NatC auxiliary subunit	NA	0.090778	3.528056	1.064944	0.4591551	0.738715
75710	Rbm12	RNA binding motif protein 12, transcript variant 1	NA	0.043774	5.650763	1.030807	0.45917894	0.738715
229003	Helz2	helicase with zinc finger 2, transcriptional coactivator	NA	0.127731	1.781356	1.092574	0.45919994	0.738715
192289	Tmlhe	trimethyllysine hydroxylase, epsilon	NA	0.088672	2.931761	1.063391	0.45932594	0.738845
15437	Hoxd8	homeobox D8, transcript variant 3	NA	0.276326	-0.52717	1.211107	0.45944968	0.738928
105245032	Gm40543	predicted gene, 40543	NA	-0.22159	0.422245	-1.16601	0.45946837	0.738928
56030	Tmem131	transmembrane protein 131	NA	0.038647	6.966807	1.02715	0.45977849	0.739353
217365	Nploc4	NPL4 homolog, ubiquitin recognition factor, transcript	NA	-0.0464	6.271927	-1.03269	0.45982424	0.739354
23821	Bace1	beta-site APP cleaving enzyme 1, transcript variant X	NA	-0.03826	6.042999	-1.02687	0.46004367	0.739613
18584	Pde8a	phosphodiesterase 8A	NA	0.096052	2.771178	1.068845	0.46007662	0.739613
66887	Lonp2	lon peptidase 2, peroxisomal, transcript variant 1	NA	-0.04236	6.104398	-1.0298	0.46017045	0.739658
108167924	Gm11613	predicted gene 11613, transcript variant X1	NA	0.183858	0.739196	1.135918	0.4602228	0.739658
11432	Acp2	acid phosphatase 2, lysosomal, transcript variant 1	NA	0.047603	6.18109	1.033546	0.46027076	0.739658
110147	Ehmt2	euchromatic histone lysine N-methyltransferase 2, tra	NA	-0.0328	7.97226	-1.023	0.46030025	0.739658
234734	Aars	alanyl-tRNA synthetase	NA	-0.03691	7.476148	-1.02591	0.46034527	0.739658
234678	D230025D16Rik	RIKEN cDNA D230025D16 gene, transcript variant 2	NA	0.04875	4.934981	1.034368	0.46039554	0.739658
30839	Fbxw5	F-box and WD-40 domain protein 5, transcript variant	NA	-0.04426	6.116402	-1.03115	0.46042247	0.739658
66297	Pantr1	POU domain, class 3, transcription factor 3 adjacent	NA	0.054659	5.237907	1.038613	0.4606179	0.739899

209354	Eif2b1	eukaryotic translation initiation factor 2B, subunit 1 (a	NA	0.047828	5.257665	1.033708	0.46071656	0.739984
109222	Rarres1	retinoic acid receptor responder (tazarotene induced)	NA	0.243131	-0.11222	1.183559	0.46090258	0.740143
104681	Slc16a6	solute carrier family 16 (monocarboxylic acid transpo	NA	0.075049	3.568369	1.053397	0.46090667	0.740143
22596	Xrcc5	X-ray repair complementing defective repair in Chines	NA	-0.05431	4.488535	-1.03836	0.46103857	0.740184
216874	Camta2	calmodulin binding transcription activator 2, transcript	NA	0.04461	5.640257	1.031405	0.46116249	0.740184
13200	Ddost	dolichyl-di-phosphooligosaccharide-protein glycotrans	NA	-0.03385	6.950283	-1.02374	0.46117803	0.740184
101148	Bmt2	base methyltransferase of 25S rRNA 2	NA	0.048747	5.392638	1.034366	0.46121265	0.740184
52348	Vps37a	vacuolar protein sorting 37A, transcript variant X3	NA	0.04715	5.527176	1.033222	0.46123678	0.740184
74238	Mterf2	mitochondrial transcription termination factor 2, trans	NA	0.06591	4.072511	1.046745	0.46124562	0.740184
21414	Tcf7	transcription factor 7, T cell specific, transcript varian	NA	0.097118	3.354727	1.069635	0.46128808	0.740184
20222	Sf3a2	splicing factor 3a, subunit 2	NA	0.043711	6.050337	1.030762	0.4613312	0.740184
66489	Rpl35	ribosomal protein L35	NA	0.046168	8.147958	1.032519	0.46134121	0.740184
243910	Nfkbid	nuclear factor of kappa light polypeptide gene enhan	NA	0.18364	1.067571	1.135746	0.46138881	0.740187
231760	Rimbp2	RIMS binding protein 2, transcript variant 2	NA	-0.05579	5.680991	-1.03943	0.46152434	0.740286
271813	Agbl2	ATP/GTP binding protein-like 2, transcript variant X1	NA	-0.24969	0.379011	-1.18895	0.46154145	0.740286
102238433	Gm28042	predicted gene, 28042	NA	-0.19309	2.563567	-1.14321	0.46161324	0.740315
107035	Fbxo38	F-box protein 38, transcript variant X8	NA	0.041795	5.754109	1.029394	0.4616593	0.740315
239114	Il17d	interleukin 17D	NA	0.107877	2.891939	1.077641	0.46169587	0.740315
19707	Reps1	RalBP1 associated Eps domain containing protein, tr	NA	0.040714	6.192777	1.028623	0.46183661	0.740456
67422	Dhdds	dehydrodolichyl diphosphate synthase, transcript vari	NA	-0.04792	5.819915	-1.03377	0.46189802	0.740456
83410	Cstf2t	cleavage stimulation factor, 3' pre-RNA subunit 2, tau	NA	0.041282	6.701191	1.029028	0.46195764	0.740456
16651	Sspn	sarcospan, transcript variant 2	NA	-0.08942	3.132894	-1.06394	0.46198219	0.740456
105246961	AB010352	cDNA sequence AB010352	NA	-0.102	2.710587	-1.07326	0.46204935	0.740456
232078	Thnsl2	threonine synthase-like 2 (bacterial), transcript varian	NA	0.163674	1.233632	1.120136	0.46207102	0.740456
20716	Serpina3n	serine (or cysteine) peptidase inhibitor, clade A, mem	NA	0.264602	-0.05711	1.201304	0.46213033	0.740456
215748	Cnksr3	Cnksr family member 3, transcript variant X1	NA	0.105626	2.539138	1.075961	0.46220182	0.740456
18759	Prkci	protein kinase C, iota	NA	0.044996	5.817338	1.031681	0.46226833	0.740456
20588	Smarcc1	SWI/SNF related, matrix associated, actin dependent	NA	-0.04436	7.532278	-1.03122	0.46227352	0.740456
77609	Ccdc151	coiled-coil domain containing 151, transcript variant 2	NA	-0.10252	2.390119	-1.07365	0.4622847	0.740456
12296	Cacnb2	calcium channel, voltage-dependent, beta 2 subunit,	NA	0.072117	4.182398	1.051258	0.46239661	0.740499
105246529	Gm41804	predicted gene, 41804	NA	-0.11159	2.629529	-1.08042	0.46240232	0.740499
225131	Wac	WW domain containing adaptor with coiled-coil, trans	NA	0.039366	7.158801	1.027662	0.46250908	0.740527
232400	A2ml1	alpha-2-macroglobulin like 1	NA	-0.25465	0.432847	-1.19305	0.46252716	0.740527
13617	Ednra	endothelin receptor type A	NA	0.074909	3.554864	1.053295	0.46255663	0.740527
140629	Ubox5	U box domain containing 5, transcript variant 1	NA	-0.08013	3.714178	-1.05711	0.46278986	0.740772
66990	Tmem134	transmembrane protein 134, transcript variant 2	NA	0.070073	4.024563	1.04977	0.46280054	0.740772
319888	Oacyl	O-acyltransferase like	NA	0.222171	0.29205	1.166488	0.46299478	0.740906
319583	Lig4	ligase IV, DNA, ATP-dependent, transcript variant 2	NA	-0.05649	4.584878	-1.03993	0.46299837	0.740906
637776	Zfp977	zinc finger protein 977	NA	0.231925	0.046088	1.174401	0.46302881	0.740906
319719	Simc1	SUMO-interacting motifs containing 1, transcript varia	NA	-0.04776	5.312225	-1.03366	0.46306638	0.740906
115486069	Gm23119	small Cajal body-specific RNA 17	NA	0.136429	1.776059	1.099181	0.46314054	0.740924
72278	Ccpg1	cell cycle progression 1, transcript variant 1	NA	-0.04775	5.598699	-1.03365	0.46316852	0.740924
100043761	Gm14399	predicted gene 14399, transcript variant X2	NA	-0.06812	4.055049	-1.04835	0.46326632	0.741008
18124	Nr4a3	nuclear receptor subfamily 4, group A, member 3, tra	NA	0.061121	5.098699	1.043276	0.46336197	0.741019
78251	Zfp712	zinc finger protein 712	NA	0.091422	3.386837	1.06542	0.46336436	0.741019
268451	Rab11fip4	RAB11 family interacting protein 4 (class II), transcrip	NA	-0.04994	7.500591	-1.03522	0.46351795	0.741191
56040	Rplp1	ribosomal protein, large, P1	NA	0.040443	8.197764	1.02843	0.46363272	0.741255
71810	Ranbp3	RAN binding protein 3, transcript variant X5	NA	-0.03995	6.551949	-1.02808	0.46364866	0.741255
65972	Ifi30	interferon gamma inducible protein 30	NA	0.077223	3.380783	1.054985	0.46371411	0.741258
71393	Kctd6	potassium channel tetramerisation domain containing	NA	0.060887	4.669248	1.043107	0.46379407	0.741258
75739	Mpp7	membrane protein, palmitoylated 7 (MAGUK p55 sub	NA	-0.10335	2.935172	-1.07426	0.46382803	0.741258
100044193	Gm20939	predicted gene, 20939	NA	-0.20485	0.46265	-1.15257	0.46383261	0.741258
21665	Tdg	thymine DNA glycosylase, transcript variant X1	NA	-0.0345	7.545231	-1.0242	0.46422534	0.741806
98053	Gtf2f1	general transcription factor IIF, polypeptide 1	NA	-0.05334	5.891458	-1.03767	0.46426703	0.741806
105243058	Gm39090	predicted gene, 39090, transcript variant X1	NA	-0.19939	0.259649	-1.14821	0.46434828	0.741857
12609	Cebpd	CCAAT/enhancer binding protein (C/EBP), delta	NA	0.160221	1.257822	1.117458	0.46442294	0.741857
228866	Pcif1	phosphorylated CTD interacting factor 1, transcript va	NA	0.044413	5.778596	1.031263	0.46443588	0.741857
13595	Ebp	phenylalkylamine Ca2+ antagonist (emopamil) bindin	NA	-0.07418	3.553065	-1.05276	0.46458394	0.742021
58245	Gpr180	G protein-coupled receptor 180, transcript variant X1	NA	0.062097	4.340162	1.043982	0.46464845	0.742051
30055	Timm13	translocase of inner mitochondrial membrane 13	NA	-0.06703	4.965881	-1.04756	0.46469785	0.742054
27225	Ddx24	DEAD box helicase 24, transcript variant 2	NA	0.038999	6.756893	1.027401	0.46474138	0.742054
54563	Nup210	nucleoporin 210	NA	0.042286	5.694397	1.029744	0.46483037	0.742123
102247	Gpat4	glycerol-3-phosphate acyltransferase 4	NA	-0.03594	6.9591	-1.02522	0.46506514	0.742425
13397	Dlx6os2	distal-less homeobox 6, opposite strand 2	NA	-0.13538	2.065234	-1.09838	0.46518703	0.742476
50759	Fbxo16	F-box protein 16, transcript variant 1	NA	0.071204	3.835081	1.050593	0.46518815	0.742476
224132	Slc49a4	solute carrier family 49 member 4	NA	0.048831	6.266928	1.034427	0.46541617	0.742767
217219	Fam171a2	family with sequence similarity 171, member A2	NA	-0.05164	7.437211	-1.03644	0.465604	0.742932
269700	Hectd4	HECT domain E3 ubiquitin protein ligase 4, transcript	NA	0.039532	7.794769	1.02778	0.46568415	0.742932

67856	Echdc3	enoyl Coenzyme A hydratase domain containing 3	NA	-0.25052	-0.30952	-1.18964	0.46568808	0.742932
17112	Tm4sf1	transmembrane 4 superfamily member 1, transcript v	NA	0.087869	2.838308	1.062799	0.46571731	0.742932
268480	Rapgefl1	Rap guanine nucleotide exchange factor (GEF)-like 1	NA	-0.05585	5.243176	-1.03947	0.46574783	0.742932
75316	Taf1d	TATA-box binding protein associated factor, RNA pol	NA	0.046294	5.687551	1.032609	0.46586276	0.74296
216867	Slc16a11	solute carrier family 16 (monocarboxylic acid transpo	NA	-0.20175	0.68056	-1.15009	0.46587865	0.74296
14867	Gstm6	glutathione S-transferase, mu 6, transcript variant 4	NA	0.104067	2.300979	1.074799	0.46590218	0.74296
102635337	Gm32703	predicted gene, 32703, transcript variant X1	NA	-0.18195	0.479214	-1.13441	0.46599814	0.743006
225908	Myrf	myelin regulatory factor, transcript variant X10	NA	0.131048	1.596276	1.095089	0.46604913	0.743006
52717	Anapc16	anaphase promoting complex subunit 16, transcript v	NA	0.044883	5.210866	1.031599	0.46606797	0.743006
13040	Ctss	cathepsin S, transcript variant 2	NA	0.092512	2.812162	1.066225	0.46624519	0.743143
320022	Terb1	telomere repeat binding bouquet formation protein 1,	NA	0.256941	0.100885	1.194942	0.46624761	0.743143
20930	Surf1	surfeit gene 1, transcript variant X6	NA	0.051875	4.857785	1.036611	0.46629129	0.743143
18742	Pitx3	paired-like homeodomain transcription factor 3	NA	-0.27555	-0.22786	-1.21046	0.46637598	0.743205
216019	Hkdc1	hexokinase domain containing 1	NA	-0.22807	-0.09651	-1.17127	0.46644826	0.743248
12192	Zfp36l1	zinc finger protein 36, C3H type-like 1	NA	-0.04825	6.318337	-1.03401	0.4665224	0.743293
15191	Hdgf	heparin binding growth factor, transcript variant 1	NA	-0.03879	7.829766	-1.02725	0.46658052	0.743313
330998	Ankrd34c	ankyrin repeat domain 34C, transcript variant X2	NA	0.253066	-0.34113	1.191737	0.46663839	0.743332
59041	Stk25	serine/threonine kinase 25 (yeast), transcript variant)	NA	0.03683	7.491861	1.025857	0.46682564	0.743554
26992	Brd7	bromodomain containing 7, transcript variant 1	NA	-0.04121	6.48337	-1.02898	0.46686862	0.743554
75288	Slc35f4	solute carrier family 35, member F4	NA	0.121056	2.840108	1.087531	0.4669512	0.743612
239618	Pdzrn4	PDZ domain containing RING finger 4, transcript vari	NA	0.061752	4.716455	1.043732	0.46703346	0.743616
664949	Gm7420	predicted gene 7420	NA	-0.11411	2.183938	-1.08231	0.46704526	0.743616
102638167	Gm38506	predicted gene, 38506	NA	-0.18991	0.413799	-1.14069	0.46711556	0.743656
12729	Clns1a	chloride channel, nucleotide-sensitive, 1A	NA	0.038417	6.339642	1.026986	0.46726185	0.743816
51792	Ppp2r1a	protein phosphatase 2, regulatory subunit A, alpha	NA	0.034739	9.024251	1.024371	0.46776672	0.744432
71751	Map3k13	mitogen-activated protein kinase kinase kinase 13, tr	NA	-0.05488	5.079794	-1.03877	0.46784802	0.744432
118567559	LOC118567559	uncharacterized LOC118567559	NA	-0.25815	1.635206	-1.19595	0.46788843	0.744432
72325	Vps9d1	VPS9 domain containing 1, transcript variant 2	NA	-0.04551	5.804229	-1.03205	0.46794265	0.744432
14588	Gfra4	glial cell line derived neurotrophic factor family recept	NA	0.083867	3.683465	1.059855	0.46794724	0.744432
67693	Hypk	huntingtin interacting protein K	NA	-0.05262	5.718698	-1.03714	0.46797167	0.744432
19775	Xpr1	xenotropic and polytropic retrovirus receptor 1	NA	0.035383	8.619484	1.024829	0.46800679	0.744432
192120	Bspry	B-box and SPRY domain containing, transcript variar	NA	-0.28876	-0.38182	-1.22159	0.46805583	0.744432
245886	Ankrd27	ankyrin repeat domain 27 (VPS9 domain), transcript v	NA	0.053265	5.225964	1.037611	0.46806071	0.744432
14056	Ezh2	enhancer of zeste 2 polycomb repressive complex 2 :	NA	-0.0447	6.7498	-1.03147	0.46811122	0.74444
66578	Mis18a	MIS18 kinetochore protein A	NA	-0.07566	3.771794	-1.05385	0.46823536	0.744514
239319	Card6	caspase recruitment domain family, member 6, transc	NA	-0.18762	1.168257	-1.13888	0.46827716	0.744514
18045	Nfyb	nuclear transcription factor-Y beta	NA	-0.04419	6.114722	-1.03111	0.46829532	0.744514
105083	Pelo	pelota mRNA surveillance and ribosome rescue facto	NA	-0.05784	4.248456	-1.04091	0.46845292	0.744557
13043	Cttn	cortactin, transcript variant 2	NA	-0.03632	7.567189	-1.0255	0.46845684	0.744557
230145	Galnt12	polypeptide N-acetylgalactosaminyltransferase 12, tr	NA	-0.1753	0.819657	-1.1292	0.4684955	0.744557
328643	Vwa5b2	von Willebrand factor A domain containing 5B2, trans	NA	0.068201	4.622402	1.048409	0.46852521	0.744557
115486294	Gm51438	predicted gene, 51438	NA	-0.18644	1.494661	-1.13795	0.46856514	0.744557
23924	Katna1	katanin p60 (ATPase-containing) subunit A1	NA	0.059115	4.410065	1.041827	0.46864037	0.744557
53892	Ppm1d	protein phosphatase 1D magnesium-dependent, delt	NA	0.051242	5.035642	1.036157	0.46864243	0.744557
67834	Idh3a	isocitrate dehydrogenase 3 (NAD+) alpha	NA	0.036269	6.700585	1.025458	0.46893986	0.744957
69663	Ddx51	DEAD box helicase 51, transcript variant X1	NA	0.064096	4.722968	1.04543	0.46905241	0.745063
20639	Snrpb2	U2 small nuclear ribonucleoprotein B	NA	0.043238	5.785833	1.030424	0.46909905	0.745064
69821	Mterf4	mitochondrial transcription termination factor 4, transc	NA	-0.07335	3.928648	-1.05215	0.4692247	0.745167
233805	Dcun1d3	DCN1, defective in cullin neddylation 1, domain conta	NA	-0.05397	4.939475	-1.03812	0.46925553	0.745167
78249	Adgrf4	adhesion G protein-coupled receptor F4, transcript va	NA	-0.24967	0.128108	-1.18893	0.4694145	0.745347
22248	Unc119	unc-119 lipid binding chaperone, transcript variant 4	NA	0.051153	5.405137	1.036093	0.46951014	0.745358
68090	Yif1a	Yip1 interacting factor homolog A (S. cerevisiae)	NA	-0.07935	3.798784	-1.05654	0.46951342	0.745358
50768	Dlc1	deleted in liver cancer 1, transcript variant 2	NA	0.054392	4.898893	1.038421	0.46980897	0.745755
240174	Thada	thyroid adenoma associated, transcript variant X2	NA	-0.06295	4.066447	-1.0446	0.46989352	0.745816
434423	Dppa5a	developmental pluripotency associated 5A	NA	0.226805	0.629619	1.17024	0.46994974	0.745833
116914	Slc19a2	solute carrier family 19 (thiamine transporter), membe	NA	0.091533	3.172152	1.065502	0.47011322	0.746019
71785	Pdgfd	platelet-derived growth factor, D polypeptide, transcri	NA	0.15572	1.43268	1.113977	0.4702358	0.74612
214897	Csnk1g1	casein kinase 1, gamma 1	NA	0.038684	5.991862	1.027177	0.47026857	0.74612
14465	Gata6	GATA binding protein 6	NA	0.274079	-0.37939	1.209222	0.47032351	0.746128
12703	Socs1	suppressor of cytokine signaling 1, transcript variant	NA	-0.19991	0.233374	-1.14863	0.47039377	0.746128
12946	Cr1l	complement component (3b/4b) receptor 1-like, trans	NA	0.06132	4.096291	1.04342	0.47041086	0.746128
627191	Syndig1l	synapse differentiation inducing 1 like	NA	0.076569	3.747838	1.054508	0.47062737	0.746242
14268	Fn1	fibronectin 1, transcript variant 4	NA	0.042545	7.39547	1.029929	0.47064812	0.746242
70450	Unc13d	unc-13 homolog D, transcript variant X1	NA	-0.2365	-0.10552	-1.17813	0.47067034	0.746242
239647	Pced1b	PC-esterase domain containing 1B, transcript variant	NA	-0.07329	3.483473	-1.05212	0.47070901	0.746242
15481	Hspa8	heat shock protein 8, transcript variant 1	NA	-0.03808	11.148	-1.02674	0.47075343	0.746242
213464	Rbbp5	retinoblastoma binding protein 5, histone lysine meth	NA	-0.04834	5.637613	-1.03408	0.47075788	0.746242
240034	Zfp760	zinc finger protein 760	NA	0.055913	4.869001	1.039516	0.47093524	0.74645

109151	Chd9	chromodomain helicase DNA binding protein 9, trans	NA	0.044011	6.284605	1.030976	0.47105718	0.746513
66505	Zmynd11	zinc finger, MYND domain containing 11, transcript va	NA	0.032994	7.986576	1.023133	0.47106637	0.746513
115487387	Gm48893	predicted gene, 48893	NA	-0.11613	2.155633	-1.08382	0.47118388	0.746626
16590	Kit	KIT proto-oncogene receptor tyrosine kinase, transcri	NA	-0.04431	6.222863	-1.03119	0.47144925	0.746974
27049	Etv3	ets variant 3, transcript variant 2	NA	0.058535	4.34095	1.041408	0.47176635	0.747312
97159	A430005L14Rik	RIKEN cDNA A430005L14 gene, transcript variant 1	NA	-0.07397	3.653498	-1.05261	0.47176686	0.747312
18576	Pde3b	phosphodiesterase 3B, cGMP-inhibited	NA	0.072496	4.448124	1.051534	0.47188991	0.747312
108012	Ap1s2	adaptor-related protein complex 1, sigma 2 subunit, t	NA	0.035762	6.748329	1.025098	0.47191741	0.747312
66853	Pnpla2	patatin-like phospholipase domain containing 2, trans	NA	0.065959	3.76294	1.046781	0.47193424	0.747312
106894	Hmgxb3	HMG box domain containing 3, transcript variant 1	NA	-0.04594	5.412602	-1.03235	0.47193809	0.747312
19766	Ripk1	receptor (TNFRSF)-interacting serine-threonine kinas	NA	-0.07293	3.550537	-1.05185	0.4720076	0.747319
28036	Larp7	La ribonucleoprotein domain family, member 7	NA	-0.05372	5.237987	-1.03794	0.47209295	0.747319
433375	Creg1	cellular repressor of E1A-stimulated genes 1, transcri	NA	0.067455	3.824407	1.047867	0.47210815	0.747319
51786	Cpsf2	cleavage and polyadenylation specific factor 2	NA	0.037301	6.166743	1.026192	0.47226304	0.747319
212124	Cfap46	cilia and flagella associated protein 46	NA	-0.11742	1.934012	-1.08479	0.47226683	0.747319
71999	Fbxo22	F-box protein 22	NA	-0.04773	6.034693	-1.03364	0.47229937	0.747319
115489346	Gm52485	predicted gene, 52485	NA	-0.24135	0.382449	-1.1821	0.47230598	0.747319
75623	Tex30	testis expressed 30, transcript variant 3	NA	-0.07677	3.757858	-1.05466	0.47231012	0.747319
226861	Hhat	hedgehog acyltransferase	NA	-0.14387	1.301075	-1.10486	0.47241671	0.747415
19324	Rab1a	RAB1A, member RAS oncogene family	NA	0.034745	8.182375	1.024375	0.47248912	0.747457
210529	Mettl14	methyltransferase like 14	NA	-0.0502	5.006655	-1.03541	0.47267551	0.747679
11482	Acvrl1	activin A receptor, type II-like 1, transcript variant 4	NA	0.074406	3.70688	1.052927	0.47274011	0.747709
93716	Pcdhga8	protocadherin gamma subfamily A, 8	NA	0.068403	4.165217	1.048555	0.47291653	0.747915
105247116	Gm42274	predicted gene, 42274	NA	-0.23377	-0.13804	-1.1759	0.47326164	0.748388
15078	H3f3a	H3.3 histone A	NA	-0.05801	10.0057	-1.04103	0.47344879	0.748482
16517	Kcnj16	potassium inwardly-rectifying channel, subfamily J, m	NA	0.133512	1.466408	1.096961	0.47345369	0.748482
232679	Zc3hc1	zinc finger, C3HC type 1, transcript variant 1	NA	0.049534	5.218599	1.03493	0.47349086	0.748482
22239	Ugt8a	UDP galactosyltransferase 8A, transcript variant 1	NA	0.126663	2.308085	1.091765	0.47350494	0.748482
100041585	Amd2	S-adenosylmethionine decarboxylase 2	NA	0.546191	-1.69974	1.460225	0.47361221	0.748549
170461	Stard6	StAR-related lipid transfer (START) domain containin	NA	0.171771	0.703635	1.12644	0.47363963	0.748549
67464	Entpd4	ectonucleoside triphosphate diphosphohydrolase 4, t	NA	0.618668	-1.54791	1.535456	0.47377714	0.748694
217738	Ism2	isthmin 2	NA	-0.20113	0.602702	-1.1496	0.47386336	0.748757
70448	Atad3aos	ATPase family, AAA domain containing 3A, opposite	NA	0.215893	-0.12544	1.161423	0.47396355	0.748843
24017	Rnf13	ring finger protein 13, transcript variant X1	NA	0.043166	5.55314	1.030372	0.47410666	0.748996
118567547	LOC118567547	uncharacterized LOC118567547	NA	-0.24198	-0.15467	-1.18261	0.47429756	0.749208
381201	Ap5b1	adaptor-related protein complex 5, beta 1 subunit, tra	NA	0.151831	1.035226	1.110979	0.4743819	0.749208
80912	Pum1	pumilio RNA-binding family member 1, transcript vari	NA	-0.03858	7.420208	-1.0271	0.4744137	0.749208
442805	D130007C19Rik	RIKEN cDNA D130007C19 gene, transcript variant X	NA	-0.13437	2.349594	-1.09762	0.47449156	0.749208
18105	Nqo2	N-ribosyldihydronicotinamide quinone reductase 2, tr	NA	-0.09947	2.791684	-1.07138	0.47453476	0.749208
69367	Glrx2	glutaredoxin 2 (thioltransferase), transcript variant 2	NA	-0.04942	5.861823	-1.03485	0.47454217	0.749208
16177	Il1r1	interleukin 1 receptor, type I, transcript variant X12	NA	-0.13679	1.188977	-1.09946	0.47456305	0.749208
84094	Plvap	plasmalemma vesicle associated protein	NA	-0.12221	2.181614	-1.0884	0.47490064	0.749669
64383	Sirt2	sirtuin 2, transcript variant 2	NA	-0.03864	6.66558	-1.02715	0.47504099	0.749798
243529	H1f10	H1.10 linker histone	NA	-0.05594	6.129323	-1.03954	0.47520287	0.749798
112405	Egln1	egl-9 family hypoxia-inducible factor 1, transcript vari	NA	0.044096	6.739938	1.031037	0.47521655	0.749798
381229	Cfap58	cilia and flagella associated protein 58	NA	0.286076	-0.33695	1.219319	0.47525607	0.749798
26896	Med14	mediator complex subunit 14, transcript variant 1	NA	-0.04121	5.937962	-1.02898	0.47533579	0.749798
76793	Snip1	Smad nuclear interacting protein 1, transcript variant	NA	-0.05171	4.753073	-1.03649	0.47535944	0.749798
72747	Ttc39c	tetratricopeptide repeat domain 39C	NA	-0.06149	4.290902	-1.04354	0.47538308	0.749798
17470	Cd200	CD200 antigen, transcript variant 1	NA	0.032077	7.849309	1.022483	0.47539153	0.749798
12371	Casp9	caspase 9, transcript variant 1	NA	-0.04203	5.405236	-1.02956	0.47542965	0.749798
230279	Tmem268	transmembrane protein 268, transcript variant X3	NA	0.094585	2.816581	1.067758	0.47544348	0.749798
436240	Foxr2	forkhead box R2	NA	0.189944	0.743651	1.140719	0.47562105	0.750005
72482	Acbd6	acyl-Coenzyme A binding domain containing 6, trans	NA	0.042285	5.51387	1.029744	0.47580825	0.750227
231583	Slc26a1	solute carrier family 26 (sulfate transporter), member	NA	0.117495	2.072996	1.08485	0.47591086	0.750227
381695	N4bp2l2	NEDD4 binding protein 2-like 2, transcript variant 2	NA	-0.04781	6.089138	-1.03369	0.47592464	0.750227
170638	Hpcal4	hippocalcin-like 4	NA	-0.05789	5.247517	-1.04094	0.47597591	0.750227
432839	Gprin2	G protein regulated inducer of neurite outgrowth 2, tra	NA	0.05059	5.53158	1.035689	0.47604589	0.750227
17169	Mark3	MAP/microtubule affinity regulating kinase 3, transcrip	NA	0.034847	7.218916	1.024448	0.47606397	0.750227
102632297	Gm30409	predicted gene, 30409	NA	-0.15688	1.755646	-1.11487	0.47609763	0.750227
27375	Tjp3	tight junction protein 3, transcript variant 2	NA	-0.11551	2.663598	-1.08336	0.47613029	0.750227
71971	Zswim1	zinc finger SWIM-type containing 1	NA	0.064862	4.08245	1.045985	0.47625532	0.750329
328971	Spink10	serine peptidase inhibitor, Kazal type 10, transcript va	NA	0.099113	2.23818	1.071115	0.47630163	0.750329
97287	Mtmr14	myotubularin related protein 14, transcript variant 1	NA	-0.04754	4.913834	-1.0335	0.47636879	0.750329
110750	Cse1l	chromosome segregation 1-like (S. cerevisiae)	NA	-0.0397	6.707081	-1.0279	0.47637982	0.750329
218963	Ubb-ps	ubiquitin B, pseudogene	NA	-0.06586	6.54984	-1.04671	0.47654069	0.75051
28064	Yipf3	Yip1 domain family, member 3, transcript variant X1	NA	0.047831	5.199508	1.03371	0.47666725	0.750576
100043911	Ppp4r1l-ps	protein phosphatase 4, regulatory subunit 1-like, pseu	NA	0.091736	2.84394	1.065652	0.47667471	0.750576

230235	Frrs1l	ferric-chelate reductase 1 like	NA	0.107593	3.811128	1.077429	0.47698507	0.750992
474156	Zbtb9	zinc finger and BTB domain containing 9	NA	-0.0597	4.164796	-1.04225	0.47706609	0.751047
18805	Pld1	phospholipase D1, transcript variant 2	NA	0.08466	3.117928	1.060438	0.47713015	0.751075
329777	Pigk	phosphatidylinositol glycan anchor biosynthesis, clas:	NA	0.043378	5.492412	1.030524	0.47720079	0.751113
93689	Lmod1	leiomodin 1 (smooth muscle)	NA	0.2896	-0.41608	1.222302	0.47729217	0.751175
269587	Epb41	erythrocyte membrane protein band 4.1, transcript va	NA	-0.04068	7.654944	-1.0286	0.47734005	0.751175
75689	Higd1b	HIG1 domain family, member 1B, transcript variant 2	NA	-0.13671	1.258172	-1.09939	0.47737846	0.751175
75416	Nop14	NOP14 nucleolar protein	NA	-0.04625	5.243172	-1.03258	0.47749266	0.75119
68521	Fam189b	family with sequence similarity 189, member B, transc	NA	-0.05778	4.930399	-1.04086	0.47752447	0.75119
433702	Ncbp1	nuclear cap binding protein subunit 1	NA	0.033292	6.641252	1.023344	0.47754868	0.75119
76263	Gstk1	glutathione S-transferase kappa 1	NA	-0.13079	1.802792	-1.09489	0.47761133	0.75119
110312	Pmch	pro-melanin-concentrating hormone	NA	0.148203	1.436273	1.108188	0.47761873	0.75119
30060	Meltf	melanotransferrin	NA	-0.37438	-0.62167	-1.29628	0.47771752	0.751202
228368	Slc35c1	solute carrier family 35, member C1, transcript varian	NA	-0.06118	4.048331	-1.04332	0.47786061	0.751202
381347	4930412O13Rik	RIKEN cDNA 4930412O13 gene	NA	-0.16496	1.351943	-1.12114	0.47788628	0.751202
19356	Rad17	RAD17 checkpoint clamp loader component, transcrip	NA	-0.05443	4.583065	-1.03845	0.47792087	0.751202
14403	Gabrd	gamma-aminobutyric acid (GABA) A receptor, subuni	NA	0.204042	-0.00121	1.151921	0.47803906	0.751202
72479	Hsdl2	hydroxysteroid dehydrogenase like 2, transcript varia	NA	0.051211	4.811429	1.036134	0.4781006	0.751202
213811	BC002059	cDNA sequence BC002059, transcript variant 2	NA	0.069007	3.817219	1.048995	0.47813013	0.751202
19231	Ptma	prothymosin alpha, transcript variant 1	NA	-0.03509	10.77692	-1.02462	0.47820794	0.751202
330050	Fam185a	family with sequence similarity 185, member A	NA	0.072941	3.650424	1.051859	0.47823076	0.751202
24136	Zeb2	zinc finger E-box binding homeobox 2, transcript vari	NA	-0.04671	7.474631	-1.0329	0.47824692	0.751202
53420	Syt5	synaptotagmin V, transcript variant 2	NA	-0.0369	6.18821	-1.02591	0.47827042	0.751202
231931	Gimap6	GTPase, IMAP family member 6	NA	0.104313	2.520885	1.074982	0.47827298	0.751202
69065	Chac1	ChaC, cation transport regulator 1	NA	0.159666	1.646413	1.117029	0.47830588	0.751202
19817	Rn7sk	RNA, 7SK, nuclear	NA	0.309211	4.158881	1.23903	0.47831766	0.751202
18631	Pex11a	peroxisomal biogenesis factor 11 alpha, transcript va	NA	-0.08664	3.390208	-1.06189	0.47831904	0.751202
80837	Rhoj	ras homolog family member J	NA	-0.0736	3.731643	-1.05234	0.47849491	0.751406
268706	Slc38a9	solute carrier family 38, member 9, transcript variant	NA	-0.05097	4.863404	-1.03596	0.47864794	0.751438
66333	Aqp11	aquaporin 11	NA	0.119722	2.316079	1.086526	0.47869245	0.751438
11487	Adam10	a disintegrin and metallopeptidase domain 10	NA	0.037701	6.9923	1.026477	0.47869924	0.751438
19933	Rpl21	ribosomal protein L21	NA	0.03811	8.957693	1.026768	0.47869968	0.751438
108100	Baiap2	brain-specific angiogenesis inhibitor 1-associated pro	NA	0.051897	5.167454	1.036627	0.47889232	0.751667
244551	Nanos3	nanos C2HC-type zinc finger 3, transcript variant 2	NA	0.206044	0.274352	1.153521	0.47914352	0.751989
66587	Fastk	Fas-activated serine/threonine kinase	NA	0.056614	5.442469	1.040022	0.47929984	0.752013
14873	Gsto1	glutathione S-transferase omega 1	NA	0.045584	5.435386	1.032101	0.47933097	0.752013
12752	Cln3	ceroid lipofuscinosis, neuronal 3, juvenile (Batten, Sp	NA	-0.06465	3.829068	-1.04583	0.47933537	0.752013
13172	Dbx1	developing brain homeobox 1	NA	-0.10562	2.665485	-1.07596	0.47935131	0.752013
22187	Ubb	ubiquitin B, transcript variant 2	NA	0.039331	10.58096	1.027637	0.47940182	0.752013
18431	Oca2	oculocutaneous albinism II, transcript variant 3	NA	0.207414	0.092342	1.154617	0.47943623	0.752013
16371	Irx1	Iroquois homeobox 1	NA	0.064844	4.353439	1.045972	0.47961212	0.752217
69288	Rhobtb1	Rho-related BTB domain containing 1, transcript vari	NA	0.062839	4.553721	1.04452	0.4796853	0.752259
110391	Qdpr	quinoid dihydropteridine reductase	NA	0.050534	5.701586	1.035648	0.47988699	0.752388
246313	Prokr2	prokineticin receptor 2	NA	0.088368	3.564664	1.063167	0.47996254	0.752388
17314	Mgmt	O-6-methylguanine-DNA methyltransferase, transcrip	NA	-0.24066	-0.0389	-1.18153	0.47998319	0.752388
231633	Tmem119	transmembrane protein 119	NA	-0.12999	2.186742	-1.09429	0.47999682	0.752388
553127	Rtl8b	retrotransposon Gag like 8B	NA	0.040574	6.627621	1.028523	0.48005562	0.752388
22348	Slc32a1	solute carrier family 32 (GABA vesicular transporter),	NA	-0.04579	5.860571	-1.03225	0.480073	0.752388
71909	Haus5	HAUS augmin-like complex, subunit 5	NA	0.065113	3.954564	1.046167	0.48009105	0.752388
15229	Foxd1	forkhead box D1	NA	0.09971	2.661873	1.071558	0.48014246	0.752396
654798	D030055H07Rik	RIKEN cDNA D030055H07 gene, transcript variant X	NA	-0.06827	4.41509	-1.04846	0.48025853	0.752505
54604	Pcnx	pecanex homolog	NA	0.039461	6.805392	1.027729	0.48040271	0.752638
192191	Med9	mediator complex subunit 9	NA	0.052606	4.572987	1.037137	0.48049571	0.752638
330812	Rnf150	ring finger protein 150	NA	0.05307	4.858723	1.03747	0.48054689	0.752638
213056	Fam126b	family with sequence similarity 126, member B, trans	NA	0.06737	4.793518	1.047805	0.48056271	0.752638
208727	Hdac4	histone deacetylase 4, transcript variant X4	NA	0.046016	5.342016	1.03241	0.48059887	0.752638
74385	Ap5m1	adaptor-related protein complex 5, mu 1 subunit, tran	NA	0.05582	4.690769	1.03945	0.48062052	0.752638
224105	Pak2	p21 (RAC1) activated kinase 2	NA	0.033967	7.009682	1.023823	0.48079264	0.752693
13207	Ddx5	DEAD box helicase 5	NA	0.03115	10.18805	1.021826	0.48081455	0.752693
67905	Ppm1m	protein phosphatase 1M, transcript variant 1	NA	-0.07898	3.250488	-1.05627	0.48085076	0.752693
93878	Pcdhb7	protocadherin beta 7	NA	0.072923	3.659673	1.051846	0.4808772	0.752693
19411	Rarg	retinoic acid receptor, gamma, transcript variant 2	NA	-0.07841	3.727451	-1.05585	0.48088691	0.752693
14812	Grin2b	glutamate receptor, ionotropic, NMDA2B (epsilon 2),	NA	0.047392	7.649583	1.033395	0.48101118	0.752815
18256	Oc90	otoconin 90, transcript variant X3	NA	0.232882	0.914311	1.17518	0.48109521	0.752874
18793	Plaur	plasminogen activator, urokinase receptor	NA	0.220599	0.016673	1.165217	0.48120815	0.752978
66824	Pycard	PYD and CARD domain containing	NA	-0.21633	0.480148	-1.16178	0.48137189	0.753162
382010	Cep44	centrosomal protein 44, transcript variant X12	NA	0.072261	4.298921	1.051363	0.48145614	0.753221
102637404	Gm26008	predicted gene, 26008, transcript variant X2	NA	-0.23622	-0.26815	-1.1779	0.4818013	0.753645

228139	P2rx3	purinergic receptor P2X, ligand-gated ion channel, 3	NA		0.117465	2.55884	1.084827	0.48190142	0.753645
118568133	LOC118568133	uncharacterized LOC118568133	NA		0.075575	3.647025	1.053781	0.4819989	0.753645
237781	Mief2	mitochondrial elongation factor 2	NA		-0.06997	3.996419	-1.04969	0.48201097	0.753645
24071	Synj2bp	synaptojanin 2 binding protein, transcript variant 3	NA		-0.06857	5.564029	-1.04868	0.48203871	0.753645
68725	1110032F04Rik	RIKEN cDNA 1110032F04 gene	NA		0.088171	2.696136	1.063022	0.48204435	0.753645
76477	Pcolce2	procollagen C-endopeptidase enhancer 2	NA		0.146133	1.059399	1.1066	0.48205121	0.753645
77945	Rpgrip1	retinitis pigmentosa GTPase regulator interacting pro	NA		-0.15384	1.012651	-1.11253	0.48218955	0.753697
235472	Prtg	protogenin	NA		-0.1263	1.506501	-1.09149	0.48219082	0.753697
19046	Ppp1cb	protein phosphatase 1 catalytic subunit beta	NA		0.043026	8.50021	1.030273	0.48235428	0.753697
78670	Plekhj1	pleckstrin homology domain containing, family J mem	NA		-0.05758	4.973655	-1.04072	0.48236114	0.753697
67484	Eepd1	endonuclease/exonuclease/phosphatase family doma	NA		-0.07055	3.37227	-1.05012	0.48237194	0.753697
56307	Metap2	methionine aminopeptidase 2, transcript variant X2	NA		0.032766	7.587253	1.022971	0.48240072	0.753697
54631	Nphs1	nephrosis 1, nephrin	NA		0.218451	-0.1244	1.163484	0.48242643	0.753697
77573	Vps33a	VPS33A CORVET/HOPS core subunit, transcript var	NA		0.036529	6.06807	1.025643	0.48245497	0.753697
11790	Speg	SPEG complex locus, transcript variant X17	NA		0.066645	5.510545	1.047278	0.48257019	0.753805
20463	Cox7a2l	cytochrome c oxidase subunit 7A2 like, transcript vari	NA		0.03508	7.215342	1.024613	0.48269121	0.753921
54214	Golga4	golgi autoantigen, golgin subfamily a, 4, transcript va	NA		0.054104	6.309336	1.038214	0.48275495	0.753948
269855	Ssc5d	scavenger receptor cysteine rich family, 5 domains	NA		-0.10884	2.478242	-1.07836	0.48312332	0.754296
14911	Thumpd3	THUMP domain containing 3, transcript variant 1	NA		-0.04899	5.10406	-1.03454	0.48315748	0.754296
106757	Catsperd	cation channel sperm associated auxiliary subunit de	NA		0.178087	1.125677	1.131383	0.48315992	0.754296
211949	Spsb4	splA/ryanodine receptor domain and SOCS box conta	NA		0.059866	4.379658	1.042369	0.48316302	0.754296
74533	Gzf1	GDNF-inducible zinc finger protein 1, transcript variar	NA		0.052313	5.173582	1.036926	0.48325993	0.754375
217325	Llgl2	LLGL2 scribble cell polarity complex component, tran	NA		-0.17387	0.632855	-1.12808	0.48331633	0.754383
66367	2310022A10Rik	RIKEN cDNA 2310022A10 gene, transcript variant 1	NA		-0.04612	4.900531	-1.03248	0.48335773	0.754383
101604	E430018J23Rik	RIKEN cDNA E430018J23 gene	NA		-0.09263	2.849509	-1.06631	0.4836482	0.754693
380918	Siah3	siah E3 ubiquitin protein ligase family member 3	NA		0.161638	1.241719	1.118557	0.48366583	0.754693
225187	Ankrd29	ankyrin repeat domain 29, transcript variant 2	NA		-0.06584	3.808921	-1.0467	0.48369535	0.754693
14566	Gdf9	growth differentiation factor 9, transcript variant X3	NA		0.145708	1.228405	1.106274	0.48395155	0.754861
70385	Spdl1	spindle apparatus coiled-coil protein 1	NA		-0.12324	2.44862	-1.08918	0.48396409	0.754861
12814	Col11a1	collagen, type XI, alpha 1, transcript variant X4	NA		0.062483	5.692052	1.044261	0.48402253	0.754861
26373	Clcn7	chloride channel, voltage-sensitive 7, transcript variar	NA		-0.04296	5.463896	-1.03022	0.48405895	0.754861
245468	Pnma3	paraneoplastic antigen MA3	NA		-0.06627	4.733894	-1.04701	0.48406841	0.754861
73473	Iws1	IWS1, SUPT6 interacting protein	NA		-0.04051	6.382758	-1.02847	0.48408179	0.754861
75423	Arl5a	ADP-ribosylation factor-like 5A	NA		0.036968	7.110379	1.025955	0.48430354	0.755135
66715	Henmt1	HEN1 methyltransferase homolog 1 (Arabidopsis), tra	NA		0.270346	0.013403	1.206097	0.48440038	0.755213
20091	Rps3a1	ribosomal protein S3A1	NA		0.04522	9.296343	1.03184	0.48454998	0.755374
18810	Plec	plectin, transcript variant 9	NA		-0.05026	5.774653	-1.03545	0.48462886	0.755425
105246062	D330022H12Rik	RIKEN cDNA D330022H12 gene	NA		-0.11696	2.007908	-1.08445	0.48469233	0.755432
23882	Gadd45g	growth arrest and DNA-damage-inducible 45 gamma	NA		-0.05902	3.866838	-1.04176	0.48472601	0.755432
56490	Zbtb20	zinc finger and BTB domain containing 20, transcript	NA		-0.03932	7.77168	-1.02763	0.48482849	0.755432
50530	Mfap5	microfibrillar associated protein 5, transcript variant 1	NA		-0.20112	0.655229	-1.14959	0.48486475	0.755432
320558	Sycp2	synaptonemal complex protein 2	NA		-0.22639	-0.13663	-1.1699	0.48487096	0.755432
407821	Znrf3	zinc and ring finger 3, transcript variant 2	NA		0.059942	3.962953	1.042424	0.48491327	0.755432
14772	Grk4	G protein-coupled receptor kinase 4, transcript varian	NA		-0.11776	1.850653	-1.08505	0.48495865	0.755432
20018	Polr1d	polymerase (RNA) I polypeptide D, transcript variant	NA		0.043389	6.106894	1.030532	0.48501215	0.755443
26562	Ncdn	neurochondrin, transcript variant 1	NA		-0.03413	7.079545	-1.02394	0.48513162	0.755557
66481	Rps21	ribosomal protein S21, transcript variant 3	NA		0.040393	7.020474	1.028394	0.48518164	0.755563
67576	4930429B21Rik	RIKEN cDNA 4930429B21 gene	NA		-0.17042	0.675426	-1.12539	0.48526144	0.755615
107522	Ece2	endothelin converting enzyme 2, transcript variant 5	NA		0.067371	3.947963	1.047806	0.48543895	0.755819
69772	Bdh2	3-hydroxybutyrate dehydrogenase, type 2, transcript	NA		0.163919	0.6107	1.120326	0.48561731	0.756024
118567914	LOC118567914	uncharacterized LOC118567914	NA		-0.24469	-0.16738	-1.18484	0.48574407	0.756149
102636909	Gm33851	predicted gene, 33851	NA		0.281153	-0.25876	1.215165	0.48584085	0.756162
85305	Kars	lysyl-tRNA synthetase, transcript variant 2	NA		-0.03145	7.090531	-1.02204	0.48584534	0.756162
68875	Tmcc2	transmembrane and coiled-coil domains 2, transcript	NA		-0.03469	7.026091	-1.02433	0.48592827	0.756219
58207	Slc43a3	solute carrier family 43, member 3, transcript variant	NA		-0.11214	2.357793	-1.08083	0.48599203	0.756246
68292	Stt3b	STT3, subunit of the oligosaccharyltransferase compl	NA		0.034208	7.286881	1.023994	0.48609827	0.756339
277353	Tcfl5	transcription factor-like 5 (basic helix-loop-helix), tran	NA		0.131922	1.580643	1.095753	0.48619861	0.756345
110606	Fntb	farnesyltransferase, CAAX box, beta	NA		-0.0445	5.188602	-1.03133	0.48622356	0.756345
54648	Ccdc120	coiled-coil domain containing 120	NA		-0.04913	5.295888	-1.03464	0.48624192	0.756345
59008	Anapc5	anaphase-promoting complex subunit 5, transcript va	NA		-0.03153	8.556998	-1.0221	0.48630654	0.756374
228790	Asxl1	additional sex combs like 1, transcript variant X2	NA		0.033316	6.674735	1.023362	0.48637518	0.756408
229521	Syt11	synaptotagmin XI	NA		-0.03079	8.21193	-1.02157	0.48657313	0.756573
72258	Kcnk10	potassium channel, subfamily K, member 10, transcri	NA		-0.04098	5.653111	-1.02881	0.48657449	0.756573
13016	Ctbp1	C-terminal binding protein 1, transcript variant 4	NA		-0.03808	8.368815	-1.02674	0.48684607	0.756923
16529	Kcnk5	potassium channel, subfamily K, member 5	NA		-0.17892	1.225219	-1.13204	0.48695583	0.757003
76265	Tsen54	tRNA splicing endonuclease subunit 54	NA		-0.0866	3.495111	-1.06187	0.48702145	0.757003
11441	Chrna7	cholinergic receptor, nicotinic, alpha polypeptide 7	NA		0.16922	1.244824	1.12445	0.48704841	0.757003
73407	Tepp	testis, prostate and placenta expressed, transcript va	NA		-0.15825	0.849585	-1.11593	0.48711845	0.757003

78134	Lpar4	lysophosphatidic acid receptor 4	NA	0.079046	3.218241	1.056319	0.48713017	0.757003
102632238	Gm30371	predicted gene, 30371	NA	0.304138	-0.77702	1.234681	0.48733847	0.757255
108167623	Gm46091	predicted gene, 46091, transcript variant 1	NA	0.178788	0.513409	1.131933	0.48749392	0.757338
102634926	Gm12426	predicted gene 12426, transcript variant X19	NA	-0.11518	2.091514	-1.08311	0.48755422	0.757338
319740	Zfyve27	zinc finger, FYVE domain containing 27, transcript va	NA	-0.04202	6.012884	-1.02955	0.4875754	0.757338
269397	Ss18l1	SS18, nBAF chromatin remodeling complex subunit l	NA	0.033845	6.890996	1.023737	0.48759615	0.757338
20606	Sstr2	somatostatin receptor 2, transcript variant 1	NA	0.051155	4.991956	1.036094	0.48764281	0.757338
17928	Myog	myogenin	NA	-0.26044	-0.05383	-1.19785	0.48768147	0.757338
102633818	Gm31550	predicted gene, 31550	NA	0.109817	2.0882	1.079091	0.48771758	0.757338
329540	Nol4l	nucleolar protein 4-like, transcript variant X22	NA	-0.0387	7.488542	-1.02719	0.48787365	0.757508
74442	Sgms2	sphingomyelin synthase 2, transcript variant 7	NA	0.13516	2.21511	1.098215	0.48800923	0.757646
74254	Gpn1	GPN-loop GTPase 1, transcript variant X3	NA	-0.04463	5.436398	-1.03142	0.48816011	0.757808
67089	Psmc6	proteasome (prosome, macropain) 26S subunit, ATP	NA	0.033044	7.279296	1.023169	0.4882524	0.757879
105734	Tigd5	tigger transposable element derived 5	NA	-0.07286	3.514174	-1.0518	0.48837787	0.757996
216459	Myl6b	myosin, light polypeptide 6B	NA	-0.05058	4.858765	-1.03568	0.4884213	0.757996
237436	Gas2l3	growth arrest-specific 2 like 3, transcript variant 3	NA	-0.09183	3.522648	-1.06572	0.48848325	0.75802
102632865	Gm30827	predicted gene, 30827, transcript variant X5	NA	0.16965	1.243814	1.124786	0.48860133	0.758083
18505	Pax3	paired box 3, transcript variant 1	NA	-0.08856	3.044583	-1.06331	0.48861666	0.758083
66402	Sln	sarcolipin	NA	-0.24175	-0.16432	-1.18243	0.48867409	0.758099
103136	Pwp1	PWP1 homolog, endonuclein	NA	0.03797	5.809691	1.026668	0.48882935	0.758267
71985	Acad10	acyl-Coenzyme A dehydrogenase family, member 10	NA	0.155551	1.209829	1.113847	0.48887563	0.758267
75180	Tmem269	transmembrane protein 269, transcript variant X3	NA	0.165915	1.465646	1.121877	0.48898108	0.758322
83674	Cnnm1	cyclin M1, transcript variant 2	NA	0.064738	3.590507	1.045895	0.48901083	0.758322
110648	Lmx1a	LIM homeobox transcription factor 1 alpha	NA	-0.08481	3.1154	-1.06055	0.4891196	0.758322
56369	Apip	APAF1 interacting protein, transcript variant 1	NA	-0.06519	3.546889	-1.04622	0.48912211	0.758322
76826	Nubpl	nucleotide binding protein-like	NA	0.10662	2.467545	1.076702	0.48918849	0.758322
16869	Lhx1	LIM homeobox protein 1	NA	0.059731	5.270391	1.042271	0.48919032	0.758322
108167812	Gm46226	predicted gene, 46226, transcript variant X2	NA	0.22774	0.141923	1.170999	0.48930305	0.758424
57170	Dolpp1	dolichyl pyrophosphate phosphatase 1, transcript var	NA	-0.04523	4.889326	-1.03185	0.4894967	0.758611
66355	Gmpr	guanosine monophosphate reductase	NA	0.06536	3.528083	1.046346	0.48957233	0.758611
276852	D11Wsu47e	DNA segment, Chr 11, Wayne State University 47, e›	NA	0.067586	4.081338	1.047961	0.48958692	0.758611
102631657	Gm29941	predicted gene, 29941, transcript variant X3	NA	0.23041	-0.29125	1.173168	0.48960965	0.758611
319722	E330033B04Rik	RIKEN cDNA E330033B04 gene	NA	0.082795	3.306548	1.059068	0.48975539	0.758764
20744	Strbp	spermatid perinuclear RNA binding protein, transcript	NA	0.033721	8.129998	1.023649	0.48989503	0.758908
22153	Tubb4a	tubulin, beta 4A class IVA	NA	0.030815	8.158513	1.021589	0.48999982	0.758945
20535	Slc4a2	solute carrier family 4 (anion exchanger), member 2,	NA	-0.05305	4.956362	-1.03746	0.49001212	0.758945
223753	Cerk	ceramide kinase, transcript variant X4	NA	0.041851	5.453144	1.029434	0.49006994	0.758962
93713	Pcdhga5	protocadherin gamma subfamily A, 5	NA	-0.0623	4.050223	-1.04413	0.4901328	0.758988
211468	Kcnh8	potassium voltage-gated channel, subfamily H (eag-r	NA	0.082023	3.019341	1.058501	0.49025701	0.759078
77630	Prdm8	PR domain containing 8	NA	-0.05874	5.847873	-1.04155	0.49028482	0.759078
100504141	Gm20083	predicted gene, 20083, transcript variant X2	NA	-0.18827	0.577981	-1.1394	0.49037795	0.75915
624582	Morf4l1-ps1	mortality factor 4 like 1, pseudogene 1	NA	-0.21741	0.282666	-1.16264	0.49057564	0.75932
56358	Copz2	coatomer protein complex, subunit zeta 2	NA	0.093322	2.365585	1.066824	0.49061662	0.75932
75905	Dipk2b	divergent protein kinase domain 2B, transcript variant	NA	0.211611	0.588796	1.15798	0.49062725	0.75932
69731	Gemin7	gem nuclear organelle associated protein 7	NA	-0.05276	4.324361	-1.03725	0.49069705	0.759355
11834	Aqr	aquarius, transcript variant 1	NA	-0.0355	6.065612	-1.02491	0.49098506	0.759729
70025	Acot7	acyl-CoA thioesterase 7, transcript variant X5	NA	0.031387	7.276408	1.021994	0.49109192	0.759822
78611	Btbd19	BTB (POZ) domain containing 19	NA	0.131496	1.549445	1.095429	0.49117386	0.759876
57742	Abhd1	abhydrolase domain containing 1, transcript variant 1	NA	-0.1806	0.340912	-1.13336	0.49129545	0.759992
66416	Ndufa7	NADH:ubiquinone oxidoreductase subunit A7, transc	NA	0.064092	5.569389	1.045427	0.49144127	0.760146
234852	Chmp1a	charged multivesicular body protein 1A	NA	-0.05499	6.546065	-1.03885	0.49164274	0.76022
68723	Hrnr	hornerin, transcript variant X3	NA	-0.94485	0.378627	-1.92499	0.49175051	0.76022
12514	Cd68	CD68 antigen, transcript variant 1	NA	-0.11902	2.557979	-1.086	0.49179652	0.76022
13716	Ell	elongation factor RNA polymerase II	NA	-0.05141	4.793535	-1.03628	0.49185796	0.76022
50518	a	nonagouti	NA	-0.28287	-0.05889	-1.21662	0.49194101	0.76022
17095	Lyl1	lymphoblastomic leukemia 1, transcript variant X1	NA	0.117338	2.208729	1.084732	0.491942	0.76022
100043387	Gm14305	predicted gene 14305	NA	0.155386	0.992166	1.113719	0.49194237	0.76022
99412	Golga2	golgi autoantigen, golgin subfamily a, 2, transcript var	NA	0.038081	6.296457	1.026747	0.49196869	0.76022
381066	Zfp948	zinc finger protein 948, transcript variant 1	NA	0.057182	4.931467	1.040431	0.49201368	0.76022
83679	Pde4dip	phosphodiesterase 4D interacting protein (myomegal	NA	-0.03355	7.298126	-1.02352	0.4920146	0.76022
226757	Wdr26	WD repeat domain 26, transcript variant X7	NA	0.037629	7.764543	1.026425	0.4920489	0.76022
72691	Calhm2	calcium homeostasis modulator family member 2	NA	-0.2055	0.059281	-1.15309	0.49205021	0.76022
66151	Prr13	proline rich 13, transcript variant 2	NA	-0.04648	5.00553	-1.03274	0.49213904	0.760285
100038712	Gm10516	predicted gene 10516	NA	0.104079	2.479948	1.074808	0.49223341	0.760359
56309	Mycbp	MYC binding protein	NA	0.098549	2.774346	1.070696	0.49239162	0.760428
218952	Fermt2	fermitin family member 2, transcript variant 3	NA	-0.04175	6.025511	-1.02936	0.49240668	0.760428
70258	1500035N22Rik	RIKEN cDNA 1500035N22 gene	NA	-0.10085	2.109078	-1.07241	0.4924357	0.760428
320174	A830082K12Rik	RIKEN cDNA A830082K12 gene	NA	0.069929	3.614526	1.049665	0.49249933	0.760428

50772	Mapk6	mitogen-activated protein kinase 6, transcript variant	NA	0.035162	7.818736	1.024672	0.4925119	0.760428
19419	Rasgrp1	RAS guanyl releasing protein 1	NA	0.07863	3.340563	1.056015	0.49259539	0.760485
100216343	Gm17501	predicted gene, 17501	NA	-0.19339	0.319518	-1.14345	0.49294005	0.760937
216810	Tom1l2	target of myb1-like 2 (chicken), transcript variant X7	NA	-0.0402	6.6786	-1.02826	0.4930748	0.760937
72895	Setd5	SET domain containing 5, transcript variant 2	NA	-0.0315	7.583425	-1.02207	0.49310106	0.760937
224703	Marchf2	membrane associated ring-CH-type finger 2, transcrip	NA	0.046512	5.088435	1.032765	0.49310366	0.760937
17354	Mllt10	myeloid/lymphoid or mixed-lineage leukemia; translo	NA	0.036852	5.795429	1.025873	0.49317685	0.760937
57874	Hacd3	3-hydroxyacyl-CoA dehydratase 3	NA	0.03298	7.464953	1.023123	0.49324039	0.760937
111175	Pecr	peroxisomal trans-2-enoyl-CoA reductase, transcript	NA	-0.11344	1.826844	-1.0818	0.49332161	0.760937
67344	Tctex1d1	Tctex1 domain containing 1, transcript variant 2	NA	-0.26527	-0.22198	-1.20186	0.49333549	0.760937
233806	Tmem159	transmembrane protein 159	NA	-0.12389	2.168304	-1.08967	0.49335888	0.760937
19221	Ptgfrn	prostaglandin F2 receptor negative regulator	NA	-0.03867	6.54834	-1.02716	0.49343841	0.760937
16409	Itgam	integrin alpha M, transcript variant 2	NA	0.178159	1.130941	1.131439	0.49344904	0.760937
73208	3110083C13Rik	RIKEN cDNA 3110083C13 gene	NA	0.219825	-0.01423	1.164592	0.4934492	0.760937
102688	AU023762	expressed sequence AU023762	NA	0.271211	-0.57683	1.20682	0.49357706	0.761015
230597	Zfyve9	zinc finger, FYVE domain containing 9, transcript vari	NA	0.049693	6.050122	1.035045	0.49361781	0.761015
26427	Creb3l1	cAMP responsive element binding protein 3-like 1	NA	-0.0866	3.088461	-1.06186	0.49364019	0.761015
56805	Zbtb33	zinc finger and BTB domain containing 33, transcript	NA	0.050092	5.862801	1.035331	0.49399656	0.761492
67510	Tvp23b	trans-golgi network vesicle protein 23B, transcript var	NA	-0.05146	4.697215	-1.03631	0.49409757	0.761576
71099	Tssk4	testis-specific serine kinase 4, transcript variant X3	NA	0.177361	0.617817	1.130814	0.49421041	0.761594
67131	Acbd4	acyl-Coenzyme A binding domain containing 4	NA	0.101265	2.281798	1.072714	0.49423847	0.761594
75860	Tex26	testis expressed 26, transcript variant 3	NA	-0.23533	0.186209	-1.17717	0.49432084	0.761594
102634598	Gm32139	predicted gene, 32139, transcript variant 1	NA	0.230422	-0.10455	1.173178	0.49432192	0.761594
102634652	Gm32186	predicted gene, 32186, transcript variant X5	NA	-0.2056	-0.03908	-1.15317	0.49434325	0.761594
64945	Cldn12	claudin 12, transcript variant X4	NA	-0.05454	4.550838	-1.03853	0.4944626	0.761611
105246241	Gm41553	predicted gene, 41553	NA	0.206316	1.051979	1.153738	0.49447468	0.761611
100515	Zfp518b	zinc finger protein 518B, transcript variant X3	NA	-0.03388	6.341197	-1.02376	0.49450676	0.761611
72960	Top1mt	DNA topoisomerase 1, mitochondrial, transcript varia	NA	0.071777	3.424005	1.05101	0.49454509	0.761611
20856	Stc2	stanniocalcin 2	NA	0.132714	1.161685	1.096354	0.49459527	0.761611
68262	Agpat4	1-acylglycerol-3-phosphate O-acyltransferase 4 (lyso	NA	-0.04361	5.323956	-1.03069	0.49465836	0.761611
239405	Rspo2	R-spondin 2, transcript variant 2	NA	0.077545	2.894117	1.055221	0.4947259	0.761611
20382	Srsf2	serine and arginine-rich splicing factor 2	NA	0.043519	8.451806	1.030625	0.49472869	0.761611
71779	Marchf8	membrane associated ring-CH-type finger 8, transcrip	NA	0.031656	6.672193	1.022185	0.49485211	0.761729
105246846	Gm42067	predicted gene, 42067	NA	0.210385	0.043939	1.156997	0.49493123	0.761778
67080	1700019D03Rik	RIKEN cDNA 1700019D03 gene, transcript variant 7	NA	0.119077	1.680114	1.08604	0.49499679	0.761807
115487822	Gm51929	predicted gene, 51929	NA	0.088635	2.839166	1.063364	0.49517085	0.761999
11776	Ap3d1	adaptor-related protein complex 3, delta 1 subunit	NA	-0.03292	7.354248	-1.02308	0.49521483	0.761999
57778	Fmnl1	formin-like 1, transcript variant 2	NA	-0.05778	4.59831	-1.04086	0.49528928	0.762018
17754	Map1a	microtubule-associated protein 1 A, transcript variant	NA	-0.0481	7.119606	-1.0339	0.49532104	0.762018
67486	Polr3g	polymerase (RNA) III (DNA directed) polypeptide G	NA	-0.10437	2.182856	-1.07503	0.49566315	0.762397
102747	Lrrc49	leucine rich repeat containing 49, transcript variant X	NA	0.034887	6.458345	1.024477	0.49570352	0.762397
233067	Lrfn3	leucine rich repeat and fibronectin type III domain co	NA	-0.04787	5.011007	-1.03374	0.49575249	0.762397
66422	Dctpp1	dCTP pyrophosphatase 1	NA	0.065401	4.204861	1.046376	0.49577401	0.762397
66985	Rassf7	Ras association (RalGDS/AF-6) domain family (N-ter	NA	0.089894	2.857428	1.064292	0.49582704	0.762397
104709	Pik3r6	phosphoinositide-3-kinase regulatory subunit 5, trans	NA	-0.14896	1.040353	-1.10877	0.49584875	0.762397
84653	Hes7	hes family bHLH transcription factor 7, transcript vari	NA	0.236119	-0.02965	1.17782	0.495957	0.762492
75540	Fpgt	fucose-1-phosphate guanylyltransferase	NA	-0.08088	4.039147	-1.05767	0.49612144	0.762569
243905	Zfp568	zinc finger protein 568, transcript variant X1	NA	0.066883	3.809752	1.047451	0.49621561	0.762569
330369	Fbxo41	F-box protein 41, transcript variant 3	NA	0.044531	6.007277	1.031348	0.49626106	0.762569
11771	Ap2a1	adaptor-related protein complex 2, alpha 1 subunit, t	NA	-0.03285	7.141179	-1.02303	0.49628009	0.762569
78266	Zfp687	zinc finger protein 687, transcript variant 1	NA	-0.04613	5.60799	-1.03249	0.49634499	0.762569
215999	Mcu	mitochondrial calcium uniporter	NA	-0.04788	5.096314	-1.03374	0.49634973	0.762569
246703	Naxe	NAD(P)HX epimerase	NA	0.048978	5.396418	1.034531	0.49635611	0.762569
245827	Fat2	FAT atypical cadherin 2	NA	-0.1334	1.308183	-1.09687	0.49638226	0.762569
16211	Kpnb1	karyopherin (importin) beta 1	NA	-0.03059	8.329506	-1.02143	0.49644541	0.762594
244548	Elmod2	ELMO/CED-12 domain containing 2, transcript varian	NA	-0.0423	5.252966	-1.02976	0.49661671	0.762785
93707	Pcdhgc4	protocadherin gamma subfamily C, 4	NA	-0.04519	6.25284	-1.03182	0.49679852	0.762965
378462	Morn2	MORN repeat containing 2, transcript variant 1	NA	-0.08369	3.283893	-1.05972	0.49682742	0.762965
100504156	Fam181a	family with sequence similarity 181, member A	NA	0.143849	1.620316	1.104848	0.49695779	0.763093
71864	Fam217a	family with sequence similarity 217, member A, trans	NA	0.204415	0.022604	1.152219	0.49731586	0.763571
105246033	Gm41395	predicted gene, 41395	NA	0.210554	-0.024	1.157132	0.49745569	0.763713
67044	Higd2a	HIG1 domain family, member 2A	NA	0.04697	5.258648	1.033093	0.49772755	0.764059
59012	Moxd1	monooxygenase, DBH-like 1	NA	-0.10538	2.060991	-1.07578	0.49791021	0.764224
71713	Cdc40	cell division cycle 40	NA	0.045535	5.261689	1.032066	0.49792945	0.764224
13429	Dnm1	dynamin 1, transcript variant X12	NA	-0.03182	7.466063	-1.0223	0.49799877	0.764259
69089	Oxa1l	oxidase assembly 1-like	NA	0.036798	5.989904	1.025834	0.4981982	0.764475
104718	Ttc7b	tetratricopeptide repeat domain 7B, transcript variant	NA	-0.04146	5.469449	-1.02915	0.49823388	0.764475
102634502	2900060L22Rik	RIKEN cDNA 2900060L22 gene, transcript variant X	NA	0.172685	1.469377	1.127154	0.49835025	0.764582

118567689	LOC118567689	uncharacterized LOC118567689	NA	0.112059	1.96481	1.08077	0.49839773	0.764583
53333	Tomm40	translocase of outer mitochondrial membrane 40, trar	NA	0.04909	5.703199	1.034612	0.49860421	0.764827
68774	Ms4a6d	membrane-spanning 4-domains, subfamily A, membe	NA	-0.18304	0.299608	-1.13528	0.4986889	0.764885
105203	Tasor2	transcription activation suppressor family member 2,	NA	-0.04574	5.970415	-1.03222	0.49877527	0.764895
70113	Odf3b	outer dense fiber of sperm tails 3B	NA	0.229781	-0.31049	1.172657	0.49878964	0.764895
22420	Wnt6	wingless-type MMTV integration site family, member	NA	-0.15515	1.322965	-1.11354	0.4989283	0.764998
209586	Nudcd3	NudC domain containing 3, transcript variant 1	NA	-0.03528	7.058669	-1.02476	0.49897098	0.764998
66592	Stoml2	stomatin (Epb7.2)-like 2	NA	0.03877	5.686984	1.027238	0.49901808	0.764998
20672	Sox18	SRY (sex determining region Y)-box 18	NA	-0.07879	3.230631	-1.05613	0.49908947	0.764998
75104	Mmd2	monocyte to macrophage differentiation-associated 2	NA	0.0442	6.114214	1.031111	0.49911299	0.764998
27632	Nelfe	negative elongation factor complex member E, Rdbp,	NA	-0.04946	4.681341	-1.03488	0.4991725	0.764998
268449	Rpl23a	ribosomal protein L23A	NA	0.034615	9.075806	1.024284	0.49918589	0.764998
100561	Slc15a4	solute carrier family 15, member 4	NA	0.050215	4.356346	1.035419	0.49930234	0.765075
242505	Rasef	RAS and EF hand domain containing, transcript varia	NA	0.225755	0.037805	1.169389	0.49933013	0.765075
115488002	LOC115488002	zinc finger protein 431-like	NA	-0.10134	2.399983	-1.07277	0.49945334	0.765106
56773	Chst5	carbohydrate (N-acetylglucosamine 6-O) sulfotransfe	NA	-0.18873	0.627676	-1.13976	0.49947861	0.765106
73608	Marveld3	MARVEL (membrane-associating) domain containing	NA	-0.1918	0.150886	-1.14219	0.49951457	0.765106
115488671	LOC115488671	uncharacterized LOC115488671	NA	0.166565	1.099321	1.122383	0.4995383	0.765106
68352	Aspdh	aspartate dehydrogenase domain containing	NA	-0.14492	1.071212	-1.10567	0.49967881	0.765177
77590	Chst15	carbohydrate sulfotransferase 15, transcript variant 1	NA	-0.06098	5.129584	-1.04317	0.49972213	0.765177
319982	5930430L01Rik	RIKEN cDNA 5930430L01 gene, transcript variant 1	NA	-0.18943	0.1732	-1.14031	0.49975288	0.765177
66050	0610009B22Rik	RIKEN cDNA 0610009B22 gene	NA	-0.05781	4.268858	-1.04089	0.49977436	0.765177
18232	Nxph2	neurexophilin 2	NA	-0.11556	2.046239	-1.0834	0.49981979	0.765177
68294	Mfsd10	major facilitator superfamily domain containing 10, tra	NA	0.055342	4.465364	1.039106	0.50001573	0.765333
12372	Casq1	calsequestrin 1	NA	-0.1477	2.086474	-1.1078	0.50002119	0.765333
74284	1700086L19Rik	RIKEN cDNA 1700086L19 gene, transcript variant 3	NA	0.139067	1.526285	1.101193	0.50006285	0.765333
23890	Gpr34	G protein-coupled receptor 34, transcript variant X2	NA	-0.1326	1.169495	-1.09627	0.5001532	0.7654
76577	Faf2	Fas associated factor family member 2	NA	-0.03865	6.046705	-1.02715	0.50020334	0.765404
66253	Aig1	androgen-induced 1, transcript variant 2	NA	0.049242	5.037727	1.034721	0.50030814	0.765493
93710	Pcdhga2	protocadherin gamma subfamily A, 2	NA	-0.06742	4.744663	-1.04784	0.50058026	0.765778
20308	Ccl9	chemokine (C-C motif) ligand 9	NA	0.232868	0.258071	1.175169	0.50058863	0.765778
67383	Carnmt1	carnosine N-methyltransferase 1, transcript variant 1	NA	0.058123	4.907408	1.04111	0.50066778	0.765827
68481	Mpzl1	myelin protein zero-like 1, transcript variant 2	NA	0.036302	6.645784	1.025482	0.50107648	0.76638
67618	Aasdhppt	aminoadipate-semialdehyde dehydrogenase-phosph	NA	0.048167	5.005302	1.033951	0.50112497	0.766382
17301	Foxd2	forkhead box D2	NA	-0.20128	0.540661	-1.14972	0.50117555	0.766387
215351	Senp6	SUMO/sentrin specific peptidase 6, transcript variant	NA	0.039418	6.942653	1.027699	0.50130725	0.766517
18715	Pim2	proviral integration site 2	NA	0.057287	4.06659	1.040508	0.50143986	0.766627
216345	Zfc3h1	zinc finger, C3H1-type containing, transcript variant X	NA	0.049111	5.722832	1.034627	0.50147357	0.766627
15403	Hoxa6	homeobox A6	NA	0.356743	0.001485	1.280532	0.50168542	0.766879
70052	Prpf4	pre-mRNA processing factor 4, transcript variant X1	NA	-0.04458	5.062102	-1.03138	0.50175707	0.766916
68790	Fendrr	Foxf1 adjacent non-coding developmental regulatory	NA	-0.2675	-0.13417	-1.20372	0.50181116	0.766927
218035	Vps41	VPS41 HOPS complex subunit	NA	0.037761	6.905568	1.026519	0.50194594	0.766944
11920	Atm	ataxia telangiectasia mutated	NA	0.044554	4.96881	1.031364	0.50196039	0.766944
67466	Pdcl	phosducin-like	NA	0.050194	5.870945	1.035404	0.50196506	0.766944
80732	Mynn	myoneurin, transcript variant 1	NA	0.042675	5.066186	1.030022	0.50201081	0.766944
117591	Slc2a9	solute carrier family 2 (facilitated glucose transporter)	NA	0.184014	0.284534	1.13604	0.50215647	0.767094
70536	Qpct	glutaminyl-peptide cyclotransferase (glutaminyl cycla	NA	0.068139	3.419331	1.048363	0.50222734	0.76711
93708	Pcdhgc5	protocadherin gamma subfamily C, 5	NA	0.09238	3.616007	1.066127	0.50227238	0.76711
12028	Bax	BCL2-associated X protein	NA	-0.04696	5.594712	-1.03309	0.50233228	0.76711
12289	Cacna1d	calcium channel, voltage-dependent, L type, alpha 1D	NA	0.044047	5.869629	1.031002	0.50239251	0.76711
108168933	LOC108168933	uncharacterized LOC108168933	NA	-0.22086	-0.09033	-1.16543	0.50240227	0.76711
18114	Rrp1	ribosomal RNA processing 1	NA	0.035362	7.818933	1.024814	0.50258174	0.767312
414123	Mir670hg	MIR670 host gene (non-protein coding), transcript va	NA	0.101923	2.932069	1.073203	0.50264401	0.767333
19934	Rpl22	ribosomal protein L22, transcript variant 1	NA	0.030929	7.975498	1.02167	0.50273203	0.767333
67891	Rpl4	ribosomal protein L4, transcript variant X1	NA	0.0318	9.714119	1.022287	0.50273698	0.767333
115488304	5830448L01Rik	RIKEN cDNA 5830448L01 gene	NA	0.221754	0.78988	1.166151	0.50279576	0.767351
29813	Zfp385a	zinc finger protein 385A, transcript variant 1	NA	0.045017	6.052345	1.031695	0.50299134	0.767484
192195	Ash1l	ASH1 like histone lysine methyltransferase, transcript	NA	0.032863	6.57342	1.02304	0.50301837	0.767484
115487206	Gm51701	predicted gene, 51701	NA	-0.13557	1.526557	-1.09852	0.50302448	0.767484
16784	Lamp2	lysosomal-associated membrane protein 2, transcript	NA	0.043028	6.116981	1.030274	0.50318304	0.767604
70083	Metrn	meteorin, glial cell differentiation regulator	NA	0.064523	4.355134	1.045739	0.50319792	0.767604
70123	Nbdy	negative regulator of P-body association	NA	0.048473	4.709264	1.03417	0.50338967	0.767721
74155	Errfi1	ERBB receptor feedback inhibitor 1, transcript varian	NA	0.048013	4.700633	1.03384	0.503392	0.767721
14104	Fasn	fatty acid synthase, transcript variant X1	NA	-0.03292	8.692928	-1.02308	0.50341608	0.767721
17760	Map6	microtubule-associated protein 6, transcript variant 2	NA	-0.03274	8.120229	-1.02295	0.50353589	0.767776
100041306	Gm3264	predicted gene 3264	NA	0.072289	4.517515	1.051384	0.50354608	0.767776
30939	Pttg1	pituitary tumor-transforming gene 1, transcript variant	NA	-0.07845	2.98878	-1.05588	0.50359418	0.767777
106248	Qtrt2	queuine tRNA-ribosyltransferase accessory subunit 2	NA	-0.04672	4.645155	-1.03291	0.50368902	0.76785

240186	Zfp438	zinc finger protein 438, transcript variant 2	NA	-0.09508	2.3986	-1.06813	0.50378115	0.767883
12815	Col11a2	collagen, type XI, alpha 2, transcript variant X11	NA	-0.08276	4.776832	-1.05904	0.50385859	0.767883
664968	Tmem238	transmembrane protein 238	NA	0.191793	0.037414	1.142183	0.50389216	0.767883
100336	Ppp1r8	protein phosphatase 1, regulatory subunit 8, transcrip	NA	-0.03685	6.115041	-1.02587	0.50393772	0.767883
59009	Sh3rf1	SH3 domain containing ring finger 1, transcript varian	NA	0.033163	6.418721	1.023253	0.50394669	0.767883
232855	Zfp772	zinc finger protein 772	NA	-0.06567	3.696736	-1.04657	0.50406773	0.767963
11972	Atp6v0d1	ATPase, H+ transporting, lysosomal V0 subunit D1	NA	0.033377	7.333296	1.023405	0.50409374	0.767963
75692	Nr2c2ap	nuclear receptor 2C2-associated protein, transcript va	NA	-0.07344	3.632115	-1.05222	0.50441623	0.768317
27357	Gyg	glycogenin, transcript variant 2	NA	0.042882	4.973799	1.030169	0.50442023	0.768317
14190	Fgl2	fibrinogen-like protein 2	NA	0.115757	1.793393	1.083543	0.50449291	0.768355
12819	Col15a1	collagen, type XV, alpha 1	NA	0.050848	4.586464	1.035873	0.50460848	0.768459
68185	Coa4	cytochrome c oxidase assembly factor 4	NA	0.119915	2.063624	1.086671	0.50478026	0.768649
230584	Yipf1	Yip1 domain family, member 1, transcript variant 1	NA	-0.05751	4.422281	-1.04067	0.50495207	0.768839
23872	Ets2	E26 avian leukemia oncogene 2, 3' domain	NA	0.043615	5.063469	1.030693	0.50499948	0.768839
328580	Tubgcp6	tubulin, gamma complex associated protein 6, transcı	NA	-0.04047	5.179547	-1.02845	0.50510207	0.768923
108871	4930447M23Rik	RIKEN cDNA 4930447M23 gene	NA	-0.11017	2.02218	-1.07936	0.50516069	0.768941
115486129	Gm51431	predicted gene, 51431	NA	-0.20525	0.579077	-1.15289	0.50545528	0.769317
76816	Sdccag8	serologically defined colon cancer antigen 8, transcrip	NA	-0.07001	3.533464	-1.04972	0.50561025	0.769481
15201	Hells	helicase, lymphoid specific	NA	0.053335	4.454163	1.037661	0.50579991	0.769685
118568277	LOC118568277	uncharacterized LOC118568277	NA	0.076417	4.157516	1.054396	0.50584183	0.769685
104348	Zfp120	zinc finger protein 120, transcript variant 1	NA	0.057157	4.342423	1.040414	0.50588633	0.769685
110175	Ggct	gamma-glutamyl cyclotransferase	NA	0.124369	2.258618	1.090031	0.50617711	0.770056
100756	Usp30	ubiquitin specific peptidase 30, transcript variant X1	NA	0.047291	5.201814	1.033323	0.50649394	0.770465
73166	Tm7sf2	transmembrane 7 superfamily member 2	NA	-0.05231	4.320041	-1.03692	0.50664021	0.770504
449000	Zfp960	zinc finger protein 960, transcript variant 1	NA	-0.13143	1.81618	-1.09538	0.50664301	0.770504
94284	Ugt1a6a	UDP glucuronosyltransferase 1 family, polypeptide A(	NA	-0.20698	0.972955	-1.15427	0.50666122	0.770504
223227	Sox21	SRY (sex determining region Y)-box 21	NA	0.062581	4.912749	1.044333	0.50676476	0.770589
209456	Trp53bp2	transformation related protein 53 binding protein 2	NA	-0.04363	5.745344	-1.0307	0.50682874	0.770615
104885	Tmem179	transmembrane protein 179	NA	0.040528	5.426707	1.02849	0.50689941	0.77065
12846	Comt	catechol-O-methyltransferase, transcript variant 3	NA	-0.04802	5.21784	-1.03384	0.50704553	0.770712
56317	Anapc7	anaphase promoting complex subunit 7, transcript va	NA	-0.03859	5.849304	-1.02711	0.50704737	0.770712
100532	Rell1	RELT-like 1	NA	-0.05587	4.083932	-1.03949	0.50712724	0.770712
118567916	LOC118567916	uncharacterized LOC118567916	NA	-0.16957	0.657065	-1.12472	0.50712978	0.770712
106957	Slc39a6	solute carrier family 39 (metal ion transporter), memb	NA	0.032528	7.048561	1.022803	0.50725885	0.770836
100047123	Gm15743	predicted gene 15743	NA	0.223764	0.274929	1.167777	0.50734734	0.770846
66531	Cmc2	COX assembly mitochondrial protein 2	NA	-0.09942	2.50614	-1.07134	0.50735982	0.770846
114301	Palmd	palmdelphin	NA	0.055125	4.866206	1.038949	0.50750568	0.770995
102634380	Gm31976	predicted gene, 31976	NA	-0.17678	0.247454	-1.13036	0.50756811	0.771018
102632924	Gm30875	predicted gene, 30875, transcript variant X4	NA	-0.16916	0.681335	-1.12441	0.50777255	0.771257
15444	Hpca	hippocalcin, transcript variant 4	NA	0.04392	5.63422	1.030911	0.50785735	0.771258
66156	Anapc11	anaphase promoting complex subunit 11, transcript v	NA	-0.04241	5.516843	-1.02983	0.50792386	0.771258
74132	Rnf6	ring finger protein (C3H2C3 type) 6, transcript variant	NA	0.058349	4.306855	1.041273	0.50796236	0.771258
22661	Zfp148	zinc finger protein 148, transcript variant 2	NA	0.037723	6.681617	1.026492	0.5079631	0.771258
230598	Nrd1	nardilysin, N-arginine dibasic convertase, NRD conve	NA	0.030351	7.282582	1.02126	0.50811943	0.771424
195531	Zfp982	zinc finger protein 982, transcript variant 2	NA	-0.1217	2.223523	-1.08802	0.5083759	0.771692
170643	Kirrel	kirre like nephrin family adhesion molecule 1, transcri	NA	-0.0407	5.52125	-1.02861	0.50840944	0.771692
73178	Wasl	WASP like actin nucleation promoting factor, transcrip	NA	0.037356	6.119786	1.026231	0.50844115	0.771692
55983	Pdzrn3	PDZ domain containing RING finger 3, transcript vari	NA	-0.04695	5.423005	-1.03308	0.50848579	0.771692
12033	Bcap29	B cell receptor associated protein 29, transcript varia	NA	0.054585	4.096256	1.03856	0.50887201	0.772141
74189	Phactr3	phosphatase and actin regulator 3, transcript variant :	NA	0.038486	6.515779	1.027035	0.50888676	0.772141
13664	Eif1a	eukaryotic translation initiation factor 1A, transcript va	NA	0.041615	5.78151	1.029265	0.50892412	0.772141
51869	Rif1	replication timing regulatory factor 1, transcript varian	NA	-0.03718	5.487284	-1.02611	0.50905367	0.772255
108672	Zdhhc15	zinc finger, DHHC domain containing 15, transcript va	NA	0.065004	3.606685	1.046088	0.50910633	0.772255
219140	Spata13	spermatogenesis associated 13, transcript variant X1	NA	-0.04818	5.398087	-1.03396	0.50914137	0.772255
66111	Tmed3	transmembrane p24 trafficking protein 3	NA	-0.05804	4.252357	-1.04105	0.50926357	0.772363
207182	Ggt7	gamma-glutamyltransferase 7, transcript variant 2	NA	0.041161	5.833553	1.028942	0.50930786	0.772363
208624	Alg3	asparagine-linked glycosylation 3 (alpha-1,3-mannos	NA	-0.08117	3.6781	-1.05788	0.50947121	0.772518
73246	Rassf6	Ras association (RalGDS/AF-6) domain family memb	NA	0.193163	0.285964	1.143268	0.50950475	0.772518
319388	Irx3os	iroquois homeobox 3, opposite strand, transcript varia	NA	-0.20889	0.530039	-1.1558	0.50958058	0.772561
209584	Tyw3	tRNA-yW synthesizing protein 3 homolog (S. cerevisi	NA	-0.07319	3.54022	-1.05204	0.50970682	0.77268
102632463	Prox1os	prospero homeobox 1, opposite strand	NA	0.142779	1.000206	1.104029	0.50983742	0.772766
11909	Atf2	activating transcription factor 2, transcript variant X15	NA	0.04564	7.02083	1.032141	0.50985821	0.772766
22066	Trpc4	transient receptor potential cation channel, subfamily	NA	0.059584	3.630842	1.042165	0.51000975	0.772792
12663	Chml	choroideremia-like	NA	-0.08477	4.019864	-1.06052	0.51001961	0.772792
214763	Cgas	cyclic GMP-AMP synthase, transcript variant 1	NA	0.159905	0.832065	1.117213	0.51005867	0.772792
208080	Ubap1l	ubiquitin-associated protein 1-like, transcript variant 2	NA	-0.07509	3.541978	-1.05342	0.51007862	0.772792
66477	Atp5md	ATP synthase membrane subunit DAPIT	NA	0.043058	6.462821	1.030295	0.51011328	0.772792
69010	Anapc13	anaphase promoting complex subunit 13, transcript v	NA	-0.04878	4.311647	-1.03439	0.51031368	0.772965

77480	Kidins220	kinase D-interacting substrate 220, transcript variant	NA	0.030526	9.146845	1.021384	0.51032228	0.772965
76303	Osbp	oxysterol binding protein	NA	0.033094	6.298239	1.023204	0.51048906	0.773146
18415	Hspa4l	heat shock protein 4 like, transcript variant X1	NA	-0.04265	5.827598	-1.03001	0.51067594	0.773337
12795	Plk3	polo like kinase 3, transcript variant 1	NA	-0.06132	3.909453	-1.04342	0.5107641	0.773337
11974	Atp6v0e	ATPase, H+ transporting, lysosomal V0 subunit E	NA	0.049736	4.388178	1.035075	0.51079892	0.773337
329934	Foxo6	forkhead box O6	NA	0.053343	5.317076	1.037667	0.51080559	0.773337
68972	Tatdn3	TatD DNase domain containing 3, transcript variant 4	NA	0.127137	2.468304	1.092124	0.51085834	0.773345
381925	Plpp4	phospholipid phosphatase 4	NA	0.128724	2.421092	1.093326	0.51095124	0.773405
18797	Plcb3	phospholipase C, beta 3, transcript variant X2	NA	-0.04716	4.519496	-1.03323	0.51101687	0.773405
100045778	Rnf223	ring finger 223	NA	-0.15374	0.96746	-1.11245	0.51104055	0.773405
217030	Synrg	synergin, gamma	NA	-0.03226	6.246952	-1.02261	0.51120502	0.773582
78889	Wsb1	WD repeat and SOCS box-containing 1, transcript va	NA	0.030186	8.178615	1.021144	0.5113924	0.773724
72053	Tmub2	transmembrane and ubiquitin-like domain containing	NA	0.048329	5.661844	1.034066	0.51142708	0.773724
102632727	Gm30723	predicted gene, 30723, transcript variant X3	NA	-0.15564	0.673067	-1.11391	0.51145198	0.773724
115487474	Gm51815	predicted gene, 51815, transcript variant X2	NA	0.236415	-0.36338	1.178062	0.51148884	0.773724
59015	Nup160	nucleoporin 160	NA	-0.0448	5.025642	-1.03154	0.51159652	0.773815
54371	Chst2	carbohydrate sulfotransferase 2	NA	0.049152	6.370342	1.034657	0.51172846	0.773942
76183	Celf6	CUGBP, Elav-like family member 6, transcript variant	NA	-0.04518	5.356671	-1.03181	0.51197849	0.774249
268345	Kcnc2	potassium voltage gated channel, Shaw-related subfa	NA	0.068487	4.032091	1.048616	0.51206114	0.774251
18393	Orc2	origin recognition complex, subunit 2, transcript varia	NA	0.042744	5.014663	1.030071	0.51209518	0.774251
406220	Krt77	keratin 77, transcript variant X3	NA	-0.49717	1.614657	-1.41144	0.51217043	0.774251
76527	Il34	interleukin 34, transcript variant 1	NA	-0.13535	1.510105	-1.09836	0.51217068	0.774251
74173	Rab10os	RAB10, member RAS oncogene family, opposite stra	NA	0.093406	2.241874	1.066886	0.51235542	0.774368
107358	Tm9sf3	transmembrane 9 superfamily member 3	NA	0.043419	7.757689	1.030554	0.51236814	0.774368
54670	Atp8b1	ATPase, class I, type 8B, member 1	NA	-0.20623	0.274994	-1.15367	0.51239034	0.774368
20892	Cenpx	centromere protein X, transcript variant 1	NA	0.061638	3.987944	1.04365	0.51260517	0.774596
380601	Fastkd5	FAST kinase domains 5, transcript variant 3	NA	0.065532	3.51014	1.04647	0.51263644	0.774596
268935	Scube3	signal peptide, CUB domain, EGF-like 3, transcript va	NA	0.039426	5.631664	1.027705	0.51272701	0.774654
68730	Dus1l	dihydrouridine synthase 1-like (S. cerevisiae), transcr	NA	-0.0535	4.440069	-1.03778	0.51278167	0.774654
56705	Ranbp9	RAN binding protein 9	NA	0.034361	6.254595	1.024103	0.51283968	0.774654
75304	4930563E22Rik	RIKEN cDNA 4930563E22 gene	NA	0.093836	2.833667	1.067204	0.51291912	0.774654
114664	Hsd17b11	hydroxysteroid (17-beta) dehydrogenase 11	NA	-0.08052	4.008148	-1.0574	0.51306681	0.774654
66594	Uqcr11	ubiquinol-cytochrome c reductase, complex III subuni	NA	0.048053	5.116587	1.033869	0.51310019	0.774654
68465	Adipor2	adiponectin receptor 2, transcript variant 1	NA	-0.03628	6.267079	-1.02547	0.51310995	0.774654
216456	Gls2	glutaminase 2 (liver, mitochondrial), transcript variant	NA	-0.14982	0.791036	-1.10943	0.51312867	0.774654
71770	Ap2b1	adaptor-related protein complex 2, beta 1 subunit, tra	NA	-0.02936	8.595315	-1.02056	0.51314876	0.774654
66475	Rps23	ribosomal protein S23	NA	0.041108	8.650839	1.028903	0.51316775	0.774654
319520	Dusp4	dual specificity phosphatase 4	NA	-0.04395	5.474956	-1.03093	0.51319843	0.774654
55950	Bri3	brain protein I3, transcript variant 1	NA	0.058001	5.431513	1.041022	0.51332888	0.774655
67916	Plpp3	phospholipid phosphatase 3	NA	0.037048	6.446721	1.026012	0.51333583	0.774655
16796	Lasp1	LIM and SH3 protein 1	NA	-0.03225	7.985128	-1.02261	0.51334202	0.774655
77940	A930004D18Rik	RIKEN cDNA A930004D18 gene, transcript variant 1	NA	-0.0916	2.863393	-1.06555	0.51340368	0.774676
320705	Bend6	BEN domain containing 6, transcript variant 1	NA	0.041221	6.17408	1.028984	0.51353896	0.774786
70598	Filip1	filamin A interacting protein 1, transcript variant X1	NA	0.104339	3.034368	1.075002	0.51357166	0.774786
13177	Eci1	enoyl-Coenzyme A delta isomerase 1	NA	-0.10295	3.009211	-1.07396	0.51389404	0.7752
74412	Gle1	GLE1 RNA export mediator (yeast)	NA	0.037269	6.084183	1.026169	0.51407008	0.775279
105245682	Gm41099	predicted gene, 41099, transcript variant X1	NA	0.222364	0.125178	1.166644	0.51407674	0.775279
105245406	Gm5954	predicted gene 5954	NA	0.24769	0.017105	1.187305	0.51408903	0.775279
118568798	LOC118568798	uncharacterized LOC118568798	NA	0.175682	0.386153	1.129498	0.51421886	0.77539
17190	Mbd1	methyl-CpG binding domain protein 1, transcript varia	NA	-0.03756	5.933685	-1.02637	0.5142581	0.77539
667705	Gm8773	predicted gene 8773	NA	-0.10635	2.086872	-1.0765	0.51436247	0.775394
109905	Rap1a	RAS-related protein 1a, transcript variant X4	NA	0.038677	5.826899	1.027171	0.51452075	0.775394
217012	Unc45b	unc-45 myosin chaperone B	NA	0.155116	1.655046	1.113511	0.51452136	0.775394
102639570	Gm35850	predicted gene, 35850, transcript variant X7	NA	-0.20047	0.580232	-1.14907	0.51454124	0.775394
11651	Akt1	thymoma viral proto-oncogene 1, transcript variant 1	NA	-0.02864	8.42889	-1.02005	0.51454171	0.775394
223691	Eif3l	eukaryotic translation initiation factor 3, subunit L	NA	0.030684	7.155665	1.021496	0.51459561	0.775394
18830	Pltp	phospholipid transfer protein	NA	-0.04591	5.251291	-1.03233	0.51461683	0.775394
102632008	Gm30189	predicted gene, 30189, transcript variant 2	NA	-0.07859	2.89348	-1.05598	0.51464189	0.775394
71777	Ing3	inhibitor of growth family, member 3, transcript varian	NA	0.050562	4.673598	1.035668	0.51482856	0.775484
192170	Eif4a3	eukaryotic translation initiation factor 4A3	NA	0.038245	6.624081	1.026864	0.5148377	0.775484
13654	Egr2	early growth response 2, transcript variant 6	NA	0.240838	-0.27927	1.181679	0.51484468	0.775484
19125	Prodh	proline dehydrogenase	NA	-0.15242	1.305648	-1.11143	0.51495156	0.775547
13822	Epb41l2	erythrocyte membrane protein band 4.1 like 2, transc	NA	0.048486	5.841187	1.034179	0.51498207	0.775547
74645	Tent5c	terminal nucleotidyltransferase 5C, transcript variant	NA	0.148345	4.117121	1.108297	0.51508212	0.775626
17692	Msl3	MSL complex subunit 3, transcript variant 5	NA	0.052264	5.07974	1.036891	0.51512947	0.775626
224454	Zdhhc14	zinc finger, DHHC domain containing 14, transcript va	NA	-0.05956	4.536542	-1.04215	0.51527107	0.775767
192173	Mcrip1	MAPK regulated corepressor interacting protein 1, tra	NA	0.043406	6.007555	1.030544	0.51545577	0.775973
69804	Tmem147	transmembrane protein 147	NA	0.046188	5.356648	1.032533	0.51580468	0.776427

243983	Zdhhc13	zinc finger, DHHC domain containing 13, transcript va	NA	0.041008	5.042583	1.028832	0.51589924	0.776485
66390	Prelid3b	PRELI domain containing 3B	NA	-0.04326	5.910433	-1.03044	0.51594924	0.776485
100503019	Gm16551	predicted gene 16551, transcript variant 1	NA	-0.1032	2.055623	-1.07415	0.51599112	0.776485
18387	Oprk1	opioid receptor, kappa 1, transcript variant 2	NA	0.140156	1.198965	1.102024	0.51603443	0.776485
76138	Ccdc138	coiled-coil domain containing 138, transcript variant X	NA	0.080631	3.21447	1.057481	0.51630803	0.776825
266692	Cpne1	copine I, transcript variant 2	NA	-0.03569	5.990609	-1.02505	0.51639043	0.776877
70248	Dazap1	DAZ associated protein 1, transcript variant 1	NA	-0.03159	7.275012	-1.02213	0.51670943	0.777126
226278	Prlhr	prolactin releasing hormone receptor, transcript varia	NA	-0.33706	-0.93282	-1.26318	0.51673911	0.777126
102643210	Gm38664	predicted gene, 38664	NA	-0.21167	-0.26592	-1.15803	0.51677905	0.777126
11519	Add2	adducin 2 (beta), transcript variant 4	NA	-0.04124	8.245762	-1.029	0.51684882	0.777126
71983	Tmco6	transmembrane and coiled-coil domains 6, transcript	NA	-0.07356	3.274187	-1.05231	0.51694043	0.777126
103220	Ttc41	tetratricopeptide repeat domain 41, transcript variant	NA	0.116936	2.002345	1.084429	0.5170128	0.777126
56217	Mpp5	membrane protein, palmitoylated 5 (MAGUK p55 sub	NA	0.057058	4.563815	1.040342	0.51702923	0.777126
22700	Zfp40	zinc finger protein 40, transcript variant 1	NA	0.037111	5.673761	1.026057	0.51704016	0.777126
102636679	Gm33682	predicted gene, 33682	NA	-0.22082	-0.0645	-1.1654	0.51705829	0.777126
30841	Kdm2b	lysine (K)-specific demethylase 2B, transcript variant	NA	-0.03215	7.380126	-1.02253	0.5170718	0.777126
56494	Gosr2	golgi SNAP receptor complex member 2, transcript va	NA	-0.03248	6.673663	-1.02277	0.51708103	0.777126
52428	Rhpn2	rhophilin, Rho GTPase binding protein 2	NA	-0.13104	1.631361	-1.09508	0.51725841	0.777321
56047	Msln	mesothelin, transcript variant 2	NA	0.262673	-0.18782	1.199699	0.51732098	0.777343
74035	Nol9	nucleolar protein 9, transcript variant 1	NA	-0.0425	5.27892	-1.0299	0.51742216	0.777423
329650	Med12l	mediator complex subunit 12-like	NA	0.054762	5.043442	1.038688	0.51751514	0.777459
20597	Smpd1	sphingomyelin phosphodiesterase 1, acid lysosomal	NA	0.041456	6.000488	1.029152	0.51754163	0.777459
30956	Aass	aminoadipate-semialdehyde synthase	NA	-0.18683	0.250943	-1.13826	0.51761194	0.777493
666920	Gm8364	predicted gene 8364	NA	0.122243	1.482768	1.088426	0.51784603	0.777769
66395	Ahnak	AHNAK nucleoprotein (desmoyokin), transcript variar	NA	-0.04399	5.522143	-1.03096	0.51789126	0.777769
14709	Gng8	guanine nucleotide binding protein (G protein), gamm	NA	0.172403	0.595781	1.126934	0.51795667	0.777795
192292	Nrbp1	nuclear receptor binding protein 1, transcript variant 2	NA	-0.03104	6.582091	-1.02175	0.51807068	0.777895
545085	Wdr70	WD repeat domain 70, transcript variant 2	NA	0.039341	5.559054	1.027644	0.51823984	0.778052
57138	Slc12a5	solute carrier family 12, member 5, transcript variant	NA	0.035557	7.002202	1.024952	0.51827386	0.778052
66497	Cmss1	cms small ribosomal subunit 1	NA	0.070618	3.420279	1.050166	0.51831872	0.778052
230709	Zmpste24	zinc metallopeptidase, STE24	NA	0.042188	5.02679	1.029675	0.51836667	0.778052
72149	Strada	STE20-related kinase adaptor alpha, transcript variar	NA	-0.043	4.978901	-1.03026	0.51844466	0.778097
30940	Usp25	ubiquitin specific peptidase 25	NA	0.05315	4.544337	1.037528	0.51872941	0.778453
56196	Tdp2	tyrosyl-DNA phosphodiesterase 2	NA	0.044797	5.061452	1.031538	0.51881106	0.778504
73121	Rflna	refilin A	NA	-0.15281	1.36E+00	-1.11173	0.5189618	0.778606
102641105	Gm38534	predicted gene, 38534, transcript variant X2	NA	0.239896	0.054958	1.180908	0.51897485	0.778606
15565	Htr6	5-hydroxytryptamine (serotonin) receptor 6	NA	-0.1306	1.250485	-1.09475	0.51906663	0.778672
269424	Jade1	jade family PHD finger 1, transcript variant X1	NA	-0.05346	4.717291	-1.03775	0.51914151	0.778712
78618	Acap2	ArfGAP with coiled-coil, ankyrin repeat and PH doma	NA	0.03735	5.745825	1.026227	0.51932716	0.778919
65107	Lrp10	low-density lipoprotein receptor-related protein 10	NA	-0.04145	5.295158	-1.02915	0.51941981	0.778986
72585	Lypd1	Ly6/Plaur domain containing 1, transcript variant 1	NA	0.038114	5.676152	1.026771	0.51951206	0.779053
108168022	Gm46371	predicted gene, 46371	NA	-0.12976	1.945239	-1.09411	0.52004616	0.779782
12017	Bag1	BCL2-associated athanogene 1, transcript variant 1	NA	0.037364	6.583453	1.026237	0.52010047	0.779791
56214	Scamp4	secretory carrier membrane protein 4	NA	-0.04143	4.923047	-1.02914	0.52017424	0.77983
118567930	LOC118567930	uncharacterized LOC118567930	NA	-0.04893	5.599393	-1.0345	0.52022971	0.779841
218832	Polr3a	polymerase (RNA) III (DNA directed) polypeptide A	NA	0.047885	4.546132	1.033748	0.52033937	0.779865
72580	Zup1	zinc finger containing ubiquitin peptidase 1, transcript	NA	0.067698	4.082125	1.048043	0.52034108	0.779865
664837	Gm7361	predicted gene 7361, transcript variant X3	NA	-0.14709	0.9202	-1.10733	0.52045444	0.779918
235907	Zfp65	zinc finger protein 65	NA	-0.0536	4.695219	-1.03785	0.52047271	0.779918
319760	D130020L05Rik	RIKEN cDNA D130020L05 gene, transcript variant 1	NA	0.135237	0.985645	1.098273	0.520557	0.779943
237806	Dnah9	dynein, axonemal, heavy chain 9, transcript variant X	NA	0.088931	2.751388	1.063582	0.52061961	0.779943
241118	Asic4	acid-sensing (proton-gated) ion channel family memb	NA	0.049897	4.902472	1.035191	0.52065207	0.779943
212448	9330159F19Rik	RIKEN cDNA 9330159F19 gene	NA	-0.03151	7.952951	-1.02208	0.52070865	0.779943
75758	9130401M01Rik	RIKEN cDNA 9130401M01 gene	NA	-0.05263	4.601504	-1.03715	0.52072905	0.779943
24010	Ik	IK cytokine	NA	0.032618	7.220197	1.022867	0.52078476	0.779955
19013	Ppara	peroxisome proliferator activated receptor alpha, tran	NA	0.124948	1.283124	1.090469	0.52087746	0.780022
28035	Usp39	ubiquitin specific peptidase 39	NA	-0.04384	5.535521	-1.03085	0.52099612	0.780088
14584	Gfpt2	glutamine fructose-6-phosphate transaminase 2	NA	-0.06555	3.556637	-1.04648	0.52101749	0.780088
56356	Gltp	glycolipid transfer protein	NA	-0.06243	4.386761	-1.04422	0.52109786	0.780137
12558	Cdh2	cadherin 2	NA	0.03062	8.472063	1.021451	0.52120065	0.780178
20466	Sin3a	transcriptional regulator, SIN3A (yeast), transcript var	NA	-0.03855	5.938168	-1.02708	0.52122161	0.780178
20648	Snta1	syntrophin, acidic 1	NA	0.068401	3.468452	1.048554	0.52133161	0.780267
13591	Ebf1	early B cell factor 1, transcript variant 2	NA	0.031742	6.7363	1.022245	0.52137664	0.780267
69282	1700001J03Rik	RIKEN cDNA 1700001J03 gene, transcript variant X2	NA	0.18956	0.407655	1.140416	0.52150565	0.780388
69504	Zfp932	zinc finger protein 932, transcript variant X2	NA	0.050384	5.975087	1.035541	0.52158117	0.780429
268395	Mpg	N-methylpurine-DNA glycosylase	NA	0.064033	4.26886	1.045384	0.52164659	0.780455
58229	Efcc1	EF hand and coiled-coil domain containing 1, transcri	NA	0.074359	3.278369	1.052893	0.52173723	0.780519
629242	Gm6958	predicted gene 6958	NA	-0.06381	4.283573	-1.04522	0.5219297	0.780735

54672	Adgrg3	adhesion G protein-coupled receptor G3	NA		-0.21817	0.219271	-1.16326	0.52205096	0.780845
227937	Pkp4	plakophilin 4, transcript variant X23	NA		-0.03019	6.647511	-1.02115	0.52228776	0.781127
75612	Gns	glucosamine (N-acetyl)-6-sulfatase, transcript variant	NA		-0.03371	6.356507	-1.02364	0.52243619	0.781171
105243282	4922502B01Rik	RIKEN cDNA 4922502B01 gene	NA		0.049144	4.665461	1.034651	0.52248766	0.781171
66042	Sostdc1	sclerostin domain containing 1	NA		-0.09519	3.292915	-1.06821	0.52252915	0.781171
23863	Dand5	DAN domain family member 5, BMP antagonist, trans	NA		-0.09935	2.274612	-1.07129	0.52253014	0.781171
328186	Gm10336	predicted gene 10336	NA		-0.06317	4.016836	-1.04476	0.52255709	0.781171
27060	Tcirg1	T cell, immune regulator 1, ATPase, H+ transporting,	NA		-0.07588	3.183446	-1.05401	0.52278411	0.78138
79560	Ublcp1	ubiquitin-like domain containing CTD phosphatase 1	NA		0.036709	5.467584	1.025771	0.52279293	0.78138
76041	Ccdc125	coiled-coil domain containing 125, transcript variant X	NA		-0.15655	0.915497	-1.11462	0.52301679	0.781572
72107	Dscc1	DNA replication and sister chromatid cohesion 1, tran	NA		-0.1128	1.483414	-1.08132	0.52301731	0.781572
216543	Cep68	centrosomal protein 68	NA		0.039197	5.358019	1.027542	0.52311465	0.781599
26406	Map3k3	mitogen-activated protein kinase kinase kinase 3	NA		0.04199	5.247846	1.029533	0.52316552	0.781599
230967	Cep104	centrosomal protein 104, transcript variant X1	NA		-0.04	5.235433	-1.02812	0.52317971	0.781599
207932	Urb1	URB1 ribosome biogenesis 1 homolog (S. cerevisiae	NA		-0.05489	4.063578	-1.03878	0.52334508	0.781751
216963	Git1	GIT ArfGAP 1, transcript variant 1	NA		-0.03112	7.079015	-1.02181	0.52337732	0.781751
20496	Slc12a2	solute carrier family 12, member 2	NA		0.038285	5.93823	1.026892	0.52352782	0.781801
69923	Agk	acylglycerol kinase	NA		-0.04186	5.439935	-1.02944	0.52360015	0.781801
22340	Vegfb	vascular endothelial growth factor B, transcript varian	NA		-0.05288	5.081966	-1.03734	0.52363712	0.781801
74148	Cluh	clustered mitochondria (cluA/CLU1) homolog, transcr	NA		-0.03986	5.742028	-1.02802	0.52367256	0.781801
320655	Pgap3	post-GPI attachment to proteins 3	NA		0.076017	2.996784	1.054104	0.5237216	0.781801
68966	Ngdn	neuroguidin, EIF4E binding protein	NA		-0.05287	4.425701	-1.03733	0.52372459	0.781801
74901	Kbtbd11	kelch repeat and BTB (POZ) domain containing 11, tr	NA		0.034314	5.982909	1.02407	0.52374688	0.781801
217779	Lysmd1	LysM, putative peptidoglycan-binding, domain contair	NA		-0.04093	4.990816	-1.02878	0.52387944	0.781869
11754	Aoc3	amine oxidase, copper containing 3, transcript varian	NA		-0.16634	0.963684	-1.12221	0.52388907	0.781869
100503868	Gm19935	predicted gene, 19935	NA		0.221991	0.13449	1.166342	0.5239703	0.78187
22245	Uck1	uridine-cytidine kinase 1, transcript variant 3	NA		0.039257	5.514808	1.027585	0.52399278	0.78187
80888	Hspb8	heat shock protein 8	NA		0.102505	2.915048	1.073636	0.52403376	0.78187
15898	Icam5	intercellular adhesion molecule 5, telencephalin	NA		-0.16719	1.122152	-1.12287	0.52414194	0.78196
102631979	LOC102631979	uncharacterized LOC102631979	NA		0.058748	3.60934	1.041562	0.52428364	0.78205
69639	Exosc8	exosome component 8, transcript variant 2	NA		0.050812	4.829396	1.035848	0.52429865	0.78205
74552	Nipal3	NIPA-like domain containing 3, transcript variant 2	NA		-0.04297	5.493875	-1.03023	0.52438511	0.782108
104346	Gas8	growth arrest specific 8	NA		-0.04172	5.440981	-1.02934	0.52450767	0.782219
102638768	Gm35253	predicted gene, 35253, transcript variant X1	NA		-0.1712	1.533559	-1.126	0.52464938	0.782358
105244956	Gm40474	predicted gene, 40474, transcript variant X3	NA		0.164754	1.38623	1.120975	0.5247747	0.782474
57775	Usp29	ubiquitin specific peptidase 29, transcript variant X1	NA		0.037915	6.777475	1.026629	0.5248956	0.782582
208691	Eif5a2	eukaryotic translation initiation factor 5A2	NA		0.069986	4.267374	1.049707	0.5249586	0.782592
27998	Exosc5	exosome component 5, transcript variant 1	NA		-0.07098	3.022588	-1.05043	0.52501962	0.782592
280668	Adam1a	a disintegrin and metallopeptidase domain 1a	NA		0.079704	4.037638	1.056801	0.52504652	0.782592
77035	Kdm8	lysine (K)-specific demethylase 8	NA		-0.09088	2.521512	-1.06502	0.52520823	0.782701
18030	Nfil3	nuclear factor, interleukin 3, regulated	NA		-0.0437	5.157419	-1.03076	0.5252233	0.782701
22123	Psmd3	proteasome (prosome, macropain) 26S subunit, non-	NA		-0.0316	6.896913	-1.02215	0.52526391	0.782701
69581	Rhou	ras homolog family member U	NA		-0.04074	5.525718	-1.02864	0.52537027	0.782777
22225	Usp5	ubiquitin specific peptidase 5 (isopeptidase T), transc	NA		-0.03079	7.443061	-1.02157	0.5254365	0.782777
12503	Cd247	CD247 antigen, transcript variant X4	NA		0.16132	0.451121	1.11831	0.52545944	0.782777
245174	Zfp937	zinc finger protein 937	NA		-0.05368	3.87498	-1.03791	0.52574446	0.78313
67087	Ctnnbip1	catenin beta interacting protein 1, transcript variant 1	NA		0.036402	6.653979	1.025553	0.52579575	0.783135
68041	Mid1ip1	Mid1 interacting protein 1 (gastrulation specific G12-li	NA		0.041957	6.093563	1.029509	0.52587098	0.783175
13446	Doc2a	double C2, alpha, transcript variant 2	NA		0.067191	4.02452	1.047675	0.52603221	0.783269
69053	1810013L24Rik	RIKEN cDNA 1810013L24 gene	NA		-0.03772	5.968804	-1.02649	0.52604265	0.783269
108169046	Gm46914	predicted gene, 46914	NA		-0.16275	1.463193	-1.11942	0.52607845	0.783269
66694	Uqcrfs1	ubiquinol-cytochrome c reductase, Rieske iron-sulfur	NA		0.036039	6.746072	1.025295	0.52616738	0.78333
22038	Plscr1	phospholipid scramblase 1, transcript variant X1	NA		0.104856	2.057441	1.075387	0.52630968	0.783464
104625	Cnot6	CCR4-NOT transcription complex, subunit 6, transcri	NA		0.038574	7.526552	1.027098	0.52635409	0.783464
234388	Ccdc124	coiled-coil domain containing 124, transcript variant X	NA		-0.0388	5.702587	-1.02726	0.52659511	0.783752
118567557	LOC118567557	uncharacterized LOC118567557	NA		-0.18159	0.873575	-1.13413	0.52670892	0.783849
22773	Zic3	zinc finger protein of the cerebellum 3	NA		0.051732	5.059311	1.036509	0.5267644	0.78386
12387	Ctnnb1	catenin (cadherin associated protein), beta 1, transcri	NA		-0.02882	9.639546	-1.02018	0.52683505	0.783894
105242891	Gm31024	predicted gene, 31024	NA		0.19987	0.04286	1.148595	0.5269683	0.78402
244958	Mrap2	melanocortin 2 receptor accessory protein 2, transcri	NA		0.154413	1.120461	1.112969	0.52707261	0.784088
20393	Sgk1	serum/glucocorticoid regulated kinase 1, transcript va	NA		0.044255	5.031846	1.03115	0.52716812	0.784088
18013	Neurod2	neurogenic differentiation 2	NA		-0.0542	7.70747	-1.03829	0.52717189	0.784088
66662	Fbxl12os	F-box and leucine-rich repeat protein 12, opposite str	NA		-0.0931	2.125695	-1.06666	0.52724223	0.784088
67556	Pigm	phosphatidylinositol glycan anchor biosynthesis, clas	NA		-0.04088	4.876292	-1.02874	0.52728017	0.784088
23871	Ets1	E26 avian leukemia oncogene 1, 5' domain, transcrip	NA		-0.03983	5.122028	-1.02799	0.5273133	0.784088
78928	Pigt	phosphatidylinositol glycan anchor biosynthesis, clas	NA		0.030706	6.908396	1.021512	0.5274608	0.784088
59038	Pxmp4	peroxisomal membrane protein 4, transcript variant X	NA		-0.07165	3.350203	-1.05092	0.52746649	0.784088
107173	Gpr137	G protein-coupled receptor 137, transcript variant 1	NA		-0.0386	5.331066	-1.02712	0.52748268	0.784088

76932	Arfip2	ADP-ribosylation factor interacting protein 2	NA	-0.04125	6.198229	-1.02901	0.52752988	0.784088
23988	Pin1	protein (peptidyl-prolyl cis/trans isomerase) NIMA-inte	NA	0.037786	5.309346	1.026537	0.52761299	0.784088
72140	Cep89	centrosomal protein 89, transcript variant X3	NA	-0.05213	4.654464	-1.03679	0.52762881	0.784088
229517	Slc25a44	solute carrier family 25, member 44, transcript variant	NA	-0.03446	5.910697	-1.02418	0.52768151	0.784088
56274	Stk3	serine/threonine kinase 3, transcript variant 1	NA	0.047171	4.487489	1.033237	0.52768864	0.784088
212679	Mars2	methionine-tRNA synthetase 2 (mitochondrial)	NA	-0.08599	2.308343	-1.06142	0.52804246	0.784542
66214	Rgcc	regulator of cell cycle	NA	0.071411	2.961237	1.050744	0.5283278	0.784799
93679	Trim8	tripartite motif-containing 8	NA	0.031293	6.754409	1.021928	0.52833435	0.784799
93880	Pcdhb9	protocadherin beta 9	NA	0.078391	2.92921	1.05584	0.52835988	0.784799
102634481	9330159H11Rik	RIKEN cDNA 9330159H11 gene	NA	0.099005	3.164756	1.071034	0.52844289	0.784851
76938	Rbm17	RNA binding motif protein 17	NA	0.037276	6.495049	1.026175	0.52860611	0.785021
108138	Xrcc4	X-ray repair complementing defective repair in Chines	NA	0.093077	3.401657	1.066643	0.52866657	0.785039
66257	Nicn1	nicolin 1, transcript variant 1	NA	0.041301	6.370414	1.029042	0.52883924	0.785138
118567625	LOC118567625	uncharacterized LOC118567625	NA	-0.1839	0.06983	-1.13595	0.52886023	0.785138
19253	Ptpn18	protein tyrosine phosphatase, non-receptor type 18, t	NA	-0.1918	-0.13541	-1.14219	0.52887796	0.785138
67474	Snap29	synaptosomal-associated protein 29	NA	-0.04304	5.885902	-1.03028	0.52898756	0.785229
53861	Zranb2	zinc finger, RAN-binding domain containing 2, transcı	NA	0.032297	7.222529	1.022639	0.5291361	0.785279
21835	Thrsp	thyroid hormone responsive	NA	0.157817	0.881565	1.115598	0.52915127	0.785279
13494	Drg1	developmentally regulated GTP binding protein 1	NA	0.031611	6.463189	1.022153	0.52916564	0.785279
216829	Mmgt2	membrane magnesium transporter 2, transcript variai	NA	0.056298	3.849388	1.039794	0.52928884	0.78539
73182	Pear1	platelet endothelial aggregation receptor 1, transcript	NA	-0.0691	3.352499	-1.04906	0.52948108	0.785604
102634738	Gm28154	predicted gene 28154, transcript variant X11	NA	0.203439	0.461505	1.15144	0.52961189	0.785691
77446	Heg1	heart development protein with EGF-like domains 1, t	NA	-0.03502	5.812118	-1.02457	0.52963631	0.785691
20378	Frzb	frizzled-related protein	NA	0.103768	2.605205	1.074577	0.52980036	0.785848
243834	Zfp324	zinc finger protein 324, transcript variant 1	NA	0.050725	4.363874	1.035785	0.52983899	0.785848
269701	Wdr66	WD repeat domain 66, transcript variant X1	NA	-0.10575	2.422659	-1.07605	0.52993032	0.785871
22685	Zfp239	zinc finger protein 239, transcript variant 1	NA	0.057341	3.976944	1.040546	0.52995075	0.785871
21682	Tec	tec protein tyrosine kinase, transcript variant X5	NA	-0.09021	2.34686	-1.06453	0.53002004	0.785902
195646	Hs3st2	heparan sulfate (glucosamine) 3-O-sulfotransferase 2	NA	0.059079	3.77345	1.041801	0.53006829	0.785902
59054	Mrps30	mitochondrial ribosomal protein S30	NA	0.057923	4.720215	1.040966	0.53016822	0.785978
12043	Bcl2	B cell leukemia/lymphoma 2, transcript variant 1	NA	0.043035	5.623321	1.030279	0.53034475	0.786168
50915	Grb14	growth factor receptor bound protein 14, transcript va	NA	0.065575	4.475641	1.046502	0.53040578	0.786187
233016	Blvrb	biliverdin reductase B (flavin reductase (NADPH)), tra	NA	0.120903	2.641643	1.087415	0.530525	0.786292
13717	Eln	elastin	NA	-0.03796	5.600944	-1.02666	0.53066867	0.786397
193670	Rnf185	ring finger protein 185, transcript variant X1	NA	0.040126	5.483277	1.028204	0.53069203	0.786397
11739	Slc25a4	solute carrier family 25 (mitochondrial carrier, adenine	NA	0.035696	8.740985	1.025052	0.53076394	0.786432
14381	G6pdx	glucose-6-phosphate dehydrogenase X-linked	NA	-0.04249	6.054195	-1.02989	0.53090253	0.786565
69459	Ubl7	ubiquitin-like 7 (bone marrow stromal cell-derived), tra	NA	-0.05368	5.739415	-1.03791	0.53112656	0.786747
213575	Dync2li1	dynein cytoplasmic 2 light intermediate chain 1	NA	-0.05269	4.370199	-1.0372	0.53115431	0.786747
118568007	LOC118568007	uncharacterized LOC118568007	NA	0.265693	-0.24479	1.202214	0.53121848	0.786747
12941	Pcdha5	protocadherin alpha 5	NA	0.063914	4.24246	1.045298	0.53122698	0.786747
115490374	Gm52878	predicted gene, 52878	NA	-0.12344	1.35235	-1.08933	0.53130659	0.786747
217995	Heatr1	HEAT repeat containing 1	NA	-0.04712	4.98681	-1.0332	0.53131516	0.786747
407243	Tmem189	transmembrane protein 189	NA	0.046644	4.714563	1.03286	0.53146944	0.786786
70101	Cyp4f16	cytochrome P450, family 4, subfamily f, polypeptide 1	NA	-0.06487	3.499089	-1.04599	0.53148534	0.786786
77200	5430403G16Rik	RIKEN cDNA 5430403G16 gene	NA	-0.13198	1.046497	-1.0958	0.53153137	0.786786
52538	Acaa2	acetyl-Coenzyme A acyltransferase 2 (mitochondrial	NA	-0.04868	4.687612	-1.03432	0.53154999	0.786786
406217	Bex4	brain expressed X-linked 4, transcript variant 1	NA	0.038652	5.780862	1.027154	0.53158384	0.786786
17764	Mtf1	metal response element binding transcription factor 1	NA	0.044536	4.694072	1.031352	0.53166226	0.786786
218236	Fam120a	family with sequence similarity 120, member A	NA	-0.03219	7.068846	-1.02256	0.53168038	0.786786
330171	Kctd10	potassium channel tetramerisation domain containing	NA	-0.03146	6.224808	-1.02205	0.53190341	0.787045
17087	Ly96	lymphocyte antigen 96, transcript variant 1	NA	-0.2671	-0.33883	-1.20338	0.53201397	0.787098
15170	Ptpn6	protein tyrosine phosphatase, non-receptor type 6, tra	NA	-0.12082	1.416801	-1.08735	0.53203602	0.787098
118567379	LOC118567379	uncharacterized LOC118567379	NA	-0.09077	2.138736	-1.06494	0.53209681	0.787108
72016	Tedc2	tubulin epsilon and delta complex 2, transcript variant	NA	-0.05703	3.795613	-1.04032	0.53213958	0.787108
20530	Slc31a2	solute carrier family 31, member 2, transcript variant	NA	-0.06425	3.555018	-1.04554	0.53227981	0.787222
13797	Emx2	empty spiracles homeobox 2	NA	-0.04641	4.97297	-1.03269	0.53232444	0.787222
75826	Senp2	SUMO/sentrin specific peptidase 2, transcript variant	NA	-0.03689	6.362516	-1.0259	0.53240958	0.787222
74480	Samd4	sterile alpha motif domain containing 4, transcript var	NA	-0.05021	4.381489	-1.03542	0.53241008	0.787222
99586	Dpyd	dihydropyrimidine dehydrogenase	NA	-0.11085	2.101077	-1.07986	0.53250552	0.787291
338523	Kdm7a	lysine (K)-specific demethylase 7A, transcript variant	NA	-0.04843	5.172199	-1.03414	0.5326397	0.787381
239591	Ttll8	tubulin tyrosine ligase-like family, member 8, transcrip	NA	0.21166	0.382986	1.15802	0.53269084	0.787381
14069	F8	coagulation factor VIII, transcript variant X3	NA	0.127485	1.090019	1.092388	0.53272487	0.787381
80292	Zxdc	ZXD family zinc finger C, transcript variant 2	NA	0.042013	4.90093	1.02955	0.53275961	0.787381
22163	Tnfrsf4	tumor necrosis factor receptor superfamily, member 4	NA	0.197223	0.059838	1.146489	0.5328491	0.787442
69577	Fastkd3	FAST kinase domains 3, transcript variant X7	NA	0.067118	3.682392	1.047622	0.53307162	0.787528
116810	Foxn4	forkhead box N4	NA	-0.23271	-0.49488	-1.17504	0.53308203	0.787528
17434	Mocs2	molybdenum cofactor synthesis 2, transcript variant X	NA	0.04118	5.535114	1.028955	0.53309296	0.787528

74782	Glt8d2	glycosyltransferase 8 domain containing 2	NA	-0.16587	1.376222	-1.12185	0.53310134	0.787528
666794	Rbm24	RNA binding motif protein 24	NA	-0.04399	4.515115	-1.03096	0.53324063	0.787636
22687	Zpr1	ZPR1 zinc finger	NA	-0.03954	5.429787	-1.02778	0.53327092	0.787636
93886	Pcdhb15	protocadherin beta 15	NA	0.156151	1.388829	1.114311	0.53345522	0.787837
105245236	Gm19802	predicted gene, 19802	NA	-0.544	-1.70061	-1.45801	0.53357921	0.787918
79554	Cptp	ceramide-1-phosphate transfer protein	NA	0.068837	4.364412	1.048871	0.53360752	0.787918
19682	Rdh5	retinol dehydrogenase 5, transcript variant X3	NA	-0.12021	1.575142	-1.08689	0.53367643	0.787949
68876	Atp23	ATP23 metallopeptidase and ATP synthase assembl	NA	0.089197	2.70342	1.063778	0.53375212	0.787989
68197	Ndufc2	NADH:ubiquinone oxidoreductase subunit C2	NA	0.056534	6.459405	1.039964	0.5340821	0.788405
108030	Lin7a	lin-7 homolog A (C. elegans), transcript variant 1	NA	-0.0442	5.523388	-1.03111	0.53413975	0.788418
21763	Tex2	testis expressed gene 2	NA	-0.04444	5.954926	-1.03128	0.53426927	0.788508
22040	Trex1	three prime repair exonuclease 1, transcript variant 1	NA	-0.0662	3.058892	-1.04695	0.53429761	0.788508
102640165	Gm36298	predicted gene, 36298	NA	0.110375	1.899757	1.079509	0.53439813	0.788543
269831	Tspan12	tetraspanin 12, transcript variant X2	NA	0.046885	4.612509	1.033032	0.53441835	0.788543
69757	Leng1	leukocyte receptor cluster (LRC) member 1	NA	-0.06193	3.635839	-1.04386	0.53465089	0.78872
80287	Apobec3	apolipoprotein B mRNA editing enzyme, catalytic poly	NA	-0.11385	2.467609	-1.08211	0.53472855	0.78872
21374	Tbp	TATA box binding protein	NA	0.046237	5.122581	1.032568	0.53473098	0.78872
99010	Lpcat4	lysophosphatidylcholine acyltransferase 4	NA	-0.05549	4.24592	-1.03921	0.53473177	0.78872
30795	Fkbp3	FK506 binding protein 3	NA	0.032581	6.774574	1.022841	0.53483519	0.788801
245631	Pwwp3b	PWWP domain containing 3B, transcript variant 2	NA	0.069163	3.213879	1.049108	0.53499036	0.788958
100764	Rita1	RBPJ interacting and tubulin associated 1, transcript	NA	-0.06692	3.314446	-1.04748	0.53508645	0.78898
72244	1600014C10Rik	RIKEN cDNA 1600014C10 gene, transcript variant 2	NA	-0.05048	4.530976	-1.03561	0.53519466	0.78898
72179	Fbxl2	F-box and leucine-rich repeat protein 2	NA	-0.04531	4.778842	-1.03191	0.53522283	0.78898
102639339	Gm26559	predicted gene, 26559, transcript variant 1	NA	-0.22928	-0.36958	-1.17225	0.5352932	0.78898
104010	Cdh22	cadherin 22	NA	-0.06698	4.596956	-1.04753	0.53530553	0.78898
72289	Malat1	metastasis associated lung adenocarcinoma transcri	NA	-0.06291	10.5558	-1.04457	0.53534359	0.78898
19227	Pthlh	parathyroid hormone-like peptide	NA	-0.20939	0.090628	-1.1562	0.53534486	0.78898
108167911	Gm46287	predicted gene, 46287, transcript variant X5	NA	-0.17001	1.021104	-1.12507	0.53543718	0.788989
71517	Vps35l	VPS35 endosomal protein sorting factor like	NA	-0.03002	6.48183	-1.02103	0.53544769	0.788989
54683	Prdx5	peroxiredoxin 5, transcript variant 1	NA	0.042553	6.317129	1.029935	0.53562794	0.789183
76524	Cln6	ceroid-lipofuscinosis, neuronal 6	NA	0.067307	3.267169	1.047759	0.53589592	0.789341
12794	Cnih2	cornichon family AMPA receptor auxiliary protein 2, tr	NA	0.041589	7.505809	1.029247	0.53590653	0.789341
320478	Sox2ot	SOX2 overlapping transcript (non-protein coding)	NA	-0.03993	5.574376	-1.02807	0.53596773	0.789341
226144	Erlin1	ER lipid raft associated 1, transcript variant 3	NA	-0.04489	4.715768	-1.03161	0.5359826	0.789341
629147	Ctxn3	cortexin 3	NA	0.183524	0.481503	1.135654	0.53601884	0.789341
20084	Rps18	ribosomal protein S18	NA	0.031775	8.233556	1.022269	0.53602628	0.789341
94215	Ugt2a1	UDP glucuronosyltransferase 2 family, polypeptide A	NA	0.418394	-0.59884	1.336439	0.53615403	0.789378
78321	Ankrd23	ankyrin repeat domain 23, transcript variant X2	NA	-0.07435	2.908435	-1.05289	0.53621054	0.789378
245049	Myrip	myosin VIIA and Rab interacting protein	NA	0.061603	3.745639	1.043625	0.53622927	0.789378
108679	Cops8	COP9 signalosome subunit 8	NA	0.03328	6.435404	1.023336	0.53624543	0.789378
19364	Rad51d	RAD51 paralog D, transcript variant X1	NA	-0.05687	4.052184	-1.0402	0.53636151	0.789457
108755	Lyrm2	LYR motif containing 2	NA	-0.04195	5.072913	-1.02951	0.53644815	0.789457
66245	Hspbp1	HSPA (heat shock 70kDa) binding protein, cytoplasm	NA	0.039865	5.168231	1.028018	0.53648255	0.789457
21678	Tead3	TEA domain family member 3, transcript variant 2	NA	-0.06104	3.620126	-1.04322	0.53665635	0.789457
75914	Exoc6b	exocyst complex component 6B	NA	0.031871	6.508294	1.022337	0.53667419	0.789457
15159	Hccs	holocytochrome c synthetase, transcript variant 3	NA	0.048252	4.747477	1.034012	0.53669252	0.789457
22648	Zfp11	zinc finger protein 11	NA	-0.06442	3.445145	-1.04566	0.53672699	0.789457
102636484	Gm33539	predicted gene, 33539, transcript variant X3	NA	0.243561	-0.48977	1.183911	0.53675339	0.789457
60364	Donson	downstream neighbor of SON	NA	-0.04465	4.694931	-1.03144	0.53677115	0.789457
239133	Dleu7	deleted in lymphocytic leukemia, 7	NA	-0.08508	3.359308	-1.06075	0.53678441	0.789457
320706	Soga1	suppressor of glucose, autophagy associated 1, trans	NA	-0.0318	8.266272	-1.02229	0.53684597	0.789477
102636677	Gm33680	predicted gene, 33680, transcript variant X3	NA	-0.13385	0.803746	-1.09722	0.53690768	0.789496
73836	Slc35b2	solute carrier family 35, member B2	NA	-0.0392	5.049852	-1.02754	0.53713178	0.789697
11889	Asgr1	asialoglycoprotein receptor 1, transcript variant 2	NA	0.162635	0.465335	1.11933	0.53714126	0.789697
208650	Cblb	Casitas B-lineage lymphoma b	NA	-0.03291	6.114386	-1.02307	0.5372787	0.789763
12427	Ccna1	cyclin A1, transcript variant X15	NA	0.231813	0.312952	1.17431	0.53728327	0.789763
16924	Lnx1	ligand of numb-protein X 1, transcript variant 4	NA	-0.06169	3.439425	-1.04369	0.53737717	0.789797
75016	4930480K23Rik	RIKEN cDNA 4930480K23 gene, transcript variant 1	NA	0.139471	1.187052	1.101501	0.53744855	0.789797
66206	1110059E24Rik	RIKEN cDNA 1110059E24 gene	NA	0.044778	4.920098	1.031524	0.53745239	0.789797
28126	Nop16	NOP16 nucleolar protein	NA	0.050778	4.960784	1.035823	0.53750746	0.789802
105245433	Gm40889	predicted gene, 40889	NA	0.184807	0.110178	1.136665	0.53757045	0.789802
228005	Ppig	peptidyl-prolyl isomerase G (cyclophilin G), transcript	NA	0.032428	6.781941	1.022732	0.53764368	0.789802
83946	Phip	pleckstrin homology domain interacting protein	NA	-0.0326	7.267047	-1.02286	0.53764966	0.789802
109304	B230118I11Rik	RIKEN cDNA B230118I11 gene, transcript variant X2	NA	0.20379	-0.35642	1.15172	0.53784693	0.79002
243914	Lgi4	leucine-rich repeat LGI family, member 4, transcript v	NA	-0.18394	0.547336	-1.13598	0.53799119	0.790066
76199	Med13l	mediator complex subunit 13-like, transcript variant 2	NA	0.036991	6.521076	1.025972	0.53800583	0.790066
68841	Lppos	LIM domain containing preferred translocation partne	NA	-0.16035	0.864445	-1.11756	0.5380234	0.790066
56453	Mbtps1	membrane-bound transcription factor peptidase, site	NA	-0.03232	6.683177	-1.02265	0.53809255	0.790096

245440	Gm4988	predicted gene 4988	NA	0.134824	1.236818	1.097959	0.53814644	0.790104
238693	Zfp58	zinc finger protein 58	NA	-0.06844	3.391219	-1.04859	0.53867762	0.790812
12816	Col12a1	collagen, type XII, alpha 1	NA	0.06341	4.961056	1.044933	0.53877149	0.790879
414072	BC031361	cDNA sequence BC031361	NA	-0.07959	2.556221	-1.05672	0.53903395	0.791107
76157	Slc35d3	solute carrier family 35, member D3	NA	0.114015	1.546605	1.082236	0.53905586	0.791107
74268	Aven	apoptosis, caspase activation inhibitor, transcript vari	NA	0.080159	2.637835	1.057134	0.53907263	0.791107
69807	Trim32	tripartite motif-containing 32, transcript variant 1	NA	-0.02748	7.397666	-1.01923	0.53933905	0.791409
74414	Polr3c	polymerase (RNA) III (DNA directed) polypeptide C, t	NA	-0.04909	4.921623	-1.03461	0.53942177	0.791409
235674	Acaa1b	acetyl-Coenzyme A acyltransferase 1B, transcript var	NA	-0.19089	0.076535	-1.14146	0.53942454	0.791409
73172	Exo5	exonuclease 5, transcript variant 1	NA	-0.05115	4.97651	-1.03609	0.5396687	0.79167
328108	Togaram1	TOG array regulator of axonemal microtubules 1, trar	NA	-0.04441	4.808684	-1.03126	0.53969957	0.79167
238722	Zfp72	zinc finger protein 72, transcript variant 2	NA	0.08584	3.299547	1.061306	0.53977368	0.791707
20410	Sorbs3	sorbin and SH3 domain containing 3, transcript variar	NA	0.052393	4.161304	1.036983	0.53999738	0.791964
118568137	LOC118568137	uncharacterized LOC118568137	NA	0.21478	-0.33459	1.160527	0.54006606	0.791993
252876	Gin1	gypsy retrotransposon integrase 1, transcript variant :	NA	-0.06129	3.526142	-1.0434	0.54021349	0.792138
735265	Mir703	microRNA 703	NA	0.107756	1.826399	1.077551	0.54034036	0.792253
22757	Zkscan5	zinc finger with KRAB and SCAN domains 5, transcri|	NA	0.046048	4.536731	1.032433	0.54060325	0.792484
229731	Slc25a24	solute carrier family 25 (mitochondrial carrier, phosph	NA	-0.07392	3.652932	-1.05257	0.54063155	0.792484
17427	Mns1	meiosis-specific nuclear structural protein 1	NA	-0.09002	2.811136	-1.06438	0.54064465	0.792484
17330	Minpp1	multiple inositol polyphosphate histidine phosphatase	NA	0.041762	5.408858	1.02937	0.54075384	0.792573
67444	Ilkap	integrin-linked kinase-associated serine/threonine ph	NA	-0.037	5.859856	-1.02598	0.54089367	0.792683
27370	Rps26	ribosomal protein S26	NA	0.052261	8.424594	1.036889	0.54092602	0.792683
235584	Dusp7	dual specificity phosphatase 7	NA	-0.04326	5.19466	-1.03044	0.54097661	0.792685
68852	Lrrn4cl	LRRN4 C-terminal like, transcript variant 2	NA	-0.20161	0.832641	-1.14998	0.54112206	0.792781
14225	Fkbp1a	FK506 binding protein 1a, transcript variant 1	NA	-0.02656	8.079149	-1.01858	0.54113935	0.792781
544752	Tug1	taurine upregulated gene 1, transcript variant a	NA	-0.02686	7.963048	-1.01879	0.54124822	0.792808
320541	Slc35e2	solute carrier family 35, member E2, transcript varian	NA	0.038616	5.985654	1.027128	0.54125505	0.792808
78892	Crispld2	cysteine-rich secretory protein LCCL domain containi	NA	-0.12389	1.637802	-1.08967	0.54131952	0.792831
30954	Siva1	SIVA1, apoptosis-inducing factor, transcript variant 1	NA	-0.08185	3.12887	-1.05837	0.54141313	0.792897
65079	Rtn4r	reticulon 4 receptor	NA	0.050787	4.499974	1.03583	0.54154103	0.79296
109689	Arrb1	arrestin, beta 1, transcript variant X6	NA	0.033903	7.227897	1.023778	0.541617	0.79296
12810	Coch	cochlin, transcript variant 2	NA	-0.06406	3.494451	-1.0454	0.54165084	0.79296
100503120	A930006K02Rik	RIKEN cDNA A930006K02 gene	NA	0.125346	1.111038	1.090769	0.54172398	0.79296
102636130	Gm14061	predicted gene 14061, transcript variant X3	NA	0.225111	-0.58797	1.168867	0.54177678	0.79296
110350	Dync2h1	dynein cytoplasmic 2 heavy chain 1, transcript varian	NA	0.044152	4.841024	1.031077	0.54180257	0.79296
66548	Adamtsl5	ADAMTS-like 5, transcript variant 3	NA	-0.26428	-0.37315	-1.20104	0.54181234	0.79296
76602	1700040D17Rik	RIKEN cDNA 1700040D17 gene	NA	-0.14638	0.65013	-1.10679	0.5418463	0.79296
11304	Abca4	ATP-binding cassette, sub-family A (ABC1), member	NA	0.094906	2.126607	1.067996	0.54191131	0.79297
57439	Tmem183a	transmembrane protein 183A, transcript variant 1	NA	-0.031	7.050952	-1.02172	0.54195036	0.79297
107527	Il1rl2	interleukin 1 receptor-like 2, transcript variant X4	NA	-0.15046	0.963136	-1.10992	0.54211258	0.793136
12942	Pcdha11	protocadherin alpha 11	NA	0.095378	3.664401	1.068345	0.5423575	0.793422
80283	Abtb1	ankyrin repeat and BTB (POZ) domain containing 1, t	NA	0.059091	4.209226	1.041809	0.54240767	0.793422
11733	Ank1	ankyrin 1, erythroid, transcript variant 11	NA	-0.04346	5.243101	-1.03058	0.54245436	0.793422
112418	1700102P08Rik	RIKEN cDNA 1700102P08 gene, transcript variant X	NA	-0.15282	1.684953	-1.11174	0.54254858	0.793488
23837	Cfdp1	craniofacial development protein 1, transcript variant	NA	-0.02893	6.758128	-1.02026	0.54263343	0.793541
100041515	Gm3383	predicted gene 3383, transcript variant 1	NA	0.056559	4.253099	1.039982	0.54276585	0.793607
19725	Rfx2	regulatory factor X, 2 (influences HLA class II expres	NA	-0.09331	2.699492	-1.06681	0.54279921	0.793607
11480	Acvr2a	activin receptor IIA	NA	0.03924	7.050758	1.027572	0.54288768	0.793607
16170	Il16	interleukin 16, transcript variant 4	NA	-0.16735	0.652738	-1.12299	0.54291364	0.793607
18033	Nfkb1	nuclear factor of kappa light polypeptide gene enhan	NA	-0.04506	4.492311	-1.03173	0.5429223	0.793607
20195	S100a11	S100 calcium binding protein A11	NA	0.059765	4.697583	1.042296	0.54302868	0.793684
68323	Nudt22	nudix (nucleoside diphosphate linked moiety X)-type	NA	0.093441	2.210353	1.066912	0.54307275	0.793684
70717	Medag	mesenteric estrogen dependent adipogenesis, transc	NA	-0.0868	2.179869	-1.06201	0.54333673	0.793999
115487471	LOC115487471	uncharacterized LOC115487471	NA	-0.06931	6.047024	-1.04922	0.54343824	0.794076
76505	1500009C09Rik	RIKEN cDNA 1500009C09 gene, transcript variant 1	NA	-0.04099	5.077282	-1.02882	0.54363431	0.794262
19152	Prtn3	proteinase 3	NA	-0.13272	1.029786	-1.09636	0.54369883	0.794262
66498	Dda1	DET1 and DDB1 associated 1, transcript variant 1	NA	-0.03154	6.747881	-1.0221	0.54372775	0.794262
70484	Slc35d2	solute carrier family 35, member D2, transcript varian	NA	-0.17526	0.047769	-1.12917	0.54376519	0.794262
210146	Irgq	immunity-related GTPase family, Q	NA	-0.0283	7.242649	-1.01981	0.5438407	0.794262
66070	Cwc15	CWC15 spliceosome-associated protein	NA	0.04593	6.144207	1.032349	0.54385881	0.794262
16764	Aff3	AF4/FMR2 family, member 3, transcript variant X7	NA	0.031334	6.78901	1.021957	0.54390863	0.794264
69878	Snrpf	small nuclear ribonucleoprotein polypeptide F	NA	-0.0456	5.381314	-1.03212	0.54410753	0.794483
66337	Fam229b	family with sequence similarity 229, member B, trans	NA	0.083629	2.631984	1.05968	0.54419254	0.794526
17192	Mbd3	methyl-CpG binding domain protein 3, transcript varia	NA	-0.02969	6.711199	-1.02079	0.544294	0.794526
69672	Txndc15	thioredoxin domain containing 15	NA	0.033204	5.764755	1.023282	0.54431177	0.794526
171211	Edaradd	EDAR (ectodysplasin-A receptor)-associated death d	NA	-0.11065	2.127646	-1.07971	0.5443326	0.794526
14375	Xrcc6	X-ray repair complementing defective repair in Chine	NA	0.041628	4.825223	1.029275	0.5446133	0.794865
22319	Vamp3	vesicle-associated membrane protein 3	NA	0.038449	5.59797	1.027009	0.54468182	0.794894

378954	3000002C10Rik	RIKEN cDNA 3000002C10 gene	NA	-0.09636	1.986406	-1.06907	0.54489637	0.795065
118568381	LOC118568381	uncharacterized LOC118568381	NA	-0.17186	0.196193	-1.12651	0.54489692	0.795065
102639537	Gm35828	predicted gene, 35828, transcript variant X2	NA	-0.11387	1.457809	-1.08213	0.54496191	0.795088
13544	Dvl3	dishevelled segment polarity protein 3, transcript vari	NA	0.030866	7.234987	1.021625	0.54506047	0.795121
104360	Isl2	insulin related protein 2 (islet 2), transcript variant X1	NA	0.239895	0.499817	1.180906	0.54508201	0.795121
27275	Nufip1	nuclear fragile X mental retardation protein interacting	NA	0.053881	4.543715	1.038054	0.54519686	0.7952
68431	Fbxl15	F-box and leucine-rich repeat protein 15, transcript va	NA	0.08741	3.078805	1.062461	0.5452341	0.7952
67392	4833420G17Rik	RIKEN cDNA 4833420G17 gene, transcript variant 4	NA	0.033289	6.589589	1.023342	0.54529436	0.795217
55927	Hes6	hairy and enhancer of split 6, transcript variant 1	NA	0.037819	5.703519	1.026561	0.54541225	0.795318
16765	Stmn1	stathmin 1	NA	0.028714	11.13605	1.020102	0.54563402	0.795536
11736	Ankfy1	ankyrin repeat and FYVE domain containing 1, transc	NA	0.030524	6.587776	1.021383	0.54570009	0.795536
15936	Ier2	immediate early response 2	NA	-0.08701	3.261903	-1.06217	0.54570846	0.795536
58994	Smpd3	sphingomyelin phosphodiesterase 3, neutral, transcri	NA	-0.03016	7.001241	-1.02113	0.54581265	0.795601
23996	Psmc4	proteasome (prosome, macropain) 26S subunit, ATP	NA	0.03034	6.938457	1.021252	0.54585091	0.795601
58220	Pard6b	par-6 family cell polarity regulator beta	NA	-0.05983	3.640061	-1.04234	0.54593323	0.795634
238123	Cog5	component of oligomeric golgi complex 5, transcript v	NA	0.045405	5.291347	1.031973	0.54597157	0.795634
64295	Tmub1	transmembrane and ubiquitin-like domain containing	NA	0.052973	3.849621	1.037401	0.54606061	0.795659
622665	Ccdc17	coiled-coil domain containing 17	NA	0.203708	0.121735	1.151654	0.54608669	0.795659
237107	Gnl3l	guanine nucleotide binding protein-like 3 (nucleolar)-l	NA	0.031187	7.519698	1.021853	0.54621608	0.795777
99334	Zscan29	zinc finger SCAN domains 29, transcript variant 1	NA	-0.07061	3.994154	-1.05016	0.54628619	0.795807
105245825	Gm41215	predicted gene, 41215	NA	-0.20398	-0.27365	-1.15187	0.54634134	0.795817
70885	Ints10	integrator complex subunit 10, transcript variant X6	NA	-0.03295	5.967327	-1.0231	0.54642938	0.795867
76775	Slc10a7	solute carrier family 10 (sodium/bile acid cotransporte	NA	-0.05335	3.855453	-1.03767	0.54649852	0.795867
102209	Snapc2	small nuclear RNA activating complex, polypeptide 2	NA	0.040611	4.809986	1.028549	0.54652293	0.795867
14784	Grb2	growth factor receptor bound protein 2, transcript vari	NA	-0.02762	7.704422	-1.01933	0.54657162	0.795867
73254	Ccdc18	coiled-coil domain containing 18	NA	0.113273	2.223137	1.081679	0.54694995	0.796193
15267	H2ac18	H2A clustered histone 18	NA	0.460334	-0.59342	1.37586	0.54701538	0.796193
56258	Hnrnph2	heterogeneous nuclear ribonucleoprotein H2, transcri	NA	0.028674	7.065042	1.020074	0.54702032	0.796193
17127	Smad3	SMAD family member 3, transcript variant X2	NA	0.048697	4.834556	1.03433	0.54710989	0.796193
115490161	Gm52800	predicted gene, 52800	NA	-0.08494	2.566011	-1.06064	0.5471153	0.796193
217331	Unk	unkempt family zinc finger, transcript variant X3	NA	-0.04042	5.654606	-1.02841	0.54712842	0.796193
118567733	LOC118567733	uncharacterized LOC118567733	NA	-0.12157	1.482423	-1.08792	0.54713837	0.796193
105450	Mmrn2	multimerin 2	NA	-0.08603	2.86211	-1.06145	0.5471979	0.796209
102640515	Gm36556	predicted gene, 36556, transcript variant X3	NA	0.041586	4.837864	1.029245	0.54742704	0.796471
321006	Dcaf1	DDB1 and CUL4 associated factor 1, transcript varia	NA	-0.04212	5.601182	-1.02963	0.54759313	0.796542
56449	Ybx3	Y box protein 3, transcript variant 1	NA	-0.0385	6.779613	-1.02705	0.54759328	0.796542
217356	Tmc8	transmembrane channel-like gene family 8, transcript	NA	0.1587	0.528146	1.116281	0.54762266	0.796542
382913	Neil2	nei like 2 (E. coli), transcript variant X6	NA	0.143381	0.671645	1.10449	0.54794057	0.796789
68423	Ankrd13d	ankyrin repeat domain 13 family, member D	NA	0.045977	4.935723	1.032382	0.54795806	0.796789
69833	Polr2f	polymerase (RNA) II (DNA directed) polypeptide F	NA	-0.05669	4.888426	-1.04008	0.54795841	0.796789
14863	Gstm2	glutathione S-transferase, mu 2	NA	0.094524	1.944044	1.067713	0.54798819	0.796789
26379	Esrra	estrogen related receptor, alpha, transcript variant X3	NA	-0.05849	4.32235	-1.04138	0.54806967	0.796836
269997	Zfp747	zinc finger protein 747	NA	-0.07633	3.041591	-1.05433	0.54817026	0.796911
67073	Pi4k2b	phosphatidylinositol 4-kinase type 2 beta, transcript v	NA	-0.07252	3.12837	-1.05155	0.54827875	0.796998
118568020	LOC118568020	uncharacterized LOC118568020	NA	-0.0922	2.033921	-1.06599	0.5483678	0.797047
69536	Hemk1	HemK methyltransferase family member 1	NA	-0.05813	3.4527	-1.04112	0.54841087	0.797047
107932	Chd4	chromodomain helicase DNA binding protein 4	NA	-0.02575	8.512401	-1.01801	0.54849629	0.7971
791312	Gm9997	predicted gene 9997	NA	-0.17285	0.292862	-1.12728	0.54859667	0.797175
22625	Map3k19	mitogen-activated protein kinase kinase kinase 19	NA	0.120252	1.129172	1.086924	0.54866281	0.7972
13631	Eef2k	eukaryotic elongation factor-2 kinase, transcript varia	NA	0.038809	6.036054	1.027266	0.54876258	0.797274
78767	Efcab11	EF-hand calcium binding domain 11	NA	-0.0987	1.762372	-1.07081	0.54896409	0.797386
68473	Mob1b	MOB kinase activator 1B	NA	0.111957	2.894488	1.080694	0.54897599	0.797386
73078	Pmpcb	peptidase (mitochondrial processing) beta	NA	-0.03539	5.881352	-1.02483	0.54898664	0.797386
102640772	2810414N06Rik	RIKEN cDNA 2810414N06 gene, transcript variant 2	NA	0.201848	-0.14758	1.150171	0.54935222	0.797845
66413	Psmd6	proteasome (prosome, macropain) 26S subunit, non-	NA	0.030197	6.737096	1.021151	0.5494992	0.797904
217732	Cipc	CLOCK interacting protein, circadian, transcript varia	NA	-0.03172	6.349583	-1.02223	0.54961169	0.797904
75717	Cul5	cullin 5, transcript variant 1	NA	0.031978	6.169707	1.022413	0.54964707	0.797904
68859	Smim1	small integral membrane protein 1, transcript variant	NA	-0.09602	2.018925	-1.06882	0.54972494	0.797904
235169	Foxred1	FAD-dependent oxidoreductase domain containing 1	NA	0.03857	5.250557	1.027095	0.5497272	0.797904
619331	Zfp551	zinc fingr protein 551, transcript variant X2	NA	0.060489	3.775031	1.042819	0.5497375	0.797904
268288	Samd3	sterile alpha motif domain containing 3, transcript var	NA	0.113209	1.903096	1.081631	0.54976147	0.797904
16875	Lhx8	LIM homeobox protein 8, transcript variant X1	NA	0.080496	3.405297	1.057381	0.54978495	0.797904
14263	Fmo5	flavin containing monooxygenase 5, transcript variant	NA	0.151715	0.488262	1.110889	0.549994	0.798136
106522	Pkdcc	protein kinase domain containing, cytoplasmic, transc	NA	0.050726	4.665755	1.035786	0.55005297	0.798151
54387	Mcm3ap	minichromosome maintenance complex component 3	NA	-0.03312	6.078082	-1.02322	0.55023593	0.798345
66993	Smarcd3	SWI/SNF related, matrix associated, actin dependent	NA	-0.02975	7.082851	-1.02084	0.55040267	0.798467
105242571	Gm38745	predicted gene, 38745	NA	0.124441	1.192218	1.090086	0.55041802	0.798467
69368	Wdfy1	WD repeat and FYVE domain containing 1, transcript	NA	0.0301	6.901326	1.021083	0.55048966	0.7985

100042100	Gm3667	predicted gene 3667, transcript variant 5	NA	0.051393	4.362205	1.036265	0.55066052	0.798592
74519	Cyp2j9	cytochrome P450, family 2, subfamily j, polypeptide 9	NA	0.107705	1.882664	1.077513	0.5507135	0.798592
192163	Pcdha3	protocadherin alpha 3	NA	-0.09304	4.141145	-1.06661	0.55072981	0.798592
230979	Tnfrsf14	tumor necrosis factor receptor superfamily, member ´	NA	-0.15523	0.518387	-1.1136	0.55081035	0.798592
21810	Tgfbi	transforming growth factor, beta induced, transcript va	NA	-0.07662	3.139963	-1.05454	0.5508679	0.798592
100039901	2810407A14Rik	RIKEN cDNA 2810407A14 gene, transcript variant X	NA	0.148856	0.582382	1.10869	0.55091092	0.798592
276919	Gemin4	gem nuclear organelle associated protein 4	NA	-0.05964	3.821734	-1.0422	0.55093695	0.798592
115486904	Gm51556	predicted gene, 51556	NA	-0.15937	0.546694	-1.1168	0.55094573	0.798592
545681	Gm12992	predicted gene 12992, transcript variant 1	NA	-0.09168	2.402277	-1.06561	0.55111617	0.798767
67586	Ubxn11	UBX domain protein 11, transcript variant X3	NA	-0.06209	3.34775	-1.04398	0.55119659	0.798813
67883	Uxs1	UDP-glucuronate decarboxylase 1, transcript variant	NA	0.041508	4.722715	1.029189	0.55142892	0.799032
14670	Gnl1	guanine nucleotide binding protein-like 1	NA	0.028781	6.353299	1.02015	0.55144571	0.799032
23945	Mgll	monoglyceride lipase, transcript variant 2	NA	0.04502	4.806439	1.031697	0.55189236	0.799511
13799	En2	engrailed 2	NA	0.059289	4.339176	1.041952	0.55189272	0.799511
108168326	Gm46583	predicted gene, 46583	NA	-0.09359	3.131945	-1.06702	0.55197182	0.799511
69185	Dtwd1	DTW domain containing 1, transcript variant 2	NA	0.0718	3.062571	1.051027	0.55197332	0.799511
54376	Cacng3	calcium channel, voltage-dependent, gamma subunit	NA	-0.0554	4.014771	-1.03915	0.55206619	0.799527
53319	Nxf1	nuclear RNA export factor 1, transcript variant 1	NA	-0.032	7.208868	-1.02243	0.55210591	0.799527
21854	Timm17a	translocase of inner mitochondrial membrane 17a, tra	NA	0.048279	4.805936	1.034031	0.55213186	0.799527
18779	Pla2r1	phospholipase A2 receptor 1, transcript variant X1	NA	0.18919	0.150984	1.140123	0.55220611	0.799536
56690	Mlycd	malonyl-CoA decarboxylase, transcript variant X1	NA	0.057632	3.892258	1.040756	0.55227582	0.799536
213119	Itga10	integrin, alpha 10	NA	-0.07616	2.729321	-1.05421	0.55228494	0.799536
56520	Nme4	NME/NM23 nucleoside diphosphate kinase 4	NA	0.078087	2.407474	1.055617	0.5524574	0.799585
12929	Crkl	v-crk avian sarcoma virus CT10 oncogene homolog-l	NA	0.028008	6.691044	1.019603	0.55255478	0.799585
59036	Dact1	dishevelled-binding antagonist of beta-catenin 1, tran	NA	0.030807	6.603826	1.021584	0.55255677	0.799585
115488335	Gm52131	predicted gene, 52131	NA	0.224907	-0.02111	1.168702	0.55256404	0.799585
226652	Arhgap30	Rho GTPase activating protein 30	NA	-0.21928	0.165635	-1.16415	0.55257486	0.799585
66102	Cxcl16	chemokine (C-X-C motif) ligand 16, transcript variant	NA	0.141998	0.797751	1.103432	0.55271276	0.799585
78394	Ddx52	DExD box helicase 52	NA	0.040635	4.989909	1.028567	0.5527399	0.799585
54473	Tollip	toll interacting protein, transcript variant 1	NA	0.030989	6.695021	1.021712	0.55277001	0.799585
217410	Trib2	tribbles pseudokinase 2, transcript variant X2	NA	0.029693	7.702239	1.020795	0.55280471	0.799585
269623	Rbm48	RNA binding motif protein 48	NA	-0.06959	3.54047	-1.04942	0.55281027	0.799585
15213	Hey1	hairy/enhancer-of-split related with YRPW motif 1	NA	-0.04529	4.515557	-1.03189	0.55311599	0.799956
115490136	Gm15860	predicted gene 15860, transcript variant 1	NA	0.109181	1.724878	1.078616	0.55336904	0.800197
76408	Abcc3	ATP-binding cassette, sub-family C (CFTR/MRP), me	NA	0.169212	0.355436	1.124444	0.55343716	0.800197
50760	Fbxo17	F-box protein 17, transcript variant X1	NA	0.115175	1.754232	1.083106	0.55346514	0.800197
66681	Pgm2	phosphoglucomutase 2, transcript variant 1	NA	0.047909	4.716864	1.033765	0.55348413	0.800197
68106	Nt5c3b	5'-nucleotidase, cytosolic IIIB, transcript variant 3	NA	-0.03925	5.716741	-1.02758	0.55352827	0.800197
14151	Fech	ferrochelatase, transcript variant 1	NA	0.048411	5.489894	1.034126	0.55369164	0.80033
56200	Ddx21	DExD box helicase 21	NA	0.032827	6.380975	1.023015	0.55375063	0.80033
14824	Grn	granulin	NA	-0.03938	5.618463	-1.02767	0.55382074	0.80033
13650	Rhbdf1	rhomboid 5 homolog 1, transcript variant 1	NA	0.051567	4.160571	1.03639	0.55384013	0.80033
56392	Shoc2	Shoc2, leucine rich repeat scaffold protein, transcript	NA	0.031054	6.624733	1.021759	0.55386668	0.80033
105246895	Gm11772	predicted gene 11772, transcript variant X2	NA	0.185582	-0.00944	1.137276	0.55410014	0.800596
229317	Eif2a	eukaryotic translation initiation factor 2A	NA	0.035853	5.930052	1.025163	0.55417397	0.800632
102639188	Gm27008	predicted gene, 27008, transcript variant X4	NA	-0.18072	0.247164	-1.13345	0.55435538	0.800757
24084	Tekt2	tektin 2, transcript variant 1	NA	-0.07081	2.839429	-1.0503	0.55435893	0.800757
19038	Ppic	peptidylprolyl isomerase C	NA	0.050998	4.945606	1.035981	0.55465589	0.801115
12161	Bmp6	bone morphogenetic protein 6	NA	0.05759	3.835558	1.040726	0.55470893	0.80112
115486889	Gm16183	predicted gene 16183, transcript variant X1	NA	0.184746	0.091715	1.136617	0.5547995	0.801125
101739	Psip1	PC4 and SFRS1 interacting protein 1, transcript varia	NA	0.02694	8.438738	1.018849	0.55485676	0.801125
102636078	Gm33250	predicted gene, 33250, transcript variant X4	NA	-0.16547	0.136062	-1.12153	0.55486009	0.801125
231605	Galnt9	polypeptide N-acetylgalactosaminyltransferase 9, trar	NA	0.038845	4.836779	1.027291	0.5549387	0.801143
18585	Pde9a	phosphodiesterase 9A, transcript variant X6	NA	0.040987	5.391697	1.028817	0.55499424	0.801143
66916	Ndufb7	NADH:ubiquinone oxidoreductase subunit B7	NA	0.050377	5.283035	1.035535	0.55503167	0.801143
105244829	Gm40365	predicted gene, 40365, transcript variant X1	NA	0.284632	3.233279	1.218099	0.5550689	0.801143
77987	Ascc3	activating signal cointegrator 1 complex subunit 3, tra	NA	-0.04535	4.92838	-1.03194	0.55524075	0.80132
26363	Btd	biotinidase, transcript variant 2	NA	-0.05133	3.890395	-1.03622	0.55546143	0.801473
69706	Lrr1	leucine rich repeat protein 1, transcript variant 1	NA	-0.17565	1.151415	-1.12947	0.55546443	0.801473
68832	Ldah	lipid droplet associated hydrolase, transcript variant 4	NA	-0.03846	5.380928	-1.02702	0.55549504	0.801473
269338	Vps39	VPS39 HOPS complex subunit, transcript variant 2	NA	-0.03857	6.336552	-1.0271	0.5556507	0.801627
75062	Sf3a3	splicing factor 3a, subunit 3	NA	0.034548	5.985635	1.024236	0.555997	0.802013
100609	Nsun5	NOL1/NOP2/Sun domain family, member 5, transcrip	NA	-0.04823	4.210963	-1.03399	0.556033	0.802013
102636164	Gm14502	predicted gene 14502, transcript variant X1	NA	0.107257	2.006837	1.077178	0.55606651	0.802013
100041231	Gm3219	predicted pseudogene 3219	NA	0.088683	3.047488	1.063399	0.55637403	0.802337
18803	Plcg1	phospholipase C, gamma 1	NA	-0.0381	6.492599	-1.02676	0.55648437	0.802337
319922	Vwc2	von Willebrand factor C domain containing 2, transcri	NA	0.098792	2.871668	1.070876	0.55648573	0.802337
102638959	Gm35394	predicted gene, 35394, transcript variant X4	NA	-0.12619	1.2942	-1.09141	0.55648784	0.802337

14590	Ggh	gamma-glutamyl hydrolase	NA	0.0506	4.223572	1.035696	0.55666163	0.802416
383619	Aim2	absent in melanoma 2	NA	0.092647	2.31726	1.066325	0.55667095	0.802416
118568762	LOC118568762	uncharacterized LOC118568762	NA	-0.15436	1.218144	-1.11293	0.55669093	0.802416
192164	Pcdha12	protocadherin alpha 12	NA	0.083195	4.479132	1.059362	0.55704746	0.802788
93704	Pcdhgb7	protocadherin gamma subfamily B, 7	NA	-0.04556	4.735312	-1.03208	0.55704765	0.802788
14149	Fdxr	ferredoxin reductase	NA	-0.05818	3.811368	-1.04115	0.55715167	0.802819
74002	Psd2	pleckstrin and Sec7 domain containing 2, transcript v	NA	-0.03101	7.039017	-1.02173	0.55716753	0.802819
105247200	Gm42339	predicted gene, 42339	NA	-0.12468	5.392128	-1.09027	0.55726362	0.802886
18451	P4ha1	procollagen-proline, 2-oxoglutarate 4-dioxygenase (p	NA	-0.06782	5.350528	-1.04813	0.55733211	0.802914
216021	Stox1	storkhead box 1, transcript variant 1	NA	-0.10751	2.066522	-1.07737	0.55766748	0.803274
64085	Clstn2	calsyntenin 2, transcript variant X2	NA	-0.03873	6.802891	-1.02721	0.55779734	0.803274
74241	Chpf	chondroitin polymerizing factor, transcript variant 1	NA	-0.04488	5.237346	-1.0316	0.55783325	0.803274
12267	C3ar1	complement component 3a receptor 1	NA	-0.11958	1.286894	-1.08642	0.55787618	0.803274
27681	Snf8	SNF8, ESCRT-II complex subunit, homolog (S. cerev	NA	-0.05548	5.459276	-1.03921	0.55791177	0.803274
13003	Vcan	versican, transcript variant 4	NA	-0.02851	7.488746	-1.01996	0.5579524	0.803274
19339	Rab3a	RAB3A, member RAS oncogene family, transcript va	NA	0.026186	7.759921	1.018317	0.55796315	0.803274
54612	Sfrp5	secreted frizzled-related sequence protein 5	NA	0.155721	0.794177	1.113978	0.55797718	0.803274
65019	Rpl23	ribosomal protein L23	NA	-0.0347	8.860253	-1.02434	0.55803481	0.803286
102641244	Gm27239	predicted gene 27239	NA	0.211936	-0.24925	1.158242	0.55817675	0.803419
70693	Adgra3	adhesion G protein-coupled receptor A3	NA	-0.03464	6.209124	-1.0243	0.55834186	0.803586
116731	Pcdha1	protocadherin alpha 1	NA	-0.11997	2.027963	-1.08671	0.55844217	0.803604
53375	Mtx2	metaxin 2	NA	0.034662	6.068823	1.024317	0.558536	0.803604
66273	Aamdc	adipogenesis associated Mth938 domain containing,	NA	0.05661	3.559336	1.040019	0.55854812	0.803604
20498	Slc12a4	solute carrier family 12, member 4, transcript variant :	NA	-0.05193	3.788652	-1.03665	0.55857137	0.803604
271842	Rpusd2	RNA pseudouridylate synthase domain containing 2	NA	-0.06605	3.315682	-1.04685	0.55860158	0.803604
14598	Ggt1	gamma-glutamyltransferase 1, transcript variant X2	NA	0.14446	0.753711	1.105317	0.5588431	0.803869
66488	Fam136a	family with sequence similarity 136, member A, trans	NA	-0.03806	5.400038	-1.02673	0.55888471	0.803869
99045	Mrps26	mitochondrial ribosomal protein S26	NA	0.049265	4.617784	1.034737	0.55893709	0.803873
20439	Siah2	siah E3 ubiquitin protein ligase 2	NA	0.064385	4.232236	1.045639	0.55908595	0.804016
50912	Exosc10	exosome component 10, transcript variant 1	NA	-0.03975	6.281116	-1.02793	0.5591582	0.804049
100861615	Gm3411	predicted gene 3411	NA	-0.17303	2.740635	-1.12742	0.55930793	0.804194
67801	Pllp	plasma membrane proteolipid	NA	0.189951	-0.00806	1.140725	0.5594951	0.804337
399591	Tmsb15l	thymosin beta 15b like	NA	-0.05671	3.432	-1.04009	0.55951155	0.804337
209776	Gpr139	G protein-coupled receptor 139	NA	0.055539	3.894189	1.039247	0.55955579	0.804337
14011	Etv6	ets variant 6, transcript variant X3	NA	0.045102	4.714531	1.031756	0.55969617	0.80444
18654	Pgf	placental growth factor, transcript variant 2	NA	0.092657	2.00102	1.066332	0.55979618	0.80444
209039	Tns2	tensin 2, transcript variant X5	NA	-0.05619	3.968074	-1.03972	0.55980043	0.80444
19240	Tmsb10	thymosin, beta 10, transcript variant 1	NA	0.031493	11.01108	1.022069	0.55982524	0.80444
16493	Kcna5	potassium voltage-gated channel, shaker-related sub	NA	0.112018	1.984561	1.080739	0.55992426	0.804511
66494	Prelid1	PRELI domain containing 1	NA	0.032461	7.091688	1.022755	0.55999723	0.804545
15571	Elavl3	ELAV like RNA binding protein 3	NA	-0.02507	9.522783	-1.01753	0.56004995	0.80455
72568	Lin9	lin-9 homolog (C. elegans), transcript variant X2	NA	-0.05563	3.866685	-1.03931	0.56013412	0.804599
238680	Cntnap3	contactin associated protein-like 3	NA	-0.10483	1.885472	-1.07537	0.56032248	0.804799
24018	Rngtt	RNA guanylyltransferase and 5'-phosphatase, transc	NA	-0.03715	5.306623	-1.02609	0.560478	0.804911
68205	Urm1	ubiquitin related modifier 1	NA	0.043048	4.805677	1.030288	0.56049975	0.804911
210719	Mkx	mohawk homeobox	NA	0.138798	1.17072	1.100988	0.56066983	0.805085
52335	Atxn1l	ataxin 1-like, transcript variant 3	NA	0.048161	5.055978	1.033946	0.56084259	0.805191
192656	Ripk2	receptor (TNFRSF)-interacting serine-threonine kinas	NA	-0.08538	2.52259	-1.06097	0.56084305	0.805191
68097	Dynll2	dynein light chain LC8-type 2, transcript variant 1	NA	-0.03007	8.970438	-1.02106	0.56089636	0.805197
19259	Ptpn5	protein tyrosine phosphatase, non-receptor type 5, tra	NA	0.035672	7.420848	1.025034	0.56117375	0.805463
66142	Cox7b	cytochrome c oxidase subunit 7B	NA	0.029119	6.487935	1.020389	0.56118074	0.805463
66398	Commd5	COMM domain containing 5	NA	0.067984	3.33269	1.048251	0.56123296	0.805467
242939	Cpz	carboxypeptidase Z	NA	-0.16292	0.659927	-1.11955	0.56133816	0.805513
66078	Tsen34	tRNA splicing endonuclease subunit 34, transcript va	NA	-0.02765	6.386728	-1.01935	0.56136427	0.805513
59010	Sqor	sulfide quinone oxidoreductase, transcript variant 3	NA	0.12757	0.84966	1.092452	0.56161352	0.8058
67121	Mastl	microtubule associated serine/threonine kinase-like, t	NA	-0.07357	2.691514	-1.05231	0.56168476	0.805831
215201	Trmt2b	TRM2 tRNA methyltransferase 2B, transcript variant :	NA	0.089201	2.889067	1.063781	0.56182231	0.805957
103213	Traf3ip2	TRAF3 interacting protein 2, transcript variant X1	NA	0.210318	0.046448	1.156943	0.56191001	0.806012
104445	Cdc42ep1	CDC42 effector protein (Rho GTPase binding) 1	NA	0.06068	3.605644	1.042957	0.56198059	0.806042
227960	Gca	grancalcin	NA	0.073816	2.811098	1.052497	0.56207314	0.806049
74098	Czib	CXXC motif containing zinc binding protein, transcript	NA	-0.04794	4.730521	-1.03379	0.56211566	0.806049
73405	1700055D18Rik	RIKEN cDNA 1700055D18 gene	NA	-0.20217	-0.12208	-1.15043	0.5621771	0.806049
239099	Homez	homeodomain leucine zipper-encoding gene, transcri	NA	-0.05018	4.017733	-1.03539	0.56224094	0.806049
14866	Gstm5	glutathione S-transferase, mu 5	NA	0.037431	5.689828	1.026284	0.56228085	0.806049
234407	Colgalt1	collagen beta(1-O)galactosyltransferase 1, transcript	NA	-0.03408	6.505903	-1.02391	0.56240561	0.806049
238505	Mtr	5-methyltetrahydrofolate-homocysteine methyltransfe	NA	0.074953	3.200633	1.053327	0.56248119	0.806049
66241	Tmem9	transmembrane protein 9, transcript variant 2	NA	0.036753	5.534191	1.025802	0.56256159	0.806049
16854	Lgals3	lectin, galactose binding, soluble 3, transcript variant	NA	-0.22037	1.048058	-1.16504	0.56258861	0.806049

72265	Tram1	translocating chain-associating membrane protein 1	NA	0.033112	5.588742	1.023217	0.56258867	0.806049
18074	Nid2	nidogen 2	NA	0.032724	5.787032	1.022942	0.56259624	0.806049
74530	9030612E09Rik	RIKEN cDNA 9030612E09 gene	NA	-0.11996	1.133698	-1.0867	0.56261279	0.806049
17775	Laptm4a	lysosomal-associated protein transmembrane 4A	NA	-0.02929	7.994964	-1.02051	0.56271129	0.806049
218100	Zfp322a	zinc finger protein 322A, transcript variant 2	NA	-0.04271	5.775573	-1.03005	0.56274921	0.806049
76484	Kndc1	kinase non-catalytic C-lobe domain (KIND) containinç	NA	0.036393	5.941184	1.025547	0.56278075	0.806049
74018	Als2	alsin Rho guanine nucleotide exchange factor, transc	NA	0.034477	5.728373	1.024185	0.56286193	0.806049
68968	Cdan1	congenital dyserythropoietic anemia, type I (human)	NA	-0.04257	4.799953	-1.02995	0.56286339	0.806049
380773	Slirp	SRA stem-loop interacting RNA binding protein	NA	0.049102	4.384179	1.034621	0.56290204	0.806049
170745	Xpnpep2	X-prolyl aminopeptidase (aminopeptidase P) 2, meml	NA	0.151672	0.309624	1.110856	0.56292656	0.806049
140483	Hnmt	histamine N-methyltransferase	NA	0.081849	2.367138	1.058374	0.56298579	0.806063
208936	Adamts18	a disintegrin-like and metallopeptidase (reprolysin typ	NA	-0.07465	4.076082	-1.05311	0.56304604	0.806078
22094	Tshb	thyroid stimulating hormone, beta subunit, transcript \	NA	-0.4978	-0.98268	-1.41206	0.5631515	0.806131
21676	Tead1	TEA domain family member 1, transcript variant 3	NA	-0.03092	6.268791	-1.02166	0.56319782	0.806131
69654	Dctn2	dynactin 2, transcript variant 1	NA	0.027829	7.758926	1.019477	0.56325701	0.806131
18263	Odc1	ornithine decarboxylase, structural 1	NA	-0.03015	7.691575	-1.02112	0.56328122	0.806131
26385	Grk6	G protein-coupled receptor kinase 6, transcript varian	NA	0.032413	6.30772	1.022721	0.5633834	0.8062
76500	Ip6k2	inositol hexaphosphate kinase 2, transcript variant 3	NA	-0.03219	7.192418	-1.02256	0.56346119	0.8062
17165	Mapkapk5	MAP kinase-activated protein kinase 5	NA	0.036233	5.503822	1.025433	0.56353726	0.8062
16500	Kcnb1	potassium voltage gated channel, Shab-related subfa	NA	0.035943	5.336392	1.025227	0.56354773	0.8062
213649	Arhgef19	Rho guanine nucleotide exchange factor (GEF) 19, tr	NA	-0.11241	1.667183	-1.08103	0.56357715	0.8062
217342	Ube2o	ubiquitin-conjugating enzyme E2O, transcript variant	NA	-0.03875	6.995602	-1.02722	0.56367631	0.806212
52397	Zfp644	zinc finger protein 644, transcript variant X43	NA	0.044524	6.212224	1.031343	0.5636844	0.806212
140917	Dclre1b	DNA cross-link repair 1B, transcript variant 2	NA	0.048909	3.857246	1.034482	0.56385404	0.806353
102032	Smim19	small integral membrane protein 19, transcript varian	NA	0.050831	4.094682	1.035862	0.56390637	0.806353
13667	Eif2b4	eukaryotic translation initiation factor 2B, subunit 4 de	NA	0.038655	5.22978	1.027156	0.5639338	0.806353
13603	Opn3	opsin 3, transcript variant X1	NA	0.090776	2.214255	1.064943	0.56398131	0.806353
329002	Zfp236	zinc finger protein 236, transcript variant X5	NA	-0.03393	5.470523	-1.0238	0.56413716	0.806498
67460	Decr1	2,4-dienoyl CoA reductase 1, mitochondrial	NA	-0.05316	3.827428	-1.03754	0.56418216	0.806498
110006	Gusb	glucuronidase, beta, transcript variant 1	NA	-0.05204	4.352288	-1.03673	0.56440022	0.806739
17256	Mea1	male enhanced antigen 1, transcript variant 6	NA	0.039073	5.970744	1.027453	0.56452391	0.80676
233532	Rsf1	remodeling and spacing factor 1	NA	0.029302	6.556123	1.020519	0.56453649	0.80676
54615	Npff	neuropeptide FF-amide peptide precursor	NA	0.163295	0.031945	1.119842	0.56456374	0.80676
15122	Hba-a1	hemoglobin alpha, adult chain 1	NA	-0.12768	10.79121	-1.09253	0.56471949	0.806912
224019	Tmem191c	transmembrane protein 191C	NA	0.068447	3.333246	1.048587	0.56491007	0.807114
68119	Cmtm3	CKLF-like MARVEL transmembrane domain containi	NA	-0.04394	4.517416	-1.03093	0.56502128	0.807202
74315	Rnf145	ring finger protein 145, transcript variant 3	NA	0.026432	7.284933	1.01849	0.56514812	0.807312
100038739	Gm10615	predicted gene 10615	NA	-0.09325	2.08534	-1.06677	0.5652396	0.807341
78449	2700046A07Rik	RIKEN cDNA 2700046A07 gene	NA	0.170189	0.283821	1.125206	0.56526804	0.807341
69732	2410018L13Rik	RIKEN cDNA 2410018L13 gene	NA	0.075721	3.196707	1.053888	0.56537862	0.807389
19357	Rad21	RAD21 cohesin complex component, transcript variai	NA	0.02486	7.969402	1.017381	0.56543375	0.807389
57257	Vav3	vav 3 oncogene, transcript variant 2	NA	0.051293	4.435235	1.036193	0.56545011	0.807389
19224	Ptgs1	prostaglandin-endoperoxide synthase 1	NA	0.121429	1.898235	1.087812	0.56551115	0.807405
70335	Reep6	receptor accessory protein 6, transcript variant 1	NA	0.081745	2.674976	1.058297	0.56560034	0.807438
436022	Dnaaf3	dynein, axonemal assembly factor 3	NA	-0.1151	1.699607	-1.08305	0.56563357	0.807438
15572	Elavl4	ELAV like RNA binding protein 4, transcript variant X	NA	0.029288	7.938205	1.020508	0.56579927	0.807528
76899	Golga1	golgi autoantigen, golgin subfamily a, 1, transcript vai	NA	0.039922	5.312062	1.028059	0.56585498	0.807528
28015	Polr2m	polymerase (RNA) II (DNA directed) polypeptide M, tı	NA	0.024832	7.837897	1.017361	0.56588586	0.807528
102639677	Gm35932	predicted gene, 35932, transcript variant 2	NA	-0.12751	1.09178	-1.09241	0.56591412	0.807528
217057	Ptrh2	peptidyl-tRNA hydrolase 2, transcript variant 1	NA	0.042066	5.148266	1.029587	0.56594473	0.807528
331374	Dgkk	diacylglycerol kinase kappa	NA	-0.06088	3.871278	-1.0431	0.56600153	0.807539
100038526	Gm16008	predicted gene 16008, transcript variant 1	NA	0.069964	3.197157	1.049691	0.56624025	0.807761
214305	Hhipl1	hedgehog interacting protein-like 1	NA	-0.18939	0.350085	-1.14028	0.56625665	0.807761
74164	Nfx1	nuclear transcription factor, X-box binding 1, transcrip	NA	0.027637	6.865869	1.019341	0.56630611	0.807761
68618	Eola1	endothelium and lymphocyte associated ASCH doma	NA	0.047709	4.003678	1.033622	0.56635659	0.807762
16372	Irx2	Iroquois homeobox 2	NA	0.057566	4.975247	1.040709	0.56654918	0.807897
53374	Chst3	carbohydrate sulfotransferase 3	NA	-0.05892	3.367911	-1.04169	0.56659093	0.807897
268822	Adck5	aarF domain containing kinase 5, transcript variant 1	NA	-0.05921	3.97292	-1.0419	0.56663046	0.807897
73916	Ift57	intraflagellar transport 57	NA	-0.03946	4.902954	-1.02773	0.56664968	0.807897
22062	Trp73	transformation related protein 73, transcript variant 3	NA	-0.0739	3.40026	-1.05256	0.56671047	0.807897
106298	Rrn3	RRN3 RNA polymerase I transcription factor homoloç	NA	-0.02789	6.726404	-1.01952	0.56674947	0.807897
64930	Tsc1	TSC complex subunit 1, transcript variant 2	NA	0.032389	6.456683	1.022704	0.56700787	0.80817
102640100	Gm36251	predicted gene, 36251, transcript variant X7	NA	0.157895	0.127083	1.115658	0.56703995	0.80817
101565	Ccp110	centriolar coiled coil protein 110, transcript variant 3	NA	-0.03505	6.037714	-1.02459	0.56712629	0.808222
14538	Gcnt2	glucosaminyl (N-acetyl) transferase 2, I-branching en	NA	0.059965	3.440056	1.042441	0.56729452	0.808391
387524	Znrf2	zinc and ring finger 2	NA	0.056701	5.837535	1.040085	0.56744987	0.808542
73340	Nptxr	neuronal pentraxin receptor	NA	-0.17327	5.797514	-1.12761	0.5675216	0.808573
243372	Zfp775	zinc finger protein 775	NA	-0.05054	3.86367	-1.03566	0.56798051	0.809156

78653	Bola3	bolA-like 3 (E. coli), transcript variant X2	NA	-0.07889	2.95686	-1.05621	0.56813348	0.809273
240843	Brinp2	bone morphogenic protein/retinoic acid inducible neu	NA	0.035363	6.065417	1.024814	0.56827875	0.809273
329251	Ppp1r12b	protein phosphatase 1, regulatory subunit 12B, trans	NA	-0.0331	6.152997	-1.02321	0.56828552	0.809273
17300	Foxc1	forkhead box C1	NA	0.065193	4.274786	1.046225	0.5683358	0.809273
216760	Mfap3	microfibrillar-associated protein 3, transcript variant X	NA	-0.03185	6.321135	-1.02232	0.56836789	0.809273
666584	BC024063	cDNA sequence BC024063, transcript variant 2	NA	0.092838	2.745809	1.066466	0.56845198	0.809273
629777	Zfp335os	zinc finger protein 335, opposite strand	NA	-0.10232	1.837773	-1.0735	0.56846321	0.809273
54722	Gsdme	gasdermin E	NA	-0.05184	4.093177	-1.03658	0.5685071	0.809273
67405	Nts	neurotensin	NA	0.053568	3.91116	1.037829	0.5685106	0.809273
13340	Slc29a2	solute carrier family 29 (nucleoside transporters), me	NA	0.054505	3.975881	1.038503	0.56884389	0.809572
19656	Rbmxl1	RNA binding motif protein, X-linked like-1, transcript v	NA	-0.02977	7.123158	-1.02085	0.56888104	0.809572
70617	Fam241a	family with sequence similarity 241, member A	NA	0.067128	3.291083	1.047629	0.56890341	0.809572
107227	Macrod1	mono-ADP ribosylhydrolase 1, transcript variant 2	NA	-0.14659	1.696492	-1.10695	0.56896168	0.809572
68874	Klhdc9	kelch domain containing 9	NA	0.173051	0.567475	1.12744	0.56903905	0.809572
101240	Wdr91	WD repeat domain 91	NA	-0.04706	4.404107	-1.03316	0.56909934	0.809572
20168	Rtn3	reticulon 3, transcript variant X1	NA	0.024981	9.530482	1.017466	0.56911419	0.809572
227545	Proser2	proline and serine rich 2, transcript variant 2	NA	0.106877	1.968955	1.076895	0.56913354	0.809572
26961	Rpl8	ribosomal protein L8	NA	0.031206	8.959494	1.021866	0.56920403	0.809572
83704	Slc12a9	solute carrier family 12 (potassium/chloride transport	NA	-0.04757	5.036611	-1.03352	0.56922938	0.809572
53881	Slc5a3	solute carrier family 5 (inositol transporters), member	NA	-0.03684	5.671217	-1.02586	0.56931769	0.809572
18567	Pdcd2	programmed cell death 2	NA	0.062903	4.177155	1.044565	0.56941654	0.809572
52808	Tspyl2	TSPY-like 2, transcript variant 1	NA	-0.03257	6.237993	-1.02284	0.56941894	0.809572
18207	Nthl1	nth (endonuclease III)-like 1 (E.coli), transcript varian	NA	0.090606	2.068631	1.064817	0.56942641	0.809572
67501	Ccdc50	coiled-coil domain containing 50, transcript variant X1	NA	0.029356	7.155976	1.020556	0.56946642	0.809572
142682	Zcchc14	zinc finger, CCHC domain containing 14	NA	-0.02476	7.576946	-1.01731	0.56980425	0.809981
17293	Mesp2	mesoderm posterior 2	NA	-0.16096	0.034273	-1.11803	0.56988536	0.810025
381218	4430402I18Rik	RIKEN cDNA 4430402I18 gene, transcript variant 5	NA	0.127067	0.974408	1.092071	0.56996432	0.810025
77619	Prelid2	PRELI domain containing 2	NA	0.162814	0.213086	1.119469	0.56998449	0.810025
11727	Ang	angiogenin, ribonuclease, RNase A family, 5, transcri	NA	0.180813	-0.02644	1.133522	0.57004087	0.810034
108168478	Gm14853	predicted gene 14853	NA	0.134066	0.954998	1.097382	0.57012258	0.81004
66757	Adat2	adenosine deaminase, tRNA-specific 2	NA	-0.10364	1.634935	-1.07448	0.5701789	0.81004
21881	Tkt	transketolase	NA	-0.02747	7.971149	-1.01922	0.57019406	0.81004
22157	Tulp1	tubby like protein 1, transcript variant X1	NA	-0.2102	-0.43585	-1.15685	0.57030742	0.810047
15567	Slc6a4	solute carrier family 6 (neurotransmitter transporter, s	NA	0.076343	2.826801	1.054342	0.57033068	0.810047
118568763	LOC118568763	uncharacterized LOC118568763	NA	0.172396	0.329011	1.126928	0.5703485	0.810047
238323	Rps6kl1	ribosomal protein S6 kinase-like 1, transcript variant 2	NA	0.041656	4.645082	1.029294	0.57041753	0.810075
67903	Gipc1	GIPC PDZ domain containing family, member 1	NA	-0.03175	5.877948	-1.02225	0.57053277	0.810167
330256	Gm20559	predicted gene, 20559	NA	-0.09987	1.872239	-1.07168	0.57083518	0.810432
72630	Hspa12b	heat shock protein 12B	NA	-0.06884	3.26177	-1.04888	0.57085348	0.810432
14159	Fes	feline sarcoma oncogene	NA	-0.11819	1.814108	-1.08537	0.5708795	0.810432
118568205	LOC118568205	uncharacterized LOC118568205	NA	0.145561	0.39083	1.106161	0.57098888	0.810432
104725	Sptssa	serine palmitoyltransferase, small subunit A	NA	0.038426	5.9503	1.026993	0.57107087	0.810432
70387	Ttc9c	tetratricopeptide repeat domain 9C, transcript variant	NA	0.029182	6.010041	1.020433	0.57110684	0.810432
102636167	Gm33310	predicted gene, 33310	NA	0.185169	-0.20103	1.13695	0.57111745	0.810432
67091	Trappc6a	trafficking protein particle complex 6A, transcript varia	NA	-0.11318	1.924025	-1.08161	0.57111754	0.810432
666329	Gm3317	predicted gene 3317	NA	-0.19374	3.850172	-1.14372	0.57143729	0.810727
104252	Cdc42ep2	CDC42 effector protein (Rho GTPase binding) 2	NA	0.056786	4.02285	1.040146	0.57147474	0.810727
72727	B3gat3	beta-1,3-glucuronyltransferase 3 (glucuronosyltransfe	NA	0.031866	5.938939	1.022333	0.5714749	0.810727
226525	Rasal2	RAS protein activator like 2, transcript variant X8	NA	-0.02806	6.321977	-1.01964	0.57155047	0.810764
58998	Nectin3	nectin cell adhesion molecule 3, transcript variant X9	NA	0.03792	5.744296	1.026633	0.57163874	0.810818
18627	Per2	period circadian clock 2, transcript variant X3	NA	0.061451	3.937247	1.043515	0.57172553	0.810871
59016	Thap11	THAP domain containing 11	NA	-0.0441	4.914008	-1.03104	0.5719642	0.811136
19188	Psme2	proteasome (prosome, macropain) activator subunit 2	NA	-0.0397	5.157244	-1.0279	0.57206223	0.811136
115487458	Gm51806	predicted gene, 51806	NA	-0.05144	3.917056	-1.0363	0.57210518	0.811136
66999	Med28	mediator complex subunit 28	NA	0.037346	5.630851	1.026224	0.57211196	0.811136
102632794	Gm30774	predicted gene, 30774	NA	0.196292	-0.18284	1.145749	0.57219587	0.811184
72330	Klhl40	kelch-like 40	NA	0.168552	0.479166	1.12393	0.5722773	0.811193
18046	Nfyc	nuclear transcription factor-Y gamma, transcript varia	NA	0.035805	5.946936	1.025129	0.57230189	0.811193
118568683	LOC118568683	MLV-related proviral Env polyprotein-like, transcript v	NA	0.06742	4.52068	1.047841	0.57244984	0.811332
114332	Lyve1	lymphatic vessel endothelial hyaluronan receptor 1	NA	0.124334	2.937609	1.090005	0.57252117	0.811363
67281	Rpl37	ribosomal protein L37	NA	0.036999	6.919306	1.025978	0.57271068	0.811527
99003	Qser1	glutamine and serine rich 1	NA	-0.02917	6.420349	-1.02043	0.57273712	0.811527
20515	Slc20a1	solute carrier family 20, member 1, transcript variant 3	NA	-0.03645	6.648409	-1.02559	0.57281757	0.811571
22365	Vps45	vacuolar protein sorting 45	NA	-0.03497	5.283027	-1.02453	0.57308866	0.811855
664903	Rps15a-ps4	ribosomal protein S15A, pseudogene 4	NA	0.044784	4.085482	1.031529	0.57313655	0.811855
27414	Sergef	secretion regulating guanine nucleotide exchange fac	NA	-0.03871	4.707462	-1.0272	0.57327055	0.811855
66114	Dnajc30	DnaJ heat shock protein family (Hsp40) member C30	NA	0.052215	4.280581	1.036855	0.57327236	0.811855
72459	Htatsf1	HIV TAT specific factor 1, transcript variant 2	NA	0.03185	6.660578	1.022322	0.57331649	0.811855

20238	Atxn1	ataxin 1, transcript variant 2	NA	0.033695	5.631096	1.023631	0.57331782	0.811855
66525	Timm50	translocase of inner mitochondrial membrane 50	NA	-0.03542	5.049832	-1.02486	0.57339932	0.8119
66104	Tceal6	transcription elongation factor A (SII)-like 6	NA	0.043627	4.634204	1.030702	0.57375822	0.812337
330788	Zfp866	zinc finger protein 866	NA	-0.03833	5.867667	-1.02692	0.57386965	0.812424
67138	Herc6	hect domain and RLD 6	NA	0.138571	1.000613	1.100814	0.57398792	0.812521
66815	Mcub	mitochondrial calcium uniporter dominant negative be	NA	-0.11261	1.319709	-1.08118	0.57411069	0.812624
75597	Ndufaf2	NADH:ubiquinone oxidoreductase complex assembly	NA	0.052974	3.913905	1.037401	0.57417907	0.812627
16330	Inpp5b	inositol polyphosphate-5-phosphatase B, transcript va	NA	-0.03749	5.03576	-1.02633	0.57421271	0.812627
225283	Rprd1a	regulation of nuclear pre-mRNA domain containing 1/	NA	-0.03394	6.15392	-1.02381	0.5744555	0.8129
434246	Trim72	tripartite motif-containing 72	NA	-0.19881	0.73814	-1.14775	0.57469142	0.813108
21333	Tac1	tachykinin 1, transcript variant 2	NA	0.044356	4.287089	1.031223	0.57485292	0.813108
12830	Col4a5	collagen, type IV, alpha 5, transcript variant 2	NA	0.038512	4.707221	1.027054	0.57487733	0.813108
233332	Adamts17	a disintegrin-like and metallopeptidase (reprolysin typ	NA	0.057517	3.332112	1.040673	0.57492116	0.813108
66877	Crnkl1	crooked neck pre-mRNA splicing factor 1	NA	-0.03691	5.110967	-1.02592	0.5749222	0.813108
75691	Anks6	ankyrin repeat and sterile alpha motif domain contain	NA	-0.05629	3.872161	-1.03979	0.57494652	0.813108
100859931	Gm20604	predicted gene 20604	NA	-0.10672	5.300935	-1.07678	0.5749523	0.813108
100101919	Dnah7c	dynein, axonemal, heavy chain 7C, transcript variant	NA	-0.16952	0.345184	-1.12469	0.57502009	0.813118
118567927	LOC118567927	uncharacterized LOC118567927	NA	-0.17238	0.446695	-1.12692	0.57505938	0.813118
210530	P3h2	prolyl 3-hydroxylase 2	NA	-0.10528	1.578626	-1.0757	0.57518506	0.813166
12034	Phb2	prohibitin 2	NA	0.035029	6.838322	1.024578	0.57519326	0.813166
17128	Smad4	SMAD family member 4, transcript variant 1	NA	0.027845	7.209874	1.019488	0.57533214	0.813223
102098	Arhgef18	rho/rac guanine nucleotide exchange factor (GEF) 18	NA	0.036103	5.453753	1.025341	0.57534055	0.813223
16416	Itgb3	integrin beta 3	NA	-0.1209	1.531213	-1.08742	0.57538297	0.813223
217578	Baz1a	bromodomain adjacent to zinc finger domain 1A, tran	NA	-0.06653	3.87775	-1.04719	0.57557457	0.813395
13680	Ddx19a	DEAD box helicase 19a	NA	-0.02874	6.03599	-1.02012	0.57569069	0.813395
12390	Cav2	caveolin 2, transcript variant 1	NA	0.062987	3.718673	1.044627	0.57570054	0.813395
105244940	Gm40462	predicted gene, 40462	NA	-0.09588	1.680306	-1.06871	0.57573772	0.813395
63955	Cables1	CDK5 and Abl enzyme substrate 1, transcript variant	NA	0.054701	3.536736	1.038644	0.57580122	0.813395
27984	Efhd2	EF hand domain containing 2	NA	-0.03091	5.674701	-1.02166	0.57583007	0.813395
52118	Pvr	poliovirus receptor	NA	0.060626	3.351047	1.042918	0.57591962	0.813395
241062	Pgap1	post-GPI attachment to proteins 1	NA	0.034158	6.829249	1.023959	0.57592902	0.813395
56480	Tbk1	TANK-binding kinase 1	NA	0.036149	5.171927	1.025373	0.57595517	0.813395
19782	Rmrp	RNA component of mitochondrial RNAase P	NA	0.193696	1.534008	1.14369	0.5760345	0.813437
54169	Kat6b	K(lysine) acetyltransferase 6B, transcript variant X14	NA	-0.03777	6.792062	-1.02653	0.57610782	0.81347
434693	Mrto4-ps2	mRNA turnover 4, pseudogene 2	NA	-0.21968	-0.20097	-1.16448	0.57616471	0.81348
66700	Chmp3	charged multivesicular body protein 3, transcript varia	NA	-0.03365	6.051877	-1.0236	0.5762673	0.813554
102640413	Gm36478	predicted gene, 36478	NA	0.186913	0.101799	1.138326	0.57632436	0.813564
102640312	Gm36411	predicted gene, 36411, transcript variant X5	NA	0.129643	1.104836	1.094023	0.57664565	0.813882
18194	Nsdhl	NAD(P) dependent steroid dehydrogenase-like	NA	0.032291	5.77915	1.022635	0.57664992	0.813882
231148	Ablim2	actin-binding LIM protein 2, transcript variant 5	NA	0.048795	4.189965	1.0344	0.57683094	0.814067
101314	Brk1	BRICK1, SCAR/WAVE actin-nucleating complex subu	NA	0.030862	6.5492	1.021622	0.57726252	0.814555
114584	Clic1	chloride intracellular channel 1	NA	-0.03797	5.331129	-1.02667	0.57732593	0.814555
60315	Myg1	melanocyte proliferating gene 1	NA	-0.03853	4.764794	-1.02707	0.57735949	0.814555
751865	Sap25	sin3 associated polypeptide	NA	-0.25029	0.861196	-1.18944	0.57740539	0.814555
242274	Lrrc7	leucine rich repeat containing 7, transcript variant 4	NA	0.027597	6.768966	1.019313	0.57742733	0.814555
100504056	Gm20036	predicted gene, 20036	NA	0.119654	0.885294	1.086474	0.57783479	0.81506
218210	Nup153	nucleoporin 153, transcript variant X2	NA	-0.03133	6.363675	-1.02196	0.57804631	0.815246
68845	Pih1d1	PIH1 domain containing 1, transcript variant 3	NA	-0.03988	5.24428	-1.02802	0.57812949	0.815246
270110	Irf2bp2	interferon regulatory factor 2 binding protein 2	NA	-0.0298	6.206825	-1.02087	0.57816999	0.815246
105675	Ppif	peptidylprolyl isomerase F (cyclophilin F)	NA	0.04046	4.709642	1.028441	0.57820786	0.815246
22388	Wdr1	WD repeat domain 1	NA	-0.02822	7.365128	-1.01975	0.57827707	0.815246
22247	Umps	uridine monophosphate synthetase, transcript variant	NA	0.04322	4.898202	1.030411	0.57831712	0.815246
214931	Fbxl16	F-box and leucine-rich repeat protein 16, transcript va	NA	-0.02799	6.67822	-1.01959	0.57832214	0.815246
16452	Jak2	Janus kinase 2, transcript variant X4	NA	0.028917	6.411676	1.020246	0.57836784	0.815246
16588	Kin	Kin17 DNA and RNA binding protein	NA	-0.04112	4.482823	-1.02892	0.57850242	0.815365
20354	Sema4d	sema domain, immunoglobulin domain (Ig), transmem	NA	-0.03845	5.236581	-1.02701	0.57862017	0.81544
12512	Cd63	CD63 antigen, transcript variant 1	NA	0.039548	5.634403	1.027792	0.57868623	0.81544
229055	Zbtb10	zinc finger and BTB domain containing 10	NA	-0.04069	5.318118	-1.0286	0.57874385	0.81544
207259	Zbtb7c	zinc finger and BTB domain containing 7C, transcript	NA	0.053384	3.94202	1.037696	0.57875598	0.81544
68817	Ddi2	DNA-damage inducible protein 2	NA	0.073535	3.452004	1.052292	0.57886744	0.815526
67838	Dnajb11	DnaJ heat shock protein family (Hsp40) member B11	NA	0.029359	6.159095	1.020559	0.5789724	0.815604
67934	1700124L16Rik	RIKEN cDNA 1700124L16 gene	NA	-0.18277	0.188544	-1.13506	0.5791242	0.815735
66315	Senp7	SUMO1/sentrin specific peptidase 7, transcript varian	NA	0.032463	5.978444	1.022757	0.57916619	0.815735
224224	Impg2	interphotoreceptor matrix proteoglycan 2, transcript v	NA	0.080165	3.155576	1.057139	0.57947567	0.816091
74476	4933439C10Rik	RIKEN cDNA 4933439C10 gene, transcript variant 1	NA	0.041805	4.992069	1.029401	0.57958185	0.816091
30843	Fbxl12	F-box and leucine-rich repeat protein 12, transcript va	NA	-0.05489	4.046917	-1.03878	0.57958994	0.816091
66090	Ypel3	yippee like 3, transcript variant 2	NA	0.042052	5.578713	1.029577	0.5796195	0.816091
118567660	LOC118567660	uncharacterized LOC118567660	NA	0.057756	3.149527	1.040846	0.580057	0.816637

71446	Get1	guided entry of tail-anchored proteins factor 1	NA		-0.02758	6.438479	-1.0193	0.58011299	0.816645
242721	Klhdc7a	kelch domain containing 7A	NA		-0.1221	1.478502	-1.08832	0.58024335	0.816758
67811	Poldip2	polymerase (DNA-directed), delta interacting protein :	NA		0.030504	6.59177	1.021369	0.580383	0.816884
235627	Nbeal2	neurobeachin-like 2	NA		0.06303	3.607273	1.044657	0.58056599	0.816965
231440	Parm1	prostate androgen-regulated mucin-like protein 1	NA		0.099644	1.776629	1.071509	0.58058587	0.816965
13014	Cstb	cystatin B	NA		0.05338	4.156427	1.037693	0.58059156	0.816965
13185	Vps26c	VPS26 endosomal protein sorting factor C	NA		-0.03029	5.657734	-1.02122	0.58068406	0.817017
71458	Bcor	BCL6 interacting corepressor, transcript variant X10	NA		0.03481	6.00594	1.024422	0.58074642	0.817017
17769	Mthfr	methylenetetrahydrofolate reductase, transcript varia	NA		-0.04176	4.937207	-1.02937	0.58078172	0.817017
66165	Bccip	BRCA2 and CDKN1A interacting protein	NA		0.036754	5.541109	1.025803	0.58082954	0.817017
214469	Fam168b	family with sequence similarity 168, member B, trans	NA		0.024035	8.810643	1.016799	0.58093602	0.817097
381410	Zfp408	zinc finger protein 408, transcript variant 2	NA		0.042238	5.004808	1.02971	0.58118329	0.817374
108168421	Gm46660	predicted gene, 46660	NA		-0.11373	1.525241	-1.08202	0.58125351	0.817402
110075	Bmp3	bone morphogenetic protein 3, transcript variant 2	NA		0.072646	2.840999	1.051644	0.58136357	0.817425
26893	Cops6	COP9 signalosome subunit 6	NA		0.029662	6.404183	1.020773	0.58137071	0.817425
68031	Rnf146	ring finger protein 146, transcript variant 5	NA		0.029496	6.40876	1.020655	0.58143155	0.81744
241303	Fam78a	family with sequence similarity 78, member A, transc	NA		0.129271	1.455688	1.093741	0.58148638	0.817447
117109	Pop5	processing of precursor 5, ribonuclease P/MRP famil	NA		-0.06279	3.396758	-1.04448	0.58187356	0.81792
56542	Cilk1	ciliogenesis associated kinase 1, transcript variant X	NA		0.027872	6.887529	1.019507	0.58234638	0.818514
108168109	Gm46436	predicted gene, 46436	NA		0.179295	0.224406	1.13233	0.58247513	0.818625
12476	Cd151	CD151 antigen, transcript variant 2	NA		-0.04513	4.847439	-1.03178	0.58258828	0.818647
223626	Them6	thioesterase superfamily member 6	NA		0.093492	1.74298	1.06695	0.58259554	0.818647
170574	Sp7	Sp7 transcription factor 7, transcript variant 1	NA		0.211537	0.420443	1.157921	0.5827184	0.818647
115487114	Gm51642	predicted gene, 51642	NA		0.27957	0.024991	1.213833	0.5827418	0.818647
224617	Tbc1d24	TBC1 domain family, member 24, transcript variant 3	NA		0.025478	6.996791	1.017817	0.58274253	0.818647
67238	Fam220a	family with sequence similarity 220, member A, trans	NA		0.029938	6.272059	1.020968	0.58291055	0.818812
102637642	Gm34402	predicted gene, 34402	NA		0.243775	-0.69745	1.184087	0.58306338	0.818956
230696	AU022252	expressed sequence AU022252, transcript variant X	NA		-0.0538	5.017282	-1.038	0.58330631	0.819227
69479	1700029J07Rik	RIKEN cDNA 1700029J07 gene, transcript variant 1	NA		-0.10098	2.764254	-1.0725	0.58354849	0.819496
112415	Zfp607b	zinc finger protein 607B, transcript variant X6	NA		0.042461	4.264296	1.029869	0.58384179	0.819837
72355	Cdpf1	cysteine rich, DPF motif domain containing 1, transcr	NA		-0.0787	2.438683	-1.05606	0.5841868	0.820251
102634735	Gm32247	predicted gene, 32247, transcript variant X1	NA		-0.13588	0.8706	-1.09876	0.58431052	0.820354
108167755	Gm46178	predicted gene, 46178, transcript variant X2	NA		0.251646	4.127529	1.190565	0.5844251	0.820385
66964	Golt1b	golgi transport 1B	NA		-0.03975	5.652523	-1.02794	0.58443325	0.820385
64660	Mrps24	mitochondrial ribosomal protein S24, transcript variar	NA		0.044793	4.438569	1.031535	0.58459401	0.820539
240869	Zbtb37	zinc finger and BTB domain containing 37, transcript	NA		0.034577	5.722281	1.024256	0.58469339	0.820608
66320	Tmem208	transmembrane protein 208, transcript variant 3	NA		0.047614	4.317214	1.033554	0.58491264	0.820788
17304	Mfge8	milk fat globule-EGF factor 8 protein, transcript variar	NA		0.03713	6.431811	1.02607	0.58492272	0.820788
277978	Exoc3l	exocyst complex component 3-like, transcript variant	NA		0.084227	1.983621	1.06012	0.58500623	0.820835
66617	Ntmt1	N-terminal Xaa-Pro-Lys N-methyltransferase 1, trans	NA		0.041892	4.948935	1.029463	0.58506663	0.820849
57320	Park7	Parkinson disease (autosomal recessive, early onset	NA		0.03049	6.622239	1.021359	0.58519453	0.820957
67072	Cdc37l1	cell division cycle 37-like 1, transcript variant X2	NA		-0.02609	6.618816	-1.01825	0.58543929	0.821188
245643	Frmpd3	FERM and PDZ domain containing 3, transcript varia	NA		0.054268	4.158236	1.038332	0.58545946	0.821188
245828	Trappc1	trafficking protein particle complex 1	NA		-0.03825	5.379281	-1.02687	0.58559014	0.8213
623230	Tmem200b	transmembrane protein 200B, transcript variant X1	NA		-0.1776	0.171774	-1.131	0.5857171	0.821407
66874	Ncbp3	nuclear cap binding subunit 3	NA		0.032941	5.400399	1.023096	0.58595561	0.821644
71914	Antxr2	anthrax toxin receptor 2	NA		-0.07374	2.854815	-1.05244	0.58598694	0.821644
56032	Nprl2	NPR2 like, GATOR1 complex subunit	NA		-0.03655	4.91219	-1.02566	0.586154	0.821808
12748	Clk2	CDC-like kinase 2, transcript variant 1	NA		-0.04247	5.67429	-1.02987	0.58657453	0.822326
102638851	Gm17224	predicted gene 17224, transcript variant X1	NA		-0.16701	0.357689	-1.12273	0.58671434	0.822348
69606	Mtfmt	mitochondrial methionyl-tRNA formyltransferase	NA		-0.05673	3.848203	-1.04011	0.58673777	0.822348
244416	Ppp1r3b	protein phosphatase 1, regulatory subunit 3B, transcr	NA		-0.07181	3.03803	-1.05104	0.58674149	0.822348
320299	Iqcb1	IQ calmodulin-binding motif containing 1	NA		0.031475	5.404388	1.022056	0.58680005	0.822359
72832	Crtac1	cartilage acidic protein 1, transcript variant 1	NA		0.053205	4.370495	1.037567	0.58697292	0.82253
16980	Lrrn2	leucine rich repeat protein 2, neuronal	NA		-0.02873	6.194262	-1.02011	0.58726539	0.822719
115488020	Gm52009	predicted gene, 52009	NA		-0.14878	0.406475	-1.10863	0.5873615	0.822719
70873	Cnbd2	cyclic nucleotide binding domain containing 2, transc	NA		-0.07791	2.537641	-1.05549	0.58739705	0.822719
353155	Gjd3	gap junction protein, delta 3	NA		-0.15698	0.026402	-1.11495	0.58740099	0.822719
229672	Bcl2l15	BCLl2-like 15, transcript variant X1	NA		0.164581	0.328035	1.12084	0.58742189	0.822719
239250	Slitrk6	SLIT and NTRK-like family, member 6	NA		0.092688	2.701735	1.066355	0.58748654	0.822719
16949	Loxl1	lysyl oxidase-like 1	NA		0.056564	3.856616	1.039986	0.58750192	0.822719
100169874	Gm11110	predicted gene 11110	NA		0.119204	1.068233	1.086136	0.5875416	0.822719
245884	Fam71f2	family with sequence similarity 71, member F2, trans	NA		0.20856	0.290819	1.155535	0.58756234	0.822719
109050	Inka2	inka box actin regulator 2, transcript variant 1	NA		-0.05121	4.259748	-1.03614	0.58765899	0.822783
171095	Il17rc	interleukin 17 receptor C, transcript variant 2	NA		-0.14532	0.429347	-1.10597	0.58779368	0.822841
654318	C530005A16Rik	RIKEN cDNA C530005A16 gene	NA		0.083856	1.982217	1.059847	0.58785164	0.822841
18155	Pnoc	prepronociceptin, transcript variant X2	NA		0.05111	3.647611	1.036062	0.58789214	0.822841
26358	Aldh1a7	aldehyde dehydrogenase family 1, subfamily A7	NA		0.140888	0.753028	1.102584	0.5879027	0.822841

72792	2810459M11Rik	RIKEN cDNA 2810459M11 gene, transcript variant 1	NA	0.053494	3.665429	1.037775	0.58799389	0.822898
27756	Lsm2	LSM2 homolog, U6 small nuclear RNA and mRNA de	NA	-0.06202	4.652414	-1.04393	0.58811017	0.822948
276905	Armc7	armadillo repeat containing 7, transcript variant 1	NA	-0.05536	3.657821	-1.03912	0.5881727	0.822948
231887	Pdap1	PDGFA associated protein 1, transcript variant 1	NA	0.024584	7.098424	1.017187	0.58819479	0.822948
239510	Phf20l1	PHD finger protein 20-like 1, transcript variant X18	NA	0.029887	6.903761	1.020932	0.58823147	0.822948
16155	Il10rb	interleukin 10 receptor, beta	NA	0.060467	2.939089	1.042803	0.58837679	0.82304
235345	Hoatz	HOATZ cilia and flagella associated protein, transcrip	NA	-0.18308	-0.06007	-1.1353	0.58843486	0.82304
71591	Zfp251	zinc finger protein 251	NA	0.029617	5.653267	1.020741	0.58852002	0.82304
64339	Fndc4	fibronectin type III domain containing 4, transcript var	NA	0.033983	6.29014	1.023835	0.58856664	0.82304
237403	Lingo3	leucine rich repeat and Ig domain containing 3, transc	NA	-0.06096	3.439017	-1.04316	0.58858504	0.82304
229096	Ythdf3	YTH N6-methyladenosine RNA binding protein 3, tran	NA	0.035263	6.793891	1.024744	0.58862781	0.82304
118568094	LOC118568094	translation initiation factor IF-2-like	NA	-0.06934	2.99262	-1.04923	0.58865945	0.82304
407819	BC031181	cDNA sequence BC031181, transcript variant 1	NA	0.033316	6.291122	1.023362	0.58871339	0.82304
407800	Ecm2	extracellular matrix protein 2, female organ and adipo	NA	0.093981	2.259683	1.067311	0.58875299	0.82304
72083	Mzt2	mitotic spindle organizing protein 2, transcript variant	NA	0.063048	3.989429	1.04467	0.58890922	0.823094
13653	Egr1	early growth response 1	NA	0.081679	2.670525	1.058249	0.58891679	0.823094
75563	Dnali1	dynein, axonemal, light intermediate polypeptide 1	NA	0.139428	0.704109	1.101469	0.58894337	0.823094
21841	Tia1	cytotoxic granule-associated RNA binding protein 1, t	NA	-0.02742	7.890917	-1.01919	0.589137	0.823294
105242688	Gm38837	predicted gene, 38837	NA	-0.10829	2.484128	-1.07795	0.58925037	0.823358
15115	Hars	histidyl-tRNA synthetase	NA	0.026101	6.546216	1.018256	0.58932007	0.823358
67937	Tmem59l	transmembrane protein 59-like, transcript variant 1	NA	0.039579	5.148803	1.027814	0.58933434	0.823358
72139	2610044O15Rik8	RIKEN cDNA 2610044O15 gene	NA	0.064471	3.496654	1.045702	0.5894805	0.823491
223726	Mpped1	metallophosphoesterase domain containing 1, transc	NA	-0.03887	8.018978	-1.02731	0.58953595	0.823498
230657	Tmem69	transmembrane protein 69	NA	-0.05775	4.180512	-1.04084	0.58963711	0.823569
12465	Cct5	chaperonin containing Tcp1, subunit 5 (epsilon), trans	NA	0.023477	8.377468	1.016406	0.58995065	0.823869
106869	Tnfaip8	tumor necrosis factor, alpha-induced protein 8, transc	NA	0.069745	3.476785	1.049531	0.58996938	0.823869
20397	Sgpl1	sphingosine phosphate lyase 1, transcript variant 3	NA	0.027865	6.295661	1.019503	0.5901007	0.823869
227094	Nemp2	nuclear envelope integral membrane protein 2, transc	NA	-0.05123	4.058885	-1.03615	0.59013679	0.823869
118568625	LOC118568625	uncharacterized LOC118568625	NA	-0.13143	1.167497	-1.09538	0.59015643	0.823869
72057	Phf10	PHD finger protein 10, transcript variant 2	NA	-0.03559	6.255539	-1.02497	0.59019195	0.823869
72433	Rab38	RAB38, member RAS oncogene family	NA	-0.19214	-0.33625	-1.14246	0.59026567	0.823869
209003	Rbmx2	RNA binding motif protein, X-linked 2	NA	0.057116	3.965558	1.040384	0.59028558	0.823869
17025	Alad	aminolevulinate, delta-, dehydratase, transcript variar	NA	0.058836	4.617586	1.041625	0.59034244	0.823869
14404	Gabre	gamma-aminobutyric acid (GABA) A receptor, subuni	NA	-0.12897	0.721793	-1.09352	0.59039132	0.823869
225791	Zadh2	zinc binding alcohol dehydrogenase, domain containi	NA	0.038094	4.476024	1.026756	0.59040902	0.823869
58230	Rnf8	ring finger protein 8	NA	0.036688	5.096233	1.025756	0.59052126	0.823955
12409	Cbr2	carbonyl reductase 2	NA	-0.26078	0.687811	-1.19812	0.59075299	0.824208
51798	Ech1	enoyl coenzyme A hydratase 1, peroxisomal	NA	0.035237	4.843806	1.024725	0.5911665	0.824643
207565	Camkk2	calcium/calmodulin-dependent protein kinase kinase	NA	-0.02823	6.618755	-1.01976	0.59126209	0.824643
16904	Gzmm	granzyme M (lymphocyte met-ase 1), transcript variar	NA	-0.1494	0.465978	-1.10911	0.59127604	0.824643
13854	Epn1	epsin 1, transcript variant X21	NA	0.02994	7.694919	1.02097	0.5912885	0.824643
194952	Jmjd4	jumonji domain containing 4, transcript variant X1	NA	-0.04149	5.515929	-1.02918	0.59134172	0.824643
103511	Calhm5	calcium homeostasis modulator family member 5	NA	-0.15052	0.271102	-1.10997	0.59136942	0.824643
17188	Maz	MYC-associated zinc finger protein (purine-binding tr	NA	-0.02344	8.487354	-1.01638	0.59143326	0.824662
27385	Magel2	MAGE family member L2	NA	-0.03772	5.946419	-1.02649	0.59159106	0.824764
15446	Hpgd	hydroxyprostaglandin dehydrogenase 15 (NAD)	NA	-0.09045	1.977555	-1.0647	0.59160832	0.824764
66576	Uqcrh	ubiquinol-cytochrome c reductase hinge protein	NA	0.03209	6.914308	1.022492	0.59192991	0.825093
68939	Rasl11b	RAS-like, family 11, member B	NA	-0.05476	3.732115	-1.03868	0.5919884	0.825093
68436	Rpl34	ribosomal protein L34, transcript variant 2	NA	0.033847	7.366546	1.023738	0.59201953	0.825093
20130	Rras	related RAS viral (r-ras) oncogene, transcript variant	NA	0.075106	2.500205	1.053439	0.5920467	0.825093
67459	Nvl	nuclear VCP-like	NA	0.025462	6.455562	1.017805	0.59218031	0.825199
102642832	Phf2os1	PHD finger protein 2, opposite strand 1, transcript var	NA	0.081152	3.374398	1.057863	0.59235406	0.825199
12803	Cntf	ciliary neurotrophic factor	NA	-0.13448	0.878612	-1.0977	0.59237615	0.825199
75847	Crppa	CDP-L-ribitol pyrophosphorylase A, transcript variant	NA	0.091652	3.099227	1.065589	0.59237642	0.825199
231834	Snx8	sorting nexin 8, transcript variant X1	NA	-0.03501	4.857387	-1.02456	0.59237787	0.825199
23954	Nek3	NIMA (never in mitosis gene a)-related expressed kin	NA	-0.06556	3.131862	-1.04649	0.59242747	0.825199
105243451	Gm27188	predicted gene 27188, transcript variant X4	NA	-0.20129	-0.29839	-1.14972	0.59263003	0.825411
16491	Kcna3	potassium voltage-gated channel, shaker-related sub	NA	0.142241	0.581568	1.103618	0.59275282	0.825505
109672	Cyb5a	cytochrome b5 type A (microsomal), transcript varian	NA	0.031346	5.738619	1.021965	0.59279895	0.825505
270097	Vat1l	vesicle amine transport protein 1 like	NA	-0.02647	6.996553	-1.01852	0.59296029	0.825659
69605	Lnpk	lunapark, ER junction formation factor, transcript varia	NA	0.026223	6.331859	1.018343	0.59307344	0.825746
100041874	Gm3558	predicted gene 3558	NA	0.079242	3.190994	1.056463	0.5931366	0.825763
74569	Ttc17	tetratricopeptide repeat domain 17, transcript variant	NA	0.033052	5.369087	1.023174	0.59323503	0.825829
105245593	Gm41025	predicted gene, 41025, transcript variant X2	NA	0.147	0.256573	1.107265	0.59363434	0.826315
243277	Adgrd1	adhesion G protein-coupled receptor D1, transcript va	NA	0.159386	0.168723	1.116812	0.59390744	0.8266
105246049	Gm41408	predicted gene, 41408	NA	0.089964	1.61565	1.064344	0.59394071	0.8266
233908	Fus	fused in sarcoma, transcript variant 1	NA	-0.02412	8.881011	-1.01686	0.5939977	0.826608
70207	Taco1	translational activator of mitochondrially encoded cyto	NA	-0.06233	3.353296	-1.04415	0.59412859	0.82672

75785	Klhl24	kelch-like 24, transcript variant X2	NA		0.027259	6.998956	1.019074	0.59419117	0.826736
53945	Slc40a1	solute carrier family 40 (iron-regulated transporter), m	NA		-0.04495	4.460818	-1.03165	0.59435319	0.826812
20977	Syp	synaptophysin	NA		0.025248	8.087799	1.017655	0.59436459	0.826812
22696	Zfp37	zinc finger protein 37, transcript variant 5	NA		0.033641	5.298299	1.023592	0.59439788	0.826812
50875	Tmod3	tropomodulin 3, transcript variant 1	NA		-0.0272	6.152743	-1.01903	0.59449929	0.826864
100503949	Zfp965	zinc finger protein 965, transcript variant X6	NA		-0.0547	6.175806	-1.03865	0.59453719	0.826864
97086	Slc9b2	solute carrier family 9, subfamily B (NHA2, cation pro	NA		-0.12852	0.852219	-1.09317	0.59464782	0.826947
67302	Zc3h13	zinc finger CCCH type containing 13, transcript variar	NA		-0.02702	6.691997	-1.01891	0.59478715	0.82707
381259	Tmem237	transmembrane protein 237, transcript variant 2	NA		-0.03726	4.741914	-1.02616	0.59496471	0.827247
109801	Glo1	glyoxalase 1, transcript variant 1	NA		0.032989	6.225599	1.02313	0.5950634	0.827313
11848	Rhoa	ras homolog family member A, transcript variant 3	NA		-0.02332	7.986022	-1.01629	0.59520359	0.827437
13123	Cyp7b1	cytochrome P450, family 7, subfamily b, polypeptide	NA		-0.07805	2.034395	-1.05559	0.59528765	0.827472
69470	Tmem127	transmembrane protein 127, transcript variant 2	NA		-0.03094	7.063037	-1.02168	0.59533032	0.827472
27373	Csnk1e	casein kinase 1, epsilon, transcript variant X21	NA		-0.02488	8.391422	-1.0174	0.59538859	0.827482
71755	Dhdh	dihydrodiol dehydrogenase (dimeric), transcript variar	NA		-0.07846	2.809298	-1.05589	0.59546875	0.827523
105242978	Gm39038	predicted gene, 39038, transcript variant X1	NA		-0.16966	1.450309	-1.1248	0.5956753	0.827739
230596	Prpf38a	PRP38 pre-mRNA processing factor 38 (yeast) doma	NA		-0.02915	5.844974	-1.02041	0.59590907	0.827828
237353	Sh3rf3	SH3 domain containing ring finger 3, transcript varian	NA		0.037527	5.468792	1.026353	0.59591419	0.827828
11746	Anxa4	annexin A4, transcript variant X2	NA		-0.0788	2.183969	-1.05614	0.59600213	0.827828
329371	Dbhos	dopamine beta hydroxylase, opposite strand	NA		0.122773	0.93023	1.088826	0.59602161	0.827828
69902	Mrto4	mRNA turnover 4, ribosome maturation factor, transc	NA		-0.04148	5.099257	-1.02917	0.59602889	0.827828
18168	Npy5r	neuropeptide Y receptor Y5, transcript variant 3	NA		-0.16647	0.215256	-1.12231	0.59608342	0.827828
102920	Cenpi	centromere protein I, transcript variant X1	NA		-0.05687	3.117005	-1.04021	0.59614037	0.827828
235633	Als2cl	ALS2 C-terminal like, transcript variant X7	NA		0.09758	2.320153	1.069977	0.59614611	0.827828
67016	Tbc1d2b	TBC1 domain family, member 2B, transcript variant X	NA		-0.04191	4.614308	-1.02948	0.59622739	0.82787
16559	Kif17	kinesin family member 17, transcript variant 1	NA		0.080035	2.090955	1.057043	0.59640267	0.82792
118567481	LOC118567481	MLV-related proviral Env polyprotein-like, transcript v	NA		0.085653	2.64388	1.061168	0.59644675	0.82792
21664	Phlda1	pleckstrin homology like domain, family A, member 1	NA		0.03944	4.873917	1.027715	0.59645385	0.82792
75736	Bcl2l12	BCL2-like 12 (proline rich), transcript variant X2	NA		-0.06832	2.513852	-1.04849	0.59646695	0.82792
114893	Dcun1d1	DCN1, defective in cullin neddylation 1, domain conta	NA		0.031475	6.136139	1.022057	0.59657072	0.827948
105246577	Gm41848	predicted gene, 41848	NA		0.112786	1.471666	1.081314	0.59659148	0.827948
381792	2310040G24Rik	RIKEN cDNA 2310040G24 gene, transcript variant 1	NA		-0.15768	0.101089	-1.11549	0.59678276	0.827948
20443	St3gal4	ST3 beta-galactoside alpha-2,3-sialyltransferase 4, tr	NA		0.036125	5.424536	1.025356	0.59680696	0.827948
80905	Polh	polymerase (DNA directed), eta (RAD 30 related), tra	NA		-0.04672	3.973576	-1.03292	0.59683125	0.827948
64656	Mrps23	mitochondrial ribosomal protein S23, transcript variar	NA		-0.03831	5.156182	-1.02691	0.59683545	0.827948
244556	Zfp791	zinc finger protein 791, transcript variant X1	NA		0.084634	2.842059	1.060419	0.59684272	0.827948
227333	Dgkd	diacylglycerol kinase, delta	NA		0.02629	6.707281	1.01839	0.59693806	0.827951
67951	Tubb6	tubulin, beta 6 class V	NA		-0.07042	3.717463	-1.05002	0.59694732	0.827951
23853	Def6	differentially expressed in FDCP 6	NA		-0.09367	1.960416	-1.06708	0.59707259	0.828055
70544	Tmem242	transmembrane protein 242	NA		-0.03667	5.419332	-1.02575	0.59713871	0.828074
18769	Pkig	protein kinase inhibitor, gamma, transcript variant 3	NA		0.033319	5.017035	1.023364	0.59722687	0.828074
230484	Usp1	ubiquitin specific peptidase 1, transcript variant 3	NA		0.033371	5.876537	1.023401	0.59725245	0.828074
330166	Miat	myocardial infarction associated transcript (non-prote	NA		0.026847	9.654599	1.018783	0.59728994	0.828074
67815	Sec14l2	SEC14-like lipid binding 2	NA		0.068076	2.90687	1.048318	0.59740857	0.828168
18096	Nkx6-1	NK6 homeobox 1	NA		0.101471	1.324304	1.072867	0.59753929	0.828278
12151	Bmi1	Bmi1 polycomb ring finger oncogene, transcript variar	NA		-0.0274	6.792983	-1.01918	0.59763697	0.828302
18439	P2rx7	purinergic receptor P2X, ligand-gated ion channel, 7,	NA		0.092734	2.480289	1.066389	0.59765848	0.828302
56749	Dhodh	dihydroorotate dehydrogenase, transcript variant X1	NA		-0.04453	3.90593	-1.03135	0.59787376	0.8285
67863	Slc25a11	solute carrier family 25 (mitochondrial carrier oxogluta	NA		-0.02699	6.151563	-1.01888	0.59799428	0.8285
76742	Snx27	sorting nexin family member 27, transcript variant 1	NA		0.025026	6.931463	1.017498	0.59803857	0.8285
57314	Nelfcd	negative elongation factor complex member C/D, Th1	NA		-0.03003	5.791848	-1.02103	0.59805396	0.8285
54127	Rps28	ribosomal protein S28, transcript variant 2	NA		0.024994	7.423494	1.017476	0.59806936	0.8285
67370	Zfp606	zinc finger protein 606, transcript variant X1	NA		-0.02824	5.96132	-1.01977	0.59813647	0.8285
209318	Gps1	G protein pathway suppressor 1, transcript variant 1	NA		-0.02725	6.800129	-1.01907	0.59816918	0.8285
210009	Mtrr	5-methyltetrahydrofolate-homocysteine methyltransfe	NA		-0.06528	3.646627	-1.04629	0.59836641	0.8285
11855	Arhgap5	Rho GTPase activating protein 5, transcript variant X	NA		0.042585	6.156721	1.029958	0.59841919	0.8285
13511	Dsg2	desmoglein 2	NA		-0.13033	0.573009	-1.09455	0.59852596	0.8285
547253	Parp14	poly (ADP-ribose) polymerase family, member 14	NA		0.149354	0.12477	1.109073	0.5986038	0.8285
50496	E2f6	E2F transcription factor 6, transcript variant X2	NA		-0.04167	5.14362	-1.02931	0.59861246	0.8285
217333	Trim47	tripartite motif-containing 47, transcript variant 2	NA		0.073304	2.538092	1.052124	0.59868002	0.8285
52874	Pum3	pumilio RNA-binding family member 3, transcript vari	NA		-0.03092	5.938336	-1.02166	0.59869221	0.8285
72512	Sting1	stimulator of interferon response cGAMP interactor 1	NA		-0.14706	0.984433	-1.10731	0.5987277	0.8285
18420	Otp	orthopedia homeobox	NA		0.046755	4.815876	1.032939	0.59875133	0.8285
17210	Mcl1	myeloid cell leukemia sequence 1	NA		0.023934	7.474084	1.016728	0.59875159	0.8285
67955	Sugt1	SGT1, suppressor of G2 allele of SKP1 (S. cerevisiae	NA		0.029258	6.101228	1.020487	0.59876947	0.8285
67847	Sncaip	synuclein, alpha interacting protein (synphilin), transc	NA		0.0322	6.025993	1.02257	0.59878739	0.8285
66857	Plbd1	phospholipase B domain containing 1	NA		-0.15987	-0.11068	-1.11719	0.59881931	0.8285
77125	Il33	interleukin 33, transcript variant 2	NA		0.184997	0.546696	1.136814	0.59887422	0.828505

228731	Nkx2-4	NK2 homeobox 4, transcript variant X1	NA	0.141295	0.881719	1.102895	0.59908197	0.828691
214321	Gm4787	predicted gene 4787	NA	-0.20431	-0.00364	-1.15214	0.59911921	0.828691
67180	Yipf5	Yip1 domain family, member 5	NA	0.031949	5.896289	1.022392	0.59916085	0.828691
241116	Cfap65	cilia and flagella associated protein 65, transcript vari	NA	-0.08645	2.260968	-1.06175	0.59930949	0.828826
18216	Ntsr1	neurotensin receptor 1	NA	0.05236	4.078227	1.03696	0.59941135	0.828853
381802	Tsen2	tRNA splicing endonuclease subunit 2, transcript vari	NA	0.053529	4.233804	1.0378	0.59949492	0.828853
64817	Svep1	sushi, von Willebrand factor type A, EGF and pentrax	NA	0.070998	2.608535	1.050443	0.59952457	0.828853
22240	Dpysl3	dihydropyrimidinase-like 3, transcript variant 1	NA	0.025483	11.22985	1.01782	0.5995329	0.828853
232196	C87436	expressed sequence C87436, transcript variant 6	NA	-0.04544	4.244825	-1.032	0.59969022	0.829
22166	Txn1	thioredoxin 1	NA	-0.03021	6.621563	-1.02116	0.59982289	0.829113
118567992	LOC118567992	zinc finger protein 844-like	NA	0.053551	3.536729	1.037816	0.59998006	0.82926
211232	Cpne9	copine family member IX	NA	0.119327	1.31877	1.086228	0.6000961	0.82929
67795	Rnls	renalase, FAD-dependent amine oxidase, transcript v	NA	-0.13377	0.495583	-1.09716	0.60017577	0.82929
68020	Coa8	cytochrome c oxidase assembly factor 8, transcript va	NA	0.044241	4.289723	1.031141	0.60031915	0.82929
22631	Ywhaz	tyrosine 3-monooxygenase/tryptophan 5-monooxyge	NA	0.02364	10.22122	1.016521	0.60039739	0.82929
105833	Ccdc65	coiled-coil domain containing 65, transcript variant 1	NA	0.114536	2.053976	1.082627	0.60041064	0.82929
330192	Vps37b	vacuolar protein sorting 37B	NA	-0.03223	6.07149	-1.02259	0.60041747	0.82929
114255	Dok4	docking protein 4	NA	-0.02473	6.616423	-1.01729	0.60043295	0.82929
229900	Gbp7	guanylate binding protein 7, transcript variant 2	NA	0.097732	1.440098	1.07009	0.60045837	0.82929
231004	Samd11	sterile alpha motif domain containing 11, transcript va	NA	-0.12252	1.724804	-1.08863	0.60052861	0.82929
330721	Nek5	NIMA (never in mitosis gene a)-related expressed kin	NA	-0.11853	0.781536	-1.08563	0.60058495	0.82929
54399	Bet1l	Bet1 golgi vesicular membrane trafficking protein like	NA	0.044599	4.323364	1.031397	0.60059818	0.82929
54138	Atxn10	ataxin 10	NA	0.022615	8.482102	1.015799	0.60061348	0.82929
105246120	Gm41461	predicted gene, 41461	NA	0.088325	2.278806	1.063135	0.60066763	0.829294
106861	Abhd3	abhydrolase domain containing 3	NA	0.117751	1.120501	1.085042	0.60080852	0.829419
12315	Calm3	calmodulin 3	NA	-0.02917	9.704844	-1.02042	0.60098701	0.82953
99681	Tchh	trichohyalin	NA	0.115352	1.03296	1.083239	0.60107577	0.82953
216148	Shc2	SHC (Src homology 2 domain containing) transformin	NA	-0.03147	5.575426	-1.02205	0.60112308	0.82953
51813	Ccnc	cyclin C, transcript variant 1	NA	-0.03754	5.233861	-1.02637	0.60124464	0.82953
74470	Cep72	centrosomal protein 72	NA	-0.06412	2.943825	-1.04545	0.60127879	0.82953
226252	Fam160b1	family with sequence similarity 160, member B1	NA	0.037537	5.546851	1.02636	0.60129623	0.82953
58859	Efemp2	epidermal growth factor-containing fibulin-like extrace	NA	-0.04186	4.291189	-1.02944	0.60132885	0.82953
233046	Rasgrp4	RAS guanyl releasing protein 4, transcript variant 3	NA	-0.09621	1.562696	-1.06896	0.60135433	0.82953
227700	Sh3glb2	SH3-domain GRB2-like endophilin B2, transcript varia	NA	0.025517	6.479415	1.017845	0.60139145	0.82953
217715	Eif2b2	eukaryotic translation initiation factor 2B, subunit 2 be	NA	0.03354	5.149885	1.023521	0.60139871	0.82953
66308	Mplkip	M-phase specific PLK1 intereacting protein	NA	-0.04531	4.36762	-1.03191	0.60177315	0.829976
20204	Prrx2	paired related homeobox 2	NA	-0.14122	0.93011	-1.10284	0.60183493	0.829991
320095	6430550D23Rik	RIKEN cDNA 6430550D23 gene, transcript variant X	NA	-0.13036	0.478805	-1.09457	0.60194827	0.830076
69601	Dab2ip	disabled 2 interacting protein, transcript variant X20	NA	-0.02674	7.239132	-1.01871	0.60209884	0.830214
381356	Cacfd1	calcium channel flower domain containing 1, transcrip	NA	-0.03017	6.620606	-1.02113	0.60215971	0.830227
66072	Sdhaf2	succinate dehydrogenase complex assembly factor 2	NA	0.031216	5.211387	1.021873	0.60236006	0.830433
399558	Flrt2	fibronectin leucine rich transmembrane protein 2, trar	NA	0.038135	6.507111	1.026786	0.6024421	0.830476
28193	Reep3	receptor accessory protein 3, transcript variant X1	NA	0.044549	6.140446	1.03136	0.60255634	0.830563
320707	Atp2b3	ATPase, Ca++ transporting, plasma membrane 3, tra	NA	0.0335	5.209766	1.023492	0.60262058	0.830581
104836	Cbll1	Casitas B-lineage lymphoma-like 1, transcript variant	NA	-0.03011	5.568296	-1.02109	0.60268663	0.830602
50708	H1f2	H1.2 linker histone, cluster member	NA	-0.14154	1.096504	-1.10308	0.60274987	0.830619
330662	Dock1	dedicator of cytokinesis 1	NA	0.043185	4.623374	1.030386	0.60292684	0.830646
22017	Tpmt	thiopurine methyltransferase	NA	-0.05489	3.663067	-1.03878	0.60296599	0.830646
18843	Bpifa1	BPI fold containing family A, member 1	NA	-0.40866	-0.43887	-1.32745	0.60297148	0.830646
98970	Fibcd1	fibrinogen C domain containing 1	NA	-0.04825	4.108572	-1.03401	0.60297409	0.830646
13018	Ctcf	CCCTC-binding factor, transcript variant X6	NA	-0.026	7.086572	-1.01818	0.60304557	0.830674
52637	Cisd1	CDGSH iron sulfur domain 1	NA	0.03108	5.604245	1.021777	0.60313123	0.830722
17713	Grpel1	GrpE-like 1, mitochondrial	NA	0.031112	5.29742	1.021799	0.60318816	0.83073
328417	Parp4	poly (ADP-ribose) polymerase family, member 4, tran	NA	-0.09176	1.917657	-1.06567	0.60332273	0.830845
19935	Mrpl23	mitochondrial ribosomal protein L23, transcript varian	NA	-0.04031	4.596026	-1.02833	0.60347263	0.830981
103737	Pex12	peroxisomal biogenesis factor 12, transcript variant 1	NA	-0.04486	3.911592	-1.03159	0.60361551	0.830986
12495	Entpd1	ectonucleoside triphosphate diphosphohydrolase 1, t	NA	-0.05356	3.550271	-1.03783	0.60363596	0.830986
15248	Hic1	hypermethylated in cancer 1, transcript variant 2	NA	0.057707	3.584607	1.04081	0.60367806	0.830986
22057	Tob1	transducer of ErbB-2.1	NA	-0.04903	5.265262	-1.03457	0.60368002	0.830986
75698	Shld2	shieldin complex subunit 2, transcript variant X7	NA	0.075349	2.123931	1.053616	0.60383832	0.831133
16979	Lrrn1	leucine rich repeat protein 1, neuronal	NA	0.032188	6.424433	1.022562	0.60408426	0.831401
68036	Zfp706	zinc finger protein 706	NA	0.027927	7.7276	1.019546	0.60445444	0.831841
226413	Lct	lactase	NA	0.168506	-0.17864	1.123894	0.60451616	0.831855
226352	Epb41l5	erythrocyte membrane protein band 4.1 like 5, transc	NA	-0.03632	4.536571	-1.02549	0.6046049	0.83187
72421	Ttc30b	tetratricopeptide repeat domain 30B	NA	0.042791	4.157328	1.030105	0.60465224	0.83187
14605	Tsc22d3	TSC22 domain family, member 3, transcript variant 3	NA	-0.03105	5.589971	-1.02176	0.60468008	0.83187
104799	Vipas39	VPS33B interacting protein, apical-basolateral polarit	NA	0.028523	6.082254	1.019967	0.60484266	0.831995
100503353	Gm14440	predicted gene 14440	NA	0.074559	2.709448	1.053039	0.60490685	0.831995

212442	Lactb2	lactamase, beta 2	NA	-0.08295	2.007182	-1.05918	0.60492488	0.831995
230935	Dnajc11	DnaJ heat shock protein family (Hsp40) member C11	NA	0.027922	6.351776	1.019542	0.60502218	0.83201
319618	Dcp1b	decapping mRNA 1B, transcript variant X1	NA	0.044217	4.087336	1.031123	0.60505645	0.83201
75974	Dock11	dedicator of cytokinesis 11	NA	0.030584	5.413115	1.021426	0.60515547	0.83201
237775	Zfp867	zinc finger protein 867, transcript variant X1	NA	0.046303	4.187391	1.032615	0.60517691	0.83201
68734	Ppp4r3a	protein phosphatase 4 regulatory subunit 3A, transcri	NA	-0.03083	6.3803	-1.0216	0.60522188	0.83201
15356	Hmgcl	3-hydroxy-3-methylglutaryl-Coenzyme A lyase, transc	NA	-0.03812	4.547068	-1.02677	0.60526457	0.83201
109305	Orai1	ORAI calcium release-activated calcium modulator 1,	NA	0.057003	2.984242	1.040302	0.60537049	0.83201
20431	Pmel	premelanosome protein, transcript variant X2	NA	-0.19237	-0.34012	-1.14264	0.60541811	0.83201
14782	Gsr	glutathione reductase	NA	0.033667	5.169657	1.023611	0.60551714	0.83201
67876	Coq10b	coenzyme Q10B, transcript variant 1	NA	-0.03828	4.576167	-1.02689	0.60556042	0.83201
70571	Tcerg1l	transcription elongation regulator 1-like	NA	0.043928	5.063759	1.030917	0.60566869	0.83201
101568	Vrk3	vaccinia related kinase 3	NA	-0.04419	4.364369	-1.03111	0.60569616	0.83201
100041241	Gm3227	predicted gene 3227	NA	0.164236	-0.20788	1.120573	0.60572177	0.83201
18545	Pcp2	Purkinje cell protein 2 (L7), transcript variant 12	NA	-0.18998	-0.62628	-1.14075	0.60575968	0.83201
216987	Utp6	UTP6 small subunit processome component	NA	-0.03306	6.048331	-1.02318	0.60577814	0.83201
69002	1500026H17Rik	RIKEN cDNA 1500026H17 gene	NA	0.149663	0.689997	1.10931	0.60580103	0.83201
22643	Zfp101	zinc finger protein 101	NA	-0.04598	4.31669	-1.03239	0.60580471	0.83201
102636808	Gm38491	predicted gene, 38491	NA	-0.08272	1.819777	-1.05901	0.60593019	0.832017
264064	Cdk8	cyclin-dependent kinase 8, transcript variant 3	NA	0.028631	5.928248	1.020044	0.60597242	0.832017
26879	B3galnt1	UDP-GalNAc:betaGlcNAc beta 1,3-galactosaminyltra	NA	0.032385	5.768488	1.022701	0.60599375	0.832017
69009	Thap7	THAP domain containing 7	NA	-0.04889	3.578443	-1.03447	0.60601461	0.832017
229584	Pogz	pogo transposable element with ZNF domain, transcr	NA	-0.02366	7.072242	-1.01653	0.60612805	0.832103
67123	Ubap1	ubiquitin-associated protein 1, transcript variant 1	NA	0.029402	5.756716	1.020589	0.60629806	0.832178
75541	Nat8f4	N-acetyltransferase 8 (GCN5-related) family member	NA	0.061732	2.986274	1.043718	0.60631569	0.832178
13559	E2f5	E2F transcription factor 5	NA	0.039107	4.422288	1.027478	0.60633589	0.832178
330463	Zfp78	zinc finger protein 78, transcript variant 1	NA	0.067296	2.648482	1.047751	0.60644821	0.832262
77519	Zfp266	zinc finger protein 266, transcript variant 1	NA	0.025624	7.34638	1.01792	0.60668485	0.832474
235086	Igsf9b	immunoglobulin superfamily, member 9B, transcript v	NA	-0.02761	6.037666	-1.01932	0.60670506	0.832474
76441	Daam2	dishevelled associated activator of morphogenesis 2,	NA	-0.04254	4.733923	-1.02993	0.60679818	0.832526
225659	Cep76	centrosomal protein 76, transcript variant 1	NA	-0.05123	3.841053	-1.03615	0.6068641	0.832526
69634	Clybl	citrate lyase beta like, transcript variant X4	NA	-0.06234	3.122261	-1.04415	0.60689677	0.832526
228003	Klhl41	kelch-like 41	NA	-0.12152	0.930163	-1.08788	0.60711129	0.832729
105246454	Gm41741	predicted gene, 41741, transcript variant X3	NA	0.073178	2.20582	1.052032	0.60719375	0.832729
225888	Kmt5b	lysine methyltransferase 5B, transcript variant X14	NA	0.027431	6.743843	1.019196	0.60720627	0.832729
19181	Psmc2	proteasome (prosome, macropain) 26S subunit, ATP	NA	0.026737	6.799868	1.018706	0.60724932	0.832729
17974	Nck2	non-catalytic region of tyrosine kinase adaptor protei	NA	-0.02939	5.97295	-1.02058	0.6074163	0.832888
226562	Prrc2c	proline-rich coiled-coil 2C	NA	0.029048	7.711765	1.020338	0.60759356	0.833061
15242	Hhex	hematopoietically expressed homeobox	NA	-0.10512	1.542069	-1.07558	0.60764717	0.833064
118568241	LOC118568241	protein NYNRIN-like, transcript variant X1	NA	-0.08774	2.65731	-1.0627	0.60770896	0.833078
69736	Nup37	nucleoporin 37, transcript variant 1	NA	-0.08136	3.283623	-1.05802	0.6077665	0.833087
109093	Rars2	arginyl-tRNA synthetase 2, mitochondrial, transcript v	NA	0.037651	4.74105	1.026441	0.60783593	0.833112
75507	Pou5f2	POU domain class 5, transcription factor 2	NA	-0.13739	0.493191	-1.09992	0.60794133	0.833149
26415	Mapk13	mitogen-activated protein kinase 13	NA	-0.19524	-0.09422	-1.14492	0.60796548	0.833149
71774	Shroom1	shroom family member 1, transcript variant 1	NA	-0.09216	1.528259	-1.06596	0.60826291	0.833473
67483	1700028P14Rik	RIKEN cDNA 1700028P14 gene	NA	0.1681	-0.19365	1.123577	0.60833848	0.833473
229589	Prune1	prune exopolyphosphatase, transcript variant X1	NA	-0.02704	6.576327	-1.01892	0.6083624	0.833473
93677	Lmod2	leiomodin 2 (cardiac)	NA	-0.2266	-1.32697	-1.17008	0.60840659	0.833473
26408	Map3k5	mitogen-activated protein kinase kinase kinase 5	NA	0.062066	3.044219	1.04396	0.608527	0.833527
17864	Mybl1	myeloblastosis oncogene-like 1, transcript variant 2	NA	0.073196	2.787172	1.052045	0.60859418	0.833527
12009	Cep131	centrosomal protein 131	NA	-0.03657	5.496843	-1.02568	0.60862456	0.833527
115485859	Gm51419	predicted gene, 51419	NA	-0.09161	3.992116	-1.06556	0.60866803	0.833527
69806	Slc39a11	solute carrier family 39 (metal ion transporter), memb	NA	0.047274	4.60264	1.033311	0.60870229	0.833527
76850	Ago4	argonaute RISC catalytic subunit 4, transcript variant	NA	-0.03714	5.38877	-1.02608	0.60910547	0.833857
791413	Gm12592	predicted gene 12592	NA	0.174694	-0.08289	1.128725	0.60916993	0.833857
26888	Clec4a2	C-type lectin domain family 4, member a2, transcript	NA	-0.20035	-0.49047	-1.14898	0.60919308	0.833857
67102	D16Ertd472e	DNA segment, Chr 16, ERATO Doi 472, expressed, t	NA	-0.04745	3.695257	-1.03344	0.60925112	0.833857
18789	Papola	poly (A) polymerase alpha, transcript variant X2	NA	0.023359	7.613529	1.016323	0.609276	0.833857
102633750	Gm10130	predicted gene 10130	NA	-0.09697	1.272821	-1.06953	0.60929248	0.833857
102902673	Gm21992	predicted gene 21992, transcript variant 3	NA	0.06273	2.690835	1.04444	0.60933224	0.833857
19696	Rel	reticuloendotheliosis oncogene	NA	-0.10857	1.183402	-1.07816	0.60940354	0.833857
227334	Usp40	ubiquitin specific peptidase 40, transcript variant X13	NA	0.04169	4.8901	1.029319	0.609404	0.833857
102638783	Gm35265	predicted gene, 35265	NA	-0.15931	-0.01212	-1.11675	0.6095119	0.83388
100040531	Dynlt1f	dynein light chain Tctex-type 1F, transcript variant 1	NA	-0.0751	5.927631	-1.05344	0.60953462	0.83388
333088	Kcp	kielin/chordin-like protein, transcript variant X8	NA	-0.03985	4.236198	-1.02801	0.60961647	0.83388
232431	Gprc5a	G protein-coupled receptor, family C, group 5, memb	NA	0.189109	-0.46199	1.14006	0.60963463	0.83388
11844	Arf5	ADP-ribosylation factor 5	NA	0.033007	6.664773	1.023142	0.60969632	0.83388
50720	Sacs	sacsin, transcript variant X4	NA	0.034707	6.086499	1.024349	0.60974541	0.83388

277250	Kdm3b	KDM3B lysine (K)-specific demethylase 3B	NA		-0.0257	6.549094	-1.01797	0.60981695	0.83388
16468	Jarid2	jumonji, AT rich interactive domain 2, transcript variar	NA		0.027982	6.258983	1.019585	0.60983119	0.83388
52331	Stbd1	starch binding domain 1	NA		0.067868	2.353814	1.048167	0.61002139	0.834046
102636931	Gm33869	predicted gene, 33869, transcript variant X2	NA		0.095441	3.182005	1.068392	0.61005484	0.834046
72097	Cracdl	capping protein inhibiting regulator of actin like, trans	NA		-0.06179	2.948594	-1.04376	0.61035885	0.834333
77034	2510039O18Rik	RIKEN cDNA 2510039O18 gene	NA		-0.03378	4.968289	-1.02369	0.61038264	0.834333
22630	Ywhaq	tyrosine 3-monooxygenase/tryptophan 5-monooxyge	NA		0.024876	9.298141	1.017392	0.61041879	0.834333
102640526	Gm10353	predicted gene 10353	NA		0.097582	1.967162	1.069979	0.61075546	0.834622
105242850	Gm38946	predicted gene, 38946	NA		-0.15185	0.011572	-1.11099	0.61078259	0.834622
108167777	Gm46195	predicted gene, 46195	NA		0.148027	0.225725	1.108053	0.61078433	0.834622
102614	Rpp25	ribonuclease P/MRP 25 subunit	NA		-0.06323	3.235971	-1.04481	0.61090298	0.834714
114875	Plcz1	phospholipase C, zeta 1, transcript variant X7	NA		0.177838	-0.37431	1.131187	0.6109741	0.834741
13198	Ddit3	DNA-damage inducible transcript 3, transcript variant	NA		0.077716	3.485512	1.055346	0.61104101	0.834763
30045	Dnajc12	DnaJ heat shock protein family (Hsp40) member C12	NA		-0.06923	2.765553	-1.04916	0.61114843	0.834823
52535	Mettl17	methyltransferase like 17	NA		0.038461	4.487039	1.027018	0.61118783	0.834823
100041289	Gm3255	predicted gene 3255, transcript variant 3	NA		0.110509	2.470694	1.079609	0.61133979	0.834899
320827	Cracd	capping protein inhibiting regulator of actin, transcript	NA		-0.02228	7.725702	-1.01556	0.61134625	0.834899
244871	Zc3h12c	zinc finger CCCH type containing 12C, transcript vari	NA		0.036685	5.052663	1.025754	0.61140206	0.834901
50770	Atp11a	ATPase, class VI, type 11A, transcript variant X2	NA		-0.02626	6.212994	-1.01837	0.61145022	0.834901
620695	Gm13889	predicted gene 13889	NA		0.050397	4.047341	1.03555	0.61154196	0.83493
73332	Ccdc30	coiled-coil domain containing 30, transcript variant 1	NA		-0.05493	4.104365	-1.03881	0.61157377	0.83493
60344	Fign	fidgetin, transcript variant 1	NA		0.040813	5.095583	1.028693	0.61181613	0.835101
26885	Casp8ap2	caspase 8 associated protein 2, transcript variant 2	NA		-0.03808	4.928981	-1.02675	0.61183558	0.835101
102193	Zdhhc7	zinc finger, DHHC domain containing 7	NA		-0.04394	4.505975	-1.03093	0.61185342	0.835101
223453	Dap	death-associated protein	NA		0.041558	4.095847	1.029225	0.61210908	0.83538
102634058	Gm20208	predicted gene, 20208, transcript variant X3	NA		0.153142	0.245636	1.111989	0.61231471	0.835591
14127	Fcer1g	Fc receptor, IgE, high affinity I, gamma polypeptide	NA		-0.09164	1.637782	-1.06558	0.61253233	0.835776
104582	Rprml	reprimo-like	NA		-0.12556	0.531109	-1.09093	0.61255311	0.835776
73668	Ttc21b	tetratricopeptide repeat domain 21B, transcript varian	NA		-0.03882	4.31186	-1.02727	0.61271309	0.835924
545975	Cers3	ceramide synthase 3	NA		0.156973	-0.13385	1.114945	0.61280383	0.835956
14180	Fgf9	fibroblast growth factor 9	NA		0.049102	3.434806	1.034621	0.61283925	0.835956
320172	E230016M11Rik	RIKEN cDNA E230016M11 gene	NA		0.114105	1.42349	1.082303	0.6129661	0.836059
115489981	LOC115489981	prothymosin alpha-like, transcript variant X2	NA		-0.11742	1.166792	-1.0848	0.61307794	0.836142
22682	Zfand5	zinc finger, AN1-type domain 5, transcript variant X1	NA		0.033595	7.481064	1.023559	0.61316135	0.836153
22689	Zfp27	zinc finger protein 27, transcript variant 1	NA		0.043733	4.594671	1.030778	0.613189	0.836153
211577	Mrgprf	MAS-related GPR, member F	NA		-0.12048	1.730709	-1.0871	0.6133318	0.836246
14255	Flt3	FMS-like tyrosine kinase 3	NA		0.147368	0.459734	1.107547	0.61336021	0.836246
67487	Dhx40	DEAH (Asp-Glu-Ala-His) box polypeptide 40	NA		-0.03166	5.437644	-1.02218	0.6135194	0.836393
70261	Chp2	calcineurin-like EF hand protein 2, transcript variant X	NA		0.101595	1.530638	1.072959	0.61365142	0.836419
235315	Rnf214	ring finger protein 214, transcript variant X1	NA		-0.02931	7.352602	-1.02052	0.61366352	0.836419
65964	Map3k20	mitogen-activated protein kinase kinase kinase 20, tra	NA		0.047511	3.845413	1.03348	0.61369267	0.836419
15461	Hras	Harvey rat sarcoma virus oncogene, transcript varian	NA		0.032971	6.031065	1.023117	0.61394729	0.836696
140482	Zfp358	zinc finger protein 358	NA		-0.03077	5.749776	-1.02156	0.61399946	0.836697
19988	Rpl6	ribosomal protein L6, transcript variant X1	NA		0.023354	9.166101	1.016319	0.61425413	0.836974
78754	Galnt15	polypeptide N-acetylgalactosaminyltransferase 15, tra	NA		0.10063	1.485576	1.072241	0.61434716	0.837031
74211	1700017B05Rik	RIKEN cDNA 1700017B05 gene	NA		-0.06828	2.973411	-1.04847	0.61441749	0.837033
67388	Rab5if	RAB5 interacting factor	NA		-0.04414	5.534611	-1.03107	0.61445206	0.837033
66201	Vta1	vesicle (multivesicular body) trafficking 1	NA		0.028632	6.003927	1.020045	0.6146115	0.837181
93875	Pcdhb4	protocadherin beta 4	NA		-0.06893	2.353901	-1.04894	0.61478749	0.83735
232966	Zfp114	zinc finger protein 114	NA		-0.08568	1.586109	-1.06119	0.61489032	0.837399
244484	Wdr17	WD repeat domain 17, transcript variant 10	NA		-0.04272	4.276857	-1.03005	0.61497072	0.837399
54391	Rfk	riboflavin kinase	NA		0.028279	5.921492	1.019795	0.61502042	0.837399
105440	Kctd9	potassium channel tetramerisation domain containing	NA		-0.04662	3.804965	-1.03284	0.61502957	0.837399
101100	Ttll3	tubulin tyrosine ligase-like family, member 3, transcrip	NA		-0.06848	3.156598	-1.04861	0.61516885	0.837519
330921	Pate2	prostate and testis expressed 2	NA		-0.20075	-0.35945	-1.14929	0.61526451	0.837523
103765	Tmem17	transmembrane protein 17	NA		-0.05484	3.065788	-1.03875	0.61533375	0.837523
242523	Dmrta1	doublesex and mab-3 related transcription factor like	NA		-0.1393	0.465175	-1.10137	0.61537344	0.837523
67008	Yae1d1	Yae1 domain containing 1	NA		0.031907	5.238667	1.022363	0.6154551	0.837523
105244068	Gm39746	predicted gene, 39746	NA		-0.10852	1.07179	-1.07812	0.61545808	0.837523
228875	Slc35c2	solute carrier family 35, member C2, transcript varian	NA		-0.0443	4.967161	-1.03118	0.61554385	0.837523
67242	Gemin6	gem nuclear organelle associated protein 6, transcrip	NA		-0.06152	2.803054	-1.04356	0.61560074	0.837523
75973	Ccdc162	coiled-coil domain containing 162, transcript variant X	NA		0.139328	0.715417	1.101392	0.61563736	0.837523
70078	Nol7	nucleolar protein 7	NA		0.029049	5.732926	1.020339	0.61566249	0.837523
227738	Lrsam1	leucine rich repeat and sterile alpha motif containing	NA		0.039985	4.88762	1.028103	0.61578717	0.837523
12022	Barx1	BarH-like homeobox 1	NA		-0.22937	-1.18354	-1.17233	0.61579066	0.837523
68198	Ndufb2	NADH:ubiquinone oxidoreductase subunit B2, transc	NA		-0.03951	4.619336	-1.02777	0.61580896	0.837523
69596	Ap5s1	adaptor-related protein 5 complex, sigma 1 subunit, t	NA		0.052448	3.390098	1.037023	0.61588496	0.837523
19349	Rab7	RAB7, member RAS oncogene family, transcript varia	NA		0.021922	8.17409	1.015311	0.61589281	0.837523

69662	2310061I04Rik	RIKEN cDNA 2310061I04 gene	NA	0.031935	5.241338	1.022383	0.61594384	0.837523
78923	Chsy3	chondroitin sulfate synthase 3	NA	0.074723	2.765512	1.053159	0.6160386	0.837582
69697	Camsap3	calmodulin regulated spectrin-associated protein fam	NA	-0.02887	6.605275	-1.02022	0.61678594	0.838516
170706	Tmem37	transmembrane protein 37	NA	0.136817	0.605792	1.099477	0.61682887	0.838516
208211	Alg1	asparagine-linked glycosylation 1 (beta-1,4-mannosy	NA	0.05671	3.76281	1.040091	0.6170112	0.838617
11684	Alox12	arachidonate 12-lipoxygenase, transcript variant 1	NA	-0.18786	-0.16901	-1.13908	0.61704517	0.838617
70601	Ecd	ecdysoneless cell cycle regulator	NA	-0.03069	5.570506	-1.0215	0.61705742	0.838617
208748	Prrg3	proline rich Gla (G-carboxyglutamic acid) 3 (transmer	NA	-0.03162	5.41953	-1.02216	0.6172824	0.838732
77697	Mmab	methylmalonic aciduria (cobalamin deficiency) cblB ty	NA	0.035054	5.101837	1.024595	0.61733081	0.838732
226351	Tmem185b	transmembrane protein 185B	NA	-0.05199	3.927673	-1.0367	0.61739432	0.838732
54678	Zfp108	zinc finger protein 108, transcript variant X2	NA	0.075651	2.19782	1.053836	0.61743532	0.838732
75533	Nme5	NME/NM23 family member 5, transcript variant 1	NA	0.090207	1.892184	1.064523	0.61744212	0.838732
94216	Col4a6	collagen, type IV, alpha 6	NA	0.051409	3.557673	1.036277	0.61745135	0.838732
11906	Zfhx3	zinc finger homeobox 3, transcript variant X4	NA	0.033795	7.682311	1.023702	0.61750647	0.838737
73132	Slc25a16	solute carrier family 25 (mitochondrial carrier, Graves	NA	0.059369	4.428753	1.04201	0.61781423	0.839047
69179	Stimate	STIM activating enhancer	NA	0.046637	4.278252	1.032854	0.61783829	0.839047
433904	Ociad2	OCIA domain containing 2, transcript variant 1	NA	0.050258	4.153297	1.03545	0.61807582	0.839268
102502	Pls1	plastin 1 (I-isoform), transcript variant X2	NA	0.080842	2.114756	1.057635	0.61810371	0.839268
234258	Neil3	nei like 3 (E. coli)	NA	-0.05979	3.575667	-1.04231	0.61819306	0.839281
382118	Zkscan7	zinc finger with KRAB and SCAN domains 7, transcri	NA	-0.04895	3.766181	-1.03451	0.61829535	0.839281
14066	F3	coagulation factor III	NA	-0.06516	2.802688	-1.0462	0.61839467	0.839281
21399	Tcea1	transcription elongation factor A (SII) 1, transcript var	NA	0.027067	7.25837	1.018939	0.6184012	0.839281
209018	Vps8	VPS8 CORVET complex subunit, transcript variant 3	NA	-0.02781	5.732776	-1.01946	0.61844022	0.839281
23959	Nt5e	5' nucleotidase, ecto	NA	0.103433	1.194394	1.074327	0.61849188	0.839281
100101807	Fam177a2	family with sequence similarity 177 member A2	NA	0.037178	6.239591	1.026105	0.61851124	0.839281
414066	BC037032	cDNA Sequence BC037032	NA	0.10752	0.989611	1.077375	0.61856831	0.839281
100503068	Smim43	small integral membrane protein 43	NA	0.141435	4.56E-05	1.103002	0.61857782	0.839281
21844	Tiam1	T cell lymphoma invasion and metastasis 1, transcrip	NA	0.034479	5.811377	1.024187	0.61888048	0.839622
102637087	Gm33989	predicted gene, 33989, transcript variant 1	NA	-0.03266	5.676055	-1.0229	0.61895284	0.839647
53627	Porcn	porcupine O-acyltransferase, transcript variant X3	NA	0.032518	5.301428	1.022796	0.61901494	0.839647
30931	Tor1a	torsin family 1, member A (torsin A)	NA	0.039979	4.925153	1.028099	0.61905404	0.839647
115489522	D930003E18Rik	RIKEN cDNA D930003E18 gene	NA	0.155742	-0.22487	1.113994	0.619115	0.83966
67293	3110039M20Rik	RIKEN cDNA 3110039M20 gene	NA	-0.07834	2.947541	-1.05581	0.61924179	0.839762
217154	Stac2	SH3 and cysteine rich domain 2	NA	-0.05175	3.836187	-1.03652	0.61931368	0.839789
100038689	Gm10421	predicted gene 10421	NA	0.152641	0.161608	1.111603	0.61956159	0.840056
66462	Rex1bd	required for excision 1-B domain containing, transcrip	NA	0.055296	4.270928	1.039072	0.61965507	0.840112
329941	Col8a2	collagen, type VIII, alpha 2	NA	0.049819	3.770949	1.035135	0.61996596	0.840464
102638784	Gm35266	predicted gene, 35266	NA	0.117486	1.274613	1.084843	0.62009308	0.840483
59057	Zfp24	zinc finger protein 24, transcript variant 1	NA	0.025969	6.134925	1.018163	0.6201619	0.840483
68742	Tmem219	transmembrane protein 219, transcript variant 3	NA	0.067192	2.968532	1.047676	0.62029979	0.840483
26364	Adgre5	adhesion G protein-coupled receptor E5, transcript va	NA	0.046838	4.066749	1.032999	0.6203157	0.840483
211255	Kbtbd7	kelch repeat and BTB (POZ) domain containing 7	NA	-0.03855	5.684481	-1.02708	0.62035937	0.840483
16970	Lrmp	lymphoid-restricted membrane protein, transcript vari	NA	0.19055	-0.38184	1.141199	0.62039194	0.840483
53378	Sdcbp	syndecan binding protein, transcript variant X1	NA	0.027049	6.775514	1.018926	0.62058708	0.840483
238726	Fam81b	family with sequence similarity 81, member B, transcı	NA	-0.16532	0.223911	-1.12141	0.62060323	0.840483
67064	Chmp1b	charged multivesicular body protein 1B	NA	-0.03246	5.279601	-1.02275	0.62060951	0.840483
214552	Cep164	centrosomal protein 164	NA	-0.05148	5.082491	-1.03632	0.62070798	0.840483
74104	Abcb6	ATP-binding cassette, sub-family B (MDR/TAP), men	NA	-0.03312	4.917303	-1.02322	0.62076965	0.840483
66356	Knop1	lysine rich nucleolar protein 1, transcript variant 1	NA	-0.02515	6.438164	-1.01758	0.62077609	0.840483
433813	Pusl1	pseudouridylate synthase-like 1, transcript variant X1	NA	0.057641	3.756279	1.040762	0.62079906	0.840483
70784	Rasl12	RAS-like, family 12, transcript variant 1	NA	-0.0646	2.48472	-1.0458	0.62089097	0.840483
67130	Ndufa6	NADH:ubiquinone oxidoreductase subunit A6	NA	0.044226	5.194219	1.03113	0.62090577	0.840483
102639711	Gm35959	predicted gene, 35959	NA	0.155465	-0.12858	1.11378	0.6209255	0.840483
243906	Zfp14	zinc finger protein 14, transcript variant 3	NA	0.040227	4.167148	1.028275	0.62093434	0.840483
69038	Tmem258	transmembrane protein 258, transcript variant 2	NA	-0.03367	4.958637	-1.02361	0.62094633	0.840483
12193	Zfp36l2	zinc finger protein 36, C3H type-like 2	NA	0.042761	5.30635	1.030083	0.62099903	0.840483
74747	Ddit4	DNA-damage-inducible transcript 4	NA	0.036412	6.274557	1.02556	0.62101319	0.840483
13063	Cycs	cytochrome c, somatic	NA	0.022595	7.301392	1.015785	0.62110172	0.840532
14260	Fmn1	formin 1, transcript variant 6	NA	-0.12378	0.640158	-1.08959	0.62115317	0.840532
102640670	Gm36684	predicted gene, 36684	NA	0.180446	-0.08971	1.133235	0.62123534	0.840532
16580	Kifc5b	kinesin family member C5B	NA	0.074871	2.598554	1.053267	0.62144213	0.840532
24063	Spry1	sprouty RTK signaling antagonist 1, transcript variant	NA	0.042721	4.144929	1.030055	0.62145548	0.840532
108168201	Gm46485	predicted gene, 46485	NA	-0.07504	2.28584	-1.05339	0.62145795	0.840532
12953	Cry2	cryptochrome 2 (photolyase-like)	NA	0.036828	5.633363	1.025856	0.62147961	0.840532
279766	Rhbdd3	rhomboid domain containing 3, transcript variant 2	NA	-0.04992	3.830464	-1.03521	0.62151351	0.840532
407823	Baz2b	bromodomain adjacent to zinc finger domain, 2B, trar	NA	0.029668	6.389235	1.020777	0.62155564	0.840532
78185	Flicr	Foxp3 regulating long intergenic noncoding RNA	NA	-0.14912	-0.05203	-1.10889	0.62161035	0.840532
67145	Tomm34	translocase of outer mitochondrial membrane 34, trar	NA	0.036197	5.110077	1.025407	0.62161765	0.840532

68889	Ubac2	ubiquitin associated domain containing 2	NA	0.033303	4.885141	1.023352	0.62177489	0.840675
100040935	Gm3050	predicted gene 3050, transcript variant X6	NA	0.180555	0.05709	1.13332	0.62186704	0.84073
18476	Pafah1b3	platelet-activating factor acetylhydrolase, isoform 1b,	NA	-0.02406	6.901476	-1.01681	0.62191944	0.840731
21990	Tph1	tryptophan hydroxylase 1, transcript variant 2	NA	0.135039	0.531143	1.098123	0.62202717	0.840755
15161	Hcfc1	host cell factor C1	NA	-0.02214	7.673811	-1.01547	0.62208709	0.840755
102633551	Gm31349	predicted gene, 31349	NA	-0.09112	2.884051	-1.06519	0.62211595	0.840755
22228	Ucp2	uncoupling protein 2 (mitochondrial, proton carrier)	NA	-0.03912	4.859126	-1.02749	0.62214424	0.840755
237716	Gpr75	G protein-coupled receptor 75	NA	-0.05712	3.149192	-1.04039	0.62230984	0.840821
208449	Sgms1	sphingomyelin synthase 1, transcript variant X5	NA	0.046912	4.04341	1.033051	0.62233049	0.840821
115490443	LOC115490443	uncharacterized LOC115490443	NA	0.247671	-0.76518	1.187289	0.62234957	0.840821
215798	Adgrg6	adhesion G protein-coupled receptor G6, transcript va	NA	-0.07649	2.218391	-1.05445	0.62240001	0.840821
14678	Gnai2	guanine nucleotide binding protein (G protein), alpha	NA	0.022543	8.217023	1.015748	0.6225407	0.840942
118567529	LOC118567529	uncharacterized LOC118567529, transcript variant X	NA	-0.1232	0.740706	-1.08915	0.62270467	0.840998
67031	Upf3a	UPF3 regulator of nonsense transcripts homolog A (y	NA	0.039449	5.210194	1.027721	0.62273521	0.840998
15126	Hba-x	hemoglobin X, alpha-like embryonic chain in Hba com	NA	-0.12003	1.705004	-1.08676	0.62273721	0.840998
66904	Pccb	propionyl Coenzyme A carboxylase, beta polypeptide	NA	0.038504	5.927191	1.027048	0.6229027	0.841151
13162	Slc6a3	solute carrier family 6 (neurotransmitter transporter, c	NA	0.085533	1.988281	1.06108	0.62297027	0.841173
18938	Ppp1r14b	protein phosphatase 1, regulatory inhibitor subunit 14	NA	-0.03908	7.182325	-1.02746	0.62324302	0.841448
78779	Spata2l	spermatogenesis associated 2-like, transcript variant	NA	0.084102	1.784122	1.060028	0.62327775	0.841448
67223	Rrp15	ribosomal RNA processing 15 homolog	NA	0.055198	3.824414	1.039002	0.62341304	0.841561
239833	Lmln	leishmanolysin-like (metallopeptidase M8 family), tran	NA	0.061164	3.627498	1.043307	0.62349758	0.841587
72117	Naa50	N(alpha)-acetyltransferase 50, NatE catalytic subunit	NA	0.027776	6.879829	1.019439	0.62353602	0.841587
102633322	A730094K22Rik	RIKEN cDNA A730094K22 gene, transcript variant X	NA	-0.07245	2.356121	-1.0515	0.62359489	0.841597
100503519	Gm19744	predicted gene, 19744	NA	0.102418	1.365366	1.073572	0.623742	0.841726
17207	Mcf2l	mcf.2 transforming sequence-like, transcript variant 1	NA	-0.0237	7.839234	-1.01656	0.62390777	0.841734
20753	Sprr1a	small proline-rich protein 1A	NA	-0.27241	-0.72615	-1.20783	0.62392606	0.841734
71923	Borcs6	BLOC-1 related complex subunit 6	NA	0.050853	4.020889	1.035877	0.6239395	0.841734
74032	Sdr42e1	short chain dehydrogenase/reductase family 42E, me	NA	-0.09675	1.374659	-1.06936	0.62395512	0.841734
76773	Wdyhv1	WDYHV motif containing 1, transcript variant X2	NA	-0.05214	3.502569	-1.0368	0.62402113	0.841753
232944	Mark4	MAP/microtubule affinity regulating kinase 4, transcrip	NA	-0.02378	6.921165	-1.01662	0.62424339	0.841898
102635226	Gm32618	predicted gene, 32618	NA	0.136528	1.503367	1.099256	0.62435302	0.841898
227377	Farp2	FERM, RhoGEF and pleckstrin domain protein 2	NA	0.083194	2.451897	1.059361	0.6243642	0.841898
14234	Foxc2	forkhead box C2	NA	-0.07328	2.190727	-1.05211	0.62442594	0.841898
93893	Pcdhb22	protocadherin beta 22	NA	0.071283	2.888084	1.05065	0.62445265	0.841898
74685	Lrrc74b	leucine rich repeat containing 74B, transcript variant :	NA	-0.18393	0.088429	-1.13597	0.62446304	0.841898
76626	Msi2	musashi RNA-binding protein 2, transcript variant X2	NA	0.023636	7.736401	1.016518	0.62449036	0.841898
29810	Bag3	BCL2-associated athanogene 3	NA	-0.07076	2.752974	-1.05027	0.62456344	0.841927
68010	Bambi	BMP and activin membrane-bound inhibitor, transcrip	NA	0.063905	2.856234	1.045291	0.62470615	0.842016
18700	Piga	phosphatidylinositol glycan anchor biosynthesis, clas	NA	-0.04977	3.249668	-1.0351	0.62473323	0.842016
110842	Etfa	electron transferring flavoprotein, alpha polypeptide	NA	0.029353	5.388687	1.020554	0.62479974	0.842031
232164	Paip2b	poly(A) binding protein interacting protein 2B	NA	0.027218	6.833736	1.019045	0.62487669	0.842031
71918	Zcchc24	zinc finger, CCHC domain containing 24	NA	0.030338	5.168752	1.021252	0.62500277	0.842031
213541	Ythdf2	YTH N6-methyladenosine RNA binding protein 2	NA	0.026916	6.591185	1.018832	0.62500915	0.842031
67144	Lrrc40	leucine rich repeat containing 40, transcript variant 1	NA	0.030119	5.422532	1.021097	0.62503641	0.842031
100040724	Mirg	miRNA containing gene	NA	-0.0438	4.0915	-1.03083	0.62505508	0.842031
59092	Pcbp4	poly(rC) binding protein 4	NA	0.027791	8.028874	1.01945	0.62531389	0.84231
101437	Dhx32	DEAH (Asp-Glu-Ala-His) box polypeptide 32, transcri	NA	-0.02911	5.238404	-1.02038	0.62540478	0.842363
216961	Coro6	coronin 6, transcript variant C	NA	-0.11108	1.005227	-1.08004	0.62558896	0.842477
105837	Mtbp	Mdm2, transformed 3T3 cell double minute p53 bindi	NA	-0.06343	3.333316	-1.04494	0.6256411	0.842477
13865	Nr2f1	nuclear receptor subfamily 2, group F, member 1, tra	NA	0.025327	7.572558	1.01771	0.62564511	0.842477
228966	Ppp1r3d	protein phosphatase 1, regulatory subunit 3D	NA	-0.07637	2.596638	-1.05436	0.62571527	0.842502
53334	Gosr1	golgi SNAP receptor complex member 1, transcript va	NA	0.03226	5.5027	1.022613	0.62584345	0.842605
12919	Crhbp	corticotropin releasing hormone binding protein	NA	0.117603	0.871666	1.084931	0.6258968	0.842607
16194	Il6ra	interleukin 6 receptor, alpha, transcript variant 2	NA	0.154657	0.191148	1.113157	0.62603999	0.842665
243547	Grip2	glutamate receptor interacting protein 2, transcript va	NA	-0.03171	5.463525	-1.02222	0.62604332	0.842665
118567699	LOC118567699	uncharacterized LOC118567699	NA	-0.10056	2.164606	-1.07219	0.62623236	0.84285
72341	Elp6	elongator acetyltransferase complex subunit 6	NA	-0.04382	4.296604	-1.03084	0.6264097	0.843019
68177	Ebpl	emopamil binding protein-like	NA	-0.06008	3.1527	-1.04252	0.62656889	0.843163
74407	Ttc25	tetratricopeptide repeat domain 25, transcript variant	NA	0.108415	1.222675	1.078043	0.6268272	0.843327
83429	Ctns	cystinosis, nephropathic, transcript variant X1	NA	0.042123	4.428752	1.029628	0.62683262	0.843327
54188	Cpsf4	cleavage and polyadenylation specific factor 4, transc	NA	-0.03172	5.069417	-1.02223	0.62687774	0.843327
230866	Emc1	ER membrane protein complex subunit 1, transcript v	NA	0.027831	6.099262	1.019479	0.62689815	0.843327
30059	Timm10	translocase of inner mitochondrial membrane 10	NA	0.043251	3.927078	1.030433	0.62697983	0.843367
269823	Pon3	paraoxonase 3, transcript variant 1	NA	-0.07977	2.433102	-1.05685	0.6270745	0.843425
70918	Nsun7	NOL1/NOP2/Sun domain family, member 7, transcrip	NA	-0.05868	3.309519	-1.04151	0.62723759	0.8435
58242	Nudt11	nudix (nucleoside diphosphate linked moiety X)-type	NA	-0.02817	5.80664	-1.01972	0.6272889	0.8435
72748	Hdhd3	haloacid dehalogenase-like hydrolase domain contair	NA	-0.0873	1.413461	-1.06238	0.62734419	0.8435
20276	Scnn1a	sodium channel, nonvoltage-gated 1 alpha, transcript	NA	0.115702	0.647029	1.083502	0.62738726	0.8435

20971	Sdc4	syndecan 4	NA		0.058224	2.90833	1.041183	0.6273891	0.8435
84004	Mcam	melanoma cell adhesion molecule, transcript variant	NA		0.031496	4.88694	1.022071	0.62747441	0.843504
108168297	Gm46558	predicted gene, 46558, transcript variant X1	NA		-0.11349	0.799771	-1.08184	0.62749586	0.843504
13608	Edar	ectodysplasin-A receptor, transcript variant X4	NA		0.219093	-0.04199	1.164002	0.62770925	0.843721
102631731	Gm29998	predicted gene, 29998	NA		0.088755	1.731131	1.063452	0.62781649	0.843795
22775	Zik1	zinc finger protein interacting with K protein 1	NA		-0.03251	5.934717	-1.02279	0.62813515	0.844154
27406	Abcf3	ATP-binding cassette, sub-family F (GCN20), membe	NA		-0.02679	5.785803	-1.01874	0.62827298	0.844251
619605	Zcchc17	zinc finger, CCHC domain containing 17, transcript va	NA		0.030375	5.278247	1.021277	0.62831139	0.844251
21390	Tbxa2r	thromboxane A2 receptor, transcript variant 2	NA		0.120463	0.879116	1.087084	0.62850642	0.844328
227695	Spout1	SPOUT domain containing methyltransferase 1	NA		-0.03282	5.042061	-1.02301	0.62851185	0.844328
14231	Fkbp7	FK506 binding protein 7, transcript variant 1	NA		0.073073	2.36189	1.051955	0.628551	0.844328
12370	Casp8	caspase 8, transcript variant X2	NA		-0.07448	2.488471	-1.05298	0.62861223	0.844328
320752	Dpy19l2	dpy-19-like 2 (C. elegans), transcript variant X8	NA		-0.14791	-0.10037	-1.10796	0.62864044	0.844328
21417	Zeb1	zinc finger E-box binding homeobox 1, transcript varia	NA		0.023315	6.914215	1.016292	0.62875923	0.844328
382522	H2bu1-ps	H2B.U histone 1, pseudogene	NA		0.216439	-0.43323	1.161862	0.62883217	0.844328
73694	Ndufaf7	NADH:ubiquinone oxidoreductase complex assembly	NA		0.034904	4.819732	1.024489	0.62884282	0.844328
66374	2310011J03Rik	RIKEN cDNA 2310011J03 gene	NA		0.05241	3.695545	1.036996	0.62887921	0.844328
66047	Mrpl54	mitochondrial ribosomal protein L54	NA		0.042069	3.893964	1.02959	0.62893547	0.844328
329260	Dennd1b	DENN/MADD domain containing 1B, transcript variar	NA		0.047892	4.009608	1.033754	0.62896641	0.844328
243308	A430033K04Rik	RIKEN cDNA A430033K04 gene	NA		-0.0389	4.800738	-1.02733	0.62899149	0.844328
102634351	Gm31953	predicted gene, 31953	NA		-0.05762	4.831406	-1.04075	0.62917387	0.844404
19726	Rfx3	regulatory factor X, 3 (influences HLA class II expres	NA		0.032905	6.123711	1.02307	0.62917721	0.844404
73721	1110017D15Rik	RIKEN cDNA 1110017D15 gene, transcript variant 7	NA		0.078245	1.80606	1.055733	0.6292036	0.844404
13001	Csnk2b	casein kinase 2, beta polypeptide, transcript variant 2	NA		0.025667	6.566947	1.01795	0.62933359	0.844509
270802	BC048403	cDNA sequence BC048403, transcript variant 1	NA		-0.06774	2.816746	-1.04807	0.62944706	0.844592
17121	Mxd3	Max dimerization protein 3	NA		-0.06521	2.958743	-1.04623	0.62953449	0.844639
116972	Tlcd3a	TLC domain containing 3A, transcript variant 1	NA		0.046531	4.239519	1.032778	0.6296305	0.844679
245945	Rbm47	RNA binding motif protein 47, transcript variant X2	NA		-0.08156	2.023786	-1.05816	0.6296914	0.844679
72554	Utp14a	UTP14A small subunit processome component	NA		-0.04712	5.046408	-1.0332	0.62975033	0.844679
20813	Srp14	signal recognition particle 14	NA		0.025978	6.061801	1.01817	0.62977167	0.844679
100993	AW549542	expressed sequence AW549542	NA		0.156942	0.053752	1.114921	0.62984589	0.844709
15194	Htt	huntingtin	NA		-0.0246	6.136216	-1.0172	0.63003281	0.844839
546524	Gm5953	predicted gene 5953, transcript variant X1	NA		0.072878	2.38214	1.051813	0.63004653	0.844839
14129	Fcgr1	Fc receptor, IgG, high affinity I	NA		0.173019	0.263388	1.127415	0.6301329	0.844885
71131	Zfp689	zinc finger protein 689	NA		-0.03958	4.166865	-1.02781	0.63020408	0.844911
217198	Plekhh3	pleckstrin homology domain containing, family H (with	NA		0.067247	3.007957	1.047716	0.63030514	0.844977
73490	Mipol1	mirror-image polydactyly 1	NA		0.10376	1.253603	1.074571	0.63042759	0.84505
69635	Dapk1	death associated protein kinase 1, transcript variant 1	NA		-0.0287	7.140598	-1.02009	0.63046352	0.84505
102636476	Gm33533	predicted gene, 33533, transcript variant X4	NA		0.1527	0.451623	1.111648	0.63058983	0.845149
104362	Meig1	meiosis expressed gene 1, transcript variant 3	NA		0.149251	0.201701	1.108994	0.63071979	0.845229
17134	Mafg	v-maf musculoaponeurotic fibrosarcoma oncogene fa	NA		-0.02495	6.548653	-1.01745	0.63076275	0.845229
170790	Mlc1	megalencephalic leukoencephalopathy with subcortic	NA		0.033585	4.842912	1.023552	0.63083012	0.845229
98999	Znfx1	zinc finger, NFX1-type containing 1, transcript variant	NA		-0.03726	4.848237	-1.02616	0.63085729	0.845229
76846	Rps9	ribosomal protein S9	NA		-0.02834	8.856989	-1.01984	0.63093515	0.845264
219257	Pcdh20	protocadherin 20	NA		-0.06447	3.30141	-1.0457	0.63110268	0.845419
66349	Dmac2	distal membrane arm assembly complex 2, transcript	NA		0.055695	4.357621	1.03936	0.63139756	0.845428
17979	Ncoa3	nuclear receptor coactivator 3, transcript variant 2	NA		0.03036	5.639285	1.021267	0.6314092	0.845428
66375	Dhrs7	dehydrogenase/reductase (SDR family) member 7	NA		0.067953	2.371995	1.048228	0.63143067	0.845428
117171	1110038F14Rik	RIKEN cDNA 1110038F14 gene, transcript variant 1	NA		0.039474	4.170196	1.027739	0.63145671	0.845428
233529	Kctd14	potassium channel tetramerisation domain containing	NA		-0.11568	0.970542	-1.08349	0.63145847	0.845428
18720	Pip5k1a	phosphatidylinositol-4-phosphate 5-kinase, type 1 alp	NA		-0.02858	5.296438	-1.02001	0.63150575	0.845428
75909	Vmp1	vacuole membrane protein 1, transcript variant 1	NA		0.035054	5.922585	1.024595	0.63155018	0.845428
109042	Cavin3	caveolae associated 3	NA		0.047356	3.385234	1.033369	0.63156241	0.845428
56018	Stard10	START domain containing 10, transcript variant X2	NA		-0.05219	3.254296	-1.03684	0.63157717	0.845428
269389	Tox2	TOX high mobility group box family member 2, transc	NA		0.040437	5.450117	1.028425	0.63162917	0.845428
17075	Epcam	epithelial cell adhesion molecule	NA		-0.08329	1.930448	-1.05943	0.6318227	0.845618
140859	Nek8	NIMA (never in mitosis gene a)-related expressed kin	NA		-0.07394	2.315642	-1.05259	0.6319944	0.84575
219114	Ska3	spindle and kinetochore associated complex subunit	NA		-0.05862	3.188107	-1.04147	0.63202563	0.84575
26411	Map4k1	mitogen-activated protein kinase kinase kinase kinas	NA		0.060232	2.697176	1.042633	0.63208217	0.845756
105245	Txndc5	thioredoxin domain containing 5, transcript variant 1	NA		-0.02503	6.2214	-1.0175	0.63213857	0.845762
233765	Plekha7	pleckstrin homology domain containing, family A men	NA		0.03774	4.292693	1.026505	0.63230996	0.84592
234728	Cmtr2	cap methyltransferase 2	NA		-0.04299	3.945166	-1.03024	0.63236051	0.84592
20723	Serpinb9	serine (or cysteine) peptidase inhibitor, clade B, mem	NA		0.110405	1.304419	1.079531	0.63245135	0.845945
78795	Armc9	armadillo repeat containing 9, transcript variant X16	NA		0.033905	5.040723	1.02378	0.63248343	0.845945
106878	Smim3	small integral membrane protein 3	NA		0.095138	1.14137	1.068168	0.63255381	0.845947
93836	Rnf111	ring finger 111, transcript variant 2	NA		0.026064	6.046183	1.01823	0.63258853	0.845947
66988	Lap3	leucine aminopeptidase 3	NA		-0.03467	4.737484	-1.02432	0.63265332	0.845964
18127	Nos3	nitric oxide synthase 3, endothelial cell	NA		-0.04424	3.593577	-1.03114	0.63282446	0.846123

54405	Ndufa1	NADH:ubiquinone oxidoreductase subunit A1	NA	-0.03361	4.970818	-1.02357	0.63300728	0.846174
19921	Rpl19	ribosomal protein L19, transcript variant 1	NA	0.026837	8.79619	1.018776	0.63304621	0.846174
102640142	Gm36279	predicted gene, 36279, transcript variant X5	NA	-0.16155	-0.1537	-1.11849	0.63310731	0.846174
100038538	Gm10767	predicted gene 10767	NA	0.092042	1.52513	1.065878	0.63320321	0.846174
12581	Cdkn2d	cyclin dependent kinase inhibitor 2D	NA	0.038141	4.934161	1.02679	0.63321179	0.846174
20256	Clec11a	C-type lectin domain family 11, member a	NA	0.105387	1.395549	1.075783	0.63322596	0.846174
19243	Ptp4a1	protein tyrosine phosphatase 4a1	NA	0.022817	8.432949	1.015941	0.63322649	0.846174
68475	Ssna1	SS nuclear autoantigen 1	NA	-0.03769	4.699286	-1.02647	0.63342503	0.846355
228775	Trib3	tribbles pseudokinase 3	NA	0.151974	0.15988	1.111089	0.63346618	0.846355
68176	Inka1	inka box actin regulator 1	NA	0.086786	1.780664	1.062001	0.63355658	0.846407
66921	Prpf38b	PRP38 pre-mRNA processing factor 38 (yeast) doma	NA	-0.02639	6.391903	-1.01846	0.63368915	0.846421
56395	Tmem115	transmembrane protein 115	NA	-0.03908	4.908195	-1.02746	0.63371382	0.846421
231769	Sfswap	splicing factor SWAP, transcript variant 1	NA	-0.02122	7.090275	-1.01482	0.6338008	0.846421
70208	Med23	mediator complex subunit 23, transcript variant X1	NA	0.028074	5.969502	1.01965	0.63393008	0.846421
102633373	Gm31214	predicted gene, 31214, transcript variant X5	NA	0.094258	2.891011	1.067516	0.63394515	0.846421
66229	Rpl7l1	ribosomal protein L7-like 1	NA	-0.02882	6.149576	-1.02018	0.63399122	0.846421
14462	Gata3	GATA binding protein 3, transcript variant 1	NA	0.050705	4.763342	1.035771	0.63399484	0.846421
434233	Ppp1ccb	protein phosphatase 1 catalytic subunit gamma B	NA	0.574301	-1.59399	1.488956	0.63399997	0.846421
66109	Tspan13	tetraspanin 13	NA	-0.0214	7.909442	-1.01494	0.63407427	0.846421
212862	Chpt1	choline phosphotransferase 1, transcript variant 2	NA	0.041298	4.027511	1.029039	0.63408755	0.846421
244723	Olfm2	olfactomedin 2, transcript variant 3	NA	0.023753	6.974229	1.016601	0.63433444	0.846436
14719	Got2	glutamatic-oxaloacetic transaminase 2, mitochondrial	NA	-0.02312	7.619836	-1.01615	0.6344404	0.846436
74479	Snx11	sorting nexin 11	NA	-0.02998	5.361624	-1.021	0.63446305	0.846436
53614	Reck	reversion-inducing-cysteine-rich protein with kazal mo	NA	0.04837	4.211785	1.034096	0.63447183	0.846436
23943	Esyt1	extended synaptotagmin-like protein 1	NA	-0.04341	4.294587	-1.03054	0.63449143	0.846436
67213	Cmtm6	CKLF-like MARVEL transmembrane domain containi	NA	-0.03521	4.407363	-1.02471	0.6345354	0.846436
11843	Arf4	ADP-ribosylation factor 4	NA	0.021698	8.29843	1.015154	0.63455873	0.846436
674895	Nek10	NIMA (never in mitosis gene a)- related kinase 10, tra	NA	-0.1489	0.058995	-1.10872	0.63469574	0.846436
93879	Pcdhb8	protocadherin beta 8	NA	0.100007	1.013206	1.071779	0.63473437	0.846436
14694	Rack1	receptor for activated C kinase 1	NA	0.022437	9.041869	1.015674	0.63476197	0.846436
83383	Tfap4	transcription factor AP4	NA	-0.04275	4.049086	-1.03007	0.63481209	0.846436
11837	Rplp0	ribosomal protein, large, P0	NA	-0.02109	9.802925	-1.01473	0.63482967	0.846436
331480	Gm5126	predicted pseudogene 5126	NA	0.114487	1.096987	1.08259	0.63485056	0.846436
118568353	LOC118568353	uncharacterized LOC118568353	NA	-0.06424	2.832951	-1.04553	0.63486103	0.846436
56715	Rabgef1	RAB guanine nucleotide exchange factor (GEF) 1, tra	NA	0.031189	5.148667	1.021854	0.63490659	0.846436
108116	Slco3a1	solute carrier organic anion transporter family, memb	NA	0.028836	5.259309	1.020188	0.63493107	0.846436
14063	F2rl1	coagulation factor II (thrombin) receptor-like 1	NA	-0.16326	-0.08906	-1.11981	0.63509278	0.84651
11610	Agtrap	angiotensin II, type I receptor-associated protein, trar	NA	-0.05518	3.067131	-1.03899	0.63513748	0.84651
102633471	Gm31288	predicted gene, 31288, transcript variant X4	NA	-0.13574	0.252175	-1.09866	0.63514245	0.84651
18571	Pdcd6ip	programmed cell death 6 interacting protein, transcrip	NA	0.026899	5.982313	1.01882	0.63535254	0.846694
12452	Ccng2	cyclin G2	NA	-0.02742	6.760061	-1.01919	0.63538482	0.846694
232016	Itprid1	ITPR interacting domain containing 1, transcript varia	NA	0.098304	1.880925	1.070514	0.6355081	0.846789
18637	Pfdn2	prefoldin 2, transcript variant 1	NA	-0.03028	6.283906	-1.02121	0.63596153	0.847265
67848	Ddx55	DEAD box helicase 55, transcript variant 1	NA	-0.0429	4.851005	-1.03018	0.63598613	0.847265
213499	Fbxo42	F-box protein 42	NA	-0.0272	5.849165	-1.01903	0.63602156	0.847265
66408	Aptx	aprataxin, transcript variant 1	NA	-0.03013	5.347532	-1.0211	0.63619783	0.847393
109267	Ssc4d	scavenger receptor cysteine rich family, 4 domains	NA	-0.10829	0.690409	-1.07795	0.63625755	0.847393
21346	Tagln2	transgelin 2	NA	0.033276	5.075324	1.023333	0.63627364	0.847393
226250	Afap1l2	actin filament associated protein 1-like 2, transcript va	NA	0.050596	3.897876	1.035693	0.63636158	0.847403
115490200	LOC115490200	zinc finger protein 431-like	NA	0.031112	4.770703	1.021799	0.63639447	0.847403
67725	Nudt13	nudix (nucleoside diphosphate linked moiety X)-type	NA	-0.05019	3.42487	-1.0354	0.63643765	0.847403
213673	9530068E07Rik	RIKEN cDNA 9530068E07 gene	NA	0.022119	6.91271	1.01545	0.63665059	0.847617
101966	D8Ertd738e	DNA segment, Chr 8, ERATO Doi 738, expressed	NA	0.037051	5.344922	1.026014	0.63671874	0.847639
68170	Iftap	intraflagellar transport associated protein, transcript v	NA	0.040982	4.293388	1.028814	0.63678935	0.847663
115487097	LOC115487097	uncharacterized LOC115487097	NA	-0.0778	2.08795	-1.05541	0.63699861	0.847806
434064	Gm5577	predicted gene 5577	NA	0.061393	2.918866	1.043472	0.63700052	0.847806
319601	Zfp653	zinc finger protein 653, transcript variant 1	NA	0.047142	4.4361	1.033216	0.63715689	0.847944
228993	Slc17a9	solute carrier family 17, member 9, transcript variant 1	NA	-0.11784	0.497114	-1.08511	0.63720917	0.847945
11854	Rhod	ras homolog family member D, transcript variant X1	NA	0.102773	1.469675	1.073835	0.63735117	0.847986
330406	B4galnt3	beta-1,4-N-acetyl-galactosaminyl transferase 3, trans	NA	0.176036	0.792098	1.129775	0.63736912	0.847986
16453	Jak3	Janus kinase 3, transcript variant 2	NA	-0.06762	2.479939	-1.04799	0.63743289	0.847986
17159	Man2b1	mannosidase 2, alpha B1	NA	0.031986	4.709778	1.022419	0.63744904	0.847986
51812	Mcrs1	microspherule protein 1, transcript variant 1	NA	0.02392	6.544922	1.016718	0.63751735	0.848008
14609	Gja1	gap junction protein, alpha 1	NA	-0.02983	6.790161	-1.02089	0.6376535	0.848108
218543	Srek1	splicing regulatory glutamine/lysine-rich protein 1, trai	NA	0.030437	6.877284	1.021321	0.63769658	0.848108
55994	Smad9	SMAD family member 9	NA	-0.08803	1.740843	-1.06292	0.63787348	0.848111
78929	Polr3h	polymerase (RNA) III (DNA directed) polypeptide H	NA	0.044006	4.412082	1.030973	0.6378884	0.848111
108943	Trmt10a	tRNA methyltransferase 10A, transcript variant X4	NA	-0.05553	3.134431	-1.03924	0.63794034	0.848111

108909	Aida	axin interactor, dorsalization associated	NA	0.027835	6.302223	1.019481	0.63794133	0.848111
54525	Syt7	synaptotagmin VII, transcript variant 4	NA	0.022642	7.618726	1.015818	0.63795959	0.848111
665574	Gm7694	predicted gene 7694	NA	-0.05128	3.137369	-1.03619	0.63803238	0.848138
115486875	Gm51536	predicted gene, 51536	NA	0.172978	-0.2903	1.127383	0.63810581	0.848167
17909	Myo10	myosin X, transcript variant 3	NA	-0.02559	5.721327	-1.0179	0.63825699	0.848259
373864	Col27a1	collagen, type XXVII, alpha 1, transcript variant X1	NA	-0.04687	3.868062	-1.03302	0.63827913	0.848259
12411	Cbs	cystathionine beta-synthase, transcript variant 2	NA	0.052657	3.035429	1.037173	0.63845516	0.848423
18537	Pcmt1	protein-L-isoaspartate (D-aspartate) O-methyltransfe	NA	0.025184	6.412721	1.017609	0.63851241	0.84843
102637959	Gm38504	predicted gene, 38504	NA	0.084152	2.008151	1.060065	0.63867291	0.84855
68505	Vps51	VPS51 GARP complex subunit	NA	-0.02857	5.253755	-1.02	0.63870716	0.84855
224014	Fgd4	FYVE, RhoGEF and PH domain containing 4, transcr	NA	-0.03409	5.658334	-1.02391	0.63878293	0.848555
21339	Taf1a	TATA-box binding protein associated factor, RNA pol	NA	0.048115	4.463491	1.033913	0.63881532	0.848555
73747	Shld1	shieldin complex subunit 1, transcript variant 1	NA	-0.05355	2.935892	-1.03782	0.63911221	0.84888
353025	Caps2	calcyphosphine 2, transcript variant X8	NA	0.133481	0.083386	1.096937	0.63945685	0.849134
14683	Gnas	GNAS (guanine nucleotide binding protein, alpha stin	NA	0.022112	10.35631	1.015445	0.63948676	0.849134
102638005	Gm11734	predicted gene 11734, transcript variant 2	NA	-0.20335	-0.45617	-1.15137	0.63950961	0.849134
69225	Naxd	NAD(P)HX dehydratase, transcript variant 2	NA	-0.02782	5.314602	-1.01947	0.63951217	0.849134
218038	Amph	amphiphysin, transcript variant 1	NA	-0.02345	6.573598	-1.01639	0.63960146	0.849184
52712	Zkscan6	zinc finger with KRAB and SCAN domains 6, transcri	NA	0.040178	4.266933	1.028241	0.639827	0.849414
225742	St8sia5	ST8 alpha-N-acetyl-neuraminide alpha-2,8-sialyltrans	NA	0.113006	1.439399	1.081479	0.63989088	0.849429
235132	Zbtb44	zinc finger and BTB domain containing 44, transcript	NA	0.029414	5.299669	1.020597	0.64013745	0.849536
67568	Mrfap1	Morf4 family associated protein 1	NA	0.022758	8.121385	1.0159	0.64019647	0.849536
100910	Chpf2	chondroitin polymerizing factor 2, transcript variant 2	NA	0.036441	4.653749	1.025581	0.64019871	0.849536
100503924	Fcor	Foxo1 corepressor	NA	0.163625	-0.19323	1.120098	0.64022448	0.849536
67608	Narf	nuclear prelamin A recognition factor	NA	0.033428	7.05053	1.023441	0.64023252	0.849536
105243391	Gm39326	predicted gene, 39326, transcript variant X2	NA	0.091857	1.499585	1.065741	0.64058606	0.849852
71820	Wdr34	WD repeat domain 34	NA	0.032019	5.076172	1.022442	0.6406358	0.849852
210503	Zfp677	zinc finger protein 677, transcript variant X2	NA	0.058889	2.714231	1.041663	0.64067826	0.849852
217716	Mlh3	mutL homolog 3, transcript variant 2	NA	-0.03948	4.213459	-1.02775	0.64067921	0.849852
14765	Gpr50	G-protein-coupled receptor 50, transcript variant 1	NA	-0.09476	1.755748	-1.06789	0.64078375	0.849921
29861	Dpf1	D4, zinc and double PHD fingers family 1, transcript v	NA	-0.0253	7.111641	-1.01769	0.64083706	0.849922
14287	Fpgs	folylpolyglutamyl synthetase	NA	0.050666	3.775788	1.035743	0.6408891	0.849922
71924	Tube1	tubulin, epsilon 1	NA	0.046407	3.343668	1.03269	0.64107168	0.850095
320808	Dcaf5	DDB1 and CUL4 associated factor 5, transcript variar	NA	0.022473	6.703785	1.015699	0.64124474	0.850255
74840	Manf	mesencephalic astrocyte-derived neurotrophic factor	NA	-0.02662	5.978366	-1.01862	0.64147524	0.850492
20068	Rps17	ribosomal protein S17	NA	0.031539	8.145034	1.022102	0.64156749	0.850545
115487153	LOC115487153	uncharacterized LOC115487153	NA	0.150169	0.266047	1.109699	0.64168185	0.850602
277010	Marveld1	MARVEL (membrane-associating) domain containing	NA	-0.0504	3.658684	-1.03555	0.64171541	0.850602
11553	Adra2c	adrenergic receptor, alpha 2c	NA	0.074466	2.345198	1.052971	0.64179009	0.850623
21821	Ift88	intraflagellar transport 88	NA	0.037944	4.6994	1.02665	0.64184571	0.850623
54447	Asah2	N-acylsphingosine amidohydrolase 2	NA	-0.08949	1.689924	-1.064	0.64188791	0.850623
20352	Sema4b	sema domain, immunoglobulin domain (Ig), transmen	NA	-0.03269	5.219552	-1.02292	0.64194604	0.850631
19108	Prkx	protein kinase, X-linked	NA	0.02522	5.860096	1.017635	0.64216876	0.850782
20563	Slit2	slit guidance ligand 2, transcript variant 3	NA	0.036728	5.405543	1.025785	0.64229679	0.850782
54667	Atp8b2	ATPase, class I, type 8B, member 2, transcript variar	NA	-0.02475	5.81115	-1.01731	0.64235866	0.850782
107607	Nod1	nucleotide-binding oligomerization domain containing	NA	0.098717	1.895076	1.070821	0.64236215	0.850782
22646	Zfp105	zinc finger protein 105	NA	-0.03763	4.079081	-1.02643	0.64240379	0.850782
67874	Rprm	reprimo, TP53 dependent G2 arrest mediator candida	NA	-0.02453	6.376648	-1.01715	0.6424329	0.850782
16010	Igfbp4	insulin-like growth factor binding protein 4	NA	0.023248	6.851361	1.016245	0.64246113	0.850782
79233	Zfp319	zinc finger protein 319	NA	-0.02967	5.29797	-1.02078	0.64254071	0.850782
73137	Prrc1	proline-rich coiled-coil 1, transcript variant X1	NA	0.029434	5.652491	1.020612	0.64261674	0.850782
22184	Zrsr2	zinc finger (CCCH type), RNA binding motif and serin	NA	-0.02712	5.344338	-1.01898	0.64264241	0.850782
103551	Epop	elongin BC and polycomb repressive complex 2 asso	NA	0.056371	3.144062	1.039847	0.64264607	0.850782
56069	Il17b	interleukin 17B	NA	-0.22981	-1.41907	-1.17268	0.64271001	0.850782
102633622	Gm31406	predicted gene, 31406, transcript variant X3	NA	0.153308	0.153964	1.112117	0.64281591	0.850782
94275	Maged1	MAGE family member D1, transcript variant 1	NA	-0.02306	9.739852	-1.01612	0.6428216	0.850782
214917	Antkmt	adenine nucleotide translocase lysine methyltransfera	NA	0.05718	4.016632	1.04043	0.64284424	0.850782
29859	Sult4a1	sulfotransferase family 4A, member 1, transcript varia	NA	0.024246	6.520456	1.016948	0.64290369	0.850791
71355	Col24a1	collagen, type XXIV, alpha 1, transcript variant X1	NA	0.099715	1.46628	1.071562	0.64301132	0.850864
320214	Maats1	MYCBP-associated, testis expressed 1	NA	-0.10919	0.967108	-1.07862	0.64352027	0.851453
67920	Mak16	MAK16 homolog	NA	0.029604	5.542779	1.020732	0.64356045	0.851453
54451	Cpsf3	cleavage and polyadenylation specificity factor 3, trar	NA	0.02624	6.284894	1.018355	0.64371422	0.851487
12328	Caml	calcium modulating ligand	NA	0.029377	5.453204	1.020571	0.64377069	0.851487
110333	Rmst	rhabdomyosarcoma 2 associated transcript (non-codi	NA	-0.0502	3.532846	-1.03541	0.64378515	0.851487
100034739	Gm17762	predicted gene, 17762	NA	0.087289	1.429771	1.062372	0.64384752	0.851487
70673	Prdm16	PR domain containing 16, transcript variant 4	NA	0.033055	4.790529	1.023177	0.64384796	0.851487
11640	Akap1	A kinase (PRKA) anchor protein 1, transcript variant '	NA	0.025546	6.115595	1.017865	0.64396425	0.851571
22213	Ube2g2	ubiquitin-conjugating enzyme E2G 2	NA	0.02857	5.663403	1.020001	0.64407474	0.851588

71163	Zfp626	zinc finger protein 626	NA	-0.04378	4.280075	-1.03081	0.64409844	0.851588
101056072	Gm29773	predicted gene, 29773, transcript variant X2	NA	0.159087	-0.08064	1.116581	0.64414187	0.851588
192897	Itgb4	integrin beta 4, transcript variant X9	NA	-0.10068	1.640321	-1.07228	0.64429007	0.851588
382089	Ripply2	ripply transcriptional repressor 2, transcript variant 1	NA	0.12759	0.406773	1.092467	0.64438685	0.851588
27878	Tada1	transcriptional adaptor 1	NA	-0.03063	5.831269	-1.02146	0.64459346	0.851588
57783	Tnip1	TNFAIP3 interacting protein 1, transcript variant X6	NA	-0.04979	4.05921	-1.03512	0.64459576	0.851588
118568618	LOC118568618	uncharacterized LOC118568618	NA	-0.08411	2.986583	-1.06003	0.64464439	0.851588
93877	Pcdhb6	protocadherin beta 6	NA	-0.09179	1.638943	-1.06569	0.64471668	0.851588
66720	Klhl10	kelch-like 10, transcript variant X3	NA	-0.1597	-0.20481	-1.11706	0.64476819	0.851588
15436	Hoxd4	homeobox D4	NA	-0.1759	0.42538	-1.12967	0.64484157	0.851588
104416	Bap1	Brca1 associated protein 1	NA	-0.02568	6.989962	-1.01796	0.64485378	0.851588
100861698	Gm21145	predicted gene, 21145	NA	-0.09693	1.130679	-1.06949	0.64485524	0.851588
71653	Shtn1	shootin 1, transcript variant 2	NA	0.024304	7.593032	1.016989	0.64491551	0.851588
629159	1700008J07Rik	RIKEN cDNA 1700008J07 gene	NA	-0.07947	2.239248	-1.05663	0.64491584	0.851588
76630	Stambpl1	STAM binding protein like 1, transcript variant 1	NA	-0.05738	3.527972	-1.04058	0.64494164	0.851588
19075	Prim1	DNA primase, p49 subunit	NA	0.030331	4.927892	1.021246	0.64505303	0.851588
72351	Ptar1	protein prenyltransferase alpha subunit repeat contai	NA	-0.04412	5.310736	-1.03105	0.64508939	0.851588
211064	Alkbh1	alkB homolog 1, histone H2A dioxygenase	NA	0.039094	3.958052	1.027468	0.64511468	0.851588
233057	Zfp940	zinc finger protein 940	NA	-0.03913	4.399791	-1.02749	0.64511574	0.851588
106073	Mfsd5	major facilitator superfamily domain containing 5	NA	0.034978	4.251972	1.024541	0.64511874	0.851588
227659	Slc2a6	solute carrier family 2 (facilitated glucose transporter)	NA	-0.10331	1.899673	-1.07424	0.64512842	0.851588
14051	Eya4	EYA transcriptional coactivator and phosphatase 4, tr	NA	0.080614	2.198531	1.057468	0.64540904	0.851752
229722	Elapor1	endosome-lysosome associated apoptosis and autop	NA	-0.03863	4.274688	-1.02714	0.64542585	0.851752
50523	Lats2	large tumor suppressor 2, transcript variant A	NA	0.040517	4.024856	1.028482	0.6454544	0.851752
241447	Cers6	ceramide synthase 6, transcript variant 1	NA	0.027955	6.435623	1.019566	0.64551154	0.851752
66192	Lage3	L antigen family, member 3	NA	-0.04645	3.937624	-1.03272	0.64562306	0.851752
71670	Acy3	aspartoacylase (aminoacylase) 3, transcript variant 1	NA	0.176412	-0.0298	1.13007	0.64564442	0.851752
381418	Ctxn2	cortexin 2, transcript variant X1	NA	0.088017	2.198241	1.062908	0.64566183	0.851752
18209	Ntn3	netrin 3	NA	-0.06155	3.265354	-1.04359	0.64567117	0.851752
108167748	Gm46174	predicted gene, 46174, transcript variant X3	NA	-0.13135	0.301258	-1.09532	0.64576465	0.851806
381820	Smim10l1	small integral membrane protein 10 like 1, transcript v	NA	-0.02434	6.347443	-1.01701	0.64587101	0.851851
52585	Dhrs1	dehydrogenase/reductase (SDR family) member 1	NA	0.029784	5.212593	1.020859	0.64590315	0.851851
12396	Cbfa2t2	CBFA2/RUNX1 translocation partner 2, transcript var	NA	-0.02376	6.285873	-1.01661	0.64602568	0.851874
108167553	LOC108167553	40S ribosomal protein S2-like	NA	0.049347	3.193943	1.034797	0.64605453	0.851874
218294	Cdc14b	CDC14 cell division cycle 14B, transcript variant 2	NA	0.053439	3.04819	1.037736	0.64611804	0.851874
105243280	Gm39244	predicted gene, 39244, transcript variant 2	NA	-0.04813	3.54675	-1.03392	0.64613001	0.851874
238023	Hexdc	hexosaminidase (glycosyl hydrolase family 20, cataly	NA	0.045371	5.716122	1.031948	0.64638388	0.85214
93690	Gpr45	G protein-coupled receptor 45	NA	-0.04506	3.562347	-1.03173	0.64665546	0.852349
230868	Igsf21	immunoglobulin superfamily, member 21	NA	0.029104	5.260827	1.020378	0.64668217	0.852349
72392	Tmem175	transmembrane protein 175, transcript variant X2	NA	-0.03901	4.016649	-1.02741	0.64672016	0.852349
108167704	LOC108167704	uncharacterized LOC108167704, transcript variant X	NA	-0.10122	1.592425	-1.07268	0.64675201	0.852349
238257	Tmem30b	transmembrane protein 30B	NA	-0.17523	-0.40344	-1.12915	0.64693558	0.852522
50914	Olig1	oligodendrocyte transcription factor 1	NA	0.035442	5.346762	1.024871	0.64714041	0.852706
216877	Dhx33	DEAH (Asp-Glu-Ala-His) box polypeptide 33, transcri	NA	-0.03256	5.289804	-1.02283	0.64723185	0.852706
52466	Slc46a1	solute carrier family 46, member 1	NA	-0.05824	2.627397	-1.04119	0.6473588	0.852706
76886	Fam81a	family with sequence similarity 81, member A	NA	-0.04583	3.358791	-1.03228	0.64743	0.852706
16543	Mdfic	MyoD family inhibitor domain containing	NA	-0.06204	3.319692	-1.04394	0.64746688	0.852706
244141	Nars2	asparaginyl-tRNA synthetase 2 (mitochondrial)(putati	NA	0.043256	3.947306	1.030436	0.64747573	0.852706
70294	Rnf126	ring finger protein 126, transcript variant 1	NA	0.030246	5.901742	1.021187	0.64749365	0.852706
114601	Ehbp1l1	EH domain binding protein 1-like 1, transcript variant	NA	0.034626	4.941511	1.024292	0.64752454	0.852706
57748	Jmy	junction-mediating and regulatory protein	NA	0.027371	6.032027	1.019153	0.64756777	0.852706
18040	Nefm	neurofilament, medium polypeptide	NA	0.033512	7.460042	1.023501	0.64768336	0.852706
107260	Otub1	OTU domain, ubiquitin aldehyde binding 1	NA	-0.02182	6.903562	-1.01524	0.64768483	0.852706
28135	Cep63	centrosomal protein 63, transcript variant X19	NA	0.033952	4.81517	1.023813	0.64770417	0.852706
66387	Nudt8	nudix (nucleoside diphosphate linked moiety X)-type	NA	0.061129	2.770483	1.043282	0.64777602	0.852731
21356	Tapbp	TAP binding protein, transcript variant 2	NA	-0.03354	4.641314	-1.02352	0.64796616	0.852913
26442	Psma5	proteasome subunit alpha 5	NA	-0.02584	6.533664	-1.01807	0.64813237	0.853062
14613	Gja5	gap junction protein, alpha 5, transcript variant 2	NA	-0.10451	1.708831	-1.07513	0.64821476	0.853102
14365	Fzd3	frizzled class receptor 3	NA	-0.0244	7.519718	-1.01706	0.64832356	0.853176
78801	Ak7	adenylate kinase 7	NA	-0.07364	2.132458	-1.05237	0.64844165	0.853262
216551	Lgalsl	lectin, galactoside binding-like, transcript variant X1	NA	0.027928	6.377228	1.019547	0.64859745	0.853331
57344	As3mt	arsenite methyltransferase	NA	-0.04795	3.908267	-1.03379	0.64859853	0.853331
12334	Capn2	calpain 2	NA	0.023849	6.683323	1.016668	0.64879203	0.853486
26942	Spag1	sperm associated antigen 1, transcript variant X4	NA	-0.04171	3.943183	-1.02933	0.64882158	0.853486
245880	Wasf3	WASP family, member 3, transcript variant X1	NA	-0.0267	6.500099	-1.01868	0.64894186	0.85356
320302	Glt28d2	glycosyltransferase 28 domain containing 2	NA	-0.13896	0.058567	-1.10111	0.64905032	0.85356
94065	Mrpl34	mitochondrial ribosomal protein L34	NA	-0.04477	4.615552	-1.03152	0.64905383	0.85356
14385	Slc37a4	solute carrier family 37 (glucose-6-phosphate transpo	NA	0.050439	4.188236	1.03558	0.6491516	0.85356

108037	Shmt2	serine hydroxymethyltransferase 2 (mitochondrial), tr	NA	-0.0274	5.577263	-1.01918	0.64920173	0.85356
13972	Gnb1l	guanine nucleotide binding protein (G protein), beta p	NA	-0.06351	2.870743	-1.045	0.64920899	0.85356
244666	Sprtn	SprT-like N-terminal domain, transcript variant X2	NA	0.030957	4.9583	1.02169	0.64924483	0.85356
29815	Bcar3	breast cancer anti-estrogen resistance 3, transcript v	NA	-0.05492	2.871259	-1.0388	0.64933507	0.85361
76965	Slitrk1	SLIT and NTRK-like family, member 1	NA	-0.02894	5.705744	-1.02026	0.64941844	0.85365
21832	Thpo	thrombopoietin, transcript variant X1	NA	-0.10674	1.274918	-1.07679	0.64951633	0.853689
109552	Sri	sorcin, transcript variant 1	NA	0.026148	5.374901	1.01829	0.6495525	0.853689
28295	Gatd3a	glutamine amidotransferase like class 1 domain conta	NA	-0.03687	4.531688	-1.02588	0.64985301	0.854015
20017	Polr1b	polymerase (RNA) I polypeptide B	NA	-0.03134	4.872355	-1.02196	0.64994949	0.854072
21652	Phf1	PHD finger protein 1, transcript variant 1	NA	0.02947	5.212094	1.020637	0.65008533	0.854148
215114	Hip1	huntingtin interacting protein 1, transcript variant 1	NA	-0.02597	7.006161	-1.01816	0.65011202	0.854148
100210	Gpn2	GPN-loop GTPase 2, transcript variant 1	NA	-0.05635	3.016731	-1.03983	0.65022109	0.854222
56471	Stmn4	stathmin-like 4, transcript variant 2	NA	-0.02378	8.231843	-1.01662	0.65043785	0.854238
22070	Tpt1	tumor protein, translationally-controlled 1	NA	-0.02777	10.24246	-1.01944	0.65047888	0.854238
74133	Smg8	smg-8 homolog, nonsense mediated mRNA decay fa	NA	-0.03075	4.732008	-1.02154	0.65051703	0.854238
15400	Hoxa3	homeobox A3	NA	0.116414	2.887152	1.084037	0.65053656	0.854238
109323	C1qtnf7	C1q and tumor necrosis factor related protein 7, trans	NA	-0.13661	0.392707	-1.09932	0.6505457	0.854238
72608	2700069I18Rik	RIKEN cDNA 2700069I18 gene	NA	-0.07217	2.028226	-1.0513	0.6505834	0.854238
97998	Deptor	DEP domain containing MTOR-interacting protein, tra	NA	-0.06039	2.844324	-1.04275	0.65060041	0.854238
21944	Tnfsf12	tumor necrosis factor (ligand) superfamily, member 1	NA	0.088699	1.500503	1.063411	0.65068464	0.854256
20933	Med22	mediator complex subunit 22, transcript variant 2	NA	0.031618	5.862253	1.022158	0.65071931	0.854256
72056	1810055G02Rik	RIKEN cDNA 1810055G02 gene, transcript variant 1	NA	-0.03625	4.704901	-1.02545	0.65086211	0.854366
218693	Paip1	polyadenylate binding protein-interacting protein 1, tra	NA	-0.02147	7.110643	-1.015	0.65090782	0.854366
17536	Meis2	Meis homeobox 2, transcript variant X8	NA	-0.02191	8.44624	-1.0153	0.65115251	0.854618
54608	Abhd2	abhydrolase domain containing 2, transcript variant X	NA	0.021935	6.980631	1.01532	0.65128296	0.85472
18148	Npm1	nucleophosmin 1, transcript variant 1	NA	-0.024	8.906588	-1.01678	0.65134142	0.854728
100504715	Tmppe	transmembrane protein with metallophosphoesterase	NA	-0.03678	4.067252	-1.02582	0.65144578	0.854785
68750	Rreb1	ras responsive element binding protein 1, transcript v	NA	0.045383	3.645847	1.031957	0.65149025	0.854785
66414	Ndufa12	NADH:ubiquinone oxidoreductase subunit A12, trans	NA	0.026994	5.754597	1.018887	0.65158013	0.854834
75613	Med25	mediator complex subunit 25, transcript variant 3	NA	-0.02594	6.697127	-1.01815	0.6519229	0.855215
67397	Erp29	endoplasmic reticulum protein 29	NA	0.024074	6.279222	1.016827	0.65200967	0.85526
93697	Ice2	interactor of little elongation complex ELL subunit 2	NA	-0.0444	3.698099	-1.03126	0.65213398	0.855354
234582	Ccdc102a	coiled-coil domain containing 102A	NA	-0.05255	2.733467	-1.03709	0.65226432	0.855392
66046	Ndufb5	NADH:ubiquinone oxidoreductase subunit B5, transcı	NA	-0.02631	6.12574	-1.0184	0.65228604	0.855392
110083	Dnah12	dynein, axonemal, heavy chain 12, transcript variant	NA	0.162734	-0.27284	1.119406	0.65232036	0.855392
14455	Gas5	growth arrest specific 5, transcript variant 7	NA	-0.02723	6.764659	-1.01906	0.65242167	0.855392
71296	Crnde	colorectal neoplasia differentially expressed (non-pro	NA	0.069387	2.085985	1.049271	0.65242573	0.855392
22654	Zfp13	zinc finger protein 13, transcript variant 2	NA	0.027325	5.448609	1.019121	0.65260713	0.855404
72454	Ccdc71	coiled-coil domain containing 71, transcript variant 2	NA	-0.02666	5.418103	-1.01865	0.65261892	0.855404
67605	Akt1s1	AKT1 substrate 1 (proline-rich), transcript variant 2	NA	0.027105	5.944224	1.018965	0.65263841	0.855404
15402	Hoxa5	homeobox A5	NA	0.170366	3.952491	1.125344	0.65264475	0.855404
56292	Naa10	N(alpha)-acetyltransferase 10, NatA catalytic subunit	NA	0.038688	4.892045	1.027179	0.65274959	0.855472
75600	Calml4	calmodulin-like 4, transcript variant X4	NA	0.075082	2.04068	1.053421	0.65288366	0.855579
77767	Ermn	ermin, ERM-like protein	NA	0.095941	1.246325	1.068763	0.65296915	0.855622
320802	Ifitm10	interferon induced transmembrane protein 10, transcı	NA	0.117537	0.812288	1.084881	0.65308136	0.8557
30963	Hacd1	3-hydroxyacyl-CoA dehydratase 1, transcript variant	NA	-0.0417	4.28395	-1.02933	0.65314264	0.855712
102634755	Gm32261	predicted gene, 32261, transcript variant 1	NA	0.143216	0.55743	1.104364	0.65323834	0.855733
19646	Rbbp4	retinoblastoma binding protein 4, chromatin remodeli	NA	-0.02202	8.206677	-1.01538	0.65340917	0.855733
72635	Lins1	lines homolog 1, transcript variant 2	NA	0.039139	4.32114	1.027501	0.65344315	0.855733
100038492	F630048H11Rik	RIKEN cDNA F630048H11 gene	NA	-0.03684	4.438578	-1.02587	0.65347005	0.855733
319955	Ercc6	excision repair cross-complementing rodent repair de	NA	0.029394	5.261944	1.020583	0.6535483	0.855733
52513	Ddx56	DEAD box helicase 56, transcript variant X2	NA	-0.02907	5.258659	-1.02035	0.65358026	0.855733
100087	Kti12	KTI12 homolog, chromatin associated	NA	-0.04793	3.70566	-1.03378	0.65359785	0.855733
78797	Ndor1	NADPH dependent diflavin oxidoreductase 1, transcr	NA	-0.04182	5.017689	-1.02941	0.6536049	0.855733
240028	Lnpep	leucyl/cystinyl aminopeptidase	NA	-0.0856	2.091941	-1.06113	0.65363918	0.855733
107071	Wdr74	WD repeat domain 74	NA	0.031493	4.77653	1.022069	0.65368478	0.855733
74120	Zfp263	zinc finger protein 263	NA	0.02883	5.294319	1.020185	0.65418225	0.856315
320790	Chd7	chromodomain helicase DNA binding protein 7, trans	NA	0.027814	6.203749	1.019466	0.65431545	0.856342
619287	Rtl4	retrotransposon Gag like 4, transcript variant X7	NA	-0.11831	0.469449	-1.08546	0.65437976	0.856342
68241	Mcrip2	MAPK regulated corepressor interacting protein 2, tra	NA	0.084583	1.81059	1.060381	0.65449879	0.856342
69068	Tcim	transcriptional and immune response regulator	NA	-0.0516	3.360517	-1.03642	0.65451055	0.856342
71795	Pitpnc1	phosphatidylinositol transfer protein, cytoplasmic 1, tr	NA	0.027733	6.632164	1.019409	0.6545211	0.856342
14623	Gjb6	gap junction protein, beta 6, transcript variant 2	NA	0.058023	3.422465	1.041039	0.65456767	0.856342
17684	Cited2	Cbp/p300-interacting transactivator, with Glu/Asp-rich	NA	-0.0364	6.463351	-1.02555	0.65457115	0.856342
432940	Otulin	OTU deubiquitinase with linear linkage specificity	NA	0.035215	4.29254	1.024709	0.65481427	0.856591
76972	Snhg20	small nucleolar RNA host gene 20	NA	0.055944	3.54559	1.039539	0.65502059	0.856748
382395	Gm5174	predicted gene 5174, transcript variant X2	NA	0.148462	-0.31551	1.108387	0.65503907	0.856748
17449	Mdh1	malate dehydrogenase 1, NAD (soluble), transcript va	NA	0.019872	7.831728	1.01387	0.65518091	0.856796

26466	Zfp260	zinc finger protein 260	NA	0.021426	6.39734	1.014962	0.6551856	0.856796
19285	Cavin1	caveolae associated 1, transcript variant 1	NA	-0.03226	5.507538	-1.02261	0.65523374	0.856796
26426	Nubp2	nucleotide binding protein 2, transcript variant 7	NA	-0.04484	4.327326	-1.03157	0.65532311	0.856829
240913	Adamts4	a disintegrin-like and metallopeptidase (reprolysin typ	NA	-0.04527	3.66688	-1.03188	0.65540982	0.856829
330959	Snapc5	small nuclear RNA activating complex, polypeptide 5	NA	-0.04136	3.931605	-1.02908	0.65542829	0.856829
68545	Ecscr	endothelial cell surface expressed chemotaxis and ap	NA	0.072883	2.843963	1.051816	0.65546977	0.856829
233575	Pgap2	post-GPI attachment to proteins 2, transcript variant X	NA	-0.03071	4.742891	-1.02152	0.65578462	0.857171
327799	Usp44	ubiquitin specific peptidase 44, transcript variant X12	NA	0.187694	-0.75014	1.138942	0.65595124	0.85732
16978	Lrrfip1	leucine rich repeat (in FLII) interacting protein 1, trans	NA	0.033168	5.387915	1.023256	0.65607516	0.857413
77929	Yipf6	Yip1 domain family, member 6, transcript variant X1	NA	-0.0296	5.221659	-1.02073	0.65615567	0.85745
69623	Zfp33b	zinc finger protein 33B, transcript variant X1	NA	-0.03689	4.089205	-1.0259	0.65627699	0.857519
12468	Cct7	chaperonin containing Tcp1, subunit 7 (eta)	NA	0.019512	8.159849	1.013617	0.65632101	0.857519
52626	Cdkn2aipnl	CDKN2A interacting protein N-terminal like	NA	0.023281	6.032522	1.016268	0.65638813	0.857519
67513	Faap20	Fanconi anemia core complex associated protein 20,	NA	-0.0368	4.820265	-1.02584	0.65643104	0.857519
56188	Fxyd1	FXYD domain-containing ion transport regulator 1, tra	NA	0.101879	1.437056	1.07317	0.65653573	0.857519
212892	Rsph4a	radial spoke head 4 homolog A (Chlamydomonas)	NA	0.076781	1.749326	1.054662	0.65656223	0.857519
12939	Pcdha7	protocadherin alpha 7	NA	-0.05176	4.333547	-1.03653	0.65665988	0.857519
108167889	Gm46274	predicted gene, 46274	NA	-0.11622	0.850991	-1.08389	0.65667391	0.857519
102639983	Gm36166	predicted gene, 36166	NA	-0.0994	1.379797	-1.07132	0.65672589	0.857519
171167	Fut10	fucosyltransferase 10, transcript variant 5	NA	-0.05948	3.26956	-1.04209	0.65676185	0.857519
18605	Enpp1	ectonucleotide pyrophosphatase/phosphodiesterase	NA	0.059759	2.712396	1.042291	0.65678844	0.857519
69441	Efcab15	EF-hand calcium binding domain 15, transcript variar	NA	0.134494	-0.1633	1.097708	0.657098	0.857744
66174	Nudt14	nudix (nucleoside diphosphate linked moiety X)-type	NA	-0.05611	2.98725	-1.03966	0.65714558	0.857744
83921	Cemip2	cell migration inducing hyaluronidase 2, transcript var	NA	-0.02467	6.18704	-1.01724	0.65720988	0.857744
329387	C230014O12Rik	RIKEN cDNA C230014O12 gene	NA	-0.1024	0.916695	-1.07356	0.65722125	0.857744
238076	Kcns3	potassium voltage-gated channel, delayed-rectifier, s	NA	-0.13615	-0.1382	-1.09897	0.65722406	0.857744
233276	Tubgcp5	tubulin, gamma complex associated protein 5, transc	NA	0.031144	4.939491	1.021822	0.65730272	0.857778
68646	Nadk2	NAD kinase 2, mitochondrial, transcript variant 2	NA	0.035066	5.417622	1.024604	0.65755247	0.857999
23828	Bves	blood vessel epicardial substance, transcript variant X	NA	0.092283	1.711205	1.066056	0.65757793	0.857999
230735	Epha10	Eph receptor A10, transcript variant X1	NA	0.043855	4.836703	1.030864	0.65789238	0.858227
229801	Tram1l1	translocation associated membrane protein 1-like 1	NA	0.025144	5.581581	1.017581	0.65790452	0.858227
67255	Zfp422	zinc finger protein 422, transcript variant X1	NA	-0.02165	7.031682	-1.01512	0.65792247	0.858227
18441	P2ry1	purinergic receptor P2Y, G-protein coupled 1, transcr	NA	0.061919	2.582473	1.043853	0.65796376	0.858227
72183	Snx6	sorting nexin 6	NA	-0.02481	6.403499	-1.01735	0.65810529	0.858343
381668	Fbrsl1	fibrosin-like 1, transcript variant 2	NA	0.022232	6.376823	1.015529	0.65821368	0.8584
80880	Kank3	KN motif and ankyrin repeat domains 3, transcript var	NA	-0.05633	4.157023	-1.03981	0.65825402	0.8584
100043772	Zfp850	zinc finger protein 850	NA	-0.05729	3.748154	-1.04051	0.65834832	0.858454
67728	Dph2	DPH2 homolog	NA	-0.04178	3.928205	-1.02939	0.65842021	0.858466
23874	Farsb	phenylalanyl-tRNA synthetase, beta subunit, transcrip	NA	-0.02161	6.408217	-1.01509	0.65846324	0.858466
74245	Ctbs	chitobiase, transcript variant 1	NA	0.085968	2.007053	1.061399	0.65851575	0.858466
70358	Steap1	six transmembrane epithelial antigen of the prostate	NA	0.146714	-0.12277	1.107045	0.65863628	0.858554
18107	Nmt1	N-myristoyltransferase 1	NA	-0.0223	6.461358	-1.01558	0.65870587	0.858576
15901	Id1	inhibitor of DNA binding 1, HLH protein, transcript var	NA	-0.06016	4.243732	-1.04258	0.65879987	0.85863
16526	Kcnk2	potassium channel, subfamily K, member 2, transcrip	NA	-0.023	5.811988	-1.01607	0.65891337	0.858709
170677	Cdhr1	cadherin-related family member 1	NA	0.055985	3.822065	1.039568	0.65939903	0.859244
71492	Bbs7	Bardet-Biedl syndrome 7 (human), transcript variant X	NA	0.03357	4.27523	1.023542	0.65942999	0.859244
73314	Lrrc69	leucine rich repeat containing 69, transcript variant X	NA	0.086563	1.745554	1.061837	0.65973917	0.85954
102640987	Gm36917	predicted gene, 36917, transcript variant X1	NA	0.183024	0.180079	1.135261	0.65976232	0.85954
115487193	Gm51691	predicted gene, 51691	NA	-0.11218	0.517705	-1.08086	0.65983599	0.859567
102634841	Gm38469	predicted gene, 38469, transcript variant X1	NA	0.190748	-0.31453	1.141355	0.65997952	0.859573
76299	Erp44	endoplasmic reticulum protein 44, transcript variant 1	NA	-0.02434	5.88908	-1.01701	0.65998841	0.859573
21929	Tnfaip3	tumor necrosis factor, alpha-induced protein 3, transc	NA	0.08292	2.290178	1.05916	0.65999892	0.859573
108816	4933409K07Rik	RIKEN cDNA 4933409K07 gene	NA	0.048921	4.007484	1.034491	0.6601615	0.859716
11540	Adora2a	adenosine A2a receptor, transcript variant 1	NA	0.049517	3.312345	1.034918	0.66021713	0.859719
77220	Tmem200a	transmembrane protein 200A, transcript variant X3	NA	0.029451	4.921714	1.020624	0.66032928	0.859797
78783	Brpf1	bromodomain and PHD finger containing, 1, transcrip	NA	-0.03286	5.456104	-1.02304	0.66061606	0.860047
214597	Sidt2	SID1 transmembrane family, member 2, transcript va	NA	-0.02565	6.208399	-1.01794	0.66062742	0.860047
68837	Foxk2	forkhead box K2, transcript variant 1	NA	-0.02073	7.362792	-1.01447	0.6606813	0.860048
73739	Cby1	chibby family member 1, beta catenin antagonist	NA	0.026244	5.339364	1.018357	0.66079153	0.860049
215194	Kri1	KRI1 homolog	NA	-0.02938	5.217841	-1.02057	0.66082443	0.860049
75695	Rilpl1	Rab interacting lysosomal protein-like 1	NA	-0.04184	3.906729	-1.02943	0.66089179	0.860049
56072	Lgals12	lectin, galactose binding, soluble 12, transcript varian	NA	0.124071	0.35218	1.089806	0.66089341	0.860049
232086	Tmem150a	transmembrane protein 150A	NA	0.035719	4.36507	1.025068	0.66109315	0.860227
102640525	Gm36565	predicted gene, 36565, transcript variant X1	NA	0.143299	-0.17721	1.104428	0.66113596	0.860227
382686	3110053B16Rik	RIKEN cDNA 3110053B16 gene, transcript variant X	NA	0.026428	5.518378	1.018487	0.66141943	0.860454
71532	Fam217b	family with sequence similarity 217, member B, trans	NA	-0.03325	4.843825	-1.02331	0.6614553	0.860454
381835	Sbk3	SH3 domain binding kinase family, member 3, transc	NA	-0.08215	1.87643	-1.0586	0.66146909	0.860454
14579	Gem	GTP binding protein (gene overexpressed in skeletal	NA	0.084495	1.492509	1.060317	0.66157997	0.86053

98682	Mfsd6	major facilitator superfamily domain containing 6, trar	NA	0.033212	5.130745	1.023288	0.66178492	0.860728
118568708	LOC118568708	uncharacterized LOC118568708	NA	-0.06042	2.334538	-1.04277	0.66196231	0.860843
69863	Ttc39b	tetratricopeptide repeat domain 39B, transcript varian	NA	0.049619	4.362536	1.034992	0.66197948	0.860843
72368	Borcs8	BLOC-1 related complex subunit 8, transcript variant	NA	-0.03457	4.622531	-1.02425	0.66220628	0.861069
26370	Cetn2	centrin 2	NA	0.033356	4.928796	1.02339	0.66239968	0.861197
115487194	LOC115487194	uncharacterized LOC115487194	NA	-0.0709	2.141175	-1.05037	0.66244995	0.861197
320435	Rinl	Ras and Rab interactor-like, transcript variant 1	NA	-0.12038	0.415094	-1.08702	0.66246344	0.861197
80385	Tusc2	tumor suppressor 2, mitochondrial calcium regulator	NA	0.025105	5.623638	1.017554	0.66254186	0.86123
13396	Dlx6	distal-less homeobox 6	NA	-0.0589	2.828811	-1.04167	0.66295469	0.861698
13393	Dlx3	distal-less homeobox 3	NA	-0.14603	-0.45263	-1.10652	0.66305326	0.861733
235072	Septin7	septin 7, transcript variant 2	NA	0.020091	7.804339	1.014023	0.66316879	0.861733
12974	Cs	citrate synthase	NA	-0.01962	8.176264	-1.01369	0.66323154	0.861733
12889	Cplx1	complexin 1	NA	-0.0248	6.244259	-1.01734	0.66333529	0.861733
108124	Napa	N-ethylmaleimide sensitive fusion protein attachment	NA	-0.02235	6.587693	-1.01561	0.66338847	0.861733
22412	Wnt9b	wingless-type MMTV integration site family, member	NA	0.139824	0.531716	1.101771	0.66341253	0.861733
13202	Ddt	D-dopachrome tautomerase	NA	0.036748	3.972685	1.025799	0.66343394	0.861733
238377	Gpr68	G protein-coupled receptor 68, transcript variant 2	NA	0.070635	2.102619	1.050179	0.66346983	0.861733
432879	Kbtbd6	kelch repeat and BTB (POZ) domain containing 6	NA	-0.07141	1.634784	-1.05074	0.66347888	0.861733
66126	Elof1	ELF1 homolog, elongation factor 1, transcript variant	NA	0.033154	5.368771	1.023247	0.66354703	0.861733
69920	Polr2i	polymerase (RNA) II (DNA directed) polypeptide I	NA	-0.05152	4.25742	-1.03636	0.6635688	0.861733
66736	Emc2	ER membrane protein complex subunit 2	NA	0.033426	5.268392	1.02344	0.66363931	0.861733
105734727	Gm27021	predicted gene, 27021	NA	-0.1438	0.651275	-1.10481	0.66367003	0.861733
20182	Rxrb	retinoid X receptor beta, transcript variant 2	NA	-0.02474	5.918417	-1.0173	0.66374118	0.861757
654801	Zfp784	zinc finger protein 784	NA	0.057853	2.637997	1.040915	0.66411159	0.862169
74026	Msl1	male specific lethal 1, transcript variant 4	NA	0.018404	7.860797	1.012838	0.66420988	0.862181
245671	Klf8	Kruppel-like factor 8, transcript variant 1	NA	0.033534	5.063732	1.023516	0.66426894	0.862181
59001	Pole3	polymerase (DNA directed), epsilon 3 (p17 subunit)	NA	0.042201	3.752246	1.029684	0.66428009	0.862181
12675	Chuk	conserved helix-loop-helix ubiquitous kinase, transcri	NA	0.027314	5.925154	1.019113	0.66453658	0.862438
105245239	Gm40720	predicted gene, 40720, transcript variant X1	NA	0.13379	0.067778	1.097172	0.6646082	0.862438
320508	Cachd1	cache domain containing 1	NA	-0.02762	5.528672	-1.01933	0.66467536	0.862438
102634821	Gm32316	predicted gene, 32316, transcript variant X3	NA	-0.13763	0.261944	-1.1001	0.66468975	0.862438
195209	Zfp469	zinc finger protein 469	NA	-0.07477	2.010452	-1.05319	0.66478876	0.862497
20392	Sgce	sarcoglycan, epsilon, transcript variant 2	NA	-0.02666	5.100692	-1.01865	0.66484248	0.862498
330577	Saxo2	stabilizer of axonemal microtubules 2, transcript varia	NA	0.076304	1.85121	1.054313	0.66495152	0.862571
236366	5730507C01Rik	RIKEN cDNA 5730507C01 gene, transcript variant 2	NA	-0.10242	1.260929	-1.07357	0.66522748	0.862594
56330	Pdcd5	programmed cell death 5	NA	0.028689	5.661358	1.020085	0.66523507	0.862594
66817	Tmem170	transmembrane protein 170, transcript variant X1	NA	-0.0499	2.867571	-1.03519	0.66524477	0.862594
68033	Cox19	cytochrome c oxidase assembly protein 19	NA	0.033293	4.870109	1.023345	0.66530699	0.862594
109685	Hyal3	hyaluronoglucosaminidase 3	NA	-0.12287	0.1997	-1.0889	0.66531373	0.862594
111368	Prn	prion protein readthrough transcript, transcript variant	NA	0.289441	-1.34863	1.222167	0.66535281	0.862594
668940	Myh7b	myosin, heavy chain 7B, cardiac muscle, beta, transc	NA	-0.15216	0.095314	-1.11123	0.6653701	0.862594
118568698	LOC118568698	uncharacterized LOC118568698	NA	0.17433	-0.26167	1.12844	0.66543141	0.862594
64929	Scel	sciellin, transcript variant X1	NA	-0.13078	-0.0213	-1.09488	0.66544934	0.862594
16154	Il10ra	interleukin 10 receptor, alpha, transcript variant 3	NA	-0.13759	0.158039	-1.10007	0.66551173	0.862594
100502874	Gm19426	predicted gene, 19426	NA	-0.06587	2.352092	-1.04671	0.66555221	0.862594
12921	Crhr1	corticotropin releasing hormone receptor 1, transcript	NA	0.042443	3.759988	1.029856	0.66566671	0.862623
209683	Ttc28	tetratricopeptide repeat domain 28, transcript variant	NA	0.023985	8.247433	1.016764	0.66569995	0.862623
102639129	Gm35515	predicted gene, 35515	NA	-0.09665	0.992873	-1.06929	0.66575401	0.862623
237847	Rtn4rl1	reticulon 4 receptor-like 1	NA	0.037491	4.035552	1.026328	0.66584259	0.862623
74443	P4htm	prolyl 4-hydroxylase, transmembrane (endoplasmic r	NA	0.043037	3.829852	1.030281	0.66588861	0.862623
100042132	Gm12371	predicted gene 12371	NA	0.07165	1.99247	1.050918	0.66603629	0.862623
102632244	Gm30375	predicted gene, 30375	NA	-0.1415	-0.23127	-1.10305	0.66608445	0.862623
232156	Slc4a5	solute carrier family 4, sodium bicarbonate cotranspo	NA	-0.07399	2.492419	-1.05263	0.6661172	0.862623
115489683	Gm52604	predicted gene, 52604	NA	0.129016	0.038765	1.093548	0.66619144	0.862623
85029	Rpph1	ribonuclease P RNA component H1	NA	0.163089	-0.24385	1.119682	0.66619301	0.862623
102635192	Gm16835	predicted gene, 16835	NA	0.146717	0.865128	1.107047	0.66619447	0.862623
14761	Gpr27	G protein-coupled receptor 27	NA	0.032833	6.430859	1.023019	0.66621113	0.862623
16872	Lhx4	LIM homeobox protein 4, transcript variant X1	NA	-0.09692	1.068689	-1.06949	0.6663816	0.86268
654409	Stk35os1	serine/threonine kinase 35, opposite strand 1	NA	-0.04742	3.041178	-1.03342	0.66638244	0.86268
71765	Klhdc3	kelch domain containing 3, transcript variant 1	NA	0.023239	7.203322	1.016238	0.6664193	0.86268
105244074	Gm39752	predicted gene, 39752	NA	0.065075	5.142462	1.04614	0.66648648	0.86268
69597	Afg3l2	AFG3-like AAA ATPase 2	NA	0.029086	5.590556	1.020365	0.66656618	0.86268
209200	Dtx3l	deltex 3-like, E3 ubiquitin ligase	NA	0.07852	1.790409	1.055934	0.6666186	0.86268
13491	Drd4	dopamine receptor D4	NA	-0.18675	-0.38443	-1.1382	0.66662596	0.86268
228602	4930402H24Rik	RIKEN cDNA 4930402H24 gene, transcript variant 1	NA	-0.02024	7.333216	-1.01413	0.66673329	0.86275
19182	Psmc3	proteasome (prosome, macropain) 26S subunit, ATP	NA	0.021823	7.136368	1.015241	0.66703319	0.862996
19416	Rasd1	RAS, dexamethasone-induced 1	NA	0.153032	0.059477	1.111904	0.66706912	0.862996
208266	Dot1l	DOT1-like, histone H3 methyltransferase (S. cerevisia	NA	-0.02067	6.564408	-1.01443	0.66708241	0.862996

56205	Ensa	endosulfine alpha, transcript variant 2	NA	0.024359	7.142135	1.017028	0.66731896	0.863083
27355	Pald1	phosphatase domain containing, paladin 1, transcript	NA	-0.03186	4.897626	-1.02233	0.66736493	0.863083
383103	Tvp23a	trans-golgi network vesicle protein 23A, transcript var	NA	-0.03347	6.011825	-1.02347	0.66737821	0.863083
50797	Copb2	coatomer protein complex, subunit beta 2 (beta prime	NA	-0.01914	7.35696	-1.01335	0.66744532	0.863083
67041	Oxct1	3-oxoacid CoA transferase 1	NA	-0.01908	8.013625	-1.01331	0.66749348	0.863083
59287	Ncstn	nicastrin	NA	0.021718	6.616535	1.015168	0.66751641	0.863083
68169	Ndnf	neuron-derived neurotrophic factor, transcript variant	NA	0.041938	4.549206	1.029496	0.6676016	0.863083
75729	Fam227a	family with sequence similarity 227, member A, transc	NA	-0.03649	4.709392	-1.02562	0.66761288	0.863083
212190	Ubxn10	UBX domain protein 10, transcript variant 2	NA	-0.07275	1.995066	-1.05172	0.66762669	0.863083
77252	9430038I01Rik	RIKEN cDNA 9430038I01 gene	NA	0.07157	1.852431	1.050859	0.66775001	0.863106
15214	Hey2	hairy/enhancer-of-split related with YRPW motif 2	NA	0.048637	2.95593	1.034287	0.6677509	0.863106
140781	Myh7	myosin, heavy polypeptide 7, cardiac muscle, beta, tr	NA	0.045755	3.060182	1.032224	0.6679763	0.863211
108660	Rnf187	ring finger protein 187	NA	-0.01926	8.421705	-1.01344	0.66802378	0.863211
14738	Gpr12	G-protein coupled receptor 12, transcript variant 2	NA	-0.04239	3.642123	-1.02982	0.66806885	0.863211
18577	Pde4a	phosphodiesterase 4A, cAMP specific, transcript vari	NA	-0.02986	5.555682	-1.02091	0.66811246	0.863211
105244644	Lrp8os3	low density lipoprotein receptor-related protein 8, apo	NA	-0.10827	0.925622	-1.07794	0.66812147	0.863211
15353	Hmg20b	high mobility group 20B, transcript variant 1	NA	-0.03614	4.165779	-1.02536	0.66815062	0.863211
330790	Hapln4	hyaluronan and proteoglycan link protein 4	NA	0.14481	0.029262	1.105585	0.6682308	0.863246
208890	Slc26a7	solute carrier family 26, member 7, transcript variant	NA	0.066963	2.340223	1.047509	0.66843904	0.863447
14745	Lpar1	lysophosphatidic acid receptor 1, transcript variant X	NA	-0.0338	4.460973	-1.02371	0.66870755	0.863653
17174	Masp1	mannan-binding lectin serine peptidase 1, transcript v	NA	-0.02303	6.32195	-1.01609	0.66878624	0.863653
227331	Gigyf2	GRB10 interacting GYF protein 2, transcript variant X	NA	-0.02812	6.231333	-1.01968	0.6687873	0.863653
66394	Nosip	nitric oxide synthase interacting protein, transcript var	NA	0.028978	5.208688	1.020289	0.66881118	0.863653
18550	Furin	furin (paired basic amino acid cleaving enzyme), tran	NA	0.032157	5.270881	1.02254	0.66893651	0.863732
328440	Npm2	nucleophosmin/nucleoplasmin 2	NA	0.182618	-0.41686	1.134942	0.66898964	0.863732
232341	Wnk1	WNK lysine deficient protein kinase 1, transcript varia	NA	0.020218	7.424622	1.014113	0.66912008	0.863732
235180	Fez1	fasciculation and elongation protein zeta 1 (zygin I), t	NA	-0.0248	8.012911	-1.01734	0.66916247	0.863732
574437	Xlr3b	X-linked lymphocyte-regulated 3B	NA	0.103431	2.654499	1.074325	0.66919893	0.863732
115489937	Gm52702	predicted gene, 52702	NA	-0.10833	0.450763	-1.07798	0.66923983	0.863732
100039864	Snhg12	small nucleolar RNA host gene 12	NA	0.044752	3.925803	1.031506	0.66926872	0.863732
22629	Ywhah	tyrosine 3-monooxygenase/tryptophan 5-monooxyge	NA	0.018111	8.593774	1.012632	0.66929682	0.863732
72269	Cda	cytidine deaminase	NA	0.148683	0.076622	1.108557	0.66939641	0.863765
30948	Bin1	bridging integrator 1, transcript variant 3	NA	0.019872	6.978259	1.01387	0.66942866	0.863765
16502	Kcnc1	potassium voltage gated channel, Shaw-related subfa	NA	-0.02482	6.133608	-1.01735	0.66952838	0.863802
240726	Slco5a1	solute carrier organic anion transporter family, memb	NA	-0.02869	5.771205	-1.02009	0.66961863	0.863802
16001	Igf1r	insulin-like growth factor I receptor, transcript variant	NA	-0.02055	7.319675	-1.01435	0.66963834	0.863802
67158	Sft2d3	SFT2 domain containing 3	NA	0.061351	2.566374	1.043443	0.66973787	0.863802
71147	Oxsm	3-oxoacyl-ACP synthase, mitochondrial, transcript va	NA	-0.05835	3.059687	-1.04128	0.66976889	0.863802
14028	Evx1	even-skipped homeobox 1	NA	-0.11326	0.786384	-1.08167	0.66977592	0.863802
69544	Wdr5b	WD repeat domain 5B	NA	-0.04496	3.147183	-1.03166	0.66983252	0.863807
13841	Epha7	Eph receptor A7, transcript variant 1	NA	0.025482	6.296556	1.01782	0.66999145	0.863918
109054	Pfdn4	prefoldin 4, transcript variant 2	NA	0.030547	5.141755	1.021399	0.67007724	0.863918
16477	Junb	jun B proto-oncogene	NA	0.07761	1.984097	1.055268	0.67012374	0.863918
434215	Lrrc32	leucine rich repeat containing 32, transcript variant X	NA	0.054674	2.919956	1.038624	0.67014905	0.863918
12750	Clk4	CDC like kinase 4, transcript variant 8	NA	0.028725	5.95876	1.02011	0.67018445	0.863918
216227	Slc17a8	solute carrier family 17 (sodium-dependent inorganic	NA	0.062948	2.717319	1.044598	0.67029469	0.863947
100503380	Snhg4	small nucleolar RNA host gene 4	NA	0.043402	3.337073	1.030541	0.67035921	0.863947
63830	Kcnq1ot1	KCNQ1 overlapping transcript 1	NA	0.02799	6.889791	1.019591	0.67036617	0.863947
228410	Cstf3	cleavage stimulation factor, 3' pre-RNA, subunit 3, tra	NA	-0.02486	6.007984	-1.01738	0.67078981	0.864371
68929	Mospd3	motile sperm domain containing 3, transcript variant 2	NA	0.034261	5.061334	1.024032	0.67084092	0.864371
55932	Gbp3	guanylate binding protein 3, transcript variant 2	NA	-0.16463	-0.06507	-1.12088	0.67090429	0.864371
230752	Eva1b	eva-1 homolog B (C. elegans)	NA	-0.07116	1.929083	-1.05056	0.67092597	0.864371
16867	Lhcgr	luteinizing hormone/choriogonadotropin receptor, tran	NA	0.119966	1.484005	1.086709	0.67103084	0.864371
15484	Hsd11b2	hydroxysteroid 11-beta dehydrogenase 2	NA	-0.10748	1.462591	-1.07735	0.67108662	0.864371
69551	2310022B05Rik	RIKEN cDNA 2310022B05 gene	NA	-0.02154	7.00466	-1.01504	0.67110964	0.864371
73068	Fut11	fucosyltransferase 11	NA	0.032611	4.55558	1.022862	0.6713052	0.864371
67681	Mrpl18	mitochondrial ribosomal protein L18	NA	-0.02602	5.457296	-1.0182	0.67135191	0.864371
12091	Glb1	galactosidase, beta 1, transcript variant 3	NA	-0.0302	4.767106	-1.02116	0.67138001	0.864371
68519	Eml1	echinoderm microtubule associated protein like 1, tra	NA	0.027377	5.952097	1.019157	0.67138635	0.864371
226422	Rab29	RAB29, member RAS oncogene family	NA	0.070327	1.735927	1.049955	0.67138833	0.864371
667171	Gm8493	predicted gene 8493, transcript variant X2	NA	-0.09517	2.117924	-1.06819	0.67139532	0.864371
23999	Twf2	twinfilin actin binding protein 2	NA	-0.03023	4.657359	-1.02117	0.67143859	0.864371
19698	Relb	avian reticuloendotheliosis viral (v-rel) oncogene rela	NA	0.080696	1.765631	1.057528	0.67161669	0.864521
237052	Tceal1	transcription elongation factor A (SII)-like 1, transcript	NA	0.034501	4.498081	1.024202	0.67166165	0.864521
170736	Parvb	parvin, beta	NA	-0.02754	5.161211	-1.01927	0.67185287	0.864674
102162	Taf5l	TATA-box binding protein associated factor 5 like, tra	NA	0.028545	4.827445	1.019983	0.67188674	0.864674
235534	Pxylp1	2-phosphoxylose phosphatase 1, transcript variant 4	NA	-0.03378	4.819836	-1.02369	0.67202455	0.86475
109299	Tmem250-ps	transmembrane protein 250, pseudogene, transcript	NA	0.028902	4.739913	1.020235	0.67206285	0.86475

11647	Alpl	alkaline phosphatase, liver/bone/kidney, transcript va	NA	-0.0536	3.540893	-1.03785	0.67214728	0.86475
67943	Mesd	mesoderm development LRP chaperone, transcript v	NA	0.024802	5.743847	1.01734	0.67215785	0.86475
105246016	C730034F03Rik	RIKEN cDNA C730034F03 gene, transcript variant X	NA	0.16273	-0.34127	1.119403	0.67223301	0.864778
74485	Lrrc71	leucine rich repeat containing 71	NA	0.118946	0.020579	1.085942	0.67234329	0.864851
208606	Rsrc2	arginine/serine-rich coiled-coil 2, transcript variant 1	NA	0.019304	6.829706	1.013471	0.67257158	0.864994
66256	Ssr2	signal sequence receptor, beta, transcript variant 2	NA	0.037159	6.318862	1.026091	0.67259746	0.864994
22235	Ugdh	UDP-glucose dehydrogenase, transcript variant X1	NA	-0.02315	5.835041	-1.01617	0.67263683	0.864994
69155	1810030O07Rik	RIKEN cDNA 1810030O07 gene	NA	-0.02702	5.250949	-1.0189	0.67266652	0.864994
118567667	LOC118567667	uncharacterized LOC118567667, transcript variant X	NA	-0.12386	0.343795	-1.08965	0.67283015	0.865136
100503962	Gm16701	predicted gene, 16701	NA	-0.10473	1.685111	-1.07529	0.67332403	0.865702
218232	Ptpdc1	protein tyrosine phosphatase domain containing 1, tra	NA	0.025816	5.227128	1.018055	0.67352261	0.865853
100503178	2810013P06Rik	RIKEN cDNA 2810013P06 gene	NA	-0.03793	4.918888	-1.02664	0.67354772	0.865853
83396	Glis2	GLIS family zinc finger 2, transcript variant X2	NA	-0.03113	4.914016	-1.02181	0.6736807	0.865956
545938	Zfp607a	zinc finger protein 607A, transcript variant X2	NA	-0.10193	0.774531	-1.0732	0.67377479	0.865959
72562	Pcbd2	pterin 4 alpha carbinolamine dehydratase/dimerizatio	NA	0.0849	1.192457	1.060615	0.67378965	0.865959
381204	Naaladl1	N-acetylated alpha-linked acidic dipeptidase-like 1	NA	-0.11054	0.407102	-1.07963	0.67386512	0.865987
56748	Nfu1	NFU1 iron-sulfur cluster scaffold, transcript variant 2	NA	0.034195	5.159861	1.023985	0.67392874	0.866001
18024	Nfe2l2	nuclear factor, erythroid derived 2, like 2, transcript va	NA	0.034355	3.979839	1.024099	0.67399779	0.866021
100503622	Gm16630	predicted gene, 16630, transcript variant 2	NA	0.140676	-0.09838	1.102421	0.67417384	0.866127
622675	Zfp827	zinc finger protein 827, transcript variant 1	NA	0.023273	6.356073	1.016262	0.67421551	0.866127
71911	Bdh1	3-hydroxybutyrate dehydrogenase, type 1, transcript	NA	-0.01957	6.85402	-1.01366	0.67424002	0.866127
70415	Stk26	serine/threonine kinase 26, transcript variant 1	NA	0.093709	1.818114	1.06711	0.67432574	0.866169
99686	AI606473	expressed sequence AI606473	NA	0.065523	2.175048	1.046464	0.67445353	0.866198
14842	Gsx1	GS homeobox 1	NA	0.083724	1.321371	1.05975	0.67445504	0.866198
17089	Lyar	Ly1 antibody reactive clone	NA	0.03708	4.45419	1.026035	0.67456377	0.86627
17096	Lyn	LYN proto-oncogene, Src family tyrosine kinase, tran	NA	0.048769	3.141881	1.034382	0.67475272	0.866402
231876	Lmtk2	lemur tyrosine kinase 2	NA	0.024488	6.182531	1.017118	0.67477326	0.866402
68135	Eif3h	eukaryotic translation initiation factor 3, subunit H	NA	0.022873	7.903627	1.015981	0.67483549	0.866413
654309	Nrp	neural regeneration protein	NA	-0.07623	1.788453	-1.05426	0.67489558	0.866422
15424	Hoxc5	homeobox C5	NA	-0.21759	1.177908	-1.16279	0.67504891	0.866482
15410	Hoxb3	homeobox B3, transcript variant X6	NA	0.110721	3.775705	1.079768	0.67504931	0.866482
244886	Tmem266	transmembrane protein 266, transcript variant 1	NA	0.047176	3.335542	1.03324	0.67513294	0.866482
230654	Lrrc41	leucine rich repeat containing 41, transcript variant X	NA	-0.02182	5.924595	-1.01524	0.67515512	0.866482
320858	L3mbtl4	L3MBTL4 histone methyl-lysine binding protein	NA	-0.06805	1.837899	-1.0483	0.67522083	0.866498
72500	Ier5l	immediate early response 5-like	NA	0.051347	2.837717	1.036232	0.67527452	0.866498
69260	Ing2	inhibitor of growth family, member 2, transcript varian	NA	-0.04448	3.598394	-1.03131	0.6753444	0.866501
11492	Adam19	a disintegrin and metallopeptidase domain 19 (meltrin	NA	-0.02523	5.764554	-1.01764	0.67543128	0.866501
232807	Ppp1r12c	protein phosphatase 1, regulatory subunit 12C	NA	0.022262	6.035281	1.015551	0.67553024	0.866501
13389	Dll3	delta like canonical Notch ligand 3	NA	0.064836	3.186095	1.045966	0.67553611	0.866501
13480	Dpm1	dolichol-phosphate (beta-D) mannosyltransferase 1, t	NA	0.055299	3.074041	1.039074	0.67554248	0.866501
545527	Erich6	glutamate rich 6	NA	-0.1673	-0.36835	-1.12296	0.67575071	0.866614
30878	Apln	apelin	NA	-0.04568	3.473589	-1.03217	0.67588344	0.866614
319634	Efcab5	EF-hand calcium binding domain 5, transcript variant	NA	0.062163	2.30202	1.04403	0.67590229	0.866614
93737	Pard6g	par-6 family cell polarity regulator gamma	NA	0.024058	5.327363	1.016816	0.67591507	0.866614
17874	Myd88	myeloid differentiation primary response gene 88	NA	0.062637	2.302073	1.044373	0.67593467	0.866614
14778	Gpx3	glutathione peroxidase 3, transcript variant 1	NA	-0.06832	3.980458	-1.04849	0.67598178	0.866614
72873	Bbof1	basal body orientation factor 1, transcript variant X4	NA	0.052361	3.245965	1.03696	0.67600359	0.866614
67270	Mrpl42	mitochondrial ribosomal protein L42, transcript varian	NA	0.025884	5.27559	1.018103	0.67619926	0.866768
76281	Tax1bp3	Tax1 (human T cell leukemia virus type I) binding pro	NA	0.022747	5.735095	1.015892	0.67623064	0.866768
60365	Rbm8a	RNA binding motif protein 8a, transcript variant 2	NA	-0.0202	6.718257	-1.0141	0.67656436	0.867052
668501	Zfp507	zinc finger protein 507, transcript variant X2	NA	-0.02484	5.376356	-1.01737	0.67662109	0.867052
76055	Oga	O-GlcNAcase, transcript variant X3	NA	0.02219	8.197283	1.015499	0.67662187	0.867052
231602	P2rx2	purinergic receptor P2X, ligand-gated ion channel, 2,	NA	0.054162	2.816597	1.038256	0.67666509	0.867052
81702	Ankrd17	ankyrin repeat domain 17, transcript variant X7	NA	0.021736	7.370914	1.01518	0.67678927	0.867088
217708	Lin52	lin-52 homolog (C. elegans)	NA	0.034298	4.534736	1.024059	0.67680599	0.867088
111365222	Rps18-ps4	ribosomal protein S18, pseudogene 4	NA	0.092047	0.991028	1.065881	0.67685318	0.867088
67844	Rab32	RAB32, member RAS oncogene family	NA	-0.09504	1.57762	-1.0681	0.67691067	0.867094
72560	Naalad2	N-acetylated alpha-linked acidic dipeptidase 2, transc	NA	-0.09473	1.446978	-1.06787	0.6769721	0.867104
30838	Fbxw4	F-box and WD-40 domain protein 4	NA	-0.033	4.198099	-1.02314	0.67736988	0.867531
108897	Aif1l	allograft inflammatory factor 1-like	NA	-0.03287	5.368515	-1.02304	0.6774124	0.867531
66940	Shisa5	shisa family member 5, transcript variant X3	NA	-0.03054	5.000916	-1.02139	0.67756197	0.867655
19354	Rac2	Rac family small GTPase 2	NA	-0.1098	1.097953	-1.07908	0.67785383	0.867754
15373	Hmx3	H6 homeobox 3, transcript variant X3	NA	-0.07428	1.825364	-1.05283	0.67787156	0.867754
102639705	Gm35956	predicted gene, 35956	NA	0.098279	1.054977	1.070496	0.67790803	0.867754
12709	Ckb	creatine kinase, brain	NA	0.028196	8.466614	1.019736	0.67793118	0.867754
319247	9530080O11Rik	RIKEN cDNA 9530080O11 gene, transcript variant 2	NA	0.170602	-0.12109	1.125528	0.67794015	0.867754
68048	Aen	apoptosis enhancing nuclease, transcript variant 1	NA	-0.0242	5.559485	-1.01692	0.67796227	0.867754
14563	Gdf5	growth differentiation factor 5	NA	0.093279	1.016896	1.066792	0.67801291	0.867754

227298	Retreg2	reticulophagy regulator family member 2, transcript va	NA	-0.0239	6.928725	-1.01671	0.67822239	0.867845
72672	Zfp518a	zinc finger protein 518A	NA	0.034701	4.113054	1.024344	0.67823292	0.867845
171580	Mical1	microtubule associated monooxygenase, calponin an	NA	-0.03063	4.838598	-1.02146	0.67824374	0.867845
102637025	Gm33937	predicted gene, 33937, transcript variant X3	NA	0.119995	1.115175	1.086731	0.67831096	0.867863
16649	Kpna4	karyopherin (importin) alpha 4	NA	0.024226	6.755359	1.016934	0.6783966	0.867904
115488768	Gm52285	predicted gene, 52285, transcript variant X2	NA	0.130466	0.201469	1.094647	0.67852287	0.867997
664994	Isoc2a	isochorismatase domain containing 2a	NA	-0.05103	3.200646	-1.036	0.67867076	0.868118
53323	Ube2k	ubiquitin-conjugating enzyme E2K, transcript variant	NA	0.019848	7.223141	1.013853	0.67881289	0.868232
243371	Lrrc61	leucine rich repeat containing 61, transcript variant 1	NA	-0.02786	4.954102	-1.0195	0.6789677	0.868361
227059	Slc39a10	solute carrier family 39 (zinc transporter), member 10	NA	0.021188	7.002864	1.014795	0.67912877	0.868499
56041	Uso1	USO1 vesicle docking factor	NA	0.021128	6.458244	1.014753	0.67943736	0.868725
102636239	Gm27042	predicted gene, 27042	NA	-0.12484	0.19477	-1.09039	0.67950989	0.868725
320234	Ccdc66	coiled-coil domain containing 66, transcript variant X3	NA	-0.03744	4.466274	-1.02629	0.6795183	0.868725
381290	Atp2b4	ATPase, Ca++ transporting, plasma membrane 4, tra	NA	-0.02077	6.501817	-1.0145	0.67951914	0.868725
66294	Fam3a	family with sequence similarity 3, member A, transcrip	NA	0.031656	4.290853	1.022184	0.67970402	0.868893
217119	Xylt2	xylosyltransferase II	NA	-0.03034	4.327648	-1.02125	0.67978585	0.86893
80883	Ntng1	netrin G1, transcript variant e	NA	-0.03314	5.575334	-1.02324	0.67997249	0.869062
70225	Ppil3	peptidylprolyl isomerase (cyclophilin)-like 3, transcript	NA	0.032872	4.280049	1.023047	0.68003768	0.869062
215090	Maneal	mannosidase, endo-alpha-like, transcript variant X2	NA	0.024918	5.735196	1.017422	0.68018922	0.869062
56532	Ripk3	receptor-interacting serine-threonine kinase 3, transc	NA	-0.1087	0.419779	-1.07826	0.68022249	0.869062
320295	C920006O11Rik	RIKEN cDNA C920006O11 gene	NA	0.070941	2.198734	1.050401	0.68023594	0.869062
230726	Rhbdl2	rhomboid like 2, transcript variant X1	NA	0.142458	-0.00123	1.103784	0.68026948	0.869062
52150	Kcnk6	potassium inwardly-rectifying channel, subfamily K, m	NA	0.08396	1.228801	1.059923	0.68027074	0.869062
69938	Scrn1	secernin 1, transcript variant X1	NA	-0.02698	6.656796	-1.01887	0.68033061	0.869062
52065	Mfhas1	malignant fibrous histiocytoma amplified sequence 1,	NA	-0.03055	5.477721	-1.0214	0.68042072	0.869062
26903	Dysf	dysferlin, transcript variant X25	NA	0.063623	2.655795	1.045087	0.68047801	0.869062
102638847	Gm35315	predicted gene, 35315, transcript variant X3	NA	-0.07609	1.440223	-1.05416	0.68056999	0.869062
14797	Tle5	TLE family member 5, transcriptional modulator, trans	NA	0.024699	8.030112	1.017267	0.68065417	0.869062
21856	Timm44	translocase of inner mitochondrial membrane 44	NA	-0.02887	5.120694	-1.02021	0.68075225	0.869062
66770	4933429O19Rik	RIKEN cDNA 4933429O19 gene	NA	0.132418	-0.25337	1.096129	0.68076172	0.869062
53951	Gpatch11	G patch domain containing 11	NA	-0.0271	4.832265	-1.01896	0.68087797	0.869062
57319	Smpdl3a	sphingomyelin phosphodiesterase, acid-like 3A	NA	0.039202	4.204332	1.027545	0.6808933	0.869062
116871	Mta3	metastasis associated 3, transcript variant 7	NA	-0.03209	5.815156	-1.02249	0.68091264	0.869062
216080	Ube2d1	ubiquitin-conjugating enzyme E2D 1, transcript variar	NA	0.021519	7.395065	1.015028	0.68097574	0.869062
112406	Egln2	egl-9 family hypoxia-inducible factor 2, transcript vari	NA	-0.02108	6.184893	-1.01472	0.68098127	0.869062
56351	Ptges3	prostaglandin E synthase 3	NA	-0.01763	7.835929	-1.01229	0.68103665	0.869062
66787	Gskip	GSK3B interacting protein	NA	0.0377	4.084606	1.026476	0.68108909	0.869062
320159	Togaram2	TOG array regulator of axonemal microtubules 2, trar	NA	0.132549	0.237725	1.096229	0.68109918	0.869062
14810	Grin1	glutamate receptor, ionotropic, NMDA1 (zeta 1), trans	NA	-0.02105	6.046779	-1.0147	0.68116046	0.869062
269693	Ccdc60	coiled-coil domain containing 60, transcript variant 2	NA	-0.06805	1.916382	-1.0483	0.68117874	0.869062
18553	Pcsk6	proprotein convertase subtilisin/kexin type 6, transcrip	NA	0.040236	3.312412	1.028282	0.68124864	0.869062
68616	Gdpd3	glycerophosphodiester phosphodiesterase domain cc	NA	0.120373	0.213522	1.087016	0.68130025	0.869062
21334	Tac2	tachykinin 2, transcript variant 1	NA	-0.13023	0.169129	-1.09447	0.68133116	0.869062
22344	Vezf1	vascular endothelial zinc finger 1	NA	-0.02387	6.905703	-1.01669	0.68141089	0.869095
18582	Pde6d	phosphodiesterase 6D, cGMP-specific, rod, delta, tra	NA	-0.02268	5.678485	-1.01585	0.68170725	0.869405
226414	Dars	aspartyl-tRNA synthetase, transcript variant 1	NA	0.021448	6.485253	1.014978	0.68184334	0.869471
15228	Foxg1	forkhead box G1, transcript variant 1	NA	-0.03933	8.298212	-1.02764	0.68189827	0.869471
67049	Pus3	pseudouridine synthase 3	NA	0.040776	3.731694	1.028667	0.68191906	0.869471
100038384	Gm10610	predicted gene 10610, transcript variant X1	NA	0.111416	0.334812	1.080288	0.68201786	0.869529
72400	Pinx1	PIN2/TERF1 interacting, telomerase inhibitor 1	NA	0.044682	3.647846	1.031456	0.68222111	0.86972
12555	Cdh15	cadherin 15	NA	0.104822	1.362878	1.075362	0.68233916	0.869802
102634132	B230208B08Rik	RIKEN cDNA B230208B08 gene	NA	0.061482	2.62142	1.043537	0.68247958	0.869823
12321	Calu	calumenin, transcript variant 1	NA	0.022642	7.610168	1.015818	0.68249109	0.869823
115486472	Gm51454	predicted gene, 51454	NA	-0.10232	0.472217	-1.0735	0.68256011	0.869823
320100	Relt	RELT tumor necrosis factor receptor, transcript variar	NA	0.058195	2.179973	1.041163	0.68256972	0.869823
71801	Plekhf2	pleckstrin homology domain containing, family F (with	NA	-0.03177	4.483933	-1.02226	0.68289077	0.870164
71752	Gtf3c2	general transcription factor IIIC, polypeptide 2, beta	NA	-0.01953	6.680121	-1.01363	0.68302412	0.870266
67419	Armh4	armadillo-like helical domain containing 4, transcript v	NA	-0.03616	4.105196	-1.02538	0.68309667	0.87029
102436	Lars2	leucyl-tRNA synthetase, mitochondrial, transcript vari	NA	0.188434	10.00036	1.139526	0.68330746	0.870491
105243496	Gm39400	predicted gene, 39400, transcript variant X2	NA	0.152545	-0.13938	1.111529	0.68345352	0.870609
69674	Mif4gd	MIF4G domain containing, transcript variant 2	NA	0.060514	2.633123	1.042837	0.68358113	0.870703
66967	Edem3	ER degradation enhancer, mannosidase alpha-like 3,	NA	-0.0323	6.061708	-1.02264	0.68374002	0.870837
24127	Xrn1	5'-3' exoribonuclease 1, transcript variant X7	NA	-0.02543	5.872026	-1.01778	0.68392329	0.870971
52023	Pibf1	progesterone immunomodulatory binding factor 1, tra	NA	-0.03739	4.122789	-1.02625	0.68395205	0.870971
18036	Nfkbib	nuclear factor of kappa light polypeptide gene enhanc	NA	0.036643	4.150267	1.025724	0.68402001	0.870989
613264	1810020O05Rik	Riken cDNA 1810020O05 gene, transcript variant X4	NA	0.098025	1.207341	1.070307	0.68421643	0.871158
24116	Nelfa	negative elongation factor complex member A, Whsc	NA	-0.02547	5.385699	-1.01781	0.68455303	0.871158
208795	Tmem63a	transmembrane protein 63a	NA	0.069401	1.679719	1.049281	0.68455363	0.871158

432628	Mfsd2b	major facilitator superfamily domain containing 2B	NA	-0.07617	1.718163	-1.05421	0.68457811	0.871158
102639045	Gm15564	predicted gene 15564, transcript variant X1	NA	0.185227	7.870539	1.136996	0.68461421	0.871158
83436	Plekha2	pleckstrin homology domain-containing, family A (phc	NA	0.05381	2.828006	1.038002	0.68467781	0.871158
140488	Igf2bp3	insulin-like growth factor 2 mRNA binding protein 3, tı	NA	-0.02607	5.689636	-1.01823	0.68468616	0.871158
56248	Ak3	adenylate kinase 3, transcript variant 1	NA	0.020212	6.252157	1.014109	0.68475875	0.871158
102638401	Gm34978	predicted gene, 34978, transcript variant X2	NA	0.153586	-0.28384	1.112331	0.68481375	0.871158
118568569	LOC118568569	uncharacterized LOC118568569	NA	0.119126	0.139714	1.086077	0.68484543	0.871158
102633730	Gm31485	predicted gene, 31485	NA	0.218173	-0.66889	1.16326	0.68494801	0.871158
233802	Thumpd1	THUMP domain containing 1	NA	0.020094	6.392432	1.014026	0.68494995	0.871158
216169	Abhd17a	abhydrolase domain containing 17A	NA	0.034036	5.663851	1.023872	0.68501424	0.871158
102015	AI225912	expressed sequence AI225912	NA	0.135718	0.441846	1.098639	0.68501889	0.871158
65963	Tmem176b	transmembrane protein 176B, transcript variant 6	NA	-0.02617	5.158694	-1.01831	0.68503936	0.871158
102635502	Gm17396	predicted gene, 17396	NA	-0.0948	1.628957	-1.06792	0.68504238	0.871158
100503884	Ccdc149	coiled-coil domain containing 149, transcript variant 2	NA	0.038804	4.329871	1.027262	0.68506233	0.871158
67099	Mettl21a	methyltransferase like 21A, transcript variant 3	NA	-0.03868	3.674494	-1.02717	0.68519701	0.871201
227693	Zer1	zyg-11 related, cell cycle regulator, transcript variant	NA	-0.02027	6.241321	-1.01415	0.68520368	0.871201
19084	Prkar1a	protein kinase, cAMP dependent regulatory, type I, al	NA	-0.01848	9.015546	-1.01289	0.68528712	0.871239
21833	Thra	thyroid hormone receptor alpha, transcript variant 1	NA	0.017883	9.397461	1.012473	0.685482	0.871411
226265	Eno4	enolase 4	NA	-0.1231	1.179715	-1.08907	0.68555879	0.871411
56530	Cnpy2	canopy FGF signaling regulator 2	NA	-0.03296	5.388384	-1.02311	0.68561938	0.871411
66847	Hint3	histidine triad nucleotide binding protein 3, transcript	NA	-0.13358	3.104572	-1.09702	0.68563641	0.871411
11504	Adamts1	a disintegrin-like and metallopeptidase (reprolysin typ	NA	-0.04173	4.039005	-1.02935	0.68581397	0.871569
105348	Golm1	golgi membrane protein 1, transcript variant 2	NA	-0.03365	5.634687	-1.0236	0.68595965	0.871658
26394	Lypla2	lysophospholipase 2	NA	-0.02371	6.226614	-1.01657	0.68599157	0.871658
15369	Hmox2	heme oxygenase 2, transcript variant 1	NA	0.026765	5.725222	1.018725	0.68627039	0.871944
27056	Irf5	interferon regulatory factor 5, transcript variant 2	NA	0.089982	0.77511	1.064357	0.68633313	0.871956
68339	Ccdc88c	coiled-coil domain containing 88C, transcript variant X	NA	0.02659	6.237795	1.018602	0.68648406	0.872066
19273	Ptpru	protein tyrosine phosphatase, receptor type, U, transc	NA	-0.03117	5.182966	-1.02184	0.68665747	0.872066
16663	Krt13	keratin 13, transcript variant 1	NA	-0.16583	0.096932	-1.12181	0.68666441	0.872066
83490	Pik3ap1	phosphoinositide-3-kinase adaptor protein 1, transcrip	NA	0.06893	1.711391	1.048938	0.68667085	0.872066
26447	Poli	polymerase (DNA directed), iota, transcript variant X2	NA	0.035875	3.773436	1.025178	0.68668786	0.872066
329831	Fam166b	family with sequence similarity 166, member B, transc	NA	-0.12481	-0.1976	-1.09036	0.6867782	0.872113
67770	Caap1	caspase activity and apoptosis inhibitor 1	NA	-0.03727	3.890881	-1.02617	0.68691552	0.872219
12767	Cxcr4	chemokine (C-X-C motif) receptor 4, transcript varian	NA	0.025611	5.335672	1.017911	0.68735703	0.872712
13360	Dhcr7	7-dehydrocholesterol reductase, transcript variant X1	NA	0.022371	6.058594	1.015628	0.68744783	0.872759
67988	Tmx3	thioredoxin-related transmembrane protein 3	NA	-0.02484	5.744049	-1.01736	0.68767366	0.872862
115488316	Gm52112	predicted gene, 52112	NA	0.059436	2.069163	1.042058	0.68768108	0.872862
76133	6230400D17Rik	RIKEN cDNA 6230400D17 gene	NA	0.169082	-0.47171	1.124343	0.68772943	0.872862
73261	1700037C18Rik	RIKEN cDNA 1700037C18 gene	NA	0.099281	1.446781	1.07124	0.68774334	0.872862
227620	Uap1l1	UDP-N-acteylglucosamine pyrophosphorylase 1-like	NA	-0.07985	1.400772	-1.05691	0.68783806	0.872914
236511	Ago1	argonaute RISC catalytic subunit 1, transcript variant	NA	0.020086	7.719768	1.01402	0.68799103	0.872983
24015	Abce1	ATP-binding cassette, sub-family E (OABP), member	NA	0.020309	7.10316	1.014177	0.68808453	0.872983
19258	Ptpn4	protein tyrosine phosphatase, non-receptor type 4, tra	NA	-0.02823	6.093156	-1.01976	0.68810818	0.872983
319552	Spx	spexin hormone, transcript variant 1	NA	-0.07528	1.837987	-1.05356	0.68814508	0.872983
102637946	Gm34632	predicted gene, 34632	NA	0.129893	0.3019	1.094212	0.68816066	0.872983
93757	Immp2l	IMP2 inner mitochondrial membrane peptidase-like (S	NA	-0.14264	0.050902	-1.10392	0.68848938	0.873332
108900	Fam72a	family with sequence similarity 72, member A	NA	-0.07535	1.340875	-1.05362	0.68865569	0.873475
16562	Kif1c	kinesin family member 1C, transcript variant X6	NA	-0.02742	5.823709	-1.01919	0.68877193	0.873554
69071	Tmem97	transmembrane protein 97	NA	-0.03411	4.119465	-1.02393	0.68910662	0.873666
50525	Spag6l	sperm associated antigen 6-like	NA	-0.04264	3.096155	-1.03	0.68914631	0.873666
105246572	Gm41844	predicted gene, 41844	NA	0.062561	2.776907	1.044318	0.68914948	0.873666
67186	Rplp2	ribosomal protein, large P2, transcript variant 1	NA	0.035769	7.250491	1.025103	0.68920724	0.873666
16456	F11r	F11 receptor	NA	-0.04067	3.355863	-1.02859	0.68925507	0.873666
98396	Slc41a1	solute carrier family 41, member 1, transcript variant	NA	-0.02727	5.329468	-1.01908	0.6892834	0.873666
102635503	Gm32822	predicted gene, 32822	NA	-0.15446	-0.09633	-1.11301	0.68932393	0.873666
83431	Ndel1	nudE neurodevelopment protein 1 like 1, transcript va	NA	0.020113	6.022151	1.014039	0.68939964	0.873666
12015	Bad	BCL2-associated agonist of cell death, transcript vari	NA	0.029564	4.594507	1.020704	0.68940075	0.873666
103140	Gstt3	glutathione S-transferase, theta 3, transcript variant 1	NA	-0.13578	-0.25756	-1.09869	0.68943445	0.873666
18710	Pik3r3	phosphoinositide-3-kinase regulatory subunit 3, trans	NA	-0.02209	7.195182	-1.01543	0.68945076	0.873666
70020	Ino80b	INO80 complex subunit B	NA	-0.03967	3.858184	-1.02788	0.6895491	0.873722
14107	Fat1	FAT atypical cadherin 1	NA	0.024029	6.689465	1.016795	0.68964654	0.873738
234699	Edc4	enhancer of mRNA decapping 4, transcript variant X2	NA	-0.02442	6.114768	-1.01707	0.68966851	0.873738
75465	Dynlrb2	dynein light chain roadblock-type 2	NA	0.079689	1.169214	1.05679	0.68980981	0.873849
67222	Srfbp1	serum response factor binding protein 1	NA	0.036581	4.14512	1.02568	0.68986797	0.873854
108159	Ubxn8	UBX domain protein 8	NA	0.038112	3.475559	1.026769	0.69010213	0.87406
74122	Tmem43	transmembrane protein 43	NA	-0.02275	5.683921	-1.01589	0.69013794	0.87406
20341	Selenbp1	selenium binding protein 1	NA	0.059832	2.752955	1.042344	0.69028633	0.87418
22635	Zan	zonadhesin, transcript variant X7	NA	-0.10935	0.374766	-1.07874	0.69053572	0.874428

72175	Mfsd8	major facilitator superfamily domain containing 8, trar	NA	0.03482	4.325108	1.024429	0.69070387	0.874558
70081	Zfp995	zinc finger protein 995, transcript variant X6	NA	-0.04263	3.799594	-1.02999	0.690746	0.874558
545611	Fam205a2	family with sequence similarity 205, member A2, tran	NA	-0.06565	2.750665	-1.04655	0.69084055	0.87461
207686	Cfap69	cilia and flagella associated protein 69, transcript vari	NA	0.037091	4.137366	1.026043	0.69094164	0.87467
67842	Nop9	NOP9 nucleolar protein	NA	-0.03074	4.772251	-1.02153	0.69100403	0.87468
102634922	Gm32391	predicted gene, 32391, transcript variant 2	NA	0.072978	1.876413	1.051886	0.6912338	0.874893
66264	Ccdc28b	coiled coil domain containing 28B	NA	0.029332	5.164779	1.02054	0.69128952	0.874893
105244244	Gm39887	predicted gene, 39887	NA	-0.10504	0.58406	-1.07553	0.69133362	0.874893
18750	Prkca	protein kinase C, alpha	NA	0.023119	5.853543	1.016154	0.69138954	0.874896
320736	Vstm4	V-set and transmembrane domain containing 4, trans	NA	-0.04779	3.151833	-1.03368	0.69149292	0.874959
546134	Gramd2	GRAM domain containing 2, transcript variant 4	NA	0.042668	3.288769	1.030017	0.69164445	0.875083
68801	Elovl5	ELOVL family member 5, elongation of long chain fat	NA	-0.01977	6.22636	-1.0138	0.69200043	0.875465
228788	Ccm2l	cerebral cavernous malformation 2-like, transcript var	NA	0.062368	2.245187	1.044178	0.69209004	0.87551
320502	Lmod3	leiomodin 3 (fetal), transcript variant X2	NA	-0.1693	-0.73106	-1.12451	0.69214837	0.875516
18557	Cdk18	cyclin-dependent kinase 18	NA	-0.10106	0.796125	-1.07256	0.69225776	0.875586
234959	Med17	mediator complex subunit 17, transcript variant 1	NA	0.024577	5.142223	1.017182	0.69234475	0.875628
58248	1700123O20Rik	RIKEN cDNA 1700123O20 gene, transcript variant X	NA	0.029022	4.842321	1.02032	0.69240665	0.875631
14269	Fnbp1	formin binding protein 1, transcript variant 5	NA	0.01893	6.433624	1.013208	0.69245456	0.875631
100504195	Micalcl	MICAL C-terminal like, transcript variant 2	NA	0.108011	0.523744	1.077741	0.69255464	0.875636
378435	Mafa	v-maf musculoaponeurotic fibrosarcoma oncogene fa	NA	-0.07312	2.87587	-1.05199	0.69256596	0.875636
78308	Gpr108	G protein-coupled receptor 108, transcript variant 2	NA	-0.03736	3.963118	-1.02624	0.6927651	0.87582
22380	Wbp4	WW domain binding protein 4	NA	-0.02477	5.908368	-1.01732	0.69291435	0.87594
68161	A930005H10Rik	RIKEN cDNA A930005H10 gene, transcript variant 2	NA	-0.06835	1.64349	-1.04851	0.69307045	0.876069
22271	Upp1	uridine phosphorylase 1, transcript variant 3	NA	0.131785	-0.33984	1.095648	0.69323282	0.876207
66658	Ccdc51	coiled-coil domain containing 51	NA	-0.0481	3.175576	-1.03391	0.69363278	0.876644
105148	Iars	isoleucine-tRNA synthetase	NA	0.01978	6.339534	1.013805	0.69394359	0.87694
70726	Angptl6	angiopoietin-like 6, transcript variant X2	NA	0.052982	2.583561	1.037407	0.69398235	0.87694
260409	Cdc42ep3	CDC42 effector protein (Rho GTPase binding) 3	NA	0.026004	5.302214	1.018188	0.69402831	0.87694
100504455	Gm15834	predicted gene 15834, transcript variant X4	NA	0.089822	1.059656	1.064239	0.69419626	0.877084
66358	Adprm	ADP-ribose/CDP-alcohol diphosphatase, manganese	NA	-0.04114	3.595476	-1.02893	0.69426919	0.877108
210004	B3gntl1	UDP-GlcNAc:betaGal beta-1,3-N-acetylglucosaminylt	NA	0.063996	2.846268	1.045358	0.69440826	0.877215
276770	Eif5a	eukaryotic translation initiation factor 5A, transcript va	NA	-0.02011	8.517019	-1.01404	0.69453732	0.877235
14198	Fhit	fragile histidine triad gene, transcript variant 3	NA	-0.08595	1.501139	-1.06138	0.69462077	0.877235
595139	E030024N20Rik	RIKEN cDNA E030024N20 gene	NA	0.076165	1.942151	1.054212	0.69466046	0.877235
105246878	Gm42084	predicted gene, 42084	NA	0.113092	0.556301	1.081544	0.69466102	0.877235
72141	Adpgk	ADP-dependent glucokinase, transcript variant 1	NA	0.031558	4.577187	1.022115	0.6947905	0.877235
72504	Taf4b	TATA-box binding protein associated factor 4b, trans	NA	0.097198	1.136155	1.069694	0.69479349	0.877235
231503	Tmem150c	transmembrane protein 150C, transcript variant 3	NA	0.021161	5.80431	1.014776	0.69480086	0.877235
19822	Rnf4	ring finger protein 4, transcript variant 3	NA	-0.0188	6.8384	-1.01312	0.69505299	0.877466
102631912	Ndufb1	NADH:ubiquinone oxidoreductase subunit B1, transc	NA	-0.02478	5.042447	-1.01732	0.69509164	0.877466
21413	Tcf4	transcription factor 4, transcript variant X26	NA	0.020611	9.293545	1.014389	0.69535612	0.877608
102634944	Gm32404	predicted gene, 32404	NA	-0.05469	2.412971	-1.03863	0.69536409	0.877608
102639545	Gm35835	predicted gene, 35835, transcript variant X1	NA	-0.0335	4.153445	-1.02349	0.6953661	0.877608
244310	Dlgap2	DLG associated protein 2, transcript variant X3	NA	-0.03415	4.143417	-1.02395	0.69550451	0.877674
69894	Fam241b	family with sequence similarity 241, member B, trans	NA	-0.02372	5.577156	-1.01658	0.69557637	0.877674
56441	Naa80	N(alpha)-acetyltransferase 80, NatH catalytic subunit	NA	0.040619	3.699947	1.028555	0.69558027	0.877674
240725	Sulf1	sulfatase 1, transcript variant X7	NA	-0.02611	5.706778	-1.01826	0.69575848	0.877804
240660	Slc35g1	solute carrier family 35, member G1	NA	-0.0774	1.373311	-1.05512	0.69579104	0.877804
338367	Myo1d	myosin ID, transcript variant X6	NA	-0.07739	2.006604	-1.05511	0.69585096	0.877811
21418	Tfap2a	transcription factor AP-2, alpha, transcript variant X3	NA	0.044135	3.949493	1.031065	0.69608708	0.878041
56505	Ruvbl1	RuvB-like protein 1	NA	-0.02248	5.598235	-1.01571	0.6962304	0.878099
58175	Rgs20	regulator of G-protein signaling 20, transcript variant	NA	0.032296	4.03401	1.022638	0.69624068	0.878099
68108	Snhg17	small nucleolar RNA host gene 17, transcript variant	NA	0.028877	4.609148	1.020218	0.69635941	0.878138
72709	C1qtnf6	C1q and tumor necrosis factor related protein 6, trans	NA	-0.0399	3.633506	-1.02804	0.6963797	0.878138
15212	Hexb	hexosaminidase B	NA	-0.02683	4.869504	-1.01877	0.69650417	0.878227
269233	Fam171a1	family with sequence similarity 171, member A1, tran	NA	0.023531	6.258261	1.016444	0.69662349	0.878309
18768	Pkib	protein kinase inhibitor beta, cAMP dependent, testis	NA	-0.08391	0.832162	-1.05989	0.69668522	0.878319
69718	Ipmk	inositol polyphosphate multikinase, transcript variant	NA	0.024817	5.607806	1.01735	0.69692424	0.878552
104215	Rhoq	ras homolog family member Q	NA	0.027237	5.20541	1.019059	0.6971777	0.878799
15478	Hs3st3a1	heparan sulfate (glucosamine) 3-O-sulfotransferase 3	NA	-0.06808	1.615008	-1.04832	0.69722823	0.878799
66262	Ing5	inhibitor of growth family, member 5, transcript varian	NA	-0.04091	4.047698	-1.02876	0.69735453	0.878874
19173	Psmb5	proteasome (prosome, macropain) subunit, beta type	NA	0.029941	6.278187	1.02097	0.69739528	0.878874
57751	Rnf25	ring finger protein 25, transcript variant 1	NA	0.028245	5.246901	1.019771	0.6975575	0.878908
15289	Hmgb1	high mobility group box 1, transcript variant 2	NA	0.01635	9.397606	1.011397	0.69757439	0.878908
269224	Pask	PAS domain containing serine/threonine kinase, trans	NA	-0.05725	3.018534	-1.04048	0.69758483	0.878908
66680	Oser1	oxidative stress responsive serine rich 1, transcript va	NA	0.026352	5.049692	1.018434	0.69763964	0.878909
105243955	Gm39662	predicted gene, 39662, transcript variant X1	NA	0.122379	0.313853	1.088528	0.69780625	0.878949
22701	Zfp41	zinc finger protein 41, transcript variant 2	NA	0.020659	6.069103	1.014423	0.69787603	0.878949

68146	Arl13b	ADP-ribosylation factor-like 13B	NA	-0.02811	4.595221	-1.01967	0.69792553	0.878949
211535	Ccdc114	coiled-coil domain containing 114, transcript variant 3	NA	-0.05633	2.36502	-1.03982	0.69793707	0.878949
13516	Epyc	epiphycan, transcript variant 1	NA	-0.12613	0.097796	-1.09136	0.69794141	0.878949
22194	Ube2e1	ubiquitin-conjugating enzyme E2E 1	NA	0.022268	6.586249	1.015555	0.69803698	0.879002
72318	Cyth4	cytohesin 4, transcript variant X3	NA	0.064589	1.972553	1.045787	0.69810612	0.879021
14910	Gt(ROSA)26Sor	gene trap ROSA 26, Philippe Soriano, transcript varia	NA	-0.03011	4.188339	-1.02109	0.69831846	0.879207
81913	Bambi-ps1	BMP and activin membrane-bound inhibitor, pseudog	NA	0.09997	0.682927	1.071751	0.69836194	0.879207
78802	Ttc30a1	tetratricopeptide repeat domain 30A1	NA	-0.0369	3.585771	-1.02591	0.69855649	0.879369
18648	Pgam1	phosphoglycerate mutase 1	NA	0.020398	8.161799	1.014239	0.69866325	0.879369
56447	Copz1	coatomer protein complex, subunit zeta 1, transcript v	NA	-0.02063	6.670363	-1.0144	0.69871112	0.879369
69574	Cmbl	carboxymethylenebutenolidase-like (Pseudomonas)	NA	-0.07764	2.090621	-1.05529	0.6987494	0.879369
211383	Amer3	APC membrane recruitment 3	NA	0.023519	5.91576	1.016436	0.6987615	0.879369
17101	Lyst	lysosomal trafficking regulator, transcript variant X1	NA	0.037904	4.578252	1.026621	0.69882741	0.879384
77622	Apex2	apurinic/apyrimidinic endonuclease 2	NA	-0.0341	4.269585	-1.02392	0.69920309	0.879789
320183	Msrb3	methionine sulfoxide reductase B3	NA	0.046909	3.003521	1.033049	0.69951662	0.880061
102637464	Gm34267	predicted gene, 34267	NA	0.108265	0.13777	1.077931	0.69952728	0.880061
384281	Gatc	glutamyl-tRNA(Gln) amidotransferase, subunit C	NA	-0.02326	5.307868	-1.01625	0.69987789	0.880434
71063	Zfp597	zinc finger protein 597, transcript variant X1	NA	-0.02511	4.891659	-1.01756	0.7	0.880458
218214	Kdm1b	lysine (K)-specific demethylase 1B, transcript variant	NA	0.023259	5.353903	1.016252	0.70000527	0.880458
414758	Zfp950	zinc finger protein 950, transcript variant X2	NA	0.035443	5.2252	1.024871	0.70007495	0.880478
19819	Rnaseh1	ribonuclease H1, transcript variant 1	NA	0.036945	3.619665	1.025939	0.70017982	0.880536
269608	Plekhg5	pleckstrin homology domain containing, family G (witl	NA	-0.03166	4.522283	-1.02219	0.70023042	0.880536
12350	Car3	carbonic anhydrase 3	NA	-0.15455	1.959039	-1.11307	0.70028368	0.880536
108155	Ogt	O-linked N-acetylglucosamine (GlcNAc) transferase (	NA	0.020366	8.331719	1.014217	0.70043963	0.880662
77582	Mboat7	membrane bound O-acyltransferase domain containi	NA	0.019393	6.098529	1.013533	0.70051696	0.880662
103988	Gck	glucokinase, transcript variant X5	NA	0.068617	2.579993	1.048711	0.70062237	0.880662
11636	Ak1	adenylate kinase 1, transcript variant 3	NA	0.023834	5.998276	1.016658	0.70071026	0.880662
105243563	Gm39452	predicted gene, 39452	NA	0.137715	0.985622	1.100161	0.70072845	0.880662
76491	Abhd14b	abhydrolase domain containing 14b	NA	-0.05058	2.87558	-1.03568	0.70074393	0.880662
218506	Mrps27	mitochondrial ribosomal protein S27	NA	0.032292	4.671576	1.022635	0.70082796	0.880662
320394	Cenpt	centromere protein T, transcript variant X9	NA	0.024739	5.264823	1.017296	0.70086919	0.880662
74354	Lrguk	leucine-rich repeats and guanylate kinase domain co	NA	0.06732	2.139897	1.047768	0.70090072	0.880662
105242624	Gm38785	predicted gene, 38785	NA	-0.13177	1.777159	-1.09563	0.70094075	0.880662
56640	Klk4	kallikrein related-peptidase 4 (prostase, enamel matri	NA	-0.14956	-0.50698	-1.10923	0.70099396	0.880662
20657	Sod3	superoxide dismutase 3, extracellular	NA	0.071461	1.395125	1.05078	0.7010335	0.880662
105244306	Gm39941	predicted gene, 39941	NA	-0.11176	0.907276	-1.08055	0.7011589	0.880747
13195	Ddc	dopa decarboxylase, transcript variant 2	NA	0.037177	3.602065	1.026104	0.70126172	0.880747
19765	Ralbp1	ralA binding protein 1, transcript variant 2	NA	-0.02176	5.991998	-1.0152	0.70130009	0.880747
68112	Entr1	endosome associated trafficking regulator 1, transcrip	NA	0.018773	6.71737	1.013097	0.70134091	0.880747
236792	Mmgt1	membrane magnesium transporter 1	NA	0.022524	6.488821	1.015735	0.70137173	0.880747
19220	Ptgfr	prostaglandin F receptor	NA	0.12446	0.049179	1.0901	0.7014969	0.880836
12798	Cnn2	calponin 2	NA	-0.02977	4.467388	-1.02085	0.70185441	0.881187
227721	Plpp7	phospholipid phosphatase 7 (inactive), transcript vari	NA	0.047147	3.458689	1.033219	0.70196297	0.881187
75678	Ippk	inositol 1,3,4,5,6-pentakisphosphate 2-kinase, transc	NA	0.034704	3.759117	1.024347	0.70202541	0.881187
102634302	Gm27199	predicted gene 27199	NA	-0.05907	2.51111	-1.0418	0.70207673	0.881187
50780	Rgs3	regulator of G-protein signaling 3, transcript variant X	NA	-0.02137	6.369354	-1.01492	0.7021009	0.881187
15247	Mfsd14a	major facilitator superfamily domain containing 14A	NA	0.021228	5.651495	1.014823	0.70211984	0.881187
20841	Zfp143	zinc finger protein 143, transcript variant 2	NA	-0.03205	4.523397	-1.02246	0.70215565	0.881187
69903	Rasip1	Ras interacting protein 1	NA	-0.03097	4.711613	-1.0217	0.70221921	0.881199
67830	Rer1	retention in endoplasmic reticulum sorting receptor 1,	NA	-0.01969	6.315692	-1.01374	0.70261945	0.8816
80796	Calm4	calmodulin 4	NA	-0.38158	-0.29726	-1.30277	0.70264718	0.8816
17850	Mmut	methylmalonyl-Coenzyme A mutase	NA	0.032453	4.265248	1.022749	0.70284606	0.881761
22146	Tuba1c	tubulin, alpha 1C	NA	-0.02043	8.037917	-1.01426	0.70288362	0.881761
66808	9030624G23Rik	RIKEN cDNA 9030624G23 gene, transcript variant 1	NA	-0.05178	2.65997	-1.03654	0.70316274	0.882023
68632	Myct1	myc target 1	NA	-0.09481	0.744023	-1.06793	0.70323827	0.882023
77559	Agl	amylo-1,6-glucosidase, 4-alpha-glucanotransferase, t	NA	0.022799	5.535586	1.015929	0.70331165	0.882023
17117	Amacr	alpha-methylacyl-CoA racemase, transcript variant X	NA	-0.05338	2.627361	-1.0377	0.70344087	0.882023
100642166	Gm15446	predicted gene 15446	NA	-0.09336	0.692971	-1.06685	0.70346514	0.882023
29807	Tpk1	thiamine pyrophosphokinase, transcript variant 1	NA	-0.05001	2.512814	-1.03527	0.70346685	0.882023
330222	Sdk1	sidekick cell adhesion molecule 1, transcript variant X	NA	-0.02816	4.645062	-1.01971	0.70347167	0.882023
14423	Galnt1	polypeptide N-acetylgalactosaminyltransferase 1, tra	NA	0.022406	6.311478	1.015652	0.70356236	0.882064
13528	Dtnb	dystrobrevin, beta, transcript variant X20	NA	-0.02652	5.07434	-1.01855	0.70361309	0.882064
21379	Tbrg4	transforming growth factor beta regulated gene 4, tra	NA	-0.02584	5.279747	-1.01807	0.70373083	0.882067
20348	Sema3c	sema domain, immunoglobulin domain (Ig), short bas	NA	-0.02484	5.400173	-1.01736	0.70375292	0.882067
75033	Mei4	meiotic double-stranded break formation protein 4, tr	NA	0.128878	0.344759	1.093443	0.70380008	0.882067
22378	Wbp2	WW domain binding protein 2, transcript variant 1	NA	0.017755	6.983685	1.012383	0.7038322	0.882067
98736	1700034H15Rik	RIKEN cDNA 1700034H15 gene	NA	-0.08477	0.976716	-1.06052	0.7039866	0.882166
56315	Rhcg	Rhesus blood group-associated C glycoprotein, trans	NA	0.087404	1.081399	1.062457	0.70401946	0.882166

321019	Gpr183	G protein-coupled receptor 183, transcript variant X1	NA	-0.15	-0.47737	-1.10957	0.70421602	0.882194
102636766	Gm33747	predicted gene, 33747, transcript variant X1	NA	-0.10527	0.162089	-1.0757	0.70423379	0.882194
67753	Eqtn	equatorin, sperm acrosome associated, transcript var	NA	-0.15381	-0.28425	-1.11251	0.70423794	0.882194
68165	Fdx2	ferredoxin 2, transcript variant 1	NA	-0.03744	3.8161	-1.02629	0.70429294	0.882194
15519	Hsp90aa1	heat shock protein 90, alpha (cytosolic), class A mem	NA	0.017265	10.067	1.012039	0.70431284	0.882194
18583	Pde7a	phosphodiesterase 7A, transcript variant 1	NA	0.01969	6.649557	1.013742	0.70442809	0.88227
216974	Proca1	protein interacting with cyclin A1, transcript variant X	NA	-0.09831	0.459732	-1.07052	0.70456128	0.882369
12491	Cd36	CD36 molecule, transcript variant X1	NA	-0.05184	2.218614	-1.03659	0.70463078	0.882387
67475	Ero1b	endoplasmic reticulum oxidoreductase 1 beta	NA	-0.03481	3.846306	-1.02442	0.70468444	0.882387
74320	Wdr33	WD repeat domain 33, transcript variant X1	NA	-0.02315	6.620293	-1.01617	0.7048066	0.882436
381812	Cracr2a	calcium release activated channel regulator 2A, trans	NA	-0.11752	0.223478	-1.08487	0.70483182	0.882436
68947	Chst8	carbohydrate sulfotransferase 8	NA	0.065292	2.811671	1.046297	0.70492703	0.882448
12616	Cenpb	centromere protein B	NA	-0.01857	7.284566	-1.01295	0.7049496	0.882448
219148	Fam167a	family with sequence similarity 167, member A, transc	NA	-0.0309	4.734839	-1.02165	0.7050485	0.882504
333329	Cngb1	cyclic nucleotide gated channel beta 1, transcript vari	NA	0.144955	-0.21912	1.105696	0.70513494	0.882542
217011	Nle1	notchless homolog 1	NA	-0.03382	3.761853	-1.02372	0.70518712	0.882542
56364	Zmym3	zinc finger, MYM-type 3, transcript variant X24	NA	-0.0168	7.390855	-1.01171	0.70526084	0.882565
227525	Dclre1c	DNA cross-link repair 1C, transcript variant X9	NA	0.032926	4.151371	1.023085	0.70536832	0.882565
19248	Ptpn12	protein tyrosine phosphatase, non-receptor type 12, t	NA	0.02168	6.363052	1.015141	0.70536849	0.882565
67245	Peli1	pellino 1	NA	-0.02499	6.783169	-1.01747	0.70562914	0.882823
19229	Ptk2b	PTK2 protein tyrosine kinase 2 beta, transcript varian	NA	0.059757	2.327486	1.042291	0.7057861	0.882845
102640673	Gm5165	predicted gene 5165	NA	-0.05102	2.764991	-1.036	0.70580691	0.882845
67792	Rgs8	regulator of G-protein signaling 8, transcript variant X	NA	-0.01971	6.373251	-1.01376	0.70580907	0.882845
94112	Med15	mediator complex subunit 15, transcript variant X8	NA	0.02008	5.813737	1.014016	0.70600923	0.883027
66060	Cystm1	cysteine-rich transmembrane module containing 1, tra	NA	0.041776	3.108665	1.02938	0.70622715	0.883232
76453	Prss23	protease, serine 23, transcript variant X2	NA	-0.04095	3.29281	-1.02879	0.70669729	0.883752
329252	Lgr6	leucine-rich repeat-containing G protein-coupled rece	NA	-0.06924	1.589544	-1.04917	0.70685972	0.88379
19671	Rce1	Ras converting CAAX endopeptidase 1, transcript var	NA	0.035384	3.829364	1.02483	0.70690842	0.88379
18176	Nras	neuroblastoma ras oncogene, transcript variant X4	NA	-0.01995	7.280449	-1.01393	0.70695062	0.88379
71660	Rarres2	retinoic acid receptor responder (tazarotene induced)	NA	0.086264	0.744506	1.061617	0.70698114	0.88379
69178	Snx5	sorting nexin 5, transcript variant 2	NA	0.019019	6.09275	1.013271	0.70700432	0.88379
14421	B4galnt1	beta-1,4-N-acetyl-galactosaminyl transferase 1, trans	NA	-0.01984	6.262038	-1.01385	0.70709123	0.88379
19303	Pxn	paxillin, transcript variant alpha	NA	-0.02225	5.699373	-1.01554	0.70710824	0.88379
170835	Inpp5j	inositol polyphosphate 5-phosphatase J	NA	-0.04245	2.95572	-1.02986	0.70729754	0.883926
17125	Smad1	SMAD family member 1	NA	-0.01917	6.156983	-1.01338	0.7073252	0.883926
71449	Eef1aknmt	EEF1A lysine and N-terminal methyltransferase, trans	NA	0.037761	3.951063	1.02652	0.70767852	0.884076
21892	Tll1	tolloid-like, transcript variant X3	NA	-0.04616	2.746761	-1.03251	0.70775818	0.884076
229534	Pbxip1	pre B cell leukemia transcription factor interacting pro	NA	-0.03338	4.725708	-1.02341	0.70780632	0.884076
13058	Cybb	cytochrome b-245, beta polypeptide, transcript varian	NA	-0.1285	-0.44855	-1.09316	0.70782946	0.884076
269061	Cpsf7	cleavage and polyadenylation specific factor 7, transc	NA	-0.01997	7.330712	-1.01394	0.70785387	0.884076
544864	Gm5785	predicted gene 5785	NA	-0.0734	1.869092	-1.05219	0.70786971	0.884076
16522	Kcnj6	potassium inwardly-rectifying channel, subfamily J, m	NA	-0.02527	4.945442	-1.01767	0.70787913	0.884076
69683	Emc10	ER membrane protein complex subunit 10, transcript	NA	0.023107	6.52815	1.016145	0.70797526	0.884076
66620	Pantr2	POU domain, class 3, transcription factor 3 adjacent	NA	0.139614	-0.24011	1.101611	0.70800567	0.884076
53859	Map3k14	mitogen-activated protein kinase kinase kinase 14	NA	-0.04979	2.722009	-1.03511	0.70803634	0.884076
211484	Tsga10	testis specific 10, transcript variant 1	NA	-0.0437	3.356537	-1.03076	0.70807363	0.884076
70359	Gtpbp3	GTP binding protein 3, transcript variant 5	NA	0.037325	3.720143	1.026209	0.70809747	0.884076
225471	Ticam2	toll-like receptor adaptor molecule 2	NA	-0.11794	-0.08591	-1.08519	0.7085049	0.884391
108767	Pnrc1	proline-rich nuclear receptor coactivator 1	NA	-0.02836	6.01055	-1.01985	0.70856922	0.884391
11991	Hnrnpd	heterogeneous nuclear ribonucleoprotein D, transcrip	NA	0.019726	8.83494	1.013767	0.70862787	0.884391
17533	Mrc1	mannose receptor, C type 1	NA	0.05501	4.214892	1.038867	0.70864848	0.884391
194268	9930104L06Rik	RIKEN cDNA 9930104L06 gene, transcript variant 1	NA	-0.02978	4.296224	-1.02085	0.70866165	0.884391
237504	Rassf9	Ras association (RalGDS/AF-6) domain family (N-ter	NA	0.051073	2.275687	1.036035	0.70874135	0.884391
170441	Slc2a10	solute carrier family 2 (facilitated glucose transporter)	NA	0.062107	1.609925	1.043989	0.70879647	0.884391
574405	DXBay18	DNA segment, Chr X, Baylor 18	NA	0.245856	0.209835	1.185796	0.70884561	0.884391
115489534	Gm52565	predicted gene, 52565	NA	-0.12707	0.057359	-1.09208	0.70890557	0.884391
108112	Eif4ebp3	eukaryotic translation initiation factor 4E binding prote	NA	0.039795	3.651924	1.027968	0.70890582	0.884391
72865	Rtl8c	retrotransposon Gag like 8C	NA	0.024871	6.154661	1.017388	0.70897831	0.884391
68611	Mrpl28	mitochondrial ribosomal protein L28	NA	-0.02646	5.116034	-1.01851	0.70905586	0.884391
20619	Snap23	synaptosomal-associated protein 23, transcript variar	NA	-0.031	4.204474	-1.02172	0.70905632	0.884391
74102	Slc35a5	solute carrier family 35, member A5, transcript varian	NA	-0.02635	5.136067	-1.01843	0.70914908	0.884439
66722	Spag16	sperm associated antigen 16, transcript variant 3	NA	-0.09867	1.165754	-1.07079	0.70947023	0.884772
13139	Dgka	diacylglycerol kinase, alpha, transcript variant X3	NA	-0.04048	3.33083	-1.02845	0.7095774	0.884837
14155	Fem1b	fem 1 homolog b	NA	-0.01864	8.173572	-1.01301	0.70969047	0.884879
272031	Plppr1	phospholipid phosphatase related 1	NA	0.021712	6.443938	1.015164	0.70971931	0.884879
66943	Slc66a2	solute carrier family 66 member 2, transcript variant 3	NA	-0.02956	4.610906	-1.0207	0.70992404	0.884983
14600	Ghr	growth hormone receptor, transcript variant 4	NA	0.036888	3.657081	1.025899	0.70994739	0.884983
54199	Ccrl2	chemokine (C-C motif) receptor-like 2, transcript varia	NA	-0.12523	-0.31073	-1.09068	0.7100274	0.884983

66460	Sys1	SYS1 Golgi-localized integral membrane protein hom	NA	0.030452	4.516438	1.021332	0.71003035	0.884983
100040792	Gm11627	predicted gene 11627	NA	-0.084	0.993348	-1.05995	0.71007496	0.884983
399510	Map4k5	mitogen-activated protein kinase kinase kinase kinas	NA	0.027249	6.074093	1.019067	0.71030237	0.885034
207740	Ubald1	UBA-like domain containing 1	NA	0.023366	5.335317	1.016328	0.71031595	0.885034
108168793	Gm46764	predicted gene, 46764, transcript variant 1	NA	0.095853	0.537563	1.068697	0.71031663	0.885034
20103	Rps5	ribosomal protein S5	NA	0.021646	8.475313	1.015117	0.71033335	0.885034
20810	Srm	spermidine synthase	NA	-0.02146	5.670113	-1.01498	0.71040983	0.885062
23991	Cib1	calcium and integrin binding 1 (calmyrin), transcript v	NA	-0.04963	2.943307	-1.035	0.71063588	0.885204
108169155	Gm46967	predicted gene, 46967, transcript variant X2	NA	-0.09395	1.292509	-1.06729	0.71066295	0.885204
17921	Myo7a	myosin VIIA, transcript variant X4	NA	-0.02234	5.231824	-1.01561	0.71068778	0.885204
171281	Acot3	acyl-CoA thioesterase 3, transcript variant 1	NA	0.131589	0.148948	1.0955	0.71074519	0.885208
53872	Caprin1	cell cycle associated protein 1, transcript variant X1	NA	-0.01793	9.152063	-1.01251	0.71092513	0.885276
319974	Auts2	autism susceptibility candidate 2	NA	0.019347	7.941792	1.013501	0.71093479	0.885276
20776	Tmie	transmembrane inner ear, transcript variant X6	NA	-0.07157	1.993958	-1.05086	0.71099297	0.885276
14865	Gstm4	glutathione S-transferase, mu 4, transcript variant 1	NA	0.047447	3.134533	1.033435	0.71101733	0.885276
20643	Snrpe	small nuclear ribonucleoprotein E	NA	0.021497	6.225623	1.015012	0.71113015	0.885297
105244574	Gm40167	predicted gene, 40167	NA	-0.0943	0.302131	-1.06755	0.71114288	0.885297
74191	P2ry13	purinergic receptor P2Y, G-protein coupled 13	NA	-0.07696	0.993442	-1.05479	0.71131363	0.885323
71817	Tmem50a	transmembrane protein 50A, transcript variant X1	NA	0.023075	6.044966	1.016123	0.71139093	0.885323
109857	Cbr3	carbonyl reductase 3	NA	-0.1182	-0.19377	-1.08538	0.71144912	0.885323
30794	Pdlim4	PDZ and LIM domain 4	NA	-0.04738	3.028186	-1.03339	0.71154007	0.885323
270669	Mbtps2	membrane-bound transcription factor peptidase, site	NA	0.022227	5.70415	1.015526	0.71160053	0.885323
22619	Siae	sialic acid acetylesterase	NA	-0.03793	3.434137	-1.02664	0.7116406	0.885323
70951	Spata1	spermatogenesis associated 1, transcript variant 3	NA	-0.07935	1.33231	-1.05655	0.71164777	0.885323
230857	Ece1	endothelin converting enzyme 1, transcript variant 4	NA	0.026869	5.376727	1.018799	0.71177823	0.885323
268534	Sntg2	syntrophin, gamma 2, transcript variant 1	NA	0.082715	0.782618	1.059009	0.71179122	0.885323
106064	AW549877	expressed sequence AW549877	NA	0.02006	6.319455	1.014001	0.71179433	0.885323
102633665	Gm31436	predicted gene, 31436, transcript variant X2	NA	-0.10737	0.104115	-1.07726	0.7118093	0.885323
100043915	Gm4724	predicted gene 4724, transcript variant X12	NA	0.038228	3.968845	1.026852	0.71181682	0.885323
81910	Rrbp1	ribosome binding protein 1, transcript variant X5	NA	-0.02659	6.46745	-1.0186	0.71188619	0.885342
27660	1700088E04Rik	RIKEN cDNA 1700088E04 gene, transcript variant 3	NA	0.060727	2.304636	1.042991	0.71200878	0.885427
19049	Ppp1r1b	protein phosphatase 1, regulatory inhibitor subunit 1E	NA	0.051388	4.033594	1.036262	0.71208077	0.885448
13557	E2f3	E2F transcription factor 3, transcript variant 3	NA	-0.02674	5.073111	-1.01871	0.71229781	0.885651
11932	Atp1b2	ATPase, Na+/K+ transporting, beta 2 polypeptide	NA	-0.02092	7.043388	-1.01461	0.71245749	0.885726
67878	Tmem33	transmembrane protein 33, transcript variant 1	NA	0.020973	6.768048	1.014644	0.71251118	0.885726
107508	Eprs	glutamyl-prolyl-tRNA synthetase, transcript variant 1	NA	0.018052	7.34949	1.012591	0.71254657	0.885726
23796	Aplnr	apelin receptor	NA	-0.03268	4.956099	-1.02291	0.71258368	0.885726
66439	Borcs7	BLOC-1 related complex subunit 7	NA	0.032152	4.187696	1.022536	0.71263042	0.885726
14725	Lrp2	low density lipoprotein receptor-related protein 2	NA	-0.0503	2.818888	-1.03548	0.71284145	0.88592
208098	Panx3	pannexin 3	NA	0.177527	-0.99103	1.130943	0.71291733	0.885929
72160	Tmem163	transmembrane protein 163	NA	0.032659	5.086531	1.022896	0.71298363	0.885929
18483	Palm	paralemmin, transcript variant 2	NA	-0.01592	8.160343	-1.0111	0.71306126	0.885929
105244932	Gm40454	predicted gene, 40454	NA	0.093331	0.619549	1.06683	0.71315266	0.885929
68818	Zfand2b	zinc finger, AN1 type domain 2B, transcript variant 1	NA	0.035279	3.532929	1.024755	0.71318464	0.885929
574428	Zmynd15	zinc finger, MYND-type containing 15, transcript varia	NA	-0.1111	0.494038	-1.08005	0.71318676	0.885929
667118	Zbed6	zinc finger, BED type containing 6	NA	0.100286	2.638092	1.071986	0.71323417	0.885929
102640259	Gm36371	predicted gene, 36371	NA	0.10063	0.281739	1.072242	0.71328393	0.885929
66870	Serbp1	serpine1 mRNA binding protein 1, transcript variant 3	NA	0.015969	8.733844	1.01113	0.71353382	0.886057
20613	Snai1	snail family zinc finger 1	NA	-0.08122	1.360731	-1.05791	0.71353848	0.886057
68642	Tmem216	transmembrane protein 216, transcript variant 2	NA	0.035183	3.508993	1.024687	0.7136275	0.886057
69784	1500009L16Rik	RIKEN cDNA 1500009L16 gene	NA	-0.02864	4.452454	-1.02005	0.71366013	0.886057
223435	Trio	triple functional domain (PTPRF interacting), transcri	NA	-0.01759	7.553705	-1.01227	0.71366129	0.886057
232210	Hmces	5-hydroxymethylcytosine (hmC) binding, ES cell spec	NA	0.027427	4.591751	1.019193	0.71373657	0.886057
12739	Cldn3	claudin 3	NA	0.083161	1.329721	1.059337	0.71386496	0.886057
11423	Ache	acetylcholinesterase, transcript variant 2	NA	0.035029	4.927222	1.024577	0.7138924	0.886057
240672	Dusp5	dual specificity phosphatase 5	NA	-0.09391	1.089551	-1.06726	0.71397464	0.886057
381798	4930590J08Rik	RIKEN cDNA 4930590J08 gene, transcript variant X4	NA	-0.07174	1.240309	-1.05099	0.71404962	0.886057
21683	Tecta	tectorin alpha, transcript variant 2	NA	-0.07989	1.082975	-1.05694	0.71407421	0.886057
78244	Dnajc21	DnaJ heat shock protein family (Hsp40) member C21	NA	0.027022	4.675881	1.018906	0.71410393	0.886057
319651	Usp37	ubiquitin specific peptidase 37, transcript variant 2	NA	-0.02293	5.427737	-1.01602	0.71414892	0.886057
54712	Plxnc1	plexin C1	NA	-0.02019	6.523701	-1.0141	0.7141496	0.886057
72480	Tspyl4	TSPY-like 4	NA	0.01845	8.288799	1.012871	0.71447891	0.886398
109082	Fbxw17	F-box and WD-40 domain protein 17, transcript varia	NA	-0.03292	4.074651	-1.02308	0.71458511	0.886448
12425	Cckar	cholecystokinin A receptor, transcript variant 1	NA	-0.11064	0.120375	-1.07971	0.71462763	0.886448
224613	Flywch1	FLYWCH-type zinc finger 1, transcript variant 2	NA	-0.01818	6.838239	-1.01268	0.71472252	0.886498
108167550	Gm38250	predicted gene, 38250, transcript variant 4	NA	-0.07304	1.656503	-1.05193	0.71494978	0.886652
68058	Chd1l	chromodomain helicase DNA binding protein 1-like	NA	-0.04124	3.107935	-1.029	0.71495559	0.886652
100503468	Gm14023	predicted gene 14023	NA	-0.11166	0.097509	-1.08047	0.71504763	0.886698

80982	Cemip	cell migration inducing protein, hyaluronan binding	NA	-0.05328	3.051341	-1.03762	0.71514674	0.886754
52713	Ccdc59	coiled-coil domain containing 59	NA	0.032811	4.337883	1.023003	0.71550946	0.886919
234796	Klhl36	kelch-like 36	NA	0.04838	2.669454	1.034103	0.71553742	0.886919
20115	Rps7	ribosomal protein S7	NA	0.017997	9.251222	1.012553	0.7155415	0.886919
105246363	Gm41662	predicted gene, 41662	NA	-0.05671	2.12747	-1.04009	0.71557112	0.886919
223827	Gxylt1	glucoside xylosyltransferase 1, transcript variant 3	NA	-0.02859	4.87401	-1.02002	0.71559647	0.886919
14172	Fgf18	fibroblast growth factor 18, transcript variant 1	NA	0.049733	2.371844	1.035073	0.71560705	0.886919
67434	Ankrd33b	ankyrin repeat domain 33B, transcript variant 4	NA	-0.1185	0.088344	-1.08561	0.71568911	0.886953
102633625	Gm31409	predicted gene, 31409, transcript variant X7	NA	0.198086	-0.70493	1.147175	0.71592212	0.887131
72046	Urgcp	upregulator of cell proliferation, transcript variant X4	NA	-0.02697	5.354468	-1.01887	0.71594156	0.887131
23972	Papss2	3'-phosphoadenosine 5'-phosphosulfate synthase 2, t	NA	0.101143	1.91971	1.072623	0.71603191	0.887175
115489888	Gm52666	predicted gene, 52666, transcript variant X1	NA	-0.44766	1.27479	-1.36382	0.71625522	0.887222
228576	Mall	mal, T cell differentiation protein-like	NA	-0.10346	0.94124	-1.07434	0.71626772	0.887222
100504703	A730063M14Rik	RIKEN cDNA A730063M14 gene	NA	-0.087	0.571137	-1.06216	0.71628973	0.887222
118568404	LOC118568404	uncharacterized LOC118568404	NA	-0.10631	0.163317	-1.07647	0.71631417	0.887222
118568345	LOC118568345	igE-binding protein-like	NA	-0.09639	1.092208	-1.06909	0.71634251	0.887222
53312	Nub1	negative regulator of ubiquitin-like proteins 1, transcri	NA	-0.02158	6.696295	-1.01507	0.71650843	0.887285
114602	Zmynd10	zinc finger, MYND domain containing 10, transcript va	NA	-0.06221	1.779606	-1.04406	0.71665055	0.887285
72701	Zfp618	zinc finger protein 618, transcript variant X23	NA	0.020554	6.019114	1.014349	0.71665634	0.887285
15399	Hoxa2	homeobox A2	NA	-0.09531	2.245769	-1.06829	0.7166587	0.887285
20687	Sp3	trans-acting transcription factor 3, transcript variant 2	NA	-0.03082	6.591841	-1.0216	0.71666583	0.887285
14706	Gng4	guanine nucleotide binding protein (G protein), gamm	NA	0.024139	6.210197	1.016873	0.71672238	0.887287
97884	B3galnt2	UDP-GalNAc:betaGlcNAc beta 1,3-galactosaminyltra	NA	0.032546	4.900375	1.022815	0.71695843	0.887512
69752	Zfp511	zinc finger protein 511	NA	-0.02831	4.448849	-1.01982	0.71702717	0.88753
18012	Neurod1	neurogenic differentiation 1	NA	0.027694	5.616664	1.019381	0.71716156	0.887629
226654	Tstd1	thiosulfate sulfurtransferase (rhodanese)-like domain	NA	-0.13396	-0.28695	-1.09731	0.71727901	0.887706
11950	Atp5pb	ATP synthase peripheral stalk-membrane subunit b, t	NA	0.023191	8.026942	1.016205	0.71740677	0.887797
27057	Ncoa4	nuclear receptor coactivator 4, transcript variant X1	NA	0.019982	6.798294	1.013947	0.71801743	0.888485
29857	Mapk12	mitogen-activated protein kinase 12, transcript varian	NA	-0.06142	2.55295	-1.04349	0.71827022	0.88868
237858	Trarg1	trafficking regulator of GLUT4 (SLC2A4) 1	NA	0.104825	0.03601	1.075364	0.71833028	0.88868
71683	Gypc	glycophorin C, transcript variant 1	NA	-0.04438	3.092182	-1.03124	0.71835005	0.88868
56484	Foxo3	forkhead box O3, transcript variant 1	NA	0.023161	5.634367	1.016184	0.71845508	0.88868
68867	Rnf122	ring finger protein 122, transcript variant 3	NA	-0.02619	5.159437	-1.01832	0.71850109	0.88868
118567995	LOC118567995	uncharacterized LOC118567995	NA	-0.07625	1.332622	-1.05427	0.71850227	0.88868
67068	Dynlrb1	dynein light chain roadblock-type 1, transcript variant	NA	0.019874	7.201584	1.013871	0.71874055	0.888882
66913	Kdelr2	KDEL (Lys-Asp-Glu-Leu) endoplasmic reticulum prote	NA	0.022505	5.831885	1.015722	0.71879557	0.888882
72185	Dbndd1	dysbindin (dystrobrevin binding protein 1) domain cor	NA	-0.02051	5.583664	-1.01432	0.71882954	0.888882
552913	Gm14634	predicted gene 14634	NA	0.067275	1.636021	1.047736	0.71889572	0.888896
18111	Nnat	neuronatin, transcript variant 2	NA	0.020741	11.22737	1.01448	0.71897415	0.888925
73046	Glrx5	glutaredoxin 5	NA	0.034383	5.133158	1.024119	0.71904906	0.88895
12669	Chrm1	cholinergic receptor, muscarinic 1, CNS, transcript va	NA	-0.04767	2.973079	-1.0336	0.71914335	0.888971
53621	Cnot4	CCR4-NOT transcription complex, subunit 4, transcri	NA	-0.02282	5.83125	-1.01594	0.71917519	0.888971
239027	Arhgap22	Rho GTPase activating protein 22, transcript variant)	NA	-0.08552	0.610749	-1.06107	0.71944531	0.88922
266614	Ly6g5b	lymphocyte antigen 6 complex, locus G5B, transcript	NA	0.06459	1.898888	1.045788	0.71948594	0.88922
18103	Nme2	NME/NM23 nucleoside diphosphate kinase 2, transcr	NA	0.028222	7.169753	1.019755	0.71955436	0.889237
66455	Cnpy4	canopy FGF signaling regulator 4	NA	-0.03183	4.410804	-1.02231	0.7196205	0.889252
22652	Mkrn3	makorin, ring finger protein, 3	NA	-0.03698	4.14017	-1.02596	0.71989938	0.889529
224624	Rab40c	Rab40C, member RAS oncogene family	NA	0.021903	5.51257	1.015297	0.71999367	0.889578
101142	Itfg2	integrin alpha FG-GAP repeat containing 2	NA	-0.02694	4.633173	-1.01885	0.72017074	0.889729
80744	Cwc22	CWC22 spliceosome-associated protein, transcript va	NA	-0.02341	5.132183	-1.01636	0.72032751	0.889799
66251	Arfgap3	ADP-ribosylation factor GTPase activating protein 3,	NA	-0.02384	5.030198	-1.01666	0.72033666	0.889799
27366	Txnl4a	thioredoxin-like 4A, transcript variant 3	NA	0.016363	6.888994	1.011407	0.72101539	0.890569
78541	Asb8	ankyrin repeat and SOCS box-containing 8, transcrip	NA	-0.02551	5.062796	-1.01784	0.7211087	0.890617
69020	Zfp707	zinc finger protein 707, transcript variant X1	NA	-0.03667	3.507093	-1.02574	0.72124974	0.890638
12660	Chka	choline kinase alpha, transcript variant 3	NA	0.018108	6.014972	1.012631	0.7212663	0.890638
234577	Cpne2	copine II	NA	-0.02303	4.862065	-1.01609	0.72128994	0.890638
108168395	Gm45871	predicted gene 45871	NA	0.036205	3.334891	1.025413	0.72140551	0.890713
268490	Lsm12	LSM12 homolog, transcript variant X1	NA	-0.02274	6.347256	-1.01588	0.72153683	0.890808
246086	Onecut3	one cut domain, family member 3	NA	0.045662	3.020962	1.032157	0.7217259	0.890898
16822	Lcp2	lymphocyte cytosolic protein 2	NA	-0.06339	1.73058	-1.04491	0.72176537	0.890898
13982	Esr1	estrogen receptor 1 (alpha), transcript variant 2	NA	0.106438	-0.1383	1.076567	0.72177396	0.890898
57773	Wdr4	WD repeat domain 4, transcript variant X9	NA	-0.0271	5.308789	-1.01896	0.7220256	0.891141
66468	Ska1	spindle and kinetochore associated complex subunit	NA	-0.05858	2.326507	-1.04144	0.72232345	0.891441
68789	Trmt61b	tRNA methyltransferase 61B, transcript variant 2	NA	0.051192	2.832387	1.036121	0.7226155	0.891627
66958	Tmx2	thioredoxin-related transmembrane protein 2, transcri	NA	0.017838	6.91905	1.012441	0.72262198	0.891627
15186	Hdc	histidine decarboxylase	NA	0.143189	1.621277	1.104343	0.72276439	0.891627
66597	Trim13	tripartite motif-containing 13, transcript variant 1	NA	-0.03304	5.012793	-1.02317	0.72279264	0.891627
115490393	Gm52889	predicted gene, 52889	NA	-0.0819	1.241354	-1.05841	0.72287211	0.891627

18245	Oaz1	ornithine decarboxylase antizyme 1, transcript varian	NA	0.019503	7.655246	1.01361	0.72298572	0.891627
66286	Sec11c	SEC11 homolog C, signal peptidase complex subuni	NA	0.032646	4.387209	1.022887	0.72300479	0.891627
215814	Ccdc28a	coiled-coil domain containing 28A, transcript variant 2	NA	0.056912	1.868652	1.040237	0.72302778	0.891627
209497	Tmem164	transmembrane protein 164, transcript variant X7	NA	-0.02257	5.688799	-1.01577	0.72306296	0.891627
110082	Dnah5	dynein, axonemal, heavy chain 5	NA	-0.07786	1.261813	-1.05545	0.7231322	0.891627
77110	Gpbp1l1	GC-rich promoter binding protein 1-like 1, transcript v	NA	0.021939	5.361611	1.015323	0.72313572	0.891627
66480	Rpl15	ribosomal protein L15, transcript variant 1	NA	-0.01552	9.24594	-1.01081	0.7231731	0.891627
229004	Gmeb2	glucocorticoid modulatory element binding protein 2,	NA	0.024072	5.242873	1.016826	0.72318642	0.891627
102638384	Gm34963	predicted gene, 34963	NA	0.101916	0.052049	1.073197	0.72329382	0.891692
78317	Ccdc88b	coiled-coil domain containing 88B	NA	-0.10526	-0.04657	-1.07569	0.72335002	0.891693
72029	Cnpy3	canopy FGF signaling regulator 3, transcript variant 1	NA	-0.02493	5.024866	-1.01743	0.72392794	0.892281
78893	Cnot10	CCR4-NOT transcription complex, subunit 10, transc	NA	0.019658	5.99559	1.013719	0.72393682	0.892281
117592	B3galt6	UDP-Gal:betaGal beta 1,3-galactosyltransferase, pol	NA	-0.02713	4.583948	-1.01898	0.72406935	0.892377
76877	Rab36	RAB36, member RAS oncogene family, transcript va	NA	0.032259	4.296372	1.022612	0.72431954	0.892488
67689	Aldh3b1	aldehyde dehydrogenase 3 family, member B1	NA	0.094098	0.399464	1.067398	0.72432082	0.892488
11923	Neurod4	neurogenic differentiation 4, transcript variant 3	NA	-0.06156	2.025296	-1.04359	0.72432418	0.892488
69961	Rpp25l	ribonuclease P/MRP 25 subunit-like	NA	0.030584	3.752996	1.021426	0.72444851	0.892548
192199	Rspo1	R-spondin 1	NA	-0.065	1.705441	-1.04608	0.72448188	0.892548
67220	Plekho1	pleckstrin homology domain containing, family O mer	NA	-0.0221	6.162802	-1.01544	0.72463299	0.892588
108169152	Gm46965	predicted gene, 46965, transcript variant X3	NA	0.107601	0.937952	1.077435	0.7246439	0.892588
14055	Ezh1	enhancer of zeste 1 polycomb repressive complex 2	NA	0.024214	4.849236	1.016926	0.72467913	0.892588
66832	Rsph3a	radial spoke 3A homolog (Chlamydomonas)	NA	-0.02476	4.5237	-1.01731	0.72491213	0.892649
105243499	Gm39401	predicted gene, 39401	NA	-0.11405	-0.05865	-1.08226	0.72492514	0.892649
237758	Zfp454	zinc finger protein 454, transcript variant 9	NA	-0.03313	3.757982	-1.02323	0.7249368	0.892649
544971	Bdp1	B double prime 1, subunit of RNA polymerase III tran	NA	-0.02284	5.258203	-1.01596	0.7249482	0.892649
338320	Mia2	MIA SH3 domain ER export factor 2, transcript varian	NA	-0.03128	5.181667	-1.02192	0.7250632	0.892707
27973	Vkorc1	vitamin K epoxide reductase complex, subunit 1	NA	-0.04386	3.496226	-1.03087	0.72523369	0.892707
108168731	LOC108168731	uncharacterized LOC108168731	NA	0.036695	5.210587	1.025761	0.72523999	0.892707
54396	Irgm2	immunity-related GTPase family M member 2	NA	0.092262	0.889175	1.066041	0.72529349	0.892707
21452	Tcn2	transcobalamin 2, transcript variant X3	NA	-0.03002	3.906196	-1.02103	0.72538659	0.892707
228889	Ddx27	DEAD box helicase 27	NA	0.024484	5.205281	1.017116	0.72542115	0.892707
68201	Ccdc34	coiled-coil domain containing 34, transcript variant 1	NA	0.022679	5.06471	1.015844	0.7254228	0.892707
71973	Rbpms2	RNA binding protein with multiple splicing 2, transcrip	NA	-0.05897	1.808255	-1.04172	0.72545649	0.892707
224045	Eif2b5	eukaryotic translation initiation factor 2B, subunit 5 e	NA	-0.02025	6.145509	-1.01414	0.72552854	0.892707
29873	Cspg5	chondroitin sulfate proteoglycan 5, transcript variant	NA	0.017516	7.23129	1.012215	0.72554419	0.892707
13417	Dnah8	dynein, axonemal, heavy chain 8, transcript variant X	NA	-0.10122	1.192998	-1.07268	0.72565574	0.892777
75560	Ep400	E1A binding protein p400, transcript variant 2	NA	0.018343	7.097266	1.012796	0.72572021	0.892789
211798	Mfsd9	major facilitator superfamily domain containing 9	NA	-0.04133	2.948028	-1.02906	0.72601439	0.893083
192651	Zfp286	zinc finger protein 286, transcript variant X5	NA	-0.03804	4.736264	-1.02672	0.72611335	0.893138
110954	Rpl10	ribosomal protein L10	NA	-0.01496	9.267813	-1.01042	0.72623651	0.893222
16572	Kif5a	kinesin family member 5A, transcript variant 1	NA	0.018605	8.299701	1.012979	0.72650848	0.893434
16948	Lox	lysyl oxidase, transcript variant 3	NA	-0.02944	4.618083	-1.02062	0.72651948	0.893434
94281	Sfxn4	sideroflexin 4, transcript variant X2	NA	-0.0535	2.929801	-1.03778	0.7267617	0.893649
16399	Itga2b	integrin alpha 2b	NA	-0.0828	1.025691	-1.05907	0.72680379	0.893649
19650	Rbl1	RB transcriptional corepressor like 1, transcript varia	NA	-0.036	4.241116	-1.02527	0.72693134	0.893738
18806	Pld2	phospholipase D2, transcript variant 4	NA	0.036028	4.38365	1.025287	0.72703466	0.893798
623131	Prr19	proline rich 19	NA	0.089329	0.622264	1.063875	0.72711208	0.893825
77048	Cep83	centrosomal protein 83	NA	-0.02684	4.979717	-1.01878	0.72735752	0.893932
208194	Exog	endo/exonuclease (5'-3'), endonuclease G-like, trans	NA	-0.0219	5.376234	-1.0153	0.72739536	0.893932
115487947	Gm51955	predicted gene, 51955, transcript variant X1	NA	0.127219	-0.15657	1.092186	0.72741681	0.893932
54636	Wdr45	WD repeat domain 45, transcript variant 3	NA	-0.02724	4.238422	-1.01906	0.7274183	0.893932
227120	Plcl1	phospholipase C-like 1	NA	0.026066	4.598554	1.018232	0.7276773	0.894183
408062	Zfp873	zinc finger protein 873, transcript variant X4	NA	-0.03026	4.073662	-1.0212	0.72777329	0.894233
102638997	Gm35422	predicted gene, 35422, transcript variant X1	NA	-0.11081	0.202289	-1.07983	0.72788889	0.894307
71974	Prmt3	protein arginine N-methyltransferase 3	NA	-0.02595	5.030255	-1.01815	0.72796263	0.894331
12895	Cpt1b	carnitine palmitoyltransferase 1b, muscle	NA	0.045642	2.914901	1.032142	0.7280507	0.894339
52708	Zfp410	zinc finger protein 410, transcript variant X1	NA	0.02085	5.55519	1.014557	0.72810649	0.894339
216705	Clint1	clathrin interactor 1, transcript variant 1	NA	-0.02166	6.100703	-1.01513	0.72813468	0.894339
100502736	Gm19345	predicted gene, 19345	NA	-0.05279	2.130548	-1.03727	0.72825751	0.894423
27387	Sh2d3c	SH2 domain containing 3C, transcript variant 2	NA	-0.02641	4.893768	-1.01848	0.72835908	0.894462
94315	Prcc	papillary renal cell carcinoma (translocation-associat	NA	-0.01991	6.397097	-1.0139	0.72839931	0.894462
58988	Rps6kb2	ribosomal protein S6 kinase, polypeptide 2, transcript	NA	0.019914	5.398899	1.013899	0.72850618	0.894467
228961	Npepl1	aminopeptidase-like 1, transcript variant 2	NA	-0.03065	4.464552	-1.02147	0.72851309	0.894467
55978	Ift20	intraflagellar transport 20	NA	0.025063	4.921456	1.017524	0.7286992	0.894628
14629	Gclc	glutamate-cysteine ligase, catalytic subunit, transcrip	NA	0.023402	5.264194	1.016353	0.72891498	0.894825
16570	Kif3c	kinesin family member 3C	NA	0.016252	7.976313	1.011328	0.72897474	0.894831
20088	Rps24	ribosomal protein S24, transcript variant X1	NA	0.02125	9.212159	1.014838	0.72908119	0.89485
108654	Fam210a	family with sequence similarity 210, member A	NA	0.023123	4.986024	1.016157	0.72910056	0.89485

13711	Elf5	E74-like factor 5, transcript variant 2	NA	-0.1748	-0.63241	-1.12881	0.72928504	0.894951
105244835	Gm40371	predicted gene, 40371, transcript variant X1	NA	0.117987	0.364797	1.085219	0.72931538	0.894951
13115	Cyp27b1	cytochrome P450, family 27, subfamily b, polypeptide	NA	-0.11785	-0.08832	-1.08512	0.72934803	0.894951
21825	Thbs1	thrombospondin 1, transcript variant 2	NA	0.025932	4.709554	1.018137	0.72946235	0.894965
118568005	LOC118568005	uncharacterized LOC118568005	NA	0.104279	0.017292	1.074957	0.72947853	0.894965
234362	Zfp868	zinc finger protein 868, transcript variant 1	NA	0.028152	4.975879	1.019705	0.72956029	0.894965
69666	Psmg4	proteasome (prosome, macropain) assembly chapero	NA	0.043201	3.035156	1.030398	0.72961592	0.894965
56150	Mad2l1	MAD2 mitotic arrest deficient-like 1, transcript variant	NA	-0.02986	4.501857	-1.02091	0.72963412	0.894965
104732	Tedc1	tubulin epsilon and delta complex 1	NA	-0.03327	3.610518	-1.02333	0.72969818	0.894976
14924	Magi1	membrane associated guanylate kinase, WW and PD	NA	0.018629	7.427745	1.012996	0.73019976	0.895411
22139	Ttr	transthyretin, transcript variant X1	NA	0.026742	9.153418	1.018709	0.73021557	0.895411
227613	Tubb4b	tubulin, beta 4B class IVB	NA	-0.01638	7.511628	-1.01142	0.73021779	0.895411
14587	Gfra3	glial cell line derived neurotrophic factor family recept	NA	0.121018	-0.62101	1.087502	0.73054331	0.895671
66541	Immp1l	IMP1 inner mitochondrial membrane peptidase-like (S	NA	0.02711	4.57845	1.018969	0.73055697	0.895671
320438	Alg6	asparagine-linked glycosylation 6 (alpha-1,3,-glucosy	NA	0.044444	3.614733	1.031286	0.73059534	0.895671
27050	Rps3	ribosomal protein S3	NA	0.017572	8.828104	1.012255	0.73068449	0.895713
67775	Rtp4	receptor transporter protein 4, transcript variant X1	NA	0.095667	1.137759	1.068559	0.73088509	0.895832
66701	Spryd4	SPRY domain containing 4	NA	0.038715	3.253639	1.027198	0.73094537	0.895832
100040944	Gm3055	predicted gene 3055, transcript variant 2	NA	-0.03852	4.055339	-1.02706	0.73094682	0.895832
383563	Gpr25	G protein-coupled receptor 25	NA	0.048326	2.435968	1.034065	0.73101364	0.895847
224833	AI661453	expressed sequence AI661453	NA	-0.08328	1.237554	-1.05943	0.73121433	0.895962
13589	Mapre1	microtubule-associated protein, RP/EB family, memb	NA	-0.02763	8.476985	-1.01933	0.73123591	0.895962
97440	B3gnt9	UDP-GlcNAc:betaGal beta-1,3-N-acetylglucosaminylt	NA	-0.05767	2.380216	-1.04078	0.73132055	0.895962
28077	Med10	mediator complex subunit 10, transcript variant 1	NA	0.0234	5.409779	1.016352	0.73136997	0.895962
14349	Fv1	Friend virus susceptibility 1	NA	-0.07229	1.938402	-1.05139	0.73138255	0.895962
20532	Slc3a1	solute carrier family 3, member 1	NA	0.109025	-0.15296	1.078499	0.73152804	0.896062
68420	Ankrd13a	ankyrin repeat domain 13a, transcript variant 1	NA	-0.0236	6.047872	-1.01649	0.73158058	0.896062
14451	Gas1	growth arrest specific 1	NA	-0.03345	5.183461	-1.02346	0.73170241	0.896062
214162	Kmt2a	lysine (K)-specific methyltransferase 2A, transcript va	NA	-0.01878	7.39549	-1.0131	0.73174021	0.896062
116748	Lsm10	U7 snRNP-specific Sm-like protein LSM10, transcript	NA	-0.03254	3.487749	-1.02281	0.73176588	0.896062
66052	Sdhc	succinate dehydrogenase complex, subunit C, integra	NA	-0.02075	6.878012	-1.01448	0.73182738	0.896062
14302	Frk	fyn-related kinase, transcript variant 3	NA	-0.09114	0.368356	-1.06521	0.73184979	0.896062
69953	2810025M15Rik	RIKEN cDNA 2810025M15 gene	NA	0.027348	4.474377	1.019137	0.73200047	0.896127
72669	2810032G03Rik	RIKEN cDNA 2810032G03 gene	NA	-0.04223	3.005365	-1.0297	0.73206748	0.896127
20315	Cxcl12	chemokine (C-X-C motif) ligand 12, transcript variant	NA	-0.01742	6.221991	-1.01215	0.73206871	0.896127
66774	Carlr	cardiac and apoptosis-related long non-coding RNA	NA	0.12333	-0.75234	1.089246	0.73218686	0.896138
76516	2010001A14Rik	RIKEN cDNA 2010001A14 gene	NA	-0.11127	0.16804	-1.08018	0.73222457	0.896138
19126	Prom1	prominin 1, transcript variant 7	NA	-0.02409	4.903012	-1.01684	0.73227883	0.896138
217140	Scrn2	secernin 2, transcript variant X3	NA	-0.04869	2.606251	-1.03433	0.73229744	0.896138
269881	Map3k10	mitogen-activated protein kinase kinase kinase 10	NA	-0.02049	6.825556	-1.0143	0.73236557	0.896154
94044	Bcl2l13	BCL2-like 13 (apoptosis facilitator), transcript variant	NA	0.020602	5.820157	1.014382	0.73254364	0.896271
69325	1700012B09Rik	RIKEN cDNA 1700012B09 gene	NA	-0.16887	-0.65857	-1.12418	0.73257176	0.896271
16573	Kif5b	kinesin family member 5B	NA	0.019079	7.223703	1.013312	0.73266615	0.896319
13008	Csrp2	cysteine and glycine-rich protein 2	NA	0.028951	5.754299	1.02027	0.73284175	0.8964
665268	1600029O15Rik	RIKEN cDNA 1600029O15 gene	NA	-0.04167	3.192077	-1.0293	0.73284198	0.8964
108168260	Gm46533	predicted gene, 46533	NA	-0.04352	2.451623	-1.03062	0.73293431	0.896445
213980	Fbxw10	F-box and WD-40 domain protein 10, transcript variar	NA	-0.1543	-0.69442	-1.11288	0.73308839	0.896514
77866	E130102H24Rik	RIKEN cDNA E130102H24 gene	NA	-0.06046	1.818068	-1.0428	0.73310041	0.896514
28018	Ubfd1	ubiquitin family domain containing 1	NA	-0.01585	7.21681	-1.01104	0.73337162	0.896778
75216	Cep128	centrosomal protein 128	NA	-0.04165	3.345818	-1.02929	0.73345004	0.896801
68666	Svop	SV2 related protein, transcript variant 1	NA	-0.02047	6.501535	-1.01429	0.73350077	0.896801
215493	A3galt2	alpha 1,3-galactosyltransferase 2 (isoglobotriaosylcer	NA	-0.07619	0.901803	-1.05423	0.73367477	0.896946
72320	Kifbp	kinesin family binding protein	NA	0.018248	6.420607	1.012729	0.73389801	0.897102
77613	Prss36	protease, serine 36, transcript variant X30	NA	0.049405	2.22017	1.034838	0.73397153	0.897102
108841	Rdh13	retinol dehydrogenase 13 (all-trans and 9-cis), transc	NA	0.022563	5.323214	1.015762	0.73397967	0.897102
20692	Sparc	secreted acidic cysteine rich glycoprotein, transcript v	NA	0.018493	8.282035	1.012901	0.73402302	0.897102
69170	Chaserr	CHD2 adjacent suppressive regulatory RNA	NA	0.021205	5.244077	1.014806	0.73425015	0.897313
105242430	Gm38699	predicted gene, 38699, transcript variant X13	NA	0.141274	-0.26162	1.102878	0.73433311	0.897322
115488826	Gm52320	predicted gene, 52320	NA	-0.11038	0.662201	-1.07951	0.73436801	0.897322
244152	Tsku	tsukushi, small leucine rich proteoglycan, transcript va	NA	-0.03407	4.121394	-1.0239	0.73457086	0.897502
51801	Ramp1	receptor (calcitonin) activity modifying protein 1, trans	NA	0.061626	2.05194	1.043641	0.73468152	0.89757
73582	Camkmt	calmodulin-lysine N-methyltransferase, transcript vari	NA	-0.02971	4.02598	-1.0208	0.73479124	0.897588
245305	B230307C23Rik	RIKEN cDNA B230307C23 gene, transcript variant 1	NA	0.035754	3.450171	1.025092	0.73480658	0.897588
21957	Tnnt3	troponin T3, skeletal, fast, transcript variant 4	NA	-0.0948	2.099693	-1.06792	0.73499048	0.897661
380752	Eipr1	EARP complex and GARP complex interacting protei	NA	0.024719	4.898796	1.017282	0.73500061	0.897661
115488819	Gm17251	predicted gene, 17251	NA	0.073962	1.621364	1.052603	0.7350315	0.897661
100503085	Klhl3	kelch-like 3, transcript variant X2	NA	-0.02584	4.677579	-1.01807	0.73513992	0.897726
118568793	LOC118568793	uncharacterized LOC118568793	NA	-0.05999	1.502638	-1.04246	0.73535125	0.897894

100532730	C230037L18Rik	RIKEN cDNA C230037L18 gene	NA	-0.11991	-0.3514	-1.08667	0.73542581	0.897894
66911	Nudt16l1	nudix (nucleoside diphosphate linked moiety X)-type	NA	-0.02344	5.162355	-1.01638	0.73544345	0.897894
102636546	Gm33582	predicted gene, 33582, transcript variant X1	NA	0.032787	3.402381	1.022987	0.73552377	0.897925
71956	Rnf135	ring finger protein 135, transcript variant 1	NA	-0.06742	1.209993	-1.04784	0.73558559	0.897933
100504661	Gm20337	predicted gene, 20337	NA	0.106709	0.629777	1.076769	0.7356923	0.897992
114886	Cygb	cytoglobin	NA	0.025821	4.614286	1.018059	0.73574428	0.897992
13655	Egr3	early growth response 3, transcript variant 1	NA	0.048524	2.278637	1.034206	0.73588366	0.898091
116905	Dph1	diphthamide biosynthesis 1	NA	0.047867	2.596528	1.033735	0.73593568	0.898091
74838	Naa15	N(alpha)-acetyltransferase 15, NatA auxiliary subunit	NA	0.015527	7.062996	1.010821	0.73607408	0.898133
78070	Cpt1c	carnitine palmitoyltransferase 1c, transcript variant X	NA	-0.01969	5.675945	-1.01374	0.73611869	0.898133
109904	Mcf2	mcf.2 transforming sequence, transcript variant 2	NA	0.062518	1.722531	1.044287	0.73613598	0.898133
118568702	LOC118568702	translation initiation factor IF-2-like, transcript variant	NA	0.101724	-0.11296	1.073055	0.73628024	0.898242
22370	Vtn	vitronectin	NA	0.019022	5.736378	1.013272	0.73646601	0.898401
211253	Mtrf1	mitochondrial translational release factor 1	NA	0.045005	2.775875	1.031687	0.73657739	0.89847
98258	Txndc9	thioredoxin domain containing 9, transcript variant X2	NA	-0.01717	6.152208	-1.01197	0.73680224	0.898622
100038453	Gm12522	predicted gene 12522	NA	-0.14599	-0.19993	-1.10649	0.73681291	0.898622
381270	Marchf4	membrane associated ring-CH-type finger 4, transcrip	NA	0.021574	6.1055	1.015066	0.73707906	0.89888
115489152	LOC115489152	uncharacterized LOC115489152	NA	0.071755	1.299795	1.050994	0.7371467	0.898895
102634341	Gm31946	predicted gene, 31946, transcript variant X1	NA	0.115211	-0.18981	1.083133	0.73720274	0.898896
109275	Actr5	ARP5 actin-related protein 5, transcript variant X7	NA	0.032254	3.533227	1.022609	0.7372846	0.898928
319229	Sctr	secretin receptor, transcript variant X6	NA	-0.14361	-0.13515	-1.10467	0.73738107	0.898978
66706	Ndufaf3	NADH:ubiquinone oxidoreductase complex assembly	NA	0.029803	4.190565	1.020873	0.73745523	0.898995
93883	Pcdhb12	protocadherin beta 12	NA	0.057925	1.778398	1.040967	0.73750519	0.898995
26441	Psma4	proteasome subunit alpha 4	NA	0.021898	6.969705	1.015294	0.73764302	0.899087
270150	Ccdc153	coiled-coil domain containing 153, transcript variant X	NA	0.163916	-0.95697	1.120324	0.73778051	0.899087
70567	Fra10ac1	FRA10AC1 homolog (human), transcript variant 1	NA	0.02826	4.424591	1.019781	0.73790744	0.899087
69125	Cnot8	CCR4-NOT transcription complex, subunit 8, transcri	NA	-0.0201	6.245832	-1.01403	0.73790774	0.899087
329942	Csmd2	CUB and Sushi multiple domains 2	NA	-0.01866	6.198158	-1.01302	0.7379181	0.899087
15375	Foxa1	forkhead box A1	NA	0.042871	2.842646	1.030162	0.73796706	0.899087
117606	Boc	biregional cell adhesion molecule-related/down-regul	NA	-0.02157	4.888742	-1.01507	0.73798917	0.899087
434377	Zfp560	zinc finger protein 560	NA	-0.02897	4.094746	-1.02028	0.73802302	0.899087
72834	Cerox1	cytoplasmic endogenous regulator of oxidative phosp	NA	-0.06764	1.728955	-1.048	0.73807974	0.899089
67708	Pcnx4	pecanex homolog 4	NA	0.021875	4.949731	1.015278	0.73832959	0.899305
70428	Polr3b	polymerase (RNA) III (DNA directed) polypeptide B	NA	0.02347	4.865136	1.016401	0.73839136	0.899305
224024	Scarf2	scavenger receptor class F, member 2	NA	-0.05409	3.22286	-1.03821	0.73846652	0.899305
97423	R74862	expressed sequence R74862, transcript variant 1	NA	0.032207	3.519749	1.022575	0.73847806	0.899305
102638607	Gm15927	predicted gene 15927, transcript variant X1	NA	-0.07677	0.927186	-1.05466	0.73872422	0.899538
319830	1500004A13Rik	RIKEN cDNA 1500004A13 gene, transcript variant 4	NA	0.017681	5.99141	1.012331	0.73884106	0.899613
115486908	Gm51558	predicted gene, 51558	NA	-0.0983	2.191833	-1.07051	0.73916884	0.899919
73656	Ms4a6c	membrane-spanning 4-domains, subfamily A, membe	NA	-0.09605	0.869543	-1.06884	0.73928107	0.899919
105245730	Gm41133	predicted gene, 41133	NA	-0.13282	-0.13482	-1.09643	0.7393013	0.899919
78330	Ndufv3	NADH:ubiquinone oxidoreductase core subunit V3, tr	NA	0.02561	5.638478	1.01791	0.73937761	0.899919
66973	Mrps18b	mitochondrial ribosomal protein S18B, transcript varia	NA	-0.02738	4.260088	-1.01916	0.73938851	0.899919
219151	Scara3	scavenger receptor class A, member 3	NA	-0.04012	3.396618	-1.0282	0.73942471	0.899919
66279	Tmem218	transmembrane protein 218, transcript variant 2	NA	0.039935	3.107751	1.028068	0.73957414	0.899977
269951	Idh2	isocitrate dehydrogenase 2 (NADP+), mitochondrial	NA	-0.02034	6.615094	-1.0142	0.73958269	0.899977
16404	Itga7	integrin alpha 7, transcript variant 1	NA	-0.0435	2.808566	-1.03061	0.73974766	0.900065
320351	Tmem251	transmembrane protein 251, transcript variant 2	NA	-0.0382	3.626549	-1.02683	0.73976608	0.900065
67855	Asprv1	aspartic peptidase, retroviral-like 1	NA	-0.17366	1.408675	-1.12791	0.73997984	0.900217
207742	Rnf43	ring finger protein 43, transcript variant 2	NA	-0.09007	0.748998	-1.06442	0.74000127	0.900217
15412	Hoxb4	homeobox B4	NA	-0.12556	1.587569	-1.09093	0.74008442	0.90022
327978	Slfn5	schlafen 5, transcript variant X7	NA	-0.06586	1.743188	-1.04671	0.74016297	0.90022
115487436	Gm51792	predicted gene, 51792	NA	0.299667	-0.93113	1.230861	0.74016988	0.90022
29812	Ndrg3	N-myc downstream regulated gene 3, transcript varia	NA	0.014905	7.056067	1.010385	0.74035645	0.90038
227733	Pip5kl1	phosphatidylinositol-4-phosphate 5-kinase-like 1, tran	NA	0.045322	2.276978	1.031913	0.7404901	0.900411
64655	Mrps22	mitochondrial ribosomal protein S22	NA	-0.02546	4.710461	-1.01781	0.74049293	0.900411
22323	Vasp	vasodilator-stimulated phosphoprotein, transcript vari	NA	-0.01804	5.768471	-1.01259	0.74058952	0.900461
77627	Efcab6	EF-hand calcium binding domain 6, transcript variant	NA	-0.0735	1.465396	-1.05226	0.74070442	0.90049
100019	Mdn1	midasin AAA ATPase 1, transcript variant X1	NA	0.024616	5.881369	1.017209	0.74072392	0.90049
118568080	LOC118568080	MLV-related proviral Env polyprotein-like, transcript v	NA	-0.08063	2.097868	-1.05748	0.74083263	0.900523
212503	Paox	polyamine oxidase (exo-N4-amino), transcript variant	NA	-0.04853	2.824585	-1.03421	0.7408613	0.900523
105245097	Gm40595	predicted gene, 40595	NA	0.042052	2.5895	1.029577	0.74108549	0.900718
233280	Nipa1	non imprinted in Prader-Willi/Angelman syndrome 1 h	NA	0.031552	4.439368	1.022111	0.74120173	0.900718
12593	Cdyl	chromodomain protein, Y chromosome-like, transcrip	NA	-0.04132	3.299538	-1.02906	0.74128975	0.900718
229675	Rsbn1	rosbin, round spermatid basic protein 1, transcript va	NA	0.024338	6.265509	1.017013	0.74129859	0.900718
17130	Smad6	SMAD family member 6	NA	0.053183	1.962426	1.037552	0.74129885	0.900718
102414	Clk3	CDC-like kinase 3, transcript variant X2	NA	-0.01991	6.17981	-1.0139	0.74148612	0.90086
100502695	Gm17399	predicted gene, 17399	NA	-0.1246	-0.41344	-1.09021	0.74152899	0.90086

105242449	Gm21103	predicted gene, 21103, transcript variant X2	NA	0.154086	0.192454	1.112717	0.7416705	0.90086
227644	Snapc4	small nuclear RNA activating complex, polypeptide 4,	NA	-0.02281	5.305958	-1.01594	0.74167648	0.90086
100039861	Gm2464	predicted gene 2464	NA	0.08434	0.785297	1.060203	0.74169246	0.90086
78787	Usp54	ubiquitin specific peptidase 54, transcript variant X7	NA	0.02176	5.395427	1.015197	0.74188524	0.901027
110323	Cox6b1	cytochrome c oxidase, subunit 6B1, transcript variant	NA	0.023239	7.248655	1.016238	0.7420062	0.901095
666190	Gm7972	predicted gene 7972	NA	0.076315	0.999376	1.054322	0.7421062	0.901095
14356	Timm10b	translocase of inner mitochondrial membrane 10B, tra	NA	0.036193	3.855618	1.025404	0.74210759	0.901095
64103	Tnmd	tenomodulin	NA	0.124197	-0.62889	1.089901	0.74225947	0.901101
68634	Tm2d3	TM2 domain containing 3, transcript variant 1	NA	0.032542	3.941258	1.022813	0.74229911	0.901101
26458	Slc27a2	solute carrier family 27 (fatty acid transporter), memb	NA	0.037256	3.08276	1.02616	0.7424021	0.901101
102637583	Gm34354	predicted gene, 34354, transcript variant X3	NA	-0.17984	-0.41254	-1.13276	0.74241227	0.901101
13046	Celf1	CUGBP, Elav-like family member 1, transcript variant	NA	0.013931	8.029164	1.009703	0.74243846	0.901101
70028	Dop1b	DOP1 leucine zipper like protein B, transcript variant	NA	0.021917	5.87385	1.015307	0.74244438	0.901101
16939	Lor	loricrin	NA	-0.17666	2.078759	-1.13027	0.7425268	0.901133
80893	Tmprss5	transmembrane protease, serine 5 (spinesin), transcr	NA	0.120295	0.298235	1.086957	0.74266035	0.901228
19255	Ptpn2	protein tyrosine phosphatase, non-receptor type 2, tra	NA	0.021328	5.357982	1.014893	0.74277203	0.901297
269799	Clec4a1	C-type lectin domain family 4, member a1	NA	0.092105	0.27114	1.065924	0.74290332	0.901389
74202	Fblim1	filamin binding LIM protein 1, transcript variant X5	NA	0.023019	5.252521	1.016083	0.74316482	0.901639
231134	Dok7	docking protein 7, transcript variant 3	NA	0.037491	3.042441	1.026327	0.74335575	0.901732
56412	Noa1	nitric oxide associated 1	NA	-0.02204	4.832873	-1.0154	0.74342407	0.901732
228994	Ythdf1	YTH N6-methyladenosine RNA binding protein 1	NA	0.017803	6.404376	1.012416	0.74345477	0.901732
382137	Fdxacb1	ferredoxin-fold anticodon binding domain containing ´	NA	-0.04645	2.43627	-1.03272	0.74348046	0.901732
244962	Snx14	sorting nexin 14, transcript variant X1	NA	0.021433	5.06801	1.014967	0.74353177	0.901732
333789	N4bp2	NEDD4 binding protein 2, transcript variant X1	NA	0.017687	6.119914	1.012335	0.74357389	0.901732
231571	Rpap2	RNA polymerase II associated protein 2, transcript va	NA	0.029923	4.305931	1.020958	0.74376211	0.901853
72416	Lrpprc	leucine-rich PPR-motif containing, transcript variant X	NA	0.019415	6.047347	1.013548	0.74383303	0.901853
56208	Becn1	beclin 1, autophagy related, transcript variant 1	NA	-0.01685	6.544584	-1.01175	0.74387463	0.901853
246788	Trpv3	transient receptor potential cation channel, subfamily	NA	-0.1252	-0.26931	-1.09066	0.74389574	0.901853
234723	Txnl4b	thioredoxin-like 4B	NA	-0.03463	3.555793	-1.02429	0.74397198	0.901878
118568566	LOC118568566	uncharacterized LOC118568566	NA	0.059039	2.519413	1.041772	0.74411805	0.901988
17762	Mapt	microtubule-associated protein tau, transcript variant	NA	0.015885	10.73512	1.011071	0.74417494	0.90199
442799	9330154J02Rik	RIKEN cDNA 9330154J02 gene, transcript variant X6	NA	-0.09612	0.527838	-1.0689	0.7443231	0.902039
66212	Sec61b	Sec61 beta subunit	NA	-0.02715	5.044353	-1.019	0.74432639	0.902039
227683	Coq4	coenzyme Q4, transcript variant 1	NA	0.030855	3.962038	1.021617	0.74448279	0.902104
233545	Emsy	EMSY, BRCA2-interacting transcriptional repressor, t	NA	0.018284	6.441754	1.012754	0.74461035	0.902104
18530	Pcdh8	protocadherin 8, transcript variant 1	NA	-0.02202	6.130792	-1.01538	0.74461815	0.902104
102638091	Gm34736	predicted gene, 34736, transcript variant X2	NA	0.078972	0.524491	1.056265	0.74463656	0.902104
57246	Tbx20	T-box 20, transcript variant 1	NA	-0.11221	0.166642	-1.08088	0.74468747	0.902104
226982	Eif5b	eukaryotic translation initiation factor 5B, transcript va	NA	0.015979	7.019315	1.011138	0.74471295	0.902104
18574	Pde1b	phosphodiesterase 1B, Ca2+-calmodulin dependent,	NA	0.022691	6.557693	1.015853	0.74477441	0.902112
240518	Peli3	pellino 3, transcript variant X1	NA	-0.02921	4.119388	-1.02046	0.74483819	0.902122
56419	Diaph3	diaphanous related formin 3	NA	-0.04451	2.792611	-1.03134	0.74491642	0.902149
94063	Mrpl16	mitochondrial ribosomal protein L16	NA	0.026542	4.176311	1.018568	0.74512094	0.90233
11541	Adora2b	adenosine A2b receptor	NA	-0.09049	0.686505	-1.06473	0.74523751	0.902404
74355	Smchd1	SMC hinge domain containing 1, transcript variant X1	NA	0.018786	6.162026	1.013107	0.74545947	0.902606
52033	Pbk	PDZ binding kinase	NA	-0.02713	4.923026	-1.01898	0.74554044	0.902636
118567794	LOC118567794	uncharacterized LOC118567794	NA	-0.134	-0.03875	-1.09733	0.74563334	0.902682
22029	Traf1	TNF receptor-associated factor 1, transcript variant X	NA	-0.10467	0.462549	-1.07525	0.74585624	0.902884
54198	Snx3	sorting nexin 3	NA	0.01683	6.824733	1.011734	0.74591909	0.902893
108168740	Gm46731	predicted gene, 46731	NA	-0.06002	1.489567	-1.04248	0.74615909	0.903058
22712	Zfp54	zinc finger protein 54, transcript variant X1	NA	0.050935	2.086405	1.035936	0.7461917	0.903058
13560	E4f1	E4F transcription factor 1, transcript variant 2	NA	-0.02527	5.001285	-1.01767	0.74622166	0.903058
27494	Amot	angiomotin, transcript variant X3	NA	-0.02269	5.142469	-1.01585	0.74635444	0.903104
319899	Dock6	dedicator of cytokinesis 6, transcript variant X14	NA	-0.02443	5.007616	-1.01708	0.74637059	0.903104
67788	Sfr1	SWI5 dependent recombination repair 1, transcript va	NA	-0.01757	6.882607	-1.01225	0.74652063	0.903218
12933	Crmp1	collapsin response mediator protein 1, transcript varia	NA	-0.0158	10.33913	-1.01101	0.74675649	0.903437
227526	Cdnf	cerebral dopamine neurotrophic factor	NA	0.116215	-0.26358	1.083887	0.74701109	0.903625
105242638	Gm38797	predicted gene, 38797	NA	-0.12534	-0.76757	-1.09077	0.74706989	0.903625
208967	Thnsl1	threonine synthase-like 1 (bacterial), transcript varian	NA	-0.02267	4.854066	-1.01583	0.74707848	0.903625
52710	Slc52a2	solute carrier protein 52, member 2, transcript variant	NA	-0.0259	4.395716	-1.01812	0.747142	0.903626
26563	Ror1	receptor tyrosine kinase-like orphan receptor 1, trans	NA	0.054519	2.095524	1.038513	0.74719099	0.903626
16976	Lrpap1	low density lipoprotein receptor-related protein assoc	NA	0.016198	6.549806	1.011291	0.74745117	0.903874
213081	Wdr19	WD repeat domain 19, transcript variant 1	NA	-0.02956	4.068848	-1.0207	0.74774159	0.904158
12054	Bcl7b	B cell CLL/lymphoma 7B	NA	-0.02641	4.283139	-1.01848	0.74779989	0.904161
217149	Cisd3	CDGSH iron sulfur domain 3, transcript variant 2	NA	0.045554	2.515289	1.03208	0.74786463	0.904172
67456	Ergic2	ERGIC and golgi 2, transcript variant 1	NA	0.015402	6.347306	1.010733	0.7479929	0.90426
69064	Fuom	fucose mutarotase, transcript variant X2	NA	0.050201	2.287401	1.03541	0.7481594	0.904394
12419	Cbx5	chromobox 5, transcript variant 2	NA	-0.01804	8.584967	-1.01258	0.748479	0.904565

320440	9530091C08Rik	RIKEN cDNA 9530091C08 gene	NA	-0.06598	1.664065	-1.0468	0.74849721	0.904565
67669	Hikeshi	heat shock protein nuclear import factor, transcript va	NA	0.020491	5.22671	1.014305	0.748621	0.904565
100045653	Gm17769	predicted gene, 17769	NA	0.085269	0.313116	1.060885	0.74862721	0.904565
74959	Platr14	pluripotency associated transcript 14, transcript varia	NA	-0.10771	-0.10774	-1.07752	0.74870684	0.904565
240332	Slc6a7	solute carrier family 6 (neurotransmitter transporter, L	NA	0.026171	4.977823	1.018306	0.74871815	0.904565
22215	Ube3a	ubiquitin protein ligase E3A, transcript variant 3	NA	-0.01689	7.337927	-1.01178	0.74872387	0.904565
70713	Gpr137c	G protein-coupled receptor 137C, transcript variant X	NA	0.023598	5.251349	1.016491	0.74874523	0.904565
21389	Tbx6	T-box 6	NA	0.085887	0.439409	1.06134	0.74885283	0.904628
268880	Xxylt1	xyloside xylosyltransferase 1, transcript variant X2	NA	-0.02274	4.61134	-1.01589	0.74900696	0.904672
268882	Fbxo45	F-box protein 45	NA	0.019394	6.454416	1.013534	0.74902546	0.904672
67941	Rps27l	ribosomal protein S27-like, transcript variant 2	NA	-0.02596	4.756292	-1.01816	0.7490565	0.904672
67563	Ciao3	cytosolic iron-sulfur assembly component 3, transcrip	NA	-0.02808	4.115125	-1.01965	0.74920225	0.904728
235611	Plxnb1	plexin B1, transcript variant X12	NA	-0.01733	7.252572	-1.01208	0.74921386	0.904728
58875	Hibadh	3-hydroxyisobutyrate dehydrogenase	NA	0.020148	5.097576	1.014063	0.74946406	0.904889
74326	Hnrnpr	heterogeneous nuclear ribonucleoprotein R, transcrip	NA	-0.01445	8.243394	-1.01007	0.74948256	0.904889
20454	St3gal5	ST3 beta-galactoside alpha-2,3-sialyltransferase 5, tr	NA	0.021563	5.640431	1.015059	0.74955069	0.904889
57764	Ntn4	netrin 4	NA	-0.04386	2.308838	-1.03087	0.74957008	0.904889
245368	Zfp300	zinc finger protein 300, transcript variant X1	NA	-0.04635	2.269879	-1.03265	0.74962927	0.904894
67106	Zbtb8os	zinc finger and BTB domain containing 8 opposite str	NA	0.029034	3.623159	1.020328	0.74988761	0.905044
14419	Gal	galanin and GMAP prepropeptide, transcript variant 1	NA	-0.06917	0.956984	-1.04911	0.74991157	0.905044
231727	B3gnt4	UDP-GlcNAc:betaGal beta-1,3-N-acetylglucosaminylt	NA	-0.08223	0.937846	-1.05866	0.7499207	0.905044
26417	Mapk3	mitogen-activated protein kinase 3	NA	-0.01802	6.91011	-1.01257	0.75000137	0.905073
105247153	Gm11267	predicted gene 11267	NA	-0.09999	-0.07417	-1.07176	0.75006005	0.905073
118567453	LOC118567453	uncharacterized LOC118567453	NA	0.068869	2.094774	1.048894	0.75011182	0.905073
224093	Fam43a	family with sequence similarity 43, member A	NA	-0.03275	3.673936	-1.02296	0.75018146	0.90509
66894	Wwp2	WW domain containing E3 ubiquitin protein ligase 2,	NA	0.02175	5.703591	1.01519	0.75055309	0.90545
217480	Dgkb	diacylglycerol kinase, beta, transcript variant 2	NA	-0.03554	3.414344	-1.02494	0.75062275	0.90545
20102	Rps4x	ribosomal protein S4, X-linked	NA	0.014931	9.360182	1.010403	0.75064683	0.90545
67896	Ccdc80	coiled-coil domain containing 80	NA	-0.02917	4.145467	-1.02042	0.75078828	0.905521
105244756	Gm40309	predicted gene, 40309	NA	-0.04164	4.124528	-1.02928	0.75081657	0.905521
109129	Mmadhc	methylmalonic aciduria (cobalamin deficiency) cblD ty	NA	0.017333	5.819882	1.012087	0.75102655	0.905549
20615	Snapin	SNAP-associated protein	NA	0.02609	5.309476	1.018248	0.75105327	0.905549
67205	Utp11	UTP11 small subunit processome component	NA	-0.02302	4.689402	-1.01609	0.75112573	0.905549
19703	Renbp	renin binding protein, transcript variant 1	NA	0.049107	1.930811	1.034624	0.75113657	0.905549
327743	Ccn6	cellular communication network factor 6, transcript va	NA	-0.0467	2.580633	-1.0329	0.75120363	0.905549
269999	Orai3	ORAI calcium release-activated calcium modulator 3	NA	-0.04443	3.138505	-1.03128	0.75129055	0.905549
212555	Slc66a1	solute carrier family 66 member 1, transcript variant X	NA	0.026496	4.054919	1.018535	0.75132698	0.905549
12362	Casp1	caspase 1	NA	-0.13693	-0.26268	-1.09956	0.75133353	0.905549
117229	Stk33	serine/threonine kinase 33, transcript variant X15	NA	-0.047	2.493283	-1.03311	0.75134114	0.905549
244349	Kat6a	K(lysine) acetyltransferase 6A, transcript variant X2	NA	-0.01689	6.934009	-1.01177	0.75147493	0.905588
243923	Rgs9bp	regulator of G-protein signalling 9 binding protein	NA	-0.08937	0.139754	-1.06391	0.75148431	0.905588
70285	Rpf1	ribosome production factor 1 homolog, transcript vari	NA	0.023349	4.889619	1.016316	0.75165912	0.905731
68659	Gask1b	golgi associated kinase 1B	NA	0.055587	2.475725	1.039282	0.75180136	0.905836
54446	Nfat5	nuclear factor of activated T cells 5, transcript variant	NA	0.01964	6.640896	1.013706	0.75189027	0.905856
16492	Kcna4	potassium voltage-gated channel, shaker-related sub	NA	-0.03299	3.843172	-1.02313	0.75192971	0.905856
66163	Mrpl4	mitochondrial ribosomal protein L4, transcript variant	NA	-0.02958	4.692862	-1.02072	0.75218852	0.906101
18760	Prkd1	protein kinase D1, transcript variant 2	NA	0.038641	3.618453	1.027146	0.75226563	0.906127
67036	Mrpl45	mitochondrial ribosomal protein L45	NA	-0.02144	5.031962	-1.01497	0.75232518	0.906127
74341	G630025P09Rik	RIKEN cDNA G630025P09 gene	NA	0.073029	1.202645	1.051923	0.75237709	0.906127
21754	Tesk1	testis specific protein kinase 1	NA	-0.01752	6.69196	-1.01222	0.75244094	0.90613
15959	Ifit3	interferon-induced protein with tetratricopeptide repea	NA	0.124791	0.019197	1.09035	0.752491	0.90613
319767	Atp10b	ATPase, class V, type 10B, transcript variant X2	NA	-0.08755	0.627073	-1.06256	0.7525536	0.906138
115487155	LOC115487155	uncharacterized LOC115487155	NA	0.041119	2.720125	1.028912	0.75263149	0.906165
14695	Gnb3	guanine nucleotide binding protein (G protein), beta 3	NA	0.1159	-0.39136	1.083651	0.75286235	0.906248
17966	Nbr1	NBR1, autophagy cargo receptor, transcript variant 1	NA	-0.0141	7.444192	-1.00982	0.75287422	0.906248
13423	Dnase2a	deoxyribonuclease II alpha, transcript variant 1	NA	0.054216	1.616437	1.038294	0.75290151	0.906248
68015	Trap1	TNF receptor-associated protein 1, transcript variant	NA	-0.02244	5.438853	-1.01568	0.75292355	0.906248
70252	Jpx	Jpx transcript, Xist activator (non-protein coding)	NA	-0.04445	2.303467	-1.03129	0.75311456	0.906369
328274	Zfp459	zinc finger protein 459	NA	-0.08258	0.945019	-1.05891	0.75314172	0.906369
271711	Tmem169	transmembrane protein 169, transcript variant X3	NA	0.021992	5.282491	1.015361	0.75320076	0.906369
100041958	Lamtor3-ps	late endosomal/lysosomal adaptor, MAPK and MTOF	NA	0.115922	0.909343	1.083667	0.75324667	0.906369
20055	Rps16	ribosomal protein S16	NA	-0.02734	7.919073	-1.01913	0.75347457	0.90657
231050	Galnt11	polypeptide N-acetylgalactosaminyltransferase 11, tra	NA	0.01797	5.477336	1.012534	0.75355178	0.90657
71382	Pex1	peroxisomal biogenesis factor 1, transcript variant X2	NA	0.02545	4.422573	1.017797	0.75358069	0.90657
408066	Cspg4b	chondroitin sulfate proteoglycan 4B	NA	-0.04853	2.51462	-1.03421	0.75363714	0.906571
70494	5730409K12Rik	RIKEN cDNA 5730409K12 gene	NA	-0.13152	-0.44088	-1.09545	0.75392686	0.906852
75735	Pank1	pantothenate kinase 1, transcript variant 2	NA	0.021452	4.899791	1.01498	0.75409572	0.906904
19655	Rbmx	RNA binding motif protein, X chromosome, transcript	NA	-0.01422	7.980473	-1.0099	0.75411351	0.906904

329278	Tnn	tenascin N, transcript variant X1	NA	-0.11297	-0.23919	-1.08145	0.75413745	0.906904
226442	Zfp281	zinc finger protein 281, transcript variant 2	NA	0.022172	5.858182	1.015487	0.75431715	0.906943
23922	Jtb	jumping translocation breakpoint	NA	0.022254	4.858236	1.015545	0.75435101	0.906943
78688	Nol3	nucleolar protein 3 (apoptosis repressor with CARD c	NA	-0.07992	1.659944	-1.05696	0.75438169	0.906943
105244875	Gm40406	predicted gene, 40406, transcript variant X1	NA	0.135739	-0.30152	1.098656	0.75443545	0.906943
229487	Gatb	glutamyl-tRNA(Gln) amidotransferase, subunit B	NA	0.034985	3.684446	1.024546	0.75444835	0.906943
53317	Plrg1	pleiotropic regulator 1	NA	0.019082	6.055909	1.013314	0.75471048	0.907108
76813	Armc6	armadillo repeat containing 6, transcript variant X4	NA	-0.01994	5.309705	-1.01392	0.7547389	0.907108
72136	Chst14	carbohydrate sulfotransferase 14	NA	-0.04933	2.679091	-1.03478	0.75475266	0.907108
13850	Ephx2	epoxide hydrolase 2, cytoplasmic, transcript variant 1	NA	-0.06665	1.275514	-1.04728	0.75482189	0.907124
14252	Flot2	flotillin 2, transcript variant 7	NA	0.015465	6.47695	1.010777	0.75495146	0.907168
118567959	LOC118567959	uncharacterized LOC118567959	NA	-0.12725	-0.05135	-1.09221	0.75497004	0.907168
111173	Erc1	ELKS/RAB6-interacting/CAST family member 1, trans	NA	0.018184	7.157597	1.012684	0.75513847	0.907285
16186	Il2rg	interleukin 2 receptor, gamma chain, transcript varian	NA	0.066075	1.508568	1.046865	0.75517889	0.907285
22041	Trf	transferrin	NA	-0.03144	4.406352	-1.02203	0.75530486	0.90737
20319	Sfrp2	secreted frizzled-related protein 2	NA	-0.03117	5.078149	-1.02184	0.75540936	0.907415
105244117	Gm13524	predicted gene 13524, transcript variant 2	NA	0.065332	1.038216	1.046326	0.75547132	0.907415
54354	Rassf5	Ras association (RalGDS/AF-6) domain family memb	NA	0.044661	2.447315	1.031441	0.75551023	0.907415
56524	Mpp6	membrane protein, palmitoylated 6 (MAGUK p55 sub	NA	0.021691	5.136017	1.015149	0.75561587	0.907475
58916	Myot	myotilin, transcript variant X3	NA	0.129579	-0.61716	1.093975	0.75577304	0.907525
102633464	Gm31282	predicted gene, 31282	NA	0.068675	1.20854	1.048753	0.7558528	0.907525
368203	Gm5136	predicted gene 5136	NA	-0.08011	0.480078	-1.0571	0.755921	0.907525
78439	A930037H05Rik	RIKEN cDNA A930037H05 gene	NA	0.072955	0.901064	1.051869	0.75592271	0.907525
233875	Ino80e	INO80 complex subunit E, transcript variant X2	NA	-0.01886	5.583683	-1.01316	0.75593631	0.907525
100043508	Ptges3-ps	prostaglandin E synthase 3, pseudogene	NA	-0.04838	3.43249	-1.0341	0.75613194	0.907606
68045	Rtraf	RNA transcription, translation and transport factor	NA	-0.01686	6.80116	-1.01176	0.75614891	0.907606
66120	Fkbp11	FK506 binding protein 11, transcript variant 1	NA	0.097569	-0.19508	1.069969	0.75623806	0.907606
13411	Dnah11	dynein, axonemal, heavy chain 11, transcript variant :	NA	0.085307	1.160014	1.060913	0.75624021	0.907606
68498	Tspan11	tetraspanin 11, transcript variant X1	NA	-0.02948	3.553787	-1.02064	0.7562827	0.907606
50771	Atp9b	ATPase, class II, type 9B, transcript variant 2	NA	0.022194	5.555851	1.015502	0.75658609	0.907862
19896	Rpl10a	ribosomal protein L10A, transcript variant 1	NA	0.020308	8.089803	1.014176	0.75665822	0.907862
18991	Pou3f1	POU domain, class 3, transcription factor 1	NA	0.02929	5.594782	1.02051	0.75666312	0.907862
21380	Tbx1	T-box 1, transcript variant 1	NA	-0.06936	0.788086	-1.04925	0.75676036	0.907912
14043	Ext2	exostosin glycosyltransferase 2, transcript variant 2	NA	0.018798	5.8247	1.013115	0.75695749	0.907919
320679	Samd12	sterile alpha motif domain containing 12, transcript va	NA	-0.0272	5.075868	-1.01903	0.75696139	0.907919
27029	Sgsh	N-sulfoglucosamine sulfohydrolase (sulfamidase)	NA	-0.06132	2.469986	-1.04342	0.75696601	0.907919
12212	Chic1	cysteine-rich hydrophobic domain 1	NA	-0.0247	4.712482	-1.01727	0.75698942	0.907919
474332	Dnm3os	dynamin 3, opposite strand	NA	-0.07641	1.162921	-1.05439	0.75714924	0.90792
320398	Lrig3	leucine-rich repeats and immunoglobulin-like domains	NA	-0.04628	2.617325	-1.0326	0.7571589	0.90792
64424	Polr1e	polymerase (RNA) I polypeptide E, transcript variant :	NA	-0.02983	3.852369	-1.02089	0.75717591	0.90792
59014	Rrs1	ribosome biogenesis regulator 1	NA	0.023168	5.032604	1.016189	0.75726368	0.90792
622645	Tmem200c	transmembrane protein 200C	NA	-0.03734	2.843491	-1.02622	0.75726969	0.90792
77053	Sun1	Sad1 and UNC84 domain containing 1, transcript var	NA	0.016907	6.151428	1.011788	0.75740198	0.908012
69048	Slc30a5	solute carrier family 30 (zinc transporter), member 5	NA	-0.0259	5.401137	-1.01811	0.75748818	0.908048
320950	9330104G04Rik	RIKEN cDNA 9330104G04 gene	NA	-0.03366	3.081712	-1.0236	0.7576143	0.908133
81877	Tnxb	tenascin XB, transcript variant X6	NA	0.067318	1.749706	1.047767	0.75771384	0.908173
15163	Hcls1	hematopoietic cell specific Lyn substrate 1	NA	-0.08142	1.195385	-1.05806	0.75775987	0.908173
22710	Zfp52	zinc finger protein 52, transcript variant X1	NA	-0.05099	2.069505	-1.03598	0.75791506	0.908292
66797	Cntnap2	contactin associated protein-like 2, transcript variant :	NA	0.014644	6.759863	1.010202	0.75823478	0.908609
66818	Smim7	small integral membrane protein 7	NA	0.017494	6.5405	1.0122	0.75837233	0.908707
70737	Cgn	cingulin, transcript variant X2	NA	-0.03459	4.328571	-1.02426	0.75849988	0.908749
67664	Rnf125	ring finger protein 125, transcript variant 1	NA	0.065303	1.171455	1.046304	0.7585744	0.908749
11749	Anxa6	annexin A6, transcript variant 1	NA	0.016827	5.873619	1.011732	0.75857559	0.908749
65116	Prrg2	proline-rich Gla (G-carboxyglutamic acid) polypeptide	NA	0.056518	2.035761	1.039953	0.75874149	0.908862
22200	Uba3	ubiquitin-like modifier activating enzyme 3, transcript	NA	-0.01666	6.673697	-1.01161	0.75884032	0.908862
280408	Rilp	Rab interacting lysosomal protein	NA	0.084302	0.075134	1.060175	0.75896636	0.908862
102633572	Gm31364	predicted gene, 31364, transcript variant X1	NA	-0.08785	0.103679	-1.06278	0.75897986	0.908862
71030	4933403O08Rik	RIKEN cDNA 4933403O08 gene, transcript variant X	NA	-0.08291	0.420229	-1.05916	0.75908133	0.908862
56794	Hacl1	2-hydroxyacyl-CoA lyase 1	NA	0.05251	2.493767	1.037068	0.75909492	0.908862
94185	Tnfrsf21	tumor necrosis factor receptor superfamily, member 2	NA	0.018572	6.340148	1.012956	0.7591238	0.908862
108151	Sema3d	sema domain, immunoglobulin domain (Ig), short bas	NA	0.042782	2.362651	1.030098	0.75913678	0.908862
52892	Sco1	SCO1 cytochrome c oxidase assembly protein	NA	-0.02735	4.021533	-1.01914	0.75931466	0.908862
118567342	LOC118567342	uncharacterized LOC118567342	NA	-0.07905	2.055409	-1.05632	0.75933208	0.908862
56495	Get3	guided entry of tail-anchored proteins factor 3, ATPas	NA	0.017905	5.979886	1.012488	0.75945082	0.908862
382245	Tmem29	transmembrane protein 29, transcript variant 7	NA	0.020565	4.874861	1.014357	0.75963592	0.908862
14739	S1pr2	sphingosine-1-phosphate receptor 2	NA	-0.03326	3.253458	-1.02332	0.75966383	0.908862
16773	Lama2	laminin, alpha 2	NA	0.028678	3.951756	1.020077	0.75972199	0.908862
12345	Capzb	capping protein (actin filament) muscle Z-line, beta, tr	NA	-0.01301	7.532059	-1.00906	0.75976367	0.908862

320727	Ipo8	importin 8	NA	-0.02084	5.349192	-1.01455	0.75976692	0.908862
228543	Rhov	ras homolog family member V	NA	0.032631	3.71414	1.022876	0.75991731	0.908862
103729	AI450353	expressed sequence AI450353	NA	0.05709	1.626327	1.040365	0.75991855	0.908862
74729	Setmar	SET domain without mariner transposase fusion, tran	NA	0.06304	2.125678	1.044665	0.75995135	0.908862
66898	Baiap2l1	BAI1-associated protein 2-like 1	NA	-0.07553	0.653257	-1.05375	0.75996985	0.908862
67752	Ppp1r32	protein phosphatase 1, regulatory subunit 32, transcr	NA	0.065915	1.012805	1.046748	0.75998164	0.908862
102545	Cmtm7	CKLF-like MARVEL transmembrane domain containi	NA	-0.06445	1.244791	-1.04569	0.76005012	0.908862
52864	Slx4	SLX4 structure-specific endonuclease subunit homol	NA	-0.01909	5.641953	-1.01332	0.76010155	0.908862
13726	Emd	emerin	NA	0.018471	5.61801	1.012886	0.76012781	0.908862
72503	2610507B11Rik	RIKEN cDNA 2610507B11 gene	NA	-0.01365	7.194126	-1.00951	0.76013873	0.908862
100043074	Gm4211	predicted gene 4211, transcript variant X2	NA	0.079637	0.444763	1.056752	0.76015542	0.908862
239845	Gpr156	G protein-coupled receptor 156, transcript variant X1	NA	0.030657	3.45379	1.021477	0.76017797	0.908862
102633974	Gm28905	predicted gene 28905, transcript variant X3	NA	0.122274	-0.31579	1.088449	0.76024935	0.908862
230789	Fam76a	family with sequence similarity 76, member A, transc	NA	0.021746	5.140992	1.015187	0.76028981	0.908862
14843	Gsx2	GS homeobox 2	NA	0.074234	1.26382	1.052802	0.76043783	0.908973
17827	Mtx1	metaxin 1, transcript variant 1	NA	-0.02203	4.490491	-1.01539	0.76061364	0.909105
214547	She	src homology 2 domain-containing transforming prote	NA	-0.03642	2.813916	-1.02557	0.76066	0.909105
69221	2410006H16Rik	RIKEN cDNA 2410006H16 gene	NA	0.025096	4.367501	1.017547	0.76074768	0.909143
12879	Cys1	cystin 1, transcript variant X2	NA	-0.0567	1.66638	-1.04008	0.76095229	0.90932
77591	Ddx10	DEAD box helicase 10	NA	-0.0241	4.749031	-1.01685	0.76107752	0.909403
14296	Frat1	frequently rearranged in advanced T cell lymphomas	NA	0.021434	4.460458	1.014968	0.76124314	0.909534
66222	Serpinb1a	serine (or cysteine) peptidase inhibitor, clade B, mem	NA	-0.06751	0.779248	-1.0479	0.76137991	0.909631
70257	Atp5mpl	ATP synthase membrane subunit 6.8PL, transcript va	NA	0.017288	5.983377	1.012055	0.7614897	0.909695
67308	Mrpl46	mitochondrial ribosomal protein L46	NA	-0.03642	3.857314	-1.02557	0.76158271	0.90974
320046	F730043M19Rik	RIKEN cDNA F730043M19 gene	NA	0.073712	0.617784	1.052421	0.76165733	0.909762
94242	Tinagl1	tubulointerstitial nephritis antigen-like 1, transcript var	NA	-0.05099	2.286255	-1.03598	0.76186232	0.90994
330554	Fan1	FANCD2/FANCI-associated nuclease 1, transcript va	NA	0.030978	3.620052	1.021705	0.76200782	0.909959
100042493	Ccl21b	chemokine (C-C motif) ligand 21B (leucine), transcrip	NA	-0.16611	-0.79737	-1.12203	0.76205232	0.909959
77323	9430041J12Rik	RIKEN cDNA 9430041J12 gene	NA	-0.07606	0.785763	-1.05413	0.76213749	0.909959
28088	Rtcb	RNA 2',3'-cyclic phosphate and 5'-OH ligase	NA	0.013981	7.157125	1.009738	0.76218855	0.909959
56869	Zfp109	zinc finger protein 109, transcript variant X3	NA	-0.04167	2.439417	-1.0293	0.76219513	0.909959
115488362	Gm47814	predicted gene, 47814	NA	-0.02865	3.639714	-1.02005	0.76228268	0.909959
18286	Odf2	outer dense fiber of sperm tails 2, transcript variant X	NA	0.015413	6.588631	1.010741	0.76230039	0.909959
13199	Ddn	dendrin	NA	-0.03809	2.91076	-1.02676	0.76232585	0.909959
102637759	Gm34489	predicted gene, 34489, transcript variant X4	NA	-0.09053	0.921648	-1.06476	0.76247595	0.910016
246727	Oas3	2'-5' oligoadenylate synthetase 3	NA	-0.10265	-0.08341	-1.07374	0.76248523	0.910016
22417	Wnt4	wingless-type MMTV integration site family, member	NA	-0.038	3.418611	-1.02669	0.76279987	0.910325
115489936	Gm12743	predicted gene 12743, transcript variant X1	NA	0.072741	0.677936	1.051713	0.76288747	0.910357
108699	Chn1	chimerin 1, transcript variant X4	NA	-0.01347	7.086981	-1.00938	0.76297777	0.910357
103724	Tbc1d10a	TBC1 domain family, member 10a	NA	-0.0319	3.847197	-1.02236	0.76299493	0.910357
108168868	LOC108168868	uncharacterized LOC108168868	NA	0.083442	0.257838	1.059543	0.76315806	0.910402
74335	Xrcc3	X-ray repair complementing defective repair in Chine	NA	0.045118	2.080615	1.031768	0.76316313	0.910402
107815	Scml2	Scm polycomb group protein like 2, transcript variant	NA	-0.03327	3.107119	-1.02333	0.76326941	0.910402
69072	Ebna1bp2	EBNA1 binding protein 2	NA	0.018424	6.024064	1.012852	0.76334548	0.910402
212937	Tifab	TRAF-interacting protein with forkhead-associated dc	NA	-0.08804	0.514249	-1.06292	0.76338865	0.910402
16150	Ikbkb	inhibitor of kappaB kinase beta, transcript variant 2	NA	-0.01949	5.564424	-1.0136	0.76340087	0.910402
223864	Rapgef3	Rap guanine nucleotide exchange factor (GEF) 3, tra	NA	0.030018	3.888081	1.021025	0.76344574	0.910402
67529	Fgfr1op2	FGFR1 oncogene partner 2, transcript variant 2	NA	-0.01829	5.271617	-1.01276	0.76349653	0.910402
18861	Pms2	PMS1 homolog2, mismatch repair system componen	NA	0.019503	4.826889	1.01361	0.76353614	0.910402
71726	Smug1	single-strand selective monofunctional uracil DNA gly	NA	-0.02309	4.734769	-1.01613	0.76369416	0.910524
12918	Crh	corticotropin releasing hormone	NA	-0.07762	0.357603	-1.05528	0.76376162	0.910538
115487771	LOC115487771	uncharacterized LOC115487771	NA	-0.08592	0.387874	-1.06137	0.76387903	0.910611
77117	Zfp934	zinc finger protein 934, transcript variant 3	NA	0.044157	2.287916	1.031081	0.76400066	0.910661
19217	Ptger2	prostaglandin E receptor 2 (subtype EP2)	NA	-0.10853	-0.05627	-1.07813	0.76403324	0.910661
13626	Eed	embryonic ectoderm development	NA	-0.02478	4.867442	-1.01733	0.7642747	0.910731
108099	Prkag2	protein kinase, AMP-activated, gamma 2 non-catalyti	NA	-0.01614	6.541503	-1.01125	0.76428576	0.910731
69562	Cdk13	cyclin-dependent kinase 13, transcript variant X4	NA	0.01997	6.445494	1.013939	0.76430643	0.910731
268396	Sh3pxd2b	SH3 and PX domains 2B	NA	-0.01737	6.859071	-1.01211	0.76431589	0.910731
71954	Suds3	suppressor of defective silencing 3 homolog (S. cere	NA	-0.01503	6.54939	-1.01047	0.76449302	0.910876
19158	Cyth2	cytohesin 2, transcript variant 1	NA	0.016238	7.45289	1.011319	0.76461345	0.910923
11764	Ap1b1	adaptor protein complex AP-1, beta 1 subunit, transc	NA	-0.01638	6.748831	-1.01142	0.76465995	0.910923
20534	Slc4a1ap	solute carrier family 4 (anion exchanger), member 1,	NA	0.018822	5.463017	1.013132	0.76471255	0.910923
18986	Pou2f1	POU domain, class 2, transcription factor 1, transcrip	NA	-0.01653	5.915824	-1.01152	0.76475618	0.910923
17763	Mtcp1	mature T cell proliferation 1	NA	0.064589	1.405576	1.045787	0.76481778	0.910929
66610	Abi3	ABI gene family, member 3, transcript variant X7	NA	-0.09532	0.598582	-1.0683	0.76494299	0.911012
76808	Rpl18a	ribosomal protein L18A	NA	-0.01339	8.702873	-1.00932	0.76501097	0.911026
216892	Spns2	spinster homolog 2, transcript variant 2	NA	-0.0205	5.289319	-1.01431	0.765162	0.911101
24109	Ubl3	ubiquitin-like 3, transcript variant 3	NA	-0.01593	5.932582	-1.01111	0.76518569	0.911101

100504501	Gm20257	predicted gene, 20257	NA	-0.09875	0.050292	-1.07084	0.76525241	0.911113
75841	Rnf139	ring finger protein 139	NA	0.01742	5.32019	1.012148	0.76539777	0.911154
70611	Fbxo33	F-box protein 33	NA	0.029548	4.057307	1.020692	0.76539832	0.911154
233824	Cog7	component of oligomeric golgi complex 7	NA	0.022022	4.63191	1.015381	0.76568438	0.911412
16362	Irf1	interferon regulatory factor 1, transcript variant 1	NA	0.059543	1.404734	1.042136	0.76572739	0.911412
12723	Clcn1	chloride channel, voltage-sensitive 1, transcript variar	NA	0.036648	2.779584	1.025728	0.76580969	0.911443
67224	Med29	mediator complex subunit 29	NA	-0.02696	4.042776	-1.01886	0.76589748	0.911445
218030	Pou6f2	POU domain, class 6, transcription factor 2, transcrip	NA	0.031178	3.814514	1.021846	0.76598793	0.911445
240038	Zfp994	zinc finger protein 994, transcript variant X2	NA	0.03528	3.41708	1.024756	0.76599248	0.911445
626854	Gm38396	predicted gene, 38396, transcript variant X1	NA	-0.04182	2.639078	-1.02941	0.7660346	0.911445
66682	Trappc5	trafficking protein particle complex 5	NA	0.017188	5.687709	1.011985	0.76613196	0.911494
100504234	Ccdc170	coiled-coil domain containing 170, transcript variant X	NA	-0.07031	0.753602	-1.04994	0.76622479	0.911537
56086	Set	SET nuclear oncogene, transcript variant 2	NA	-0.01415	9.069957	-1.00986	0.76642175	0.911705
114644	Slc13a3	solute carrier family 13 (sodium-dependent dicarboxy	NA	-0.03859	3.049998	-1.02711	0.76665843	0.91192
103889	Hoxb2	homeobox B2	NA	0.107727	1.785103	1.077529	0.76679864	0.91202
72075	Ogfr	opioid growth factor receptor	NA	-0.01996	6.654379	-1.01393	0.76690003	0.912074
114229	Kiss1r	KISS1 receptor, transcript variant X5	NA	-0.04868	2.113048	-1.03432	0.76707265	0.912213
81701	Egfl8	EGF-like domain 8	NA	-0.06601	1.683065	-1.04682	0.76734649	0.912404
102636836	Gm17202	predicted gene 17202, transcript variant X1	NA	-0.08162	0.490146	-1.05821	0.76738506	0.912404
17988	Ndrg1	N-myc downstream regulated gene 1	NA	0.018911	5.90406	1.013194	0.76740144	0.912404
18193	Nsd1	nuclear receptor-binding SET-domain protein 1, trans	NA	0.015171	7.789904	1.010571	0.76754904	0.912512
433256	Acsl5	acyl-CoA synthetase long-chain family member 5, tra	NA	-0.02396	4.38109	-1.01675	0.76769071	0.912614
232288	Frmd4b	FERM domain containing 4B, transcript variant X5	NA	-0.02327	6.293453	-1.01626	0.76781513	0.912695
66989	Kctd20	potassium channel tetramerisation domain containing	NA	-0.01554	7.096766	-1.01083	0.76796041	0.912794
19124	Procr	protein C receptor, endothelial	NA	0.064211	0.920471	1.045513	0.76809086	0.912794
22418	Wnt5a	wingless-type MMTV integration site family, member	NA	0.020884	4.960301	1.014581	0.76811974	0.912794
102640667	Gm36681	predicted gene, 36681	NA	0.079952	0.500962	1.056983	0.76812209	0.912794
18129	Notch2	notch 2	NA	-0.01756	5.886687	-1.01224	0.76822031	0.912809
100534296	Gm9805	predicted gene 9805, transcript variant 1	NA	0.075894	0.714082	1.054014	0.76824687	0.912809
115488942	Gm29417	predicted gene 29417	NA	-0.10663	-0.33085	-1.07671	0.76840055	0.912924
18673	Phb	prohibitin	NA	-0.0165	6.626764	-1.0115	0.76855742	0.912986
74670	Zfp943	zinc finger prtoein 943	NA	0.022111	4.484655	1.015445	0.76856432	0.912986
74195	Elp3	elongator acetyltransferase complex subunit 3, transc	NA	0.016886	7.04764	1.011773	0.76863688	0.913005
99311	Commd7	COMM domain containing 7, transcript variant 1	NA	-0.01628	5.518029	-1.01135	0.76872511	0.913043
16859	Lgals9	lectin, galactose binding, soluble 9, transcript variant	NA	-0.04043	2.54658	-1.02842	0.76889871	0.913051
22209	Ube2a	ubiquitin-conjugating enzyme E2A, transcript variant	NA	-0.01933	5.333113	-1.01349	0.76892724	0.913051
381373	Sp9	trans-acting transcription factor 9	NA	0.027032	4.747056	1.018914	0.76903776	0.913051
269198	Nbeal1	neurobeachin like 1, transcript variant X17	NA	0.028746	4.784311	1.020125	0.76904634	0.913051
102636299	Gm9836	predicted pseudogene 9836	NA	0.115	0.26405	1.082975	0.76911485	0.913051
227099	Pms1	PMS1 homolog 1, mismatch repair system componer	NA	0.038147	2.786179	1.026794	0.76915693	0.913051
21981	Ppp1r13b	protein phosphatase 1, regulatory subunit 13B	NA	0.018089	6.183456	1.012617	0.76918651	0.913051
244923	Klhl31	kelch-like 31	NA	-0.11596	-0.06343	-1.0837	0.76919234	0.913051
100972	Rab28	RAB28, member RAS oncogene family, transcript vai	NA	0.02049	5.21638	1.014304	0.76923656	0.913051
246179	Fktn	fukutin, transcript variant X11	NA	0.017871	5.225086	1.012464	0.76946621	0.91312
110893	Slc8a3	solute carrier family 8 (sodium/calcium exchanger), m	NA	0.018034	5.164327	1.012579	0.76954034	0.91312
72544	Exosc6	exosome component 6	NA	0.078778	2.793389	1.056123	0.76955618	0.91312
78772	Hhipl2	hedgehog interacting protein-like 2, transcript variant	NA	-0.08609	0.074778	-1.06149	0.76956354	0.91312
75608	Chmp4b	charged multivesicular body protein 4B	NA	0.018989	7.119408	1.013249	0.76963996	0.91312
109163	3010003L21Rik	RIKEN cDNA 3010003L21 gene	NA	-0.07087	0.720686	-1.05035	0.76968388	0.91312
67819	Derl1	Der1-like domain family, member 1	NA	0.017063	6.235375	1.011897	0.76968768	0.91312
15216	Hfe	homeostatic iron regulator, transcript variant 1	NA	0.059832	1.878309	1.042345	0.76989266	0.913297
53612	Vti1b	vesicle transport through interaction with t-SNAREs 1	NA	0.014344	6.180737	1.009992	0.77010661	0.913484
22722	Zfp64	zinc finger protein 64, transcript variant 1	NA	-0.02049	4.579581	-1.01431	0.77020353	0.913532
20340	Glg1	golgi apparatus protein 1, transcript variant 2	NA	0.013592	7.808095	1.009466	0.77027133	0.913546
11777	Ap3s1	adaptor-related protein complex 3, sigma 1 subunit, t	NA	-0.01984	6.92769	-1.01384	0.77042683	0.913642
77041	Arsk	arylsulfatase K, transcript variant X1	NA	0.035581	2.828606	1.02497	0.77046392	0.913642
66491	Polr2l	polymerase (RNA) II (DNA directed) polypeptide L	NA	-0.02317	4.474677	-1.01619	0.77054183	0.913644
104771	Jkamp	JNK1/MAPK8-associated membrane protein, transcri	NA	0.015872	5.640656	1.011062	0.77059237	0.913644
217207	Dhx8	DEAH (Asp-Glu-Ala-His) box polypeptide 8	NA	-0.01616	5.744323	-1.01126	0.77063415	0.913644
217721	Flvcr2	feline leukemia virus subgroup C cellular receptor 2	NA	0.047617	2.411127	1.033556	0.77069891	0.913654
18133	Ccn3	cellular communication network factor 3	NA	0.044089	3.820165	1.031032	0.77109357	0.914055
102639186	Gm26644	predicted gene, 26644, transcript variant 1	NA	0.040967	2.109019	1.028804	0.77141803	0.914132
64290	Foxb1	forkhead box B1	NA	0.02931	3.271567	1.020524	0.77148282	0.914132
56622	Adam21	a disintegrin and metallopeptidase domain 21	NA	-0.10404	0.05813	-1.07478	0.77155184	0.914132
105245941	Gm41313	predicted gene, 41313	NA	0.057475	1.30195	1.040643	0.77158493	0.914132
26462	Txnrd2	thioredoxin reductase 2, transcript variant 1	NA	0.030879	3.732669	1.021634	0.77159234	0.914132
76969	Chst1	carbohydrate sulfotransferase 1, transcript variant 3	NA	0.02223	5.91084	1.015528	0.77159665	0.914132
207819	Bnip5	BCL2 interacting protein 5	NA	0.05022	1.722764	1.035423	0.77159789	0.914132

257635	Sdsl	serine dehydratase-like, transcript variant X2	NA	0.069931	1.357383	1.049667	0.77160817	0.914132
115490215	Gm52832	predicted gene, 52832	NA	0.103395	-0.06428	1.074299	0.7717101	0.914145
13358	Slc25a1	solute carrier family 25 (mitochondrial carrier, citrate t	NA	-0.0175	6.279218	-1.01221	0.77175379	0.914145
74096	Hvcn1	hydrogen voltage-gated channel 1, transcript variant	NA	0.074941	0.959938	1.053318	0.77183887	0.914145
227648	Sec16a	SEC16 homolog A, endoplasmic reticulum export fac	NA	-0.01593	6.500356	-1.01111	0.77187187	0.914145
19684	Rdx	radixin, transcript variant 2	NA	0.013444	7.094352	1.009362	0.77198656	0.914145
59028	Rcl1	RNA terminal phosphate cyclase-like 1	NA	-0.02899	4.181666	-1.0203	0.77200267	0.914145
20853	Stau1	staufen double-stranded RNA binding protein 1, trans	NA	0.014265	7.196415	1.009937	0.77211809	0.914145
20826	Snu13	SNU13 homolog, small nuclear ribonucleoprotein (U4	NA	0.016293	6.91723	1.011358	0.7721871	0.914145
11908	Atf1	activating transcription factor 1	NA	-0.02728	4.504563	-1.01909	0.77227662	0.914145
331401	Thoc2	THO complex 2	NA	0.016167	7.048066	1.011269	0.77232049	0.914145
74931	4930481A15Rik	RIKEN cDNA 4930481A15 gene, transcript variant 1	NA	-0.08954	0.266631	-1.06403	0.77232907	0.914145
232449	Dera	deoxyribose-phosphate aldolase (putative)	NA	0.048727	2.577977	1.034352	0.77234228	0.914145
115488771	Gm52287	predicted gene, 52287	NA	0.095295	-0.19153	1.068284	0.77234899	0.914145
70481	Pnma1	paraneoplastic antigen MA1	NA	-0.04946	2.368919	-1.03487	0.772453	0.914201
12336	Capns1	calpain, small subunit 1	NA	-0.01411	6.867687	-1.00983	0.77256211	0.914248
210711	Mcmbp	minichromosome maintenance complex binding prote	NA	0.017138	6.114636	1.01195	0.77260442	0.914248
52633	Nit2	nitrilase family, member 2	NA	0.029049	4.144233	1.020339	0.77278689	0.914292
71793	Ints12	integrator complex subunit 12	NA	-0.022	4.485411	-1.01536	0.77279653	0.914292
224796	Clic5	chloride intracellular channel 5	NA	-0.09839	0.106791	-1.07058	0.7728107	0.914292
102636745	Gm33733	predicted gene, 33733, transcript variant X1	NA	0.103914	0.089764	1.074685	0.77300067	0.914309
56544	Vmn2r1	vomeronasal 2, receptor 1, transcript variant X10	NA	-0.06761	1.181331	-1.04798	0.77304438	0.914309
380614	Intu	inturned planar cell polarity protein	NA	0.025993	3.940138	1.01818	0.77308547	0.914309
12922	Crhr2	corticotropin releasing hormone receptor 2, transcript	NA	0.087443	0.079816	1.062485	0.7731012	0.914309
67219	Med18	mediator complex subunit 18	NA	0.035435	2.999694	1.024866	0.77310546	0.914309
231991	Creb5	cAMP responsive element binding protein 5, transcrip	NA	-0.03123	3.522177	-1.02188	0.77317885	0.914329
68346	Sirt5	sirtuin 5	NA	0.029256	3.737653	1.020486	0.77324329	0.914339
66067	Gtpbp8	GTP-binding protein 8 (putative), transcript variant 1	NA	0.033963	2.985826	1.02382	0.77335512	0.914404
70989	Jhy	junctional cadherin complex regulator, transcript varia	NA	-0.06747	0.711484	-1.04788	0.77351351	0.914525
16202	Ilk	integrin linked kinase, transcript variant 1	NA	-0.01467	6.884574	-1.01022	0.77384501	0.914809
106369	Ypel1	yippee like 1, transcript variant 2	NA	0.016386	6.443562	1.011423	0.77386621	0.914809
70465	Wdr77	WD repeat domain 77	NA	0.013789	6.349895	1.009604	0.77396347	0.914858
77006	Ddrgk1	DDRGK domain containing 1	NA	0.01892	5.027266	1.013201	0.77430268	0.915192
72151	Rfc5	replication factor C (activator 1) 5	NA	-0.02449	4.204065	-1.01712	0.77446565	0.91526
12224	Klf5	Kruppel-like factor 5	NA	0.049518	1.749976	1.034919	0.77450459	0.91526
115486533	LOC115486533	uncharacterized LOC115486533	NA	0.025492	4.141388	1.017827	0.77452879	0.91526
666938	Bend4	BEN domain containing 4	NA	-0.02597	4.614422	-1.01817	0.77474118	0.915276
65103	Arl6ip6	ADP-ribosylation factor-like 6 interacting protein 6	NA	0.022399	4.803623	1.015647	0.77481482	0.915276
15278	Tfb2m	transcription factor B2, mitochondrial, transcript varia	NA	0.021038	4.896425	1.01469	0.77483031	0.915276
77809	Lrrc42	leucine rich repeat containing 42	NA	0.019645	5.257536	1.01371	0.77485517	0.915276
239188	Enox1	ecto-NOX disulfide-thiol exchanger 1, transcript varia	NA	-0.01874	5.562186	-1.01308	0.77508124	0.915276
12402	Cbl	Casitas B-lineage lymphoma, transcript variant X3	NA	-0.01556	6.518167	-1.01085	0.77512755	0.915276
107585	Dio3	deiodinase, iodothyronine type III	NA	-0.05926	1.943595	-1.04193	0.77516112	0.915276
11772	Ap2a2	adaptor-related protein complex 2, alpha 2 subunit, tr	NA	0.013848	7.548543	1.009645	0.77517653	0.915276
14853	Gspt2	G1 to S phase transition 2	NA	0.023687	4.455797	1.016554	0.77521102	0.915276
217143	Gpr179	G protein-coupled receptor 179	NA	0.086317	0.709503	1.061656	0.77521278	0.915276
27260	Plek2	pleckstrin 2	NA	-0.08833	-0.0457	-1.06314	0.77521472	0.915276
235043	Tmem205	transmembrane protein 205, transcript variant 2	NA	-0.03542	2.754372	-1.02485	0.77522579	0.915276
56722	Litaf	LPS-induced TN factor	NA	0.024029	4.357113	1.016795	0.77527383	0.915276
66926	Trmt6	tRNA methyltransferase 6, transcript variant 1	NA	0.028677	4.780877	1.020076	0.77544925	0.915278
22190	Ubc	ubiquitin C	NA	0.016383	9.397564	1.011421	0.77545161	0.915278
14645	Glul	glutamate-ammonia ligase (glutamine synthetase)	NA	0.013232	7.908426	1.009214	0.77548199	0.915278
320208	Tmem91	transmembrane protein 91, transcript variant 2	NA	-0.03872	2.713044	-1.0272	0.77550015	0.915278
59069	Tpm3	tropomyosin 3, gamma, transcript variant 3	NA	0.014351	8.113634	1.009997	0.77566107	0.915369
66559	Metap1d	methionyl aminopeptidase type 1D (mitochondrial), tr	NA	-0.01959	4.934701	-1.01367	0.77569005	0.915369
71877	Efhc1	EF-hand domain (C-terminal) containing 1	NA	-0.05633	1.487574	-1.03982	0.77583313	0.915468
102632737	Gm30732	predicted gene, 30732	NA	0.107356	-0.32593	1.077252	0.77596469	0.915468
109145	Gins4	GINS complex subunit 4 (Sld5 homolog)	NA	-0.02746	4.31347	-1.01922	0.77598058	0.915468
232946	Bloc1s3	biogenesis of lysosomal organelles complex-1, subur	NA	-0.02524	3.802501	-1.01765	0.77602649	0.915468
228715	Smim26	small integral membrane protein 26	NA	-0.03281	2.83999	-1.023	0.776087	0.915468
52430	Echdc2	enoyl Coenzyme A hydratase domain containing 2, tr	NA	-0.0414	2.812627	-1.02911	0.77625457	0.915468
140579	Elmo2	engulfment and cell motility 2, transcript variant X2	NA	-0.01372	6.554324	-1.00955	0.7762884	0.915468
58185	Rsad2	radical S-adenosyl methionine domain containing 2	NA	0.099551	-0.20232	1.07144	0.77634811	0.915468
100101806	Srp54c	signal recognition particle 54C, transcript variant 2	NA	-0.01764	6.088335	-1.01231	0.77635733	0.915468
102640439	Gm36500	predicted gene, 36500, transcript variant X4	NA	-0.08527	0.366258	-1.06089	0.77638553	0.915468
67785	Zmym4	zinc finger, MYM-type 4, transcript variant X2	NA	0.014596	6.774947	1.010169	0.77639213	0.915468
74199	Vit	vitrin, transcript variant X1	NA	-0.0267	4.03814	-1.01868	0.77647748	0.915502
108015	Chrnb4	cholinergic receptor, nicotinic, beta polypeptide 4	NA	-0.05704	1.078929	-1.04033	0.77654354	0.915513

74843	Mss51	MSS51 mitochondrial translational activator, transcrip	NA	-0.09046	0.580674	-1.06471	0.77663211	0.915552
110119	Mpi	mannose phosphate isomerase, transcript variant 1	NA	0.017722	5.116351	1.01236	0.77670971	0.915577
67554	Slc25a30	solute carrier family 25, member 30	NA	0.035906	2.629037	1.025201	0.7769563	0.915747
21950	Tnfsf9	tumor necrosis factor (ligand) superfamily, member 9	NA	0.046979	1.740471	1.033099	0.77699938	0.915747
66317	Wdr61	WD repeat domain 61, transcript variant 1	NA	0.017534	5.239345	1.012227	0.77705538	0.915747
110379	Sec13	SEC13 homolog, nuclear pore and COPII coat compl	NA	0.016679	6.423191	1.011628	0.77710382	0.915747
330863	Trim67	tripartite motif-containing 67	NA	0.013393	8.356212	1.009326	0.7771587	0.915747
11838	Arc	activity regulated cytoskeletal-associated protein, trar	NA	-0.05175	1.660257	-1.03653	0.77724399	0.915747
12812	Coil	coilin	NA	0.018239	5.193046	1.012722	0.77724807	0.915747
102638470	Gm35025	predicted gene, 35025	NA	0.094025	0.053467	1.067344	0.77747483	0.915851
20768	Sephs2	selenophosphate synthetase 2	NA	0.019655	5.208904	1.013717	0.77749744	0.915851
13038	Ctsk	cathepsin K	NA	-0.04767	2.8153	-1.03359	0.77750783	0.915851
381560	Xkr8	X-linked Kx blood group related 8, transcript variant X	NA	0.034685	2.871794	1.024333	0.77757091	0.915851
66866	Nhlrc2	NHL repeat containing 2, transcript variant X2	NA	-0.01865	5.066293	-1.01301	0.77761757	0.915851
100862375	Entpd4b	ectonucleoside triphosphate diphosphohydrolase 4B,	NA	-0.03028	4.88581	-1.02121	0.77805518	0.916235
99890	Prmt6	protein arginine N-methyltransferase 6, transcript vari	NA	0.020042	4.656838	1.013989	0.77807464	0.916235
243328	Slc29a4	solute carrier family 29 (nucleoside transporters), me	NA	0.014184	6.629046	1.00988	0.77812693	0.916235
228980	Taf4	TATA-box binding protein associated factor 4, transc	NA	-0.01851	5.573606	-1.01291	0.77826974	0.916235
22644	Rnf103	ring finger protein 103, transcript variant 1	NA	-0.02154	5.579848	-1.01505	0.77831999	0.916235
56375	B4galt4	UDP-Gal:betaGlcNAc beta 1,4-galactosyltransferase,	NA	-0.02369	4.650064	-1.01656	0.77833013	0.916235
52184	Odf2l	outer dense fiber of sperm tails 2-like, transcript varia	NA	-0.02706	4.141031	-1.01893	0.77835652	0.916235
57437	Golga7	golgi autoantigen, golgin subfamily a, 7, transcript va	NA	0.013647	6.920982	1.009504	0.77839428	0.916235
72198	Mtrex	Mtr4 exosome RNA helicase	NA	0.01378	6.350885	1.009597	0.77853492	0.916319
100044509	Tgfbr3l	transforming growth factor, beta receptor III-like, tran	NA	-0.05938	1.444272	-1.04202	0.77857815	0.916319
14132	Fcgrt	Fc receptor, IgG, alpha chain transporter, transcript v	NA	-0.0261	3.70479	-1.01825	0.77863604	0.916321
69297	Lrrc46	leucine rich repeat containing 46	NA	0.057134	1.292253	1.040397	0.77875467	0.916394
69239	Pdzph1	PDZ and pleckstrin homology domains 1	NA	0.062792	1.024721	1.044485	0.77904038	0.916447
94094	Trim34a	tripartite motif-containing 34A, transcript variant X2	NA	-0.0721	0.862131	-1.05124	0.7790419	0.916447
14236	Foxn2	forkhead box N2, transcript variant X3	NA	-0.02334	4.251771	-1.01631	0.77904608	0.916447
102638425	Gm34996	predicted gene, 34996	NA	-0.05132	1.867876	-1.03622	0.77905827	0.916447
17532	Mras	muscle and microspikes RAS, transcript variant X3	NA	0.016304	6.863563	1.011365	0.77908105	0.916447
14547	Gdap2	ganglioside-induced differentiation-associated-protei	NA	-0.02187	4.696506	-1.01528	0.77913896	0.916449
66048	Emc6	ER membrane protein complex subunit 6, transcript v	NA	-0.02062	5.075585	-1.0144	0.77941857	0.91648
71169	Nbas	neuroblastoma amplified sequence, transcript variant	NA	-0.01754	5.079363	-1.01223	0.77948502	0.91648
70640	Dcp2	decapping mRNA 2	NA	-0.01666	5.800293	-1.01161	0.77952888	0.91648
23836	Cdh20	cadherin 20	NA	0.039674	3.277753	1.027881	0.77953078	0.91648
66590	Farsa	phenylalanyl-tRNA synthetase, alpha subunit, transc	NA	0.015689	5.730623	1.010934	0.77959002	0.91648
66388	Cutc	cutC copper transporter, transcript variant 1	NA	0.045574	2.035797	1.032094	0.77960954	0.91648
81906	Cyp4x1	cytochrome P450, family 4, subfamily x, polypeptide	NA	-0.03126	3.754344	-1.0219	0.77975366	0.91648
115488163	Gm52047	predicted gene, 52047, transcript variant X1	NA	-0.09321	0.001198	-1.06674	0.77977526	0.91648
170721	Papln	papilin, proteoglycan-like sulfated glycoprotein, trans	NA	-0.07428	0.748589	-1.05284	0.77982401	0.91648
231042	Nupl2	nucleoporin like 2, transcript variant 1	NA	0.027531	3.580496	1.019266	0.77995621	0.91648
22678	Zfp2	zinc finger protein 2, transcript variant 4	NA	-0.01674	5.668638	-1.01167	0.77996418	0.91648
23894	Gtf2h2	general transcription factor II H, polypeptide 2, transc	NA	-0.01996	4.785286	-1.01393	0.78002196	0.91648
319622	Itpripl2	inositol 1,4,5-triphosphate receptor interacting protei	NA	-0.0285	3.346373	-1.01995	0.78002758	0.91648
13171	Dbt	dihydrolipoamide branched chain transacylase E2, tr	NA	0.023459	4.200735	1.016394	0.78002869	0.91648
20383	Srsf3	serine and arginine-rich splicing factor 3, transcript va	NA	-0.01223	8.783403	-1.00851	0.78004353	0.91648
104156	Etv5	ets variant 5, transcript variant 2	NA	-0.01751	5.343645	-1.01221	0.78006656	0.91648
18453	P4hb	prolyl 4-hydroxylase, beta polypeptide	NA	0.014209	8.015089	1.009898	0.78025496	0.916585
225392	Rell2	RELT-like 2	NA	-0.0156	5.702287	-1.01087	0.78034734	0.916585
59048	C1galt1c1	C1GALT1-specific chaperone 1	NA	-0.02582	3.698891	-1.01806	0.78041745	0.916585
623169	Gm6402	predicted pseudogene 6402	NA	-0.03687	2.908945	-1.02589	0.78043019	0.916585
231872	Aimp2	aminoacyl tRNA synthetase complex-interacting mult	NA	-0.02521	4.622027	-1.01763	0.78043806	0.916585
114674	Gtf2ird2	GTF2I repeat domain containing 2, transcript variant	NA	0.018639	4.792246	1.013003	0.78068734	0.916749
382038	Urb2	URB2 ribosome biogenesis 2 homolog (S. cerevisiae	NA	0.022829	4.262793	1.01595	0.78073538	0.916749
76642	1700113A16Rik	RIKEN cDNA 1700113A16 gene	NA	-0.06511	0.702761	-1.04616	0.78074643	0.916749
71145	Scara5	scavenger receptor class A, member 5, transcript var	NA	-0.05494	1.678085	-1.03882	0.78084434	0.916756
234875	Ttc13	tetratricopeptide repeat domain 13, transcript variant	NA	0.016825	5.966752	1.01173	0.78091381	0.916756
269959	Adamtsl3	ADAMTS-like 3	NA	0.057646	1.154688	1.040766	0.78096507	0.916756
53867	Col5a3	collagen, type V, alpha 3, transcript variant 2	NA	-0.04291	2.446357	-1.03019	0.78097785	0.916756
102636035	Gm33219	predicted gene, 33219	NA	0.116986	0.411218	1.084467	0.78122408	0.916979
665700	Hmcn2	hemicentin 2, transcript variant X3	NA	0.087348	0.046134	1.062416	0.78145601	0.917185
18217	Ntsr2	neurotensin receptor 2, transcript variant X1	NA	0.089615	0.032059	1.064086	0.78166549	0.917347
19771	Rlbp1	retinaldehyde binding protein 1, transcript variant 1	NA	0.021645	5.278861	1.015116	0.78170689	0.917347
67724	Pop1	processing of precursor 1, ribonuclease P/MRP famil	NA	-0.02807	3.786023	-1.01965	0.7822452	0.917913
108954	Ppp1r15b	protein phosphatase 1, regulatory subunit 15B	NA	0.01363	6.375182	1.009492	0.78239279	0.91802
56808	Cacna2d2	calcium channel, voltage-dependent, alpha 2/delta su	NA	-0.01606	6.805533	-1.01119	0.78259397	0.91818
100504029	Gm16740	predicted gene, 16740	NA	0.103821	-0.2855	1.074616	0.7827114	0.91818

115486927	Gm51569	predicted gene, 51569	NA	0.088265	0.032652	1.063091	0.78274599	0.91818
106581	Fam234a	family with sequence similarity 234, member A, transc	NA	-0.02055	4.685082	-1.01434	0.78283553	0.91818
14026	Evl	Ena-vasodilator stimulated phosphoprotein, transcript	NA	0.012717	8.751179	1.008854	0.78291277	0.91818
18221	Nudc	nudC nuclear distribution protein	NA	-0.0137	6.902851	-1.00954	0.78291615	0.91818
218121	Mboat1	membrane bound O-acyltransferase domain containi	NA	0.062003	1.486122	1.043914	0.78295256	0.91818
74666	4930432K21Rik	RIKEN cDNA 4930432K21 gene, transcript variant X	NA	0.05731	1.41533	1.040524	0.78303173	0.91818
76976	Arxes2	adipocyte-related X-chromosome expressed sequenc	NA	0.018268	6.730144	1.012743	0.78303722	0.91818
11539	Adora1	adenosine A1 receptor, transcript variant 5	NA	-0.01706	5.363927	-1.0119	0.78309496	0.918181
50767	Pnpla6	patatin-like phospholipase domain containing 6, trans	NA	-0.01771	5.593735	-1.01235	0.78331635	0.918353
381413	Gpr176	G protein-coupled receptor 176	NA	0.028139	3.619716	1.019696	0.78335436	0.918353
15929	Idh3g	isocitrate dehydrogenase 3 (NAD+), gamma, transcri	NA	0.018516	6.300335	1.012917	0.7838445	0.918862
51793	Ddah2	dimethylarginine dimethylaminohydrolase 2, transcrip	NA	-0.01647	7.058422	-1.01148	0.78398216	0.918918
320961	A630026N12Rik	RIKEN cDNA A630026N12 gene	NA	0.035726	3.128944	1.025073	0.78400528	0.918918
382051	Pdp2	pyruvate dehyrogenase phosphatase catalytic subuni	NA	-0.02001	4.858802	-1.01396	0.7840981	0.918944
170763	Zfp87	zinc finger protein 87	NA	0.021935	4.524268	1.01532	0.78414061	0.918944
70417	Megf10	multiple EGF-like-domains 10	NA	-0.02887	4.73821	-1.02021	0.78420572	0.918954
633979	Ak9	adenylate kinase 9	NA	-0.09213	-0.01627	-1.06594	0.78428173	0.918977
68260	Trmt12	tRNA methyltransferase 12	NA	0.036965	3.130714	1.025953	0.78437321	0.919014
18974	Pole2	polymerase (DNA directed), epsilon 2 (p59 subunit)	NA	-0.04093	2.411552	-1.02878	0.78442658	0.919014
216820	Dhrs7b	dehydrogenase/reductase (SDR family) member 7B,	NA	0.030578	3.767667	1.021421	0.78461953	0.919109
101835	AW146154	expressed sequence AW146154	NA	0.039181	2.243697	1.027531	0.78462069	0.919109
93739	Gabarapl2	gamma-aminobutyric acid (GABA) A receptor-associa	NA	-0.0125	6.97513	-1.0087	0.78468706	0.919109
330485	Tmem145	transmembrane protein 145	NA	-0.01584	6.584734	-1.01104	0.78473366	0.919109
244198	Olfml1	olfactomedin-like 1	NA	0.056745	1.631671	1.040116	0.78486168	0.919193
18718	Pip4k2a	phosphatidylinositol-5-phosphate 4-kinase, type II, al	NA	0.016711	5.05344	1.011651	0.78494108	0.91922
54484	Mkrn1	makorin, ring finger protein, 1, transcript variant 1	NA	0.013692	7.76182	1.009536	0.78501598	0.919242
192678	Rassf3	Ras association (RalGDS/AF-6) domain family memb	NA	0.024753	3.93595	1.017305	0.78521737	0.919411
17164	Mapkapk2	MAP kinase-activated protein kinase 2	NA	0.018074	5.907127	1.012607	0.78538691	0.919514
78372	Snrnp25	small nuclear ribonucleoprotein 25 (U11/U12)	NA	0.032995	3.446031	1.023134	0.78541824	0.919514
75746	Morc4	microrchidia 4, transcript variant B	NA	0.034949	2.861269	1.024521	0.78553151	0.91958
19701	Ren1	renin 1 structural	NA	-0.10904	-0.26678	-1.07851	0.78568754	0.919622
13384	Mpp3	membrane protein, palmitoylated 3 (MAGUK p55 sub	NA	-0.01748	5.442757	-1.01219	0.78570471	0.919622
70317	Arl16	ADP-ribosylation factor-like 16	NA	0.022345	4.114193	1.015609	0.78573761	0.919622
234395	Ushbp1	USH1 protein network component harmonin binding p	NA	-0.03993	2.517484	-1.02806	0.78589436	0.919622
93874	Pcdhb3	protocadherin beta 3	NA	-0.03811	2.50823	-1.02677	0.78589728	0.919622
118568317	LOC118568317	uncharacterized LOC118568317	NA	-0.06207	1.166459	-1.04397	0.78590564	0.919622
214585	Spg11	SPG11, spatacsin vesicle trafficking associated	NA	0.021944	4.767667	1.015327	0.78598697	0.919651
118568479	LOC118568479	uncharacterized LOC118568479	NA	-0.1263	0.519342	-1.09149	0.78616651	0.919752
52668	Ifi27	interferon, alpha-inducible protein 27, transcript varia	NA	-0.05813	1.55166	-1.04112	0.7862546	0.919752
70579	Zc3h11a	zinc finger CCCH type containing 11A, transcript vari	NA	-0.01301	7.444533	-1.00906	0.78628023	0.919752
329375	Cfap77	cilia and flagella associated protein 77	NA	-0.10088	-0.34449	-1.07243	0.78632227	0.919752
330323	Mindy4	MINDY lysine 48 deubiquitinase 4, transcript variant	NA	0.038427	3.253665	1.026993	0.7863559	0.919752
225030	Kcng3	potassium voltage-gated channel, subfamily G, meml	NA	0.047008	2.304496	1.03312	0.78655565	0.919919
70383	Cox10	heme A:farnesyltransferase cytochrome c oxidase as	NA	0.032063	4.395648	1.022473	0.78673004	0.919988
229524	Msto1	misato 1, mitochondrial distribution and morphology r	NA	-0.02083	4.586538	-1.01454	0.78683703	0.919988
13481	Dpm2	dolichol-phosphate (beta-D) mannosyltransferase 2	NA	-0.02159	4.902979	-1.01508	0.78688417	0.919988
66423	Coprs	coordinator of PRMT5, differentiation stimulator, trans	NA	0.02397	4.293349	1.016754	0.78691274	0.919988
223665	C030006K11Rik	RIKEN cDNA C030006K11 gene, transcript variant 1	NA	-0.03831	2.669986	-1.02691	0.78701912	0.919988
72324	Plxdc1	plexin domain containing 1, transcript variant X2	NA	-0.06209	0.890513	-1.04398	0.78707103	0.919988
213109	Phf3	PHD finger protein 3, transcript variant X15	NA	-0.01523	6.691157	-1.01061	0.78714255	0.919988
21933	Tnfrsf10b	tumor necrosis factor receptor superfamily, member	NA	-0.06331	1.085708	-1.04486	0.78716282	0.919988
11443	Chrnb1	cholinergic receptor, nicotinic, beta polypeptide 1 (mu	NA	-0.05641	0.937243	-1.03987	0.78717568	0.919988
16527	Kcnk3	potassium channel, subfamily K, member 3	NA	0.033673	3.756138	1.023615	0.78727046	0.919988
68267	Slc25a22	solute carrier family 25 (mitochondrial carrier, glutama	NA	-0.01422	6.478459	-1.00991	0.78737613	0.919988
81601	Kat5	K(lysine) acetyltransferase 5, transcript variant 1	NA	0.013821	6.110606	1.009626	0.78738397	0.919988
21454	Tcp1	t-complex protein 1, transcript variant 1	NA	0.011791	8.418027	1.008206	0.7874274	0.919988
319998	Tmem198	transmembrane protein 198	NA	0.021874	4.438181	1.015277	0.78753608	0.919988
102636414	Gm38487	predicted gene, 38487	NA	-0.08192	0.130806	-1.05843	0.78761492	0.919988
319939	Tns3	tensin 3, transcript variant X11	NA	0.01677	5.154405	1.011692	0.78761904	0.919988
73420	Ccsap	centriole, cilia and spindle associated protein, transcr	NA	-0.02822	3.556823	-1.01975	0.78765083	0.919988
192976	Lrrc75a	leucine rich repeat containing 75A, transcript variant	NA	-0.02229	4.137845	-1.01557	0.78768596	0.919988
546071	Mast3	microtubule associated serine/threonine kinase 3, tra	NA	-0.01523	5.734984	-1.01061	0.78773489	0.919988
100041286	Snhg15	small nucleolar RNA host gene 15	NA	0.072669	1.053135	1.05166	0.78774505	0.919988
242050	Igsf10	immunoglobulin superfamily, member 10	NA	0.027094	4.231524	1.018957	0.78781118	0.919999
102636892	Gm33841	predicted gene, 33841, transcript variant X4	NA	0.109591	-0.55004	1.078922	0.78799631	0.920076
11922	Neurod6	neurogenic differentiation 6	NA	0.022553	8.346202	1.015755	0.78802866	0.920076
56692	Lamtor3	late endosomal/lysosomal adaptor, MAPK and MTOF	NA	-0.01548	5.580411	-1.01079	0.78804652	0.920076
259279	Tubgcp3	tubulin, gamma complex associated protein 3, transc	NA	-0.01495	5.759075	-1.01042	0.7881482	0.920128

73390	Msl3l2	MSL3 like 2	NA	0.025438	3.541931	1.017789	0.78829588	0.920223
16535	Kcnq1	potassium voltage-gated channel, subfamily Q, meml	NA	0.073532	0.528957	1.05229	0.78834596	0.920223
100503605	Hbb-bs	hemoglobin, beta adult s chain	NA	-0.06309	10.93479	-1.0447	0.78849298	0.920223
214616	Spata5l1	spermatogenesis associated 5-like 1, transcript variai	NA	0.025219	3.640886	1.017634	0.78850657	0.920223
74463	Exoc3l2	exocyst complex component 3-like 2	NA	-0.09024	-0.17625	-1.06455	0.78856771	0.920223
73467	1700066M21Rik	RIKEN cDNA 1700066M21 gene	NA	0.01993	4.676475	1.01391	0.78862114	0.920223
19177	Psmb7	proteasome (prosome, macropain) subunit, beta type	NA	-0.01642	7.236644	-1.01145	0.78862557	0.920223
105501	Abhd4	abhydrolase domain containing 4, transcript variant 1	NA	-0.01642	5.397409	-1.01145	0.78870323	0.920248
319817	Rc3h2	ring finger and CCCH-type zinc finger domains 2, trar	NA	-0.01487	7.130189	-1.01036	0.78885216	0.92026
72774	Neil1	nei endonuclease VIII-like 1 (E. coli), transcript variar	NA	0.036225	3.066138	1.025427	0.78887563	0.92026
51789	Tnk2	tyrosine kinase, non-receptor, 2, transcript variant 2	NA	-0.01275	6.676179	-1.00888	0.78888348	0.92026
20669	Sox14	SRY (sex determining region Y)-box 14	NA	0.030536	3.858426	1.021392	0.78894452	0.920266
227290	Aamp	angio-associated migratory protein, transcript variant	NA	-0.01541	6.783315	-1.01074	0.78914061	0.920406
100198	H6pd	hexose-6-phosphate dehydrogenase (glucose 1-dehy	NA	0.031412	3.233004	1.022012	0.7891781	0.920406
14297	Fxn	frataxin, transcript variant 1	NA	0.022114	4.050551	1.015447	0.78935864	0.920476
170718	Idh3b	isocitrate dehydrogenase 3 (NAD+) beta, transcript va	NA	-0.0147	6.671967	-1.01024	0.78948739	0.920476
72552	Hsdl1	hydroxysteroid dehydrogenase like 1, transcript varia	NA	-0.01337	6.997927	-1.00931	0.78950576	0.920476
114874	Ddhd1	DDHD domain containing 1, transcript variant 1	NA	-0.01479	5.935768	-1.0103	0.78952553	0.920476
245474	Dkc1	dyskeratosis congenita 1, dyskerin, transcript variant	NA	-0.0182	5.189414	-1.01269	0.78960174	0.920476
195040	Tmem199	transmembrane protein 199	NA	0.019493	4.809524	1.013603	0.78963755	0.920476
238896	Cdc20b	cell division cycle 20B, transcript variant X3	NA	0.063005	0.728588	1.044639	0.78968952	0.920476
14528	Gch1	GTP cyclohydrolase 1	NA	-0.04715	1.975541	-1.03322	0.7896907	0.920476
77574	Tcaf1	TRPM8 channel-associated factor 1, transcript varian	NA	0.011715	8.48936	1.008153	0.78981645	0.920557
72344	Usp36	ubiquitin specific peptidase 36, transcript variant X9	NA	-0.01635	5.170975	-1.01139	0.78993775	0.920568
13808	Eno3	enolase 3, beta muscle, transcript variant 2	NA	0.027748	4.61853	1.01942	0.78995788	0.920568
102462	Imp3	IMP3, U3 small nucleolar ribonucleoprotein	NA	0.022532	4.574068	1.01574	0.79000811	0.920568
15502	Dnaja1	DnaJ heat shock protein family (Hsp40) member A1,	NA	0.011755	9.018625	1.008181	0.79005243	0.920568
72692	Hnrnpll	heterogeneous nuclear ribonucleoprotein L-like, trans	NA	0.015477	6.665771	1.010785	0.79016844	0.920637
100090	Zbtb48	zinc finger and BTB domain containing 48, transcript	NA	-0.02491	4.022156	-1.01742	0.79033202	0.920762
193116	Slu7	SLU7 splicing factor homolog (S. cerevisiae), transcri	NA	0.013981	5.788292	1.009738	0.79068131	0.920962
17132	Maf	avian musculoaponeurotic fibrosarcoma oncogene hc	NA	0.018662	5.38364	1.01302	0.79070548	0.920962
58186	Rad18	RAD18 E3 ubiquitin protein ligase, transcript variant 3	NA	-0.03259	2.7768	-1.02285	0.79072677	0.920962
21924	Tnnc1	troponin C, cardiac/slow skeletal	NA	-0.09619	1.20808	-1.06894	0.7907298	0.920962
64898	Lpin2	lipin 2, transcript variant 2	NA	0.016417	5.852918	1.011444	0.7908825	0.921074
12521	Cd82	CD82 antigen, transcript variant 1	NA	0.027808	3.812844	1.019462	0.79100496	0.92115
226751	Cdc42bpa	CDC42 binding protein kinase alpha, transcript variar	NA	0.013864	7.345536	1.009656	0.79118343	0.921292
211286	Cln5	ceroid-lipofuscinosis, neuronal 5	NA	-0.02542	3.392791	-1.01778	0.79124622	0.921299
73674	Wdr75	WD repeat domain 75	NA	0.01587	5.379869	1.011061	0.7918623	0.921951
56149	Grasp	GRP1 (general receptor for phosphoinositides 1)-ass	NA	0.042916	2.658526	1.030194	0.79206251	0.922051
381990	Zbtb2	zinc finger and BTB domain containing 2, transcript v	NA	-0.02126	4.682749	-1.01485	0.79210895	0.922051
59024	Med12	mediator complex subunit 12	NA	-0.01383	5.859711	-1.00963	0.79211833	0.922051
50934	Slc7a8	solute carrier family 7 (cationic amino acid transporte	NA	-0.02022	4.870621	-1.01411	0.79232137	0.922051
445007	Nup85	nucleoporin 85	NA	0.013557	5.849165	1.009441	0.79232299	0.922051
11910	Atf3	activating transcription factor 3	NA	0.082872	0.17404	1.059124	0.79233439	0.922051
73296	Rhobtb3	Rho-related BTB domain containing 3, transcript varia	NA	-0.01559	6.266533	-1.01086	0.7923454	0.922051
17160	Man2b2	mannosidase 2, alpha B2	NA	-0.02337	3.868165	-1.01633	0.79249222	0.922151
77189	A430105J06Rik	RIKEN cDNA A430105J06 gene, transcript variant X1	NA	0.05491	1.065515	1.038794	0.79262882	0.922151
105243361	Gm39308	predicted gene, 39308	NA	0.037496	2.365165	1.026331	0.79268845	0.922151
17261	Mef2d	myocyte enhancer factor 2D, transcript variant X3	NA	0.013806	6.6178	1.009615	0.79269071	0.922151
627828	Gm6793	predicted gene 6793	NA	-0.07457	6.708466	-1.05305	0.79271446	0.922151
319702	A830011K09Rik	RIKEN cDNA A830011K09 gene, transcript variant 1	NA	0.050406	1.557042	1.035556	0.79279343	0.922177
72615	Anks3	ankyrin repeat and sterile alpha motif domain contain	NA	-0.01628	5.368011	-1.01135	0.79305505	0.922276
17776	Mast2	microtubule associated serine/threonine kinase 2, tra	NA	-0.0133	7.72477	-1.00926	0.79305813	0.922276
72088	Ush1c	USH1 protein network component harmonin, transcrip	NA	-0.12159	-0.61463	-1.08793	0.79307912	0.922276
103611158	Gm38666	predicted gene, 38666	NA	0.041659	3.12426	1.029297	0.79310511	0.922276
11488	Adam11	a disintegrin and metallopeptidase domain 11, transc	NA	0.018002	5.955836	1.012556	0.79325536	0.922298
77810	A930015D03Rik	RIKEN cDNA A930015D03 gene	NA	-0.06559	0.717853	-1.04651	0.79333539	0.922298
108645	Mat2b	methionine adenosyltransferase II, beta, transcript va	NA	0.015989	5.440423	1.011144	0.79333604	0.922298
73681	Trmt11	tRNA methyltransferase 11, transcript variant 1	NA	0.025563	3.644399	1.017877	0.79335058	0.922298
78656	Brd8	bromodomain containing 8, transcript variant 2	NA	0.014423	6.748118	1.010047	0.79358084	0.922499
115487965	Gm51971	predicted gene, 51971	NA	-0.01839	5.626717	-1.01283	0.7936799	0.922549
230125	Slc25a51	solute carrier family 25, member 51	NA	0.011902	6.899261	1.008284	0.79384271	0.922618
192775	Kcnh6	potassium voltage-gated channel, subfamily H (eag-r	NA	-0.04221	2.457747	-1.02969	0.79394779	0.922618
108121	U2af1	U2 small nuclear ribonucleoprotein auxiliary factor (U	NA	-0.01682	6.354624	-1.01173	0.79394863	0.922618
107375	Slc25a45	solute carrier family 25, member 45, transcript variant	NA	0.068212	1.243368	1.048416	0.79396597	0.922618
118568284	LOC118568284	igE-binding protein-like	NA	0.064285	0.884206	1.045567	0.7942367	0.922743
71778	Klhl5	kelch-like 5, transcript variant 1	NA	0.012526	6.37593	1.00872	0.79427832	0.922743
242022	Frem2	Fras1 related extracellular matrix protein 2	NA	0.029026	3.824456	1.020323	0.79433349	0.922743

210029	Metrnl	meteorin, glial cell differentiation regulator-like	NA	-0.04048	2.138401	-1.02845	0.79442952	0.922743
67800	Dgat2	diacylglycerol O-acyltransferase 2	NA	-0.02464	3.549109	-1.01722	0.79445168	0.922743
271981	Tbck	TBC1 domain containing kinase, transcript variant X1	NA	-0.0156	5.610854	-1.01087	0.79450753	0.922743
207352	Sec23ip	Sec23 interacting protein	NA	-0.01372	5.804125	-1.00956	0.79458679	0.922743
237500	Tmtc3	transmembrane and tetratricopeptide repeat containi	NA	0.028246	4.462885	1.019772	0.79462635	0.922743
102632389	Gm24463	predicted gene, 24463, transcript variant X3	NA	0.056831	0.788352	1.040179	0.79472195	0.922743
320842	C230035I16Rik	RIKEN cDNA C230035I16 gene	NA	0.078742	-0.01041	1.056097	0.79477527	0.922743
353258	Ltv1	LTV1 ribosome biogenesis factor	NA	-0.02212	4.458043	-1.01545	0.79480771	0.922743
72018	Fundc1	FUN14 domain containing 1, transcript variant 1	NA	-0.01747	5.340164	-1.01218	0.79481272	0.922743
67736	Ccdc130	coiled-coil domain containing 130, transcript variant X	NA	-0.03391	3.39597	-1.02378	0.79491409	0.922743
14731	Gpaa1	GPI anchor attachment protein 1	NA	-0.01555	5.356769	-1.01084	0.79494765	0.922743
13610	S1pr3	sphingosine-1-phosphate receptor 3	NA	-0.02129	4.244043	-1.01487	0.79497606	0.922743
66091	Ndufa3	NADH:ubiquinone oxidoreductase subunit A3	NA	0.018998	5.127493	1.013255	0.79504428	0.922743
382117	Tcaim	T cell activation inhibitor, mitochondrial, transcript var	NA	0.030933	3.5178	1.021673	0.79504504	0.922743
58804	Cdc42ep5	CDC42 effector protein (Rho GTPase binding) 5	NA	-0.06276	0.770965	-1.04446	0.79509481	0.922743
26374	Cop1	COP1, E3 ubiquitin ligase, transcript variant X1	NA	0.012244	7.002057	1.008523	0.79553476	0.923134
229488	Fam160a1	family with sequence similarity 160, member A1	NA	-0.0589	1.054797	-1.04167	0.79559482	0.923134
768252	Foxl2os	forkhead box L2, opposite strand	NA	-0.05334	1.343075	-1.03767	0.79560206	0.923134
240590	Dmrt3	doublesex and mab-3 related transcription factor 3	NA	0.049009	1.526985	1.034554	0.7956865	0.923146
211446	Exoc3	exocyst complex component 3	NA	-0.01211	6.811685	-1.00843	0.79572576	0.923146
320203	C130071C03Rik	RIKEN cDNA C130071C03 gene, transcript variant 4	NA	0.014595	8.03182	1.010168	0.79587886	0.923193
15465	Hrh1	histamine receptor H1, transcript variant X1	NA	0.071541	0.338858	1.050839	0.79592224	0.923193
83485	Ngrn	neugrin, neurite outgrowth associated, transcript varia	NA	0.017852	5.056986	1.012451	0.79593918	0.923193
12617	Cenpc1	centromere protein C1, transcript variant 2	NA	0.014797	5.627731	1.010309	0.79599307	0.923193
102634534	Gm32091	predicted gene, 32091	NA	-0.0334	3.066192	-1.02342	0.7960664	0.923212
17938	Naca	nascent polypeptide-associated complex alpha polyp	NA	-0.01488	8.610162	-1.01036	0.79626923	0.923382
105886298	Cmc4	C-x(9)-C motif containing 4, transcript variant 2	NA	0.033951	2.673018	1.023812	0.79666829	0.923779
105246108	Gm41450	predicted gene, 41450	NA	0.061607	1.229609	1.043628	0.7968151	0.923801
115487365	Gm51751	predicted gene, 51751	NA	-0.09477	-0.17986	-1.0679	0.79684007	0.923801
11363	Acadl	acyl-Coenzyme A dehydrogenase, long-chain	NA	-0.02053	5.02233	-1.01433	0.79685771	0.923801
67489	Ap4b1	adaptor-related protein complex AP-4, beta 1, transcr	NA	0.018673	4.541352	1.013027	0.79692402	0.923812
105827	Amigo2	adhesion molecule with Ig like domain 2, transcript va	NA	0.0294	4.049566	1.020587	0.79711153	0.923834
105000	Dnal1	dynein, axonemal, light chain 1, transcript variant 1	NA	-0.01475	5.644837	-1.01028	0.79724847	0.923834
115488642	Gm52248	predicted gene, 52248	NA	0.082383	0.657915	1.058766	0.79725412	0.923834
23808	Ash2l	ASH2 like histone lysine methyltransferase complex s	NA	0.013317	6.801849	1.009274	0.79727669	0.923834
17246	Mdm2	transformed mouse 3T3 cell double minute 2, transcr	NA	-0.01299	6.119108	-1.00904	0.79733249	0.923834
170755	Sgk3	serum/glucocorticoid regulated kinase 3, transcript va	NA	0.028745	3.054278	1.020125	0.79733367	0.923834
52840	Dbndd2	dysbindin (dystrobrevin binding protein 1) domain cor	NA	0.021037	4.397512	1.014689	0.79734081	0.923834
231999	Plekha8	pleckstrin homology domain containing, family A (phc	NA	0.017767	5.237565	1.012391	0.79745576	0.923865
16396	Itch	itchy, E3 ubiquitin protein ligase, transcript variant 1	NA	0.016688	5.886427	1.011634	0.79748083	0.923865
22774	Zic4	zinc finger protein of the cerebellum 4, transcript varia	NA	-0.01478	6.411539	-1.0103	0.79762961	0.923879
70556	Slc25a33	solute carrier family 25, member 33	NA	-0.03218	3.73164	-1.02256	0.79767317	0.923879
28114	Nsun2	NOL1/NOP2/Sun domain family member 2	NA	0.014179	6.12074	1.009877	0.79786064	0.923879
69116	Ubr4	ubiquitin protein ligase E3 component n-recognin 4	NA	-0.01516	7.511139	-1.01056	0.7979214	0.923879
68178	Cgnl1	cingulin-like 1, transcript variant 2	NA	-0.02497	4.419867	-1.01746	0.79795057	0.923879
227634	Camsap1	calmodulin regulated spectrin-associated protein 1, tr	NA	0.012434	7.638189	1.008656	0.79800965	0.923879
68533	Mphosph6	M phase phosphoprotein 6, transcript variant 1	NA	-0.02276	4.033449	-1.0159	0.79801796	0.923879
17193	Mbd4	methyl-CpG binding domain protein 4, transcript varia	NA	0.023712	3.51611	1.016572	0.79804736	0.923879
105244530	Gm19391	predicted gene, 19391	NA	0.067876	0.704239	1.048173	0.79810085	0.923879
76205	Stard3nl	STARD3 N-terminal like, transcript variant X3	NA	0.018037	5.949902	1.012581	0.79811072	0.923879
55943	Stx8	syntaxin 8, transcript variant 1	NA	0.031356	4.298998	1.021972	0.79811717	0.923879
105244669	Gm40235	predicted gene, 40235	NA	0.1232	-0.04275	1.089148	0.79829147	0.923972
78610	Uvrag	UV radiation resistance associated gene	NA	-0.0172	4.710423	-1.01199	0.79832303	0.923972
69723	Rpain	RPA interacting protein, transcript variant 1	NA	-0.02738	3.37657	-1.01916	0.79836805	0.923972
101055754	Gm2974	predicted gene 2974, transcript variant X6	NA	0.09714	0.465942	1.069651	0.79852279	0.924021
105243583	Gm39468	predicted gene, 39468	NA	-0.07735	-0.0639	-1.05508	0.79855006	0.924021
320080	Zbtb39	zinc finger and BTB domain containing 39	NA	-0.01574	5.222671	-1.01097	0.7985858	0.924021
432731	Zscan26	zinc finger and SCAN domain containing 26, transcrip	NA	-0.01444	5.659919	-1.01006	0.79863774	0.924021
546058	AA386476	expressed sequence AA386476	NA	0.047377	1.660337	1.033384	0.79880855	0.924153
67201	Glod4	glyoxalase domain containing 4, transcript variant 1	NA	0.014581	6.118606	1.010158	0.79895223	0.924225
71902	Cand1	cullin associated and neddylation disassociated 1	NA	0.011473	7.364104	1.007984	0.79898463	0.924225
115486414	LOC115486414	uncharacterized LOC115486414	NA	0.070419	0.461601	1.050021	0.79908027	0.92427
67211	Armc10	armadillo repeat containing 10, transcript variant 1	NA	-0.01303	6.591869	-1.00907	0.79924044	0.924354
102636640	Gm33649	predicted gene, 33649	NA	0.019186	5.918189	1.013387	0.79928483	0.924354
67840	Mrpl57	mitochondrial ribosomal protein L57, transcript varian	NA	0.02096	4.162543	1.014634	0.79932309	0.924354
58180	Hic2	hypermethylated in cancer 2	NA	0.022965	4.648144	1.016045	0.79949274	0.924472
58226	Cacna1h	calcium channel, voltage-dependent, T type, alpha 1I	NA	0.012207	7.004597	1.008497	0.79953865	0.924472
102636360	LOC102636360	uncharacterized LOC102636360	NA	0.03482	2.696159	1.024429	0.79978054	0.924631

19769	Rit1	Ras-like without CAAX 1, transcript variant 1	NA	-0.01647	4.866807	-1.01148	0.79982058	0.924631
100504116	Brd3os	bromodomain containing 3, opposite strand, transcrip	NA	-0.01872	4.396586	-1.01306	0.79984657	0.924631
20019	Polr1a	polymerase (RNA) I polypeptide A, transcript variant :	NA	-0.01969	5.063204	-1.01374	0.80005117	0.924764
76568	Ift46	intraflagellar transport 46, transcript variant X2	NA	0.017225	6.04848	1.012011	0.80012327	0.924764
71132	Cabyr	calcium-binding tyrosine-(Y)-phosphorylation regulate	NA	0.067788	0.851962	1.048108	0.80020612	0.924764
52036	Ppp6r3	protein phosphatase 6, regulatory subunit 3, transcrip	NA	0.012337	6.892484	1.008588	0.8003968	0.924764
70561	Txndc16	thioredoxin domain containing 16, transcript variant X	NA	0.015069	5.662201	1.0105	0.80041668	0.924764
103694	Tmed4	transmembrane p24 trafficking protein 4	NA	-0.01522	5.917111	-1.0106	0.8004916	0.924764
65969	Cubn	cubilin (intrinsic factor-cobalamin receptor)	NA	0.048856	1.311894	1.034444	0.8005098	0.924764
67305	Gpx7	glutathione peroxidase 7	NA	0.024081	3.45926	1.016832	0.80051831	0.924764
66271	Tmem126a	transmembrane protein 126A, transcript variant 1	NA	0.019163	4.499397	1.013371	0.80069184	0.924764
68250	Ciao2a	cytosolic iron-sulfur assembly component 2A, transcr	NA	0.015316	5.119693	1.010673	0.80073736	0.924764
17305	Mfng	MFNG O-fucosylpeptide 3-beta-N-acetylglucosaminy	NA	-0.0192	4.406258	-1.0134	0.8007505	0.924764
56378	Arpc3	actin related protein 2/3 complex, subunit 3	NA	-0.01222	6.849149	-1.00851	0.80090681	0.924764
23892	Grem1	gremlin 1, DAN family BMP antagonist	NA	0.063952	0.424745	1.045325	0.80092981	0.924764
17268	Meis1	Meis homeobox 1, transcript variant X5	NA	-0.01861	5.601785	-1.01298	0.8010248	0.924764
244954	Prss35	protease, serine 35, transcript variant X2	NA	0.035288	2.664943	1.024762	0.80104134	0.924764
102122	Psme3ip1	proteasome activator subunit 3 interacting protein 1, t	NA	0.013457	6.13295	1.009371	0.80106861	0.924764
14406	Gabrg2	gamma-aminobutyric acid (GABA) A receptor, subuni	NA	0.015323	6.788141	1.010678	0.80107072	0.924764
11676	Aldoc	aldolase C, fructose-bisphosphate, transcript variant :	NA	-0.01338	6.713862	-1.00932	0.80112969	0.924764
83486	Rbm5	RNA binding motif protein 5, transcript variant X7	NA	0.012186	7.722185	1.008482	0.8011403	0.924764
67713	Dnajc19	DnaJ heat shock protein family (Hsp40) member C19	NA	0.024689	4.218653	1.01726	0.80115395	0.924764
238831	Ppwd1	peptidylprolyl isomerase domain and WD repeat cont	NA	0.026982	3.790974	1.018879	0.80115525	0.924764
227449	Zcchc2	zinc finger, CCHC domain containing 2, transcript var	NA	-0.01761	4.621966	-1.01228	0.8013392	0.924911
53380	Psmd10	proteasome (prosome, macropain) 26S subunit, non-	NA	0.020783	4.16205	1.01451	0.80143178	0.924928
234384	Mpv17l2	MPV17 mitochondrial membrane protein-like 2	NA	0.028867	3.03958	1.020211	0.80146757	0.924928
71521	Pds5a	PDS5 cohesin associated factor A, transcript variant :	NA	-0.01522	6.693977	-1.01061	0.80184642	0.925235
12934	Dpysl2	dihydropyrimidinase-like 2, transcript variant 2	NA	-0.01216	9.463388	-1.00847	0.80184771	0.925235
71213	Cage1	cancer antigen 1, transcript variant X3	NA	0.055561	0.973294	1.039263	0.80214812	0.925503
98388	Chst10	carbohydrate sulfotransferase 10, transcript variant 1	NA	0.013591	6.345476	1.009465	0.80224708	0.925503
224273	Crybg3	beta-gamma crystallin domain containing 3, transcrip	NA	0.033996	3.227387	1.023844	0.80227247	0.925503
72399	Brap	BRCA1 associated protein, transcript variant 1	NA	0.014452	5.734881	1.010068	0.80236217	0.925503
192176	Flna	filamin, alpha, transcript variant X4	NA	-0.01077	8.121351	-1.0075	0.80241832	0.925503
13136	Cd55	CD55 molecule, decay accelerating factor for comple	NA	-0.02932	3.114213	-1.02053	0.80243476	0.925503
105245627	Gm41056	predicted gene, 41056	NA	-0.08505	-0.0003	-1.06072	0.80247831	0.925503
71860	Cfap52	cilia and flagella associated protein 52	NA	0.042639	1.784711	1.029996	0.80261498	0.925574
115487767	Gm51891	predicted gene, 51891	NA	0.087652	-0.12486	1.062639	0.80288044	0.925574
71981	Tdrd12	tudor domain containing 12, transcript variant X3	NA	-0.07609	0.219849	-1.05416	0.80288737	0.925574
15423	Hoxc4	homeobox C4	NA	-0.08253	2.821153	-1.05887	0.80289608	0.925574
18414	Osmr	oncostatin M receptor, transcript variant X1	NA	0.033578	2.278063	1.023547	0.8029558	0.925574
84092	Usp8	ubiquitin specific peptidase 8, transcript variant 2	NA	-0.01388	5.669082	-1.00967	0.8029612	0.925574
338369	Tmem220	transmembrane protein 220, transcript variant 3	NA	-0.08183	0.101038	-1.05836	0.80304168	0.925574
66445	Cyc1	cytochrome c-1, transcript variant X1	NA	-0.0141	6.580738	-1.00982	0.80307857	0.925574
67028	2610002M06Rik	RIKEN cDNA 2610002M06 gene	NA	-0.0166	5.74395	-1.01157	0.80310243	0.925574
381845	Rnf225	ring finger protein 225	NA	0.049686	1.179508	1.03504	0.8031678	0.925574
108169083	Gm46928	predicted gene, 46928	NA	-0.11156	-0.27266	-1.08039	0.80323032	0.925574
677289	Prr33	proline rich 33	NA	0.078923	-0.16098	1.05623	0.80323891	0.925574
115487357	Gm51744	predicted gene, 51744	NA	0.040633	3.146208	1.028565	0.80331358	0.925574
58800	Trpm7	transient receptor potential cation channel, subfamily	NA	0.015486	6.025589	1.010792	0.80335178	0.925574
329727	Dennd2c	DENN/MADD domain containing 2C, transcript variar	NA	0.044703	1.549834	1.031471	0.80341028	0.925574
217707	Coq6	coenzyme Q6 monooxygenase	NA	0.020444	4.17401	1.014271	0.80344987	0.925574
14251	Flot1	flotillin 1, transcript variant X1	NA	0.014657	5.536264	1.010211	0.80356451	0.925641
18701	Pigf	phosphatidylinositol glycan anchor biosynthesis, clas	NA	-0.02777	3.476519	-1.01944	0.80376905	0.925757
11655	Alas1	aminolevulinic acid synthase 1, transcript variant 2	NA	0.015621	5.185753	1.010886	0.80377914	0.925757
212139	Cc2d1a	coiled-coil and C2 domain containing 1A, transcript va	NA	0.013999	5.923818	1.009751	0.80385052	0.925774
70237	Bhlhb9	basic helix-loop-helix domain containing, class B9, tra	NA	0.011748	6.86045	1.008176	0.80397072	0.925812
69788	1600023N17Rik	RIKEN cDNA 1600023N17 gene	NA	-0.06823	0.281196	-1.04843	0.80399782	0.925812
68800	Prr32	proline rich 32	NA	-0.04494	1.782542	-1.03164	0.80406337	0.925822
67836	Wdr83	WD repeat domain containing 83	NA	-0.02276	4.3477	-1.0159	0.80420537	0.92592
18227	Nr4a2	nuclear receptor subfamily 4, group A, member 2, tra	NA	0.014832	5.330895	1.010334	0.8044305	0.926114
76233	Dnttip1	deoxynucleotidyltransferase, terminal, interacting pro	NA	0.016306	4.898722	1.011366	0.80448987	0.926117
12494	Cd38	CD38 antigen	NA	0.053872	1.534857	1.038047	0.80464257	0.926197
28169	Agpat3	1-acylglycerol-3-phosphate O-acyltransferase 3, trans	NA	-0.01199	6.837157	-1.00835	0.80474257	0.926197
14608	Gpr83	G protein-coupled receptor 83, transcript variant 2	NA	0.051665	1.482655	1.03646	0.80479059	0.926197
65099	Irak1bp1	interleukin-1 receptor-associated kinase 1 binding prc	NA	0.01679	4.804206	1.011706	0.80481257	0.926197
66945	Sdha	succinate dehydrogenase complex, subunit A, flavopi	NA	-0.01092	7.445464	-1.0076	0.80490068	0.926197
414108	9230114K14Rik	RIKEN cDNA 9230114K14 gene	NA	0.078842	-0.08974	1.05617	0.80490321	0.926197
76654	Upp2	uridine phosphorylase 2, transcript variant X13	NA	-0.03461	2.840061	-1.02428	0.80497725	0.926197

72958	Zfp493	zinc finger protein 493	NA	-0.03579	2.195398	-1.02512	0.80501458	0.926197
65221	Slc15a3	solute carrier family 15, member 3	NA	0.078416	-0.32876	1.055858	0.80507501	0.926201
108169205	Gm50599	predicted gene, 50599	NA	0.047895	4.417639	1.033756	0.80519544	0.926274
26416	Mapk14	mitogen-activated protein kinase 14, transcript varian	NA	-0.01494	5.923502	-1.01041	0.80545781	0.92651
382639	Zbtb42	zinc finger and BTB domain containing 42	NA	-0.059	0.976443	-1.04174	0.80553581	0.926519
320616	B130006D01Rik	RIKEN cDNA B130006D01 gene	NA	0.06544	0.57299	1.046404	0.80557931	0.926519
231580	Gak	cyclin G associated kinase, transcript variant X1	NA	0.016478	6.473518	1.011488	0.80593354	0.926762
66614	Gpatch4	G patch domain containing 4, transcript variant 2	NA	0.021549	4.522971	1.015048	0.80595606	0.926762
231842	Amz1	archaelysin family metallopeptidase 1	NA	0.052149	2.074046	1.036808	0.80596137	0.926762
229937	Znhit6	zinc finger, HIT type 6, transcript variant X1	NA	0.018551	4.976327	1.012941	0.80603243	0.926778
68118	Atg101	autophagy related 101	NA	-0.02276	4.385517	-1.0159	0.80611672	0.926809
320230	C130023O10Rik	RIKEN cDNA C130023O10 gene	NA	-0.09183	-0.21111	-1.06572	0.80619074	0.926826
213452	Dstyk	dual serine/threonine and tyrosine protein kinase, tra	NA	0.016129	4.892023	1.011242	0.80625086	0.926826
71020	Spats1	spermatogenesis associated, serine-rich 1, transcript	NA	-0.07188	0.770594	-1.05109	0.80632995	0.926826
71228	Dlg5	discs large MAGUK scaffold protein 5, transcript varia	NA	-0.01204	6.914614	-1.00838	0.80635878	0.926826
74166	Tmem38a	transmembrane protein 38A, transcript variant X2	NA	-0.01616	5.015064	-1.01127	0.80660087	0.927038
56009	Alyref2	Aly/REF export factor 2	NA	-0.02667	3.543717	-1.01865	0.80682352	0.927229
328234	Rnf182	ring finger protein 182	NA	0.013795	5.812844	1.009608	0.80705442	0.927337
70807	Arrdc2	arrestin domain containing 2	NA	0.029422	3.039857	1.020603	0.80714529	0.927337
12484	Cd24a	CD24a antigen	NA	0.011438	10.00824	1.00796	0.80719117	0.927337
66627	Ogfod2	2-oxoglutarate and iron-dependent oxygenase domai	NA	0.025449	3.994766	1.017796	0.80730338	0.927337
268297	Scml4	Scm polycomb group protein like 4, transcript variant	NA	0.02181	4.360233	1.015233	0.80733054	0.927337
226123	Morn4	MORN repeat containing 4	NA	0.012807	6.73437	1.008917	0.80738807	0.927337
100039863	Gm13306	predicted gene 13306, transcript variant X3	NA	0.178676	0.621092	1.131844	0.80740862	0.927337
231125	Zfyve28	zinc finger, FYVE domain containing 28, transcript va	NA	0.031484	3.006498	1.022063	0.8074155	0.927337
652925	Tmem243	transmembrane protein 243, mitochondrial	NA	-0.04742	1.237034	-1.03342	0.80743064	0.927337
67851	Mtres1	mitochondrial transcription rescue factor 1	NA	0.020094	4.572427	1.014026	0.80749074	0.927341
545156	Kalrn	kalirin, RhoGEF kinase, transcript variant X43	NA	0.012649	7.631217	1.008806	0.80755926	0.927354
226049	Dmrt2	doublesex and mab-3 related transcription factor 2, tr	NA	-0.07762	0.758721	-1.05527	0.80770495	0.927426
625638	Fam43b	family with sequence similarity 43, member B	NA	0.031995	2.870217	1.022425	0.80777047	0.927426
27426	Nagpa	N-acetylglucosamine-1-phosphodiester alpha-N-acet	NA	-0.03436	2.355385	-1.02411	0.80783218	0.927426
18987	Pou2f2	POU domain, class 2, transcription factor 2, transcrip	NA	-0.01719	6.190698	-1.01199	0.80784981	0.927426
319370	Ubald2	UBA-like domain containing 2	NA	-0.01811	5.857874	-1.01263	0.80800244	0.927536
17150	Mfap2	microfibrillar-associated protein 2, transcript variant X	NA	-0.01447	5.702059	-1.01008	0.80807166	0.92755
242681	Rab42	RAB42, member RAS oncogene family, transcript va	NA	-0.07135	0.140117	-1.0507	0.80830678	0.927754
67530	Uqcrb	ubiquinol-cytochrome c reductase binding protein	NA	-0.01342	5.901911	-1.00934	0.80856328	0.927983
12607	Cebpz	CCAAT/enhancer binding protein zeta	NA	-0.01546	5.826504	-1.01078	0.80866335	0.928032
214444	Cdk5rap2	CDK5 regulatory subunit associated protein 2, transc	NA	-0.01854	5.742354	-1.01294	0.80886814	0.928202
14775	Gpx1	glutathione peroxidase 1, transcript variant 1	NA	-0.01767	6.328021	-1.01233	0.808972	0.928256
71365	Pdss2	prenyl (solanesyl) diphosphate synthase, subunit 2, t	NA	0.023878	3.76453	1.016689	0.80923942	0.928497
193742	Abhd16a	abhydrolase domain containing 16A	NA	0.015745	5.20226	1.010973	0.80931599	0.928519
56771	Med20	mediator complex subunit 20, transcript variant 1	NA	0.016229	5.028614	1.011312	0.8094198	0.928573
19414	Rasa3	RAS p21 protein activator 3	NA	0.014695	5.74657	1.010238	0.80953855	0.928644
320244	Ttll5	tubulin tyrosine ligase-like family, member 5, transcri	NA	-0.03151	4.490945	-1.02208	0.80959606	0.928644
70967	Eva1c	eva-1 homolog C (C. elegans), transcript variant X3	NA	-0.04723	1.387982	-1.03328	0.8099151	0.928915
71544	Arhgap42	Rho GTPase activating protein 42, transcript variant	NA	-0.01833	4.623945	-1.01279	0.80998571	0.928915
269401	Zfp512b	zinc finger protein 512B	NA	0.011281	7.563029	1.00785	0.81013067	0.928915
15504	Dnajb3	DnaJ heat shock protein family (Hsp40) member B3	NA	-0.03846	1.909671	-1.02702	0.81013361	0.928915
74737	Pcf11	PCF11 cleavage and polyadenylation factor subunit	NA	0.013697	5.678517	1.009539	0.81017957	0.928915
228413	Prrg4	proline rich Gla (G-carboxyglutamic acid) 4 (transmer	NA	-0.05427	0.997996	-1.03834	0.81023074	0.928915
69151	Lzic	leucine zipper and CTNNBIP1 domain containing, tra	NA	-0.01856	5.018199	-1.01295	0.81027812	0.928915
69029	Smdt1	single-pass membrane protein with aspartate rich tail	NA	-0.01564	5.431311	-1.0109	0.81028862	0.928915
20607	Sstr3	somatostatin receptor 3, transcript variant 1	NA	-0.04628	1.40612	-1.0326	0.81043844	0.928956
14786	Grb7	growth factor receptor bound protein 7, transcript vari	NA	-0.09011	-0.023	-1.06445	0.81050068	0.928956
12580	Cdkn2c	cyclin dependent kinase inhibitor 2C, transcript variar	NA	-0.02503	3.626538	-1.0175	0.8105613	0.928956
66075	Chchd3	coiled-coil-helix-coiled-coil-helix domain containing 3,	NA	-0.01601	5.864652	-1.01116	0.81058438	0.928956
66082	Abhd6	abhydrolase domain containing 6, transcript variant 1	NA	-0.02048	4.205022	-1.0143	0.81060965	0.928956
237221	Gemin8	gem nuclear organelle associated protein 8, transcrip	NA	-0.02938	3.096979	-1.02058	0.8108127	0.929084
72309	Tmem158	transmembrane protein 158	NA	0.022313	4.860674	1.015586	0.81083549	0.929084
271005	Klhdc1	kelch domain containing 1, transcript variant X4	NA	0.02686	3.43333	1.018792	0.81094606	0.929145
67279	Med31	mediator complex subunit 31	NA	0.026707	3.643946	1.018684	0.81123479	0.92941
77975	Tmem50b	transmembrane protein 50B	NA	-0.01396	6.091532	-1.00972	0.81148206	0.929628
210126	Lpp	LIM domain containing preferred translocation partne	NA	0.017394	4.583888	1.01213	0.81165875	0.929765
100043468	Zfp955b	zinc finger protein 955B, transcript variant X1	NA	0.01552	5.312508	1.010816	0.81171568	0.929765
231051	Kmt2c	lysine (K)-specific methyltransferase 2C, transcript va	NA	-0.01323	7.036622	-1.00921	0.81190015	0.929911
115486031	Gm51425	predicted gene, 51425, transcript variant 1	NA	0.037071	2.401221	1.026029	0.81218128	0.930167
218503	Fcho2	FCH domain only 2, transcript variant X1	NA	0.014654	5.683049	1.010209	0.81233478	0.930212
66129	Prxl2c	peroxiredoxin like 2C	NA	-0.02592	3.418472	-1.01813	0.81238013	0.930212

54394	Crlf3	cytokine receptor-like factor 3, transcript variant 1	NA	-0.02043	4.05016	-1.01426	0.81239229	0.930212
71146	Golga7b	golgi autoantigen, golgin subfamily a, 7B, transcript v	NA	-0.01516	5.551548	-1.01057	0.81255453	0.930333
319468	Ppm1h	protein phosphatase 1H (PP2C domain containing), t	NA	-0.01607	5.42627	-1.0112	0.81266985	0.930399
11465	Actg1	actin, gamma, cytoplasmic 1, transcript variant 1	NA	0.011914	11.69846	1.008292	0.81277398	0.930453
216991	Adap2	ArfGAP with dual PH domains 2, transcript variant X1	NA	0.040466	2.456245	1.028446	0.81290132	0.930533
66118	Sarnp	SAP domain containing ribonucleoprotein, transcript v	NA	0.014224	6.064758	1.009908	0.81303911	0.930626
80284	Smim12	small integral membrane protein 12	NA	0.026139	3.474409	1.018284	0.81319321	0.930736
231841	Brat1	BRCA1-associated ATM activator 1, transcript varian	NA	0.024158	4.418211	1.016886	0.81328102	0.930771
16591	Kl	klotho	NA	-0.05351	0.771267	-1.03778	0.81337442	0.930789
54364	Rpp30	ribonuclease P/MRP 30 subunit, transcript variant X1	NA	-0.01896	4.361509	-1.01323	0.81343667	0.930789
118568297	LOC118568297	igE-binding protein-like	NA	0.049959	2.149228	1.035235	0.81346789	0.930789
56444	Actr10	ARP10 actin-related protein 10	NA	0.011229	7.101126	1.007814	0.81360818	0.930884
53404	Atoh7	atonal bHLH transcription factor 7, transcript variant 1	NA	-0.05749	0.68597	-1.04066	0.81366504	0.930884
67178	Zmat5	zinc finger, matrin type 5, transcript variant 1	NA	-0.02405	3.56322	-1.01681	0.81378029	0.930922
56422	Hbs1l	Hbs1-like (S. cerevisiae), transcript variant 1	NA	-0.01238	6.371445	-1.00862	0.81381272	0.930922
74337	Palm3	paralemmin 3	NA	-0.03774	2.398594	-1.02651	0.81387807	0.930931
20420	Shd	src homology 2 domain-containing transforming prote	NA	0.016603	5.617669	1.011575	0.81394882	0.930946
105243712	Gm39529	predicted gene, 39529, transcript variant 2	NA	0.094948	-0.33179	1.068027	0.81419218	0.931139
14615	Gjc1	gap junction protein, gamma 1, transcript variant 3	NA	0.014042	5.505629	1.009781	0.81423133	0.931139
66923	Pbrm1	polybromo 1, transcript variant 6	NA	0.011144	7.195004	1.007754	0.81463549	0.931505
384783	Irs2	insulin receptor substrate 2	NA	-0.01562	5.530402	-1.01089	0.8146659	0.931505
80280	Cdk5rap3	CDK5 regulatory subunit associated protein 3, transc	NA	0.018204	5.31972	1.012698	0.81478838	0.931531
56526	Septin6	septin 6, transcript variant 2	NA	0.011697	6.774581	1.008141	0.8148033	0.931531
102635799	Gm33047	predicted gene, 33047	NA	0.110719	-0.39051	1.079766	0.81503356	0.931711
268857	Nlrc3	NLR family, CARD domain containing 3, transcript va	NA	0.058377	1.473247	1.041294	0.81507514	0.931711
22092	Rsph1	radial spoke head 1 homolog (Chlamydomonas), tran	NA	0.041772	2.033742	1.029378	0.81519985	0.931788
12330	Canx	calnexin, transcript variant 1	NA	0.01043	8.501938	1.007256	0.81557165	0.932125
12166	Bmpr1a	bone morphogenetic protein receptor, type 1A	NA	0.015539	6.197482	1.010829	0.81560948	0.932125
268930	Pkmyt1	protein kinase, membrane associated tyrosine/threon	NA	-0.02472	3.618179	-1.01728	0.81605539	0.932527
140492	Kcnn2	potassium intermediate/small conductance calcium-a	NA	0.018104	4.320501	1.012628	0.81612591	0.932527
240354	Malt1	MALT1 paracaspase, transcript variant X2	NA	-0.03197	2.823235	-1.02241	0.81623085	0.932527
27061	Bcap31	B cell receptor associated protein 31, transcript varia	NA	-0.01832	5.318209	-1.01278	0.81624016	0.932527
50996	Pdcd7	programmed cell death 7	NA	0.0172	4.413905	1.011994	0.81624789	0.932527
54624	Paf1	Paf1, RNA polymerase II complex component	NA	-0.0129	6.204658	-1.00899	0.81643258	0.932673
216438	Marchf9	membrane associated ring-CH-type finger 9	NA	0.016034	6.071357	1.011176	0.81675848	0.932931
11765	Ap1g1	adaptor protein complex AP-1, gamma 1 subunit, tran	NA	0.012048	7.348848	1.008386	0.81677334	0.932931
68291	Mto1	mitochondrial tRNA translation optimization 1	NA	-0.01976	4.177462	-1.01379	0.81713523	0.933269
67149	Nkain1	Na+/K+ transporting ATPase interacting 1, transcript	NA	0.009645	8.006871	1.006708	0.81735747	0.933269
67973	Mphosph10	M-phase phosphoprotein 10 (U3 small nucleolar ribor	NA	0.018493	4.572914	1.012901	0.81735801	0.933269
20523	Slc25a14	solute carrier family 25 (mitochondrial carrier, brain),	NA	0.020191	4.681476	1.014094	0.81739764	0.933269
94187	Zfp423	zinc finger protein 423, transcript variant 2	NA	-0.01308	6.141681	-1.00911	0.81745279	0.933269
105513	Chmp7	charged multivesicular body protein 7	NA	-0.01177	6.857173	-1.00819	0.81752098	0.933269
433520	Gm14403	predicted gene 14403	NA	-0.04452	1.566821	-1.03134	0.81760133	0.933269
12937	Pcdha6	protocadherin alpha 6	NA	-0.08755	0.888941	-1.06256	0.81769813	0.933269
115488777	Gm52290	predicted gene, 52290	NA	-0.09292	0.264619	-1.06652	0.81772828	0.933269
67636	Etfrf1	electron transfer flavoprotein regulatory factor 1, tran	NA	0.022193	3.832626	1.015502	0.81773335	0.933269
70508	Bbx	bobby sox HMG box containing, transcript variant X3	NA	0.02177	4.613587	1.015204	0.81780451	0.933269
108168350	Gm27217	predicted gene 27217	NA	-0.07406	-0.11425	-1.05268	0.81784548	0.933269
72144	Slc37a3	solute carrier family 37 (glycerol-3-phosphate transpo	NA	0.011454	6.177028	1.007971	0.81786401	0.933269
68115	Maip1	matrix AAA peptidase interacting protein 1	NA	0.015841	4.614095	1.01104	0.81787178	0.933269
80708	Pacsin3	protein kinase C and casein kinase substrate in neur	NA	-0.02265	3.560321	-1.01582	0.81800041	0.933283
140580	Elmo1	engulfment and cell motility 1, transcript variant X4	NA	-0.01242	6.601853	-1.00864	0.81803561	0.933283
59053	Hgh1	HGH1 homolog	NA	-0.02086	3.822458	-1.01457	0.81806296	0.933283
20288	Msr1	macrophage scavenger receptor 1, transcript variant	NA	-0.07028	0.386637	-1.04992	0.81814403	0.933283
15203	Heph	hephaestin, transcript variant 3	NA	0.027195	3.026037	1.019029	0.81820014	0.933283
235956	Zfp825	zinc finger protein 825, transcript variant X1	NA	-0.0273	3.572945	-1.0191	0.81827584	0.933283
71566	Clmp	CXADR-like membrane protein, transcript variant X1	NA	-0.01056	7.18814	-1.00734	0.81828574	0.933283
12683	Cidea	cell death-inducing DNA fragmentation factor, alpha s	NA	0.075201	-0.01735	1.053508	0.81837144	0.933306
57753	Noc3l	NOC3 like DNA replication regulator	NA	-0.01761	4.838283	-1.01228	0.81842046	0.933306
18294	Ogg1	8-oxoguanine DNA-glycosylase 1, transcript variant 1	NA	-0.0294	2.914057	-1.02059	0.81893159	0.933823
20649	Sntb1	syntrophin, basic 1, transcript variant X1	NA	0.031799	2.179701	1.022286	0.8190094	0.933846
67609	4930453N24Rik	RIKEN cDNA 4930453N24 gene	NA	0.016751	5.057067	1.011678	0.81910752	0.933893
78896	Ecrg4	ECRG4 augurin precursor	NA	0.027361	3.081236	1.019146	0.8192308	0.933968
13345	Twist2	twist basic helix-loop-helix transcription factor 2	NA	0.089934	0.618166	1.064321	0.81952242	0.934199
666048	Trabd2b	TraB domain containing 2B, transcript variant X3	NA	0.044496	2.415257	1.031323	0.81954798	0.934199
381339	Tmem182	transmembrane protein 182, transcript variant X2	NA	0.109651	-0.76348	1.078967	0.8197358	0.934278
74007	Btbd11	BTB (POZ) domain containing 11, transcript variant 2	NA	0.015438	4.847964	1.010758	0.81978529	0.934278
100040608	Fancf	Fanconi anemia, complementation group F	NA	0.034433	2.470854	1.024154	0.81979031	0.934278

68016	Cavin4	caveolae associated 4	NA	-0.04213	2.309519	-1.02963	0.81988354	0.934319
623661	Lipt1	lipoyltransferase 1, transcript variant 2	NA	0.032418	2.253568	1.022725	0.82014497	0.934348
18260	Ocln	occludin, transcript variant 1	NA	0.026652	3.095557	1.018645	0.82015835	0.934348
108169043	Gm46911	predicted gene, 46911, transcript variant X3	NA	-0.04951	1.886176	-1.03491	0.82018486	0.934348
330230	Zfp853	zinc finger protein 853, transcript variant X8	NA	-0.03361	2.337878	-1.02357	0.82021747	0.934348
244864	Layn	layilin, transcript variant X1	NA	-0.04412	1.271063	-1.03106	0.82029758	0.934348
75593	Malsu1	mitochondrial assembly of ribosomal large subunit 1	NA	-0.02382	3.273419	-1.01665	0.82031604	0.934348
12913	Creb3	cAMP responsive element binding protein 3	NA	0.015623	4.833159	1.010888	0.82035603	0.934348
94218	Cnnm3	cyclin M3, transcript variant 1	NA	0.013337	5.415754	1.009288	0.82037054	0.934348
68240	Rpa3	replication protein A3	NA	-0.02315	3.987506	-1.01618	0.82043402	0.934348
77090	Ocel1	occludin/ELL domain containing 1, transcript variant ´	NA	0.058107	0.650723	1.041099	0.82061446	0.934348
226539	Dars2	aspartyl-tRNA synthetase 2 (mitochondrial)	NA	-0.01802	4.189742	-1.01257	0.82062218	0.934348
102632234	Gm30368	predicted gene, 30368, transcript variant X7	NA	0.088879	-0.05663	1.063544	0.82063968	0.934348
67013	Oma1	OMA1 zinc metallopeptidase	NA	0.025547	3.69332	1.017866	0.82070706	0.934348
67542	Cog6	component of oligomeric golgi complex 6	NA	0.012832	5.643907	1.008934	0.8207128	0.934348
170935	Grid2ip	glutamate receptor, ionotropic, delta 2 (Grid2) interac	NA	-0.06744	0.364435	-1.04786	0.82102465	0.934638
68758	Abhd11	abhydrolase domain containing 11, transcript variant	NA	0.015949	4.840584	1.011117	0.82143126	0.93484
30930	Vps26a	VPS26 retromer complex component A, transcript vai	NA	-0.01271	5.913129	-1.00885	0.82143671	0.93484
69976	Galk2	galactokinase 2, transcript variant X2	NA	-0.01843	4.371592	-1.01286	0.82149166	0.93484
19164	Psen1	presenilin 1	NA	-0.01375	6.044181	-1.00958	0.82149744	0.93484
102640971	CJ186046Rik	Riken cDNA CJ186046 gene, transcript variant X1	NA	-0.0808	-0.68255	-1.0576	0.82152283	0.93484
17524	Mpp1	membrane protein, palmitoylated	NA	-0.01998	4.255826	-1.01394	0.82154669	0.93484
108737	Oxsr1	oxidative-stress responsive 1, transcript variant 3	NA	-0.01487	6.411735	-1.01036	0.8218589	0.935076
20471	Six1	sine oculis-related homeobox 1	NA	-0.05126	1.519153	-1.03617	0.8218692	0.935076
67231	Tbc1d20	TBC1 domain family, member 20, transcript variant 1	NA	-0.01302	5.887836	-1.00906	0.82194039	0.935092
320817	Atad2b	ATPase family, AAA domain containing 2B, transcript	NA	0.014358	5.796587	1.010002	0.82201749	0.935114
21843	Tial1	Tia1 cytotoxic granule-associated RNA binding protei	NA	-0.01418	7.237985	-1.00988	0.82211871	0.935164
71881	Apmap	adipocyte plasma membrane associated protein, tran	NA	-0.01606	4.911777	-1.01119	0.82224749	0.935208
102640671	Gm36685	predicted gene, 36685	NA	-0.0586	0.632732	-1.04146	0.82227309	0.935208
110208	Pgd	phosphogluconate dehydrogenase, transcript variant	NA	-0.01266	6.421818	-1.00882	0.82233881	0.935218
224694	Zfp81	zinc finger protein 81, transcript variant X3	NA	-0.01265	6.193331	-1.00881	0.82249151	0.935317
101401	Adamts9	a disintegrin-like and metallopeptidase (reprolysin typ	NA	0.021668	3.740164	1.015132	0.82254062	0.935317
13167	Dbi	diazepam binding inhibitor, transcript variant 2	NA	0.02145	7.147264	1.014979	0.82265559	0.935349
26891	Cops4	COP9 signalosome subunit 4, transcript variant 1	NA	0.011598	6.396885	1.008071	0.82268445	0.935349
66461	Ptpmt1	protein tyrosine phosphatase, mitochondrial 1	NA	0.019605	4.061954	1.013682	0.8227865	0.935353
69434	Snhg10	small nucleolar RNA host gene 10	NA	0.052223	0.991659	1.036862	0.8228022	0.935353
319475	Zfp672	zinc finger protein 672, transcript variant X18	NA	-0.01643	5.031222	-1.01145	0.82297682	0.935486
19070	Mob4	MOB family member 4, phocein, transcript variant 1	NA	-0.01136	6.67463	-1.00791	0.82304655	0.935492
102632483	Gm17330	predicted gene, 17330, transcript variant X1	NA	0.036678	3.196539	1.025749	0.82309743	0.935492
73649	Cybrd1	cytochrome b reductase 1	NA	0.023742	4.135438	1.016593	0.82327181	0.935625
14163	Fgd1	FYVE, RhoGEF and PH domain containing 1, transcr	NA	-0.01584	5.976043	-1.01104	0.8234784	0.935729
14472	Gbx2	gastrulation brain homeobox 2	NA	-0.02564	3.710446	-1.01793	0.82350052	0.935729
56368	Cyb561d2	cytochrome b-561 domain containing 2, transcript vai	NA	0.023388	3.214132	1.016344	0.82354425	0.935729
100188919	Gm45915	predicted gene, 45915	NA	0.087467	-0.07101	1.062503	0.82361181	0.935729
70361	Lman1	lectin, mannose-binding, 1, transcript variant 1	NA	0.016106	5.565145	1.011226	0.82369402	0.935729
69550	Bst2	bone marrow stromal cell antigen 2	NA	0.043869	1.158315	1.030875	0.82370843	0.935729
224903	Safb	scaffold attachment factor B, transcript variant 2	NA	0.009668	7.432058	1.006724	0.82389109	0.935853
13057	Cyba	cytochrome b-245, alpha polypeptide, transcript varia	NA	-0.03656	2.085683	-1.02566	0.82395688	0.935853
242653	Cldn19	claudin 19, transcript variant 2	NA	-0.05446	0.877541	-1.03847	0.82398999	0.935853
52389	Adgra1	adhesion G protein-coupled receptor A1	NA	0.023459	3.852302	1.016393	0.82410982	0.935886
52882	Rgs7bp	regulator of G-protein signalling 7 binding protein, tra	NA	0.018361	4.501498	1.012808	0.82413378	0.935886
269639	Zfp512	zinc finger protein 512, transcript variant 1	NA	-0.01144	7.050454	-1.00796	0.82437573	0.93599
73205	C9orf72	C9orf72, member of C9orf72-SMCR8 complex, trans	NA	0.013153	5.669986	1.009158	0.82439395	0.93599
83561	Tdrd1	tudor domain containing 1, transcript variant X7	NA	0.057145	0.891237	1.040405	0.82443769	0.93599
55942	Sertad1	SERTA domain containing 1	NA	-0.05613	1.537068	-1.03967	0.82445565	0.93599
230806	Crybg2	crystallin beta-gamma domain containing 2, transcrip	NA	0.079589	-0.32254	1.056717	0.82455106	0.935995
76650	Srxn1	sulfiredoxin 1 homolog (S. cerevisiae)	NA	-0.01819	4.142799	-1.01269	0.82457566	0.935995
17069	Ly6e	lymphocyte antigen 6 complex, locus E, transcript vai	NA	-0.01313	5.599316	-1.00914	0.82468268	0.936052
12652	Chga	chromogranin A	NA	-0.01887	6.312883	-1.01317	0.82475545	0.936069
110310	Krt7	keratin 7	NA	-0.07512	0.716286	-1.05345	0.82486327	0.936096
118567589	LOC118567589	uncharacterized LOC118567589	NA	-0.05077	0.962327	-1.03582	0.82495358	0.936096
209012	Ulk4	unc-51-like kinase 4, transcript variant X1	NA	-0.02689	2.759254	-1.01882	0.82508338	0.936096
75302	Asxl2	additional sex combs like 2, transcriptional regulator,	NA	0.018757	5.036081	1.013087	0.82509994	0.936096
66884	Appbp2	amyloid beta precursor protein (cytoplasmic tail) bind	NA	-0.02533	4.561961	-1.01771	0.82512936	0.936096
73667	2410004P03Rik	RIKEN cDNA 2410004P03 gene, transcript variant 1	NA	0.038474	1.924206	1.027027	0.82518979	0.936096
54485	Dll4	delta like canonical Notch ligand 4	NA	0.035567	2.949273	1.02496	0.82525274	0.936096
67773	Kat8	K(lysine) acetyltransferase 8, transcript variant 1	NA	0.014804	4.780904	1.010314	0.82527637	0.936096
56428	Mtch2	mitochondrial carrier 2, transcript variant 1	NA	-0.01666	6.958886	-1.01162	0.82529711	0.936096

23854	Def8	differentially expressed in FDCP 8, transcript variant :	NA	-0.01119	6.182815	-1.00779	0.82576067	0.936325
70396	Asnsd1	asparagine synthetase domain containing 1, transcrip	NA	0.012708	5.753249	1.008847	0.82577135	0.936325
19210	Ptdss1	phosphatidylserine synthase 1	NA	0.012939	6.288102	1.009009	0.82581649	0.936325
75605	Kdm5b	lysine (K)-specific demethylase 5B, transcript variant	NA	-0.01113	7.558078	-1.00775	0.82583188	0.936325
22346	Vhl	von Hippel-Lindau tumor suppressor	NA	0.012555	6.179717	1.008741	0.82585984	0.936325
215653	Rassf2	Ras association (RalGDS/AF-6) domain family memb	NA	0.012966	6.182398	1.009028	0.82596053	0.936325
626832	Gm6710	predicted gene 6710	NA	0.146048	0.7115	1.106534	0.82601908	0.936325
20090	Rps29	ribosomal protein S29	NA	-0.02674	7.862699	-1.01871	0.82612786	0.936325
19245	Ptp4a3	protein tyrosine phosphatase 4a3, transcript variant X	NA	0.013571	5.557531	1.009451	0.82613459	0.936325
68836	Mrpl52	mitochondrial ribosomal protein L52	NA	0.016404	4.436116	1.011435	0.82615466	0.936325
78755	Fam122b	family with sequence similarity 122, member B, trans	NA	0.033884	2.984589	1.023765	0.82616658	0.936325
94282	Sfxn5	sideroflexin 5	NA	-0.01631	5.343256	-1.01137	0.82623646	0.936325
214804	Syde2	synapse defective 1, Rho GTPase, homolog 2 (C. ele	NA	-0.02938	2.554487	-1.02057	0.82624679	0.936325
228850	Ralgapb	Ral GTPase activating protein, beta subunit (non-cata	NA	-0.01168	6.674881	-1.00813	0.82639926	0.936423
17701	Msx1	msh homeobox 1	NA	-0.02588	3.029422	-1.0181	0.82644851	0.936423
17463	Psmd7	proteasome (prosome, macropain) 26S subunit, non-	NA	0.010468	6.812616	1.007282	0.82657608	0.936502
242297	Fam110b	family with sequence similarity 110, member B, trans	NA	0.011402	5.98584	1.007934	0.82674228	0.936625
108167429	Gm33136	predicted gene, 33136, transcript variant X1	NA	0.056189	1.021612	1.039716	0.82696049	0.936807
12914	Crebbp	CREB binding protein, transcript variant X4	NA	0.010503	7.071493	1.007306	0.8270852	0.936883
319673	9330159M07Rik	RIKEN cDNA 9330159M07 gene, transcript variant 2	NA	0.053437	0.608378	1.037734	0.82739466	0.937169
50913	Olig2	oligodendrocyte transcription factor 2	NA	-0.01628	4.655855	-1.01135	0.82748969	0.937173
52855	Lair1	leukocyte-associated Ig-like receptor 1, transcript var	NA	0.047593	1.329149	1.033539	0.82751391	0.937173
100503390	Gm17518	predicted gene, 17518, transcript variant X1	NA	-0.08093	-0.14718	-1.0577	0.82757785	0.937181
101023	Zfp513	zinc finger protein 513, transcript variant 1	NA	0.015502	5.041685	1.010803	0.82784843	0.937252
72674	Adipor1	adiponectin receptor 1, transcript variant 2	NA	-0.01064	6.430503	-1.0074	0.827897	0.937252
338346	Gpr21	G protein-coupled receptor 21	NA	0.023056	3.643731	1.016109	0.82792595	0.937252
58237	Nkain4	Na+/K+ transporting ATPase interacting 4, transcript	NA	0.020752	3.866546	1.014488	0.82793144	0.937252
230775	Adgrb2	adhesion G protein-coupled receptor B2, transcript va	NA	-0.01044	7.51746	-1.00726	0.82795705	0.937252
67446	Dusp28	dual specificity phosphatase 28	NA	0.028599	2.557927	1.020021	0.82798683	0.937252
118568082	LOC118568082	uncharacterized LOC118568082	NA	-0.05603	0.999628	-1.0396	0.82817068	0.937269
83673	Snhg1	small nucleolar RNA host gene 1	NA	-0.02246	4.159792	-1.01569	0.82817261	0.937269
66641	Sike1	suppressor of IKBKE 1	NA	-0.01318	5.2294	-1.00918	0.82818434	0.937269
218885	Oxnad1	oxidoreductase NAD-binding domain containing 1	NA	-0.01852	4.161701	-1.01292	0.82826174	0.937269
108014	Srsf9	serine and arginine-rich splicing factor 9, transcript va	NA	-0.01962	5.542929	-1.01369	0.82828949	0.937269
215632	Psd4	pleckstrin and Sec7 domain containing 4, transcript v	NA	0.068066	0.003654	1.048311	0.82834835	0.93727
12839	Col9a1	collagen, type IX, alpha 1, transcript variant 1	NA	0.041881	2.899486	1.029455	0.82841662	0.937282
13033	Ctsd	cathepsin D	NA	0.009557	7.370515	1.006646	0.82856324	0.937325
78100	Msantd4	Myb/SANT-like DNA-binding domain containing 4 witl	NA	0.011474	6.296773	1.007985	0.82856957	0.937325
239743	Klhl6	kelch-like 6, transcript variant X1	NA	0.046548	1.674327	1.032791	0.82869628	0.937395
118568281	LOC118568281	40S ribosomal protein S24-like, transcript variant X4	NA	0.030192	2.263633	1.021148	0.82874616	0.937395
69237	Gtpbp4	GTP binding protein 4	NA	-0.01343	5.648845	-1.00935	0.82893322	0.937446
27367	Rpl3	ribosomal protein L3	NA	0.010019	9.411555	1.006969	0.82896306	0.937446
73327	Pradc1	protease-associated domain containing 1, transcript v	NA	-0.03915	2.39668	-1.02751	0.82896469	0.937446
78552	E130201H02Rik	RIKEN cDNA E130201H02 gene	NA	-0.07706	-0.30819	-1.05486	0.82908987	0.937523
56312	Nupr1	nuclear protein transcription regulator 1	NA	0.04578	1.24419	1.032241	0.82928658	0.93768
626082	Gm20754	predicted gene, 20754	NA	0.070926	0.182529	1.050391	0.82937585	0.9377
115488332	Gm52128	predicted gene, 52128	NA	0.035998	3.246967	1.025266	0.82941978	0.9377
27207	Rps11	ribosomal protein S11	NA	0.01351	8.546349	1.009409	0.82963467	0.93783
320799	Zhx3	zinc fingers and homeoboxes 3	NA	0.022142	3.760322	1.015466	0.82964949	0.93783
26451	Rpl27a	ribosomal protein L27A	NA	-0.01006	8.475963	-1.00699	0.8298185	0.937936
67433	Ccdc127	coiled-coil domain containing 127, transcript variant 1	NA	0.013562	6.087281	1.009444	0.82996819	0.937936
544848	Gm5784	predicted gene 5784, transcript variant X1	NA	-0.03615	3.233423	-1.02537	0.82998058	0.937936
66536	Nipsnap3b	nipsnap homolog 3B, transcript variant 1	NA	-0.01165	5.612555	-1.00811	0.83006871	0.937936
69430	1700048O20Rik	RIKEN cDNA 1700048O20 gene	NA	-0.02634	4.013734	-1.01843	0.83012373	0.937936
26564	Ror2	receptor tyrosine kinase-like orphan receptor 2	NA	-0.02485	2.934719	-1.01737	0.8301956	0.937936
115489722	LOC115489722	uncharacterized LOC115489722	NA	0.175718	-1.48336	1.129526	0.83020607	0.937936
330409	Cecr2	CECR2, histone acetyl-lysine reader, transcript variar	NA	-0.0246	4.28779	-1.0172	0.83025428	0.937936
108167746	Gm46172	predicted gene, 46172	NA	0.061201	0.009805	1.043334	0.83026232	0.937936
68499	Mrpl53	mitochondrial ribosomal protein L53	NA	-0.02367	4.490469	-1.01654	0.83037888	0.937987
211135	D130040H23Rik	RIKEN cDNA D130040H23 gene, transcript variant X	NA	-0.01339	5.22356	-1.00932	0.83046106	0.937987
226101	Myof	myoferlin, transcript variant 2	NA	0.032629	2.648977	1.022874	0.8304806	0.937987
326622	Upf2	UPF2 regulator of nonsense transcripts homolog (yea	NA	0.011847	5.618243	1.008246	0.8307014	0.938171
244329	Mcph1	microcephaly, primary autosomal recessive 1, transcı	NA	-0.01787	4.383003	-1.01246	0.83076847	0.938182
215627	Zbtb8b	zinc finger and BTB domain containing 8b	NA	0.023571	3.723379	1.016472	0.83085179	0.938211
105245681	Gm41098	predicted gene, 41098	NA	-0.05008	0.733608	-1.03532	0.83094097	0.938247
233246	Ano5	anoctamin 5, transcript variant 2	NA	-0.05024	1.039713	-1.03544	0.8310251	0.938276
69146	Gsdmd	gasdermin D	NA	0.043396	1.223753	1.030536	0.83115395	0.938277
11576	Afp	alpha fetoprotein	NA	-0.15985	1.091359	-1.11717	0.83118237	0.938277

11792	Apex1	apurinic/apyrimidinic endonuclease 1	NA	-0.01145	6.279309	-1.00797	0.83119881	0.938277
81000	Rad54l2	RAD54 like 2 (S. cerevisiae)	NA	0.013878	6.209009	1.009666	0.83147495	0.938402
115488470	LOC115488470	uncharacterized LOC115488470	NA	-0.02344	3.433873	-1.01638	0.83150641	0.938402
12258	Serping1	serine (or cysteine) peptidase inhibitor, clade G, mem	NA	-0.02557	3.848513	-1.01788	0.83155714	0.938402
214498	Cdc73	cell division cycle 73, Paf1/RNA polymerase II comple	NA	-0.01311	5.135271	-1.00913	0.83159365	0.938402
102580	Alg9	asparagine-linked glycosylation 9 (alpha 1,2 mannosy	NA	0.013738	5.266873	1.009568	0.83163294	0.938402
11785	Apbb1	amyloid beta (A4) precursor protein-binding, family B	NA	-0.00973	7.658658	-1.00677	0.83168487	0.938402
56305	Pitpnb	phosphatidylinositol transfer protein, beta, transcript v	NA	-0.00992	6.730763	-1.0069	0.83171859	0.938402
228359	Arhgap1	Rho GTPase activating protein 1, transcript variant X	NA	0.011915	6.449087	1.008293	0.83181369	0.938402
118568792	LOC118568792	uncharacterized LOC118568792	NA	-0.01873	4.073459	-1.01307	0.83185223	0.938402
11522	Adh1	alcohol dehydrogenase 1 (class I)	NA	0.083084	-0.16301	1.05928	0.83188565	0.938402
246293	Klhl8	kelch-like 8, transcript variant 2	NA	0.013066	5.668549	1.009098	0.83205785	0.938531
14227	Fkbp2	FK506 binding protein 2, transcript variant 4	NA	0.01615	4.337649	1.011257	0.83216696	0.938545
13506	Dsc2	desmocollin 2, transcript variant Dsc2a	NA	-0.04756	1.385203	-1.03352	0.8321858	0.938545
14208	Ppm1g	protein phosphatase 1G (formerly 2C), magnesium-d	NA	-0.00958	7.414087	-1.00666	0.83247215	0.938698
50529	Mrps7	mitchondrial ribosomal protein S7	NA	-0.0171	5.158921	-1.01193	0.83249399	0.938698
17973	Nck1	non-catalytic region of tyrosine kinase adaptor protein	NA	-0.01625	4.681947	-1.01133	0.83255096	0.938698
11639	Ak4	adenylate kinase 4, transcript variant X2	NA	0.025027	3.93566	1.017499	0.83262939	0.938698
545389	Cep170	centrosomal protein 170, transcript variant X14	NA	-0.01086	8.361298	-1.00756	0.83263339	0.938698
100503199	5430416N02Rik	RIKEN cDNA 5430416N02 gene	NA	0.026978	3.127177	1.018876	0.83266738	0.938698
94280	Sfxn3	sideroflexin 3, transcript variant 1	NA	0.010229	6.774158	1.007116	0.83280539	0.938734
319481	Wdr59	WD repeat domain 59, transcript variant 3	NA	0.013297	5.035696	1.009259	0.83295079	0.938734
228564	Frmd5	FERM domain containing 5, transcript variant 1	NA	-0.01172	6.248312	-1.00815	0.83296296	0.938734
67861	Akr1b10	aldo-keto reductase family 1, member B10 (aldose re	NA	-0.03427	2.149605	-1.02404	0.83296504	0.938734
71807	Tars2	threonyl-tRNA synthetase 2, mitochondrial (putative),	NA	-0.01572	4.859748	-1.01095	0.8329882	0.938734
17937	Nab2	Ngfi-A binding protein 2, transcript variant 1	NA	-0.02427	3.316122	-1.01697	0.8331153	0.938812
22761	Zfpm1	zinc finger protein, multitype 1, transcript variant X1	NA	0.021731	3.728355	1.015177	0.83339329	0.939061
118568424	Akap17a	A-kinase anchoring protein 17A, transcript variant X3	NA	0.024616	4.974046	1.017209	0.83346104	0.939072
56491	Vapb	vesicle-associated membrane protein, associated pro	NA	0.009765	7.009742	1.006791	0.83361448	0.93918
71904	Paqr7	progestin and adipoQ receptor family member VII, tra	NA	0.020259	4.229198	1.014141	0.8337082	0.93922
78890	Trmt44	tRNA methyltransferase 44	NA	0.026983	2.817891	1.018879	0.833954	0.939238
213417	Klhdc8a	kelch domain containing 8A, transcript variant X1	NA	0.026986	4.026566	1.018881	0.83411589	0.939238
20680	Sox7	SRY (sex determining region Y)-box 7	NA	-0.02375	3.00153	-1.0166	0.83412382	0.939238
109778	Blvra	biliverdin reductase A, transcript variant X2	NA	-0.03343	3.153626	-1.02344	0.83415139	0.939238
17285	Meox1	mesenchyme homeobox 1	NA	0.056902	0.425963	1.04023	0.83423713	0.939238
100043902	Six3os1	SIX homeobox 3, opposite strand 1, transcript variant	NA	-0.01759	4.448184	-1.01226	0.83425198	0.939238
11595	Acan	aggrecan, transcript variant 2	NA	-0.04527	1.481878	-1.03187	0.83433224	0.939238
118568777	LOC118568777	uncharacterized LOC118568777	NA	-0.05039	1.063374	-1.03554	0.83437672	0.939238
170676	Peg10	paternally expressed 10, transcript variant 1	NA	0.011656	7.88715	1.008112	0.83446489	0.939238
76893	Cers2	ceramide synthase 2, transcript variant 2	NA	0.01087	5.948235	1.007563	0.83448579	0.939238
105242946	Gm39017	predicted gene, 39017	NA	0.069788	0.461011	1.049563	0.83451561	0.939238
65111	Dap3	death associated protein 3, transcript variant X2	NA	-0.01194	6.204934	-1.00831	0.83452606	0.939238
12903	Crabp1	cellular retinoic acid binding protein I, transcript variar	NA	-0.02866	4.022947	-1.02007	0.8345314	0.939238
269593	Luzp1	leucine zipper protein 1, transcript variant X2	NA	0.014648	5.112104	1.010205	0.83465213	0.939238
20438	Siah1b	siah E3 ubiquitin protein ligase 1B, transcript variant	NA	0.023407	3.248295	1.016357	0.83470976	0.939238
27062	Cadps	Ca2+-dependent secretion activator, transcript varian	NA	0.01053	7.231146	1.007326	0.83472765	0.939238
102639686	Gm35940	predicted gene, 35940, transcript variant X2	NA	0.060258	0.329123	1.042652	0.83485762	0.939238
13807	Eno2	enolase 2, gamma neuronal, transcript variant 1	NA	0.012256	6.351458	1.008532	0.83487396	0.939238
239556	Cacna1i	calcium channel, voltage-dependent, alpha 1I subuni	NA	0.015995	4.440942	1.011148	0.83488147	0.939238
225849	Ppp2r5b	protein phosphatase 2, regulatory subunit B', beta	NA	-0.01093	6.964184	-1.0076	0.83492909	0.939238
622175	E430024I08Rik	RIKEN cDNA E430024I08 gene, transcript variant X1	NA	0.017677	4.082646	1.012328	0.83493569	0.939238
83433	Trem2	triggering receptor expressed on myeloid cells 2, tran	NA	0.064764	0.677311	1.045914	0.83505633	0.939308
108168883	4930481B07Rik	RIKEN cDNA 4930481B07 gene	NA	0.047982	1.409595	1.033818	0.83521367	0.939416
216177	AU041133	expressed sequence AU041133	NA	0.023164	2.935	1.016185	0.83526699	0.939416
76501	Commd9	COMM domain containing 9	NA	0.017017	4.8212	1.011865	0.83560762	0.939731
620631	Ttc30a2	tetratricopeptide repeat domain 30A2	NA	-0.09746	-0.28036	-1.06989	0.83579613	0.939731
209966	Pgbd5	piggyBac transposable element derived 5	NA	0.013662	5.040929	1.009515	0.83595068	0.939731
17931	Ppp1r12a	protein phosphatase 1, regulatory subunit 12A, transc	NA	0.010673	6.58391	1.007426	0.83595398	0.939731
19359	Rad23b	RAD23 homolog B, nucleotide excision repair protein	NA	-0.00885	8.290533	-1.00615	0.83605034	0.939731
209357	Gtf2h3	general transcription factor IIH, polypeptide 3	NA	-0.01423	4.809259	-1.00991	0.83609851	0.939731
80289	Lysmd3	LysM, putative peptidoglycan-binding, domain contai	NA	0.01723	4.323255	1.012015	0.83612145	0.939731
97541	Qars	glutaminyl-tRNA synthetase	NA	0.010933	6.108769	1.007607	0.83614707	0.939731
107589	Mylk	myosin, light polypeptide kinase, transcript variant X4	NA	-0.01688	4.199064	-1.01177	0.83615636	0.939731
56847	Aldh1a3	aldehyde dehydrogenase family 1, subfamily A3	NA	-0.03707	1.874783	-1.02603	0.83616163	0.939731
114663	Impa2	inositol monophosphatase 2, transcript variant X1	NA	0.039741	2.131551	1.027929	0.83618272	0.939731
14186	Fgfr4	fibroblast growth factor receptor 4, transcript variant X	NA	-0.05356	1.03488	-1.03782	0.83632717	0.939828
243499	Lrrtm4	leucine rich repeat transmembrane neuronal 4, transc	NA	-0.01467	4.803827	-1.01022	0.83645338	0.939885
329470	Accs	1-aminocyclopropane-1-carboxylate synthase (non-fu	NA	0.024972	3.719316	1.01746	0.83649314	0.939885

272589	Tbcel	tubulin folding cofactor E-like, transcript variant 3	NA		0.014157	5.286995	1.009861	0.8366683	0.940001
228812	Pigu	phosphatidylinositol glycan anchor biosynthesis, clas:	NA		-0.01715	5.069003	-1.01196	0.83678815	0.940001
66487	Smim4	small integral membrane protein 4, transcript variant :	NA		0.036451	1.559071	1.025588	0.83682103	0.940001
434178	Zfp141	zinc finger protein 141, transcript variant 1	NA		-0.01428	4.505716	-1.00994	0.83693438	0.940001
217666	L2hgdh	L-2-hydroxyglutarate dehydrogenase	NA		0.019245	4.037769	1.013429	0.83694503	0.940001
103948	Rn7s1	7S RNA 1	NA		0.083383	3.607133	1.059499	0.8370479	0.940001
108150	Galnt7	polypeptide N-acetylgalactosaminyltransferase 7, trar	NA		-0.01782	4.025426	-1.01243	0.83706551	0.940001
14430	Galt	galactose-1-phosphate uridyl transferase, transcript v	NA		0.021499	3.867721	1.015013	0.8370927	0.940001
241226	Itga8	integrin alpha 8, transcript variant 1	NA		0.027901	2.639751	1.019527	0.83711584	0.940001
209361	Taf3	TATA-box binding protein associated factor 3	NA		-0.0157	4.677687	-1.01094	0.83740273	0.940066
20668	Sox13	SRY (sex determining region Y)-box 13, transcript va	NA		0.019965	4.293893	1.013935	0.83741844	0.940066
67647	4930523C07Rik	RIKEN cDNA 4930523C07 gene, transcript variant 1	NA		0.049807	0.980738	1.035126	0.83747522	0.940066
93834	Peli2	pellino 2, transcript variant X1	NA		0.01387	5.085753	1.00966	0.83749115	0.940066
20618	Sncg	synuclein, gamma	NA		0.033364	3.765588	1.023395	0.83768397	0.940066
13877	Erh	ERH mRNA splicing and mitosis factor, transcript var	NA		0.009178	7.448135	1.006382	0.83788623	0.940066
68480	Card19	caspase recruitment domain family, member 19	NA		-0.03076	2.168659	-1.02155	0.83790914	0.940066
77134	Hnrnpa0	heterogeneous nuclear ribonucleoprotein A0	NA		0.018473	9.203933	1.012887	0.83795308	0.940066
403174	Msantd1	Myb/SANT-like DNA-binding domain containing 1, tra	NA		0.028761	2.653345	1.020135	0.83805608	0.940066
240063	Zfp811	zinc finger protein 811, transcript variant 1	NA		0.015708	6.462233	1.010947	0.83808573	0.940066
71916	Dus4l	dihydrouridine synthase 4-like (S. cerevisiae)	NA		-0.02509	2.913312	-1.01755	0.8380874	0.940066
80902	Zfp202	zinc finger protein 202, transcript variant 3	NA		0.017548	4.193667	1.012238	0.83814738	0.940066
207798	Gramd1c	GRAM domain containing 1C, transcript variant 3	NA		0.039126	1.915612	1.027491	0.83816304	0.940066
12068	Bet1	Bet1 golgi vesicular membrane trafficking protein	NA		-0.01833	4.13427	-1.01279	0.83820763	0.940066
22174	Tyro3	TYRO3 protein tyrosine kinase 3, transcript variant X	NA		-0.01134	5.54695	-1.00789	0.83824074	0.940066
66622	Ubr7	ubiquitin protein ligase E3 component n-recognin 7 (p	NA		0.013792	5.897448	1.009606	0.83836741	0.940066
12654	Chil1	chitinase-like 1, transcript variant 2	NA		0.06614	0.386614	1.046912	0.83837053	0.940066
67239	Rpf2	ribosome production factor 2 homolog, transcript vari	NA		-0.01442	5.073675	-1.01004	0.83838039	0.940066
619719	6430590A07Rik	RIKEN cDNA 6430590A07 gene	NA		0.046179	0.747477	1.032526	0.83838457	0.940066
17688	Msh6	mutS homolog 6	NA		-0.01379	5.690443	-1.0096	0.83850625	0.940066
240055	Neurl1b	neuralized E3 ubiquitin protein ligase 1B	NA		-0.01292	5.182708	-1.00899	0.83852153	0.940066
626802	Gm14322	predicted gene 14322, transcript variant 2	NA		0.027156	3.233045	1.019001	0.83854165	0.940066
109660	Ctrl	chymotrypsin-like	NA		-0.061	0.478526	-1.04319	0.83859272	0.940066
52665	Echdc1	enoyl Coenzyme A hydratase domain containing 1, tr	NA		-0.01877	4.349918	-1.0131	0.83865558	0.940066
73847	Fam110a	family with sequence similarity 110, member A, trans	NA		-0.0161	4.77567	-1.01122	0.83873762	0.940066
20556	Slfn2	schlafen 2	NA		0.071703	-0.02721	1.050957	0.83874307	0.940066
26889	Cln8	CLN8 transmembrane ER and ERGIC protein	NA		-0.01463	4.968325	-1.01019	0.83877169	0.940066
22003	Tpm1	tropomyosin 1, alpha, transcript variant X7	NA		0.011606	7.043288	1.008077	0.83881195	0.940066
668166	Zxdb	zinc finger, X-linked, duplicated B	NA		0.018378	4.014994	1.01282	0.83884933	0.940066
14718	Got1	glutamic-oxaloacetic transaminase 1, soluble	NA		-0.01106	6.059104	-1.0077	0.83912671	0.940312
21941	Tnfrsf8	tumor necrosis factor receptor superfamily, member 8	NA		0.067594	-0.14214	1.047968	0.83920689	0.940337
74682	Wdr35	WD repeat domain 35, transcript variant 1	NA		0.015503	5.008776	1.010804	0.8395852	0.940656
20675	Sox3	SRY (sex determining region Y)-box 3	NA		-0.02107	4.122468	-1.01471	0.8396068	0.940656
140571	Plxnb3	plexin B3	NA		0.055992	0.423146	1.039573	0.83968889	0.940683
225372	Apbb3	amyloid beta (A4) precursor protein-binding, family B	NA		-0.01641	4.439803	-1.01144	0.83984965	0.940798
286942	Kif19a	kinesin family member 19A	NA		0.048936	0.940356	1.034502	0.84046026	0.941159
118568348	LOC118568348	igE-binding protein-like	NA		-0.03585	1.465349	-1.02516	0.8405219	0.941159
102636262	Gm20544	predicted gene 20544	NA		0.022385	3.157686	1.015637	0.84058338	0.941159
140743	Rem2	rad and gem related GTP binding protein 2	NA		0.018247	4.280267	1.012728	0.84059174	0.941159
68999	Anapc10	anaphase promoting complex subunit 10, transcript v	NA		-0.01618	4.409354	-1.01128	0.84059322	0.941159
23937	Mab21l2	mab-21-like 2	NA		-0.02112	5.796665	-1.01475	0.84062651	0.941159
74251	Ankrd9	ankyrin repeat domain 9, transcript variant X1	NA		-0.04805	0.73426	-1.03387	0.84063101	0.941159
12300	Cacng2	calcium channel, voltage-dependent, gamma subunit	NA		0.013295	6.138542	1.009258	0.8406348	0.941159
12159	Bmp4	bone morphogenetic protein 4, transcript variant 1	NA		0.03224	2.21471	1.022599	0.84076795	0.941244
80285	Parp9	poly (ADP-ribose) polymerase family, member 9, tran	NA		-0.03523	1.602523	-1.02472	0.84090696	0.941334
234683	Elmo3	engulfment and cell motility 3	NA		-0.04815	0.786462	-1.03394	0.84106831	0.941382
319535	Zfp182	zinc finger protein 182, transcript variant X2	NA		0.015117	4.473968	1.010533	0.84106898	0.941382
606496	Gsk3a	glycogen synthase kinase 3 alpha, transcript variant)	NA		0.009082	7.712404	1.006315	0.8411588	0.941382
66776	Pisd-ps3	phosphatidylserine decarboxylase, pseudogene 3	NA		0.011535	8.397795	1.008027	0.84121246	0.941382
66059	Krtcap2	keratinocyte associated protein 2, transcript variant X	NA		-0.01628	4.974407	-1.01135	0.84128627	0.941382
60510	Syt9	synaptotagmin IX, transcript variant X2	NA		0.01527	4.668573	1.010641	0.84136446	0.941382
23920	Insrr	insulin receptor-related receptor, transcript variant X2	NA		0.053811	0.672626	1.038003	0.84142803	0.941382
330286	D630045J12Rik	RIKEN cDNA D630045J12 gene	NA		-0.01106	7.317286	-1.00769	0.84149847	0.941382
791299	Gm10069	predicted gene 10069, transcript variant 1	NA		0.085691	0.096714	1.061196	0.84154831	0.941382
66116	Nat8f1	N-acetyltransferase 8 (GCN5-related) family member	NA		0.036406	1.888424	1.025556	0.84160754	0.941382
208285	Cyp4f17	cytochrome P450, family 4, subfamily f, polypeptide 1	NA		0.024378	3.595266	1.017041	0.84162837	0.941382
12807	Hps3	HPS3, biogenesis of lysosomal organelles complex 2	NA		0.017862	4.150487	1.012458	0.84168167	0.941382
109594	Lmo1	LIM domain only 1, transcript variant 1	NA		0.021146	5.056091	1.014765	0.84173823	0.941382
225745	Haus1	HAUS augmin-like complex, subunit 1, transcript vari	NA		0.023548	3.711518	1.016456	0.8417872	0.941382

77022	2700099C18Rik	RIKEN cDNA 2700099C18 gene	NA	-0.02778	2.401208	-1.01945	0.84181737	0.941382
27399	Ip6k1	inositol hexaphosphate kinase 1	NA	0.008721	7.64586	1.006063	0.84192029	0.94143
105298	Epdr1	ependymin related protein 1 (zebrafish)	NA	-0.02001	4.023051	-1.01397	0.84197581	0.94143
100504360	D730003I15Rik	RIKEN cDNA D730003I15 gene	NA	0.052576	0.749597	1.037115	0.84228235	0.94166
11936	Fxyd2	FXYD domain-containing ion transport regulator 2, tra	NA	0.049384	1.10055	1.034823	0.84229691	0.94166
330427	9330102E08Rik	RIKEN cDNA 9330102E08 gene	NA	-0.03	2.125278	-1.02101	0.84239845	0.941708
71876	Cenpu	centromere protein U, transcript variant X1	NA	-0.02923	2.591767	-1.02047	0.84256562	0.941831
109333	Pkn2	protein kinase N2	NA	-0.01123	5.664771	-1.00782	0.84268082	0.941895
73910	Arhgap18	Rho GTPase activating protein 18	NA	-0.01584	4.576767	-1.01104	0.84288928	0.942052
102639087	Gm35486	predicted gene, 35486	NA	0.022512	3.420996	1.015727	0.8429372	0.942052
241494	Zfp385b	zinc finger protein 385B, transcript variant 2	NA	0.025532	3.612066	1.017855	0.84301866	0.942078
105243252	Gm39223	predicted gene, 39223, transcript variant X2	NA	0.057752	0.750504	1.040843	0.84318404	0.942176
56177	Olfm1	olfactomedin 1, transcript variant 3	NA	0.009628	8.419075	1.006696	0.8432748	0.942176
71865	Fbxo30	F-box protein 30, transcript variant X1	NA	-0.01657	5.22972	-1.01155	0.84330874	0.942176
115488282	Gm3512	predicted gene 3512, transcript variant X10	NA	0.035846	2.147445	1.025158	0.84333818	0.942176
23962	Oasl2	2'-5' oligoadenylate synthetase-like 2, transcript varia	NA	0.042351	1.283474	1.029791	0.84348339	0.942274
238317	Mideas	mitotic deacetylase associated SANT domain protein	NA	-0.01528	4.535664	-1.01065	0.84365549	0.942323
78913	Ltn1	listerin E3 ubiquitin protein ligase 1	NA	0.01307	5.625771	1.0091	0.84365739	0.942323
100038734	Gm10845	predicted gene 10845	NA	0.043744	1.053043	1.030785	0.843701	0.942323
69513	1700030C10Rik	RIKEN cDNA 1700030C10 gene	NA	0.058172	0.202636	1.041146	0.84379446	0.942363
19335	Rab23	RAB23, member RAS oncogene family, transcript var	NA	-0.01159	5.383847	-1.00806	0.8439622	0.942462
80509	Med8	mediator complex subunit 8, transcript variant X2	NA	0.013571	5.243838	1.009451	0.84399928	0.942462
66440	Cdc26	cell division cycle 26, transcript variant 1	NA	0.013244	5.925882	1.009222	0.84406766	0.942462
243362	Stard13	StAR-related lipid transfer (START) domain containin	NA	-0.01948	3.483241	-1.01359	0.84411531	0.942462
211948	Pde12	phosphodiesterase 12	NA	0.017368	4.592192	1.012111	0.8442002	0.942492
74665	Drc3	dynein regulatory complex subunit 3, transcript variar	NA	-0.02972	2.19707	-1.02082	0.84425831	0.942493
100042584	Gm16523	predicted gene, 16523	NA	-0.07185	-0.17567	-1.05106	0.84440401	0.942591
75627	Snapc1	small nuclear RNA activating complex, polypeptide 1	NA	0.015343	5.751114	1.010692	0.84472805	0.942888
22693	Zfp30	zinc finger protein 30, transcript variant 2	NA	-0.01477	4.556596	-1.01029	0.84479088	0.942893
69602	Otop3	otopetrin 3, transcript variant 2	NA	0.066356	-0.4211	1.047069	0.8449146	0.942933
27081	Zfp275	zinc finger protein 275, transcript variant 2	NA	-0.01503	5.412472	-1.01047	0.84502816	0.942933
50789	Fbxl3	F-box and leucine-rich repeat protein 3, transcript var	NA	0.016452	5.697878	1.011469	0.84505122	0.942933
17859	Mxi1	MAX interactor 1, dimerization protein, transcript varia	NA	0.01102	6.195597	1.007668	0.84512708	0.942933
13537	Dusp2	dual specificity phosphatase 2	NA	0.034021	1.869663	1.023862	0.8451381	0.942933
75624	Metap1	methionyl aminopeptidase 1	NA	-0.01087	5.757293	-1.00756	0.84521988	0.942933
239122	Setdb2	SET domain, bifurcated 2, transcript variant 1	NA	-0.03629	2.349914	-1.02547	0.8452325	0.942933
331004	Slc9a9	solute carrier family 9 (sodium/hydrogen exchanger),	NA	0.026129	3.004829	1.018276	0.84533617	0.942984
71592	Pogk	pogo transposable element with KRAB domain, trans	NA	0.009165	7.300326	1.006373	0.84548608	0.943087
71767	Tysnd1	trypsin domain containing 1, transcript variant 5	NA	0.016149	4.368984	1.011256	0.84565462	0.94321
67416	Armcx2	armadillo repeat containing, X-linked 2, transcript vari	NA	-0.00899	7.527358	-1.00625	0.84572836	0.943228
404634	Macroh2a2	macroH2A.2 histone	NA	-0.00853	7.902244	-1.00593	0.84584321	0.943256
20912	Stxbp3	syntaxin binding protein 3	NA	0.021032	3.515172	1.014685	0.84591115	0.943256
223648	Ccdc166	coiled-coil domain containing 166, transcript variant 2	NA	0.017951	3.68124	1.01252	0.84592749	0.943256
12608	Cebpb	CCAAT/enhancer binding protein (C/EBP), beta, tran	NA	0.05082	0.972833	1.035854	0.8461339	0.943385
99543	Olfml3	olfactomedin-like 3	NA	-0.01924	3.542957	-1.01342	0.84615904	0.943385
118568236	LOC118568236	uncharacterized LOC118568236	NA	-0.04864	0.967767	-1.03429	0.84622382	0.943392
226412	R3hdm1	R3H domain containing 1, transcript variant X20	NA	0.009419	7.255409	1.00655	0.84629875	0.943411
625098	Slc38a6	solute carrier family 38, member 6, transcript variant	NA	-0.02155	4.843604	-1.01505	0.84653481	0.94361
244745	Dpy19l1	dpy-19-like 1 (C. elegans), transcript variant 1	NA	0.011788	6.843113	1.008204	0.84661525	0.943635
74646	Spsb1	splA/ryanodine receptor domain and SOCS box conta	NA	0.031474	2.883993	1.022056	0.8467534	0.943695
59093	Pcbp3	poly(rC) binding protein 3, transcript variant X17	NA	-0.01197	5.351055	-1.00833	0.84683454	0.943695
94254	Rcc1l	reculator of chromosome condensation 1 like	NA	0.016208	4.0194	1.011298	0.84689692	0.943695
66441	Magohb	mago homolog B, exon junction complex core compo	NA	0.020652	3.568566	1.014418	0.84690135	0.943695
210274	Shank2	SH3 and multiple ankyrin repeat domains 2, transcrip	NA	0.011072	6.700965	1.007704	0.84697135	0.943708
55949	Eef1b2	eukaryotic translation elongation factor 1 beta 2	NA	0.008599	8.193062	1.005978	0.84708539	0.943771
96957	Tmem62	transmembrane protein 62, transcript variant X2	NA	-0.01726	3.910886	-1.01204	0.84717826	0.94381
108098	Med21	mediator complex subunit 21	NA	-0.01532	4.366669	-1.01068	0.84736138	0.943944
105727	Slc38a1	solute carrier family 38, member 1, transcript variant	NA	0.010944	7.983026	1.007615	0.84741461	0.943944
76161	Lamp5	lysosomal-associated membrane protein family, mem	NA	0.034633	3.968978	1.024296	0.84762528	0.944045
65247	Asb1	ankyrin repeat and SOCS box-containing 1, transcrip	NA	0.014212	4.966777	1.0099	0.84773988	0.944045
18949	Pnn	pinin	NA	-0.00851	7.341483	-1.00591	0.84774824	0.944045
76594	Dnajc18	DnaJ heat shock protein family (Hsp40) member C18	NA	-0.00947	6.236629	-1.00659	0.84785352	0.944045
68980	Wdr53	WD repeat domain 53, transcript variant 1	NA	-0.0223	3.062785	-1.01558	0.84785846	0.944045
268656	Sptlc1	serine palmitoyltransferase, long chain base subunit	NA	0.016973	4.872952	1.011834	0.84790445	0.944045
17113	M6pr	mannose-6-phosphate receptor, cation dependent	NA	-0.01066	5.742031	-1.00742	0.84791163	0.944045
102638376	Gm34957	predicted gene, 34957	NA	-0.03152	2.098222	-1.02209	0.84798421	0.944061
100504114	Gm16907	predicted gene, 16907	NA	0.053098	0.786743	1.037491	0.84828249	0.944326
66433	Chchd7	coiled-coil-helix-coiled-coil-helix domain containing 7,	NA	0.016476	3.933607	1.011486	0.848406	0.944326

114479	Slc5a5	solute carrier family 5 (sodium iodide symporter), mei	NA	0.051517	1.029421	1.036354	0.84852399	0.944326
14950	H13	histocompatibility 13, transcript variant 4	NA	0.010352	7.240031	1.007202	0.84852868	0.944326
230027	Coq3	coenzyme Q3 methyltransferase, transcript variant 1	NA	-0.01786	4.123405	-1.01246	0.84854493	0.944326
71986	Ddx28	DEAD box helicase 28	NA	0.018911	3.87288	1.013194	0.84856988	0.944326
102633880	A730081D07Rik	RIKEN cDNA A730081D07 gene	NA	-0.06194	0.09027	-1.04387	0.84871481	0.944381
73873	Fam161a	family with sequence similarity 161, member A, transc	NA	0.041025	2.400683	1.028845	0.84878021	0.944381
57429	Sult5a1	sulfotransferase family 5A, member 1	NA	-0.05581	0.198678	-1.03944	0.84879417	0.944381
100039968	Tmem35b	transmembrane protein 35B	NA	-0.02873	2.355593	-1.02011	0.84900503	0.944551
115488769	LOC115488769	uncharacterized LOC115488769	NA	0.047916	2.067676	1.033771	0.84912154	0.944617
330217	Gal3st4	galactose-3-O-sulfotransferase 4, transcript variant 2	NA	0.051857	0.145194	1.036599	0.8492128	0.944653
68202	Ndufa5	NADH:ubiquinone oxidoreductase subunit A5, transci	NA	0.011639	5.362734	1.0081	0.84955949	0.944975
16431	Itm2a	integral membrane protein 2A	NA	0.009825	6.293825	1.006833	0.8497749	0.945149
67532	Mfap1a	microfibrillar-associated protein 1A	NA	-0.01109	6.14845	-1.00772	0.85004589	0.945324
108853	Mtrf1l	mitochondrial translational release factor 1-like	NA	0.020589	3.438124	1.014373	0.85004771	0.945324
226518	Nmnat2	nicotinamide nucleotide adenylyltransferase 2	NA	0.009927	7.427627	1.006904	0.85016278	0.945358
213539	Bag2	BCL2-associated athanogene 2	NA	-0.01764	3.545388	-1.0123	0.85019472	0.945358
17876	Myef2	myelin basic protein expression factor 2, repressor, tr	NA	-0.00835	8.33277	-1.0058	0.85066263	0.945814
269952	Gdpgp1	GDP-D-glucose phosphorylase 1	NA	-0.03442	2.054122	-1.02414	0.85075423	0.945851
64384	Sirt3	sirtuin 3, transcript variant 2	NA	-0.01577	3.901282	-1.01099	0.85089683	0.945945
94222	Olig3	oligodendrocyte transcription factor 3	NA	0.05377	0.655485	1.037974	0.85104184	0.945974
228136	Zdhhc5	zinc finger, DHHC domain containing 5	NA	0.010905	5.924498	1.007588	0.8511802	0.945974
69837	Pcgf1	polycomb group ring finger 1	NA	-0.01934	3.44522	-1.0135	0.85118178	0.945974
19672	Rcn1	reticulocalbin 1	NA	0.01124	6.126107	1.007821	0.85121214	0.945974
230801	Pigv	phosphatidylinositol glycan anchor biosynthesis, clas	NA	0.017043	3.902381	1.011883	0.85129629	0.945974
57354	Cramp1l	cramped chromatin regulator homolog 1, transcript va	NA	-0.01166	5.808136	-1.00811	0.85130338	0.945974
207596	Thsd4	thrombospondin, type I, domain containing 4, transcri	NA	0.01649	4.023793	1.011495	0.85136085	0.945974
73010	Gpr22	G protein-coupled receptor 22	NA	-0.01739	3.938947	-1.01213	0.85139262	0.945974
64050	Yeats4	YEATS domain containing 4	NA	0.010113	6.416029	1.007035	0.85152321	0.945974
140484	Pofut1	protein O-fucosyltransferase 1	NA	0.013795	5.190689	1.009608	0.85153054	0.945974
71908	Cldn23	claudin 23	NA	-0.06304	-0.10305	-1.04466	0.85156263	0.945974
223690	Ankrd54	ankyrin repeat domain 54, transcript variant 1	NA	-0.01653	4.585522	-1.01152	0.85183071	0.946126
72307	2510002D24Rik	RIKEN cDNA 2510002D24 gene, transcript variant X	NA	0.022187	3.731492	1.015498	0.85186349	0.946126
211147	Marchf11	membrane associated ring-CH-type finger 11, transcr	NA	0.030917	2.309489	1.021661	0.85187397	0.946126
72383	Cnfn	cornifelin, transcript variant 1	NA	0.137319	-1.27095	1.099859	0.85193441	0.946129
67948	Fbxo28	F-box protein 28	NA	-0.01033	6.029523	-1.00718	0.85204476	0.946158
234839	Piezo1	piezo-type mechanosensitive ion channel component	NA	-0.02085	4.059728	-1.01455	0.85208706	0.946158
320111	Prr18	proline rich 18, transcript variant 1	NA	0.010984	5.329383	1.007642	0.85213567	0.946158
234356	Csgalnact1	chondroitin sulfate N-acetylgalactosaminyltransferase	NA	-0.02717	2.591398	-1.01901	0.85225257	0.946224
21898	Tlr4	toll-like receptor 4, transcript variant X1	NA	-0.03791	1.153155	-1.02663	0.85247542	0.94637
18038	Nfkbil1	nuclear factor of kappa light polypeptide gene enhanc	NA	-0.02293	3.224778	-1.01602	0.85257934	0.94637
107094	Rrp12	ribosomal RNA processing 12 homolog, transcript vai	NA	-0.0116	5.018895	-1.00807	0.85259718	0.94637
68347	Mettl26	methyltransferase like 26	NA	-0.02165	3.104745	-1.01512	0.85264445	0.94637
225010	Lclat1	lysocardiolipin acyltransferase 1, transcript variant 2	NA	0.012521	5.014895	1.008717	0.85267511	0.94637
12799	Cnp	2',3'-cyclic nucleotide 3' phosphodiesterase, transcrip	NA	0.013383	4.611926	1.009319	0.85276636	0.946394
29808	Mga	MAX gene associated, transcript variant X16	NA	0.010284	6.283398	1.007154	0.85281304	0.946394
105245048	Gm40557	predicted gene, 40557, transcript variant X1	NA	-0.05655	0.188905	-1.03998	0.85297344	0.946507
102635912	C030005K06Rik	RIKEN cDNA C030005K06 gene	NA	-0.03698	1.409306	-1.02597	0.85313613	0.946623
26432	Plod2	procollagen lysine, 2-oxoglutarate 5-dioxygenase 2, t	NA	0.012816	4.58516	1.008923	0.85341086	0.946799
225215	Rsl24d1	ribosomal L24 domain containing 1	NA	0.00955	6.054415	1.006642	0.85344487	0.946799
109270	Prr5	proline rich 5 (renal), transcript variant X1	NA	-0.0141	4.682445	-1.00982	0.85346937	0.946799
80719	Igsf6	immunoglobulin superfamily, member 6	NA	-0.06475	-0.24893	-1.04591	0.85359734	0.946819
228730	Kiz	kizuna centrosomal protein	NA	-0.01438	4.380769	-1.01002	0.85360334	0.946819
319887	E030030I06Rik	RIKEN cDNA E030030I06 gene, transcript variant 1	NA	-0.06401	-0.15941	-1.04537	0.85383749	0.946981
11536	Gpr182	G protein-coupled receptor 182	NA	0.046398	0.60323	1.032684	0.85386587	0.946981
71354	Wdr31	WD repeat domain 31, transcript variant X2	NA	0.020084	3.505855	1.014019	0.85411214	0.947182
108168812	Gm46774	predicted gene, 46774	NA	-0.0631	0.61114	-1.04471	0.85416377	0.947182
67580	Lrrc18	leucine rich repeat containing 18, transcript variant X:	NA	0.076903	-0.33879	1.054751	0.85431154	0.947282
71872	Aox4	aldehyde oxidase 4, transcript variant X2	NA	-0.06581	1.263334	-1.04667	0.85437073	0.947283
79044	Mrps34	mitochondrial ribosomal protein S34	NA	-0.02047	4.257113	-1.01429	0.85446639	0.947324
14664	Slc6a9	solute carrier family 6 (neurotransmitter transporter, g	NA	0.014181	4.864776	1.009878	0.85460681	0.947346
108673	Ccdc86	coiled-coil domain containing 86	NA	-0.01667	3.865193	-1.01162	0.85465128	0.947346
52530	Nhp2	NHP2 ribonucleoprotein, transcript variant 1	NA	0.016696	4.703851	1.01164	0.8547253	0.947346
67706	Tmem179b	transmembrane protein 179B, transcript variant 1	NA	-0.03022	2.054395	-1.02117	0.85477122	0.947346
11831	Aqp6	aquaporin 6	NA	-0.06722	-0.32938	-1.04769	0.854825	0.947346
217166	Nr1d1	nuclear receptor subfamily 1, group D, member 1	NA	0.02624	2.613313	1.018355	0.85489989	0.947346
208501	Ndufaf8	NADH:ubiquinone oxidoreductase complex assembly	NA	-0.03509	1.544432	-1.02462	0.85494176	0.947346
319352	Pianp	PILR alpha associated neural protein, transcript varia	NA	0.010981	6.014023	1.007641	0.85495201	0.947346
213895	Bms1	BMS1, ribosome biogenesis factor	NA	-0.01026	5.824437	-1.00714	0.85507054	0.94737

60455	Pgap6	post-glycosylphosphatidylinositol attachment to prote	NA	-0.02412	2.540537	-1.01686	0.85508964	0.94737
54609	Ubqln2	ubiquilin 2	NA	-0.0091	8.111029	-1.00633	0.85516247	0.947386
380608	Tagap1	T cell activation GTPase activating protein 1	NA	-0.01238	5.274695	-1.00862	0.85544137	0.94763
70356	St13	suppression of tumorigenicity 13, transcript variant X	NA	-0.01003	7.749555	-1.00697	0.8555345	0.947661
72338	Wdr89	WD repeat domain 89	NA	-0.02979	2.104975	-1.02086	0.85558548	0.947661
102635360	Gm32719	predicted gene, 32719	NA	-0.04649	0.349923	-1.03275	0.85569568	0.947717
224823	Rrp36	ribosomal RNA processing 36, transcript variant 1	NA	0.014779	4.160085	1.010297	0.8558048	0.947717
19085	Prkar1b	protein kinase, cAMP dependent regulatory, type I be	NA	0.0088	7.927514	1.006119	0.85587555	0.947717
230770	Tmem39b	transmembrane protein 39b, transcript variant X5	NA	-0.01204	4.918032	-1.00838	0.85589908	0.947717
319477	Insyn1	inhibitory synaptic factor 1	NA	0.010658	5.623537	1.007415	0.85608633	0.947717
108167802	Gm46218	predicted gene, 46218	NA	0.027202	2.353543	1.019034	0.85610383	0.947717
53970	Rfx5	regulatory factor X, 5 (influences HLA class II expres	NA	0.014195	4.786748	1.009888	0.85613704	0.947717
94067	Mrpl43	mitochondrial ribosomal protein L43	NA	0.013975	4.980856	1.009734	0.85619745	0.947717
26356	Ing1	inhibitor of growth family, member 1, transcript varian	NA	0.011643	6.257023	1.008103	0.85620126	0.947717
67249	Tbc1d19	TBC1 domain family, member 19, transcript variant X	NA	0.011168	5.759422	1.007771	0.85621884	0.947717
66642	Ctnnbl1	catenin, beta like 1	NA	-0.01089	5.683009	-1.00757	0.85647263	0.947816
69241	Polr2d	polymerase (RNA) II (DNA directed) polypeptide D, tr	NA	0.012143	5.083703	1.008452	0.85650368	0.947816
209462	Hace1	HECT domain and ankyrin repeat containing, E3 ubic	NA	0.016317	4.471173	1.011374	0.85652863	0.947816
223978	Cpped1	calcineurin-like phosphoesterase domain containing	NA	0.014019	4.65403	1.009764	0.8565865	0.947816
69860	Eif1ad	eukaryotic translation initiation factor 1A domain cont	NA	0.010917	5.355217	1.007596	0.85663099	0.947816
12010	B2m	beta-2 microglobulin	NA	0.017627	4.650576	1.012293	0.85665759	0.947816
67025	Rpl11	ribosomal protein L11, transcript variant X1	NA	0.011605	8.446834	1.008076	0.85694644	0.947971
102632594	A330001L22Rik	RIKEN cDNA A330001L22 gene, transcript variant X	NA	-0.03712	1.438906	-1.02607	0.85697427	0.947971
11767	Ap1m1	adaptor-related protein complex AP-1, mu subunit 1	NA	0.008883	7.286289	1.006176	0.85703162	0.947971
73884	Zdbf2	zinc finger, DBF-type containing 2, transcript variant	NA	-0.01077	6.434053	-1.00749	0.85704153	0.947971
14276	Folr2	folate receptor 2 (fetal), transcript variant 3	NA	0.059093	-0.35631	1.041811	0.85713115	0.947971
68979	Nol11	nucleolar protein 11, transcript variant 1	NA	-0.01228	5.635809	-1.00855	0.85717081	0.947971
382236	Brwd3	bromodomain and WD repeat domain containing 3	NA	-0.0137	5.031012	-1.00954	0.85720566	0.947971
19340	Rab3d	RAB3D, member RAS oncogene family, transcript va	NA	-0.01304	4.974701	-1.00908	0.8573905	0.948111
240476	Zfp407	zinc finger protein 407	NA	-0.01398	4.356482	-1.00974	0.85758189	0.948259
100504601	Gm16702	predicted gene, 16702	NA	0.025446	2.441636	1.017794	0.85769426	0.948318
14174	Fgf3	fibroblast growth factor 3	NA	-0.04747	0.695544	-1.03345	0.85779919	0.94837
115488122	Gm36445	predicted gene, 36445	NA	0.04905	0.295532	1.034583	0.85794115	0.948454
233066	Syne4	spectrin repeat containing, nuclear envelope family m	NA	-0.03947	1.081166	-1.02773	0.85799159	0.948454
18685	Phtf1	putative homeodomain transcription factor 1, transcri	NA	-0.01142	5.468704	-1.00795	0.85810922	0.948501
72789	Veph1	ventricular zone expressed PH domain-containing 1	NA	-0.02039	3.440753	-1.01423	0.85821743	0.948501
21823	Th	tyrosine hydroxylase	NA	-0.02193	2.667237	-1.01531	0.85837701	0.948501
215512	Fam117a	family with sequence similarity 117, member A	NA	0.025521	2.740917	1.017847	0.85846804	0.948501
216150	Cdc34	cell division cycle 34, transcript variant X1	NA	-0.01394	5.170277	-1.00971	0.85851196	0.948501
64540	Tspan4	tetraspanin 4, transcript variant 2	NA	0.015183	4.038838	1.01058	0.85853848	0.948501
102635406	Gm32756	predicted gene, 32756, transcript variant X2	NA	0.045917	0.623437	1.032339	0.85860302	0.948501
223774	Alg12	asparagine-linked glycosylation 12 (alpha-1,6-manno	NA	-0.01788	3.397748	-1.01247	0.85860581	0.948501
102636338	4930500F10Rik	RIKEN cDNA 4930500F10 gene, transcript variant X	NA	0.05126	0.245187	1.03617	0.85869259	0.948501
68981	Snrpa1	small nuclear ribonucleoprotein polypeptide A', transc	NA	0.014679	5.614393	1.010227	0.85870055	0.948501
65967	Eefsec	eukaryotic elongation factor, selenocysteine-tRNA-sp	NA	-0.01378	4.30941	-1.0096	0.85870919	0.948501
50784	Plpp2	phospholipid phosphatase 2, transcript variant 3	NA	0.022703	2.787945	1.015861	0.8587335	0.948501
66768	Pacrgl	PARK2 co-regulated-like	NA	-0.02503	3.011178	-1.0175	0.85886006	0.948501
232314	Ppp4r2	protein phosphatase 4, regulatory subunit 2, transcrip	NA	0.010673	7.188273	1.007425	0.85892101	0.948501
20587	Smarcb1	SWI/SNF related, matrix associated, actin dependent	NA	-0.0082	7.201749	-1.0057	0.85896471	0.948501
215051	Bud13	BUD13 homolog	NA	-0.01711	4.12681	-1.01193	0.85900815	0.948501
210741	Kcnk12	potassium channel, subfamily K, member 12, transcri	NA	0.033245	1.905332	1.023311	0.85902509	0.948501
231474	Paqr3	progestin and adipoQ receptor family member III, tran	NA	0.014971	5.697645	1.010431	0.85911942	0.948541
12062	Bdkrb2	bradykinin receptor, beta 2, transcript variant X3	NA	-0.03963	1.1315	-1.02785	0.85918583	0.94855
381404	Pabpc1l	poly(A) binding protein, cytoplasmic 1-like, transcript	NA	-0.0547	0.491546	-1.03864	0.8593589	0.948676
320051	Exph5	exophilin 5, transcript variant X4	NA	0.071294	-0.04234	1.050659	0.85951705	0.948768
380694	Ccnjl	cyclin J-like	NA	-0.02059	3.127253	-1.01438	0.8595584	0.948768
278795	Lrrc10b	leucine rich repeat containing 10B	NA	-0.0358	1.08258	-1.02512	0.85966267	0.948774
74375	Gcc1	golgi coiled coil 1	NA	0.014776	4.398664	1.010295	0.85970649	0.948774
13486	Dr1	down-regulator of transcription 1	NA	0.011204	5.85278	1.007796	0.85973863	0.948774
75693	3010001F23Rik	RIKEN cDNA 3010001F23 gene, transcript variant 1	NA	0.026102	2.158405	1.018257	0.85983942	0.948816
434437	Amt	aminomethyltransferase	NA	-0.01228	4.950535	-1.00855	0.85992037	0.948816
211499	Tmem87a	transmembrane protein 87A, transcript variant 2	NA	-0.01201	5.128409	-1.00836	0.8599531	0.948816
320145	Sp8	trans-acting transcription factor 8, transcript variant 1	NA	-0.01941	3.922502	-1.01355	0.86002234	0.948816
239667	Dip2b	disco interacting protein 2 homolog B, transcript varia	NA	-0.00906	6.444822	-1.0063	0.86015656	0.948816
328906	Gm15328	predicted gene 15328	NA	0.035398	2.209708	1.024839	0.86016377	0.948816
56336	B4galt5	UDP-Gal:betaGlcNAc beta 1,4-galactosyltransferase,	NA	-0.00995	6.171192	-1.00692	0.86025787	0.948816
111002	Marcksl1-ps4	MARCKS-like 1, pseudogene 4	NA	0.058617	0.040586	1.041467	0.86026394	0.948816
68040	Zfp593	zinc finger protein 593	NA	-0.01791	3.266743	-1.01249	0.86031977	0.948816

56807	Scamp5	secretory carrier membrane protein 5, transcript varia	NA	0.008108	7.689224	1.005636	0.86039075	0.948816
223843	Dbx2	developing brain homeobox 2, transcript variant X1	NA	0.032504	1.323172	1.022786	0.86046373	0.948816
28240	Trpm2	transient receptor potential cation channel, subfamily	NA	-0.02994	2.827978	-1.02097	0.86052233	0.948816
216343	Tph2	tryptophan hydroxylase 2	NA	0.032867	1.980528	1.023043	0.86053515	0.948816
66335	Atp6v1c1	ATPase, H+ transporting, lysosomal V1 subunit C1	NA	-0.00914	6.185499	-1.00636	0.86061878	0.948844
54353	Skap2	src family associated phosphoprotein 2	NA	-0.02442	2.504338	-1.01707	0.86074559	0.948862
76510	Trappc9	trafficking protein particle complex 9, transcript variar	NA	-0.01004	5.706337	-1.00698	0.8607513	0.948862
18807	Pld3	phospholipase D family, member 3, transcript variant	NA	-0.0087	6.555031	-1.00605	0.86086341	0.948921
12738	Cldn2	claudin 2	NA	-0.04013	0.991121	-1.0282	0.86155942	0.949411
218772	Rarb	retinoic acid receptor, beta, transcript variant beta4	NA	0.015198	4.04428	1.01059	0.86169171	0.949411
118568655	LOC118568655	uncharacterized LOC118568655	NA	0.040051	1.078579	1.02815	0.86169516	0.949411
16410	Itgav	integrin alpha V	NA	0.018024	4.546974	1.012572	0.86180423	0.949411
69660	Tmbim1	transmembrane BAX inhibitor motif containing 1	NA	0.022306	2.59638	1.015582	0.86188977	0.949411
216767	Mrpl22	mitochondrial ribosomal protein L22, transcript varian	NA	0.020818	4.028498	1.014535	0.86189102	0.949411
22032	Traf4	TNF receptor associated factor 4	NA	-0.00894	6.556016	-1.00621	0.86191166	0.949411
78894	Aacs	acetoacetyl-CoA synthetase	NA	-0.00937	6.12459	-1.00652	0.86197326	0.949411
231147	Sh3tc1	SH3 domain and tetratricopeptide repeats 1, transcrip	NA	-0.03526	1.477423	-1.02474	0.86200862	0.949411
100039441	Gm2237	predicted gene 2237, transcript variant 1	NA	-0.02168	3.774643	-1.01514	0.86213178	0.949411
380753	Atxn7l1	ataxin 7-like 1, transcript variant X15	NA	0.013964	5.151361	1.009726	0.86215256	0.949411
257633	Acsf3	acyl-CoA synthetase family member 3, transcript vari	NA	0.019191	3.902111	1.013391	0.86216761	0.949411
50909	C1ra	complement component 1, r subcomponent A	NA	-0.0673	-0.42177	-1.04775	0.86216882	0.949411
238386	Btbd7	BTB (POZ) domain containing 7, transcript variant 2	NA	0.010897	6.077403	1.007582	0.86218931	0.949411
230761	Zfp362	zinc finger protein 362	NA	-0.0084	6.812587	-1.00584	0.8622068	0.949411
67458	Ergic1	endoplasmic reticulum-golgi intermediate compartme	NA	-0.00826	7.410684	-1.00574	0.86224132	0.949411
320784	C230072F16Rik	RIKEN cDNA C230072F16 gene, transcript variant 1	NA	-0.04734	0.393449	-1.03336	0.86245435	0.949581
69051	Pycr2	pyrroline-5-carboxylate reductase family, member 2	NA	-0.01514	4.514697	-1.01055	0.86252325	0.949591
72446	Prr5l	proline rich 5 like, transcript variant 4	NA	0.025659	2.037346	1.017945	0.86258043	0.949591
57914	Crlf2	cytokine receptor-like factor 2, transcript variant 2	NA	-0.02046	3.17553	-1.01428	0.86274855	0.949712
193740	Hspa1a	heat shock protein 1A	NA	-0.02593	2.67206	-1.01814	0.86286779	0.949718
57315	Wdr46	WD repeat domain 46	NA	-0.01344	4.743476	-1.00936	0.86287082	0.949718
269800	Zfp384	zinc finger protein 384, transcript variant 3	NA	-0.00888	6.166858	-1.00617	0.86303037	0.949775
20909	Stx4a	syntaxin 4A (placental)	NA	0.013304	4.358833	1.009264	0.86311032	0.949775
75705	Eif4b	eukaryotic translation initiation factor 4B	NA	-0.00853	8.456228	-1.00593	0.8631317	0.949775
238276	Akap5	A kinase (PRKA) anchor protein 5	NA	0.013016	4.8543	1.009063	0.86315562	0.949775
16598	Klf2	Kruppel-like factor 2 (lung)	NA	0.02655	3.059246	1.018574	0.86327728	0.949844
234684	Lrrc29	leucine rich repeat containing 29, transcript variant X:	NA	-0.05728	-0.23043	-1.0405	0.86333866	0.949848
55992	Trim3	tripartite motif-containing 3, transcript variant 2	NA	0.008529	6.372037	1.005929	0.86347887	0.949874
229541	Dennd4b	DENN/MADD domain containing 4B, transcript variar	NA	-0.01212	4.909282	-1.00843	0.86347918	0.949874
66950	Pacc1	proton activated chloride channel 1	NA	-0.01128	5.021814	-1.00785	0.8635723	0.949912
56809	Gmeb1	glucocorticoid modulatory element binding protein 1,	NA	0.009403	5.975915	1.006539	0.86376264	0.950057
77532	Jrkl	Jrk-like	NA	0.014765	4.042265	1.010287	0.86387013	0.95009
53417	Hif3a	hypoxia inducible factor 3, alpha subunit, transcript va	NA	0.015362	4.361788	1.010705	0.86391779	0.95009
56515	Rnf138	ring finger protein 138, transcript variant 1	NA	0.012488	5.844515	1.008694	0.86396787	0.95009
20184	Uimc1	ubiquitin interaction motif containing 1, transcript vari	NA	-0.01174	4.745315	-1.00817	0.86404672	0.950113
230234	Abitram	actin binding transcription modulator	NA	-0.01243	4.680191	-1.00865	0.86429278	0.950319
105245463	Gm40912	predicted gene, 40912	NA	-0.06893	-0.26063	-1.04894	0.86457249	0.950537
17389	Mmp16	matrix metallopeptidase 16, transcript variant 1	NA	0.015374	3.889937	1.010713	0.86460758	0.950537
13004	Ncan	neurocan	NA	0.008205	8.865599	1.005703	0.86480803	0.950693
80915	Dusp12	dual specificity phosphatase 12, transcript variant 3	NA	0.014632	4.12393	1.010194	0.86498324	0.950821
102638347	Gm34934	predicted gene, 34934	NA	0.054029	0.02743	1.03816	0.86519984	0.950944
13495	Drg2	developmentally regulated GTP binding protein 2	NA	0.01018	5.655859	1.007081	0.86521205	0.950944
70355	Gprc5c	G protein-coupled receptor, family C, group 5, memb	NA	-0.02867	2.888939	-1.02007	0.86533812	0.950976
66362	Exosc3	exosome component 3, transcript variant 1	NA	0.01801	3.649429	1.012562	0.86545896	0.950976
66899	Fip1l1	FIP1 like 1 (S. cerevisiae), transcript variant 1	NA	-0.00785	6.808874	-1.00546	0.86546602	0.950976
102635069	Gm32498	predicted gene, 32498, transcript variant 2	NA	-0.0361	1.056728	-1.02534	0.8654747	0.950976
66585	Snrnp40	small nuclear ribonucleoprotein 40 (U5)	NA	0.010185	5.802102	1.007085	0.86564008	0.951094
20689	Sall3	spalt like transcription factor 3, transcript variant 2	NA	-0.01349	5.089401	-1.0094	0.86580642	0.951212
78103	8430431K14Rik	RIKEN cDNA 8430431K14 gene	NA	-0.04523	0.185337	-1.03185	0.8658991	0.951224
71409	Fmnl2	formin-like 2, transcript variant X4	NA	0.009137	6.973293	1.006353	0.86601753	0.951224
68328	Rab13	RAB13, member RAS oncogene family, transcript va	NA	0.016736	3.769933	1.011668	0.86605721	0.951224
14874	Gstz1	glutathione transferase zeta 1 (maleylacetoacetate is	NA	0.01454	4.912467	1.010129	0.86609394	0.951224
207785	Csrnp2	cysteine-serine-rich nuclear protein 2	NA	0.008985	6.11523	1.006248	0.86610968	0.951224
11629	Aif1	allograft inflammatory factor 1, transcript variant 3	NA	-0.04884	0.116175	-1.03443	0.86629215	0.95136
70530	Lrfn2	leucine rich repeat and fibronectin type III domain cor	NA	0.014147	4.065472	1.009854	0.86645805	0.951478
217734	Pomt2	protein-O-mannosyltransferase 2	NA	-0.01348	4.796308	-1.00939	0.86677164	0.951758
94061	Mrpl1	mitochondrial ribosomal protein L1, transcript variant	NA	-0.01481	4.382942	-1.01032	0.86715259	0.952112
67746	4930577N17Rik	RIKEN cDNA 4930577N17 gene	NA	0.053252	-0.17858	1.037601	0.86733757	0.952251
235047	Zfp809	zinc finger protein 809, transcript variant X3	NA	-0.0247	2.121977	-1.01727	0.86740406	0.95226

214932	Hdhd5	haloacid dehalogenase like hydrolase domain contair	NA	0.014335	4.162562	1.009986	0.86756712	0.952333
70238	Rnf168	ring finger protein 168	NA	-0.00998	5.570716	-1.00694	0.86760186	0.952333
115486239	Gm51436	predicted gene, 51436	NA	-0.05	0.015991	-1.03526	0.86764599	0.952333
320938	Tnpo3	transportin 3, transcript variant X2	NA	0.007595	6.913732	1.005279	0.86794545	0.952597
219072	Haus4	HAUS augmin-like complex, subunit 4	NA	-0.02319	3.304067	-1.0162	0.86804542	0.952643
108937	Rnf169	ring finger protein 169	NA	-0.0098	5.38084	-1.00682	0.86813276	0.952674
170788	Crb1	crumbs family member 1, photoreceptor morphogene	NA	0.035573	2.307494	1.024964	0.86828004	0.952772
243912	Hspb6	heat shock protein, alpha-crystallin-related, B6, trans	NA	0.020576	2.558185	1.014364	0.8684761	0.95291
115488669	LOC115488669	uncharacterized LOC115488669	NA	0.031264	1.727784	1.021907	0.86852296	0.95291
94176	Dock2	dedicator of cyto-kinesis 2	NA	-0.04218	0.800882	-1.02967	0.86859988	0.952911
110911	Cds2	CDP-diacylglycerol synthase (phosphatidate cytidylyl	NA	-0.00816	7.596952	-1.00567	0.86864127	0.952911
16898	Rps2	ribosomal protein S2	NA	-0.00948	9.321457	-1.00659	0.86894864	0.953176
236643	Sytl5	synaptotagmin-like 5, transcript variant X17	NA	-0.03059	1.707851	-1.02143	0.86899987	0.953176
74131	Sash3	SAM and SH3 domain containing 3, transcript variant	NA	-0.0613	-0.1056	-1.0434	0.86957507	0.95366
230103	Npr2	natriuretic peptide receptor 2, transcript variant X2	NA	-0.01073	5.544008	-1.00746	0.86958457	0.95366
13135	Dad1	defender against cell death 1, transcript variant 1	NA	0.011832	5.477784	1.008235	0.86968077	0.95366
18132	Notch4	notch 4	NA	-0.01727	3.659355	-1.01204	0.86968205	0.95366
102638888	Gm20346	predicted gene, 20346	NA	-0.03959	0.829933	-1.02782	0.86973404	0.95366
71586	Ifih1	interferon induced with helicase C domain 1, transcri	NA	-0.04009	0.833398	-1.02818	0.87024519	0.954008
333182	Cox6b2	cytochrome c oxidase subunit 6B2, transcript variant	NA	-0.03698	1.300146	-1.02597	0.87024893	0.954008
67912	1600012H06Rik	RIKEN cDNA 1600012H06 gene, transcript variant 2	NA	0.011745	4.608804	1.008174	0.8703215	0.954008
115487189	9430062P05Rik	RIKEN cDNA 9430062P05 gene	NA	0.027509	1.642895	1.019251	0.87037165	0.954008
21408	Zfp354a	zinc finger protein 354A, transcript variant X3	NA	-0.01548	4.012282	-1.01078	0.87040201	0.954008
414115	D330050I16Rik	RIKEN cDNA D330050I16 gene	NA	0.036328	0.901729	1.0255	0.87040328	0.954008
545207	E130008D07Rik	RIKEN cDNA E130008D07 gene, transcript variant 2	NA	0.015128	4.336293	1.010541	0.8704845	0.954033
50926	Hnrnpdl	heterogeneous nuclear ribonucleoprotein D-like, tran	NA	0.008768	8.805927	1.006096	0.87080668	0.954247
16511	Kcnh2	potassium voltage-gated channel, subfamily H (eag-r	NA	0.014993	4.929264	1.010446	0.87091319	0.954247
75556	Cfap161	cilia and flagella associated protein 161, transcript va	NA	-0.06885	-0.22506	-1.04888	0.87101277	0.954247
77805	Esco1	establishment of sister chromatid cohesion N-acetyltr	NA	0.013177	4.489825	1.009176	0.87102891	0.954247
78473	Skap1	src family associated phosphoprotein 1, transcript va	NA	-0.05411	0.700228	-1.03822	0.87119815	0.954247
18121	Nog	noggin	NA	-0.01932	3.23681	-1.01348	0.87125939	0.954247
108168481	Gm50594	predicted gene, 50594, transcript variant X2	NA	0.022775	3.311186	1.015911	0.87126964	0.954247
57905	Isy1	ISY1 splicing factor homolog	NA	-0.01177	4.46395	-1.00819	0.87132418	0.954247
24050	Septin3	septin 3, transcript variant 3	NA	0.007015	9.630791	1.004874	0.87138758	0.954247
225997	Trpm6	transient receptor potential cation channel, subfamily	NA	-0.05259	0.0068	-1.03713	0.87140342	0.954247
14048	Eya1	EYA transcriptional coactivator and phosphatase 1, t	NA	0.02291	3.10765	1.016007	0.87141439	0.954247
72776	Sass6	SAS-6 centriolar assembly protein, transcript variant	NA	-0.01476	3.947553	-1.01029	0.87142033	0.954247
75939	4930579G24Rik	RIKEN cDNA 4930579G24 gene	NA	0.02892	2.137817	1.020248	0.87159837	0.954247
12587	Mia	melanoma inhibitory activity	NA	0.038732	1.200851	1.027211	0.87161997	0.954247
19326	Rab11b	RAB11B, member RAS oncogene family, transcript v	NA	-0.00711	8.141358	-1.00494	0.8716387	0.954247
319960	4930513N10Rik	RIKEN cDNA 4930513N10 gene	NA	-0.04414	0.589363	-1.03107	0.87166045	0.954247
18596	Pdgfrb	platelet derived growth factor receptor, beta polypepti	NA	-0.01073	5.299053	-1.00747	0.87173107	0.954247
100036568	Gm3373	predicted gene 3373, transcript variant X8	NA	0.062	-0.79975	1.043912	0.87180647	0.954247
118567375	LOC118567375	uncharacterized LOC118567375	NA	0.0555	0.806502	1.03922	0.87186388	0.954247
103236	Csnk1g2	casein kinase 1, gamma 2, transcript variant 2	NA	0.008343	6.635447	1.0058	0.87187082	0.954247
434778	Ccdc160	coiled-coil domain containing 160	NA	-0.0292	2.252861	-1.02045	0.87194235	0.954247
208718	Dis3l2	DIS3 like 3'-5' exoribonuclease 2, transcript variant 1	NA	0.011421	5.233715	1.007948	0.87196989	0.954247
102636879	Gm33829	predicted gene, 33829	NA	0.043766	0.32627	1.030801	0.87212276	0.954331
68691	Kansl1l	KAT8 regulatory NSL complex subunit 1-like, transcri	NA	0.013776	4.93664	1.009595	0.8723828	0.954331
227154	Stradb	STE20-related kinase adaptor beta, transcript variant	NA	0.010725	6.033369	1.007462	0.87240097	0.954331
54369	Nme6	NME/NM23 nucleoside diphosphate kinase 6, transcr	NA	-0.0159	3.448883	-1.01108	0.87248572	0.954331
84113	Ptov1	prostate tumor over expressed gene 1	NA	-0.00979	7.898826	-1.00681	0.87249892	0.954331
12702	Socs3	suppressor of cytokine signaling 3	NA	-0.01834	3.279171	-1.01279	0.87251077	0.954331
105242888	Gm38973	predicted gene, 38973, transcript variant X2	NA	0.062909	-0.10177	1.04457	0.8725224	0.954331
79263	Trim39	tripartite motif-containing 39, transcript variant 1	NA	-0.00954	5.47653	-1.00663	0.87256959	0.954331
81004	Tbl1xr1	transducin (beta)-like 1X-linked receptor 1, transcript	NA	-0.0091	6.274576	-1.00633	0.87257503	0.954331
213573	Cracr2b	calcium release activated channel regulator 2B	NA	0.037386	0.782513	1.026252	0.8727629	0.954473
102632415	LOC102632415	uncharacterized LOC102632415, transcript variant X	NA	0.065817	-0.30814	1.046677	0.87299171	0.954659
214572	Prmt7	protein arginine N-methyltransferase 7	NA	0.00985	5.347663	1.006851	0.87312075	0.954736
76628	1700112J16Rik	RIKEN cDNA 1700112J16 gene, transcript variant 1	NA	0.060679	0.137294	1.042957	0.87339698	0.954974
13197	Gadd45a	growth arrest and DNA-damage-inducible 45 alpha	NA	-0.02339	2.310962	-1.01634	0.87353477	0.955027
118567827	LOC118567827	uncharacterized LOC118567827	NA	-0.03795	0.763421	-1.02666	0.8735646	0.955027
102638333	Gm34923	predicted gene, 34923	NA	-0.04463	0.224059	-1.03142	0.87362201	0.955027
89867	Sec16b	SEC16 homolog B (S. cerevisiae), transcript variant 2	NA	-0.04542	0.617953	-1.03198	0.87376329	0.95511
329078	Emx2os	Emx2 opposite strand/antisense transcript (non-prote	NA	0.021103	2.468568	1.014735	0.8738206	0.95511
100040529	Gm2824	predicted gene 2824, transcript variant X12	NA	0.01904	3.224146	1.013285	0.8738818	0.95511
14362	Fzd1	frizzled class receptor 1	NA	-0.00886	5.87627	-1.00616	0.87393238	0.95511
18504	Pax2	paired box 2, transcript variant 7	NA	-0.02876	3.528183	-1.02013	0.87428965	0.95529

71768	Vwce	von Willebrand factor C and EGF domains, transcript	NA	0.032899	1.342304	1.023066	0.87440132	0.95529
66199	Commd4	COMM domain containing 4, transcript variant X1	NA	0.012699	4.751025	1.008841	0.87444905	0.95529
11773	Ap2m1	adaptor-related protein complex 2, mu 1 subunit, tran	NA	-0.00691	9.115084	-1.0048	0.87450912	0.95529
13424	Dync1h1	dynein cytoplasmic 1 heavy chain 1	NA	0.00824	9.059368	1.005728	0.87464566	0.95529
264134	Ttc26	tetratricopeptide repeat domain 26	NA	-0.01269	4.043389	-1.00884	0.87465934	0.95529
101685	Spty2d1	SPT2 chromatin protein domain containing 1	NA	-0.01764	3.910281	-1.0123	0.87470461	0.95529
73737	1110008P14Rik	RIKEN cDNA 1110008P14 gene	NA	-0.02101	3.694581	-1.01467	0.87471481	0.95529
209027	Pycr1	pyrroline-5-carboxylate reductase 1, transcript varian	NA	0.017053	3.787465	1.011891	0.87473905	0.95529
19293	Pvalb	parvalbumin, transcript variant 1	NA	0.058864	-0.1838	1.041645	0.87476434	0.95529
67184	Ndufa13	NADH:ubiquinone oxidoreductase subunit A13, trans	NA	-0.0146	5.56324	-1.01017	0.87479188	0.95529
51796	Srrm1	serine/arginine repetitive matrix 1, transcript variant 9	NA	0.007264	7.672298	1.005048	0.87480622	0.95529
239559	A4galt	alpha 1,4-galactosyltransferase, transcript variant X2	NA	0.028023	1.630671	1.019614	0.8749031	0.95529
97387	Strn4	striatin, calmodulin binding protein 4, transcript variar	NA	-0.00778	7.717201	-1.00541	0.87491964	0.95529
64296	Abhd8	abhydrolase domain containing 8	NA	0.008582	6.2355	1.005967	0.87513895	0.955395
52897	Rbfox3	RNA binding protein, fox-1 homolog (C. elegans) 3, tr	NA	-0.00842	6.364324	-1.00585	0.87519591	0.955395
54324	Arhgef5	Rho guanine nucleotide exchange factor (GEF) 5, tra	NA	-0.02083	2.798328	-1.01454	0.87521595	0.955395
105193	Nhlrc1	NHL repeat containing 1	NA	-0.01662	3.618205	-1.01158	0.87527898	0.955395
72085	Osgepl1	O-sialoglycoprotein endopeptidase-like 1, transcript v	NA	0.018044	3.357288	1.012586	0.87530888	0.955395
320973	D330023K18Rik	RIKEN cDNA D330023K18 gene	NA	-0.03408	1.101148	-1.0239	0.87564268	0.955508
73998	Herc3	hect domain and RLD 3, transcript variant X11	NA	-0.00855	5.62725	-1.00594	0.87575623	0.955508
11783	Apaf1	apoptotic peptidase activating factor 1, transcript vari	NA	0.00915	5.587907	1.006363	0.87592527	0.955508
12175	Bnip2	BCL2/adenovirus E1B interacting protein 2, transcript	NA	-0.00907	6.239448	-1.00631	0.87596962	0.955508
19027	Sypl	synaptophysin-like protein, transcript variant 2	NA	-0.01281	5.305706	-1.00892	0.87597207	0.955508
67826	Snap47	synaptosomal-associated protein, 47, transcript varia	NA	0.007463	7.720489	1.005186	0.87601484	0.955508
233189	Ctu1	cytosolic thiouridylase subunit 1	NA	0.010756	4.644242	1.007483	0.87602368	0.955508
11740	Slc25a5	solute carrier family 25 (mitochondrial carrier, adenin	NA	0.007652	7.680954	1.005318	0.87606563	0.955508
20264	Scn10a	sodium channel, voltage-gated, type X, alpha, transc	NA	0.04716	0.210465	1.033229	0.87610629	0.955508
56747	Sez6l	seizure related 6 homolog like, transcript variant 3	NA	-0.00803	7.815234	-1.00558	0.87615152	0.955508
225523	Cep120	centrosomal protein 120	NA	0.007567	6.369485	1.005259	0.87621091	0.955508
207318	Gm4750	predicted gene 4750	NA	-0.0377	0.490676	-1.02647	0.87621504	0.955508
240697	Mcmdc2	minichromosome maintenance domain containing 2	NA	0.037996	0.584986	1.026686	0.87621741	0.955508
52705	Krr1	KRR1, small subunit (SSU) processome component,	NA	0.008786	5.722227	1.006108	0.87628159	0.955508
22758	Zscan12	zinc finger and SCAN domain containing 12	NA	0.017619	4.094789	1.012288	0.87630081	0.955508
626848	Zfp971	zinc finger protein 971, transcript variant 1	NA	0.022603	2.698067	1.015791	0.87640602	0.955508
104130	Ndufb11	NADH:ubiquinone oxidoreductase subunit B11, trans	NA	0.009041	5.866259	1.006286	0.87641126	0.955508
217217	Asb16	ankyrin repeat and SOCS box-containing 16	NA	0.046152	0.279115	1.032508	0.87677767	0.955844
20476	Six6	sine oculis-related homeobox 6	NA	-0.03125	1.326786	-1.0219	0.87685551	0.955865
240041	Zfp945	zinc finger protein 945, transcript variant 1	NA	-0.01283	4.770215	-1.00894	0.87708979	0.955921
68055	Dmac2l	distal membrane arm assembly complex 2 like, trans	NA	0.01495	3.491517	1.010417	0.87711582	0.955921
21872	Tjp1	tight junction protein 1, transcript variant 2	NA	0.007934	6.457666	1.005515	0.87711702	0.955921
57423	Atp5j2	ATP synthase, H+ transporting, mitochondrial F0 cor	NA	-0.0081	6.448202	-1.00563	0.87714874	0.955921
100039815	Gm2436	predicted gene 2436	NA	-0.01772	2.819124	-1.01236	0.87725724	0.955921
66162	Bola2	bolA-like 2 (E. coli)	NA	0.018012	3.135341	1.012564	0.87726	0.955921
20375	Spi1	spleen focus forming virus (SFFV) proviral integratior	NA	0.041766	0.6849	1.029373	0.8773694	0.955962
244281	Myo16	myosin XVI	NA	-0.00839	6.062577	-1.00583	0.87743553	0.955962
67338	Rffl	ring finger and FYVE like domain containing protein,	NA	-0.01692	3.127792	-1.0118	0.87747375	0.955962
108168930	Gm46845	predicted gene, 46845	NA	0.025367	1.934696	1.017739	0.8776472	0.956061
620419	Zfp963	zinc finger protein 963	NA	0.015069	3.775331	1.0105	0.87771959	0.956061
241547	Harbi1	harbinger transposase derived 1, transcript variant 1	NA	-0.01442	3.715491	-1.01005	0.87774045	0.956061
99382	Abtb2	ankyrin repeat and BTB (POZ) domain containing 2	NA	-0.01628	3.55671	-1.01135	0.87781971	0.956083
233979	Tpcn2	two pore segment channel 2, transcript variant 1	NA	-0.02594	2.357512	-1.01814	0.87819221	0.956364
56706	Ccnl1	cyclin L1, transcript variant 1	NA	0.007971	5.972361	1.005541	0.8781954	0.956364
22415	Wnt3	wingless-type MMTV integration site family, member	NA	-0.01451	3.476189	-1.01011	0.87830945	0.956382
11656	Alas2	aminolevulinic acid synthase 2, erythroid, transcript v	NA	0.042514	5.767912	1.029907	0.87832973	0.956382
110524	Dgkq	diacylglycerol kinase, theta, transcript variant 2	NA	-0.00955	5.486816	-1.00664	0.87847256	0.956382
53356	Eif3g	eukaryotic translation initiation factor 3, subunit G	NA	-0.00823	6.393932	-1.00572	0.8784793	0.956382
18610	Pdyn	prodynorphin, transcript variant 1	NA	0.019844	3.133195	1.01385	0.87850553	0.956382
194388	Tet3	tet methylcytosine dioxygenase 3, transcript variant 2	NA	0.008212	7.310495	1.005708	0.87898835	0.956763
225861	Snx32	sorting nexin 32, transcript variant 1	NA	-0.01028	5.706705	-1.00715	0.8790281	0.956763
27274	Zfp354b	zinc finger protein 354B	NA	-0.01988	2.407219	-1.01388	0.87903215	0.956763
16785	Rpsa	ribosomal protein SA	NA	0.006479	9.847979	1.004501	0.87919062	0.956795
50995	Uba2	ubiquitin-like modifier activating enzyme 2	NA	-0.00716	7.227781	-1.00498	0.87922258	0.956795
105242636	Gm38795	predicted gene, 38795	NA	-0.02627	1.929453	-1.01838	0.87926519	0.956795
54200	Sult2b1	sulfotransferase family, cytosolic, 2B, member 1, tran	NA	-0.05674	-0.22497	-1.04011	0.87932555	0.956795
210094	Iglon5	IgLON family member 5	NA	-0.00768	7.014758	-1.00534	0.87935523	0.956795
75141	Rasd2	RASD family, member 2	NA	-0.02776	2.47463	-1.01943	0.87947355	0.95686
78796	Zcchc4	zinc finger, CCHC domain containing 4, transcript var	NA	0.014555	4.008012	1.01014	0.87967768	0.956934
654795	Sdr39u1	short chain dehydrogenase/reductase family 39U, me	NA	0.009904	5.346823	1.006889	0.87970194	0.956934

407812	Zfp941	zinc finger protein 941	NA	0.012255	4.455506	1.008531	0.87976741	0.956934
94092	Trim16	tripartite motif-containing 16	NA	-0.02079	2.564254	-1.01451	0.87977733	0.956934
19347	Dennd5a	DENN/MADD domain containing 5A, transcript variar	NA	-0.00739	7.383703	-1.00514	0.87985634	0.956949
625591	Cldn34c2	claudin 34C2	NA	-0.14766	-1.10702	-1.10777	0.87995799	0.956949
70568	Cpne3	copine III, transcript variant 1	NA	0.012724	4.621014	1.008859	0.87996763	0.956949
12426	Cckbr	cholecystokinin B receptor	NA	0.028313	1.604487	1.019819	0.8802293	0.95717
27886	Ess2	ess-2 splicing factor, transcript variant 1	NA	0.009639	5.433873	1.006704	0.88033875	0.957172
229694	AI504432	expressed sequence AI504432	NA	-0.01317	5.705341	-1.00917	0.88037423	0.957172
228858	Gdap1l1	ganglioside-induced differentiation-associated protei	NA	0.007266	6.764184	1.005049	0.88041134	0.957172
12925	Crip1	cysteine-rich protein 1 (intestinal)	NA	-0.03286	1.399025	-1.02304	0.8804664	0.957172
210762	Ppp1r36	protein phosphatase 1, regulatory subunit 36	NA	-0.05002	-0.0499	-1.03528	0.88078235	0.957451
22259	Nr1h3	nuclear receptor subfamily 1, group H, member 3, tra	NA	0.045313	-0.0117	1.031907	0.88098515	0.957608
67212	Mrpl55	mitochondrial ribosomal protein L55, transcript varian	NA	-0.01463	3.87951	-1.01019	0.88108808	0.957608
67812	Ubxn4	UBX domain protein 4, transcript variant 1	NA	0.007032	6.750923	1.004886	0.88122612	0.957608
14912	Nkx6-2	NK6 homeobox 2, transcript variant 2	NA	0.038444	0.804863	1.027005	0.88123527	0.957608
70470	Rprd1b	regulation of nuclear pre-mRNA domain containing 1I	NA	0.009652	5.426775	1.006713	0.88130427	0.957608
12931	Crlf1	cytokine receptor-like factor 1, transcript variant 2	NA	0.027864	2.099903	1.019501	0.88142163	0.957608
16777	Lamb1	laminin B1, transcript variant X1	NA	0.008513	5.939055	1.005918	0.88151522	0.957608
11568	Aebp1	AE binding protein 1, transcript variant 2	NA	-0.01364	4.730903	-1.0095	0.88161709	0.957608
24030	Mrps12	mitochondrial ribosomal protein S12, transcript variar	NA	0.013528	3.816824	1.009421	0.88163909	0.957608
105244627	Gm40208	predicted gene, 40208	NA	0.069925	-0.21713	1.049662	0.88166439	0.957608
80721	Slc19a3	solute carrier family 19, member 3, transcript variant	NA	-0.03551	0.604413	-1.02492	0.88166477	0.957608
622208	Gm6297	predicted gene 6297, transcript variant 2	NA	0.030094	1.431597	1.021079	0.88169076	0.957608
13032	Ctsc	cathepsin C, transcript variant X2	NA	-0.01582	3.528185	-1.01103	0.88171267	0.957608
20850	Stat5a	signal transducer and activator of transcription 5A, tra	NA	-0.03553	1.322988	-1.02493	0.88175003	0.957608
215821	Arfgef3	ARFGEF family member 3	NA	0.022451	3.349548	1.015683	0.88199129	0.957744
403178	Plcxd1	phosphatidylinositol-specific phospholipase C, X dom	NA	-0.02487	2.025097	-1.01739	0.88202112	0.957744
105246804	D830024N08Rik	RIKEN cDNA D830024N08 gene, transcript variant X	NA	0.040771	0.141008	1.028663	0.88205214	0.957744
100034684	Cstdc5	cystatin domain containing 5	NA	0.105588	-0.47344	1.075933	0.8821408	0.957774
11472	Actn2	actinin alpha 2	NA	-0.01955	3.488087	-1.01364	0.88220241	0.957774
11806	Apoa1	apolipoprotein A-I	NA	0.054956	-0.02535	1.038827	0.88225639	0.957774
232157	Mob1a	MOB kinase activator 1A, transcript variant X1	NA	0.01023	4.896184	1.007116	0.88233277	0.957793
80861	Dhx58	DEXH (Asp-Glu-X-His) box polypeptide 58, transcript	NA	-0.0329	0.693467	-1.02307	0.88257857	0.957965
108167775	Gm8112	predicted gene 8112	NA	-0.05026	-0.14639	-1.03545	0.88265992	0.957965
100310809	Gm10509	predicted gene 10509	NA	-0.01428	3.689161	-1.00995	0.88266777	0.957965
216439	Agap2	ArfGAP with GTPase domain, ankyrin repeat and PH	NA	0.007218	8.478327	1.005016	0.88294276	0.9582
22419	Wnt5b	wingless-type MMTV integration site family, member	NA	-0.027	1.786463	-1.01889	0.88334482	0.958572
66659	Acp6	acid phosphatase 6, lysophosphatidic	NA	-0.02024	2.868995	-1.01413	0.88365126	0.958766
67326	1700037H04Rik	RIKEN cDNA 1700037H04 gene, transcript variant X	NA	-0.01065	4.528439	-1.00741	0.88382245	0.958766
56489	Ikbke	inhibitor of kappaB kinase epsilon	NA	0.036283	0.942276	1.025469	0.88385229	0.958766
70082	Lysmd2	LysM, putative peptidoglycan-binding, domain contai	NA	0.014932	3.651014	1.010404	0.88388345	0.958766
107250	Kazald1	Kazal-type serine peptidase inhibitor domain 1, trans	NA	-0.04123	0.137628	-1.02899	0.88389191	0.958766
66365	Ccdc90b	coiled-coil domain containing 90B, transcript variant	NA	-0.01096	4.982253	-1.00763	0.88392122	0.958766
76357	Trmt5	TRM5 tRNA methyltransferase 5	NA	0.01555	3.578674	1.010837	0.88393606	0.958766
69024	Snx15	sorting nexin 15, transcript variant X1	NA	0.010912	4.632441	1.007592	0.88402217	0.958795
12648	Chd1	chromodomain helicase DNA binding protein 1	NA	-0.00871	5.524481	-1.00605	0.88410104	0.958817
75991	Slain2	SLAIN motif family, member 2, transcript variant X1	NA	0.008682	6.22367	1.006036	0.88427386	0.95888
207911	Mchr1	melanin-concentrating hormone receptor 1	NA	0.029899	1.514003	1.020941	0.88427689	0.95888
240444	Kcng2	potassium voltage-gated channel, subfamily G, meml	NA	-0.05213	0.017336	-1.03679	0.88453136	0.958994
72170	Chchd4	coiled-coil-helix-coiled-coil-helix domain containing 4	NA	0.010005	4.724874	1.006959	0.88454827	0.958994
269717	Orai2	ORAI calcium release-activated calcium modulator 2	NA	-0.00803	6.161739	-1.00558	0.88455919	0.958994
115488816	Gm52313	predicted gene, 52313	NA	-0.05223	0.221247	-1.03687	0.8846924	0.959041
245666	Iqsec2	IQ motif and Sec7 domain 2, transcript variant 2	NA	-0.01007	5.197141	-1.007	0.88471984	0.959041
242202	Pde5a	phosphodiesterase 5A, cGMP-specific	NA	0.012811	4.301634	1.00892	0.88509643	0.959323
75956	Srrm2	serine/arginine repetitive matrix 2, transcript variant 2	NA	-0.00808	9.240805	-1.00561	0.88512253	0.959323
243407	Qrfprl	pyroglutamylated RFamide peptide receptor like	NA	-0.04783	0.021869	-1.03371	0.88521213	0.959323
100503636	1700123M08Rik	RIKEN cDNA 1700123M08 gene	NA	0.03733	0.50099	1.026213	0.88527334	0.959323
12583	Cdo1	cysteine dioxygenase 1, cytosolic, transcript variant 1	NA	-0.00808	6.041228	-1.00561	0.88540347	0.959323
107528	Magee1	MAGE family member E1	NA	0.007472	7.576414	1.005192	0.88541474	0.959323
20112	Rps6ka2	ribosomal protein S6 kinase, polypeptide 2	NA	0.009149	5.725627	1.006361	0.88556489	0.959323
208666	Diras1	DIRAS family, GTP-binding RAS-like 1	NA	0.009543	6.354115	1.006637	0.88559785	0.959323
100900	Hscb	HscB iron-sulfur cluster co-chaperone, transcript vari	NA	-0.0233	1.81786	-1.01628	0.88560124	0.959323
110084	Dnah1	dynein, axonemal, heavy chain 1	NA	-0.01532	3.04025	-1.01067	0.88561136	0.959323
115489006	Gm52381	predicted gene, 52381	NA	0.047699	-0.23408	1.033615	0.88565567	0.959323
67588	Rnf41	ring finger protein 41, transcript variant 1	NA	-0.00741	6.483785	-1.00515	0.88568765	0.959323
80751	Rnf34	ring finger protein 34	NA	-0.00787	5.6761	-1.00547	0.88590256	0.959479
78408	Fam131a	family with sequence similarity 131, member A	NA	-0.0097	5.363747	-1.00674	0.88594995	0.959479
56485	Slc2a5	solute carrier family 2 (facilitated glucose transporter)	NA	0.04367	0.503694	1.030732	0.88606614	0.959482

215031	Vgll2	vestigial like family member 2, transcript variant 2	NA	-0.03619	0.677381	-1.0254	0.88607053	0.959482
21916	Tmod1	tropomodulin 1	NA	0.036364	1.382089	1.025526	0.88618163	0.959539
70503	Ddo	D-aspartate oxidase, transcript variant 1	NA	0.032383	0.742884	1.0227	0.88638329	0.959619
75259	4930556M19Rik	RIKEN cDNA 4930556M19 gene, transcript variant 3	NA	-0.05576	-0.27308	-1.03941	0.88640339	0.959619
70802	Pwwp2a	PWWP domain containing 2A, transcript variant 4	NA	0.007363	5.926639	1.005117	0.8864324	0.959619
14201	Fhl3	four and a half LIM domains 3	NA	-0.02211	2.143265	-1.01545	0.88666339	0.959767
74732	Stx11	syntaxin 11, transcript variant 3	NA	-0.04501	0.812895	-1.03169	0.88668767	0.959767
110821	Pcca	propionyl-Coenzyme A carboxylase, alpha polypeptid	NA	-0.01075	4.446126	-1.00748	0.88683282	0.959861
27280	Phlda3	pleckstrin homology like domain, family A, member 3	NA	0.018056	2.899669	1.012594	0.88697293	0.959948
67564	Tmem35a	transmembrane protein 35A	NA	-0.00698	6.886532	-1.00485	0.88712347	0.960047
77604	Rbm12b2	RNA binding motif protein 12 B2, transcript variant 2	NA	-0.0089	5.618506	-1.00619	0.88722778	0.960096
69276	Sec62	SEC62 homolog (S. cerevisiae), transcript variant 2	NA	-0.00684	7.211592	-1.00475	0.88753957	0.960299
210105	Zfp719	zinc finger protein 719, transcript variant X1	NA	-0.01131	4.386544	-1.00787	0.887554	0.960299
19334	Rab22a	RAB22A, member RAS oncogene family	NA	0.012663	4.755777	1.008816	0.88759178	0.960299
56418	Ykt6	YKT6 v-SNARE homolog (S. cerevisiae)	NA	0.006477	7.56471	1.004499	0.88785083	0.960502
66897	Naa16	N(alpha)-acetyltransferase 16, NatA auxiliary subunit	NA	0.012874	3.71202	1.008963	0.88796756	0.960502
11538	Adnp	activity-dependent neuroprotective protein, transcript	NA	0.00694	7.260609	1.004822	0.88798651	0.960502
67196	Ube2t	ubiquitin-conjugating enzyme E2T, transcript variant	NA	0.020658	2.275492	1.014422	0.88808811	0.960502
72590	Ppme1	protein phosphatase methylesterase 1	NA	0.009151	6.882211	1.006363	0.88812418	0.960502
245865	Spag4	sperm associated antigen 4, transcript variant X1	NA	0.029664	1.362195	1.020775	0.88813365	0.960502
14247	Fli1	Friend leukemia integration 1	NA	-0.01314	3.519458	-1.00915	0.88820191	0.960511
381269	Mreg	melanoregulin, transcript variant 1	NA	-0.03084	1.046693	-1.02161	0.88826053	0.960511
18858	Pmp22	peripheral myelin protein 22, transcript variant 7	NA	0.011919	4.502477	1.008295	0.88845741	0.96066
72982	Tmem138	transmembrane protein 138, transcript variant X1	NA	-0.01271	3.986588	-1.00885	0.88854105	0.960687
84682	Cox4i2	cytochrome c oxidase subunit 4I2	NA	0.038087	0.425558	1.026751	0.88901105	0.960951
66074	Tmem167	transmembrane protein 167	NA	0.008576	6.635469	1.005962	0.88903916	0.960951
93891	Pcdhb20	protocadherin beta 20	NA	0.012267	3.988812	1.008539	0.8890739	0.960951
16513	Kcnj10	potassium inwardly-rectifying channel, subfamily J, m	NA	0.009854	4.571272	1.006854	0.88916768	0.960951
12722	Clca3a1	chloride channel accessory 3A1, transcript variant 2	NA	-0.03486	0.902376	-1.02446	0.88923167	0.960951
21386	Tbx3	T-box 3, transcript variant 1	NA	0.019435	2.615711	1.013562	0.88923254	0.960951
20851	Stat5b	signal transducer and activator of transcription 5B, tra	NA	-0.00951	4.770912	-1.00661	0.8892642	0.960951
69942	Rnf113a1	ring finger protein 113A1	NA	0.019036	2.589715	1.013282	0.88931881	0.960951
115487119	6030442K20Rik	RIKEN cDNA 6030442K20 gene	NA	-0.05289	0.001403	-1.03734	0.88943987	0.960951
71729	Rgs12	regulator of G-protein signaling 12, transcript variant	NA	0.007892	6.480867	1.005485	0.88946943	0.960951
327824	5330438D12Rik	RIKEN cDNA 5330438D12 gene	NA	-0.02385	1.950716	-1.01667	0.88948808	0.960951
107650	Pi4kb	phosphatidylinositol 4-kinase beta, transcript variant	NA	0.008951	5.789955	1.006223	0.88949411	0.960951
225845	Plaat3	phospholipase A and acyltransferase 3, transcript var	NA	-0.02154	2.030121	-1.01504	0.88966421	0.961022
57330	Gigyf1	GRB10 interacting GYF protein 1	NA	0.007089	6.854793	1.004926	0.88969927	0.961022
22784	Slc30a3	solute carrier family 30 (zinc transporter), member 3,	NA	0.018338	2.882489	1.012792	0.88973709	0.961022
213053	Slc39a14	solute carrier family 39 (zinc transporter), member 14	NA	-0.01064	4.363988	-1.0074	0.88981143	0.961039
110599584	Eef1akmt4-ece2	Eef1akmt4-Ece2 readthrough, transcript variant 2	NA	0.118872	-1.38067	1.085885	0.88990823	0.961053
16882	Lig3	ligase III, DNA, ATP-dependent, transcript variant 2	NA	0.007143	6.113095	1.004963	0.88994241	0.961053
242409	Tmem8b	transmembrane protein 8B, transcript variant X6	NA	0.007809	6.130542	1.005428	0.89010276	0.961092
13363	Dhh	desert hedgehog	NA	0.048326	0.0023	1.034064	0.89020468	0.961092
216292	Mettl25	methyltransferase like 25, transcript variant X21	NA	-0.0157	3.396113	-1.01094	0.89026425	0.961092
20502	Slc16a2	solute carrier family 16 (monocarboxylic acid transpo	NA	-0.00769	6.409386	-1.00534	0.89030147	0.961092
72665	2810039B14Rik	RIKEN cDNA 2810039B14 gene	NA	0.031424	1.329777	1.02202	0.89033206	0.961092
72844	Kctd17	potassium channel tetramerisation domain containing	NA	-0.00698	6.858001	-1.00485	0.89034536	0.961092
171180	Syt12	synaptotagmin XII	NA	-0.02446	1.973864	-1.0171	0.89042346	0.961092
22632	Yy1	YY1 transcription factor	NA	0.008343	6.804572	1.0058	0.89045111	0.961092
12142	Prdm1	PR domain containing 1, with ZNF domain, transcript	NA	0.03523	0.546419	1.02472	0.89055167	0.961137
70316	Ndufab1	NADH:ubiquinone oxidoreductase subunit AB1, trans	NA	0.00922	5.669948	1.006411	0.89079197	0.961312
11938	Atp2a2	ATPase, Ca++ transporting, cardiac muscle, slow twi	NA	-0.00701	8.36658	-1.00487	0.89088321	0.961312
66404	Rtf2	replication termination factor 2	NA	-0.00958	6.09741	-1.00666	0.89089663	0.961312
14154	Fem1a	fem 1 homolog a	NA	-0.00886	6.101604	-1.00616	0.89095043	0.961312
15039	H2-T22	histocompatibility 2, T region locus 22, transcript varia	NA	-0.0201	2.711845	-1.01403	0.89117081	0.961486
244058	Rgma	repulsive guidance molecule family member A	NA	-0.00664	7.242205	-1.00462	0.8913055	0.961568
320429	Trank1	tetratricopeptide repeat and ankyrin repeat containing	NA	-0.00931	6.743216	-1.00648	0.89137824	0.961582
14300	Frg1	FSHD region gene 1	NA	-0.0092	5.25585	-1.0064	0.89174614	0.961829
68263	Pdhb	pyruvate dehydrogenase (lipoamide) beta	NA	-0.00701	6.81712	-1.00487	0.8917587	0.961829
75284	Bcdin3d	BCDIN3 domain containing	NA	0.017953	3.064313	1.012522	0.89188357	0.961829
23921	Sh2b2	SH2B adaptor protein 2, transcript variant X3	NA	0.008353	5.286473	1.005806	0.89188645	0.961829
19024	Ppfibp2	PTPRF interacting protein, binding protein 2 (liprin be	NA	0.019543	2.394852	1.013638	0.89190275	0.961829
69234	Zfp688	zinc finger protein 688	NA	0.016649	2.859003	1.011607	0.89206081	0.961912
26412	Map4k2	mitogen-activated protein kinase kinase kinase kinas	NA	-0.00835	5.735385	-1.0058	0.89217503	0.961912
22680	Zfp207	zinc finger protein 207, transcript variant 1	NA	0.006865	8.145747	1.00477	0.8922236	0.961912
71891	Cdadc1	cytidine and dCMP deaminase domain containing 1,	NA	-0.00862	5.181739	-1.00599	0.89223522	0.961912
105245152	A230054N11Rik	RIKEN cDNA A230054N11 gene	NA	-0.03779	0.557389	-1.02654	0.89227475	0.961912

59031	Chst12	carbohydrate sulfotransferase 12	NA	0.014076	3.75007	1.009805	0.89257949	0.962176
108168347	5830433I10Rik	RIKEN cDNA 5830433I10 gene	NA	0.037215	0.107308	1.026131	0.89270407	0.962176
17005	Ltk	leukocyte tyrosine kinase, transcript variant 1	NA	0.038647	0.777355	1.02715	0.89275557	0.962176
319776	Tmem72	transmembrane protein 72, transcript variant X1	NA	-0.02846	1.09084	-1.01992	0.89280645	0.962176
11981	Atp9a	ATPase, class II, type 9A, transcript variant 1	NA	0.006484	8.511948	1.004504	0.89281603	0.962176
234964	Deup1	deuterosome assembly protein 1, transcript variant X	NA	0.034214	1.084489	1.023999	0.89288754	0.962187
50497	Hspa14	heat shock protein 14, transcript variant 1	NA	0.008124	5.611648	1.005647	0.89294423	0.962187
218460	Wdr41	WD repeat domain 41	NA	0.007887	5.315753	1.005482	0.89303001	0.962216
66061	Tctex1d2	Tctex1 domain containing 2, transcript variant 1	NA	0.015251	3.287679	1.010627	0.89314789	0.962224
102633908	Gm31621	predicted gene, 31621, transcript variant X1	NA	0.047846	0.180661	1.03372	0.89327752	0.962224
83493	Sacm1l	SAC1 suppressor of actin mutations 1-like (yeast), tra	NA	0.008201	5.882111	1.005701	0.89327864	0.962224
22764	Zfx	zinc finger protein X-linked, transcript variant X6	NA	0.012087	5.392015	1.008413	0.89330152	0.962224
13663	Ei24	etoposide induced 2.4 mRNA, transcript variant 1	NA	-0.00609	7.038818	-1.00423	0.89333344	0.962224
320116	Fndc9	fibronectin type III domain containing 9	NA	0.015705	2.844218	1.010945	0.89344894	0.962285
18972	Pold2	polymerase (DNA directed), delta 2, regulatory subur	NA	0.010889	4.524654	1.007576	0.89357009	0.962352
22673	Zfp185	zinc finger protein 185, transcript variant X2	NA	-0.02934	1.296774	-1.02055	0.89386127	0.962544
192216	Tmem47	transmembrane protein 47	NA	-0.00926	5.605886	-1.00644	0.89402619	0.962544
28106	Mydgf	myeloid derived growth factor	NA	0.010195	4.591927	1.007092	0.89410414	0.962544
320586	A630089N07Rik	RIKEN cDNA A630089N07 gene	NA	0.03514	0.247574	1.024657	0.8941728	0.962544
57441	Gmnn	geminin	NA	0.015743	3.201469	1.010972	0.89417773	0.962544
237831	Slc13a5	solute carrier family 13 (sodium-dependent citrate tra	NA	0.028473	1.378788	1.019932	0.89417938	0.962544
73469	Rnf38	ring finger protein 38, transcript variant 5	NA	0.006526	7.451673	1.004534	0.89427752	0.962544
20508	Slc18a3	solute carrier family 18 (vesicular monoamine), meml	NA	-0.02808	1.401356	-1.01965	0.89432557	0.962544
24058	Sigirr	single immunoglobulin and toll-interleukin 1 receptor (	NA	0.040876	0.095222	1.028739	0.89432698	0.962544
17286	Meox2	mesenchyme homeobox 2	NA	0.04102	0.297279	1.028841	0.89441996	0.962544
105242910	Gm38989	predicted gene, 38989, transcript variant X2	NA	-0.03366	0.704954	-1.02361	0.89445432	0.962544
76459	Car12	carbonic anhydrase 12, transcript variant 1	NA	0.022393	3.37014	1.015643	0.89450575	0.962544
20451	St8sia3	ST8 alpha-N-acetyl-neuraminide alpha-2,8-sialyltrans	NA	-0.00634	6.494017	-1.00441	0.89451753	0.962544
20935	Surf6	surfeit gene 6	NA	-0.01062	4.830268	-1.00739	0.89462717	0.962567
50505	Ercc4	excision repair cross-complementing rodent repair de	NA	0.011522	4.482564	1.008018	0.89465701	0.962567
102640173	Gm28175	predicted gene 28175, transcript variant X18	NA	-0.01344	4.17681	-1.00936	0.89486966	0.962704
12995	Csnk2a1	casein kinase 2, alpha 1 polypeptide	NA	0.005675	7.968634	1.003941	0.89490307	0.962704
21983	Tpbg	trophoblast glycoprotein, transcript variant 2	NA	-0.01366	4.274843	-1.00951	0.89503313	0.962725
237943	Gpatch8	G patch domain containing 8, transcript variant X14	NA	0.008633	6.849777	1.006002	0.89504059	0.962725
16582	Kifc3	kinesin family member C3, transcript variant 7	NA	0.011991	5.017116	1.008346	0.89514357	0.962772
102635129	Gm32547	predicted gene, 32547, transcript variant X7	NA	-0.03149	0.667958	-1.02207	0.89520683	0.962776
12647	Chat	choline acetyltransferase	NA	0.056522	-0.50343	1.039956	0.89571246	0.96309
71943	Tom1l1	target of myb1-like 1 (chicken), transcript variant 1	NA	0.011886	3.900835	1.008273	0.89573307	0.96309
20528	Slc2a4	solute carrier family 2 (facilitated glucose transporter)	NA	-0.02219	2.379615	-1.0155	0.89575375	0.96309
215494	Pomgnt2	protein O-linked mannose beta 1,4-N-acetylglucosam	NA	0.010058	4.463459	1.006996	0.89588005	0.96309
66310	Dpy30	dpy-30, histone methyltransferase complex regulatory	NA	0.008132	5.228396	1.005653	0.8959328	0.96309
75116	4930520O04Rik	RIKEN cDNA 4930520O04 gene, transcript variant 2	NA	-0.04668	0.347449	-1.03289	0.8959381	0.96309
16426	Itih3	inter-alpha trypsin inhibitor, heavy chain 3	NA	-0.03676	0.488559	-1.02581	0.89597281	0.96309
101543	Wtip	WT1-interacting protein	NA	0.012237	4.04888	1.008518	0.89603433	0.96309
72612	Hpf1	histone PARylation factor 1, transcript variant X3	NA	0.012329	5.572645	1.008583	0.89608717	0.96309
16434	Itpa	inosine triphosphatase (nucleoside triphosphate pyro	NA	0.006862	6.000953	1.004767	0.89609022	0.96309
70422	Ints2	integrator complex subunit 2, transcript variant X3	NA	-0.00911	5.238973	-1.00634	0.89625212	0.9632
229542	Gatad2b	GATA zinc finger domain containing 2B, transcript va	NA	0.006647	7.348392	1.004618	0.89641246	0.96326
13710	Elf3	E74-like factor 3, transcript variant 2	NA	-0.06578	-0.81563	-1.04665	0.89642583	0.96326
212111	Inpp5a	inositol polyphosphate-5-phosphatase A, transcript va	NA	-0.01051	5.079032	-1.00731	0.89658436	0.963336
239336	Rxfp3	relaxin family peptide receptor 3	NA	0.029143	1.492991	1.020406	0.89675896	0.963336
17873	Gadd45b	growth arrest and DNA-damage-inducible 45 beta	NA	0.023218	1.567931	1.016224	0.89693583	0.963336
13436	Dnmt3b	DNA methyltransferase 3B, transcript variant X13	NA	0.019119	2.439814	1.013341	0.89694018	0.963336
103468	Nup107	nucleoporin 107	NA	-0.00792	5.327224	-1.0055	0.89697427	0.963336
319865	E130114P18Rik	RIKEN cDNA E130114P18 gene	NA	0.012992	3.524324	1.009046	0.89708383	0.963336
102636475	Gm33532	predicted gene, 33532	NA	0.036797	0.141881	1.025834	0.89710731	0.963336
12890	Cplx2	complexin 2, transcript variant 1	NA	-0.00638	8.05517	-1.00443	0.89711166	0.963336
15015	H2-Q4	histocompatibility 2, Q region locus 4	NA	0.025952	1.407572	1.018152	0.8971125	0.963336
77219	Ptgr2	prostaglandin reductase 2, transcript variant 1	NA	0.010367	4.22954	1.007212	0.897131	0.963336
68089	Arpc4	actin related protein 2/3 complex, subunit 4, transcrip	NA	-0.00648	6.955702	-1.0045	0.89714827	0.963336
18711	Pikfyve	phosphoinositide kinase, FYVE type zinc finger conta	NA	0.010159	5.435519	1.007066	0.89723874	0.963352
118567488	LOC118567488	uncharacterized LOC118567488	NA	0.039626	-0.01861	1.027847	0.897319	0.963352
71691	Pnmal1	PNMA-like 1	NA	-0.00669	6.277515	-1.00465	0.89734033	0.963352
102693	Phldb1	pleckstrin homology like domain, family B, member 1	NA	0.006764	6.900571	1.004699	0.89764364	0.96361
66153	Fbxo36	F-box protein 36	NA	0.031416	0.937145	1.022015	0.89773104	0.96361
26434	Prnd	prion like protein doppel, transcript variant 1	NA	0.019833	3.189043	1.013842	0.89775823	0.96361
74203	Eif4enif1	eukaryotic translation initiation factor 4E nuclear impc	NA	0.006803	6.409137	1.004727	0.89790083	0.963637
15220	Foxq1	forkhead box Q1	NA	-0.01696	2.822522	-1.01182	0.89791155	0.963637

142980	Tlr3	toll-like receptor 3, transcript variant 3	NA	0.026908	1.245882	1.018826	0.89796113	0.963637
231044	Gbx1	gastrulation brain homeobox 1	NA	0.032116	0.511165	1.02251	0.89804425	0.963662
108167895	Gm46277	predicted gene, 46277	NA	0.020808	3.464145	1.014528	0.89826493	0.963754
69694	Tatdn1	TatD DNase domain containing 1, transcript variant 3	NA	0.010368	4.317154	1.007213	0.89826902	0.963754
57261	Brd4	bromodomain containing 4, transcript variant X29	NA	-0.0064	7.554134	-1.00445	0.89837024	0.963754
72632	Smim18	small integral membrane protein 18	NA	0.014322	3.284273	1.009977	0.89839451	0.963754
67480	Cwc25	CWC25 spliceosome-associated protein	NA	0.00869	4.866508	1.006042	0.89846815	0.963754
69659	2310069G16Rik	RIKEN cDNA 2310069G16 gene	NA	0.037344	0.168187	1.026223	0.89851198	0.963754
215280	Wipf1	WAS/WASL interacting protein family, member 1, trai	NA	0.014272	3.454798	1.009942	0.89858545	0.963754
218850	Tasor	transcription activation suppressor, transcript variant	NA	-0.00833	6.036428	-1.00579	0.89860322	0.963754
17349	Mlf1	myeloid leukemia factor 1, transcript variant 2	NA	-0.01529	3.260207	-1.01066	0.89880266	0.963891
72502	Cwf19l1	CWF19-like 1, cell cycle control (S. pombe), transcrip	NA	0.008973	4.842773	1.006239	0.89890557	0.963891
105244208	Rps6-ps4	ribosomal protein S6, pseudogene 4	NA	-0.00632	9.028752	-1.00439	0.89894862	0.963891
68350	Mul1	mitochondrial ubiquitin ligase activator of NFKB 1	NA	-0.00932	4.817883	-1.00648	0.89896788	0.963891
56437	Rrad	Ras-related associated with diabetes	NA	0.027488	0.970983	1.019236	0.89904472	0.963908
14934	Gypa	glycophorin A	NA	-0.05766	1.743168	-1.04078	0.89910248	0.963908
329795	Tmem67	transmembrane protein 67, transcript variant 1	NA	0.009944	4.407587	1.006916	0.89922656	0.963939
347722	Agap1	ArfGAP with GTPase domain, ankyrin repeat and PH	NA	0.006095	7.899373	1.004234	0.89924954	0.963939
70021	Nt5dc2	5'-nucleotidase domain containing 2, transcript variar	NA	-0.00608	6.542286	-1.00422	0.89934976	0.963983
67994	Mrps11	mitochondrial ribosomal protein S11, transcript variar	NA	-0.00983	4.171378	-1.00684	0.89956816	0.964117
77721	Mrps5	mitochondrial ribosomal protein S5	NA	0.007938	5.747239	1.005517	0.89966075	0.964117
268783	Mtmr12	myotubularin related protein 12, transcript variant 1	NA	-0.01109	4.413457	-1.00772	0.89969827	0.964117
15937	Ier3	immediate early response 3	NA	0.022639	1.880864	1.015816	0.8997119	0.964117
17083	Tmed1	transmembrane p24 trafficking protein 1, transcript va	NA	0.012427	3.857739	1.008651	0.89978446	0.964131
68861	Dipk2a	divergent protein kinase domain 2A	NA	-0.00928	5.372149	-1.00645	0.90009143	0.964396
67374	Jam2	junction adhesion molecule 2, transcript variant X1	NA	0.007916	5.231742	1.005502	0.90025205	0.964428
74144	Robo4	roundabout guidance receptor 4, transcript variant 2	NA	-0.02033	3.287408	-1.01419	0.90025466	0.964428
13836	Epha2	Eph receptor A2	NA	-0.02034	1.884474	-1.0142	0.90030295	0.964428
11987	Slc7a1	solute carrier family 7 (cationic amino acid transporte	NA	-0.00698	6.563927	-1.00485	0.90040583	0.964428
56709	Dnajb12	DnaJ heat shock protein family (Hsp40) member B12	NA	-0.00931	5.157879	-1.00648	0.90041774	0.964428
108168931	Gm46846	predicted gene, 46846	NA	-0.03281	0.285337	-1.023	0.90052138	0.964476
12569	Cdk5r1	cyclin-dependent kinase 5, regulatory subunit 1 (p35)	NA	0.006936	8.738813	1.00482	0.90070597	0.964522
242702	Myom3	myomesin family, member 3, transcript variant X3	NA	0.03496	0.750581	1.024528	0.90081761	0.964522
76800	Usp42	ubiquitin specific peptidase 42	NA	-0.00665	5.924888	-1.00462	0.90087913	0.964522
67921	Ube2f	ubiquitin-conjugating enzyme E2F (putative), transcri	NA	-0.00927	4.846279	-1.00644	0.90089345	0.964522
108857	Ankhd1	ankyrin repeat and KH domain containing 1	NA	-0.00842	5.959128	-1.00585	0.90091339	0.964522
102639653	Gm2007	predicted gene 2007, transcript variant X4	NA	0.135166	0.973559	1.098219	0.90096282	0.964522
237860	Ssh2	slingshot protein phosphatase 2, transcript variant X5	NA	0.007671	6.924947	1.005331	0.90103611	0.964522
22324	Vav1	vav 1 oncogene, transcript variant 3	NA	0.035776	0.552086	1.025108	0.90103838	0.964522
68440	Dusp23	dual specificity phosphatase 23	NA	0.021975	1.966833	1.015349	0.90117788	0.964608
215436	Slc35e3	solute carrier family 35, member E3	NA	-0.00646	5.884216	-1.00449	0.90126674	0.964639
331487	Uprt	uracil phosphoribosyltransferase	NA	-0.01293	4.704359	-1.009	0.90141173	0.964731
17240	Mdfi	MyoD family inhibitor, transcript variant 3	NA	-0.0167	2.547143	-1.01164	0.90182467	0.96511
19943	Rpl28	ribosomal protein L28	NA	0.008045	8.259296	1.005592	0.90192474	0.965153
241576	Ldlrad3	low density lipoprotein receptor class A domain conta	NA	0.008001	5.470347	1.005561	0.90206376	0.9652
668212	Efr3b	EFR3 homolog B, transcript variant X4	NA	0.006026	7.527874	1.004185	0.9021726	0.9652
231506	Lin54	lin-54 homolog (C. elegans), transcript variant 2	NA	-0.01061	4.037115	-1.00738	0.90219682	0.9652
217866	Cdc42bpb	CDC42 binding protein kinase beta	NA	0.005913	7.323446	1.004107	0.90237255	0.9652
100502745	2900009J06Rik	RIKEN cDNA 2900009J06 gene	NA	0.02841	1.049552	1.019888	0.90237881	0.9652
380850	Gm5141	predicted gene 5141	NA	0.012254	3.264862	1.00853	0.90245443	0.9652
212569	Zfp273	zinc finger protein 273	NA	0.013561	3.538704	1.009444	0.90248502	0.9652
433667	Ankrd13c	ankyrin repeat domain 13c, transcript variant 1	NA	0.007835	5.965107	1.005445	0.90250002	0.9652
66456	2810001G20Rik	RIKEN cDNA 2810001G20 gene	NA	0.012736	3.629018	1.008867	0.90252965	0.9652
433693	Akirin2	akirin 2	NA	0.006955	6.821207	1.004832	0.90256166	0.9652
69028	Mitd1	MIT, microtubule interacting and transport, domain cc	NA	0.015275	3.195613	1.010644	0.90277593	0.965286
212090	Tmem60	transmembrane protein 60	NA	0.012925	3.96326	1.008999	0.90288724	0.965286
69546	Mapk1ip1	mitogen-activated protein kinase 1 interacting protein	NA	0.008442	5.099815	1.005869	0.90291899	0.965286
114774	Pawr	PRKC, apoptosis, WT1, regulator, transcript variant X	NA	-0.01593	2.359687	-1.0111	0.9029257	0.965286
66354	Snw1	SNW domain containing 1	NA	-0.00655	6.451964	-1.00455	0.90296643	0.965286
224893	Zfp959	zinc finger protein 959, transcript variant 2	NA	-0.01397	3.441403	-1.00973	0.90302733	0.965286
142688	Asb13	ankyrin repeat and SOCS box-containing 13, transcri	NA	0.009461	4.702113	1.00658	0.90305716	0.965286
100862203	Gm6712	predicted gene 6712	NA	-0.01823	2.210928	-1.01271	0.90316694	0.965299
22034	Traf6	TNF receptor-associated factor 6, transcript variant 2	NA	0.008069	4.740263	1.005609	0.90318802	0.965299
50709	H1f4	H1.4 linker histone, cluster member	NA	-0.05412	-0.02912	-1.03823	0.9033258	0.965383
115489466	LOC115489466	uncharacterized LOC115489466	NA	0.049553	0.722104	1.034944	0.90347815	0.965433
16145	Igtp	interferon gamma induced GTPase	NA	0.030968	0.606486	1.021697	0.90353862	0.965433
11768	Ap1m2	adaptor protein complex AP-1, mu 2 subunit, transcri	NA	0.041126	0.69379	1.028917	0.90357119	0.965433
19712	Rest	RE1-silencing transcription factor, transcript variant X	NA	0.009541	4.501162	1.006635	0.90361045	0.965433

20778	Scarb1	scavenger receptor class B, member 1, transcript var	NA		0.007347	5.122405	1.005105	0.90374047	0.965463
77533	C030034I22Rik	RIKEN cDNA C030034I22 gene	NA		0.019755	2.505708	1.013788	0.90378626	0.965463
225998	Rorb	RAR-related orphan receptor beta, transcript variant :	NA		0.008144	5.027189	1.005661	0.90381665	0.965463
104086	Cyp27a1	cytochrome P450, family 27, subfamily a, polypeptide	NA		-0.03686	0.100074	-1.02588	0.90404113	0.965562
320152	4930412C18Rik	RIKEN cDNA 4930412C18 gene	NA		0.041832	-0.23064	1.02942	0.90416542	0.965562
223255	Stk24	serine/threonine kinase 24, transcript variant X2	NA		0.005904	6.3043	1.004101	0.90417544	0.965562
21873	Tjp2	tight junction protein 2, transcript variant 1	NA		-0.00907	4.814645	-1.00631	0.90423704	0.965562
66799	Ube2w	ubiquitin-conjugating enzyme E2W (putative), transcr	NA		0.006533	6.083	1.004538	0.9042656	0.965562
72096	Eef1akmt2	EEF1A lysine methyltransferase 2, transcript variant	NA		0.009604	4.639702	1.006679	0.9042731	0.965562
20917	Suclg2	succinate-Coenzyme A ligase, GDP-forming, beta su	NA		0.008823	4.467133	1.006134	0.90432391	0.965562
14173	Fgf2	fibroblast growth factor 2, transcript variant X1	NA		0.030467	1.429008	1.021342	0.90462939	0.965824
27397	Mrpl17	mitochondrial ribosomal protein L17	NA		0.00906	4.733304	1.0063	0.9050148	0.966172
545192	Baiap3	BAI1-associated protein 3	NA		-0.01325	3.990253	-1.00923	0.90523411	0.966312
18503	Pax1	paired box 1	NA		0.060482	-1.53906	1.042814	0.90528914	0.966312
15221	Foxd3	forkhead box D3	NA		0.039058	0.451626	1.027443	0.90535217	0.966312
69743	Casz1	castor zinc finger 1, transcript variant 1	NA		-0.0194	2.839122	-1.01354	0.90541496	0.966312
26448	Mok	MOK protein kinase, transcript variant X36	NA		0.013376	3.201645	1.009315	0.90550278	0.966312
80879	Slc16a3	solute carrier family 16 (monocarboxylic acid transpo	NA		-0.02593	2.272291	-1.01814	0.90553657	0.966312
320720	Fastkd1	FAST kinase domains 1, transcript variant X1	NA		0.009944	4.464306	1.006917	0.90556153	0.966312
66603	Gemin2	gem nuclear organelle associated protein 2	NA		-0.01568	3.040237	-1.01093	0.90563131	0.966323
72572	Spats2	spermatogenesis associated, serine-rich 2, transcript	NA		-0.00668	6.209244	-1.00464	0.90598547	0.966449
23880	Fyb	FYN binding protein, transcript variant X3	NA		-0.02164	1.44441	-1.01512	0.90602801	0.966449
100502825	Rpl37rt	ribosomal protein L37, retrotransposed, transcript var	NA		-0.00822	6.243914	-1.00571	0.9061146	0.966449
56529	Sec11a	SEC11 homolog A, signal peptidase complex subunit	NA		0.007363	5.571287	1.005116	0.90620042	0.966449
69310	Pacrg	PARK2 co-regulated	NA		0.013189	3.028319	1.009184	0.90621482	0.966449
12524	Cd86	CD86 antigen	NA		-0.02811	0.601662	-1.01967	0.90626648	0.966449
170776	Cd209c	CD209c antigen	NA		-0.03228	0.619441	-1.02263	0.90628961	0.966449
104079	Nxph3	neurexophilin 3	NA		-0.00922	4.64152	-1.00641	0.90636672	0.966449
245847	Amdhd2	amidohydrolase domain containing 2	NA		0.014055	3.212263	1.00979	0.90640002	0.966449
192231	Hexim1	hexamethylene bis-acetamide inducible 1	NA		-0.00809	5.342229	-1.00562	0.9064152	0.966449
24105	Rbck1	RanBP-type and C3HC4-type zinc finger containing 1	NA		0.006521	5.96892	1.00453	0.90648073	0.966449
230700	Foxj3	forkhead box J3, transcript variant 2	NA		-0.00558	6.55204	-1.00387	0.90650471	0.966449
22117	Tst	thiosulfate sulfurtransferase, mitochondrial	NA		0.013976	3.227639	1.009734	0.90654962	0.966449
26946	Trpc7	transient receptor potential cation channel, subfamily	NA		0.01716	2.580988	1.011965	0.9066294	0.966449
118567721	LOC118567721	uncharacterized LOC118567721, transcript variant X	NA		-0.01822	2.830723	-1.01271	0.9066399	0.966449
66352	Blzf1	basic leucine zipper nuclear factor 1, transcript variar	NA		0.009268	4.669729	1.006445	0.90686046	0.966621
12462	Cct3	chaperonin containing Tcp1, subunit 3 (gamma)	NA		-0.00585	7.930413	-1.00406	0.90692763	0.966629
102641552	Gm38563	predicted gene, 38563, transcript variant X1	NA		-0.03582	0.076938	-1.02514	0.90710489	0.966662
232227	Iqsec1	IQ motif and Sec7 domain 1, transcript variant X19	NA		0.005493	6.991376	1.003815	0.90713111	0.966662
225182	Rbbp8	retinoblastoma binding protein 8, endonuclease, trans	NA		-0.01019	3.933526	-1.00709	0.90714762	0.966662
331578	AW822252	expressed sequence AW822252	NA		0.047636	-0.34797	1.03357	0.90722225	0.966662
66568	Rwdd3	RWD domain containing 3, transcript variant 2	NA		0.012956	3.280696	1.009021	0.90725589	0.966662
20430	Cyfip1	cytoplasmic FMR1 interacting protein 1, transcript vai	NA		0.006741	5.947355	1.004684	0.90735188	0.966701
279610	E530001F21Rik	RIKEN cDNA E530001F21 gene	NA		0.031252	0.238033	1.021898	0.90751507	0.966792
74450	Pank2	pantothenate kinase 2, transcript variant 3	NA		0.005997	5.862376	1.004166	0.90762597	0.966792
415115	Neurl2	neuralized E3 ubiquitin protein ligase 2	NA		-0.0281	1.210416	-1.01967	0.90762793	0.966792
72634	Tdrkh	tudor and KH domain containing protein, transcript va	NA		0.007128	6.035626	1.004953	0.90767474	0.966792
56088	Psmg1	proteasome (prosome, macropain) assembly chaperc	NA		-0.00928	4.242543	-1.00645	0.90795153	0.967024
66220	Zdhhc12	zinc finger, DHHC domain containing 12, transcript va	NA		-0.01508	2.583648	-1.0105	0.90807333	0.96709
320268	B930095G15Rik	RIKEN cDNA B930095G15 gene	NA		-0.00575	6.350639	-1.00399	0.90814412	0.967102
78926	Gas2l1	growth arrest-specific 2 like 1, transcript variant delta	NA		-0.00917	4.205866	-1.00638	0.90828087	0.967184
118568307	LOC118568307	igE-binding protein-like	NA		0.021912	2.342983	1.015305	0.90834573	0.96719
67170	2610306M01Rik	RIKEN cDNA 2610306M01 gene	NA		-0.01492	2.50141	-1.01039	0.90840528	0.96719
76894	Mettl15	methyltransferase like 15	NA		0.017679	2.217996	1.012329	0.90848805	0.967215
19989	Rpl7	ribosomal protein L7	NA		-0.00556	9.431283	-1.00386	0.90860247	0.967223
54445	Unc93b1	unc-93 homolog B1, TLR signaling regulator, transcri	NA		0.014882	2.386052	1.010369	0.90871827	0.967223
108735	Sft2d2	SFT2 domain containing 2, transcript variant 1	NA		0.009859	4.044289	1.006857	0.9087221	0.967223
225631	Onecut2	one cut domain, family member 2	NA		-0.01163	5.095913	-1.00809	0.90877418	0.967223
244895	Peak1	pseudopodium-enriched atypical kinase 1	NA		-0.00956	4.183485	-1.00665	0.90885675	0.967223
20974	Syngr3	synaptogyrin 3	NA		0.006431	6.335629	1.004468	0.90887616	0.967223
72720	Zfp248	zinc finger protein 248, transcript variant 1	NA		-0.00978	4.314379	-1.0068	0.90891154	0.967223
73191	Fezf1	Fez family zinc finger 1	NA		-0.01687	2.759252	-1.01176	0.90902719	0.967283
226971	Plekhb2	pleckstrin homology domain containing, family B (eve	NA		-0.00662	5.809092	-1.0046	0.90929272	0.967289
100038388	Gm10614	predicted gene 10614, transcript variant X7	NA		0.04052	-0.03259	1.028485	0.90935181	0.967289
102636806	Gm33777	predicted gene, 33777	NA		0.01222	4.389538	1.008506	0.90946356	0.967289
59308	Emcn	endomucin, transcript variant 1	NA		0.013308	3.166059	1.009267	0.90947914	0.967289
242773	Slc45a1	solute carrier family 45, member 1, transcript variant	NA		-0.0089	4.900842	-1.00619	0.9095249	0.967289
21411	Tcf20	transcription factor 20, transcript variant X3	NA		0.007215	7.181652	1.005014	0.90952619	0.967289

58996	Arhgap23	Rho GTPase activating protein 23, transcript variant)	NA	0.006381	6.341365	1.004433	0.90956196	0.967289
230259	E130308A19Rik	RIKEN cDNA E130308A19 gene, transcript variant X	NA	-0.00672	5.684845	-1.00467	0.90960747	0.967289
24082	Map2k3os	mitogen-activated protein kinase kinase 3, opposite s	NA	-0.03142	0.186852	-1.02201	0.90961311	0.967289
19014	Med1	mediator complex subunit 1, transcript variant X4	NA	-0.00667	6.222728	-1.00463	0.90962705	0.967289
29820	Tnfrsf19	tumor necrosis factor receptor superfamily, member '	NA	-0.00721	5.822987	-1.00501	0.9097413	0.967347
68083	Pak1ip1	PAK1 interacting protein 1	NA	-0.00664	5.39989	-1.00461	0.90981596	0.967363
105246679	Gm37053	predicted gene, 37053	NA	-0.05971	-0.63656	-1.04225	0.91019391	0.967702
19720	Trim27	tripartite motif-containing 27, transcript variant X2	NA	-0.00679	5.81212	-1.00472	0.9103082	0.967756
72008	Zfyve19	zinc finger, FYVE domain containing 19, transcript va	NA	0.009339	4.474689	1.006494	0.91036427	0.967756
268527	Greb1	gene regulated by estrogen in breast cancer protein,	NA	-0.02355	1.657744	-1.01646	0.91055139	0.967892
118568243	LOC118568243	uncharacterized LOC118568243	NA	0.017678	2.123327	1.012329	0.91065949	0.967944
54637	Praf2	PRA1 domain family 2	NA	0.006167	5.814355	1.004284	0.91076755	0.967956
21689	Tekt1	tektin 1, transcript variant 1	NA	0.014296	2.747977	1.009959	0.91078986	0.967956
224129	Adcy5	adenylate cyclase 5, transcript variant X1	NA	-0.00557	6.353162	-1.00387	0.91102826	0.968083
102637537	Gm34321	predicted gene, 34321	NA	-0.022	3.475981	-1.01537	0.91108374	0.968083
442801	Arhgef15	Rho guanine nucleotide exchange factor (GEF) 15, tr	NA	0.00768	4.764944	1.005337	0.91112941	0.968083
211488	Ado	2-aminoethanethiol (cysteamine) dioxygenase	NA	0.006332	6.042334	1.004398	0.91115414	0.968083
59056	Evc	EvC ciliary complex subunit 1, transcript variant X8	NA	0.012798	3.542119	1.008911	0.91120708	0.968083
234309	Cbr4	carbonyl reductase 4	NA	-0.01485	2.88426	-1.01034	0.91129952	0.968118
234889	Gucy1a2	guanylate cyclase 1, soluble, alpha 2	NA	0.010589	4.85906	1.007367	0.91145889	0.968211
108101	Fermt3	fermitin family member 3, transcript variant 2	NA	0.018345	1.790431	1.012797	0.91150579	0.968211
20491	Sla	src-like adaptor, transcript variant X3	NA	-0.01089	5.900683	-1.00758	0.91190006	0.968516
66980	Zdhhc6	zinc finger, DHHC domain containing 6, transcript var	NA	0.0078	5.052776	1.005421	0.91191242	0.968516
382073	Ccdc84	coiled-coil domain containing 84, transcript variant X9	NA	0.009175	4.451114	1.00638	0.91218461	0.968685
73830	Eif3k	eukaryotic translation initiation factor 3, subunit K, tra	NA	-0.00657	6.722985	-1.00456	0.91219032	0.968685
75410	Kmt2b	lysine (K)-specific methyltransferase 2B, transcript va	NA	0.00551	6.691273	1.003827	0.91228494	0.968722
209176	Ido2	indoleamine 2,3-dioxygenase 2, transcript variant X2	NA	-0.03079	0.445152	-1.02157	0.91289861	0.968819
57443	Fbxo3	F-box protein 3, transcript variant 1	NA	0.006717	6.527045	1.004667	0.9129205	0.968819
97761	Sgsm2	small G protein signaling modulator 2, transcript varia	NA	0.006034	6.247915	1.004191	0.91299378	0.968819
70266	Kyat1	kynurenine aminotransferase 1, transcript variant 2	NA	-0.01364	3.87526	-1.0095	0.91300543	0.968819
21781	Tfdp1	transcription factor Dp 1, transcript variant 3	NA	0.005897	6.714014	1.004096	0.91301652	0.968819
328370	Rft1	RFT1 homolog	NA	0.012612	3.290424	1.00878	0.91302107	0.968819
105245351	Gm40823	predicted gene, 40823, transcript variant X2	NA	0.04236	-0.34025	1.029797	0.913047	0.968819
19052	Ppp2ca	protein phosphatase 2 (formerly 2A), catalytic subunit	NA	0.004716	8.757135	1.003274	0.91306312	0.968819
20239	Atxn2	ataxin 2, transcript variant 1	NA	0.005802	6.949159	1.00403	0.91308277	0.968819
105243369	Gm15521	predicted gene 15521, transcript variant X2	NA	0.010555	5.371774	1.007343	0.91316413	0.968819
77766	Elp4	elongator acetyltransferase complex subunit 4	NA	0.007032	5.153243	1.004886	0.91325117	0.968819
68318	Aph1c	aph1 homolog C, gamma secretase subunit	NA	0.020478	1.821797	1.014296	0.9132564	0.968819
77579	Myh10	myosin, heavy polypeptide 10, non-muscle	NA	0.005541	8.331587	1.003848	0.9132589	0.968819
18197	Nsg2	neuron specific gene family member 2, transcript vari	NA	-0.00507	10.20337	-1.00352	0.91330282	0.968819
21336	Tacr1	tachykinin receptor 1, transcript variant X3	NA	-0.01388	2.641801	-1.00967	0.91333941	0.968819
71887	Ppm1j	protein phosphatase 1J	NA	0.03089	0.070881	1.021642	0.9134583	0.968819
18994	Pou3f4	POU domain, class 3, transcription factor 4	NA	0.007038	5.310169	1.00489	0.91346137	0.968819
59047	Pnkp	polynucleotide kinase 3'- phosphatase, transcript vari	NA	-0.00906	4.290521	-1.0063	0.91350318	0.968819
68539	Tmem109	transmembrane protein 109	NA	-0.00733	4.737341	-1.0051	0.91350732	0.968819
217449	Trappc12	trafficking protein particle complex 12, transcript varia	NA	-0.00674	5.433424	-1.00468	0.91363109	0.968887
212627	Prpsap2	phosphoribosyl pyrophosphate synthetase-associate	NA	-0.00745	5.820578	-1.00518	0.91396791	0.969181
114841036	LOC114841036	Uncharacterized protein LOC114841036	NA	-0.01543	2.259528	-1.01075	0.91431239	0.969433
20893	Bhlhe40	basic helix-loop-helix family, member e40	NA	0.01201	3.568868	1.008359	0.91442231	0.969433
109359	Abraxas2	BRISC complex subunit	NA	0.006732	5.265786	1.004677	0.91444151	0.969433
66948	Acad8	acyl-Coenzyme A dehydrogenase family, member 8,	NA	0.00872	4.419431	1.006062	0.91444325	0.969433
69713	Pin4	protein (peptidyl-prolyl cis/trans isomerase) NIMA-inte	NA	-0.0112	4.126	-1.00779	0.91459191	0.969527
105245981	Gm41349	predicted gene, 41349, transcript variant X2	NA	-0.03301	0.533726	-1.02315	0.91473208	0.96959
102635731	Gm32994	predicted gene, 32994, transcript variant X5	NA	-0.03617	-0.32436	-1.02539	0.9147709	0.96959
408022	Primpol	primase and polymerase (DNA-directed), transcript va	NA	0.011584	3.67318	1.008061	0.91485402	0.969615
21761	Morf4l1	mortality factor 4 like 1, transcript variant 2	NA	-0.00525	8.994209	-1.00364	0.91504934	0.969759
12704	Cit	citron, transcript variant X36	NA	0.00695	5.117055	1.004829	0.9152621	0.969922
56451	Suclg1	succinate-CoA ligase, GDP-forming, alpha subunit	NA	-0.0055	5.964518	-1.00382	0.91554502	0.97012
13628	Eef1a2	eukaryotic translation elongation factor 1 alpha 2	NA	-0.00531	7.720265	-1.00369	0.91556867	0.97012
100504309	Smim27	small integral membrane protein 27	NA	-0.0331	-0.24492	-1.02321	0.91581838	0.970284
14371	Fzd9	frizzled class receptor 9	NA	0.015133	3.232022	1.010545	0.91588217	0.970284
13483	Dpp6	dipeptidylpeptidase 6, transcript variant 1	NA	-0.00549	7.405404	-1.00381	0.91590215	0.970284
217431	Nol10	nucleolar protein 10	NA	0.009501	4.811959	1.006607	0.91611013	0.97032
403185	Cfap97d2	CFAP97 domain containing 2, transcript variant X3	NA	-0.04193	0.209619	-1.02949	0.91619359	0.97032
234912	Cfap300	cilia and flagella associated protein 300, transcript va	NA	0.011677	3.450585	1.008126	0.91621467	0.97032
207175	Cetn4	centrin 4	NA	-0.02208	1.14091	-1.01542	0.9162394	0.97032
216873	Spag7	sperm associated antigen 7, transcript variant 1	NA	-0.00582	5.657534	-1.00404	0.91628044	0.97032
13732	Emp3	epithelial membrane protein 3, transcript variant 1	NA	0.017291	1.89485	1.012057	0.91640196	0.97032

22329	Vcam1	vascular cell adhesion molecule 1	NA	0.007335	5.386192	1.005097	0.91644292	0.97032
668171	Zxda	zinc finger, X-linked, duplicated A	NA	0.011082	3.530003	1.007711	0.91656176	0.97032
230751	Oscp1	organic solute carrier partner 1, transcript variant 1	NA	-0.00805	5.044047	-1.00559	0.91660017	0.97032
26940	Ecsit	ECSIT signalling integrator, transcript variant 1	NA	-0.00781	4.872385	-1.00543	0.91661648	0.97032
319786	B930059L03Rik	RIKEN cDNA B930059L03 gene	NA	-0.03962	-0.25282	-1.02784	0.91666207	0.97032
230025	Prdm13	PR domain containing 13, transcript variant X2	NA	-0.03799	0.480993	-1.02668	0.91669193	0.97032
223775	Pim3	proviral integration site 3	NA	-0.00761	5.177783	-1.00529	0.91679834	0.97032
20741	Sptb	spectrin beta, erythrocytic	NA	-0.01144	4.817478	-1.00796	0.91682299	0.97032
102634715	Gm32234	predicted gene, 32234	NA	0.035071	0.258984	1.024607	0.91686566	0.97032
105244926	Gm40448	predicted gene, 40448	NA	0.014883	2.321835	1.010369	0.91689036	0.97032
20167	Rtn2	reticulon 2 (Z-band associated protein), transcript var	NA	0.005899	6.752862	1.004097	0.91695216	0.970322
11951	Atp5g1	ATP synthase, H+ transporting, mitochondrial F0 con	NA	-0.00615	5.998103	-1.00427	0.91726323	0.97058
70574	Cpm	carboxypeptidase M, transcript variant X2	NA	0.011424	3.174571	1.00795	0.91731455	0.97058
67704	1810037I17Rik	RIKEN cDNA 1810037I17 gene	NA	-0.00771	4.913265	-1.00536	0.91745874	0.970615
140858	Wdr5	WD repeat domain 5	NA	0.0057	6.052398	1.003958	0.91751162	0.970615
107769	Tm6sf1	transmembrane 6 superfamily member 1, transcript v	NA	-0.01183	3.148118	-1.00824	0.91760397	0.970615
231821	Adap1	ArfGAP with dual PH domains 1	NA	-0.00576	6.309261	-1.004	0.917648	0.970615
636104	Cfap47	cilia and flagella associated protein 47, transcript vari	NA	0.021299	1.047894	1.014873	0.91769119	0.970615
74034	4632404H12Rik	RIKEN cDNA 4632404H12 gene	NA	0.012289	3.22276	1.008555	0.91783502	0.970615
67426	Coq8a	coenzyme Q8A, transcript variant X2	NA	-0.01705	1.797018	-1.01189	0.91789255	0.970615
213988	Tnrc6b	trinucleotide repeat containing 6b, transcript variant 2	NA	0.005074	7.035179	1.003523	0.9179414	0.970615
14148	Fdx1	ferredoxin 1, transcript variant 1	NA	-0.0138	3.217356	-1.00961	0.91795857	0.970615
233060	Zfp382	zinc finger protein 382, transcript variant 1	NA	-0.00698	5.054329	-1.00485	0.91798336	0.970615
67230	Zfp329	zinc finger protein 329	NA	0.005774	6.025055	1.00401	0.91804246	0.970615
118567339	LOC118567339	uncharacterized LOC118567339	NA	-0.01584	3.052507	-1.01104	0.91812748	0.970615
207806	Usf3	upstream transcription factor family member 3	NA	-0.00714	5.393714	-1.00496	0.91815954	0.970615
101202	Hepacam2	HEPACAM family member 2, transcript variant X1	NA	-0.0376	0.190681	-1.0264	0.91821302	0.970615
98267	Stk17b	serine/threonine kinase 17b (apoptosis-inducing)	NA	0.017323	2.60087	1.01208	0.91825604	0.970615
22411	Wnt11	wingless-type MMTV integration site family, member	NA	-0.03698	0.37576	-1.02597	0.91835466	0.970615
70762	Dclk2	doublecortin-like kinase 2, transcript variant 4	NA	-0.0048	7.974743	-1.00333	0.91842393	0.970615
102632286	Gm30401	predicted gene, 30401, transcript variant X2	NA	0.026751	1.388287	1.018716	0.91842564	0.970615
102638270	Gm34872	predicted gene, 34872, transcript variant X4	NA	-0.03123	0.01209	-1.02188	0.91848168	0.970615
100043636	AI662270	expressed sequence AI662270	NA	0.043429	0.033804	1.030561	0.91862296	0.970655
214505	Gnptg	N-acetylglucosamine-1-phosphotransferase, gamma	NA	0.006311	5.744195	1.004384	0.9186387	0.970655
232087	Mat2a	methionine adenosyltransferase II, alpha, transcript v	NA	0.004595	8.083618	1.00319	0.91876268	0.970723
118567855	LOC118567855	uncharacterized LOC118567855	NA	-0.01672	3.890638	-1.01166	0.91894147	0.970849
14275	Folr1	folate receptor 1 (adult), transcript variant X6	NA	0.013712	2.792269	1.00955	0.91930969	0.971103
381605	Tbc1d2	TBC1 domain family, member 2, transcript variant X5	NA	0.022148	0.851026	1.01547	0.91939933	0.971103
100041677	Zfp984	zinc finger protein 984	NA	-0.00997	3.432739	-1.00694	0.91943604	0.971103
69716	Trip13	thyroid hormone receptor interactor 13	NA	-0.01091	3.606502	-1.00759	0.91944327	0.971103
74493	Tnks2	tankyrase, TRF1-interacting ankyrin-related ADP-ribc	NA	-0.00714	7.699577	-1.00496	0.91951073	0.971103
217882	Cep170b	centrosomal protein 170B, transcript variant X1	NA	-0.00565	7.05323	-1.00392	0.91954034	0.971103
237422	Ric8b	RIC8 guanine nucleotide exchange factor B, transcrip	NA	0.005866	6.042569	1.004074	0.91966305	0.971144
102636582	Gm26555	predicted gene, 26555	NA	0.019499	1.200499	1.013607	0.91978286	0.971144
320977	A330023F24Rik	RIKEN cDNA A330023F24 gene	NA	-0.01052	3.17743	-1.00732	0.91980783	0.971144
68017	Mrm2	mitochondrial rRNA methyltransferase 2	NA	-0.01539	3.024317	-1.01073	0.91987499	0.971144
22637	Zap70	zeta-chain (TCR) associated protein kinase, transcrip	NA	-0.02979	0.793377	-1.02086	0.91991474	0.971144
84585	Rnf123	ring finger protein 123, transcript variant 2	NA	0.006317	5.990265	1.004388	0.91993682	0.971144
100040822	Gm1604a	predicted gene 1604A, transcript variant 6	NA	-0.03307	0.242227	-1.02318	0.92000108	0.971149
268749	Rnf31	ring finger protein 31	NA	0.007159	4.442259	1.004975	0.92012634	0.971218
227612	Tor4a	torsin family 4, member A	NA	-0.02135	1.623163	-1.01491	0.92042546	0.971373
78749	Filip1l	filamin A interacting protein 1-like, transcript variant 2	NA	-0.01471	2.135484	-1.01025	0.92044725	0.971373
74706	4930507D05Rik	RIKEN cDNA 4930507D05 gene	NA	-0.03107	-0.06754	-1.02177	0.92047319	0.971373
217733	Tmem63c	transmembrane protein 63c, transcript variant 3	NA	-0.00778	4.865939	-1.00541	0.92057397	0.971373
68607	Serhl	serine hydrolase-like	NA	-0.01996	1.807135	-1.01393	0.92062256	0.971373
69952	Tunar	Tcl1 upstream neural differentiation associated RNA,	NA	0.009658	5.146581	1.006717	0.92063159	0.971373
102639538	Gm35829	predicted gene, 35829	NA	0.035645	-0.12402	1.025015	0.92071895	0.971402
208213	Tmem132c	transmembrane protein 132C	NA	-0.00679	5.012414	-1.00472	0.92078606	0.97141
320770	A630072M18Rik	RIKEN cDNA A630072M18 gene	NA	0.014762	2.688728	1.010285	0.92091577	0.971468
115488174	Gm52054	predicted gene, 52054, transcript variant X3	NA	-0.04257	0.026822	-1.02995	0.92095991	0.971468
69721	Nkiras1	NFKB inhibitor interacting Ras-like protein 1, transcrip	NA	-0.00741	4.504303	-1.00515	0.92122781	0.971687
13356	Dgcr2	DiGeorge syndrome critical region gene 2, transcript	NA	-0.00468	7.290495	-1.00325	0.92128886	0.971689
21946	Pglyrp1	peptidoglycan recognition protein 1	NA	-0.01704	2.265325	-1.01188	0.92148297	0.971776
26465	Zfp146	zinc finger protein 146, transcript variant 1	NA	-0.00506	6.694818	-1.00351	0.92149081	0.971776
115488820	Gm52316	predicted gene, 52316	NA	-0.02155	1.187921	-1.01505	0.92175979	0.971996
76355	Tgds	TDP-glucose 4,6-dehydratase	NA	-0.00942	3.66579	-1.00655	0.92183556	0.972013
71738	Mamdc2	MAM domain containing 2	NA	-0.02093	1.73329	-1.01461	0.92190425	0.972023
115488162	LOC115488162	uncharacterized LOC115488162	NA	-0.05532	0.135525	-1.03909	0.92207631	0.972141

217294	BC006965	cDNA sequence BC006965	NA	0.033753	0.142188	1.023672	0.92213852	0.972144
17904	Myl6	myosin, light polypeptide 6, alkali, smooth muscle and	NA	0.004396	7.75819	1.003052	0.92224509	0.972164
67942	Atp5g2	ATP synthase, H+ transporting, mitochondrial F0 com	NA	0.006155	6.491013	1.004276	0.92233425	0.972164
234779	Plcg2	phospholipase C, gamma 2	NA	-0.02124	1.100985	-1.01483	0.92233683	0.972164
67019	Actr6	ARP6 actin-related protein 6, transcript variant 1	NA	-0.00675	4.655368	-1.00469	0.92261845	0.972276
224691	Zfp472	zinc finger protein 472	NA	0.011419	3.176188	1.007947	0.92281783	0.972276
286940	Flnb	filamin, beta, transcript variant X1	NA	0.006336	5.896268	1.004401	0.92281949	0.972276
79202	Tnfrsf22	tumor necrosis factor receptor superfamily, member 2	NA	-0.01418	2.346212	-1.00988	0.92285191	0.972276
225152	Gjd4	gap junction protein, delta 4, transcript variant X1	NA	0.047262	-1.52976	1.033302	0.92305382	0.972276
66119	Tomm6	translocase of outer mitochondrial membrane 6, trans	NA	0.007302	5.919614	1.005074	0.92328002	0.972276
328778	Rab26	RAB26, member RAS oncogene family	NA	-0.04463	0.393287	-1.03142	0.92335755	0.972276
353326	Rtl1	retrotransposon Gaglike 1, transcript variant X1	NA	0.012773	3.443044	1.008893	0.92336977	0.972276
74194	Rnd3	Rho family GTPase 3	NA	-0.00453	7.303538	-1.00315	0.92337488	0.972276
108167945	Gm46305	predicted gene, 46305	NA	-0.02958	0.356723	-1.02071	0.92341602	0.972276
100504446	Prr22	proline rich 22	NA	0.032363	-0.13233	1.022685	0.92345059	0.972276
12962	Crybb3	crystallin, beta B3, transcript variant X2	NA	0.035006	0.522273	1.024561	0.92350126	0.972276
69587	Pcgf3	polycomb group ring finger 3, transcript variant 1	NA	0.004655	7.008758	1.003232	0.92352992	0.972276
105246555	Gm41829	predicted gene, 41829	NA	-0.00799	3.897459	-1.00555	0.92355325	0.972276
207214	Larp4	La ribonucleoprotein domain family, member 4, transc	NA	-0.00797	5.819105	-1.00554	0.92357822	0.972276
109229	Fam118b	family with sequence similarity 118, member B, transc	NA	-0.00577	5.185047	-1.00401	0.92361208	0.972276
17768	Mthfd2	methylenetetrahydrofolate dehydrogenase (NAD+ de	NA	-0.00799	4.364616	-1.00556	0.92369425	0.972276
18143	Npas2	neuronal PAS domain protein 2, transcript variant X1	NA	0.018673	1.841652	1.013027	0.92374491	0.972276
14131	Fcgr3	Fc receptor, IgG, low affinity III, transcript variant 1	NA	-0.01665	2.538321	-1.01161	0.92378277	0.972276
72181	Nsun4	NOL1/NOP2/Sun domain family, member 4, transcrip	NA	-0.00975	3.472494	-1.00678	0.9238705	0.972276
241490	Rbm45	RNA binding motif protein 45	NA	-0.00707	5.279724	-1.00491	0.92392249	0.972276
66922	Rras2	related RAS viral (r-ras) oncogene 2	NA	0.008073	5.253005	1.005612	0.92392582	0.972276
12408	Cbr1	carbonyl reductase 1	NA	-0.00566	5.832362	-1.00393	0.92393219	0.972276
12448	Ccne2	cyclin E2, transcript variant X4	NA	-0.01232	3.421137	-1.00857	0.92393778	0.972276
67574	Alg13	asparagine-linked glycosylation 13, transcript variant	NA	-0.00974	3.980136	-1.00677	0.92398802	0.972276
328734	Pisd-ps2	phosphatidylserine decarboxylase, pseudogene 2	NA	-0.01663	1.832012	-1.0116	0.92407289	0.972276
59027	Nampt	nicotinamide phosphoribosyltransferase	NA	-0.00524	5.988894	-1.00364	0.92412397	0.972276
50905	Il17rb	interleukin 17 receptor B	NA	-0.04637	-0.33887	-1.03266	0.92412902	0.972276
66096	Lamtor4	late endosomal/lysosomal adaptor, MAPK and MTOF	NA	-0.00813	4.206818	-1.00565	0.92417632	0.972276
118567669	LOC118567669	uncharacterized LOC118567669	NA	-0.02096	1.223963	-1.01464	0.92429915	0.972343
21807	Tsc22d1	TSC22 domain family, member 1, transcript variant 2	NA	-0.00401	9.304571	-1.00278	0.92444871	0.972359
352968	D830050J10Rik	RIKEN cDNA D830050J10 gene	NA	-0.03251	0.260078	-1.02279	0.9244875	0.972359
26450	Rbbp9	retinoblastoma binding protein 9, serine hydrolase	NA	0.006374	5.522757	1.004428	0.92455671	0.972359
435626	Rufy4	RUN and FYVE domain containing 4, transcript varia	NA	0.034347	-0.11876	1.024093	0.92465723	0.972359
19288	Ptx3	pentraxin related gene	NA	-0.00916	3.866638	-1.00637	0.92473888	0.972359
16706	Ksr1	kinase suppressor of ras 1, transcript variant X5	NA	0.007375	5.071937	1.005125	0.92474933	0.972359
360013	Myo18a	myosin XVIIIA, transcript variant 4	NA	0.005222	7.325887	1.003626	0.92479923	0.972359
217826	Kcnk13	potassium channel, subfamily K, member 13, transcri	NA	0.029405	0.467081	1.020591	0.92482174	0.972359
64933	Ap3m2	adaptor-related protein complex 3, mu 2 subunit, tran	NA	0.005628	6.021538	1.003909	0.92499509	0.972359
224023	Klhl22	kelch-like 22, transcript variant 1	NA	0.004616	6.22898	1.003205	0.9250071	0.972359
98741	Kcnb2	potassium voltage gated channel, Shab-related subfa	NA	0.00732	4.473922	1.005087	0.92500863	0.972359
69228	Zfp746	zinc finger protein 746, transcript variant 2	NA	-0.0057	5.452929	-1.00396	0.92523107	0.972359
11438	Chrna4	cholinergic receptor, nicotinic, alpha polypeptide 4, tra	NA	-0.00599	5.741865	-1.00416	0.92524156	0.972359
51788	H2az1	H2A.Z variant histone 1, transcript variant 1	NA	0.005902	8.125359	1.0041	0.92529902	0.972359
73844	Ankrd45	ankyrin repeat domain 45, transcript variant X9	NA	0.006856	4.967713	1.004764	0.92539565	0.972359
67694	Ift74	intraflagellar transport 74, transcript variant X9	NA	0.00697	4.770878	1.004843	0.9254856	0.972359
243272	Sbno1	strawberry notch 1, transcript variant 2	NA	0.004671	7.232316	1.003243	0.92552275	0.972359
234290	BC030500	cDNA sequence BC030500, transcript variant X1	NA	0.011941	3.068901	1.008311	0.92554037	0.972359
74760	Rab3il1	RAB3A interacting protein (rabin3)-like 1, transcript va	NA	0.007983	4.093791	1.005549	0.92555864	0.972359
381650	Thap6	THAP domain containing 6	NA	0.0137	2.215204	1.009542	0.92567282	0.972359
69834	Rab43	RAB43, member RAS oncogene family, transcript var	NA	-0.00722	4.751332	-1.00502	0.92567598	0.972359
56390	Znrd2	zinc ribbon domain containing 2	NA	0.009658	3.83349	1.006717	0.92571013	0.972359
11461	Actb	actin, beta	NA	-0.00459	11.4644	-1.00319	0.92572232	0.972359
338364	Trim65	tripartite motif-containing 65, transcript variant X2	NA	-0.01323	2.382146	-1.00921	0.92574881	0.972359
11988	Slc7a2	solute carrier family 7 (cationic amino acid transporte	NA	0.011178	3.344854	1.007778	0.92598145	0.972402
56856	Insm2	insulinoma-associated 2	NA	-0.01933	0.968419	-1.01349	0.92601029	0.972402
75079	Zbtb49	zinc finger and BTB domain containing 49, transcript	NA	0.009601	3.992023	1.006677	0.92605465	0.972402
107702	Rnh1	ribonuclease/angiogenin inhibitor 1, transcript variant	NA	0.008395	4.307089	1.005836	0.92608811	0.972402
30058	Timm8a1	translocase of inner mitochondrial membrane 8A1	NA	0.00764	4.244304	1.00531	0.92613934	0.972402
58184	Cnot9	CCR4-NOT transcription complex, subunit 9, transcri	NA	0.005435	6.208671	1.003774	0.92614826	0.972402
17955	Nap1l4	nucleosome assembly protein 1-like 4, transcript varia	NA	-0.00472	7.740771	-1.00328	0.92633515	0.972424
235320	Zbtb16	zinc finger and BTB domain containing 16, transcript	NA	0.009769	4.07148	1.006795	0.92639082	0.972424
208263	Tor1aip1	torsin A interacting protein 1, transcript variant 3	NA	-0.00605	5.232529	-1.0042	0.92640169	0.972424
67661	Ift172	intraflagellar transport 172	NA	0.006243	5.258757	1.004337	0.92640857	0.972424

66225	Llph	LLP homolog, long-term synaptic facilitation (Aplysia)	NA		0.008834	4.399278	1.006142	0.92648421	0.972441
235599	6430571L13Rik	RIKEN cDNA 6430571L13 gene	NA		0.01214	2.871262	1.008451	0.9267063	0.972561
27015	Polk	polymerase (DNA directed), kappa, transcript variant	NA		-0.01152	3.078131	-1.00802	0.92671865	0.972561
232785	Zfp783	zinc finger protein 783	NA		0.009049	3.339264	1.006292	0.92678277	0.972566
240066	Zfp870	zinc finger protein 870	NA		-0.00847	3.888954	-1.00589	0.92687831	0.972604
106021	Topors	topoisomerase I binding, arginine/serine-rich, transcri	NA		-0.00753	5.405448	-1.00523	0.92699403	0.972662
77951	Cyp20a1	cytochrome P450, family 20, subfamily a, polypeptide	NA		0.009163	4.013628	1.006371	0.92740219	0.973028
269630	5031425E22Rik	RIKEN cDNA 5031425E22 gene, transcript variant 3	NA		-0.00798	4.330326	-1.00555	0.92752596	0.973095
108154	Adamts6	a disintegrin-like and metallopeptidase (reprolysin typ	NA		-0.01062	3.198079	-1.00739	0.92762777	0.973139
21428	Mlx	MAX-like protein X, transcript variant 2	NA		-0.00675	5.111234	-1.00469	0.9277791	0.973235
102635498	Gm32819	predicted gene, 32819, transcript variant X7	NA		0.051434	-0.94495	1.036294	0.92800271	0.973316
83675	Bicc1	BicC family RNA binding protein 1, transcript variant	NA		0.008306	4.019012	1.005774	0.9280041	0.973316
56360	Acot9	acyl-CoA thioesterase 9, transcript variant 1	NA		-0.00732	4.635789	-1.00509	0.92803698	0.973316
68270	Dnaaf1	dynein, axonemal assembly factor 1	NA		-0.04033	-0.69149	-1.02835	0.92809563	0.973316
66039	Tmem254a	transmembrane protein 254a, transcript variant 1	NA		0.006474	5.50504	1.004497	0.92826748	0.973434
223664	Lrrc14	leucine rich repeat containing 14, transcript variant 3	NA		-0.008	4.142368	-1.00556	0.92835081	0.973458
118568450	LOC118568450	uncharacterized LOC118568450	NA		0.024503	0.338003	1.017129	0.92851367	0.973564
30937	Lmcd1	LIM and cysteine-rich domains 1	NA		-0.01022	2.866164	-1.00711	0.92857099	0.973564
12908	Crat	carnitine acetyltransferase, transcript variant X5	NA		0.006042	5.042161	1.004197	0.92864342	0.973577
231713	Naa25	N(alpha)-acetyltransferase 25, NatB auxiliary subunit	NA		-0.00466	5.844951	-1.00324	0.92876181	0.973586
16776	Lama5	laminin, alpha 5, transcript variant X3	NA		-0.00872	3.701137	-1.00606	0.92877203	0.973586
627375	Gm6750	predicted gene 6750	NA		-0.00738	4.057608	-1.00513	0.92893328	0.973664
215819	Nhsl1	NHS-like 1, transcript variant 2	NA		0.005416	6.037102	1.003761	0.92896603	0.973664
319562	9630028B13Rik	RIKEN cDNA 9630028B13 gene	NA		-0.01522	1.945101	-1.01061	0.92908999	0.973731
13607	Eda	ectodysplasin-A, transcript variant 7	NA		-0.01077	3.010897	-1.00749	0.92917895	0.973758
23980	Pebp1	phosphatidylethanolamine binding protein 1	NA		-0.00393	8.581176	-1.00273	0.92923507	0.973758
12716	Ckmt1	creatine kinase, mitochondrial 1, ubiquitous, transcrip	NA		-0.00576	4.917875	-1.004	0.92931718	0.973776
105246380	Gm41676	predicted gene, 41676	NA		0.022172	1.149308	1.015487	0.92937226	0.973776
13627	Eef1a1	eukaryotic translation elongation factor 1 alpha 1	NA		0.004868	12.76229	1.00338	0.92949697	0.973816
67059	Ola1	Obg-like ATPase 1, transcript variant 1	NA		0.004604	6.481744	1.003196	0.92966101	0.973816
330189	Tmem120b	transmembrane protein 120B	NA		0.011446	3.428031	1.007965	0.92966392	0.973816
100041979	Gm3604	predicted gene 3604, transcript variant X7	NA		-0.01531	1.516284	-1.01067	0.92971698	0.973816
13180	Pcbd1	pterin 4 alpha carbinolamine dehydratase/dimerizatio	NA		0.01169	2.936086	1.008136	0.9298459	0.973816
93892	Pcdhb21	protocadherin beta 21	NA		0.026943	-0.15123	1.018851	0.92987984	0.973816
629133	Gm20472	predicted gene 20472	NA		0.014533	1.812777	1.010125	0.92989847	0.973816
19193	Pipox	pipecolic acid oxidase	NA		-0.0158	2.453866	-1.01101	0.92997026	0.973816
100503659	Cbarp	calcium channel, voltage-dependent, beta subunit as	NA		-0.00472	7.171642	-1.00327	0.93005529	0.973816
108169031	Gm46903	predicted gene, 46903	NA		0.036683	-0.69141	1.025753	0.93005613	0.973816
13839	Epha5	Eph receptor A5	NA		-0.00472	6.552037	-1.00328	0.93009327	0.973816
105245869	Gm4675	predicted gene 4675, transcript variant 1	NA		-0.02254	0.750788	-1.01574	0.93012825	0.973816
15387	Hnrnpk	heterogeneous nuclear ribonucleoprotein K, transcrip	NA		0.003858	9.88795	1.002678	0.93030085	0.973849
170737	Znrf1	zinc and ring finger 1, transcript variant 1	NA		0.004556	7.82827	1.003163	0.93032281	0.973849
16195	Il6st	interleukin 6 signal transducer	NA		-0.00556	5.326308	-1.00386	0.930362	0.973849
74525	Fam234b	family with sequence similarity 234, member B, trans	NA		0.003861	7.010248	1.00268	0.9304328	0.973849
99100	Cep152	centrosomal protein 152, transcript variant X9	NA		-0.01174	3.080409	-1.00817	0.93055956	0.973849
232943	Klc3	kinesin light chain 3, transcript variant 3	NA		0.02771	0.165479	1.019393	0.93059832	0.973849
56046	Uqcc1	ubiquinol-cytochrome c reductase complex assembly	NA		-0.00639	5.384384	-1.00444	0.93064969	0.973849
102641860	Gm26578	predicted gene, 26578, transcript variant X2	NA		-0.02573	0.504042	-1.018	0.93066821	0.973849
66263	1810014B01Rik	RIKEN cDNA 1810014B01 gene	NA		0.014382	2.090204	1.010018	0.93077064	0.973849
56503	Ankrd49	ankyrin repeat domain 49, transcript variant X1	NA		0.007471	4.494019	1.005192	0.9308605	0.973849
106512	Gpsm3	G-protein signalling modulator 3 (AGS3-like, C. elega	NA		0.017532	1.295311	1.012226	0.93087782	0.973849
545007	Gm5796	predicted gene 5796	NA		-0.0081	3.741593	-1.00563	0.93093654	0.973849
68183	Bcas2	breast carcinoma amplified sequence 2, transcript va	NA		0.004753	6.441205	1.0033	0.93095812	0.973849
623796	Ptma-ps2	prothymosin alpha, pseudogene 2	NA		-0.0298	0.356589	-1.02087	0.93104817	0.973849
20411	Sorbs1	sorbin and SH3 domain containing 1, transcript varia	NA		0.004122	6.989831	1.002861	0.93109626	0.973849
70966	4931415C17Rik	RIKEN cDNA 4931415C17 gene	NA		-0.02781	0.215048	-1.01947	0.93111712	0.973849
80877	Lrba	LPS-responsive beige-like anchor, transcript variant	NA		-0.00676	4.701797	-1.00469	0.93122373	0.973898
80906	Kcnip2	Kv channel-interacting protein 2, transcript variant d	NA		-0.00822	3.83781	-1.00572	0.93143904	0.974052
78748	Rassf10	Ras association (RalGDS/AF-6) domain family (N-ter	NA		0.016358	1.607164	1.011403	0.93149116	0.974052
118567332	LOC118567332	uncharacterized LOC118567332	NA		-0.02719	0.647574	-1.01903	0.93161038	0.974114
67841	Atg3	autophagy related 3, transcript variant 1	NA		-0.00507	5.536229	-1.00352	0.93172746	0.974174
320026	A330076H08Rik	RIKEN cDNA A330076H08 gene	NA		0.013499	2.182432	1.009401	0.93200633	0.974245
14654	Glra1	glycine receptor, alpha 1 subunit, transcript variant 2	NA		-0.02448	0.259439	-1.01711	0.93201978	0.974245
73729	Zfp383	zinc finger protein 383	NA		-0.0059	4.80486	-1.0041	0.93204	0.974245
320923	Map7d3	MAP7 domain containing 3	NA		-0.02696	-0.02079	-1.01886	0.93209549	0.974245
233058	Zfp420	zinc finger protein 420, transcript variant 2	NA		-0.00584	4.744398	-1.00405	0.93213018	0.974245
338348	Ttc16	tetratricopeptide repeat domain 16, transcript variant	NA		-0.02926	-0.17744	-1.02049	0.932155	0.974245
71991	Ercc8	excision repaiross-complementing rodent repair defic	NA		-0.00715	4.476384	-1.00497	0.93226612	0.974299

22401	Zmat3	zinc finger matrin type 3	NA	-0.0053	5.648309	-1.00368	0.93241159	0.974329
68693	Hnrnpul2	heterogeneous nuclear ribonucleoprotein U-like 2	NA	-0.00401	8.249158	-1.00278	0.93245603	0.974329
17350	Mlh1	mutL homolog 1, transcript variant 1	NA	0.007066	4.414412	1.00491	0.93247478	0.974329
215476	Prr14l	proline rich 14-like, transcript variant X13	NA	0.006741	5.287797	1.004683	0.9326922	0.974445
102631705	Gm29975	predicted gene, 29975, transcript variant X6	NA	-0.0091	4.475181	-1.00633	0.93270507	0.974445
76892	Rnft1	ring finger protein, transmembrane 1	NA	0.008061	3.843239	1.005603	0.93298353	0.974673
22666	Zbtb14	zinc finger and BTB domain containing 14, transcript	NA	0.004752	5.715112	1.003299	0.93350627	0.975157
18762	Prkcz	protein kinase C, zeta, transcript variant 2	NA	-0.00408	6.687888	-1.00283	0.93387157	0.975448
102640681	Gm36693	predicted gene, 36693	NA	0.026017	-0.17408	1.018197	0.93390494	0.975448
382066	Prdm10	PR domain containing 10, transcript variant 1	NA	0.005212	5.41433	1.003619	0.93446305	0.975918
245350	AA414768	expressed sequence AA414768	NA	0.013774	2.58305	1.009593	0.93449744	0.975918
115486508	Gm37292	predicted gene, 37292	NA	-0.01591	1.418675	-1.01109	0.93453492	0.975918
20686	Spa17	sperm autoantigenic protein 17, transcript variant 1	NA	0.013342	1.975565	1.009291	0.93466828	0.97596
56220	Zfp386	zinc finger protein 386 (Kruppel-like), transcript variar	NA	0.004501	5.607596	1.003125	0.93469524	0.97596
73130	Tmed5	transmembrane p24 trafficking protein 5, transcript va	NA	0.006529	4.689008	1.004536	0.93496821	0.976148
216161	Sbno2	strawberry notch 2, transcript variant 2	NA	-0.00685	4.638687	-1.00476	0.93500168	0.976148
12273	C5ar1	complement component 5a receptor 1, transcript vari	NA	0.024309	0.098608	1.016993	0.93505519	0.976148
118568581	LOC118568581	uncharacterized LOC118568581, transcript variant X	NA	0.01063	2.934683	1.007395	0.93513819	0.97615
223922	Atf7	activating transcription factor 7, transcript variant 2	NA	-0.00522	5.122061	-1.00363	0.93517719	0.97615
105246114	Gm30445	predicted gene, 30445	NA	0.012176	2.003669	1.008475	0.93529221	0.976207
105500046	Gm30731	predicted gene, 30731	NA	-0.02353	0.231007	-1.01645	0.93535285	0.976208
108168734	2900093K20Rik	RIKEN cDNA 2900093K20 gene	NA	-0.0173	1.28486	-1.01206	0.93542685	0.976223
233878	Sez6l2	seizure related 6 homolog like 2, transcript variant X4	NA	-0.00402	7.500095	-1.00279	0.9356362	0.976379
233919	Gpr26	G protein-coupled receptor 26	NA	-0.00997	2.661932	-1.00694	0.93570866	0.976392
75616	Smim15	small integral membrane protein 15	NA	-0.00615	5.217474	-1.00428	0.93579423	0.97641
75788	Smurf1	SMAD specific E3 ubiquitin protein ligase 1, transcrip	NA	0.004397	6.581782	1.003052	0.93589197	0.97641
108168104	Gm46433	predicted gene, 46433	NA	0.02606	0.321465	1.018228	0.93597834	0.97641
234365	Yjefn3	YjeF N-terminal domain containing 3	NA	-0.02915	0.239944	-1.02041	0.93600781	0.97641
229357	Gpr149	G protein-coupled receptor 149, transcript variant 1	NA	0.010521	2.376078	1.007319	0.93602581	0.97641
58222	Rab37	RAB37, member RAS oncogene family, transcript vai	NA	0.02317	0.11005	1.01619	0.93621682	0.976438
69681	Cdk3	cyclin-dependent kinase 3	NA	-0.02287	0.961089	-1.01598	0.93623929	0.976438
320916	Wscd2	WSC domain containing 2	NA	-0.00731	4.012923	-1.00508	0.93627846	0.976438
19157	Cyth1	cytohesin 1, transcript variant 4	NA	-0.00447	5.954893	-1.0031	0.93629299	0.976438
19090	Prkdc	protein kinase, DNA activated, catalytic polypeptide	NA	-0.00704	3.992143	-1.00489	0.93642887	0.976504
100177	Zmym6	zinc finger, MYM-type 6, transcript variant 3	NA	-0.00505	5.589646	-1.0035	0.9364767	0.976504
80281	Cttnbp2nl	CTTNBP2 N-terminal like, transcript variant 2	NA	-0.00404	6.819131	-1.00281	0.93664856	0.976614
102640201	Gm36325	predicted gene, 36325, transcript variant X2	NA	-0.02787	-0.23607	-1.0195	0.93674903	0.976614
13418	Dnajc1	DnaJ heat shock protein family (Hsp40) member C1,	NA	0.006144	4.724268	1.004268	0.93677781	0.976614
76178	Coa5	cytochrome C oxidase assembly factor 5	NA	-0.00405	6.280497	-1.00281	0.93684619	0.976614
69900	Mfsd11	major facilitator superfamily domain containing 11, tra	NA	-0.00548	5.099088	-1.00381	0.93688244	0.976614
56289	Rassf1	Ras association (RalGDS/AF-6) domain family memb	NA	0.006727	4.376981	1.004674	0.93705815	0.976735
66914	Vps28	vacuolar protein sorting 28, transcript variant 1	NA	0.004468	6.017979	1.003101	0.93720913	0.976792
64074	Smoc2	SPARC related modular calcium binding 2	NA	-0.01234	2.325403	-1.00859	0.93723288	0.976792
211480	Kcnj14	potassium inwardly-rectifying channel, subfamily J, m	NA	-0.02168	0.689375	-1.01514	0.93730224	0.976802
110157	Raf1	v-raf-leukemia viral oncogene 1, transcript variant X3	NA	-0.0039	7.429369	-1.0027	0.93737202	0.976812
107271	Yars	tyrosyl-tRNA synthetase, transcript variant 1	NA	0.004021	6.646672	1.002791	0.93747905	0.976861
50490	Nox4	NADPH oxidase 4, transcript variant X2	NA	0.01369	1.847909	1.009534	0.93761457	0.97694
74490	Mamstr	MEF2 activating motif and SAP domain containing tra	NA	-0.02048	1.756198	-1.0143	0.93777004	0.977022
100041621	Gm3435	predicted gene 3435, transcript variant X5	NA	-0.01073	3.594279	-1.00747	0.93781411	0.977022
207425	Wdr11	WD repeat domain 11	NA	-0.00459	5.710687	-1.00319	0.93800278	0.977156
68520	Zfyve21	zinc finger, FYVE domain containing 21, transcript va	NA	0.008886	3.327851	1.006178	0.93817006	0.977268
22320	Vamp8	vesicle-associated membrane protein 8	NA	-0.01154	2.486625	-1.00803	0.93831028	0.977302
93876	Pcdhb5	protocadherin beta 5	NA	0.010725	2.526374	1.007462	0.93832751	0.977302
68816	Ppil1	peptidylprolyl isomerase (cyclophilin)-like 1	NA	0.005033	5.440499	1.003495	0.9383829	0.977302
226777	C130074G19Rik	RIKEN cDNA C130074G19 gene	NA	0.008425	4.132277	1.005857	0.93885698	0.977361
72195	Supt7l	SPT7-like, STAGA complex gamma subunit	NA	0.006785	4.529336	1.004714	0.93896593	0.977361
69315	1700001L19Rik	RIKEN cDNA 1700001L19 gene	NA	-0.01242	1.969979	-1.00865	0.93900679	0.977361
101056014	AY036118	cDNA sequence AY036118	NA	0.029239	3.613835	1.020473	0.93910785	0.977361
108167408	Gm45971	predicted gene, 45971, transcript variant X4	NA	0.02476	-0.00066	1.01731	0.93912599	0.977361
54562	Lrrc6	leucine rich repeat containing 6 (testis)	NA	-0.02103	0.685839	-1.01469	0.93915292	0.977361
20148	Dhrs3	dehydrogenase/reductase (SDR family) member 3, tr	NA	0.007758	3.700288	1.005392	0.93916557	0.977361
245026	Col6a6	collagen, type VI, alpha 6, transcript variant 1	NA	-0.02226	0.473775	-1.01555	0.93929395	0.977361
16328	Cep250	centrosomal protein 250, transcript variant X7	NA	-0.0066	5.758918	-1.00459	0.93929431	0.977361
66471	Anp32e	acidic (leucine-rich) nuclear phosphoprotein 32 family	NA	0.003627	7.561881	1.002518	0.93939342	0.977361
17079	Cd180	CD180 antigen, transcript variant 2	NA	0.029833	0.261794	1.020894	0.93944175	0.977361
105245650	Gm41077	predicted gene, 41077	NA	0.02099	0.427831	1.014656	0.93944755	0.977361
18548	Pcsk1	proprotein convertase subtilisin/kexin type 1, transcrip	NA	0.011667	2.3698	1.00812	0.93947863	0.977361
66177	Ubl5	ubiquitin-like 5, transcript variant 3	NA	-0.00454	6.474041	-1.00315	0.93951066	0.977361

76380	Cep112	centrosomal protein 112, transcript variant X8	NA	0.01178	3.125009	1.008199	0.9395412	0.977361
108167537	Gm46043	predicted gene, 46043, transcript variant X1	NA	-0.02715	0.008346	-1.019	0.93954198	0.977361
28030	Gfm1	G elongation factor, mitochondrial 1	NA	-0.0051	4.922385	-1.00354	0.93958658	0.977361
72091	Snhg7	small nucleolar RNA host gene 7	NA	-0.00923	2.710904	-1.00642	0.93959212	0.977361
102633439	Gm14154	predicted gene 14154, transcript variant X1	NA	0.025158	-0.20586	1.017591	0.93965335	0.977361
105244278	Gm39918	predicted gene, 39918, transcript variant 1	NA	0.021292	0.023892	1.014868	0.93967006	0.977361
64075	Smoc1	SPARC related modular calcium binding 1, transcript	NA	0.004662	5.715789	1.003237	0.9397008	0.977361
97112	Nmd3	NMD3 ribosome export adaptor	NA	0.005378	4.865116	1.003734	0.94011067	0.977676
12896	Cpt2	carnitine palmitoyltransferase 2	NA	0.011531	3.142342	1.008024	0.94012356	0.977676
218695	Gm10044	predicted gene 10044, transcript variant X3	NA	-0.04067	-0.55032	-1.02859	0.94018394	0.977676
67832	Brix1	BRX1, biogenesis of ribosomes	NA	-0.00503	5.569639	-1.00349	0.94031606	0.977751
213409	Lemd1	LEM domain containing 1, transcript variant 4	NA	0.011849	2.673419	1.008247	0.94040314	0.977752
211347	Pank3	pantothenate kinase 3	NA	-0.00558	6.988954	-1.00387	0.94054913	0.977752
67997	Ddx59	DEAD box helicase 59	NA	-0.00695	3.539908	-1.00483	0.94057342	0.977752
16913	Psmb8	proteasome (prosome, macropain) subunit, beta type	NA	0.027075	-0.149	1.018944	0.94063718	0.977752
108960	Irak2	interleukin-1 receptor-associated kinase 2, transcript	NA	-0.01519	1.667415	-1.01058	0.94067435	0.977752
12190	Brca2	breast cancer 2, early onset, transcript variant X3	NA	-0.00673	3.698687	-1.00468	0.94067813	0.977752
19338	Rab33b	RAB33B, member RAS oncogene family	NA	-0.00645	4.955974	-1.00448	0.94089865	0.977919
69707	Iqcg	IQ motif containing G, transcript variant X1	NA	0.011385	2.69878	1.007923	0.94097685	0.977938
93765	Ube2n	ubiquitin-conjugating enzyme E2N	NA	-0.00406	8.022645	-1.00282	0.94119109	0.978098
272538	Tango6	transport and golgi organization 6	NA	-0.01166	2.479451	-1.00812	0.94144027	0.978244
57249	Gabrq	gamma-aminobutyric acid (GABA) A receptor, subuni	NA	-0.00934	2.841957	-1.0065	0.94145145	0.978244
102634769	Gm32273	predicted gene, 32273, transcript variant 2	NA	0.025802	-0.16292	1.018046	0.94201018	0.978733
66143	Eef1e1	eukaryotic translation elongation factor 1 epsilon 1	NA	0.004695	5.945741	1.00326	0.94204231	0.978733
378460	Pram1	PML-RAR alpha-regulated adaptor molecule 1, trans	NA	0.024616	-0.07477	1.017209	0.94253257	0.97918
20116	Rps8	ribosomal protein S8	NA	0.005478	8.534356	1.003804	0.94272422	0.979287
381260	Gm973	predicted gene 973, transcript variant X24	NA	0.010294	2.184771	1.00716	0.94279148	0.979287
121022	Mrps6	mitochondrial ribosomal protein S6	NA	0.004867	5.026095	1.003379	0.94286492	0.979287
67134	Nop56	NOP56 ribonucleoprotein, transcript variant 1	NA	0.003453	7.095104	1.002396	0.94293379	0.979287
546336	Prrg1	proline rich Gla (G-carboxyglutamic acid) 1, transcript	NA	0.009059	3.11044	1.006299	0.94297411	0.979287
77038	Arfgap2	ADP-ribosylation factor GTPase activating protein 2,	NA	0.00511	6.000497	1.003548	0.94299722	0.979287
216853	Wrap53	WD repeat containing, antisense to Trp53, transcript	NA	-0.007	3.87645	-1.00486	0.94354843	0.979652
213012	Abhd10	abhydrolase domain containing 10, transcript variant	NA	-0.00572	5.045679	-1.00397	0.94383864	0.979652
106344	Rfc4	replication factor C (activator 1) 4	NA	-0.0067	4.06711	-1.00466	0.9439715	0.979652
18220	Nucb1	nucleobindin 1, transcript variant X1	NA	0.00418	6.144145	1.002901	0.94407215	0.979652
67544	Fam120b	family with sequence similarity 120, member B, trans	NA	-0.00366	6.045639	-1.00254	0.94418302	0.979652
218271	B4galt7	xylosylprotein beta1,4-galactosyltransferase, polypep	NA	0.004972	4.790371	1.003452	0.94419695	0.979652
16922	Phyh	phytanoyl-CoA hydroxylase	NA	0.004464	5.088241	1.003099	0.94421619	0.979652
56458	Foxo1	forkhead box O1	NA	0.004953	5.022838	1.003439	0.9442657	0.979652
52898	Rnasek	ribonuclease, RNase K	NA	-0.00581	5.550666	-1.00404	0.94427269	0.979652
242466	Zfp462	zinc finger protein 462, transcript variant X21	NA	-0.0036	7.85154	-1.0025	0.9442734	0.979652
12549	Arhgap31	Rho GTPase activating protein 31	NA	0.005375	4.455273	1.003733	0.94428537	0.979652
11551	Adra2a	adrenergic receptor, alpha 2a	NA	0.006233	4.454298	1.00433	0.94430139	0.979652
56424	Stub1	STIP1 homology and U-Box containing protein 1, trar	NA	-0.00322	6.593757	-1.00224	0.94432219	0.979652
70235	Poc1a	POC1 centriolar protein A	NA	0.01216	2.849431	1.008464	0.94434087	0.979652
12323	Camk2b	calcium/calmodulin-dependent protein kinase II, beta	NA	0.003484	8.049193	1.002418	0.94436237	0.979652
66180	P3h4	prolyl 3-hydroxylase family member 4 (non-enzymatic	NA	0.00777	3.709058	1.0054	0.94437592	0.979652
69684	Aarsd1	alanyl-tRNA synthetase domain containing 1	NA	-0.00421	5.294773	-1.00292	0.94442577	0.979652
268417	Zkscan17	zinc finger with KRAB and SCAN domains 17, transc	NA	0.004422	5.223313	1.00307	0.94449929	0.979652
21945	Dedd	death effector domain-containing, transcript variant 3	NA	-0.00481	5.529968	-1.00334	0.94452595	0.979652
223870	Senp1	SUMO1/sentrin specific peptidase 1, transcript varian	NA	0.004214	5.434921	1.002925	0.94458584	0.979652
53791	Tlr5	toll-like receptor 5, transcript variant X7	NA	0.026984	-0.59492	1.01888	0.94461313	0.979652
106026	AW121686	expressed sequence AW121686	NA	-0.0228	0.031573	-1.01593	0.944778	0.979706
22327	Vbp1	von Hippel-Lindau binding protein 1	NA	0.003459	7.086437	1.0024	0.94478547	0.979706
108168254	Gm29666	predicted gene 29666	NA	0.017478	0.754939	1.012188	0.94492934	0.979793
71687	Tmem25	transmembrane protein 25, transcript variant 2	NA	0.008209	3.5794	1.005707	0.94510599	0.979914
66340	Psenen	presenilin enhancer gamma secretase subunit, trans	NA	0.00494	4.744822	1.00343	0.94520385	0.979953
74919	Slc35f6	solute carrier family 35, member F6	NA	0.005839	4.200334	1.004056	0.94558401	0.980284
19941	Rpl26	ribosomal protein L26	NA	-0.00416	8.381488	-1.00289	0.94592443	0.980491
102633185	Gm31075	predicted gene, 31075, transcript variant X8	NA	0.018391	0.205847	1.012829	0.94593518	0.980491
76302	Pcnp	PEST proteolytic signal containing nuclear protein, tra	NA	-0.00323	7.284446	-1.00224	0.94596428	0.980491
66979	Pole4	polymerase (DNA-directed), epsilon 4 (p12 subunit),	NA	0.004437	5.520087	1.00308	0.94624261	0.980683
114716	Spred2	sprouty-related EVH1 domain containing 2, transcript	NA	0.004102	6.090682	1.002847	0.94627036	0.980683
14182	Fgfr1	fibroblast growth factor receptor 1, transcript variant 2	NA	-0.00341	6.750107	-1.00237	0.94652858	0.980889
269941	Chsy1	chondroitin sulfate synthase 1, transcript variant X1	NA	0.003691	5.751243	1.002562	0.94669566	0.980975
228608	Smox	spermine oxidase, transcript variant X15	NA	-0.00489	5.130322	-1.00339	0.94673218	0.980975
68992	Zfp580	zinc finger protein 580	NA	-0.00768	4.874915	-1.00534	0.94688186	0.981067
99982	Kdm1a	lysine (K)-specific demethylase 1A, transcript variant	NA	-0.00326	7.777674	-1.00226	0.94706378	0.981128

19128	Pros1	protein S (alpha), transcript variant X1	NA	0.007146	3.584929	1.004966	0.94709887	0.981128
319701	Fbxo48	F-box protein 48, transcript variant X7	NA	-0.01033	2.317555	-1.00719	0.94716523	0.981128
56838	Ccl28	chemokine (C-C motif) ligand 28	NA	-0.01671	1.181985	-1.01165	0.94718174	0.981128
18674	Slc25a3	solute carrier family 25 (mitochondrial carrier, phosph	NA	0.003737	8.119503	1.002594	0.94733172	0.981221
638502	Gm7240	predicted gene 7240	NA	0.016553	0.574535	1.01154	0.94750902	0.981239
65106	Arl6ip5	ADP-ribosylation factor-like 6 interacting protein 5	NA	-0.00316	6.687508	-1.0022	0.94751967	0.981239
76219	Arxes1	adipocyte-related X-chromosome expressed sequenc	NA	-0.00565	5.89889	-1.00392	0.94768803	0.981239
319625	Galm	galactose mutarotase	NA	0.014848	1.424666	1.010345	0.94770203	0.981239
74196	Ttc27	tetratricopeptide repeat domain 27	NA	-0.00542	4.223076	-1.00377	0.94770731	0.981239
57279	Slc25a20	solute carrier family 25 (mitochondrial carnitine/acylca	NA	0.005042	4.742123	1.003501	0.94772401	0.981239
13661	Ehf	ets homologous factor, transcript variant X3	NA	-0.02949	-0.52029	-1.02065	0.9477885	0.981239
115487412	LOC115487412	uncharacterized LOC115487412	NA	0.018198	0.4432	1.012694	0.94790363	0.981239
74486	Osbpl10	oxysterol binding protein-like 10, transcript variant X5	NA	-0.00543	4.545211	-1.00377	0.9479529	0.981239
320119	Rps6kc1	ribosomal protein S6 kinase polypeptide 1, transcript	NA	0.005774	5.300681	1.00401	0.94796617	0.981239
69090	Ascc1	activating signal cointegrator 1 complex subunit 1, tra	NA	-0.00506	4.984772	-1.00351	0.94802046	0.981239
101994	Champ1	chromosome alignment maintaining phosphoprotein 1	NA	-0.00338	6.277779	-1.00234	0.94812039	0.981239
13170	Dbp	D site albumin promoter binding protein	NA	-0.00887	2.753557	-1.00617	0.94813324	0.981239
613254	AA465934	expressed sequence AA465934	NA	-0.01886	0.383344	-1.01316	0.94820766	0.981254
71840	Tekt4	tektin 4	NA	-0.01973	0.125948	-1.01377	0.94840139	0.981372
245695	Tceanc	transcription elongation factor A (SII) N-terminal and (	NA	-0.00835	2.699846	-1.00581	0.94845767	0.981372
72225	1700003M07Rik	RIKEN cDNA 1700003M07 gene, transcript variant 2	NA	0.012615	2.609755	1.008782	0.94850295	0.981372
72308	Brf1	BRF1, RNA polymerase III transcription initiation facto	NA	0.003358	6.55267	1.00233	0.94866614	0.981479
269523	Vcp	valosin containing protein	NA	-0.00274	9.030523	-1.0019	0.94880404	0.981559
270198	Pfkfb4	6-phosphofructo-2-kinase/fructose-2,6-biphosphatase	NA	-0.00517	4.734549	-1.00359	0.94897619	0.981632
68277	2310057M21Rik	RIKEN cDNA 2310057M21 gene, transcript variant X	NA	0.00563	4.060592	1.00391	0.94902669	0.981632
71086	4933412E12Rik	RIKEN cDNA 4933412E12 gene, transcript variant 2	NA	-0.02049	0.332644	-1.01431	0.94913057	0.981632
230119	Zbtb5	zinc finger and BTB domain containing 5, transcript v	NA	0.003403	5.891776	1.002362	0.9491887	0.981632
19893	Rpgr	retinitis pigmentosa GTPase regulator, transcript varia	NA	0.003984	5.664445	1.002765	0.94919059	0.981632
213006	Mfsd4a	major facilitator superfamily domain containing 4A, tra	NA	0.011535	1.643925	1.008027	0.94923668	0.981632
208595	Mterf1b	mitochondrial transcription termination factor 1b, trans	NA	-0.00869	2.410786	-1.00604	0.94936024	0.981677
245020	Slc35g2	solute carrier family 35, member G2	NA	0.010033	2.8189	1.006979	0.94940073	0.981677
67382	Brd3	bromodomain containing 3, transcript variant 2	NA	0.00282	7.955494	1.001957	0.94959999	0.981821
66457	2810002D19Rik	RIKEN cDNA 2810002D19 gene	NA	-0.00782	2.871838	-1.00543	0.9497971	0.981901
70998	Phf6	PHD finger protein 6, transcript variant 2	NA	-0.00395	6.789923	-1.00274	0.94982433	0.981901
382423	Atxn7l3b	ataxin 7-like 3B	NA	-0.0026	9.037563	-1.00181	0.94985912	0.981901
13051	Cx3cr1	chemokine (C-X3-C motif) receptor 1	NA	0.007679	3.423569	1.005337	0.95033363	0.982247
16521	Kcnj5	potassium inwardly-rectifying channel, subfamily J, m	NA	0.013389	1.198758	1.009324	0.95040323	0.982247
100504568	Gm17634	predicted gene, 17634, transcript variant 1	NA	-0.02099	-0.22813	-1.01466	0.95041983	0.982247
268752	Wdfy2	WD repeat and FYVE domain containing 2, transcript	NA	-0.0041	5.044836	-1.00285	0.9504349	0.982247
219094	Khnyn	KH and NYN domain containing, transcript variant 1	NA	-0.00443	4.746764	-1.00307	0.9505412	0.982289
107652	Uap1	UDP-N-acetylglucosamine pyrophosphorylase 1, tran	NA	0.003775	5.610766	1.00262	0.95059682	0.982289
26920	Cntrl	centriolin, transcript variant 5	NA	-0.0046	4.724064	-1.0032	0.95075097	0.982386
12393	Runx2	runt related transcription factor 2, transcript variant 3	NA	0.014708	1.092734	1.010247	0.95098001	0.982432
67993	Nudt12	nudix (nucleoside diphosphate linked moiety X)-type	NA	-0.01017	2.032771	-1.00708	0.95100807	0.982432
269955	Rccd1	RCC1 domain containing 1	NA	-0.0073	3.730756	-1.00507	0.9510101	0.982432
17880	Myh11	myosin, heavy polypeptide 11, smooth muscle, transc	NA	0.011216	1.492057	1.007804	0.95103669	0.982432
11430	Acox1	acyl-Coenzyme A oxidase 1, palmitoyl, transcript vari	NA	0.003051	6.994013	1.002117	0.95127098	0.982445
78885	Coro7	coronin 7	NA	-0.0041	5.300332	-1.00285	0.95137537	0.982445
69109	Ccnq	cyclin Q	NA	-0.00699	3.303635	-1.00485	0.95145687	0.982445
327954	Dnah2	dynein, axonemal, heavy chain 2, transcript variant X	NA	-0.00674	3.402693	-1.00469	0.95153272	0.982445
16797	Lat	linker for activation of T cells, transcript variant X4	NA	-0.02166	-0.34701	-1.01513	0.95157032	0.982445
56324	Stam2	signal transducing adaptor molecule (SH3 domain an	NA	0.003663	5.586776	1.002542	0.95158667	0.982445
69573	Hilpda	hypoxia inducible lipid droplet associated, transcript v	NA	0.007752	3.134561	1.005387	0.95160856	0.982445
19289	Igdcc3	immunoglobulin superfamily, DCC subclass, member	NA	-0.00472	4.355837	-1.00328	0.95165459	0.982445
17129	Smad5	SMAD family member 5, transcript variant 3	NA	0.002877	6.981992	1.001996	0.95165991	0.982445
24000	Ptpn21	protein tyrosine phosphatase, non-receptor type 21, t	NA	-0.00632	3.597475	-1.00439	0.95169792	0.982445
226122	Ubtd1	ubiquitin domain containing 1	NA	-0.00712	3.071891	-1.00494	0.95172486	0.982445
243373	AI854703	expressed sequence AI854703	NA	0.005187	4.331177	1.003602	0.95177389	0.982445
68776	Taf11	TATA-box binding protein associated factor 11, transc	NA	-0.00386	5.072197	-1.00268	0.95189102	0.982461
102954	Nudt10	nudix (nucleoside diphosphate linked moiety X)-type	NA	0.003496	5.413705	1.002426	0.95191045	0.982461
219022	Ttc5	tetratricopeptide repeat domain 5, transcript variant 1	NA	0.003606	6.138685	1.002502	0.95220601	0.982701
11852	Rhob	ras homolog family member B	NA	-0.00322	7.505914	-1.00223	0.95226348	0.982701
110826	Etfb	electron transferring flavoprotein, beta polypeptide, tr	NA	-0.00581	4.662406	-1.00403	0.9523447	0.982723
237397	C2cd4c	C2 calcium-dependent domain containing 4C, transcr	NA	0.005584	4.127673	1.003878	0.95261356	0.982873
22258	Usp4	ubiquitin specific peptidase 4 (proto-oncogene), trans	NA	-0.00298	7.102008	-1.00207	0.95262801	0.982873
70093	Ube2q1	ubiquitin-conjugating enzyme E2Q family member 1	NA	0.002914	6.744339	1.002022	0.95267211	0.982873
13041	Ctsw	cathepsin W, transcript variant X1	NA	-0.0167	0.059784	-1.01164	0.95280372	0.982947
64051	Sv2a	synaptic vesicle glycoprotein 2 a	NA	0.002817	7.458514	1.001955	0.95318461	0.982963

57740	Stk32c	serine/threonine kinase 32C, transcript variant 1	NA	0.004561	4.87639	1.003167	0.9532275	0.982963
98932	Myl9	myosin, light polypeptide 9, regulatory, transcript vari	NA	-0.00473	4.113	-1.00328	0.95326361	0.982963
214704	Iqub	IQ motif and ubiquitin domain containing	NA	0.011591	1.463438	1.008066	0.95327564	0.982963
64143	Ralb	v-ral simian leukemia viral oncogene B, transcript var	NA	-0.00459	5.172441	-1.00318	0.95329885	0.982963
72519	Pip4p2	phosphatidylinositol-4,5-bisphosphate 4-phosphatase	NA	-0.00384	5.885453	-1.00266	0.95331459	0.982963
70675	Vcpip1	valosin containing protein (p97)/p47 complex interact	NA	0.004015	6.108636	1.002787	0.95346059	0.982963
56361	Pus1	pseudouridine synthase 1, transcript variant X5	NA	-0.00567	3.681596	-1.00394	0.95351326	0.982963
236266	Alms1	ALMS1, centrosome and basal body associated	NA	-0.00549	4.225646	-1.00381	0.95367124	0.982963
58231	Stk4	serine/threonine kinase 4	NA	-0.00412	5.833896	-1.00286	0.95370312	0.982963
320162	Cep95	centrosomal protein 95, transcript variant X3	NA	-0.00591	4.402679	-1.0041	0.95373406	0.982963
76479	Smndc1	survival motor neuron domain containing 1, transcript	NA	-0.00358	5.708622	-1.00248	0.95375919	0.982963
67767	Jagn1	jagunal homolog 1, transcript variant 1	NA	-0.00417	4.668169	-1.0029	0.95379072	0.982963
12292	Cacna1s	calcium channel, voltage-dependent, L type, alpha 1S	NA	-0.02077	0.416696	-1.0145	0.95379939	0.982963
230126	Shb	src homology 2 domain-containing transforming prote	NA	-0.00417	5.246942	-1.00289	0.9538032	0.982963
319876	Cobll1	Cobl-like 1, transcript variant X19	NA	-0.00801	3.056433	-1.00557	0.95381181	0.982963
241274	Pnpla7	patatin-like phospholipase domain containing 7	NA	0.008783	2.509929	1.006106	0.95384661	0.982963
67676	Rpp21	ribonuclease P 21 subunit	NA	-0.00671	2.904381	-1.00466	0.95392184	0.982979
15900	Irf8	interferon regulatory factor 8, transcript variant 2	NA	-0.01524	1.085654	-1.01062	0.95406849	0.983068
434402	Gm5617	predicted gene 5617	NA	0.014035	0.879763	1.009776	0.95418542	0.98307
22754	Zfp92	zinc finger protein 92, transcript variant 1	NA	0.008968	1.875456	1.006236	0.95419167	0.98307
66631	Mfsd14b	major facilitator superfamily domain containing 14B, t	NA	-0.00353	5.188099	-1.00245	0.95426575	0.983084
100038755	Gm9917	predicted gene 9917	NA	0.009986	1.757534	1.006946	0.95449105	0.983192
629945	Cep112it	centrosomal protein 112, intronic transcript	NA	-0.01682	0.084401	-1.01172	0.95449151	0.983192
16536	Kcnq2	potassium voltage-gated channel, subfamily Q, meml	NA	-0.00309	7.650938	-1.00215	0.9545546	0.983195
547176	Zc3h12b	zinc finger CCCH-type containing 12B, transcript vari	NA	-0.0087	2.787331	-1.00605	0.95480177	0.983387
69714	Tfpt	TCF3 (E2A) fusion partner, transcript variant 2	NA	0.005018	4.18592	1.003484	0.95496295	0.983453
22196	Ube2i	ubiquitin-conjugating enzyme E2I, transcript variant 1	NA	-0.00251	8.23266	-1.00174	0.95498664	0.983453
102635541	Gm32849	predicted gene, 32849	NA	-0.00684	5.673169	-1.00475	0.95514655	0.983483
218335	Clptm1l	CLPTM1-like	NA	-0.00271	6.665573	-1.00188	0.9551786	0.983483
14411	Slc6a12	solute carrier family 6 (neurotransmitter transporter, b	NA	0.015948	0.60628	1.011116	0.95519693	0.983483
13722	Aimp1	aminoacyl tRNA synthetase complex-interacting mult	NA	-0.00338	5.552911	-1.00235	0.95547377	0.983641
72391	Cdkn3	cyclin-dependent kinase inhibitor 3, transcript variant	NA	0.010888	1.550571	1.007576	0.95549573	0.983641
14368	Fzd6	frizzled class receptor 6, transcript variant 2	NA	0.005269	3.916941	1.003659	0.95553165	0.983641
240064	Zfp799	zinc finger protein 799, transcript variant 1	NA	-0.00336	5.574029	-1.00233	0.95563637	0.983686
239555	Mief1	mitochondrial elongation factor 1, transcript variant 3	NA	0.003295	5.476134	1.002287	0.95587825	0.983848
27395	Mrpl15	mitochondrial ribosomal protein L15, transcript varian	NA	-0.00353	5.430502	-1.00245	0.95608208	0.983848
78284	Creb3l4	cAMP responsive element binding protein 3-like 4, tra	NA	0.016797	0.458336	1.011711	0.95612831	0.983848
434438	Iho1	interactor of HORMAD1 1, transcript variant X1	NA	0.011309	1.344552	1.00787	0.95618448	0.983848
20429	Shox2	short stature homeobox 2, transcript variant 3	NA	0.005227	5.151135	1.003629	0.95622989	0.983848
81879	Tfcp2l1	transcription factor CP2-like 1, transcript variant X1	NA	-0.01036	1.797543	-1.00721	0.95625785	0.983848
234378	Klhl26	kelch-like 26, transcript variant 3	NA	-0.00478	4.657585	-1.00332	0.95627935	0.983848
27223	Trp53bp1	transformation related protein 53 binding protein 1, tra	NA	-0.00273	8.047616	-1.0019	0.95632479	0.983848
94279	Sfxn2	sideroflexin 2, transcript variant X2	NA	-0.00426	4.240412	-1.00296	0.95639655	0.983848
53901	Rcan2	regulator of calcineurin 2, transcript variant 4	NA	-0.00355	6.832402	-1.00247	0.95639762	0.983848
240442	Adnp2	ADNP homeobox 2, transcript variant X2	NA	0.003463	5.179855	1.002403	0.95653495	0.983927
108167592	Gm33082	predicted gene, 33082	NA	-0.01909	-0.34111	-1.01332	0.95671624	0.984051
245522	Zc4h2	zinc finger, C4H2 domain containing, transcript variar	NA	-0.00402	6.100637	-1.00279	0.95690341	0.984181
245650	Gucy2f	guanylate cyclase 2f	NA	-0.01922	-0.15679	-1.01341	0.95712953	0.984315
72780	Rspo3	R-spondin 3	NA	-0.00477	3.944017	-1.00331	0.95715431	0.984315
66066	Gng11	guanine nucleotide binding protein (G protein), gamm	NA	-0.00636	2.899443	-1.00442	0.95723178	0.984332
22427	Wrn	Werner syndrome RecQ like helicase, transcript varia	NA	-0.00523	3.505412	-1.00363	0.95731086	0.984351
12700	Cish	cytokine inducible SH2-containing protein, transcript v	NA	-0.00492	3.580714	-1.00342	0.95761874	0.984606
[truncated: 56,935 more chars]
