# Supplementary material for: Influenza A virus during pregnancy disrupts maternal intestinal immunity and fetal cortical development in a dose- and time-dependent manner
Source: Mol Psychiatry. 2024 Jul 3;30(1):13–28. doi: 10.1038/s41380-024-02648-9 (PMC11649561; doi:10.1038/s41380-024-02648-9)
Supplement: Supplementary file 12 — Supplemental Table S11 [file 41380_2024_2648_MOESM12_ESM.pdf]

Supplemental Table S11. Fetal brain cell counts and MFI of BAMs and microglia at E11.5 and 16.5, 2 and 7 dpi.

Timepoint	Brain Region	Analysis	Cell Markers	Control	$X31_{mod}$	$X31_{hi}$	p-value	Test	Statistic
E11.5, 2 dpi	Whole Brain	Counts	Iba1+CD206-	60.2 ± 3.74	53.3 ± 4.83	62.1 ± 4.84	0.38	One-way	F(2, 22) = 1.02
			Iba1+CD206+	91.9 ± 4.65	93.7 ± 4.16	91.1 ± 7.58	0.95	One-way	F(2, 22) = 0.05
	Fore- and mid-brain	Counts	Iba1+CD206-	30.1 ± 1.77	27.0 ± 2.59	30.5 ± 2.43	0.52	One-way	F(2, 22) = 0.68
			Iba1+CD206+	44.8 ± 3.72	43.3 ± 3.39	45.0 ± 5.90	0.96	One-way	F(2, 22) = 0.04
	Hindbrain	Counts	Iba1+CD206-	30.0 ± 2.25	26.3 ± 2.82	31.6 ± 3.23	0.42	One-way	F(2, 22) = 0.91
			Iba1+CD206+	47.0 ± 2.71	50.4 ± 4.92	46.1 ± 2.40	0.67	B-F + W	F*(2, 14.77) = 0.42
E16.5, 7 dpi	Whole Brain	Counts	Iba1+CD206-	20.3 ± 2.58	25.4 ± 3.93	27.4 ± 4.32	0.36	One-way	F(2, 22) = 1.07
			Iba1+CD206+	23.3 ± 2.44	29.9 ± 4.75	37.7 ± 2.54	**0.01**	One-way	F(2, 22) = 5.55
			Iba1+Ki67+	5.11 ± 0.64	4.40 ± 0.63	5.80 ± 1.37	0.58	One-way	F(2, 23) = 0.55
			Iba1+CD68+	44.0 ± 3.21	49.3 ± 3.55	59.7 ± 4.76	**0.03**	One-way	F(2, 23) = 4.47
		MFI	Ki67	68.6 ± 5.59	60.5 ± 9.21	59.1 ± 10.3	0.68	One-way	F(2, 24) = 0.39
	Right Hemisphere	Counts	Iba1+CD206-	21.5 ± 3.10	23.7 ± 3.21	28.9 ± 4.03	0.32	One-way	F(2, 21) = 1.21
			Iba1+CD206+	22.9 ± 3.98	29.7 ± 4.34	40.2 ± 3.25	**0.01**	One-way	F(2, 21) = 5.56
	Left Hemisphere	Counts	Iba1+CD206-	18.4 ± 2.49	27.8 ± 6.00	26.2 ± 4.80	0.32	One-way	F(2, 21) = 1.19
			Iba1+CD206+	24.1 ± 3.00	30.1 ± 5.26	35.8 ± 2.47	0.08	One-way	F(2, 21) = 2.92
	Parenchyma	Counts	Iba+Ki67+	2.70 ± 0.33	2.17 ± 0.55	2.18 ± 0.50	0.63	One-way	F(2, 23) = 0.48
			Iba1+CD68+	19.8 ± 1.41	21.4 ± 1.83	24.0 ± 1.70	0.18	One-way	F(2, 23) = 1.83
		%Iba1+	Iba1+CD68+	19.8 ± 1.41	21.4 ± 1.83	24.0 ± 1.70	0.10	One-way	F(2, 23) = 2.53
	Meninges	Counts	Iba1+CD206+	7.85 ± 2.05	16.0 ± 4.72	20.2 ± 2.62	**0.02**	One-way	F(2, 22) = 4.51
			Iba+Ki67+	1.27 ± 0.29	1.08 ± 0.30	1.95 ± 0.52	0.28	B-F + W	F*(2, 16.5) = 1.37
			Iba1+CD68+	11.6 ± 1.81	14.4 ± 3.39	18.9 ± 2.59	0.12	One-way	F(2, 23) = 2.33
		%Iba1+	Iba1+CD68+	81.4 ± 2.06	83.0 ± 2.73	83.7 ± 2.14	0.76	One-way	F(2, 23) = 0.28
	Choroid Plexus	Counts	Iba1+CD206+	15.5 ± 2.03	14.0 ± 0.84	17.5 ± 1.60	0.33	B-F + W	F*(2, 17.7) = 1.18
			Iba+Ki67+	1.10 ± 0.18	1.15 ± 0.17	1.65 ± 0.47	0.40	B-F + W	F*(2, 11.3) = 0.99
			Iba1+CD68+	12.6 ± 0.85	13.6 ± 0.86	16.9 ± 1.37	**0.02**	One-way	F(2, 23) = 4.54
		%Iba1+	Iba1+CD68+	85.4 ± 2.03	85.2 ± 3.66	89.6 ± 1.26	0.33	One-way	F(2, 23) = 1.15

Quantification of brain macrophages in E11.5 and 16.5 fetal brains. Forebrain = telencephalon and diencephalon, midbrain = mesencephalon, hindbrain = metencephelon and myencephelon. *MFI* = mean fluorescence intensity, *IAV* = influenza A virus, *dpi* = days post-inoculation, $X31_{mod}$ = IAV-X31 10^3 $TCID_{50}$, $X31_{hi}$ = IAV-X31 10^4 $TCID_{50}$. One-way ANOVA is the default statistical test unless residuals fail to meet normality (use K-W = Kruskal-Wallis) or homogeneity of variance (use B-F + W = Brown-Forsythe + Welch). Data are means ± SEM; bold font = $p < 0.05$, 2 dpi n = 12-14, 7 dpi n = 9-10 per treatment group.
